# SARS-CoV-2 genetic variations associated with COVID-19 pathogenicity

**DOI:** 10.1099/mgen.0.000734

**Published:** 2021-12-06

**Authors:** Pakorn Aiewsakun, Patrawee Nilplub, Patompon Wongtrakoongate, Suradej Hongeng, Arunee Thitithanyanont

**Affiliations:** ^1^​ Department of Microbiology, Faculty of Science, Mahidol University, 272, Rama VI Road, Ratchathewi, Bangkok, 10400, Thailand; ^2^​ Pornchai Matangkasombut Center for Microbial Genomics, Department of Microbiology, Faculty of Science, Mahidol University, 272, Rama VI Road, Ratchathewi, Bangkok, 10400, Thailand; ^3^​ Department of Biochemistry, Faculty of Science, Mahidol University, 272, Rama VI Road, Ratchathewi, Bangkok, 10400, Thailand; ^4^​ Center for Neuroscience, Faculty of Science, Mahidol University, 272, Rama VI Road, Ratchathewi, Bangkok, 10400, Thailand; ^5^​ Division of Hematology and Oncology, Department of Pediatrics, Faculty of Medicine, Ramathibodi Hospital, Mahidol University, 270, Rama VI Road, Ratchathewi, Bangkok, 10400, Thailand

**Keywords:** SARS-CoV-2, GWAS, nsp6

## Abstract

In this study, we performed genome-wide association analyses on SARS-CoV-2 genomes to identify genetic mutations associated with pre-symptomatic/asymptomatic COVID-19 cases. Various potential covariates and confounding factors of COVID-19 severity, including patient age, gender and country, as well as virus phylogenetic relatedness were adjusted for. In total, 3021 full-length genomes of SARS-CoV-2 generated from original clinical samples and whose patient status could be determined conclusively as either ‘pre-symptomatic/asymptomatic’ or ‘symptomatic’ were retrieved from the GISAID database. We found that the mutation 11 083G>T, located in the coding region of non-structural protein 6, is significantly associated with asymptomatic COVID-19. Patient age is positively correlated with symptomatic infection, while gender is not significantly correlated with the development of the disease. We also found that the effects of the mutation, patient age and gender do not vary significantly among countries, although each country appears to have varying baseline chances of COVID-19 symptom development.

## Data Summary

All sequence data used in this study were retrieved from the GISAID database. All supplementary information is available with the article as PDF files online. Supplementary material can be found in Figshare: https://doi.org/10.6084/m9.figshare.16528950.v1 [1].

**Impact Statement**

Coronavirus disease 2019 (COVID-19) is a major public health concern, caused by a novel coronavirus called SARS-CoV-2. As of now, more than 240 million cases of COVID-19 with more than 4.9 million deaths have been reported worldwide, and the rapid surge in the number of COVID-19 patients has overwhelmed hospitals’ capacity around the world. It has been reported that up to 80 % of SARS-CoV-2 infection might be asymptomatic, and this probably plays an important part in the rapid global transmission of the virus. To effectively contain the disease, it is thus imperative to understand the disease progression and the factors that drive the process. Here, we performed genome-wide association analyses on all SARS-CoV-2 genomes in the GISAID database, and found that a mutation at genomic position 11 083, namely the 11083G>T mutation, located in the coding region of non-structural protein 6, is significantly associated with asymptomatic COVID-19, adjusted for various confounders and covariates, including virus phylogenetic relatedness, patient age, gender and country. Our results have implications for the development of better and more informative test kits, for example, allowing potentially symptomatic cases to be distinguished from asymptomatic ones, and this could lead to more effective disease management and control.

## Introduction

Severe acute respiratory syndrome coronavirus 2 (SARS-CoV-2), the causative agent of coronavirus disease 2019 (COVID-19), was first reported in Wuhan, Hubei, China, in late December 2019 [2]. SARS-CoV-2 is a positive-sense ssRNA virus in the family *Coronaviridae* [3]. It is the seventh known coronavirus capable of infecting humans, after HCoV-229E, HCoV-OC43, HCoV NL63, HKU1, MERS-CoV and the original SARS-CoV. The first four typically cause non-lethal mild upper respiratory diseases, while the last two, as well as SARS-CoV-2, can cause severe lethal respiratory illnesses [4, 5].

The virus was found to spread around the globe, and as of now (October 2021) has infected more than 240 million people globally [6], which has overwhelmed hospitals in many countries. Although the case–fatality ratio of SARS-CoV-2 (~1.4–2.29 % [6, 7]) is lower than those of SARS-CoV (11 %) [8, 9] and MERS-CoV (34–37 %) [10], due to the greater number of infected cases, the number of deaths caused by SARS-CoV-2 is much greater than those caused by SARS-CoV and MERS-CoV. To date, at least 4.9 million deaths have been reported to be associated with SARS-CoV-2 infection [6]. A systematic review [11] showed that the serial interval time of COVID-19 (i.e. the time between illness onset in the primary case to the onset in the secondary case) is ~5.2 (mean range from 23 studies: 4.2–7.5) days while the incubation period (i.e. the time from infection to the onset of illness) is ~6.5 (mean range from 14 studies: 4.8–9) days. This shorter serial interval time suggests a substantial role of pre-symptomatic/asymptomatic transmission [11]. Mathematical model analyses suggested that more than half of all transmissions may be attributable to pre-symptomatic/asymptomatic transmission [12]. In order to effectively control the virus, it is thus important to understand the factors that underlie the disease severity and pathogenesis.

Several host factors correlated with COVID-19 severity have been identified, including patient age [13–15], patient gender [15–18], pre-existing medical conditions such as diabetes and chronic liver disease [15, 19], levels of CD4+ and CD8+ T cell counts, levels of IL-6 and IL-8 [20], and genotypes of *human leucocyte antigen* genes [21]. A number of viral genetic factors associated with the increase in COVID-19 severity have also been characterized. For example, nucleotide mutations 14 408C>T (i.e. the mutation P323L in RNA-dependent RNA polymerase protein) and 23 403A>G (i.e. the mutation D614G in the virus spike protein), which tended to be found together, were reported to show significant positive correlations with death [21], and to be found more frequently in severe cases than mild cases [22]. The latter mutation was also reported to increase infectivity of the virus in multiple human cell types [23]. In addition, a study has shown that ORF3a mutations are associated with higher infection and mortality rates of SARS-CoV-2 [24]. However, viral mutations associated with pre-symptomatic/asymptomatic SARS-CoV-2 infection are still poorly characterized.

There are currently millions of SARS-CoV-2 genomes from around the world made publicly available on the database of the Global Initiative on Sharing All Influenza Data (GISAID) [25], and some of which have patient status made publicly available. While most of the sequences were from symptomatic cases, some were from asymptomatic cases, presenting us an opportunity to examine viral genetic factors that might be associated with the virus’s pathogenicity on a large scale.

## Methods

### SARS-CoV-2 genome sequences with patient status

In total, 18 705 full-length/almost full-length genome sequences of SARS-CoV-2 (>29 000 nt) with patient status were downloaded from the GISAID database on 28 December 2020 together with their metadata. A table of acknowledgements can be found in Table S1 (available in the online version of this article), and the metadata can be found in Table S2. To allow for accurate determination of the genetic factors associated with COVID-19 pathogenicity, we only analysed sequences whose patient status could be unambiguously determined as either ‘pre-symptomatic/asymptomatic’ or ‘symptomatic’. We categorized ‘Asymptomatic/Released’, ‘Asymptomatic’, ‘Asymptomatic, identified as positive during preoperation investigation’, ‘No clinical signs’ and ‘No clinical signs without hospitalization’ as ‘pre-symptomatic/asymptomatic’ (257 sequences). If the patient status was ‘Acute upper respiratory infection, unspecified’, ‘Death’, ‘Deceased’, ‘Hospitalized (Critical)’, ‘Hospitalized/Deceased’, ‘Hospitalized, deceased’, ‘Hospitalized in ICU’, ‘Hospitalized (Intensive care unit)’, ‘Hospitalized (Mild)”, ‘Hospitalized (Moderate)’, ‘Hospitalized, oxygenotherapy, diarrhoea’, ‘Hospitalized (Severe)’, ‘Hospsitalized, ICU, fully recovered’, ‘ICD-10 Disease: J00-J06 Acute upper respiratory infections’, ‘ICD-10 Disease: J06.9 Acute upper respiratory infection, unspecified’, ‘ICD-10 Disease: J18.1 Lobar pneumonia, unspecified organism’, ‘ICD-10 Disease: J18.9 Pneumonia, unspecified organism’, ‘ICU’, ‘ICU; Serious’, ‘Intensive Care Unit’, ‘Live, mild symptoms, at home’, ‘Mild’, ‘Mild, at home’, ‘Mild case’, ‘Mild clinical signs without hospitalization’, ‘Mild clinical signs without hospitalization. Diarrhoea’, ‘Mild clinical signs without hospitalization. Distorted ability to smell’, ‘Mild clinical signs without hospitalization. Distorted ability to smell and taste’, ‘Mild clinical signs without hospitalization. Distorted ability to taste’, ‘Mild symptoms (fever, cardiovascular disorders)’, ‘Mild symptoms inpatient for observation’, ‘Moderate/Outpatient’, ‘Paucisymptomatic’, ‘Severe/ICU’, ‘Severe’, ‘Symptomatic’ or ‘Symptoms indicative of upper respiratory infection’, we annotated them as ‘symptomatic’ (10 172 sequences). Sequences that were not generated from original clinical samples or human samples were excluded (three asymptomatic samples and 10 symptomatic samples), leaving 10 416 sequences in the whole dataset. Virus genomes associated with symptomatic Japanese cases were subsampled to reduce the data redundancy (see main text). Fifteen sequences with unusually high sequence diversity were also removed. In total, our dataset comprised 3021 sequences, 252 of which were associated with pre-symptomatic/asymptomatic infections, while 2769 sequences were associated with symptomatic cases. Taxonomic and geographical distributions of the sequences can be found in Tables S3 and S4, respectively.

### Phylogenetic reconstruction

A manually curated full-length multiple sequence alignment of the 3021 SARS-CoV-2 genomes was constructed. Potential recombination within the alignment was checked by using the Phi test implemented in SplitsTree4 [26], but no evidence was found (*P*=0.91). A maximum-likelihood phylogeny was estimated by using IQ-TREE [27]. ModelFinder [28] determined the general time reversible model (GTR) with empirical base frequencies (+F) and the 5-discrete-rate-category FreeRate model (+R5) to be the best-fit nucleotide substitution model under the Bayesian information criterion, and was used for tree reconstruction. Bootstrap clade support was computed based on 1000 pseudoreplicate datasets with ultrafast bootstrap approximation. The terminal branch leading to sample EPI_ISL_407976 was determined as a suitable root placement by comparing the estimated tree with the global audacity tree from the GISAID database. The maximum-likelihood tree in Newick format can be found in Data S1.

### Genetic variation detection

Polymorphic sites with less than 50 % ambiguous bases and aggregated minor allele frequencies of more than 5 % of the collected sequences were identified. For initial screening of candidate sites, genetic variations present in the reference genome (RefSeq accession number: NC_045512.2) were considered ‘reference’ variations, or otherwise as ‘non-reference’ variations, and for each position, a binary profile of genetic variations was constructed. We believed this was reasonable as all positions had only one major alternative variant, with the frequencies of the third most common allele being less than 1 % for all sites except one (Table 1). Uncertainty coefficients were computed for all pairs of positions using the *UncertCoef* function in R (Table S5). Pairs with scores of >0.90 (out of 1) were grouped together as sites with co-varying variants, and were analysed as strongly linked loci. Likewise, for each set of strongly linked loci, a sequence was considered to be a ‘reference’ variant if all of its genetic variations on the sites were those present in the reference genome, and otherwise as a ‘non-reference’ variant.

**Table 1.** SARS-CoV-2 polymorphic sites under the study Twenty-six polymorphic sites with <50 % ambiguous bases and aggregated minor allele frequencies of >5 % of the collected sequences were analysed in our analyses. Sites with co-occurring variants, i.e. those with pairwise uncertainty coefficients of >0.90 (Table S5), were grouped together for analysis.

Nucleotide position*	Nucleotide grouping*	Reference variant [frequency (%)]*	Alternative variant [frequency (%)]	Gene location	Substitution type	Amino acid change†
313	313	C(70.18)	T(29.66), N(0.1), –(0.03), Y(0.03)	ORF1ab:NSP1	Synonymous	L16
1059	1059	C(91.43)	T(8.47), Y(0.07), N(0.03)	ORF1ab:NSP2	Non-synonymous	T85I
3037	3037	C(17.71)	T(82.16), N(0.07), Y(0.07)	ORF1ab:NSP3	Synonymous	F106
11 083	11 083	G(92.62)	T(6.79), N(0.56), K(0.03)	ORF1ab:NSP6	Non-synonymous	L37F
18 877	18 877	C(94.21)	T(5.76), N(0.03)	ORF1ab:NSP14	Synonymous	L280
20 268	20 268	A(93.02)	G(6.59), N(0.4)	ORF1ab:NSP15	Synonymous	L216
23 403	23 403	A(18.21)	G(81.53), N(0.2), R(0.07)	S	Non-synonymous	D614G
25 563	25 563	G(83.88)	T(14.96), C(1.06), N(0.1)	ORF3a	Non-synonymous	Q57H
26 730	26 730	G(94.64)	A(5.1), T(0.13), C(0.07), N(0.07)	M	Non-synonymous	V70I, V70F, V70L
26 735	26 735	C(94.87)	T(5.06), N(0.07)	M	Synonymous	Y71
28 975	28 975	G(92.98)	T(6.29), C(0.53), N(0.2)	N	Non-synonymous	M234I
29 692	29 692	G(92.65)	T(5.66), –(1.42), N(0.26)	3′UTR	Non-coding	–
241	241/14 408	C(16.95)	T(82.75), G(0.13), A(0.07), Y(0.07), –(0.03)	5′UTR	Non-coding	–
14 408		C(17.28)	T(82.36), Y(0.2), N(0.17)	ORF1ab:RDRP	Non-synonymous	P323L
8782	8782/28 144	C(92.98)	T(6.92), N(0.1)	ORF1ab:NSP4	Synonymous	S76
28 144		T(93.25)	C(6.75)	ORF8	Non-synonymous	L84S
18 167	18167/21 518	C(94.14)	T(5.79), A(0.07)	ORF1ab:NSP14	Non-synonymous	P43L, P43H
21 518		G(93.88)	T(5.83), N(0.3)	ORF1ab:NSP16	Non-synonymous	R287I
28 881	28 881/28 882/28 883	G(49.75)	A(49.85), N(0.3), R(0.1)	N	Non-synonymous	R203K, K204R
28 882		G(49.95)	A(49.65), N(0.26), R(0.1), T(0.03)			
28 883		G(50.05)	C(49.59), N(0.26), S(0.1)			
4346	4346/9286/10 376/14 708/28 725	T(94.24)	C(5.76)	ORF1ab:NSP3	Non-synonymous	S543P
9286		C(94.11)	T(5.79), N(0.1)	ORF1ab:NSP4	Synonymous	N244
10 376		C(93.88)	T(6.06), N(0.07)	ORF1ab:NSP5	Non-synonymous	P108S
14 708		C(94.17)	T(5.79), N(0.03)	ORF1ab:RDRP	Non-synonymous	A423V
28 725		C(94.11)	T(5.86), N(0.03)	N	Non-synonymous	P151L

*With respect to the reference SARS-CoV-2 genome (RefSeq accession number: NC_045512.2).

†Reported for non-ambiguous base changes only.

### Phylogenetic-based approach to GWAS

TreeWAS [29] was used for initial screening of SARS-CoV-2 genetic variations that might be associated with COVID-19 pathogenicity, defined as two discrete traits: ‘symptomatic’ and ‘pre-symptomatic/asymptomatic’, conditioning on the estimated virus phylogeny. Three separate tests of association were performed: the ‘terminal’, ‘simultaneous’ and ‘subsequent’ tests. Analysis-wide and separate Bonferroni multiple-testing corrections were applied. Ancestral states of both genetic and phenotypic states were inferred by using the TreeWAS software package [29] under the maximum-parsimony framework. The analysis was applied to all of the 1000 trees in the bootstrap tree distribution obtained from the phylogenetic analysis described above to account for phylogenetic uncertainty. In each test, 1000 genetic loci were simulated to estimate the null distributions of association scores, fixing the tree topology and the distribution of phenotypic states but reassigning genetic substitutions to new branches. The direction of association was determined based on crude odds ratios, estimated using the *oddsratio.wald* function, implemented in the R software package *epitools*.

### Examination of the undetermined/ambiguous base found at site 11083

Our initial screening suggested that genetic variations at site 11 083 are associated with COVID-19 pathogenicity, and examination revealed that 18 sequences had either an undetermined (N) or ambiguous (K) base at the position (see Results). To examine the nature of the genetic variations at this site, we were able to obtain raw sequencing data for 14 of them, and mapped them against the reference genome (NC_045512.2) using *bwa* [30] and *samtools* [31]. Crude read mapping depths and base/indel calling statistics were computed by using *Integrative Genomics Viewer* [32] (Fig. S1).

### Generalized linear mixed model fitting

**Fig. 1.**
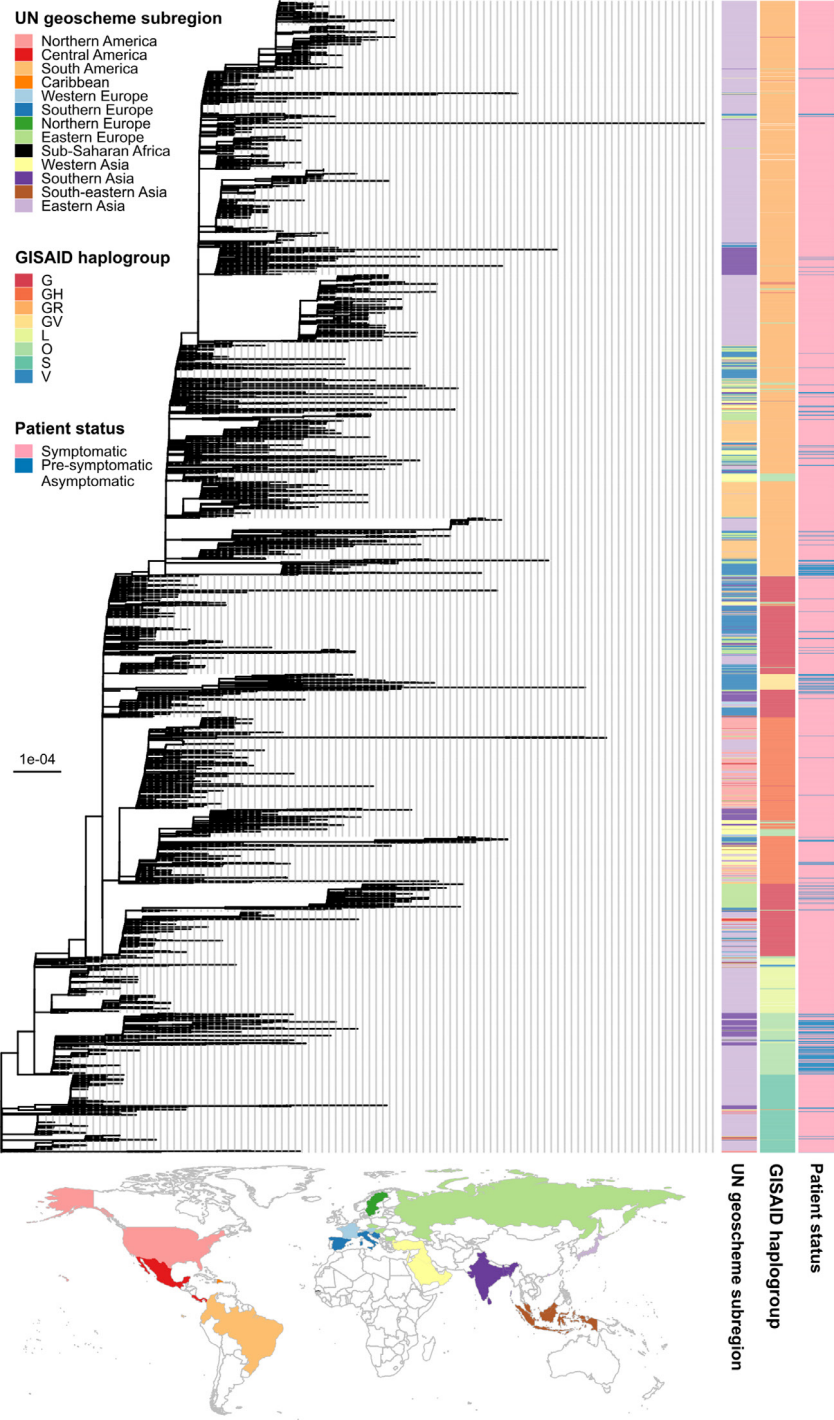
SARS-CoV-2 phylogeny. The tree was estimated under the maximum-likelihood framework implemented in IQ-TREE2 [27] based on a manually curated alignment of 3021 full-length SARS-CoV-2 genomes. Potential recombination within the alignment was checked by using the Phi test implemented in SplitsTree4 [26], but no evidence was found (*P*=0.91). The best-fit nucleotide substitution model was determined to be GTR+F+R5 (the general time reversible model+empirical base frequencies+the 5-discrete-rate-category FreeRate model) by ModelFinder [28] under the Bayesian information criterion and was used for tree reconstruction. We compared our tree with the global tree obtained from GISAID, and determined the terminal branch leading to sample EPI_ISL_407976 as a suitable location for root placement. Bar, substitutions per site. The tree file in Newick format with bootstrap clade-support values, computed based on 1000 bootstrap trees, can be found in Data S1. The three columns on the right indicate the United Nations (UN) geoscheme subregion, the GISAID haplogroup assignment and the patient status of the sequences, respectively (see keys). The World map below the tree shows the countries from which the sequences were sampled, colored according to the UN geoscheme subregions.

To further test the association detected by the initial screening, we fitted various binomial generalized linear mixed models to the data. In addition to the patient status and virus genetic variations, this analysis also incorporated patient age, gender and sequence country of origin, as well as virus phylogenetic structure to the models. The virus phylogenetic structure was simply the variance/co-variance matrix of the estimated phylogeny (Fig. 1), standardized to have a determinant of 1. The effects of the virus genetic mutation, patient age and gender were treated as fixed effects, while the effects of virus phylogenetic structure and patient country were treated as random effects. Four models were examined in total (see Table S6 for model specifications), and were fitted to the data by using the *relmatGLmer* function, implemented in the *lme4qtl* R package [33]. The *anova* function was used to perform the likelihood ratio test to identify the best-fit model. The estimated parameter values can be found in Table S7.

## Results

### SARS-CoV-2 genome sequences with patient status

SARS-CoV-2 genomes with patient status data were retrieved from the GISAID database [25] on 28 December 2020. At the time of the study, 18 705 genome sequences were full-length/almost full-length (>29 000 nt), and had patient status information (Table S1). The metadata of the sequences can be found in Table S2. Of the 18 705 sequences, 10 416 sequences were generated from original clinical samples, and had patient statuses that could be determined as either ‘pre-symptomatic/asymptomatic’ (254 sequences) or ‘symptomatic’ (10 162 sequences). See the Methods section for more details about patient grouping. Further inspection revealed that the majority of the sequences associated with symptomatic infections were from Japan (8776/10 162=86.36 %), and they were highly redundant – according to the classification scheme described previously [34] and the GISAID haplogroup assignment; 8136/10 162 (80.06 %) of all symptomatic sequences comprised just five distinct lineages sampled from Japan, namely B.1.1.284 haplogroup GR (*N*=4005, 39.41%), B.1.1 haplogroup GR (*N*=2612, 25.70%), B.1.1.214 haplogroup GR (*N*=1020, 10.04%), B.1.1.48 haplogroup GR (*N*=343, 3.38%) and A haplogroup S (*N*=150, 1.48%). We thus randomly subsampled the Japanese data so that there were at most 150 sequences from each of these lineages. Preliminary phylogenetic analysis showed that 15 sequences had unusually long terminal branches and had unusually high sequence diversities (>9×10^−4^ mutations per site) compared to the reference SARS-CoV-2 genome (RefSeq accession number: NC_045512.2), probably due to primer contamination. Thirteen were associated with symptomatic cases, while two were obtained from pre-symptomatic/asymptomatic cases, and they were removed from the dataset. In total, our curated dataset comprised 3021 sequences, 252 of which were associated with pre-symptomatic/asymptomatic infections while 2769 sequences were associated with symptomatic infections.

The curated dataset covered a wide diversity of SARS-CoV-2, comprising 148 distinct viral lineages and eight GISAID haplogroups (Table S3) sampled from 36 countries/territories and 13 United Nations (UN) geoscheme subregions (Table S4). Fig. 1 shows a maximum-likelihood phylogeny estimated from the SARS-CoV-2 genomes collected. We found that sequences of different geographical origins often clustered together phylogenetically, indicating substantial cross-country and cross-continent transmissions. Viruses of the same GISAID haplogroups tended to form distinct and tight clusters of sequences in the tree, with the exception of haplogroup G. Haplogroup G was estimated to be a paraphyletic group basal to haplogroups GR, GV and GH. This phylogenetic pattern is expected however, since the haplotype defining haplogroup G is parental/a subset of those defining haplogroups GR, GV and GH [35].

The distribution of SARS-CoV-2 genomes associated with pre-symptomatic/asymptomatic and symptomatic infections were significantly different among lineages [χ^2^ test: χ^2^ score=1094.3, degrees of freedom (d.f.)=147, *P*<2.2×10^−16^) and GISAID haplogroups (χ^2^ test: χ^2^ score=499.22, d.f.=7, *P*<2.2×10^−16^) as well as among countries/territories (χ^2^ test: χ^2^ score=406.33, d.f.=35, *P*<2.2×10^−16^) and broader geographical regions (χ^2^ test: χ^2^ score=230.17, d.f.=12, *P*<2.2×10^−16^). The majority of the genomes associated with pre-symptomatic/asymptomatic cases were from the Czech Republic (34/252=13.49 %), India (52/252=20.63 %), Italy (69/252=27.38 %), Japan (61/252=24.21 %) and Turkey (21/252=8.33 %), while genomes associated with symptomatic cases were from around the globe (Table S4).

### Identification of polymorphic sites

Twenty-six polymorphic sites with less than 50 % ambiguous bases and aggregated minor allele frequencies of more than 5 % of the collected sequences were considered in our analyses, corresponding to sites 241, 313, 1059, 3037, 4346, 8782, 9286, 10 376, 11 083, 14 408, 14 708, 18 167, 18 877, 20 268, 21 518, 23 403, 25 563, 26 730, 26 735, 28 144, 28 725, 28 881, 28 882, 28 883, 28 975 and 29 692 in the reference SARS-CoV-2 genome (RefSeq accession number: NC_045512.2) (Table 1). Of these 26 sites, two were in non-coding regions, seven harbored synonymous changes and the rest harbored non-synonymous changes (Table 1). Analyses have shown that synonymous changes in viruses might not be neutral [36, 37], and since there are many ways that a pathogen and its host could interact, including through protein–protein interactions, protein–nucleotide interactions or even nucleotide–nucleotide interactions, to be as inclusive and as hypothesis-free as possible, we included all of these sites in our analyses.

**Fig. 2.**
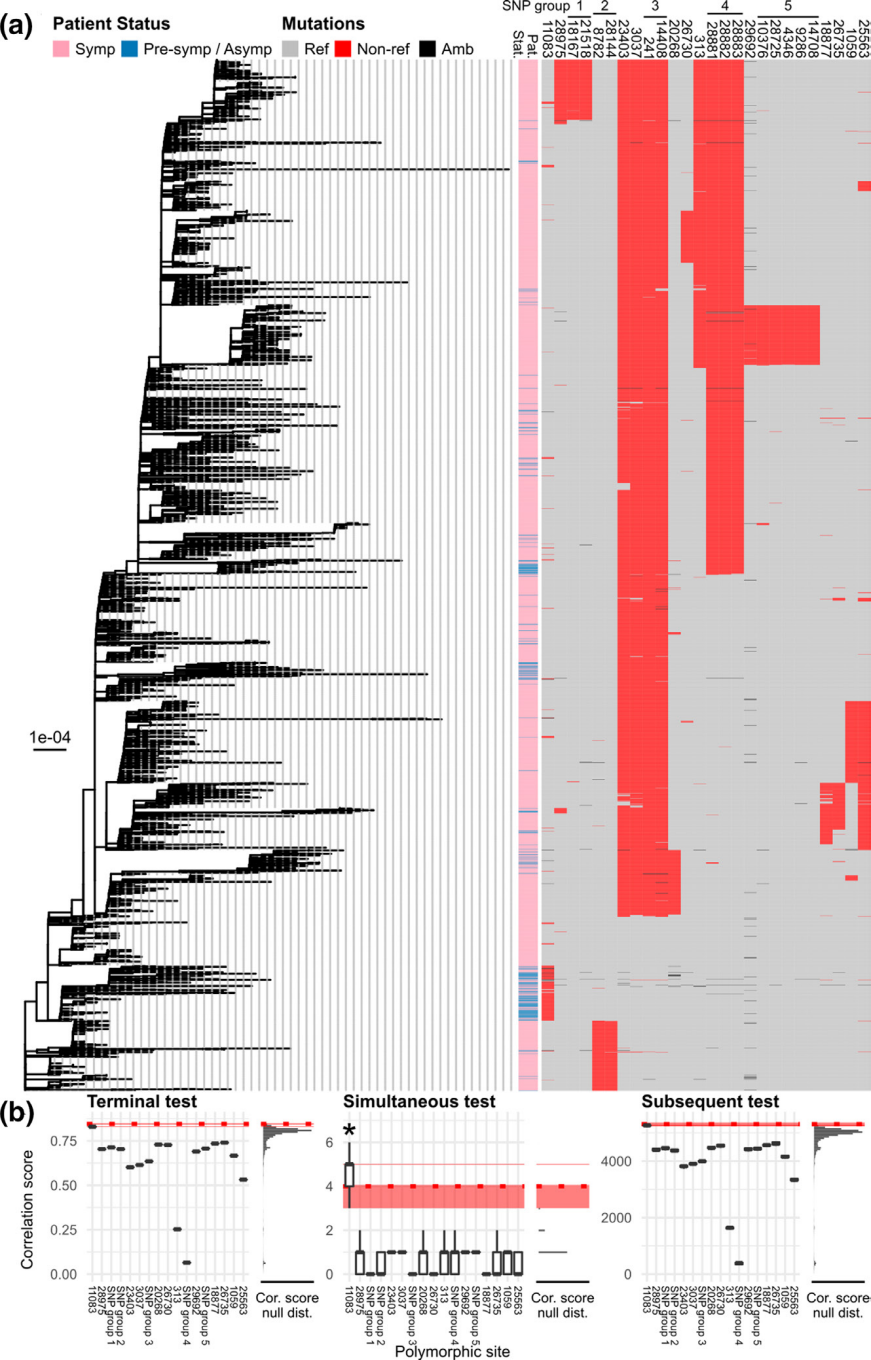
Screening for candidate sites with genetic variations associated with COVID-19 pathogenicity by using TreeWAS [29]. (**a**) Maximum-likelihood tree of SARS-CoV-2 (as shown in Fig. 1) is shown on the left. Bar, substitutions per site. The viruses’ patient status (pat. stat.) and mutational profiles of the 26 polymorphic sites investigated (Table 1) are shown on the right (see keys for details). Sites determined as strongly linked loci are indicated with black horizontal bars and numbers on the top. (**b**) Three separate tests of genotype–phenotype association implemented in the software TreeWAS [29] were performed, namely ‘Terminal’ (left), ‘Simultaneous’ (middle) and ‘Subsequent’ tests (right) with Bonferroni multiple-testing correction (adjusted *P* value threshold=5 %/17 sets of polymorphic sites analysed=0.294 %). To account for phylogenetic uncertainty, the tests were applied to the entire distribution of the 1000 bootstrap trees to obtain the distributions of correlation scores and null scores (Cor. score null dist.). The horizontal red strips indicate the 95 % highest density intervals of the score cut-offs obtained from the 1000 bootstrap analyses. The horizontal red dotted lines indicate the score cut-off obtained from the maximum-likelihood tree analysis. All tests revealed that site 11 083 had the highest scores (horizontal red solid lines). Simultaneous tests suggested that site 11 083 was the only site with genetic variations significantly associated with COVID-19 patient status (marked with an asterisk, positive bootstrap testing rate=58.5 %), while the other two tests did not detect significant signals.

For each position, a binary profile of genetic variations (reference variation vs. non-reference variation) was constructed, and pairwise uncertainty coefficients (*U*) were computed (Table S5). We considered pairs with *U*>0.90 (out of 1) to be sites with co-occurring variations. Five sets of co-varying sites were detected under this criterion, namely (i) sites 241 and 14 408, (ii) sites 8782 and 28 144, (iii) sites 18 167 and 21 518, (iv) sites 28881, 28 882 and 28 883, and (v) sites 4346, 9286, 10376 14 708 and 28 725. Indeed, genetic variations of these five sets of sites were phylogenetically highly correlated, as could been seen pictorially in Fig. 2a, and they were thus analysed together in subsequent analyses as strongly linked sets of loci. Binary profiles of these linked loci were constructed in a similar manner (reference variant vs. non-reference variant). In total, 17 sets of polymorphic sites were analysed.

### Initial screening for candidate polymorphic sites with genetic variations associated with COVID-19 pathogenicity

To identify genetic mutations associated with COVID-19 pathogenicity, we first screened for potential candidates by using a phylogenetic-based approach implemented in TreeWAS [29], which was designed specifically to accommodate the great diversity of virus (and bacterial) genomes.

Three measurements of association between the observed patient status and virus genotype were computed: ‘terminal score’, ‘simultaneous score’ and ‘subsequent score’. The ‘terminal score’ indicates the degree of sample-wide association across the phylogeny’s leaves, the ‘simultaneous score’ is the number of times that the examined phenotype and genotype change in parallel together, and the ‘subsequent score’ measures the total tree length that the genotype and investigated phenotype are found to co-exist [29]. Ancestral states of both genetic and phenotypic data were inferred by using TreeWAS [29] under the maximum-parsimony principle. For each test, Bonferroni multiple-testing correction was applied (adjusted *P* value threshold=5 %/17 sets of polymorphic sites analysed=0.294 %). Three-test wide Bonferroni multiple-testing correction was also applied (adjusted *P* value threshold=5 %/17/3=0.098 %). To account for phylogenetic uncertainty, the analysis was applied to the entire distribution of the 1000 bootstrap trees.

Our results showed that site 11 083 had the highest association scores in all of the three tests (Fig. 2b). Conditioning on the maximum-likelihood tree (Fig. 2a), and under both analysis-wide and separate Bonferroni multiple-testing corrections, the simultaneous test detected the genomic position 11 083 as the only site with variations significantly associated with COVID-19 patient status, while terminal and subsequent tests did not detect any significant signals, although the scores for site 11 083 were very close to the thresholds. Analyses of bootstrap trees showed that site 11 083 was detected as positive by the simultaneous test 37.6 and 58.5 % of the times under the analysis-wide and separate Bonferroni multiple-testing correction, respectively. These relatively low bootstrap positive rates indicated substantial phylogenetic uncertainty, which perhaps was not too surprising given that the sequences were sampled from the same pandemic and were quite similar. These rates, nonetheless, were still much greater than expected had the scores been truly randomly sampled from the null distribution, in which there was no phylogenetic signal or genetic–phenotype association in the sequence data at all (0.098 and 0.294 % per site per test, respectively). Biologically, these results support that no single site with genetic variations could fully explain the distinctions between pre-symptomatic/asymptomatic and symptomatic SARS-CoV-2 infections, but mutations at site 11 083 may cause changes in the disease symptoms through some complex complementary pathways involving many host and virus components.

### Genetic variations at site 11083 and the direction of association

Two major nucleotide variations were observed at genomic position 11 083, namely thymine (T11 083, 205/3021=6.78 %) and guanine (G11 083, 2798/3021=92.62 %). The reference genome (RefSeq accession number: NC_045512.2), which is one of the earliest genomes from the COVID-19 outbreak in Wuhan, was identified as a G11 083 variant (Table 1). One of the closest relatives of SAR-CoV-2 currently known is the bat coronavirus RaTG13 (GenBank accession number: MN996532.2) [28], and it also has a G at the homologous position, suggesting that the G11 083 variant is the wild-type/ancestral state while the T11 083 variant is a mutant variant. The rest (18 sequences, 18/3021=0.60 %) had undetermined nucleotides at this site (Table 2).

**Table 2.** GISAID sequences with ambiguous bases at position 11 083 The sequence NC_045512.2 is included as the reference sequence of the original strain. Ambiguous bases are in bold type. The base at position 11 083 is underlined.

Accession no.	Country	Lineage – GISAID haplogroup	Patient status	Sequence (11073–11093)
*Reference sequence*				
NC_045512.2	China	B – L	Symptomatic	TCTTTTTTTTGTATGAAAATG
*GISAID sequences with an ambiguous base at position 11 083*				
EPI_ISL_454602	Croatia	B.1.1 – GR	Pre-symptomatic/Asymptomatic	TCTTTTTTTT **N** TATGAAAATG
EPI_ISL_539777	Czech Republic	B.1 – GH	Pre-symptomatic/Asymptomatic	TCTTTTTTTT **K** TATGAAAATG
EPI_ISL_626570	Czech Republic	B.1 – GH	Symptomatic	TCTTTTTTT**NN** TATGAAAATG
EPI_ISL_626613	Czech Republic	B.1.258 – G	Symptomatic	TCTTTTTTT**NN** TATGAAAATG
EPI_ISL_437454	India	B.6 – O	Symptomatic	TCTTTTTTTT **N** TATGAAAATG
EPI_ISL_479520	India	B.6 – O	Pre-symptomatic/Asymptomatic	TCTTTTTTTT **N** TATGAAAATG
EPI_ISL_436137	India	B.6 – O	Pre-symptomatic/Asymptomatic	TCTTTTTTTT **N** TATGAAAATG
EPI_ISL_436140	India	B.1.80 – G	Pre-symptomatic/Asymptomatic	TCTTTTTTTT **N** TATGAAAATG
EPI_ISL_436141	India	B.1.80 – G	Pre-symptomatic/Asymptomatic	TCTTTTTTTT **N** TATGAAAATG
EPI_ISL_436156	India	B.6 – O	Pre-symptomatic/Asymptomatic	TCTTTTTTTT **N** TATGAAAATG
EPI_ISL_436157	India	B.6 – O	Pre-symptomatic/Asymptomatic	TCTTTTTTTT **N** TATGAAAATG
EPI_ISL_486386	India	B.6 – O	Pre-symptomatic/Asymptomatic	TCTTTTTTT**NN** TATGAAAATG
EPI_ISL_486394	India	B.6 – O	Pre-symptomatic/Asymptomatic	TCTTTTTTTT **N** TATGAAAATG
EPI_ISL_486403	India	B.6 – O	Pre-symptomatic/Asymptomatic	TCTTTTTTT**NN** TATGAAAATG
EPI_ISL_486407	India	B.1 – S	Pre-symptomatic/Asymptomatic	TCTTTTTTTT **N** TATGAAAATG
EPI_ISL_486384	India	B.1 – O	Symptomatic	TCT**NNNNNNNNNNNNNNNNNN**
EPI_ISL_447776	Colombia	B.1 – GH	Symptomatic	**NNNNNNNNNNNNNNNNNNNNN**
EPI_ISL_447801	Colombia	B.1.5 – G	Symptomatic	**NNNNNNNNNNNNNNNNNNNNN**

Across the 2769 sequences associated with symptomatic cases, 95.88 % (2655 sequences) were found to be the wild-type G11 083 variants, while only 3.90 % (108 sequences) were found to be the mutant T11 083 variants. By contrast, of the 252 sequences associated with pre-symptomatic/asymptomatic infections, only 56.75 %(143 sequences) were found to be the wild-type G11 083 variants, while 38.49 % (97 sequences) were the T11 083 mutant variants. Based on this dataset, the crude odds ratio for causing a symptomatic infection of the mutant T11 083 variant compared to the reference G11 083 variant was estimated to be 0.060 by the Wald method (95 % confidence interval=0.043–0.083; Fisher’s exact test *P*-value=2.11×10^−58^). The association was robust to the inclusion of the Japanese sequences excluded from the dataset due to data redundancy (crude odds ratio=0.037; 95 % confidence interval=0.028–0.050; Fisher’s exact test *P*-value=1.52×10^−82^). Altogether, these results suggested that the 11 083G>T mutation is significantly associated with pre-symptomatic/asymptomatic infection.

Regarding the 18 sequences with undetermined or ambiguous bases at position 11 083 (Table 2), further inspection revealed that three of them had a very long stretch of ‘N’s spanning across the position, suggesting genuine sequencing failures. Fourteen sequences, however, either had only one or two N bases placed precisely at, or spanning across, site 11 083 amidst a long stretch of the T homopolymer, and one sequence had a K ambiguous base (either T or G) at the position. Among these 15 sequences, we found that most of them were from pre-symptomatic/asymptomatic cases (12/15=80 %), and remarkably, they appeared to have an even stronger association with pre-symptomatic/asymptomatic infections compared to the T11 083 variant (crude odds ratio=0.225; 95 % confidence interval=0.062–0.819; Fisher’s exact test *P*-value=0.016). In addition, most of these sequences belonged to lineage B.6 (8/15=53.33 %), which is the lineage that has a very high ratio of T11 083 to G11 083 variants (47 : 3 sequences).

It is well known that a long homopolymer stretch is difficult to sequence, and is prone to indel sequencing errors [38, 39], in particular deletions [40]. Coupled with the fact that site 11 083 is the only polymorphic site amidst the long homopolymer stretch, if such a deletion error were to occur in a T11 083 variant, it would be natural that a sequence aligner would place the gaps spanning the position to maximize the alignment score. Some of these sequences might also be a result of mixed viral populations – indeed, one of these sequences was reported to have an ambiguous base K at site 11 083, indicating that the original sample contained both the T11 083 and G11 083 variants. Alternatively, some of them might genuinely be the G11 083 (or other) variant, but sequencing errors just happened to occur precisely at site 11 083, resulting in undetermined bases. Among these three scenarios, we believed that the last scenario was the most unlikely.

Of these 18 sequences, we were able to obtain raw sequence data for 14, and mapped them against the reference genome to examine the underlying nature of the genetic variations at this site (Fig. S1). We were able to confirm that, among the three sequences with the long stretches of Ns, one was indeed a result of complete sequencing failure, and for the rest, it was often the case that majority of the raw sequence data (>50 %) would support a deletion at the site. However, considering the remaining reads that resulted in base calling, all of them were either Ts or Ks, and none were Gs. Combining this information, we therefore deemed reasonable to group the remaining 15 sequences with other T11 083 variants for further analyses.

### Generalized linear mixed model-based method

One of the limitations of TreeWAS [29] was that, while it directly uses a pathogen’s phylogenetic tree to compute genotype–phenotype association scores, it could not explicitly account for multiple host and virus covariates and confounding factors at the same time. Patient age, gender and country data were available from the GISAID database for some of the sampled sequences, allowing us to further test the association detected by TreeWAS [29], accounting for the effects of these factors. Indeed, various studies have suggested that age and gender may be correlated with the disease severity [13–18], and the incidence of COVID-19 does vary from one country to another [6].

To examine if the association detected by TreeWAS [29] was robust to the inclusion of other covariates and confounding factors, we fitted four binomial generalized linear mixed models to the data (Table S6). In all of the four models, the effects of the mutation 11 083G>T, patient gender and age on disease outcome were treated as fixed effects, while the effects of country sampling and virus phylogenetic relatedness were considered random effects. In the most complex model, each individual virus and county was allowed to have varying baseline chances of symptom development while accounting for virus phylogenetic structure, and the effects of the mutation 11 083G>T, gender and age may also vary from country to country (Table S6; M1). In the second most complex model, each individual virus and country may still have varying baseline chances of symptom development adjusted for virus phylogenetic structure, but the effects of the mutation, gender, and age were ‘fixed’ (i.e. do not vary randomly) across countries (Table S6; M2). In the other two models, one excluded the effect of country sampling (Table S6; M3), and the other excluded the effect of individual virus and phylogenetic structure (Table S6; M4). Sequences with missing patient gender and/or age values were excluded from the model fittings, leaving 1546 sequences in the whole dataset. We also noted that sequence availability varied considerably among countries; most of the countries had fewer than ten sequences in each disease category (Table S4), and this might cause the model fittings and parameter estimates to be unreliable and have unnecessarily high uncertainty. We thus also performed the same analysis on a subsample dataset removing sequences from countries with fewer than ten samples in each disease category. Excluding those with missing patient gender and/or age values, the curated subsample dataset comprised 859 sequences from five countries, namely Czech Republic, India, Italy, Japan and Turkey.

**Fig. 3.**
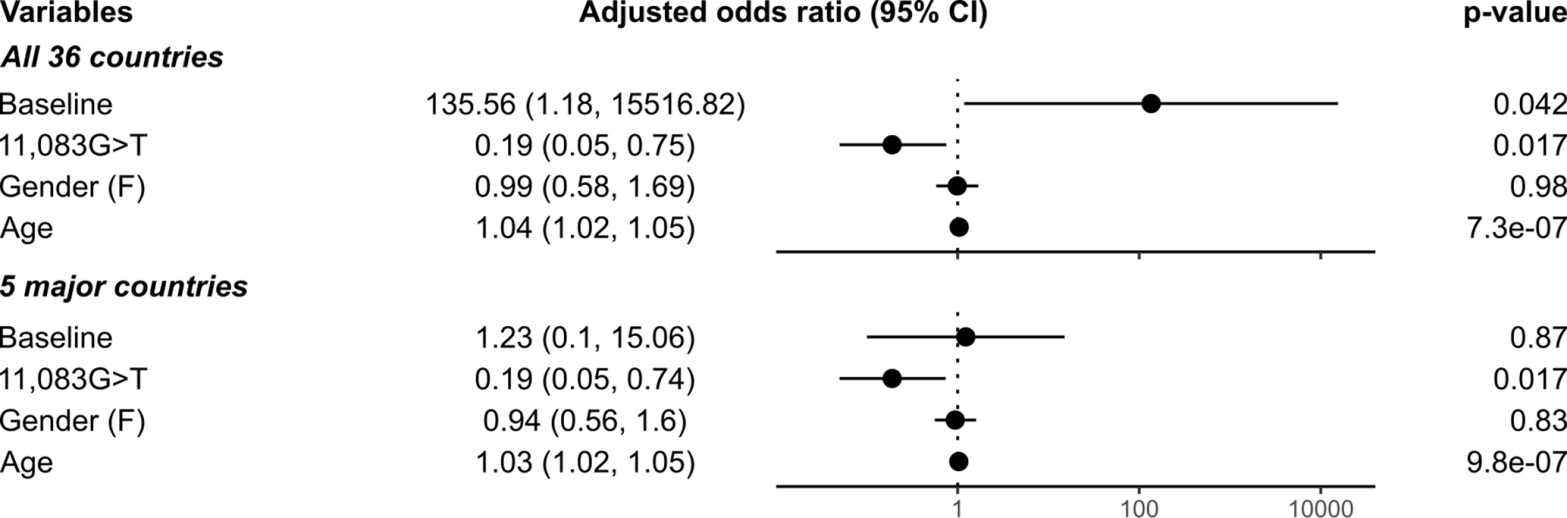
Adjusted odds ratios and 95 % confidence intervals of various potential risk factors for COVID-19 symptom development. The values were estimated based on the best-fit binomial generalized linear-mixed model M2, in which the effects of the mutation 11 083G>T, patient gender and age on the disease outcome were treated as fixed effects, and the effects of country sampling and virus phylogenetic relatedness were considered random effects. The model allowed each individual virus and country to have varying baseline chances of symptom development while adjusting for the virus phylogenetic structure. See model specification in Table S6 and estimated parameter values in Table S7.

Based on the analysis of the whole dataset, we found that M1 did not significantly better fit the data than M2 (Table S6; χ^2^ test: χ^2^ score=7.85, d.f.=9, *P*=0.55), supporting that the effects of the mutation 11 083G>T, patient gender and age on COVID-19 pathogenicity do not vary significantly among countries. However, M2 was found to fit the data significantly better than M3 (Table S6; χ^2^ test: χ^2^ score=72.84, d.f.=1, *P*<2.2×10^−16^), suggesting that populations of different counties may have varying baseline chances of developing COVID-19 symptoms. Similarly, M2 was found to better fit than M4 (Table S6; χ^2^ test: χ^2^ score=113.55, d.f.=1, *P*<2.2×10^−16^), suggesting that phylogenetic relatedness among viruses plays a significant role in COVID-19 pathogenicity (i.e. closely related viruses tend to cause the same kind of infection). Estimated parameter values of the best-fit model can be found in Table S7, and their corresponding adjusted odds ratios are given in Fig. 3.

According to the best-fit model M2 (Fig. 3), we found that patient age is positively associated with symptomatic infection (adjusted odds ratio for increasing an age by 1 year=1.04; 95 % confidence interval=1.02–1.05; *P*=7.3×10^−7^), but patient gender does not significantly correlate with the disease outcome (adjusted odds ratio for being female=0.99, 95 % confidence interval=0.58–1.69; *P*=0.98). The baseline odds for developing COVID-19 symptoms when getting infected was estimated to be (just) significantly different from 1 (*P*=4.2×10^−2^), computed to be 135.56 with an extremely large uncertainty (95 % confidence interval=1.18–15 516.82), and the value was estimated to vary substantially among countries (variance=49.66) and also among viruses, although to a much lesser extent (variance=0.38). At face value, this estimate is consistent with the overall baseline chance of developing symptoms being greater than that of not developing symptoms. Nonetheless, it is noteworthy that there might be a tendency for this value to be overestimated, due to the fact that viruses associated with symptomatic infection may be more likely to be sequenced and submitted to the GISAID database than those causing asymptomatic infection given typical healthcare seeking behaviour, making the database systematically biased towards viruses causing symptoms. Thus, this value should be interpreted with care. Nevertheless, taking all of these factors into account, the 11 083G>T mutation was still found to be significantly associated with pre-symptomatic/asymptomatic infection (adjusted odds ratio=0.19, 95 % confidence interval=0.05–0.75; *P*=1.70×10^−2^).

Analysis of the subsample data yielded largely consistent results. M1 was not found to significantly better fit than M2 (Table S6; χ^2^ test: score=12.46, d.f.=9, *P*=0.19), but M2 significantly better fitted the data than M3 (Table S6; χ^2^ test: score=15.49, d.f.=1, *P*=8.31×10^−5^) and M4 (Table S6; χ^2^ test: score=106.12, d.f.=1, *P*<2.2×10^−16^). According to the M2 model (Table S7, and Fig. 3), again, we found that age is positively associated with symptomatic infection (adjusted odds ratio for increasing an age by 1 year=1.03; 95 % confidence interval=1.02–1.05; *P*=9.8×10^−7^), while gender does not significantly correlate with the pathogenicity of the virus (adjusted odds ratio for being female=0.94, 95 % confidence interval=0.56–1.60; *P*=0.83). Unlike the results from the whole dataset analysis, the baseline odds was estimated to be insignificantly different from 1 (odds ratio=1.23, 95 % confidence interval=0.10–15.06; *P*=0.87), but the analysis still suggested that the value does vary considerably among countries (variance=2.04) and among viruses (variance=0.32). The overall strength of the random country-specific effect was estimated to be less than that estimated based on the whole dataset (variance=49.66), probably due to fewer countries being included in this dataset. Nonetheless, again, a significant (negative) effect of the 11 083G>T mutation on SARS-CoV-2 pathogenicity could still be detected (adjusted odds ratio=0.19, 95 % confidence interval=0.05–0.74; *P*=1.74×10^−2^).

## Discussion

An unprecedented number of SARS-CoV-2 genomes have been generated at a rapid rate and made publicly available in near real-time. Millions of sequences of SARS-CoV-2 genomes have been made publicly available on the GISAID database [25], and some sequences have patient status available, as well as patient age, gender and country. This allowed us to investigate viral genetic factors that might be associated with SARS-CoV-2 pathogenicity.

In this study, we performed GWAS analyses on 3021 SARS-CoV-2 genomes, and identified variations at genomic position 11 083 to be associated with SARS-CoV-2 pathogenicity. Specifically, we found that the mutation 11 083G>T is associated with pre-symptomatic/asymptomatic cases (Fig. 3, *P*=1.70–1.74×10^−2^), adjusted for various factors that might also be associated with disease severity, including patient age, gender and country of origin, as well as virus phylogenetic relatedness and structure.

Our analysis detected a positive correlation between age and COVID-19 symptom development, consistent with results from previous studies [13–15]. A significant association between gender and pathogenicity could not be detected by our study. In contrast, several other studies have detected a gender effect on COVID-19 severity. For example, an analysis of a global survey of hospital records suggested that male patients have higher odds of requiring intensive treatment unit admission and death compared to females [18]. A similar trend was observed in the USA, adjusted for various potential covariates including age, race, ethnicity, marital status, insurance type, median income, body mass index, smoking and other 17 comorbidities [17]. It has been suggested that this could be due to differences in social and cultural behaviours between men and women, and differences in the sex hormone influencing, for example, virus entry and priming, immune and inflammatory response, and coagulation and thrombosis diathesis (see [16] for review). The lack of signal in this study could be because we focused on virus pathogenicity, grouping death, severe and mild cases together as symptomatic. Nonetheless, it is of note that there are also studies that did not find a significant association between gender and COVID-19 severity. For example, Raimondi *et al*. found that gender did not play a significant role as an independent predictor of death in an Italian cohort after adjusting for various confounding factors such as age and severity of the disease at hospital presentation [41]. This suggested that such a trend may vary from country to country.

Paralleling this, we found that patient country is, indeed, a significant random effect, suggesting that the baseline chance of COVID-19 symptom development varies from country to country (although country-specific random effects of the virus mutation, patient age and gender are not significant). In part, this may be because different countries may differ in the way they recorded COVID-19 symptoms and/or how the disease surveillances or treatments were performed, resulting in different distributions of pre-symptomatic/asymptomatic vs. symptomatic cases. This result is also consistent with different human populations having varying degrees of susceptibility to the virus infection. Several studies have revealed that background genetic makeup does, indeed, associate with susceptibility to SARS-CoV-2 infection and COVID-19 severity. For example, a study of two case-control cohorts of Italian and Spanish patients identified a 3p21.31 gene cluster on chromosome 3 to be a genetic susceptibility locus in COVID-19 patients, and showed a higher risk in blood group A than in other blood groups, corresponding to the rs657152 variant at locus 9q34.2 on chromosome 9 [42]. A separate analysis of a dataset from the COVID-19 Host Genetics Initiative confirmed the former region to be significantly associated with COVID-19 severity, and determined that the risk variant was inherited from Neanderthals, carried by around 50 % of South Asians and around 16 % of Europeans [43]. There are several more host genetic factors that have been suggested as COVID-19 severity predictors, such as genetic variability in *human leucocyte antigen* genes [21], *major histocompatibility complex class I* genes [44] and *TLR7* gene on chromosome X [45], for example. Different human populations have different genetic backgrounds, and this could potentially partly explain our finding.

A study identified the 11 083G>T mutation to be associated with asymptomatic cases by Fisher’s exact test (*P*=8.45×10^−35^) and examination of Pearson’s correlation coefficient (Pearson correlation=0.61, *P*=5.42×10^−56^) [46]; however, the analysis neither accounted for phylogenetic relatedness among viruses nor potential host factors that might also correlate with COVID-19 severity. In this study, we identified the genetic mutation 11 083G>T to be associated with pre-symptomatic/asymptomatic cases by using a phylogenetic approach, and after correcting for patient gender, age, country of origin and virus phylogenetic relatedness, the association still remained significant, suggesting that this association is robust. An earlier study of SARS-CoV-2 from a Shanghai cohort identified the T11 083 variant to be more prevalent in pre-symptomatic/asymptomatic cases (nine in 91 cases=9.89 %) compared to symptomatic cases (one in 21 cases=4.76 %), but the association was not significant [20]. This could be due to their relatively small data set (*N*=112), and different groupings of the disease outcome (mild symptomatic and pre-symptomatic/asymptomatic cases vs. severe and critical cases), which could potentially mask the effect we observed. Epidemiological data examination showed that, indeed, countries with a high prevalence of the T11 083 variant tended to have lower rates of COVID-19 mortality than those that had low prevalence of the mutation [46], further supporting the association.

This mutation is located in the coding region of non-structural protein 6 (NSP6, nt 10973–11842), coded by ORF1ab (nt 266–21 555). NSP6 is a multi-functional protein, and can be found in many coronaviruses. It has been demonstrated that, located to the endoplasmic reticulum, SARS-CoV NSP6 can form a protein complex with NSP3 and NSP4 to induce double-membrane vesicles, crucial for the formation of the virus replication/transcription complex [47]. Studies of an avian gamma-coronavirus, infectious bronchitis virus, showed that NSP6 can activate formation of autophagosomes [48], but ones with small and restricted sizes [49], which may interfere with the host’s ability to deliver viral components to lysosomes for degradation. A recent study [50] showed that SARS-CoV-2 NSP6 can bind TANK binding kinase 1, and suppress phosphorylation of IFN regulatory factor 3, which in turn antagonizes type I IFN production. The study also showed that the protein can suppress STAT1 and STAT2 phosphorylation, inhibiting type I IFN signalling, which might mitigate the host immune response even further. In addition, the study found that SARS-CoV NSP6 could not inhibit type I IFN signalling, at least not as efficiently as that of SARS-CoV-2, potentially explaining the relatively more delayed disease onset and less systemic and severe clinical manifestations of COVID-19 compared to SARS.

The 11 083G>T mutation confers an amino acid change from leucine (L) to phenylalanine (F) at the 37^th^ position in the NSP6 protein (L37F). *In silico* 3D structure analyses suggested that the mutation may reduce the stability of the protein structure [46, 51], and at the same time may increase the rigidity of the protein, reducing interactions with the endoplasmic reticulum, and interfering with the protein functions in turn [46]. As discussed above, the functions of NSP6 are to antagonize host immune responses, interacting with many virus and host components. Nonetheless, the physical interactions between them are yet to be characterized, and it would be interesting to see if the mutation affects or alters the interactions or not. Our results warrant further experimental confirmations to validate the biological significance of this mutation and its consequences.

The mutation 11 083G>T has occurred independently many times over the course of this on-going pandemic. In fact, this genomic position has been quantified as having one of the highest rates of homoplasy [52]. Some previous studies suggested that this might suggest on-going adaptation/positive selection [51, 53]; however, concrete evidence supporting this notion is lacking [54]. In fact, according to examination of the sequences in the GISAID database, it was reported that the frequencies of the T11 083 variant relative to the wild-type G11 083 variant decreased globally, supporting that the mutation may hinder the virus transmissibility [46]. However, an alternative possibility is that, as the numbers of COVID-19 patients have surged very rapidly and overwhelmed public healthcare systems in many countries around the globe, limited central disease surveillance and medical resources may tend to be allocated to search for and treat patients with severe clinical manifestations of COVID-19. Asymptomatic cases (perhaps with the 11 083G>T mutation) may thus be overlooked and less likely to be tested, and in turn have fewer virus sequences deposited in public databases. Continual surveillance of COVID-19 should monitor this genomic region as it might affect the virus’s pathogenicity and control of COVID-19. These results have potential applications for the development of better, and more informative test kits, potentially allowing for asymptomatic cases to be distinguished from symptomatic ones.

## Supplementary Data

Supplementary material 1
