# Supplementary material for: SARS-CoV-2 genetic variations associated with COVID-19 pathogenicity
Source: Microb Genom. 2021 Dec 6;7(12):000734. doi: 10.1099/mgen.0.000734 (PMC8767342; doi:10.1099/mgen.0.000734)
Supplement: Supplementary material 1 [file mgen-7-0734-s001.pdf]

Supplementary Data 1: The maximum likelihood phylogeny shown in **Figure 1** in the Newick format, with bootstrap clade-support values, computed based on 1,000 bootstrap trees.

(EPI_ISL_513050:0.0001033908,((
(((
EPI_ISL_513051:3.44122e-
05,((EPI_ISL_513053:0,EPI_ISL_512890:0):0,EPI_ISL_428671:0):
0,EPI_ISL_477202:0):2.425e-07,(((((((EPI_ISL_513067:2.425e-07,EPI_ISL_513022:3.44085e-
05)100:2.427e-07,EPI_ISL_732674:0.0003134638)100:3.44083e-
05,(((((((((((((((((((((((((((((((((((EPI_ISL_516974:3.51132e-
05,(EPI_ISL_479505:0.0001389489,(((((((((((((((((((((((((((((((EPI_ISL_479507:2.425e-
07,EPI_ISL_479522:2.425e-07)100:3.47279e-
05,((((((((((((((EPI_ISL_479509:0.0001043557,(((((EPI_ISL_479526:2.425e-07,((EPI_ISL_479538:6.94747e-
05,(((EPI_ISL_524764:2.425e-07,EPI_ISL_524765:3.48315e-
05)100:0.0002091164,EPI_ISL_700039:6.92134e-05)30:2.427e-07,EPI_ISL_699779:3.50048e-
05)30:2.427e-07)30:2.427e-07,EPI_ISL_479549:0.0007012632)30:2.427e-07)30:2.427e-
07,(EPI_ISL_454524:0.0001391092,EPI_ISL_452201:2.425e-07)100:6.94482e-05)30:2.427e-
07,EPI_ISL_452212:3.47207e-05)30:2.427e-07,EPI_ISL_479551:0.0002787421)30:2.427e-
07,(EPI_ISL_452197:0.0001042222,(EPI_ISL_452198:6.94572e-05,EPI_ISL_452200:2.425e-07)39:2.427e-
07)100:6.94479e-05)30:2.427e-07)30:2.427e-07,(EPI_ISL_454522:7.02636e-05,EPI_ISL_452194:2.425e-
07)100:3.47236e-05)30:2.427e-07,EPI_ISL_699926:6.95898e-05)30:2.427e-07,EPI_ISL_479498:3.4813e-
05)30:2.427e-07,EPI_ISL_479529:0.000104206)30:2.427e-07,EPI_ISL_452192:2.425e-07)30:2.427e-
07,EPI_ISL_479499:2.425e-07)30:2.427e-07,EPI_ISL_699970:0.0001043746)30:2.427e-
07,(((((EPI_ISL_479537:0.0001042352,EPI_ISL_479502:3.47415e-05)83:3.47191e-
05,(EPI_ISL_479544:0.0003092574,EPI_ISL_479658:0.0001777075)83:3.84542e-05)36:2.427e-
07,EPI_ISL_479501:0.0001737764)73:3.47299e-05,EPI_ISL_479547:2.425e-07)32:2.427e-
07,EPI_ISL_454550:0.0002120597)55:3.47243e-05)30:2.427e-07,EPI_ISL_452211:3.47246e-
05)30:2.427e-07,(EPI_ISL_479540:0.0001389554,EPI_ISL_452193:2.425e-07)98:3.47198e-05)30:2.427e-
07,EPI_ISL_479533:3.47303e-05)30:2.427e-
07,(EPI_ISL_479530:0.0001393297,EPI_ISL_479550:0.000173894)100:6.9431e-05)30:2.427e-
07,(EPI_ISL_479513:6.9492e-05,EPI_ISL_479521:6.9494e-05)100:3.47231e-05)30:2.427e-07)30:2.427e-
07,EPI_ISL_699737:0.0001043965)30:2.427e-07,EPI_ISL_479512:2.425e-07)30:2.427e-
07,EPI_ISL_452199:6.94979e-05)30:2.427e-07,EPI_ISL_452207:2.425e-07)30:2.427e-
07,EPI_ISL_700066:0.0002087258)30:2.427e-07,EPI_ISL_479525:6.97963e-05)30:2.427e-
07,EPI_ISL_699845:0.0001751199)30:2.427e-
07,((EPI_ISL_454556:0.00052395,EPI_ISL_454521:0.000351006)100:3.077e-
07,EPI_ISL_479548:0.0002087032)100:6.94706e-05)30:2.427e-07,EPI_ISL_479536:3.47325e-
05)30:2.427e-07,EPI_ISL_479552:0.0001739661)30:2.427e-07,EPI_ISL_479543:6.94672e-05)30:2.427e-
07,EPI_ISL_454557:2.425e-07)30:2.427e-07,EPI_ISL_699996:0.0002073205)30:2.427e-
07,EPI_ISL_479534:2.425e-07)30:2.427e-07,EPI_ISL_479541:0.0001042377)30:2.427e-
07,EPI_ISL_479510:0.0001043193)30:2.427e-07,EPI_ISL_479519:2.425e-07)30:2.427e-
07,EPI_ISL_479535:6.95131e-05)30:2.427e-07,EPI_ISL_479532:6.9513e-05)30:2.427e-
07,EPI_ISL_479528:6.9517e-05)30:2.427e-07,EPI_ISL_700118:0.0002088706)30:2.427e-
07,EPI_ISL_479493:3.47271e-05)30:2.427e-07,EPI_ISL_699963:0.0001035784)30:2.427e-
07,EPI_ISL_479517:3.47277e-05)30:2.427e-07,EPI_ISL_479523:3.47262e-05)30:2.427e-

07,EPI_ISL_479514:2.425e-07)30:2.427e-07,EPI_ISL_479531:2.425e-07)30:2.427e-
07,EPI_ISL_699894:0.0001035608)30:2.427e-07)30:2.427e-07)30:3.45178e-
05,((EPI_ISL_480139:3.48648e-
05,(((((((((((((((((((((((((((((EPI_ISL_480194:2.425e-
07,((EPI_ISL_428485:0.0001950345,((((((((((((((((((((((EPI_ISL_689299:2.425e-
07,(EPI_ISL_689238:6.97484e-05,((EPI_ISL_686202:2.425e-07,EPI_ISL_687544:6.97392e-05)30:2.427e-
07,EPI_ISL_686203:2.425e-07)30:2.427e-07)30:2.427e-07)100:3.48102e-05,EPI_ISL_689137:3.48683e-
05)100:3.48147e-05,((((((((((((((((((((EPI_ISL_689270:3.48667e-
05,(((((((EPI_ISL_692509:0,EPI_ISL_686161:0):0,EPI_ISL_692473:0):0,EPI_ISL_686170:0):0,EPI_ISL_69209
3:0):2.425e-07,(EPI_ISL_688875:2.425e-07,EPI_ISL_688579:3.52223e-05)30:2.427e-07)30:2.427e-
07,EPI_ISL_690525:6.97373e-05)30:2.427e-07,EPI_ISL_688896:2.425e-07)30:2.427e-07)30:2.427e-
07,EPI_ISL_688877:6.95592e-05)30:2.427e-07,EPI_ISL_689668:2.425e-07)30:2.427e-
07,EPI_ISL_688697:2.425e-07)30:2.427e-07,EPI_ISL_689304:2.425e-07)30:2.427e-
07,EPI_ISL_692353:2.425e-07)30:2.427e-07,(EPI_ISL_689263:3.47831e-05,EPI_ISL_689352:6.97376e-
05)100:3.47828e-05)30:2.427e-07,(EPI_ISL_686194:0,EPI_ISL_687884:0):2.425e-07)30:2.427e-
07,EPI_ISL_687573:3.47944e-05)30:2.427e-07,EPI_ISL_689177:6.95739e-05)30:2.427e-
07,EPI_ISL_691870:2.425e-07)30:2.427e-07,EPI_ISL_689272:2.425e-07)30:2.427e-
07,(EPI_ISL_688663:2.425e-07,EPI_ISL_688668:2.425e-07)100:3.47851e-05)30:2.427e-
07,EPI_ISL_687899:2.425e-07)30:2.427e-07,(EPI_ISL_686807:2.425e-07,EPI_ISL_687881:2.425e-
07)100:3.47754e-05)30:2.427e-07,EPI_ISL_689986:3.47753e-05)30:2.427e-
07,((((EPI_ISL_689253:2.425e-07,(EPI_ISL_690478:2.425e-07,EPI_ISL_687133:3.48667e-05)30:2.427e-
07)30:2.427e-07,EPI_ISL_688963:6.96718e-05)30:2.427e-07,EPI_ISL_686213:2.425e-07)30:2.427e-
07,EPI_ISL_686325:6.97403e-05)100:3.47754e-05)30:2.427e-07,(EPI_ISL_689464:3.48314e-
05,EPI_ISL_688954:2.425e-07)100:6.95576e-05)30:2.427e-07,EPI_ISL_686838:2.425e-07)30:2.427e-
07,(EPI_ISL_690598:2.425e-07,EPI_ISL_690356:2.425e-07)100:6.96598e-05)30:2.427e-07)30:2.427e-
07,EPI_ISL_689144:3.48667e-05)30:2.427e-07,(EPI_ISL_686162:2.425e-07,EPI_ISL_686183:2.425e-
07)100:3.48657e-05)30:2.427e-07,EPI_ISL_688912:0.0001045286)30:2.427e-
07,((((EPI_ISL_692650:2.425e-07,((EPI_ISL_690560:2.425e-07,EPI_ISL_690561:2.425e-07)30:2.427e-
07,EPI_ISL_690563:2.425e-07)100:0.0001043773)100:6.95673e-
05,EPI_ISL_689309:0.0001046509)99:2.427e-07,EPI_ISL_692406:3.4784e-05)100:3.47888e-
05,EPI_ISL_687505:6.96172e-05)100:3.47751e-05)30:2.427e-
07,(((((((EPI_ISL_689193:0,EPI_ISL_688655:0):0,EPI_ISL_689505:0):0,EPI_ISL_687900:0):0,EPI_ISL_68867
5:0):0,EPI_ISL_687895:0):0,EPI_ISL_692571:0):0,EPI_ISL_689665:0):2.425e-07)30:2.427e-
07,(EPI_ISL_689147:3.48681e-05,(EPI_ISL_689660:2.425e-07,((((((((EPI_ISL_690406:2.425e-
07,((EPI_ISL_690426:2.425e-07,EPI_ISL_689354:2.425e-07)100:6.95752e-05,(EPI_ISL_688713:2.425e-
07,(EPI_ISL_687220:6.9747e-05,((EPI_ISL_687226:2.425e-07,((EPI_ISL_687229:2.425e-
07,EPI_ISL_687247:2.425e-07)30:2.427e-07,EPI_ISL_687233:3.48683e-05)30:2.427e-07)30:2.427e-
07,EPI_ISL_687228:2.425e-07)99:3.4777e-05)30:2.427e-07)30:2.427e-07)30:2.427e-07)30:2.427e-
07,EPI_ISL_689340:3.44841e-05)30:2.427e-07,EPI_ISL_687200:2.425e-07)30:2.427e-
07,EPI_ISL_689329:3.47834e-05)30:2.427e-07,EPI_ISL_688711:2.425e-07)30:2.427e-
07,(EPI_ISL_688614:2.425e-07,EPI_ISL_687160:2.425e-07)100:6.95756e-05)30:2.427e-
07,EPI_ISL_689338:0.0001391742)30:2.427e-07,EPI_ISL_689333:0.0001388534)98:3.44631e-
05)30:2.427e-07)100:3.44632e-05)30:2.427e-07,EPI_ISL_688664:3.48638e-05)30:2.427e-
07,EPI_ISL_689817:3.48267e-05)30:2.427e-07,EPI_ISL_689200:3.48683e-05)30:2.427e-

07,(((EPI_ISL_690212:2.425e-07,(((((((EPI_ISL_690158:3.48666e-05,(((((((EPI_ISL_692732:2.425e-07,(((EPI_ISL_690559:3.48678e-05,(EPI_ISL_690556:2.425e-07,(EPI_ISL_689989:2.425e-07,EPI_ISL_689990:2.425e-07)30:2.427e-07)30:2.427e-07)30:2.427e-07,EPI_ISL_689988:2.425e-07)30:2.427e-07,EPI_ISL_690562:2.425e-07)100:3.47775e-05)30:2.427e-07,(EPI_ISL_692657:2.425e-07,EPI_ISL_688975:0.0001053172)100:6.96809e-05)31:2.427e-07,(EPI_ISL_686525:2.425e-07,EPI_ISL_686530:3.48632e-05)100:6.95751e-05)30:2.427e-07,EPI_ISL_688746:3.48328e-05)30:2.427e-07,EPI_ISL_689948:2.425e-07)100:3.47775e-05,(((((EPI_ISL_689098:6.9749e-05,EPI_ISL_688393:2.425e-07)100:3.48634e-05,EPI_ISL_687099:2.425e-07)100:2.427e-07,EPI_ISL_686226:3.48684e-05)100:3.48679e-05,EPI_ISL_688586:6.9741e-05)100:2.427e-07,((EPI_ISL_686458:6.95597e-05,EPI_ISL_686462:3.48642e-05)30:2.427e-07,EPI_ISL_686459:2.425e-07)100:3.47765e-05)99:3.47809e-05)82:2.427e-07,EPI_ISL_686753:6.96694e-05)30:2.427e-07)30:2.427e-07,((EPI_ISL_690159:2.425e-07,EPI_ISL_688383:2.425e-07)30:2.427e-07,EPI_ISL_688389:2.425e-07)100:3.48323e-05)30:2.427e-07,EPI_ISL_688600:0.0001395442)30:2.427e-07,EPI_ISL_687823:6.97498e-05)30:2.427e-07,EPI_ISL_687153:6.95339e-05)30:2.427e-07,EPI_ISL_687824:2.425e-07)30:2.427e-07,(EPI_ISL_692664:0,EPI_ISL_687838:0):2.425e-07)30:2.427e-07)30:2.427e-07,EPI_ISL_686812:3.48681e-05)30:2.427e-07,EPI_ISL_686781:3.48683e-05)100:3.47589e-05)30:2.427e-07,(((EPI_ISL_689508:3.48635e-05,((EPI_ISL_689511:2.425e-07,EPI_ISL_687529:3.48645e-05)100:3.48636e-05,EPI_ISL_688674:2.425e-07)54:2.427e-07)30:2.427e-07,EPI_ISL_690355:2.425e-07)30:2.427e-07,EPI_ISL_689857:2.425e-07)97:3.47805e-05)30:2.427e-07,((((((((EPI_ISL_691583:0,EPI_ISL_691605:0):2.425e-07,EPI_ISL_691591:2.425e-07)92:2.427e-07,EPI_ISL_692142:3.48332e-05)30:2.427e-07,EPI_ISL_691393:2.425e-07)30:2.427e-07,EPI_ISL_692139:2.425e-07)30:2.427e-07,EPI_ISL_692150:6.9568e-05)30:2.427e-07,EPI_ISL_691760:6.96686e-05)30:2.427e-07,EPI_ISL_691763:2.425e-07)31:2.427e-07,(EPI_ISL_692524:3.48683e-05,EPI_ISL_691761:2.425e-07)97:3.48366e-05)97:3.4776e-05)100:3.44628e-05,(EPI_ISL_689009:2.425e-07,EPI_ISL_686224:3.48646e-05)100:0.0001046274)30:2.427e-07,(EPI_ISL_692360:3.48334e-05,EPI_ISL_692182:2.425e-07)100:0.0001394077)30:2.427e-07,(((((((((((EPI_ISL_686904:6.96915e-05,(EPI_ISL_686913:2.425e-07,EPI_ISL_686433:2.425e-07)30:2.427e-07)30:2.427e-07,(EPI_ISL_686504:3.48647e-05,(EPI_ISL_686497:2.425e-07,EPI_ISL_686499:2.425e-07)100:2.427e-07)100:3.4868e-05)30:2.427e-07,EPI_ISL_686907:2.425e-07)30:2.427e-07,EPI_ISL_692681:3.48681e-05)30:2.427e-07,(EPI_ISL_686905:2.425e-07,EPI_ISL_686923:3.48647e-05)100:3.48672e-05)30:2.427e-07,EPI_ISL_686479:3.48697e-05)30:2.427e-07,EPI_ISL_689429:2.425e-07)30:2.427e-07,EPI_ISL_688305:6.96747e-05)30:2.427e-07,EPI_ISL_688583:6.97487e-05)30:2.427e-07,EPI_ISL_687806:2.425e-07)30:2.427e-07,EPI_ISL_686480:2.425e-07)30:2.427e-07,EPI_ISL_689402:2.425e-07)76:3.47959e-05)30:2.427e-07,(((((((((EPI_ISL_686909:6.96683e-05,EPI_ISL_686658:6.97451e-05)32:2.427e-07,EPI_ISL_686842:6.97347e-05)30:2.427e-07,EPI_ISL_686833:3.48683e-05)30:2.427e-07,EPI_ISL_686841:3.48642e-05)30:2.427e-07,EPI_ISL_687560:0.0001046518)30:2.427e-07,EPI_ISL_689099:2.425e-07)30:2.427e-07,((EPI_ISL_686839:0,EPI_ISL_686440:0):0,EPI_ISL_687673:0):2.425e-07)30:2.427e-07,EPI_ISL_689096:2.425e-07)30:2.427e-07,EPI_ISL_686615:2.425e-07)30:2.427e-07,EPI_ISL_690536:3.48647e-05)100:3.47825e-05)30:2.427e-07,(EPI_ISL_689444:2.425e-07,EPI_ISL_689447:2.425e-07)100:0.0001744236)100:6.89358e-05,EPI_ISL_685866:0.0001392147)30:2.427e-07,EPI_ISL_690649:3.48698e-05)30:2.427e-07)30:2.427e-07,(((EPI_ISL_685233:2.425e-07,EPI_ISL_685235:2.425e-07)99:2.427e-07,EPI_ISL_685236:2.425e-

07)99:2.427e-07,EPI_ISL_685234:2.425e-07)100:0.000139172)30:2.427e-07)30:2.427e-07,((EPI_ISL_685972:3.48622e-05,(EPI_ISL_685975:2.425e-07,EPI_ISL_692126:2.425e-07)30:2.427e-07)30:2.427e-07,EPI_ISL_692119:2.425e-07)100:6.96738e-05)30:2.427e-07,EPI_ISL_685210:2.425e-07)30:3.4465e-05,EPI_ISL_684345:3.47781e-05)30:2.427e-07,EPI_ISL_684328:3.48371e-05)30:2.427e-07,EPI_ISL_692012:6.9744e-05)30:2.427e-07,EPI_ISL_684234:3.48683e-05)30:2.427e-07,EPI_ISL_691912:2.425e-07)30:2.427e-07,EPI_ISL_685931:0.0001046438)30:2.427e-07,EPI_ISL_684286:0.0001046158)30:2.427e-07,EPI_ISL_685406:2.425e-07)30:2.427e-07,EPI_ISL_684289:2.425e-07)30:2.427e-07,(EPI_ISL_690758:6.97405e-05,EPI_ISL_685266:6.94994e-05)99:3.47449e-05)30:2.427e-07,EPI_ISL_480039:2.425e-07)30:2.427e-07,EPI_ISL_690753:3.48343e-05)30:2.427e-07,EPI_ISL_495446:0.0001790147)30:2.427e-07,EPI_ISL_684478:3.47831e-05)30:2.427e-07,EPI_ISL_480080:3.48647e-05)30:2.427e-07,EPI_ISL_688795:6.97443e-05)30:2.427e-07,EPI_ISL_684431:2.425e-07)30:2.427e-07,EPI_ISL_684363:2.425e-07)30:2.427e-07,EPI_ISL_691937:3.48386e-05)30:2.427e-07,EPI_ISL_684826:3.48702e-05)30:2.427e-07,(EPI_ISL_684608:2.425e-07,(EPI_ISL_685287:3.47972e-05,EPI_ISL_685290:3.48697e-05)30:2.427e-07)100:3.4797e-05)30:2.427e-07,EPI_ISL_684449:3.48659e-05)30:2.427e-07,(EPI_ISL_691353:2.425e-07,EPI_ISL_684538:2.425e-07)100:3.47609e-05)30:2.427e-07,EPI_ISL_691356:3.48659e-05)30:2.427e-07,EPI_ISL_685739:0.0001071902)30:2.427e-07,(((EPI_ISL_685914:6.9685e-05,EPI_ISL_686059:2.425e-07)30:2.427e-07,EPI_ISL_686063:3.48659e-05)99:2.427e-07,EPI_ISL_686082:3.48169e-05)100:3.48122e-05)30:2.427e-07)30:2.427e-07,EPI_ISL_692032:3.48705e-05)30:2.427e-07,EPI_ISL_691429:3.48659e-05)30:2.427e-07,EPI_ISL_691409:2.425e-07)30:2.427e-07,EPI_ISL_690622:2.425e-07)30:2.427e-07,(EPI_ISL_691249:2.425e-07,EPI_ISL_690759:2.425e-07)100:3.47828e-05)30:2.427e-07,EPI_ISL_685360:2.425e-07)30:2.427e-07,EPI_ISL_685661:3.48374e-05)30:2.427e-07,EPI_ISL_480012:3.48697e-05)30:2.427e-07,(EPI_ISL_685543:2.425e-07,EPI_ISL_684937:3.48705e-05)100:6.97451e-05)30:2.427e-07,EPI_ISL_690623:2.425e-07)30:2.427e-07,EPI_ISL_684847:6.9748e-05)30:2.427e-07,(((EPI_ISL_684387:0,EPI_ISL_684750:0):0,EPI_ISL_684809:0):0,EPI_ISL_691413:0):2.425e-07)30:2.427e-07,EPI_ISL_692019:3.48705e-05)30:2.427e-07,EPI_ISL_722896:0.0002756862)30:2.427e-07,(((EPI_ISL_452210:6.94692e-05,EPI_ISL_699949:0.0001044558)30:3.47037e-05,(EPI_ISL_710483:4.76805e-05,EPI_ISL_710484:3.43593e-05)100:0.0005729746)30:2.427e-07,EPI_ISL_732665:0.0004528136)30:3.4396e-05)30:2.427e-07,(EPI_ISL_685773:2.425e-07,EPI_ISL_685779:3.478e-05)100:6.95729e-05)30:2.427e-07,((EPI_ISL_684842:2.425e-07,EPI_ISL_684854:2.425e-07)100:3.48706e-05,EPI_ISL_684915:2.425e-07)99:3.48396e-05)30:2.427e-07,EPI_ISL_685960:0.0001045846)30:2.427e-07,EPI_ISL_684459:2.425e-07)30:2.427e-07,((EPI_ISL_692565:6.97491e-05,EPI_ISL_691869:2.425e-07)100:3.48745e-05,EPI_ISL_687592:3.48377e-05)100:0.0001394366)30:2.427e-07,EPI_ISL_685232:2.425e-07)30:2.427e-07,EPI_ISL_684754:6.95915e-05)30:2.427e-07,EPI_ISL_685086:3.48702e-05)30:2.427e-07,EPI_ISL_685076:0.0001045489)30:2.427e-07,EPI_ISL_480008:3.48702e-05)30:2.427e-07,EPI_ISL_684573:6.96097e-05)30:2.427e-07,(EPI_ISL_685520:0.0001394124,EPI_ISL_684879:2.425e-07)100:3.47847e-05)30:2.427e-07,((EPI_ISL_685024:3.48702e-05,EPI_ISL_685091:2.425e-07)30:2.427e-07,EPI_ISL_685058:2.425e-07)100:3.48354e-05)30:2.427e-07,EPI_ISL_691497:2.425e-07)30:2.427e-07,((EPI_ISL_684540:2.425e-07,EPI_ISL_684551:2.425e-07)30:2.427e-07,EPI_ISL_684553:2.425e-07)100:3.47836e-05)30:2.427e-07,((((((((((((((((((((EPI_ISL_480020:0,EPI_ISL_684586:0):0,EPI_ISL_691507:0):0,EPI_ISL_684934:0):0,EPI_ISL_684326:0):0,EPI_ISL_684981:0):0,EPI_ISL_691407:0):0,EPI_ISL_691875:0):0,EPI_ISL_691474:0):0,EP

I_ISL_691332:0):0,EPI_ISL_691434:0):0,EPI_ISL_684575:0):0,EPI_ISL_684771:0):0,EPI_ISL_684355:0):0,E
PI_ISL_691246:0):0,EPI_ISL_690777:0):0,EPI_ISL_691292:0):0,EPI_ISL_684250:0):0,EPI_ISL_691073:0):0,
EPI_ISL_685045:0):0,EPI_ISL_684789:0):0,EPI_ISL_691321:0):0,EPI_ISL_691457:0):2.425e-07)30:2.427e-
07,EPI_ISL_690751:3.48666e-05)30:2.427e-07,((((EPI_ISL_722856:2.425e-07,(EPI_ISL_722858:6.95733e-
05,EPI_ISL_722872:6.88289e-05)30:2.427e-07)30:2.427e-07,EPI_ISL_722857:2.425e-07)100:3.44173e-
05,EPI_ISL_722897:0.0001376398)99:6.88253e-05,EPI_ISL_690990:6.97194e-05)31:2.427e-
07)30:2.427e-07,(((EPI_ISL_547966:0,EPI_ISL_577624:0):2.425e-07,(EPI_ISL_546436:2.425e-
07,(EPI_ISL_577625:0.0001045505,EPI_ISL_626614:6.96966e-05)61:2.427e-07)32:2.427e-07)60:2.427e-
07,EPI_ISL_547967:3.48357e-05)100:0.0001743086)30:2.427e-07,(EPI_ISL_480160:2.425e-
07,(((((EPI_ISL_690676:2.425e-07,EPI_ISL_685066:0.0001050127)30:2.427e-
07,EPI_ISL_685019:3.48667e-05)30:2.427e-07,EPI_ISL_700147:0.0001392047)30:2.427e-
07,EPI_ISL_685178:3.48701e-05)30:2.427e-07,EPI_ISL_685174:0.0001043922)30:2.427e-
07)44:3.47845e-05)30:2.427e-07,EPI_ISL_685156:2.425e-07)30:2.427e-07,EPI_ISL_691159:2.425e-
07)30:2.427e-07,((((((((EPI_ISL_685669:3.48729e-05,(EPI_ISL_691063:3.48683e-
05,EPI_ISL_685047:6.95819e-05)31:2.427e-07)30:2.427e-07,EPI_ISL_686032:2.425e-07)30:2.427e-
07,EPI_ISL_685679:0.000978299)31:2.427e-07,EPI_ISL_685672:2.425e-07)100:3.4781e-
05,EPI_ISL_685230:6.98791e-05)30:2.427e-07,EPI_ISL_685507:3.4872e-05)30:2.427e-
07,EPI_ISL_685536:2.425e-07)30:2.427e-07,EPI_ISL_690667:3.48702e-05)30:2.427e-
07,EPI_ISL_685683:3.49554e-05)59:3.47854e-05)30:2.427e-07,(EPI_ISL_691520:3.48736e-
05,(EPI_ISL_685820:2.425e-07,EPI_ISL_685837:2.425e-07)100:3.48781e-05)67:3.48736e-05)30:2.427e-
07,(EPI_ISL_685603:6.97524e-05,(((((EPI_ISL_685528:2.425e-07,((((EPI_ISL_691220:2.425e-
07,(EPI_ISL_688763:2.425e-07,(EPI_ISL_690927:0.0001395419,EPI_ISL_690972:2.425e-
07)100:3.48721e-05)30:2.427e-07)30:2.427e-07,EPI_ISL_691120:2.425e-07)31:2.427e-
07,EPI_ISL_691116:2.425e-07)30:2.427e-07,EPI_ISL_691195:6.96933e-05)30:2.427e-07)30:2.427e-
07,(EPI_ISL_688779:2.425e-07,EPI_ISL_688791:2.425e-07)100:3.48683e-05)30:2.427e-
07,EPI_ISL_691134:3.48394e-05)30:2.427e-07,EPI_ISL_688767:2.425e-07)30:2.427e-
07,EPI_ISL_688762:2.425e-07)30:2.427e-07)100:3.47807e-05)30:2.427e-07,EPI_ISL_685191:3.48683e-
05)30:2.427e-07,EPI_ISL_690783:6.95949e-05)30:2.427e-07,(EPI_ISL_690684:2.425e-
07,(((((EPI_ISL_690685:2.425e-07,EPI_ISL_685894:2.425e-07)100:2.427e-07,EPI_ISL_685889:2.425e-
07)100:3.48368e-05,EPI_ISL_690686:3.48039e-05)31:2.427e-07,EPI_ISL_685888:2.425e-07)31:2.427e-
07,EPI_ISL_685891:3.48717e-05)30:2.427e-07)100:3.47861e-05)30:2.427e-
07,EPI_ISL_691042:3.48683e-05)30:2.427e-07,EPI_ISL_685556:3.48683e-05)30:2.427e-
07,((((EPI_ISL_685733:3.48681e-05,EPI_ISL_685638:2.425e-07)100:3.48679e-
05,(((((((EPI_ISL_685746:3.49252e-05,(((EPI_ISL_687062:2.425e-07,((EPI_ISL_687064:2.425e-
07,EPI_ISL_687066:2.425e-07)100:3.48689e-05,EPI_ISL_687067:2.425e-07)100:3.48689e-05)30:2.427e-
07,EPI_ISL_687768:3.48689e-05)30:2.427e-07,EPI_ISL_687065:2.425e-07)100:3.48721e-05)39:2.427e-
07,((EPI_ISL_684980:3.48718e-05,(EPI_ISL_685110:2.425e-07,EPI_ISL_685111:2.425e-07)100:3.48721e-
05)56:2.427e-07,EPI_ISL_691094:6.97443e-05)100:6.97506e-05)31:2.427e-
07,EPI_ISL_685500:6.95799e-05)30:2.427e-07,EPI_ISL_685752:3.48371e-05)30:2.427e-
07,EPI_ISL_686029:3.48687e-05)30:2.427e-07,EPI_ISL_684799:3.47893e-05)100:3.47864e-
05,(((EPI_ISL_684359:0,EPI_ISL_684969:0):2.425e-07,((EPI_ISL_685549:2.425e-
07,((EPI_ISL_685557:3.478e-05,EPI_ISL_685558:2.425e-07)100:3.47803e-05,((EPI_ISL_684897:2.425e-
07,EPI_ISL_685010:3.48037e-05)33:2.427e-07,EPI_ISL_685991:3.48411e-05)30:2.427e-07)30:2.427e-
07)30:2.427e-07,EPI_ISL_684903:6.95753e-05)30:2.427e-07)30:2.427e-07,EPI_ISL_684968:2.425e-

07)100:3.47848e-05)72:2.427e-07)30:2.427e-07,EPI_ISL_684379:2.425e-07)31:2.427e-
07,EPI_ISL_685220:3.48846e-05)83:3.47847e-05)30:2.427e-
07,(EPI_ISL_534339:0.0001044049,EPI_ISL_685078:6.95849e-05)69:3.47993e-05)30:2.427e-
07,((((EPI_ISL_685623:3.48377e-05,EPI_ISL_685627:2.425e-07)82:2.427e-07,(EPI_ISL_685624:2.425e-
07,(EPI_ISL_685645:2.425e-07,EPI_ISL_685646:2.425e-07)31:2.427e-07)100:0.0001043778)42:2.427e-
07,(((EPI_ISL_685626:0,EPI_ISL_685630:0):2.425e-07,EPI_ISL_685629:2.425e-07)100:2.427e-
07,EPI_ISL_685631:2.425e-07)100:3.47823e-05)30:2.427e-07,EPI_ISL_685628:2.425e-07)100:3.47868e-
05)30:2.427e-07,EPI_ISL_684696:3.4794e-05)30:2.427e-07,EPI_ISL_690624:3.48452e-05)30:2.427e-
07,((EPI_ISL_684430:2.425e-07,EPI_ISL_691331:2.425e-07)100:2.427e-07,EPI_ISL_691330:3.48736e-
05)100:3.48723e-05)30:2.427e-07,(((((((((((((EPI_ISL_685714:6.97078e-05,EPI_ISL_685716:3.48757e-
05)100:3.48456e-05,(EPI_ISL_685719:0.0001046518,EPI_ISL_685276:2.425e-07)98:3.48746e-
05)98:3.48475e-05,((((EPI_ISL_684293:3.47865e-05,((EPI_ISL_685541:0,EPI_ISL_684912:0):2.425e-
07,((((((EPI_ISL_691961:3.48377e-05,(((EPI_ISL_691970:2.425e-07,EPI_ISL_684648:2.425e-07)30:2.427e-
07,EPI_ISL_685883:2.425e-07)30:2.427e-07,EPI_ISL_692014:3.48697e-05)30:2.427e-07)30:2.427e-
07,EPI_ISL_691872:3.487e-05)30:2.427e-07,EPI_ISL_684713:3.48698e-05)100:6.9689e-
05,EPI_ISL_685815:2.425e-07)30:2.427e-07,EPI_ISL_690724:3.48698e-05)30:2.427e-
07,EPI_ISL_690966:2.425e-07)30:2.427e-07)30:2.427e-07)30:2.427e-07,(EPI_ISL_685539:6.97552e-
05,(EPI_ISL_691993:6.97648e-05,(EPI_ISL_684649:2.425e-07,EPI_ISL_684687:2.425e-07)100:3.4838e-
05)30:2.427e-07)100:3.48379e-05)30:2.427e-07,((EPI_ISL_690731:2.425e-07,EPI_ISL_690736:2.425e-
07)30:2.427e-07,EPI_ISL_690739:2.425e-07)100:3.48346e-05)30:2.427e-
07,EPI_ISL_685869:0.0001046518)30:2.427e-07)30:2.427e-07,(EPI_ISL_691099:2.425e-
07,EPI_ISL_691085:3.48737e-05)100:6.97531e-05)30:2.427e-07,EPI_ISL_685172:3.4781e-05)30:2.427e-
07,EPI_ISL_690821:6.97545e-05)30:2.427e-07,(((EPI_ISL_691542:2.425e-07,EPI_ISL_690842:2.425e-
07)31:2.427e-07,EPI_ISL_690841:2.425e-07)30:2.427e-07,EPI_ISL_690843:2.425e-
07)100:0.0001392025)30:2.427e-07,EPI_ISL_685994:3.48458e-05)30:2.427e-07,EPI_ISL_686004:2.425e-
07)30:2.427e-07,EPI_ISL_691907:3.48407e-05)30:2.427e-07,EPI_ISL_685126:6.98027e-05)30:2.427e-
07,EPI_ISL_685084:2.425e-07)30:2.427e-07,EPI_ISL_684213:3.48731e-05)36:2.427e-
07,EPI_ISL_686106:6.9588e-05)89:3.47808e-05)30:2.427e-07,((((((((((((((((((((EPI_ISL_480069:2.425e-
07,EPI_ISL_690946:6.96932e-05)100:3.47877e-05,((((((((((((((EPI_ISL_591479:2.425e-
07,((((((((((((((((((((((EPI_ISL_591480:0,EPI_ISL_690995:0):0,EPI_ISL_684191:0):0,EPI_ISL_690947:0):0,EPI
_ISL_684294:0):0,EPI_ISL_690914:0):0,EPI_ISL_691049:0):0,EPI_ISL_686017:0):0,EPI_ISL_685597:0):0,EP
I_ISL_690934:0):0,EPI_ISL_684673:0):0,EPI_ISL_691352:0):2.425e-07,((((((EPI_ISL_684311:2.425e-
07,EPI_ISL_685900:3.47831e-05)100:3.47862e-05,(((((((EPI_ISL_684393:3.48394e-
05,((((EPI_ISL_685614:3.48757e-05,(((EPI_ISL_685615:3.5096e-
05,((((((((EPI_ISL_685491:0,EPI_ISL_685487:0):0,EPI_ISL_685485:0):0,EPI_ISL_685486:0):2.425e-
07,EPI_ISL_685496:2.425e-07)100:3.48712e-05,EPI_ISL_685468:2.425e-07)32:2.427e-
07,((((EPI_ISL_685494:0,EPI_ISL_690671:0):0,EPI_ISL_685488:0):0,EPI_ISL_685473:0):0,EPI_ISL_685475:
0):2.425e-07)44:2.427e-07,(((EPI_ISL_685471:2.425e-07,EPI_ISL_685481:3.48705e-05)100:3.48737e-
05,EPI_ISL_685476:2.425e-07)30:2.427e-07,EPI_ISL_685484:2.425e-07)100:3.48705e-05)38:2.427e-
07,EPI_ISL_685472:3.47833e-05)30:2.427e-07)30:2.427e-07,EPI_ISL_685479:2.425e-07)30:2.427e-
07,EPI_ISL_685477:2.425e-07)38:2.427e-07)100:6.95739e-05,(EPI_ISL_686861:2.425e-
07,((EPI_ISL_686867:2.425e-07,EPI_ISL_686868:2.425e-07)100:3.48749e-05,EPI_ISL_686869:3.48394e-
05)30:2.427e-07)100:3.48425e-05)30:2.427e-07,EPI_ISL_684394:0.0001045379)31:2.427e-
07,EPI_ISL_684390:2.425e-07)30:2.427e-07)100:3.47905e-05,((((EPI_ISL_684186:2.425e-

07,EPI_ISL_684749:3.48757e-05)100:3.48393e-05,EPI_ISL_688793:2.425e-07)31:2.427e-
07,EPI_ISL_687931:0.0002435968)31:2.427e-07,(EPI_ISL_685850:0,EPI_ISL_690915:0):2.425e-
07)100:3.47636e-05)100:6.9547e-05,(((((EPI_ISL_687052:2.425e-07,EPI_ISL_687043:2.425e-
07)30:2.427e-07,(((EPI_ISL_687057:3.48753e-05,EPI_ISL_687059:2.425e-07)30:2.427e-
07,(EPI_ISL_687058:2.425e-07,EPI_ISL_687060:2.425e-07)100:3.48754e-05)100:2.427e-
07,EPI_ISL_687047:2.425e-07)100:3.45744e-05)30:2.427e-
07,((EPI_ISL_687054:0,EPI_ISL_687048:0):0,EPI_ISL_690834:0):2.425e-07)30:2.427e-
07,EPI_ISL_687044:3.48284e-05)99:3.45789e-05,EPI_ISL_691093:6.97542e-05)100:6.91623e-
05)30:2.427e-07,EPI_ISL_684725:3.47852e-05)30:2.427e-07,EPI_ISL_685596:3.48732e-05)30:2.427e-
07,EPI_ISL_686037:0.0001046553)30:2.427e-07,EPI_ISL_688788:6.97618e-05)30:2.427e-07)30:2.427e-
07,EPI_ISL_686060:3.51683e-05)30:2.427e-07,(EPI_ISL_684681:2.425e-07,((EPI_ISL_684682:2.425e-
07,(EPI_ISL_684692:0,EPI_ISL_685918:0):2.425e-07)30:2.427e-07,EPI_ISL_685922:2.425e-07)99:2.427e-
07)100:3.48341e-05)30:2.427e-07,(((((EPI_ISL_685832:2.425e-07,EPI_ISL_685875:2.425e-07)30:2.427e-
07,EPI_ISL_685846:2.425e-07)30:2.427e-07,EPI_ISL_685845:2.425e-07)99:2.427e-
07,EPI_ISL_685863:2.425e-07)100:3.47898e-05,((EPI_ISL_685839:2.425e-07,EPI_ISL_685865:2.425e-
07)30:2.427e-07,EPI_ISL_685849:2.425e-07)100:3.48441e-05)100:3.47852e-05)30:2.427e-
07,EPI_ISL_690993:2.425e-07)30:2.427e-07)30:2.427e-07,EPI_ISL_690967:2.425e-07)30:2.427e-
07,EPI_ISL_684744:6.96385e-05)30:2.427e-07,EPI_ISL_685708:2.425e-07)30:2.427e-
07,(EPI_ISL_684200:2.425e-07,EPI_ISL_684593:0.0001046588)100:6.96931e-05)30:2.427e-
07,EPI_ISL_691509:2.425e-07)30:2.427e-07,EPI_ISL_685838:2.425e-07)30:2.427e-
07,EPI_ISL_685604:3.48424e-05)30:2.427e-07,EPI_ISL_685598:3.48738e-05)30:2.427e-
07,EPI_ISL_591482:3.48705e-05)30:2.427e-07,EPI_ISL_691537:3.51103e-05)30:2.427e-
07,EPI_ISL_684463:2.425e-07)30:2.427e-07)30:2.427e-07,EPI_ISL_684741:2.425e-07)30:2.427e-
07,EPI_ISL_684873:3.47851e-05)30:2.427e-07,(EPI_ISL_684714:3.47916e-05,EPI_ISL_685933:2.425e-
07)100:3.47912e-05)30:2.427e-07,EPI_ISL_691536:6.97212e-05)30:2.427e-
07,EPI_ISL_684752:0.0001046309)30:2.427e-07,EPI_ISL_685593:3.48386e-05)30:2.427e-
07,(EPI_ISL_685710:3.48383e-05,(EPI_ISL_686061:2.425e-07,(EPI_ISL_686064:2.425e-
07,EPI_ISL_686067:6.97471e-05)99:2.427e-07)31:2.427e-07)100:3.47684e-05)30:2.427e-
07,EPI_ISL_684320:6.96902e-05)30:2.427e-07,EPI_ISL_684582:6.88442e-05)30:2.427e-
07,EPI_ISL_685959:3.51203e-05)30:2.427e-07,(EPI_ISL_684300:2.425e-07,EPI_ISL_684319:2.425e-
07)100:0.0001032198)30:2.427e-07,EPI_ISL_685897:2.425e-07)30:2.427e-07,EPI_ISL_684680:3.47815e-
05)30:2.427e-07)30:2.427e-07,(EPI_ISL_685802:3.4804e-05,EPI_ISL_686027:2.425e-07)100:3.48044e-
05)30:2.427e-07,(EPI_ISL_684206:2.425e-07,(EPI_ISL_684208:2.425e-07,EPI_ISL_684761:6.97469e-
05)30:2.427e-07)100:3.47864e-05)30:2.427e-07,EPI_ISL_690992:2.425e-07)30:2.427e-
07,EPI_ISL_691538:6.96831e-05)30:2.427e-07,EPI_ISL_685792:2.425e-07)30:2.427e-
07,EPI_ISL_591477:2.425e-07)30:2.427e-07,EPI_ISL_690941:6.97362e-05)30:2.427e-
07,EPI_ISL_690940:3.48442e-05)30:2.427e-07,EPI_ISL_690928:2.425e-07)30:2.427e-
07,EPI_ISL_690994:2.425e-07)30:2.427e-07,EPI_ISL_684724:2.425e-07)30:2.427e-
07,EPI_ISL_690909:3.47865e-05)30:2.427e-07,EPI_ISL_685665:6.96929e-05)30:2.427e-
07,EPI_ISL_690917:6.9756e-05)30:2.427e-07,EPI_ISL_690938:2.425e-07)30:2.427e-
07,((((((((((((((((((EPI_ISL_591474:0,EPI_ISL_684748:0):0,EPI_ISL_685785:0):0,EPI_ISL_685599:0):0,EPI_I
SL_685595:0):0,EPI_ISL_684864:0):0,EPI_ISL_685806:0):0,EPI_ISL_690898:0):0,EPI_ISL_684339:0):0,EPI_
ISL_690883:0):0,EPI_ISL_691978:0):0,EPI_ISL_690968:0):0,EPI_ISL_685243:0):0,EPI_ISL_685793:0):0,EPI
_ISL_591471:0):0,EPI_ISL_686024:0):0,EPI_ISL_690918:0):0,EPI_ISL_685699:0):0,EPI_ISL_685783:0):0,EP

I_ISL_685800:0):0,EPI_ISL_690886:0):2.425e-07)30:2.427e-07,EPI_ISL_684742:3.48442e-05)30:2.427e-
07,EPI_ISL_684679:3.48409e-05)30:2.427e-07,EPI_ISL_690942:3.47925e-05)49:3.44169e-05)30:2.427e-
07,((((((EPI_ISL_480022:2.425e-07,(EPI_ISL_684424:2.425e-07,EPI_ISL_684656:3.48422e-05)30:2.427e-
07)100:3.48429e-05,(((((EPI_ISL_685426:2.425e-07,EPI_ISL_685438:3.48759e-05)30:2.427e-
07,EPI_ISL_685449:2.425e-07)31:2.427e-07,EPI_ISL_685413:2.425e-07)100:6.97636e-
05,EPI_ISL_685199:3.47831e-05)30:2.427e-07,EPI_ISL_684797:6.96865e-05)30:2.427e-07)30:2.427e-
07,EPI_ISL_685580:0.0001392145)30:2.427e-07,EPI_ISL_684568:0.0001056563)30:2.427e-
07,EPI_ISL_685717:0.0001046611)30:2.427e-07,EPI_ISL_690659:2.425e-07)80:3.47829e-05)30:2.427e-
07,((((((((((EPI_ISL_684832:2.425e-07,EPI_ISL_684835:2.425e-07)100:3.48716e-
05,((((((EPI_ISL_684834:3.48717e-05,EPI_ISL_684853:2.425e-07)31:2.427e-07,EPI_ISL_684855:2.425e-
07)30:2.427e-07,EPI_ISL_684806:2.425e-07)100:6.9677e-05,(EPI_ISL_684846:2.425e-
07,EPI_ISL_684801:2.425e-07)100:3.47893e-05)30:2.427e-07,EPI_ISL_684836:2.425e-07)30:2.427e-
07,((EPI_ISL_684818:2.425e-07,EPI_ISL_684828:2.425e-07)30:2.427e-07,EPI_ISL_684827:2.45e-
07)97:8.04895e-05)31:2.455e-07)30:2.455e-
07,((EPI_ISL_684844:0,EPI_ISL_684852:0):0,EPI_ISL_684803:0):2.425e-07)30:2.455e-
07,EPI_ISL_684802:2.425e-07)30:2.455e-
07,((EPI_ISL_684849:0,EPI_ISL_684804:0):0,EPI_ISL_684805:0):2.425e-07)31:2.455e-
07,EPI_ISL_684851:3.47872e-05)30:2.455e-07,EPI_ISL_684800:2.425e-07)30:2.455e-
07,EPI_ISL_684845:2.425e-07)30:2.455e-07,EPI_ISL_684833:2.425e-07)37:2.455e-
07,EPI_ISL_684850:2.425e-07)100:0.0001613992)30:2.427e-
07,((EPI_ISL_479553:0.000139074,(EPI_ISL_685194:2.425e-07,EPI_ISL_687747:6.97559e-
05)100:3.48741e-05)33:2.427e-07,EPI_ISL_542427:3.47146e-05)33:3.45796e-05)30:2.427e-
07,((EPI_ISL_684321:3.48718e-05,(EPI_ISL_684323:3.48718e-05,EPI_ISL_685282:6.97593e-
05)44:2.427e-07)44:2.427e-07,EPI_ISL_684613:6.97593e-05)100:6.97504e-05)30:2.427e-
07,(((EPI_ISL_603154:2.425e-07,(EPI_ISL_603166:6.93928e-05,(EPI_ISL_603140:3.47072e-
05,EPI_ISL_603147:2.425e-07)30:2.427e-07)30:2.427e-07)30:2.427e-07,EPI_ISL_603144:2.425e-
07)31:2.427e-07,EPI_ISL_603170:3.47072e-05)100:3.46711e-05)30:2.427e-
07,EPI_ISL_685510:0.0001045502)30:2.427e-07)30:2.427e-07,(EPI_ISL_685789:2.425e-
07,((((((((((((((((((((((EPI_ISL_690280:0,EPI_ISL_688176:0):2.425e-07,(((((EPI_ISL_688245:6.97593e-
05,EPI_ISL_688259:2.425e-07)30:2.427e-07,EPI_ISL_690335:2.425e-07)30:2.427e-
07,EPI_ISL_688166:2.425e-07)30:2.427e-07,EPI_ISL_688169:6.96964e-05)30:2.427e-
07,(EPI_ISL_688252:2.425e-07,EPI_ISL_688253:2.425e-07)100:6.97051e-05)30:2.427e-07)30:2.427e-
07,EPI_ISL_688195:3.48763e-05)100:3.48478e-05,EPI_ISL_686754:2.425e-07)30:2.427e-
07,EPI_ISL_687722:3.48794e-05)100:3.48491e-
05,(((EPI_ISL_689280:6.9763e-05,(EPI_ISL_689229:2.425e-
07,((EPI_ISL_688958:2.425e-07,EPI_ISL_688944:6.97697e-05)30:2.427e-07,EPI_ISL_687550:6.96349e-
05)30:2.427e-07)30:2.427e-07)98:3.48092e-05,((EPI_ISL_690495:2.425e-07,EPI_ISL_687427:3.48775e-
05)100:3.48772e-05,EPI_ISL_687649:3.47947e-05)30:2.427e-07)30:2.427e-
07,EPI_ISL_688868:0.0001046674)30:2.427e-07,((EPI_ISL_688420:2.425e-07,EPI_ISL_688463:2.425e-
07)31:2.427e-07,EPI_ISL_688459:2.425e-07)99:6.96024e-05)30:2.427e-07,EPI_ISL_686166:3.48798e-
05)30:2.427e-07,EPI_ISL_686157:2.425e-07)30:2.427e-07,EPI_ISL_688455:3.48767e-05)30:2.427e-
07,EPI_ISL_689609:3.48821e-05)30:2.427e-07,EPI_ISL_690268:2.425e-07)100:3.47991e-
05,EPI_ISL_690014:2.425e-07)30:2.427e-07,((EPI_ISL_690005:2.425e-07,EPI_ISL_689995:2.425e-
07)100:3.47997e-05,(EPI_ISL_689655:2.425e-07,((((EPI_ISL_689515:2.425e-07,((EPI_ISL_688871:2.425e-

07,EPI_ISL_687121:2.425e-07)100:6.97141e-05,EPI_ISL_688881:2.425e-07)31:2.427e-07)31:2.427e-07,EPI_ISL_688694:3.52197e-05)30:2.427e-07,EPI_ISL_688924:0.0001394733)100:3.4799e-05,((EPI_ISL_688266:6.9602e-05,EPI_ISL_686406:2.425e-07)31:2.427e-07,EPI_ISL_692248:2.425e-07)30:2.427e-07)30:2.427e-07)97:3.47952e-05)90:3.47997e-05)30:2.427e-07,EPI_ISL_690317:3.48138e-05)30:2.427e-07,EPI_ISL_687322:3.48803e-05)30:2.427e-07,EPI_ISL_690360:3.48766e-05)30:2.427e-07,EPI_ISL_690552:3.48803e-05)30:2.427e-07,EPI_ISL_689866:3.4792e-05)30:2.427e-07,((EPI_ISL_686158:6.96452e-05,(EPI_ISL_686167:2.425e-07,EPI_ISL_686150:6.98099e-05)31:2.427e-07)31:2.427e-07,EPI_ISL_686152:3.48942e-05)100:3.48158e-05)30:2.427e-07,EPI_ISL_688281:6.97708e-05)30:2.427e-07,EPI_ISL_690469:2.425e-07)30:2.427e-07,(EPI_ISL_686270:2.425e-07,EPI_ISL_686272:2.425e-07)100:0.0001046657)30:2.427e-07,EPI_ISL_689213:3.488e-05)30:2.427e-07,(EPI_ISL_688012:2.425e-07,EPI_ISL_688041:3.488e-05)100:0.0002094631)30:2.427e-07,EPI_ISL_690142:3.53352e-05)30:2.427e-07,EPI_ISL_687277:2.425e-07)30:2.427e-07,EPI_ISL_692234:3.48769e-05)30:2.427e-07,(((((((EPI_ISL_691560:0,EPI_ISL_687460:0):0,EPI_ISL_687380:0):0,EPI_ISL_692219:0):0,EPI_ISL_692183:0):0,EPI_ISL_687004:0):0,EPI_ISL_687284:0):0,EPI_ISL_692268:0):2.425e-07)30:2.427e-07,EPI_ISL_687394:3.48765e-05)30:2.427e-07,EPI_ISL_690048:2.425e-07)30:2.427e-07,EPI_ISL_689798:2.425e-07)30:2.427e-07,EPI_ISL_692438:6.97677e-05)30:2.427e-07,EPI_ISL_692403:3.48765e-05)30:2.427e-07,EPI_ISL_692257:3.48811e-05)30:2.427e-07,EPI_ISL_688249:0.0001745138)30:2.427e-07,EPI_ISL_690531:3.48763e-05)30:2.427e-07,EPI_ISL_688562:3.48487e-05)30:2.427e-07,EPI_ISL_692666:2.425e-07)30:2.427e-07,EPI_ISL_687409:2.425e-07)30:2.427e-07,EPI_ISL_690107:2.425e-07)59:3.47878e-05,((((((((((EPI_ISL_690179:6.97697e-05,((EPI_ISL_689040:3.48666e-05,EPI_ISL_689043:6.97832e-05)100:6.97828e-05,EPI_ISL_689045:3.47945e-05)30:2.427e-07)30:2.427e-07,EPI_ISL_692208:2.425e-07)30:2.427e-07,EPI_ISL_692684:2.425e-07)30:2.427e-07,EPI_ISL_687030:2.425e-07)100:3.47943e-05,(((EPI_ISL_686801:3.48795e-05,((((((EPI_ISL_686876:2.425e-07,((((EPI_ISL_686799:2.425e-07,EPI_ISL_692415:0.0001045605)30:2.427e-07,EPI_ISL_687580:2.425e-07)30:2.427e-07,EPI_ISL_688145:3.48767e-05)30:2.427e-07,EPI_ISL_688121:2.425e-07)30:2.427e-07)30:2.427e-07,EPI_ISL_692225:2.425e-07)30:2.427e-07,EPI_ISL_686670:3.47893e-05)30:2.427e-07,EPI_ISL_692502:2.425e-07)30:2.427e-07,EPI_ISL_686744:0.0001046509)100:3.47924e-05,(((EPI_ISL_688081:2.425e-07,EPI_ISL_688078:3.48767e-05)100:3.48769e-05,EPI_ISL_688069:2.425e-07)100:2.427e-07,EPI_ISL_688843:2.425e-07)100:3.47893e-05)44:2.427e-07)30:2.427e-07,(((EPI_ISL_692641:2.425e-07,EPI_ISL_692644:2.425e-07)100:0.0001401302,EPI_ISL_691850:3.48489e-05)95:2.427e-07,EPI_ISL_692106:2.425e-07)96:3.48483e-05)30:2.427e-07,EPI_ISL_687174:3.46916e-05)30:2.427e-07)30:2.427e-07,EPI_ISL_690807:2.425e-07)30:2.427e-07,(EPI_ISL_687950:3.48814e-05,EPI_ISL_687693:2.425e-07)100:6.97691e-05)30:2.427e-07,(EPI_ISL_689017:2.425e-07,(EPI_ISL_689039:2.425e-07,EPI_ISL_687832:0.0001046824)30:2.427e-07)88:3.48797e-05)30:2.427e-07,(((((EPI_ISL_686760:2.425e-07,EPI_ISL_686769:2.425e-07)100:2.427e-07,EPI_ISL_686768:2.425e-07)100:3.48758e-05,EPI_ISL_692073:2.425e-07)30:2.427e-07,EPI_ISL_692084:3.48758e-05)40:2.427e-07,EPI_ISL_692072:3.48765e-05)100:6.97646e-05)30:2.427e-07,EPI_ISL_689347:6.97593e-05)30:3.46958e-05,EPI_ISL_687486:3.48394e-05)30:2.427e-07)30:2.427e-07,EPI_ISL_689884:2.425e-07)30:2.427e-07)30:2.427e-07,EPI_ISL_692520:0.0001045678)30:2.427e-07,EPI_ISL_690236:3.47925e-05)30:2.427e-07,(EPI_ISL_692676:3.48438e-05,EPI_ISL_688529:2.425e-07)100:0.0001043994)30:2.427e-07,EPI_ISL_692316:3.48804e-05)30:2.427e-

07,EPI_ISL_688817:3.48794e-05)30:2.427e-07,(((EPI_ISL_691552:3.48659e-05,((((((((EPI_ISL_691555:3.48487e-05,(((((((((EPI_ISL_686804:0,EPI_ISL_687770:0):0,EPI_ISL_688215:0):0,EPI_ISL_687705:0):0,EPI_ISL_692051:0):2.425e-07,(((EPI_ISL_691371:2.425e-07,(((((EPI_ISL_688088:2.425e-07,EPI_ISL_688823:2.425e-07)30:2.427e-07,EPI_ISL_688555:2.425e-07)100:6.96266e-05,EPI_ISL_687804:0.0001052497)42:3.47901e-05,(EPI_ISL_687687:2.425e-07,EPI_ISL_687696:2.425e-07)100:0.0001044147)30:2.427e-07,EPI_ISL_688608:6.97687e-05)30:2.427e-07)30:2.427e-07,EPI_ISL_690007:0.000104662)30:2.427e-07,EPI_ISL_688291:0.0001395779)30:2.427e-07)30:2.427e-07,EPI_ISL_692079:3.488e-05)30:2.427e-07,EPI_ISL_687873:2.425e-07)30:2.427e-07,EPI_ISL_687776:0.0001395741)30:2.427e-07,EPI_ISL_686736:2.425e-07)30:2.427e-07)30:2.427e-07,EPI_ISL_690031:3.48787e-05)30:2.427e-07,EPI_ISL_691860:3.48758e-05)30:2.427e-07,EPI_ISL_689803:0.0002092575)30:2.427e-07,EPI_ISL_688362:3.48757e-05)30:2.427e-07,EPI_ISL_688342:0.0001395859)30:2.427e-07,EPI_ISL_690098:0.0001046636)30:2.427e-07,EPI_ISL_692327:3.48483e-05)30:2.427e-07)30:2.427e-07,EPI_ISL_686729:2.425e-07)30:2.427e-07,EPI_ISL_689265:0.0001395734)30:3.47932e-05)30:2.427e-07,EPI_ISL_692573:6.97577e-05)30:2.427e-07,((((EPI_ISL_689059:0.0002093754,EPI_ISL_687075:2.425e-07)31:2.427e-07,EPI_ISL_687723:2.425e-07)99:2.427e-07,EPI_ISL_687850:2.425e-07)98:2.427e-07,EPI_ISL_688496:2.425e-07)100:3.4787e-05)30:2.427e-07,EPI_ISL_690226:3.48111e-05)30:2.427e-07,EPI_ISL_690234:3.4875e-05)30:2.427e-07,EPI_ISL_690176:3.48424e-05)30:2.427e-07,EPI_ISL_686743:3.48795e-05)30:2.427e-07,EPI_ISL_690981:2.425e-07)30:2.427e-07,EPI_ISL_692205:3.47929e-05)30:2.427e-07,EPI_ISL_687724:0.0001744812)30:2.427e-07,EPI_ISL_688472:2.425e-07)30:2.427e-07,EPI_ISL_690230:2.425e-07)44:3.46981e-05,EPI_ISL_689091:0.0001395747)100:0.0001388423)98:3.46944e-05)30:2.427e-07,EPI_ISL_684195:3.4846e-05)30:2.427e-07,(((EPI_ISL_684306:3.47917e-05,(((EPI_ISL_688771:2.425e-07,EPI_ISL_688778:2.425e-07)30:2.427e-07,EPI_ISL_688777:2.425e-07)100:3.48461e-05,EPI_ISL_691070:3.48814e-05)86:2.427e-07)43:2.427e-07,EPI_ISL_687927:3.47956e-05)30:2.427e-07,EPI_ISL_686041:2.425e-07)100:3.47913e-05)30:3.44081e-05,EPI_ISL_434546:2.425e-07)30:2.427e-07,EPI_ISL_732672:0.0001740389)30:2.427e-07,EPI_ISL_513023:3.44035e-05)30:2.427e-07,EPI_ISL_542372:2.425e-07)30:2.427e-07,EPI_ISL_486822:0.0001044042)30:2.427e-07,EPI_ISL_542238:2.425e-07)30:2.427e-07,EPI_ISL_542426:3.47203e-05)30:2.427e-07,EPI_ISL_486820:3.4792e-05)30:2.427e-07,EPI_ISL_471540:3.4848e-05)30:2.427e-07,EPI_ISL_512884:3.44068e-05)30:2.427e-07,EPI_ISL_542386:6.94561e-05)30:2.427e-07,EPI_ISL_539299:6.95915e-05)30:2.427e-07,EPI_ISL_513043:6.88291e-05)30:2.427e-07,(((EPI_ISL_479621:0,EPI_ISL_534346:0):0,EPI_ISL_610238:0):0,EPI_ISL_486826:0):2.425e-07)30:2.427e-07,(EPI_ISL_468914:2.425e-07,EPI_ISL_469020:2.425e-07)100:3.44039e-05)30:2.427e-07,EPI_ISL_653815:3.44921e-05)30:2.427e-07,EPI_ISL_542198:3.47262e-05)30:2.427e-07,EPI_ISL_653818:6.88365e-05)30:2.427e-07,EPI_ISL_525570:3.44036e-05)30:2.427e-07,EPI_ISL_542166:6.94592e-05)30:2.427e-07,EPI_ISL_653817:3.45195e-05)30:2.427e-07,EPI_ISL_542262:2.425e-07)30:2.427e-07,(EPI_ISL_542158:2.425e-07,EPI_ISL_542162:2.425e-07)100:6.94564e-05)30:2.427e-07,EPI_ISL_542210:6.9507e-05)30:2.427e-07,EPI_ISL_542119:6.945e-05)30:2.427e-07,EPI_ISL_542362:0.0001040914)30:2.427e-07,EPI_ISL_491092:3.48466e-05)30:2.427e-07,EPI_ISL_498164:2.425e-07)30:2.427e-07,EPI_ISL_450241:6.95852e-05)30:2.427e-07)30:2.427e-07,EPI_ISL_468320:6.9434e-05)30:2.427e-07,EPI_ISL_653816:3.44507e-05)30:2.427e-07,EPI_ISL_516946:3.51041e-05)30:2.427e-07,EPI_ISL_525639:3.47282e-05)30:2.427e-07)30:2.427e-

07,EPI_ISL_456156:2.425e-07)30:2.427e-07,(EPI_ISL_542170:2.425e-07,EPI_ISL_542172:2.425e-
07)100:6.94508e-05)30:2.427e-07,EPI_ISL_542146:3.47218e-05)30:2.427e-07,EPI_ISL_542099:2.425e-
07)30:2.427e-07,(EPI_ISL_732664:0.0002092012,EPI_ISL_717968:0.0003138896)53:3.45572e-
05)30:2.427e-07,(EPI_ISL_542422:2.425e-07,EPI_ISL_542175:3.47223e-05)99:3.47255e-05)30:2.427e-
07,EPI_ISL_542180:3.47223e-05)30:2.427e-07,EPI_ISL_475663:0.0001032341)30:2.427e-
07,EPI_ISL_603223:0.0001032432)30:2.427e-07,EPI_ISL_477194:6.89235e-05)30:2.427e-
07,EPI_ISL_479620:0.0001392479)30:2.427e-07,EPI_ISL_421652:2.425e-07)30:2.427e-
07,(EPI_ISL_732668:0.0004534795,EPI_ISL_691519:0.0001747549)33:3.46002e-05)30:2.427e-
07,EPI_ISL_542195:3.47262e-05)30:2.427e-07,EPI_ISL_539781:2.425e-07)30:2.427e-
07,EPI_ISL_469016:3.44052e-05)30:2.427e-
07,((((((((((((((((((((EPI_ISL_542348:0,EPI_ISL_542214:0):0,EPI_ISL_542131:0):0,EPI_ISL_542326:0):0,EPI_
ISL_542287:0):0,EPI_ISL_542237:0):0,EPI_ISL_542242:0):0,EPI_ISL_542199:0):0,EPI_ISL_542193:0):0,EPI
_ISL_542415:0):0,EPI_ISL_542258:0):0,EPI_ISL_542108:0):0,EPI_ISL_542219:0):0,EPI_ISL_542256:0):0,EP
I_ISL_542420:0):0,EPI_ISL_542431:0):0,EPI_ISL_542249:0):0,EPI_ISL_542122:0):0,EPI_ISL_542118:0):0,E
PI_ISL_542200:0):0,EPI_ISL_542433:0):0,EPI_ISL_542332:0):2.425e-07)30:2.427e-
07,EPI_ISL_684938:3.4848e-05)30:2.427e-07,EPI_ISL_636972:0.0001721066)30:2.427e-
07,((EPI_ISL_486828:2.425e-07,EPI_ISL_653800:0.0002788274)57:2.427e-
07,EPI_ISL_717972:0.0003135998)75:3.47943e-05)30:2.427e-07,(EPI_ISL_610243:3.48039e-
05,EPI_ISL_717974:0.0002089549)100:0.0002089782)30:2.427e-07,EPI_ISL_542311:6.94536e-
05)30:2.427e-07,EPI_ISL_729848:0.0001394706)30:2.427e-07,EPI_ISL_450244:3.47834e-05)30:2.427e-
07,EPI_ISL_422424:2.425e-07)30:2.427e-07,EPI_ISL_542120:3.47279e-05)30:2.427e-
07,(((EPI_ISL_513021:2.425e-07,EPI_ISL_513046:3.44057e-05)100:2.427e-
07,EPI_ISL_479572:0.0002434493)43:3.441e-05,(EPI_ISL_513056:3.44057e-05,EPI_ISL_512906:2.425e-
07)100:2.427e-07)32:3.44102e-05)30:2.427e-07,EPI_ISL_422427:2.425e-07)30:2.427e-
07,EPI_ISL_479624:3.47937e-05)30:2.427e-07,EPI_ISL_732673:0.0005229308)30:2.427e-
07,(EPI_ISL_491117:0,EPI_ISL_471544:0):2.425e-07)30:2.427e-07,EPI_ISL_542106:3.47272e-
05)30:2.427e-07,(EPI_ISL_491095:6.9718e-05,EPI_ISL_491118:3.48578e-05)100:3.48481e-05)30:2.427e-
07,EPI_ISL_454558:0.0003495143)30:2.427e-07,EPI_ISL_717967:0.0005929052)30:2.427e-
07,((((EPI_ISL_513060:2.425e-07,EPI_ISL_512905:2.425e-07)100:3.44148e-
05,EPI_ISL_513059:3.44147e-05)100:3.44162e-05,EPI_ISL_513058:2.425e-07)97:3.44065e-
05,EPI_ISL_513029:2.425e-07)97:3.44065e-05)30:2.427e-07,EPI_ISL_512972:3.44111e-05)30:2.427e-
07,EPI_ISL_513511:2.425e-07)30:2.427e-07,(EPI_ISL_513066:3.44114e-05,EPI_ISL_513030:2.425e-
07)55:3.44097e-05)30:2.427e-
07,(((EPI_ISL_603238:0.0001378243,EPI_ISL_603239:0.0002067083)88:3.43205e-
05,EPI_ISL_603240:0.0001376785)30:2.427e-07,EPI_ISL_603241:0.0002410771)31:3.44096e-
05)30:2.427e-07,EPI_ISL_525553:2.425e-07)30:2.427e-07,EPI_ISL_454602:0.0004166934)30:2.427e-
07,EPI_ISL_434572:2.425e-07)30:2.427e-07,(EPI_ISL_542152:2.425e-07,EPI_ISL_542154:2.425e-
07)100:3.469e-05)30:2.427e-07,EPI_ISL_490112:2.425e-07)30:2.427e-07,EPI_ISL_475602:6.88297e-
05)30:2.427e-07,(EPI_ISL_735263:3.48111e-05,EPI_ISL_735324:3.4804e-
05)100:0.0001392798)30:2.427e-07,(EPI_ISL_542201:3.47848e-
05,EPI_ISL_462990:0.0004529999)52:3.45329e-05)30:2.427e-07,EPI_ISL_513048:3.44077e-
05)30:2.427e-07,((((EPI_ISL_542173:3.47251e-05,EPI_ISL_471553:2.425e-07)30:2.427e-
07,EPI_ISL_471528:2.425e-07)30:2.427e-07,EPI_ISL_688781:2.425e-07)30:2.427e-
07,EPI_ISL_471547:6.97138e-05)32:3.45898e-05)30:2.427e-07,EPI_ISL_447749:7.12788e-05)30:2.427e-

07,EPI_ISL_469018:6.88218e-05)30:1.0213e-06,((EPI_ISL_513025:1.0213e-06,EPI_ISL_513196:1.0213e-06)89:3.23735e-05,(EPI_ISL_513039:3.44166e-05,EPI_ISL_512969:3.44166e-05)95:0.0001033081)30:2.976e-06)30:1.0213e-06)30:2.427e-07,(((EPI_ISL_528809:6.97142e-05,EPI_ISL_700200:0.0002777338)100:6.93412e-05,EPI_ISL_528814:0.0001395185)100:6.93637e-05,EPI_ISL_631775:3.5073e-05)100:3.46688e-05)30:2.427e-07,EPI_ISL_469023:7.07914e-05)30:2.427e-07,EPI_ISL_486818:6.96049e-05)30:2.427e-07,EPI_ISL_456142:2.425e-07)30:2.427e-07,EPI_ISL_574431:0.0001046327)30:2.427e-07,(((((((EPI_ISL_480244:2.425e-07,((((EPI_ISL_480249:2.425e-07,EPI_ISL_735297:6.96136e-05)30:2.427e-07,EPI_ISL_480264:2.425e-07)100:3.47988e-05,((EPI_ISL_480284:6.99677e-05,EPI_ISL_735336:2.425e-07)30:2.427e-07,EPI_ISL_735333:2.425e-07)100:3.47957e-05)36:2.427e-07,EPI_ISL_576275:6.88866e-05)30:2.427e-07)30:2.427e-07,EPI_ISL_576268:0.0003847613)30:2.427e-07,(EPI_ISL_732669:2.425e-07,EPI_ISL_717971:2.425e-07)100:0.000557979)30:2.427e-07,((EPI_ISL_596233:7.02573e-05,EPI_ISL_596234:2.425e-07)100:3.48772e-05,EPI_ISL_732661:0.0002437891)100:0.0001044307)30:2.427e-07,EPI_ISL_480268:3.47954e-05)30:2.427e-07,EPI_ISL_735303:6.96748e-05)30:2.427e-07,EPI_ISL_735327:0.0001044672)30:3.44407e-05)30:2.427e-07,EPI_ISL_717966:0.0002786452)30:2.427e-07,EPI_ISL_542178:3.48277e-05)30:2.427e-07,(EPI_ISL_512896:6.8833e-05,EPI_ISL_636965:2.425e-07)74:3.44089e-05)30:2.427e-07,((((((((((EPI_ISL_547968:2.425e-07,EPI_ISL_577637:6.9711e-05)30:2.427e-07,EPI_ISL_546936:2.425e-07)30:2.427e-07,(EPI_ISL_577627:2.425e-07,EPI_ISL_577628:2.425e-07)100:6.97173e-05)35:2.427e-07,EPI_ISL_626575:6.97251e-05)30:2.427e-07,EPI_ISL_626588:6.97267e-05)33:2.427e-07,EPI_ISL_546935:6.97109e-05)100:6.97171e-05,(EPI_ISL_545954:0,EPI_ISL_545957:0):2.425e-07)30:2.427e-07,EPI_ISL_545956:2.425e-07)100:0.0001046091,EPI_ISL_577633:3.48645e-05)100:6.97517e-05,EPI_ISL_545580:6.97283e-05)100:3.48507e-05,((EPI_ISL_539782:3.48548e-05,(EPI_ISL_541333:2.425e-07,EPI_ISL_541334:2.425e-07)100:3.48549e-05)100:6.97325e-05,EPI_ISL_541332:6.97113e-05)100:3.48697e-05)100:6.9734e-05)30:2.427e-07,((((((((((((EPI_ISL_515542:6.94468e-05,(EPI_ISL_515560:0.000104137,EPI_ISL_468315:2.425e-07)32:2.427e-07)100:6.94231e-05,((((((((((((((EPI_ISL_515548:3.47366e-05,((((((((EPI_ISL_515555:0,EPI_ISL_527860:0):0,EPI_ISL_468314:0):2.425e-07,((((((((((EPI_ISL_523955:0.0001042938,EPI_ISL_603028:3.47134e-05)33:3.31913e-05,EPI_ISL_527866:0.0001053149)20:1.0213e-06,EPI_ISL_583503:0.0001041939)73:3.4721e-05,EPI_ISL_523989:0.0001044212)30:2.427e-07,EPI_ISL_523984:0.000138998)30:2.427e-07,EPI_ISL_527857:3.47192e-05)30:2.427e-07,EPI_ISL_690635:2.425e-07)30:2.427e-07,EPI_ISL_471648:3.47318e-05)30:2.427e-07,EPI_ISL_603034:0.0001389912)30:2.427e-07,EPI_ISL_468321:2.425e-07)30:2.427e-07)30:2.427e-07,EPI_ISL_523982:2.425e-07)30:2.427e-07,EPI_ISL_534311:6.9485e-05)30:2.427e-07,EPI_ISL_534317:0.0001042275)30:2.427e-07,EPI_ISL_471546:2.425e-07)30:2.427e-07,EPI_ISL_523981:0.0001042377)30:2.427e-07)30:2.427e-07,EPI_ISL_547575:0.0001389968)30:3.47062e-05,((EPI_ISL_515551:3.4738e-05,EPI_ISL_534314:2.425e-07)30:2.427e-07,EPI_ISL_515564:3.4732e-05)30:2.427e-07)30:2.427e-07,EPI_ISL_471556:0.0001042235)30:2.427e-07,EPI_ISL_523970:0.000104104)30:3.46988e-05,(EPI_ISL_547576:8.33104e-05,EPI_ISL_603035:0.0001042476)100:0.0001250601)30:2.427e-07,EPI_ISL_534324:6.94686e-05)30:2.427e-07,EPI_ISL_515565:2.425e-07)30:2.427e-07,EPI_ISL_523967:6.94682e-05)30:2.427e-07,EPI_ISL_515552:2.425e-07)30:2.427e-

07,((EPI_ISL_471542:3.47241e-05,EPI_ISL_524466:0.0001739418)89:3.47237e-
05,(EPI_ISL_468319:6.94375e-05,EPI_ISL_574594:0.0001736867)30:2.427e-07)48:3.47029e-
05)30:2.427e-07,EPI_ISL_515562:0.0001042262)30:2.427e-07,EPI_ISL_523978:2.425e-07)30:2.427e-
07,EPI_ISL_527859:6.95649e-05)30:2.427e-07,EPI_ISL_603021:0.0001737587)30:2.427e-07)30:2.427e-
07,(EPI_ISL_468305:0.0002285027,EPI_ISL_603022:0.0001388382)61:7.63379e-05)30:7.63334e-
05,EPI_ISL_693198:3.44114e-05)30:2.427e-07,EPI_ISL_603027:0.0002084539)30:2.427e-
07,EPI_ISL_523985:3.4593e-05)30:2.427e-07,EPI_ISL_471582:6.94829e-05)30:2.427e-
07,EPI_ISL_583497:0.0002433146)30:2.427e-07,EPI_ISL_524463:6.96285e-05)30:2.427e-
07,EPI_ISL_523971:3.46675e-05)30:2.427e-07,EPI_ISL_534321:3.47303e-05)30:2.427e-
07,EPI_ISL_515547:2.425e-07)30:3.44143e-05,(EPI_ISL_525685:3.47308e-05,(EPI_ISL_458116:4.62792e-
05,EPI_ISL_492010:0.0001042342)100:4.63447e-05)99:4.63443e-05)30:2.427e-07)30:2.427e-
07,EPI_ISL_447779:3.48663e-05)30:2.427e-07,(((EPI_ISL_542440:2.425e-07,(EPI_ISL_542211:2.425e-
07,EPI_ISL_631732:2.425e-07)51:2.427e-07)46:2.427e-07,EPI_ISL_542110:3.47339e-05)47:2.427e-
07,EPI_ISL_542263:2.425e-07)100:0.0001034787)30:2.427e-07,EPI_ISL_542344:3.4732e-05)30:2.427e-
07,(EPI_ISL_475691:0.0001032675,((EPI_ISL_495423:7.00036e-05,EPI_ISL_735309:3.48601e-
05)100:3.48065e-05,(EPI_ISL_512846:0.0002435604,EPI_ISL_631767:3.48586e-05)31:2.427e-
07)36:2.427e-07)30:3.44157e-05)30:2.427e-07,EPI_ISL_542428:3.47182e-05)30:2.427e-
07,(EPI_ISL_542101:0.0001042541,(EPI_ISL_541336:3.49625e-05,EPI_ISL_541337:3.47581e-
05)100:0.0001744268)53:2.427e-07)30:2.427e-07,EPI_ISL_445080:3.45016e-05)30:2.427e-
07,EPI_ISL_525557:3.44253e-05)30:2.427e-07,EPI_ISL_542364:6.94717e-05)30:2.427e-
07,((EPI_ISL_542361:0.0002085148,EPI_ISL_542306:2.425e-07)100:2.427e-
07,EPI_ISL_626590:0.0003140718)70:3.45925e-05)30:2.427e-07,((((((((EPI_ISL_480253:2.425e-
07,(EPI_ISL_480248:2.425e-07,EPI_ISL_480262:2.425e-07)100:3.47991e-05)30:2.427e-
07,EPI_ISL_480232:6.9614e-05)30:2.427e-07,EPI_ISL_735305:6.962e-05)30:2.427e-
07,(EPI_ISL_735296:3.49217e-05,EPI_ISL_735313:6.96125e-05)100:0.0001044359)30:2.427e-
07,EPI_ISL_480281:3.48895e-05)30:2.427e-07,EPI_ISL_480282:3.47994e-05)32:2.427e-
07,EPI_ISL_735337:2.425e-07)33:2.427e-07,EPI_ISL_735329:3.48537e-05)44:3.48021e-05)30:2.427e-
07,(EPI_ISL_542389:0.0001042249,(EPI_ISL_542206:2.425e-07,EPI_ISL_542209:2.425e-
07)100:3.47321e-05)100:3.47321e-05)30:2.427e-07,(((((EPI_ISL_486824:3.48031e-
05,((EPI_ISL_542363:2.425e-
07,(((EPI_ISL_534337:0.0001044222,(EPI_ISL_610244:0.0003485237,(EPI_ISL_732670:2.425e-
07,EPI_ISL_717973:2.425e-07)100:0.0002438752)41:2.427e-07)41:2.427e-
07,(EPI_ISL_626615:3.48557e-05,EPI_ISL_693675:2.425e-07)100:0.0002442909)100:6.96165e-
05,((EPI_ISL_539778:2.425e-07,EPI_ISL_539779:3.48553e-05)100:3.48598e-
05,(EPI_ISL_513512:3.48553e-05,(EPI_ISL_626595:3.48323e-
05,EPI_ISL_693681:0.0001046493)100:0.0001395547)47:2.427e-07)50:2.427e-07)31:2.427e-
07)30:2.427e-07,EPI_ISL_737026:0.0004891981)30:2.427e-07)30:2.427e-07,EPI_ISL_542145:3.47298e-
05)30:2.427e-07,EPI_ISL_542358:2.425e-07)30:2.427e-07,(EPI_ISL_542349:0,EPI_ISL_542317:0):2.425e-
07)30:2.427e-07,EPI_ISL_486827:3.48e-05)53:3.459e-05)30:2.427e-07,EPI_ISL_419663:3.53612e-
05)30:2.427e-07,EPI_ISL_685003:2.425e-07)30:2.427e-07,EPI_ISL_542319:2.425e-07)30:2.427e-
07,((EPI_ISL_603221:0.0002758227,EPI_ISL_603222:0.0002758244)100:0.0001376459,(EPI_ISL_653931:
2.425e-07,EPI_ISL_732659:0.0003135354)100:0.0001741103)30:2.427e-07)30:2.427e-
07,EPI_ISL_732663:0.0002438023)30:2.427e-07,(((((EPI_ISL_690670:6.97834e-
05,EPI_ISL_690673:3.48878e-05)30:2.427e-

07,((EPI_ISL_690672:0,EPI_ISL_690665:0):0,EPI_ISL_690664:0):2.425e-07)30:2.427e-
07,EPI_ISL_690666:2.425e-07)30:2.427e-07,EPI_ISL_690669:2.445e-07)30:2.446e-
07,EPI_ISL_690680:2.425e-07)100:0.0001378605)30:2.427e-
07,EPI_ISL_732667:0.0004532969)30:2.427e-07,(EPI_ISL_542354:2.425e-
07,((EPI_ISL_542377:0.0001042304,EPI_ISL_542321:3.46955e-05)95:3.46955e-
05,EPI_ISL_542388:0.0001042418)30:2.427e-07)93:3.46932e-05)30:2.427e-07,((EPI_ISL_524733:2.425e-
07,EPI_ISL_524734:3.4848e-05)100:0.0002443173,EPI_ISL_699778:0.0002785707)100:3.45731e-
05)30:2.427e-07,(((EPI_ISL_542371:0,EPI_ISL_542384:0):2.425e-07,EPI_ISL_542392:3.4732e-
05)30:2.427e-07,EPI_ISL_542383:2.425e-07)100:6.94768e-05)30:2.427e-
07,((((((((((((((EPI_ISL_513054:0,EPI_ISL_512893:0):0,EPI_ISL_513036:0):0,EPI_ISL_513008:0):0,EPI_ISL_
513049:0):0,EPI_ISL_512889:0):2.425e-07,((((EPI_ISL_513014:0,EPI_ISL_513019:0):2.425e-
07,EPI_ISL_513017:2.425e-07)100:3.44194e-05,EPI_ISL_513027:3.44191e-05)100:3.4415e-
05,EPI_ISL_512886:6.8835e-05)34:2.427e-07)30:2.427e-07,EPI_ISL_513069:0.0001032624)30:2.427e-
07,EPI_ISL_513045:3.44187e-05)30:2.427e-07,EPI_ISL_513068:6.88504e-05)30:2.427e-
07,EPI_ISL_512986:3.44166e-05)30:2.427e-07,EPI_ISL_512885:3.44166e-05)30:2.427e-
07,EPI_ISL_512876:0.0001032715)30:2.427e-07,EPI_ISL_513055:2.425e-07)30:2.427e-
07,EPI_ISL_513057:6.88346e-05)30:2.427e-07,EPI_ISL_513037:3.44187e-05)30:3.44186e-05)30:2.427e-
07,EPI_ISL_486817:2.425e-07)30:2.427e-07,EPI_ISL_513035:2.425e-07)30:2.427e-
07,(((((((((((((((((((((((((((EPI_ISL_515520:6.945e-05,((EPI_ISL_523977:3.47656e-
05,(((EPI_ISL_527870:2.425e-07,EPI_ISL_583498:8.68317e-05)100:8.68869e-
05,((EPI_ISL_524465:2.425e-07,EPI_ISL_524469:0.0001042609)30:2.427e-07,EPI_ISL_468316:3.46378e-
05)30:2.427e-07)30:2.427e-07,EPI_ISL_471549:6.94971e-05)58:2.427e-07)51:3.46308e-
05,((EPI_ISL_523983:3.47377e-05,(((EPI_ISL_527862:6.94346e-05,((EPI_ISL_471562:6.94643e-
05,EPI_ISL_693207:0.0001390031)74:3.4678e-05,EPI_ISL_524462:2.425e-07)30:2.427e-07)30:2.427e-
07,EPI_ISL_603030:0.0001389536)30:2.427e-07,EPI_ISL_583502:6.94307e-05)30:2.427e-07)30:2.427e-
07,(EPI_ISL_468312:2.425e-07,(EPI_ISL_583504:6.95065e-05,EPI_ISL_583505:4.85713e-
05)100:0.0001457678)81:4.86022e-05)30:2.427e-07)30:2.427e-07)30:2.427e-
07,(EPI_ISL_547573:0.0001390826,EPI_ISL_583500:6.9491e-05)87:3.46998e-05)30:2.427e-
07,(EPI_ISL_534322:0.0001390857,EPI_ISL_693206:6.94657e-05)92:3.46865e-05)30:2.427e-
07,(EPI_ISL_534319:2.425e-07,(EPI_ISL_547579:0.0001043188,(EPI_ISL_603024:2.425e-
07,EPI_ISL_603038:0.0002085959)30:2.427e-07)100:6.9418e-05)97:3.47101e-05)30:2.427e-
07,(EPI_ISL_515561:2.425e-07,EPI_ISL_527861:6.94869e-05)99:3.46899e-05)30:2.427e-
07,EPI_ISL_515563:6.94896e-05)30:2.427e-07,EPI_ISL_603033:0.0001389612)30:2.427e-
07,EPI_ISL_690818:2.425e-07)30:2.427e-07,EPI_ISL_523988:0.0001043478)30:2.427e-
07,EPI_ISL_527865:6.94554e-05)30:2.427e-07,(EPI_ISL_523958:6.9266e-
05,EPI_ISL_693211:0.0003124082)99:3.46505e-05)30:2.427e-
07,EPI_ISL_693208:0.000172571)30:2.427e-07,EPI_ISL_693212:0.000243177)30:2.427e-
07,EPI_ISL_603037:0.0001041822)30:2.427e-07,EPI_ISL_515559:3.47237e-05)30:2.427e-
07,EPI_ISL_693196:0.0001033811)30:2.427e-07,EPI_ISL_693209:0.0001387965)30:2.427e-
07,EPI_ISL_523986:2.425e-07)30:2.427e-07,EPI_ISL_515524:2.425e-07)30:2.427e-
07,EPI_ISL_583496:6.93086e-05)30:2.427e-07,EPI_ISL_603036:0.000104158)30:3.44512e-
05,((((((EPI_ISL_515526:2.425e-07,EPI_ISL_471581:3.47244e-05)85:3.47243e-
05,(((((((((EPI_ISL_515544:2.425e-07,EPI_ISL_523975:2.425e-07)81:3.47202e-
05,((((EPI_ISL_515554:2.425e-07,EPI_ISL_603023:0.0001042511)30:2.427e-

07,EPI_ISL_523965:3.47407e-05)30:2.427e-07,((EPI_ISL_523990:2.425e-07,((EPI_ISL_471541:3.47051e-05,EPI_ISL_574597:0.0001042639)30:2.427e-07,EPI_ISL_471647:3.47237e-05)30:2.427e-07)30:2.427e-07,EPI_ISL_534316:6.9479e-05)30:2.427e-07)30:3.4709e-05,((EPI_ISL_524468:0.0001044555,EPI_ISL_693204:0.0002168549)100:4.32834e-05,EPI_ISL_534326:0.0001042769)31:4.33632e-05)30:2.446e-07)30:2.446e-07,EPI_ISL_693199:3.47155e-05)30:4.3359e-05,EPI_ISL_471548:6.94872e-05)30:2.446e-07,((((((EPI_ISL_515566:3.47204e-05,(EPI_ISL_527856:6.9078e-05,EPI_ISL_534320:3.4705e-05)30:2.427e-07)30:2.427e-07,EPI_ISL_471552:3.47251e-05)30:2.427e-07,EPI_ISL_471545:2.425e-07)36:3.45359e-05,((EPI_ISL_527868:0.0001044569,EPI_ISL_574598:0.0001736871)30:2.427e-07,EPI_ISL_524464:3.47231e-05)30:2.427e-07)30:2.427e-07,EPI_ISL_468311:3.47354e-05)30:2.427e-07,EPI_ISL_523957:0.0001390314)30:2.427e-07,EPI_ISL_523969:2.425e-07)93:6.90687e-05)30:2.427e-07,EPI_ISL_471539:3.47244e-05)30:2.427e-07,EPI_ISL_534318:6.9588e-05)30:2.427e-07,EPI_ISL_515546:3.47277e-05)30:2.427e-07,(EPI_ISL_523980:2.425e-07,EPI_ISL_468313:2.425e-07)65:3.46771e-05)30:2.427e-07)30:2.427e-07,(EPI_ISL_515529:0,EPI_ISL_523974:0):2.425e-07)30:2.427e-07,EPI_ISL_515541:2.425e-07)30:2.427e-07,EPI_ISL_583499:0.0001041952)30:3.45375e-05,EPI_ISL_515545:3.47091e-05)30:2.427e-07)30:2.427e-07,EPI_ISL_515553:3.47202e-05)30:2.427e-07,EPI_ISL_527863:3.47354e-05)30:2.427e-07,EPI_ISL_468308:3.46927e-05)30:2.427e-07,(EPI_ISL_523993:6.94929e-05,EPI_ISL_468307:3.47034e-05)98:3.47131e-05)30:2.427e-07,EPI_ISL_468318:3.46966e-05)30:2.427e-07,EPI_ISL_693210:0.000104154)30:3.44471e-05)30:2.427e-07,(EPI_ISL_491093:3.48602e-05,EPI_ISL_530347:2.425e-07)100:6.97359e-05)30:2.427e-07,EPI_ISL_512966:3.44199e-05)30:2.427e-07,(EPI_ISL_614396:2.425e-07,EPI_ISL_614397:2.425e-07)100:6.94917e-05)30:2.427e-07,((((((((((EPI_ISL_515521:2.425e-07,((((((((((((((((((((EPI_ISL_515522:0,EPI_ISL_515527:0):2.425e-07,(((((EPI_ISL_515525:2.777e-07,(EPI_ISL_527864:0.0001042288,EPI_ISL_527869:2.425e-07)77:3.47132e-05)61:0.0002759221,EPI_ISL_534323:6.95317e-05)11:3.9414e-06,EPI_ISL_492041:6.94658e-05)9:1.0213e-06,(EPI_ISL_603039:0.0002087219,EPI_ISL_693213:3.46758e-05)95:6.94869e-05)7:1.0213e-06,(((EPI_ISL_523976:0.0001041847,(EPI_ISL_534313:0.0001735935,((EPI_ISL_547580:2.425e-07,(EPI_ISL_603026:3.47308e-05,EPI_ISL_603032:3.46721e-05)100:3.46675e-05)100:0.0001735105,EPI_ISL_603031:0.0002435842)92:6.93277e-05)29:2.427e-07)31:2.073e-06,(EPI_ISL_603025:6.06841e-05,EPI_ISL_613708:0.0001047398)35:4.74105e-05)6:3.0392e-06,EPI_ISL_729849:0.0002469087)6:3.08976e-05)30:1.0213e-06)30:2.427e-07,(((EPI_ISL_524467:6.95976e-05,EPI_ISL_603029:0.0001042541)31:2.427e-07,EPI_ISL_524470:2.425e-07)30:2.427e-07,EPI_ISL_534312:0.0001738165)99:6.94934e-05)30:2.427e-07,EPI_ISL_471554:2.425e-07)30:2.427e-07,EPI_ISL_527867:2.425e-07)30:2.427e-07,EPI_ISL_574596:0.0001040373)30:2.427e-07,EPI_ISL_523959:3.47277e-05)30:2.427e-07,EPI_ISL_534325:0.000104254)30:3.44671e-05,EPI_ISL_523991:6.94295e-05)30:2.427e-07,EPI_ISL_515550:2.425e-07)30:2.427e-07,EPI_ISL_583495:0.0001737264)30:2.427e-07,EPI_ISL_684859:3.48968e-05)30:2.427e-07,EPI_ISL_515528:2.425e-07)30:2.427e-07,EPI_ISL_523973:6.94552e-05)30:2.427e-07,EPI_ISL_547570:3.47407e-05)30:2.427e-07,EPI_ISL_547574:0.0001730653)30:2.427e-07,EPI_ISL_471551:3.47438e-05)30:2.427e-07,EPI_ISL_534315:0.0002082426)30:2.427e-07,EPI_ISL_523956:6.94908e-05)30:2.427e-07,EPI_ISL_515543:3.47274e-05)30:2.427e-07,EPI_ISL_515523:2.425e-07)30:2.427e-07)30:2.427e-07,EPI_ISL_523992:6.94701e-05)30:2.427e-07,EPI_ISL_515557:3.47443e-05)30:2.427e-07,EPI_ISL_527858:6.9492e-05)30:2.427e-

07,EPI_ISL_547571:6.95678e-05)30:2.427e-07,EPI_ISL_468310:6.94009e-05)30:2.427e-
07,EPI_ISL_722899:0.0001377808)30:2.427e-07,EPI_ISL_729800:0.000104559)85:3.44353e-
05,EPI_ISL_547577:0.0001738361)85:3.44353e-05,(((((((((((EPI_ISL_569885:6.95097e-
05,(((((((((((((((EPI_ISL_686902:3.48647e-05,(EPI_ISL_686881:2.425e-07,EPI_ISL_692398:2.425e-
07)30:2.427e-07)30:2.427e-07,((EPI_ISL_686886:2.425e-07,EPI_ISL_686887:2.425e-07)100:2.427e-
07,EPI_ISL_686890:2.425e-07)79:3.47915e-05)30:2.427e-07,((EPI_ISL_686882:6.98012e-
05,((EPI_ISL_686883:2.425e-07,(EPI_ISL_686892:2.425e-07,EPI_ISL_686895:3.48078e-05)40:2.427e-
07)30:2.427e-07,EPI_ISL_686899:2.425e-07)30:2.427e-07)30:2.427e-07,EPI_ISL_686885:2.425e-
07)54:3.48094e-05)30:2.427e-07,EPI_ISL_686891:2.425e-07)30:2.427e-07,(((EPI_ISL_689409:2.425e-
07,EPI_ISL_692407:2.425e-07)31:2.427e-07,EPI_ISL_689400:2.425e-07)30:2.427e-
07,EPI_ISL_689401:2.425e-07)100:3.48276e-05)30:2.427e-
07,((((((EPI_ISL_686888:0,EPI_ISL_692320:0):0,EPI_ISL_686897:0):0,EPI_ISL_686893:0):0,EPI_ISL_692322
:0):0,EPI_ISL_686898:0):0,EPI_ISL_692325:0):2.425e-07)30:2.427e-07,EPI_ISL_692217:2.425e-
07)30:2.427e-07,EPI_ISL_686894:2.425e-07)30:2.427e-07,EPI_ISL_692321:2.425e-07)30:2.427e-
07,EPI_ISL_686896:2.425e-07)30:2.427e-07,EPI_ISL_692324:2.425e-07)30:2.427e-
07,EPI_ISL_692405:2.425e-07)30:2.427e-07,EPI_ISL_686884:2.425e-07)100:3.47928e-
05,EPI_ISL_691277:2.425e-07)100:0.0003832078,EPI_ISL_687718:0.0002795382)99:3.47756e-
05)30:2.427e-
07,(EPI_ISL_653782:0.0001378042,((EPI_ISL_653787:0.0001743667,EPI_ISL_653789:2.425e-
07)100:2.427e-07,EPI_ISL_653790:0.0001741267)99:6.89021e-05)100:0.0001377078)30:2.427e-
07,(((EPI_ISL_626580:2.425e-07,((EPI_ISL_626585:3.4865e-
05,((EPI_ISL_626591:0.0001046394,EPI_ISL_626606:2.425e-07)100:6.97348e-
05,EPI_ISL_693687:2.425e-07)30:2.427e-07)100:3.48609e-05,EPI_ISL_626605:2.425e-07)31:2.427e-
07)31:2.427e-07,EPI_ISL_722871:3.45491e-05)30:2.427e-07,EPI_ISL_737027:6.97346e-
05)100:0.0001036802)30:2.427e-07,((EPI_ISL_626579:6.97373e-
05,(EPI_ISL_584082:0.0001046115,EPI_ISL_693682:0.0001744406)31:2.427e-07)43:2.427e-
07,EPI_ISL_660555:0.0001046174)100:0.0002792566)30:2.427e-07,EPI_ISL_542156:3.47354e-
05)30:2.427e-07,(EPI_ISL_542367:2.425e-07,EPI_ISL_542339:2.425e-07)100:6.94772e-05)30:2.427e-
07,EPI_ISL_452140:2.425e-07)30:2.427e-07,EPI_ISL_452150:3.4738e-05)30:2.427e-
07,EPI_ISL_576266:0.0002411446)30:2.427e-07,EPI_ISL_530350:2.425e-
07)100:0.0001033354,EPI_ISL_722855:0.0001740939)100:3.43779e-05)30:2.427e-07)30:2.427e-
07,(((EPI_ISL_560408:3.48683e-05,((EPI_ISL_560409:3.48638e-05,EPI_ISL_560411:2.425e-07)31:2.427e-
07,(EPI_ISL_560410:0,EPI_ISL_560412:0):2.425e-07)99:2.427e-
07)100:0.0001045874,(EPI_ISL_626586:0.0001395252,((EPI_ISL_653784:2.425e-
07,(EPI_ISL_653785:0.0003841049,(EPI_ISL_653792:3.44379e-05,(EPI_ISL_653794:2.425e-
07,EPI_ISL_653821:3.44641e-05)100:2.427e-07)100:0.0001033628)99:2.427e-07)100:6.88844e-
05,EPI_ISL_577634:2.425e-07)31:2.427e-07)32:2.446e-07)79:0.0001033797,EPI_ISL_653814:6.89908e-
05)100:0.0002413327)30:2.427e-07,((EPI_ISL_653763:3.49389e-
05,((((((((((((((((EPI_ISL_653764:0.0001390586,((((EPI_ISL_722852:3.46062e-05,(EPI_ISL_722867:2.425e-
07,((EPI_ISL_722874:3.44498e-05,EPI_ISL_722906:3.44834e-05)100:2.427e-
07,EPI_ISL_722903:0.0001033069)100:6.88583e-05)30:2.427e-07)30:2.427e-07,EPI_ISL_722860:2.425e-
07)30:2.427e-07,EPI_ISL_722900:2.425e-07)30:2.427e-07,EPI_ISL_722862:3.44212e-05)30:2.427e-
07)30:2.427e-07,EPI_ISL_722864:3.44239e-05)30:2.427e-07,((EPI_ISL_722868:2.425e-
07,EPI_ISL_722870:2.425e-07)30:2.427e-07,EPI_ISL_722869:2.425e-07)100:3.44497e-05)30:2.427e-

07,EPI_ISL_722851:2.425e-07)30:2.427e-07,EPI_ISL_722866:0.0001036778)30:2.427e-
07,EPI_ISL_722853:2.425e-07)30:2.427e-07,EPI_ISL_653805:3.44212e-05)30:2.427e-
07,EPI_ISL_722854:3.44278e-05)30:2.427e-07,EPI_ISL_722861:2.425e-07)30:2.427e-
07,(EPI_ISL_653802:3.44533e-05,EPI_ISL_653803:2.425e-07)100:6.89058e-05)30:2.427e-
07,EPI_ISL_722898:3.44492e-05)30:2.427e-07,EPI_ISL_653791:0.0002066894)30:2.427e-
07,EPI_ISL_649938:0.0004162617)30:2.427e-07,EPI_ISL_722859:2.425e-07)30:2.427e-
07,EPI_ISL_649939:0.0001390079)30:2.427e-07,EPI_ISL_722863:3.45399e-05)30:2.427e-07)32:2.427e-
07,EPI_ISL_722865:3.44276e-05)100:0.0002412115)34:3.44245e-05,EPI_ISL_486823:3.481e-
05)30:2.427e-07,EPI_ISL_577635:2.425e-07)34:3.44306e-05,EPI_ISL_437300:0.000137817)30:3.44298e-
05,EPI_ISL_542279:0.0001042752)30:2.427e-07,EPI_ISL_479999:6.9631e-05)30:2.427e-
07,EPI_ISL_542257:3.47399e-05)30:2.427e-07,EPI_ISL_480316:2.425e-07)30:2.427e-
07,EPI_ISL_431102:2.425e-07)30:2.427e-07,EPI_ISL_542216:6.94981e-05)30:2.427e-
07,EPI_ISL_542316:3.48051e-05)30:2.427e-07,EPI_ISL_476896:6.99329e-05)30:2.427e-
07,EPI_ISL_735361:6.97499e-05)30:2.427e-07,EPI_ISL_542233:2.425e-07)30:2.427e-
07,EPI_ISL_636966:2.425e-07)30:2.427e-07,EPI_ISL_475690:0.0001377511)30:2.427e-
07,EPI_ISL_542322:3.47401e-05)30:2.427e-07,EPI_ISL_685990:2.425e-07)30:2.427e-
07,(EPI_ISL_542391:2.425e-07,EPI_ISL_542334:2.425e-07)100:3.47399e-05)30:2.427e-
07,EPI_ISL_690708:2.425e-07)30:2.427e-07,EPI_ISL_542225:2.425e-07)30:2.427e-
07,EPI_ISL_542387:6.94861e-05)30:2.427e-07,((EPI_ISL_542369:0,EPI_ISL_542342:0):2.425e-
07,EPI_ISL_542307:2.425e-07)100:3.47399e-05)30:2.427e-07,(EPI_ISL_479516:2.425e-
07,(EPI_ISL_452195:6.95027e-05,EPI_ISL_452196:3.4751e-05)100:6.95032e-05)100:3.47474e-
05)30:2.427e-07,EPI_ISL_444477:0.0001044621)30:2.427e-07,EPI_ISL_542217:3.47401e-05)30:2.427e-
07,EPI_ISL_577629:6.97445e-05)30:2.427e-07,EPI_ISL_542208:3.47401e-05)30:2.427e-
07,EPI_ISL_479937:3.48966e-05)30:2.427e-07,EPI_ISL_542252:3.47444e-05)30:2.427e-
07,EPI_ISL_542230:2.425e-07)30:2.427e-07,EPI_ISL_542297:6.94091e-05)30:2.427e-
07,EPI_ISL_542335:3.47773e-05)30:2.427e-07,EPI_ISL_542254:6.94868e-05)30:2.427e-
07,EPI_ISL_542314:0.0001390259)30:2.427e-07,EPI_ISL_542205:3.47402e-05)30:2.427e-
07,(EPI_ISL_631562:3.51475e-05,EPI_ISL_653795:0.0007688731)42:3.47207e-05)30:2.427e-
07,(EPI_ISL_526949:3.48518e-05,EPI_ISL_526950:0.0001055535)100:0.0001047265)30:2.427e-
07,EPI_ISL_498163:2.425e-07)30:2.427e-07,(EPI_ISL_569880:3.47521e-
05,EPI_ISL_653757:0.0002463859)72:3.47519e-05)30:2.427e-07,EPI_ISL_542226:3.4741e-05)30:2.427e-
07,EPI_ISL_685989:3.49705e-05)30:2.427e-07,EPI_ISL_542423:3.47386e-05)30:2.427e-
07,EPI_ISL_542239:2.425e-07)30:2.427e-07,EPI_ISL_586564:0.0001394759)30:2.427e-
07,EPI_ISL_498159:2.425e-07)30:2.427e-07,EPI_ISL_542221:3.47411e-05)30:2.427e-
07,(EPI_ISL_542286:2.425e-07,EPI_ISL_542289:2.425e-07)100:3.47455e-05)30:2.427e-
07,EPI_ISL_542153:2.425e-07)30:2.427e-07,EPI_ISL_584080:0.0001395044)30:2.427e-
07,EPI_ISL_476067:3.48667e-05)30:2.427e-07,(EPI_ISL_684884:2.425e-07,EPI_ISL_684885:2.425e-
07)100:0.0001047373)30:2.427e-07,EPI_ISL_483874:6.94694e-05)30:2.427e-07,EPI_ISL_495419:2.425e-
07)30:2.427e-07,EPI_ISL_542352:6.94891e-05)30:2.427e-07,EPI_ISL_542299:3.4746e-05)30:2.427e-
07,EPI_ISL_542435:3.47412e-05)30:2.427e-07,EPI_ISL_495062:0.000104487)30:2.427e-
07,EPI_ISL_542429:3.47443e-05)30:2.427e-07,EPI_ISL_542243:3.47465e-05)30:2.427e-
07,EPI_ISL_501169:3.45428e-05)30:2.427e-
07,(((((((EPI_ISL_513052:0.0001377512,(EPI_ISL_513063:6.88713e-05,EPI_ISL_513200:3.443e-
05)58:3.4426e-05)34:2.427e-07,(EPI_ISL_513211:2.425e-07,EPI_ISL_513197:0.0001033035)30:2.427e-

07)30:3.4428e-05,EPI_ISL_513097:6.88685e-05)30:2.427e-07,EPI_ISL_513201:3.44265e-05)30:2.427e-07,EPI_ISL_513206:6.88534e-05)30:3.44278e-05,(EPI_ISL_437302:0.0001378502,EPI_ISL_688084:0.0001397533)100:6.8854e-05)30:2.427e-07,EPI_ISL_461494:0.000103922)30:3.44276e-05,((EPI_ISL_542114:0,EPI_ISL_542183:0):2.425e-07,(EPI_ISL_542128:3.47444e-05,EPI_ISL_542169:2.425e-07)30:2.427e-07)100:3.47412e-05)30:2.427e-07)30:2.427e-07,(((EPI_ISL_542255:2.425e-07,EPI_ISL_542277:3.47451e-05)30:2.427e-07,EPI_ISL_542331:2.425e-07)100:2.427e-07,EPI_ISL_542116:3.47308e-05)100:3.47054e-05)30:2.427e-07,EPI_ISL_542379:0.00010427)30:2.427e-07,EPI_ISL_542274:3.47415e-05)30:2.427e-07,EPI_ISL_469019:0.0001033094)30:2.427e-07,EPI_ISL_454532:3.48261e-05)30:2.427e-07,EPI_ISL_542366:0.0001042552)30:2.427e-07,EPI_ISL_542350:0.0001042689)30:2.427e-07,EPI_ISL_525467:2.425e-07)30:2.427e-07,(EPI_ISL_542292:2.425e-07,EPI_ISL_684361:3.48717e-05)100:3.4602e-05)30:2.427e-07,EPI_ISL_542313:2.425e-07)30:2.427e-07,EPI_ISL_542281:6.94906e-05)30:2.427e-07,(EPI_ISL_631605:3.50787e-05,EPI_ISL_690832:0.0001047435)99:3.48683e-05)30:2.427e-07,EPI_ISL_542419:2.425e-07)30:2.427e-07,EPI_ISL_498167:2.425e-07)30:2.427e-07,EPI_ISL_542234:3.47454e-05)30:2.427e-07,(EPI_ISL_542325:6.9516e-05,EPI_ISL_685242:3.49059e-05)65:3.4609e-05)30:2.427e-07,EPI_ISL_542291:3.47457e-05)30:2.427e-07,(EPI_ISL_542359:3.4743e-05,EPI_ISL_542385:0.0001042495)99:3.47428e-05)30:2.427e-07,EPI_ISL_542360:0.0001042562)30:2.427e-07,(((((((((((((((((((((((EPI_ISL_542375:0,EPI_ISL_542253:0):0,EPI_ISL_542213:0):0,EPI_ISL_542124:0):0,EPI_ISL_542133:0):0,EPI_ISL_542278:0):0,EPI_ISL_542276:0):0,EPI_ISL_542333:0):0,EPI_ISL_542330:0):0,EPI_ISL_542159:0):0,EPI_ISL_542324:0):0,EPI_ISL_542202:0):0,EPI_ISL_542220:0):0,EPI_ISL_542104:0):0,EPI_ISL_542264:0):0,EPI_ISL_542223:0):0,EPI_ISL_542261:0):0,EPI_ISL_542224:0):0,EPI_ISL_542309:0):0,EPI_ISL_542327:0):0,EPI_ISL_542310:0):0,EPI_ISL_542165:0):0,EPI_ISL_542107:0):0,EPI_ISL_542181:0):2.425e-07)30:2.427e-07,EPI_ISL_483866:6.94091e-05)30:2.427e-07,EPI_ISL_542231:2.425e-07)30:2.427e-07,EPI_ISL_542232:6.97525e-05)30:2.427e-07,((EPI_ISL_542373:2.425e-07,(EPI_ISL_542374:3.47425e-05,EPI_ISL_542390:2.425e-07)30:2.427e-07)100:6.94975e-05,EPI_ISL_542228:2.425e-07)100:3.47029e-05)30:2.427e-07,(EPI_ISL_524760:2.425e-07,EPI_ISL_524761:3.4856e-05)100:0.0001394812)30:2.427e-07,EPI_ISL_542365:6.95818e-05)30:2.427e-07,(EPI_ISL_542280:3.47125e-05,EPI_ISL_542303:6.95062e-05)98:3.47032e-05)30:2.427e-07,(EPI_ISL_469021:2.425e-07,EPI_ISL_469022:2.425e-07)100:3.44245e-05)30:2.427e-07,EPI_ISL_542288:0.0001042475)30:2.427e-07,EPI_ISL_542282:6.94978e-05)30:2.427e-07,EPI_ISL_542381:0.0001042609)30:2.427e-07,EPI_ISL_542442:3.47427e-05)30:2.427e-07,EPI_ISL_685988:2.425e-07)30:2.427e-07,EPI_ISL_542329:0.0001042705)30:2.427e-07,(EPI_ISL_471543:3.47346e-05,(((((EPI_ISL_542302:2.425e-07,EPI_ISL_686039:2.425e-07)30:2.427e-07,(EPI_ISL_684435:2.425e-07,EPI_ISL_684436:2.425e-07)100:3.48997e-05)30:2.427e-07,EPI_ISL_542340:6.94979e-05)31:2.427e-07,EPI_ISL_542197:2.425e-07)31:2.427e-07,EPI_ISL_684421:6.98297e-05)30:2.427e-07)100:3.46027e-05)30:2.427e-07,(((EPI_ISL_541335:0,EPI_ISL_577631:0):2.425e-07,EPI_ISL_577630:2.425e-07)100:2.427e-07,EPI_ISL_577632:2.425e-07)100:6.96369e-05)30:2.427e-07,(((EPI_ISL_542196:2.425e-07,EPI_ISL_542174:2.425e-07)100:2.427e-07,EPI_ISL_542203:3.4743e-05)99:3.47435e-05,EPI_ISL_542204:3.47469e-05)44:3.47466e-05)30:2.427e-07,(((EPI_ISL_495038:6.9466e-05,EPI_ISL_495039:0.0001042194)93:6.94659e-05,(EPI_ISL_469037:3.4719e-05,((((EPI_ISL_626582:2.425e-07,((EPI_ISL_626620:2.425e-07,EPI_ISL_626621:2.425e-07)100:3.48684e-05,EPI_ISL_693669:6.97438e-05)58:2.427e-07)34:2.427e-07,EPI_ISL_693666:6.97525e-05)36:2.427e-

07,EPI_ISL_626583:6.97525e-05)33:2.427e-07,EPI_ISL_577636:3.48683e-
05)100:0.0001395681)30:2.427e-07)30:2.427e-07,(EPI_ISL_495061:6.96436e-
05,EPI_ISL_469034:6.94663e-05)100:6.94537e-05)44:3.47189e-05)30:2.427e-
07,EPI_ISL_471267:3.48705e-05)30:2.427e-07,(EPI_ISL_542347:3.4748e-05,EPI_ISL_542308:2.425e-
07)100:3.47432e-05)30:2.427e-07,((EPI_ISL_542443:3.47428e-05,(EPI_ISL_542227:3.51015e-
05,EPI_ISL_542285:3.47438e-05)31:2.427e-07)74:2.427e-
07,(EPI_ISL_541082:0.0001046278,EPI_ISL_577626:0.0002094324)100:0.0001046969)74:3.46076e-
05)30:2.427e-07,EPI_ISL_513047:6.88659e-05)30:2.427e-07,(EPI_ISL_542229:3.4789e-
05,EPI_ISL_542312:2.425e-07)100:3.47435e-05)30:2.427e-07,EPI_ISL_542336:6.95002e-05)30:2.427e-
07,((((((EPI_ISL_626587:3.48812e-05,EPI_ISL_626617:3.48812e-05)39:2.427e-
07,EPI_ISL_626603:2.425e-07)30:2.427e-07,EPI_ISL_626608:3.48812e-05)30:2.427e-
07,(EPI_ISL_737029:3.48811e-05,(EPI_ISL_737013:3.48841e-05,EPI_ISL_693667:3.48812e-05)83:2.427e-
07)100:3.48809e-05)37:2.427e-07,EPI_ISL_577638:6.97752e-
05)100:0.0004193014,(EPI_ISL_584075:6.97499e-
05,(EPI_ISL_626604:0.0002094447,EPI_ISL_693678:0.0002793467)100:0.0001046547)99:3.4873e-
05)97:3.48714e-05)30:2.427e-07,EPI_ISL_477193:0.0001034643)30:2.427e-07,((EPI_ISL_516948:2.425e-
07,EPI_ISL_684506:6.98175e-05)100:2.427e-07,EPI_ISL_480003:6.96523e-05)99:3.48167e-
05)30:2.427e-07,EPI_ISL_542382:2.425e-07)30:2.427e-07,(((((((((((((((((((((((((((((EPI_ISL_480180:2.425e-
07,(EPI_ISL_480184:2.425e-07,EPI_ISL_480189:3.49017e-05)94:2.427e-07)94:2.427e-
07,EPI_ISL_480023:2.425e-07)100:3.48188e-05,(EPI_ISL_480000:3.49017e-
05,(((EPI_ISL_684444:2.425e-07,(EPI_ISL_685734:2.425e-07,(EPI_ISL_685736:2.425e-
07,(EPI_ISL_685738:2.425e-07,EPI_ISL_684776:2.425e-07)100:3.4902e-05)30:2.427e-07)30:2.427e-
07)31:2.427e-07,EPI_ISL_685735:2.425e-07)70:2.427e-07,EPI_ISL_685737:0.000104498)100:3.48014e-
05)30:2.427e-07)30:2.427e-07,EPI_ISL_684992:3.47971e-05)30:2.427e-
07,(EPI_ISL_480029:0,EPI_ISL_686031:0):2.425e-07)30:2.427e-07,EPI_ISL_691029:2.425e-07)30:2.427e-
07,EPI_ISL_691032:2.425e-07)30:2.427e-07,EPI_ISL_684740:3.48162e-05)30:2.427e-
07,EPI_ISL_684207:6.98195e-05)30:2.427e-07,EPI_ISL_480092:2.425e-07)30:2.427e-
07,EPI_ISL_480181:2.425e-07)34:2.427e-07,EPI_ISL_690820:6.98195e-05)100:6.96002e-
05,(EPI_ISL_542424:2.425e-07,EPI_ISL_480035:0.0001047232)30:2.427e-07)30:2.427e-
07,EPI_ISL_636485:0.0001036799)30:2.427e-07,EPI_ISL_525556:0.0001033185)30:2.427e-
07,((EPI_ISL_479790:0.0001033135,EPI_ISL_542283:2.425e-07)30:2.427e-07,(EPI_ISL_479618:2.425e-
07,EPI_ISL_479619:2.425e-07)100:0.0001390685)100:3.44266e-05)30:2.427e-
07,(EPI_ISL_437301:0.0001378282,EPI_ISL_437203:0.0001033711)75:3.43836e-05)30:2.427e-
07,((EPI_ISL_542194:3.47438e-05,(EPI_ISL_542212:0.000104253,EPI_ISL_542218:2.425e-07)30:2.427e-
07)51:2.427e-07,EPI_ISL_542207:3.47835e-05)100:3.47481e-05)30:2.427e-
07,EPI_ISL_542121:3.47476e-05)30:2.427e-07,EPI_ISL_480310:2.425e-07)30:2.427e-
07,EPI_ISL_542318:2.425e-07)30:2.427e-07,EPI_ISL_542345:3.47443e-05)30:2.427e-
07,EPI_ISL_542236:6.95899e-05)30:2.427e-07,EPI_ISL_454527:3.47944e-05)32:2.427e-
07,EPI_ISL_542241:2.425e-07)30:2.427e-07,(EPI_ISL_542421:0,EPI_ISL_542432:0):2.425e-07)30:2.427e-
07,EPI_ISL_471530:3.48697e-05)30:2.427e-07,EPI_ISL_542112:3.47443e-05)30:2.427e-
07,EPI_ISL_525650:0.0001390721)46:3.44262e-05)30:2.427e-07,((EPI_ISL_626597:2.425e-
07,(EPI_ISL_737021:2.425e-07,EPI_ISL_737024:2.425e-
07)100:0.0001744577)100:0.000349322,((((((EPI_ISL_653765:0.0002756856,((((EPI_ISL_722879:2.425e-
07,EPI_ISL_722905:3.48147e-05)30:2.427e-07,EPI_ISL_722904:2.425e-07)30:2.427e-

07,EPI_ISL_722886:2.425e-07)30:2.427e-07,EPI_ISL_722888:2.425e-07)100:0.0001033831)47:2.427e-07,(((EPI_ISL_653786:6.88846e-05,(EPI_ISL_653804:6.88876e-05,((EPI_ISL_653806:2.425e-07,EPI_ISL_722889:0.0001044408)45:2.427e-07,(EPI_ISL_722875:3.44428e-05,EPI_ISL_722876:2.425e-07)100:6.88925e-05)45:3.44472e-05)30:2.427e-07)100:6.8897e-05,(EPI_ISL_722884:0.0001380847,EPI_ISL_722907:3.45553e-05)31:3.45003e-05)30:2.427e-07,EPI_ISL_722901:6.91085e-05)100:3.44399e-05)48:2.427e-07,EPI_ISL_653768:0.0002412977)100:3.44287e-05,((((((EPI_ISL_653769:3.4432e-05,EPI_ISL_653773:2.425e-07)30:2.427e-07,EPI_ISL_653774:2.425e-07)100:3.44286e-05,(EPI_ISL_653819:2.425e-07,(EPI_ISL_722883:3.44569e-05,EPI_ISL_722890:2.425e-07)100:0.0001378922)100:6.88973e-05)31:2.427e-07,EPI_ISL_653779:0.0003103582)39:2.427e-07,EPI_ISL_653822:0.0003448735)100:0.0001033138,(EPI_ISL_722878:2.425e-07,(EPI_ISL_722882:6.89102e-05,EPI_ISL_722887:3.44469e-05)100:3.44473e-05)100:0.0001723506)36:2.427e-07)30:2.427e-07,(EPI_ISL_653808:0.0001378175,(EPI_ISL_653788:0.0003491963,(((EPI_ISL_653796:2.425e-07,EPI_ISL_653798:2.425e-07)31:2.427e-07,EPI_ISL_653797:2.425e-07)100:0.0001378952,EPI_ISL_722885:0.0001728393)62:2.427e-07)68:2.427e-07)100:3.44458e-05)48:2.427e-07,((EPI_ISL_653775:0.000241279,EPI_ISL_722891:0.0001393599)100:3.44436e-05,((EPI_ISL_653801:2.425e-07,EPI_ISL_653812:3.44408e-05)100:3.44413e-05,(EPI_ISL_653799:0.0004882096,(EPI_ISL_722880:2.425e-07,EPI_ISL_722881:2.425e-07)100:3.44707e-05)98:2.427e-07)100:0.0001723295)100:0.0001033206)100:3.43345e-05,EPI_ISL_653781:0.0002068373)100:0.0001378866)100:6.89262e-05)30:2.427e-07,EPI_ISL_584079:3.48728e-05)30:2.427e-07,((((((((((((((((((((((((((((EPI_ISL_479527:3.47599e-05,(EPI_ISL_479495:2.425e-07,EPI_ISL_479657:3.47861e-05)100:3.47621e-05)100:2.427e-07,EPI_ISL_479546:6.95377e-05)100:0.0001043133,((((EPI_ISL_486383:2.425e-07,EPI_ISL_486881:6.95281e-05)100:3.47629e-05,(EPI_ISL_486396:7.11105e-05,EPI_ISL_436138:2.425e-07)100:6.92472e-05)61:1.0213e-06,((EPI_ISL_436139:3.54119e-05,EPI_ISL_436140:1.0213e-06)100:2.0954e-06,EPI_ISL_436141:2.097e-06)73:6.90866e-05)100:0.0001038694,EPI_ISL_689415:0.0002093876)30:2.427e-07)30:2.427e-07,(EPI_ISL_467042:3.46289e-05,EPI_ISL_467046:2.425e-07)100:3.46332e-05)30:2.427e-07,EPI_ISL_699705:6.92957e-05)30:2.427e-07,EPI_ISL_469035:0.0001041975)30:2.427e-07,EPI_ISL_469031:2.425e-07)30:2.427e-07,EPI_ISL_467047:2.425e-07)30:2.427e-07,EPI_ISL_467043:2.425e-07)30:2.427e-07,EPI_ISL_454560:0.0001044249)30:2.427e-07,EPI_ISL_700072:0.0001741992)30:2.427e-07,EPI_ISL_479539:3.4762e-05)30:2.427e-07,EPI_ISL_454530:0.0003480619)30:2.427e-07,EPI_ISL_454529:2.425e-07)30:2.427e-07,(EPI_ISL_461493:3.46335e-05,(EPI_ISL_467044:2.425e-07,EPI_ISL_467048:2.425e-07)30:2.427e-07)99:3.46329e-05)93:3.44758e-05,(EPI_ISL_479928:0,EPI_ISL_479930:0):2.425e-07)30:2.427e-07,EPI_ISL_636477:3.48503e-05)30:2.427e-07,EPI_ISL_452151:0.0001042102)30:2.427e-07,EPI_ISL_700371:3.48758e-05)30:2.427e-07,EPI_ISL_452152:3.4751e-05)30:2.427e-07,EPI_ISL_636476:0.0001034599)30:2.427e-07,EPI_ISL_691021:0.0001393831)30:2.427e-07,EPI_ISL_479929:2.425e-07)30:2.427e-07,EPI_ISL_690660:6.98226e-05)30:2.427e-07,EPI_ISL_691917:3.49041e-05)30:2.427e-07,(EPI_ISL_452148:2.425e-07,EPI_ISL_690826:0.0001396764)99:3.47505e-05)30:2.427e-07,EPI_ISL_523972:6.95167e-05)30:2.427e-07,EPI_ISL_451935:2.425e-07)30:2.427e-07,EPI_ISL_684737:0.0001047532)90:3.44722e-05)30:2.427e-07,((((EPI_ISL_542355:3.47486e-

05,(((((((((EPI_ISL_542351:0,EPI_ISL_542315:0):0,EPI_ISL_542343:0):0,EPI_ISL_542338:0):0,EPI_ISL_5423
05:0):2.425e-07,(((((EPI_ISL_542353:0.0001042853,EPI_ISL_542337:3.47528e-05)35:2.427e-
07,EPI_ISL_542328:3.4749e-05)30:2.427e-07,EPI_ISL_542357:3.4749e-05)30:2.427e-
07,EPI_ISL_542290:2.425e-07)30:2.427e-07,EPI_ISL_542284:3.47521e-05)30:2.427e-07)30:2.427e-
07,EPI_ISL_542370:3.47521e-05)30:2.427e-07,EPI_ISL_542130:0.0002086724)30:2.427e-
07,EPI_ISL_542378:0.0001042885)30:2.427e-07,EPI_ISL_542240:3.47425e-05)30:2.427e-07)30:2.427e-
07,EPI_ISL_542294:3.47486e-05)30:2.427e-07,EPI_ISL_542155:0.0001042655)30:2.427e-
07,EPI_ISL_542304:3.47486e-05)64:3.47122e-05)30:2.427e-07,(EPI_ISL_525571:2.425e-
07,EPI_ISL_525572:2.425e-07)100:0.0001033211)30:2.427e-07,((EPI_ISL_539780:3.48738e-
05,EPI_ISL_496744:3.58634e-05)31:2.427e-07,EPI_ISL_458236:7.17825e-05)100:3.48166e-
05)30:2.427e-07,(EPI_ISL_444483:2.425e-07,EPI_ISL_542323:6.94346e-05)98:3.45059e-05)30:2.427e-
07,((EPI_ISL_516940:2.425e-
07,(EPI_ISL_516942:2.425e-07,((EPI_ISL_516943:2.425e-07,(((((((EPI_ISL_516944:3.51563e-
05,(((((((EPI_ISL_516960:2.425e-07,((EPI_ISL_516971:7.16412e-05,EPI_ISL_516972:2.425e-07)30:2.427e-
07,((EPI_ISL_486394:2.425e-07,EPI_ISL_486398:0.0001393774)100:3.4815e-
05,EPI_ISL_486399:4.12868e-05)100:0.0001243176)30:2.427e-07)30:2.427e-
07,EPI_ISL_516961:3.58519e-05)31:2.427e-07,EPI_ISL_436137:0.000141677)31:2.427e-
07,EPI_ISL_516962:2.425e-07)74:4.14446e-05,EPI_ISL_486388:6.90892e-
05)76:0.0001014168,(((((((((((EPI_ISL_516970:7.17143e-05,(EPI_ISL_454543:3.48191e-
05,EPI_ISL_452209:2.425e-07)30:2.427e-07)30:2.427e-07,EPI_ISL_454561:0.0002808533)63:2.427e-
07,EPI_ISL_454547:2.425e-07)100:3.47648e-05,(EPI_ISL_454542:2.425e-07,EPI_ISL_452206:3.48666e-
05)100:3.47647e-05)38:2.427e-07,((EPI_ISL_486382:2.425e-
07,EPI_ISL_486397:0.0001402864)100:2.427e-07,(EPI_ISL_436156:2.425e-07,EPI_ISL_436157:4.68365e-
05)100:9.37671e-05)93:0.0001044134)60:2.427e-07,EPI_ISL_454552:0.0001398931)88:3.47658e-
05,((EPI_ISL_454540:3.4768e-05,EPI_ISL_454551:3.47929e-05)31:2.427e-
07,EPI_ISL_454544:0.0001745517)100:3.47643e-05)30:2.427e-07,EPI_ISL_690989:3.48877e-
05)30:2.427e-07,((EPI_ISL_480226:2.425e-07,EPI_ISL_685298:2.425e-07)100:3.48835e-
05,EPI_ISL_686022:2.425e-07)100:6.97799e-05)30:2.427e-07,((EPI_ISL_486385:2.425e-
07,EPI_ISL_486386:0.0002721512)92:3.51181e-05,EPI_ISL_486392:3.48332e-05)91:3.48107e-
05)30:2.427e-07,EPI_ISL_454546:6.96991e-05)30:2.427e-07)30:2.446e-07,(EPI_ISL_479861:2.425e-
07,EPI_ISL_684133:3.52226e-05)100:3.4883e-05)30:2.446e-07)30:2.446e-
07,(EPI_ISL_486384:0.0003000323,((EPI_ISL_486389:2.425e-
07,EPI_ISL_486409:0.0001042168)30:2.427e-07,EPI_ISL_486408:3.47835e-
05)100:0.0001042294)35:3.46341e-05)30:2.446e-07,EPI_ISL_486395:7.01599e-05)30:2.446e-
07,((EPI_ISL_479520:3.47882e-05,(EPI_ISL_437453:2.425e-
07,EPI_ISL_437454:0.0003491199)84:6.97085e-05)80:3.47728e-05,(EPI_ISL_480118:2.425e-
07,EPI_ISL_480036:2.425e-07)100:0.0001045719)48:3.47402e-05)30:2.446e-07,EPI_ISL_428856:2.425e-
07)30:2.446e-07,EPI_ISL_690852:2.425e-07)30:2.446e-07,(EPI_ISL_486387:7.15212e-
05,((EPI_ISL_486401:0.0001057838,EPI_ISL_486403:3.53543e-05)49:7.02365e-
05,EPI_ISL_486402:3.51726e-05)30:2.427e-07)49:6.96664e-05)30:2.446e-07)30:2.446e-
07,EPI_ISL_452208:2.425e-07)30:2.446e-07)30:2.446e-07)30:2.446e-07,EPI_ISL_438138:2.425e-
07)30:2.427e-07,EPI_ISL_486393:2.425e-07)30:2.427e-07,EPI_ISL_516941:2.425e-07)44:3.44707e-
05,EPI_ISL_454555:0.0004692105)31:2.427e-07,EPI_ISL_454548:0.0003263332)31:2.427e-
07,EPI_ISL_516945:7.58799e-05)78:0.0001034602,EPI_ISL_479825:3.48817e-05)30:2.427e-

07,((((EPI_ISL_479862:0.0001393561,EPI_ISL_479863:2.425e-07)100:3.48259e-
05,EPI_ISL_416567:3.48284e-05)32:2.427e-07,EPI_ISL_416615:0.0001053284)100:2.427e-
07,EPI_ISL_416599:2.425e-07)98:3.46663e-05)30:2.427e-07,EPI_ISL_416566:6.96603e-05)30:2.427e-
07,((EPI_ISL_454549:6.95407e-05,EPI_ISL_684930:7.05163e-05)100:6.95601e-
05,((EPI_ISL_419213:7.16574e-05,EPI_ISL_419221:3.49879e-05)51:7.1244e-
05,EPI_ISL_419243:0.0003218485)51:0.0001395777)30:3.47576e-05)30:2.427e-
07,EPI_ISL_416630:3.49116e-05)30:2.427e-07,EPI_ISL_416578:3.48295e-05)30:2.427e-
07,EPI_ISL_416589:0.0001044944)30:2.427e-07,((((EPI_ISL_486400:7.03316e-
05,EPI_ISL_486404:7.02617e-05)100:0.0001392477,(EPI_ISL_524749:2.425e-
07,EPI_ISL_524750:3.4872e-
05)100:0.0001395715)100:0.000139233,EPI_ISL_454528:0.0002088764)100:6.95921e-
05,((EPI_ISL_454525:6.98577e-05,EPI_ISL_452213:2.425e-07)30:2.427e-07,EPI_ISL_454526:2.425e-
07)100:0.000139115)100:0.0001043056)30:2.427e-07,(EPI_ISL_479850:2.425e-
07,EPI_ISL_416581:3.48282e-05)100:3.45527e-05)30:2.427e-07,EPI_ISL_690745:6.96787e-
05)30:2.427e-
07,(((EPI_ISL_416606:0,EPI_ISL_416595:0):0,EPI_ISL_416592:0):0,EPI_ISL_416586:0):2.425e-
07)30:2.427e-07,(EPI_ISL_416571:2.425e-07,EPI_ISL_416593:2.425e-07)100:3.48244e-05)30:2.427e-
07,(EPI_ISL_416605:3.49118e-05,EPI_ISL_480031:2.425e-07)100:6.98371e-05)30:2.427e-
07,((((((((EPI_ISL_413459:2.425e-07,((((((EPI_ISL_416602:2.425e-07,(((EPI_ISL_416603:2.425e-
07,EPI_ISL_416625:3.4886e-05)31:2.427e-07,EPI_ISL_416613:0.0001047648)30:2.427e-
07,EPI_ISL_480030:2.425e-07)30:2.427e-07)30:2.427e-07,EPI_ISL_416624:2.425e-07)30:2.427e-
07,EPI_ISL_416619:2.425e-07)30:2.427e-07,EPI_ISL_416565:2.425e-07)30:2.427e-
07,EPI_ISL_480032:2.425e-07)30:2.427e-07,EPI_ISL_416616:2.425e-07)100:3.4465e-
05,EPI_ISL_416568:7.05125e-05)30:2.427e-07)30:2.427e-07,EPI_ISL_416604:6.98397e-05)30:2.427e-
07,EPI_ISL_685585:2.425e-07)30:2.427e-07,EPI_ISL_416598:2.425e-07)30:2.427e-
07,EPI_ISL_416588:2.425e-07)30:2.427e-07,EPI_ISL_416587:3.48846e-05)30:2.427e-
07,EPI_ISL_416610:2.425e-07)30:2.427e-07,EPI_ISL_416582:3.48243e-05)100:3.4468e-05)30:2.427e-
07,(EPI_ISL_416607:2.425e-07,EPI_ISL_416608:2.425e-07)100:3.4825e-05)30:2.427e-
07,EPI_ISL_416574:2.425e-07)30:2.427e-07,EPI_ISL_416609:2.425e-07)30:2.427e-
07,EPI_ISL_454749:2.425e-07)30:2.427e-
07,((((EPI_ISL_479851:0,EPI_ISL_416596:0):0,EPI_ISL_684163:0):0,EPI_ISL_684139:0):0,EPI_ISL_416594:
0):2.425e-07)30:2.427e-07,EPI_ISL_416622:3.44832e-05)30:2.427e-
07,((((((EPI_ISL_416629:0,EPI_ISL_480033:0):0,EPI_ISL_416580:0):0,EPI_ISL_416575:0):0,EPI_ISL_416572
:0):0,EPI_ISL_416576:0):0,EPI_ISL_416573:0):2.425e-07)30:2.427e-07,EPI_ISL_416614:3.48269e-
05)30:2.427e-07,EPI_ISL_416591:3.44681e-05)30:2.427e-07,EPI_ISL_416577:2.425e-07)30:2.427e-
07,(EPI_ISL_416570:0.0001047984,EPI_ISL_416585:3.48292e-05)100:3.48294e-05)30:2.427e-
07,EPI_ISL_416634:2.425e-07)30:2.427e-07,EPI_ISL_416601:0.0001045169)30:2.427e-
07,EPI_ISL_416618:2.425e-07)30:2.427e-07,EPI_ISL_416623:2.425e-07)30:2.427e-
07,EPI_ISL_685780:6.97733e-05)30:2.427e-07,EPI_ISL_416633:3.48267e-05)30:2.427e-
07,EPI_ISL_416612:3.47581e-05)30:2.427e-07,EPI_ISL_416631:2.425e-07)30:2.427e-
07,EPI_ISL_416583:3.48232e-05)30:2.427e-07,(EPI_ISL_416632:0,EPI_ISL_416597:0):2.425e-
07)30:2.427e-
07,(((EPI_ISL_416600:0,EPI_ISL_416627:0):0,EPI_ISL_416617:0):0,EPI_ISL_416590:0):2.425e-
07)30:2.427e-07,EPI_ISL_416628:2.425e-07)30:2.427e-07,EPI_ISL_416569:2.425e-07)30:2.427e-

07,EPI_ISL_416620:3.48282e-05)30:2.427e-07,EPI_ISL_479852:3.48601e-05)30:2.427e-07,EPI_ISL_416579:6.96546e-05)30:2.427e-07,EPI_ISL_416621:3.48282e-05)30:2.427e-07,EPI_ISL_416626:3.50869e-05)30:2.427e-07,EPI_ISL_416611:2.425e-07)30:3.44691e-05,((((((((((((((EPI_ISL_480103:3.48289e-05,(((((((((((((((((((((((((((((((((((((EPI_ISL_480104:2.425e-07,EPI_ISL_479931:3.4916e-05)100:3.49139e-05,((((EPI_ISL_480227:2.425e-07,EPI_ISL_684128:3.5201e-05)99:3.48843e-05,((EPI_ISL_479855:2.425e-07,EPI_ISL_685203:6.97758e-05)30:2.427e-07,(EPI_ISL_479858:2.425e-07,EPI_ISL_479860:6.98358e-05)100:3.49115e-05)30:2.427e-07)30:2.427e-07,EPI_ISL_479905:2.425e-07)30:2.427e-07,EPI_ISL_684168:2.425e-07)30:2.427e-07)30:2.427e-07,EPI_ISL_690850:6.98297e-05)30:2.427e-07,(EPI_ISL_479927:2.425e-07,EPI_ISL_480065:3.49144e-05)100:6.95715e-05)30:2.427e-07,EPI_ISL_479833:2.425e-07)30:2.427e-07,EPI_ISL_688758:0.0001045473)30:2.427e-07,(EPI_ISL_684414:2.425e-07,EPI_ISL_684422:2.425e-07)100:3.48796e-05)30:2.427e-07,EPI_ISL_479807:2.425e-07)30:2.427e-07,EPI_ISL_479952:3.49106e-05)30:2.427e-07,EPI_ISL_479823:3.48269e-05)30:2.427e-07,EPI_ISL_479890:3.49141e-05)30:2.427e-07,((EPI_ISL_479809:2.425e-07,EPI_ISL_479810:2.425e-07)100:2.427e-07,EPI_ISL_479811:6.97221e-05)100:3.48575e-05)30:2.427e-07,EPI_ISL_479856:2.425e-07)30:2.427e-07,EPI_ISL_685695:3.49112e-05)30:2.427e-07,EPI_ISL_479958:6.96588e-05)30:2.427e-07,EPI_ISL_686038:6.98162e-05)30:2.427e-07,EPI_ISL_688757:3.48794e-05)30:2.427e-07,EPI_ISL_479868:2.425e-07)30:2.427e-07,EPI_ISL_479945:2.425e-07)30:2.427e-07,(EPI_ISL_479946:2.425e-07,EPI_ISL_480034:2.425e-07)100:3.48226e-05)30:2.427e-07,((((EPI_ISL_479819:0,EPI_ISL_479947:0):0,EPI_ISL_684156:0):0,EPI_ISL_684142:0):0,EPI_ISL_685647:0):2.425e-07)30:2.427e-07,EPI_ISL_479925:3.49111e-05)30:2.427e-07,(EPI_ISL_479820:0,EPI_ISL_479835:0):2.425e-07)30:2.427e-07,(((((((((((((((((EPI_ISL_479822:0,EPI_ISL_479956:0):0,EPI_ISL_479910:0):0,EPI_ISL_684152:0):0,EPI_ISL_479954:0):0,EPI_ISL_479903:0):0,EPI_ISL_479906:0):0,EPI_ISL_479909:0):0,EPI_ISL_479953:0):0,EPI_ISL_479857:0):0,EPI_ISL_479908:0):0,EPI_ISL_479912:0):0,EPI_ISL_690848:0):0,EPI_ISL_690845:0):0,EPI_ISL_479955:0):0,EPI_ISL_479926:0):0,EPI_ISL_479907:0):0,EPI_ISL_479859:0):2.425e-07)30:2.427e-07,EPI_ISL_690847:2.425e-07)30:2.427e-07,(EPI_ISL_479805:2.425e-07,EPI_ISL_479806:2.425e-07)100:3.48287e-05)30:2.427e-07,EPI_ISL_479815:2.425e-07)30:2.427e-07,(EPI_ISL_479944:0,EPI_ISL_479951:0):2.425e-07)30:2.427e-07,EPI_ISL_480002:2.425e-07)30:2.427e-07,EPI_ISL_685696:3.49094e-05)30:2.427e-07,EPI_ISL_684153:3.48794e-05)30:2.427e-07,EPI_ISL_479986:6.98377e-05)30:2.427e-07,EPI_ISL_480105:2.425e-07)30:2.427e-07,(EPI_ISL_479949:2.425e-07,EPI_ISL_684151:3.48792e-05)100:6.9658e-05)30:2.427e-07,EPI_ISL_479821:2.425e-07)30:2.427e-07,EPI_ISL_479948:3.49108e-05)30:2.427e-07,EPI_ISL_479888:2.425e-07)30:2.427e-07,EPI_ISL_479904:3.48289e-05)30:2.427e-07)30:2.427e-07,EPI_ISL_479957:6.98287e-05)30:2.427e-07,EPI_ISL_479950:3.48419e-05)30:2.427e-07,EPI_ISL_684132:7.12034e-05)100:6.95887e-05,(((((EPI_ISL_486405:2.425e-07,(EPI_ISL_486406:8.52459e-05,EPI_ISL_486407:8.04278e-05)100:6.44317e-05)100:6.15617e-05,(((EPI_ISL_454531:1.0213e-06,EPI_ISL_452204:1.0213e-06)89:6.33577e-05,(EPI_ISL_454536:2.425e-07,EPI_ISL_454537:0.000209114)99:5.58241e-05)90:0.0003647816,(EPI_ISL_454534:0.0002121559,EPI_ISL_452205:1.0213e-06)97:2.0756e-06)100:6.86139e-05)30:2.427e-07,EPI_ISL_452203:3.47566e-05)34:2.427e-07,EPI_ISL_513155:0.000140667)30:2.427e-07,EPI_ISL_586541:0.0002793284)79:3.43955e-05)89:3.44451e-05,(EPI_ISL_512989:3.43688e-05,EPI_ISL_512990:0.0001379214)32:6.89418e-05)32:3.44792e-05,(((EPI_ISL_479939:2.425e-07,EPI_ISL_479940:2.425e-07)100:3.4887e-

05,EPI_ISL_690824:6.9867e-05)100:0.0002096476,((((((EPI_ISL_412970:3.46772e-05,(EPI_ISL_434541:3.49724e-05,(EPI_ISL_434547:0.0001399287,EPI_ISL_426364:6.8885e-05)30:2.427e-07)100:3.44349e-05)30:2.427e-07,EPI_ISL_452202:6.95341e-05)30:2.427e-07,EPI_ISL_445082:3.45699e-05)30:2.427e-07,EPI_ISL_684693:0.0001397106)30:2.427e-07,EPI_ISL_434543:2.425e-07)30:2.427e-07,EPI_ISL_455432:0.0001442142)100:0.0001033365)30:2.427e-07)30:2.427e-07,(((((((((((((((((((((EPI_ISL_480109:2.425e-07,((((((((((((((((((EPI_ISL_480112:0,EPI_ISL_480053:0):0,EPI_ISL_480042:0):0,EPI_ISL_480063:0):0,EPI_ISL_480047:0):0,EPI_ISL_480051:0):0,EPI_ISL_480054:0):0,EPI_ISL_480046:0):0,EPI_ISL_480055:0):0,EPI_ISL_479984:0):0,EPI_ISL_480057:0):0,EPI_ISL_480048:0):0,EPI_ISL_480062:0):0,EPI_ISL_480060:0):0,EPI_ISL_480064:0):0,EPI_ISL_480058:0):0,EPI_ISL_479983:0):0,EPI_ISL_480043:0):2.425e-07,EPI_ISL_684158:3.48775e-05)31:2.427e-07,EPI_ISL_480116:2.425e-07)30:2.427e-07)30:2.427e-07,EPI_ISL_480056:2.425e-07)30:2.427e-07,(((((EPI_ISL_480083:0,EPI_ISL_480087:0):0,EPI_ISL_480089:0):0,EPI_ISL_480088:0):2.425e-07,(EPI_ISL_480084:2.425e-07,EPI_ISL_480086:2.425e-07)30:2.427e-07)30:2.427e-07,EPI_ISL_480085:3.49138e-05)100:6.96569e-05)30:2.427e-07,EPI_ISL_480113:2.425e-07)30:2.427e-07,EPI_ISL_480044:3.48775e-05)30:2.427e-07,EPI_ISL_480114:2.425e-07)30:2.427e-07,EPI_ISL_690987:2.425e-07)30:2.427e-07,EPI_ISL_480061:3.48261e-05)30:2.427e-07,EPI_ISL_480117:2.425e-07)30:2.427e-07,(((EPI_ISL_480050:0,EPI_ISL_690988:0):0,EPI_ISL_684171:0):0,EPI_ISL_684172:0):2.425e-07)30:2.427e-07,EPI_ISL_480052:2.425e-07)30:2.427e-07,EPI_ISL_480115:2.425e-07)30:2.427e-07,EPI_ISL_480049:3.48228e-05)30:2.427e-07,EPI_ISL_684170:6.97722e-05)30:2.427e-07,EPI_ISL_480045:3.48772e-05)30:2.427e-07,EPI_ISL_480111:3.48248e-05)30:2.427e-07,EPI_ISL_684164:2.425e-07)30:2.427e-07,EPI_ISL_480110:0.0001047125)30:2.427e-07,(EPI_ISL_479979:2.425e-07,EPI_ISL_479980:2.425e-07)100:3.45702e-05)30:2.427e-07,EPI_ISL_479981:2.425e-07)30:2.427e-07,EPI_ISL_480059:2.425e-07)30:2.427e-07,EPI_ISL_479982:3.48586e-05)100:6.91378e-05)30:2.427e-07,(((((EPI_ISL_417034:6.97529e-05,(((EPI_ISL_542346:3.4856e-05,(((EPI_ISL_489708:2.425e-07,((EPI_ISL_684736:2.425e-07,EPI_ISL_685238:2.425e-07)30:2.427e-07,EPI_ISL_685245:2.425e-07)100:3.48776e-05)100:3.48776e-05,(EPI_ISL_496607:2.425e-07,EPI_ISL_498162:3.48846e-05)30:2.427e-07)30:2.427e-07,EPI_ISL_496632:2.425e-07)30:2.427e-07)30:2.427e-07,EPI_ISL_496605:3.58999e-05)30:2.427e-07,EPI_ISL_496670:2.425e-07)30:2.427e-07)30:2.427e-07,EPI_ISL_686021:6.98314e-05)30:2.427e-07,EPI_ISL_496679:0.0001481414)30:2.427e-07,EPI_ISL_452515:3.54774e-05)30:2.427e-07,EPI_ISL_688759:6.98248e-05)100:0.0001731867)30:2.427e-07,(((EPI_ISL_479869:3.48817e-05,((((((((EPI_ISL_479892:2.425e-07,(((EPI_ISL_479924:6.97306e-05,EPI_ISL_479959:6.97797e-05)30:2.427e-07,(EPI_ISL_684143:0,EPI_ISL_684512:0):2.425e-07)30:2.427e-07,EPI_ISL_479960:2.425e-07)30:2.427e-07)30:2.427e-07,EPI_ISL_684154:2.425e-07)99:2.427e-07,EPI_ISL_479932:3.45504e-05)100:3.45542e-05,((EPI_ISL_479916:2.425e-07,EPI_ISL_684861:2.425e-07)30:2.427e-07,(((((EPI_ISL_479918:2.425e-07,(EPI_ISL_479920:2.425e-07,EPI_ISL_479922:3.49132e-05)100:3.48267e-05)31:2.427e-07,EPI_ISL_479921:2.425e-07)30:2.427e-07,EPI_ISL_479919:2.425e-07)31:2.427e-07,EPI_ISL_479923:2.425e-07)30:2.427e-07,EPI_ISL_685297:2.425e-07)100:3.48222e-05)30:2.427e-07)30:2.427e-07,EPI_ISL_479914:3.48244e-05)30:2.427e-07,EPI_ISL_684145:2.425e-07)30:2.427e-07,EPI_ISL_684858:3.49118e-05)30:2.427e-07,EPI_ISL_479915:2.425e-07)30:2.427e-07,EPI_ISL_684146:2.425e-07)30:2.427e-07)30:2.427e-07,EPI_ISL_479913:2.425e-07)30:2.427e-

07,EPI_ISL_479917:2.425e-07)100:6.9112e-05)30:2.427e-07,EPI_ISL_685606:6.9831e-05)30:2.427e-07,(((EPI_ISL_501167:0.0001728022,EPI_ISL_631808:6.97765e-05)100:3.077e-07,EPI_ISL_445084:2.425e-07)100:0.0001873031,(EPI_ISL_525621:2.425e-07,EPI_ISL_496610:2.425e-07)100:6.9548e-05)100:0.0002623798)30:2.427e-07,EPI_ISL_407976:2.425e-07)32:3.44444e-05,EPI_ISL_684129:0.0001050567)32:3.44374e-05)30:2.427e-07,(((((((EPI_ISL_479870:2.425e-07,EPI_ISL_479871:3.49073e-05)100:6.98399e-05,(((((((((((EPI_ISL_479872:2.425e-07,EPI_ISL_479847:3.48261e-05)30:2.427e-07,EPI_ISL_479842:2.425e-07)92:2.427e-07,EPI_ISL_479837:2.425e-07)99:3.4825e-05,(((((((((EPI_ISL_479873:2.425e-07,EPI_ISL_684126:2.425e-07)30:2.427e-07,EPI_ISL_479874:2.425e-07)100:6.95995e-05,EPI_ISL_479841:2.425e-07)36:3.47919e-05,((((((((EPI_ISL_479875:6.98319e-05,EPI_ISL_479876:2.425e-07)100:3.48259e-05,EPI_ISL_479849:2.425e-07)97:3.48304e-05,((((((((((EPI_ISL_479879:3.49083e-05,((((EPI_ISL_479838:2.425e-07,EPI_ISL_684114:2.425e-07)30:2.427e-07,EPI_ISL_691016:2.425e-07)30:2.427e-07,(EPI_ISL_684111:3.4825e-05,EPI_ISL_684120:2.425e-07)100:3.48253e-05)30:2.427e-07,EPI_ISL_479795:2.425e-07)30:2.427e-07)30:2.427e-07,EPI_ISL_479828:2.425e-07)30:2.427e-07,EPI_ISL_479800:2.425e-07)30:2.427e-07,EPI_ISL_684113:2.425e-07)98:3.48253e-05,((((((EPI_ISL_479817:2.425e-07,EPI_ISL_684116:2.425e-07)66:2.427e-07,EPI_ISL_479839:2.425e-07)30:2.427e-07,EPI_ISL_684121:2.425e-07)30:2.427e-07,EPI_ISL_479818:2.425e-07)100:3.4826e-05,EPI_ISL_684123:3.48765e-05)31:2.427e-07,EPI_ISL_479827:3.48215e-05)30:2.427e-07)30:2.427e-07,(EPI_ISL_479812:0,EPI_ISL_479831:0):2.425e-07)30:2.427e-07,EPI_ISL_479881:3.48586e-05)30:2.427e-07,EPI_ISL_479830:2.425e-07)30:2.427e-07,EPI_ISL_684125:3.48795e-05)30:2.427e-07,EPI_ISL_684115:3.48795e-05)30:2.427e-07)30:2.427e-07,(((EPI_ISL_479801:0,EPI_ISL_684110:0):0,EPI_ISL_684119:0):0,EPI_ISL_684118:0):2.425e-07)30:2.427e-07,(EPI_ISL_685313:6.97593e-05,(((((EPI_ISL_685261:2.425e-07,(EPI_ISL_685267:3.52035e-05,(EPI_ISL_685271:6.96674e-05,EPI_ISL_685255:2.425e-07)30:2.427e-07)30:2.427e-07)30:2.427e-07,EPI_ISL_685259:3.48261e-05)30:2.427e-07,EPI_ISL_685258:3.48194e-05)30:2.427e-07,EPI_ISL_685253:2.425e-07)30:2.427e-07,EPI_ISL_685269:3.48421e-05)40:2.427e-07)100:6.9646e-05)30:2.427e-07,EPI_ISL_479877:3.49081e-05)30:2.427e-07,((((((((((EPI_ISL_479816:0,EPI_ISL_479794:0):0,EPI_ISL_479848:0):0,EPI_ISL_479998:0):0,EPI_ISL_479997:0):0,EPI_ISL_479799:0):0,EPI_ISL_479843:0):0,EPI_ISL_479846:0):0,EPI_ISL_479832:0):0,EPI_ISL_479793:0):0,EPI_ISL_479845:0):0,EPI_ISL_479834:0):2.425e-07)30:2.427e-07,EPI_ISL_479840:2.425e-07)30:2.427e-07)30:2.427e-07,EPI_ISL_684124:2.425e-07)30:2.427e-07,EPI_ISL_479829:2.425e-07)30:2.427e-07,EPI_ISL_479878:3.48393e-05)30:2.427e-07,EPI_ISL_479836:3.49081e-05)30:2.427e-07,(EPI_ISL_479814:2.425e-07,EPI_ISL_479792:2.425e-07)100:3.48817e-05)30:2.427e-07)30:2.427e-07,EPI_ISL_479813:2.425e-07)30:2.427e-07,EPI_ISL_684122:2.425e-07)30:2.427e-07,EPI_ISL_479808:2.425e-07)30:2.427e-07,EPI_ISL_479824:2.425e-07)30:2.427e-07,EPI_ISL_479826:3.48759e-05)30:2.427e-07,EPI_ISL_479844:3.49081e-05)100:3.45517e-05,(((((EPI_ISL_480221:6.9756e-05,EPI_ISL_684622:2.425e-07)100:6.97487e-05,(((((((((((EPI_ISL_479941:3.49112e-05,((((EPI_ISL_479964:2.425e-07,(((((((EPI_ISL_689643:2.425e-07,EPI_ISL_684734:6.98391e-05)30:2.427e-07,EPI_ISL_684514:3.49099e-05)30:2.427e-07,EPI_ISL_684735:2.425e-07)30:2.427e-07,EPI_ISL_684728:7.0337e-05)30:2.427e-07,EPI_ISL_684732:3.49112e-05)30:2.427e-07,EPI_ISL_684629:2.425e-07)30:2.427e-07,EPI_ISL_684625:3.48776e-05)30:2.427e-07)30:2.427e-07,EPI_ISL_479965:2.425e-07)30:2.427e-07,EPI_ISL_684731:3.49112e-05)30:2.427e-07,(((EPI_ISL_684617:0,EPI_ISL_684633:0):0,EPI_ISL_684513:0):0,EPI_ISL_684515:0):2.425e-

07)30:2.427e-07)100:3.48255e-05,(EPI_ISL_684155:2.425e-07,((EPI_ISL_684620:6.97224e-05,(EPI_ISL_684628:3.49099e-05,(EPI_ISL_684632:2.425e-07,EPI_ISL_684525:3.49099e-05)31:2.427e-07)30:2.427e-07)30:2.427e-07,EPI_ISL_684627:6.98372e-05)99:6.97249e-05)30:2.427e-07)30:2.427e-07,(EPI_ISL_684920:2.425e-07,EPI_ISL_684940:2.425e-07)100:3.48795e-05)30:2.427e-07,EPI_ISL_684624:2.425e-07)30:2.427e-07,EPI_ISL_684623:2.425e-07)30:2.427e-07,EPI_ISL_684626:3.4882e-05)30:2.427e-07,EPI_ISL_684619:2.425e-07)30:2.427e-07,EPI_ISL_684616:2.425e-07)30:2.427e-07,EPI_ISL_684631:3.48821e-05)30:2.427e-07,EPI_ISL_684516:0.0001046546)30:2.427e-07,(EPI_ISL_684729:6.98386e-05,EPI_ISL_684730:2.425e-07)100:3.48802e-05)30:2.427e-07)30:2.427e-07,EPI_ISL_685667:2.425e-07)30:2.427e-07,EPI_ISL_684615:2.425e-07)30:2.427e-07,EPI_ISL_684630:2.425e-07)100:0.0001044821)30:2.427e-07)31:2.427e-07,(EPI_ISL_479797:3.49073e-05,EPI_ISL_479798:2.425e-07)100:0.0001045045)30:2.427e-07,EPI_ISL_479804:2.425e-07)30:2.427e-07,EPI_ISL_479803:2.425e-07)31:2.427e-07,EPI_ISL_479802:3.47865e-05)30:2.427e-07,EPI_ISL_479889:3.49106e-05)98:3.45512e-05)30:2.427e-07,EPI_ISL_718137:0.0001377676)30:2.427e-07,((EPI_ISL_416032:2.425e-07,((((EPI_ISL_525683:0.0001390963,EPI_ISL_631624:0.0002456724)100:3.47648e-05,EPI_ISL_685781:6.9764e-05)36:2.427e-07,EPI_ISL_684374:0.0001395851)90:2.427e-07,EPI_ISL_437303:6.88797e-05)54:3.44364e-05)94:6.88795e-05,EPI_ISL_684136:3.5122e-05)30:3.56492e-05)30:2.427e-07,(EPI_ISL_479886:3.48258e-05,EPI_ISL_479887:2.425e-07)100:3.48286e-05)30:2.427e-07,EPI_ISL_541138:0.0001046605)30:2.427e-07,EPI_ISL_437189:0.0001033262)30:2.427e-07,(EPI_ISL_455435:2.425e-07,EPI_ISL_455454:7.08378e-05)100:3.45505e-05)30:2.427e-07,EPI_ISL_542215:3.47548e-05)30:4.854e-07,(EPI_ISL_479866:3.48373e-05,(EPI_ISL_479867:2.425e-07,((EPI_ISL_479864:2.425e-07,EPI_ISL_479865:3.48173e-05)30:2.427e-07,EPI_ISL_684130:2.425e-07)30:2.427e-07)30:2.427e-07)100:0.0001044939)30:4.854e-07,EPI_ISL_430846:7.62426e-05)30:2.427e-07,((EPI_ISL_419662:6.98745e-05,EPI_ISL_684429:0.0001046382)31:2.427e-07,EPI_ISL_471550:6.97635e-05)100:6.971e-05)30:6.91292e-05,EPI_ISL_631773:3.4879e-05)30:2.427e-07,EPI_ISL_718155:0.0001733041)30:3.44353e-05,EPI_ISL_437198:2.425e-07)30:2.427e-07,EPI_ISL_513020:3.44354e-05)30:2.427e-07,(EPI_ISL_437197:0,EPI_ISL_437201:0):2.425e-07)30:3.44353e-05)30:2.427e-07,((EPI_ISL_480196:6.98035e-05,((((((((((((((((((((EPI_ISL_462449:3.52607e-05,EPI_ISL_452521:2.425e-07)30:2.427e-07,EPI_ISL_525469:7.02234e-05)30:2.427e-07,EPI_ISL_452492:2.425e-07)30:2.427e-07,EPI_ISL_454533:3.47905e-05)30:2.427e-07,((((((((((((((((((((EPI_ISL_626571:3.49042e-05,((((((((EPI_ISL_626573:2.425e-07,EPI_ISL_660553:3.49039e-05)100:3.49043e-05,EPI_ISL_626601:6.98219e-05)40:2.427e-07,((EPI_ISL_626578:0,EPI_ISL_626612:0):0,EPI_ISL_626600:0):2.425e-07)30:2.427e-07,((((((((EPI_ISL_626577:2.425e-07,((EPI_ISL_626581:2.425e-07,EPI_ISL_626618:3.49042e-05)100:3.49047e-05,(((EPI_ISL_660561:0.0001047344,EPI_ISL_660564:2.425e-07)30:2.427e-07,EPI_ISL_626623:2.425e-07)100:0.000104767,EPI_ISL_736998:6.98311e-05)39:2.427e-07)30:2.427e-07)30:2.427e-07,((EPI_ISL_660565:2.425e-07,EPI_ISL_660568:2.425e-07)100:3.49086e-05,EPI_ISL_693661:3.49085e-05)100:3.49054e-05)30:2.427e-07,EPI_ISL_737015:0.0001396966)31:2.427e-07,EPI_ISL_693672:0.0001747146)30:2.427e-07,EPI_ISL_737023:6.98156e-05)30:2.427e-07,EPI_ISL_693685:0.0001397015)38:2.427e-07,EPI_ISL_660573:6.98155e-05)100:3.49097e-05,(EPI_ISL_626619:2.425e-07,(EPI_ISL_626602:0.0001396701,EPI_ISL_693683:0.0001047514)30:2.427e-07)100:3.4905e-

05)100:3.49096e-05)30:2.427e-07,(EPI_ISL_737028:3.48907e-05,EPI_ISL_737012:6.98358e-05)100:6.98363e-05)30:2.427e-07,((EPI_ISL_626594:2.425e-07,EPI_ISL_693660:0.0001397108)30:2.427e-07,EPI_ISL_737010:2.425e-07)100:3.49086e-05)30:2.427e-07,EPI_ISL_626609:0.0001047318)30:2.427e-07,((EPI_ISL_626596:2.425e-07,(EPI_ISL_626613:2.425e-07,EPI_ISL_737002:2.425e-07)100:3.49041e-05)100:3.48991e-05,(EPI_ISL_737031:2.425e-07,EPI_ISL_737032:2.425e-07)100:6.98349e-05)100:6.98348e-05)30:2.427e-07)30:2.427e-07,(((EPI_ISL_660563:2.425e-07,EPI_ISL_660570:2.425e-07)100:3.49039e-05,EPI_ISL_660572:2.425e-07)30:2.427e-07,EPI_ISL_660571:2.425e-07)100:0.000174648)30:2.427e-07,EPI_ISL_660562:0.0002096054)30:2.427e-07,(EPI_ISL_660569:3.49034e-05,EPI_ISL_737003:2.425e-07)100:6.98232e-05)30:2.427e-07,EPI_ISL_737009:0.0001396984)30:2.427e-07,EPI_ISL_693679:0.0001746077)30:2.427e-07,((EPI_ISL_737011:3.49033e-05,EPI_ISL_737018:3.49094e-05)100:2.427e-07,EPI_ISL_693674:0.0001047397)100:6.98292e-05)30:2.427e-07,(EPI_ISL_626592:2.425e-07,EPI_ISL_660559:6.98297e-05)100:3.49081e-05)30:2.427e-07,EPI_ISL_626625:6.9823e-05)30:2.427e-07,EPI_ISL_626599:2.425e-07)30:2.427e-07,EPI_ISL_693677:6.98227e-05)30:2.427e-07,EPI_ISL_660566:0.0001396789)30:2.427e-07,EPI_ISL_737025:3.49029e-05)36:2.427e-07,EPI_ISL_737019:0.000139653)91:3.49033e-05,(EPI_ISL_660560:2.425e-07,EPI_ISL_660567:3.49062e-05)100:0.0001396895)67:2.427e-07,(EPI_ISL_626616:3.48973e-05,EPI_ISL_737008:3.49201e-05)100:0.0001047672)32:2.427e-07,EPI_ISL_737022:0.0003145763)37:2.427e-07,EPI_ISL_660554:0.0001746358)31:2.427e-07,EPI_ISL_737016:0.0001396884)100:0.0001747509,(((EPI_ISL_653780:3.44752e-05,EPI_ISL_653811:3.48335e-05)100:2.427e-07,EPI_ISL_653810:6.88979e-05)100:6.89352e-05,(EPI_ISL_722873:3.45114e-05,EPI_ISL_722902:6.92971e-05)100:0.0001381203)100:3.4307e-05)99:0.000172457)30:2.427e-07,EPI_ISL_452480:2.425e-07)99:3.44468e-05,(((EPI_ISL_455437:6.9844e-05,EPI_ISL_493338:7.12363e-05)100:2.427e-07,EPI_ISL_493336:3.48622e-05)100:3.48623e-05,(EPI_ISL_516611:0.0001045773,((EPI_ISL_516623:0.0001058714,(EPI_ISL_493346:3.50172e-05,(((((((((((EPI_ISL_688808:4.04715e-05,EPI_ISL_690895:2.425e-07)37:2.427e-07,EPI_ISL_690919:2.425e-07)30:2.427e-07,EPI_ISL_688810:2.425e-07)30:2.427e-07,EPI_ISL_690975:3.49166e-05)30:2.427e-07,EPI_ISL_690912:2.425e-07)30:2.427e-07,EPI_ISL_690961:3.48487e-05)30:2.427e-07,EPI_ISL_690913:2.425e-07)31:2.427e-07,EPI_ISL_690959:2.425e-07)30:2.427e-07,EPI_ISL_690970:6.98024e-05)30:2.427e-07,EPI_ISL_690962:6.96764e-05)30:2.427e-07,((((EPI_ISL_690921:0,EPI_ISL_690965:0):0,EPI_ISL_690831:0):0,EPI_ISL_690977:0):0,EPI_ISL_690974:0):2.425e-07)100:0.0002025325,EPI_ISL_735360:0.0002440091)50:2.427e-07)30:2.427e-07)44:2.427e-07,EPI_ISL_576278:0.0004494976)30:2.427e-07)81:3.45265e-05)30:2.427e-07)30:2.427e-07,EPI_ISL_447801:2.425e-07)30:2.427e-07,EPI_ISL_690876:3.48846e-05)30:2.427e-07,EPI_ISL_498165:3.48997e-05)30:2.427e-07,(EPI_ISL_685749:2.425e-07,((EPI_ISL_685750:2.425e-07,EPI_ISL_685760:2.425e-07)100:2.427e-07,EPI_ISL_685762:2.425e-07)100:3.49161e-05)100:3.49205e-05)30:2.427e-07,EPI_ISL_480093:2.425e-07)30:2.427e-07,EPI_ISL_493348:0.0001046335)30:2.427e-07,EPI_ISL_684219:3.48846e-05)30:2.427e-07,EPI_ISL_690892:2.425e-07)30:2.427e-07,EPI_ISL_480200:2.425e-07)30:2.427e-07,EPI_ISL_419664:2.425e-07)30:2.427e-07,(EPI_ISL_691147:2.425e-07,EPI_ISL_691160:2.425e-07)100:3.48849e-05)30:2.427e-07,EPI_ISL_516625:0.0001743663)30:2.427e-07,(EPI_ISL_690872:2.425e-07,EPI_ISL_690879:3.48295e-05)100:3.48297e-05)30:2.427e-07)30:2.427e-

07,EPI_ISL_542260:2.425e-07)30:2.427e-07,EPI_ISL_690874:3.49163e-05)30:2.427e-
07,EPI_ISL_735354:0.0001393546)30:2.427e-07,(EPI_ISL_447747:2.425e-07,EPI_ISL_498154:2.425e-
07)100:3.48847e-05)30:2.427e-07,EPI_ISL_690979:3.48874e-05)30:2.427e-
07,EPI_ISL_690858:3.48874e-05)30:2.427e-07,EPI_ISL_690937:2.425e-07)30:2.427e-
07,EPI_ISL_542275:0.000104194)30:2.427e-07,EPI_ISL_462448:7.02566e-05)30:2.427e-
07,((EPI_ISL_690920:3.49163e-05,EPI_ISL_690889:2.425e-07)100:2.427e-07,EPI_ISL_690880:2.425e-
07)100:3.48744e-05)30:2.427e-07,EPI_ISL_690881:3.48891e-05)30:2.427e-
07,(((((((((EPI_ISL_479934:0,EPI_ISL_690956:0):0,EPI_ISL_690865:0):0,EPI_ISL_688776:0):0,EPI_ISL_6852
56:0):0,EPI_ISL_688761:0):0,EPI_ISL_690878:0):0,EPI_ISL_690855:0):0,EPI_ISL_685648:0):0,EPI_ISL_690
900:0):2.425e-07)30:2.427e-07,EPI_ISL_542248:3.47599e-05)30:2.427e-07,EPI_ISL_416426:3.48844e-
05)30:2.427e-07,(EPI_ISL_542320:0,EPI_ISL_542222:0):2.425e-07)30:2.427e-
07,EPI_ISL_637109:6.94682e-05)30:2.427e-07,EPI_ISL_479911:3.49191e-05)30:2.427e-
07,EPI_ISL_498156:2.425e-07)30:2.427e-07,EPI_ISL_483566:0.0001043339)30:2.427e-
07,EPI_ISL_527753:0.0002093776)30:2.427e-07,EPI_ISL_480202:2.425e-07)30:2.427e-
07,EPI_ISL_542425:3.47203e-05)30:2.427e-07,EPI_ISL_480199:2.425e-07)30:2.427e-
07,EPI_ISL_690891:3.49162e-05)30:2.427e-07,EPI_ISL_480198:2.425e-07)30:2.427e-
07,EPI_ISL_690871:3.49154e-05)30:2.427e-07,EPI_ISL_688624:6.97908e-05)30:2.427e-
07,EPI_ISL_685608:2.425e-07)30:2.427e-07,EPI_ISL_684196:6.97822e-05)30:2.427e-
07,EPI_ISL_690877:6.98547e-05)30:2.427e-07,EPI_ISL_684149:2.425e-07)30:2.427e-
07,EPI_ISL_686040:2.425e-07)30:2.427e-07,EPI_ISL_684694:6.98372e-05)30:2.427e-
07,EPI_ISL_542341:2.425e-07)30:2.427e-07,EPI_ISL_690860:2.425e-07)30:2.427e-
07,EPI_ISL_493340:3.56936e-05)30:2.427e-
07,((((((((EPI_ISL_480197:0,EPI_ISL_684296:0):0,EPI_ISL_690861:0):0,EPI_ISL_480203:0):0,EPI_ISL_6908
73:0):0,EPI_ISL_690870:0):0,EPI_ISL_690862:0):0,EPI_ISL_691113:0):0,EPI_ISL_691114:0):2.425e-
07)30:2.427e-07,(EPI_ISL_690863:3.48332e-05,EPI_ISL_690868:2.425e-07)100:6.96632e-05)30:2.427e-
07,EPI_ISL_690875:2.425e-07)30:2.427e-07,EPI_ISL_542192:3.47617e-05)30:2.427e-
07,EPI_ISL_542376:0.0001391)30:2.427e-07,EPI_ISL_485603:6.92278e-05)30:2.427e-
07,((((((EPI_ISL_685666:0,EPI_ISL_690936:0):0,EPI_ISL_691139:0):0,EPI_ISL_690884:0):0,EPI_ISL_690866
:0):0,EPI_ISL_690945:0):0,EPI_ISL_690952:0):2.425e-07)30:2.427e-07,EPI_ISL_690867:2.425e-
07)33:3.4445e-05,EPI_ISL_542413:6.95337e-05)30:2.427e-07)30:3.44487e-
05,EPI_ISL_447776:8.27123e-05)30:2.427e-
07,(EPI_ISL_513031:0.0001083106,EPI_ISL_525468:9.29986e-05)59:4.58869e-05)30:2.427e-
07,EPI_ISL_475701:0.0002757996)30:2.427e-07,(((((((EPI_ISL_479936:2.425e-
07,EPI_ISL_684372:2.425e-07)100:2.427e-07,EPI_ISL_684371:0.0001048042)100:3.48423e-
05,(EPI_ISL_471555:3.48645e-05,((EPI_ISL_584074:3.9129e-06,EPI_ISL_584076:2.425e-
07)100:3.09755e-05,EPI_ISL_584077:2.425e-07)100:0.0002444635)99:3.49225e-05)31:2.427e-
07,EPI_ISL_525627:3.47663e-05)30:2.427e-07,(EPI_ISL_568577:0.0005948951,(EPI_ISL_690853:2.425e-
07,EPI_ISL_690856:2.425e-07)100:0.000104937)99:3.46794e-05)30:2.427e-07,EPI_ISL_691018:2.425e-
07)30:2.427e-07,EPI_ISL_447294:2.425e-07)98:3.4766e-05)30:2.427e-
07,((EPI_ISL_435051:0.0001046723,((((((((((((((((((((((((EPI
_ISL_523963:0.00020749,((((((((((((((((((EPI_ISL_475625:0.0002413448,((((((((((EPI_ISL_475616:6.89282e
-05,((((((((((((((((((EPI_ISL_475710:2.425e-07,(((((((((((((EPI_ISL_434549:3.49929e-
05,EPI_ISL_631706:7.01768e-05)100:3.49005e-05,(((EPI_ISL_631772:2.425e-
07,EPI_ISL_631712:3.49652e-05)30:2.427e-07,EPI_ISL_631735:2.425e-07)100:3.49024e-

05,EPI_ISL_631568:2.425e-07)30:2.427e-07)30:2.427e-07,EPI_ISL_631740:3.50608e-05)30:2.427e-
07,EPI_ISL_631813:6.98113e-05)30:2.427e-07,EPI_ISL_631711:6.9937e-05)30:2.427e-
07,EPI_ISL_631806:2.425e-07)30:2.427e-07,EPI_ISL_631812:3.51517e-05)30:2.427e-
07,EPI_ISL_631556:2.425e-07)30:2.427e-07,(EPI_ISL_631835:2.425e-07,EPI_ISL_631597:2.425e-
07)100:6.98174e-05)30:2.427e-07,EPI_ISL_631613:2.425e-07)30:2.427e-
07,EPI_ISL_631777:0.0001047171)30:2.427e-07,EPI_ISL_631801:3.51548e-05)30:2.427e-
07,EPI_ISL_631698:7.03103e-05)30:2.427e-07)30:2.427e-07,EPI_ISL_631758:3.49415e-05)30:2.427e-
07,EPI_ISL_631577:2.425e-07)30:2.427e-07,EPI_ISL_631546:2.425e-07)30:2.427e-
07,EPI_ISL_525686:6.95753e-05)30:2.427e-07,EPI_ISL_631759:2.425e-07)30:2.427e-
07,EPI_ISL_631616:7.01715e-05)30:2.427e-07,EPI_ISL_631761:3.48983e-05)30:2.427e-
07,EPI_ISL_631782:2.425e-07)100:3.44567e-05,EPI_ISL_434548:0.0001067215)100:3.44566e-
05,EPI_ISL_493334:2.425e-07)30:2.427e-07,EPI_ISL_631617:7.01615e-05)30:2.427e-
07,EPI_ISL_686025:2.425e-07)30:2.427e-07,EPI_ISL_525658:2.425e-07)30:2.427e-
07,EPI_ISL_631720:3.51516e-05)100:3.44578e-05,EPI_ISL_631704:7.01648e-05)30:2.427e-
07,EPI_ISL_626593:2.425e-07)30:2.427e-07,EPI_ISL_631594:7.03095e-05)30:2.427e-07)30:2.427e-
07,EPI_ISL_631769:3.48981e-05)30:2.427e-07,(EPI_ISL_501172:0.0002434194,EPI_ISL_691084:2.425e-
07)54:3.46684e-05)30:2.427e-07,((((((EPI_ISL_525646:3.47764e-05,EPI_ISL_691027:3.49303e-
05)30:2.427e-07,EPI_ISL_631829:3.48979e-05)100:3.47762e-05,EPI_ISL_631815:3.49057e-
05)30:2.427e-07,(EPI_ISL_583479:2.425e-07,EPI_ISL_583480:0.0001396485)100:3.49035e-
05)31:2.427e-07,EPI_ISL_631554:3.5151e-05)30:2.427e-07,EPI_ISL_631811:0.0001054831)100:3.4776e-
05)30:2.427e-07,((EPI_ISL_539494:2.425e-07,EPI_ISL_539777:2.425e-07)100:9.30414e-
05,EPI_ISL_626570:2.425e-07)32:4.65702e-05)30:2.427e-07,((((EPI_ISL_593553:2.425e-
07,EPI_ISL_593480:5.47169e-
05)100:0.0001093244,EPI_ISL_593556:0.0002095673)100:0.0006044641,EPI_ISL_593479:2.425e-
07)30:2.427e-07,EPI_ISL_593478:2.425e-07)100:0.0001394326)30:2.427e-07,EPI_ISL_526686:6.98263e-
05)30:2.427e-07,EPI_ISL_631689:3.51507e-05)30:2.427e-
07,((EPI_ISL_479991:0,EPI_ISL_479994:0):2.425e-07,(EPI_ISL_479992:2.425e-
07,EPI_ISL_479993:2.425e-07)30:2.427e-07)99:6.971e-05)30:2.427e-07,((((EPI_ISL_480222:2.425e-
07,(((((((((((((EPI_ISL_684403:0,EPI_ISL_684360:0):0,EPI_ISL_684406:0):2.425e-
07,EPI_ISL_684358:2.425e-07)30:2.427e-07,EPI_ISL_685563:2.425e-07)30:2.427e-
07,(EPI_ISL_684405:0,EPI_ISL_684524:0):2.425e-07)37:2.427e-07,(EPI_ISL_685635:2.425e-
07,EPI_ISL_685639:6.98657e-05)99:3.49294e-05)44:2.427e-07,EPI_ISL_684407:2.425e-07)31:2.427e-
07,EPI_ISL_684404:2.425e-07)100:3.48977e-05,EPI_ISL_684375:2.425e-07)31:2.427e-
07,EPI_ISL_685535:2.425e-07)30:2.427e-07,EPI_ISL_685525:2.425e-07)30:2.427e-
07,EPI_ISL_686856:2.425e-07)30:2.427e-07,EPI_ISL_685530:2.425e-07)30:2.427e-
07,EPI_ISL_685529:2.425e-07)30:2.427e-07)30:2.427e-07,EPI_ISL_684378:2.425e-07)30:2.427e-
07,(((EPI_ISL_684376:0,EPI_ISL_684389:0):0,EPI_ISL_684384:0):0,EPI_ISL_684395:0):2.425e-
07)30:2.427e-07,EPI_ISL_684377:2.425e-07)99:3.48462e-05)30:2.427e-07)30:2.427e-
07,(EPI_ISL_684522:2.425e-07,EPI_ISL_684556:2.425e-07)100:6.98018e-05)30:2.427e-
07,((EPI_ISL_631770:2.425e-07,EPI_ISL_690819:6.97073e-05)30:2.427e-07,EPI_ISL_583472:6.98232e-
05)100:6.97002e-05)30:2.427e-07,EPI_ISL_525681:3.47785e-05)30:2.427e-
07,EPI_ISL_631840:3.50388e-05)30:2.427e-07,EPI_ISL_631776:0.000174999)30:2.427e-
07,EPI_ISL_475619:6.89194e-05)30:2.427e-07,EPI_ISL_479796:2.425e-07)30:2.427e-
07,(EPI_ISL_690854:2.425e-07,(EPI_ISL_690857:2.425e-07,EPI_ISL_690864:2.425e-07)100:3.49284e-

05)100:3.49283e-05)30:2.427e-07,(EPI_ISL_631803:0,EPI_ISL_631542:0):2.425e-07)30:2.427e-07,((EPI_ISL_475614:3.44578e-05,(EPI_ISL_495426:0.000175178,((EPI_ISL_684889:2.425e-07,EPI_ISL_685132:2.425e-07)100:0.0001396727,EPI_ISL_691019:3.48998e-05)44:2.427e-07)40:2.427e-07)40:2.427e-07,EPI_ISL_539493:3.49183e-05)32:3.44578e-05)30:2.427e-07,EPI_ISL_525677:3.47746e-05)30:2.427e-07,EPI_ISL_631584:2.425e-07)30:2.427e-07,EPI_ISL_631686:3.51499e-05)30:2.427e-07,((EPI_ISL_525678:3.47796e-05,EPI_ISL_631511:2.425e-07)30:2.427e-07,EPI_ISL_631510:2.425e-07)100:3.47748e-05)30:2.427e-07,(EPI_ISL_434542:0.0001066535,EPI_ISL_434550:3.55198e-05)99:3.54897e-05)30:2.427e-07,EPI_ISL_690859:3.49327e-05)30:2.427e-07,EPI_ISL_434551:3.49799e-05)30:2.427e-07)30:2.427e-07,(EPI_ISL_682242:7.00222e-05,(EPI_ISL_682250:2.425e-07,EPI_ISL_682252:2.425e-07)100:3.49084e-05)100:0.0001398014)30:2.427e-07,EPI_ISL_685241:2.425e-07)30:2.427e-07,EPI_ISL_491456:3.47937e-05)30:2.427e-07,EPI_ISL_631637:2.425e-07)30:2.427e-07,(EPI_ISL_445079:0.0001401774,EPI_ISL_444022:5.15722e-05)99:8.436e-05)30:2.427e-07,EPI_ISL_631558:2.425e-07)30:2.427e-07,EPI_ISL_631736:0.0001047099)30:2.427e-07,((EPI_ISL_525684:6.95709e-05,(EPI_ISL_525679:2.425e-07,EPI_ISL_631781:6.98996e-05)30:2.427e-07)30:2.427e-07,EPI_ISL_631513:2.425e-07)100:3.47742e-05)30:2.427e-07,EPI_ISL_631615:3.49012e-05)30:2.427e-07,EPI_ISL_475715:0.0001379029)30:2.427e-07,((((EPI_ISL_491094:0,EPI_ISL_631867:0):0,EPI_ISL_631716:0):0,EPI_ISL_631701:0):0,EPI_ISL_631831:0):2.425e-07)30:2.427e-07,((EPI_ISL_479883:0,EPI_ISL_479885:0):2.425e-07,(EPI_ISL_479884:2.425e-07,(EPI_ISL_685260:2.425e-07,EPI_ISL_685247:2.425e-07)100:3.48997e-05)30:2.427e-07)100:6.97996e-05)30:2.427e-07,(((EPI_ISL_501168:6.89123e-05,EPI_ISL_501174:0.0002070225)97:6.89461e-05,(EPI_ISL_463287:0.0001047639,EPI_ISL_684893:3.49312e-05)100:3.49036e-05)30:2.427e-07,EPI_ISL_501173:0.0001385249)54:3.44793e-05)30:2.427e-07,EPI_ISL_631690:7.03049e-05)30:2.427e-07,EPI_ISL_631733:2.425e-07)30:2.427e-07,EPI_ISL_631778:3.49005e-05)30:2.427e-07,EPI_ISL_631606:7.03136e-05)30:2.427e-07,EPI_ISL_479938:0.0001747452)30:2.427e-07,EPI_ISL_685784:7.05231e-05)30:2.427e-07,EPI_ISL_631618:3.51517e-05)30:2.427e-07,EPI_ISL_525641:3.47736e-05)30:2.427e-07,EPI_ISL_631697:0.0001054795)30:2.427e-07,EPI_ISL_631561:0.0001055037)30:2.427e-07)30:2.427e-07,EPI_ISL_525640:0.0001043671)30:2.427e-07,EPI_ISL_610237:6.96977e-05)30:2.427e-07,EPI_ISL_447774:2.425e-07)30:2.427e-07,EPI_ISL_631574:7.03172e-05)30:2.427e-07,EPI_ISL_684857:6.98668e-05)30:2.427e-07,(EPI_ISL_631687:0.0001055275,EPI_ISL_685620:7.00317e-05)99:3.49181e-05)30:2.427e-07,(EPI_ISL_526688:2.425e-07,EPI_ISL_548244:2.425e-07)100:0.0001047273)30:2.427e-07,EPI_ISL_631726:3.51529e-05)30:2.427e-07,EPI_ISL_445081:2.425e-07)30:2.427e-07,EPI_ISL_493335:6.92953e-05)30:2.427e-07,EPI_ISL_631570:2.425e-07)30:2.427e-07,(EPI_ISL_631593:7.03073e-05,EPI_ISL_684187:0.0001746026)100:3.49154e-05)30:2.427e-07,EPI_ISL_631755:3.48954e-05)30:2.427e-07,EPI_ISL_691022:2.425e-07)30:2.427e-07,EPI_ISL_496651:2.425e-07)30:2.427e-07,EPI_ISL_434544:0.0002911893)30:2.427e-07,EPI_ISL_475591:0.0004830658)30:2.427e-07,(EPI_ISL_631760:2.425e-07,EPI_ISL_631538:2.425e-07)100:3.50749e-05)30:2.427e-07,EPI_ISL_475676:3.45136e-05)30:2.427e-07,EPI_ISL_631705:0.0001055319)30:2.427e-07,EPI_ISL_631719:3.50745e-05)30:2.427e-07,EPI_ISL_489709:2.425e-07)30:2.427e-07,EPI_ISL_631633:7.03088e-05)30:2.427e-07,EPI_ISL_434553:0.0001065687)30:2.427e-07,EPI_ISL_525648:6.95586e-05)30:2.427e-07,EPI_ISL_631625:3.51475e-05)30:2.427e-07,EPI_ISL_434545:3.49816e-05)30:2.427e-07,EPI_ISL_475615:0.0001723588)30:2.427e-07,EPI_ISL_631628:3.48994e-05)30:2.427e-

07,EPI_ISL_525638:3.4773e-05)30:2.427e-07,EPI_ISL_631757:3.48979e-05)30:2.427e-
07,EPI_ISL_631841:0.0001409442)30:2.427e-
07,(((((EPI_ISL_525682:0,EPI_ISL_529035:0):0,EPI_ISL_525642:0):0,EPI_ISL_525649:0):0,EPI_ISL_525578:
0):0,EPI_ISL_525676:0):2.425e-07)30:2.427e-07,EPI_ISL_530348:2.425e-07)30:2.427e-
07,EPI_ISL_631619:0.0001051941)30:2.427e-07,EPI_ISL_525631:2.425e-07)30:2.427e-
07,EPI_ISL_447791:2.425e-07)30:2.427e-07,EPI_ISL_631598:7.03017e-05)30:2.427e-
07,((EPI_ISL_475667:0.0001033856,EPI_ISL_475652:3.4455e-05)82:2.427e-07,EPI_ISL_475713:3.4455e-
05)84:3.44508e-05)30:2.427e-07,((EPI_ISL_631504:0,EPI_ISL_631573:0):0,EPI_ISL_631595:0):2.425e-
07)30:2.427e-07,EPI_ISL_631703:3.51517e-05)30:2.427e-07,EPI_ISL_475621:2.425e-07)30:2.427e-
07,EPI_ISL_525654:3.47729e-05)30:2.427e-07,EPI_ISL_498155:2.425e-07)30:2.427e-
07,EPI_ISL_631724:3.48991e-05)30:2.427e-07,EPI_ISL_507008:0.0001033821)30:2.427e-
07,EPI_ISL_631836:3.4956e-05)30:2.427e-07,EPI_ISL_631728:3.48987e-05)30:2.427e-
07,EPI_ISL_475685:2.425e-07)30:2.427e-07,EPI_ISL_525647:6.9551e-05)30:2.427e-
07,EPI_ISL_447306:6.9805e-05)30:2.427e-07,EPI_ISL_631762:6.98022e-05)30:2.427e-
07,EPI_ISL_631768:0.000174561)30:2.427e-07,EPI_ISL_631774:0.0001047358)30:2.427e-
07,EPI_ISL_631845:3.51168e-05)30:2.427e-07,EPI_ISL_434552:0.0001452305)30:2.427e-
07,EPI_ISL_631797:2.425e-07)30:2.427e-07,EPI_ISL_447296:2.425e-07)30:2.427e-
07,EPI_ISL_631710:3.50548e-05)30:2.427e-07,EPI_ISL_525659:0.0001391579)30:2.427e-
07,EPI_ISL_452149:6.95341e-05)30:2.427e-07,(EPI_ISL_480106:2.425e-07,EPI_ISL_480107:2.425e-
07)100:0.0001047181)30:2.427e-07,EPI_ISL_631591:0.0001054599)30:2.427e-
07,EPI_ISL_631629:3.5147e-05)71:3.44548e-05)30:3.44495e-05,EPI_ISL_529036:3.48487e-
05)30:2.427e-07,(EPI_ISL_419296:0,EPI_ISL_631754:0):2.425e-07)30:2.427e-
07,EPI_ISL_684894:3.49206e-05)30:2.427e-07,EPI_ISL_516969:3.48895e-05)30:2.427e-
07,EPI_ISL_582030:0.0001402533)30:2.427e-07,EPI_ISL_419298:2.425e-07)30:2.427e-
07,EPI_ISL_475657:2.425e-07)30:2.427e-07,EPI_ISL_631694:7.02958e-05)30:2.427e-
07,EPI_ISL_631866:2.425e-07)30:2.427e-07,EPI_ISL_631602:2.425e-07)30:2.427e-
07,EPI_ISL_525680:6.95463e-05)30:2.427e-07,EPI_ISL_450508:6.97421e-05)30:2.427e-
07,EPI_ISL_690849:2.425e-07)30:2.427e-07,EPI_ISL_498160:2.425e-07)30:2.427e-
07,(EPI_ISL_631607:2.425e-07,EPI_ISL_684929:3.48882e-05)100:3.4889e-05)30:2.427e-
07,EPI_ISL_456125:3.52735e-05)30:2.427e-07,EPI_ISL_526934:6.98063e-05)30:2.427e-
07,EPI_ISL_498166:3.4895e-05)30:2.427e-07,EPI_ISL_475637:2.425e-07)30:2.427e-
07,EPI_ISL_631603:2.425e-07)30:2.427e-07,EPI_ISL_684918:3.49206e-05)30:2.427e-
07,EPI_ISL_583465:3.48979e-05)30:2.427e-07,EPI_ISL_498157:2.425e-07)30:2.427e-
07,EPI_ISL_419297:2.425e-07)30:2.427e-07,EPI_ISL_690846:2.425e-07)30:2.427e-
07,EPI_ISL_498161:3.49129e-05)30:2.427e-
07,(EPI_ISL_513180:0.0001384523,EPI_ISL_631695:3.50876e-05)45:3.45752e-05)30:2.427e-
07,EPI_ISL_631596:0.000140652)30:2.427e-07,EPI_ISL_631587:0.0001054649)30:2.427e-
07,(((EPI_ISL_452142:2.425e-07,((EPI_ISL_735271:2.425e-07,EPI_ISL_735293:2.425e-07)100:3.48336e-
05,EPI_ISL_735289:0.000174676)99:2.427e-07)100:3.47703e-05,(EPI_ISL_480237:7.00136e-
05,EPI_ISL_735352:0.0002092002)100:3.48619e-05)31:2.427e-07,(EPI_ISL_495439:2.425e-
07,EPI_ISL_495457:2.425e-07)100:0.0001045526)100:6.95406e-05)30:2.427e-
07,((EPI_ISL_419300:2.425e-07,EPI_ISL_419299:2.425e-07)100:3.49245e-05,EPI_ISL_631610:3.51475e-
05)30:2.427e-07)30:3.44502e-05,EPI_ISL_495046:6.93928e-05)30:2.427e-07,EPI_ISL_479854:2.425e-
07)30:2.427e-07,EPI_ISL_636967:3.44507e-05)30:2.427e-07,EPI_ISL_479901:2.425e-07)30:2.427e-

07,EPI_ISL_513096:0.0001378587)30:2.427e-07,EPI_ISL_513169:3.44485e-05)30:2.427e-07,EPI_ISL_513158:0.00013784)30:2.427e-07,EPI_ISL_513203:2.425e-07)30:2.427e-07,EPI_ISL_513038:3.44461e-05)30:2.427e-07,EPI_ISL_513132:3.44461e-05)30:2.427e-07,EPI_ISL_512967:3.44851e-05)30:2.427e-07,EPI_ISL_479853:3.49216e-05)30:2.427e-07,EPI_ISL_513152:0.0001725332)30:2.427e-07,(EPI_ISL_479896:2.425e-07,(((EPI_ISL_479897:2.425e-07,EPI_ISL_479898:6.97221e-05)30:2.427e-07,EPI_ISL_479900:2.425e-07)30:2.427e-07,EPI_ISL_479899:3.49206e-05)30:2.427e-07)100:3.48377e-05)30:2.427e-07,EPI_ISL_513153:0.0001033578)30:2.427e-07,EPI_ISL_512913:6.89047e-05)30:2.427e-07,(((((((EPI_ISL_513032:0,EPI_ISL_513124:0):0,EPI_ISL_513122:0):0,EPI_ISL_512941:0):0,EPI_ISL_513145:0):0,EPI_ISL_512963:0):0,EPI_ISL_512992:0):0,EPI_ISL_512974:0):2.425e-07)30:2.427e-07,EPI_ISL_512962:2.425e-07)30:2.427e-07,EPI_ISL_529963:0.0002423289)30:2.427e-07,EPI_ISL_513150:3.44462e-05)30:2.427e-07,(EPI_ISL_513024:6.89191e-05,EPI_ISL_512887:3.44569e-05)99:3.44478e-05)30:2.427e-07,EPI_ISL_513138:3.44462e-05)30:2.427e-07,EPI_ISL_513141:6.88993e-05)30:2.427e-07,EPI_ISL_513009:3.44511e-05)30:2.427e-07,EPI_ISL_512881:6.89175e-05)30:2.427e-07,(EPI_ISL_483824:0.0001043509,(EPI_ISL_576113:2.425e-07,EPI_ISL_576115:2.425e-07)100:0.0004487123)99:3.44379e-05)30:2.427e-07,EPI_ISL_513006:6.89153e-05)30:2.427e-07,(EPI_ISL_512077:0.0002087916,EPI_ISL_524732:0.0001046561)100:6.95957e-05)30:2.427e-07,(EPI_ISL_524762:6.97743e-05,EPI_ISL_524763:2.425e-07)100:6.97771e-05)30:2.427e-07,((((((((((EPI_ISL_513028:3.44543e-05,(EPI_ISL_513033:2.425e-07,(EPI_ISL_513034:2.425e-07,EPI_ISL_512878:0.0001033898)84:3.44533e-05)76:3.44572e-05)64:3.44579e-05,EPI_ISL_513098:3.44587e-05)55:3.44584e-05,(((EPI_ISL_513083:6.89375e-05,EPI_ISL_513099:3.44587e-05)52:3.44508e-05,(EPI_ISL_513094:6.89223e-05,EPI_ISL_513095:2.425e-07)100:6.89156e-05)48:3.44586e-05,(((((EPI_ISL_447035:2.425e-07,EPI_ISL_467045:3.47237e-05)100:2.427e-07,EPI_ISL_467041:2.425e-07)100:3.46517e-05,((((((((EPI_ISL_495047:0.0002248433,(EPI_ISL_495063:2.425e-07,(EPI_ISL_475047:2.425e-07,EPI_ISL_475048:3.48576e-05)100:3.48528e-05)100:5.19215e-05)21:3.40715e-05,EPI_ISL_469038:4.87397e-05)100:0.0001243622,EPI_ISL_512069:0.0002088016)30:2.427e-07,EPI_ISL_524723:2.425e-07)74:3.46974e-05,((EPI_ISL_512067:6.94106e-05,EPI_ISL_512068:6.94106e-05)100:3.47081e-05,EPI_ISL_524747:2.425e-07)100:3.46959e-05)54:2.427e-07,(EPI_ISL_495056:3.48361e-05,EPI_ISL_495057:2.425e-07)100:6.96879e-05)30:2.427e-07,(EPI_ISL_512072:2.425e-07,EPI_ISL_512073:3.47712e-05)100:6.95582e-05)30:2.427e-07,EPI_ISL_512912:3.47261e-05)30:2.427e-07,EPI_ISL_495064:0.000139451)30:2.427e-07)30:2.427e-07,EPI_ISL_524748:0.0001046647)30:2.427e-07,((((EPI_ISL_514611:0.0001043652,EPI_ISL_514612:3.47476e-05)100:6.95793e-05,((EPI_ISL_512074:0.0002786861,(EPI_ISL_586565:2.425e-07,EPI_ISL_586566:3.48846e-05)100:0.000209735)90:3.45675e-05,EPI_ISL_461500:3.47552e-05)30:2.427e-07)30:2.427e-07,(EPI_ISL_461501:6.95027e-05,EPI_ISL_461502:2.425e-07)100:0.0001390147)30:2.427e-07,(EPI_ISL_514596:0.0001739359,EPI_ISL_514597:2.425e-07)100:6.95661e-05)30:2.427e-07)31:3.44536e-05)30:2.427e-07)30:2.427e-07,EPI_ISL_513149:2.425e-07)30:2.427e-07,(((EPI_ISL_461485:0.0001042495,EPI_ISL_514587:0.0002088302)30:2.427e-07,EPI_ISL_512058:0.0001391385)84:3.47468e-05,EPI_ISL_512066:0.000278588)31:3.47461e-05)30:2.427e-07,EPI_ISL_524724:0.0001746406)30:2.427e-07,(EPI_ISL_513081:0.0001033656,(EPI_ISL_513202:3.44533e-05,EPI_ISL_513168:0.0001033873)44:2.427e-07)47:3.44528e-05)30:2.427e-

07,(EPI_ISL_480289:0.0001045615,EPI_ISL_735288:6.96878e-05)100:3.48433e-05)30:2.427e-07,EPI_ISL_513151:2.425e-07)30:2.427e-07,((EPI_ISL_513213:2.425e-07,EPI_ISL_513191:2.425e-07)100:3.44445e-05,EPI_ISL_513190:0.0001034167)38:3.44669e-05)34:2.427e-07,EPI_ISL_733570:0.0002795938)61:6.89205e-05)30:2.427e-07,(EPI_ISL_447053:0.0001045468,EPI_ISL_444480:0.0001394545)100:3.47757e-05)30:2.427e-07,((((((((((EPI_ISL_653766:3.48729e-05,EPI_ISL_653771:2.425e-07)31:2.427e-07,EPI_ISL_653783:2.425e-07)33:2.427e-07,EPI_ISL_653770:2.425e-07)32:2.427e-07,EPI_ISL_653809:6.96613e-05)97:2.427e-07,EPI_ISL_653772:3.44475e-05)97:2.427e-07,EPI_ISL_653776:0.0001033928)100:6.89167e-05,((EPI_ISL_653767:2.425e-07,EPI_ISL_653807:6.89586e-05)100:3.44552e-05,EPI_ISL_722877:6.89974e-05)100:6.89119e-05)30:2.427e-07,EPI_ISL_653793:6.89941e-05)100:0.0001033753,(EPI_ISL_653777:3.48676e-05,EPI_ISL_653778:6.9134e-05)100:6.91346e-05)30:2.427e-07,(EPI_ISL_737014:0.0001746697,EPI_ISL_693663:0.0001397143)100:3.48441e-05)31:2.427e-07,EPI_ISL_653820:0.0001034117)100:0.0004486608)30:2.427e-07,(EPI_ISL_513018:3.44525e-05,(EPI_ISL_483820:6.95555e-05,((((EPI_ISL_437199:0,EPI_ISL_437202:0):0,EPI_ISL_437299:0):0,EPI_ISL_437200:0):2.425e-07,EPI_ISL_437298:2.425e-07)100:6.89124e-05)78:3.44555e-05)30:2.427e-07)32:3.44523e-05,EPI_ISL_513013:3.47796e-05)30:2.427e-07,EPI_ISL_513010:2.425e-07)30:2.427e-07,EPI_ISL_513040:6.89084e-05)30:2.427e-07,EPI_ISL_513188:3.44476e-05)30:2.427e-07,(EPI_ISL_513062:6.89019e-05,((EPI_ISL_513002:3.44511e-05,(EPI_ISL_513016:3.44525e-05,(EPI_ISL_513089:0.0001033865,(EPI_ISL_513100:6.8912e-05,EPI_ISL_512883:0.0001033963)32:2.427e-07)30:2.427e-07)30:2.427e-07)34:2.427e-07,EPI_ISL_513011:0.00013986)41:3.44522e-05)30:2.427e-07);

Supplementary Table 1: GISAID sequence acknowledgement table

We gratefully acknowledge the following Authors from the Originating laboratories responsible for obtaining the specimens, as well as the Submitting laboratories where the genome data were generated and shared via GISAID, on which this research is based.

All Submitters of data may be contacted directly via www.gisaid.org

Authors are sorted alphabetically.

Accession ID	Originating Laboratory	Submitting Laboratory	Authors
EPI_ISL_402124, EPI_ISL_402127, EPI_ISL_402128, EPI_ISL_402129, EPI_ISL_402130	Wuhan Jinyintan Hospital	Wuhan Institute of Virology, Chinese Academy of Sciences	Peng Zhou, Xing-Lou Yang, Ding-Yu Zhang, Lei Zhang, Yan Zhu, Hao-Rui Si, Zhengli Shi
EPI_ISL_402132	Wuhan Jinyintan Hospital	Hubei Provincial Center for Disease Control and Prevention	Bin Fang, Xiang Li, Xiao Yu, Linlin Liu, Bo Yang, Faxian Zhan, Guojun Ye, Xixiang Huo, Junqiang Xu, Bo Yu, Kun Cai, Jing Li, Yongzhong Jiang.
EPI_ISL_403962, EPI_ISL_403963	Bamrasnaradura Hospital	1. Department of Medical Sciences, Ministry of Public Health, Thailand 2. Thai Red Cross Emerging Infectious Diseases - Health Science Centre 3. Department of Disease Control, Ministry of Public Health, Thailand	Pilailuk,Okada; Siripaporn,Phuygun; Thanutsapa,Thanadachakul; Supaporn,Wacharapluesadee; Sittiporn,Parnmen; Warawan,Wongboot; Sunthareeya,Waicharoen; Rome,Buathong; Malinee,Chittaganpitch; Nanthawan,Mekha
EPI_ISL_404227	Zhejiang Provincial Center for Disease Control and Prevention	Department of Microbiology, Zhejiang Provincial Center for Disease Control and Prevention	Yin Chen, Yanjun Zhang, Haiyan Mao, Junhang Pan, Xiuyu Lou, Yiyu Lu, Juying Yan, Hanping Zhu, Jian Gao, Yan Feng, Yi Sun, Hao Yan, Zhen Li, Yisheng Sun, Liming Gong, Qiong Ge, Wen Shi, Xinying Wang, Wenwu Yao, Zhangnv Yang, Fang Xu, Chen Chen, Enfu Chen, Zhen Wang, Zhiping Chen, Jianmin Jiang, Chonggao Hu
EPI_ISL_404228	Zhejiang Provincial Center for Disease Control and Prevention	Department of Microbiology, Zhejiang Provincial Center for Disease Control and Prevention	Yanjun Zhang, Yin Chen, Haiyan Mao, Junhang Pan, Xiuyu Lou, Yiyu Lu, Juying Yan, Hanping Zhu, Jian Gao, Yan Feng, Yi Sun, Hao Yan, Zhen Li, Yisheng Sun, Liming Gong, Qiong Ge, Wen Shi, Xinying Wang, Wenwu Yao, Zhangnv Yang, Fang Xu, Chen Chen, Enfu Chen, Zhen Wang, Zhiping Chen, Jianmin Jiang, Chonggao Hu
EPI_ISL_406592	Shenzhen Third People's Hospital	Shenzhen Key Laboratory of Pathogen and Immunity, National Clinical Research Center for Infectious Disease,Shenzhen Third People's Hospital	Yang Yang, Chenguang Shen, Li Xing, Zhixiang Xu, Haixia Zheng, Yingxia Liu
EPI_ISL_406593, EPI_ISL_406594, EPI_ISL_406595	Shenzhen Key Laboratory of Pathogen and Immunity, National Clinical Research Center for Infectious Disease, Shenzhen Third People's Hospital	Shenzhen Key Laboratory of Pathogen and Immunity, National Clinical Research Center for Infectious Disease, Shenzhen Third People's Hospital	Yang Yang, Chenguang Shen, Li Xing, Zhixiang Xu, Haixia Zheng, Yingxia Liu
EPI_ISL_406862	Charité Universitätsmedizin Berlin, Institute of Virology; Institut für Mikrobiologie der Bundeswehr, Munich	Charité Universitätsmedizin Berlin, Institute of Virology	Victor M Corman, Julia Schneider, Talitha Veith, Barbara Mühlemann, Markus Antwerpen, Christian Drosten, Roman Wölfel
EPI_ISL_406970	Hangzhou Center for Disease and Control Microbiology Lab	Hangzhou Center for Disease and Control Microbiology Lab	Yu Hua, Wang Haoqiu, Li Jun, Yu Xinfeng
EPI_ISL_407079	Lapland Central Hospital	Department of Virology, University of Helsinki and Helsinki University Hospital, Helsinki, Finland	Teemu Smura, Suvi Kuivanen, Hannimari Kallio-Kokko, Olli Vapalahti
EPI_ISL_407084	Department of Virology III, National Institute of Infectious Diseases	Pathogen Genomics Center, National Institute of Infectious Diseases	Tsuyoshi Sekizuka, Shutoku Matsuyama, Naganori Nao, Kazuya Shirato, Shinji Watanabe, Makoto Takeda, Makoto Kuroda
EPI_ISL_407313	Hangzhou Center for Disease Control and Prevention	Hangzhou Center for Disease Control and Prevention	Jun Li, Haoqiu Wang, Hua Yu, Lingfeng Mao, Xinfen Yu, Zhou Sun, Qingxin Kong, Xin Qian, Shuchang Chen, Xuchu Wang
EPI_ISL_407893	Centre for Infectious Diseases and Microbiology Laboratory Services	NSW Health Pathology - Institute of Clinical Pathology and Medical Research; Westmead Hospital; University of Sydney	Eden J-S, Carter I, Rahman H, Holmes EC, Rockett R, O'Sullivan MV, Sintchenko V, Chen SC, Maddocks S, Kok J and Dwyer DE for the 2019-nCoV Study Group
EPI_ISL_407976	KU Leuven, Clinical and Epidemiological Virology	KU Leuven, Clinical and Epidemiological Virology	Bert Vanmechelen, Elke Wollants, Annabel Rector, Els Keyaerts, Lies Laenen, Marc Van Ranst, and Piet Maes
EPI_ISL_408430	Department of Infectious and Tropical Diseases, Bichat Claude Bernard Hospital, Paris	National Reference Center for Viruses of Respiratory Infections, Institut Pasteur, Paris	Mélanie Albert, Marion Barbet, Sylvie Behillil, Méline Bizard, Angela Brisebarre, Flora Donati, Vincent Enouf, Maud Vanpeene, Sylvie van der Werf, Yazdan Yazdanpanah, Xavier Lescure
EPI_ISL_408431	Sorbonne Université, Inserm et Assistance Publique-Hôpitaux de Paris (Pitié Salpétrière)	National Reference Center for Viruses of Respiratory Infections, Institut Pasteur, Paris	Mélanie Albert, Marion Barbet, Sylvie Behillil, Méline Bizard, Angela Brisebarre, Flora Donati, Vincent Enouf, Maud Vanpeene, Sylvie van der Werf, Sonia Burrel, Anne-Geneviève Marcelin, Vincent Calvez, David Boutolleau, Elise Klément, Valérie Pourcher, Eric Caumes.
EPI_ISL_408480	National Institute for Viral Disease Control and Prevention, China CDC	National Institute for Viral Disease Control & Prevention, CCDC	Wenjie TanXiaoqing FuXiang ZhaoWenling Wang Peihua NiuRoujian Lu,Yanhong SunBaoying HuangLi ZhaoFei YeWenbo XuGeorge F. GaoGuizhen Wu
EPI_ISL_408481	National Institute for Viral Disease Control and Prevention, China CDC	National Institute for Viral Disease Control & Prevention, CCDC	Wenjie Tan, Hengqin Wang, Xiang Zhao, Wenling Wang, Peihua Niu, Roujian Lu, Sheng Ye, Baoying Huang, Li Zhao, Fei Ye, Wenbo Xu, George F. Gao, Guizhen Wu
EPI_ISL_408482	National Institute for Viral Disease Control and Prevention, China CDC	National Institute for Viral Disease Control & Prevention, CCDC	Wenjie Tan, Zhaoguo Wang, Xiang Zhao, Wenling Wang, Peihua Niu, Roujian Lu, Ti Liu, Baoying Huang, Li Zhao, Fei Ye, Wenbo Xu, George F. Gao, Guizhen Wu
EPI_ISL_408483	National Institute for Viral Disease Control and Prevention, China CDC	National Institute for Viral Disease Control & Prevention, CCDC	Wenjie TanZhen Teng,Xiang ZhaoWenling Wang Peihua NiuRoujian Lu,Chongshan Li,Baoying HuangLi ZhaoFei YeWenbo XuGeorge F. GaoGuizhen Wu
EPI_ISL_408484	National Institute for Viral Disease Control and Prevention, China CDC	National Institute for Viral Disease Control & Prevention, CCDC	Wenjie Tan, Jianan Xu, Wenling Wang, Peihua Niu, Roujian Lu, Huiping Yang, Xiang Zhao, Baoying Huang, Li Zhao, Fei Ye, Wenbo Xu, George F. Gao, Guizhen Wu
EPI_ISL_408485	National Institute for Viral Disease Control and Prevention, China CDC	National Institute for Viral Disease Control & Prevention, CCDC	Wenjie Tan,Quanyi Wang,Wenling Wang, Peihua Niu,Roujian Lu,Yang Pan,Xiang Zhao,Baoying Huang,Li Zhao,Fei Ye,Wenbo Xu,George F. Gao,Guizhen Wu
EPI_ISL_408486	National Institute for Viral Disease Control and Prevention, China CDC	National Institute for Viral Disease Control & Prevention, CCDC	Wenjie Tan, Yong Shi, Wenling Wang, Peihua Niu, Roujian Lu, Jianxiong Li, Xiang Zhao, Baoying Huang, Li Zhao, Fei Ye, Wenbo Xu, George F. Gao, Guizhen Wu
EPI_ISL_408487	National Institute for Viral Disease Control and Prevention, China CDC	National Institute for Viral Disease Control & Prevention, China CDC	Wenjie Tan, Jin Xu, Wenling Wang, Peihua Niu, Roujian Lu, Xueyong Huang, Xiang Zhao, Baoying Huang, Li Zhao, Fei Ye, Wenbo Xu, George F. Gao, Guizhen Wu
EPI_ISL_408488	National Institute for Viral Disease Control and Prevention, China CDC	National Institute for Viral Disease Control & Prevention, CCDC	Wenjie Tan, Shenjiao Wang, Wenling Wang, Peihua Niu, Roujian Lu, Kangchen Zhao, Xiang Zhao, Baoying Huang, Li Zhao, Fei Ye, Wenbo Xu, George F. Gao, Guizhen Wu
EPI_ISL_408489	Department of Laboratory Medicine, National Taiwan University Hospital	Microbial Genomics Core Lab, National Taiwan University Centers of Genomic and Precision Medicine	Shiou-Hwei Yeh, You-Yu Lin, Ya-Yun Lai, Chiao-Ling Li, Shan-Chwen Chang, Pei-Jer Chen, Sui-Yuan Chang
EPI_ISL_408666, EPI_ISL_408669	Dept. of Virology III, National Institute of Infectious Diseases	Pathogen Genomics Center, National Institute of Infectious Diseases	Tsuyoshi Sekizuka, Shutoku Matsuyama, Naganori Nao, Kazuya Shirato, Makoto Takeda, Makoto Kuroda
EPI_ISL_408976	Centre for Infectious Diseases and Microbiology Laboratory Services	NSW Health Pathology - Institute of Clinical Pathology and Medical Research; Westmead Hospital; University of Sydney	Rockett R, Sadsad R, Eden J-S, Carter I, Rahman H, Holmes EC, O'Sullivan MV, Sintchenko V, Chen SC, Maddocks S, Kok J and Dwyer DE for the 2019-nCoV Study Group*
EPI_ISL_408977	Serology, Virology and OTDS Laboratories (SAViD), NSW Health Pathology Randwick	NSW Health Pathology - Institute of Clinical Pathology and Medical Research; Centre for Infectious Diseases and	Eden J-S, Carter I, Rahman H, Rawlinson W, Holmes EC, Rockett R, O'Sullivan MV, Sintchenko V, Chen SC, Maddocks S, Kok J and Dwyer DE for the 2019-nCoV Study Group*

		Microbiology Laboratory Services; Westmead Hospital; University of Sydney	
EPI_ISL_410218	Department of Laboratory Medicine, National Taiwan University Hospital	Microbial Genomics Core Lab, National Taiwan University Centers of Genomic and Precision Medicine	Shiou-Hwei Yeh, You-Yu Lin, Ya-Yun Lai, Chiao-Ling Li, Shan-Chwen Chang, Pei-Jer Chen, Sui-Yuan Chang
EPI_ISL_410301	National Influenza Centre, National Public Health Laboratory, Kathmandu, Nepal	The University of Hong Kong	Ranjit Sah , Runa Jha, Daniel Chu, Haogao Gu, Malik Peiris, Anup Bastola, Alfonso J. Rodriguez-Morales, Bibek Kumar Lal, Basu Dev Pandey, Leo Poon
EPI_ISL_410531, EPI_ISL_410532	Dept. of Pathology, National Institute of Infectious Diseases	Pathogen Genomics Center, National Institute of Infectious Diseases	Tsuyoshi Sekizuka, Harutaka Katano, Shutoku Matsuyama, Naganori Nao, Kazuya Shirato, Motoi Suzuki, Hideki Hasegawa, Takaji Wakita, Makoto Takeda, Tadaki Suzuki, Makoto Kuroda
EPI_ISL_410545	INMI Lazzaro Spallanzani IRCCS	Laboratory of Virology, INMI Lazzaro Spallanzani IRCCS	Maria R. Capobianchi, Cesare E. M. Gruber, Martina Rueca, Barbara Bartolini, Francesco Messina, Emanuela Giombini, Francesca Colavita, Concetta Castilletti, Eleonora Lalle, Fabrizio Carletti, Emanuele Nicastri, Giuseppe Ippolito.
EPI_ISL_410546	INMI Lazzaro Spallanzani IRCCS	Laboratory of Virology, INMI Lazzaro Spallanzani IRCCS	Maria R. Capobianchi, Cesare E. M. Gruber, Martina Rueca, Fabrizio Carletti, Barbara Bartolini, Francesco Messina, Emanuela Giombini, Francesca Colavita, Concetta Castilletti, Eleonora Lalle, Emanuele Nicastri, Giuseppe Ippolito.
EPI_ISL_410984	Department of Infectious and Tropical Diseases, Bichat Claude Bernard Hospital, Paris	National Reference Center for Viruses of Respiratory Infections, Institut Pasteur, Paris	Mélanie Albert, Marion Barbet, Sylvie Behillil, Méline Bizard, Angela Brisebarre, Flora Donati, Vincent Enouf, Maud Vanpeene, Sylvie van der Werf, Yazdan Yazdanpanah, Xavier Lescure
EPI_ISL_411218	Department of Infectious and Tropical Diseases, Bichat Claude Bernard Hospital, Paris	Laboratoire Virpath, CIRI U111, UCBL1, INSERM, CNRS, ENS Lyon	Olivier Terrier, Aurélien Traversier, Julien Fouret, Yazdan Yazdanpanah, Xavier Lescure, Catherine Legras-Lachuer, Alexandre Gaymard, Bruno Lina, Manuel Rosa-Calatrava
EPI_ISL_411219, EPI_ISL_411220	Department of Infectious and Tropical Diseases, Bichat Claude Bernard Hospital, Paris	Laboratoire Virpath, CIRI U111, UCBL1, INSERM, CNRS, ENS Lyon	Olivier Terrier, Aurélien Traversier, Julien Fouret, Yazdan Yazdanpanah, Xavier Lescure, Alexandre Gaymard, Bruno Lina, Manuel Rosa-Calatrava
EPI_ISL_411902	Virology Unit, Institut Pasteur du Cambodge.	Virology Unit, Institut Pasteur du Cambodge (Sequencing done by: Jessica E Manning/Jennifer A Bohl at Malaria and Vector Research Research Laboratory, National Institute of Allergy and Infectious Diseases and Vida Ahyong from Chan-Zuckerberg Biohub)	Erik A Karlsson, Jennifer A Bohl, Vida Ahyong, Veasna Duong, Philippe Dussart, Jessica E Manning.
EPI_ISL_412386	Beijing Ditan Hospital, Capital Medical University	National Institute for Communicable Disease Control and Prevention, Chinese Center for Disease Control and Prevention	Xinmin Xu, Xin Lu, Pan Xiang, Haijian Zhou, Biao Kan, Yajie Wang, Jingyuan Liu, Yanwen Xiong, Huizhu Wang, Ruihong Li, Fangfang Jin, Jie Gong, Xiaoping Chen, Lili Gao, Haofeng Xiong, Lin Pu, Chuansheng Li, Ming Zhang, Jianbo Tan, Yao Sun, Yufeng Liu, Hebing Guo, Jingjing Hao
EPI_ISL_412459	Jingzhou Center for Disease Control and Prevention	Hubei Provincial Center for Disease Control and Prevention	Bin Fang, Xiang Li, Xiao Yu, Linlin Liu, Bo Yang, Faxian Zhan, Guojun Ye, Xixiang Huo, Junqiang Xu, Bo Yu, Kun Cai, Jing Li, Maoyi Chen,Jie Hu, Chunlin Mao, Yongzhong Jiang.
EPI_ISL_412964	Hospital Israelita Albert Einstein	Instituto Adolfo Lutz Interdisciplinary Procedures Center Strategic Laboratory	Jaqueline Goes de Jesus, Claudio Tavares Sacchi, Daniela Bernardes Borges da Silva, Ingra Morales Claro, Flávia Cristina da Silva Sales, Claudia Regina Gonçalves, Joshua Quick, Maria do Carmo, Sampaio Tavares Timenetsky, Nicholas James Loman, Andrew Rambaut, Ester Cerdeira Sabino, Nuno Rodrigues Faria
EPI_ISL_412970	Washington State Department of Health	Seattle Flu Study	Helen Chu, Michael Boeckh, Janet Englund, Michael Famulare, Barry Lutz, Deborah Nickerson, Mark Rieder, Lea Starita, Matthew Thompson, Jay Shendure, and Trevor Bedford
EPI_ISL_412971	HUS Diagnostiikkakeskus, Hallinto	Department of Virology Faculty of Medicine, Medicum University of Helsinki	Teemu Smura, Suvi Kuivanen, Hannimari Kallio-Kokko, Olli Vapalahti
EPI_ISL_412972	Instituto Nacional de Enfermedades Respiratorias	Instituto de Diagnostico y Referencia Epidemiologicos (INDRE)	Ramirez-Gonzalez Ernesto, Garces-Ayala Fabiola, Araiza-Rodriguez Adnan, Mendieta-Condado Edgar, Rodriguez-Maldonado Abril, Wong-Arambula Claudia, Vazquez-Perez Joel, Martinez Arturo, Boukadida Celia, Munoz-Medina Esteban, Sanchez Alejandro, Isa Pavel, Taboada Blanca, Lopez Susana, Arias Carlos, Barrera-Badillo Gisela, Hernandez-Rivas Lucia, Lopez-Martinez Irma
EPI_ISL_412975	Centre for Infectious Diseases and Microbiology Laboratory Services	NSW Health Pathology - Institute of Clinical Pathology and Medical Research; Westmead Hospital; University of Sydney	Eden J-S, Carter I, Rahman H, Holmes EC, Rockett R, O'Sullivan MV, Sintchenko V, Chen SC, Maddocks S, Kok J and Dwyer DE for the 2019-nCoV Study Group
EPI_ISL_412978	The Central Hospital Of Wuhan	Hubei Provincial Center for Disease Control and Prevention	Bin Fang, Xiang Li, Xiao Yu, Linlin Liu, Bo Yang, Faxian Zhan, Guojun Ye, Xixiang Huo, Junqiang Xu, Bo Yu, Kun Cai, Jing Li, Yongzhong Jiang.
EPI_ISL_412979, EPI_ISL_412980	Union Hospital of Tongji Medical College, Huazhong University of Science and Technology	Hubei Provincial Center for Disease Control and Prevention	Bin Fang, Xiang Li, Xiao Yu, Linlin Liu, Bo Yang, Faxian Zhan, Guojun Ye, Xixiang Huo, Junqiang Xu, Bo Yu, Kun Cai, Jing Li, Yongzhong Jiang.
EPI_ISL_412981	CR&WISCO GENERAL HOSPITAL	Hubei Provincial Center for Disease Control and Prevention	Bin Fang, Xiang Li, Xiao Yu, Linlin Liu, Bo Yang, Faxian Zhan, Guojun Ye, Xixiang Huo, Junqiang Xu, Bo Yu, Kun Cai, Jing Li, Yongzhong Jiang.
EPI_ISL_412982	Wuhan Lung Hospital	Hubei Provincial Center for Disease Control and Prevention	Bin Fang, Xiang Li, Xiao Yu, Linlin Liu, Bo Yang, Faxian Zhan, Guojun Ye, Xixiang Huo, Junqiang Xu, Bo Yu, Kun Cai, Jing Li, Yongzhong Jiang.
EPI_ISL_412983	Tianmen Center for Disease Control and Prevention	Hubei Provincial Center for Disease Control and Prevention	Bin Fang, Xiang Li, Xiao Yu, Linlin Liu, Bo Yang, Faxian Zhan, Guojun Ye, Xixiang Huo, Junqiang Xu, Bo Yu, Kun Cai, Jing Li, YiFa Zhu, Yangyang Tao,Xierong Li,Yongzhong Jiang.
EPI_ISL_413016	Hospital Israelita Albert Einstein	Instituto Adolfo Lutz, Interdisciplinary Procedures Center, Strategic Laboratory	Jaqueline Goes de Jesus, Claudio Tavares Sacchi, Fabiana Cristina Pereira dos Santos, Ingra Morales Claro, Flávia Cristina da Silva Sales, Claudia Regina Gonçalves, Joshua Quick, Maria do Carmo Sampaio Tavares Timenetsky, Nicholas James Loman, Andrew Rambaut, Ester Cerdeira Sabino, Nuno Rodrigues Faria
EPI_ISL_413213, EPI_ISL_413214	Centre for Infectious Diseases and Microbiology Laboratory Services	NSW Health Pathology - Institute of Clinical Pathology and Medical Research; Westmead Hospital; University of Sydney	Eden J-S, Carter I, Rahman H, Holmes EC, Rockett R, O'Sullivan MV, Sintchenko V, Chen SC, Maddocks S, Kok J and Dwyer DE for the 2019-nCoV Study Group*
EPI_ISL_413459	Department of Pathology, Toshima Hospital	Pathogen Genomics Center, National Institute of Infectious Diseases	Tsuyoshi Sekizuka, Kentaro Itokawa, Takuya Adachi, Masahiro Sano, Jun Yamazaki, Ippei Miyamoto, Haruka Nishioka, Ja-Mun Chong, Noriko Nakajima, Yuko Sato, Minoru Tobiume, Harutaka Katano, Tadaki Suzuki, Makoto Kuroda
EPI_ISL_413490	Auckland Hospital	Institute of Environmental Science and Research (ESR)	Matt Storey, Xiaoyun Ren, Gary McAuliffe, Sally Roberts, Matthew Blakiston, Erasmus Smit, Lauren Jelly, Joep de Ligt
EPI_ISL_413522	Indian Council of Medical Research - National Institute of Virology	National Influenza Center, Indian Council of Medical Research - National Institute of Virology	Potdar V, Yadav PD, Choudhary ML, Shete-Aich A
EPI_ISL_413523	Indian Council of Medical Research-National Institute of Virology	National Influenza Center, Indian Council of Medical Research-National Institute of Virology	Potdar V, Yadav PD, Choudhary ML, Shete-Aich A
EPI_ISL_413550	Centre for Human and Zoonotic Virology (CHAZVY), College of Medicine University of Lagos/Lagos University Teaching Hospital (LUTH), part of the Laboratory Network of the Nigeria Centre for Disease Control (NCDC)	African Centre of Excellence for Genomics of Infectious Diseases (ACEGID), Redeemer's University, Ede, Osun State, Nigeria	Oluniyi P.E., Ajogbasile F.V., Kayode A., Oguzie J., Folarin O.A., Ihekweazu C. Happi C.T.
EPI_ISL_413594	Centre for Infectious Diseases and Microbiology Laboratory Services	NSW Health Pathology - Institute of Clinical Pathology and Medical Research; Westmead Hospital; University of Sydney	Rockett R, Eden J-S, Lam C, Gray K, Timms, V, Gall, M, Alicia, A, Carter I, Rahman H, Holmes EC, , O'Sullivan MV, Sintchenko V, Chen SC, Maddocks S, Kok J and Dwyer DE for the 2019-nCoV Study Group*
EPI_ISL_413595	Centre for Infectious Diseases and Microbiology Laboratory Services	NSW Health Pathology - Institute of Clinical Pathology and Medical Research; Westmead Hospital; University of Sydney	Rockett R, Eden J-S, Lam C, Gray K, Timms, V, Gall, M, Carter I, Rahman H, Holmes EC, O'Sullivan MV, Sintchenko V, Chen SC, Maddocks S, Kok J and Dwyer DE for the 2019-nCoV Study Group*
EPI_ISL_413596	Centre for Infectious Diseases and Microbiology - Public Health	NSW Health Pathology - Institute of Clinical Pathology and Medical Research; Westmead Hospital; University of Sydney	Rockett R, Eden J-S, Lam C, Gray K, Timms, V, Gall, M, Carter I, Rahman H, Holmes EC, O'Sullivan MV, Sintchenko V, Chen SC, Maddocks S, Kok J and Dwyer DE for the 2019-nCoV Study Group*
EPI_ISL_413597	Centre for Infectious Diseases and Microbiology- Public Health	NSW Health Pathology - Institute of Clinical Pathology and Medical Research; Westmead Hospital; University of Sydney	Lam C, Eden J-S, Rockett R, Gray K, Timms, V, Gall, M, Carter I, Rahman H, Holmes EC, O'Sullivan MV, Sintchenko V, Chen SC, Maddocks S, Kok J and Dwyer DE for the 2019-nCoV Study Group*

EPI_ISL_413598	Centre for Infectious Diseases and Microbiology - Public Health	NSW Health Pathology - Institute of Clinical Pathology and Medical Research; Westmead Hospital; University of Sydney	Gray K, Eden J-S, Lam C, Rockett R, Timms, V, Gall, M, Carter I, Rahman H, Holmes EC, O'Sullivan MV, Sintchenko V, Chen SC, Maddocks S, Kok J and Dwyer DE for the 2019-nCoV Study Group*
EPI_ISL_413599	Centre for Infectious Diseases and Microbiology - Public Health	NSW Health Pathology - Institute of Clinical Pathology and Medical Research; Westmead Hospital; University of Sydney	Timms, V, Eden J-S, Lam C, Gray K, Rockett R, Gall, M, Carter I, Rahman H, Holmes EC, O'Sullivan MV, Sintchenko V, Chen SC, Maddocks S, Kok J and Dwyer DE for the 2019-nCoV Study Group*
EPI_ISL_413600	Centre for Infectious Diseases and Microbiology - Public Health	NSW Health Pathology - Institute of Clinical Pathology and Medical Research; Westmead Hospital; University of Sydney	Gall, M, Eden J-S, Lam C, Gray K, Timms, V, Rockett R, Carter I, Rahman H, Holmes EC, O'Sullivan MV, Sintchenko V, Chen SC, Maddocks S, Kok J and Dwyer DE for the 2019-nCoV Study Group*
EPI_ISL_413647	Centro Hospital do Porto, E.P.E. - H. Geral de Santo Antonio	Instituto Nacional de Saude (INSA)	Raquel Guiomar, Inês Costa, Pedro Pechirra, Joana Mendonça, Luís Vieira, Helena Ramos, Joana Isidro, Vítor Borges, João Paulo Gomes
EPI_ISL_413648	Centro Hospitalar e Universitário de Sao Joao, Porto	Instituto Nacional de Saude (INSA)	Raquel Guiomar, Inês Costa, Pedro Pechirra, Joana Mendonça, Luís Vieira, João Tiago Guimarães, Joana Isidro, Vítor Borges, João Paulo Gomes
EPI_ISL_414014	Hospital Israelita Albert Einstein	Instituto Adolfo Lutz, Interdiciplinary Procedures Center, Strategic Laboratory	Claudio Tavares Sacchi, Claudia Regina Gonçalves, Katia Correia dos Santos, Carlos Henrique Camargo, Maria do Carmo Sampaito Tavares Timenetsky, Terezinha Maria de Paiva, Ester Cerdeira Sabino
EPI_ISL_414015	Hospital São Joaquim Beneficencia Portuguesa	Instituto Adolfo Lutz, Interdiciplinary Procedures Center, Strategic Laboratory	Claudio Tavares Sacchi, Claudia Regina Gonçalves, SimoneGuadagnucci Morillo, Carlos Henrique Camargo, Maria do Carmo Sampaito Tavares Timenetsky, Fabiana Cristina Pereira dos Santos Terezinha Maria de Paiva, Ester Cerdeira Sabino
EPI_ISL_414016	Hospital São Joaquim Beneficencia Portuguesa	Instituto Adolfo Lutz, Interdiciplinary Procedures Center, Strategic Laboratory	Claudio Tavares Sacchi, Claudia Regina Gonçalves, Audrey Cilli, Carlos Henrique Camargo, Maria do Carmo Sampaito Tavares Timenetsky, Daniela Bernardes Borges da Silva, Terezinha Maria de Paiva, Ester Cerdeira Sabino
EPI_ISL_414017	Hospital São Joaquim Beneficencia Portuguesa	Instituto Adolfo Lutz, Interdiciplinary Procedures Center, Strategic Laboratory	Claudio Tavares Sacchi, Claudia Regina Gonçalves, Fabiana Cristina Pereira dos Santos, Carlos Henrique Camargo, Maria do Carmo Sampaito Tavares Timenetsky, Daniela Bernardes Borges da Silva, Terezinha Maria de Paiva, Ester Cerdeira Sabino
EPI_ISL_414477	The National Institute of Public Health Center for Epidemiology and Microbiology	State Veterinary Institute Prague	Alexander Nagy, Oldrich Bartos, Helena Jirincova, Klara Labska, Ludmila Novakova, Olga Storkanova, Dusan Trnka, Jaromira Vecerova
EPI_ISL_414495	Servicio Microbiología. Hospital Clínico Universitario. Valencia.	Sequencing and Bioinformatics Service. Molecular Epidemiology Laboratory. FISABIO-Public Health	David Navarro, Maria Alma Bracho, Giuseppe D'Auria, Griselda De Marco, Neris Garcia-Gonzalez, Fernando Gonzalez-Candelas
EPI_ISL_414496	Servicio Microbiologia. Hospital Clinico Universitario. Valencia.	Sequencing and Bioinformatics Service. Molecular Epidemiology Laboratory. FISABIO-Public Health	David Navarro, María Alma Bracho, Giuseppe D'Auria, Griselda De Marco, Neris Garcia-Gonzalez, Fernando Gonzalez-Candelas
EPI_ISL_414520, EPI_ISL_414521	Bundeswehr Institute of Microbiology	Bundeswehr Institute of Microbiology	Mathias C Walter, Markus H Antwerpen and Roman Wölfel
EPI_ISL_414577	Hospital de Talca, Chile	Instituto de Salud Publica de Chile	Andrés E. Castillo, Bárbara Parra, Paz Tapia, Alejandra Acevedo, Jaime Lagos, Winston Andrade, Loredana Arata, Gabriel Leal, Gisselle Barra, Carolina Tambley, Javier Tognarelli, Patricia Bustos, Soledad Ulloa, Rodrigo Fasce, Jorge Fernández.
EPI_ISL_414598	Servicio Microbiologia, Hospital Clinico Universitario, Valencia	Sequencing and Bioinformatics Service and Molecular Epidemiology Research Group. FISABIO-Public Health.	David Navarro, Maria Alma Bracho, Giuseppe D'Auria, Griselda De Marco, Neris Garcia-Gonzalez, Fernando Gonzalez-Candelas
EPI_ISL_414623	Laboratoire de Virologie Institut de Virologie - INSERM U 1109 Hôpitaux Universitaires de Strasbourg	National Reference Center for Viruses of Respiratory Infections, Institut Pasteur, Paris	Mélnie Albert, Marion Barbet, Sylvie Behillil, Méline Bizard, Angela Brisebarre, Flora Donati Vincent Enouf, Maud Vanpeene, Sylvie van der Werf, Samira Fafi-Kremer
EPI_ISL_414624	Centre Hositalier Universitaire de Rouen Laboratoire de Virologie	National Reference Center for Viruses of Respiratory Infections, Institut Pasteur, Paris	Mélnie Albert, Marion Barbet, Sylvie Behillil, Méline Bizard, Angela Brisebarre, Flora Donati Vincent Enouf, Maud Vanpeene, Sylvie van der Werf, Jean-Christophe Plantier
EPI_ISL_414625	Centre Hospitalier Régional Universitaire de Nantes Laboratoire de Virologie	National Reference Center for Viruses of Respiratory Infections, Institut Pasteur, Paris	Mélnie Albert, Marion Barbet, Sylvie Behillil, Méline Bizard, Angela Brisebarre, Flora Donati Vincent Enouf, Maud Vanpeene, Sylvie van der Werf, Marianne Coste-Burel
EPI_ISL_414626	unknown	National Reference Center for Viruses of Respiratory Infections, Institut Pasteur, Paris	Mélnie Albert, Marion Barbet, Sylvie Behillil, Méline Bizard, Angela Brisebarre, Flora Donati Vincent Enouf, Maud Vanpeene, Sylvie van der Werf
EPI_ISL_414627, EPI_ISL_414628, EPI_ISL_414629, EPI_ISL_414630	Centre Hospitalier Compiègne Laboratoire de Biologie	National Reference Center for Viruses of Respiratory Infections, Institut Pasteur, Paris	Mélnie Albert, Marion Barbet, Sylvie Behillil, Méline Bizard, Angela Brisebarre, Flora Donati Vincent Enouf, Maud Vanpeene, Sylvie van der Werf, Raulin Olivia
EPI_ISL_414631, EPI_ISL_414632	Hôpital Robert Debré Laboratoire de Virologie	National Reference Center for Viruses of Respiratory Infections, Institut Pasteur, Paris	Mélnie Albert, Marion Barbet, Sylvie Behillil, Méline Bizard, Angela Brisebarre, Flora Donati Vincent Enouf, Maud Vanpeene, Sylvie van der Werf, Laurent Andreoletti
EPI_ISL_414633	Centre Hospitalier René Dubois Laboratoire de Microbiologie - Bât A	National Reference Center for Viruses of Respiratory Infections, Institut Pasteur, Paris	Mélnie Albert, Marion Barbet, Sylvie Behillil, Méline Bizard, Angela Brisebarre, Flora Donati Vincent Enouf, Maud Vanpeene, Sylvie van der Werf, Pascale Martres
EPI_ISL_414634, EPI_ISL_414635, EPI_ISL_414636, EPI_ISL_414637, EPI_ISL_414638	Centre Hospitalier Compiègne Laboratoire de Biologie	National Reference Center for Viruses of Respiratory Infections, Institut Pasteur, Paris	Mélnie Albert, Marion Barbet, Sylvie Behillil, Méline Bizard, Angela Brisebarre, Flora Donati Vincent Enouf, Maud Vanpeene, Sylvie van der Werf, Raulin Olivia
EPI_ISL_414647	Viral Respiratory Lab, National Institute for Biomedical Research (INRB)	Pathogen Sequencing Lab, National Institute for Biomedical Research (INRB)	Placide Mbala-Kingebeni, Edith Nkwembe, Eddy Kinganda-Lusamaki, Amuri Aziza, Catherine Pratt, Matthias Pauthner, Josh Quick, Allison Black, James Hadfield, Trevor Bedford, Ian Goodfellow, Nick Loman, Kristian Andersen, Michael Wiley, Steve Ahuka-Mundeke, Jean-Jacques Muyembe Tamfum
EPI_ISL_414663, EPI_ISL_414691	State Key Laboratory of Respiratory Disease, National Clinical Research Center for Respiratory Disease, Guangzhou Institute of Respiratory Health, the First Affiliated Hospital of Guangzhou Medical University	The First Affiliated Hospital of Guangzhou Medical University & BGI-Shenzhen	Zhao et al
EPI_ISL_415153	KU Leuven, Clinical and Epidemiological Virology	KU Leuven, Clinical and Epidemiological Virology	Bert Vanmechelen, Joan Marti-Carreras, Tony Wawina, Marc Van Ranst, Piet Maes
EPI_ISL_415154	KU Leuven, Clincal and Epidemiological Virology	KU Leuven, Clincal and Epidemiological Virology	Bert Vanmechelen, Joan Marti-Careras, Tony Wawina, Marc Van Ranst, Piet Maes.
EPI_ISL_415155	KU Leuven, Clinical and Epidemiological Virology	KU Leuven, Clinical and Epidemiological Virology	Bert Vanmechelen, Joan Marti-Carreras, Tony Wawina, Marc Van Ranst, Piet Maes
EPI_ISL_415156, EPI_ISL_415157, EPI_ISL_415158, EPI_ISL_415159	KU Leuven, Clinical and Epidemiological Virology	KU Leuven, Clinical and Epidemiological Virology	Bert Vanmechelen, Joan Marti-Carreras, Tony Wawina, Piet Maes
EPI_ISL_415641, EPI_ISL_415642, EPI_ISL_415643, EPI_ISL_415644	R. G. Lugar Center for Public Health Research, National Center for Disease Control and Public Health (NCDC) of Georgia.	R. G. Lugar Center for Public Health Research, National Center for Disease Control and Public Health (NCDC) of Georgia.	Nato Kotaria, Marine Murtskhvaladze, Ann Machablishvili, Lela Sabadze, Mari Gavashelidze, Ana Papkiauri, Meri Pantsulaia, Gvantsa Brachveli, Tata Imnadze, Tamar Jashiashvili, Tea Tevdoradze, Ketevan Sidamonidze, Ekaterine Khmaladze, Ekaterine Zhghenti, Roena Sukhiashvili, Mariam Zakalashvili, Lela Urushadze, Magda Dgebuadze, Giorgi Tomashvili, Davit Tsaguria, Ekaterine Zangaladze, Nino Berishvili, Gvantsa Chanturia, Adam Kotorashvili, Maia Alkhazashvili, Irma Burjanadze, Anna Kasradze, Khatuna Zakhashvili, Paata Imnadze, Amiran Gamkrelidze.
EPI_ISL_415649	unknown	National Reference Center for Viruses of Respiratory Infections, Institut Pasteur, Paris	Mélnie Albert, Marion Barbet, Sylvie Behillil, Méline Bizard, Angela Brisebarre, Flora Donati Vincent Enouf, Maud Vanpeene, Sylvie van der Werf
EPI_ISL_415650	Hôpital Instruction des Armées - BEGIN	National Reference Center for Viruses of Respiratory Infections, Institut Pasteur, Paris	Mélnie Albert, Marion Barbet, Sylvie Behillil, Méline Bizard, Angela Brisebarre, Flora Donati Vincent Enouf, Maud Vanpeene, Sylvie van der Werf, Christine Bigaillon
EPI_ISL_415652	unknown	National Reference Center for Viruses of Respiratory Infections, Institut Pasteur, Paris	Mélnie Albert, Marion Barbet, Sylvie Behillil, Méline Bizard, Angela Brisebarre, Flora Donati Vincent Enouf, Maud Vanpeene, Sylvie van der Werf
EPI_ISL_415653, EPI_ISL_415654	Centre Hospitalier Compiègne Laboratoire de Biologie	National Reference Center for Viruses of Respiratory Infections, Institut Pasteur, Paris	Mélnie Albert, Marion Barbet, Sylvie Behillil, Méline Bizard, Angela Brisebarre, Flora Donati Vincent Enouf, Maud Vanpeene, Sylvie van der Werf, Raulin Olivia
EPI_ISL_415709	State Key Laboratory for Diagnosis and Treatment of Infectious Diseases, National Clinical Research Center for Infectious Diseases, First Affiliated Hospital, Zhejiang University School of Medicine, Hangzhou, China. 310003	State Key Laboratory for Diagnosis and Treatment of Infectious Diseases, National Clinical Research Center for Infectious Diseases, First Affiliated Hospital, Zhejiang University School of Medicine, Hangzhou, China. 310003	Hangping Yao, Nanping Wu, Chao Jiang, Xiangyun Lu, Linfang Cheng, Fumin Liu, Zhigang Wu, Haibo Wu, Changzhong Jin, Min Zheng, Lanjuan Li

EPI_ISL_415710	WHO National Influenza Centre Russian Federation	WHO National Influenza Centre Russian Federation	Andrey Komissarov, Artem Fadeev, Anna Ivanova, Daria Danilenko
EPI_ISL_415711	State Key Laboratory for Diagnosis and Treatment of Infectious Diseases, National Clinical Research Center for Infectious Diseases, First Affiliated Hospital, Zhejiang University School of Medicine, Hangzhou, China. 310003	State Key Laboratory for Diagnosis and Treatment of Infectious Diseases, National Clinical Research Center for Infectious Diseases, First Affiliated Hospital, Zhejiang University School of Medicine, Hangzhou, China. 310003	Hangping Yao, Nanping Wu, Chao Jiang, Xiangyun Lu, Linfang Cheng, Fumin Liu, Zhigang Wu, Haibo Wu, Changzhong Jin, Min Zheng, Lanjuan Li
EPI_ISL_416028	National Influenza Center - Instituto Adolfo Lutz	Instituto Adolfo Lutz, Interdiciplinary Procedures Center, Strategic Laboratory	Claudio Tavares Sacchi, Claudia Regina Gonçalves, Carlos Henrique Camargo, Fabiana Cristina Pereira dos Santos, Daniela Bernardes Borges da Silva, Simone Guadagnucci Morillo, Adriano Abbud, Adriana Bugno, Maria do Carmo Sampaio Tavares Timenetsky, Terezinha Maria de Paiva
EPI_ISL_416029	Laboiratório Fleury	Instituto Adolfo Lutz, Interdiciplinary Procedures Center, Strategic Laboratory	Claudio Tavares Sacchi, Claudia Regina Gonçalves, Carlos Henrique Camargo, Fabiana Cristina Pereira dos Santos, Daniela Bernardes Borges da Silva, Simone Guadagnucci Morillo, Adriano Abbud, Adriana Bugno, Maria do Carmo Sampaio Tavares Timenetsky, Terezinha Maria de Paiva
EPI_ISL_416031, EPI_ISL_416032	National Influenza Center - Instituto Adolfo Lutz	Instituto Adolfo Lutz, Interdiciplinary Procedures Center, Strategic Laboratory	Claudio Tavares Sacchi, Claudia Regina Gonçalves, Carlos Henrique Camargo, Fabiana Cristina Pereira dos Santos, Daniela Bernardes Borges da Silva, Simone Guadagnucci Morillo, Adriano Abbud, Adriana Bugno, Maria do Carmo Sampaio Tavares Timenetsky, Terezinha Maria de Paiva
EPI_ISL_416033, EPI_ISL_416034	Hospital Israelita Albert Einstein	Instituto Adolfo Lutz, Interdiciplinary Procedures Center, Strategic Laboratory	Claudio Tavares Sacchi, Claudia Regina Gonçalves, Carlos Henrique Camargo, Erica Valessa Ramos Gomes, Fabiana Cristina Pereira dos Santos, Daniela Bernardes Borges da Silva, Simone Guadagnucci Morillo, Adriano Abbud, Adriana Bugno, Maria do Carmo Sampaio Tavares Timenetsky, Terezinha Maria de Paiva
EPI_ISL_416035, EPI_ISL_416036	National Influenza Center - Instituto Adolfo Lutz	Instituto Adolfo Lutz, Interdiciplinary Procedures Center, Strategic Laboratory	Claudio Tavares Sacchi, Claudia Regina Gonçalves, Carlos Henrique Camargo, Erica Valessa Ramos Gomes, Fabiana Cristina Pereira dos Santos, Daniela Bernardes Borges da Silva, Simone Guadagnucci Morillo, Adriano Abbud, Adriana Bugno, Maria do Carmo Sampaio Tavares Timenetsky, Terezinha Maria de Paiva
EPI_ISL_416042	State Key Laboratory for Diagnosis and Treatment of Infectious Diseases, National Clinical Research Center for Infectious Diseases, First Affiliated Hospital, Zhejiang University School of Medicine, Hangzhou, China. 310003	State Key Laboratory for Diagnosis and Treatment of Infectious Diseases, National Clinical Research Center for Infectious Diseases, First Affiliated Hospital, Zhejiang University School of Medicine, Hangzhou, China. 310003	Hangping Yao, Nanping Wu, Chao Jiang, Xiangyun Lu, Linfang Cheng, Fumin Liu, Zhigang Wu, Haibo Wu, Changzhong Jin, Min Zheng, Lanjuan Li
EPI_ISL_416044, EPI_ISL_416046, EPI_ISL_416047	State Key Laboratory for Diagnosis and Treatment of Infectious Diseases, National Clinical Research Center for Infectious Diseases, First Affiliated Hospital, Zhejiang University School of Medicine, Hangzhou, China 310003	State Key Laboratory for Diagnosis and Treatment of Infectious Diseases, National Clinical Research Center for Infectious Diseases, First Affiliated Hospital, Zhejiang University School of Medicine, Hangzhou, China 310003	Hangping Yao, Nanping Wu, Chao Jiang, Xiangyun Lu, Linfang Cheng, Fumin Liu, Zhigang Wu, Haibo Wu, Changzhong Jin, Min Zheng, Lanjuan Li
EPI_ISL_416142	Department of Virus and Microbiological Special diagnostics, Statens Serum Institut, Copenhagen, Denmark.	Statens Serum Institute	Morten Rasmussen, Maiken Worsoe Rosenstierne , Anders Fomsgaard
EPI_ISL_416143, EPI_ISL_416144	Department of Virus and Microbiological Special diagnostics, Statens Serum Institut, Copenhagen, Denmark.	ViFU	Morten Rasmussen, Maiken Worsoe Rosenstierne , Anders Fomsgaard
EPI_ISL_416425	State Key Laboratory for Diagnosis and Treatment of Infectious Diseases, National Clinical Research Center for Infectious Diseases, First Affiliated Hospital, Zhejiang University School of Medicine, Hangzhou, China 310003	State Key Laboratory for Diagnosis and Treatment of Infectious Diseases, National Clinical Research Center for Infectious Diseases, First Affiliated Hospital, Zhejiang University School of Medicine, Hangzhou, China 310003	Hangping Yao, Nanping Wu, Chao Jiang, Xiangyun Lu, Linfang Cheng, Fumin Liu, Zhigang Wu, Haibo Wu, Changzhong Jin, Min Zheng, Lanjuan Li
EPI_ISL_416426	Virological Research Group, Szentágothai Research Centre, University of Pécs	Bioinformatics Research Group, Szentágothai Research Centre, University of Pécs	Péter Urbán, Endre Gábor Tóth, Gábor Kemenesi, Róbert Herczeg, Attila Gyenesei, Ferenc Jakab
EPI_ISL_416458	Virology laboratory Ministry of Health Kuwait sequenced at Dasman Diabetes Institute	Dasman Diabetes Institute	Fahd Al-Mulla, Sumi John, Sara Alqabandi, Rasheeba iqbal, Motasem Melhem, Ebaa alOzairi, Qais Al-Duwairi
EPI_ISL_416469, EPI_ISL_416470, EPI_ISL_416471, EPI_ISL_416472	KU Leuven, Clinical and Epidemiological Virology	KU Leuven, Clinical and Epidemiological Virology	Bert Vanmechelen, Tony Wawina, Joan Marti-Carreras, Piet Maes
EPI_ISL_416473, EPI_ISL_416474	State Key Laboratory for Diagnosis and Treatment of Infectious Diseases, National Clinical Research Center for Infectious Diseases, First Affiliated Hospital, Zhejiang University School of Medicine, Hangzhou, China 310003	State Key Laboratory for Diagnosis and Treatment of Infectious Diseases, National Clinical Research Center for Infectious Diseases, First Affiliated Hospital, Zhejiang University School of Medicine, Hangzhou, China 310003	Hangping Yao, Nanping Wu, Chao Jiang, Xiangyun Lu, Linfang Cheng, Fumin Liu, Zhigang Wu, Haibo Wu, Changzhong Jin, Min Zheng, Lanjuan Li
EPI_ISL_416475	KU Leuven, Clinical and Epidemiological Virology	KU Leuven, Clinical and Epidemiological Virology	Bert Vanmechelen, Tony Wawina, Joan Marti-Carreras, Piet Maes
EPI_ISL_416483	Servicio de Microbiología. Consorcio Hospital General Universitario de Valencia	Sequencing and Bioinformatics Service and Molecular Epidemiology Research Group. FISABIO-Public Health	Maria Alma Bracho, Maria Dolores Ocete, Concepcion Gimeno, Giuseppe D'Auria, Griselda De Marco, Neris Garcia-Gonzalez, Fernando Gonzalez-Candelas
EPI_ISL_416485	Servicio de Microbiologia. Consorcio Hospital General Universitario de Valencia	Sequencing and Bioinformatics Service and Molecular Epidemiology Research Group. FISABIO-Public Health	Griselda De Marco, Neris Garcia-Gonzalez, Maria Alma Bracho, Maria Dolores Ocete, Concepcion Gimeno, Giuseppe D'Auria, Fernando Gonzalez-Candelas
EPI_ISL_416486	Servicio de Microbiología. Consorcio Hospital General Universitario de Valencia	Sequencing and Bioinformatics Service and Molecular Epidemiology Research Group. FISABIO-Public Health	Neris Garcia-Gonzalez, Maria Alma Bracho, Maria Dolores Ocete, Concepcion Gimeno, Giuseppe D'Auria, Griselda De Marco, Fernando Gonzalez-Candelas
EPI_ISL_416487	Servicio de Microbiología. Consorcio Hospital General Universitario de Valencia	Sequencing and Bioinformatics Service and Molecular Epidemiology Research Group. FISABIO-Public Health	Giuseppe D'Auria, Griselda De Marco, Neris Garcia-Gonzalez, Maria Alma Bracho, Maria Dolores Ocete, Concepcion Gimeno, Fernando Gonzalez-Candelas
EPI_ISL_416488	ViroGenetics - BSL3 Laboratory of Virology; Human Genome Variation Research Group & Genomics Centre MCB; Bioinformatics Research Group Department of Virology	ViroGenetics - BSL3 Laboratory of Virology; Human Genome Variation Research Group & Genomics Centre MCB; Bioinformatics Research Group Department of Virology	Aleksandra Milewska, Ewelina Popiech, Agata Jarosz, Adrianna Klajmon, Kamila Marszaek, Katarzyna Pancer, Magdalena Rzeczkowska, Tomasz Wokowicz, Katarzyna Zacharczuk, Agnieszka Koakowska-Kulesza, Natalia Wolaniuk, Ewelina Hallman-Szeliska, Pawe P abaj, Wojciech Branicki, Krzysztof Pyr
EPI_ISL_416493	CH Jean de Navarre Laboratoire de Biologie	National Reference Center for Viruses of Respiratory Infections, Institut Pasteur, Paris	Mélnie Albert, Marion Barbet, Sylvie Behillil, Méline Bizard, Angela Brisebarre, Flora Donati, Etienne Simon-Lorière, Vincent Enouf, Maud Vanpeene, Sylvie van der Werf
EPI_ISL_416494	Centre Hositalier Universitaire de Rouen Laboratoire de Virologie	National Reference Center for Viruses of Respiratory Infections, Institut Pasteur, Paris	Mélnie Albert, Marion Barbet, Sylvie Behillil, Méline Bizard, Angela Brisebarre, Flora Donati, Etienne Simon-Lorière, Vincent Enouf, Maud Vanpeene, Sylvie van der Werf, Jean-Christophe Plantier
EPI_ISL_416495, EPI_ISL_416496, EPI_ISL_416497	Centre Hospitalier Compiègne Laboratoire de Biologie	National Reference Center for Viruses of Respiratory Infections, Institut Pasteur, Paris	Mélnie Albert, Marion Barbet, Sylvie Behillil, Méline Bizard, Angela Brisebarre, Flora Donati, Etienne Simon-Lorière, Vincent Enouf, Maud Vanpeene, Sylvie van der Werf, Raulin Olivia
EPI_ISL_416498	Institut Médico légal- Hop R. Poincaré	National Reference Center for Viruses of Respiratory Infections, Institut Pasteur, Paris	Mélnie Albert, Marion Barbet, Sylvie Behillil, Méline Bizard, Angela Brisebarre, Flora Donati, Etienne Simon-Lorière, Vincent Enouf, Maud Vanpeene, Sylvie van der Werf
EPI_ISL_416499, EPI_ISL_416500	LABM GH nord Essonne	National Reference Center for Viruses of Respiratory Infections, Institut Pasteur, Paris	Mélnie Albert, Marion Barbet, Sylvie Behillil, Méline Bizard, Angela Brisebarre, Flora Donati, Etienne Simon-Lorière, Vincent Enouf, Maud Vanpeene, Sylvie van der Werf
EPI_ISL_416501	Hopital franco britannique - Service des Urgences	National Reference Center for Viruses of Respiratory Infections, Institut Pasteur, Paris	Mélnie Albert, Marion Barbet, Sylvie Behillil, Méline Bizard, Angela Brisebarre, Flora Donati, Etienne Simon-Lorière, Vincent Enouf, Maud Vanpeene, Sylvie van der Werf
EPI_ISL_416502, EPI_ISL_416503, EPI_ISL_416504, EPI_ISL_416505, EPI_ISL_416506, EPI_ISL_416507, EPI_ISL_416508, EPI_ISL_416509, EPI_ISL_416510, EPI_ISL_416511, EPI_ISL_416512, EPI_ISL_416513			
see above	CHRU Pontchaillou - Laboratoire de Virologie	National Reference Center for Viruses of Respiratory Infections, Institut Pasteur, Paris	Mélnie Albert, Marion Barbet, Sylvie Behillil, Méline Bizard, Angela Brisebarre, Flora Donati, Etienne Simon-Lorière, Vincent Enouf, Maud Vanpeene, Sylvie van der Werf, Gisèle Lagathu
EPI_ISL_416565, EPI_ISL_416566, EPI_ISL_416567, EPI_ISL_416568, EPI_ISL_416569, EPI_ISL_416570, EPI_ISL_416571, EPI_ISL_416572, EPI_ISL_416573, EPI_ISL_416574, EPI_ISL_416575, EPI_ISL_416576, EPI_ISL_416577, EPI_ISL_416578, EPI_ISL_416579, EPI_ISL_416580, EPI_ISL_416581, EPI_ISL_416582, EPI_ISL_416583, EPI_ISL_416585, EPI_ISL_416586, EPI_ISL_416587, EPI_ISL_416588, EPI_ISL_416589, EPI_ISL_416590, EPI_ISL_416591, EPI_ISL_416592, EPI_ISL_416593, EPI_ISL_416594, EPI_ISL_416595, EPI_ISL_416596, EPI_ISL_416597, EPI_ISL_416598, EPI_ISL_416599, EPI_ISL_416600, EPI_ISL_416601,			

EPI_ISL_416602, EPI_ISL_416603, EPI_ISL_416604, EPI_ISL_416605, EPI_ISL_416606, EPI_ISL_416607, EPI_ISL_416608, EPI_ISL_416609, EPI_ISL_416610, EPI_ISL_416611, EPI_ISL_416612, EPI_ISL_416613, EPI_ISL_416614, EPI_ISL_416615, EPI_ISL_416616, EPI_ISL_416617, EPI_ISL_416618, EPI_ISL_416619, EPI_ISL_416620, EPI_ISL_416621, EPI_ISL_416622, EPI_ISL_416623, EPI_ISL_416624, EPI_ISL_416625, EPI_ISL_416626, EPI_ISL_416627, EPI_ISL_416628, EPI_ISL_416629, EPI_ISL_416630, EPI_ISL_416631, EPI_ISL_416632, EPI_ISL_416633, EPI_ISL_416634			
see above	Japanese Quarantine Stations	Pathogen Genomics Center, National Institute of Infectious Diseases	Tsuyoshi Sekizuka, Kentaro Itokawa, Rina Tanaka, Masanori Hashino, Tsutomu Kageyama, Shinji Saito, Ikuyo Takayama, Hideki Hasegawa, Takuri Takahashi, Hajime Kamiya, Takuya Yamagishi, Motoi Suzuki, Takaji Wakita, Makoto Kuroda
EPI_ISL_416744	Virological Research Group, Szentágothai Research Centre	Bioinformatics Research Group, Szentágothai Research Centre	Péter Urbán, Endre Gábor Tóth, Gábor Kemenesi, Róbert Herczeg, Attila Gyenesei, Ferenc Jakab
EPI_ISL_416829	National Public Health Laboratory	Malaysia Genome Institute	Mohd Noor Mat Isa, Irni Suhayu Sapian, Yusuf Muhammad Noor, Nurhezreen Md Iqbal, Mohd Faizal Abu Bakar, Enizza Kasim, Shamsidar Sopie, Siti Noraini Othman, Azrin Ahmad, Nor Azfa Johari, Norazimah Tajudin, Noorliza Mohamad Noordin, W Afiza W Mohd Arifin, Rehan Shuhada Abu Bakar, Yu Kie Chem, Selvanesan Sengol, Hani Mat Hussin, Shahrul Hisham Zainal Ariffin
EPI_ISL_416866, EPI_ISL_416884	National Public Health Laboratory	Malaysia Genome Institute	Mohd Noor Mat Isa, Irni Suhayu Sapian, Yusuf Muhammad Noor, Nurhezreen Md Iqbal, Mohd Faizal Abu Bakar, Enizza Kasim, Shamsidar Sopie, Siti Noraini Othman, Azrin Ahmad, Nor Azfa Johari, Norazimah Tajudin, Noorliza Mohamad Noordin, W Afiza W Mohd Arifin, Rehan Shuhada Abu Bakar, Yu Kie Chem, Selvanesan Sengol, Hani Mat Hussin, Shahrul Hisham Zainal Ariffin
EPI_ISL_416885, EPI_ISL_416886, EPI_ISL_416907	National Public Health Laboratory	Malaysia Genome Institute	Mohd Noor Mat Isa, Irni Suhayu Sapian, Yusuf Muhammad Noor, Nurhezreen Md Iqbal, Mohd Faizal Abu Bakar, Enizza Kasim, Shamsidar Sopie, Siti Noraini Othman, Azrin Ahmad, Nor Azfa Johari, Norazimah Tajudin, Noorliza Mohamad Noordin, W Afiza W Mohd Arifin, Rehan Shuhada Abu Bakar, Yu Kie Chem, Selvanesan Sengol, Hani Mat Hussin, Shahrul Hisham Zainal Ariffin
EPI_ISL_416994	COMPLEJO ASISTENCIAL UNIVERSITARIO DE BURGOS	Instituto de Salud Carlos III	Iglesias-Caballero, M. Molinero Calamita, M. González-Esguevillas, M. Camarero S. Pozo F. Casas I. Jiménez P. Jiménez M. Zaballos A. Monzón, S. Varona, S. Juliá M. Cuesta I. Megias Lobón, G. Hospital: ------
EPI_ISL_417007	HOSPITAL SANTA MARIA NAI	Instituto de Salud Carlos III	Iglesias-Caballero, M. Molinero Calamita, M. González-Esguevillas, M. Camarero S. Pozo F. Casas I. Jiménez, P. Jiménez, M. Zaballos, A. Monzón, S. Varona, S. Juliá, M. Cuesta, I. García Costa, J.
EPI_ISL_417010	FUNDACION JIMENEZ DIAZ	Instituto de Salud Carlos III	Iglesias-Caballero, M. Molinero Calamita, M. González-Esguevillas, M. Camarero, S. Pozo, F. Casas, I. Jiménez, P. Jiménez, M. Zaballos, A. Monzón, S. Varona, S. Juliá, M. Cuesta, I. Fernández Roblas, R.
EPI_ISL_417015, EPI_ISL_417016, EPI_ISL_417018, EPI_ISL_417019	Department of Clinical Microbiology	GIGA Medical Genomics	Durkin Keith, Artesi Maria, Bontems Sébastien, Boreux Raphaël, Meex Cécile, Melin Pierrette, Hayette Marie-Pierre, Bours Vincent.
EPI_ISL_417030	Centre for Infectious Diseases and Microbiology Laboratory Services	NSW Health Pathology - Institute of Clinical Pathology and Medical Research; Westmead Hospital; University of Sydney	Eden J-S, Rockett R, Carter I, Rahman H, Holmes EC, O'Sullivan MV, Sintchenko V, Chen SC, Maddocks S, Kok J and Dwyer DE for the 2019-nCoV Study Group*
EPI_ISL_417034	Laboratorio de Ecologia de Doencas Transmissiveis na Amazonia, Instituto Leonidas e Maria Deane - Fiocruz Amazonia	Laboratorio de Ecologia de Doencas Transmissiveis na Amazonia, Instituto Leonidas e Maria Deane - Fiocruz Amazonia	Valdinete Nascimento, André Corado, Fernanda Nascimento, Ágatha Costa, Debora Duarte, Luciana Gonçalves, Michele Jesus, Sérgio Luz, Felipe Naveca
EPI_ISL_417176, EPI_ISL_417177, EPI_ISL_417179	Department of Pathology, Princess Margaret Hospital	Department of Health Technology and Informatics, Faculty of Health and Social Science, The Hong Kong Polytechnic University	Kenneth Siu-Sing LEUNG, Timothy Ting-Leung NG, Alan Ka-Lun WU, Miranda Chong-Yee YAU, Hiu-Yin LAO, Ming-Pan CHOI, Kingsley King-Gee TAM, Lam-Kwong LEE, Barry Kin-Chung WONG, Alex Yat-Man HO, Kam-Tong Yip, Kwok-Cheung LUNG, Raymond Wai-To LIU, Eugene Yuk-Keung TSO, Wai-Shing LEUNG, Man-Chun CHAN, Yuk-Yung NG, Kit-Man SIN, Kitty Sau-Chun FUNG, Sandy Ka-Yee CHAU, Wing-Kin TO, Tak-Lun Que, David Ho-Keung SHUM, Shea Ping YIP, Wing Cheong YAM, Gilman Kit-Hang SIU
EPI_ISL_417181, EPI_ISL_417182	Department of Pathology, United Christian Hospital	Department of Health Technology and Informatics, Faculty of Health and Social Science, The Hong Kong Polytechnic University	Kenneth Siu-Sing LEUNG, Timothy Ting-Leung NG, Alan Ka-Lun WU, Miranda Chong-Yee YAU, Hiu-Yin LAO, Ming-Pan CHOI, Kingsley King-Gee TAM, Lam-Kwong LEE, Barry Kin-Chung WONG, Alex Yat-Man HO, Kam-Tong Yip, Kwok-Cheung LUNG, Raymond Wai-To LIU, Eugene Yuk-Keung TSO, Wai-Shing LEUNG, Man-Chun CHAN, Yuk-Yung NG, Kit-Man SIN, Kitty Sau-Chun FUNG, Sandy Ka-Yee CHAU, Wing-Kin TO, Tak-Lun Que, David Ho-Keung SHUM, Shea Ping YIP, Wing Cheong YAM, Gilman Kit-Hang SIU
EPI_ISL_417183, EPI_ISL_417184	Department of Clinical Pathology, Pamela Youde Nethersole Eastern Hospital	Department of Health Technology and Informatics, Faculty of Health and Social Science, The Hong Kong Polytechnic University	Kenneth Siu-Sing LEUNG, Timothy Ting-Leung NG, Alan Ka-Lun WU, Miranda Chong-Yee YAU, Hiu-Yin LAO, Ming-Pan CHOI, Kingsley King-Gee TAM, Lam-Kwong LEE, Barry Kin-Chung WONG, Alex Yat-Man HO, Kam-Tong Yip, Kwok-Cheung LUNG, Raymond Wai-To LIU, Eugene Yuk-Keung TSO, Wai-Shing LEUNG, Man-Chun CHAN, Yuk-Yung NG, Kit-Man SIN, Kitty Sau-Chun FUNG, Sandy Ka-Yee CHAU, Wing-Kin TO, Tak-Lun Que, David Ho-Keung SHUM, Shea Ping YIP, Wing Cheong YAM, Gilman Kit-Hang SIU
EPI_ISL_417185	Department of Pathology, United Christian Hospital	Department of Health Technology and Informatics, Faculty of Health and Social Science, The Hong Kong Polytechnic University	Kenneth Siu-Sing LEUNG, Timothy Ting-Leung NG, Alan Ka-Lun WU, Miranda Chong-Yee YAU, Hiu-Yin LAO, Ming-Pan CHOI, Kingsley King-Gee TAM, Lam-Kwong LEE, Barry Kin-Chung WONG, Alex Yat-Man HO, Kam-Tong Yip, Kwok-Cheung LUNG, Raymond Wai-To LIU, Eugene Yuk-Keung TSO, Wai-Shing LEUNG, Man-Chun CHAN, Yuk-Yung NG, Kit-Man SIN, Kitty Sau-Chun FUNG, Sandy Ka-Yee CHAU, Wing-Kin TO, Tak-Lun Que, David Ho-Keung SHUM, Shea Ping YIP, Wing Cheong YAM, Gilman Kit-Hang SIU
EPI_ISL_417186	National Institute for Communicable Diseases of the National Health Laboratory Service	National Institute for Communicable Diseases of the National Health Laboratory Service	Allam M, Kwenda S, van Heusden P, Khumalo Z, Mohale T, Subramoney K, von Gottberg, A, Ismail A, Bhiman JN
EPI_ISL_417187, EPI_ISL_417188, EPI_ISL_417190, EPI_ISL_417193, EPI_ISL_417195	Department of Clinical Pathology, Pamela Youde Nethersole Eastern Hospital	Department of Health Technology and Informatics, Faculty of Health and Social Science, The Hong Kong Polytechnic University	Kenneth Siu-Sing LEUNG, Timothy Ting-Leung NG, Alan Ka-Lun WU, Miranda Chong-Yee YAU, Hiu-Yin LAO, Ming-Pan CHOI, Kingsley King-Gee TAM, Lam-Kwong LEE, Barry Kin-Chung WONG, Alex Yat-Man HO, Kam-Tong Yip, Kwok-Cheung LUNG, Raymond Wai-To LIU, Eugene Yuk-Keung TSO, Wai-Shing LEUNG, Man-Chun CHAN, Yuk-Yung NG, Kit-Man SIN, Kitty Sau-Chun FUNG, Sandy Ka-Yee CHAU, Wing-Kin TO, Tak-Lun Que, David Ho-Keung SHUM, Shea Ping YIP, Wing Cheong YAM, Gilman Kit-Hang SIU
EPI_ISL_417333, EPI_ISL_417334, EPI_ISL_417335, EPI_ISL_417336, EPI_ISL_417337, EPI_ISL_417339	Institut des Agents Infectieux (IAI), Hospices Civils de Lyon	CNR Virus des Infections Respiratoires - France SUD	Antonin Bal, Gregory Destras, Gwendolyne Burfin, Solenne Brun, Carine Moustaud, Raphaelle Lamy, Alexandre Gaymard, Maude Bouscambert-Duchamp, Florence Morfin-Sherpa, Martine Valette, Laurence Josset, Bruno Lina
EPI_ISL_417413	Ministry of Health Turkey	Ministry of Health Turkey	Fatma Bayrakdar,Aye Baak Alta,Yasemin Cogun,Gülay Korukluolu,Selçuk Klç
EPI_ISL_417443	State Key Laboratory for Emerging Infectious Diseases Department of Microbiology Li Ka Shing Faculty of Medicine The University of Hong Kong	State Key Laboratory for Emerging Infectious Diseases Department of Microbiology Li Ka Shing Faculty of Medicine The University of Hong Kong	Pui Wang, Siu-Ying Lau, Shaofeng Deng, Bobo Wing-Yee Mok, Wenjun Song, Kwok-Yung Yuen, Honglin Chen
EPI_ISL_417482	Institute of Microbiology, Universidad San Francisco de Quito	Institute of Microbiology, Universidad San Francisco de Quito	Sully Márquez, Belén Prado-Vivar, Juan José Guadalupe, Bernardo Gutiérrez, Manuel Jibaja, Milton Tobar, Verónica Barragán, Patricio Rojas-Silva, Gabriel Trueba, Michelle Grunauer, Paúl Cárdenas
EPI_ISL_417491	Virology Laboratory, Department of Biomedical Sciences and Public Health, University Politecnica delle Marche	Virology and Legal Medicine Laboratories, Department of Biomedical Sciences and Public Health, University Politecnica delle Marche	Bagnarelli,P., Caucci,S., Di Sante,L., Menzo,S., Alessandrini,F., Onofri,V., Turchi,C., Tagliabracci,A.
EPI_ISL_417877, EPI_ISL_417878, EPI_ISL_417879, EPI_ISL_417880	Institute of Virology, Biomedical Research Center of the Slovak Academy of Sciences, Bratislava; Public Health Authority of the Slovak Republic, Bratislava	Institute of Virology, Biomedical Research Center of the Slovak Academy of Sciences, Bratislava; Comenius University Science Park, Bratislava	Monika Slávikova, Martina Liková, Sabina Fumaová Havlíková, Juraj Koi, Juraj Kopáek, Elena Tichá, Edita Staroová, Jaroslav Budiš, Werner Krampl, Miroslav Böhmer, Diana Rusáková, Tomáš Szemeš, Boris Klempa
EPI_ISL_417919	Department of Medical Microbiology, University Malaya Medical Centre	Department of Medical Microbiology, Faculty of Medicine, University of Malaya	Yoong Min CHONG, Sasheela PONNAMPALAVANAR, Sharifah Faridah SYED OMAR, Adeeba KAMARULZAMAN,Vijayan MUNUSAMY, Chee Kuan WONG, Fadhil Hadi JAMALUDDIN, Han Ming GAN, Cindy Shuan Ju TEH, I-Ching SAM, Yoke Fun CHAN, University Malaya Medical Centre COVID Team
EPI_ISL_417920	Department of Medical Microbiology, University Malaya Medical Centre	Department of Medical Microbiology, Faculty of Medicine, University of Malaya	Yoong Min CHONG, Sasheela PONNAMPALAVANAR, Sharifah Faridah SYED OMAR, Adeeba KAMARULZAMAN,Vijayan MUNUSAMY, Chee Kuan WONG, Fadhil Hadi JAMALUDDIN, Cindy Shuan Ju TEH, I-Ching SAM, Yoke Fun Chan, University Malaya Medical Centre COVID Team
EPI_ISL_417924	Secretaría de Salud Medellín	Instituto Nacional de Salud, Universidad Cooperativa de Colombia, Instituto Alexander von Humboldt, Imperial College-London, London School of Hygiene & Tropical Medicine	Marcela Mercado-Reyes, Katherine Laiton-Donato, Diego A. Álvarez-Díaz, Carlos Franco-Muñoz, Jose A. Usme-Ciro, Gloria Puerto, Nicolás D. Franco-Sierra, Mailyn A. Gonzalez, Zulma M. Cucunubá, Christian Julian VillabonaArenas, Liz Villabona-Arenas, Sussy Echeverria-Londoño, Astrid C. Flórez, Sergio Gomez Rangel, Luz Dary Rodriguez, Juliana Barbosa, Erika Ospitia, Diana Marcela Walteros-Acero, Martha Lucia Ospina Martinez
EPI_ISL_418183	Virological Research Group, Szentágothai Research Centre	Bioinformatics Research Group, Szentágothai Research	Péter Urbán, Endre Gábor Tóth, Gábor Kemenesi, Róbert Herczeg, Attila Gyenesei, Ferenc Jakab

		Centre	
EPI_ISL_418206, EPI_ISL_418207, EPI_ISL_418208, EPI_ISL_418209, EPI_ISL_418210, EPI_ISL_418211	Institut Pasteur Dakar	Institut Pasteur de Dakar	Ndongo Dia, Ousmane Faye, Amadou Alpha Sall
EPI_ISL_418212	Institut Pasteur Dakar	Institut Pasteur de Dakar	Ndongo Dia, Ousmane Faye, Amadou Alpha sall
EPI_ISL_418213, EPI_ISL_418214	Institut Pasteur Dakar	Institut Pasteur de Dakar	Ndongo Dia, Ousmane Faye, Amadou Alpha Sall
EPI_ISL_418215	Instirut Pasteur Dakar	Institut Pasteur de Dakar	Ndongo Dia, Ousmane Faye, Amadou Alpha Sall
EPI_ISL_418216, EPI_ISL_418217	Institut Pasteur Dakar	Institut Pasteur de Dakar	Ndongo Dia, Ousmane Faye, Amadou Alpha Sall
EPI_ISL_418218	Centre Hospitalier Compiègne Laboratoire de Biologie	National Reference Center for Viruses of Respiratory Infections, Institut Pasteur, Paris	Mélanie Albert, Marion Barbet, Sylvie Behillil, Méline Bizard, Angela Brisebarre, Flora Donati, Fabiana Gambaro, Etienne Simon-Lorière, Vincent Enouf, Maud Vanpeene, Sylvie van der Werf, Raulin Olivia
EPI_ISL_418219	CHU - Hôpital Cavale Blanche - Labo. de Virologie	National Reference Center for Viruses of Respiratory Infections, Institut Pasteur, Paris	Mélanie Albert, Marion Barbet, Sylvie Behillil, Méline Bizard, Angela Brisebarre, Flora Donati, Fabiana Gambaro, Etienne Simon-Lorière, Vincent Enouf, Maud Vanpeene, Sylvie van der Werf, Léa Pilorge
EPI_ISL_418220, EPI_ISL_418221	Centre Hospitalier Compiègne Laboratoire de Biologie	National Reference Center for Viruses of Respiratory Infections, Institut Pasteur, Paris	Mélanie Albert, Marion Barbet, Sylvie Behillil, Méline Bizard, Angela Brisebarre, Flora Donati, Fabiana Gambaro, Etienne Simon-Lorière, Vincent Enouf, Maud Vanpeene, Sylvie van der Werf, Raulin Olivia
EPI_ISL_418222	CHRU Bretonneau - Serv. Bacterio-Virol.	National Reference Center for Viruses of Respiratory Infections, Institut Pasteur, Paris	Mélanie Albert, Marion Barbet, Sylvie Behillil, Méline Bizard, Angela Brisebarre, Flora Donati, Fabiana Gambaro, Etienne Simon-Lorière, Vincent Enouf, Maud Vanpeene, Sylvie van der Werf, Julien Marlet
EPI_ISL_418223, EPI_ISL_418224, EPI_ISL_418225	Centre Hospitalier Compiègne Laboratoire de Biologie	National Reference Center for Viruses of Respiratory Infections, Institut Pasteur, Paris	Mélanie Albert, Marion Barbet, Sylvie Behillil, Méline Bizard, Angela Brisebarre, Flora Donati, Fabiana Gambaro, Etienne Simon-Lorière, Vincent Enouf, Maud Vanpeene, Sylvie van der Werf, Raulin Olivia
EPI_ISL_418226	EHPAD - Résidences les Cèdres	National Reference Center for Viruses of Respiratory Infections, Institut Pasteur, Paris	Mélanie Albert, Marion Barbet, Sylvie Behillil, Méline Bizard, Angela Brisebarre, Flora Donati, Etienne Simon-Lorière, Vincent Enouf, Maud Vanpeene, Sylvie van der Werf
EPI_ISL_418227, EPI_ISL_418228	Centre Hospitalier Compiègne Laboratoire de Biologie	National Reference Center for Viruses of Respiratory Infections, Institut Pasteur, Paris	Mélanie Albert, Marion Barbet, Sylvie Behillil, Méline Bizard, Angela Brisebarre, Flora Donati, Etienne Simon-Lorière, Vincent Enouf, Maud Vanpeene, Sylvie van der Werf, Raulin Olivia
EPI_ISL_418229	Hopital franco britannique - Laboratoire	National Reference Center for Viruses of Respiratory Infections, Institut Pasteur, Paris	Mélanie Albert, Marion Barbet, Sylvie Behillil, Méline Bizard, Angela Brisebarre, Flora Donati, Etienne Simon-Lorière, Vincent Enouf, Maud Vanpeene, Sylvie van der Werf, Marianne Asso Bonnet
EPI_ISL_418230	Clinique AVERAY LA BROUSTE, Med. Polyvalente	National Reference Center for Viruses of Respiratory Infections, Institut Pasteur, Paris	Mélanie Albert, Marion Barbet, Sylvie Behillil, Méline Bizard, Angela Brisebarre, Flora Donati, Etienne Simon-Lorière, Vincent Enouf, Maud Vanpeene, Sylvie van der Werf, Elsa Ngwem
EPI_ISL_418231	Centre Hospitalier Compiègne Laboratoire de Biologie	National Reference Center for Viruses of Respiratory Infections, Institut Pasteur, Paris	Mélanie Albert, Marion Barbet, Sylvie Behillil, Méline Bizard, Angela Brisebarre, Flora Donati, Etienne Simon-Lorière, Vincent Enouf, Maud Vanpeene, Sylvie van der Werf, Raulin Olivia
EPI_ISL_418232, EPI_ISL_418233	Service des Urgences	National Reference Center for Viruses of Respiratory Infections, Institut Pasteur, Paris	Mélanie Albert, Marion Barbet, Sylvie Behillil, Méline Bizard, Angela Brisebarre, Flora Donati, Etienne Simon-Lorière, Vincent Enouf, Maud Vanpeene, Sylvie van der Werf, Boubkeur
EPI_ISL_418234	LABM GH nord Essonne	National Reference Center for Viruses of Respiratory Infections, Institut Pasteur, Paris	Mélanie Albert, Marion Barbet, Sylvie Behillil, Méline Bizard, Angela Brisebarre, Flora Donati, Etienne Simon-Lorière, Vincent Enouf, Maud Vanpeene, Sylvie van der Werf, Christine Lambert
EPI_ISL_418236, EPI_ISL_418237, EPI_ISL_418238, EPI_ISL_418239	Centre Hospitalier Compiègne Laboratoire de Biologie	National Reference Center for Viruses of Respiratory Infections, Institut Pasteur, Paris	Mélanie Albert, Marion Barbet, Sylvie Behillil, Méline Bizard, Angela Brisebarre, Flora Donati, Etienne Simon-Lorière, Vincent Enouf, Maud Vanpeene, Sylvie van der Werf, Raulin Olivia
EPI_ISL_418240	LABM GH nord Essonne	National Reference Center for Viruses of Respiratory Infections, Institut Pasteur, Paris	Mélanie Albert, Marion Barbet, Sylvie Behillil, Méline Bizard, Angela Brisebarre, Flora Donati, Etienne Simon-Lorière, Vincent Enouf, Maud Vanpeene, Sylvie van der Werf, Christine Lambert
EPI_ISL_418241, EPI_ISL_418242	NIC Viral Respiratory Unit - Institut Pasteur of Algeria	National Reference Center for Viruses of Respiratory Infections, Institut Pasteur, Paris	Mélanie Albert, Marion Barbet, Sylvie Behillil, Méline Bizard, Angela Brisebarre, Flora Donati, Etienne Simon-Lorière, Vincent Enouf, Maud Vanpeene, Sylvie van der Werf, Fawzi Derrar
EPI_ISL_418243, EPI_ISL_418244	HOSPITAL UNIVERSITARIO VIRGEN DE LAS NIEVES	Instituto de Salud Carlos III	Iglesias-Caballero, M. Molinero Calamita, M. González-Esguevillas, M. Camarero, S. Pozo, F. Casas, I. Jiménez, P. Jiménez, M. Zaballos, A. Monzón, S. Varona, S. Juliá, M. Cuesta, I. Sanbonmatsu S.
EPI_ISL_418245, EPI_ISL_418246	Hospital General y Universitario de Guadalajara	Instituto de Salud Carlos III	Iglesias-Caballero, M. Molinero Calamita, M. González-Esguevillas, M. Camarero, S. Pozo, F. Casas, I. Jiménez, P. Jiménez, M. Zaballos, A. Monzón, S. Varona, S. Juliá, M. Cuesta, I. Gonzalez-Praetorius A.
EPI_ISL_418247	HOSPITAL GENERAL DE SEGOVIA	Instituto de Salud Carlos III	Iglesias-Caballero, M. Molinero Calamita, M. González-Esguevillas, M. Camarero, S. Pozo, F. Casas, I. Jiménez, P. Jiménez, M. Zaballos, A. Monzón, S. Varona, S. Juliá, M. Cuesta, I. Hernando-Real S.
EPI_ISL_418248, EPI_ISL_418249	COMPLEJO ASISTENCIAL UNIVERSITARIO DE BURGOS	Instituto de Salud Carlos III	Iglesias-Caballero, M. Molinero Calamita, M. González-Esguevillas, M. Camarero, S. Pozo, F. Casas, I. Jiménez, P. Jiménez, M. Zaballos, A. Monzón, S. Varona, S. Juliá, M. Cuesta, I. Megias-Lobon G.
EPI_ISL_418250	HOSPITAL CLINIC	Instituto de Salud Carlos III	Iglesias-Caballero, M. Molinero Calamita, M. González-Esguevillas, M. Camarero, S. Pozo, F. Casas, I. Jiménez, P. Jiménez, M. Zaballos, A. Monzón, S. Varona, S. Juliá, M. Cuesta, I. Marcos M.A
EPI_ISL_418251	HOSPITAL UNIVERSITARIO LA PAZ	Instituto de Salud Carlos III	Iglesias-Caballero, M. Molinero Calamita, M. González-Esguevillas, M. Camarero, S. Pozo, F. Casas, I. Jiménez, P. Jiménez, M. Zaballos, A. Monzón, S. Varona, S. Juliá, M. Cuesta, I. Romero P.
EPI_ISL_418252	FUNDACION JIMENEZ DIAZ	Instituto de Salud Carlos III	Iglesias-Caballero, M. Molinero Calamita, M. González-Esguevillas, M. Camarero, S. Pozo, F. Casas, I. Jiménez, P. Jiménez, M. Zaballos, A. Monzón, S. Varona, S. Juliá, M. Cuesta, I. Fernández Roblas, R.
EPI_ISL_418253	HOSPITAL TXAGORRITXU	Instituto de Salud Carlos III	Iglesias-Caballero, M. Molinero Calamita, M. González-Esguevillas, M. Camarero, S. Pozo, F. Casas, I. Jiménez, P. Jiménez, M. Zaballos, A. Monzón, S. Varona, S. Juliá, M. Cuesta, I. Gomez-Gonzalez C.
EPI_ISL_418256	Ospedale "San Liberatore" di Atri	Istituto Zooprofilattico Sperimentale dell'Abruzzo e Molise "G. Caporale"	Lorusso A, Marcacci M, Di Domenico M, Puglia I, Curini V, Ancora M, Di Pasquale A, Rinaldi A, Mangone I, Cammà C, Savini G.
EPI_ISL_418257	Ospedale Civile Giuseppe Mazzini, Teramo	Istituto Zooprofilattico Sperimentale dell'Abruzzo e Molise "G.Caporale"	Lorusso A, Marcacci M, Di Domenico M, Puglia I, Curini V, Ancora M, Di Pasquale A, Rinaldi A, Mangone I, Cammà C, Savini G.
EPI_ISL_418262	Instituto Nacional de Salud	Instituto Nacional de Salud Universidad Cooperativa de Colombia Instituto Alexander von Humboldt Imperial College-London London School of Hygiene & Tropical Medicine	Marcela Mercado-Reyes, Katherine Laiton-Donato, Diego A. Álvarez-Díaz, Carlos Franco-Muñoz, Jose A. Usme-Ciro, Gloria Puerto, Nicolas D. Franco-Sierra, Mailyn A.Gonzalez, Zulma M. Cucunubá, Christian Julian VillabonaArenas, Liz Villabona-Arenas, Sussy Echeverria, Astrid C. Flórez, Sergio Gomez Rangel, Luz Dary Rodriguez, Juliana Barbosa, Erika Ospitia, Diana Marcela Walteros-Acero, Nuno Rodrigues Faria, Martha Lucia Ospina Martinez
EPI_ISL_418268	Hospital Universitari Germans Trias i Pujol(HUGTiP)/Fundació Lluita contra la SIDA (FLSida)/IRTA-CReSA	IrsiCaixa AIDS Research Lab	Pilar Armengol, Marc Noguera-Julian, Jordi Rodón, Julia Vergara, Lidia Ruiz, Nuria Izquierdo, Jorge Carrillo, Roger Paredes, Albert Bensaid, Julia Blanco, Joaquim Segalés, Bonaventura Clotet
EPI_ISL_418623, EPI_ISL_418624, EPI_ISL_418633, EPI_ISL_418643, EPI_ISL_418644, EPI_ISL_418645, EPI_ISL_418646, EPI_ISL_418647, EPI_ISL_418648, EPI_ISL_418649, EPI_ISL_418650, EPI_ISL_418651, EPI_ISL_418652, EPI_ISL_418658, EPI_ISL_418659, EPI_ISL_418660, EPI_ISL_418662, EPI_ISL_418664, EPI_ISL_418665, EPI_ISL_418666			
see above	Department of Clinical Microbiology	GIGA Medical Genomics	Keith Durkin, Maria Artesi, Sébastien Bontems, Raphaël Boreux, Cécile Meex, Pierrette Melin, Marie-Pierre Hayette, Vincent Bours.
EPI_ISL_418800	KU Leuven, Clinical and Epidemiological Virology	KU Leuven, Clinical and Epidemiological Virology	Bert Vanmechelen, Joan Marti-Carreras, Tony Wawina, Piet Maes
EPI_ISL_418815	Department of Clinical Pathology, Pamela Youde Nethersole Eastern Hospital	Department of Health Technology and Informatics, Faculty of Health and Social Science, The Hong Kong Polytechnic	Kenneth Siu-Sing LEUNG, Timothy Ting-Leung NG, Alan Ka-Lun WU, Miranda Chong-Yee YAU, Hiu-Yin LAO, Ming-Pan CHOI, Kingsley King-Gee TAM, Lam-Kwong LEE, Barry Kin-Chung WONG, Alex Yat-Man HO, Kam-Tong YIP, Kwok-Cheung LUNG, Raymond Wai-To LIU, Eugene Yuk-Keung TSO,

		University	Wai-Shing LEUNG, Man-Chun CHAN, Yuk-Yung NG, Kit-Man SIN, Kitty Sau-Chun FUNG, Sandy Ka-Yee CHAU, Wing-Kin TO, Tak-Lun QUE, David Ho-Keung SHUM, Shea Ping YIP, Wing Cheong YAM, Gilman Kit-Hang SIU
EPI_ISL_418990, EPI_ISL_418991	State Key Laboratory for Diagnosis and Treatment of Infectious Diseases, National Clinical Research Center for Infectious Diseases, First Affiliated Hospital, Zhejiang University School of Medicine, Hangzhou, China 310003	State Key Laboratory for Diagnosis and Treatment of Infectious Diseases, National Clinical Research Center for Infectious Diseases, First Affiliated Hospital, Zhejiang University School of Medicine, Hangzhou, China 310003	Hangping Yao, Nanping Wu, Chao Jiang, Xiangyun Lu, Linfang Cheng, Fumin Liu, Zhigang Wu, Haibo Wu, Changzhong Jin, Min Zheng, Lanjuan Li
EPI_ISL_419213, EPI_ISL_419214, EPI_ISL_419215, EPI_ISL_419216	Department of Clinical Pathology, Pamela Youde Nethersole Eastern Hospital	Department of Health Technology and Informatics, Faculty of Health and Social Science, The Hong Kong Polytechnic University	Kenneth Siu-Sing LEUNG, Timothy Ting-Leung NG, Alan Ka-Lun WU, Miranda Chong-Yee YAU, Hiu-Yin LAO, Ming-Pan CHOI, Kingsley King-Gee TAM, Lam-Kwong LEE, Barry Kin-Chung WONG, Alex Yat-Man HO, Kam-Tong YIP, Kwok-Cheung LUNG, Raymond Wai-To LIU, Eugene Yuk-Keung TSO, Wai-Shing LEUNG, Man-Chun CHAN, Yuk-Yung NG, Kit-Man SIN, Kitty Sau-Chun FUNG, Sandy Ka-Yee CHAU, Wing-Kin TO, Tak-Lun QUE, David Ho-Keung SHUM, Shea Ping YIP, Wing Cheong YAM, Gilman Kit-Hang SIU
EPI_ISL_419217	Department of Pathology, Princess Margaret Hospital	Department of Health Technology and Informatics, Faculty of Health and Social Science, The Hong Kong Polytechnic University	Kenneth Siu-Sing LEUNG, Timothy Ting-Leung NG, Alan Ka-Lun WU, Miranda Chong-Yee YAU, Hiu-Yin LAO, Ming-Pan CHOI, Kingsley King-Gee TAM, Lam-Kwong LEE, Barry Kin-Chung WONG, Alex Yat-Man HO, Kam-Tong YIP, Kwok-Cheung LUNG, Raymond Wai-To LIU, Eugene Yuk-Keung TSO, Wai-Shing LEUNG, Man-Chun CHAN, Yuk-Yung NG, Kit-Man SIN, Kitty Sau-Chun FUNG, Sandy Ka-Yee CHAU, Wing-Kin TO, Tak-Lun QUE, David Ho-Keung SHUM, Shea Ping YIP, Wing Cheong YAM, Gilman Kit-Hang SIU
EPI_ISL_419218, EPI_ISL_419219	Department of Clinical Pathology, Pamela Youde Nethersole Eastern Hospital	Department of Health Technology and Informatics, Faculty of Health and Social Science, The Hong Kong Polytechnic University	Kenneth Siu-Sing LEUNG, Timothy Ting-Leung NG, Alan Ka-Lun WU, Miranda Chong-Yee YAU, Hiu-Yin LAO, Ming-Pan CHOI, Kingsley King-Gee TAM, Lam-Kwong LEE, Barry Kin-Chung WONG, Alex Yat-Man HO, Kam-Tong YIP, Kwok-Cheung LUNG, Raymond Wai-To LIU, Eugene Yuk-Keung TSO, Wai-Shing LEUNG, Man-Chun CHAN, Yuk-Yung NG, Kit-Man SIN, Kitty Sau-Chun FUNG, Sandy Ka-Yee CHAU, Wing-Kin TO, Tak-Lun QUE, David Ho-Keung SHUM, Shea Ping YIP, Wing Cheong YAM, Gilman Kit-Hang SIU
EPI_ISL_419221	Department of Pathology, United Christian Hospital	Department of Health Technology and Informatics, Faculty of Health and Social Science, The Hong Kong Polytechnic University	Kenneth Siu-Sing LEUNG, Timothy Ting-Leung NG, Alan Ka-Lun WU, Miranda Chong-Yee YAU, Hiu-Yin LAO, Ming-Pan CHOI, Kingsley King-Gee TAM, Lam-Kwong LEE, Barry Kin-Chung WONG, Alex Yat-Man HO, Kam-Tong YIP, Kwok-Cheung LUNG, Raymond Wai-To LIU, Eugene Yuk-Keung TSO, Wai-Shing LEUNG, Man-Chun CHAN, Yuk-Yung NG, Kit-Man SIN, Kitty Sau-Chun FUNG, Sandy Ka-Yee CHAU, Wing-Kin TO, Tak-Lun QUE, David Ho-Keung SHUM, Shea Ping YIP, Wing Cheong YAM, Gilman Kit-Hang SIU
EPI_ISL_419222, EPI_ISL_419223	Department of Pathology, Princess Margaret Hospital	Department of Health Technology and Informatics, Faculty of Health and Social Science, The Hong Kong Polytechnic University	Kenneth Siu-Sing LEUNG, Timothy Ting-Leung NG, Alan Ka-Lun WU, Miranda Chong-Yee YAU, Hiu-Yin LAO, Ming-Pan CHOI, Kingsley King-Gee TAM, Lam-Kwong LEE, Barry Kin-Chung WONG, Alex Yat-Man HO, Kam-Tong YIP, Kwok-Cheung LUNG, Raymond Wai-To LIU, Eugene Yuk-Keung TSO, Wai-Shing LEUNG, Man-Chun CHAN, Yuk-Yung NG, Kit-Man SIN, Kitty Sau-Chun FUNG, Sandy Ka-Yee CHAU, Wing-Kin TO, Tak-Lun QUE, David Ho-Keung SHUM, Shea Ping YIP, Wing Cheong YAM, Gilman Kit-Hang SIU
EPI_ISL_419224, EPI_ISL_419225, EPI_ISL_419226, EPI_ISL_419227, EPI_ISL_419228, EPI_ISL_419229	Department of Clinical Pathology, Pamela Youde Nethersole Eastern Hospital	Department of Health Technology and Informatics, Faculty of Health and Social Science, The Hong Kong Polytechnic University	Kenneth Siu-Sing LEUNG, Timothy Ting-Leung NG, Alan Ka-Lun WU, Miranda Chong-Yee YAU, Hiu-Yin LAO, Ming-Pan CHOI, Kingsley King-Gee TAM, Lam-Kwong LEE, Barry Kin-Chung WONG, Alex Yat-Man HO, Kam-Tong YIP, Kwok-Cheung LUNG, Raymond Wai-To LIU, Eugene Yuk-Keung TSO, Wai-Shing LEUNG, Man-Chun CHAN, Yuk-Yung NG, Kit-Man SIN, Kitty Sau-Chun FUNG, Sandy Ka-Yee CHAU, Wing-Kin TO, Tak-Lun QUE, David Ho-Keung SHUM, Shea Ping YIP, Wing Cheong YAM, Gilman Kit-Hang SIU
EPI_ISL_419230	Hospital Universitario Virgen de las Nieves	Instituto de Salud Carlos III	Iglesias-Caballero, M.; Molinero Calamita, M.; González-Esguevillas, M.; Camarero, S.; Pozo, F.; Casas, I.; Jiménez, P.; Jiménez, M.; Zaballos, A.; Monzón, S.; Varona, S.; Juliá, M.; Cuesta, I.; Sanbonmatsu, S.
EPI_ISL_419231	Department of Clinical Pathology, Tuen Mun Hospital, 23 Tsing Chung Koon Road, Tuen Mun, N.T.	Department of Health Technology and Informatics, Faculty of Health and Social Science, The Hong Kong Polytechnic University	Kenneth Siu-Sing LEUNG, Timothy Ting-Leung NG, Alan Ka-Lun WU, Miranda Chong-Yee YAU, Hiu-Yin LAO, Ming-Pan CHOI, Kingsley King-Gee TAM, Lam-Kwong LEE, Barry Kin-Chung WONG, Alex Yat-Man HO, Kam-Tong YIP, Kwok-Cheung LUNG, Raymond Wai-To LIU, Eugene Yuk-Keung TSO, Wai-Shing LEUNG, Man-Chun CHAN, Yuk-Yung NG, Kit-Man SIN, Kitty Sau-Chun FUNG, Sandy Ka-Yee CHAU, Wing-Kin TO, Tak-Lun QUE, David Ho-Keung SHUM, Shea Ping YIP, Wing Cheong YAM, Gilman Kit-Hang SIU
EPI_ISL_419232	Department of Clinical Pathology, Pamela Youde Nethersole Eastern Hospital	Department of Health Technology and Informatics, Faculty of Health and Social Science, The Hong Kong Polytechnic University	Kenneth Siu-Sing LEUNG, Timothy Ting-Leung NG, Alan Ka-Lun WU, Miranda Chong-Yee YAU, Hiu-Yin LAO, Ming-Pan CHOI, Kingsley King-Gee TAM, Lam-Kwong LEE, Barry Kin-Chung WONG, Alex Yat-Man HO, Kam-Tong YIP, Kwok-Cheung LUNG, Raymond Wai-To LIU, Eugene Yuk-Keung TSO, Wai-Shing LEUNG, Man-Chun CHAN, Yuk-Yung NG, Kit-Man SIN, Kitty Sau-Chun FUNG, Sandy Ka-Yee CHAU, Wing-Kin TO, Tak-Lun QUE, David Ho-Keung SHUM, Shea Ping YIP, Wing Cheong YAM, Gilman Kit-Hang SIU
EPI_ISL_419233	Hospital Universitario de Canarias	Instituto de Salud Carlos III	Iglesias-Caballero, M.; Molinero Calamita, M.; González-Esguevillas, M.; Camarero, S.; Pozo, F.; Casas, I.; Jiménez, P.; Jiménez, M.; Zaballos, A.; Monzón, S.; Varona, S.; Juliá, M.; Cuesta, I.; Castro, B.
EPI_ISL_419234	Hospital San Pedro	Instituto de Salud Carlos III	Iglesias-Caballero, M.; Molinero Calamita, M.; González-Esguevillas, M.; Camarero, S.; Pozo, F.; Casas, I.; Jiménez, P.; Jiménez, M.; Zaballos, A.; Monzón, S.; Varona, S.; Juliá, M.; Cuesta, I.; Alonso, C.
EPI_ISL_419235, EPI_ISL_419236, EPI_ISL_419237	Fundacion Jimenez Diaz	Instituto de Salud Carlos III	Iglesias-Caballero, M.; Molinero Calamita, M.; González-Esguevillas, M.; Camarero, S.; Pozo, F.; Casas, I.; Jiménez, P.; Jiménez, M.; Zaballos, A.; Monzón, S.; Varona, S.; Juliá, M.; Cuesta, I.; Fernández, R.
EPI_ISL_419238	HOSPITAL DE CRUCES.	Instituto de Salud Carlos III	Iglesias-Caballero, M. Molinero Calamita, M. González-Esguevillas, M. Camarero, S. Pozo, F. Casas, I. Jiménez, P. Jiménez, M. Zaballos, A. Monzón, S. Varona, S. Juliá, M. Cuesta, I. Aranzamendi, M.
EPI_ISL_419240	HOSPITAL TXAGORRITXU	Instituto de Salud Carlos III	Iglesias-Caballero, M. Molinero Calamita, M. González-Esguevillas, M. Camarero, S. Pozo, F. Casas, I. Jiménez, P. Jiménez, M. Zaballos, A. Monzón, S. Varona, S. Juliá, M. Cuesta, I. Gómez, C
EPI_ISL_419241	Department of Clinical Pathology, Pamela Youde Nethersole Eastern Hospital	Department of Health Technology and Informatics, Faculty of Health and Social Science, The Hong Kong Polytechnic University	Kenneth Siu-Sing LEUNG, Timothy Ting-Leung NG, Alan Ka-Lun WU, Miranda Chong-Yee YAU, Hiu-Yin LAO, Ming-Pan CHOI, Kingsley King-Gee TAM, Lam-Kwong LEE, Barry Kin-Chung WONG, Alex Yat-Man HO, Kam-Tong YIP, Kwok-Cheung LUNG, Raymond Wai-To LIU, Eugene Yuk-Keung TSO, Wai-Shing LEUNG, Man-Chun CHAN, Yuk-Yung NG, Kit-Man SIN, Kitty Sau-Chun FUNG, Sandy Ka-Yee CHAU, Wing-Kin TO, Tak-Lun QUE, David Ho-Keung SHUM, Shea Ping YIP, Wing Cheong YAM, Gilman Kit-Hang SIU
EPI_ISL_419242	Department of Clinical Pathology, Tuen Mun Hospital	Department of Health Technology and Informatics, Faculty of Health and Social Science, The Hong Kong Polytechnic University	Kenneth Siu-Sing LEUNG, Timothy Ting-Leung NG, Alan Ka-Lun WU, Miranda Chong-Yee YAU, Hiu-Yin LAO, Ming-Pan CHOI, Kingsley King-Gee TAM, Lam-Kwong LEE, Barry Kin-Chung WONG, Alex Yat-Man HO, Kam-Tong YIP, Kwok-Cheung LUNG, Raymond Wai-To LIU, Eugene Yuk-Keung TSO, Wai-Shing LEUNG, Man-Chun CHAN, Yuk-Yung NG, Kit-Man SIN, Kitty Sau-Chun FUNG, Sandy Ka-Yee CHAU, Wing-Kin TO, Tak-Lun QUE, David Ho-Keung SHUM, Shea Ping YIP, Wing Cheong YAM, Gilman Kit-Hang SIU
EPI_ISL_419243, EPI_ISL_419244, EPI_ISL_419245, EPI_ISL_419246, EPI_ISL_419247, EPI_ISL_419248, EPI_ISL_419249, EPI_ISL_419250, EPI_ISL_419251, EPI_ISL_419252	Department of Clinical Pathology, Pamela Youde Nethersole Eastern Hospital	Department of Health Technology and Informatics, Faculty of Health and Social Science, The Hong Kong Polytechnic University	Kenneth Siu-Sing LEUNG, Timothy Ting-Leung NG, Alan Ka-Lun WU, Miranda Chong-Yee YAU, Hiu-Yin LAO, Ming-Pan CHOI, Kingsley King-Gee TAM, Lam-Kwong LEE, Barry Kin-Chung WONG, Alex Yat-Man HO, Kam-Tong YIP, Kwok-Cheung LUNG, Raymond Wai-To LIU, Eugene Yuk-Keung TSO, Wai-Shing LEUNG, Man-Chun CHAN, Yuk-Yung NG, Kit-Man SIN, Kitty Sau-Chun FUNG, Sandy Ka-Yee CHAU, Wing-Kin TO, Tak-Lun QUE, David Ho-Keung SHUM, Shea Ping YIP, Wing Cheong YAM, Gilman Kit-Hang SIU
EPI_ISL_419253	Department of Pathology, United Christian Hospital	Department of Health Technology and Informatics, Faculty of Health and Social Science, The Hong Kong Polytechnic University	Kenneth Siu-Sing LEUNG, Timothy Ting-Leung NG, Alan Ka-Lun WU, Miranda Chong-Yee YAU, Hiu-Yin LAO, Ming-Pan CHOI, Kingsley King-Gee TAM, Lam-Kwong LEE, Barry Kin-Chung WONG, Alex Yat-Man HO, Kam-Tong YIP, Kwok-Cheung LUNG, Raymond Wai-To LIU, Eugene Yuk-Keung TSO, Wai-Shing LEUNG, Man-Chun CHAN, Yuk-Yung NG, Kit-Man SIN, Kitty Sau-Chun FUNG, Sandy Ka-Yee CHAU, Wing-Kin TO, Tak-Lun QUE, David Ho-Keung SHUM, Shea Ping YIP, Wing Cheong YAM, Gilman Kit-Hang SIU
EPI_ISL_419296	Kochi Prefectural Institute of Public Health	Pathogen Genomics Center, National Institute of Infectious Diseases	Tsuyoshi Sekizuka, Akihiko Tokaji, Kentaro Itokawa, Rina Tanaka, Masanori Hashino, Hajime Kamiya, Motoi Suzuki, Makoto Kuroda
EPI_ISL_419297, EPI_ISL_419298	Chiba Prefectural Institute of Public Health	Pathogen Genomics Center, National Institute of Infectious Diseases	Tsuyoshi Sekizuka, Masakatsu Taira, Yushi Hachisu, Kentaro Itokawa, Rina Tanaka, Masanori Hashino, Hajime Kamiya, Motoi Suzuki, Makoto Kuroda
EPI_ISL_419299, EPI_ISL_419300	Ishikawa Prefectural Institute of Public Health and Environmental Science	Pathogen Genomics Center, National Institute of Infectious Diseases	Tsuyoshi Sekizuka, Sanae Kuramoto, Eri Nariai, Kentaro Itokawa, Rina Tanaka, Masanori Hashino, Hajime Kamiya, Motoi Suzuki, Makoto Kuroda
EPI_ISL_419301, EPI_ISL_419302, EPI_ISL_419303, EPI_ISL_419304, EPI_ISL_419305, EPI_ISL_419306,	Saitama Prefectural Institute of Public Health	Pathogen Genomics Center, National Institute of Infectious Diseases	Tsuyoshi Sekizuka, Michiyo Shinohara, Tsuyoshi Kishimoto, Kentaro Itokawa, Rina Tanaka, Masanori Hashino, Hajime Kamiya, Motoi Suzuki, Makoto Kuroda

EPI_ISL_419307, EPI_ISL_419308			
EPI_ISL_419309, EPI_ISL_419310, EPI_ISL_419311	Chiba Prefectural Institute of Public Health	Pathogen Genomics Center, National Institute of Infectious Diseases	Tsuyoshi Sekizuka, Masakatsu Taira, Yushi Hachisu, Kentaro Itokawa, Rina Tanaka, Masanori Hashino, Hajime Kamiya, Motoi Suzuki, Makoto Kuroda
EPI_ISL_419662, EPI_ISL_419663, EPI_ISL_419664	Center for Virology, Medical University of Vienna	Bergthaler laboratory, CeMM Research Center for Molecular Medicine of the Austrian Academy of Sciences	Alexandra Popa, Benedikt Agerer, Henrique Colaco, Lukas Endler, Jakob-Wendelin Genger, Alexander Lercher, Mark Smyth, Thomas Penz, Michael Schuster, Judith Aberle, Stephan Aberle, Elisabeth Puchhammer-Stöckl, Christoph Bock, Andreas Bergthaler
EPI_ISL_419707	HOSPITAL CLINIC	Instituto de Salud Carlos III	Iglesias-Caballero, M. Molinero Calamita, M. González-Esguevillas, M. Camarero S. Pozo F. Casas I. Jiménez, P. Jiménez, M. Zaballos, A. Monzón, S. Varona, S. Juliá, M. Cuesta, I. Marcos, M.A
EPI_ISL_419709	HOSPITAL TXAGORRITXU	Instituto de Salud Carlos III	Iglesias-Caballero, M. Molinero Calamita, M. González-Esguevillas, M. Camarero S. Pozo F. Casas I. Jiménez, P. Jiménez, M. Zaballos, A. Monzón, S. Varona, S. Juliá, M. Cuesta, I. Gómez, C.
EPI_ISL_420037	NIC Viral Respiratory Unit - Institut Pasteur of Algeria	National Reference Center for Viruses of Respiratory Infections, Institut Pasteur, Paris	Mélanie Albert, Marion Barbet, Sylvie Behillil, Méline Bizard, Angela Brisebarre, Flora Donati, Etienne Simon-Lorière, Vincent Enouf, Maud Vanpeene, Sylvie van der Werf, Fawzi Derrar
EPI_ISL_420039, EPI_ISL_420040	L'Air du Temps	National Reference Center for Viruses of Respiratory Infections, Institut Pasteur, Paris	Mélanie Albert, Marion Barbet, Sylvie Behillil, Méline Bizard, Angela Brisebarre, Flora Donati, Etienne Simon-Lorière, Vincent Enouf, Maud Vanpeene, Sylvie van der Werf
EPI_ISL_420041	CH Compiègne Laboratoire de Biologie	National Reference Center for Viruses of Respiratory Infections, Institut Pasteur, Paris	Mélanie Albert, Marion Barbet, Sylvie Behillil, Méline Bizard, Angela Brisebarre, Flora Donati, Etienne Simon-Lorière, Vincent Enouf, Maud Vanpeene, Sylvie van der Werf, Raulin Olivia
EPI_ISL_420042	Service de Biologie clinique	National Reference Center for Viruses of Respiratory Infections, Institut Pasteur, Paris	Mélanie Albert, Marion Barbet, Sylvie Behillil, Méline Bizard, Angela Brisebarre, Flora Donati, Etienne Simon-Lorière, Vincent Enouf, Maud Vanpeene, Sylvie van der Werf
EPI_ISL_420044	CH Jean de Navarre Laboratoire de Biologie	National Reference Center for Viruses of Respiratory Infections, Institut Pasteur, Paris	Mélanie Albert, Marion Barbet, Sylvie Behillil, Méline Bizard, Angela Brisebarre, Flora Donati, Etienne Simon-Lorière, Vincent Enouf, Maud Vanpeene, Sylvie van der Werf
EPI_ISL_420046, EPI_ISL_420047	Résidence Villa Caroline	National Reference Center for Viruses of Respiratory Infections, Institut Pasteur, Paris	Mélanie Albert, Marion Barbet, Sylvie Behillil, Méline Bizard, Angela Brisebarre, Flora Donati, Etienne Simon-Lorière, Vincent Enouf, Maud Vanpeene, Sylvie van der Werf
EPI_ISL_420048	Service de Biologie Médicale - BP 125	National Reference Center for Viruses of Respiratory Infections, Institut Pasteur, Paris	Mélanie Albert, Marion Barbet, Sylvie Behillil, Méline Bizard, Angela Brisebarre, Flora Donati, Etienne Simon-Lorière, Vincent Enouf, Maud Vanpeene, Sylvie van der Werf, Christine Lambert
EPI_ISL_420049, EPI_ISL_420050	CH Compiègne Laboratoire de Biologie	National Reference Center for Viruses of Respiratory Infections, Institut Pasteur, Paris	Mélanie Albert, Marion Barbet, Sylvie Behillil, Méline Bizard, Angela Brisebarre, Flora Donati, Etienne Simon-Lorière, Vincent Enouf, Maud Vanpeene, Sylvie van der Werf, Raulin Olivia
EPI_ISL_420051	Résidence Eleusis	National Reference Center for Viruses of Respiratory Infections, Institut Pasteur, Paris	Mélanie Albert, Marion Barbet, Sylvie Behillil, Méline Bizard, Angela Brisebarre, Flora Donati, Etienne Simon-Lorière, Vincent Enouf, Maud Vanpeene, Sylvie van der Werf
EPI_ISL_420052	Résidence les Marines	National Reference Center for Viruses of Respiratory Infections, Institut Pasteur, Paris	Mélanie Albert, Marion Barbet, Sylvie Behillil, Méline Bizard, Angela Brisebarre, Flora Donati, Etienne Simon-Lorière, Vincent Enouf, Maud Vanpeene, Sylvie van der Werf
EPI_ISL_420053	CH Jean de Navarre Laboratoire de Biologie	National Reference Center for Viruses of Respiratory Infections, Institut Pasteur, Paris	Mélanie Albert, Marion Barbet, Sylvie Behillil, Méline Bizard, Angela Brisebarre, Flora Donati, Etienne Simon-Lorière, Vincent Enouf, Maud Vanpeene, Sylvie van der Werf
EPI_ISL_420054	Résidence de maintenon	National Reference Center for Viruses of Respiratory Infections, Institut Pasteur, Paris	Mélanie Albert, Marion Barbet, Sylvie Behillil, Méline Bizard, Angela Brisebarre, Flora Donati, Etienne Simon-Lorière, Vincent Enouf, Maud Vanpeene, Sylvie van der Werf
EPI_ISL_420056, EPI_ISL_420057	CH Compiègne Laboratoire de Biologie	National Reference Center for Viruses of Respiratory Infections, Institut Pasteur, Paris	Mélanie Albert, Marion Barbet, Sylvie Behillil, Méline Bizard, Angela Brisebarre, Flora Donati, Etienne Simon-Lorière, Vincent Enouf, Maud Vanpeene, Sylvie van der Werf, Raulin Olivia
EPI_ISL_420058, EPI_ISL_420059, EPI_ISL_420060, EPI_ISL_420062	Service de Biologie Médicale - BP 125	National Reference Center for Viruses of Respiratory Infections, Institut Pasteur, Paris	Mélanie Albert, Marion Barbet, Sylvie Behillil, Méline Bizard, Angela Brisebarre, Flora Donati, Etienne Simon-Lorière, Vincent Enouf, Maud Vanpeene, Sylvie van der Werf, Christine Lambert
EPI_ISL_420063	Labo BM - Site de Juvisy - Hopital Général	National Reference Center for Viruses of Respiratory Infections, Institut Pasteur, Paris	Mélanie Albert, Marion Barbet, Sylvie Behillil, Méline Bizard, Angela Brisebarre, Flora Donati, Etienne Simon-Lorière, Vincent Enouf, Maud Vanpeene, Sylvie van der Werf
EPI_ISL_420064	Service de Biologie Médicale - BP 125	National Reference Center for Viruses of Respiratory Infections, Institut Pasteur, Paris	Mélanie Albert, Marion Barbet, Sylvie Behillil, Méline Bizard, Angela Brisebarre, Flora Donati, Etienne Simon-Lorière, Vincent Enouf, Maud Vanpeene, Sylvie van der Werf, Christine Lambert
EPI_ISL_420069, EPI_ISL_420070, EPI_ISL_420071	Institut Pasteur Dakar	Institut Pasteur de Dakar	Ndongo Dia, Moussa Moise Diagne, Mamadou Diop, Ousmane Faye, Amadou Alpha Sall
EPI_ISL_420072, EPI_ISL_420073, EPI_ISL_420074	Institut Pasteur Dakar	Institut Pasteur de Dakar	Ndongo Dia, Moussa Moise Diagne, Mamadou Diop, Ousmane Faye , Amadou Alpha Sall
EPI_ISL_420075	Institut pasteur Dakar	Institut Pasteur de Dakar	Ndongo Dia, Moussa Moise Diagne, Mamadou Diop, Ousmane Faye , Amadou Alpha Sall
EPI_ISL_420076	Institut Pasteur Dakar	Institut Pasteur de Dakar	Ndongo Dia, Moussa Moise Diagne, Mamadou Diop, Ousmane Faye , Ndongo Dia
EPI_ISL_420077, EPI_ISL_420078, EPI_ISL_420079	Institut Pasteur Dakar	Institut Pasteur de Dakar	Ndongo Dia, Moussa Moise Diagne, Mamadou Diop, Ousmane Faye , Amadou Alpha Sall
EPI_ISL_420080	WHO National Influenza Centre Russian Federation	WHO National Influenza Centre Russian Federation	Andrey Komissarov, Artem Fadeev, Anna Ivanova, Daria Danilenko
EPI_ISL_420081	WHO National Influenza Centre Russian Federation	WHO National Influenza Centre Russian Federation	Andrey Komissarov, Artem Fadeev, Mariia Sergeeva, Anna Ivanova, Daria Danilenko
EPI_ISL_420293	Wildlife Conservation Society, Bronx Zoo	Diagnostic Virology Laboratory, United States Department of Agriculture, National Veterinary Services Laboratories	Patrick K. Mitchell, Renee R. Anderson, Brittany Chilson, Roopa Venugopalan, D. G. Diel, Laura B. Goodman, L. Wang, F. Yuan, Y. Fang, Mary Lea Killian, Kerrie Franzen, Nichole Hines Bergeson, Ivan Kuzmin, Melinda Jenkins-Moore, Tod P. Stuber
EPI_ISL_420455	Department of Clinical Pathology, Pamela Youde Nethersole Eastern Hospital	Department of Health Technology and Informatics, Faculty of Health and Social Science, The Hong Kong Polytechnic University	Kenneth Siu-Sing LEUNG, Timothy Ting-Leung NG, Alan Ka-Lun WU, Miranda Chong-Yee YAU, Hiu-Yin LAO, Ming-Pan CHOI, Kingsley King-Gee TAM, Lam-Kwong LEE, Barry Kin-Chung WONG, Alex Yat-Man HO, Kam-Tong YIP, Kwok-Cheung LUNG, Raymond Wai-To LIU, Eugene Yuk-Keung TSO, Wai-Shing LEUNG, Man-Chun CHAN, Yuk-Yung NG, Kit-Man SIN, Kitty Sau-Chun FUNG, Sandy Ka-Yee CHAU, Wing-Kin TO, Tak-Lun QUE, David Ho-Keung SHUM, Shea Ping YIP, Wing Cheong YAM, Gilman Kit-Hang SIU
EPI_ISL_421182, EPI_ISL_421183, EPI_ISL_421184, EPI_ISL_421185, EPI_ISL_421186, EPI_ISL_421187, EPI_ISL_421188, EPI_ISL_421189, EPI_ISL_421190, EPI_ISL_421191, EPI_ISL_421192, EPI_ISL_421193, EPI_ISL_421194, EPI_ISL_421195, EPI_ISL_421196, EPI_ISL_421211			
see above	Department of Clinical Microbiology	GIGA Medical Genomics	Keith Durkin, Maria Artesi, Sébastien Bontems, Raphaël Boreux, Cécile Meex, Pierrette Melin, Marie-Pierre Hayette, Vincent Bours.
EPI_ISL_421221, EPI_ISL_421222, EPI_ISL_421223, EPI_ISL_421224, EPI_ISL_421225, EPI_ISL_421226, EPI_ISL_421227, EPI_ISL_421228, EPI_ISL_421229, EPI_ISL_421230, EPI_ISL_421231, EPI_ISL_421232, EPI_ISL_421233, EPI_ISL_421234, EPI_ISL_421235, EPI_ISL_421236			
see above	Hangzhou Center for Diseases Control and Prevention	Hangzhou Center for Diseases Control and Prevention	Jun Li, Haoqiu Wang, Lingfeng Mao, Hua Yu, Xinfen Yu, Zhou Sun, Xin Qian, Shuchang Chen, Junfang Chen, Xuchu Wang
EPI_ISL_421275	Russian State Collection of Viruses	Pathogenic Microorganisms Variability Laboratory	Alexey Shchetinin, Maria Nikiforova, Nadezhda Kuznetsova, Ekaterina Aksenova, Marina Kunda, Natalia Ryzhova, Olga Voronina, Inna Dolzhikova, Daria Grousova, Andrey Botikov, Denis Logunov, Alexander Gintsburg, Vladimir Gushchin
EPI_ISL_421500	CH Compiègne Laboratoire de Biologie	National Reference Center for Viruses of Respiratory Infections, Institut Pasteur, Paris	Mélanie Albert, Marion Barbet, Sylvie Behillil, Méline Bizard, Angela Brisebarre, Flora Donati, Etienne Simon-Lorière, Vincent Enouf, Maud Vanpeene, Sylvie van der Werf, Raulin Olivia
EPI_ISL_421501	Service de Biologie Médicale - BP 125	National Reference Center for Viruses of Respiratory Infections, Institut Pasteur, Paris	Mélanie Albert, Marion Barbet, Sylvie Behillil, Méline Bizard, Angela Brisebarre, Flora Donati, Etienne Simon-Lorière, Vincent Enouf, Maud Vanpeene, Sylvie van der Werf, Christine Lambert
EPI_ISL_421502, EPI_ISL_421503	Parc des Dames	National Reference Center for Viruses of Respiratory Infections, Institut Pasteur, Paris	Mélanie Albert, Marion Barbet, Sylvie Behillil, Méline Bizard, Angela Brisebarre, Flora Donati, Etienne Simon-Lorière, Vincent Enouf, Maud Vanpeene, Sylvie van der Werf

EPI_ISL_421504, EPI_ISL_421505, EPI_ISL_421506	Service de Biologie Médicale - BP 125	National Reference Center for Viruses of Respiratory Infections, Institut Pasteur, Paris	Mélanie Albert, Marion Barbet, Sylvie Behillil, Méline Bizard, Angela Brisebarre, Flora Donati, Etienne Simon-Lorière, Vincent Enouf, Maud Vanpeene, Sylvie van der Werf, Christine Lambert
EPI_ISL_421507, EPI_ISL_421508	Le Château de Seine-Port	National Reference Center for Viruses of Respiratory Infections, Institut Pasteur, Paris	Mélanie Albert, Marion Barbet, Sylvie Behillil, Méline Bizard, Angela Brisebarre, Flora Donati, Etienne Simon-Lorière, Vincent Enouf, Maud Vanpeene, Sylvie van der Werf
EPI_ISL_421509, EPI_ISL_421510, EPI_ISL_421511	CH Compiègne Laboratoire de Biologie	National Reference Center for Viruses of Respiratory Infections, Institut Pasteur, Paris	Mélanie Albert, Marion Barbet, Sylvie Behillil, Méline Bizard, Angela Brisebarre, Flora Donati, Etienne Simon-Lorière, Vincent Enouf, Maud Vanpeene, Sylvie van der Werf, Raulin Olivia
EPI_ISL_421512	Service de Biologie Médicale - BP 125	National Reference Center for Viruses of Respiratory Infections, Institut Pasteur, Paris	Mélanie Albert, Marion Barbet, Sylvie Behillil, Méline Bizard, Angela Brisebarre, Flora Donati, Etienne Simon-Lorière, Vincent Enouf, Maud Vanpeene, Sylvie van der Werf, Christine Lambert
EPI_ISL_421513	Service de Biologie clinique	National Reference Center for Viruses of Respiratory Infections, Institut Pasteur, Paris	Mélanie Albert, Marion Barbet, Sylvie Behillil, Méline Bizard, Angela Brisebarre, Flora Donati, Etienne Simon-Lorière, Vincent Enouf, Maud Vanpeene, Sylvie van der Werf, Christine Lambert
EPI_ISL_421572	Molecular Diagnostic Services and FLowpath	KRISP, KZN Research Innovation and Sequencing Platform	Giandhari J, Pillay S, Ngcapu S, Samsunder N, Lessells R, Chimukangara B, Deforche K, Tegally H, Wilkinson E, de Oliveira T
EPI_ISL_421574	Molecular Diagnostic Services	KRISP, KZN Research Innovation and Sequencing Platform	Giandhari J, Pillay S, Ngcapu S, Samsunder N, Lessells R, Chimukangara B, Deforche K, Tegally H, Wilkinson E, de Oliveira T
EPI_ISL_421652	Dasman Diabetes Institute	Dasman Diabetes Institute	Fahd Al-Mulla, Rasheeba Iqbal, Sumi John, Ebaa Al-Ozairi, Qais Al-Duwairi
EPI_ISL_422407, EPI_ISL_422408, EPI_ISL_422409, EPI_ISL_422410, EPI_ISL_422411, EPI_ISL_422412, EPI_ISL_422413, EPI_ISL_422414, EPI_ISL_422415, EPI_ISL_422416, EPI_ISL_422417, EPI_ISL_422418, EPI_ISL_422419, EPI_ISL_422420, EPI_ISL_422421, EPI_ISL_422422			
see above	Department of Laboratory Medicine, National Taiwan University Hospital	Microbial Genomics Core Lab, National Taiwan University Centers of Genomic and Precision Medicine	Shiou-Hwei Yeh, You-Yu Lin, Ya-Yun Lai, Chiao-Ling Li, Shan-Chwen Chang, Pei-Jer Chen, Sui-Yuan Chang
EPI_ISL_422424	Jaber Al Ahmad Al Sabah Hospital	Dasman diabetes Institute	Fahd Al-Mulla, Rasheeba Iqbal, Sumi John, Ebaa Al-Ozairi, Qais Al-Duwairi
EPI_ISL_422427	JABER AL AHMAD AL SABAH HOSPITAL - KUWAIT CITY	Dasman Diabetes Institute	Fahd Al-Mulla, Rasheeba Iqbal, Sumi John, Ebaa Al-Ozairi, Qais Al-Duwairi
EPI_ISL_422563	Institute of Microbiology Universidad San Francisco de Quito	Institute of Microbiology Universidad San Francisco de Quito	Belen Prado-Vivar, Sully Marquez, Juan Jose Guadalupe, Bernardo Gutierrez, Francisco Mora, Juan Gaviria, Alejandra Ramones, Franklin Espinoza, Edison Ligña, Jorge Reyes, Patricio Rojas-Silva, Veronica Barragan, Gabriel Trueba, Michelle Grunauer, Paul Cardenas
EPI_ISL_422564	Institute of Microbiology Universidad San Francisco de Quito	Institute of Microbiology Universidad San Francisco de Quito	Juan Jose Guadalupe, Belen Prado-Vivar, Sully Marquez, Bernardo Gutierrez, Francisco Mora, Juan Gaviria, Alejandra Ramones, Franklin Espinoza, Edison Ligña, Jorge Reyes, Patricio Rojas-Silva, Veronica Barragan, Gabriel Trueba, Michelle Grunauer, Paul Cardenas
EPI_ISL_422565	Institute of Microbiology Universidad San Francisco de Quito	Institute of Microbiology Universidad San Francisco de Quito	Sully Marquez, Belen Prado-Vivar, Juan Jose Guadalupe, Bernardo Gutierrez, Francisco Mora, Juan Gaviria, Alejandra Ramones, Franklin Espinoza, Edison Ligña, Jorge Reyes, Patricio Rojas-Silva, Veronica Barragan, Gabriel Trueba, Michelle Grunauer, Paul Cardenas
EPI_ISL_422636	The National Institute of Public Health Center for Epidemiology and Microbiology	State Veterinary Institute Prague	Alexander Nagy, Helena Jirincova, Klara Labska, Ludmila Novakova, Olga Storkanova, Dusan Trnka, Jaromira Vecerova
EPI_ISL_424366	Vaccine Research, Development and Application Center, Erciyes University	Gen Era Diagnostics Inc.	Shaikh Terkis Islam Pavel, Hazel Yetiskin, Gunsu Aydin, Can Holyavkin, Muhammet Ali Uygut, Zehra B Dursun, lhami Celik, Alper Iseri, Aykut Ozdarendeli
EPI_ISL_424628, EPI_ISL_424629, EPI_ISL_424630, EPI_ISL_424631, EPI_ISL_424633, EPI_ISL_424635, EPI_ISL_424636, EPI_ISL_424637, EPI_ISL_424638, EPI_ISL_424639, EPI_ISL_424640, EPI_ISL_424641, EPI_ISL_424642, EPI_ISL_424643, EPI_ISL_424644, EPI_ISL_424647, EPI_ISL_424648, EPI_ISL_424649, EPI_ISL_424650, EPI_ISL_424652, EPI_ISL_424653, EPI_ISL_424654, EPI_ISL_424655, EPI_ISL_424656, EPI_ISL_424657, EPI_ISL_424658, EPI_ISL_424659, EPI_ISL_424660, EPI_ISL_424661, EPI_ISL_424663, EPI_ISL_424664			
see above	Department of Clinical Microbiology	GIGA Medical Genomics	Keith Durkin, Maria Artesi, Sébastien Bontems, Raphaël Boreux, Cécile Meex, Pierrette Melin, Marie-Pierre Hayette, Vincent Bours.
EPI_ISL_426364	Instituto Nacional de Ciencias Medicas y Nutricion Salvador Zubiran	Instituto Nacional de Ciencias Medicas y Nutricion	Guillermo M. Ruiz-Palacios, Pilar Ramos Cervantes, Violeta Ibarra Gonzalez, Fernando Ledesma Barrientos, Luis Alberto García Andrade, Alfredo Ponce de León Garduño, Irma López Martínez, Lucia Hernández Rivas, Gisela Barrera Badillo, Edgar Mendieta Condado, Fabiola Garcés Ayala, Adnan Araiza Rodríguez, José Ernesto Ramírez González, Celia Boukadida, Santiago Avila Ríos, Mario Mújica Sánchez, José Arturo Martínez Orozco, Eduardo Becerril Vargas, Joel Armando Vázquez Pérez, Victor Hugo Borja Aburto, Concepción Grajales Muñiz, Cesar Raúl González Bonilla, Carolina González Torres, Francisco Javier Gaytán Cervantes, José Esteban Muñoz Medina, Blanca Taboada, Alejandro Sánchez, Pavel Isa, Ricardo Grande, Gloria Vázquez, Francisco Pulido, Carlos F. Arias
EPI_ISL_426414	Sir M P Shah Government Medical College	Gujarat Biotechnology Research Centre	Ramesh Pandit, Tejas Shah, Ankit Hinsu, Pritesh Sabara, Apurvasinh Puvar, Janvi Raval, Monika Gandhi, Pinal Trivedi, Maharshi Pandya, Amit Kanani, Akanksha Verma, Nitin Savaliya, Raghawendra Kumar, Dinesh Kumar, Zubair Saiyed, Dipa Kinariwala, Disha Patel, Binita Aring, Geeta Vaghela, Sonia Barve, Bhavesh Modi, Kairavi Joshi, Nidhi Sood, Pranay Shah, R D Dixit, Snehal Bagatharia, Madhvi Joshi, Chaitanya Joshi
EPI_ISL_426415	Sir M P Shah Government Medical College, Jamnagar	Gujarat Biotechnology Research Centre, Gandhinagar	Ramesh Pandit, Tejas Shah, Ankit Hinsu, Pritesh Sabara, Apurvasinh Puvar, Janvi Raval, Monika Gandhi, Pinal Trivedi, Maharshi Pandya, Amit Kanani, Akanksha Verma, Nitin Savaliya, Raghvendra Kumar, Dinesh Kumar, Zuber Saiyed, Dipa Kinariwala, Disha Patel, Binita Aring, Geeta Vaghela, Sonia Barve, Bhavesh Modi, Kairavi Joshi, Nidhi Sood, Pranay Shah, R D Dixit, Snehal Bagatharia, Madhvi Joshi, Chaitanya Joshi
EPI_ISL_426580	Instituto Sabin	Laboratory of Virology	Fernando L Melo,Gustavo Barra, Ticiane H Santa-Rita, Pedro G Mesquita, Ikaro A Andrade, Tatsuya Nagata, Bergmann M Ribeiro
EPI_ISL_427307, EPI_ISL_427308, EPI_ISL_427309, EPI_ISL_427310, EPI_ISL_427311, EPI_ISL_427312, EPI_ISL_427314, EPI_ISL_427315, EPI_ISL_427319, EPI_ISL_427320, EPI_ISL_427321, EPI_ISL_427322, EPI_ISL_427323, EPI_ISL_427337, EPI_ISL_427338, EPI_ISL_427339			
see above	WHO National Influenza Centre Russian Federation	WHO National Influenza Centre Russian Federation	Andrey Komissarov, Artem Fadeev, Mariia Sergeeva, Anna Ivanova, Daria Danilenko
EPI_ISL_427340, EPI_ISL_427347, EPI_ISL_427348, EPI_ISL_427349, EPI_ISL_427350, EPI_ISL_427351, EPI_ISL_427352, EPI_ISL_427353, EPI_ISL_427354, EPI_ISL_427355, EPI_ISL_427356, EPI_ISL_427357, EPI_ISL_427358, EPI_ISL_427359, EPI_ISL_427360, EPI_ISL_427361, EPI_ISL_427362, EPI_ISL_427363, EPI_ISL_427364, EPI_ISL_427365, EPI_ISL_427366, EPI_ISL_427367, EPI_ISL_427368, EPI_ISL_427369, EPI_ISL_427370, EPI_ISL_427371, EPI_ISL_427372, EPI_ISL_427373, EPI_ISL_427374, EPI_ISL_427375, EPI_ISL_427376, EPI_ISL_427377, EPI_ISL_427378, EPI_ISL_427379, EPI_ISL_427380, EPI_ISL_427381, EPI_ISL_427382, EPI_ISL_427383, EPI_ISL_427384, EPI_ISL_427385, EPI_ISL_427386, EPI_ISL_427387, EPI_ISL_427388, EPI_ISL_427390			
see above	Department of Clinical Microbiology	GIGA Medical Genomics	Keith Durkin, Maria Artesi, Sébastien Bontems, Raphaël Boreux, Cécile Meex, Pierrette Melin, Marie-Pierre Hayette, Vincent Bours.
EPI_ISL_427391	Genomic Laboratory (GLAB) (Conjoint lab of Health Directorate of Istanbul and Istanbul Technical University)	Genomic Laboratory (GLAB), Istanbul Technical University	Ilker Karacan, Tugba Kizilboga Akgun, Bugra Agaoglu, Gizem Alkurt, Jale Yildiz, Betsi Köse, Elifnaz Çelik, Mehtap Aydn, Levent Doganay, Gizem Dinler Doganay
EPI_ISL_428209	Laboratory of Molecular Biology, Diagnostyka sp. z o.o.	Laboratory of Recombinant Vaccines	Lukasz Rabalski, Anna Piotrowska-Mietelska, Boguslaw Szewczyk, Krystyna Bienkowska-Szewczyk
EPI_ISL_428229	TSGH-CP molecular lab	TSGH-CP molecular lab	Cherng-Lih Perng, Ming-Jr Jian, Chih-Kai Chang, Jung-Chung Lin, Kuo-Ming Yeh, Chien-Wen Chen, Sheng-Kang Chiu, Hsing-Yi Chung, Shih-Hung Tsai, Kuo-Sheng Hung, Tien-Yao Chang, Feng-Yee Chang, Hung-Sheng Shang
EPI_ISL_428346	Genomic Laboratory (GLAB) (Conjoint lab of Health Directorate of Istanbul and Istanbul Technical University)	Genomic Laboratory (GLAB), Istanbul Technical University	Ilker Karacan, Tugba Kizilboga Akgun, Bugra Agaoglu, Gizem Alkurt, Jale Yildiz, Betsi Köse, Elifnaz Çelik, Arzu Irvem, Yasemin Kendir Demirkol, Ozlem Akgun Dogan, Mehtap Aydn, Levent Doganay, Gizem Dinler Doganay
EPI_ISL_428347, EPI_ISL_428349	Service de Biologie Médicale - BP 125	National Reference Center for Viruses of Respiratory Infections, Institut Pasteur, Paris	Mélanie Albert, Marion Barbet, Sylvie Behillil, Méline Bizard, Angela Brisebarre, Flora Donati, Etienne Simon-Lorière, Vincent Enouf, Maud Vanpeene, Sylvie van der Werf
EPI_ISL_428350	CH Jean de Navarre Laboratoire de Biologie	National Reference Center for Viruses of Respiratory Infections, Institut Pasteur, Paris	Mélanie Albert, Marion Barbet, Sylvie Behillil, Méline Bizard, Angela Brisebarre, Flora Donati, Etienne Simon-Lorière, Vincent Enouf, Maud Vanpeene, Sylvie van der Werf
EPI_ISL_428351, EPI_ISL_428352	GH Nord Essonne Service de Biologie clinique	National Reference Center for Viruses of Respiratory Infections, Institut Pasteur, Paris	Mélanie Albert, Marion Barbet, Sylvie Behillil, Méline Bizard, Angela Brisebarre, Flora Donati, Etienne Simon-Lorière, Vincent Enouf, Maud Vanpeene, Sylvie van der Werf
EPI_ISL_428353	CH Compiègne Laboratoire de Biologie	National Reference Center for Viruses of Respiratory Infections, Institut Pasteur, Paris	Mélanie Albert, Marion Barbet, Sylvie Behillil, Méline Bizard, Angela Brisebarre, Flora Donati, Etienne Simon-Lorière, Vincent Enouf, Maud Vanpeene, Sylvie van der Werf
EPI_ISL_428354	LABM GH nord Essonne de Longjumeau - BP 125	National Reference Center for Viruses of Respiratory Infections, Institut Pasteur, Paris	Mélanie Albert, Marion Barbet, Sylvie Behillil, Méline Bizard, Angela Brisebarre, Flora Donati, Etienne Simon-Lorière, Vincent Enouf, Maud Vanpeene, Sylvie van der Werf
EPI_ISL_428355, EPI_ISL_428356, EPI_ISL_428357	Institut Médico légal- Hop R. Poincaré	National Reference Center for Viruses of Respiratory Infections, Institut Pasteur, Paris	Mélanie Albert, Marion Barbet, Sylvie Behillil, Méline Bizard, Angela Brisebarre, Flora Donati, Etienne Simon-Lorière, Vincent Enouf, Maud Vanpeene, Sylvie van der Werf

EPI_ISL_428358	CH Jeanne de Navarre Laboratoire de Biologie	National Reference Center for Viruses of Respiratory Infections, Institut Pasteur, Paris	Mélanie Albert, Marion Barbet, Sylvie Behillil, Méline Bizard, Angela Brisebarre, Flora Donati, Etienne Simon-Lorière, Vincent Enouf, Maud Vanpeene, Sylvie van der Werf
EPI_ISL_428359, EPI_ISL_428360	CH Compiègne Laboratoire de Biologie	National Reference Center for Viruses of Respiratory Infections, Institut Pasteur, Paris	Mélanie Albert, Marion Barbet, Sylvie Behillil, Méline Bizard, Angela Brisebarre, Flora Donati, Etienne Simon-Lorière, Vincent Enouf, Maud Vanpeene, Sylvie van der Werf
EPI_ISL_428361, EPI_ISL_428362	LABM GH nord Essonne de Longjumeau - BP 125	National Reference Center for Viruses of Respiratory Infections, Institut Pasteur, Paris	Mélanie Albert, Marion Barbet, Sylvie Behillil, Méline Bizard, Angela Brisebarre, Flora Donati, Etienne Simon-Lorière, Vincent Enouf, Maud Vanpeene, Sylvie van der Werf
EPI_ISL_428363	GH Nord Essonne Service de Biologie clinique	National Reference Center for Viruses of Respiratory Infections, Institut Pasteur, Paris	Mélanie Albert, Marion Barbet, Sylvie Behillil, Méline Bizard, Angela Brisebarre, Flora Donati, Etienne Simon-Lorière, Vincent Enouf, Maud Vanpeene, Sylvie van der Werf
EPI_ISL_428365	LABM GH nord Essonne de Longjumeau - BP 125	National Reference Center for Viruses of Respiratory Infections, Institut Pasteur, Paris	Mélanie Albert, Marion Barbet, Sylvie Behillil, Méline Bizard, Angela Brisebarre, Flora Donati, Etienne Simon-Lorière, Vincent Enouf, Maud Vanpeene, Sylvie van der Werf
EPI_ISL_428366	CH Jeanne de Navarre Laboratoire de Biologie	National Reference Center for Viruses of Respiratory Infections, Institut Pasteur, Paris	Mélanie Albert, Marion Barbet, Sylvie Behillil, Méline Bizard, Angela Brisebarre, Flora Donati, Etienne Simon-Lorière, Vincent Enouf, Maud Vanpeene, Sylvie van der Werf
EPI_ISL_428368	Genomic Laboratory (GLAB) (Conjoint lab of Health Directorate of Istanbul and Istanbul Technical University)	Genomic Laboratory (GLAB), Istanbul Technical University	Ilker Karacan, Tugba Kizilboga Akgun, Bugra Agaoglu, Gizem Alkurt, Jale Yildiz, Betsi Köse, Elifnaz Çelik, Arzu Irvem, Yasemin Kendir Demirkol, Ozlem Akgun Dogan, Mehtap Aydn, Levent Doganay, Gizem Dinler Doganay
EPI_ISL_428485	District Surveillence Unit	Department of Neurovirology, National Institute of Mental Health and Neuroscience (NIMHANS)	Chitra Pattabiraman, Vijayalakshmi Reddy, Harsha PK, Risha Rasheed, Shafeeq S Hameed, Manjunatha Venkataswamy, Anita Desai, Ravi Vasanthapuram
EPI_ISL_428670	Centre for Dengue Research	Centre for Dengue Research	Chandima Jeewandara, Dinuka Ariyaratne, Laksiri Gomes, Deshni Jayathilaka, Ananda Wijewickrama, Eranga Narangoda, Damayanthi Idampitiya, Neelika Malaige
EPI_ISL_428671	Centre for Dengue Research	Centre for Dengue Research	Chandima Jeewandara, Dinuka Ariyatane, Laksiri Gomes, Deshni Jayathilaka, Diyanath Ranasinghe, Ananda Wijewickrama, Eranga Narangoda, Damayanthi Tdampitiya, Neelika Malavige
EPI_ISL_428672, EPI_ISL_428673	Centre for Dengue Research	Centre for Dengue Research	Chandima Jeewandara, Dinuka Ariyaratne, Laksiri Gomes, Deshni Jayathilaka, Diyanath Ranasinghe, Ananda Wijewickrama, Eranga Narangoda, Damayanthi Idampitiya, Neelika Malavige
EPI_ISL_428851, EPI_ISL_428852	FSBSI "Chumakov Federal Scientific Center for Research and Development of Immune-and-Biological Products of Russian Academy of Sciences"	FSBSI "Chumakov Federal Scientific Center for Research and Development of Immune-and-Biological Products of Russian Academy of Sciences" & NRC "Kurchatov institute"	Liubov Kozlovskaya, Anastasia Piniaeva, Georgy Ignatyev, Anna Shishova, Aydar Ishmukhametov, Mikhail Rychev, Egor Prokhorchuk, Denis Protsenko, Anastasia Berestovskaya
EPI_ISL_428855	MRCG at LSHTM Geomics lab	MRCG at LSHTM Genomics lab	Sesay et al
EPI_ISL_428856	MRCG at LSHTM Genomics Lab	MRCG at LSHTM Genomics lab	Sesay et al
EPI_ISL_429256	Health Sciences Technology Park, Avicena, 8, 18016 Granada. Spain	Sequencing and Bioinformatics Service FISABIO-Public Health	Joaquín Mendoza, Almudena Rojas, Pablo Mendoza
EPI_ISL_429852, EPI_ISL_429853, EPI_ISL_429854, EPI_ISL_429855	Centers for Disease Control and Prevention of Lishui	Department of InspectionCenters for Disease Control and Prevention of Lishui	Wang Xiaoguang,Ji Qiaoying,Ji Jiansong,Ye Bifeng,Ye Ling
EPI_ISL_429968	Centre Hospitalier Compiègne Laboratoire de Biologie	National Reference Center for Viruses of Respiratory Infections, Institut Pasteur, Paris	Mélanie Albert, Marion Barbet, Sylvie Behillil, Méline Bizard, Angela Brisebarre, Flora Donati, Fabiana Gambaro, Etienne Simon-Lorière, Vincent Enouf, Maud Vanpeene, Sylvie van der Werf, Raulin Olivia
EPI_ISL_430067, EPI_ISL_430068, EPI_ISL_430069, EPI_ISL_430070, EPI_ISL_430071, EPI_ISL_430072, EPI_ISL_430073, EPI_ISL_430074, EPI_ISL_430075, EPI_ISL_430076, EPI_ISL_430077, EPI_ISL_430078, EPI_ISL_430079, EPI_ISL_430080, EPI_ISL_430081, EPI_ISL_430082, EPI_ISL_430083, EPI_ISL_430084, EPI_ISL_430085, EPI_ISL_430086, EPI_ISL_430087, EPI_ISL_430088, EPI_ISL_430089, EPI_ISL_430090, EPI_ISL_430100, EPI_ISL_430101, EPI_ISL_430102, EPI_ISL_430103, EPI_ISL_430104, EPI_ISL_430105, EPI_ISL_430106, EPI_ISL_430107, EPI_ISL_430108, EPI_ISL_430109, EPI_ISL_430110, EPI_ISL_430111, EPI_ISL_430112			
see above	WHO National Influenza Centre Russian Federation	WHO National Influenza Centre Russian Federation	Andrey Komissarov, Artem Fadeev, Mariia Sergeeva, Anna Ivanova, Daria Danilenko
EPI_ISL_430297	National Institute for Communicable Diseases of the National Health Laboratory Service	National Institute for Communicable Diseases of the National Health Laboratory Service	Allam M, Kwenda S, van Heusden P, Khumalo Z, Mohale T, Subramoney K, von Gottberg, A, Ismail A, Bhiman JN
EPI_ISL_430439	Institute for Medical Research, Infectious Disease Research Centre, National Institutes of Health, Ministry of Health Malaysia	Institute for Medical Research Infectious Disease Research Centre, National Institutes of Health, Ministry of Health Malaysia	Suppiah.J, Mohd-Zawawi.Z, Kalyanasundram.J, Azizan.M-A, Mat-Sharani.S, Hisham.H-A, Tan.L-P, Abdul-Wahid.M-Z, Mohd-Zain.R, Ahmad.N, Thayan.R
EPI_ISL_430440	Institute for Medical Research, Infectious Disease Research Centre, National Institutes of Health, Ministry of Health Malaysia	Institute for Medical Research, Infectious Disease Research Centre, National Institutes of Health, Ministry of Health Malaysia	Suppiah.J, Mohd-Zawawi.Z, Kalyanasundram.J, Azizan.M-A, Mat-Sharani.S, Hisham.H-A, Tan.L-P, Abdul-Wahid.M-Z, Tengku-Abd-Rashid.T-R, Mohd-Zain.R, Ahmad.N, Thayan.R
EPI_ISL_430441, EPI_ISL_430442, EPI_ISL_430443, EPI_ISL_430444	Institute for Medical Research, Infectious Disease Research Centre, National Institutes of Health, Ministry of Health Malaysia	Institute for Medical Research, Infectious Disease Research Centre, National Institutes of Health, Ministry of Health Malaysia	Suppiah.J, Mohd-Zawawi.Z, Kalyanasundram.J, Azizan.M-A, Mat-Sharani.S, Hisham.H-A, Tan.L-P, Abdul-Wahid.M-Z, Tengku-Rogayah.TAR, Mohd-Zain.R, Ahmad.N, Thayan.R
EPI_ISL_430456	Rizal Medical Center	Research Institute for Tropical Medicine	Medado,I.A.P., Bautista,C.T., Onza,O.J.T., Polotan,F.G.M., Brunker, K., Mercado,E.S., Manalo, D.L., Demetria, C.S.
EPI_ISL_430469	Hellenic Pasteur Institute, Public Health Laboratories	Hellenic Pasteur Institute, Public Health Laboratories, Unit of Bioinformatics and Applied Genomics	Vasiliki Pogka, Timokratis Karamitros, Athanasios Kossyvakis, Antonios Kalliaropoulos, Horefti Elina, Evangelidou Maria, Androniki Voulgari-Kokota, Aspasia Kontou, Andreas Mentis
EPI_ISL_430722, EPI_ISL_430723, EPI_ISL_430724, EPI_ISL_430725, EPI_ISL_430726, EPI_ISL_430727, EPI_ISL_430728, EPI_ISL_430729, EPI_ISL_430730, EPI_ISL_430731, EPI_ISL_430732, EPI_ISL_430733, EPI_ISL_430734, EPI_ISL_430735, EPI_ISL_430736, EPI_ISL_430737, EPI_ISL_430738, EPI_ISL_430739, EPI_ISL_430740, EPI_ISL_430741, EPI_ISL_430742			
see above	Chinese PLA Institute for Disease Control and Prevention	Chinese PLA Institute for Disease Control and Prevention	Peng LiJinhui Li, Lizhong Li
EPI_ISL_430819	Center of Scientific Excellence for Influenza Viruses,National Research Centre (NRC), Egypt.	Center of Scientific Excellence for Influenza Viruses,National Research Centre (NRC), Egypt.	Mohamed Ahmed Ali, Ahmed Kandeil, Ahmed Mostafa, Rabeh El-Shesheny, Mahmoud Shehata, Wael Roshdy, Shymaa Showky Ahmed , Amal Naguib, Nancy M. El Guindy, Mokhtar Gomaa, Ahmed El-Taweel, Ahmed E Kayed, Yassmin Moatasim, Omnia Kutkat, Sara Mahmoud, Mina Kamel, Abo Shama, M Noura, Mohamed El Sayes
EPI_ISL_430820	Center of Scientific Excellence for Influenza Viruses, National Research Centre (NRC), Egypt.	Center of Scientific Excellence for Influenza Viruses, National Research Centre (NRC), Egypt.	Mohamed Ahmed Ali, Ahmed Kandeil, Ahmed Mostafa, Rabeh El-Shesheny, Mahmoud Shehata, Wael Roshdy, Shymaa Showky Ahmed , Amal Naguib, Mokhtar Gomaa, Ahmed El-Taweel, Ahmed E Kayed, Yassmin Moatasim, Omnia Kutkat, Sara Mahmoud, Mina Kamel, Abo Shama, M Noura, Mohamed El Sayes, Nancy M. El Guindy
EPI_ISL_430839	Research Institute for Tropical Medicine	Research Institute for Tropical Medicine	Medado,I.A.P., Bautista,C.T., Onza,O.J.T., Polotan,F.G.M., Brunker, K., Mercado,E.S., Manalo, D.L., Demetria, C.S.
EPI_ISL_430840	Veterans Memorial Medical Center	Research Institute for Tropical Medicine	Medado,I.A.P., Bautista,C.T., Onza,O.J.T., Polotan,F.G.M., Brunker, K., Mercado,E.S., Manalo, D.L., Demetria, C.S.
EPI_ISL_430846	General Intensive Care Unit, Raymond Poincaré Hospital (AP-HP), Lab Inflammation & Infection, U1173 University Paris Saclay-UVSQ/INSERM, Garches, France.	Institut Pasteur, Laboratory for Urgent Response to biological Threats	Annane Djillali, Vanhomwegen Jessica, Caro Valérie, Manuguerra Jean-Claude
EPI_ISL_431101	Department of Microbiology,Gandhi Medical College and Hospital	Virus Research Laboratory, Department of Zoology, Osmania University,Hyderabad,India	Muttineni Radhakrishna, Nagamani K, Thrilok Chander B, Raja Rao M, Kalyani Putty, Ravikumar P, Sunitha P, Pankaj Singh D, Anand Kumar K, Amit A. Upadhyay Steven E. Bosinger, Rama Amara
EPI_ISL_431102	Department of MicroBiology,Gandhi Medical College and Hospital,Secendrabad,Hyderabad,India	Department of Microbiology, Gandhi Medical College and Hospital, Secendrabad, Hyderabad	Nagamani K, Muttineni Radhakrishna, Thrilok Chander B, Raja Rao M, Kalyani Putty, Ravikumar P, Sunitha P, Pankaj Singh D, Anand Kumar K, Amit A. Upadhyay, Steven E. Bosinger, Rama Amara
EPI_ISL_431103	Department of Microbiology, Gandhi Medical College and Hospital, Secendrabad, Hyderabad, India	Department of Microbiology, Gandhi Medical College and Hospital, Secendrabad, Hyderabad, India	Nagamani K, Muttineni Radhakrishna, Thrilok Chander B, Raja Rao M, Kalyani Putty, Ravikumar P, Sunitha P, Pankaj Singh D, Anand Kumar K, Amit A. Upadhyay, Steven E. Bosinger, Rama Amara

EPI_ISL_431117	Department of Microbiology, Gandhi Medical College and Hospital, Secendrabad, Hyderabad, India	Department of Microbiology, Gandhi Medical College and Hospital, Secendrabad, Hyderabad, India	Thrilok Chander B, Muttineni Radhakrishna, Nagamani K, Raja Rao M, Kalyani Putty, Ravikumar P, Sunitha P, Pankaj Singh D, Anand Kumar K, Amit A. Upadhyay, Steven E.Bosinger, Rama Amara
EPI_ISL_431118, EPI_ISL_431180, EPI_ISL_431240, EPI_ISL_431292, EPI_ISL_431779, EPI_ISL_431780, EPI_ISL_431781, EPI_ISL_431782, EPI_ISL_431783, EPI_ISL_431784, EPI_ISL_431785			
see above	Fujian Center for Disease Control and Prevention	Fujian Center for Disease Control and Prevention	Lin Qi, Huang Zhimiao, Zhang Yanhua, Weng Yuwei
EPI_ISL_434366, EPI_ISL_434367, EPI_ISL_434369, EPI_ISL_434371	Hospital AZ Rivierenland	Institute of Tropical Medicine	Philippe Selhorst, Colin Anthony
EPI_ISL_434372, EPI_ISL_434373, EPI_ISL_434375, EPI_ISL_434376, EPI_ISL_434377, EPI_ISL_434378, EPI_ISL_434379, EPI_ISL_434380, EPI_ISL_434381, EPI_ISL_434383	Hospital AZ Rivierenland	Institute of Tropical Medicine	Philippe Selhorst, Colin Anthony,
EPI_ISL_434384, EPI_ISL_434385	Hospital AZ Rivierenland	Institute of Tropical Medicine	Philippe Selhorst, Colin Anthony
EPI_ISL_434533	Area de Salud Alajuela Sur	Inciensa, Instituto Costarricense de Investigación y Enseñanza en Nutrición y Salud	Francisco Duarte, Hebleen Porras, Claudio Soto-Garita, Estela Cordero, Adriana Godinez & Melany Calderon
EPI_ISL_434534	National Institute for Viral Disease Control and Prevention, China CDC	National Institute for Viral Disease Control and Prevention, China CDC, Yunnan Provincial CDC	Wenjie Tan, Roujian Lu, Wenling Wang, Peihua Niu, Huijuan Wang, Baoying Huang, Li Zhao, Fei Ye, Guizhen Wu
EPI_ISL_434535	Area de Salud Alajuela Sur	Inciensa, Instituto Costarricense de Investigación y Enseñanza en Nutrición y Salud	Francisco Duarte, Hebleen Porras, Claudio Soto-Garita, Estela Cordero, Adriana Godinez & Melany Calderon
EPI_ISL_434536	Hospital San Vicente de Paul	Inciensa, Instituto Costarricense de Investigación y Enseñanza en Nutrición y Salud	Francisco Duarte, Hebleen Porras, Claudio Soto-Garita, Estela Cordero, Adriana Godinez & Melany Calderon
EPI_ISL_434538	COOPESAIN	Inciensa, Instituto Costarricense de Investigación y Enseñanza en Nutrición y Salud	Francisco Duarte, Hebleen Porras, Claudio Soto-Garita, Estela Cordero, Adriana Godinez & Melany Calderon
EPI_ISL_434539	Area de Salud Orotina	Inciensa, Instituto Costarricense de Investigación y Enseñanza en Nutrición y Salud	Francisco Duarte, Hebleen Porras, Claudio Soto-Garita, Estela Cordero, Adriana Godinez & Melany Calderon
EPI_ISL_434540	EBAIS Concepción Norte	Inciensa, Instituto Costarricense de Investigación y Enseñanza en Nutrición y Salud	Francisco Duarte, Hebleen Porras, Claudio Soto-Garita, Estela Cordero, Adriana Godinez & Melany Calderon
EPI_ISL_434541, EPI_ISL_434542, EPI_ISL_434543, EPI_ISL_434544, EPI_ISL_434545, EPI_ISL_434546, EPI_ISL_434547, EPI_ISL_434548, EPI_ISL_434549, EPI_ISL_434550, EPI_ISL_434551, EPI_ISL_434552, EPI_ISL_434553			
see above	Puerto Rico Department of Health	Centers for Disease Control and Prevention, Dengue Branch	Gilberto A. Santiago, Glenda Gonzalez, Betzabel Flores, Keyla Charriez, Fabiola Cruz, Chaney Kalinich, Joseph Fauver, Jessica I. Falcon, Nathan Grubaugh, Jorge L. Munoz-Jordan
EPI_ISL_434572	The National Institute of Public Health Center for Epidemiology and Microbiology	The National Institute of Public Health Center for Epidemiology and Microbiology	Alexander Nagy, Helena Jirincova, Ludmila Novakova, Dusan Trnka, Jaromira Vecerova
EPI_ISL_435045	Laboratory of Applied Genetics	RSE "National Center for Biotechnology"	Alexandr Shevtsov, Ilyas Akhmetollayev, Viktoriya Lutsay, Asylulan Amirgazin, Ruslan Kalendar, Yerlan Ramanculov
EPI_ISL_435049	B.J. Medical College and Civil hospital	Gujarat Biotechnology Research Centre	Pinal Trivedi, Maharshi Pandya, Amit Kanani, Akanksha Verma, Nitin Savaliya, Raghawendra Kumar, Dinesh Kumar, Zuber Saiyed, Dipa Kinariwala, Disha Patel, Binita Aring, Geeta Vaghela, Sonia Barve, Bhavesh Modi, Kairavi Joshi, Gaurishankar Shrimali, Nidhi Sood, Pranay Shah, R D Dixit, Snehal Bagatharia, Kamlesh J Upadhyay, Ramesh Pandit, Tejas Shah, Ankit Hinsu, Pritesh Sabara, Apurvasinh Puvar, Janvi Raval, Monika Gandhi, Neha Rajpara, Chaitanya Joshi, Madhvi Joshi
EPI_ISL_435050	B.J. Medical College and Civil hospital	Gujarat Biotechnology Research Centre	Ankit Hinsu, Pritesh Sabara, Apurvasinh Puvar, Janvi Raval, Monika Gandhi, Pinal Trivedi, Maharshi Pandya, Amit Kanani, Akanksha Verma, Nitin Savaliya, Raghawendra Kumar, Dinesh Kumar, Zuber Saiyed, Dipa Kinariwala, Disha Patel, Binita Aring, Geeta Vaghela, Sonia Barve, Bhavesh Modi, Kairavi Joshi, Gaurishankar Shrimali, Nidhi Sood, Pranay Shah, R D Dixit, Snehal Bagatharia, Kamlesh J Upadhyay, Ramesh Pandit, Tejas Shah, Dipeshwari Shewale, Chaitanya Joshi, Madhvi Joshi
EPI_ISL_435051	B.J. Medical College and Civil hospital	Gujarat Biotechnology Research Centre	Pritesh Sabara, Apurvasinh Puvar, Janvi Raval, Monika Gandhi, Pinal Trivedi, Maharshi Pandya, Amit Kanani, Akanksha Verma, Nitin Savaliya, Raghawendra Kumar, Dinesh Kumar, Zuber Saiyed, Dipa Kinariwala, Disha Patel, Binita Aring, Geeta Vaghela, Sonia Barve, Bhavesh Modi, Kairavi Joshi, Gaurishankar Shrimali, Nidhi Sood, Pranay Shah, R D Dixit, Snehal Bagatharia, Kamlesh J Upadhyay, Ramesh Pandit, Tejas Shah, Ankit Hinsu, Vasudha Sharma, Chaitanya Joshi, Madhvi Joshi
EPI_ISL_435052	B.J. Medical College and Civil hospital	Gujarat Biotechnology Research Centre	Apurvasinh Puvar, Janvi Raval, Monika Gandhi, Pinal Trivedi, Maharshi Pandya, Amit Kanani, Akanksha Verma, Nitin Savaliya, Raghawendra Kumar, Dinesh Kumar, Zuber Saiyed, Dipa Kinariwala, Disha Patel, Binita Aring, Geeta Vaghela, Sonia Barve, Bhavesh Modi, Kairavi Joshi, Gaurishankar Shrimali, Nidhi Sood, Pranay Shah, R D Dixit, Snehal Bagatharia, Kamlesh J Upadhyay, Ramesh Pandit, Tejas Shah, Ankit Hinsu, Pritesh Sabara, Pooja P Doshi, Chaitanya Joshi, Madhvi Joshi
EPI_ISL_435053	B.J. Medical College and Civil hospital	Gujarat Biotechnology Research Centre	Janvi Raval, Monika Gandhi, Pinal Trivedi, Maharshi Pandya, Amit Kanani, Akanksha Verma, Nitin Savaliya, Raghawendra Kumar, Dinesh Kumar, Zuber Saiyed, Dipa Kinariwala, Disha Patel, Binita Aring, Geeta Vaghela, Sonia Barve, Bhavesh Modi, Kairavi Joshi, Gaurishankar Shrimali, Nidhi Sood, Pranay Shah, R D Dixit, Snehal Bagatharia, Kamlesh J Upadhyay, Ramesh Pandit, Tejas Shah, Ankit Hinsu, Pritesh Sabara, Apurvasinh Puvar, Nidhi Patel, Chaitanya Joshi, Madhvi Joshi
EPI_ISL_435055	Gujarat Biotechnology Research Centre	Gujarat Biotechnology Research Centre	Tejas Shah, Ankit Hinsu, Pritesh Sabara, Apurvasinh Puvar, Janvi Raval, Monika Gandhi, Pinal Trivedi, Maharshi Pandya, Amit Kanani, Akanksha Verma, Nitin Savaliya, Raghawendra Kumar, Dinesh Kumar, Zuber Saiyed, Dipa Kinariwala, Disha Patel, Binita Aring, Geeta Vaghela, Sonia Barve, Bhavesh Modi, Kairavi Joshi, Gaurishankar Shrimali, Nidhi Sood, Pranay Shah, R D Dixit, Snehal Bagatharia, Kamlesh J Upadhyay, Ramesh Pandit, Anjali Rajwal, Chaitanya Joshi, Madhvi Joshi
EPI_ISL_435056	Gujarat Biotechnology Research Centre	Gujarat Biotechnology Research Centre	Maharshi Pandya, Amit Kanani, Akanksha Verma, Nitin Savaliya, Raghawendra Kumar, Dinesh Kumar, Zuber Saiyed, Dipa Kinariwala, Disha Patel, Binita Aring, Geeta Vaghela, Sonia Barve, Bhavesh Modi, Kairavi Joshi, Gaurishankar Shrimali, Nidhi Sood, Pranay Shah, R D Dixit, Snehal Bagatharia, Kamlesh J Upadhyay, Ramesh Pandit, Tejas Shah, Ankit Hinsu, Pritesh Sabara, Apurvasinh Puvar, Janvi Raval, Monika Gandhi, Pinal Trivedi, Afzal Ansari, Chaitanya Joshi, Madhvi Joshi
EPI_ISL_435057	T.C. Salk Bakanl Adyaman l Salk Müdürlüü Adyaman Eitim Ve Aratrma Hastanesi	VETAL Animal Health Products Company, BSL3+ Production Laboratuary, Turkey	Fatma Nilay Tutak, Haluk Uluca, Fethiye Sevimli, O. Ugur Sezerman
EPI_ISL_435058	National Institute for Communicable Diseases of the National Health Laboratory Service	National Institute for Communicable Diseases of the National Health Laboratory Service	Allam M, Kwenda S, van Heusden P, Khumalo Z, Mohale T, Subramoney K, von Gottberg, A, Ismail A, Bhiman JN
EPI_ISL_435145	Ospedale Civile Giuseppe Mazzini	Istituto Zooprofilattico Sperimentale dell'Abruzzo e Molise "G.Caporale"	Lorusso A, Marcacci M, Di Domenico M, Ancora M, Curini V, Mangone I, Rinaldi A, Di Pasquale A, Cammà C, Puglia I, Savini G
EPI_ISL_435146, EPI_ISL_435147	Villa Serena del Dr. Leonardo Petruzzi	Istituto Zooprofilattico Sperimentale dell'Abruzzo e Molise "G.Caporale"	Lorusso A, Marcacci M, Di Domenico M, Ancora M, Curini V, Mangone I, Rinaldi A, Di Pasquale A, Cammà C, Puglia I, Savini G
EPI_ISL_435148	Ospedale SS Annunziata	Istituto Zooprofilattico Sperimentale dell'Abruzzo e Molise "G.Caporale"	Lorusso A, Marcacci M, Di Domenico M, Ancora M, Curini V, Mangone I, Rinaldi A, Di Pasquale A, Cammà C, Puglia I, Savini G
EPI_ISL_435149	SERVIZIO DI IGIENE E SANITÀ PUBBLICA ASL Teramo	Istituto Zooprofilattico Sperimentale dell'Abruzzo e Molise "G.Caporale"	Lorusso A, Marcacci M, Di Domenico M, Ancora M, Curini V, Mangone I, Rinaldi A, Di Pasquale A, Cammà C, Puglia I, Savini G

EPI_ISL_435150, EPI_ISL_435151	Ospedale SS Annunziata	Istituto Zooprofilattico Sperimentale dell'Abruzzo e Molise "G.Caporale"	Lorusso A, Marcacci M, Di Domenico M, Ancora M, Curini V, Mangone I, Rinaldi A, Di Pasquale A, Cammà C, Puglia I, Savini G
EPI_ISL_435152	Servizio di Igiene, Epidemiologia e Sanità Pubblica (SIESP) Avezzano	Istituto Zooprofilattico Sperimentale dell'Abruzzo e Molise "G.Caporale"	Lorusso A, Marcacci M, Di Domenico M, Ancora M, Curini V, Mangone I, Rinaldi A, Di Pasquale A, Cammà C, Puglia I, Savini G
EPI_ISL_435153, EPI_ISL_435154, EPI_ISL_435155	SERVIZIO DI IGIENE E SANITÀ PUBBLICA ASL Teramo	Istituto Zooprofilattico Sperimentale dell'Abruzzo e Molise "G.Caporale"	Lorusso A, Marcacci M, Di Domenico M, Ancora M, Curini V, Mangone I, Rinaldi A, Di Pasquale A, Cammà C, Puglia I, Savini G
EPI_ISL_435281	Medistra Hospital Jakarta	Eijkman Institute for Molecular Biology, Ministry of Research and Technology/National Agency for Research and Innovation	Edison Johar, Frilasita A Yudhaputri, Hidayat Trimarsanto, David H Muljono, Safarina G Malik, Khin Saw Myint, Amin Soebandrio
EPI_ISL_435282, EPI_ISL_435283	RS Pondok Indah Hospital - Pondok Indah	Eijkman Institute for Molecular Biology, Ministry of Research and Technology/National Agency for Research and Innovation	Edison Johar, Frilasita A Yudhaputri, Hidayat Trimarsanto, David H Muljono, Safarina G Malik, Khin Saw Myint, Amin Soebandrio
EPI_ISL_435284	Central Virology Laboratory, Israel Ministry of Health	Central Virology Laboratory, Israel Ministry of Health	Neta Zuckerman, Efrat Bucris, Oran Erster, Danit Sofer, Orna Mor, Ella Mendelson, Michal Mandelboim
EPI_ISL_435286	Central Virology Laboratory, Israel Ministry of Health	Central Virology Laboratory, Israel Ministry of Health	eta Zuckerman, Efrat Bucris, Oran Erster, Orna Mor, Ella Mendelson, Michal Mandelboim, Danit Sofer
EPI_ISL_435287, EPI_ISL_435289, EPI_ISL_435291	Central Virology Laboratory, Israel Ministry of Health	Central Virology Laboratory, Israel Ministry of Health	Neta Zuckerman, Efrat Bucris, Oran Erster, Danit Sofer, Orna Mor, Ella Mendelson, Michal Mandelboim
EPI_ISL_435292	Central Virology Laboratory, Israel Ministry of Health	Central Virology Laboratory, Israel Ministry of Health	Neta Zuckerman, Efrat Bucris, Oran Erster, Danit Sofer, Ella Mendelson, Michal Mandelboim, Orna Mor
EPI_ISL_435303	National Hospital of Tropical Diseases	Oxford University Clinical Research Unit, Hanoi, Vietnam	Nguyen Thi Tam, Van Dinh Trang, Nguyen Thu Trang, Nguyen Thi Ngoc Diep, Le Nguyen Minh Hoa, Pham Ngoc Thach, H.Rogier van Doorn, on behalf of the OUCRU COVID-19 research group
EPI_ISL_435305	National Hospital of Tropical Diseases	Oxford University Clinical Research Unit, Hanoi, Vietnam	Nguyen Thi Tam, Van Dinh Trang, Nguyen Thu Trang, Nguyen Thi Ngoc Diep, Le Nguyen Minh Hoa, Pham Ngoc Thach, H. Rogier van Doorn, on behalf of the OUCRU COVID-19 research group
EPI_ISL_435405, EPI_ISL_435407, EPI_ISL_435408, EPI_ISL_435410, EPI_ISL_435412, EPI_ISL_435414, EPI_ISL_435416, EPI_ISL_435419, EPI_ISL_435422, EPI_ISL_435423, EPI_ISL_435424, EPI_ISL_435425, EPI_ISL_435430, EPI_ISL_435431			
see above	Virological Research Group, Szentágothai Research Centre	Bioinformatics Research Group, Szentágothai Research Centre	Péter Urbán, Endre Gábor Tóth, Gábor Kemenesi, Róbert Herczeg, Attila Gyenesei, Ferenc Jakab
EPI_ISL_435723	Laboratory of Genomics & Bioinformatics, Institute of Immunology and Experimental Therapy, Polish Academy of Sciences Oddzia Mikrobiologii Wojewódzkiej Stacji Sanitarno Epidemiologiczna	Laboratory of Genomics & Bioinformatics, Institute of Immunology and Experimental Therapy, Polish Academy of Sciences	Aleksandra Herud, Dorota Kujawa, Dariusz Martynowski, Krzysztof Jakub Pawlik, Joanna Sikorska, Paulina ebrowska, Grayna Zalewska, Oskar Karpiski and ukasz aczmaski
EPI_ISL_436099	TSGH-CP molecular lab	TSGH-CP molecular lab	Cherng-Lih Perng, Ming-Jr JIAN, Chih-Kai Chang, Jung-Chung Lin, Kuo-Ming Yeh, Chien-Wen Chen, Sheng-Kang Chiu, Hsing-Yi Chung, Shih-Hung Tsai, Kuo-Sheng Hung, Tien-Yao Chang, Feng-Yee Chang, Hung-Sheng Shang
EPI_ISL_436100	TSGH-CP molecular lab	TSGH-CP molecular lab	Cherng-Lih Perng, Ming-Jr Jian, Chih-Kai Chang, Jung-Chung Lin, Kuo-Ming Yeh, Chien-Wen Chen, Sheng-Kang Chiu, Hsing-Yi Chung, Shih-Hung Tsai, Kuo-Sheng Hung, Tien-Yao Chang, Feng-Yee Chang, Hung-Sheng Shang
EPI_ISL_436101, EPI_ISL_436102, EPI_ISL_436103, EPI_ISL_436104	TSGH-CP molecular lab	TSGH-CP molecular lab	Cherng-Lih Perng, Ming-Jr JIAN, Chih-Kai Chang, Jung-Chung Lin, Kuo-Ming Yeh, Chien-Wen Chen, Sheng-Kang Chiu, Hsing-Yi Chung, Shih-Hung Tsai, Kuo-Sheng Hung, Tien-Yao Chang, Feng-Yee Chang, Hung-Sheng Shang
EPI_ISL_436105	TSGH-CP molecular lab	TSGH-CP molecular lab	Cherng-Lih Perng, Ming-Jr Jian, Chih-Kai Chang, Jung-Chung Lin, Kuo-Ming Yeh, Chien-Wen Chen, Sheng-Kang Chiu, Hsing-Yi Chung, Shih-Hung Tsai, Kuo-Sheng Hung, Tien-Yao Chang, Feng-Yee Chang, Hung-Sheng Shang
EPI_ISL_436106, EPI_ISL_436107, EPI_ISL_436108	TSGH-CP molecular lab	TSGH-CP molecular lab	Cherng-Lih Perng, Ming-Jr JIAN, Chih-Kai Chang, Jung-Chung Lin, Kuo-Ming Yeh, Chien-Wen Chen, Sheng-Kang Chiu, Hsing-Yi Chung, Shih-Hung Tsai, Kuo-Sheng Hung, Tien-Yao Chang, Feng-Yee Chang, Hung-Sheng Shang
EPI_ISL_436137, EPI_ISL_436138, EPI_ISL_436139, EPI_ISL_436140, EPI_ISL_436141, EPI_ISL_436156, EPI_ISL_436157	District Surveillance Unit	Department of Neurovirology, National Institute of Mental Health and Neuroscience (NIMHANS)	Chitra Pattabiraman, Vijayalakshmi Reddy, Harsha PK, Risha Rasheed, Shafeeq S Hameed, Manjunatha Venkataswamy, Anita Desai, Ravi Vasanthapuram
EPI_ISL_436412	Viral Respiratory Lab, National Institute for Biomedical Research (INRB)	Pathogen Sequencing Lab, National Institute for Biomedical Research (INRB)	Placide Mbala-Kingebeni, Edith Nkwembe, Eddy Kinganda-Lusamaki, Amuri Aziza, Francisca Muyembe Mawete, Catherine Pratt, Matthias Pauthner, Josh Quick, Allison Black, James Hadfield, Trevor Bedford, Ian Goodfellow, Andrew Rambaut, Nick Loman, Kristian Andersen, Michael Wiley, Steve Ahuka-Mundeke, Jean-Jacques Muyembe Tamfum
EPI_ISL_436684, EPI_ISL_436685, EPI_ISL_436686, EPI_ISL_436687	KRISP, KZN Research Innovation and Sequencing Platform	KRISP, KZN Research Innovation and Sequencing Platform	Giandhari J, Pillay S, Lessells R, Chimukangara B, Deforche K, Tegally H, Wilkinson E, de Oliveira T
EPI_ISL_436715, EPI_ISL_436716, EPI_ISL_436717	Genomics and Computational Biology Lab, Scientific Research Institute of Physical-Chemical Medicine, FMBA of Russia	Genomics and Computational Biology Lab, Scientific Research Institute of Physical-Chemical Medicine, FMBA of Russia	A. Pavlenko, O. Guskova, K. Klimina, V. Veselovsky, A. Manolov, D. Fedorov, V. Govorun and E. Ilina
EPI_ISL_436718	Ospedale Regionale San Salvatore	Istituto Zooprofilattico Sperimentale dell'Abruzzo e Molise "G.Caporale"	Lorusso A, Marcacci M, Di Domenico M, Ancora M, Curini V, Mangone I, Rinaldi A, Di Pasquale A, Cammà C, Puglia I, Savini G
EPI_ISL_436719, EPI_ISL_436720, EPI_ISL_436721, EPI_ISL_436722	Ospedale Civile S. Liberatore di Atri	Istituto Zooprofilattico Sperimentale dell'Abruzzo e Molise "G.Caporale"	Lorusso A, Marcacci M, Di Domenico M, Ancora M, Curini V, Mangone I, Rinaldi A, Di Pasquale A, Cammà C, Puglia I, Savini G
EPI_ISL_436723	Ospedale Civile Giuseppe Mazzini	Istituto Zooprofilattico Sperimentale dell'Abruzzo e Molise "G.Caporale"	Lorusso A, Marcacci M, Di Domenico M, Ancora M, Curini V, Mangone I, Rinaldi A, Di Pasquale A, Cammà C, Puglia I, Savini G
EPI_ISL_436724	Ospedale Civile S. Liberatore di Atri	Istituto Zooprofilattico Sperimentale dell'Abruzzo e Molise "G.Caporale"	Lorusso A, Marcacci M, Di Domenico M, Ancora M, Curini V, Mangone I, Rinaldi A, Di Pasquale A, Cammà C, Puglia I, Savini G
EPI_ISL_436725	RSA/RP Villa San Giovanni - Gruppo Edos	Istituto Zooprofilattico Sperimentale dell'Abruzzo e Molise "G.Caporale"	Lorusso A, Marcacci M, Di Domenico M, Ancora M, Curini V, Mangone I, Rinaldi A, Di Pasquale A, Cammà C, Puglia I, Savini G
EPI_ISL_436726, EPI_ISL_436727, EPI_ISL_436728, EPI_ISL_436729	SERVIZIO DI IGIENE E SANITÀ PUBBLICA ASL Teramo	Istituto Zooprofilattico Sperimentale dell'Abruzzo e Molise "G.Caporale"	Lorusso A, Marcacci M, Di Domenico M, Ancora M, Curini V, Mangone I, Rinaldi A, Di Pasquale A, Cammà C, Puglia I, Savini G
EPI_ISL_436730	Servizio di igiene epidemiologia e sanità pubblica (Siesp) Chieti	Istituto Zooprofilattico Sperimentale dell'Abruzzo e Molise "G.Caporale"	Lorusso A, Marcacci M, Di Domenico M, Ancora M, Curini V, Mangone I, Rinaldi A, Di Pasquale A, Cammà C, Puglia I, Savini G
EPI_ISL_436731, EPI_ISL_436732	Ospedale Civile S. Liberatore di Atri	Istituto Zooprofilattico Sperimentale dell'Abruzzo e Molise "G.Caporale"	Lorusso A, Marcacci M, Di Domenico M, Ancora M, Curini V, Mangone I, Rinaldi A, Di Pasquale A, Cammà C, Puglia I, Savini G
EPI_ISL_436939, EPI_ISL_436940, EPI_ISL_436941, EPI_ISL_436942, EPI_ISL_436943, EPI_ISL_436944, EPI_ISL_436945, EPI_ISL_436946, EPI_ISL_436947, EPI_ISL_436948, EPI_ISL_436949, EPI_ISL_436950, EPI_ISL_436951, EPI_ISL_436952, EPI_ISL_436953, EPI_ISL_436954, EPI_ISL_436955, EPI_ISL_436956, EPI_ISL_436957, EPI_ISL_436958, EPI_ISL_436959, EPI_ISL_436960, EPI_ISL_436961			
see above	Ochsner Health	Bioinfoexperts, LLC	Amy Feehan, David J. Nolan, Rebecca Rose, Sissy Cross, David Moraga Amador, Tong Yang, Luke Caruso, Wayra Navia, Lydia Von Borstel, Xiao Hui Zhou, Julia-Garcia-Diaz, Susanna L. Lamers
EPI_ISL_437187	Siloam Hospitals	Institute of Tropical Disease, Universitas Airlangga	Kazufumi Shimizu, Krisnoadi Rahardjo, Aldise M Nastri, Jezzy R Dewantari, Rima R Prasetya, Maria M Padmidewi, Gatot Soegiarto, Laksmi Wulandari, Retno A Setyoningrum, Resti Y Meliana, Yohko K Shimizu, Mitsuhiro Nishimura, Yasuko Mori, Soetjipto, Maria I Lusida
EPI_ISL_437188	RSUD Dr. Soetomo	Institute of Tropical Disease, Universitas Airlangga	Krisnoadi Rahardjo, Aldise M Nastri, Jezzy R Dewantari, Rima R Prasetya, Joni Wahyuhadi, Gatot Soegiarto, Laksmi Wulandari, Retno A Setyoningrum,

			Resti Y Meliana, Yohko K Shimizu, Mitsuhiro Nishimura, Yasuko Mori, Soetjipto, Kazufumi Shimizu, Maria I Lusida
EPI_ISL_437189	Pusat Pertamina Hospital	Eijkman Institute for Molecular Biology, Ministry of Research and Technology/National Agency for Research and Innovation	Edison Johar, Frilasita A Yudhaputri, Hidayat Trimarsanto, David H Muljono, Safarina G Malik, Khin Saw Myint, Amin Soebandrio
EPI_ISL_437190, EPI_ISL_437191	RS Pondok Indah Hospital - Pondok Indah	Eijkman Institute for Molecular Biology, Ministry of Research and Technology/National Agency for Research and Innovation	Edison Johar, Frilasita A Yudhaputri, Hidayat Trimarsanto, David H Muljono, Safarina G Malik, Khin Saw Myint, Amin Soebandrio
EPI_ISL_437192	Mitra Keluarga Kelapa Gading Hospital	Eijkman Institute for Molecular Biology, Ministry of Research and Technology/National Agency for Research and Innovation	Edison Johar, Frilasita A Yudhaputri, Hidayat Trimarsanto, David H Muljono, Safarina G Malik, Khin Saw Myint, Amin Soebandrio
EPI_ISL_437193, EPI_ISL_437194	Viral Respiratory Lab, National Institute for Biomedical Research (INRB)	Pathogen Sequencing Lab, National Institute for Biomedical Research (INRB)	Placide Mbala-Kingebeni, Edith Nkwembe, Eddy Kinganda-Lusamaki, Amuri Aziza, Francisca Muyembe Mawete, Catherine Pratt, Matthias Pauthner, Josh Quick, Allison Black, James Hadfield, Trevor Bedford, Ian Goodfellow, Andrew Rambaut, Nick Loman, Kristian Andersen, Michael Wiley, Steve Ahuka-Mundeke, Jean-Jacques Muyembe Tamfum
EPI_ISL_437197, EPI_ISL_437198, EPI_ISL_437199, EPI_ISL_437200, EPI_ISL_437201, EPI_ISL_437202, EPI_ISL_437203, EPI_ISL_437298, EPI_ISL_437299, EPI_ISL_437300, EPI_ISL_437301, EPI_ISL_437302, EPI_ISL_437303			
see above	Diagnostic- and Research Institute of Pathology, Medical University of Graz	Diagnostic- and Research Institute of Pathology, Medical University of Graz	Karl Kashofer, Peter Regitnig, Martin Zacharias, Gregor Gorkiewicz
EPI_ISL_437336	TSGH-CP molecular lab, Division of Clinical Pathology, Department of Pathology	TSGH-CP molecular lab, Division of Clinical Pathology, Department of Pathology	Cherng-Lih Perng, Ming-Jr JIAN, Chih-Kai Chang, Jung-Chung Lin, Kuo-Ming Yeh, Chien-Wen Chen, Sheng-Kang Chiu, Hsing-Yi Chung, Shih-Hung Tsai, Kuo-Sheng Hung, Tien-Yao Chang, Feng-Yee Chang, Hung-Sheng Shang
EPI_ISL_437339, EPI_ISL_437342, EPI_ISL_437344, EPI_ISL_437345	Viral Respiratory Lab, National Institute for Biomedical Research (INRB)	Pathogen Sequencing Lab, National Institute for Biomedical Research (INRB)	Placide Mbala-Kingebeni, Edith Nkwembe, Eddy Kinganda-Lusamaki, Amuri Aziza, Francisca Muyembe Mawete, Catherine Pratt, Matthias Pauthner, Josh Quick, Allison Black, James Hadfield, Trevor Bedford, Ian Goodfellow, Andrew Rambaut, Nick Loman, Kristian Andersen, Michael Wiley, Steve Ahuka-Mundeke, Jean-Jacques Muyembe Tamfum
EPI_ISL_437349	Ecole nationale vétérinaire d'Alfort-laboratoire de santé animale Anses UMR 1161 de virologie ENVA-Anses-INRAE	Institut Pasteur CIBU-ERI	Sophie Le Poder, Corinne Sailleau, Marine Dumarest, Bernard Klonjkowski, Stéphan Zientara
EPI_ISL_437433	Bozeman Health Deaconess Hospital	Wiedenheft lab, Montana State University	Artem Nemudryi, Anna Nemudraia, Kevin Surya, Tanner Wiegand, Murat Buyukyoruk, Royce Wilkinson, Blake Wiedenheft
EPI_ISL_437439	Department of MicroBiology, Government Medical College, Surat	Gujarat Biotechnology Research Centre	Akanksha Verma, Nitin Savaliya, Raghawendra Kumar, Dinesh Kumar, Zuber Saiyed, Dipa Kinariwala, Disha Patel, Binita Aring, Neeta Khandelwal, Geeta Vaghela, Sonia Barve, Bhavesh Modi, Kairavi Joshi, Gaurishankar Shrimali, Nidhi Sood, Pranay Shah, R D Dixit, Snehal Bagatharia, Kamlesh J Upadhyay, Ramesh Pandit, Tejas Shah, Ankit Hinsu, Pritesh Sabara, Apurvasinh Puvar, Janvi Raval, Monika Gandhi, Pinal Trivedi, Maharshi Pandya, Amit Kanani, Armi Chaudhari, Chaitanya Joshi, Madhvi Joshi
EPI_ISL_437440	Department of MicroBiology, Government Medical College, Surat	Gujarat Biotechnology Research Centre	Nitin Savaliya, Raghawendra Kumar, Dinesh Kumar, Zuber Saiyed, Dipa Kinariwala, Disha Patel, Binita Aring, Neeta Khandelwal, Geeta Vaghela, Sonia Barve, Bhavesh Modi, Kairavi Joshi, Gaurishankar Shrimali, Nidhi Sood, Pranay Shah, R D Dixit, Snehal Bagatharia, Kamlesh J Upadhyay, Ramesh Pandit, Tejas Shah, Ankit Hinsu, Pritesh Sabara, Apurvasinh Puvar, Janvi Raval, Monika Gandhi, Pinal Trivedi, Maharshi Pandya, Amit Kanani, Akanksha Verma, Bhavya Jindal, Chaitanya Joshi, Madhvi Joshi
EPI_ISL_437442	Department of MicroBiology, Government Medical College, Surat	Gujarat Biotechnology Research Centre	Dinesh Kumar, Zuber Saiyed, Dipa Kinariwala, Disha Patel, Binita Aring, Neeta Khandelwal, Geeta Vaghela, Sonia Barve, Bhavesh Modi, Kairavi Joshi, Gaurishankar Shrimali, Nidhi Sood, Pranay Shah, R D Dixit, Snehal Bagatharia, Kamlesh J Upadhyay, Ramesh Pandit, Tejas Shah, Ankit Hinsu, Pritesh Sabara, Apurvasinh Puvar, Janvi Raval, Monika Gandhi, Pinal Trivedi, Maharshi Pandya, Amit Kanani, Akanksha Verma, Nitin Savaliya, Raghawendra Kumar, Dipeshwari Shewale, Chaitanya Joshi, Madhvi Joshi
EPI_ISL_437445	B.J. Medical College and Civil hospital	Gujarat Biotechnology Research Centre	Disha Patel, Binita Aring, Neeta Khandelwal, Geeta Vaghela, Sonia Barve, Bhavesh Modi, Kairavi Joshi, Gaurishankar Shrimali, Nidhi Sood, Pranay Shah, R D Dixit, Snehal Bagatharia, Kamlesh J Upadhyay, Ramesh Pandit, Tejas Shah, Ankit Hinsu, Pritesh Sabara, Apurvasinh Puvar, Janvi Raval, Monika Gandhi, Pinal Trivedi, Maharshi Pandya, Amit Kanani, Akanksha Verma, Nitin Savaliya, Raghawendra Kumar, Dinesh Kumar, Zuber Saiyed, Dipa Kinariwala, Nidhi Patel, Chaitanya Joshi, Madhvi Joshi
EPI_ISL_437446	B.J. Medical College and Civil hospital	Gujarat Biotechnology Research Centre	Binita Aring, Neeta Khandelwal, Geeta Vaghela, Sonia Barve, Bhavesh Modi, Kairavi Joshi, Gaurishankar Shrimali, Nidhi Sood, Pranay Shah, R D Dixit, Snehal Bagatharia, Kamlesh J Upadhyay, Ramesh Pandit, Tejas Shah, Ankit Hinsu, Pritesh Sabara, Apurvasinh Puvar, Janvi Raval, Monika Gandhi, Pinal Trivedi, Maharshi Pandya, Amit Kanani, Akanksha Verma, Nitin Savaliya, Raghawendra Kumar, Dinesh Kumar, Zuber Saiyed, Dipa Kinariwala, Disha Patel, Priti Pandita, Chaitanya Joshi, Madhvi Joshi
EPI_ISL_437447	B.J. Medical College and Civil hospital	Gujarat Biotechnology Research Centre	Neeta Khandelwal, Geeta Vaghela, Sonia Barve, Bhavesh Modi, Kairavi Joshi, Gaurishankar Shrimali, Nidhi Sood, Pranay Shah, R D Dixit, Snehal Bagatharia, Kamlesh J Upadhyay, Ramesh Pandit, Tejas Shah, Ankit Hinsu, Pritesh Sabara, Apurvasinh Puvar, Janvi Raval, Monika Gandhi, Pinal Trivedi, Maharshi Pandya, Amit Kanani, Akanksha Verma, Nitin Savaliya, Raghawendra Kumar, Dinesh Kumar, Zuber Saiyed, Dipa Kinariwala, Disha Patel, Binita Aring, Neha Rajpara, Chaitanya Joshi, Madhvi Joshi
EPI_ISL_437448	B.J. Medical College and Civil hospital	Gujarat Biotechnology Research Centre	Geeta Vaghela, Sonia Barve, Bhavesh Modi, Kairavi Joshi, Gaurishankar Shrimali, Nidhi Sood, Pranay Shah, R D Dixit, Snehal Bagatharia, Kamlesh J Upadhyay, Ramesh Pandit, Tejas Shah, Ankit Hinsu, Pritesh Sabara, Apurvasinh Puvar, Janvi Raval, Monika Gandhi, Pinal Trivedi, Maharshi Pandya, Amit Kanani, Akanksha Verma, Nitin Savaliya, Raghawendra Kumar, Dinesh Kumar, Zuber Saiyed, Dipa Kinariwala, Disha Patel, Binita Aring, Neeta Khandelwal, Afzal Ansari, Chaitanya Joshi, Madhvi Joshi
EPI_ISL_437449	B.J. Medical College and Civil hospital	Gujarat Biotechnology Research Centre	Sonia Barve, Bhavesh Modi, Kairavi Joshi, Gaurishankar Shrimali, Nidhi Sood, Pranay Shah, R D Dixit, Snehal Bagatharia, Kamlesh J Upadhyay, Ramesh Pandit, Tejas Shah, Ankit Hinsu, Pritesh Sabara, Apurvasinh Puvar, Janvi Raval, Monika Gandhi, Pinal Trivedi, Maharshi Pandya, Amit Kanani, Akanksha Verma, Nitin Savaliya, Raghawendra Kumar, Dinesh Kumar, Zuber Saiyed, Dipa Kinariwala, Disha Patel, Binita Aring, Neeta Khandelwal, Geeta Vaghela, Neelam Nathani, Chaitanya Joshi, Madhvi Joshi
EPI_ISL_437450	B.J. Medical College and Civil hospital	Gujarat Biotechnology Research Centre	Bhavesh Modi, Kairavi Joshi, Gaurishankar Shrimali, Nidhi Sood, Pranay Shah, R D Dixit, Snehal Bagatharia, Kamlesh J Upadhyay, Ramesh Pandit, Tejas Shah, Ankit Hinsu, Pritesh Sabara, Apurvasinh Puvar, Janvi Raval, Monika Gandhi, Pinal Trivedi, Maharshi Pandya, Amit Kanani, Akanksha Verma, Nitin Savaliya, Raghawendra Kumar, Dinesh Kumar, Zuber Saiyed, Dipa Kinariwala, Disha Patel, Binita Aring, Neeta Khandelwal, Geeta Vaghela, Sonia Barve, Armi Chaudhari, Chaitanya Joshi, Madhvi Joshi
EPI_ISL_437452	B.J. Medical College and Civil hospital	Gujarat Biotechnology Research Centre	Gaurishankar Shrimali, Nidhi Sood, Pranay Shah, R D Dixit, Snehal Bagatharia, Kamlesh J Upadhyay, Ramesh Pandit, Tejas Shah, Ankit Hinsu, Pritesh Sabara, Apurvasinh Puvar, Janvi Raval, Monika Gandhi, Pinal Trivedi, Maharshi Pandya, Amit Kanani, Akanksha Verma, Nitin Savaliya, Raghawendra Kumar, Dinesh Kumar, Zuber Saiyed, Dipa Kinariwala, Disha Patel, Binita Aring, Neeta Khandelwal, Geeta Vaghela, Sonia Barve, Bhavesh Modi, Kairavi Joshi, Chaitanya Joshi, Anjali Rajwar, Madhvi Joshi
EPI_ISL_437453	B.J. Medical College and Civil hospital	Gujarat Biotechnology Research Centre	Nidhi Sood, Pranay Shah, R D Dixit, Snehal Bagatharia, Kamlesh J Upadhyay, Ramesh Pandit, Tejas Shah, Ankit Hinsu, Pritesh Sabara, Apurvasinh Puvar, Janvi Raval, Monika Gandhi, Pinal Trivedi, Maharshi Pandya, Amit Kanani, Akanksha Verma, Nitin Savaliya, Raghawendra Kumar, Dinesh Kumar, Zuber Saiyed, Dipa Kinariwala, Disha Patel, Binita Aring, Neeta Khandelwal, Geeta Vaghela, Sonia Barve, Bhavesh Modi, Kairavi Joshi, Gaurishankar Shrimali, Chaitanya Joshi, Dipeshwari Shewale, Madhvi Joshi
EPI_ISL_437454	B.J. Medical College and Civil hospital	Gujarat Biotechnology Research Centre	Pranay Shah, R D Dixit, Snehal Bagatharia, Kamlesh J Upadhyay, Ramesh Pandit, Tejas Shah, Ankit Hinsu, Pritesh Sabara, Apurvasinh Puvar, Janvi Raval, Monika Gandhi, Pinal Trivedi, Maharshi Pandya, Amit Kanani, Akanksha Verma, Nitin Savaliya, Raghawendra Kumar, Dinesh Kumar, Zuber Saiyed, Dipa Kinariwala, Disha Patel, Binita Aring, Neeta Khandelwal, Geeta Vaghela, Sonia Barve, Bhavesh Modi, Kairavi Joshi, Gaurishankar Shrimali, Nidhi Sood, Chaitanya Joshi, Sharmistha Majumdar, Madhvi Joshi
EPI_ISL_437459, EPI_ISL_437460, EPI_ISL_437461, EPI_ISL_437462, EPI_ISL_437463, EPI_ISL_437464, EPI_ISL_437465, EPI_ISL_437466, EPI_ISL_437467, EPI_ISL_437468, EPI_ISL_437469, EPI_ISL_437470, EPI_ISL_437471, EPI_ISL_437472, EPI_ISL_437473, EPI_ISL_437474, EPI_ISL_437475, EPI_ISL_437476, EPI_ISL_437477, EPI_ISL_437478, EPI_ISL_437479			
see above	Pathogen Genomics Lab King Abdullah University of Science and Technology(KAUST)	Pathogen Genomics Lab King Abdullah University of Science and Technology(KAUST)	Sharif Hala,Raeece Naeem,Sara Mfarrej,Arnab Pain
EPI_ISL_437481, EPI_ISL_437482, EPI_ISL_437483, EPI_ISL_437484, EPI_ISL_437485, EPI_ISL_437486, EPI_ISL_437487, EPI_ISL_437488, EPI_ISL_437489, EPI_ISL_437490, EPI_ISL_437491, EPI_ISL_437492, EPI_ISL_437493, EPI_ISL_437494, EPI_ISL_437495, EPI_ISL_437496, EPI_ISL_437497			

see above	Pathogen Genomics Lab King Abdullah University of Science and Technology(KAUST)	Pathogen Genomics Lab King Abdullah University of Science and Technology(KAUST)	Sara Mfarrej,Raeece Naeem,Sharif Hala,Amit Subudhi,Fathia Rached,Arnab Pain
EPI_ISL_437626	Department of Microbiology,Gandhi Medical College and Hospital	Department of Veterinary Biotechnalogy, College of Veterinary Science, Rajendranagar, PV Narsimha Rao Telengana Veterinary University	Kalyani Putty, Muttineni Radhakrishna, Nagamani K, Thrilok Chander B, Raja Rao M, Ravikumar P, Sunitha P, Pankaj Singh D, Anand Kumar K, Amit A. Upadhyay, Steven Bosinger, Rama Amara
EPI_ISL_437691, EPI_ISL_437692, EPI_ISL_437693, EPI_ISL_437694, EPI_ISL_437695, EPI_ISL_437696, EPI_ISL_437697, EPI_ISL_437698, EPI_ISL_437699, EPI_ISL_437700, EPI_ISL_437701, EPI_ISL_437702, EPI_ISL_437703, EPI_ISL_437704, EPI_ISL_437705, EPI_ISL_437706, EPI_ISL_437707, EPI_ISL_437708, EPI_ISL_437709, EPI_ISL_437710, EPI_ISL_437711, EPI_ISL_437712, EPI_ISL_437713, EPI_ISL_437714, EPI_ISL_437715, EPI_ISL_437716, EPI_ISL_437717, EPI_ISL_437718, EPI_ISL_437719, EPI_ISL_437720, EPI_ISL_437721, EPI_ISL_437722, EPI_ISL_437723, EPI_ISL_437724, EPI_ISL_437725, EPI_ISL_437726, EPI_ISL_437727, EPI_ISL_437728, EPI_ISL_437729, EPI_ISL_437730, EPI_ISL_437731, EPI_ISL_437732, EPI_ISL_437733, EPI_ISL_437734, EPI_ISL_437735, EPI_ISL_437736, EPI_ISL_437737, EPI_ISL_437738, EPI_ISL_437739, EPI_ISL_437740, EPI_ISL_437741, EPI_ISL_437742, EPI_ISL_437743, EPI_ISL_437744, EPI_ISL_437745, EPI_ISL_437746, EPI_ISL_437747, EPI_ISL_437748, EPI_ISL_437749, EPI_ISL_437750, EPI_ISL_437751, EPI_ISL_437752, EPI_ISL_437753, EPI_ISL_437754, EPI_ISL_437755, EPI_ISL_437756, EPI_ISL_437757, EPI_ISL_437758, EPI_ISL_437759, EPI_ISL_437760, EPI_ISL_437761, EPI_ISL_437762			
see above	Pathogen Genomics Lab King Abdullah University of Science and Technology(KAUST)	Pathogen Genomics Lab King Abdullah University of Science and Technology(KAUST)	Sharif Hala,Fadwa Alofi,Afrah Alsomali, Asim Khogeer, Sara Mfarrej, Khaled Algithami,Raeece Naeem, Amit Kumar Subudhi,Fathia Ben-Rached, Rahul Salunke, Anwar Hashem, Naif Almontashiri, Arnab Pain
EPI_ISL_438138	Department of Microbiology,Gandhi Medical College and Hospital	Department of Microbiology, Gandhi Medical College and Hospital Secendrabad, Hyderbad, India	Raja Rao Mesipogu, Muttineni Radhakrishna, Nagamani K, Thrilok Chander B, Kalyani Putty, Ravikumar P, Sunitha P, Pankaj Singh D, Anand Kumar K, Amit A. Upadhyay, Steven Bosinger, Rama Amara
EPI_ISL_438139	Department of Microbiology,Gandhi Medical College and Hospital,Hyderabad	Virus Research Laboratory, Department of Zoology, Osmania University, Hyderabad, India	Muttineni Radhakrishna, Nagamani K, Thrilok Chander B, Raja Rao M, Kalyani Putty, Ravikumar P, Sunitha P, Pankaj Singh D, Anand Kumar K, Amit A. Upadhyay, Steven Bosinger, Rama Amara
EPI_ISL_442044	Kawsar Human Genetic Research Center	Kawsar Human Genetic Research Center	Mohammad Ali Khosravi, Maryam Abbasalipour Bashash, Sirous Zeinali, Solmaz Sabeghi, Yeganeh Keshvar, Fatemeh Hosseini, Yeganeh Haghdoost
EPI_ISL_442523	Pasteur Institute of Iran	Kawsar Human Genetic Research Company	Sirous Zeinali, Mohammad Ali Khosravi,Maryam Abbasalipour Bashash, Sanaz Mostafavi Jabbari, Maraym Firoozi, Sormeh Pourtavakoli, Elmira Khateri, Razieh Zeinali and Fahimeh Hoseini
EPI_ISL_443258, EPI_ISL_443259	Résidence Ornano	National Reference Center for Viruses of Respiratory Infections, Institut Pasteur, Paris	Mélanie Albert, Marion Barbet, Sylvie Behillil, Méline Bizard, Angela Brisebarre, Flora Donati, Etienne Simon-Lorière, Vincent Enouf, Maud Vanpeene, Sylvie van der Werf
EPI_ISL_443260	LABM GH nord Essonne de Longjumeau - BP 125	National Reference Center for Viruses of Respiratory Infections, Institut Pasteur, Paris	Mélanie Albert, Marion Barbet, Sylvie Behillil, Méline Bizard, Angela Brisebarre, Flora Donati, Etienne Simon-Lorière, Vincent Enouf, Maud Vanpeene, Sylvie van der Werf
EPI_ISL_443261, EPI_ISL_443262, EPI_ISL_443263, EPI_ISL_443264	CHU de Dijon - Laboratoire de Virologie	National Reference Center for Viruses of Respiratory Infections, Institut Pasteur, Paris	Mélanie Albert, Marion Barbet, Sylvie Behillil, Méline Bizard, Angela Brisebarre, Flora Donati, Etienne Simon-Lorière, Vincent Enouf, Maud Vanpeene, Sylvie van der Werf, Jean-Baptiste Bour
EPI_ISL_443265, EPI_ISL_443266, EPI_ISL_443267, EPI_ISL_443268, EPI_ISL_443269, EPI_ISL_443270, EPI_ISL_443271, EPI_ISL_443272, EPI_ISL_443273, EPI_ISL_443274, EPI_ISL_443275, EPI_ISL_443276, EPI_ISL_443277, EPI_ISL_443278, EPI_ISL_443279, EPI_ISL_443280, EPI_ISL_443281, EPI_ISL_443282, EPI_ISL_443283			
see above	CHU - Hôpital Cavale Blanche - Labo. de Virologie	National Reference Center for Viruses of Respiratory Infections, Institut Pasteur, Paris	Mélanie Albert, Marion Barbet, Sylvie Behillil, Méline Bizard, Angela Brisebarre, Flora Donati, Etienne Simon-Lorière, Vincent Enouf, Maud Vanpeene, Sylvie van der Werf, Léa Pilorge
EPI_ISL_443284, EPI_ISL_443285, EPI_ISL_443286, EPI_ISL_443287, EPI_ISL_443288	Laboratoire de Microbiologie - Bât A - CH René Dubois	National Reference Center for Viruses of Respiratory Infections, Institut Pasteur, Paris	Mélanie Albert, Marion Barbet, Sylvie Behillil, Méline Bizard, Angela Brisebarre, Flora Donati, Etienne Simon-Lorière, Vincent Enouf, Maud Vanpeene, Sylvie van der Werf, Pascale Martres
EPI_ISL_443289, EPI_ISL_443290, EPI_ISL_443291, EPI_ISL_443292, EPI_ISL_443293, EPI_ISL_443294	CHRU Pontchaillou - Laboratoire de Virologie	National Reference Center for Viruses of Respiratory Infections, Institut Pasteur, Paris	Mélanie Albert, Marion Barbet, Sylvie Behillil, Méline Bizard, Angela Brisebarre, Flora Donati, Etienne Simon-Lorière, Vincent Enouf, Maud Vanpeene, Sylvie van der Werf, Gisèle Lagathu
EPI_ISL_443295, EPI_ISL_443296, EPI_ISL_443297, EPI_ISL_443298, EPI_ISL_443299	Hôpital Necker - Enfants - Malades Laboratoire de Virologie	National Reference Center for Viruses of Respiratory Infections, Institut Pasteur, Paris	Mélanie Albert, Marion Barbet, Sylvie Behillil, Méline Bizard, Angela Brisebarre, Flora Donati, Etienne Simon-Lorière, Vincent Enouf, Maud Vanpeene, Sylvie van der Werf, Marianne Leruez-Ville
EPI_ISL_443303	Résidence Les Marines	National Reference Center for Viruses of Respiratory Infections, Institut Pasteur, Paris	Mélanie Albert, Marion Barbet, Sylvie Behillil, Méline Bizard, Angela Brisebarre, Flora Donati, Etienne Simon-Lorière, Vincent Enouf, Maud Vanpeene, Sylvie van der Werf
EPI_ISL_443304	Résidence Esterel	National Reference Center for Viruses of Respiratory Infections, Institut Pasteur, Paris	Mélanie Albert, Marion Barbet, Sylvie Behillil, Méline Bizard, Angela Brisebarre, Flora Donati, Etienne Simon-Lorière, Vincent Enouf, Maud Vanpeene, Sylvie van der Werf
EPI_ISL_443305	LABM GH nord Essonne de Longjumeau - BP 125	National Reference Center for Viruses of Respiratory Infections, Institut Pasteur, Paris	Mélanie Albert, Marion Barbet, Sylvie Behillil, Méline Bizard, Angela Brisebarre, Flora Donati, Etienne Simon-Lorière, Vincent Enouf, Maud Vanpeene, Sylvie van der Werf
EPI_ISL_443307	La Villa Papyri	National Reference Center for Viruses of Respiratory Infections, Institut Pasteur, Paris	Mélanie Albert, Marion Barbet, Sylvie Behillil, Méline Bizard, Angela Brisebarre, Flora Donati, Etienne Simon-Lorière, Vincent Enouf, Maud Vanpeene, Sylvie van der Werf
EPI_ISL_443308	Plaisance	National Reference Center for Viruses of Respiratory Infections, Institut Pasteur, Paris	Mélanie Albert, Marion Barbet, Sylvie Behillil, Méline Bizard, Angela Brisebarre, Flora Donati, Etienne Simon-Lorière, Vincent Enouf, Maud Vanpeene, Sylvie van der Werf
EPI_ISL_443309	CH Compiègne Laboratoire de Biologie	National Reference Center for Viruses of Respiratory Infections, Institut Pasteur, Paris	Mélanie Albert, Marion Barbet, Sylvie Behillil, Méline Bizard, Angela Brisebarre, Flora Donati, Etienne Simon-Lorière, Vincent Enouf, Maud Vanpeene, Sylvie van der Werf
EPI_ISL_443314	LABM GH nord Essonne de Longjumeau - BP 125	National Reference Center for Viruses of Respiratory Infections, Institut Pasteur, Paris	Mélanie Albert, Marion Barbet, Sylvie Behillil, Méline Bizard, Angela Brisebarre, Flora Donati, Etienne Simon-Lorière, Vincent Enouf, Maud Vanpeene, Sylvie van der Werf
EPI_ISL_443315	Château de la Source	National Reference Center for Viruses of Respiratory Infections, Institut Pasteur, Paris	Mélanie Albert, Marion Barbet, Sylvie Behillil, Méline Bizard, Angela Brisebarre, Flora Donati, Etienne Simon-Lorière, Vincent Enouf, Maud Vanpeene, Sylvie van der Werf
EPI_ISL_443316	CH Compiègne Laboratoire de Biologie	National Reference Center for Viruses of Respiratory Infections, Institut Pasteur, Paris	Mélanie Albert, Marion Barbet, Sylvie Behillil, Méline Bizard, Angela Brisebarre, Flora Donati, Etienne Simon-Lorière, Vincent Enouf, Maud Vanpeene, Sylvie van der Werf, Olivia Raulin
EPI_ISL_444022	Baylor College of Medicine	Baylor College of Medicine: HGSC	Vasanthi Avadhanula, Erin Nicholson, David Henke, Pedro Piedra, Harsha Doddapaneni, Donna Muzny, Qingchang Meng, Hsu Chao, Zeineen Momin, Hua Shen, George Weissenberger, Kavya Kottapalli, Yimiti Meiheerguli, Sejal Salvi, Ginger Metcalf, Vipin Menon, Sara J.J. Cregeen, Matthew C. Ross, Tulin Ayvaz, Richard Sucgang, Kristi L. Hoffman, Matthew Wong, Joseph F. Petrosino
EPI_ISL_444456	B.J. Medical College and Civil hospital	Gujarat Biotechnology Research Centre	R D Dixit, Snehal Bagatharia, Kamlesh J Upadhyay, Ramesh Pandit, Tejas Shah, Ankit Hinsu, Pritesh Sabara, Apurvasinh Puvar, Janvi Raval, Monika Gandhi, Pinal Trivedi, Maharshi Pandya, Amit Kanani, Akanksha Verma, Nitin Savaliya, Raghawendra Kumar, Dinesh Kumar, Zuber Saiyed, Dipa Kinariwala, Disha Patel, Binita Aring, Neeta Khandelwal, Geeta Vaghela, Sonia Barve, Bhavesh Modi, Kairavi Joshi, Gaurishankar Shrimali, Nidhi Sood, Pranay Shah, Pooja P Doshi, Chaitanya Joshi, Madhvi Joshi
EPI_ISL_444457	B.J. Medical College and Civil hospital	Gujarat Biotechnology Research Centre	Snehal Bagatharia, Kamlesh J Upadhyay, Ramesh Pandit, Tejas Shah, Ankit Hinsu, Pritesh Sabara, Apurvasinh Puvar, Janvi Raval, Monika Gandhi, Pinal Trivedi, Maharshi Pandya, Amit Kanani, Akanksha Verma, Nitin Savaliya, Raghawendra Kumar, Dinesh Kumar, Zuber Saiyed, Dipa Kinariwala, Disha Patel, Binita Aring, Neeta Khandelwal, Geeta Vaghela, Sonia Barve, Bhavesh Modi, Kairavi Joshi, Gaurishankar Shrimali, Nidhi Sood, Pranay Shah, R D Dixit, Nidhi Patel, Chaitanya Joshi, Madhvi Joshi
EPI_ISL_444458	B.J. Medical College and Civil hospital	Gujarat Biotechnology Research Centre	Kamlesh J Upadhyay, Ramesh Pandit, Tejas Shah, Ankit Hinsu, Pritesh Sabara, Apurvasinh Puvar, Janvi Raval, Monika Gandhi, Pinal Trivedi, Maharshi Pandya, Amit Kanani, Akanksha Verma, Nitin Savaliya, Raghawendra Kumar, Dinesh Kumar, Zuber Saiyed, Dipa Kinariwala, Disha Patel, Binita Aring, Neeta Khandelwal, Geeta Vaghela, Sonia Barve, Bhavesh Modi, Kairavi Joshi, Gaurishankar Shrimali, Nidhi Sood, Pranay Shah, R D Dixit, Snehal Bagatharia, Priti Pandita, Chaitanya Joshi, Madhvi Joshi
EPI_ISL_444459	B.J. Medical College and Civil hospital	Gujarat Biotechnology Research Centre	Ramesh Pandit, Tejas Shah, Ankit Hinsu, Pritesh Sabara, Apurvasinh Puvar, Janvi Raval, Monika Gandhi, Pinal Trivedi, Maharshi Pandya, Amit Kanani, Akanksha Verma, Nitin Savaliya, Raghawendra Kumar, Dinesh Kumar, Zuber Saiyed, Dipa Kinariwala, Disha Patel, Binita Aring, Neeta Khandelwal, Geeta Vaghela, Sonia Barve, Bhavesh Modi, Kairavi Joshi, Gaurishankar Shrimali, Nidhi Sood, Pranay Shah, R D Dixit, Snehal Bagatharia, Kamlesh J Upadhyay,

			Neha Rajpara, Chaitanya Joshi, Madhvi Joshi
EPI_ISL_444460	B.J. Medical College and Civil hospital	Gujarat Biotechnology Research Centre	Tejas Shah, Ankit Hinsu, Pritesh Sabara, Apurvasinh Puvar, Janvi Raval, Monika Gandhi, Pinal Trivedi, Maharshi Pandya, Amit Kanani, Akanksha Verma, Nitin Savaliya, Raghawendra Kumar, Dinesh Kumar, Zuber Saiyed, Dipa Kinariwala, Disha Patel, Binita Aring, Neeta Khandelwal, Geeta Vaghela, Sonia Barve, Bhavesh Modi, Kairavi Joshi, Gaurishankar Shrimali, Nidhi Sood, Pranay Shah, R D Dixit, Snehal Bagatharia, Kamlesh J Upadhyay, Ramesh Pandit, Afzal Ansari, Chaitanya Joshi, Madhvi Joshi
EPI_ISL_444462	B.J. Medical College and Civil hospital	Gujarat Biotechnology Research Centre	Pritesh Sabara, Apurvasinh Puvar, Janvi Raval, Monika Gandhi, Pinal Trivedi, Maharshi Pandya, Amit Kanani, Akanksha Verma, Nitin Savaliya, Raghawendra Kumar, Dinesh Kumar, Zuber Saiyed, Dipa Kinariwala, Disha Patel, Binita Aring, Neeta Khandelwal, Geeta Vaghela, Sonia Barve, Bhavesh Modi, Kairavi Joshi, Gaurishankar Shrimali, Nidhi Sood, Pranay Shah, R D Dixit, Snehal Bagatharia, Kamlesh J Upadhyay, Ramesh Pandit, Tejas Shah, Ankit Hinsu, Armi Chaudhari, Chaitanya Joshi, Madhvi Joshi
EPI_ISL_444465	B.J. Medical College and Civil hospital	Gujarat Biotechnology Research Centre	Monika Gandhi, Pinal Trivedi, Maharshi Pandya, Amit Kanani, Akanksha Verma, Nitin Savaliya, Raghawendra Kumar, Dinesh Kumar, Zuber Saiyed, Dipa Kinariwala, Disha Patel, Binita Aring, Neeta Khandelwal, Geeta Vaghela, Sonia Barve, Bhavesh Modi, Kairavi Joshi, Gaurishankar Shrimali, Nidhi Sood, Pranay Shah, R D Dixit, Snehal Bagatharia, Kamlesh J Upadhyay, Ramesh Pandit, Tejas Shah, Ankit Hinsu, Pritesh Sabara, Apurvasinh Puvar, Janvi Raval, Anjali Rajwar, Chaitanya Joshi, Madhvi Joshi
EPI_ISL_444466	B.J. Medical College and Civil hospital	Gujarat Biotechnology Research Centre	Pinal Trivedi, Maharshi Pandya, Amit Kanani, Akanksha Verma, Nitin Savaliya, Raghawendra Kumar, Dinesh Kumar, Zuber Saiyed, Dipa Kinariwala, Disha Patel, Binita Aring, Neeta Khandelwal, Geeta Vaghela, Sonia Barve, Bhavesh Modi, Kairavi Joshi, Gaurishankar Shrimali, Nidhi Sood, Pranay Shah, R D Dixit, Snehal Bagatharia, Kamlesh J Upadhyay, Ramesh Pandit, Tejas Shah, Ankit Hinsu, Pritesh Sabara, Apurvasinh Puvar, Janvi Raval, Monika Gandhi, Sharmistha Majumdar, Chaitanya Joshi, Madhvi Joshi
EPI_ISL_444468	B.J. Medical College and Civil hospital	Gujarat Biotechnology Research Centre	Amit Kanani, Akanksha Verma, Nitin Savaliya, Raghawendra Kumar, Dinesh Kumar, Zuber Saiyed, Dipa Kinariwala, Disha Patel, Binita Aring, Neeta Khandelwal, Geeta Vaghela, Sonia Barve, Bhavesh Modi, Kairavi Joshi, Gaurishankar Shrimali, Nidhi Sood, Pranay Shah, R D Dixit, Snehal Bagatharia, Kamlesh J Upadhyay, Ramesh Pandit, Tejas Shah, Ankit Hinsu, Pritesh Sabara, Apurvasinh Puvar, Janvi Raval, Monika Gandhi, Pinal Trivedi, Maharshi Pandya, Nidhi Patel, Chaitanya Joshi, Madhvi Joshi
EPI_ISL_444469	B.J. Medical College and Civil hospital	Gujarat Biotechnology Research Centre	Akanksha Verma, Nitin Savaliya, Raghawendra Kumar, Dinesh Kumar, Zuber Saiyed, Dipa Kinariwala, Disha Patel, Binita Aring, Neeta Khandelwal, Geeta Vaghela, Sonia Barve, Bhavesh Modi, Kairavi Joshi, Gaurishankar Shrimali, Nidhi Sood, Pranay Shah, R D Dixit, Snehal Bagatharia, Kamlesh J Upadhyay, Ramesh Pandit, Tejas Shah, Ankit Hinsu, Pritesh Sabara, Apurvasinh Puvar, Janvi Raval, Monika Gandhi, Pinal Trivedi, Maharshi Pandya, Amit Kanani, Priti Pandita, Chaitanya Joshi, Madhvi Joshi
EPI_ISL_444471	B.J. Medical College and Civil hospital	Gujarat Biotechnology Research Centre	Raghawendra Kumar, Dinesh Kumar, Zuber Saiyed, Dipa Kinariwala, Disha Patel, Binita Aring, Neeta Khandelwal, Geeta Vaghela, Sonia Barve, Bhavesh Modi, Kairavi Joshi, Gaurishankar Shrimali, Nidhi Sood, Pranay Shah, R D Dixit, Snehal Bagatharia, Kamlesh J Upadhyay, Ramesh Pandit, Tejas Shah, Ankit Hinsu, Pritesh Sabara, Apurvasinh Puvar, Janvi Raval, Monika Gandhi, Pinal Trivedi, Maharshi Pandya, Amit Kanani, Akanksha Verma, Nitin Savaliya, Afzal Ansari, Chaitanya Joshi, Madhvi Joshi
EPI_ISL_444474	B.J. Medical College and Civil hospital	Gujarat Biotechnology Research Centre	Dipa Kinariwala, Disha Patel, Binita Aring, Neeta Khandelwal, Geeta Vaghela, Sonia Barve, Bhavesh Modi, Kairavi Joshi, Gaurishankar Shrimali, Nidhi Sood, Pranay Shah, R D Dixit, Snehal Bagatharia, Kamlesh J Upadhyay, Ramesh Pandit, Tejas Shah, Ankit Hinsu, Pritesh Sabara, Apurvasinh Puvar, Janvi Raval, Monika Gandhi, Pinal Trivedi, Maharshi Pandya, Amit Kanani, Akanksha Verma, Nitin Savaliya, Raghawendra Kumar, Dinesh Kumar, Zuber Saiyed, Bhavya Jindal, Chaitanya Joshi, Madhvi Joshi
EPI_ISL_444475	B.J. Medical College and Civil hospital	Gujarat Biotechnology Research Centre	Disha Patel, Binita Aring, Neeta Khandelwal, Geeta Vaghela, Sonia Barve, Bhavesh Modi, Kairavi Joshi, Gaurishankar Shrimali, Nidhi Sood, Pranay Shah, R D Dixit, Snehal Bagatharia, Kamlesh J Upadhyay, Ramesh Pandit, Tejas Shah, Ankit Hinsu, Pritesh Sabara, Apurvasinh Puvar, Janvi Raval, Monika Gandhi, Pinal Trivedi, Maharshi Pandya, Amit Kanani, Akanksha Verma, Nitin Savaliya, Raghawendra Kumar, Dinesh Kumar, Zuber Saiyed, Dipa Kinariwala, Dipeshwari Shewale, Chaitanya Joshi, Madhvi Joshi
EPI_ISL_444476	B.J. Medical College and Civil hospital	Gujarat Biotechnology Research Centre	Binita Aring, Neeta Khandelwal, Geeta Vaghela, Sonia Barve, Bhavesh Modi, Kairavi Joshi, Gaurishankar Shrimali, Nidhi Sood, Pranay Shah, R D Dixit, Snehal Bagatharia, Kamlesh J Upadhyay, Ramesh Pandit, Tejas Shah, Ankit Hinsu, Pritesh Sabara, Apurvasinh Puvar, Janvi Raval, Monika Gandhi, Pinal Trivedi, Maharshi Pandya, Amit Kanani, Akanksha Verma, Nitin Savaliya, Raghawendra Kumar, Dinesh Kumar, Zuber Saiyed, Dipa Kinariwala, Disha Patel, Chaitanya Joshi, Madhvi Joshi, Dipeshwari Shewale
EPI_ISL_444477	B.J. Medical College and Civil hospital	Gujarat Biotechnology Research Centre	Neeta Khandelwal, Geeta Vaghela, Sonia Barve, Bhavesh Modi, Kairavi Joshi, Gaurishankar Shrimali, Nidhi Sood, Pranay Shah, R D Dixit, Snehal Bagatharia, Kamlesh J Upadhyay, Ramesh Pandit, Tejas Shah, Ankit Hinsu, Pritesh Sabara, Apurvasinh Puvar, Janvi Raval, Monika Gandhi, Pinal Trivedi, Maharshi Pandya, Amit Kanani, Akanksha Verma, Nitin Savaliya, Raghawendra Kumar, Dinesh Kumar, Zuber Saiyed, Dipa Kinariwala, Disha Patel, Binita Aring, Sharmistha Majumdar, Chaitanya Joshi, Madhvi Joshi
EPI_ISL_444480	B.J. Medical College and Civil hospital	Gujarat Biotechnology Research Centre	Bhavesh Modi, Kairavi Joshi, Gaurishankar Shrimali, Nidhi Sood, Pranay Shah, R D Dixit, Snehal Bagatharia, Kamlesh J Upadhyay, Ramesh Pandit, Tejas Shah, Ankit Hinsu, Pritesh Sabara, Apurvasinh Puvar, Janvi Raval, Monika Gandhi, Pinal Trivedi, Maharshi Pandya, Amit Kanani, Akanksha Verma, Nitin Savaliya, Raghawendra Kumar, Dinesh Kumar, Zuber Saiyed, Dipa Kinariwala, Disha Patel, Binita Aring, Neeta Khandelwal, Geeta Vaghela, Sonia Barve, Priti Pandita, Chaitanya Joshi, Madhvi Joshi
EPI_ISL_444481	B.J. Medical College and Civil hospital	Gujarat Biotechnology Research Centre	Kairavi Joshi, Gaurishankar Shrimali, Nidhi Sood, Pranay Shah, R D Dixit, Snehal Bagatharia, Kamlesh J Upadhyay, Ramesh Pandit, Tejas Shah, Ankit Hinsu, Pritesh Sabara, Apurvasinh Puvar, Janvi Raval, Monika Gandhi, Pinal Trivedi, Maharshi Pandya, Amit Kanani, Akanksha Verma, Nitin Savaliya, Raghawendra Kumar, Dinesh Kumar, Zuber Saiyed, Dipa Kinariwala, Disha Patel, Binita Aring, Neeta Khandelwal, Geeta Vaghela, Sonia Barve, Bhavesh Modi, Neha Rajpara, Chaitanya Joshi, Madhvi Joshi
EPI_ISL_444482	Gujarat Biotechnology Research Centre	Gujarat Biotechnology Research Centre	Gaurishankar Shrimali, Nidhi Sood, Pranay Shah, R D Dixit, Snehal Bagatharia, Kamlesh J Upadhyay, Ramesh Pandit, Tejas Shah, Ankit Hinsu, Pritesh Sabara, Apurvasinh Puvar, Janvi Raval, Monika Gandhi, Pinal Trivedi, Maharshi Pandya, Amit Kanani, Akanksha Verma, Nitin Savaliya, Raghawendra Kumar, Dinesh Kumar, Zuber Saiyed, Dipa Kinariwala, Disha Patel, Binita Aring, Neeta Khandelwal, Geeta Vaghela, Sonia Barve, Bhavesh Modi, Kairavi Joshi, Afzal Ansari, Chaitanya Joshi, Madhvi Joshi
EPI_ISL_444483	Gujarat Biotechnology Research Centre	Gujarat Biotechnology Research Centre	Nidhi Sood, Pranay Shah, R D Dixit, Snehal Bagatharia, Kamlesh J Upadhyay, Ramesh Pandit, Tejas Shah, Ankit Hinsu, Pritesh Sabara, Apurvasinh Puvar, Janvi Raval, Monika Gandhi, Pinal Trivedi, Maharshi Pandya, Amit Kanani, Akanksha Verma, Nitin Savaliya, Raghawendra Kumar, Dinesh Kumar, Zuber Saiyed, Dipa Kinariwala, Disha Patel, Binita Aring, Neeta Khandelwal, Geeta Vaghela, Sonia Barve, Bhavesh Modi, Kairavi Joshi, Gaurishankar Shrimali, Neelam Nathani, Chaitanya Joshi, Madhvi Joshi
EPI_ISL_444484	Gujarat Biotechnology Research Centre	Gujarat Biotechnology Research Centre	Pranay Shah, R D Dixit, Snehal Bagatharia, Kamlesh J Upadhyay, Ramesh Pandit, Tejas Shah, Ankit Hinsu, Pritesh Sabara, Apurvasinh Puvar, Janvi Raval, Monika Gandhi, Pinal Trivedi, Maharshi Pandya, Amit Kanani, Akanksha Verma, Nitin Savaliya, Raghawendra Kumar, Dinesh Kumar, Zuber Saiyed, Dipa Kinariwala, Disha Patel, Binita Aring, Neeta Khandelwal, Geeta Vaghela, Sonia Barve, Bhavesh Modi, Kairavi Joshi, Gaurishankar Shrimali, Nidhi Sood, Armi Chaudhari, Chaitanya Joshi, Madhvi Joshi
EPI_ISL_444486	Gujarat Biotechnology Research Centre	Gujarat Biotechnology Research Centre	Snehal Bagatharia, Kamlesh J Upadhyay, Ramesh Pandit, Tejas Shah, Ankit Hinsu, Pritesh Sabara, Apurvasinh Puvar, Janvi Raval, Monika Gandhi, Pinal Trivedi, Maharshi Pandya, Amit Kanani, Akanksha Verma, Nitin Savaliya, Raghawendra Kumar, Dinesh Kumar, Zuber Saiyed, Dipa Kinariwala, Disha Patel, Binita Aring, Neeta Khandelwal, Geeta Vaghela, Sonia Barve, Bhavesh Modi, Kairavi Joshi, Gaurishankar Shrimali, Nidhi Sood, Pranay Shah, R D Dixit, Dipeshwari Shewale, Chaitanya Joshi, Madhvi Joshi
EPI_ISL_444493	Departamento de Laboratorios de Salud Publica (DLSP, Division Epidemiologia, Ministerio de Salud Publica)	Facultad de Ciencias (Sección Genética Evolutiva, Sección Virología).	Panzera,Y., Delfraro,A., Ramos,N., Frabasile,S., Calleros,L., Techera,C., Grecco,S., Fuques,E., Goni,N., Coppola,L., Ramos,V., Chiparelli,H., Arbiza,J. and Perez,R.
EPI_ISL_444969	Guangzhou Eighth People's Hospital (Jiahe Sector)	Institute of Human Virology, Zhongshan School of Medicine, Sun Yat-sen University	Junsong Zhang, Fei Yu, Jun Liu, Huimin Fan, Ruosu Ying, Feng Huang, Ting Pan, Bingfeng Liu,Yiwen Zhang, Xu Zhang, Mang Shi, Fengyu Hu, Fang Li, Kai Deng, Hui Zhang
EPI_ISL_444994, EPI_ISL_444995, EPI_ISL_444996, EPI_ISL_444998, EPI_ISL_445000	Naval Health Research Center	Naval Medical Research Center Biological Defense Research Directorate	Logan Voegtly, Regina Cer, Dessiree Pena-Gomez, Adrian Paskey,Kyle Long, Roger Pan, Melinda Balansay-Ames, Chris Myers, Ewell Hollis, Nathaniel Christy, Kimberly Bishop-Lilly
EPI_ISL_445078, EPI_ISL_445079,	Baylor College of Medicine	Baylor College of Medicine: HGSC	Vasanthi Avadhanula, Erin Nicholson, David Henke, Pedro Piedra, Harsha Doddapaneni, Donna Muzny, Qingchang Meng, Hsu Chao, Zeineen Momin, Hua

EPI_ISL_445080, EPI_ISL_445081, EPI_ISL_445082, EPI_ISL_445083, EPI_ISL_445084			Shen, George Weissenberger, Kavya Kottapalli, Yimiti Meiheerguli, Sejal Salvi, Ginger Metcalf, Vipin Menon, Sara J.J. Cregeen, Matthew C. Ross, Tulin Ayvaz, Richard Sucgang, Kristi L. Hoffman, Matthew Wong, Joseph F. Petrosino
EPI_ISL_445213	DNA Solution Ltd	DNA Solution Ltd	Md. Imran Khan, Kazi Nadim Hasan, Abu Sufian, Mohammed Nafiz Imtiaz Polol, Abdul Khaleque, Mizanur Rahman, MSM Chowdhury, Hasan Ul Haider, Mamudul Hasan Razu, Mala Khan, Mohammad Fazle Alam Rabbi
EPI_ISL_445214, EPI_ISL_445215, EPI_ISL_445216, EPI_ISL_445217	DNA Solution Ltd.	DNA Solution Ltd.	Md. Imran Khan, Kazi Nadim Hasan, Abu Sufian, Mohammed Nafiz Imtiaz Polol, Abdul Khaleque, Mizanur Rahman, MSM Chowdhury, Hasan Ul Haider, Mamudul Hasan Razu, Mala Khan, Mohammad Fazle Alam Rabbi
EPI_ISL_445219	Universidad del Valle, Laboratorio de Microbiologia, VIREM	Universidad del Valle, Universidad Nacional de Colombia-Sede Palmira, International Center for Tropical Agriculture	Beatriz Parra, Diana López-Alvarez, Wilmer J. Cuellar
EPI_ISL_445220	Laborarory for Respiratory Viruses, "Cantacuzino" National Military-Medical Institute for Resararch and Development	Cantacuzino Institute	M.Lazar, L.Ustea, A.Cretu
EPI_ISL_445243	Laboratory for Respiratory Viruses, Cantacuzino National Military-Medical Institute for Research and Development	Cantacuzino Institute	M.Lazar, L.Ustea, A.Cretu
EPI_ISL_447030	B.J. Medical College and Civil hospital	Gujarat Biotechnology Research Centre	Kamlesh J Upadhyay, Ramesh Pandit, Tejas Shah, Ankit Hinsu, Pritesh Sabara, Apurvasinh Puvar, Janvi Raval, Monika Gandhi, Pinal Trivedi, Maharshi Pandya, Amit Kanani, Akanksha Verma, Nitin Savaliya, Raghawendra Kumar, Dinesh Kumar, Zuber Saiyed, Dipa Kinariwala, Disha Patel, Binita Aring, Neeta Khandelwal, Geeta Vaghela, Sonia Barve, Bhavesh Modi, Kairavi Joshi, Gaurishankar Shrimali, Nidhi Sood, Pranay Shah, R D Dixit, Snehal Bagatharia, Anjali Rajwar, Chaitanya Joshi, Madhvi Joshi
EPI_ISL_447031	B.J. Medical College and Civil hospital	Gujarat Biotechnology Research Centre	Ramesh Pandit, Tejas Shah, Ankit Hinsu, Pritesh Sabara, Apurvasinh Puvar, Janvi Raval, Monika Gandhi, Pinal Trivedi, Maharshi Pandya, Amit Kanani, Akanksha Verma, Nitin Savaliya, Raghawendra Kumar, Dinesh Kumar, Zuber Saiyed, Dipa Kinariwala, Disha Patel, Binita Aring, Neeta Khandelwal, Geeta Vaghela, Sonia Barve, Bhavesh Modi, Kairavi Joshi, Gaurishankar Shrimali, Nidhi Sood, Pranay Shah, R D Dixit, Snehal Bagatharia, Kamlesh J Upadhyay, Sharmistha Majumdar, Chaitanya Joshi, Madhvi Joshi
EPI_ISL_447033	B.J. Medical College and Civil hospital	Gujarat Biotechnology Research Centre	Ankit Hinsu, Pritesh Sabara, Apurvasinh Puvar, Janvi Raval, Monika Gandhi, Pinal Trivedi, Maharshi Pandya, Amit Kanani, Akanksha Verma, Nitin Savaliya, Raghawendra Kumar, Dinesh Kumar, Zuber Saiyed, Dipa Kinariwala, Disha Patel, Binita Aring, Neeta Khandelwal, Geeta Vaghela, Sonia Barve, Bhavesh Modi, Kairavi Joshi, Gaurishankar Shrimali, Nidhi Sood, Pranay Shah, R D Dixit, Snehal Bagatharia, Kamlesh J Upadhyay, Ramesh Pandit, Tejas Shah, Nidhi Patel, Chaitanya Joshi, Madhvi Joshi
EPI_ISL_447034	B.J. Medical College and Civil hospital	Gujarat Biotechnology Research Centre	Pritesh Sabara, Apurvasinh Puvar, Janvi Raval, Monika Gandhi, Pinal Trivedi, Maharshi Pandya, Amit Kanani, Akanksha Verma, Nitin Savaliya, Raghawendra Kumar, Dinesh Kumar, Zuber Saiyed, Dipa Kinariwala, Disha Patel, Binita Aring, Neeta Khandelwal, Geeta Vaghela, Sonia Barve, Bhavesh Modi, Kairavi Joshi, Gaurishankar Shrimali, Nidhi Sood, Pranay Shah, R D Dixit, Snehal Bagatharia, Kamlesh J Upadhyay, Ramesh Pandit, Tejas Shah, Ankit Hinsu, Priti Pandita, Chaitanya Joshi, Madhvi Joshi
EPI_ISL_447035	B.J. Medical College and Civil hospital	Gujarat Biotechnology Research Centre	Apurvasinh Puvar, Janvi Raval, Monika Gandhi, Pinal Trivedi, Maharshi Pandya, Amit Kanani, Akanksha Verma, Nitin Savaliya, Raghawendra Kumar, Dinesh Kumar, Zuber Saiyed, Dipa Kinariwala, Disha Patel, Binita Aring, Neeta Khandelwal, Geeta Vaghela, Sonia Barve, Bhavesh Modi, Kairavi Joshi, Gaurishankar Shrimali, Nidhi Sood, Pranay Shah, R D Dixit, Snehal Bagatharia, Kamlesh J Upadhyay, Ramesh Pandit, Tejas Shah, Ankit Hinsu, Pritesh Sabara, Neha Rajpara, Chaitanya Joshi, Madhvi Joshi
EPI_ISL_447038	B.J. Medical College and Civil hospital	Gujarat Biotechnology Research Centre	Pinal Trivedi, Maharshi Pandya, Amit Kanani, Akanksha Verma, Nitin Savaliya, Raghawendra Kumar, Dinesh Kumar, Zuber Saiyed, Dipa Kinariwala, Disha Patel, Binita Aring, Neeta Khandelwal, Geeta Vaghela, Sonia Barve, Bhavesh Modi, Kairavi Joshi, Gaurishankar Shrimali, Nidhi Sood, Pranay Shah, R D Dixit, Snehal Bagatharia, Kamlesh J Upadhyay, Ramesh Pandit, Tejas Shah, Ankit Hinsu, Pritesh Sabara, Apurvasinh Puvar, Janvi Raval, Monika Gandhi, Armi Chaudhari, Chaitanya Joshi, Madhvi Joshi
EPI_ISL_447039	B.J. Medical College and Civil hospital	Gujarat Biotechnology Research Centre	Maharshi Pandya, Amit Kanani, Akanksha Verma, Nitin Savaliya, Raghawendra Kumar, Dinesh Kumar, Zuber Saiyed, Dipa Kinariwala, Disha Patel, Binita Aring, Neeta Khandelwal, Geeta Vaghela, Sonia Barve, Bhavesh Modi, Kairavi Joshi, Gaurishankar Shrimali, Nidhi Sood, Pranay Shah, R D Dixit, Snehal Bagatharia, Kamlesh J Upadhyay, Ramesh Pandit, Tejas Shah, Ankit Hinsu, Pritesh Sabara, Apurvasinh Puvar, Janvi Raval, Monika Gandhi, Pinal Trivedi, Bhavya Jindal, Chaitanya Joshi, Madhvi Joshi
EPI_ISL_447042	B.J. Medical College and Civil hospital	Gujarat Biotechnology Research Centre	Nitin Savaliya, Raghawendra Kumar, Dinesh Kumar, Zuber Saiyed, Dipa Kinariwala, Disha Patel, Binita Aring, Neeta Khandelwal, Geeta Vaghela, Sonia Barve, Bhavesh Modi, Kairavi Joshi, Gaurishankar Shrimali, Nidhi Sood, Pranay Shah, R D Dixit, Snehal Bagatharia, Kamlesh J Upadhyay, Ramesh Pandit, Tejas Shah, Ankit Hinsu, Pritesh Sabara, Apurvasinh Puvar, Janvi Raval, Monika Gandhi, Pinal Trivedi, Maharshi Pandya, Amit Kanani, Akanksha Verma, Pooja P Doshi, Chaitanya Joshi, Madhvi Joshi
EPI_ISL_447043	B.J. Medical College and Civil hospital	Gujarat Biotechnology Research Centre	Raghawendra Kumar, Dinesh Kumar, Zuber Saiyed, Dipa Kinariwala, Disha Patel, Binita Aring, Neeta Khandelwal, Geeta Vaghela, Sonia Barve, Bhavesh Modi, Kairavi Joshi, Gaurishankar Shrimali, Nidhi Sood, Pranay Shah, R D Dixit, Snehal Bagatharia, Kamlesh J Upadhyay, Ramesh Pandit, Tejas Shah, Ankit Hinsu, Pritesh Sabara, Apurvasinh Puvar, Janvi Raval, Monika Gandhi, Pinal Trivedi, Maharshi Pandya, Amit Kanani, Akanksha Verma, Nitin Savaliya, Nidhi Patel, Chaitanya Joshi, Madhvi Joshi
EPI_ISL_447044	B.J. Medical College and Civil hospital	Gujarat Biotechnology Research Centre	Dinesh Kumar, Zuber Saiyed, Dipa Kinariwala, Disha Patel, Binita Aring, Neeta Khandelwal, Geeta Vaghela, Sonia Barve, Bhavesh Modi, Kairavi Joshi, Gaurishankar Shrimali, Nidhi Sood, Pranay Shah, R D Dixit, Snehal Bagatharia, Kamlesh J Upadhyay, Ramesh Pandit, Tejas Shah, Ankit Hinsu, Pritesh Sabara, Apurvasinh Puvar, Janvi Raval, Monika Gandhi, Pinal Trivedi, Maharshi Pandya, Amit Kanani, Akanksha Verma, Nitin Savaliya, Raghawendra Kumar, Priti Pandita, Chaitanya Joshi, Madhvi Joshi
EPI_ISL_447045	B.J. Medical College and Civil hospital	Gujarat Biotechnology Research Centre	Zuber Saiyed, Dipa Kinariwala, Disha Patel, Binita Aring, Neeta Khandelwal, Geeta Vaghela, Sonia Barve, Bhavesh Modi, Kairavi Joshi, Gaurishankar Shrimali, Nidhi Sood, Pranay Shah, R D Dixit, Snehal Bagatharia, Kamlesh J Upadhyay, Ramesh Pandit, Tejas Shah, Ankit Hinsu, Pritesh Sabara, Apurvasinh Puvar, Janvi Raval, Monika Gandhi, Pinal Trivedi, Maharshi Pandya, Amit Kanani, Akanksha Verma, Nitin Savaliya, Raghawendra Kumar, Dinesh Kumar, Neha Rajpara, Chaitanya Joshi, Madhvi Joshi
EPI_ISL_447048	GMERS Medical College and Hospital, Gandhinagar	Gujarat Biotechnology Research Centre	Binita Aring, Neeta Khandelwal, Geeta Vaghela, Sonia Barve, Bhavesh Modi, Kairavi Joshi, Gaurishankar Shrimali, Nidhi Sood, Pranay Shah, R D Dixit, Snehal Bagatharia, Kamlesh J Upadhyay, Ramesh Pandit, Tejas Shah, Ankit Hinsu, Pritesh Sabara, Apurvasinh Puvar, Janvi Raval, Monika Gandhi, Pinal Trivedi, Maharshi Pandya, Amit Kanani, Akanksha Verma, Nitin Savaliya, Raghawendra Kumar, Dinesh Kumar, Zuber Saiyed, Dipa Kinariwala, Disha Patel, Armi Chaudhari, Chaitanya Joshi, Madhvi Joshi
EPI_ISL_447053	GMERS Medical College and Hospital, Gandhinagar	Gujarat Biotechnology Research Centre	Kairavi Joshi, Gaurishankar Shrimali, Nidhi Sood, Pranay Shah, R D Dixit, Snehal Bagatharia, Kamlesh J Upadhyay, Ramesh Pandit, Tejas Shah, Ankit Hinsu, Pritesh Sabara, Apurvasinh Puvar, Janvi Raval, Monika Gandhi, Pinal Trivedi, Maharshi Pandya, Amit Kanani, Akanksha Verma, Nitin Savaliya, Raghawendra Kumar, Dinesh Kumar, Zuber Saiyed, Dipa Kinariwala, Disha Patel, Binita Aring, Neeta Khandelwal, Geeta Vaghela, Sonia Barve, Bhavesh Modi, Pooja P Doshi, Chaitanya Joshi, Madhvi Joshi
EPI_ISL_447054	Cantacuzino National Military-Medical Institute for Research and Development	Cantacuzino Institute	M.Lazar, L.Ustea, A.Cretu
EPI_ISL_447250, EPI_ISL_447251	Central Virology Laboratory	Central Virology Laboratory	Neta Zuckerman, Efrat Bucris, Oran Erster, Danit Sofer, Orna Mor, Ella Mendelson, Michal Mandelboim
EPI_ISL_447252, EPI_ISL_447253, EPI_ISL_447254, EPI_ISL_447255, EPI_ISL_447256, EPI_ISL_447257	TSGH-CP molecular lab	TSGH-CP molecular lab	Cherng-Lih Perng, Ming-Jr JIAN, Chih-Kai Chang, Jung-Chung Lin, Kuo-Ming Yeh, Chien-Wen Chen, Sheng-Kang Chiu, Hsing-Yi Chung, Shih-Hung Tsai, Kuo-Sheng Hung, Tien-Yao Chang, Feng-Yee Chang, Hung-Sheng Shang
EPI_ISL_447281, EPI_ISL_447282, EPI_ISL_447283, EPI_ISL_447284, EPI_ISL_447285, EPI_ISL_447286, EPI_ISL_447287, EPI_ISL_447288, EPI_ISL_447289, EPI_ISL_447290, EPI_ISL_447291, EPI_ISL_447292, EPI_ISL_447293, EPI_ISL_447294, EPI_ISL_447295, EPI_ISL_447296, EPI_ISL_447297, EPI_ISL_447298, EPI_ISL_447299, EPI_ISL_447300, EPI_ISL_447301, EPI_ISL_447302, EPI_ISL_447303, EPI_ISL_447304, EPI_ISL_447305, EPI_ISL_447306, EPI_ISL_447307, EPI_ISL_447308, EPI_ISL_447309, EPI_ISL_447310			
see above	Microbiology Division, Barzilai University Medical Center	Stern Lab	Stern Lab

EPI_ISL_447534	Gujarat Biotechnology Research Centre	Gujarat Biotechnology Research Centre	Gaurishankar Shrimali, Nidhi Sood, Pranay Shah, R D Dixit, Snehal Bagatharia, Kamlesh J Upadhyay, Ramesh Pandit, Tejas Shah, Ankit Hinsu, Pritesh Sabara, Apurvasinh Puvar, Janvi Raval, Monika Gandhi, Pinal Trivedi, Maharshi Pandya, Amit Kanani, Akanksha Verma, Nitin Savaliya, Raghawendra Kumar, Dinesh Kumar, Zuber Saiyed, Dipa Kinariwala, Disha Patel, Binita Aring, Neeta Khandelwal, Geeta Vaghela, Sonia Barve, Bhavesh Modi, Kairavi Joshi, Nidhi Patel, Chaitanya Joshi, Madhvi Joshi
EPI_ISL_447535	Gujarat Biotechnology Research Centre	Gujarat Biotechnology Research Centre	Nidhi Sood, Pranay Shah, R D Dixit, Snehal Bagatharia, Kamlesh J Upadhyay, Ramesh Pandit, Tejas Shah, Ankit Hinsu, Pritesh Sabara, Apurvasinh Puvar, Janvi Raval, Monika Gandhi, Pinal Trivedi, Maharshi Pandya, Amit Kanani, Akanksha Verma, Nitin Savaliya, Raghawendra Kumar, Dinesh Kumar, Zuber Saiyed, Dipa Kinariwala, Disha Patel, Binita Aring, Neeta Khandelwal, Geeta Vaghela, Sonia Barve, Bhavesh Modi, Kairavi Joshi, Gaurishankar Shrimali, Priti Pandita, Chaitanya Joshi, Madhvi Joshi
EPI_ISL_447536	Gujarat Biotechnology Research Centre	Gujarat Biotechnology Research Centre	Pranay Shah, R D Dixit, Snehal Bagatharia, Kamlesh J Upadhyay, Ramesh Pandit, Tejas Shah, Ankit Hinsu, Pritesh Sabara, Apurvasinh Puvar, Janvi Raval, Monika Gandhi, Pinal Trivedi, Maharshi Pandya, Amit Kanani, Akanksha Verma, Nitin Savaliya, Raghawendra Kumar, Dinesh Kumar, Zuber Saiyed, Dipa Kinariwala, Disha Patel, Binita Aring, Neeta Khandelwal, Geeta Vaghela, Sonia Barve, Bhavesh Modi, Kairavi Joshi, Gaurishankar Shrimali, Nidhi Sood, Neha Rajpara, Chaitanya Joshi, Madhvi Joshi
EPI_ISL_447537	Gujarat Biotechnology Research Centre	Gujarat Biotechnology Research Centre	R D Dixit, Snehal Bagatharia, Kamlesh J Upadhyay, Ramesh Pandit, Tejas Shah, Ankit Hinsu, Pritesh Sabara, Apurvasinh Puvar, Janvi Raval, Monika Gandhi, Pinal Trivedi, Maharshi Pandya, Amit Kanani, Akanksha Verma, Nitin Savaliya, Raghawendra Kumar, Dinesh Kumar, Zuber Saiyed, Dipa Kinariwala, Disha Patel, Binita Aring, Neeta Khandelwal, Geeta Vaghela, Sonia Barve, Bhavesh Modi, Kairavi Joshi, Gaurishankar Shrimali, Nidhi Sood, Pranay Shah, Afzal Ansari, Chaitanya Joshi, Madhvi Joshi
EPI_ISL_447542	Gujarat Biotechnology Research Centre	Gujarat Biotechnology Research Centre	Ankit Hinsu, Pritesh Sabara, Apurvasinh Puvar, Janvi Raval, Monika Gandhi, Pinal Trivedi, Maharshi Pandya, Amit Kanani, Akanksha Verma, Nitin Savaliya, Raghawendra Kumar, Dinesh Kumar, Zuber Saiyed, Dipa Kinariwala, Disha Patel, Binita Aring, Neeta Khandelwal, Geeta Vaghela, Sonia Barve, Bhavesh Modi, Kairavi Joshi, Gaurishankar Shrimali, Nidhi Sood, Pranay Shah, R D Dixit, Snehal Bagatharia, Kamlesh J Upadhyay, Ramesh Pandit, Tejas Shah, Dipeshwari Shewale, Chaitanya Joshi, Madhvi Joshi
EPI_ISL_447543	Gujarat Biotechnology Research Centre	Gujarat Biotechnology Research Centre	Pritesh Sabara, Apurvasinh Puvar, Janvi Raval, Monika Gandhi, Pinal Trivedi, Maharshi Pandya, Amit Kanani, Akanksha Verma, Nitin Savaliya, Raghawendra Kumar, Dinesh Kumar, Zuber Saiyed, Dipa Kinariwala, Disha Patel, Binita Aring, Neeta Khandelwal, Geeta Vaghela, Sonia Barve, Bhavesh Modi, Kairavi Joshi, Gaurishankar Shrimali, Nidhi Sood, Pranay Shah, R D Dixit, Snehal Bagatharia, Kamlesh J Upadhyay, Ramesh Pandit, Tejas Shah, Ankit Hinsu, Sharmistha Majumdar, Chaitanya Joshi, Madhvi Joshi
EPI_ISL_447544	Gujarat Biotechnology Research Centre	Gujarat Biotechnology Research Centre	Apurvasinh Puvar, Janvi Raval, Monika Gandhi, Pinal Trivedi, Maharshi Pandya, Amit Kanani, Akanksha Verma, Nitin Savaliya, Raghawendra Kumar, Dinesh Kumar, Zuber Saiyed, Dipa Kinariwala, Disha Patel, Binita Aring, Neeta Khandelwal, Geeta Vaghela, Sonia Barve, Bhavesh Modi, Kairavi Joshi, Gaurishankar Shrimali, Nidhi Sood, Pranay Shah, R D Dixit, Snehal Bagatharia, Kamlesh J Upadhyay, Ramesh Pandit, Tejas Shah, Ankit Hinsu, Pritesh Sabara, Pooja P Doshi, Chaitanya Joshi, Madhvi Joshi
EPI_ISL_447545	Gujarat Biotechnology Research Centre	Gujarat Biotechnology Research Centre	Janvi Raval, Monika Gandhi, Pinal Trivedi, Maharshi Pandya, Amit Kanani, Akanksha Verma, Nitin Savaliya, Raghawendra Kumar, Dinesh Kumar, Zuber Saiyed, Dipa Kinariwala, Disha Patel, Binita Aring, Neeta Khandelwal, Geeta Vaghela, Sonia Barve, Bhavesh Modi, Kairavi Joshi, Gaurishankar Shrimali, Nidhi Sood, Pranay Shah, R D Dixit, Snehal Bagatharia, Kamlesh J Upadhyay, Ramesh Pandit, Tejas Shah, Ankit Hinsu, Pritesh Sabara, Apurvasinh Puvar, Nidhi Patel, Chaitanya Joshi, Madhvi Joshi
EPI_ISL_447546	Gujarat Biotechnology Research Centre	Gujarat Biotechnology Research Centre	Monika Gandhi, Pinal Trivedi, Maharshi Pandya, Amit Kanani, Akanksha Verma, Nitin Savaliya, Raghawendra Kumar, Dinesh Kumar, Zuber Saiyed, Dipa Kinariwala, Disha Patel, Binita Aring, Neeta Khandelwal, Geeta Vaghela, Sonia Barve, Bhavesh Modi, Kairavi Joshi, Gaurishankar Shrimali, Nidhi Sood, Pranay Shah, R D Dixit, Snehal Bagatharia, Kamlesh J Upadhyay, Ramesh Pandit, Tejas Shah, Ankit Hinsu, Pritesh Sabara, Apurvasinh Puvar, Janvi Raval, Priti Pandita, Chaitanya Joshi, Madhvi Joshi
EPI_ISL_447548	GMERS Medical College and Hospital, Gandhinagar	Gujarat Biotechnology Research Centre	Maharshi Pandya, Amit Kanani, Akanksha Verma, Nitin Savaliya, Raghawendra Kumar, Dinesh Kumar, Zuber Saiyed, Dipa Kinariwala, Disha Patel, Binita Aring, Neeta Khandelwal, Geeta Vaghela, Sonia Barve, Bhavesh Modi, Kairavi Joshi, Gaurishankar Shrimali, Nidhi Sood, Pranay Shah, R D Dixit, Snehal Bagatharia, Kamlesh J Upadhyay, Ramesh Pandit, Tejas Shah, Ankit Hinsu, Pritesh Sabara, Apurvasinh Puvar, Janvi Raval, Monika Gandhi, Pinal Trivedi, Afzal Ansari, Chaitanya Joshi, Madhvi Joshi
EPI_ISL_447551	GMERS Medical College and Hospital, Gandhinagar	Gujarat Biotechnology Research Centre	Nitin Savaliya, Raghawendra Kumar, Dinesh Kumar, Zuber Saiyed, Dipa Kinariwala, Disha Patel, Binita Aring, Neeta Khandelwal, Geeta Vaghela, Sonia Barve, Bhavesh Modi, Kairavi Joshi, Gaurishankar Shrimali, Nidhi Sood, Pranay Shah, R D Dixit, Snehal Bagatharia, Kamlesh J Upadhyay, Ramesh Pandit, Tejas Shah, Ankit Hinsu, Pritesh Sabara, Apurvasinh Puvar, Janvi Raval, Monika Gandhi, Pinal Trivedi, Maharshi Pandya, Amit Kanani, Akanksha Verma, Bhavya Jindal, Chaitanya Joshi, Madhvi Joshi
EPI_ISL_447552	GMERS Medical College and Hospital, Gandhinagar	Gujarat Biotechnology Research Centre	Raghawendra Kumar, Dinesh Kumar, Zuber Saiyed, Dipa Kinariwala, Disha Patel, Binita Aring, Neeta Khandelwal, Geeta Vaghela, Sonia Barve, Bhavesh Modi, Kairavi Joshi, Gaurishankar Shrimali, Nidhi Sood, Pranay Shah, R D Dixit, Snehal Bagatharia, Kamlesh J Upadhyay, Ramesh Pandit, Tejas Shah, Ankit Hinsu, Pritesh Sabara, Apurvasinh Puvar, Janvi Raval, Monika Gandhi, Pinal Trivedi, Maharshi Pandya, Amit Kanani, Akanksha Verma, Nitin Savaliya, Anjali Rajwar, Chaitanya Joshi, Madhvi Joshi
EPI_ISL_447553	GMERS Medical College and Hospital, Gandhinagar	Gujarat Biotechnology Research Centre	Dinesh Kumar, Zuber Saiyed, Dipa Kinariwala, Disha Patel, Binita Aring, Neeta Khandelwal, Geeta Vaghela, Sonia Barve, Bhavesh Modi, Kairavi Joshi, Gaurishankar Shrimali, Nidhi Sood, Pranay Shah, R D Dixit, Snehal Bagatharia, Kamlesh J Upadhyay, Ramesh Pandit, Tejas Shah, Ankit Hinsu, Pritesh Sabara, Apurvasinh Puvar, Janvi Raval, Monika Gandhi, Pinal Trivedi, Maharshi Pandya, Amit Kanani, Akanksha Verma, Nitin Savaliya, Raghawendra Kumar, Dipeshwari Shewale, Chaitanya Joshi, Madhvi Joshi
EPI_ISL_447554	GMERS Medical College and Hospital, Gandhinagar	Gujarat Biotechnology Research Centre	Zuber Saiyed, Dipa Kinariwala, Disha Patel, Binita Aring, Neeta Khandelwal, Geeta Vaghela, Sonia Barve, Bhavesh Modi, Kairavi Joshi, Gaurishankar Shrimali, Nidhi Sood, Pranay Shah, R D Dixit, Snehal Bagatharia, Kamlesh J Upadhyay, Ramesh Pandit, Tejas Shah, Ankit Hinsu, Pritesh Sabara, Apurvasinh Puvar, Janvi Raval, Monika Gandhi, Pinal Trivedi, Maharshi Pandya, Amit Kanani, Akanksha Verma, Nitin Savaliya, Raghawendra Kumar, Dinesh Kumar, Sharmistha Majumdar, Chaitanya Joshi, Madhvi Joshi
EPI_ISL_447555	GMERS Medical College and Hospital, Gandhinagar	Gujarat Biotechnology Research Centre	Dipa Kinariwala, Disha Patel, Binita Aring, Neeta Khandelwal, Geeta Vaghela, Sonia Barve, Bhavesh Modi, Kairavi Joshi, Gaurishankar Shrimali, Nidhi Sood, Pranay Shah, R D Dixit, Snehal Bagatharia, Kamlesh J Upadhyay, Ramesh Pandit, Tejas Shah, Ankit Hinsu, Pritesh Sabara, Apurvasinh Puvar, Janvi Raval, Monika Gandhi, Pinal Trivedi, Maharshi Pandya, Amit Kanani, Akanksha Verma, Nitin Savaliya, Raghawendra Kumar, Dinesh Kumar, Zuber Saiyed, Pooja P Doshi, Chaitanya Joshi, Madhvi Joshi
EPI_ISL_447577	CSIR-Centre for Cellular and Molecular Biology	CSIR-Centre for Cellular and Molecular Biology	Namami Gaur, Sakshi Shambhavi, Lamuk Zaveri, Shagufta Khan, Tulasi Nagabandi, Purushotham Vodnala, Payel Mukherjee, Sofia Banu, Priya Singh, Dhiviya Vedagiri, Divya Gupta, Vishal Sah, Santosh Kumar Kuncha, Krishnan Harinivas Harshan, Archana Bharadwaj Siva, Karthik Bharadwaj Tallapaka, Rakesh K Mishra, Divya Tej Sowpati
EPI_ISL_447591, EPI_ISL_447592, EPI_ISL_447593	TSGH-CP molecular lab	TSGH-CP molecular lab	Cherng-Lih Perng, Ming-Jr JIAN, Chih-Kai Chang, Jung-Chung Lin, Kuo-Ming Yeh, Chien-Wen Chen, Sheng-Kang Chiu, Hsing-Yi Chung, Shih-Hung Tsai, Kuo-Sheng Hung, Tien-Yao Chang, Feng-Yee Chang, Hung-Sheng Shang
EPI_ISL_447614, EPI_ISL_447615, EPI_ISL_447616, EPI_ISL_447617, EPI_ISL_447618, EPI_ISL_447619, EPI_ISL_447620, EPI_ISL_447621, EPI_ISL_447622	Department of Laboratory Medicine, National Taiwan University Hospital	Microbial Genomics Core Lab, National Taiwan University Centers of Genomic and Precision Medicine	Shiou-Hwei Yeh, You-Yu Lin, Ya-Yun Lai, Chiao-Ling Li, Shan-Chwen Chang, Pei-Jer Chen, Sui-Yuan Chang
EPI_ISL_447734, EPI_ISL_447735, EPI_ISL_447736, EPI_ISL_447737, EPI_ISL_447738, EPI_ISL_447739, EPI_ISL_447740, EPI_ISL_447741, EPI_ISL_447742, EPI_ISL_447743, EPI_ISL_447744, EPI_ISL_447745, EPI_ISL_447746, EPI_ISL_447747, EPI_ISL_447748, EPI_ISL_447749, EPI_ISL_447750, EPI_ISL_447751, EPI_ISL_447752, EPI_ISL_447753, EPI_ISL_447754			
see above	Grupo de Investigaciones Microbiológicas-UR (GIMUR), Departamento de Biología, Facultad de Ciencias Naturales, Universidad del Rosario, Bogotá, Colombia	Grupo de Investigaciones Microbiológicas-UR (GIMUR), Departamento de Biología, Facultad de Ciencias Naturales, Universidad del Rosario, Bogotá, Colombia Instituto Nacional de Salud, Bogotá, Colombia Icahn School of Medicine at Mount Sinai, New York, USA	Juan David Ramírez, Carolina Florez, Marina Muñoz, Carolina Hernandez, Adriana Castillo, Sergio Castañeda, Nathalia Ballesteros, David Martínez, Laura Vega, Jesús E. Jaimes, Sergio Gomez, Angelica Rico, Lisseth Pardo, Esther C. Barros, Martha L. Ospina, Aníbal A. Teherán, Ana S. Gonzalez-Reiche, Matthew M. Hernandez, Emilia Mia Sordillo, Viviana Simon, Harm van Bakel, Alberto Paniz-Mondolfi

EPI_ISL_447755, EPI_ISL_447756, EPI_ISL_447757, EPI_ISL_447758, EPI_ISL_447759, EPI_ISL_447760, EPI_ISL_447761, EPI_ISL_447762, EPI_ISL_447763, EPI_ISL_447764, EPI_ISL_447765, EPI_ISL_447766, EPI_ISL_447767, EPI_ISL_447768, EPI_ISL_447769, EPI_ISL_447771, EPI_ISL_447772, EPI_ISL_447774, EPI_ISL_447775, EPI_ISL_447776, EPI_ISL_447777, EPI_ISL_447778, EPI_ISL_447779, EPI_ISL_447780, EPI_ISL_447781, EPI_ISL_447782, EPI_ISL_447783, EPI_ISL_447784, EPI_ISL_447785, EPI_ISL_447786, EPI_ISL_447787, EPI_ISL_447789, EPI_ISL_447790, EPI_ISL_447791, EPI_ISL_447792, EPI_ISL_447793, EPI_ISL_447794, EPI_ISL_447795, EPI_ISL_447796, EPI_ISL_447797, EPI_ISL_447798, EPI_ISL_447799, EPI_ISL_447800, EPI_ISL_447801, EPI_ISL_447802, EPI_ISL_447803, EPI_ISL_447804, EPI_ISL_447805, EPI_ISL_447806, EPI_ISL_447807, EPI_ISL_447808, EPI_ISL_447809, EPI_ISL_447810, EPI_ISL_447811, EPI_ISL_447812, EPI_ISL_447813, EPI_ISL_447814, EPI_ISL_447815, EPI_ISL_447816, EPI_ISL_447817			
see above	Instituto Nacional de Salud, Bogotá, Colombia	Grupo de Investigaciones Microbiológicas-UR (GIMUR), Departamento de Biología, Facultad de Ciencias Naturales, Universidad del Rosario, Bogotá, Colombia Instituto Nacional de Salud, Bogotá, Colombia Icahn School of Medicine at Mount Sinai, New York, USA	Juan David Ramírez, Carolina Florez, Marina Muñoz, Carolina Hernandez, Adriana Castillo, Sergio Castañeda, Nathalia Ballesteros, David Martínez, Laura Vega, Jesús E. Jaimes, Sergio Gomez, Angelica Rico, Lisseth Pardo, Esther C. Barros, Martha L. Ospina, Aníbal A. Teherán, Ana S. Gonzalez-Reiche, Matthew M. Hernandez, Emilia Mia Sordillo, Viviana Simon, Harm van Bakel, Alberto Paniz-Mondolfi
EPI_ISL_447854	CSIR-Centre for Cellular and Molecular Biology	CSIR-Centre for Cellular and Molecular Biology	Payel Mukherjee, Sofia Banu, Priya Singh, Dhiviya Vedagiri, Divya Gupta, Vishal Sah, Santosh Kumar Kuncha, Krishnan Harinivas Harshan, Archana Bharadwaj Siva, Karthik Bharadwaj Tallapaka, Shagufta Khan, Lamuk Zaveri, Namami Gaur, Sakshi Shambhavi, Tulasi Nagabandi, Purushotham Vodnala, Rakesh K Mishra, Divya Tej Sowpati
EPI_ISL_447855	CSIR-Centre for Cellular and Molecular Biology	CSIR-Centre for Cellular and Molecular Biology	Lamuk Zaveri, Shagufta Khan, Namami Gaur, Sakshi Shambhavi, Tulasi Nagabandi, Purushotham Vodnala, Payel Mukherjee, Sofia Banu, Priya Singh, Dhiviya Vedagiri, Divya Gupta, Vishal Sah, Santosh Kumar Kuncha, Krishnan Harinivas Harshan, Archana Bharadwaj Siva, Karthik Bharadwaj Tallapaka, Rakesh K Mishra, Divya Tej Sowpati
EPI_ISL_448222	Pasteur Insitute Ho Chi Minh City	National Key Laboratory of Gene Technology, Institute of Biotechnology, Vietnam Academy of Science and Technology	Le Tung Lam, Nguyen Trung Hieu, Nguyen Hong Trang, Ho Thi Thuong, Nguyen Thi Ngoc Thao, Huynh Thi Kim Loan, Luu Thuy Tien, Tran Huyen Linh, Pham Duy Quang, Luong Chan Quang, Cao Minh Thang, Nguyen Vu Thuong, Hoang Ha, Chu Hoang Ha, Phan Trong Lan, Truong Nam Hai
EPI_ISL_450241, EPI_ISL_450242, EPI_ISL_450243, EPI_ISL_450244, EPI_ISL_450245, EPI_ISL_450248, EPI_ISL_450249, EPI_ISL_450250, EPI_ISL_450251, EPI_ISL_450252, EPI_ISL_450258, EPI_ISL_450259, EPI_ISL_450260, EPI_ISL_450261, EPI_ISL_450262, EPI_ISL_450263, EPI_ISL_450264, EPI_ISL_450265, EPI_ISL_450266, EPI_ISL_450267, EPI_ISL_450271, EPI_ISL_450272, EPI_ISL_450273, EPI_ISL_450274, EPI_ISL_450275, EPI_ISL_450276, EPI_ISL_450277, EPI_ISL_450278, EPI_ISL_450279, EPI_ISL_450280, EPI_ISL_450281, EPI_ISL_450282, EPI_ISL_450283, EPI_ISL_450284, EPI_ISL_450285, EPI_ISL_450286, EPI_ISL_450287, EPI_ISL_450288, EPI_ISL_450289, EPI_ISL_450290, EPI_ISL_450291, EPI_ISL_450292, EPI_ISL_450293			
see above	WHO National Influenza Centre Russian Federation	WHO National Influenza Centre Russian Federation	Andrey Komissarov, Artem Fadeev, Mariia Sergeeva, Anna Ivanova, Tamila Musaeva, Ksenia Komissarova, Mariia Timofeeva, Veronica Eder, Mariia Pisareva, Daria Danilenko
EPI_ISL_450321	NIV Pune	CSIR-Centre for Cellular and Molecular Biology	Dr V A Potdar, Dr ML Choudhary,Dr Priya Abraham,V. Vipat, S. Jadhav, U. Saha, H. Kengle, A. Awhale, A. Jagtap, A. Gondhalikar, V Malik, N Srivastava, S. Digraskar, P. Malsane, S. Hundekar, K. Patel, Yogesh Balakartik, M. Kakade, S. Jadhav, R. Gunjikar, V. Awtade, S. Bhorekar, P Shinde, S. Salve, B. Minhas S. Bharadwaj, H Kaushal Y. Gurav, S. Tomar,Payel Mukherjee, Sofia Banu, Priya Singh, Dhiviya Vedagiri, Divya Gupta, Vishal Sah, Santosh Kumar Kuncha, Krishnan Harinivas Harshan, Archana Bharadwaj Siva, Karthik Bharadwaj Tallapaka, Shagufta Khan, Lamuk Zaveri, Namami Gaur, Sakshi Shambhavi, Tulasi Nagabandi, Purushotham Vodnala,G. Aditya Kumar, Koushick Sivakumar, Pooja Ramesh Gupta, Rajan Kumar Jha, Shraddha Vijay Lahoti, Deepak Kumar, Devi Prasad Vijayashankara, Disha Nanda, Divya Das, Jotin Gogoi, Manish
EPI_ISL_450322	NIV Pune	CSIR-Centre for Cellular and Molecular Biology	Dr V A Potdar, Dr ML Choudhary,Dr Priya Abraham,V. Vipat, S. Jadhav, U. Saha, H. Kengle, A. Awhale, A. Jagtap, A. Gondhalikar, V Malik, N Srivastava, S. Digraskar, P. Malsane, S. Hundekar, K. Patel, Yogesh Balakartik, M. Kakade, S. Jadhav, R. Gunjikar, V. Awtade, S. Bhorekar, P Shinde, S. Salve, B. Minhas S. Bharadwaj, H Kaushal Y. Gurav, S. Tomar,Sofia Banu, Payel Mukherjee, Priya Singh, Dhiviya Vedagiri, Divya Gupta, Vishal Sah, Santosh Kumar Kuncha, Krishnan Harinivas Harshan, Archana Bharadwaj Siva, Karthik Bharadwaj Tallapaka, Shagufta Khan, Lamuk Zaveri, Namami Gaur, Sakshi Shambhavi, Tulasi Nagabandi, Purushotham Vodnala, Disha Nanda, Divya Das, Jotin Gogoi, Manish Bhattacharjee, Ravi Prasad Mukku, Renu Sudhakar, Somesh Gorde, Gangumala Srinivas Reddy, Sujoy Deb, Swati Bayyana, Zeba Rizvi, Rakesh K Mishra
EPI_ISL_450323	NIV Pune	CSIR-Centre for Cellular and Molecular Biology	Dr V A Potdar, Dr ML Choudhary,Dr Priya Abraham,V. Vipat, S. Jadhav, U. Saha, H. Kengle, A. Awhale, A. Jagtap, A. Gondhalikar, V Malik, N Srivastava, S. Digraskar, P. Malsane, S. Hundekar, K. Patel, Yogesh Balakartik, M. Kakade, S. Jadhav, R. Gunjikar, V. Awtade, S. Bhorekar, P Shinde, S. Salve, B. Minhas S. Bharadwaj, H Kaushal Y. Gurav, S. Tomar,Payel Mukherjee, Sofia Banu, Priya Singh, Dhiviya Vedagiri, Divya Gupta, Vishal Sah, Santosh Kumar Kuncha, Krishnan Harinivas Harshan, Archana Bharadwaj Siva, Karthik Bharadwaj Tallapaka, Shagufta Khan, Lamuk Zaveri, Namami Gaur, Sakshi Shambhavi, Tulasi Nagabandi, Purushotham Vodnala,G. Aditya Kumar, Koushick Sivakumar, Pooja Ramesh Gupta, Rajan Kumar Jha, Shraddha Vijay Lahoti, Deepak Kumar, Devi Prasad Vijayashankara, Disha Nanda, Divya Das, Jotin Gogoi, Manish
EPI_ISL_450324	NIV Pune	CSIR-Centre for Cellular and Molecular Biology	Dr V A Potdar, Dr ML Choudhary,Dr Priya Abraham,V. Vipat, S. Jadhav, U. Saha, H. Kengle, A. Awhale, A. Jagtap, A. Gondhalikar, V Malik, N Srivastava, S. Digraskar, P. Malsane, S. Hundekar, K. Patel, Yogesh Balakartik, M. Kakade, S. Jadhav, R. Gunjikar, V. Awtade, S. Bhorekar, P Shinde, S. Salve, B. Minhas S. Bharadwaj, H Kaushal Y. Gurav, S. Tomar,Sofia Banu, Payel Mukherjee, Priya Singh, Dhiviya Vedagiri, Divya Gupta, Vishal Sah, Santosh Kumar Kuncha, Krishnan Harinivas Harshan, Archana Bharadwaj Siva, Karthik Bharadwaj Tallapaka, Shagufta Khan, Lamuk Zaveri, Namami Gaur, Sakshi Shambhavi, Tulasi Nagabandi, Purushotham Vodnala, Disha Nanda, Divya Das, Jotin Gogoi, Manish Bhattacharjee, Ravi Prasad Mukku, Renu Sudhakar, Somesh Gorde, Gangumala Srinivas Reddy, Sujoy Deb, Swati Bayyana, Zeba Rizvi, Rakesh K Mishra
EPI_ISL_450325	NIV Pune	CSIR-Centre for Cellular and Molecular Biology	Dr V A Potdar, Dr ML Choudhary,Dr Priya Abraham,V. Vipat, S. Jadhav, U. Saha, H. Kengle, A. Awhale, A. Jagtap, A. Gondhalikar, V Malik, N Srivastava, S. Digraskar, P. Malsane, S. Hundekar, K. Patel, Yogesh Balakartik, M. Kakade, S. Jadhav, R. Gunjikar, V. Awtade, S. Bhorekar, P Shinde, S. Salve, B. Minhas S. Bharadwaj, H Kaushal Y. Gurav, S. Tomar,Payel Mukherjee, Sofia Banu, Priya Singh, Dhiviya Vedagiri, Divya Gupta, Vishal Sah, Santosh Kumar Kuncha, Krishnan Harinivas Harshan, Archana Bharadwaj Siva, Karthik Bharadwaj Tallapaka, Shagufta Khan, Lamuk Zaveri, Namami Gaur, Sakshi Shambhavi, Tulasi Nagabandi, Purushotham Vodnala,G. Aditya Kumar, Koushick Sivakumar, Pooja Ramesh Gupta, Rajan Kumar Jha, Shraddha Vijay Lahoti, Deepak Kumar, Devi Prasad Vijayashankara, Disha Nanda, Divya Das, Jotin Gogoi, Manish
EPI_ISL_450340	Bangladesh Institute of Tropical & Infectious Diseases, COVID-19 Testing Laboratory	Basic and Applied Research on Jute Project	Rasel Ahmed, Md. Sabbir Hossain, Shah Md Tamim Kabir, Emdadul Mannan Emdad, Md. Nazmul Haq Rony, Eaftekhar Ahmed Rana, Paritous Kumar Biswas, M A Hassan Chowdhury, Md. Shakeel Ahmed, Md. Samiul Haque, Md. Monjurul Alam, Md. Sharifur Rahman, A S M Anwarul Huq, Md. Shahidul Islam, Goutam Buddha Das, AMAM Zonaed Siddiki
EPI_ISL_450341	Bangladesh Institute of Tropical & Infectious Diseases, COVID-19 Testing Laboratory	Basic and Applied Research on Jute Project	Md. Sabbir Hossain, Rasel Ahmed, Shah Md Tamim Kabir, Emdadul Mannan Emdad, Md. Nazmul Haq Rony, Eaftekhar Ahmed Rana, Paritous Kumar Biswas, M A Hassan Chowdhury, Md. Shakeel Ahmed, Md. Samiul Haque, Md. Monjurul Alam, Md. Sharifur Rahman, A S M Anwarul Huq, Md. Shahidul Islam, Goutam Buddha Das, AMAM Zonaed Siddiki
EPI_ISL_450342	Bangladesh Institute of Tropical & Infectious Diseases, COVID-19 Testing Laboratory	Basic and Applied Research on Jute Project	Rasel Ahmed, Md. Sabbir Hossain, Shah Md Tamim Kabir, Emdadul Mannan Emdad, Md. Nazmul Haq Rony, Eaftekhar Ahmed Rana, Paritous Kumar Biswas, M A Hassan Chowdhury, Md. Shakeel Ahmed, Md. Samiul Haque, Md. Monjurul Alam, Md. Sharifur Rahman, A S M Anwarul Huq, Md. Shahidul Islam, Goutam Buddha Das, AMAM Zonaed Siddiki
EPI_ISL_450343	Bangladesh Institute of Tropical & Infectious Diseases, COVID-19 Testing Laboratory	Basic and Applied Research on Jute Project	Md. Sabbir Hossain, Rasel Ahmed, Shah Md Tamim Kabir, Emdadul Mannan Emdad, Md. Nazmul Haq Rony, Eaftekhar Ahmed Rana, Paritous Kumar Biswas, M A Hassan Chowdhury, Md. Shakeel Ahmed, Md. Samiul Haque, Md. Monjurul Alam, Md. Sharifur Rahman, A S M Anwarul Huq, Md. Shahidul Islam, Goutam Buddha Das, AMAM Zonaed Siddiki
EPI_ISL_450508, EPI_ISL_450509, EPI_ISL_450510, EPI_ISL_450511, EPI_ISL_450512, EPI_ISL_450513, EPI_ISL_450514, EPI_ISL_450515, EPI_ISL_450516, EPI_ISL_450517	Rafik Hariri University Hospital	Rafik Hariri University Hospital	Rita Feghali
EPI_ISL_450724, EPI_ISL_450725, EPI_ISL_450726, EPI_ISL_450727, EPI_ISL_450728, EPI_ISL_450729, EPI_ISL_450730, EPI_ISL_450733, EPI_ISL_450735	Hospital AZ Rivierenland	Institute of Tropical Medicine	Philippe Selhorst, Colin Anthony
EPI_ISL_450746	Laboratory of Molecular Biology, Diagnostyka sp. z o.o.	Laboratory of Recombinant Vaccines	Lukasz Rabalski, Anna Piotrowska-Mietelska, Maciej Kosinski, Boguslaw Szewczyk, Krystyna Bienkowska-Szewczyk
EPI_ISL_450781	Government Medical College-Bhavnagar	Gujarat Biotechnology Research Centre	Kairavi Desai, Saklain Malek, Shirish Patel, Ramesh Pandit, Tejas Shah, Ankit Hinsu, Pritesh Sabara, Apurvasinh Puvar, Janvi Raval, Zarna Patel, Monika Gandhi, Pinal Trivedi, Maharshi Pandya, Amit Kanani, Nidhi Patel, Nitin Savaliya, Raghawendra Kumar, Dinesh Kumar, Zuber Saiyed, Komal Patel, Labdhi Pandya, Snehal Bagatharia, Bhavesh Modi, Gaurishankar Shrimali, R D Dixit, A M Kadri, Akanksha Verma, Chaitanya Joshi, Madhvi Joshi

EPI_ISL_450783	Government Medical College-Bhavnagar	Gujarat Biotechnology Research Centre	Shirish Patel, Kairavi Desai, Saklain Malek, Ankit Hinsu, Pritesh Sabara, Apurvasinh Puvar, Janvi Raval, Zarna Patel, Monika Gandhi, Pinal Trivedi, Maharshi Pandya, Amit Kanani, Nidhi Patel, Nitin Savaliya, Raghawendra Kumar, Dinesh Kumar, Zuber Saiyed, Komal Patel, Labdhi Pandya, Snehal Bagatharia, Ramesh Pandit, Tejas Shah, Bhavesh Modi, Gaurishankar Shrimali, R D Dixit, A M Kadri, Neha Rajpara, Chaitanya Joshi, Madhvi Joshi
EPI_ISL_450784	Government Medical College-Bhavnagar	Gujarat Biotechnology Research Centre	Zarna Patel, Ramesh Pandit, Tejas Shah, Ankit Hinsu, Pritesh Sabara, Apurvasinh Puvar, Janvi Raval, Monika Gandhi, Pinal Trivedi, Maharshi Pandya, Amit Kanani, Nidhi Patel, Nitin Savaliya, Raghawendra Kumar, Dinesh Kumar, Zuber Saiyed, Komal Patel, Labdhi Pandya, Snehal Bagatharia, Kairavi Desai, Saklain Malek, Shirish Patel, Bhavesh Modi, Gaurishankar Shrimali, R D Dixit, A M Kadri, Afzal Ansari, Chaitanya Joshi, Madhvi Joshi
EPI_ISL_450785	Pandit Deendayal Upadhyay Government Medical College, Rajkot	Gujarat Biotechnology Research Centre	Prakash Modi, Sejul Antala, Manish Pattani, Apurvasinh Puvar, Janvi Raval, Zarna Patel, Monika Gandhi, Pinal Trivedi, Maharshi Pandya, Amit Kanani, Nidhi Patel, Nitin Savaliya, Raghawendra Kumar, Dinesh Kumar, Zuber Saiyed, Komal Patel, Labdhi Pandya, Snehal Bagatharia, Ramesh Pandit, Tejas Shah, Ankit Hinsu, Pritesh Sabara, Bhavesh Modi, Gaurishankar Shrimali, R D Dixit, A M Kadri, Neelam Nathani, Chaitanya Joshi, Madhvi Joshi
EPI_ISL_450786	Pandit Deendayal Upadhyay Government Medical College, Rajkot	Gujarat Biotechnology Research Centre	Sejul Antala, Manish Pattani, Prakash Modi, Janvi Raval, Zarna Patel, Monika Gandhi, Pinal Trivedi, Maharshi Pandya, Amit Kanani, Nidhi Patel, Nitin Savaliya, Raghawendra Kumar, Dinesh Kumar, Zuber Saiyed, Komal Patel, Labdhi Pandya, Snehal Bagatharia, Ramesh Pandit, Tejas Shah, Ankit Hinsu, Pritesh Sabara, Apurvasinh Puvar, Bhavesh Modi, Gaurishankar Shrimali, R D Dixit, A M Kadri, Armi Chaudhari, Chaitanya Joshi, Madhvi Joshi
EPI_ISL_450788	Pandit Deendayal Upadhyay Government Medical College, Rajkot	Gujarat Biotechnology Research Centre	Zarna Patel, Tejas Shah, Ankit Hinsu, Pritesh Sabara, Apurvasinh Puvar, Janvi Raval, Monika Gandhi, Pinal Trivedi, Maharshi Pandya, Amit Kanani, Nidhi Patel, Nitin Savaliya, Raghawendra Kumar, Dinesh Kumar, Zuber Saiyed, Komal Patel, Labdhi Pandya, Snehal Bagatharia, Prakash Modi, Sejul Antala, Manish Pattani, Ramesh Pandit, Bhavesh Modi, Gaurishankar Shrimali, R D Dixit, A M Kadri, Camellia Chakraborty, Chaitanya Joshi, Madhvi Joshi
EPI_ISL_450789	Pandit Deendayal Upadhyay Government Medical College, Rajkot	Gujarat Biotechnology Research Centre	Ankit Hinsu, Pritesh Sabara, Apurvasinh Puvar, Janvi Raval, Zarna Patel, Monika Gandhi, Pinal Trivedi, Maharshi Pandya, Amit Kanani, Nidhi Patel, Nitin Savaliya, Raghawendra Kumar, Dinesh Kumar, Zuber Saiyed, Komal Patel, Labdhi Pandya, Snehal Bagatharia, Prakash Modi, Sejul Antala, Manish Pattani, Ramesh Pandit, Tejas Shah, Bhavesh Modi, Gaurishankar Shrimali, R D Dixit, A M Kadri, Siddhant Kumar, Chaitanya Joshi, Madhvi Joshi
EPI_ISL_450790	Pandit Deendayal Upadhyay Government Medical College, Rajkot	Gujarat Biotechnology Research Centre	Zarna Patel, Pritesh Sabara, Apurvasinh Puvar, Janvi Raval, Monika Gandhi, Pinal Trivedi, Maharshi Pandya, Amit Kanani, Nidhi Patel, Nitin Savaliya, Raghawendra Kumar, Dinesh Kumar, Zuber Saiyed, Komal Patel, Labdhi Pandya, Snehal Bagatharia, Prakash Modi, Sejul Antala, Manish Pattani, Ramesh Pandit, Tejas Shah, Ankit Hinsu, Bhavesh Modi, Gaurishankar Shrimali, R D Dixit, A M Kadri, Sharmistha Majumdar, Chaitanya Joshi, Madhvi Joshi
EPI_ISL_450791	Pandit Deendayal Upadhyay Government Medical College, Rajkot	Gujarat Biotechnology Research Centre	Zarna Patel, Apurvasinh Puvar, Janvi Raval, Monika Gandhi, Pinal Trivedi, Maharshi Pandya, Amit Kanani, Nidhi Patel, Nitin Savaliya, Raghawendra Kumar, Dinesh Kumar, Zuber Saiyed, Komal Patel, Labdhi Pandya, Snehal Bagatharia, Prakash Modi, Sejul Antala, Manish Pattani, Ramesh Pandit, Tejas Shah, Ankit Hinsu, Pritesh Sabara, Bhavesh Modi, Gaurishankar Shrimali, R D Dixit, A M Kadri, Pooja P Doshi, Chaitanya Joshi, Madhvi Joshi
EPI_ISL_450839	COVID-19 Laboratory Centre for Advanced Research in Sciences (CARS), University of Dhaka, Dhaka-1000, Bangladesh	DNA Solution Ltd	Sharif Akhteruzzaman, Zeba Islam Seraj, Nazmul Ahsan, Md Imdadul Hoque, MA Malek, Shahryar Nabi, Sabrina Moriom Elius, ABM Khademul Islam, Richard Malo, Imran Khan, Abu Sufian, Sabita Rezwana Rahman, Habibul Bari Shozib, Mamun Ahmed, AHM Nurun Nabi, Mohammad Riazul Islam, Md Mizanur Rahman, Md Ismail Hosen, Latiful Bari, Gazi Nurun Nahar, Haseena Khan, M Anwar Hossain.
EPI_ISL_450840	COVID-19 Laboratory	DNA Solution Ltd. L-5	Sharif Akhteruzzaman, Zeba Islam Seraj, Nazmul Ahsan, Md Imdadul Hoque, MA Malek, Shahryar Nabi, Sabrina Moriom Elius, ABM Khademul Islam, Richard Malo, Imran Khan, Abu Sufian, Sabita Rezwana Rahman, Habibul Bari Shozib, Mamun Ahmed, AHM Nurun Nabi, Mohammad Riazul Islam, Md Mizanur Rahman, Md Ismail Hosen, Latiful Bari, Gazi Nurun Nahar, Haseena Khan, M Anwar Hossain.
EPI_ISL_450841	COVID-19 Laboratory	DNA Solution Ltd	Sharif Akhteruzzaman, Zeba Islam Seraj, Nazmul Ahsan, Md Imdadul Hoque, MA Malek, Shahryar Nabi, Sabrina Moriom Elius, ABM Khademul Islam, Richard Malo, Imran Khan, Abu Sufian, Sabita Rezwana Rahman, Habibul Bari Shozib, Mamun Ahmed, AHM Nurun Nabi, Mohammad Riazul Islam, Md Mizanur Rahman, Md Ismail Hosen, Latiful Bari, Gazi Nurun Nahar, Haseena Khan, M Anwar Hossain.
EPI_ISL_450842, EPI_ISL_450843	COVID-19 Laboratory	DNA Solution Ltd.	Sharif Akhteruzzaman, Zeba Islam Seraj, Nazmul Ahsan, Md Imdadul Hoque, MA Malek, Shahryar Nabi, Sabrina Moriom Elius, ABM Khademul Islam, Richard Malo, Imran Khan, Abu Sufian, Sabita Rezwana Rahman, Habibul Bari Shozib, Mamun Ahmed, AHM Nurun Nabi, Mohammad Riazul Islam, Md Mizanur Rahman, Md Ismail Hosen, Latiful Bari, Gazi Nurun Nahar, Haseena Khan, M Anwar Hossain.
EPI_ISL_451076	West China Hospital of Sichuan University	State Key Laboratory of Biotherapy of Sichuan University	Baowen Du, Minjin Wang, Chao Tanga, Chuan Chena, Yongzhao Zhou, Mingxia Yu, Han-Cheng Wei, Weimin Li, Jing-wen Lin, Jia Geng, Binwu Ying, Lu Chen
EPI_ISL_451149	M.P Shah Government Medocal college Jamnagar	Gujarat Biotechnology Research Centre	Janvi Raval, Zarna Patel, Monika Gandhi, Pinal Trivedi, Maharshi Pandya, Amit Kanani, Nidhi Patel, Nitin Savaliya, Raghawendra Kumar, Dinesh Kumar, Zuber Saiyed, Komal Patel, Labdhi Pandya, Snehal Bagatharia, Ramesh Pandit, Tejas Shah, Ankit Hinsu, Pritesh Sabara, Apurvasinh Puvar, Binita Aring, Bhavesh Modi, Gaurishankar Shrimali, R D Dixit, A M Kadri, Priti Pandita, Chaitanya Joshi, Madhvi Joshi,
EPI_ISL_451150	M.P Shah Government Medocal college Jamnagar	Gujarat Biotechnology Research Centre	Zarna Patel, Monika Gandhi, Pinal Trivedi, Maharshi Pandya, Amit Kanani, Nidhi Patel, Nitin Savaliya, Raghawendra Kumar, Dinesh Kumar, Zuber Saiyed, Komal Patel, Labdhi Pandya, Snehal Bagatharia, Ramesh Pandit, Tejas Shah, Ankit Hinsu, Pritesh Sabara, Apurvasinh Puvar, Binita Aring, Janvi Raval, Bhavesh Modi, Gaurishankar Shrimali, R D Dixit, A M Kadri, Pragya Sharma, Chaitanya Joshi, Madhvi Joshi,
EPI_ISL_451151	M.P Shah Government Medocal college Jamnagar	Gujarat Biotechnology Research Centre	Monika Gandhi, Pinal Trivedi, Maharshi Pandya, Amit Kanani, Nidhi Patel, Nitin Savaliya, Raghawendra Kumar, Dinesh Kumar, Zuber Saiyed, Komal Patel, Labdhi Pandya, Snehal Bagatharia, Ramesh Pandit, Tejas Shah, Ankit Hinsu, Pritesh Sabara, Apurvasinh Puvar, Binita Aring, Janvi Raval, Zarna Patel, Bhavesh Modi, Gaurishankar Shrimali, R D Dixit, A M Kadri, Neha Rajpara, Chaitanya Joshi, Madhvi Joshi,
EPI_ISL_451152	M.P Shah Government Medocal college Jamnagar	Gujarat Biotechnology Research Centre	Pinal Trivedi, Maharshi Pandya, Amit Kanani, Nidhi Patel, Nitin Savaliya, Raghawendra Kumar, Dinesh Kumar, Zuber Saiyed, Komal Patel, Labdhi Pandya, Snehal Bagatharia, Ramesh Pandit, Tejas Shah, Ankit Hinsu, Pritesh Sabara, Apurvasinh Puvar, Binita Aring, Janvi Raval, Zarna Patel, Monika Gandhi, Bhavesh Modi, Gaurishankar Shrimali, R D Dixit, A M Kadri, Afzal Ansari, Chaitanya Joshi, Madhvi Joshi,
EPI_ISL_451157	Government Medical College, Vadodara	Gujarat Biotechnology Research Centre	Nidhi Patel, Nitin Savaliya, Raghawendra Kumar, Dinesh Kumar, Zuber Saiyed, Komal Patel, Labdhi Pandya, Snehal Bagatharia, Ramesh Pandit, Tejas Shah, Ankit Hinsu, Pritesh Sabara, Apurvasinh Puvar, Janvi Raval, Zarna Patel, Monika Gandhi, Pinal Trivedi, Maharshi Pandya, Manish Pattani, Tanuja Javadekar , Amit Kanani, Bhavesh Modi, Gaurishankar Shrimali, R D Dixit, A M Kadri, Camellia Chakraborty, Chaitanya Joshi, Madhvi Joshi
EPI_ISL_451158	Government Medical College, Vadodara	Gujarat Biotechnology Research Centre	Nitin Savaliya, Raghawendra Kumar, Dinesh Kumar, Zuber Saiyed, Komal Patel, Labdhi Pandya, Snehal Bagatharia, Ramesh Pandit, Tejas Shah, Ankit Hinsu, Pritesh Sabara, Apurvasinh Puvar, Janvi Raval, Zarna Patel, Monika Gandhi, Pinal Trivedi, Maharshi Pandya, Manish Pattani, Tanuja Javadekar , Amit Kanani, Nidhi Patel, Bhavesh Modi, Gaurishankar Shrimali, R D Dixit, A M Kadri, Siddhant Kumar, Chaitanya Joshi, Madhvi Joshi
EPI_ISL_451161	Government Medical College, Vadodara	Gujarat Biotechnology Research Centre	Zuber Saiyed, Komal Patel, Labdhi Pandya, Snehal Bagatharia, Ramesh Pandit, Tejas Shah, Ankit Hinsu, Pritesh Sabara, Apurvasinh Puvar, Janvi Raval, Zarna Patel, Monika Gandhi, Pinal Trivedi, Maharshi Pandya, Manish Pattani, Tanuja Javadekar , Amit Kanani, Nidhi Patel, Nitin Savaliya, Raghawendra Kumar, Dinesh Kumar, Bhavesh Modi, Gaurishankar Shrimali, R D Dixit, A M Kadri, Akanksha Verma, Chaitanya Joshi, Madhvi Joshi
EPI_ISL_451162	Government Medical College, Vadodara	Gujarat Biotechnology Research Centre	Komal Patel, Labdhi Pandya, Snehal Bagatharia, Ramesh Pandit, Tejas Shah, Ankit Hinsu, Pritesh Sabara, Apurvasinh Puvar, Janvi Raval, Zarna Patel, Monika Gandhi, Pinal Trivedi, Maharshi Pandya, Manish Pattani, Tanuja Javadekar , Amit Kanani, Nidhi Patel, Nitin Savaliya, Raghawendra Kumar, Dinesh Kumar, Zuber Saiyed, Bhavesh Modi, Gaurishankar Shrimali, R D Dixit, A M Kadri, Priti Pandita, Chaitanya Joshi, Madhvi Joshi
EPI_ISL_451298	Laboratory of Virology, INMI Lazzaro Spallanzani IRCCS	Laboratory of Virology, INMI Lazzaro Spallanzani IRCCS	Cesare E.M. Gruber, Martina Rueca, Barbara Bartolini, Francesco Messina, Antonino Di Caro, Maria R. Capobianchi, Giuseppe Ippolito
EPI_ISL_451299	Laboratory of Virology, INMI Lazzaro Spallanzani IRCCS	Laboratory of Virology, INMI Lazzaro Spallanzani IRCCS	Martina Rueca, Cesare E.M. Gruber, Barbara Bartolini, Francesco Messina, Antonino Di Caro, Maria R. Capobianchi, Giuseppe Ippolito
EPI_ISL_451300	Laboratory of Virology, INMI Lazzaro Spallanzani IRCCS	Laboratory of Virology, INMI Lazzaro Spallanzani IRCCS	Cesare E.M. Gruber, Martina Rueca, Barbara Bartolini, Francesco Messina, Antonino Di Caro, Maria R. Capobianchi, Giuseppe Ippolito
EPI_ISL_451301	Laboratory of Virology, INMI Lazzaro Spallanzani IRCCS	Laboratory of Virology, INMI Lazzaro Spallanzani IRCCS	Martina Rueca, Cesare E.M. Gruber, Barbara Bartolini, Francesco Messina, Antonino Di Caro, Maria R. Capobianchi, Giuseppe Ippolito
EPI_ISL_451302	Laboratory of Virology, INMI Lazzaro Spallanzani IRCCS	Laboratory of Virology, INMI Lazzaro Spallanzani IRCCS	Cesare E.M. Gruber, Martina Rueca, Barbara Bartolini, Francesco Messina, Antonino Di Caro, Maria R. Capobianchi, Giuseppe Ippolito
EPI_ISL_451303	Laboratory of Virology, INMI Lazzaro Spallanzani IRCCS	Laboratory of Virology, INMI Lazzaro Spallanzani IRCCS	Martina Rueca, Cesare E.M. Gruber, Barbara Bartolini, Francesco Messina, Antonino Di Caro, Maria R. Capobianchi, Giuseppe Ippolito
EPI_ISL_451304	Laboratory of Virology, INMI Lazzaro Spallanzani IRCCS	Laboratory of Virology, INMI Lazzaro Spallanzani IRCCS	Cesare E.M. Gruber, Martina Rueca, Barbara Bartolini, Francesco Messina, Antonino Di Caro, Maria R. Capobianchi, Giuseppe Ippolito
EPI_ISL_451305	Laboratory of Virology, INMI Lazzaro Spallanzani IRCCS	Laboratory of Virology, INMI Lazzaro Spallanzani IRCCS	Martina Rueca, Cesare E.M. Gruber, Barbara Bartolini, Francesco Messina, Antonino Di Caro, Maria R. Capobianchi, Giuseppe Ippolito
EPI_ISL_451306	Molecular Virology Unit, Fondazione IRCCS Policlinico San Matteo , Pavia	Laboratory of Virology, INMI Lazzaro Spallanzani IRCCS	Antonio Piralla, Fausto Baldanti, Martina Rueca, Antonino Di Caro, Maria R. Capobianchi, Cesare E.M. Gruber, Barbara Bartolini

EPI_ISL_451307	Molecular Virology Unit, Fondazione IRCCS Policlinico San Matteo , Pavia	Laboratory of Virology, INMI Lazzaro Spallanzani IRCCS	Fausto Baldanti, Antonio Piralla, Antonino Di Caro, Cesare E.M. Gruber, Martina Rueca, Barbara Bartolini, Maria R. Capobianchi
EPI_ISL_451308	Molecular Virology Unit, Fondazione IRCCS Policlinico San Matteo , Pavia	Laboratory of Virology, INMI Lazzaro Spallanzani IRCCS	Antonio Piralla, Fausto Baldanti, Maria R. Capobianchi, Cesare E.M. Gruber, Martina Rueca, Barbara Bartolini, Antonino Di Caro
EPI_ISL_451309	Molecular Virology Unit, Fondazione IRCCS Policlinico San Matteo , Pavia	Laboratory of Virology, INMI Lazzaro Spallanzani IRCCS	Fausto Baldanti, Antonio Piralla, Cesare E.M. Gruber, Maria R. Capobianchi, Antonino Di Caro, Martina Rueca, Barbara Bartolini
EPI_ISL_451310, EPI_ISL_451311	Hellenic Pasteur Institute, National Influenza Reference laboratory of Southern Greece & Unit of Bioinformatics and Applied Genomics	Hellenic Pasteur Institute, National Influenza Reference laboratory of Southern Greece & Unit of Bioinformatics and Applied Genomics	Vasiliki Pogka, Timokratis Karamitros, Athanasios Kossyvakis, Antonios Kalliaropoulos, Horefti Elina, Evangelidou Maria, Androniki Voulgari-Kokota, Aspasia Kontou, Andreas Mentis
EPI_ISL_451312, EPI_ISL_451313, EPI_ISL_451314, EPI_ISL_451315, EPI_ISL_451316, EPI_ISL_451317, EPI_ISL_451318, EPI_ISL_451319, EPI_ISL_451320, EPI_ISL_451321, EPI_ISL_451322, EPI_ISL_451323, EPI_ISL_451324, EPI_ISL_451325, EPI_ISL_451326, EPI_ISL_451327, EPI_ISL_451328, EPI_ISL_451329, EPI_ISL_451330, EPI_ISL_451331, EPI_ISL_451332, EPI_ISL_451333, EPI_ISL_451334, EPI_ISL_451335, EPI_ISL_451336, EPI_ISL_451337, EPI_ISL_451338, EPI_ISL_451339, EPI_ISL_451340, EPI_ISL_451341, EPI_ISL_451342, EPI_ISL_451343, EPI_ISL_451344, EPI_ISL_451345, EPI_ISL_451346, EPI_ISL_451347, EPI_ISL_451348, EPI_ISL_451349, EPI_ISL_451350, EPI_ISL_451351, EPI_ISL_451352, EPI_ISL_451353, EPI_ISL_451354, EPI_ISL_451355, EPI_ISL_451356, EPI_ISL_451357, EPI_ISL_451358, EPI_ISL_451359, EPI_ISL_451360, EPI_ISL_451361, EPI_ISL_451362, EPI_ISL_451363, EPI_ISL_451364, EPI_ISL_451365, EPI_ISL_451366, EPI_ISL_451367, EPI_ISL_451368, EPI_ISL_451369, EPI_ISL_451370, EPI_ISL_451371, EPI_ISL_451372, EPI_ISL_451373, EPI_ISL_451374, EPI_ISL_451375, EPI_ISL_451376, EPI_ISL_451377, EPI_ISL_451378, EPI_ISL_451379, EPI_ISL_451380, EPI_ISL_451381, EPI_ISL_451382, EPI_ISL_451383, EPI_ISL_451384, EPI_ISL_451385, EPI_ISL_451386, EPI_ISL_451387, EPI_ISL_451388, EPI_ISL_451389, EPI_ISL_451390, EPI_ISL_451391, EPI_ISL_451392, EPI_ISL_451393, EPI_ISL_451394, EPI_ISL_451395, EPI_ISL_451396, EPI_ISL_451397, EPI_ISL_451398, EPI_ISL_451399			
see above	West China Hospital of Sichuan University	State Key Laboratory of Biotherapy of Sichuan University	Baowen Du, Minjin Wang, Chao Tang, Chuan Chen, Yongzhao Zhou, Mingxia Yu, Hancheng Wei, Weimin Li, Jing-wen Lin, Jia Geng, Binwu Ying, Lu Chen
EPI_ISL_451644, EPI_ISL_451645, EPI_ISL_451646, EPI_ISL_451647	Laboratory of Molecular Biology, Diagnostyka sp. z o.o.	Laboratory of Recombinant Vaccines	Lukasz Rabalski, Anna Piotrowska-Mietelska, Maciej Kosinski, Boguslaw Szewczyk, Krystyna Bienkowska-Szewczyk
EPI_ISL_451655, EPI_ISL_451656, EPI_ISL_451657, EPI_ISL_451658, EPI_ISL_451659, EPI_ISL_451660, EPI_ISL_451661, EPI_ISL_451662, EPI_ISL_451663, EPI_ISL_451664	State Sanitary Inspectorate	Laboratory of Recombinant Vaccines	Lukasz Rabalski, Boguslaw Szewczyk, Krystyna Bienkowska-Szewczyk, Jaroslaw Pinkas
EPI_ISL_451666	M.P Shah Government Medocal college Jamnagar	Gujarat Biotechnology Research Centre	Binita Aring, Janvi Raval, Zarna Patel, Monika Gandhi, Pinal Trivedi, Maharshi Pandya, Amit Kanani, Nidhi Patel, Nitin Savaliya, Raghawendra Kumar, Dinesh Kumar, Zuber Saiyed, Komal Patel, Labdhi Pandya, Snehal Bagatharia, Ramesh Pandit, Tejas Shah, Ankit Hinsu, Pritesh Sabara, Apurvasinh Puvar, Bhavesh Modi, Gaurishankar Shrimali, R D Dixit, A M Kadri, Akanksha Verma, Chaitanya Joshi, Madhvi Joshi,
EPI_ISL_451934	Research Unit, University Hospital for Infectious Diseases "Dr. Fran Mihaljevi"	Cicin Sain lab, Helmholtz Centre for Infection Research	Zeeshan Chaudhry, Kathrin Eschke, Željka Maak Šafranko, Ivan-Christian Kurolt
EPI_ISL_451935	CUB Hopital Erasme Laboratoire d'Anatomie Pathologique	CUB Hopital Erasme Laboratoire d'Anatomie Pathologique	Prof. Isabelle Salmon, Dr. Nicky D'Haene
EPI_ISL_451961	Istituto Zooprofilattico Sperimentale Puglia e Basilicata; Dipartimento di Bioscienze, Biotecnologie e Biofarmaceutica dell'Università degli Studi di Bari "A.Moro"; Istituto di Biomembrane. Bioenergetica e Biotecnologie Molecolari del Consiglio Nazionale delle Ricerche di Bari	Beaconlab (Bioinformatics Evolution and Comparative Genomics lab), Dept of Biosciences, University of Milan	Parisi A.,Pesole G., Manzari C., Chiara M.
EPI_ISL_451962	Istituto Zooprofilattico Sperimentale Puglia e Basilicata; Dipartimento di Bioscienze, Biotecnologie e Biofarmaceutica dell'Università degli Studi di Bari "A.Moro"; Istituto di Biomembrane. Bioenergetica e Biotecnologie Molecolari del Consiglio Nazionale delle Ricerche di Bari	Beaconlab (Bioinformatics, Evolution and Comparative Genomics lab), Dept of Biosciences, University on Milan	Parisi A.,Pesole G., Manzari C., Chiara M.
EPI_ISL_452139	Instituto de Diagnostico y Referencia Epidemiologicos (INDRE)	Instituto de diagnóstico y Referencia Epidemiologicos (INDRE)	Ramirez-Gonzalez Ernesto, Garces-Ayala Fabiola, Araiza-Rodriguez Adnan, Mendieta-Condado Edgar, Rodriguez-Maldonado Abril, Wong-Arambula Claudia, Barrera-Badillo Gisela, Hernandez-Rivas Lucia, Lopez-Martinez Irma
EPI_ISL_452140	CUB Hopital Erasme Laboratoire d'Anatomie Pathologique	CUB Hopital Erasme Laboratoire d'Anatomie Pathologique	Isabelle Salmon, Nicky D'Haene
EPI_ISL_452141	Instituto de Diagnostico y Referencia Epidemiologicos (INDRE)	Instituto de diagnóstico y Referencia Epidemiologicos (INDRE)	Ramirez-Gonzalez Ernesto, Garces-Ayala Fabiola, Araiza-Rodriguez Adnan, Mendieta-Condado Edgar, Rodriguez-Maldonado Abril, Wong-Arambula Claudia, Barrera-Badillo Gisela, Hernandez-Rivas Lucia, Lopez-Martinez Irma.
EPI_ISL_452142	CUB Hopital Erasme Laboratoire d'Anatomie Pathologique	CUB Hopital Erasme Laboratoire d'Anatomie Pathologique	Isabelle Salmon, Nikcy D'Haene
EPI_ISL_452148, EPI_ISL_452149	CUB Hopital Erasme Laboratoire d'Anatomie Pathologique	CUB Hopital Erasme Laboratoire d'Anatomie Pathologique	Isabelle Salmon, Nicky D'Haene
EPI_ISL_452150	CUB Hopital Erasme Laboratoire d'Anatomie Pathologique	CUB Hopital Erasme Laboratoire d'Anatomie Pathologique	Prof. Isabelle Salmon, Dr Nicky D'Haene
EPI_ISL_452151	CUB Hopital Erasme Laboratoire d'Anatomie Pathologique	CUB Hopital Erasme Laboratoire d'Anatomie Pathologique	Prof. Isabelle Salmon, Dr.Nikcy D'Haene
EPI_ISL_452152	CUB Hopital Erasme Laboratoire d'Anatomie Pathologique	CUB Hopital Erasme Laboratoire d'Anatomie Pathologique	Prof. Isabelle Salmon, Dr Nicky D'Haene
EPI_ISL_452192, EPI_ISL_452193, EPI_ISL_452194, EPI_ISL_452195, EPI_ISL_452196, EPI_ISL_452197, EPI_ISL_452198, EPI_ISL_452199, EPI_ISL_452200, EPI_ISL_452201, EPI_ISL_452202, EPI_ISL_452203, EPI_ISL_452204, EPI_ISL_452205, EPI_ISL_452206, EPI_ISL_452207, EPI_ISL_452208, EPI_ISL_452209, EPI_ISL_452210, EPI_ISL_452211, EPI_ISL_452212, EPI_ISL_452213			
see above	NIV Influenza	NIV Influenza	Potdar V
EPI_ISL_452218, EPI_ISL_452219, EPI_ISL_452220, EPI_ISL_452221, EPI_ISL_452222, EPI_ISL_452223	Goethe University Hospital Frankfurt	Institute for Medical Virology, Goethe University Hospital Frankfurt	Tuna Toptan, Sebastian Hoehl, Sandra Westhaus, Denisa Bojkova, Annemarie Berger, Björn Rotter, Klaus Hoffmeier, Jindrich Cinatl, Sandra Ciesek, and Marek Widera
EPI_ISL_452327, EPI_ISL_452328, EPI_ISL_452329, EPI_ISL_452330, EPI_ISL_452331, EPI_ISL_452332, EPI_ISL_452333, EPI_ISL_452334, EPI_ISL_452335, EPI_ISL_452336, EPI_ISL_452337, EPI_ISL_452338, EPI_ISL_452339, EPI_ISL_452340, EPI_ISL_452341, EPI_ISL_452342, EPI_ISL_452343, EPI_ISL_452344, EPI_ISL_452345, EPI_ISL_452346, EPI_ISL_452347, EPI_ISL_452348, EPI_ISL_452349, EPI_ISL_452350, EPI_ISL_452351, EPI_ISL_452352, EPI_ISL_452353, EPI_ISL_452354, EPI_ISL_452355, EPI_ISL_452356, EPI_ISL_452357, EPI_ISL_452358, EPI_ISL_452359, EPI_ISL_452360, EPI_ISL_452361, EPI_ISL_452362, EPI_ISL_452363, EPI_ISL_452364			
see above	Laboratory of Infectious Diseases Center of Beijing Ditan Hospital	Laboratory of Infectious Diseases Center of Beijing Ditan Hospital	Siyuan Yang, Chengjie Jie, Fengting Yu, Yunxia Tang, Liting Yan, Linghang Wang
EPI_ISL_452472, EPI_ISL_452473, EPI_ISL_452480, EPI_ISL_452481, EPI_ISL_452484, EPI_ISL_452487, EPI_ISL_452488, EPI_ISL_452489, EPI_ISL_452492, EPI_ISL_452496, EPI_ISL_452498, EPI_ISL_452500, EPI_ISL_452503, EPI_ISL_452509, EPI_ISL_452515, EPI_ISL_452521, EPI_ISL_452534			
see above	Clínica Universidad de Navarra. Servicio de Enfermedades Infecciosas y Microbiología clínica	SeqCOVID-SPAIN consortium/IBV(CSIC)	Mirian Fernández-Alonso, Jose Luis del Pozo and SeqCOVID-SPAIN consortium
EPI_ISL_454420	Rafik Hariri University Hospital	Rafik Hariri University Hospital	Rita Feghali
EPI_ISL_454497, EPI_ISL_454498, EPI_ISL_454499, EPI_ISL_454500, EPI_ISL_454501, EPI_ISL_454502, EPI_ISL_454503, EPI_ISL_454504, EPI_ISL_454505, EPI_ISL_454506, EPI_ISL_454507, EPI_ISL_454508, EPI_ISL_454509, EPI_ISL_454510, EPI_ISL_454511, EPI_ISL_454512, EPI_ISL_454513, EPI_ISL_454514, EPI_ISL_454515, EPI_ISL_454516, EPI_ISL_454517, EPI_ISL_454518, EPI_ISL_454519, EPI_ISL_454520			
see above	RSE "National Center for Biotechnology"	RSE "National Center for Biotechnology"	Alexandr Shevtsov, Ilyas Akhmetollayev, Viktoriya Lutsay, Asylulan Amirgazin, Askar Abdaliyev, Akbota Rakhmetova, Zabira Aushakhmetova, Ruslan Kalendar, Yerlan Ramankulov
EPI_ISL_454521, EPI_ISL_454522, EPI_ISL_454523, EPI_ISL_454524, EPI_ISL_454525, EPI_ISL_454526, EPI_ISL_454527, EPI_ISL_454528, EPI_ISL_454529, EPI_ISL_454530, EPI_ISL_454531, EPI_ISL_454532, EPI_ISL_454533, EPI_ISL_454534, EPI_ISL_454535, EPI_ISL_454536, EPI_ISL_454537, EPI_ISL_454538, EPI_ISL_454539, EPI_ISL_454540, EPI_ISL_454541, EPI_ISL_454542, EPI_ISL_454543, EPI_ISL_454544, EPI_ISL_454545, EPI_ISL_454546, EPI_ISL_454547, EPI_ISL_454548, EPI_ISL_454549, EPI_ISL_454550, EPI_ISL_454551, EPI_ISL_454552, EPI_ISL_454553, EPI_ISL_454554, EPI_ISL_454555, EPI_ISL_454556, EPI_ISL_454557, EPI_ISL_454558, EPI_ISL_454559, EPI_ISL_454560, EPI_ISL_454561, EPI_ISL_454562			
see above	NIV Influenza	NIV Influenza	Potdar V

EPI_ISL_454574	nstitute for Public Health	Laboratory for advanced genomics	Filip Roki, Lovro Trgovec-Greif, Neven Sui, Tomislav Rukavina, Igor Jurak, Oliver Vugrek
EPI_ISL_454578, EPI_ISL_454581, EPI_ISL_454583, EPI_ISL_454588, EPI_ISL_454592, EPI_ISL_454595	University Hospital for Infectious Diseases "Dr. Fran Mihaljevi", Research Unit	University of Zagreb, Centre for research and knowledge transfer in biotechnology	Ivan-Christian Kurolt, Jelena Ivancic Jelecki, Anamarija Slovic
EPI_ISL_454602	Croatian Institute of Public Health	University of Zagreb, Centre for research and knowledge transfer in biotechnology	Irena Tabain, Tatjana Vilibic-Cavlek, Jelena Ivancic Jelecki, Anamarija Slovic
EPI_ISL_454605, EPI_ISL_454606	Institute for Public Health	Laboratory for advanced genomics	Filip Roki, Lovro Trgovec-Greif, Neven Sui, Tomislav Rukavina, Igor Jurak, Oliver Vugrek
EPI_ISL_454732	Russian State Collection of Viruses	Pathogenic Microorganisms Variability Laboratory	Denis Protsenko, Alexey Shchetinin, Maria Nikiforova, Elena Shidlovskaya, Nadezhda Kuznetsova, Vladimir Gushchin, Inna Dolzhikova, Daria Grousova, Andrey Botikov, Denis Logunov, Alexander Gintsburg, Alexey Mazus
EPI_ISL_454749	Japanese Quarantine Stations	Pathogen Genomics Center, National Institute of Infectious Diseases	Tsuyoshi Sekizuka, Kentaro Itokawa, Rina Tanaka, Masanori Hashino, Tsutomu Kageyama, Shinji Saito, Ikuyo Takayama, Hideki Hasegawa, Takuri Takahashi, Hajime Kamiya, Takuya Yamagishi, Motoi Suzuki, Takaji Wakita, Makoto Kuroda
EPI_ISL_454830, EPI_ISL_454831, EPI_ISL_454832, EPI_ISL_454833	SMS Medical College, Jaipur	CSIR Institute of Genomics and Integrative Biology	Sudhir Bhandari, Rahul Bhoyar, Mohammed Imran, Mohit Divakar, Disha Sharma, Anshul Kumar, Bani Jolly, Rahul Sahlot, Abhinav Jain, Paras Sehgal, Gyan Ranjan, Vinod Scaria, Sridhar Sivasubbu, Sandeep K Mathur
EPI_ISL_454858, EPI_ISL_454859, EPI_ISL_454860, EPI_ISL_454861, EPI_ISL_454862, EPI_ISL_454863, EPI_ISL_454864, EPI_ISL_454865, EPI_ISL_454866, EPI_ISL_454867	Translational Health Science and Technology Institute -ESIC medical college and hospital, Faridabad	THSTI Bioassay laboratory	Saurabh Kumar, Jigme Wangchuk, Anil Kumar Pandey, Asim Das, Guruprasad R. Medigeshi
EPI_ISL_455015	Pandit Deendayal Upadhyay Government Medical College, Rajkot	Gujarat Biotechnology Research Centre	Snehal Bagatharia, Prakash Modi, Sejul Antala, Manish Pattani, Ramesh Pandit, Tejas Shah, Ankit Hinsu, Pritesh Sabara, Apurvasinh Puvar, Janvi Raval, Zarna Patel, Monika Gandhi, Pinal Trivedi, Maharshi Pandya, Amit Kanani, Nidhi Patel, Nitin Savaliya, Raghawendra Kumar, Dinesh Kumar, Zuber Saiyed, Komal Patel, Labdhi Pandya, Neha Rajpara, Bhavesh Modi, Gaurishankar Shrimali, R D Dixit, A M Kadri, Umang Mishra, Chaitanya Joshi, Madhvi Joshi
EPI_ISL_455016	Pandit Deendayal Upadhyay Government Medical College, Rajkot	Gujarat Biotechnology Research Centre	Prakash Modi, Sejul Antala, Manish Pattani, Ramesh Pandit, Tejas Shah, Ankit Hinsu, Pritesh Sabara, Apurvasinh Puvar, Janvi Raval, Zarna Patel, Monika Gandhi, Pinal Trivedi, Maharshi Pandya, Amit Kanani, Nidhi Patel, Nitin Savaliya, Raghawendra Kumar, Dinesh Kumar, Zuber Saiyed, Komal Patel, Labdhi Pandya, Snehal Bagatharia, Afzal Ansari, Bhavesh Modi, Gaurishankar Shrimali, R D Dixit, A M Kadri, Umang Mishra, Chaitanya Joshi, Madhvi Joshi
EPI_ISL_455017	Government Medical College, Vadodara	Gujarat Biotechnology Research Centre	Tanuja Javadekar , R N Daveshwar, Ramesh Pandit, Tejas Shah, Ankit Hinsu, Pritesh Sabara, Apurvasinh Puvar, Janvi Raval, Zarna Patel, Monika Gandhi, Pinal Trivedi, Maharshi Pandya, Amit Kanani, Nidhi Patel, Nitin Savaliya, Raghawendra Kumar, Dinesh Kumar, Zuber Saiyed, Komal Patel, Labdhi Pandya, Snehal Bagatharia, Fenil Patel, Bhavesh Modi, Gaurishankar Shrimali, R D Dixit, A M Kadri, Umang Mishra, Chaitanya Joshi, Madhvi Joshi,
EPI_ISL_455018	Government Medical College, Vadodara	Gujarat Biotechnology Research Centre	R N Daveshwar, Ramesh Pandit, Tejas Shah, Ankit Hinsu, Pritesh Sabara, Apurvasinh Puvar, Janvi Raval, Zarna Patel, Monika Gandhi, Pinal Trivedi, Maharshi Pandya, Amit Kanani, Nidhi Patel, Nitin Savaliya, Raghawendra Kumar, Dinesh Kumar, Zuber Saiyed, Komal Patel, Labdhi Pandya, Snehal Bagatharia, Tanuja Javadekar , Neelam Nathani, Bhavesh Modi, Gaurishankar Shrimali, R D Dixit, A M Kadri, Umang Mishra, Chaitanya Joshi, Madhvi Joshi,
EPI_ISL_455019	Government Medical College, Vadodara	Gujarat Biotechnology Research Centre	Ramesh Pandit, Tejas Shah, Ankit Hinsu, Pritesh Sabara, Apurvasinh Puvar, Janvi Raval, Zarna Patel, Monika Gandhi, Pinal Trivedi, Maharshi Pandya, Amit Kanani, Nidhi Patel, Nitin Savaliya, Raghawendra Kumar, Dinesh Kumar, Zuber Saiyed, Komal Patel, Labdhi Pandya, Snehal Bagatharia, Tanuja Javadekar , R N Daveshwar, Armi Chaudhari, Bhavesh Modi, Gaurishankar Shrimali, R D Dixit, A M Kadri, Umang Mishra, Chaitanya Joshi, Madhvi Joshi,
EPI_ISL_455020	Government Medical College, Vadodara	Gujarat Biotechnology Research Centre	Tejas Shah, Ankit Hinsu, Pritesh Sabara, Apurvasinh Puvar, Janvi Raval, Zarna Patel, Monika Gandhi, Pinal Trivedi, Maharshi Pandya, Amit Kanani, Nidhi Patel, Nitin Savaliya, Raghawendra Kumar, Dinesh Kumar, Zuber Saiyed, Komal Patel, Labdhi Pandya, Snehal Bagatharia, Tanuja Javadekar , R N Daveshwar, Ramesh Pandit, Bhavya Jindal, Bhavesh Modi, Gaurishankar Shrimali, R D Dixit, A M Kadri, Umang Mishra, Chaitanya Joshi, Madhvi Joshi,
EPI_ISL_455021	Government Medical College, Vadodara	Gujarat Biotechnology Research Centre	Ankit Hinsu, Pritesh Sabara, Apurvasinh Puvar, Janvi Raval, Zarna Patel, Monika Gandhi, Pinal Trivedi, Maharshi Pandya, Amit Kanani, Nidhi Patel, Nitin Savaliya, Raghawendra Kumar, Dinesh Kumar, Zuber Saiyed, Komal Patel, Labdhi Pandya, Snehal Bagatharia, Tanuja Javadekar , R N Daveshwar, Ramesh Pandit, Tejas Shah, Camellia Chakraborty, Bhavesh Modi, Gaurishankar Shrimali, R D Dixit, A M Kadri, Umang Mishra, Chaitanya Joshi, Madhvi Joshi,
EPI_ISL_455022	Government Medical College, Vadodara	Gujarat Biotechnology Research Centre	Pritesh Sabara, Apurvasinh Puvar, Janvi Raval, Zarna Patel, Monika Gandhi, Pinal Trivedi, Maharshi Pandya, Amit Kanani, Nidhi Patel, Nitin Savaliya, Raghawendra Kumar, Dinesh Kumar, Zuber Saiyed, Komal Patel, Labdhi Pandya, Snehal Bagatharia, Tanuja Javadekar , R N Daveshwar, Ramesh Pandit, Tejas Shah, Ankit Hinsu, Siddhant Kumar, Bhavesh Modi, Gaurishankar Shrimali, R D Dixit, A M Kadri, Umang Mishra, Chaitanya Joshi, Madhvi Joshi,
EPI_ISL_455023	Government Medical College, Vadodara	Gujarat Biotechnology Research Centre	Apurvasinh Puvar, Janvi Raval, Zarna Patel, Monika Gandhi, Pinal Trivedi, Maharshi Pandya, Amit Kanani, Nidhi Patel, Nitin Savaliya, Raghawendra Kumar, Dinesh Kumar, Zuber Saiyed, Komal Patel, Labdhi Pandya, Snehal Bagatharia, Tanuja Javadekar , R N Daveshwar, Ramesh Pandit, Tejas Shah, Ankit Hinsu, Pritesh Sabara, Priyanka P Vatsa, Bhavesh Modi, Gaurishankar Shrimali, R D Dixit, A M Kadri, Umang Mishra, Chaitanya Joshi, Madhvi Joshi,
EPI_ISL_455024	Government Medical College, Vadodara	Gujarat Biotechnology Research Centre	Janvi Raval, Zarna Patel, Monika Gandhi, Pinal Trivedi, Maharshi Pandya, Amit Kanani, Nidhi Patel, Nitin Savaliya, Raghawendra Kumar, Dinesh Kumar, Zuber Saiyed, Komal Patel, Labdhi Pandya, Snehal Bagatharia, Tanuja Javadekar , R N Daveshwar, Ramesh Pandit, Tejas Shah, Ankit Hinsu, Pritesh Sabara, Apurvasinh Puvar, Pooja P Doshi, Bhavesh Modi, Gaurishankar Shrimali, R D Dixit, A M Kadri, Umang Mishra, Chaitanya Joshi, Madhvi Joshi,
EPI_ISL_455025	Government Medical College, Vadodara	Gujarat Biotechnology Research Centre	Zarna Patel, Monika Gandhi, Pinal Trivedi, Maharshi Pandya, Amit Kanani, Nidhi Patel, Nitin Savaliya, Raghawendra Kumar, Dinesh Kumar, Zuber Saiyed, Komal Patel, Labdhi Pandya, Snehal Bagatharia, Tanuja Javadekar , R N Daveshwar, Ramesh Pandit, Tejas Shah, Ankit Hinsu, Pritesh Sabara, Apurvasinh Puvar, Janvi Raval, Akanksha Verma, Bhavesh Modi, Gaurishankar Shrimali, R D Dixit, A M Kadri, Umang Mishra, Chaitanya Joshi, Madhvi Joshi,
EPI_ISL_455026	Government Medical College, Vadodara	Gujarat Biotechnology Research Centre	Monika Gandhi, Pinal Trivedi, Maharshi Pandya, Amit Kanani, Nidhi Patel, Nitin Savaliya, Raghawendra Kumar, Dinesh Kumar, Zuber Saiyed, Komal Patel, Labdhi Pandya, Snehal Bagatharia, Tanuja Javadekar , R N Daveshwar, Ramesh Pandit, Tejas Shah, Ankit Hinsu, Pritesh Sabara, Apurvasinh Puvar, Janvi Raval, Zarna Patel, Priti Pandita, Bhavesh Modi, Gaurishankar Shrimali, R D Dixit, A M Kadri, Umang Mishra, Chaitanya Joshi, Madhvi Joshi,
EPI_ISL_455027	Government Medical College, Vadodara	Gujarat Biotechnology Research Centre	Pinal Trivedi, Maharshi Pandya, Amit Kanani, Nidhi Patel, Nitin Savaliya, Raghawendra Kumar, Dinesh Kumar, Zuber Saiyed, Komal Patel, Labdhi Pandya, Snehal Bagatharia, Tanuja Javadekar , R N Daveshwar, Ramesh Pandit, Tejas Shah, Ankit Hinsu, Pritesh Sabara, Apurvasinh Puvar, Janvi Raval, Zarna Patel, Monika Gandhi, Pragya Sharma, Bhavesh Modi, Gaurishankar Shrimali, R D Dixit, A M Kadri, Umang Mishra, Chaitanya Joshi, Madhvi Joshi,
EPI_ISL_455312	Microbiology Unit, Department of Pathology & Laboratory Medicine, IIUM Medical Centre	SEA Microbiome Unit, Faculty of Industrial Sciences & Technology, Universiti Malaysia Pahang	Norhidayah Binti Kamarudin, Ahmad Hafiz Bin Zulkifly, Hajar Fauzan Ahmad, Muhammad Adam Lee Abdullah, Mohd Fazli Farida Asras, Ahmad Mahfuz Gazali, Mohd Nazli Bin Kamarulzaman, IIUM Medical Centre Covid19 Taskforce, UMP Covid19 Team
EPI_ISL_455313	Microbiology Unit, Department of Pathology & Laboratory Medicine, IIUM Medical Centre	SEA Microbiome Unit, Faculty of Industrial Sciences & Technology, Universiti Malaysia Pahang	Hajar Fauzan Ahmad, Norhidayah Kamarudin, Ahmad Hafiz Zulkifly, IIUM Medical Centre Covid19 Taskforce, UMP Covid19 Team
EPI_ISL_455314	Hospital Virgen del Rocio	Instituto de Salud Carlos III	Iglesias-Caballero, M. Molinero Calamita, M. González-Esguevillas, M. Camarero, S. Pozo, F. Casas, I. Jiménez, P. Jiménez, M. Zaballos, A. Monzón, S. Varona, S. Juliá, M. Cuesta, I, J. Lepe
EPI_ISL_455315, EPI_ISL_455316, EPI_ISL_455317, EPI_ISL_455318, EPI_ISL_455319, EPI_ISL_455320, EPI_ISL_455321, EPI_ISL_455322	Hospital Virgen de las Nieves	Instituto de Salud Carlos III	Iglesias-Caballero, M. Molinero Calamita, M. González-Esguevillas, M. Camarero, S. Pozo, F. Casas, I. Jiménez, P. Jiménez, M. Zaballos, A. Monzón, S. Varona, S. Juliá, M. Cuesta, I, S. Sanbonmatsu
EPI_ISL_455323	Hospital Virgen del Rocio	Instituto de Salud Carlos III	Iglesias-Caballero, M. Molinero Calamita, M. González-Esguevillas, M. Camarero, S. Pozo, F. Casas, I. Jiménez, P. Jiménez, M. Zaballos, A. Monzón, S. Varona, S. Juliá, M. Cuesta, I, J. Lepe
EPI_ISL_455324	Hospital Virgen de las Nieves	Instituto de Salud Carlos III	Iglesias-Caballero, M. Molinero Calamita, M. González-Esguevillas, M. Camarero, S. Pozo, F. Casas, I. Jiménez, P. Jiménez, M. Zaballos, A. Monzón, S. Varona, S. Juliá, M. Cuesta, I, S. Sanbonmatsu

EPI_ISL_455325	Hospital Universitario de Canarias	Instituto de Salud Carlos III	Iglesias-Caballero, M. Molinero Calamita, M. González-Esguevillas, M. Camarero, S. Pozo, F. Casas, I. Jiménez, P. Jiménez, M. Zaballos, A. Monzón, S. Varona, S. Juliá, M. Cuesta, I, B. Castro
EPI_ISL_455326	Hospital Universitario Insular de Gran Canaria	Instituto de Salud Carlos III	Iglesias-Caballero, M. Molinero Calamita, M. González-Esguevillas, M. Camarero, S. Pozo, F. Casas, I. Jiménez, P. Jiménez, M. Zaballos, A. Monzón, S. Varona, S. Juliá, M. Cuesta, I, A. Hernández
EPI_ISL_455327	Consejeria de Sanidad y Asuntos Sociales	Instituto de Salud Carlos III	Iglesias-Caballero, M. Molinero Calamita, M. González-Esguevillas, M. Camarero, S. Pozo, F. Casas, I. Jiménez, P. Jiménez, M. Zaballos, A. Monzón, S. Varona, S. Juliá, M. Cuesta, I, G. Gutiérrez
EPI_ISL_455328, EPI_ISL_455329, EPI_ISL_455330, EPI_ISL_455331	Complejo Hospitalario Universitario La Coruna	Instituto de Salud Carlos III	Iglesias-Caballero, M. Molinero Calamita, M. González-Esguevillas, M. Camarero, S. Pozo, F. Casas, I. Jiménez, P. Jiménez, M. Zaballos, A. Monzón, S. Varona, S. Juliá, M. Cuesta, I, M.A Canizares
EPI_ISL_455332	Xerencia de Xestión Integrada de Pontevedra e o Salnés	Instituto de Salud Carlos III	Iglesias-Caballero, M. Molinero Calamita, M. González-Esguevillas, M. Camarero, S. Pozo, F. Casas, I. Jiménez, P. Jiménez, M. Zaballos, A. Monzón, S. Varona, S. Juliá, M. Cuesta, I, M. Garcia
EPI_ISL_455333	Complejo Hospitalario Universitario de Santiago	Instituto de Salud Carlos III	Iglesias-Caballero, M. Molinero Calamita, M. González-Esguevillas, M. Camarero, S. Pozo, F. Casas, I. Jiménez, P. Jiménez, M. Zaballos, A. Monzón, S. Varona, S. Juliá, M. Cuesta, I, J. Llovo
EPI_ISL_455334, EPI_ISL_455335	Complejo Hospitalario de Orense	Instituto de Salud Carlos III	Iglesias-Caballero, M. Molinero Calamita, M. González-Esguevillas, M. Camarero, S. Pozo, F. Casas, I. Jiménez, P. Jiménez, M. Zaballos, A. Monzón, S. Varona, S. Juliá, M. Cuesta, I, M. Paz
EPI_ISL_455336, EPI_ISL_455337, EPI_ISL_455338, EPI_ISL_455339, EPI_ISL_455340, EPI_ISL_455341, EPI_ISL_455342, EPI_ISL_455343	Hospital San Pedro	Instituto de Salud Carlos III	Iglesias-Caballero, M. Molinero Calamita, M. González-Esguevillas, M. Camarero, S. Pozo, F. Casas, I. Jiménez, P. Jiménez, M. Zaballos, A. Monzón, S. Varona, S. Juliá, M. Cuesta, I, C. Alonso
EPI_ISL_455344, EPI_ISL_455345, EPI_ISL_455346, EPI_ISL_455347, EPI_ISL_455348, EPI_ISL_455349	Hospital Comarcal de Melilla	Instituto de Salud Carlos III	Iglesias-Caballero, M. Molinero Calamita, M. González-Esguevillas, M. Camarero, S. Pozo, F. Casas, I. Jiménez, P. Jiménez, M. Zaballos, A. Monzón, S. Varona, S. Juliá, M. Cuesta, I, I. Pérez
EPI_ISL_455350, EPI_ISL_455351	Hospital Txagorritxu	Instituto de Salud Carlos III	Iglesias-Caballero, M. Molinero Calamita, M. González-Esguevillas, M. Camarero, S. Pozo, F. Casas, I. Jiménez, P. Jiménez, M. Zaballos, A. Monzón, S. Varona, S. Juliá, M. Cuesta, I, C. Gómez
EPI_ISL_455352, EPI_ISL_455353, EPI_ISL_455354	Hospital de Cruces	Instituto de Salud Carlos III	Iglesias-Caballero, M. Molinero Calamita, M. González-Esguevillas, M. Camarero, S. Pozo, F. Casas, I. Jiménez, P. Jiménez, M. Zaballos, A. Monzón, S. Varona, S. Juliá, M. Cuesta, I, M. Aranzamendi
EPI_ISL_455362	Nigeria Centre for Disease Control (NCDC)	African Centre of Excellence for Genomics of Infectious Diseases (ACEGID), Redeemer's University, Ede, Osun State, Nigeria	Oluniyi P.E., Ajogbasile F.V., Kayode A., Olawoye I., Uwanibe J., Oguzie J., Olumade T., Folarin O.A., Ihekweazu C., Happi C.T.
EPI_ISL_455412, EPI_ISL_455413	Nigeria Centre for Disease Control (NCDC)	African Centre of Excellence for Genomics of Infectious Diseases (ACEGID), Redeemer's University, Ede, Osun State, Nigeria	Oluniyi P.E., Ajogbasile F.V., Kayode A., Oguzie J., Olawoye I., Uwanibe J., Olumade T., Folarin O.A., Ihekweazu C., Happi C.T.
EPI_ISL_455415	Nigeria Centre for Disease Control (NCDC)	African Centre of Excellence for Genomics of Infectious Diseases (ACEGID), Redeemer's University, Ede, Osun State, Nigeria	Oluniyi P.E., Ajogbasile F.V., Kayode A., Oguzie J., Olawoye I., Uwanibe J., Olumade T., Folarin O.A., Ihekweazu C., Happi C.T.
EPI_ISL_455418, EPI_ISL_455419	Nigeria Centre for Disease Control (NCDC)	African Centre of Excellence for Genomics of Infectious Diseases (ACEGID), Redeemer's University, Ede, Osun State, Nigeria	Oluniyi P.E., Ajogbasile F.V., Kayode A., Oguzie J., Olawoye I., Uwanibe J., Olumade T., Folarin O.A., Ihekweazu C., Happi C.T.
EPI_ISL_455422	Nigeria Centre for Disease Control	African Centre of Excellence for Genomics of Infectious Diseases (ACEGID), Redeemer's University, Ede, Osun State, Nigeria	Oluniyi P.E., Ajogbasile F.V., Kayode A., Oguzie J., Olawoye I., Uwanibe J., Olumade T., Folarin O.A., Ihekweazu C., Happi C.T.
EPI_ISL_455423, EPI_ISL_455424, EPI_ISL_455425	Nigeria Centre for Disease Control (NCDC)	African Centre of Excellence for Genomics of Infectious Diseases (ACEGID), Redeemer's University, Ede, Osun State, Nigeria	Oluniyi P.E., Ajogbasile F.V., Kayode A., Oguzie J., Olawoye I., Uwanibe J., Olumade T., Folarin O.A., Ihekweazu C., Happi C.T.
EPI_ISL_455426	Nigeria Centre for Disease Control	African Centre of Excellence for Genomics of Infectious Diseases (ACEGID), Redeemer's University, Ede, Osun State, Nigeria	Oluniyi P.E., Ajogbasile F.V., Kayode A., Oguzie J., Olawoye I., Uwanibe J., Olumade T., Folarin O.A., Ihekweazu C., Happi C.T.
EPI_ISL_455427, EPI_ISL_455429, EPI_ISL_455430, EPI_ISL_455431	Nigeria Centre for Disease Control (NCDC)	African Centre of Excellence for Genomics of Infectious Diseases (ACEGID), Redeemer's University, Ede, Osun State, Nigeria	Oluniyi P.E., Ajogbasile F.V., Kayode A., Oguzie J., Olawoye I., Uwanibe J., Olumade T., Folarin O.A., Ihekweazu C., Happi C.T.
EPI_ISL_455432, EPI_ISL_455434	Instituto de Diagnostico y Referencia Epidemiologicos (INDRE)	Instituto de Diagnostico y Referencia Epidemiologicos (INDRE)	Taboada Ramírez Blanca. Ramirez-Gonzalez Ernesto, Garces-Ayala Fabiola, Araiza-Rodriguez Adnan, Mendieta-Condado Edgar, Rodriguez-Maldonado Abril, Wong-Arambula Claudia, Barrera-Badillo Gisela, Hernandez-Rivas Lucia, Lopez-Martinez Irma.
EPI_ISL_455435	Instituto de Diagnostico y Referencia Epidemiologicos (INDRE)	Instituto de Diagnostico y Referencia Epidemiologicos (INDRE)	Garces-Ayala Fabiola. Taboada Ramírez Blanca. Ramirez-Gonzalez Ernesto, Araiza-Rodriguez Adnan , Mendieta-Condado Edgar, Rodriguez-Maldonado Abril, Wong-Arambula Claudia, Barrera-Badillo Gisela, Hernandez-Rivas Lucia, Lopez-Martinez Irma
EPI_ISL_455436	Instituto de Diagnostico y Referencia Epidemiologicos (INDRE)	Instituto de Diagnostico y Referencia Epidemiologicos (INDRE)	Garces-Ayala Fabiola. Taboada Ramírez Blanca. Ramirez-Gonzalez Ernesto, Araiza-Rodriguez Adnan, Mendieta-Condado Edgar, Rodriguez-Maldonado Abril, Wong-Arambula Claudia, Barrera-Badillo Gisela, Hernandez-Rivas Lucia, Lopez-Martinez Irma.
EPI_ISL_455437, EPI_ISL_455438	Instituto de Diagnostico y Referencia Epidemiologicos (INDRE)	Instituto de Diagnostico y Referencia Epidemiologicos (INDRE)	Araiza-Rodriguez Adnan, Garces-Ayala Fabiola. Ramirez-Gonzalez Ernesto, Mendieta-Condado Edgar, Rodriguez-Maldonado Abril, Wong-Arambula Claudia, Barrera-Badillo Gisela, Hernandez-Rivas Lucia, Lopez-Martinez Irma, Taboada Ramírez Blanca.
EPI_ISL_455439	Instituto de Diagnostico y Referencia Epidemiologicos (INDRE)	Instituto de Diagnostico y Referencia Epidemiologicos (INDRE)	Mendieta-Condado Edgar, Araiza-Rodriguez Adnan, Garces-Ayala Fabiola, Rodriguez-Maldonado Abril, Wong-Arambula Claudia, Barrera-Badillo Gisela, Hernandez-Rivas Lucia, Lopez-Martinez Irma, Taboada Ramirez Blanca, Ramirez-Gonzalez Ernesto.
EPI_ISL_455454	Instituto de Diagnostico y Referencia Epidemiologicos (INDRE)	Instituto de Diagnostico y Referencia Epidemiologicos (INDRE)	Mendieta-Condado Edgar, Araiza-Rodriguez Adnan, Garces-Ayala Fabiola, , Rodriguez-Maldonado Abril, Wong-Arambula Claudia, Barrera-Badillo Gisela, Taboada Ramírez Blanca, Ramirez-Gonzalez Ernesto, Hernandez-Rivas Lucia, Lopez-Martinez Irma.
EPI_ISL_455455	Instituto de Diagnostico y Referencia Epidemiologicos (INDRE)	Instituto de Diagnostico y Referencia Epidemiologicos (INDRE)	Rodriguez-Maldonado Abril, Mendieta-Condado Edgar, Araiza-Rodriguez Adnan, Garces-Ayala Fabiola. Taboada Ramirez Blanca. Ramirez-Gonzalez Ernesto, Barrera-Badillo Gisela, Hernandez-Rivas Lucia, Lopez-Martinez Irma, Wong-Arambula Claudia.
EPI_ISL_455456	Instituto de Diagnostico y Referencia Epidemiologicos (INDRE)	Instituto de Diagnostico y Referencia Epidemiologicos (INDRE)	Rodriguez-Maldonado Abril, Mendieta-Condado Edgar, Araiza-Rodriguez Adnan, Garces-Ayala Fabiola. Taboada Ramírez Blanca. Ramirez-Gonzalez Ernesto, , Barrera-Badillo Gisela, Hernandez-Rivas Lucia, Lopez-Martinez Irma, Wong-Arambula Claudia.
EPI_ISL_455460, EPI_ISL_455461, EPI_ISL_455462, EPI_ISL_455463, EPI_ISL_455464, EPI_ISL_455465, EPI_ISL_455466, EPI_ISL_455467	Jiangxi Province Center for Disease Control and Prevention	Jiangxi Province Center for Disease Control and Prevention	JianXiong Li,Ying Xiong,Tian Gong,Yong Shi,Jun Zhou,Fang Xiao,ShiWen Liu,XiaoQing Liu,Gang Xu,DaJin Xiao,Xin Ran,YanNi Zhang
EPI_ISL_455468, EPI_ISL_455469, EPI_ISL_455470, EPI_ISL_455471, EPI_ISL_455472, EPI_ISL_455473, EPI_ISL_455474	Laboratory for Respiratory Viruses, Cantacuzino National Military-Medical Institute for Research and Development	Cantacuzino Institute	M.Lazar, L.Ustea, A.Cretu, Tim Durfee

EPI_ISL_455475, EPI_ISL_455476, EPI_ISL_455477, EPI_ISL_455479	Laboratory for Respiratory Viruses, Cantacuzino National Military-Medical Institute for Research and Development	Cantacuzino Institute	M.Lazar, L.Ustea, A.Cretu, T.Durfee
EPI_ISL_455566, EPI_ISL_455567	Institute for Public Health	Laboratory for advanced genomics	Filip Roki, Lovro Trgovec-Greif, Neven Sui, Tomislav Rukavina, Igor Jurak, Oliver Vugrek
EPI_ISL_455694, EPI_ISL_455695, EPI_ISL_455696, EPI_ISL_455697, EPI_ISL_455698, EPI_ISL_455699, EPI_ISL_455700, EPI_ISL_455701, EPI_ISL_455702, EPI_ISL_455703, EPI_ISL_455704, EPI_ISL_455705, EPI_ISL_455706, EPI_ISL_455707, EPI_ISL_455708, EPI_ISL_455709, EPI_ISL_455710, EPI_ISL_455711, EPI_ISL_455712			
see above	National Hospital of Tropical Diseases	Oxford University Clinical Research Unit, Hanoi, Vietnam	Nguyen Thi Tam, Van Dinh Trang, Nguyen Thu Trang, Nguyen Thi Ngoc Diep, Le Nguyen Minh Hoa, Pham Ngoc Thach, H. Rogier van Doorn, on behalf of the OUCRU COVID-19 research group
EPI_ISL_455713, EPI_ISL_455714, EPI_ISL_455715, EPI_ISL_455716, EPI_ISL_455717, EPI_ISL_455718	National Hospital of Tropical Diseases	Oxford University Clinical Research Unit, Hanoi, Vietnam	Nguyen Thi Tam, Van Dinh Trang, Nguyen Thi Hong Thuong, Vu Thi Ngoc Bich, Nguyen Thu Trang, Nguyen Thi Ngoc Diep, Le Nguyen Minh Hoa, Pham Ngoc Thach, H. Rogier van Doorn, on behalf of the OUCRU COVID-19 research group
EPI_ISL_455719	T.C. Salk Bakanl Adyaman l Salk Müdürlüü Adyaman Eitim Ve Aratrma Hastanesi	VETAL Animal Health Products Company, BSL3+ Production Laboratuary /Turkey	Mehmet Turgut, Muhittin Önderci, Fatma Nilay Tutak, Abidin Ercan Yonucu, Murat Dönen, Haluk Uluca, Fethiye Sevimli, O. Ugur Sezerman
EPI_ISL_455790, EPI_ISL_455791	Institute for Medical Research, Infectious Disease Research Centre, National Institutes of Health, Ministry of Health Malaysia	Malaysia Genome Institute	Mohd Noor Mat Isa, Irni Suhayu Sapian, Yusuf Muhammad Noor, Jeyanthi Suppiah, Nurhezreen Md Iqbal, Enizza Kasim, Zarina Mohd Zawawi, Siti Noraini Othman, Mohd Faizal Abu Bakar, Shamsidar Sopie, Azrin Ahmad, Ravindran Thayan, Norazah Ahmad, Tahir Aris, Shahrul Hisham Zainal Ariffin
EPI_ISL_455792, EPI_ISL_455793	Institute for Medical Research, Infectious Disease Research Centre, National Institutes of Health, Ministry of Health Malaysia	Malaysia Genome Institute	Mohd Noor Mat Isa, Irni Suhayu Sapian, Yusuf Muhammad Noor, Jeyanthi Suppiah, Nurhezreen Md Iqbal, Enizza Kasim, Zarina Mohd Zawawi, Siti Noraini Othman, Mohd Faizal Abu Bakar, Shamsidar Sopie, Azrin Ahmad, Ravindran Thayan, Norazah Ahmad, Tahir Aris, Shahrul Hisham Zainal Ariffin
EPI_ISL_456116, EPI_ISL_456117, EPI_ISL_456118, EPI_ISL_456119, EPI_ISL_456120, EPI_ISL_456121, EPI_ISL_456122, EPI_ISL_456123, EPI_ISL_456124, EPI_ISL_456125, EPI_ISL_456126, EPI_ISL_456127, EPI_ISL_456128, EPI_ISL_456129, EPI_ISL_456130, EPI_ISL_456131, EPI_ISL_456132, EPI_ISL_456133, EPI_ISL_456134, EPI_ISL_456135, EPI_ISL_456136, EPI_ISL_456137, EPI_ISL_456138, EPI_ISL_456139, EPI_ISL_456140, EPI_ISL_456141, EPI_ISL_456142, EPI_ISL_456143, EPI_ISL_456145, EPI_ISL_456146, EPI_ISL_456147, EPI_ISL_456148, EPI_ISL_456149, EPI_ISL_456150, EPI_ISL_456151, EPI_ISL_456152, EPI_ISL_456155, EPI_ISL_456156			
see above	Instituto Nacional de Salud - Unidad de Secuenciación y Análisis Genómico	Instituto Nacional de Salud, Universidad Cooperativa de Colombia, Instituto Alexander von Humboldt, Imperial College-London, London School of Hygiene & Tropical Medicine	Katherine Laiton-Donato, Diego A. Álvarez-Díaz, Carlos Franco-Muñoz, Jose A. Usme-Ciro, Gloria Puerto, Nicolas D. Franco-Sierra, Mailyn A.Gonzalez, Zulma M. Cucunubá, Christian Julian VillabonaArenas, Liz Villabona-Arenas, Sussy Echeverria, Astrid C. Flórez, Sergio Gomez-Rangel, Luz Dary Rodriguez, Juliana Barbosa, Erika Ospitia, Diana Marcela Walteros-Acero, Martha Lucia Ospina Martinez, Marcela Mercado-Reyes.
EPI_ISL_457702	Oman-NIC	Microbiology laboratory- Sultan Qaboos University Hospital	Fahad Zadjali, Samira Al-Maruqi, Amina Al Jardani, Khulood Al-Mammary, Hanan Al-kindi, Fatma BaAlawi, Hamida AL Barwani, Zeyana AL-Dahmani, Intisar Al-Shukri, Aisha Al-Busaidi, Aisha Al-Amri, Ahlam Al-Amri, Mohammed Al-Tobi, Samiha Al Kharusi, Abdulla Balkhair
EPI_ISL_457703	Oman-NIC	Department of Microbiology and Immunology- SQUH	Fahad Zadjali, Samira Al-Maruqi, Amina Al Jardani, Khulood Al-Mammary, Hanan Al-kindi, Fatma BaAlawi, Hamida AL Barwani, Zeyana AL-Dahmani, Intisar Al-Shukri, Aisha Al-Busaidi, Aisha Al-Amri, Ahlam Al-Amri, Mohammed Al-Tobi, Samiha Al Kharusi, Abdulla Balkhair
EPI_ISL_457705	OMAN-NIC	Department of Microbiology and Immunology- SQUH	Fahad Zadjali, Samira Al-Maruqi, Amina Al Jardani, Khulood Al-Mammary, Hanan Al-kindi, Fatma BaAlawi, Hamida AL Barwani, Zeyana AL-Dahmani, Intisar Al-Shukri, Aisha Al-Busaidi, Aisha Al-Amri, Ahlam Al-Amri, Mohammed Al-Tobi, Samiha Al Kharusi, Abdulla Balkhair
EPI_ISL_457707	Oman-NIC	Department of Microbiology and Immunology- SQUH	Fahad Zadjali, Samira Al-Maruqi, Amina Al Jardani, Khulood Al-Mammary, Hanan Al-kindi, Fatma BaAlawi, Hamida AL Barwani, Zeyana AL-Dahmani, Intisar Al-Shukri, Aisha Al-Busaidi, Aisha Al-Amri, Ahlam Al-Amri, Mohammed Al-Tobi, Samiha Al Kharusi, Abdulla Balkhair
EPI_ISL_457726, EPI_ISL_457730	TSGH-CP molecular lab	TSGH-CP molecular lab	Cherng-Lih Perng, Ming-Jr JIAN, Chih-Kai Chang, Jung-Chung Lin, Kuo-Ming Yeh, Chien-Wen Chen, Sheng-Kang Chiu, Hsing-Yi Chung, Shih-Hung Tsai, Kuo-Sheng Hung, Tien-Yao Chang, Feng-Yee Chang, Hung-Sheng Shang
EPI_ISL_457750	Centogene AG	Centogene AG	Prof. Dr. Peter Bauer, Dr. Krishna Kumar Kandaswamy
EPI_ISL_457824	Bezmialem Vakif University, Dept Microbiology, Medical School, Fatih, Istanbul, Turkey	Bezmialem Vakif University, Medical School & Beykoz Institute of Life Sciences & Biotechnology	Mehmet Z. Doymaz, Merve Kalkan, Nesibe Cetin, Elif Karaaslan, Bilge Sumbul, Filiz Guney
EPI_ISL_457997	Oman-NIC	Oman-NIC	Samira Al-Maruqi, Fahad Zadjali, Amina Al Jardani, Khulood Al-Mammary, Hanan Al-kindi, Fatma BaAlawi, Hamida AL Barwani, Zeyana AL-Dahmani, Intisar Al-Shukri, Aisha Al-Busaidi, Aisha Al-Amri, Ahlam Al-Amri, Mohammed Al-Tobi, Samiha Al Kharusi, Abdulla Balkhair
EPI_ISL_458029	TSGH-CP molecular lab	TSGH-CP molecular lab	Cherng-Lih Perng, Ming-Jr JIAN, Chih-Kai Chang, Jung-Chung Lin, Kuo-Ming Yeh, Chien-Wen Chen, Sheng-Kang Chiu, Hsing-Yi Chung, Shih-Hung Tsai, Kuo-Sheng Hung, Tien-Yao Chang, Feng-Yee Chang, Hung-Sheng Shang
EPI_ISL_458079	Mitra Keluarga Hospital Kenjeran	Institute of Tropical Disease, Universitas Airlangga	Aldise M Nastri, Jezzy R Dewantari, Rima R Prasetya, Krisnoadi Rahardjo, Anastasia W Jefuna, Gatot Soegiarto, Laksmi Wulandari, Retno A Setyoningrum, Resti Yudhawati, Yohko K Shimizu, Mitsuhiro Nishimura, Yasuko Mori, Soetjipto, Kazufumi Shimizu, Maria I Lusida
EPI_ISL_458081	RSUD Bangil Pasuruan	Institute of Tropical Disease, Universitas Airlangga	Jezzy R Dewantari, Rima R Prasetya, Krisnoadi Rahardjo, Aldise M Nastri, Arma Roosalina, Gatot Soegiarto, Laksmi Wulandari, Retno A Setyoningrum, Resti Yudhawati, Yohko K Shimizu, Mitsuhiro Nishimura, Yasuko Mori, Soetjipto, Kazufumi Shimizu, Maria I Lusida
EPI_ISL_458082	Universitas Airlangga Hospital	Institute of Tropical Disease, Universitas Airlangga	Kazufumi Shimizu, Krisnoadi Rahardjo, Aldise M Nastri, Jezzy R Dewantari, Rima R Prasetya, Nasronudin, Gatot Soegiarto, Laksmi Wulandari, Retno A Setyoningrum, Resti Yudhawati, Yohko K Shimizu, Mitsuhiro Nishimura, Yasuko Mori, Soetjipto, Maria I Lusida
EPI_ISL_458083	Adi Husada Undaan Hospital	Institute of Tropical Disease, Universitas Airlangga	Rima R Prasetya, Krisnoadi Rahardjo, Aldise M Nastri, Jezzy R Dewantari, Irawati Marga, Gatot Soegiarto, Laksmi Wulandari, Retno A Setyoningrum, Resti Yudhawati, Yohko K Shimizu, Mitsuhiro Nishimura, Yasuko Mori, Soetjipto, Kazufumi Shimizu, Maria I Lusida
EPI_ISL_458086	B.J. Medical College and Civil hospital	Gujarat Biotechnology Research Centre	Dhaval Vaghela, Ramesh Patel, Pranay Shah, Kamlesh J Upadhyay, Ramesh Pandit, Tejas Shah, Ankit Hinsu, Pritesh Sabara, Apurvasinh Puvar, Janvi Raval, Zarna Patel, Monika Gandhi, Pinal Trivedi, Maharshi Pandya, Amit Kanani, Nidhi Patel, Nitin Savaliya, Raghawendra Kumar, Dinesh Kumar, Zuber Saiyed, Komal Patel, Labdhi Pandya, Snehal Bagatharia, Neha Rajpara, Bhavesh Modi, Gaurishankar Shrimali, R D Dixit, A M Kadri, Umang Mishra, Chaitanya Joshi, Madhvi Joshi
EPI_ISL_458087	B.J. Medical College and Civil hospital	Gujarat Biotechnology Research Centre	Ramesh Patel, Pranay Shah, Kamlesh J Upadhyay, Ramesh Pandit, Tejas Shah, Ankit Hinsu, Pritesh Sabara, Apurvasinh Puvar, Janvi Raval, Zarna Patel, Monika Gandhi, Pinal Trivedi, Maharshi Pandya, Amit Kanani, Nidhi Patel, Nitin Savaliya, Raghawendra Kumar, Dinesh Kumar, Zuber Saiyed, Komal Patel, Labdhi Pandya, Snehal Bagatharia, Dhaval Vaghela, Afzal Ansari, Bhavesh Modi, Gaurishankar Shrimali, R D Dixit, A M Kadri, Umang Mishra, Chaitanya Joshi, Madhvi Joshi
EPI_ISL_458088	B.J. Medical College and Civil hospital	Gujarat Biotechnology Research Centre	Pranay Shah, Kamlesh J Upadhyay, Ramesh Pandit, Tejas Shah, Ankit Hinsu, Pritesh Sabara, Apurvasinh Puvar, Janvi Raval, Zarna Patel, Monika Gandhi, Pinal Trivedi, Maharshi Pandya, Amit Kanani, Nidhi Patel, Nitin Savaliya, Raghawendra Kumar, Dinesh Kumar, Zuber Saiyed, Komal Patel, Labdhi Pandya, Snehal Bagatharia, Dhaval Vaghela, Ramesh Patel, Fenil Patel, Bhavesh Modi, Gaurishankar Shrimali, R D Dixit, A M Kadri, Umang Mishra, Chaitanya Joshi, Madhvi Joshi
EPI_ISL_458089	B.J. Medical College and Civil hospital	Gujarat Biotechnology Research Centre	Pranay Shah, Kamlesh J Upadhyay, Ramesh Pandit, Tejas Shah, Ankit Hinsu, Pritesh Sabara, Apurvasinh Puvar, Janvi Raval, Zarna Patel, Monika Gandhi, Pinal Trivedi, Maharshi Pandya, Amit Kanani, Nidhi Patel, Nitin Savaliya, Raghawendra Kumar, Dinesh Kumar, Zuber Saiyed, Komal Patel, Labdhi Pandya, Snehal Bagatharia, Dhaval Vaghela, Ramesh Patel, Neelam Nathani, Bhavesh Modi, Gaurishankar Shrimali, R D Dixit, A M Kadri, Umang Mishra, Chaitanya Joshi, Madhvi Joshi
EPI_ISL_458090	B.J. Medical College and Civil hospital	Gujarat Biotechnology Research Centre	Kamlesh J Upadhyay, Ramesh Pandit, Tejas Shah, Ankit Hinsu, Pritesh Sabara, Apurvasinh Puvar, Janvi Raval, Zarna Patel, Monika Gandhi, Pinal Trivedi, Maharshi Pandya, Amit Kanani, Nidhi Patel, Nitin Savaliya, Raghawendra Kumar, Dinesh Kumar, Zuber Saiyed, Komal Patel, Labdhi Pandya, Snehal Bagatharia, Dhaval Vaghela, Ramesh Patel, Pranay Shah, Armi Chaudhari, Bhavesh Modi, Gaurishankar Shrimali, R D Dixit, A M Kadri, Umang Mishra, Chaitanya Joshi, Madhvi Joshi
EPI_ISL_458091	B.J. Medical College and Civil hospital	Gujarat Biotechnology Research Centre	Maharshi Pandya, Amit Kanani, Nidhi Patel, Nitin Savaliya, Raghawendra Kumar, Dinesh Kumar, Zuber Saiyed, Komal Patel, Labdhi Pandya, Snehal Bagatharia, Dhaval Vaghela, Ramesh Patel, Pranay Shah, Kamlesh J Upadhyay, Ramesh Pandit, Tejas Shah, Ankit Hinsu, Pritesh Sabara, Apurvasinh Puvar, Janvi Raval, Zarna Patel, Monika Gandhi, Pinal Trivedi, Bhavya Jindal, Bhavesh Modi, Gaurishankar Shrimali, R D Dixit, A M Kadri, Umang Mishra,

			Chaitanya Joshi, Madhvi Joshi
EPI_ISL_458092	B.J. Medical College and Civil hospital	Gujarat Biotechnology Research Centre	Amit Kanani, Nidhi Patel, Nitin Savaliya, Raghawendra Kumar, Dinesh Kumar, Zuber Saiyed, Komal Patel, Labdhi Pandya, Snehal Bagatharia, Dhaval Vaghela, Ramesh Patel, Pranay Shah, Kamlesh J Upadhyay, Ramesh Pandit, Tejas Shah, Ankit Hinsu, Pritesh Sabara, Apurvasinh Puvar, Janvi Raval, Zarna Patel, Monika Gandhi, Pinal Trivedi, Maharshi Pandya, Camellia Chakraborty, Bhavesh Modi, Gaurishankar Shrimali, R D Dixit, A M Kadri, Umang Mishra, Chaitanya Joshi, Madhvi Joshi
EPI_ISL_458093	B.J. Medical College and Civil hospital	Gujarat Biotechnology Research Centre	Nidhi Patel, Nitin Savaliya, Raghawendra Kumar, Dinesh Kumar, Zuber Saiyed, Komal Patel, Labdhi Pandya, Snehal Bagatharia, Dhaval Vaghela, Ramesh Patel, Pranay Shah, Kamlesh J Upadhyay, Ramesh Pandit, Tejas Shah, Ankit Hinsu, Pritesh Sabara, Apurvasinh Puvar, Janvi Raval, Zarna Patel, Monika Gandhi, Pinal Trivedi, Maharshi Pandya, Amit Kanani, Siddhant Kumar, Bhavesh Modi, Gaurishankar Shrimali, R D Dixit, A M Kadri, Umang Mishra, Chaitanya Joshi, Madhvi Joshi
EPI_ISL_458094	B.J. Medical College and Civil hospital	Gujarat Biotechnology Research Centre	Nitin Savaliya, Raghawendra Kumar, Dinesh Kumar, Zuber Saiyed, Komal Patel, Labdhi Pandya, Snehal Bagatharia, Dhaval Vaghela, Ramesh Patel, Pranay Shah, Kamlesh J Upadhyay, Ramesh Pandit, Tejas Shah, Ankit Hinsu, Pritesh Sabara, Apurvasinh Puvar, Janvi Raval, Zarna Patel, Monika Gandhi, Pinal Trivedi, Maharshi Pandya, Amit Kanani, Nidhi Patel, Priyanka P Vatsa, Bhavesh Modi, Gaurishankar Shrimali, R D Dixit, A M Kadri, Umang Mishra, Chaitanya Joshi, Madhvi Joshi
EPI_ISL_458095	B.J. Medical College and Civil hospital	Gujarat Biotechnology Research Centre	Raghawendra Kumar, Dinesh Kumar, Zuber Saiyed, Komal Patel, Labdhi Pandya, Snehal Bagatharia, Dhaval Vaghela, Ramesh Patel, Pranay Shah, Kamlesh J Upadhyay, Ramesh Pandit, Tejas Shah, Ankit Hinsu, Pritesh Sabara, Apurvasinh Puvar, Janvi Raval, Zarna Patel, Monika Gandhi, Pinal Trivedi, Maharshi Pandya, Amit Kanani, Nidhi Patel, Nitin Savaliya, Pooja P Doshi, Bhavesh Modi, Gaurishankar Shrimali, R D Dixit, A M Kadri, Umang Mishra, Chaitanya Joshi, Madhvi Joshi
EPI_ISL_458096	B.J. Medical College and Civil hospital	Gujarat Biotechnology Research Centre	Dinesh Kumar, Zuber Saiyed, Komal Patel, Labdhi Pandya, Snehal Bagatharia, Dhaval Vaghela, Ramesh Patel, Pranay Shah, Kamlesh J Upadhyay, Ramesh Pandit, Tejas Shah, Ankit Hinsu, Pritesh Sabara, Apurvasinh Puvar, Janvi Raval, Zarna Patel, Monika Gandhi, Pinal Trivedi, Maharshi Pandya, Amit Kanani, Nidhi Patel, Nitin Savaliya, Raghawendra Kumar, Akanksha Verma, Bhavesh Modi, Gaurishankar Shrimali, R D Dixit, A M Kadri, Umang Mishra, Chaitanya Joshi, Madhvi Joshi
EPI_ISL_458097	B.J. Medical College and Civil hospital	Gujarat Biotechnology Research Centre	Zuber Saiyed, Komal Patel, Labdhi Pandya, Snehal Bagatharia, Dhaval Vaghela, Ramesh Patel, Pranay Shah, Kamlesh J Upadhyay, Ramesh Pandit, Tejas Shah, Ankit Hinsu, Pritesh Sabara, Apurvasinh Puvar, Janvi Raval, Zarna Patel, Monika Gandhi, Pinal Trivedi, Maharshi Pandya, Amit Kanani, Nidhi Patel, Nitin Savaliya, Raghawendra Kumar, Dinesh Kumar, Priti Pandita, Bhavesh Modi, Gaurishankar Shrimali, R D Dixit, A M Kadri, Umang Mishra, Chaitanya Joshi, Madhvi Joshi
EPI_ISL_458098	B.J. Medical College and Civil hospital	Gujarat Biotechnology Research Centre	Komal Patel, Labdhi Pandya, Snehal Bagatharia, Dhaval Vaghela, Ramesh Patel, Pranay Shah, Kamlesh J Upadhyay, Ramesh Pandit, Tejas Shah, Ankit Hinsu, Pritesh Sabara, Apurvasinh Puvar, Janvi Raval, Zarna Patel, Monika Gandhi, Pinal Trivedi, Maharshi Pandya, Amit Kanani, Nidhi Patel, Nitin Savaliya, Raghawendra Kumar, Dinesh Kumar, Zuber Saiyed, Pragya Sharma, Bhavesh Modi, Gaurishankar Shrimali, R D Dixit, A M Kadri, Umang Mishra, Chaitanya Joshi, Madhvi Joshi
EPI_ISL_458099	B.J. Medical College and Civil hospital	Gujarat Biotechnology Research Centre	Labdhi Pandya, Snehal Bagatharia, Dhaval Vaghela, Ramesh Patel, Pranay Shah, Kamlesh J Upadhyay, Ramesh Pandit, Tejas Shah, Ankit Hinsu, Pritesh Sabara, Apurvasinh Puvar, Janvi Raval, Zarna Patel, Monika Gandhi, Pinal Trivedi, Maharshi Pandya, Amit Kanani, Nidhi Patel, Nitin Savaliya, Raghawendra Kumar, Dinesh Kumar, Zuber Saiyed, Komal Patel, Neha Rajpara, Bhavesh Modi, Gaurishankar Shrimali, R D Dixit, A M Kadri, Umang Mishra, Chaitanya Joshi, Madhvi Joshi
EPI_ISL_458100	B.J. Medical College and Civil hospital	Gujarat Biotechnology Research Centre	Snehal Bagatharia, Dhaval Vaghela, Ramesh Patel, Pranay Shah, Kamlesh J Upadhyay, Ramesh Pandit, Tejas Shah, Ankit Hinsu, Pritesh Sabara, Apurvasinh Puvar, Janvi Raval, Zarna Patel, Monika Gandhi, Pinal Trivedi, Maharshi Pandya, Amit Kanani, Nidhi Patel, Nitin Savaliya, Raghawendra Kumar, Dinesh Kumar, Zuber Saiyed, Komal Patel, Labdhi Pandya, Afzal Ansari, Bhavesh Modi, Gaurishankar Shrimali, R D Dixit, A M Kadri, Umang Mishra, Chaitanya Joshi, Madhvi Joshi
EPI_ISL_458101	B.J. Medical College and Civil hospital	Gujarat Biotechnology Research Centre	Dhaval Vaghela, Ramesh Patel, Pranay Shah, Kamlesh J Upadhyay, Ramesh Pandit, Tejas Shah, Ankit Hinsu, Pritesh Sabara, Apurvasinh Puvar, Janvi Raval, Zarna Patel, Monika Gandhi, Pinal Trivedi, Maharshi Pandya, Amit Kanani, Nidhi Patel, Nitin Savaliya, Raghawendra Kumar, Dinesh Kumar, Zuber Saiyed, Komal Patel, Labdhi Pandya, Snehal Bagatharia, Fenil Patel, Bhavesh Modi, Gaurishankar Shrimali, R D Dixit, A M Kadri, Umang Mishra, Chaitanya Joshi, Madhvi Joshi
EPI_ISL_458102	B.J. Medical College and Civil hospital	Gujarat Biotechnology Research Centre	Ramesh Patel, Pranay Shah, Kamlesh J Upadhyay, Ramesh Pandit, Tejas Shah, Ankit Hinsu, Pritesh Sabara, Apurvasinh Puvar, Janvi Raval, Zarna Patel, Monika Gandhi, Pinal Trivedi, Maharshi Pandya, Amit Kanani, Nidhi Patel, Nitin Savaliya, Raghawendra Kumar, Dinesh Kumar, Zuber Saiyed, Komal Patel, Labdhi Pandya, Snehal Bagatharia, Dhaval Vaghela, Neelam Nathani, Bhavesh Modi, Gaurishankar Shrimali, R D Dixit, A M Kadri, Umang Mishra, Chaitanya Joshi, Madhvi Joshi
EPI_ISL_458103	Gujarat Biotechnology Research Centre	Gujarat Biotechnology Research Centre	Ramesh Pandit, Tejas Shah, Ankit Hinsu, Pritesh Sabara, Apurvasinh Puvar, Janvi Raval, Zarna Patel, Monika Gandhi, Pinal Trivedi, Maharshi Pandya, Amit Kanani, Nidhi Patel, Nitin Savaliya, Raghawendra Kumar, Dinesh Kumar, Zuber Saiyed, Komal Patel, Labdhi Pandya, Snehal Bagatharia, Armi Chaudhari, Bhavesh Modi, Gaurishankar Shrimali, R D Dixit, A M Kadri, Umang Mishra, Chaitanya Joshi, Madhvi Joshi, , , , ,
EPI_ISL_458104	Gujarat Biotechnology Research Centre	Gujarat Biotechnology Research Centre	Tejas Shah, Ankit Hinsu, Pritesh Sabara, Apurvasinh Puvar, Janvi Raval, Zarna Patel, Monika Gandhi, Pinal Trivedi, Maharshi Pandya, Amit Kanani, Nidhi Patel, Nitin Savaliya, Raghawendra Kumar, Dinesh Kumar, Zuber Saiyed, Komal Patel, Labdhi Pandya, Snehal Bagatharia, Ramesh Pandit, Bhavya Jindal, Bhavesh Modi, Gaurishankar Shrimali, R D Dixit, A M Kadri, Umang Mishra, Chaitanya Joshi, Madhvi Joshi, , , , ,
EPI_ISL_458105	Gujarat Biotechnology Research Centre	Gujarat Biotechnology Research Centre	Ankit Hinsu, Pritesh Sabara, Apurvasinh Puvar, Janvi Raval, Zarna Patel, Monika Gandhi, Pinal Trivedi, Maharshi Pandya, Amit Kanani, Nidhi Patel, Nitin Savaliya, Raghawendra Kumar, Dinesh Kumar, Zuber Saiyed, Komal Patel, Labdhi Pandya, Snehal Bagatharia, Ramesh Pandit, Tejas Shah, Camellia Chakraborty, Bhavesh Modi, Gaurishankar Shrimali, R D Dixit, A M Kadri, Umang Mishra, Chaitanya Joshi, Madhvi Joshi, , , , ,
EPI_ISL_458106	Gujarat Biotechnology Research Centre	Gujarat Biotechnology Research Centre	Pritesh Sabara, Apurvasinh Puvar, Janvi Raval, Zarna Patel, Monika Gandhi, Pinal Trivedi, Maharshi Pandya, Amit Kanani, Nidhi Patel, Nitin Savaliya, Raghawendra Kumar, Dinesh Kumar, Zuber Saiyed, Komal Patel, Labdhi Pandya, Snehal Bagatharia, Ramesh Pandit, Tejas Shah, Ankit Hinsu, Siddhant Kumar, Bhavesh Modi, Gaurishankar Shrimali, R D Dixit, A M Kadri, Umang Mishra, Chaitanya Joshi, Madhvi Joshi, , , , ,
EPI_ISL_458107	Gujarat Biotechnology Research Centre	Gujarat Biotechnology Research Centre	Apurvasinh Puvar, Janvi Raval, Zarna Patel, Monika Gandhi, Pinal Trivedi, Maharshi Pandya, Amit Kanani, Nidhi Patel, Nitin Savaliya, Raghawendra Kumar, Dinesh Kumar, Zuber Saiyed, Komal Patel, Labdhi Pandya, Snehal Bagatharia, Ramesh Pandit, Tejas Shah, Ankit Hinsu, Pritesh Sabara, Priyanka P Vatsa, Bhavesh Modi, Gaurishankar Shrimali, R D Dixit, A M Kadri, Umang Mishra, Chaitanya Joshi, Madhvi Joshi, , , , ,
EPI_ISL_458111	Gujarat Biotechnology Research Centre	Gujarat Biotechnology Research Centre	Pinal Trivedi, Maharshi Pandya, Amit Kanani, Nidhi Patel, Nitin Savaliya, Raghawendra Kumar, Dinesh Kumar, Zuber Saiyed, Komal Patel, Labdhi Pandya, Snehal Bagatharia, Ramesh Pandit, Tejas Shah, Ankit Hinsu, Pritesh Sabara, Apurvasinh Puvar, Janvi Raval, Zarna Patel, Monika Gandhi, Pragya Sharma, Bhavesh Modi, Gaurishankar Shrimali, R D Dixit, A M Kadri, Umang Mishra, Chaitanya Joshi, Madhvi Joshi, , , , ,
EPI_ISL_458112	Gujarat Biotechnology Research Centre	Gujarat Biotechnology Research Centre	Maharshi Pandya, Amit Kanani, Nidhi Patel, Nitin Savaliya, Raghawendra Kumar, Dinesh Kumar, Zuber Saiyed, Komal Patel, Labdhi Pandya, Snehal Bagatharia, Ramesh Pandit, Tejas Shah, Ankit Hinsu, Pritesh Sabara, Apurvasinh Puvar, Janvi Raval, Zarna Patel, Monika Gandhi, Pinal Trivedi, Neha Rajpara, Bhavesh Modi, Gaurishankar Shrimali, R D Dixit, A M Kadri, Umang Mishra, Chaitanya Joshi, Madhvi Joshi, , , , ,
EPI_ISL_458113	Gujarat Biotechnology Research Centre	Gujarat Biotechnology Research Centre	Amit Kanani, Nidhi Patel, Nitin Savaliya, Raghawendra Kumar, Dinesh Kumar, Zuber Saiyed, Komal Patel, Labdhi Pandya, Snehal Bagatharia, Ramesh Pandit, Tejas Shah, Ankit Hinsu, Pritesh Sabara, Apurvasinh Puvar, Janvi Raval, Zarna Patel, Monika Gandhi, Pinal Trivedi, Maharshi Pandya, Afzal Ansari, Bhavesh Modi, Gaurishankar Shrimali, R D Dixit, A M Kadri, Umang Mishra, Chaitanya Joshi, Madhvi Joshi, , , , ,
EPI_ISL_458116, EPI_ISL_458117, EPI_ISL_458118, EPI_ISL_458120	Oman National Influenza Centre	Department of Microbiology and Immunology-SQUH	Fahad Zadjali, Samira Al-Maruqi, Amina Al Jardani, Khulood Al-Mammary, Hanan Al-kindi, Fatma BaAlawi, Hamida AL Barwani, Zeyana AL-Dahmani, Intisar Al-Shukri, Aisha Al-Busaidi, Aisha Al-Amri, Ahlam Al-Amri, Mohammed Al-Tobi, Samiha Al Kharusi, Abdulla Balkhair
EPI_ISL_458133	National Institute of Biotechnology	Bioinformatics Division, National Institute of Biotechnology	Mohammad Uzzal Hossain, Md. Moniruzzaman, Md. Salim Khan, Md. Nazrul Islam, Md. Hadisur Rahman, Arittra Bhattacharjee, Md. Ruhul Amin, Asif Rashid, Chaman Ara Keya, Keshob Chandra Das, Md. Salimullah
EPI_ISL_458139	Evandro Chagas Institute	Evandro Chagas Institute	Santos, M.C.; Silva, A.M.; Junior, W.D.C.; Barbagelata, L.S.; Ferreira, J.A.; Sousa, E.M.A.; da Silva, P.S.; Resque, H.R; Martins, L.C.; Sousa Junior, E.C.;Viana, G.M.R
EPI_ISL_458236	Hospital Mexico	Charité Virology-University of Costa Rica	Andres Moreira-Soto, Eugenia Corrales-Aguilar, Teresita Somogyi, Jan Felix Drexler

EPI_ISL_459866, EPI_ISL_459867, EPI_ISL_459868, EPI_ISL_459869, EPI_ISL_459871, EPI_ISL_459872, EPI_ISL_459873, EPI_ISL_459874, EPI_ISL_459875, EPI_ISL_459877, EPI_ISL_459878, EPI_ISL_459879, EPI_ISL_459880, EPI_ISL_459881, EPI_ISL_459882, EPI_ISL_459883, EPI_ISL_459884, EPI_ISL_459885, EPI_ISL_459886, EPI_ISL_459887, EPI_ISL_459888, EPI_ISL_459889, EPI_ISL_459890, EPI_ISL_459891, EPI_ISL_459892			
see above	Kingston Health Sciences Center	Queen's Genomics Lab at Ongwanada (Q-GLO)	Sjaarda CP, Rustom N, Huang D, Perez-Patrigeon S, Hudson ML, Wong H,Guan H, Ayub M, Soares CN, Colautti R, Evans GA, Sheth P
EPI_ISL_459910	Zoonotic and Exotic infection Diseases Division, Harbin Veterinary Research Institute, CAAS	Zoonotic and Exotic infection Diseases Division, Harbin Veterinary Resrarch Institute, CAAS	Jinliang Wang, Lei Shuai, Chong Wang, Renqiang Liu, Xijun He, Xianfeng Zhang, Ziruo Sun, Dan Shan, Jinying Ge, Xijun Wang, Gongxun Zhong, Zhiyuan Wen, Zhigao Bu
EPI_ISL_459911	Devki Devi Foundation, a unit of Max Healthcare	CSIR-IGIB/Max	Rajesh Pandey#, Samreen Siddiqui, Pooja Sharma, Bansidhar Tarai, Vivekanand A, Bharathram Uppili, Saruchi Wadhwa, Nishu Tyagi, Mitali Mukerji, Poonam Das, Sujeet Jha, Mohammed Faruq, Vinita Jha, Anurag Agrawal
EPI_ISL_459912, EPI_ISL_459914, EPI_ISL_459915, EPI_ISL_459918, EPI_ISL_459919, EPI_ISL_459920, EPI_ISL_459921, EPI_ISL_459922, EPI_ISL_459923, EPI_ISL_459925, EPI_ISL_459931, EPI_ISL_459932, EPI_ISL_459933, EPI_ISL_459934, EPI_ISL_459935, EPI_ISL_459936, EPI_ISL_459937, EPI_ISL_459938, EPI_ISL_459939, EPI_ISL_459940, EPI_ISL_459941, EPI_ISL_459942, EPI_ISL_459943, EPI_ISL_459944, EPI_ISL_459945, EPI_ISL_459946, EPI_ISL_459947, EPI_ISL_459948, EPI_ISL_459949, EPI_ISL_459950, EPI_ISL_459951, EPI_ISL_459952			
see above	Devki Devi Foundation, a unit of Max Healthcare	CSIR-IGIB/Max	Rajesh Pandey#, Samreen Siddiqui, Pooja Sharma, Bansidhar Tarai, Vivekanand A, Bharathram Uppili, Saruchi Wadhwa, Nishu Tyagi, Mitali Mukerji, Bansidhar Tarai, Poonam Das, Sujeet Jha, Mohammed Faruq, Vinita Jha, Anurag Agrawal
EPI_ISL_459953	Institute for Medical Research, Infectious Disease Research Centre, National Institutes of Health, Ministry of Health Malaysia	Institute for Medical Research Infectious Disease Research Centre, National Institutes of Health, Ministry of Health Malaysia	Suppiah J, Mohd-Zawawi Z, Kamel KA, Ellan K, Kalyanasundram J, Mohd-Zain R, Thayan R
EPI_ISL_459954, EPI_ISL_459955, EPI_ISL_459956	Institute for Medical Research, Infectious Disease Research Centre, National Institutes of Health, Ministry of Health Malaysia	Institute for Medical Research, Infectious Disease Research Centre, National Institutes of Health, Ministry of Health Malaysia	Suppiah J, Mohd-Zawawi Z, Kamel KA, Ellan K, Kalyanasundram J, Mohd-Zain R, Thayan R
EPI_ISL_459962, EPI_ISL_459963, EPI_ISL_459964	Centogene AG	Centogene AG	Prof. Dr. Peter Bauer, Dr. Krishna Kumar Kandaswamy
EPI_ISL_460079	Molecular Virology Unit, Fondazione IRCCS Policlinico San Matteo , Pavia	Laboratory of Virology, INMI Lazzaro Spallanzani IRCCS	Barbara Bartolini, Cesare E.M. Gruber, Maria R. Capobianchi, Martina Rueca, Antonio Piralla, Fausto Baldanti, Antonino Di Caro
EPI_ISL_460080	Molecular Virology Unit, Fondazione IRCCS Policlinico San Matteo , Pavia	Laboratory of Virology, INMI Lazzaro Spallanzani IRCCS	Antonio Piralla, Barbara Bartolini, Fausto Baldanti, Martina Rueca, Antonino Di Caro, Cesare E.M. Gruber, Maria R. Capobianchi
EPI_ISL_460081	Molecular Virology Unit, Fondazione IRCCS Policlinico San Matteo , Pavia	Laboratory of Virology, INMI Lazzaro Spallanzani IRCCS	Fausto Baldanti, Martina Rueca, Antonio Piralla, Antonino Di Caro, Maria R. Capobianchi, Cesare E.M. Gruber, Barbara Bartolini
EPI_ISL_460082	Molecular Virology Unit, Fondazione IRCCS Policlinico San Matteo , Pavia	Laboratory of Virology, INMI Lazzaro Spallanzani IRCCS	Martina Rueca, Cesare E.M. Gruber, Antonio Piralla, Antonino Di Caro, Barbara Bartolini, Maria R. Capobianchi, Fausto Baldanti
EPI_ISL_460083	Molecular Virology Unit, Fondazione IRCCS Policlinico San Matteo , Pavia	Laboratory of Virology, INMI Lazzaro Spallanzani IRCCS	Martina Rueca, Antonino Di Caro, Cesare E.M. Gruber, Barbara Bartolini, Fausto Baldanti, Antonio Piralla, Maria R. Capobianchi
EPI_ISL_460084	Molecular Virology Unit, Fondazione IRCCS Policlinico San Matteo , Pavia	Laboratory of Virology, INMI Lazzaro Spallanzani IRCCS	Fausto Baldanti, Antonio Piralla, Martina Rueca, Barbara Bartolini, Maria R. Capobianchi, Cesare E.M. Gruber, Antonino Di Caro
EPI_ISL_460085	Molecular Virology Unit, Fondazione IRCCS Policlinico San Matteo , Pavia	Laboratory of Virology, INMI Lazzaro Spallanzani IRCCS	Cesare E.M. Gruber, Maria R. Capobianchi, Barbara Bartolini, Fausto Baldanti, Martina Rueca, Antonio Piralla, Antonino Di Caro
EPI_ISL_460086	Molecular Virology Unit, Fondazione IRCCS Policlinico San Matteo , Pavia	Laboratory of Virology, INMI Lazzaro Spallanzani IRCCS	Maria R. Capobianchi, Fausto Baldanti, Antonio Piralla, Antonino Di Caro, Barbara Bartolini, Cesare E.M. Gruber, Martina Rueca
EPI_ISL_460087	Molecular Virology Unit, Fondazione IRCCS Policlinico San Matteo , Pavia	Laboratory of Virology, INMI Lazzaro Spallanzani IRCCS	Cesare E.M. Gruber, Maria R. Capobianchi, Martina Rueca, Barbara Bartolini, Antonino Di Caro, Antonio Piralla, Fausto Baldanti
EPI_ISL_460088	Molecular Virology Unit, Fondazione IRCCS Policlinico San Matteo , Pavia	Laboratory of Virology, INMI Lazzaro Spallanzani IRCCS	Martina Rueca, Barbara Bartolini, Fausto Baldanti, Maria R. Capobianchi, Cesare E.M. Gruber, Antonino Di Caro, Antonio Piralla
EPI_ISL_460089	Molecular Virology Unit, Fondazione IRCCS Policlinico San Matteo , Pavia	Laboratory of Virology, INMI Lazzaro Spallanzani IRCCS	Antonino Di Caro, Barbara Bartolini, Martina Rueca, Cesare E.M. Gruber, Antonio Piralla, Fausto Baldanti, Maria R. Capobianchi
EPI_ISL_460091	Molecular Virology Unit, Fondazione IRCCS Policlinico San Matteo , Pavia	Laboratory of Virology, INMI Lazzaro Spallanzani IRCCS	Antonino Di Caro, Antonio Piralla, Martina Rueca, Fausto Baldanti, Barbara Bartolini, Maria R. Capobianchi, Cesare E.M. Gruber
EPI_ISL_460092	Molecular Virology Unit, Fondazione IRCCS Policlinico San Matteo , Pavia	Laboratory of Virology, INMI Lazzaro Spallanzani IRCCS	Cesare E.M. Gruber, Martina Rueca, Maria R. Capobianchi, Antonino Di Caro, Antonio Piralla, Barbara Bartolini, Fausto Baldanti
EPI_ISL_460093	Molecular Virology Unit, Fondazione IRCCS Policlinico San Matteo , Pavia	Laboratory of Virology, INMI Lazzaro Spallanzani IRCCS	Maria R. Capobianchi, Antonio Piralla, Antonino Di Caro, Fausto Baldanti, Martina Rueca, Cesare E.M. Gruber, Barbara Bartolini
EPI_ISL_460094	Molecular Virology Unit, Fondazione IRCCS Policlinico San Matteo , Pavia	Laboratory of Virology, INMI Lazzaro Spallanzani IRCCS	Barbara Bartolini, Maria R. Capobianchi, Antonino Di Caro, Antonio Piralla, Cesare E.M. Gruber, Martina Rueca, Fausto Baldanti
EPI_ISL_461478	Government Medical College, Vadodara	Gujarat Biotechnology Research Centre	Fenil Patel, Nidhi Patel, Nitin Savaliya, Raghawendra Kumar, Dinesh Kumar, Zuber Saiyed, Komal Patel, Labdhi Pandya, Snehal Bagatharia, Tanuja Javadekar , R N Daveshwar, Tejas Shah, Ankit Hinsu, Pritesh Sabara, Apurvasinh Puvar, Janvi Raval, Zarna Patel, Monika Gandhi, Pinal Trivedi, Maharshi Pandya, R D Dixit, A M Kadri, Harsh Bakshi, Chaitanya Joshi, Madhvi Joshi,
EPI_ISL_461479	Government Medical College, Vadodara	Gujarat Biotechnology Research Centre	Neelam Nathani, Nitin Savaliya, Raghawendra Kumar, Dinesh Kumar, Zuber Saiyed, Komal Patel, Labdhi Pandya, Snehal Bagatharia, Tanuja Javadekar , R N Daveshwar, Tejas Shah, Ankit Hinsu, Pritesh Sabara, Apurvasinh Puvar, Janvi Raval, Zarna Patel, Monika Gandhi, Pinal Trivedi, Maharshi Pandya, Nidhi Patel, R D Dixit, A M Kadri, Harsh Bakshi, Chaitanya Joshi, Madhvi Joshi,
EPI_ISL_461480	Government Medical College, Vadodara	Gujarat Biotechnology Research Centre	Armi Chaudhari, Raghawendra Kumar, Dinesh Kumar, Zuber Saiyed, Komal Patel, Labdhi Pandya, Snehal Bagatharia, Tanuja Javadekar , R N Daveshwar, Tejas Shah, Ankit Hinsu, Pritesh Sabara, Apurvasinh Puvar, Janvi Raval, Zarna Patel, Monika Gandhi, Pinal Trivedi, Maharshi Pandya, Nidhi Patel, Nitin Savaliya, R D Dixit, A M Kadri, Harsh Bakshi, Chaitanya Joshi, Madhvi Joshi,
EPI_ISL_461481	Pandit Deendayal Upadhyay Government Medical College, Rajkot	Gujarat Biotechnology Research Centre	Bhavya Jindal, Dinesh Kumar, Zuber Saiyed, Komal Patel, Labdhi Pandya, Snehal Bagatharia, Prakash Modi, Sejul Antala, Manish Pattani, Tejas Shah, Ankit Hinsu, Pritesh Sabara, Apurvasinh Puvar, Janvi Raval, Zarna Patel, Monika Gandhi, Pinal Trivedi, Maharshi Pandya, Nidhi Patel, Nitin Savaliya, Raghawendra Kumar, R D Dixit, A M Kadri, Harsh Bakshi, Chaitanya Joshi, Madhvi Joshi
EPI_ISL_461482	Pandit Deendayal Upadhyay Government Medical College, Rajkot	Gujarat Biotechnology Research Centre	Anjali Rajwar, Zuber Saiyed, Komal Patel, Labdhi Pandya, Snehal Bagatharia, Prakash Modi, Sejul Antala, Manish Pattani, Tejas Shah, Ankit Hinsu, Pritesh Sabara, Apurvasinh Puvar, Janvi Raval, Zarna Patel, Monika Gandhi, Pinal Trivedi, Maharshi Pandya, Nidhi Patel, Nitin Savaliya, Raghawendra Kumar, Dinesh Kumar, R D Dixit, A M Kadri, Harsh Bakshi, Chaitanya Joshi, Madhvi Joshi
EPI_ISL_461483	B.J. Medical College and Civil hospital	Gujarat Biotechnology Research Centre	Dipeshwari Shewale, Komal Patel, Labdhi Pandya, Snehal Bagatharia, Pranay Shah, Kamlesh J Upadhyay, Tejas Shah, Ankit Hinsu, Pritesh Sabara, Apurvasinh Puvar, Janvi Raval, Zarna Patel, Monika Gandhi, Pinal Trivedi, Maharshi Pandya, Nidhi Patel, Nitin Savaliya, Raghawendra Kumar, Dinesh Kumar, Zuber Saiyed, R D Dixit, A M Kadri, Harsh Bakshi, Chaitanya Joshi, Madhvi Joshi,
EPI_ISL_461484	B.J. Medical College and Civil hospital	Gujarat Biotechnology Research Centre	Priyanka P Vatsa, Labdhi Pandya, Snehal Bagatharia, Pranay Shah, Kamlesh J Upadhyay, Tejas Shah, Ankit Hinsu, Pritesh Sabara, Apurvasinh Puvar, Janvi Raval, Zarna Patel, Monika Gandhi, Pinal Trivedi, Maharshi Pandya, Nidhi Patel, Nitin Savaliya, Raghawendra Kumar, Dinesh Kumar, Zuber Saiyed, Komal Patel, R D Dixit, A M Kadri, Harsh Bakshi, Chaitanya Joshi, Madhvi Joshi,
EPI_ISL_461485	B.J. Medical College and Civil hospital	Gujarat Biotechnology Research Centre	Pooja P Doshi, Snehal Bagatharia, Pranay Shah, Kamlesh J Upadhyay, Tejas Shah, Ankit Hinsu, Pritesh Sabara, Apurvasinh Puvar, Janvi Raval, Zarna Patel, Monika Gandhi, Pinal Trivedi, Maharshi Pandya, Nidhi Patel, Nitin Savaliya, Raghawendra Kumar, Dinesh Kumar, Zuber Saiyed, Komal Patel, Labdhi

			Pandya, R D Dixit, A M Kadri, Harsh Bakshi, Chaitanya Joshi, Madhvi Joshi,
EPI_ISL_461486	B.J. Medical College and Civil hospital	Gujarat Biotechnology Research Centre	Akanksha Verma, Pranay Shah, Kamlesh J Upadhyay, Tejas Shah, Ankit Hinsu, Pritesh Sabara, Apurvasinh Puvar, Janvi Raval, Zarna Patel, Monika Gandhi, Pinal Trivedi, Maharshi Pandya, Nidhi Patel, Nitin Savaliya, Raghawendra Kumar, Dinesh Kumar, Zuber Saiyed, Komal Patel, Labdhi Pandya, Snehal Bagatharia, R D Dixit, A M Kadri, Harsh Bakshi, Chaitanya Joshi, Madhvi Joshi,
EPI_ISL_461487	B.J. Medical College and Civil hospital	Gujarat Biotechnology Research Centre	Priti Pandita, Kamlesh J Upadhyay, Tejas Shah, Ankit Hinsu, Pritesh Sabara, Apurvasinh Puvar, Janvi Raval, Zarna Patel, Monika Gandhi, Pinal Trivedi, Maharshi Pandya, Nidhi Patel, Nitin Savaliya, Raghawendra Kumar, Dinesh Kumar, Zuber Saiyed, Komal Patel, Labdhi Pandya, Snehal Bagatharia, Pranay Shah, R D Dixit, A M Kadri, Harsh Bakshi, Chaitanya Joshi, Madhvi Joshi,
EPI_ISL_461488	B.J. Medical College and Civil hospital	Gujarat Biotechnology Research Centre	Pragya Sharma, Tejas Shah, Ankit Hinsu, Pritesh Sabara, Apurvasinh Puvar, Janvi Raval, Zarna Patel, Monika Gandhi, Pinal Trivedi, Maharshi Pandya, Nidhi Patel, Nitin Savaliya, Raghawendra Kumar, Dinesh Kumar, Zuber Saiyed, Komal Patel, Labdhi Pandya, Snehal Bagatharia, Pranay Shah, Kamlesh J Upadhyay, R D Dixit, A M Kadri, Harsh Bakshi, Chaitanya Joshi, Madhvi Joshi,
EPI_ISL_461489	B.J. Medical College and Civil hospital	Gujarat Biotechnology Research Centre	Neha Rajpara, Ankit Hinsu, Pritesh Sabara, Apurvasinh Puvar, Janvi Raval, Zarna Patel, Monika Gandhi, Pinal Trivedi, Maharshi Pandya, Nidhi Patel, Nitin Savaliya, Raghawendra Kumar, Dinesh Kumar, Zuber Saiyed, Komal Patel, Labdhi Pandya, Snehal Bagatharia, Pranay Shah, Kamlesh J Upadhyay, Tejas Shah, R D Dixit, A M Kadri, Harsh Bakshi, Chaitanya Joshi, Madhvi Joshi,
EPI_ISL_461490	B.J. Medical College and Civil hospital	Gujarat Biotechnology Research Centre	Afzal Ansari, Pritesh Sabara, Apurvasinh Puvar, Janvi Raval, Zarna Patel, Monika Gandhi, Pinal Trivedi, Maharshi Pandya, Nidhi Patel, Nitin Savaliya, Raghawendra Kumar, Dinesh Kumar, Zuber Saiyed, Komal Patel, Labdhi Pandya, Snehal Bagatharia, Pranay Shah, Kamlesh J Upadhyay, Tejas Shah, Ankit Hinsu, R D Dixit, A M Kadri, Harsh Bakshi, Chaitanya Joshi, Madhvi Joshi,
EPI_ISL_461491	B.J. Medical College and Civil hospital	Gujarat Biotechnology Research Centre	Fenil Patel, Apurvasinh Puvar, Janvi Raval, Zarna Patel, Monika Gandhi, Pinal Trivedi, Maharshi Pandya, Nidhi Patel, Nitin Savaliya, Raghawendra Kumar, Dinesh Kumar, Zuber Saiyed, Komal Patel, Labdhi Pandya, Snehal Bagatharia, Pranay Shah, Kamlesh J Upadhyay, Tejas Shah, Ankit Hinsu, Pritesh Sabara, R D Dixit, A M Kadri, Harsh Bakshi, Chaitanya Joshi, Madhvi Joshi,
EPI_ISL_461492	B.J. Medical College and Civil hospital	Gujarat Biotechnology Research Centre	Neelam Nathani, Janvi Raval, Zarna Patel, Monika Gandhi, Pinal Trivedi, Maharshi Pandya, Nidhi Patel, Nitin Savaliya, Raghawendra Kumar, Dinesh Kumar, Zuber Saiyed, Komal Patel, Labdhi Pandya, Snehal Bagatharia, Pranay Shah, Kamlesh J Upadhyay, Tejas Shah, Ankit Hinsu, Pritesh Sabara, Apurvasinh Puvar, R D Dixit, A M Kadri, Harsh Bakshi, Chaitanya Joshi, Madhvi Joshi,
EPI_ISL_461493	B.J. Medical College and Civil hospital	Gujarat Biotechnology Research Centre	Armi Chaudhari, Zarna Patel, Monika Gandhi, Pinal Trivedi, Maharshi Pandya, Nidhi Patel, Nitin Savaliya, Raghawendra Kumar, Dinesh Kumar, Zuber Saiyed, Komal Patel, Labdhi Pandya, Snehal Bagatharia, Pranay Shah, Kamlesh J Upadhyay, Tejas Shah, Ankit Hinsu, Pritesh Sabara, Apurvasinh Puvar, Janvi Raval, R D Dixit, A M Kadri, Harsh Bakshi, Chaitanya Joshi, Madhvi Joshi,
EPI_ISL_461494	B.J. Medical College and Civil hospital	Gujarat Biotechnology Research Centre	Bhavya Jindal, Monika Gandhi, Pinal Trivedi, Maharshi Pandya, Nidhi Patel, Nitin Savaliya, Raghawendra Kumar, Dinesh Kumar, Zuber Saiyed, Komal Patel, Labdhi Pandya, Snehal Bagatharia, Pranay Shah, Kamlesh J Upadhyay, Tejas Shah, Ankit Hinsu, Pritesh Sabara, Apurvasinh Puvar, Janvi Raval, Zarna Patel, R D Dixit, A M Kadri, Harsh Bakshi, Chaitanya Joshi, Madhvi Joshi,
EPI_ISL_461495	B.J. Medical College and Civil hospital	Gujarat Biotechnology Research Centre	Anjali Rajwar, Pinal Trivedi, Maharshi Pandya, Nidhi Patel, Nitin Savaliya, Raghawendra Kumar, Dinesh Kumar, Zuber Saiyed, Komal Patel, Labdhi Pandya, Snehal Bagatharia, Pranay Shah, Kamlesh J Upadhyay, Tejas Shah, Ankit Hinsu, Pritesh Sabara, Apurvasinh Puvar, Janvi Raval, Zarna Patel, Monika Gandhi, R D Dixit, A M Kadri, Harsh Bakshi, Chaitanya Joshi, Madhvi Joshi,
EPI_ISL_461496	B.J. Medical College and Civil hospital	Gujarat Biotechnology Research Centre	Dipeshwari Shewale, Maharshi Pandya, Nidhi Patel, Nitin Savaliya, Raghawendra Kumar, Dinesh Kumar, Zuber Saiyed, Komal Patel, Labdhi Pandya, Snehal Bagatharia, Pranay Shah, Kamlesh J Upadhyay, Tejas Shah, Ankit Hinsu, Pritesh Sabara, Apurvasinh Puvar, Janvi Raval, Zarna Patel, Monika Gandhi, Pinal Trivedi, R D Dixit, A M Kadri, Harsh Bakshi, Chaitanya Joshi, Madhvi Joshi,
EPI_ISL_461497	B.J. Medical College and Civil hospital	Gujarat Biotechnology Research Centre	Priyanka P Vatsa, Nidhi Patel, Nitin Savaliya, Raghawendra Kumar, Dinesh Kumar, Zuber Saiyed, Komal Patel, Labdhi Pandya, Snehal Bagatharia, Pranay Shah, Kamlesh J Upadhyay, Tejas Shah, Ankit Hinsu, Pritesh Sabara, Apurvasinh Puvar, Janvi Raval, Zarna Patel, Monika Gandhi, Pinal Trivedi, Maharshi Pandya, R D Dixit, A M Kadri, Harsh Bakshi, Chaitanya Joshi, Madhvi Joshi,
EPI_ISL_461498	B.J. Medical College and Civil hospital	Gujarat Biotechnology Research Centre	Pooja P Doshi, Nitin Savaliya, Raghawendra Kumar, Dinesh Kumar, Zuber Saiyed, Komal Patel, Labdhi Pandya, Snehal Bagatharia, Pranay Shah, Kamlesh J Upadhyay, Tejas Shah, Ankit Hinsu, Pritesh Sabara, Apurvasinh Puvar, Janvi Raval, Zarna Patel, Monika Gandhi, Pinal Trivedi, Maharshi Pandya, Nidhi Patel, R D Dixit, A M Kadri, Harsh Bakshi, Chaitanya Joshi, Madhvi Joshi,
EPI_ISL_461499	B.J. Medical College and Civil hospital	Gujarat Biotechnology Research Centre	Akanksha Verma, Raghawendra Kumar, Dinesh Kumar, Zuber Saiyed, Komal Patel, Labdhi Pandya, Snehal Bagatharia, Pranay Shah, Kamlesh J Upadhyay, Tejas Shah, Ankit Hinsu, Pritesh Sabara, Apurvasinh Puvar, Janvi Raval, Zarna Patel, Monika Gandhi, Pinal Trivedi, Maharshi Pandya, Nidhi Patel, Nitin Savaliya, R D Dixit, A M Kadri, Harsh Bakshi, Chaitanya Joshi, Madhvi Joshi,
EPI_ISL_461500	B.J. Medical College and Civil hospital	Gujarat Biotechnology Research Centre	Priti Pandita, Dinesh Kumar, Zuber Saiyed, Komal Patel, Labdhi Pandya, Snehal Bagatharia, Pranay Shah, Kamlesh J Upadhyay, Tejas Shah, Ankit Hinsu, Pritesh Sabara, Apurvasinh Puvar, Janvi Raval, Zarna Patel, Monika Gandhi, Pinal Trivedi, Maharshi Pandya, Nidhi Patel, Nitin Savaliya, Raghawendra Kumar, R D Dixit, A M Kadri, Harsh Bakshi, Chaitanya Joshi, Madhvi Joshi,
EPI_ISL_461501	B.J. Medical College and Civil hospital	Gujarat Biotechnology Research Centre	Pragya Sharma, Zuber Saiyed, Komal Patel, Labdhi Pandya, Snehal Bagatharia, Pranay Shah, Kamlesh J Upadhyay, Tejas Shah, Ankit Hinsu, Pritesh Sabara, Apurvasinh Puvar, Janvi Raval, Zarna Patel, Monika Gandhi, Pinal Trivedi, Maharshi Pandya, Nidhi Patel, Nitin Savaliya, Raghawendra Kumar, Dinesh Kumar, R D Dixit, A M Kadri, Harsh Bakshi, Chaitanya Joshi, Madhvi Joshi,
EPI_ISL_461502	B.J. Medical College and Civil hospital	Gujarat Biotechnology Research Centre	Neha Rajpara, Komal Patel, Labdhi Pandya, Snehal Bagatharia, Pranay Shah, Kamlesh J Upadhyay, Tejas Shah, Ankit Hinsu, Pritesh Sabara, Apurvasinh Puvar, Janvi Raval, Zarna Patel, Monika Gandhi, Pinal Trivedi, Maharshi Pandya, Nidhi Patel, Nitin Savaliya, Raghawendra Kumar, Dinesh Kumar, Zuber Saiyed, R D Dixit, A M Kadri, Harsh Bakshi, Chaitanya Joshi, Madhvi Joshi,
EPI_ISL_461503	B.J. Medical College and Civil hospital	Gujarat Biotechnology Research Centre	Afzal Ansari, Labdhi Pandya, Snehal Bagatharia, Pranay Shah, Kamlesh J Upadhyay, Tejas Shah, Ankit Hinsu, Pritesh Sabara, Apurvasinh Puvar, Janvi Raval, Zarna Patel, Monika Gandhi, Pinal Trivedi, Maharshi Pandya, Nidhi Patel, Nitin Savaliya, Raghawendra Kumar, Dinesh Kumar, Zuber Saiyed, Komal Patel, R D Dixit, A M Kadri, Harsh Bakshi, Chaitanya Joshi, Madhvi Joshi,
EPI_ISL_461504	B.J. Medical College and Civil hospital	Gujarat Biotechnology Research Centre	Snehal Bagatharia, Pranay Shah, Kamlesh J Upadhyay, Tejas Shah, Ankit Hinsu, Pritesh Sabara, Apurvasinh Puvar, Janvi Raval, Zarna Patel, Monika Gandhi, Pinal Trivedi, Maharshi Pandya, Nidhi Patel, Nitin Savaliya, Raghawendra Kumar, Dinesh Kumar, Zuber Saiyed, Komal Patel, Labdhi Pandya, Fenil Patel, R D Dixit, A M Kadri, Harsh Bakshi, Chaitanya Joshi, Madhvi Joshi,
EPI_ISL_461505	B.J. Medical College and Civil hospital	Gujarat Biotechnology Research Centre	Pranay Shah, Kamlesh J Upadhyay, Tejas Shah, Ankit Hinsu, Pritesh Sabara, Apurvasinh Puvar, Janvi Raval, Zarna Patel, Monika Gandhi, Pinal Trivedi, Maharshi Pandya, Nidhi Patel, Nitin Savaliya, Raghawendra Kumar, Dinesh Kumar, Zuber Saiyed, Komal Patel, Labdhi Pandya, Snehal Bagatharia, Neelam Nathani, R D Dixit, A M Kadri, Harsh Bakshi, Chaitanya Joshi, Madhvi Joshi,
EPI_ISL_461506	B.J. Medical College and Civil hospital	Gujarat Biotechnology Research Centre	Kamlesh J Upadhyay, Tejas Shah, Ankit Hinsu, Pritesh Sabara, Apurvasinh Puvar, Janvi Raval, Zarna Patel, Monika Gandhi, Pinal Trivedi, Maharshi Pandya, Nidhi Patel, Nitin Savaliya, Raghawendra Kumar, Dinesh Kumar, Zuber Saiyed, Komal Patel, Labdhi Pandya, Snehal Bagatharia, Pranay Shah, Armi Chaudhari, R D Dixit, A M Kadri, Harsh Bakshi, Chaitanya Joshi, Madhvi Joshi,
EPI_ISL_462085, EPI_ISL_462086, EPI_ISL_462087, EPI_ISL_462088, EPI_ISL_462089	Singapore General Hospital	Department of Microbiology	Nurdyana Abdul Rahman, Kun Lee Lim, Chenhao Li, Kian Sing Chan, Lynette Oon, Kern Rei Chng, Niranjan Nagarajan, Karrie Ko
EPI_ISL_462447, EPI_ISL_462448	Fundació Lluita contra la SIDA (FLSida)/Hospital Universitari Germans Trias i Pujol	IrsiCaixa AIDS Research Lab	Marc Noguera-Julian, Mariona Parera, Maria Pilar Armengol, Marc Corbacho, Maria Ubals, Oriol Mitjà, Lidia Ruiz, Nuria Izquierdo, Jorge Carrillo, Roger Paredes, Julia Blanco, Joaquim Segalés, Bonaventura Clotet
EPI_ISL_462449	Fundació Lluita contra la SIDA (FLSida)/Hospital Universitari Germans Trias i Pujol	IrsiCaixa AIDS Research Lab	Marc Noguera-Julian, Mariona Parera, Maria Pilar Armengol, Marc Corbacho, Maria Ubals, Oriol Mitjà, Lidia Ruiz, Nuria Izquierdo, Jorge Carrillo, Roger Paredes, Julia Blanco, Bonaventura Clotet
EPI_ISL_462477	Hospital Costa del Sol	Instituto de Salud Carlos III	Iglesias-Caballero, M. Molinero Calamita, M. González-Esguevillas, M. Camarero, S. Pozo, F. Casas, I. Jiménez, P. Jiménez, M. Zaballos, A. Monzón, S. Varona, S. Juliá, M. Cuesta, I, F. Fernández
EPI_ISL_462478	Fundación Jiménez Díaz	Instituto de Salud Carlos III	Iglesias-Caballero, M. Molinero Calamita, M. González-Esguevillas, M. Camarero, S. Pozo, F. Casas, I. Jiménez, P. Jiménez, M. Zaballos, A. Monzón, S.

			Varona, S. Juliá, M. Cuesta, I, R. Fernández
EPI_ISL_462479	Hospital Clinic	Instituto de Salud Carlos III	Iglesias-Caballero, M. Molinero Calamita, M. González-Esguevillas, M. Camarero, S. Pozo, F. Casas, I. Jiménez, P. Jiménez, M. Zaballos, A. Monzón, S. Varona, S. Juliá, M. Cuesta, I, M.A Marcos
EPI_ISL_462753	University Clinical Hospital of Mostar	University of Sarajevo Veterinary Faculty	Goletic, T., Softic, A., Goletic, S., Ostojic, M., Hukic, M., Eterovic, T., Seho-Alic, A.
EPI_ISL_462990	University Clinical Centre of the Republic of Srpska	University of Sarajevo, Veterinary Faculty	Teufik, G., Šejla, G., Toni, E., Maja, T., Mirsada, H., Aida, K., Alma, Š.A.
EPI_ISL_463007	Department of Laboratory Medicine, National Taiwan University Hospital	Microbial Genomics Core Lab, National Taiwan University Centers of Genomic and Precision Medicine	Shiou-Hwei Yeh, You-Yu Lin, Ya-Yun Lai, Chiao-Ling Li, Shan-Chwen Chang, Pei-Jer Chen, Sui-Yuan Chang
EPI_ISL_463008	Institute of Molecular Virology, University Münster	Institute of Molecular Virology, University Münster	Angeles Mecate Zambrano, Linda Brunotte, Stephan Ludwig, Joachim Kühn, Alexander Mellmann
EPI_ISL_463277, EPI_ISL_463278, EPI_ISL_463279, EPI_ISL_463280, EPI_ISL_463281, EPI_ISL_463282, EPI_ISL_463283, EPI_ISL_463284, EPI_ISL_463285, EPI_ISL_463286, EPI_ISL_463287, EPI_ISL_463288, EPI_ISL_463289, EPI_ISL_463290, EPI_ISL_463291, EPI_ISL_463292, EPI_ISL_463293, EPI_ISL_463294, EPI_ISL_463295, EPI_ISL_463296, EPI_ISL_463297, EPI_ISL_463298, EPI_ISL_463299, EPI_ISL_463300			
see above	Ochsner Health	Bioinfoexperts, LLC	Susanna L. Lamers, David J. Nolan, Rebecca Rose, Sissy Cross, David Moraga Amador, Tong Yang, Luke Caruso, Wayra Navia, Lydia Von Borstel, Xiao Hui Zhou, Amy Feehan, Julia-Garcia-Diaz
EPI_ISL_463741, EPI_ISL_463742, EPI_ISL_463743, EPI_ISL_463744, EPI_ISL_463745, EPI_ISL_463746, EPI_ISL_463747, EPI_ISL_463748	Department of Molecular Virology, Cyprus Institute of Neurology and Genetics	Department of Molecular Virology, Cyprus Institute of Neurology and Genetics	Jan Richter, George Krashias, Christina Tryfonos, Stavros Bashiardes, Dana Koptides, Christina Christodoulou
EPI_ISL_463893	University Clinical Center Tuzla	Alea Genetiki Centar	Konjhodži,R;Salihefendi,L;Goleti,T;Pear,D;Tihi,N;Marjanovi,D;Huki,M.
EPI_ISL_467029	GMERS Medical College and Hospital, Gandhinagar	Gujarat Biotechnology Research Centre	Seema Bhatt, Gaurishankar Shrimali, Bhavesh Modi, Bharti Rajani, Tejas Shah, Ankit Hinsu, Pritesh Sabara, Apurvasinh Puvar, Janvi Raval, Zarna Patel, Monika Gandhi, Pinal Trivedi, Maharshi Pandya, Nidhi Patel, Nitin Savaliya, Raghawendra Kumar, Dinesh Kumar, Zuber Saiyed, Komal Patel, Labdhi Pandya, Snehal Bagatharia, Bhavya Jindal, R D Dixit, A M Kadri, Harsh Bakshi, Chaitanya Joshi, Madhvi Joshi
EPI_ISL_467030	GMERS Medical College and Hospital, Gandhinagar	Gujarat Biotechnology Research Centre	Gaurishankar Shrimali, Bhavesh Modi, Bharti Rajani, Tejas Shah, Ankit Hinsu, Pritesh Sabara, Apurvasinh Puvar, Janvi Raval, Zarna Patel, Monika Gandhi, Pinal Trivedi, Maharshi Pandya, Nidhi Patel, Nitin Savaliya, Raghawendra Kumar, Dinesh Kumar, Zuber Saiyed, Komal Patel, Labdhi Pandya, Snehal Bagatharia, Seema Bhatt, Priyanka P Vatsa, R D Dixit, A M Kadri, Harsh Bakshi, Chaitanya Joshi, Madhvi Joshi
EPI_ISL_467031	GMERS Medical College and Hospital, Gandhinagar	Gujarat Biotechnology Research Centre	Bhavesh Modi, Bharti Rajani, Tejas Shah, Ankit Hinsu, Pritesh Sabara, Apurvasinh Puvar, Janvi Raval, Zarna Patel, Monika Gandhi, Pinal Trivedi, Maharshi Pandya, Nidhi Patel, Nitin Savaliya, Raghawendra Kumar, Dinesh Kumar, Zuber Saiyed, Komal Patel, Labdhi Pandya, Snehal Bagatharia, Seema Bhatt, Gaurishankar Shrimali, Pooja P Doshi, R D Dixit, A M Kadri, Harsh Bakshi, Chaitanya Joshi, Madhvi Joshi
EPI_ISL_467032	GMERS Medical College and Hospital, Gandhinagar	Gujarat Biotechnology Research Centre	Bharti Rajani, Tejas Shah, Ankit Hinsu, Pritesh Sabara, Apurvasinh Puvar, Janvi Raval, Zarna Patel, Monika Gandhi, Pinal Trivedi, Maharshi Pandya, Nidhi Patel, Nitin Savaliya, Raghawendra Kumar, Dinesh Kumar, Zuber Saiyed, Komal Patel, Labdhi Pandya, Snehal Bagatharia, Seema Bhatt, Gaurishankar Shrimali, Bhavesh Modi, Akanksha Verma, R D Dixit, A M Kadri, Harsh Bakshi, Chaitanya Joshi, Madhvi Joshi
EPI_ISL_467033	GMERS Medical College and Hospital, Gandhinagar	Gujarat Biotechnology Research Centre	Tejas Shah, Ankit Hinsu, Pritesh Sabara, Apurvasinh Puvar, Janvi Raval, Zarna Patel, Monika Gandhi, Pinal Trivedi, Maharshi Pandya, Nidhi Patel, Nitin Savaliya, Raghawendra Kumar, Dinesh Kumar, Zuber Saiyed, Komal Patel, Labdhi Pandya, Snehal Bagatharia, Seema Bhatt, Gaurishankar Shrimali, Bhavesh Modi, Bharti Rajani, Priti Pandita, R D Dixit, A M Kadri, Harsh Bakshi, Chaitanya Joshi, Madhvi Joshi
EPI_ISL_467034	GMERS Medical College and Hospital, Gandhinagar	Gujarat Biotechnology Research Centre	Ankit Hinsu, Pritesh Sabara, Apurvasinh Puvar, Janvi Raval, Zarna Patel, Monika Gandhi, Pinal Trivedi, Maharshi Pandya, Nidhi Patel, Nitin Savaliya, Raghawendra Kumar, Dinesh Kumar, Zuber Saiyed, Komal Patel, Labdhi Pandya, Snehal Bagatharia, Seema Bhatt, Gaurishankar Shrimali, Bhavesh Modi, Bharti Rajani, Tejas Shah, Pragya Sharma, R D Dixit, A M Kadri, Harsh Bakshi, Chaitanya Joshi, Madhvi Joshi
EPI_ISL_467035	GMERS Medical College and Hospital, Gandhinagar	Gujarat Biotechnology Research Centre	Pritesh Sabara, Apurvasinh Puvar, Janvi Raval, Zarna Patel, Monika Gandhi, Pinal Trivedi, Maharshi Pandya, Nidhi Patel, Nitin Savaliya, Raghawendra Kumar, Dinesh Kumar, Zuber Saiyed, Komal Patel, Labdhi Pandya, Snehal Bagatharia, Seema Bhatt, Gaurishankar Shrimali, Bhavesh Modi, Bharti Rajani, Tejas Shah, Ankit Hinsu, Neha Rajpara, R D Dixit, A M Kadri, Harsh Bakshi, Chaitanya Joshi, Madhvi Joshi
EPI_ISL_467036	GMERS Medical College and Hospital, Gandhinagar	Gujarat Biotechnology Research Centre	Apurvasinh Puvar, Janvi Raval, Zarna Patel, Monika Gandhi, Pinal Trivedi, Maharshi Pandya, Nidhi Patel, Nitin Savaliya, Raghawendra Kumar, Dinesh Kumar, Zuber Saiyed, Komal Patel, Labdhi Pandya, Snehal Bagatharia, Seema Bhatt, Gaurishankar Shrimali, Bhavesh Modi, Bharti Rajani, Tejas Shah, Ankit Hinsu, Pritesh Sabara, Afzal Ansari, R D Dixit, A M Kadri, Harsh Bakshi, Chaitanya Joshi, Madhvi Joshi
EPI_ISL_467037	GMERS Medical College and Hospital, Gandhinagar	Gujarat Biotechnology Research Centre	Janvi Raval, Zarna Patel, Monika Gandhi, Pinal Trivedi, Maharshi Pandya, Nidhi Patel, Nitin Savaliya, Raghawendra Kumar, Dinesh Kumar, Zuber Saiyed, Komal Patel, Labdhi Pandya, Snehal Bagatharia, Seema Bhatt, Gaurishankar Shrimali, Bhavesh Modi, Bharti Rajani, Tejas Shah, Ankit Hinsu, Pritesh Sabara, Apurvasinh Puvar, Fenil Patel, R D Dixit, A M Kadri, Harsh Bakshi, Chaitanya Joshi, Madhvi Joshi
EPI_ISL_467038	GMERS Medical College and Hospital, Gandhinagar	Gujarat Biotechnology Research Centre	Zarna Patel, Monika Gandhi, Pinal Trivedi, Maharshi Pandya, Nidhi Patel, Nitin Savaliya, Raghawendra Kumar, Dinesh Kumar, Zuber Saiyed, Komal Patel, Labdhi Pandya, Snehal Bagatharia, Seema Bhatt, Gaurishankar Shrimali, Bhavesh Modi, Bharti Rajani, Tejas Shah, Ankit Hinsu, Pritesh Sabara, Apurvasinh Puvar, Janvi Raval, Neelam Nathani, R D Dixit, A M Kadri, Harsh Bakshi, Chaitanya Joshi, Madhvi Joshi
EPI_ISL_467039	Government Medical College, Vadodara	Gujarat Biotechnology Research Centre	Meenakshi Shah, Neena Doshi, Varsha Godbole, Tejas Shah, Ankit Hinsu, Pritesh Sabara, Apurvasinh Puvar, Janvi Raval, Zarna Patel, Monika Gandhi, Pinal Trivedi, Maharshi Pandya, Nidhi Patel, Nitin Savaliya, Raghawendra Kumar, Dinesh Kumar, Zuber Saiyed, Komal Patel, Labdhi Pandya, Snehal Bagatharia, Armi Chaudhari, R D Dixit, A M Kadri, Harsh Bakshi, Chaitanya Joshi, Madhvi Joshi,
EPI_ISL_467040	Government Medical College, Vadodara	Gujarat Biotechnology Research Centre	Neena Doshi, Varsha Godbole, Tejas Shah, Ankit Hinsu, Pritesh Sabara, Apurvasinh Puvar, Janvi Raval, Zarna Patel, Monika Gandhi, Pinal Trivedi, Maharshi Pandya, Nidhi Patel, Nitin Savaliya, Raghawendra Kumar, Dinesh Kumar, Zuber Saiyed, Komal Patel, Labdhi Pandya, Snehal Bagatharia, Meenakshi Shah, Bhavya Jindal, R D Dixit, A M Kadri, Harsh Bakshi, Chaitanya Joshi, Madhvi Joshi,
EPI_ISL_467041	B.J. Medical College and Civil hospital	Gujarat Biotechnology Research Centre	Monika Gandhi, Pinal Trivedi, Maharshi Pandya, Nidhi Patel, Nitin Savaliya, Raghawendra Kumar, Dinesh Kumar, Zuber Saiyed, Komal Patel, Labdhi Pandya, Snehal Bagatharia, Pranay Shah, Kamlesh J Upadhyay, Nirav Mungalpara, Tejas Shah, Ankit Hinsu, Pritesh Sabara, Apurvasinh Puvar, Janvi Raval, Zarna Patel, Priyanka P Vatsa, R D Dixit, A M Kadri, Harsh Bakshi, Chaitanya Joshi, Madhvi Joshi,
EPI_ISL_467042	B.J. Medical College and Civil hospital	Gujarat Biotechnology Research Centre	Pinal Trivedi, Maharshi Pandya, Nidhi Patel, Nitin Savaliya, Raghawendra Kumar, Dinesh Kumar, Zuber Saiyed, Komal Patel, Labdhi Pandya, Snehal Bagatharia, Pranay Shah, Kamlesh J Upadhyay, Nirav Mungalpara, Tejas Shah, Ankit Hinsu, Pritesh Sabara, Apurvasinh Puvar, Janvi Raval, Zarna Patel, Monika Gandhi, Pooja P Doshi, R D Dixit, A M Kadri, Harsh Bakshi, Chaitanya Joshi, Madhvi Joshi,
EPI_ISL_467043	B.J. Medical College and Civil hospital	Gujarat Biotechnology Research Centre	Maharshi Pandya, Nidhi Patel, Nitin Savaliya, Raghawendra Kumar, Dinesh Kumar, Zuber Saiyed, Komal Patel, Labdhi Pandya, Snehal Bagatharia, Pranay Shah, Kamlesh J Upadhyay, Nirav Mungalpara, Tejas Shah, Ankit Hinsu, Pritesh Sabara, Apurvasinh Puvar, Janvi Raval, Zarna Patel, Monika Gandhi, Pinal Trivedi, Akanksha Verma, R D Dixit, A M Kadri, Harsh Bakshi, Chaitanya Joshi, Madhvi Joshi,
EPI_ISL_467044	B.J. Medical College and Civil hospital	Gujarat Biotechnology Research Centre	Nidhi Patel, Nitin Savaliya, Raghawendra Kumar, Dinesh Kumar, Zuber Saiyed, Komal Patel, Labdhi Pandya, Snehal Bagatharia, Pranay Shah, Kamlesh J Upadhyay, Nirav Mungalpara, Tejas Shah, Ankit Hinsu, Pritesh Sabara, Apurvasinh Puvar, Janvi Raval, Zarna Patel, Monika Gandhi, Pinal Trivedi, Maharshi Pandya, Priti Pandita, R D Dixit, A M Kadri, Harsh Bakshi, Chaitanya Joshi, Madhvi Joshi,
EPI_ISL_467045	B.J. Medical College and Civil hospital	Gujarat Biotechnology Research Centre	Nitin Savaliya, Raghawendra Kumar, Dinesh Kumar, Zuber Saiyed, Komal Patel, Labdhi Pandya, Snehal Bagatharia, Pranay Shah, Kamlesh J Upadhyay, Nirav Mungalpara, Tejas Shah, Ankit Hinsu, Pritesh Sabara, Apurvasinh Puvar, Janvi Raval, Zarna Patel, Monika Gandhi, Pinal Trivedi, Maharshi Pandya, Nidhi Patel, Pragya Sharma, R D Dixit, A M Kadri, Harsh Bakshi, Chaitanya Joshi, Madhvi Joshi,
EPI_ISL_467046	B.J. Medical College and Civil hospital	Gujarat Biotechnology Research Centre	Raghawendra Kumar, Dinesh Kumar, Zuber Saiyed, Komal Patel, Labdhi Pandya, Snehal Bagatharia, Pranay Shah, Kamlesh J Upadhyay, Nirav Mungalpara, Tejas Shah, Ankit Hinsu, Pritesh Sabara, Apurvasinh Puvar, Janvi Raval, Zarna Patel, Monika Gandhi, Pinal Trivedi, Maharshi Pandya, Nidhi Patel, Nitin Savaliya, Neha Rajpara, R D Dixit, A M Kadri, Harsh Bakshi, Chaitanya Joshi, Madhvi Joshi,
EPI_ISL_467047	B.J. Medical College and Civil hospital	Gujarat Biotechnology Research Centre	Dinesh Kumar, Zuber Saiyed, Komal Patel, Labdhi Pandya, Snehal Bagatharia, Pranay Shah, Kamlesh J Upadhyay, Nirav Mungalpara, Tejas Shah, Ankit Hinsu, Pritesh Sabara, Apurvasinh Puvar, Janvi Raval, Zarna Patel, Monika Gandhi, Pinal Trivedi, Maharshi Pandya, Nidhi Patel, Nitin Savaliya,

			Raghawendra Kumar, Afzal Ansari, R D Dixit, A M Kadri, Harsh Bakshi, Chaitanya Joshi, Madhvi Joshi,
EPI_ISL_467048	B.J. Medical College and Civil hospital	Gujarat Biotechnology Research Centre	Zuber Saiyed, Komal Patel, Labdhi Pandya, Snehal Bagatharia, Pranay Shah, Kamlesh J Upadhyay, Nirav Mungalpara, Tejas Shah, Ankit Hinsu, Pritesh Sabara, Apurvasinh Puvar, Janvi Raval, Zarna Patel, Monika Gandhi, Pinal Trivedi, Maharshi Pandya, Nidhi Patel, Nitin Savaliya, Raghawendra Kumar, Dinesh Kumar, Fenil Patel, R D Dixit, A M Kadri, Harsh Bakshi, Chaitanya Joshi, Madhvi Joshi,
EPI_ISL_467049	B.J. Medical College and Civil hospital	Gujarat Biotechnology Research Centre	Komal Patel, Labdhi Pandya, Snehal Bagatharia, Pranay Shah, Kamlesh J Upadhyay, Nirav Mungalpara, Tejas Shah, Ankit Hinsu, Pritesh Sabara, Apurvasinh Puvar, Janvi Raval, Zarna Patel, Monika Gandhi, Pinal Trivedi, Maharshi Pandya, Nidhi Patel, Nitin Savaliya, Raghawendra Kumar, Dinesh Kumar, Zuber Saiyed, Neelam Nathani, R D Dixit, A M Kadri, Harsh Bakshi, Chaitanya Joshi, Madhvi Joshi,
EPI_ISL_467050	B.J. Medical College and Civil hospital	Gujarat Biotechnology Research Centre	Labdhi Pandya, Snehal Bagatharia, Pranay Shah, Kamlesh J Upadhyay, Nirav Mungalpara, Tejas Shah, Ankit Hinsu, Pritesh Sabara, Apurvasinh Puvar, Janvi Raval, Zarna Patel, Monika Gandhi, Pinal Trivedi, Maharshi Pandya, Nidhi Patel, Nitin Savaliya, Raghawendra Kumar, Dinesh Kumar, Zuber Saiyed, Komal Patel, Armi Chaudhari, R D Dixit, A M Kadri, Harsh Bakshi, Chaitanya Joshi, Madhvi Joshi,
EPI_ISL_467051	B.J. Medical College and Civil hospital	Gujarat Biotechnology Research Centre	Snehal Bagatharia, Pranay Shah, Kamlesh J Upadhyay, Nirav Mungalpara, Tejas Shah, Ankit Hinsu, Pritesh Sabara, Apurvasinh Puvar, Janvi Raval, Zarna Patel, Monika Gandhi, Pinal Trivedi, Maharshi Pandya, Nidhi Patel, Nitin Savaliya, Raghawendra Kumar, Dinesh Kumar, Zuber Saiyed, Komal Patel, Labdhi Pandya, Bhavya Jindal, R D Dixit, A M Kadri, Harsh Bakshi, Chaitanya Joshi, Madhvi Joshi,
EPI_ISL_467052	B.J. Medical College and Civil hospital	Gujarat Biotechnology Research Centre	Pranay Shah, Kamlesh J Upadhyay, Nirav Mungalpara, Tejas Shah, Ankit Hinsu, Pritesh Sabara, Apurvasinh Puvar, Janvi Raval, Zarna Patel, Monika Gandhi, Pinal Trivedi, Maharshi Pandya, Nidhi Patel, Nitin Savaliya, Raghawendra Kumar, Dinesh Kumar, Zuber Saiyed, Komal Patel, Labdhi Pandya, Snehal Bagatharia, Priyanka P Vatsa, R D Dixit, A M Kadri, Harsh Bakshi, Chaitanya Joshi, Madhvi Joshi,
EPI_ISL_467053	B.J. Medical College and Civil hospital	Gujarat Biotechnology Research Centre	Kamlesh J Upadhyay, Nirav Mungalpara, Tejas Shah, Ankit Hinsu, Pritesh Sabara, Apurvasinh Puvar, Janvi Raval, Zarna Patel, Monika Gandhi, Pinal Trivedi, Maharshi Pandya, Nidhi Patel, Nitin Savaliya, Raghawendra Kumar, Dinesh Kumar, Zuber Saiyed, Komal Patel, Labdhi Pandya, Snehal Bagatharia, Pranay Shah, Pooja P Doshi, R D Dixit, A M Kadri, Harsh Bakshi, Chaitanya Joshi, Madhvi Joshi,
EPI_ISL_467054	B.J. Medical College and Civil hospital	Gujarat Biotechnology Research Centre	Nirav Mungalpara, Tejas Shah, Ankit Hinsu, Pritesh Sabara, Apurvasinh Puvar, Janvi Raval, Zarna Patel, Monika Gandhi, Pinal Trivedi, Maharshi Pandya, Nidhi Patel, Nitin Savaliya, Raghawendra Kumar, Dinesh Kumar, Zuber Saiyed, Komal Patel, Labdhi Pandya, Snehal Bagatharia, Pranay Shah, Kamlesh J Upadhyay, Akanksha Verma, R D Dixit, A M Kadri, Harsh Bakshi, Chaitanya Joshi, Madhvi Joshi,
EPI_ISL_467299	Research and Medical Analysis Laboratory of Gendarmerie Royale	Research and Medical Analysis Laboratory of Gendarmerie Royale	Sanaâ LEMRISS Amal SOUIRI Hicham EL OSSMANI Saâd EL Kabbaj
EPI_ISL_467300	General Hospital "Abdulah Nakas"	Alea Genetic Center	Rijad Konjhodzic; Lana Salihefendic; Teufik Goletic; Sead Jazic; Dino Pecar; Nihad Fejzic; Damir Marjanovic; Enis Kandic
EPI_ISL_467352, EPI_ISL_467353, EPI_ISL_467367, EPI_ISL_467369	Laboratory of Respiratory Viruses and Measles, Oswaldo Cruz Institute, FIOCRUZ	Laboratory of Respiratory Viruses and Measles, Oswaldo Cruz Institute, FIOCRUZ	Paola Resende, Luciana Appolinario, Fernando Motta, Anna Carolina Paixão, Ana Carolina Mendonça, Aline Mattos, Milene Miranda, Cristiana Garcia, Braulia Caetano, Maria Ogrzewalska, Jonathan Lopes, Marilda Siqueira
EPI_ISL_467374	Dinkes Samarinda	Eijkman Institute for Molecular Biology, Ministry of Research and Technology/National Agency for Research and Innovation	Edison Johar, Frilasita A Yudhaputri, Hidayat Trimarsanto, David H Muljono, Safarina G Malik, Khin Saw Myint, Amin Soebandrio
EPI_ISL_467375	RSUP Prof. Dr. R. Kandou Manado	Eijkman Institute for Molecular Biology, Ministry of Research and Technology/National Agency for Research and Innovation	Edison Johar, Frilasita A Yudhaputri, Hidayat Trimarsanto, David H Muljono, Safarina G Malik, Khin Saw Myint, Amin Soebandrio
EPI_ISL_467376	RSUP Fatmawati	Eijkman Institute for Molecular Biology, Ministry of Research and Technology/National Agency for Research and Innovation	Edison Johar, Frilasita A Yudhaputri, Hidayat Trimarsanto, David H Muljono, Safarina G Malik, Khin Saw Myint, Amin Soebandrio
EPI_ISL_467666	Virology lab, NIC, NCCD, Ulaanbaatar, Mongolia	National Centre for Communicable Deseases (NCCD)	Naranzul Ts,Darmaa B,Bayasgalan N,Ankhbayar S,Tsogtbaatar B, Erdene-Ochir Ts,Nymadawa P
EPI_ISL_467774, EPI_ISL_467775	Molecular diagnostic laboratory of Federal Budget Institution of Science "Central Research Institute of Epidemiology" of The Federal Service on Customers' Rights Protection and Human Well-being Surveillance	Group of Genomics and Postgenomic Technologies of Central Research Institute of Epidemilology	Speranskaya AS, Kaptelova VV, Samoilov AE, Korneenko EV, Sizova TV, Tivanova EV, Shipulina OY, Akimkin VG
EPI_ISL_467809	Cedars-Sinai Medical Center, Department of Pathology & Laboratory Medicine, Molecular Pathology Laboratory	Cedars-Sinai Medical Center, Molecular Pathology Laboratory of Department of Pathology & Laboratory Medicine and Genomic Core	Wenjuan Zhang, John Paul Govindavari, Brian Davis, Stephanie Chen, Jong Taek Kim, Jianbo Song, Jean Lopategui, Jasmine T Plummer, Eric Vail
EPI_ISL_468134, EPI_ISL_468135, EPI_ISL_468136, EPI_ISL_468137, EPI_ISL_468138, EPI_ISL_468139, EPI_ISL_468140, EPI_ISL_468141, EPI_ISL_468142, EPI_ISL_468143, EPI_ISL_468144, EPI_ISL_468145, EPI_ISL_468146, EPI_ISL_468147, EPI_ISL_468148, EPI_ISL_468149, EPI_ISL_468150, EPI_ISL_468151, EPI_ISL_468152, EPI_ISL_468153, EPI_ISL_468154, EPI_ISL_468155, EPI_ISL_468156, EPI_ISL_468157, EPI_ISL_468158			
see above	[Romania, Bucharest] National Institute for Infectious Diseases "Prof. Dr. Matei Bal"	[Romania, Bucharest] National Institute for Infectious Diseases "Prof. Dr. Matei Bal"	Leontina Banica, Marius Cotic, Corina Casangiu, Marius Surleac, Simona Paraschiv
EPI_ISL_468305, EPI_ISL_468307	Centro de Vigilancia a Saude de Diadema	Instituto Adolfo Lutz, Interdiciplinary Procedures Center, Strategic Laboratory	Claudio Tavares Sacchi, Claudia Regina Gonçalves, Erica Valessa Ramos Gomes
EPI_ISL_468308	Hospital Municipal do Tatuape Carmino Caricchio	Instituto Adolfo Lutz, Interdiciplinary Procedures Center, Strategic Laboratory	Claudio Tavares Sacchi, Claudia Regina Gonçalves, Erica Valessa Ramos Gomes
EPI_ISL_468310	Hospital Sao Paulo de Ensino da UNIFESP	Instituto Adolfo Lutz, Interdiciplinary Procedures Center, Strategic Laboratory	Claudio Tavares Sacchi, Claudia Regina Gonçalves, Erica Valessa Ramos Gomes
EPI_ISL_468311, EPI_ISL_468312	Hospital Municipal Dr Ignacio Proenca de Gouvea	Instituto Adolfo Lutz, Interdiciplinary Procedures Center, Strategic Laboratory	Claudio Tavares Sacchi, Claudia Regina Gonçalves, Erica Valessa Ramos Gomes
EPI_ISL_468313	Vigilancia Epidemiologica de São Bernardo do Campo	Instituto Adolfo Lutz, Interdiciplinary Procedures Center, Strategic Laboratory	Claudio Tavares Sacchi, Claudia Regina Gonçalves, Erica Valessa Ramos Gomes
EPI_ISL_468314	CTA Centro de Testagem e Aconselhamento	Instituto Adolfo Lutz, Interdiciplinary Procedures Center, Strategic Laboratory	Claudio Tavares Sacchi, Claudia Regina Gonçalves, Erica Valessa Ramos Gomes
EPI_ISL_468315	Hospital Municipal do Tatuape Carmino Caricchio	Instituto Adolfo Lutz, Interdiciplinary Procedures Center, Strategic Laboratory	Claudio Tavares Sacchi, Claudia Regina Gonçalves, Erica Valessa Ramos Gomes
EPI_ISL_468316	UPA Vila Assis	Instituto Adolfo Lutz, Interdiciplinary Procedures Center, Strategic Laboratory	Claudio Tavares Sacchi, Claudia Regina Gonçalves, Erica Valessa Ramos Gomes
EPI_ISL_468318	Hospital Universitario da USP	Instituto Adolfo Lutz, Interdiciplinary Procedures Center, Strategic Laboratory	Claudio Tavares Sacchi, Claudia Regina Gonçalves, Erica Valessa Ramos Gomes
EPI_ISL_468319	Vigilancia Epidemiologica de São Bernardo do Campo	Instituto Adolfo Lutz, Interdiciplinary Procedures Center, Strategic Laboratory	Claudio Tavares Sacchi, Claudia Regina Gonçalves, Erica Valessa Ramos Gomes
EPI_ISL_468320	Secretaria Municipal de Saude de Hortolandia	Instituto Adolfo Lutz, Interdiciplinary Procedures Center, Strategic Laboratory	Claudio Tavares Sacchi, Claudia Regina Gonçalves, Erica Valessa Ramos Gomes
EPI_ISL_468321	Hospital Universitario da USP	Instituto Adolfo Lutz, Interdiciplinary Procedures Center, Strategic Laboratory	Claudio Tavares Sacchi, Claudia Regina Gonçalves, Erica Valessa Ramos Gomes
EPI_ISL_468591, EPI_ISL_468656	Institute for Public Health	Laboratory for advanced genomics	Filip Roki, Lovro Trgovec-Greif, Neven Sui, Tomislav Rukavina, Igor Jurak, Oliver Vugrek
EPI_ISL_468765, EPI_ISL_468766, EPI_ISL_468767, EPI_ISL_468768, EPI_ISL_468771, EPI_ISL_468772, EPI_ISL_468774, EPI_ISL_468775, EPI_ISL_468776, EPI_ISL_468777, EPI_ISL_468780, EPI_ISL_468782, EPI_ISL_468787, EPI_ISL_468788, EPI_ISL_468791, EPI_ISL_468793, EPI_ISL_468795, EPI_ISL_468798, EPI_ISL_468800, EPI_ISL_468801, EPI_ISL_468804, EPI_ISL_468805, EPI_ISL_468808, EPI_ISL_468809, EPI_ISL_468810, EPI_ISL_468812, EPI_ISL_468814, EPI_ISL_468815, EPI_ISL_468817, EPI_ISL_468820, EPI_ISL_468829, EPI_ISL_468830, EPI_ISL_468832, EPI_ISL_468834, EPI_ISL_468835, EPI_ISL_468839,			

EPI_ISL_468841, EPI_ISL_468848, EPI_ISL_468849, EPI_ISL_468851, EPI_ISL_468855, EPI_ISL_468857, EPI_ISL_468858, EPI_ISL_468859

see above	Servicio de Microbiología, Hospital Miguel Servet, Zaragoza	SeqCOVID-SPAIN consortium/IBV(CSIC)	Antonio Rezusta López, Alexander Tristancho Baró, Ana Milagro, Yolanda Gracia Grataloup, Nieves Martínez Cameo and SeqCOVID-SPAIN consortium
EPI_ISL_468914, EPI_ISL_469016, EPI_ISL_469018, EPI_ISL_469019, EPI_ISL_469020, EPI_ISL_469021, EPI_ISL_469022	Istituto Zooprofilattico Sperimentale Puglia e Basilicata; Dipartimento di Bioscienze, Biotecnologie e Biofarmaceutica dell'Università degli Studi di Bari "A.Moro"; Istituto di Biomembrane. Bioenergetica e Biotecnologie Molecolari del Consiglio Nazionale delle Ricerche di Bari	Beaconlab (Bioinformatics, Evolution and Comparative Genomics lab), Dept of Biosciences, University on Milan	Parisi A.,Pesole G., Manzari C., Chiara M.
EPI_ISL_469023	Istituto Zooprofilattico Sperimentale Puglia e Basilicata; Dipartimento di Bioscienze, Biotecnologie e Biofarmaceutica dell'Università degli Studi di Bari "A.Moro"; Istituto di Biomembrane. Bioenergetica e Biotecnologie Molecolari del Consiglio Nazionale delle Ricerche di Bari	Beaconlab (Bioinformatics, Evolution and Comparative Genomics lab), Dept of Biosciences, University on Milan	Parisi A.,Pesole G., Manzari C., Chiara M
EPI_ISL_469024	B.J. Medical College and Civil hospital	Gujarat Biotechnology Research Centre	Tejas Shah, Ankit Hinsu, Pritesh Sabara, Apurvasinh Puvar, Janvi Raval, Zarna Patel, Monika Gandhi, Pinal Trivedi, Maharshi Pandya, Nidhi Patel, Nitin Savaliya, Raghawendra Kumar, Dinesh Kumar, Zuber Saiyed, Komal Patel, Labdhi Pandya, Snehal Bagatharia, Pranay Shah, Kamlesh J Upadhyay, Nirav Mungalpara, Priti Pandita, R D Dixit, A M Kadri, Harsh Bakshi, Chaitanya Joshi, Madhvi Joshi
EPI_ISL_469025	B.J. Medical College and Civil hospital	Gujarat Biotechnology Research Centre	Ankit Hinsu, Pritesh Sabara, Apurvasinh Puvar, Janvi Raval, Zarna Patel, Monika Gandhi, Pinal Trivedi, Maharshi Pandya, Nidhi Patel, Nitin Savaliya, Raghawendra Kumar, Dinesh Kumar, Zuber Saiyed, Komal Patel, Labdhi Pandya, Snehal Bagatharia, Pranay Shah, Kamlesh J Upadhyay, Nirav Mungalpara, Tejas Shah, Pragya Sharma, R D Dixit, A M Kadri, Harsh Bakshi, Chaitanya Joshi, Madhvi Joshi
EPI_ISL_469026	B.J. Medical College and Civil hospital	Gujarat Biotechnology Research Centre	Pritesh Sabara, Apurvasinh Puvar, Janvi Raval, Zarna Patel, Monika Gandhi, Pinal Trivedi, Maharshi Pandya, Nidhi Patel, Nitin Savaliya, Raghawendra Kumar, Dinesh Kumar, Zuber Saiyed, Komal Patel, Labdhi Pandya, Snehal Bagatharia, Pranay Shah, Kamlesh J Upadhyay, Nirav Mungalpara, Tejas Shah, Ankit Hinsu, Neha Rajpara, R D Dixit, A M Kadri, Harsh Bakshi, Chaitanya Joshi, Madhvi Joshi
EPI_ISL_469027	B.J. Medical College and Civil hospital	Gujarat Biotechnology Research Centre	Apurvasinh Puvar, Janvi Raval, Zarna Patel, Monika Gandhi, Pinal Trivedi, Maharshi Pandya, Nidhi Patel, Nitin Savaliya, Raghawendra Kumar, Dinesh Kumar, Zuber Saiyed, Komal Patel, Labdhi Pandya, Snehal Bagatharia, Pranay Shah, Kamlesh J Upadhyay, Nirav Mungalpara, Tejas Shah, Ankit Hinsu, Pritesh Sabara, Afzal Ansari, R D Dixit, A M Kadri, Harsh Bakshi, Chaitanya Joshi, Madhvi Joshi
EPI_ISL_469028	B.J. Medical College and Civil hospital	Gujarat Biotechnology Research Centre	Janvi Raval, Zarna Patel, Monika Gandhi, Pinal Trivedi, Maharshi Pandya, Nidhi Patel, Nitin Savaliya, Raghawendra Kumar, Dinesh Kumar, Zuber Saiyed, Komal Patel, Labdhi Pandya, Snehal Bagatharia, Pranay Shah, Kamlesh J Upadhyay, Nirav Mungalpara, Tejas Shah, Ankit Hinsu, Pritesh Sabara, Apurvasinh Puvar, Fenil Patel, R D Dixit, A M Kadri, Harsh Bakshi, Chaitanya Joshi, Madhvi Joshi
EPI_ISL_469029	Government Medical College, Vadodara	Gujarat Biotechnology Research Centre	Zarna Patel, Monika Gandhi, Pinal Trivedi, Maharshi Pandya, Nidhi Patel, Nitin Savaliya, Raghawendra Kumar, Dinesh Kumar, Zuber Saiyed, Komal Patel, Labdhi Pandya, Snehal Bagatharia, Meenakshi Shah, Neena Doshi, Varsha Godbole, Tejas Shah, Ankit Hinsu, Pritesh Sabara, Apurvasinh Puvar, Janvi Raval, Neelam Nathani, R D Dixit, A M Kadri, Harsh Bakshi, Chaitanya Joshi, Madhvi Joshi
EPI_ISL_469030	Government Medical College, Vadodara	Gujarat Biotechnology Research Centre	Monika Gandhi, Pinal Trivedi, Maharshi Pandya, Nidhi Patel, Nitin Savaliya, Raghawendra Kumar, Dinesh Kumar, Zuber Saiyed, Komal Patel, Labdhi Pandya, Snehal Bagatharia, Meenakshi Shah, Neena Doshi, Varsha Godbole, Tejas Shah, Ankit Hinsu, Pritesh Sabara, Apurvasinh Puvar, Janvi Raval, Zarna Patel, Armi Chaudhari, R D Dixit, A M Kadri, Harsh Bakshi, Chaitanya Joshi, Madhvi Joshi
EPI_ISL_469031	Government Medical College, Vadodara	Gujarat Biotechnology Research Centre	Pinal Trivedi, Maharshi Pandya, Nidhi Patel, Nitin Savaliya, Raghawendra Kumar, Dinesh Kumar, Zuber Saiyed, Komal Patel, Labdhi Pandya, Snehal Bagatharia, Meenakshi Shah, Neena Doshi, Varsha Godbole, Tejas Shah, Ankit Hinsu, Pritesh Sabara, Apurvasinh Puvar, Janvi Raval, Zarna Patel, Monika Gandhi, Bhavya Jindal, R D Dixit, A M Kadri, Harsh Bakshi, Chaitanya Joshi, Madhvi Joshi
EPI_ISL_469032	Government Medical College, Vadodara	Gujarat Biotechnology Research Centre	Maharshi Pandya, Nidhi Patel, Nitin Savaliya, Raghawendra Kumar, Dinesh Kumar, Zuber Saiyed, Komal Patel, Labdhi Pandya, Snehal Bagatharia, Meenakshi Shah, Neena Doshi, Varsha Godbole, Tejas Shah, Ankit Hinsu, Pritesh Sabara, Apurvasinh Puvar, Janvi Raval, Zarna Patel, Monika Gandhi, Pinal Trivedi, Pragya Sharma, R D Dixit, A M Kadri, Harsh Bakshi, Chaitanya Joshi, Madhvi Joshi
EPI_ISL_469033	Government Medical College, Vadodara	Gujarat Biotechnology Research Centre	Nidhi Patel, Nitin Savaliya, Raghawendra Kumar, Dinesh Kumar, Zuber Saiyed, Komal Patel, Labdhi Pandya, Snehal Bagatharia, Meenakshi Shah, Neena Doshi, Varsha Godbole, Tejas Shah, Ankit Hinsu, Pritesh Sabara, Apurvasinh Puvar, Janvi Raval, Zarna Patel, Monika Gandhi, Pinal Trivedi, Maharshi Pandya, Priyanka P Vatsa, R D Dixit, A M Kadri, Harsh Bakshi, Chaitanya Joshi, Madhvi Joshi
EPI_ISL_469034	Government Medical College, Vadodara	Gujarat Biotechnology Research Centre	Nitin Savaliya, Raghawendra Kumar, Dinesh Kumar, Zuber Saiyed, Komal Patel, Labdhi Pandya, Snehal Bagatharia, Meenakshi Shah, Neena Doshi, Varsha Godbole, Tejas Shah, Ankit Hinsu, Pritesh Sabara, Apurvasinh Puvar, Janvi Raval, Zarna Patel, Monika Gandhi, Pinal Trivedi, Maharshi Pandya, Nidhi Patel, Pooja P Doshi, R D Dixit, A M Kadri, Harsh Bakshi, Chaitanya Joshi, Madhvi Joshi
EPI_ISL_469035	Government Medical College, Vadodara	Gujarat Biotechnology Research Centre	Raghawendra Kumar, Dinesh Kumar, Zuber Saiyed, Komal Patel, Labdhi Pandya, Snehal Bagatharia, Meenakshi Shah, Neena Doshi, Varsha Godbole, Tejas Shah, Ankit Hinsu, Pritesh Sabara, Apurvasinh Puvar, Janvi Raval, Zarna Patel, Monika Gandhi, Pinal Trivedi, Maharshi Pandya, Nidhi Patel, Nitin Savaliya, Akanksha Verma, R D Dixit, A M Kadri, Harsh Bakshi, Chaitanya Joshi, Madhvi Joshi
EPI_ISL_469036	Government Medical College, Vadodara	Gujarat Biotechnology Research Centre	Dinesh Kumar, Zuber Saiyed, Komal Patel, Labdhi Pandya, Snehal Bagatharia, Meenakshi Shah, Neena Doshi, Varsha Godbole, Tejas Shah, Ankit Hinsu, Pritesh Sabara, Apurvasinh Puvar, Janvi Raval, Zarna Patel, Monika Gandhi, Pinal Trivedi, Maharshi Pandya, Nidhi Patel, Nitin Savaliya, Raghawendra Kumar, Priti Pandita, R D Dixit, A M Kadri, Harsh Bakshi, Chaitanya Joshi, Madhvi Joshi
EPI_ISL_469037	GMERS Medical College & Hospital	Gujarat Biotechnology Research Centre	Zuber Saiyed, Komal Patel, Labdhi Pandya, Snehal Bagatharia, Meenakshi Shah, Neena Doshi, Varsha Godbole, Tejas Shah, Ankit Hinsu, Pritesh Sabara, Apurvasinh Puvar, Janvi Raval, Zarna Patel, Monika Gandhi, Pinal Trivedi, Maharshi Pandya, Nidhi Patel, Nitin Savaliya, Raghawendra Kumar, Dinesh Kumar, Pragya Sharma, R D Dixit, A M Kadri, Harsh Bakshi, Chaitanya Joshi, Madhvi Joshi
EPI_ISL_469038	GMERS Medical College & Hospital	Gujarat Biotechnology Research Centre	Komal Patel, Labdhi Pandya, Snehal Bagatharia, Meenakshi Shah, Neena Doshi, Varsha Godbole, Tejas Shah, Ankit Hinsu, Pritesh Sabara, Apurvasinh Puvar, Janvi Raval, Zarna Patel, Monika Gandhi, Pinal Trivedi, Maharshi Pandya, Nidhi Patel, Nitin Savaliya, Raghawendra Kumar, Dinesh Kumar, Zuber Saiyed, Neha Rajpara, R D Dixit, A M Kadri, Harsh Bakshi, Chaitanya Joshi, Madhvi Joshi
EPI_ISL_469039	GMERS Medical College & Hospital	Gujarat Biotechnology Research Centre	Labdhi Pandya, Snehal Bagatharia, Meenakshi Shah, Neena Doshi, Varsha Godbole, Tejas Shah, Ankit Hinsu, Pritesh Sabara, Apurvasinh Puvar, Janvi Raval, Zarna Patel, Monika Gandhi, Pinal Trivedi, Maharshi Pandya, Nidhi Patel, Nitin Savaliya, Raghawendra Kumar, Dinesh Kumar, Zuber Saiyed, Komal Patel, Afzal Ansari, R D Dixit, A M Kadri, Harsh Bakshi, Chaitanya Joshi, Madhvi Joshi
EPI_ISL_469040	GMERS Medical College & Hospital	Gujarat Biotechnology Research Centre	Snehal Bagatharia, Meenakshi Shah, Neena Doshi, Varsha Godbole, Tejas Shah, Ankit Hinsu, Pritesh Sabara, Apurvasinh Puvar, Janvi Raval, Zarna Patel, Monika Gandhi, Pinal Trivedi, Maharshi Pandya, Nidhi Patel, Nitin Savaliya, Raghawendra Kumar, Dinesh Kumar, Zuber Saiyed, Komal Patel, Labdhi Pandya, Fenil Patel, R D Dixit, A M Kadri, Harsh Bakshi, Chaitanya Joshi, Madhvi Joshi
EPI_ISL_469041	GMERS Medical College & Hospital	Gujarat Biotechnology Research Centre	Meenakshi Shah, Neena Doshi, Varsha Godbole, Tejas Shah, Ankit Hinsu, Pritesh Sabara, Apurvasinh Puvar, Janvi Raval, Zarna Patel, Monika Gandhi, Pinal Trivedi, Maharshi Pandya, Nidhi Patel, Nitin Savaliya, Raghawendra Kumar, Dinesh Kumar, Zuber Saiyed, Komal Patel, Labdhi Pandya, Snehal Bagatharia, Neelam Nathani, R D Dixit, A M Kadri, Harsh Bakshi, Chaitanya Joshi, Madhvi Joshi
EPI_ISL_469042	GMERS Medical College & Hospital	Gujarat Biotechnology Research Centre	Neena Doshi, Varsha Godbole, Tejas Shah, Ankit Hinsu, Pritesh Sabara, Apurvasinh Puvar, Janvi Raval, Zarna Patel, Monika Gandhi, Pinal Trivedi, Maharshi Pandya, Nidhi Patel, Nitin Savaliya, Raghawendra Kumar, Dinesh Kumar, Zuber Saiyed, Komal Patel, Labdhi Pandya, Snehal Bagatharia, Meenakshi Shah, Armi Chaudhari, R D Dixit, A M Kadri, Harsh Bakshi, Chaitanya Joshi, Madhvi Joshi
EPI_ISL_469043	Dr. N. D. Desai Medical College & Hospital	Gujarat Biotechnology Research Centre	J G Buch, Jigar Gusani, Supreet Prabhu, Tejas Shah, Ankit Hinsu, Pritesh Sabara, Apurvasinh Puvar, Janvi Raval, Zarna Patel, Monika Gandhi, Pinal Trivedi, Maharshi Pandya, Nidhi Patel, Nitin Savaliya, Raghawendra Kumar, Dinesh Kumar, Zuber Saiyed, Komal Patel, Labdhi Pandya, Snehal Bagatharia, Bhavya Jindal, R D Dixit, A M Kadri, Harsh Bakshi, Chaitanya Joshi, Madhvi Joshi
EPI_ISL_469044	Dr. N. D. Desai Medical College & Hospital	Gujarat Biotechnology Research Centre	Jigar Gusani, Supreet Prabhu, Tejas Shah, Ankit Hinsu, Pritesh Sabara, Apurvasinh Puvar, Janvi Raval, Zarna Patel, Monika Gandhi, Pinal Trivedi, Maharshi Pandya, Nidhi Patel, Nitin Savaliya, Raghawendra Kumar, Dinesh Kumar, Zuber Saiyed, Komal Patel, Labdhi Pandya, Snehal Bagatharia, J G Buch, Neha Rajpara, R D Dixit, A M Kadri, Harsh Bakshi, Chaitanya Joshi, Madhvi Joshi

EPI_ISL_469045	Dr. N. D. Desai Medical College & Hospital	Gujarat Biotechnology Research Centre	Supreet Prabhu, Tejas Shah, Ankit Hinsu, Pritesh Sabara, Apurvasinh Puvar, Janvi Raval, Zarna Patel, Monika Gandhi, Pinal Trivedi, Maharshi Pandya, Nidhi Patel, Nitin Savaliya, Raghawendra Kumar, Dinesh Kumar, Zuber Saiyed, Komal Patel, Labdhi Pandya, Snehal Bagatharia, J G Buch, Jigar Gusani, Priyanka P Vatsa, R D Dixit, A M Kadri, Harsh Bakshi, Chaitanya Joshi, Madhvi Joshi
EPI_ISL_469046	Dr. N. D. Desai Medical College & Hospital	Gujarat Biotechnology Research Centre	Tejas Shah, Ankit Hinsu, Pritesh Sabara, Apurvasinh Puvar, Janvi Raval, Zarna Patel, Monika Gandhi, Pinal Trivedi, Maharshi Pandya, Nidhi Patel, Nitin Savaliya, Raghawendra Kumar, Dinesh Kumar, Zuber Saiyed, Komal Patel, Labdhi Pandya, Snehal Bagatharia, J G Buch, Jigar Gusani, Supreet Prabhu, Pooja P Doshi, R D Dixit, A M Kadri, Harsh Bakshi, Chaitanya Joshi, Madhvi Joshi
EPI_ISL_469047	Dr. N. D. Desai Medical College & Hospital	Gujarat Biotechnology Research Centre	Ankit Hinsu, Pritesh Sabara, Apurvasinh Puvar, Janvi Raval, Zarna Patel, Monika Gandhi, Pinal Trivedi, Maharshi Pandya, Nidhi Patel, Nitin Savaliya, Raghawendra Kumar, Dinesh Kumar, Zuber Saiyed, Komal Patel, Labdhi Pandya, Snehal Bagatharia, J G Buch, Jigar Gusani, Supreet Prabhu, Tejas Shah, Akanksha Verma, R D Dixit, A M Kadri, Harsh Bakshi, Chaitanya Joshi, Madhvi Joshi
EPI_ISL_469048	Banas Medical College and Research Institute	Gujarat Biotechnology Research Centre	Radhika Khara, Sunil R Joshi, Viren s Doshi, Zarna Patel, Monika Gandhi, Pinal Trivedi, Maharshi Pandya, Nidhi Patel, Nitin Savaliya, Raghawendra Kumar, Dinesh Kumar, Zuber Saiyed, Komal Patel, Labdhi Pandya, Snehal Bagatharia, Tejas Shah, Ankit Hinsu, Pritesh Sabara, Apurvasinh Puvar, Janvi Raval, Priti Pandita, R D Dixit, A M Kadri, Harsh Bakshi, Chaitanya Joshi, Madhvi Joshi
EPI_ISL_469275	Egyptian National Cancer Institute (ENCI)	Human Genome Center	Zekri, Abdel Rahman N, Amer,K.E., Ahmed,O.S., Soliman,H.K., Hafez,M.M., Bahnassy,A.A., Abdelhamid,W., Gad,A., Ali,M., Hassan,W., Samir,M., Raouf,A., Hamdy,M.S., Soliman,M.S., Elsissy,M.H., Elkhateeb,S.M., Ezzelarab,M.H., Abouelhoda, Mohamed
EPI_ISL_469282	Service de Virologie Hôpital Saint-Louis	Laboratory of Cell Biology of viral infection, Unit INSERM-U944	Laurent Meertens, Lucie Bonnet-Madin, Constance Delaugerre, Ali Amara
EPI_ISL_469283, EPI_ISL_469284	Service de Virologie Hôpital Saint-Louis	Laboratory Cell Biology of Viral Infection-INSERM unit 944	Laurent Meertens, Lucie Bonnet-Madin, Séverine Mercier-Delarue, Maud SALMONA, Constance Delaugerre, Ali Amara
EPI_ISL_470896	Russian State Collection of Viruses	Pathogenic Microorganisms Variability Laboratory	Alexey Shchetinin, Maria Nikiforova, Elena Shidlovskaya, Nadezhda Kuznetsova, Inna Dolzhikova, Daria Grousova, Andrey Botikov, Denis Logunov, Alexander Gintsburg, Vladimir Gushchin
EPI_ISL_470897, EPI_ISL_470898, EPI_ISL_470899	Pathogenic Microorganisms Variability Laboratory	Pathogenic Microorganisms Variability Laboratory	Alexey Shchetinin, Maria Nikiforova, Elena Shidlovskaya, Nadezhda Kuznetsova, Andrey Botikov, Alexander Gintsburg, Vladimir Gushchin
EPI_ISL_470900, EPI_ISL_470901, EPI_ISL_470902	Influenza etiology and epidemiology laboratory	Pathogenic Microorganisms Variability Laboratory	Alexey Shchetinin, Maria Nikiforova, Elena Shidlovskaya, Nadezhda Kuznetsova, Vladimir Gushchin, Inna Dolzhikova, Daria Grousova, Andrey Botikov, Denis Logunov, Kirill Krasnoslobotsev, Svetlana Trushakova, Elena Burtseva, Ludmila Kolobukhina, Svetlana Smetanina, Alexander Gintsburg
EPI_ISL_470903, EPI_ISL_470904	Influenza etiology and epidemiology laboratory	Pathogenic Microorganisms Variability Laboratory	Alexey Shchetinin, Maria Nikiforova, Elena Shidlovskaya, Nadezhda Kuznetsova, Vladimir Gushchin, Inna Dolzhikova, Daria Grousova, Andrey Botikov, Denis Logunov, Anna Ignatjeva, Evgeniya Mukasheva, Elena Burtseva, Ludmila Kolobukhina, Svetlana Smetanina, Alexander Gintsburg
EPI_ISL_471158, EPI_ISL_471159, EPI_ISL_471160, EPI_ISL_471161, EPI_ISL_471163, EPI_ISL_471164, EPI_ISL_471166, EPI_ISL_471167, EPI_ISL_471168, EPI_ISL_471169, EPI_ISL_471171			
see above	MRCG at LSHTM Genomics lab	MRCG at LSHTM Genomics lab	Sesay et al
EPI_ISL_471267	Hospital IESS Babahoyo	Institute of Microbiology, Universidad San Francisco de Quito	Sully Márquez, Belén Prado-Vivar, Juan José Guadalupe, Bernardo Gutiérrez, Francisco Cordova, Ninfa Henriquez, Killen Briones-Zamora, Killen Briones-Claudette, Verónica Barragán, Patricio Rojas-Silva, Gabriel Trueba, Michelle Grunauer, Paúl Cárdenas
EPI_ISL_471269, EPI_ISL_471270, EPI_ISL_471271	Hospital Oncológico Solca Núcleo de Quito	Institute of Microbiology, Universidad San Francisco de Quito	Sully Márquez, Belén Prado-Vivar, Juan José Guadalupe, Bernardo Gutiérrez, Marcos Di Stefano, Grace Salazar, Verónica Barragán, Patricio Rojas-Silva, Gabriel Trueba, Michelle Grunauer, Paúl Cárdenas
EPI_ISL_471416, EPI_ISL_471417, EPI_ISL_471418, EPI_ISL_471419, EPI_ISL_471420, EPI_ISL_471421, EPI_ISL_471422, EPI_ISL_471423, EPI_ISL_471424	Laboratory for Respiratory Viruses, National Influenza Centre, Cantacuzino National Military-Medical Institute for Research and Development	Cantacuzino Institute	Luiza Ustea, Nicoleta Paraschiv, Tim Durfee, Mihaela Lazar
EPI_ISL_471456, EPI_ISL_471457, EPI_ISL_471458, EPI_ISL_471459, EPI_ISL_471460	Centre de Virologie des Maladies Tropicales	Functional Genomic Platform/Service Analyses Biologique/UATRS/ Centre National Pour la Recherche Scientifique Et Technique (CNRST)	Hicham ANNAZ, Elmostafa EL FAHIME, Marouane MELLOUL, Yassine AKHOUAD, Mly Abdelaziz ELALAOUI, Ahmed REGGAD, Sanaa ALAOUI-Amine , Rachid ABI, Rida TAGAJDID, Zhor KASMY, Safaa ELKORCHI, Nadia TOUIL, Farida HILALI, Abdelkader LAATIRIS , Abdelillah LARAQUI, Tahra BAJJOU , Yassine SEKHSOKH , Idriss-Amine LAHLOU, Mostafa ELOUENNASS, Khalid ENNIBI
EPI_ISL_471472	Hospital Universitari Germans Trias i Pujol(HUGTiP)/Fundació Lluita contra la SIDA (FLSida)/IRTA-CReSA	IrsiCaixa AIDS Research Lab	Marc Noguera-Julian, Pilar Armengol, Jordi Rodón, Julia Vergara, Lidia Ruiz, Nuria Izquierdo, Jorge Carrillo, Roger Paredes, Albert Bensaid, Julia Blanco, Joaquim Segalés, Bonaventura Clotet
EPI_ISL_471528, EPI_ISL_471530	The National Institute of Public Health	State Veterinary Institute Prague and The National Institute of Public Health	Nagy,A;Jirincova,H;Novakova,L;Trnka,D;Vecerova,J
EPI_ISL_471539	Hospital Universitario da USP Sao Paulo	Instituto Adolfo Lutz, Interdiciplinary Procedures Center, Strategic Laboratory	Claudio Tavares Sacchi, Claudia Regina Gonçalves, Erica Valessa Ramos Gomes
EPI_ISL_471540	The National Institute of Public Health	State Veterinary Institute Prague and The National Institute of Public Health	Nagy,A;Jirincova,H;Novakova,L;Trnka,D;Vecerova,J
EPI_ISL_471541	Hospital Geral Santa Marcelina	Instituto Adolfo Lutz, Interdiciplinary Procedures Center, Strategic Laboratory	Claudio Tavares Sacchi, Claudia Regina Gonçalves, Erica Valessa Ramos Gomes
EPI_ISL_471542	Secretaria de Saude de Mogi das Cruzes	Instituto Adolfo Lutz, Interdiciplinary Procedures Center, Strategic Laboratory	Claudio Tavares Sacchi, Claudia Regina Gonçalves, Erica Valessa Ramos Gomes
EPI_ISL_471543	Centro de Saude I Tacito Leite de Carvalho e Silva	Instituto Adolfo Lutz, Interdiciplinary Procedures Center, Strategic Laboratory	Claudio Tavares Sacchi, Claudia Regina Gonçalves, Erica Valessa Ramos Gomes
EPI_ISL_471544	The National Institute of Public Health	State Veterinary Institute Prague and The National Institute of Public Health	Nagy,A;Jirincova,H;Novakova,L;Trnka,D;Vecerova,J
EPI_ISL_471545	Hospital Sao Paulo de Ensino da Unifesp	Instituto Adolfo Lutz, Interdiciplinary Procedures Center, Strategic Laboratory	Claudio Tavares Sacchi, Claudia Regina Gonçalves, Erica Valessa Ramos Gomes
EPI_ISL_471546	AMA DR Jose Soares Hungria	Instituto Adolfo Lutz, Interdiciplinary Procedures Center, Strategic Laboratory	Claudio Tavares Sacchi, Claudia Regina Gonçalves, Erica Valessa Ramos Gomes
EPI_ISL_471547	The National Institute of Public Health	State Veterinary Institute Prague and The National Institute of Public Health	Nagy,A;Jirincova,H;Novakova,L;Trnka,D;Vecerova,J
EPI_ISL_471548	Hospital do Servidor Público Estadual Francisco Morato de Oliveira	Instituto Adolfo Lutz, Interdiciplinary Procedures Center, Strategic Laboratory	Claudio Tavares Sacchi, Claudia Regina Gonçalves, Erica Valessa Ramos Gomes
EPI_ISL_471549	Hospital Municipal Carmen Prudente	Instituto Adolfo Lutz, Interdiciplinary Procedures Center, Strategic Laboratory	Claudio Tavares Sacchi, Claudia Regina Gonçalves, Erica Valessa Ramos Gomes
EPI_ISL_471550	The National Institute of Public Health	State Veterinary Institute Prague and The National Institute of Public Health	Nagy,A;Jirincova,H;Novakova,L;Trnka,D;Vecerova,J
EPI_ISL_471551	Hospital Sao Paulo de Ensino da Unifesp	Instituto Adolfo Lutz, Interdiciplinary Procedures Center, Strategic Laboratory	Claudio Tavares Sacchi, Claudia Regina Gonçalves, Erica Valessa Ramos Gomes
EPI_ISL_471552	Hospital Sancta Maggiore	Instituto Adolfo Lutz, Interdiciplinary Procedures Center, Strategic Laboratory	Claudio Tavares Sacchi, Claudia Regina Gonçalves, Erica Valessa Ramos Gomes

EPI_ISL_471553	The National Institute of Public Health	State Veterinary Institute Prague and The National Institute of Public Health	Nagy,A;Jirincova,H;Novakova,L;Trnka,D;Vecerova,J
EPI_ISL_471554	Hospital Bosque da Saúde	Instituto Adolfo Lutz, Interdiciplinary Procedures Center, Strategic Laboratory	Claudio Tavares Sacchi, Claudia Regina Gonçalves, Erica Valessa Ramos Gomes
EPI_ISL_471555	The National Institute of Public Health	State Veterinary Institute Prague and The National Institute of Public Health	Nagy,A;Jirincova,H;Novakova,L;Trnka,D;Vecerova,J
EPI_ISL_471556	Pronto Socorro Jose Ibrahin	Instituto Adolfo Lutz, Interdiciplinary Procedures Center, Strategic Laboratory	Claudio Tavares Sacchi, Claudia Regina Gonçalves, Erica Valessa Ramos Gomes
EPI_ISL_471562, EPI_ISL_471581, EPI_ISL_471582	Hosp. Municipal Prof. Dr. Alípio Corrêa Netto	Instituto Adolfo Lutz, Interdiciplinary Procedures Center, Strategic Laboratory	Claudio Tavares Sacchi, Claudia Regina Gonçalves, Erica Valessa Ramos Gomes
EPI_ISL_471647	Hospital Municipal de Barueri Dr. Francisco Moran	Instituto Adolfo Lutz, Interdiciplinary Procedures Center, Strategic Laboratory	Claudio Tavares Sacchi, Claudia Regina Gonçalves, Erica Valessa Ramos Gomes
EPI_ISL_471648	UBS e Pronto Socorro Jd. Jacira	Instituto Adolfo Lutz, Interdiciplinary Procedures Center, Strategic Laboratory	Claudio Tavares Sacchi, Claudia Regina Gonçalves, Erica Valessa Ramos Gomes
EPI_ISL_475026	Banas Medical College and Research Institute	Gujarat Biotechnology Research Centre	Sunil R Joshi, Viren s Doshi, Pritesh Sabara, Apurvasinh Puvar, Janvi Raval, Zarna Patel, Monika Gandhi, Pinal Trivedi, Maharshi Pandya, Nidhi Patel, Nitin Savaliya, Raghawendra Kumar, Dinesh Kumar, Zuber Saiyed, Komal Patel, Labdhi Pandya, Snehal Bagatharia, Radhika Khara, Neha Rajpara, R D Dixit, A M Kadri, Harsh Bakshi, Chaitanya Joshi, Madhvi Joshi
EPI_ISL_475027	Banas Medical College and Research Institute	Gujarat Biotechnology Research Centre	Viren s Doshi, Pritesh Sabara, Apurvasinh Puvar, Janvi Raval, Zarna Patel, Monika Gandhi, Pinal Trivedi, Maharshi Pandya, Nidhi Patel, Nitin Savaliya, Raghawendra Kumar, Dinesh Kumar, Zuber Saiyed, Komal Patel, Labdhi Pandya, Snehal Bagatharia, Radhika Khara, Sunil R Joshi, Afzal Ansari, R D Dixit, A M Kadri, Harsh Bakshi, Chaitanya Joshi, Madhvi Joshi
EPI_ISL_475028	Banas Medical College and Research Institute	Gujarat Biotechnology Research Centre	Pritesh Sabara, Apurvasinh Puvar, Janvi Raval, Zarna Patel, Monika Gandhi, Pinal Trivedi, Maharshi Pandya, Nidhi Patel, Nitin Savaliya, Raghawendra Kumar, Dinesh Kumar, Zuber Saiyed, Komal Patel, Labdhi Pandya, Snehal Bagatharia, Radhika Khara, Sunil R Joshi, Viren s Doshi, Fenil Patel, R D Dixit, A M Kadri, Harsh Bakshi, Chaitanya Joshi, Madhvi Joshi
EPI_ISL_475029	Banas Medical College and Research Institute	Gujarat Biotechnology Research Centre	Apurvasinh Puvar, Janvi Raval, Zarna Patel, Monika Gandhi, Pinal Trivedi, Maharshi Pandya, Nidhi Patel, Nitin Savaliya, Raghawendra Kumar, Dinesh Kumar, Zuber Saiyed, Komal Patel, Labdhi Pandya, Snehal Bagatharia, Radhika Khara, Sunil R Joshi, Viren s Doshi, Pritesh Sabara, Neelam Nathani, R D Dixit, A M Kadri, Harsh Bakshi, Chaitanya Joshi, Madhvi Joshi
EPI_ISL_475030	Department of MicroBiology, Government Medical College, Surat	Gujarat Biotechnology Research Centre	Janvi Raval, Zarna Patel, Monika Gandhi, Pinal Trivedi, Maharshi Pandya, Nidhi Patel, Nitin Savaliya, Raghawendra Kumar, Dinesh Kumar, Zuber Saiyed, Komal Patel, Labdhi Pandya, Snehal Bagatharia, Naresh Chauhan, Summaiya Mullan, Amit gamit, Pritesh Sabara, Apurvasinh Puvar, Armi Chaudhari, R D Dixit, A M Kadri, Harsh Bakshi, Chaitanya Joshi, Madhvi Joshi
EPI_ISL_475031	Department of MicroBiology, Government Medical College, Surat	Gujarat Biotechnology Research Centre	Zarna Patel, Monika Gandhi, Pinal Trivedi, Maharshi Pandya, Nidhi Patel, Nitin Savaliya, Raghawendra Kumar, Dinesh Kumar, Zuber Saiyed, Komal Patel, Labdhi Pandya, Snehal Bagatharia, Naresh Chauhan, Summaiya Mullan, Amit gamit, Pritesh Sabara, Apurvasinh Puvar, Janvi Raval, Bhavya Jindal, R D Dixit, A M Kadri, Harsh Bakshi, Chaitanya Joshi, Madhvi Joshi
EPI_ISL_475032	Department of MicroBiology, Government Medical College, Surat	Gujarat Biotechnology Research Centre	Monika Gandhi, Pinal Trivedi, Maharshi Pandya, Nidhi Patel, Nitin Savaliya, Raghawendra Kumar, Dinesh Kumar, Zuber Saiyed, Komal Patel, Labdhi Pandya, Snehal Bagatharia, Naresh Chauhan, Summaiya Mullan, Amit gamit, Pritesh Sabara, Apurvasinh Puvar, Janvi Raval, Zarna Patel, Priyanka P Vatsa, R D Dixit, A M Kadri, Harsh Bakshi, Chaitanya Joshi, Madhvi Joshi
EPI_ISL_475033	Department of MicroBiology, Government Medical College, Surat	Gujarat Biotechnology Research Centre	Pinal Trivedi, Maharshi Pandya, Nidhi Patel, Nitin Savaliya, Raghawendra Kumar, Dinesh Kumar, Zuber Saiyed, Komal Patel, Labdhi Pandya, Snehal Bagatharia, Naresh Chauhan, Summaiya Mullan, Amit gamit, Pritesh Sabara, Apurvasinh Puvar, Janvi Raval, Zarna Patel, Monika Gandhi, Pooja P Doshi, R D Dixit, A M Kadri, Harsh Bakshi, Chaitanya Joshi, Madhvi Joshi
EPI_ISL_475034	Department of MicroBiology, Government Medical College, Surat	Gujarat Biotechnology Research Centre	Maharshi Pandya, Nidhi Patel, Nitin Savaliya, Raghawendra Kumar, Dinesh Kumar, Zuber Saiyed, Komal Patel, Labdhi Pandya, Snehal Bagatharia, Naresh Chauhan, Summaiya Mullan, Amit gamit, Pritesh Sabara, Apurvasinh Puvar, Janvi Raval, Zarna Patel, Monika Gandhi, Pinal Trivedi, Akanksha Verma, R D Dixit, A M Kadri, Harsh Bakshi, Chaitanya Joshi, Madhvi Joshi
EPI_ISL_475035	Department of MicroBiology, Government Medical College, Surat	Gujarat Biotechnology Research Centre	Nidhi Patel, Nitin Savaliya, Raghawendra Kumar, Dinesh Kumar, Zuber Saiyed, Komal Patel, Labdhi Pandya, Snehal Bagatharia, Naresh Chauhan, Summaiya Mullan, Amit gamit, Pritesh Sabara, Apurvasinh Puvar, Janvi Raval, Zarna Patel, Monika Gandhi, Pinal Trivedi, Maharshi Pandya, Priti Pandita, R D Dixit, A M Kadri, Harsh Bakshi, Chaitanya Joshi, Madhvi Joshi
EPI_ISL_475036	Department of MicroBiology, Government Medical College, Surat	Gujarat Biotechnology Research Centre	Nitin Savaliya, Raghawendra Kumar, Dinesh Kumar, Zuber Saiyed, Komal Patel, Labdhi Pandya, Snehal Bagatharia, Naresh Chauhan, Summaiya Mullan, Amit gamit, Pritesh Sabara, Apurvasinh Puvar, Janvi Raval, Zarna Patel, Monika Gandhi, Pinal Trivedi, Maharshi Pandya, Nidhi Patel, Pragya Sharma, R D Dixit, A M Kadri, Harsh Bakshi, Chaitanya Joshi, Madhvi Joshi
EPI_ISL_475037	Department of MicroBiology, Government Medical College, Surat	Gujarat Biotechnology Research Centre	Raghawendra Kumar, Dinesh Kumar, Zuber Saiyed, Komal Patel, Labdhi Pandya, Snehal Bagatharia, Naresh Chauhan, Summaiya Mullan, Amit gamit, Pritesh Sabara, Apurvasinh Puvar, Janvi Raval, Zarna Patel, Monika Gandhi, Pinal Trivedi, Maharshi Pandya, Nidhi Patel, Nitin Savaliya, Neha Rajpara, R D Dixit, A M Kadri, Harsh Bakshi, Chaitanya Joshi, Madhvi Joshi
EPI_ISL_475038	Department of MicroBiology, Government Medical College, Surat	Gujarat Biotechnology Research Centre	Dinesh Kumar, Zuber Saiyed, Komal Patel, Labdhi Pandya, Snehal Bagatharia, Naresh Chauhan, Summaiya Mullan, Amit gamit, Pritesh Sabara, Apurvasinh Puvar, Janvi Raval, Zarna Patel, Monika Gandhi, Pinal Trivedi, Maharshi Pandya, Nidhi Patel, Nitin Savaliya, Raghawendra Kumar, Afzal Ansari, R D Dixit, A M Kadri, Harsh Bakshi, Chaitanya Joshi, Madhvi Joshi
EPI_ISL_475039	Department of MicroBiology, Government Medical College, Surat	Gujarat Biotechnology Research Centre	Zuber Saiyed, Komal Patel, Labdhi Pandya, Snehal Bagatharia, Naresh Chauhan, Summaiya Mullan, Amit gamit, Pritesh Sabara, Apurvasinh Puvar, Janvi Raval, Zarna Patel, Monika Gandhi, Pinal Trivedi, Maharshi Pandya, Nidhi Patel, Nitin Savaliya, Raghawendra Kumar, Dinesh Kumar, Fenil Patel, R D Dixit, A M Kadri, Harsh Bakshi, Chaitanya Joshi, Madhvi Joshi
EPI_ISL_475040	Department of MicroBiology, Government Medical College, Surat	Gujarat Biotechnology Research Centre	Komal Patel, Labdhi Pandya, Snehal Bagatharia, Naresh Chauhan, Summaiya Mullan, Amit gamit, Pritesh Sabara, Apurvasinh Puvar, Janvi Raval, Zarna Patel, Monika Gandhi, Pinal Trivedi, Maharshi Pandya, Nidhi Patel, Nitin Savaliya, Raghawendra Kumar, Dinesh Kumar, Zuber Saiyed, Neelam Nathani, R D Dixit, A M Kadri, Harsh Bakshi, Chaitanya Joshi, Madhvi Joshi
EPI_ISL_475041	Department of MicroBiology, Government Medical College, Surat	Gujarat Biotechnology Research Centre	Labdhi Pandya, Snehal Bagatharia, Naresh Chauhan, Summaiya Mullan, Amit gamit, Pritesh Sabara, Apurvasinh Puvar, Janvi Raval, Zarna Patel, Monika Gandhi, Pinal Trivedi, Maharshi Pandya, Nidhi Patel, Nitin Savaliya, Raghawendra Kumar, Dinesh Kumar, Zuber Saiyed, Komal Patel, Armi Chaudhari, R D Dixit, A M Kadri, Harsh Bakshi, Chaitanya Joshi, Madhvi Joshi
EPI_ISL_475042	Department of MicroBiology, Government Medical College, Surat	Gujarat Biotechnology Research Centre	Snehal Bagatharia, Naresh Chauhan, Summaiya Mullan, Amit gamit, Pritesh Sabara, Apurvasinh Puvar, Janvi Raval, Zarna Patel, Monika Gandhi, Pinal Trivedi, Maharshi Pandya, Nidhi Patel, Nitin Savaliya, Raghawendra Kumar, Dinesh Kumar, Zuber Saiyed, Komal Patel, Labdhi Pandya, Bhavya Jindal, R D Dixit, A M Kadri, Harsh Bakshi, Chaitanya Joshi, Madhvi Joshi
EPI_ISL_475043	Department of MicroBiology, Government Medical College, Surat	Gujarat Biotechnology Research Centre	Naresh Chauhan, Summaiya Mullan, Amit gamit, Pritesh Sabara, Apurvasinh Puvar, Janvi Raval, Zarna Patel, Monika Gandhi, Pinal Trivedi, Maharshi Pandya, Nidhi Patel, Nitin Savaliya, Raghawendra Kumar, Dinesh Kumar, Zuber Saiyed, Komal Patel, Labdhi Pandya, Snehal Bagatharia, Priyanka P Vatsa, R D Dixit, A M Kadri, Harsh Bakshi, Chaitanya Joshi, Madhvi Joshi
EPI_ISL_475044	Department of MicroBiology, Government Medical College, Surat	Gujarat Biotechnology Research Centre	Summaiya Mullan, Amit gamit, Pritesh Sabara, Apurvasinh Puvar, Janvi Raval, Zarna Patel, Monika Gandhi, Pinal Trivedi, Maharshi Pandya, Nidhi Patel, Nitin Savaliya, Raghawendra Kumar, Dinesh Kumar, Zuber Saiyed, Komal Patel, Labdhi Pandya, Snehal Bagatharia, Naresh Chauhan, Pooja P Doshi, R D Dixit, A M Kadri, Harsh Bakshi, Chaitanya Joshi, Madhvi Joshi
EPI_ISL_475045	Department of MicroBiology, Government Medical College, Surat	Gujarat Biotechnology Research Centre	Amit gamit, Pritesh Sabara, Apurvasinh Puvar, Janvi Raval, Zarna Patel, Monika Gandhi, Pinal Trivedi, Maharshi Pandya, Nidhi Patel, Nitin Savaliya, Raghawendra Kumar, Dinesh Kumar, Zuber Saiyed, Komal Patel, Labdhi Pandya, Snehal Bagatharia, Naresh Chauhan, Summaiya Mullan, Akanksha Verma, R D Dixit, A M Kadri, Harsh Bakshi, Chaitanya Joshi, Madhvi Joshi

EPI_ISL_475046	Department of MicroBiology, Government Medical College, Surat	Gujarat Biotechnology Research Centre	Pritesh Sabara, Apurvasinh Puvar, Janvi Raval, Zarna Patel, Monika Gandhi, Pinal Trivedi, Maharshi Pandya, Nidhi Patel, Nitin Savaliya, Raghawendra Kumar, Dinesh Kumar, Zuber Saiyed, Komal Patel, Labdhi Pandya, Snehal Bagatharia, Naresh Chauhan, Summaiya Mullan, Amit gamit, Priti Pandita, R D Dixit, A M Kadri, Harsh Bakshi, Chaitanya Joshi, Madhvi Joshi
EPI_ISL_475047	GMERS Medical College & Hospital	Gujarat Biotechnology Research Centre	Apurvasinh Puvar, Janvi Raval, Zarna Patel, Monika Gandhi, Pinal Trivedi, Maharshi Pandya, Nidhi Patel, Nitin Savaliya, Raghawendra Kumar, Dinesh Kumar, Zuber Saiyed, Komal Patel, Labdhi Pandya, Snehal Bagatharia, Meenakshi Shah, Neena Doshi, Varsha Godbole, Pritesh Sabara, Pragya Sharma, R D Dixit, A M Kadri, Harsh Bakshi, Chaitanya Joshi, Madhvi Joshi
EPI_ISL_475048	GMERS Medical College & Hospital	Gujarat Biotechnology Research Centre	Janvi Raval, Zarna Patel, Monika Gandhi, Pinal Trivedi, Maharshi Pandya, Nidhi Patel, Nitin Savaliya, Raghawendra Kumar, Dinesh Kumar, Zuber Saiyed, Komal Patel, Labdhi Pandya, Snehal Bagatharia, Meenakshi Shah, Neena Doshi, Varsha Godbole, Pritesh Sabara, Apurvasinh Puvar, Neha Rajpara, R D Dixit, A M Kadri, Harsh Bakshi, Chaitanya Joshi, Madhvi Joshi
EPI_ISL_475050	GMERS Medical College & Hospital	Gujarat Biotechnology Research Centre	Monika Gandhi, Pinal Trivedi, Maharshi Pandya, Nidhi Patel, Nitin Savaliya, Raghawendra Kumar, Dinesh Kumar, Zuber Saiyed, Komal Patel, Labdhi Pandya, Snehal Bagatharia, Meenakshi Shah, Neena Doshi, Varsha Godbole, Pritesh Sabara, Apurvasinh Puvar, Janvi Raval, Zarna Patel, Fenil Patel, R D Dixit, A M Kadri, Harsh Bakshi, Chaitanya Joshi, Madhvi Joshi
EPI_ISL_475051	GMERS Medical College & Hospital	Gujarat Biotechnology Research Centre	Pinal Trivedi, Maharshi Pandya, Nidhi Patel, Nitin Savaliya, Raghawendra Kumar, Dinesh Kumar, Zuber Saiyed, Komal Patel, Labdhi Pandya, Snehal Bagatharia, Meenakshi Shah, Neena Doshi, Varsha Godbole, Pritesh Sabara, Apurvasinh Puvar, Janvi Raval, Zarna Patel, Monika Gandhi, Neelam Nathani, R D Dixit, A M Kadri, Harsh Bakshi, Chaitanya Joshi, Madhvi Joshi
EPI_ISL_475052	GMERS Medical College & Hospital	Gujarat Biotechnology Research Centre	Maharshi Pandya, Nidhi Patel, Nitin Savaliya, Raghawendra Kumar, Dinesh Kumar, Zuber Saiyed, Komal Patel, Labdhi Pandya, Snehal Bagatharia, Meenakshi Shah, Neena Doshi, Varsha Godbole, Pritesh Sabara, Apurvasinh Puvar, Janvi Raval, Zarna Patel, Monika Gandhi, Pinal Trivedi, Armi Chaudhari, R D Dixit, A M Kadri, Harsh Bakshi, Chaitanya Joshi, Madhvi Joshi
EPI_ISL_475053	GMERS Medical College & Hospital	Gujarat Biotechnology Research Centre	Nidhi Patel, Nitin Savaliya, Raghawendra Kumar, Dinesh Kumar, Zuber Saiyed, Komal Patel, Labdhi Pandya, Snehal Bagatharia, Meenakshi Shah, Neena Doshi, Varsha Godbole, Pritesh Sabara, Apurvasinh Puvar, Janvi Raval, Zarna Patel, Monika Gandhi, Pinal Trivedi, Maharshi Pandya, Bhavya Jindal, R D Dixit, A M Kadri, Harsh Bakshi, Chaitanya Joshi, Madhvi Joshi
EPI_ISL_475056	Dr. N. D. Desai Medical College & Hospital	Gujarat Biotechnology Research Centre	Dinesh Kumar, Zuber Saiyed, Komal Patel, Labdhi Pandya, Snehal Bagatharia, J G Buch, Jigar Gusani, Supreet Prabhu, Pritesh Sabara, Apurvasinh Puvar, Janvi Raval, Zarna Patel, Monika Gandhi, Pinal Trivedi, Maharshi Pandya, Nidhi Patel, Nitin Savaliya, Raghawendra Kumar, Akanksha Verma, R D Dixit, A M Kadri, Harsh Bakshi, Chaitanya Joshi, Madhvi Joshi
EPI_ISL_475057	Dr. N. D. Desai Medical College & Hospital	Gujarat Biotechnology Research Centre	Zuber Saiyed, Komal Patel, Labdhi Pandya, Supreet Prabhu, Snehal Bagatharia, Jigar Gusani, J G Buch, Pritesh Sabara, Apurvasinh Puvar, Janvi Raval, Zarna Patel, Monika Gandhi, Pinal Trivedi, Maharshi Pandya, Nidhi Patel, Nitin Savaliya, Raghawendra Kumar, Dinesh Kumar, Priti Pandita, R D Dixit, A M Kadri, Harsh Bakshi, Chaitanya Joshi, Madhvi Joshi
EPI_ISL_475059	GAIMS & G K General Hospital	Gujarat Biotechnology Research Centre	Hitesh Assudani, Babulal Babhoria, Labdhi Pandya, Snehal Bagatharia, Pritesh Sabara, Apurvasinh Puvar, Janvi Raval, Zarna Patel, Monika Gandhi, Pinal Trivedi, Maharshi Pandya, Nidhi Patel, Nitin Savaliya, Raghawendra Kumar, Dinesh Kumar, Zuber Saiyed, Komal Patel, Neha Rajpara, R D Dixit, A M Kadri, Harsh Bakshi, Chaitanya Joshi, Madhvi Joshi
EPI_ISL_475574, EPI_ISL_475575, EPI_ISL_475576, EPI_ISL_475577, EPI_ISL_475578, EPI_ISL_475579, EPI_ISL_475580, EPI_ISL_475581, EPI_ISL_475582, EPI_ISL_475583, EPI_ISL_475584, EPI_ISL_475585, EPI_ISL_475586, EPI_ISL_475587, EPI_ISL_475588, EPI_ISL_475589, EPI_ISL_475590, EPI_ISL_475591, EPI_ISL_475592, EPI_ISL_475593, EPI_ISL_475594, EPI_ISL_475595, EPI_ISL_475596, EPI_ISL_475597, EPI_ISL_475598, EPI_ISL_475599, EPI_ISL_475600, EPI_ISL_475601, EPI_ISL_475602, EPI_ISL_475603, EPI_ISL_475604, EPI_ISL_475605, EPI_ISL_475606, EPI_ISL_475607, EPI_ISL_475608, EPI_ISL_475609, EPI_ISL_475610, EPI_ISL_475611, EPI_ISL_475612, EPI_ISL_475613, EPI_ISL_475614, EPI_ISL_475615, EPI_ISL_475616, EPI_ISL_475617, EPI_ISL_475618, EPI_ISL_475619, EPI_ISL_475620, EPI_ISL_475621, EPI_ISL_475622, EPI_ISL_475623, EPI_ISL_475624, EPI_ISL_475625, EPI_ISL_475626, EPI_ISL_475627, EPI_ISL_475628, EPI_ISL_475629, EPI_ISL_475630, EPI_ISL_475631, EPI_ISL_475632, EPI_ISL_475633, EPI_ISL_475634, EPI_ISL_475635, EPI_ISL_475636, EPI_ISL_475637, EPI_ISL_475638, EPI_ISL_475639, EPI_ISL_475640, EPI_ISL_475641, EPI_ISL_475642, EPI_ISL_475643, EPI_ISL_475644, EPI_ISL_475645, EPI_ISL_475646, EPI_ISL_475647, EPI_ISL_475648, EPI_ISL_475649, EPI_ISL_475650, EPI_ISL_475651, EPI_ISL_475652, EPI_ISL_475653, EPI_ISL_475654, EPI_ISL_475655, EPI_ISL_475656, EPI_ISL_475657, EPI_ISL_475658, EPI_ISL_475659, EPI_ISL_475660, EPI_ISL_475661, EPI_ISL_475662, EPI_ISL_475663, EPI_ISL_475664, EPI_ISL_475665, EPI_ISL_475666, EPI_ISL_475667, EPI_ISL_475668, EPI_ISL_475669, EPI_ISL_475670, EPI_ISL_475671, EPI_ISL_475672, EPI_ISL_475673, EPI_ISL_475674, EPI_ISL_475675, EPI_ISL_475676, EPI_ISL_475677, EPI_ISL_475678, EPI_ISL_475679, EPI_ISL_475680, EPI_ISL_475681, EPI_ISL_475682, EPI_ISL_475683, EPI_ISL_475684, EPI_ISL_475685, EPI_ISL_475686, EPI_ISL_475687, EPI_ISL_475688, EPI_ISL_475689, EPI_ISL_475690, EPI_ISL_475691, EPI_ISL_475692, EPI_ISL_475693, EPI_ISL_475694, EPI_ISL_475695, EPI_ISL_475696, EPI_ISL_475697, EPI_ISL_475698, EPI_ISL_475699, EPI_ISL_475700, EPI_ISL_475701, EPI_ISL_475702, EPI_ISL_475703, EPI_ISL_475704, EPI_ISL_475705, EPI_ISL_475706, EPI_ISL_475707, EPI_ISL_475708, EPI_ISL_475709, EPI_ISL_475710, EPI_ISL_475711, EPI_ISL_475712, EPI_ISL_475713, EPI_ISL_475714, EPI_ISL_475715, EPI_ISL_475716			
see above	Cedars-Sinai Medical Center, Department of Pathology & Laboratory Medicine, Molecular Pathology Laboratory	Cedars-Sinai Medical Center, Molecular Pathology Laboratory of Department of Pathology & Laboratory Medicine and Genomic Core	Wenjuan Zhang, John Paul Govindavari, Brian Davis, Stephanie Chen, Jong Taek Kim, Jianbo Song, Jean Lopategui, Jasmine T Plummer, Eric Vail
EPI_ISL_475745, EPI_ISL_475746, EPI_ISL_475747, EPI_ISL_475748, EPI_ISL_475749, EPI_ISL_475750, EPI_ISL_475751, EPI_ISL_475752, EPI_ISL_475753	Medical Ain Shams Research Institute (MASRI), Ain Shams University	Medical Ain Shams Research Institute (MASRI), Ain Shams University	Hesham Elghazaly , Sara Hassan Agwa, Mahmoud Elmeteini , Ahmad Moustafa , Ashraf Omar, Osama Mansour, Samia Abdo, Hala Hafez, Ghada Ismael , Shaimaa Moustafa , Aya Mohamed, Reham Mamdouh , Hoda Abd Elsatar, Manal Hamdy Elsaid, Fatma Ebied
EPI_ISL_476022	Defence Research & Development Establishment	Defence Research & Development Establishment	Shashi Sharma, Paban Kumar Dash, Jyoti S Kumar, Sushil Kumar Sharma, Ambuj Shrivastava
EPI_ISL_476023	Defence Research & Development Establishment (DRDE)	Defence Research & Development Establishment (DRDE)	Shashi Sharma, Paban Kumar Dash, Sushil Kumar Sharma, Ambuj Shrivastava, Jyoti S. Kumar
EPI_ISL_476067	The National Institute of Public Health	State Veterinary Institute Prague and The National Institute of Public Health	Nagy,A;Jirincova,H;Novakova,L;Trnka,D;Vecerova,J
EPI_ISL_476148, EPI_ISL_476149	Institut Pasteur Dakar	Institut Pasteur de Dakar	Ndongo Dia, Moussa Moise Diagne, Mamadou Diop, Ousmane Faye, Amadou Alpha Sall
EPI_ISL_476150	Institut Pasteur Dakar	Institut Pasteur de Dakar	Ndongo Dia, Moussa Moise Diagne, Mamadou diop, Ousmane Faye, Amadou Alpha Sall
EPI_ISL_476151	Institut Pasteur Dakar	Institut Pasteur de Dakar	Ndongo Dia, Moussa Moise Diagne, Mamadou Diop, Ousmane faye, Amadou Alpha Sall
EPI_ISL_476491, EPI_ISL_476492	Institut Pasteur Dakar	Institut Pasteur de Dakar	Ndongo Dia, Moussa Moise Diagne, Mamadou Diop, Ousmane Faye, Amadou Alpha Sall
EPI_ISL_476493	Institut Pasteur Dakar	Institut Pasteur de Dakar	Ndongo Dia, Moussa Moise Diagne, Mamadou Diop, Ousmane Faye, Amadou alpha Sall
EPI_ISL_476494	Institut Pasteur Dakar	Institut Pasteur de Dakar	Ndongo Dia, Moussa Moise Diagne, Mamadou Diop, Ousmane Faye, Amadou Alpha Sall
EPI_ISL_476495, EPI_ISL_476497	Institut Pasteur Dakar	Institut Pasteur de Dakar	Ndongo Dia, Moussa Moise Diagne, Mamadou Diop, Ousmane Faye, Amadou alpha Sall
EPI_ISL_476514	Institut Pasteur Dakar	Institut Pasteur de Dakar	Ndongo Dia, Moussa Moise Diagne, Mamadou Diop, Ousmane Faye, Amadou Alpha Sall
EPI_ISL_476515	Institut Pasteur Dakar	Institut Pasteur de Dakar	Ndongo Dia, Moussa Moise Diagne, Mamadou diop, Ousmane Faye, Amadou alpha Sall
EPI_ISL_476516	Institut Pasteur Dakar	Institut Pasteur de Dakar	Ndongo Dia, Moussa Moise Diagne, mamadou Diop, Ousmane Faye, Amadou Alpha Sall
EPI_ISL_476558, EPI_ISL_476560, EPI_ISL_476562	Institut Pasteur Dakar	Institut Pasteur de Dakar	Ndongo Dia, Moussa Moise Diagne, Mamadou Diop, Ousmane Faye, Amadou Alpha Sall
EPI_ISL_476564	Institut Pasteur Dakar	Institut Pasteur de Dakar	Ndongo Dia, Moussa Moise Diagne, Mamadou diop, Ousmane Faye, Amadou alpha Sall
EPI_ISL_476566	Institut pasteur Dakar	Institut Pasteur de Dakar	Ndongo Dia, Moussa Moise Diagne, Mamadou Diop, Ousmane Faye, Amadou Alpha Sall
EPI_ISL_476569	Institut Pasteur Dakar	Institut Pasteur de Dakar	Ndongo Dia, Moussa Moise, Mamadou Diop, Ousmane Faye, Amadou Alpha Sall
EPI_ISL_476570, EPI_ISL_476572, EPI_ISL_476574	Institut Pasteur Dakar	Institut Pasteur de Dakar	Ndongo Dia, Moussa Moise Diagne, Mamadou Diop, Ousmane Faye, Amadou Alpha Sall
EPI_ISL_476702, EPI_ISL_476703, EPI_ISL_476704	Incubadora Venezolana de Ciencia, Venezuela	Incubadora Venezolana de Ciencia, Venezuela / Instituto Nacional de Salud, Bogotá, Colombia / Grupo de Investigaciones Microbiológicas-UR (GIMUR), Departamento	Alberto Paniz-Mondolfi, Marina Muñoz, Luis Perez-Garcia, Lourdes Delgado, Carolina Florez, Sergio Gomez, Angelica Rico, Lisseth Pardo, Esther C. Barros, Carolina Hernández, Jesús E. Jaimes, Aníbal A. Teherán, Ana S. Gonzalez-Reiche, Matthew M. Hernandez, Emilia Mia Sordillo, Viviana Simon, Harm van Bakel, Juan David Ramírez

		de Biología, Facultad de Ciencias Naturales, Universidad del Rosario, Bogotá, Colombia / Icahn School of Medicine at Mount Sinai, New York, USA	
EPI_ISL_476795, EPI_ISL_476796, EPI_ISL_476797	Department of Laboratory Medicine, Tan Tock Seng Hospital	Department of Laboratory Medicine, Tan Tock Seng Hospital	Chen YYC, Zair X, Li C, Tang WY, Maurer-Stroh S, Barkham TMS, Nagarajan N, Sessions OM
EPI_ISL_476801, EPI_ISL_476802, EPI_ISL_476803, EPI_ISL_476804	Hong Kong Department of Health	School of Public Health, The University of Hong Kong	Dominic N.C. Tsang, Daniel K.W. Chu, Leo L.M. Poon, Malik Peiris
EPI_ISL_476805, EPI_ISL_476806, EPI_ISL_476807, EPI_ISL_476808, EPI_ISL_476809, EPI_ISL_476810, EPI_ISL_476811, EPI_ISL_476812	Department of Laboratory Medicine Tan Tock Seng Hospital	Department of Laboratory Medicine Tan Tock Seng Hospital	Chen YYC, Zair X, Li C, Tang WY, Maurer-Stroh S, Barkham TMS, Nagarajan N, Sessions OM
EPI_ISL_476813, EPI_ISL_476814	Department of Laboratory Medicine, Tan Tock Seng Hospital	Department of Laboratory Medicine, Tan Tock Seng Hospital	Chen YYC, Zair X, Li C, Tang WY, Maurer-Stroh S, Barkham TMS, Nagarajan N, Sessions OM
EPI_ISL_476815	Department of Laboratory Medicine Tan Tock Seng Hospital	Department of Laboratory Medicine Tan Tock Seng Hospital	Chen YYC, Zair X, Li C, Tang WY, Maurer-Stroh S, Barkham TMS, Nagarajan N, Sessions OM
EPI_ISL_476816, EPI_ISL_476817, EPI_ISL_476818, EPI_ISL_476819, EPI_ISL_476820, EPI_ISL_476821	Department of Laboratory Medicine, Tan Tock Seng Hospital	Department of Laboratory Medicine, Tan Tock Seng Hospital	Chen YYC, Zair X, Li C, Tang WY, Maurer-Stroh S, Barkham TMS, Nagarajan N, Sessions OM
EPI_ISL_476832	Medical Biology Department, Kocaeli University	Medical Genetics Department, Kocaeli University	Savli H, Cine N, Sunnetci-Akkoyunlu D, Eren-Keskin S, Ilgazli A, Akhan S, Karadenizli A, Kasap M, Sayan M, Akpinar G, Canturk NZ.
EPI_ISL_476835	National Influenza Centre for Northern Greece	National Influenza Centre for Northern Greece	Maria Christoforidi
EPI_ISL_476836	National Influenza Centre for Nothern Greece	National Influenza Centre for Nothern Greece	Maria Christoforidi
EPI_ISL_476837, EPI_ISL_476838, EPI_ISL_476839	National Influenza Centre for Northern Greece	National Influenza Centre for Northern Greece	Maria Christoforidi
EPI_ISL_476840	Defence Research & Development Establishment (DRDE)	Defence Research & Development Establishment (DRDE)	Shashi Sharma, Paban Kumar Dash, Sushil Kumar Sharma, Ambuj Shrivastava, Jyoti S. Kumar
EPI_ISL_476841	National Influenza Centre for Northern Greece	National Influenza Centre for Northern Greece	Maria Christoforidi
EPI_ISL_476842	Defence Research & Development Establishment (DRDE)	Defence Research & Development Establishment (DRDE)	Shashi Sharma, Paban Kumar Dash, Sushil Kumar Sharma, Ambuj Shrivastava, Jyoti S. Kumar
EPI_ISL_476843	National Influenza Centre for Northern Greece	National Influenza Centre for Northern Greece	Maria Christoforidi
EPI_ISL_476844	Defence Research & Development Establishment (DRDE)	Defence Research & Development Establishment (DRDE)	Shashi Sharma, Paban Kumar Dash, Sushil Kumar Sharma, Ambuj Shrivastava, Jyoti S. Kumar
EPI_ISL_476845	National Influenza Centre for Northern Greece	National Influenza Centre for Northern Greece	Maria Christoforidi
EPI_ISL_476846	Defence Research & Development Establishment (DRDE)	Defence Research & Development Establishment (DRDE)	Shashi Sharma, Paban Kumar Dash, Sushil Kumar Sharma, Ambuj Shrivastava, Jyoti S. Kumar
EPI_ISL_476847	National Influenza Centre for Northern Greece	National Influenza Centre for Northern Greece	Maria Christoforidi
EPI_ISL_476848, EPI_ISL_476849, EPI_ISL_476850	Defence Research & Development Establishment (DRDE)	Defence Research & Development Establishment (DRDE)	Shashi Sharma, Paban Kumar Dash, Sushil Kumar Sharma, Ambuj Shrivastava, Jyoti S. Kumar
EPI_ISL_476851	National Influenza Centre for Northern Greece	National Influenza Centre for Northern Greece	Maria Christoforidi
EPI_ISL_476852, EPI_ISL_476853, EPI_ISL_476854	Defence Research & Development Establishment (DRDE)	Defence Research & Development Establishment (DRDE)	Shashi Sharma, Paban Kumar Dash, Sushil Kumar Sharma, Ambuj Shrivastava, Jyoti S. Kumar
EPI_ISL_476855	GMERS Medical College & Hospital, Gotri, Vadodara	Gujarat Biotechnology Research Centre	Apurvasinh Puvar, Janvi Raval, Zarna Patel, Monika Gandhi, Pinal Trivedi, Maharshi Pandya, Nidhi Patel, Nitin Savaliya, Raghawendra Kumar, Dinesh Kumar, Zuber Saiyed, Komal Patel, Labdhi Pandya, Afzal Ansari, Nikha Trivedi, Meenakshi Shah, Neena Doshi, Varsha Godbole, R D Dixit, A M Kadri, Harsh Bakshi, Chaitanya Joshi, Madhvi Joshi
EPI_ISL_476856	GMERS Medical College & Hospital, Gotri, Vadodara	Gujarat Biotechnology Research Centre	Janvi Raval, Zarna Patel, Monika Gandhi, Pinal Trivedi, Maharshi Pandya, Nidhi Patel, Nitin Savaliya, Raghawendra Kumar, Dinesh Kumar, Zuber Saiyed, Komal Patel, Labdhi Pandya, Afzal Ansari, Nikha Trivedi, Meenakshi Shah, Neena Doshi, Varsha Godbole, Apurvasinh Puvar, R D Dixit, A M Kadri, Harsh Bakshi, Chaitanya Joshi, Madhvi Joshi
EPI_ISL_476857	GMERS Medical College & Hospital, Gotri, Vadodara	Gujarat Biotechnology Research Centre	Zarna Patel, Monika Gandhi, Pinal Trivedi, Maharshi Pandya, Nidhi Patel, Nitin Savaliya, Raghawendra Kumar, Dinesh Kumar, Zuber Saiyed, Komal Patel, Labdhi Pandya, Afzal Ansari, Nikha Trivedi, Meenakshi Shah, Neena Doshi, Varsha Godbole, Apurvasinh Puvar, Janvi Raval, R D Dixit, A M Kadri, Harsh Bakshi, Chaitanya Joshi, Madhvi Joshi
EPI_ISL_476858	GMERS Medical College & Hospital, Gotri, Vadodara	Gujarat Biotechnology Research Centre	Monika Gandhi, Pinal Trivedi, Maharshi Pandya, Nidhi Patel, Nitin Savaliya, Raghawendra Kumar, Dinesh Kumar, Zuber Saiyed, Komal Patel, Labdhi Pandya, Afzal Ansari, Nikha Trivedi, Meenakshi Shah, Neena Doshi, Varsha Godbole, Apurvasinh Puvar, Janvi Raval, Zarna Patel, R D Dixit, A M Kadri, Harsh Bakshi, Chaitanya Joshi, Madhvi Joshi
EPI_ISL_476859	GMERS Medical College & Hospital, Gotri, Vadodara	Gujarat Biotechnology Research Centre	Pinal Trivedi, Maharshi Pandya, Nidhi Patel, Nitin Savaliya, Raghawendra Kumar, Dinesh Kumar, Zuber Saiyed, Komal Patel, Labdhi Pandya, Afzal Ansari, Nikha Trivedi, Meenakshi Shah, Neena Doshi, Varsha Godbole, Apurvasinh Puvar, Janvi Raval, Zarna Patel, Monika Gandhi, R D Dixit, A M Kadri, Harsh Bakshi, Chaitanya Joshi, Madhvi Joshi
EPI_ISL_476860	GMERS Medical College & Hospital, Gotri, Vadodara	Gujarat Biotechnology Research Centre	Maharshi Pandya, Nidhi Patel, Nitin Savaliya, Raghawendra Kumar, Dinesh Kumar, Zuber Saiyed, Komal Patel, Labdhi Pandya, Afzal Ansari, Nikha Trivedi, Meenakshi Shah, Neena Doshi, Varsha Godbole, Apurvasinh Puvar, Janvi Raval, Zarna Patel, Monika Gandhi, Pinal Trivedi, R D Dixit, A M Kadri, Harsh Bakshi, Chaitanya Joshi, Madhvi Joshi
EPI_ISL_476861	GMERS Medical College & Hospital, Gotri, Vadodara	Gujarat Biotechnology Research Centre	Nidhi Patel, Nitin Savaliya, Raghawendra Kumar, Dinesh Kumar, Zuber Saiyed, Komal Patel, Labdhi Pandya, Afzal Ansari, Nikha Trivedi, Meenakshi Shah, Neena Doshi, Varsha Godbole, Apurvasinh Puvar, Janvi Raval, Zarna Patel, Monika Gandhi, Pinal Trivedi, Maharshi Pandya, R D Dixit, A M Kadri, Harsh Bakshi, Chaitanya Joshi, Madhvi Joshi
EPI_ISL_476862	GMERS Medical College & Hospital, Gotri, Vadodara	Gujarat Biotechnology Research Centre	Nitin Savaliya, Raghawendra Kumar, Dinesh Kumar, Zuber Saiyed, Komal Patel, Labdhi Pandya, Afzal Ansari, Nikha Trivedi, Meenakshi Shah, Neena Doshi, Varsha Godbole, Apurvasinh Puvar, Janvi Raval, Zarna Patel, Monika Gandhi, Pinal Trivedi, Maharshi Pandya, Nidhi Patel, R D Dixit, A M Kadri, Harsh Bakshi, Chaitanya Joshi, Madhvi Joshi
EPI_ISL_476863	GMERS Medical College and Hospital, Gandhinagar	Gujarat Biotechnology Research Centre	Raghawendra Kumar, Dinesh Kumar, Zuber Saiyed, Komal Patel, Labdhi Pandya, Afzal Ansari, Nikha Trivedi, Seema Bhatt, Gaurishankar Shrimali, Bhavesh Modi, Bharti Rajani, Apurvasinh Puvar, Janvi Raval, Zarna Patel, Monika Gandhi, Pinal Trivedi, Maharshi Pandya, Nidhi Patel, Nitin Savaliya, R D Dixit, A M Kadri, Harsh Bakshi, Chaitanya Joshi, Madhvi Joshi
EPI_ISL_476864	GMERS Medical College and Hospital, Gandhinagar	Gujarat Biotechnology Research Centre	Dinesh Kumar, Zuber Saiyed, Komal Patel, Labdhi Pandya, Afzal Ansari, Nikha Trivedi, Seema Bhatt, Gaurishankar Shrimali, Bhavesh Modi, Bharti Rajani, Apurvasinh Puvar, Janvi Raval, Zarna Patel, Monika Gandhi, Pinal Trivedi, Maharshi Pandya, Nidhi Patel, Nitin Savaliya, Raghawendra Kumar, R D Dixit, A M Kadri, Harsh Bakshi, Chaitanya Joshi, Madhvi Joshi
EPI_ISL_476865	GMERS Medical College and Hospital, Gandhinagar	Gujarat Biotechnology Research Centre	Zuber Saiyed, Komal Patel, Labdhi Pandya, Afzal Ansari, Nikha Trivedi, Seema Bhatt, Gaurishankar Shrimali, Bhavesh Modi, Bharti Rajani, Apurvasinh Puvar, Janvi Raval, Zarna Patel, Monika Gandhi, Pinal Trivedi, Maharshi Pandya, Nidhi Patel, Nitin Savaliya, Raghawendra Kumar, Dinesh Kumar, R D Dixit, A M Kadri, Harsh Bakshi, Chaitanya Joshi, Madhvi Joshi
EPI_ISL_476866	GMERS Medical College and Hospital, Gandhinagar	Gujarat Biotechnology Research Centre	Komal Patel, Labdhi Pandya, Afzal Ansari, Nikha Trivedi, Seema Bhatt, Gaurishankar Shrimali, Bhavesh Modi, Bharti Rajani, Apurvasinh Puvar, Janvi Raval, Zarna Patel, Monika Gandhi, Pinal Trivedi, Maharshi Pandya, Nidhi Patel, Nitin Savaliya, Raghawendra Kumar, Dinesh Kumar, Zuber Saiyed, R D Dixit, A M Kadri, Harsh Bakshi, Chaitanya Joshi, Madhvi Joshi
EPI_ISL_476867	Banas Medical College and Research Institute	Gujarat Biotechnology Research Centre	Labdhi Pandya, Afzal Ansari, Nikha Trivedi, Radhika Khara, Sunil R Joshi, Viren s Doshi, Apurvasinh Puvar, Janvi Raval, Zarna Patel, Monika Gandhi,

			Pinal Trivedi, Maharshi Pandya, Nidhi Patel, Nitin Savaliya, Raghawendra Kumar, Dinesh Kumar, Zuber Saiyed, Komal Patel, R D Dixit, A M Kadri, Harsh Bakshi, Chaitanya Joshi, Madhvi Joshi
EPI_ISL_476868	Banas Medical College and Research Institute	Gujarat Biotechnology Research Centre	Afzal Ansari, Nikha Trivedi, Radhika Khara, Sunil R Joshi, Viren s Doshi, Apurvasinh Puvar, Janvi Raval, Zarna Patel, Monika Gandhi, Pinal Trivedi, Maharshi Pandya, Nidhi Patel, Nitin Savaliya, Raghawendra Kumar, Dinesh Kumar, Zuber Saiyed, Komal Patel, Labdhi Pandya, R D Dixit, A M Kadri, Harsh Bakshi, Chaitanya Joshi, Madhvi Joshi
EPI_ISL_476869	Department of MicroBiology, Government Medical College, Surat	Gujarat Biotechnology Research Centre	Nikha Trivedi, Naresh Chauhan, Summaiya Mullan, Amit gamit, Apurvasinh Puvar, Janvi Raval, Zarna Patel, Monika Gandhi, Pinal Trivedi, Maharshi Pandya, Nidhi Patel, Nitin Savaliya, Raghawendra Kumar, Dinesh Kumar, Zuber Saiyed, Komal Patel, Labdhi Pandya, Afzal Ansari, R D Dixit, A M Kadri, Harsh Bakshi, Chaitanya Joshi, Madhvi Joshi
EPI_ISL_476870	Department of MicroBiology, Government Medical College, Surat	Gujarat Biotechnology Research Centre	Naresh Chauhan, Summaiya Mullan, Amit gamit, Apurvasinh Puvar, Janvi Raval, Zarna Patel, Monika Gandhi, Pinal Trivedi, Maharshi Pandya, Nidhi Patel, Nitin Savaliya, Raghawendra Kumar, Dinesh Kumar, Zuber Saiyed, Komal Patel, Labdhi Pandya, Afzal Ansari, Nikha Trivedi, R D Dixit, A M Kadri, Harsh Bakshi, Chaitanya Joshi, Madhvi Joshi
EPI_ISL_476871	Department of MicroBiology, Government Medical College, Surat	Gujarat Biotechnology Research Centre	Summaiya Mullan, Amit gamit, Apurvasinh Puvar, Janvi Raval, Zarna Patel, Monika Gandhi, Pinal Trivedi, Maharshi Pandya, Nidhi Patel, Nitin Savaliya, Raghawendra Kumar, Dinesh Kumar, Zuber Saiyed, Komal Patel, Labdhi Pandya, Afzal Ansari, Nikha Trivedi, Naresh Chauhan, R D Dixit, A M Kadri, Harsh Bakshi, Chaitanya Joshi, Madhvi Joshi
EPI_ISL_476872	Department of MicroBiology, Government Medical College, Surat	Gujarat Biotechnology Research Centre	Amit gamit, Apurvasinh Puvar, Janvi Raval, Zarna Patel, Monika Gandhi, Pinal Trivedi, Maharshi Pandya, Nidhi Patel, Nitin Savaliya, Raghawendra Kumar, Dinesh Kumar, Zuber Saiyed, Komal Patel, Labdhi Pandya, Afzal Ansari, Nikha Trivedi, Naresh Chauhan, Summaiya Mullan, R D Dixit, A M Kadri, Harsh Bakshi, Chaitanya Joshi, Madhvi Joshi
EPI_ISL_476873	Department of MicroBiology, Government Medical College, Surat	Gujarat Biotechnology Research Centre	Apurvasinh Puvar, Janvi Raval, Zarna Patel, Monika Gandhi, Pinal Trivedi, Maharshi Pandya, Nidhi Patel, Nitin Savaliya, Raghawendra Kumar, Dinesh Kumar, Zuber Saiyed, Komal Patel, Labdhi Pandya, Afzal Ansari, Nikha Trivedi, Naresh Chauhan, Summaiya Mullan, Amit gamit, R D Dixit, A M Kadri, Harsh Bakshi, Chaitanya Joshi, Madhvi Joshi
EPI_ISL_476874	Department of MicroBiology, Government Medical College, Surat	Gujarat Biotechnology Research Centre	Janvi Raval, Zarna Patel, Monika Gandhi, Pinal Trivedi, Maharshi Pandya, Nidhi Patel, Nitin Savaliya, Raghawendra Kumar, Dinesh Kumar, Zuber Saiyed, Komal Patel, Labdhi Pandya, Afzal Ansari, Nikha Trivedi, Naresh Chauhan, Summaiya Mullan, Amit gamit, Apurvasinh Puvar, R D Dixit, A M Kadri, Harsh Bakshi, Chaitanya Joshi, Madhvi Joshi
EPI_ISL_476875	Department of MicroBiology, Government Medical College, Surat	Gujarat Biotechnology Research Centre	Zarna Patel, Monika Gandhi, Pinal Trivedi, Maharshi Pandya, Nidhi Patel, Nitin Savaliya, Raghawendra Kumar, Dinesh Kumar, Zuber Saiyed, Komal Patel, Labdhi Pandya, Afzal Ansari, Nikha Trivedi, Naresh Chauhan, Summaiya Mullan, Amit gamit, Apurvasinh Puvar, Janvi Raval, R D Dixit, A M Kadri, Harsh Bakshi, Chaitanya Joshi, Madhvi Joshi
EPI_ISL_476876	Department of MicroBiology, Government Medical College, Surat	Gujarat Biotechnology Research Centre	Pinal Trivedi, Maharshi Pandya, Nidhi Patel, Nitin Savaliya, Raghawendra Kumar, Dinesh Kumar, Zuber Saiyed, Komal Patel, Labdhi Pandya, Afzal Ansari, Nikha Trivedi, Naresh Chauhan, Summaiya Mullan, Amit gamit, Apurvasinh Puvar, Janvi Raval, Zarna Patel, Monika Gandhi, R D Dixit, A M Kadri, Harsh Bakshi, Chaitanya Joshi, Madhvi Joshi
EPI_ISL_476877	Department of MicroBiology, Government Medical College, Surat	Gujarat Biotechnology Research Centre	Maharshi Pandya, Nidhi Patel, Nitin Savaliya, Raghawendra Kumar, Dinesh Kumar, Zuber Saiyed, Komal Patel, Labdhi Pandya, Afzal Ansari, Nikha Trivedi, Naresh Chauhan, Summaiya Mullan, Amit gamit, Apurvasinh Puvar, Janvi Raval, Zarna Patel, Monika Gandhi, Pinal Trivedi, R D Dixit, A M Kadri, Harsh Bakshi, Chaitanya Joshi, Madhvi Joshi
EPI_ISL_476878	Department of MicroBiology, Government Medical College, Surat	Gujarat Biotechnology Research Centre	Nidhi Patel, Nitin Savaliya, Raghawendra Kumar, Dinesh Kumar, Zuber Saiyed, Komal Patel, Labdhi Pandya, Afzal Ansari, Nikha Trivedi, Naresh Chauhan, Summaiya Mullan, Amit gamit, Apurvasinh Puvar, Janvi Raval, Zarna Patel, Monika Gandhi, Pinal Trivedi, Maharshi Pandya, R D Dixit, A M Kadri, Harsh Bakshi, Chaitanya Joshi, Madhvi Joshi
EPI_ISL_476879	Department of MicroBiology, Government Medical College, Surat	Gujarat Biotechnology Research Centre	Nitin Savaliya, Raghawendra Kumar, Dinesh Kumar, Zuber Saiyed, Komal Patel, Labdhi Pandya, Afzal Ansari, Nikha Trivedi, Naresh Chauhan, Summaiya Mullan, Amit gamit, Apurvasinh Puvar, Janvi Raval, Zarna Patel, Monika Gandhi, Pinal Trivedi, Maharshi Pandya, Nidhi Patel, R D Dixit, A M Kadri, Harsh Bakshi, Chaitanya Joshi, Madhvi Joshi
EPI_ISL_476880	Department of MicroBiology, Government Medical College, Surat	Gujarat Biotechnology Research Centre	Raghawendra Kumar, Dinesh Kumar, Zuber Saiyed, Komal Patel, Labdhi Pandya, Afzal Ansari, Nikha Trivedi, Naresh Chauhan, Summaiya Mullan, Amit gamit, Apurvasinh Puvar, Janvi Raval, Zarna Patel, Monika Gandhi, Pinal Trivedi, Maharshi Pandya, Nidhi Patel, Nitin Savaliya, R D Dixit, A M Kadri, Harsh Bakshi, Chaitanya Joshi, Madhvi Joshi
EPI_ISL_476881	Department of MicroBiology, Government Medical College, Surat	Gujarat Biotechnology Research Centre	Dinesh Kumar, Zuber Saiyed, Komal Patel, Labdhi Pandya, Afzal Ansari, Nikha Trivedi, Naresh Chauhan, Summaiya Mullan, Amit gamit, Apurvasinh Puvar, Janvi Raval, Zarna Patel, Monika Gandhi, Pinal Trivedi, Maharshi Pandya, Nidhi Patel, Nitin Savaliya, Raghawendra Kumar, R D Dixit, A M Kadri, Harsh Bakshi, Chaitanya Joshi, Madhvi Joshi
EPI_ISL_476882	Department of MicroBiology, Government Medical College, Surat	Gujarat Biotechnology Research Centre	Zuber Saiyed, Komal Patel, Labdhi Pandya, Afzal Ansari, Nikha Trivedi, Naresh Chauhan, Summaiya Mullan, Amit gamit, Apurvasinh Puvar, Janvi Raval, Zarna Patel, Monika Gandhi, Pinal Trivedi, Maharshi Pandya, Nidhi Patel, Nitin Savaliya, Raghawendra Kumar, Dinesh Kumar, R D Dixit, A M Kadri, Harsh Bakshi, Chaitanya Joshi, Madhvi Joshi
EPI_ISL_476883, EPI_ISL_476884, EPI_ISL_476885, EPI_ISL_476886, EPI_ISL_476887, EPI_ISL_476888, EPI_ISL_476889, EPI_ISL_476890, EPI_ISL_476891, EPI_ISL_476892, EPI_ISL_476893, EPI_ISL_476894, EPI_ISL_476895, EPI_ISL_476896			
see above	Defence Research & Development Establishment (DRDE)	Defence Research & Development Establishment (DRDE)	Shashi Sharma, Paban Kumar Dash, Sushil Kumar Sharma, Ambuj Shrivastava, Jyoti S. Kumar
EPI_ISL_477015	Institute of Microbiology, Universidad San Francisco de Quito	Institute of Microbiology, Universidad San Francisco de Quito	Sully Márquez, Belén Prado-Vivar, Juan José Guadalupe, Monica Becerra-Wong, Carla Torres, Bernardo Gutiérrez, Jorge Luis Velez, Verónica Barragán, Patricio Rojas-Silva, Gabriel Trueba, Michelle Grunauer, Paúl Cárdenas
EPI_ISL_477016	Institute of Microbiology, Universidad San Francisco de Quito	Institute of Microbiology, Universidad San Francisco de Quito	Juan José Guadalupe, Sully Márquez, Belén Prado-Vivar, Monica Becerra-Wong, Carla Torres, Bernardo Gutiérrez, Jorge Luis Velez, Verónica Barragán, Patricio Rojas-Silva, Gabriel Trueba, Michelle Grunauer, Paúl Cárdenas
EPI_ISL_477141, EPI_ISL_477142, EPI_ISL_477143, EPI_ISL_477144, EPI_ISL_477145, EPI_ISL_477146, EPI_ISL_477147, EPI_ISL_477148, EPI_ISL_477149, EPI_ISL_477150, EPI_ISL_477151, EPI_ISL_477152, EPI_ISL_477153, EPI_ISL_477154, EPI_ISL_477155, EPI_ISL_477156, EPI_ISL_477157, EPI_ISL_477158, EPI_ISL_477159			
see above	Institut Pasteur Dakar	Institut Pasteur de Dakar	Ndongo Dia, Moussa Moise Diagne, Mamadou Diop, Mamadou Malado Jallow, Marie Henriette Dior Ndione, Safietou Sankhe, Ousmane Faye, Amadou Alpha Sall.
EPI_ISL_477163	Laboratory of Dr. John Lednicky	University of Florida	John A. Lednicky, Maha A. Elbadry, Kuttichantran Subramaniam, Thomas B. Waltzek, John Glenn Morris, Jr.
EPI_ISL_477170	Department of Laboratory, Medicine Tan Tock Seng Hospital	Department of Laboratory, Medicine Tan Tock Seng Hospital	Chen YYC, Zair X, Li C, Tang WY, Maurer-Stroh S, Barkham TMS, Nagarajan N, Sessions OM
EPI_ISL_477171	Department of Laboratory, Medicine Tan Tock Seng Hospital	Department of Laboratory, Medicine Tan Tock Seng Hospital	Chen YYC, Zair X, Li C, Tang WY, Maurer-Stroh S, Barkham TMS, Nagarajan N, Sessions OM
EPI_ISL_477172, EPI_ISL_477174, EPI_ISL_477175, EPI_ISL_477177, EPI_ISL_477178, EPI_ISL_477180, EPI_ISL_477182	Department of Laboratory Medicine Tan Tock Seng Hospital	Department of Laboratory Medicine Tan Tock Seng Hospital	Chen YYC, Zair X, Li C, Tang WY, Maurer-Stroh S, Barkham TMS, Nagarajan N, Sessions OM
EPI_ISL_477183	Department of MicroBiology, Government Medical College, Surat	Gujarat Biotechnology Research Centre	Monika Gandhi, Pinal Trivedi, Maharshi Pandya, Nidhi Patel, Nitin Savaliya, Raghawendra Kumar, Dinesh Kumar, Zuber Saiyed, Komal Patel, Labdhi Pandya, Afzal Ansari, Nikha Trivedi, Naresh Chauhan, Summaiya Mullan, Amit gamit, Apurvasinh Puvar, Janvi Raval, Zarna Patel, R D Dixit, A M Kadri, Harsh Bakshi, Chaitanya Joshi, Madhvi Joshi
EPI_ISL_477184, EPI_ISL_477187, EPI_ISL_477188, EPI_ISL_477189, EPI_ISL_477190, EPI_ISL_477191, EPI_ISL_477192	Department of Laboratory Medicine Tan Tock Seng Hospital	Department of Laboratory Medicine Tan Tock Seng Hospital	Chen YYC, Zair X, Li C, Tang WY, Maurer-Stroh S, Barkham TMS, Nagarajan N, Sessions OM
EPI_ISL_477193, EPI_ISL_477194, EPI_ISL_477202	Istituto Zooprofilattico Sperimentale Puglia e Basilicata;	Beaconlab (Bioinformatics, Evolution and Comparative Genomics lab), Dept of Biosciences, University on Mila	Parisi A.,Pesole G., Manzari C., Chiara M.

EPI_ISL_479482, EPI_ISL_479483, EPI_ISL_479484, EPI_ISL_479485, EPI_ISL_479486, EPI_ISL_479487, EPI_ISL_479488, EPI_ISL_479489, EPI_ISL_479490, EPI_ISL_479491, EPI_ISL_479492			
see above	Department of Laboratory Medicine Tan Tock Seng Hospital	Department of Laboratory Medicine Tan Tock Seng Hospital	Chen YYC, Zair X, Li C, Tang WY, Maurer-Stroh S, Barkham TMS, Nagarajan N, Sessions OM
EPI_ISL_479493, EPI_ISL_479495, EPI_ISL_479498, EPI_ISL_479499, EPI_ISL_479501, EPI_ISL_479502, EPI_ISL_479505, EPI_ISL_479507, EPI_ISL_479509, EPI_ISL_479510, EPI_ISL_479512, EPI_ISL_479513, EPI_ISL_479514, EPI_ISL_479516, EPI_ISL_479517, EPI_ISL_479519, EPI_ISL_479520, EPI_ISL_479521, EPI_ISL_479522, EPI_ISL_479523, EPI_ISL_479525, EPI_ISL_479526, EPI_ISL_479527, EPI_ISL_479528, EPI_ISL_479529, EPI_ISL_479530, EPI_ISL_479531, EPI_ISL_479532, EPI_ISL_479533, EPI_ISL_479534, EPI_ISL_479535, EPI_ISL_479536, EPI_ISL_479537, EPI_ISL_479538, EPI_ISL_479539, EPI_ISL_479540, EPI_ISL_479541, EPI_ISL_479543, EPI_ISL_479544, EPI_ISL_479546, EPI_ISL_479547, EPI_ISL_479548, EPI_ISL_479549, EPI_ISL_479550, EPI_ISL_479551, EPI_ISL_479552, EPI_ISL_479553, EPI_ISL_479572			
see above	NIV Influenza	NIV Influenza	Potdar V
EPI_ISL_479616, EPI_ISL_479617	Laboratory of Molecular Virology of the International Centre for Genetic Engineering and Biotechnology (ICGEB)	ARGO Open Lab Platform for Genome Sequencing	Licastro, D, Rajasekharan S, Dal Monego S, Segat L, D'Agaro P, Salton F, Confalonieri P, Confalonieri M Marcello A
EPI_ISL_479618, EPI_ISL_479619	Laboratory of Molecular Virology of the International Centre for Genetic Engineering and Biotechnology (ICGEB)	ARGO Open Lab Platform for Genome Sequencing	Licastro, D, Rajasekharan S, Dal Monego S, Segat L, D'Agaro P, Salton F, Confalonieri P, Confalonieri M, Marcello A
EPI_ISL_479620, EPI_ISL_479621, EPI_ISL_479622, EPI_ISL_479623, EPI_ISL_479624	Molecular diagnostic laboratory of Federal Budget Institution of Science "Central Research Institute of Epidemiology" of The Federal Service on Customers' Rights Protection and Human Well-being Surveillance	Group of Genomics and Postgenomic Technologies of Central Research Institute of Epidemilology	Speranskaya AS, Kaptelova VV,Valdokhina AV, Bulanenko VP, Samoilov AE, Korneenko EV, Sizova TV, Tivanova EV, Shipulina OY, Akimkin VG
EPI_ISL_479657, EPI_ISL_479658, EPI_ISL_479659	NIV Influenza	NIV Influenza	Potdar V
EPI_ISL_479756, EPI_ISL_479757, EPI_ISL_479758	National Institute of Hygiene and Epidemiology (NIHE)	National Key Laboratory of Gene Technology, Institute of Biotechnology (IBT)	Le Tung Lam, Nguyen Hong Trang, Ho Thi Thuong, Tran Huyen Linh, Ung Thi Hong Trang, Le Thi Thanh, Nguyen Vu Son, Vuong Duc Cuong, Tran Thu Huong, Pham Thi Hien, Nguyen Phuong Anh, Nguyen Le Khanh Hang, Hoang Vu Mai Phuong, Hoang Ha, Taichiro Takemura, Futoshi Hasebe, Chu Hoang Ha, Le Quynh Mai, Dang Duc Anh, Truong Nam Hai
EPI_ISL_479790, EPI_ISL_479791	Laboratory of Molecular Virology of the International Centre for Genetic Engineering and Biotechnology (ICGEB)	ARGO Open Lab Platform for Genome Sequencing	Licastro, D, Rajasekharan S, Dal Monego S, Segat L, D'Agaro P, Salton F, Confalonieri P, Confalonieri M, Marcello A
EPI_ISL_479792, EPI_ISL_479793, EPI_ISL_479794, EPI_ISL_479795	Hokkaido Institute of Public Health	Pathogen Genomics Center, National Institute of Infectious Diseases	Tsuyoshi Sekizuka, Rika Komagome, Kentaro Itokawa, Rina Tanaka, Masanori Hashino, Hajime Kamiya, Motoi Suzuki, Makoto Kuroda
EPI_ISL_479796	Ishikawa Prefectural Institute of Public Health and Environmental Science	Pathogen Genomics Center, National Institute of Infectious Diseases	Tsuyoshi Sekizuka, Sanae Kuramoto, Eri Nariai, Kentaro Itokawa, Rina Tanaka, Masanori Hashino, Hajime Kamiya, Motoi Suzuki, Makoto Kuroda
EPI_ISL_479797, EPI_ISL_479798	Sagamihara City Public Health Research Institute	Pathogen Genomics Center, National Institute of Infectious Diseases	Tsuyoshi Sekizuka, Hiroshi Nakamura, Kentaro Itokawa, Rina Tanaka, Masanori Hashino, Hajime Kamiya, Motoi Suzuki, Makoto Kuroda
EPI_ISL_479799, EPI_ISL_479800	Sapporo City Institute of Public Health	Pathogen Genomics Center, National Institute of Infectious Diseases	Tsuyoshi Sekizuka, Asami Ohnishi, Kentaro Itokawa, Rina Tanaka, Masanori Hashino, Hajime Kamiya, Motoi Suzuki, Makoto Kuroda
EPI_ISL_479801	Hokkaido Institute of Public Health	Pathogen Genomics Center, National Institute of Infectious Diseases	Tsuyoshi Sekizuka, Rika Komagome, Kentaro Itokawa, Rina Tanaka, Masanori Hashino, Hajime Kamiya, Motoi Suzuki, Makoto Kuroda
EPI_ISL_479802, EPI_ISL_479803, EPI_ISL_479804	Sagamihara City Public Health Research Institute	Pathogen Genomics Center, National Institute of Infectious Diseases	Tsuyoshi Sekizuka, Hiroshi Nakamura, Kentaro Itokawa, Rina Tanaka, Masanori Hashino, Hajime Kamiya, Motoi Suzuki, Makoto Kuroda
EPI_ISL_479805, EPI_ISL_479806, EPI_ISL_479807, EPI_ISL_479808	Saitama Prefectural Institute of Public Health	Pathogen Genomics Center, National Institute of Infectious Diseases	Tsuyoshi Sekizuka, Hayato Ehara, Kentaro Itokawa, Rina Tanaka, Masanori Hashino, Hajime Kamiya, Motoi Suzuki, Makoto Kuroda
EPI_ISL_479809, EPI_ISL_479810, EPI_ISL_479811	Chiba Prefectural Institute of Public Health	Pathogen Genomics Center, National Institute of Infectious Diseases	Tsuyoshi Sekizuka, Masakatsu Taira, Kentaro Itokawa, Rina Tanaka, Masanori Hashino, Hajime Kamiya, Motoi Suzuki, Makoto Kuroda
EPI_ISL_479812, EPI_ISL_479813, EPI_ISL_479814, EPI_ISL_479815, EPI_ISL_479816, EPI_ISL_479817, EPI_ISL_479818, EPI_ISL_479819, EPI_ISL_479820	Hokkaido Institute of Public Health	Pathogen Genomics Center, National Institute of Infectious Diseases	Tsuyoshi Sekizuka, Rika Komagome, Kentaro Itokawa, Rina Tanaka, Masanori Hashino, Hajime Kamiya, Motoi Suzuki, Makoto Kuroda
EPI_ISL_479821, EPI_ISL_479822	Department of Infectious Diseases, Kobe Institute of Health	Pathogen Genomics Center, National Institute of Infectious Diseases	Tsuyoshi Sekizuka, Ryohei Nomoto, Kentaro Itokawa, Rina Tanaka, Masanori Hashino, Hajime Kamiya, Motoi Suzuki, Makoto Kuroda
EPI_ISL_479823	Kochi Prefectural Institute of Public Health	Pathogen Genomics Center, National Institute of Infectious Diseases	Tsuyoshi Sekizuka, Akihiko Tokaji, Kentaro Itokawa, Rina Tanaka, Masanori Hashino, Hajime Kamiya, Motoi Suzuki, Makoto Kuroda
EPI_ISL_479824	Kumamoto PrefecturalInstituteof Public-Health and Environmental Science	Pathogen Genomics Center, National Institute of Infectious Diseases	Tsuyoshi Sekizuka, Shunsuke Yahiro, Kentaro Itokawa, Rina Tanaka, Masanori Hashino, Hajime Kamiya, Motoi Suzuki, Makoto Kuroda
EPI_ISL_479825	Tokyo Metropolitan Institute of Public Health	Pathogen Genomics Center, National Institute of Infectious Diseases	Tsuyoshi Sekizuka, Kenji Sadamasu, Takashi Chiba, Mami Nagashima, Kentaro Itokawa, Rina Tanaka, Masanori Hashino, Hajime Kamiya, Motoi Suzuki, Makoto Kuroda
EPI_ISL_479826, EPI_ISL_479827, EPI_ISL_479828, EPI_ISL_479829, EPI_ISL_479830, EPI_ISL_479831, EPI_ISL_479832, EPI_ISL_479833, EPI_ISL_479834, EPI_ISL_479835, EPI_ISL_479836, EPI_ISL_479837, EPI_ISL_479838, EPI_ISL_479839, EPI_ISL_479840, EPI_ISL_479841, EPI_ISL_479842, EPI_ISL_479843, EPI_ISL_479844, EPI_ISL_479845, EPI_ISL_479846, EPI_ISL_479847, EPI_ISL_479848, EPI_ISL_479849			
see above	Sapporo City Institute of Public Health	Pathogen Genomics Center, National Institute of Infectious Diseases	Tsuyoshi Sekizuka, Asami Ohnishi, Kentaro Itokawa, Rina Tanaka, Masanori Hashino, Hajime Kamiya, Motoi Suzuki, Makoto Kuroda
EPI_ISL_479850, EPI_ISL_479851, EPI_ISL_479852, EPI_ISL_479853, EPI_ISL_479854	Gunma Prefectural Institute of Public Health and Environmental Sciences	Pathogen Genomics Center, National Institute of Infectious Diseases	Tsuyoshi Sekizuka, Hiroyuki Tsukagoshi, Kentaro Itokawa, Rina Tanaka, Masanori Hashino, Hajime Kamiya, Motoi Suzuki, Makoto Kuroda
EPI_ISL_479855, EPI_ISL_479856, EPI_ISL_479857, EPI_ISL_479858, EPI_ISL_479859, EPI_ISL_479860, EPI_ISL_479861	Department of Infectious Diseases, Kobe Institute of Health	Pathogen Genomics Center, National Institute of Infectious Diseases	Tsuyoshi Sekizuka, Ryohei Nomoto, Kentaro Itokawa, Rina Tanaka, Masanori Hashino, Hajime Kamiya, Motoi Suzuki, Makoto Kuroda
EPI_ISL_479862, EPI_ISL_479863, EPI_ISL_479864, EPI_ISL_479865, EPI_ISL_479866, EPI_ISL_479867	Wakayama Prefectural Research Center of Environment and Public Health	Pathogen Genomics Center, National Institute of Infectious Diseases	Tsuyoshi Sekizuka, Fumio Terasoma, Yosuke Hamajima, Kentaro Itokawa, Rina Tanaka, Masanori Hashino, Hajime Kamiya, Motoi Suzuki, Makoto Kuroda
EPI_ISL_479868	Department of Infectious Diseases, Kobe Institute of Health	Pathogen Genomics Center, National Institute of Infectious Diseases	Tsuyoshi Sekizuka, Ryohei Nomoto, Kentaro Itokawa, Rina Tanaka, Masanori Hashino, Hajime Kamiya, Motoi Suzuki, Makoto Kuroda
EPI_ISL_479869	Niigata Prefectural Institute of Public Health and Environmental Sciences	Pathogen Genomics Center, National Institute of Infectious Diseases	Tsuyoshi Sekizuka, Reiko Arai, Kentaro Itokawa, Rina Tanaka, Masanori Hashino, Hajime Kamiya, Motoi Suzuki, Makoto Kuroda
EPI_ISL_479870, EPI_ISL_479871	Sagamihara City Public Health Research Institute	Pathogen Genomics Center, National Institute of Infectious Diseases	Tsuyoshi Sekizuka, Hiroshi Nakamura, Kentaro Itokawa, Rina Tanaka, Masanori Hashino, Hajime Kamiya, Motoi Suzuki, Makoto Kuroda
EPI_ISL_479872, EPI_ISL_479873, EPI_ISL_479874, EPI_ISL_479875, EPI_ISL_479876, EPI_ISL_479877, EPI_ISL_479878, EPI_ISL_479879, EPI_ISL_479880, EPI_ISL_479881, EPI_ISL_479882, EPI_ISL_479883, EPI_ISL_479884, EPI_ISL_479885			
see above	Sapporo City Institute of Public Health	Pathogen Genomics Center, National Institute of Infectious	Tsuyoshi Sekizuka, Asami Ohnishi, Kentaro Itokawa, Rina Tanaka, Masanori Hashino, Hajime Kamiya, Motoi Suzuki, Makoto Kuroda

		Diseases	
EPI_ISL_479886, EPI_ISL_479887, EPI_ISL_479888, EPI_ISL_479889, EPI_ISL_479890, EPI_ISL_479891, EPI_ISL_479892, EPI_ISL_479893, EPI_ISL_479894, EPI_ISL_479895	Tokyo Metropolitan Institute of Public Health	Pathogen Genomics Center, National Institute of Infectious Diseases	Tsuyoshi Sekizuka, Kenji Sadamasu, Takashi Chiba, Mami Nagashima, Kentaro Itokawa, Rina Tanaka, Masanori Hashino, Hajime Kamiya, Motoi Suzuki, Makoto Kuroda
EPI_ISL_479896, EPI_ISL_479897, EPI_ISL_479898, EPI_ISL_479899, EPI_ISL_479900, EPI_ISL_479901	Gunma Prefectural Institute of Public Health and Environmental Sciences	Pathogen Genomics Center, National Institute of Infectious Diseases	Tsuyoshi Sekizuka, Hiroyuki Tsukagoshi, Kentaro Itokawa, Rina Tanaka, Masanori Hashino, Hajime Kamiya, Motoi Suzuki, Makoto Kuroda
EPI_ISL_479902	Niigata Prefectural Institute of Public Health and Environmental Sciences	Pathogen Genomics Center, National Institute of Infectious Diseases	Tsuyoshi Sekizuka, Reiko Arai, Kentaro Itokawa, Rina Tanaka, Masanori Hashino, Hajime Kamiya, Motoi Suzuki, Makoto Kuroda
EPI_ISL_479903, EPI_ISL_479904, EPI_ISL_479905, EPI_ISL_479906, EPI_ISL_479907, EPI_ISL_479908, EPI_ISL_479909, EPI_ISL_479910, EPI_ISL_479911, EPI_ISL_479912	Himeji City Institute of Environment and Health	Pathogen Genomics Center, National Institute of Infectious Diseases	Tsuyoshi Sekizuka, Kentaro Itokawa, Rina Tanaka, Masanori Hashino, Hajime Kamiya, Motoi Suzuki, Makoto Kuroda
EPI_ISL_479913, EPI_ISL_479914, EPI_ISL_479915, EPI_ISL_479916, EPI_ISL_479917, EPI_ISL_479918, EPI_ISL_479919, EPI_ISL_479920, EPI_ISL_479921, EPI_ISL_479922, EPI_ISL_479923, EPI_ISL_479924			
see above	Niigata City Public Health Research Institute	Pathogen Genomics Center, National Institute of Infectious Diseases	Tsuyoshi Sekizuka, Yurie Takahashi, Kentaro Itokawa, Rina Tanaka, Masanori Hashino, Hajime Kamiya, Motoi Suzuki, Makoto Kuroda
EPI_ISL_479925, EPI_ISL_479926, EPI_ISL_479927	Sakai City Institute of Public Health	Pathogen Genomics Center, National Institute of Infectious Diseases	Tsuyoshi Sekizuka, Tatsuya Miyoshi, Kentaro Itokawa, Rina Tanaka, Masanori Hashino, Hajime Kamiya, Motoi Suzuki, Makoto Kuroda
EPI_ISL_479928, EPI_ISL_479929, EPI_ISL_479930, EPI_ISL_479931, EPI_ISL_479932, EPI_ISL_479933, EPI_ISL_479934, EPI_ISL_479935	Saitama Prefectural Institute of Public Health	Pathogen Genomics Center, National Institute of Infectious Diseases	Tsuyoshi Sekizuka, Hayato Ehara, Kentaro Itokawa, Rina Tanaka, Masanori Hashino, Hajime Kamiya, Motoi Suzuki, Makoto Kuroda
EPI_ISL_479936, EPI_ISL_479937, EPI_ISL_479938, EPI_ISL_479939, EPI_ISL_479940, EPI_ISL_479941, EPI_ISL_479942, EPI_ISL_479943	Ibaraki Prefectural Institute of Public Health	Pathogen Genomics Center, National Institute of Infectious Diseases	Tsuyoshi Sekizuka, Keiko Goto, Kentaro Itokawa, Rina Tanaka, Masanori Hashino, Hajime Kamiya, Motoi Suzuki, Makoto Kuroda
EPI_ISL_479944, EPI_ISL_479945, EPI_ISL_479946, EPI_ISL_479947, EPI_ISL_479948, EPI_ISL_479949, EPI_ISL_479950, EPI_ISL_479951, EPI_ISL_479952, EPI_ISL_479953, EPI_ISL_479954, EPI_ISL_479955, EPI_ISL_479956, EPI_ISL_479957, EPI_ISL_479958			
see above	Osaka Institute of Public Health	Pathogen Genomics Center, National Institute of Infectious Diseases	Tsuyoshi Sekizuka, Satoshi Hiroi, Saeko Morikawa, Kazushi Motomura, Kentaro Itokawa, Rina Tanaka, Masanori Hashino, Hajime Kamiya, Motoi Suzuki, Makoto Kuroda
EPI_ISL_479959, EPI_ISL_479960, EPI_ISL_479961, EPI_ISL_479962, EPI_ISL_479963, EPI_ISL_479964, EPI_ISL_479965	Tokyo Metropolitan Institute of Public Health	Pathogen Genomics Center, National Institute of Infectious Diseases	Tsuyoshi Sekizuka, Kenji Sadamasu, Takashi Chiba, Mami Nagashima, Kentaro Itokawa, Rina Tanaka, Masanori Hashino, Hajime Kamiya, Motoi Suzuki, Makoto Kuroda
EPI_ISL_479966	Osaka Institute of Public Health	Pathogen Genomics Center, National Institute of Infectious Diseases	Tsuyoshi Sekizuka, Satoshi Hiroi, Saeko Morikawa, Kazushi Motomura, Kentaro Itokawa, Rina Tanaka, Masanori Hashino, Hajime Kamiya, Motoi Suzuki, Makoto Kuroda
EPI_ISL_479967, EPI_ISL_479968, EPI_ISL_479969, EPI_ISL_479970, EPI_ISL_479971, EPI_ISL_479972, EPI_ISL_479973, EPI_ISL_479974, EPI_ISL_479975, EPI_ISL_479976, EPI_ISL_479977, EPI_ISL_479978			
see above	Fukui Prefectural Institute of Public Health and Environmental Science	Pathogen Genomics Center, National Institute of Infectious Diseases	Tsuyoshi Sekizuka, Miho Toho, Kentaro Itokawa, Rina Tanaka, Masanori Hashino, Hajime Kamiya, Motoi Suzuki, Makoto Kuroda
EPI_ISL_479979, EPI_ISL_479980, EPI_ISL_479981, EPI_ISL_479982, EPI_ISL_479983, EPI_ISL_479984	Oita Prefectural Institute of Public Health and Environmental Science	Pathogen Genomics Center, National Institute of Infectious Diseases	Tsuyoshi Sekizuka, Mari Sasaki, Kentaro Itokawa, Rina Tanaka, Masanori Hashino, Hajime Kamiya, Motoi Suzuki, Makoto Kuroda
EPI_ISL_479985	Ibaraki Prefectural Institute of Public Health	Pathogen Genomics Center, National Institute of Infectious Diseases	Tsuyoshi Sekizuka, Keiko Goto, Kentaro Itokawa, Rina Tanaka, Masanori Hashino, Hajime Kamiya, Motoi Suzuki, Makoto Kuroda
EPI_ISL_479986, EPI_ISL_479987, EPI_ISL_479988, EPI_ISL_479989	Department of Infectious Diseases, Kobe Institute of Health	Pathogen Genomics Center, National Institute of Infectious Diseases	Tsuyoshi Sekizuka, Ryohei Nomoto, Kentaro Itokawa, Rina Tanaka, Masanori Hashino, Hajime Kamiya, Motoi Suzuki, Makoto Kuroda
EPI_ISL_479990	Kitakyushu City Institute of Health and Environmental Sciences	Pathogen Genomics Center, National Institute of Infectious Diseases	Tsuyoshi Sekizuka, Katsuya Obata, Asuka Kikuchi Kentaro Itokawa, Rina Tanaka, Masanori Hashino, Hajime Kamiya, Motoi Suzuki, Makoto Kuroda
EPI_ISL_479991, EPI_ISL_479992, EPI_ISL_479993, EPI_ISL_479994, EPI_ISL_479995, EPI_ISL_479996	Kumamoto City Public Health Research Institute	Pathogen Genomics Center, National Institute of Infectious Diseases	Tsuyoshi Sekizuka, Kaori Tashiro, Kentaro Itokawa, Rina Tanaka, Masanori Hashino, Hajime Kamiya, Motoi Suzuki, Makoto Kuroda
EPI_ISL_479997, EPI_ISL_479998, EPI_ISL_479999, EPI_ISL_480000, EPI_ISL_480001	Nagano Environmental Conservation Research Institute	Pathogen Genomics Center, National Institute of Infectious Diseases	Tsuyoshi Sekizuka, Naoko Shimodaira, Kentaro Itokawa, Rina Tanaka, Masanori Hashino, Hajime Kamiya, Motoi Suzuki, Makoto Kuroda
EPI_ISL_480002, EPI_ISL_480003	Nagasaki Prefectural Institute for Environmental Research and Public Health	Pathogen Genomics Center, National Institute of Infectious Diseases	Tsuyoshi Sekizuka, Fumiaki Matsumoto, Kentaro Itokawa, Rina Tanaka, Masanori Hashino, Hajime Kamiya, Motoi Suzuki, Makoto Kuroda
EPI_ISL_480004, EPI_ISL_480005, EPI_ISL_480006, EPI_ISL_480007, EPI_ISL_480008, EPI_ISL_480009, EPI_ISL_480010, EPI_ISL_480011, EPI_ISL_480012, EPI_ISL_480013, EPI_ISL_480014			
see above	Chiba Prefectural Institute of Public Health	Pathogen Genomics Center, National Institute of Infectious Diseases	Tsuyoshi Sekizuka, Masakatsu Taira, Kentaro Itokawa, Rina Tanaka, Masanori Hashino, Hajime Kamiya, Motoi Suzuki, Makoto Kuroda
EPI_ISL_480015, EPI_ISL_480016, EPI_ISL_480017, EPI_ISL_480018, EPI_ISL_480019, EPI_ISL_480020	Gunma Prefectural Institute of Public Health and Environmental Sciences	Pathogen Genomics Center, National Institute of Infectious Diseases	Tsuyoshi Sekizuka, Hiroyuki Tsukagoshi, Kentaro Itokawa, Rina Tanaka, Masanori Hashino, Hajime Kamiya, Motoi Suzuki, Makoto Kuroda
EPI_ISL_480021, EPI_ISL_480022, EPI_ISL_480023, EPI_ISL_480024, EPI_ISL_480025, EPI_ISL_480026, EPI_ISL_480027, EPI_ISL_480028, EPI_ISL_480029	Ibaraki Prefectural Institute of Public Health	Pathogen Genomics Center, National Institute of Infectious Diseases	Tsuyoshi Sekizuka, Keiko Goto, Kentaro Itokawa, Rina Tanaka, Masanori Hashino, Hajime Kamiya, Motoi Suzuki, Makoto Kuroda
EPI_ISL_480030, EPI_ISL_480031, EPI_ISL_480032, EPI_ISL_480033, EPI_ISL_480034, EPI_ISL_480035, EPI_ISL_480036, EPI_ISL_480037, EPI_ISL_480038, EPI_ISL_480039, EPI_ISL_480040, EPI_ISL_480041			
see above	Tochigi Prefectural Institute of Public Health and Environmental Science	Pathogen Genomics Center, National Institute of Infectious Diseases	Tsuyoshi Sekizuka, Ako Nakajima, Kentaro Itokawa, Rina Tanaka, Masanori Hashino, Hajime Kamiya, Motoi Suzuki, Makoto Kuroda
EPI_ISL_480042, EPI_ISL_480043, EPI_ISL_480044, EPI_ISL_480045, EPI_ISL_480046, EPI_ISL_480047, EPI_ISL_480048, EPI_ISL_480049, EPI_ISL_480050, EPI_ISL_480051, EPI_ISL_480052, EPI_ISL_480053, EPI_ISL_480054, EPI_ISL_480055, EPI_ISL_480056, EPI_ISL_480057, EPI_ISL_480058, EPI_ISL_480059,			

EPI_ISL_480060, EPI_ISL_480061, EPI_ISL_480062, EPI_ISL_480063, EPI_ISL_480064			
see above	Nagoya City Public Health Research Institute	Pathogen Genomics Center, National Institute of Infectious Diseases	Tsuyoshi Sekizuka, Takuya Miki, Shinichiro Shibata, Kentaro Itokawa, Rina Tanaka, Masanori Hashino, Hajime Kamiya, Motoi Suzuki, Makoto Kuroda
EPI_ISL_480065, EPI_ISL_480066, EPI_ISL_480067, EPI_ISL_480068, EPI_ISL_480069, EPI_ISL_480070, EPI_ISL_480071, EPI_ISL_480072	Sakai City Institute of Public Health	Pathogen Genomics Center, National Institute of Infectious Diseases	Tsuyoshi Sekizuka, Tatsuya Miyoshi, Kentaro Itokawa, Rina Tanaka, Masanori Hashino, Hajime Kamiya, Motoi Suzuki, Makoto Kuroda
EPI_ISL_480073	Tochigi Prefectural Institute of Public Health and Environmental Science	Pathogen Genomics Center, National Institute of Infectious Diseases	Tsuyoshi Sekizuka, Ako Nakajima, Kentaro Itokawa, Rina Tanaka, Masanori Hashino, Hajime Kamiya, Motoi Suzuki, Makoto Kuroda
EPI_ISL_480074, EPI_ISL_480075, EPI_ISL_480076, EPI_ISL_480077, EPI_ISL_480078, EPI_ISL_480079, EPI_ISL_480080, EPI_ISL_480081, EPI_ISL_480082	Shizuoka City Institute of Environmental Sciences and Public Health	Pathogen Genomics Center, National Institute of Infectious Diseases	Tsuyoshi Sekizuka, Takaharu Maehata,Sou Okamura,Yuji Kanazawa,Kenji Yagi, Kentaro Itokawa, Rina Tanaka, Masanori Hashino, Hajime Kamiya, Motoi Suzuki, Makoto Kuroda
EPI_ISL_480083, EPI_ISL_480084, EPI_ISL_480085, EPI_ISL_480086, EPI_ISL_480087, EPI_ISL_480088, EPI_ISL_480089	Gifu Prefectural Institute of Public Health and Environmental Sciences	Pathogen Genomics Center, National Institute of Infectious Diseases	Tsuyoshi Sekizuka, Yoshihiko Kameyama, Kentaro Itokawa, Rina Tanaka, Masanori Hashino, Hajime Kamiya, Motoi Suzuki, Makoto Kuroda
EPI_ISL_480090, EPI_ISL_480091, EPI_ISL_480092, EPI_ISL_480093, EPI_ISL_480094, EPI_ISL_480095, EPI_ISL_480096, EPI_ISL_480097, EPI_ISL_480098, EPI_ISL_480099, EPI_ISL_480100, EPI_ISL_480101, EPI_ISL_480102			
see above	Department of Infectious Diseases, Kobe Institute of Health	Pathogen Genomics Center, National Institute of Infectious Diseases	Tsuyoshi Sekizuka, Ryohei Nomoto, Kentaro Itokawa, Rina Tanaka, Masanori Hashino, Hajime Kamiya, Motoi Suzuki, Makoto Kuroda
EPI_ISL_480103, EPI_ISL_480104, EPI_ISL_480105, EPI_ISL_480106, EPI_ISL_480107, EPI_ISL_480108	Koshigaya City Public Health Center	Pathogen Genomics Center, National Institute of Infectious Diseases	Tsuyoshi Sekizuka, Yuka Furui, Aya Tamura, Kyohei Sakata, Takumi Daimon, Yoko Togawa, Yoshiko Hamada, Kentaro Itokawa, Rina Tanaka, Masanori Hashino, Hajime Kamiya, Motoi Suzuki, Makoto Kuroda
EPI_ISL_480109, EPI_ISL_480110, EPI_ISL_480111, EPI_ISL_480112, EPI_ISL_480113, EPI_ISL_480114, EPI_ISL_480115, EPI_ISL_480116, EPI_ISL_480117, EPI_ISL_480118, EPI_ISL_480119			
see above	Oita Prefectural Institute of Public Health and Environmental Science	Pathogen Genomics Center, National Institute of Infectious Diseases	Tsuyoshi Sekizuka, Mari Sasaki, Kentaro Itokawa, Rina Tanaka, Masanori Hashino, Hajime Kamiya, Motoi Suzuki, Makoto Kuroda
EPI_ISL_480120, EPI_ISL_480121, EPI_ISL_480122, EPI_ISL_480123, EPI_ISL_480124, EPI_ISL_480125, EPI_ISL_480126, EPI_ISL_480127, EPI_ISL_480128, EPI_ISL_480129, EPI_ISL_480130, EPI_ISL_480131, EPI_ISL_480132, EPI_ISL_480133, EPI_ISL_480134, EPI_ISL_480135, EPI_ISL_480136, EPI_ISL_480137, EPI_ISL_480138, EPI_ISL_480139, EPI_ISL_480140, EPI_ISL_480141, EPI_ISL_480142, EPI_ISL_480143, EPI_ISL_480144, EPI_ISL_480145, EPI_ISL_480146, EPI_ISL_480147, EPI_ISL_480148, EPI_ISL_480149, EPI_ISL_480150, EPI_ISL_480151, EPI_ISL_480152, EPI_ISL_480153, EPI_ISL_480154, EPI_ISL_480155, EPI_ISL_480156, EPI_ISL_480157, EPI_ISL_480158, EPI_ISL_480159, EPI_ISL_480160, EPI_ISL_480161, EPI_ISL_480162, EPI_ISL_480163, EPI_ISL_480164, EPI_ISL_480165, EPI_ISL_480166, EPI_ISL_480167, EPI_ISL_480168			
see above	Fukui Prefectural Institute of Public Health and Environmental Science	Pathogen Genomics Center, National Institute of Infectious Diseases	Tsuyoshi Sekizuka, Miho Toho, Kentaro Itokawa, Rina Tanaka, Masanori Hashino, Hajime Kamiya, Motoi Suzuki, Makoto Kuroda
EPI_ISL_480169, EPI_ISL_480170, EPI_ISL_480171, EPI_ISL_480172, EPI_ISL_480173, EPI_ISL_480174, EPI_ISL_480175, EPI_ISL_480176, EPI_ISL_480177	Gunma Prefectural Institute of Public Health and Environmental Sciences	Pathogen Genomics Center, National Institute of Infectious Diseases	Tsuyoshi Sekizuka, Hiroyuki Tsukagoshi, Kentaro Itokawa, Rina Tanaka, Masanori Hashino, Hajime Kamiya, Motoi Suzuki, Makoto Kuroda
EPI_ISL_480178, EPI_ISL_480179	Hiroshima City Institute of Public Health	Pathogen Genomics Center, National Institute of Infectious Diseases	Tsuyoshi Sekizuka, Kota Noritsune, Kentaro Itokawa, Rina Tanaka, Masanori Hashino, Hajime Kamiya, Motoi Suzuki, Makoto Kuroda
EPI_ISL_480180, EPI_ISL_480181, EPI_ISL_480182, EPI_ISL_480183, EPI_ISL_480184, EPI_ISL_480185, EPI_ISL_480186, EPI_ISL_480187, EPI_ISL_480188, EPI_ISL_480189	Ibaraki Prefectural Institute of Public Health	Pathogen Genomics Center, National Institute of Infectious Diseases	Tsuyoshi Sekizuka, Keiko Goto, Kentaro Itokawa, Rina Tanaka, Masanori Hashino, Hajime Kamiya, Motoi Suzuki, Makoto Kuroda
EPI_ISL_480190, EPI_ISL_480191, EPI_ISL_480192, EPI_ISL_480193, EPI_ISL_480194, EPI_ISL_480195	Ota Health Center Welfare Section	Pathogen Genomics Center, National Institute of Infectious Diseases	Tsuyoshi Sekizuka, Chika Takahashi, Kentaro Itokawa, Rina Tanaka, Masanori Hashino, Hajime Kamiya, Motoi Suzuki, Makoto Kuroda
EPI_ISL_480196, EPI_ISL_480197, EPI_ISL_480198, EPI_ISL_480199, EPI_ISL_480200, EPI_ISL_480201, EPI_ISL_480202, EPI_ISL_480203	Toyama Institute of Health	Pathogen Genomics Center, National Institute of Infectious Diseases	Tsuyoshi Sekizuka, Masae Itamochi, Kazunori Oishi, Kentaro Itokawa, Rina Tanaka, Masanori Hashino, Hajime Kamiya, Motoi Suzuki, Makoto Kuroda
EPI_ISL_480204	Akita City Public Health Center	Pathogen Genomics Center, National Institute of Infectious Diseases	Tsuyoshi Sekizuka, Koichi Ito, Kentaro Itokawa, Rina Tanaka, Masanori Hashino, Hajime Kamiya, Motoi Suzuki, Makoto Kuroda
EPI_ISL_480205, EPI_ISL_480206, EPI_ISL_480207	Department of Infectious Diseases, Kobe Institute of Health	Pathogen Genomics Center, National Institute of Infectious Diseases	Tsuyoshi Sekizuka, Ryohei Nomoto, Kentaro Itokawa, Rina Tanaka, Masanori Hashino, Hajime Kamiya, Motoi Suzuki, Makoto Kuroda
EPI_ISL_480208	Pathogen Genomics Center, National Institute of Infectious Diseases	Pathogen Genomics Center, National Institute of Infectious Diseases	Tsuyoshi Sekizuka, Ryohei Nomoto, Kentaro Itokawa, Rina Tanaka, Masanori Hashino, Hajime Kamiya, Motoi Suzuki, Makoto Kuroda
EPI_ISL_480209, EPI_ISL_480210, EPI_ISL_480211, EPI_ISL_480212, EPI_ISL_480213, EPI_ISL_480214, EPI_ISL_480215, EPI_ISL_480216, EPI_ISL_480217, EPI_ISL_480218, EPI_ISL_480219, EPI_ISL_480220			
see above	Department of Infectious Diseases, Kobe Institute of Health	Pathogen Genomics Center, National Institute of Infectious Diseases	Tsuyoshi Sekizuka, Ryohei Nomoto, Kentaro Itokawa, Rina Tanaka, Masanori Hashino, Hajime Kamiya, Motoi Suzuki, Makoto Kuroda
EPI_ISL_480221, EPI_ISL_480222, EPI_ISL_480223	Koshigaya City Public Health Center	Pathogen Genomics Center, National Institute of Infectious Diseases	Tsuyoshi Sekizuka, Yuka Furui, Aya Tamura, Kyohei Sakata, Takumi Daimon, Yoko Togawa, Yoshiko Hamada, Kentaro Itokawa, Rina Tanaka, Masanori Hashino, Hajime Kamiya, Motoi Suzuki, Makoto Kuroda
EPI_ISL_480225	Fukui Prefectural Institute of Public Health and Environmental Science	Pathogen Genomics Center, National Institute of Infectious Diseases	Tsuyoshi Sekizuka, Miho Toho, Kentaro Itokawa, Rina Tanaka, Masanori Hashino, Hajime Kamiya, Motoi Suzuki, Makoto Kuroda
EPI_ISL_480226	Niigata Prefectural Institute of Public Health and Environmental Sciences	Pathogen Genomics Center, National Institute of Infectious Diseases	Tsuyoshi Sekizuka, Reiko Arai, Kentaro Itokawa, Rina Tanaka, Masanori Hashino, Hajime Kamiya, Motoi Suzuki, Makoto Kuroda
EPI_ISL_480227	Tokyo Metropolitan Institute of Public Health	Pathogen Genomics Center, National Institute of Infectious Diseases	Tsuyoshi Sekizuka, Kenji Sadamasu, Takashi Chiba, Mami Nagashima, Kentaro Itokawa, Rina Tanaka, Masanori Hashino, Hajime Kamiya, Motoi Suzuki, Makoto Kuroda
EPI_ISL_480228, EPI_ISL_480229, EPI_ISL_480230, EPI_ISL_480231, EPI_ISL_480232, EPI_ISL_480233, EPI_ISL_480234, EPI_ISL_480235, EPI_ISL_480236, EPI_ISL_480237, EPI_ISL_480238, EPI_ISL_480239, EPI_ISL_480240, EPI_ISL_480241, EPI_ISL_480242, EPI_ISL_480243, EPI_ISL_480244, EPI_ISL_480245, EPI_ISL_480246, EPI_ISL_480247, EPI_ISL_480248, EPI_ISL_480249, EPI_ISL_480250, EPI_ISL_480252, EPI_ISL_480253, EPI_ISL_480254, EPI_ISL_480255, EPI_ISL_480256, EPI_ISL_480257, EPI_ISL_480258, EPI_ISL_480259, EPI_ISL_480260, EPI_ISL_480261, EPI_ISL_480262, EPI_ISL_480263, EPI_ISL_480264, EPI_ISL_480265, EPI_ISL_480266, EPI_ISL_480267, EPI_ISL_480268, EPI_ISL_480269, EPI_ISL_480270, EPI_ISL_480271, EPI_ISL_480272, EPI_ISL_480273, EPI_ISL_480274, EPI_ISL_480277, EPI_ISL_480278, EPI_ISL_480281, EPI_ISL_480282, EPI_ISL_480283, EPI_ISL_480284, EPI_ISL_480285, EPI_ISL_480286, EPI_ISL_480289, EPI_ISL_480290, EPI_ISL_480291, EPI_ISL_480292			
see above	Genomic Laboratory (GLAB) (Conjoint lab of Health	Genomic Laboratory (GLAB), Istanbul Technical University	Ilker Karacan, Tugba Kizilboga Akgun, Bugra Agaoglu, Gizem Alkurt, Jale Yildiz, Betsi Köse, Elifnaz Çelik, Arzu Irvem, Yasemin Kendir Demirkol, Ozlem

	Directorate of Istanbul and Istanbul Technical University)		Akgun Dogan, Mehtap Aydn, Levent Doganay, Gizem Dinler Doganay
EPI_ISL_480310	National Reference Laboratory "Influenza and acute respiratory diseases"	NRL-HIV	Ivan Ivanov, Ivailo Alexiev, Ivva Philipova
EPI_ISL_480315, EPI_ISL_480316, EPI_ISL_480317, EPI_ISL_480318, EPI_ISL_480319, EPI_ISL_480320	Hospital Clínica Bíblica	Charité Virology-University of Costa Rica	Andres Moreira-Soto, Eugenia Corrales-Aguilar, Ignacio Postigo-Hidalgo, Karla Sofía Gutiérrez, Jan Felix Drexler
EPI_ISL_480321	Laboratorio Clínico San José	Charité Virology-University of Costa Rica	Andres Moreira-Soto, Eugenia Corrales-Aguilar, Ignacio Postigo-Hidalgo, Hugo Núñez Navas, Jan Felix Drexler
EPI_ISL_480322, EPI_ISL_480323, EPI_ISL_480324, EPI_ISL_480325, EPI_ISL_480326, EPI_ISL_480327	Hospital Nacional de Niños	Charité Virology-University of Costa Rica	Andres Moreira-Soto, Eugenia Corrales-Aguilar, Ignacio Postigo-Hidalgo, Cristian Pérez Corrales, Andrei Montero Bonilla, Jan Felix Drexler
EPI_ISL_480328	Laboratorio LABIN	Charité Virology-University of Costa Rica	Andres Moreira-Soto, Eugenia Corrales-Aguilar, Ignacio Postigo-Hidalgo, Ignacio Soto Pacheco, Jan Felix Drexler
EPI_ISL_480554, EPI_ISL_480556, EPI_ISL_480782, EPI_ISL_480783, EPI_ISL_480786, EPI_ISL_480787, EPI_ISL_480788, EPI_ISL_480789, EPI_ISL_481220, EPI_ISL_481234, EPI_ISL_481235, EPI_ISL_481236, EPI_ISL_481237, EPI_ISL_481238, EPI_ISL_481239, EPI_ISL_481240, EPI_ISL_481243			
see above	Institut Pasteur Dakar	Institut Pasteur de Dakar	Ndongo Dia, Moussa Moise Diagne, Mamadou Diop, Marie Henriette Dior Ndione, Mamadou Malado Jallow, Safietou Sanke, Ousmane Faye, Amadou Alpha Sall.
EPI_ISL_482575, EPI_ISL_482576, EPI_ISL_482577, EPI_ISL_482578, EPI_ISL_482579, EPI_ISL_482580, EPI_ISL_482581, EPI_ISL_482582, EPI_ISL_482583, EPI_ISL_482584, EPI_ISL_482585, EPI_ISL_482586			
see above	Hangzhou Center for Diseases Control and Prevention	Hangzhou Center for Diseases Control and Prevention	Jun Li, Haoqiu Wang, Lingfeng Mao, Hua Yu, Xinfen Yu, Zhou Sun, Xin Qian, Shuchang Chen, Junfang Chen, Xuchu Wang
EPI_ISL_482672, EPI_ISL_482673, EPI_ISL_482674, EPI_ISL_482675, EPI_ISL_482676, EPI_ISL_482677, EPI_ISL_482678, EPI_ISL_482679, EPI_ISL_482680, EPI_ISL_482681, EPI_ISL_482682, EPI_ISL_482683, EPI_ISL_482684, EPI_ISL_482685, EPI_ISL_482686, EPI_ISL_482687, EPI_ISL_482688, EPI_ISL_482689, EPI_ISL_482690, EPI_ISL_482691, EPI_ISL_482692, EPI_ISL_482693, EPI_ISL_482694, EPI_ISL_482695, EPI_ISL_482696, EPI_ISL_482697, EPI_ISL_482698, EPI_ISL_482699			
see above	Singapore General Hospital	Department of Microbiology	Nurdyana Abdul Rahman, Kun Lee Lim, Chenhao Li, Kian Sing Chan, Lynette Oon, Kern Rei Chng, Niranjan Nagarajan, Karrie Ko
EPI_ISL_482702, EPI_ISL_482703, EPI_ISL_482704, EPI_ISL_482705, EPI_ISL_482706, EPI_ISL_482707, EPI_ISL_482708, EPI_ISL_482709	Molecular Diagnostics Services (MDS)	KRISP, KZN Research Innovation and Sequencing Platform	Giandhari J, Pillay S, Lessells R, Chimukangara B, Mdlalose K, York D, Khan S, Tegally H, Wilkinson E, de Oliveira T
EPI_ISL_482710, EPI_ISL_482711, EPI_ISL_482712, EPI_ISL_482713	NHLS-IALCH	KRISP, KZN Research Innovation and Sequencing Platform	Giandhari J, Pillay S, Lessells R, Chimukangara B, Mdlalose K, York D, Khan S, Tegally H, Wilkinson E, de Oliveira T
EPI_ISL_482714, EPI_ISL_482715, EPI_ISL_482716, EPI_ISL_482717, EPI_ISL_482718, EPI_ISL_482719, EPI_ISL_482720, EPI_ISL_482721, EPI_ISL_482722, EPI_ISL_482723	Molecular Diagnostics Services (MDS)	KRISP, KZN Research Innovation and Sequencing Platform	Giandhari J, Pillay S, Lessells R, Chimukangara B, Mdlalose K, York D, Khan S, Tegally H, Wilkinson E, de Oliveira T
EPI_ISL_482724, EPI_ISL_482725, EPI_ISL_482726, EPI_ISL_482727, EPI_ISL_482728, EPI_ISL_482729, EPI_ISL_482730, EPI_ISL_482731	NHLS-IALCH	KRISP, KZN Research Innovation and Sequencing Platform	Giandhari J, Pillay S, Lessells R, Chimukangara B, Mdlalose K, York D, Khan S, Tegally H, Wilkinson E, de Oliveira T
EPI_ISL_482759, EPI_ISL_482760, EPI_ISL_482761, EPI_ISL_482762, EPI_ISL_482763, EPI_ISL_482764, EPI_ISL_482765, EPI_ISL_482766, EPI_ISL_482767, EPI_ISL_482768, EPI_ISL_482769, EPI_ISL_482770, EPI_ISL_482771, EPI_ISL_482772, EPI_ISL_482773, EPI_ISL_482774, EPI_ISL_482775			
see above	Medical Ain Shams Research Institute (MASRI), Ain Shams University	Medical Ain Shams Research Institute (MASRI), Ain Shams University	Hesham Elghazaly, Sara Hassan Agwa, Ahmad Moustafa, Hala Hafez, Sara Elnakeep, Shaimaa Moustafa, Aya Mohamed, Reham Mamdouh, Ghada Ismael, Ashraf Omar, Osama Mansour, Mahmoud Elmeitini
EPI_ISL_482820	Centre de Recerca en Sanitat Animal (IRTA-CReSA)	IrsiCaixa AIDS Research Lab	J. Segalés, M. Puig, J. Rodon, C. Avila-Nieto, J. Carrillo, G. Cantero, M.T. Terrón, S. Cruz, M. Parera ,M. Noguera-Julián, N. Izquierdo-Useros, V. Guallar, E. Vidal, A. Valencia, I. Blanco, J. Blanco, B. Clotet, J. Vergara-Alert
EPI_ISL_482848, EPI_ISL_482849, EPI_ISL_482850	NHLS-IALCH	KRISP, KZN Research Innovation and Sequencing Platform	Giandhari J, Pillay S, Lessells R, Chimukangara B, Mdlalose K, York D, Khan S, Tegally H, Wilkinson E, de Oliveira T
EPI_ISL_482851, EPI_ISL_482852, EPI_ISL_482853, EPI_ISL_482854, EPI_ISL_482855, EPI_ISL_482856, EPI_ISL_482857, EPI_ISL_482858, EPI_ISL_482859, EPI_ISL_482860, EPI_ISL_482861, EPI_ISL_482862, EPI_ISL_482863, EPI_ISL_482864, EPI_ISL_482865, EPI_ISL_482866, EPI_ISL_482867, EPI_ISL_482868, EPI_ISL_482869, EPI_ISL_482870, EPI_ISL_482871, EPI_ISL_482872			
see above	Molecular Diagnostics Services (MDS)	KRISP, KZN Research Innovation and Sequencing Platform	Giandhari J, Pillay S, Lessells R, Chimukangara B, Mdlalose K, York D, Khan S, Tegally H, Wilkinson E, de Oliveira T
EPI_ISL_482874, EPI_ISL_482875, EPI_ISL_482876, EPI_ISL_482877, EPI_ISL_482878	Institut Pasteur Dakar	Institut Pasteur de Dakar	Ndongo Dia, Moussa Moise Diagne, Mamadou Diop, Marie Henriette Dior Ndione, Mamadou malado Jallow, Safietou Sankhe, Ousmane Faye, Amadou Alpha Sall.
EPI_ISL_483035, EPI_ISL_483036, EPI_ISL_483037, EPI_ISL_483038	Medical Ain Shams Research Institute (MASRI), Ain Shams University	Medical Ain Shams Research Institute (MASRI), Ain Shams University	Hesham Elghazaly, Sara Hassan Agwa, Ahmad Moustafa, Hala Hafez, Sara Elnakeep, Shaimaa Moustafa, Aya Mohamed, Reham Mamdouh, Ghada Ismael, Ashraf Omar, Osama Mansour, Mahmoud Elmeitini
EPI_ISL_483059	Hospital Universitari Germans Trias i Pujol	IrsiCaixa AIDS Research Lab	J. Segalés, M. Puig, J. Rodon, C. Avila-Nieto, J. Carrillo, G. Cantero, M.T. Terrón, S. Cruz, M. Parera ,M. Noguera-Julián, N. Izquierdo-Useros, V. Guallar, E. Vidal, A. Valencia, I. Blanco, J. Blanco, B. Clotet, J. Vergara-Alert
EPI_ISL_483566	Clinical Microbiology Laboratory- Basurto University Hospital	Biocruces-Bizkaia	Mikel J. Urrutikoetxea-Gutierrez, Ana Belén Belén de la Hoz, Matxalen Vidal-García, Mº Carmen Nieto Toboso, Estibaliz Ugalde-Zarraga, José Luis Díaz de Tuesta del Arco
EPI_ISL_483570	Clinical Microbiology Laboratory- Basurto University Hospita	Biocruces-Bizkaia	Mikel J. Urrutikoetxea-Gutierrez, Ana Belén Belén de la Hoz, Matxalen Vidal-García, Mº Carmen Nieto Toboso, Estibaliz Ugalde-Zarraga, José Luis Díaz de Tuesta del Arco
EPI_ISL_483571, EPI_ISL_483572, EPI_ISL_483573	Clinical Microbiology Laboratory- Basurto University Hospital	Biocruces-Bizkaia	Mikel J. Urrutikoetxea-Gutierrez, Ana Belén Belén de la Hoz, Matxalen Vidal-García, Mº Carmen Nieto Toboso, Estibaliz Ugalde-Zarraga, José Luis Díaz de Tuesta del Arco
EPI_ISL_483637	National Laboratory of Virology, Szentágothai Research Centre	National Laboratory of Virology, Szentágothai Research Centre	Endre Gábor Tóth, Balázs Somogyi, Ferenc Jakab, Gábor Kemenesi
EPI_ISL_483820	GMERS Medical College and Hospital, Gandhinagar	Gujarat Biotechnology Research Centre	Komal Patel, Labdhi Pandya, Afzal Ansari, Nikha Trivedi, Seema Bhatt, Gaurishankar Shrimali, Bhavesh Modi, Bharti Rajani, Apurvasinh Puvar, Janvi Raval, Zarna Patel, Monika Gandhi, Pinal Trivedi, Maharshi Pandya, Nidhi Patel, Nitin Savaliya, Raghawendra Kumar, Dinesh Kumar, Zuber Saiyed, R D Dixit, A M Kadri, Harsh Bakshi, Chaitanya Joshi, Madhvi Joshi
EPI_ISL_483821	Government Medical College, Vadodara	Gujarat Biotechnology Research Centre	Labdhi Pandya, Afzal Ansari, Nikha Trivedi, Meenakshi Shah, Neena Doshi, Varsha Godbole, Apurvasinh Puvar, Janvi Raval, Zarna Patel, Monika Gandhi, Pinal Trivedi, Maharshi Pandya, Nidhi Patel, Nitin Savaliya, Raghawendra Kumar, Dinesh Kumar, Zuber Saiyed, Komal Patel, R D Dixit, A M Kadri, Harsh Bakshi, Chaitanya Joshi, Madhvi Joshi
EPI_ISL_483822	Government Medical College, Vadodara	Gujarat Biotechnology Research Centre	Afzal Ansari, Nikha Trivedi, Meenakshi Shah, Neena Doshi, Varsha Godbole, Apurvasinh Puvar, Janvi Raval, Zarna Patel, Monika Gandhi, Pinal Trivedi, Maharshi Pandya, Nidhi Patel, Nitin Savaliya, Raghawendra Kumar, Dinesh Kumar, Zuber Saiyed, Komal Patel, Labdhi Pandya, R D Dixit, A M Kadri, Harsh Bakshi, Chaitanya Joshi, Madhvi Joshi
EPI_ISL_483823	GMERS Medical College Himmatnagar	Gujarat Biotechnology Research Centre	Nikha Trivedi, Himanshu Khatri, Mayur Gandhi, Apurvasinh Puvar, Janvi Raval, Zarna Patel, Monika Gandhi, Pinal Trivedi, Maharshi Pandya, Nidhi Patel, Nitin Savaliya, Raghawendra Kumar, Dinesh Kumar, Zuber Saiyed, Komal Patel, Labdhi Pandya, Afzal Ansari, R D Dixit, A M Kadri, Harsh Bakshi, Chaitanya Joshi, Madhvi Joshi

EPI_ISL_483824	GMERS Medical College Himmatnagar	Gujarat Biotechnology Research Centre	Himanshu Khatri, Mayur Gandhi, Apurvasinh Puvar, Janvi Raval, Zarna Patel, Monika Gandhi, Pinal Trivedi, Maharshi Pandya, Nidhi Patel, Nitin Savaliya, Raghawendra Kumar, Dinesh Kumar, Zuber Saiyed, Komal Patel, Labdhi Pandya, Afzal Ansari, Nikha Trivedi, R D Dixit, A M Kadri, Harsh Bakshi, Chaitanya Joshi, Madhvi Joshi
EPI_ISL_483825	GMERS Medical College Himmatnagar	Gujarat Biotechnology Research Centre	Mayur Gandhi, Apurvasinh Puvar, Janvi Raval, Zarna Patel, Monika Gandhi, Pinal Trivedi, Maharshi Pandya, Nidhi Patel, Nitin Savaliya, Raghawendra Kumar, Dinesh Kumar, Zuber Saiyed, Komal Patel, Labdhi Pandya, Afzal Ansari, Nikha Trivedi, Himanshu Khatri, R D Dixit, A M Kadri, Harsh Bakshi, Chaitanya Joshi, Madhvi Joshi
EPI_ISL_483826	GMERS Medical College Himmatnagar	Gujarat Biotechnology Research Centre	Apurvasinh Puvar, Janvi Raval, Zarna Patel, Monika Gandhi, Pinal Trivedi, Maharshi Pandya, Nidhi Patel, Nitin Savaliya, Raghawendra Kumar, Dinesh Kumar, Zuber Saiyed, Komal Patel, Labdhi Pandya, Afzal Ansari, Nikha Trivedi, Himanshu Khatri, Mayur Gandhi, R D Dixit, A M Kadri, Harsh Bakshi, Chaitanya Joshi, Madhvi Joshi
EPI_ISL_483827	GMERS Medical College Himmatnagar	Gujarat Biotechnology Research Centre	Janvi Raval, Zarna Patel, Monika Gandhi, Pinal Trivedi, Maharshi Pandya, Nidhi Patel, Nitin Savaliya, Raghawendra Kumar, Dinesh Kumar, Zuber Saiyed, Komal Patel, Labdhi Pandya, Afzal Ansari, Nikha Trivedi, Himanshu Khatri, Mayur Gandhi, Apurvasinh Puvar, R D Dixit, A M Kadri, Harsh Bakshi, Chaitanya Joshi, Madhvi Joshi
EPI_ISL_483828	GMERS Medical College Himmatnagar	Gujarat Biotechnology Research Centre	Zarna Patel, Monika Gandhi, Pinal Trivedi, Maharshi Pandya, Nidhi Patel, Nitin Savaliya, Raghawendra Kumar, Dinesh Kumar, Zuber Saiyed, Komal Patel, Labdhi Pandya, Afzal Ansari, Nikha Trivedi, Himanshu Khatri, Mayur Gandhi, Apurvasinh Puvar, Janvi Raval, R D Dixit, A M Kadri, Harsh Bakshi, Chaitanya Joshi, Madhvi Joshi
EPI_ISL_483829	Pandit Deendayal Upadhyay Government Medical College, Rajkot	Gujarat Biotechnology Research Centre	Monika Gandhi, Pinal Trivedi, Maharshi Pandya, Nidhi Patel, Nitin Savaliya, Raghawendra Kumar, Dinesh Kumar, Zuber Saiyed, Komal Patel, Labdhi Pandya, Afzal Ansari, Nikha Trivedi, Gauravi Dhruv, Arti Trivedi, Apurvasinh Puvar, Janvi Raval, Zarna Patel, R D Dixit, A M Kadri, Harsh Bakshi, Chaitanya Joshi, Madhvi Joshi
EPI_ISL_483830	Pandit Deendayal Upadhyay Government Medical College, Rajkot	Gujarat Biotechnology Research Centre	Pinal Trivedi, Maharshi Pandya, Nidhi Patel, Nitin Savaliya, Raghawendra Kumar, Dinesh Kumar, Zuber Saiyed, Komal Patel, Labdhi Pandya, Afzal Ansari, Nikha Trivedi, Gauravi Dhruv, Arti Trivedi, Apurvasinh Puvar, Janvi Raval, Zarna Patel, Monika Gandhi, R D Dixit, A M Kadri, Harsh Bakshi, Chaitanya Joshi, Madhvi Joshi
EPI_ISL_483831	Pandit Deendayal Upadhyay Government Medical College, Rajkot	Gujarat Biotechnology Research Centre	Maharshi Pandya, Nidhi Patel, Nitin Savaliya, Raghawendra Kumar, Dinesh Kumar, Zuber Saiyed, Komal Patel, Labdhi Pandya, Afzal Ansari, Nikha Trivedi, Gauravi Dhruv, Arti Trivedi, Apurvasinh Puvar, Janvi Raval, Zarna Patel, Monika Gandhi, Pinal Trivedi, R D Dixit, A M Kadri, Harsh Bakshi, Chaitanya Joshi, Madhvi Joshi
EPI_ISL_483832	Pandit Deendayal Upadhyay Government Medical College, Rajkot	Gujarat Biotechnology Research Centre	Nidhi Patel, Nitin Savaliya, Raghawendra Kumar, Dinesh Kumar, Zuber Saiyed, Komal Patel, Labdhi Pandya, Afzal Ansari, Nikha Trivedi, Gauravi Dhruv, Arti Trivedi, Apurvasinh Puvar, Janvi Raval, Zarna Patel, Monika Gandhi, Pinal Trivedi, Maharshi Pandya, R D Dixit, A M Kadri, Harsh Bakshi, Chaitanya Joshi, Madhvi Joshi
EPI_ISL_483833	Pandit Deendayal Upadhyay Government Medical College, Rajkot	Gujarat Biotechnology Research Centre	Nitin Savaliya, Raghawendra Kumar, Dinesh Kumar, Zuber Saiyed, Komal Patel, Labdhi Pandya, Afzal Ansari, Nikha Trivedi, Gauravi Dhruv, Arti Trivedi, Apurvasinh Puvar, Janvi Raval, Zarna Patel, Monika Gandhi, Pinal Trivedi, Maharshi Pandya, Nidhi Patel, R D Dixit, A M Kadri, Harsh Bakshi, Chaitanya Joshi, Madhvi Joshi
EPI_ISL_483834	Pandit Deendayal Upadhyay Government Medical College, Rajkot	Gujarat Biotechnology Research Centre	Raghawendra Kumar, Dinesh Kumar, Zuber Saiyed, Komal Patel, Labdhi Pandya, Afzal Ansari, Nikha Trivedi, Gauravi Dhruv, Arti Trivedi, Apurvasinh Puvar, Janvi Raval, Zarna Patel, Monika Gandhi, Pinal Trivedi, Maharshi Pandya, Nidhi Patel, Nitin Savaliya, R D Dixit, A M Kadri, Harsh Bakshi, Chaitanya Joshi, Madhvi Joshi
EPI_ISL_483835	Pandit Deendayal Upadhyay Government Medical College, Rajkot	Gujarat Biotechnology Research Centre	Dinesh Kumar, Zuber Saiyed, Komal Patel, Labdhi Pandya, Afzal Ansari, Nikha Trivedi, Gauravi Dhruv, Arti Trivedi, Apurvasinh Puvar, Janvi Raval, Zarna Patel, Monika Gandhi, Pinal Trivedi, Maharshi Pandya, Nidhi Patel, Nitin Savaliya, Raghawendra Kumar, R D Dixit, A M Kadri, Harsh Bakshi, Chaitanya Joshi, Madhvi Joshi
EPI_ISL_483836	Pandit Deendayal Upadhyay Government Medical College, Rajkot	Gujarat Biotechnology Research Centre	Zuber Saiyed, Komal Patel, Labdhi Pandya, Afzal Ansari, Nikha Trivedi, Gauravi Dhruv, Arti Trivedi, Apurvasinh Puvar, Janvi Raval, Zarna Patel, Monika Gandhi, Pinal Trivedi, Maharshi Pandya, Nidhi Patel, Nitin Savaliya, Raghawendra Kumar, Dinesh Kumar, R D Dixit, A M Kadri, Harsh Bakshi, Chaitanya Joshi, Madhvi Joshi
EPI_ISL_483837	Pandit Deendayal Upadhyay Government Medical College, Rajkot	Gujarat Biotechnology Research Centre	Komal Patel, Labdhi Pandya, Afzal Ansari, Nikha Trivedi, Gauravi Dhruv, Arti Trivedi, Apurvasinh Puvar, Janvi Raval, Zarna Patel, Monika Gandhi, Pinal Trivedi, Maharshi Pandya, Nidhi Patel, Nitin Savaliya, Raghawendra Kumar, Dinesh Kumar, Zuber Saiyed, R D Dixit, A M Kadri, Harsh Bakshi, Chaitanya Joshi, Madhvi Joshi
EPI_ISL_483838	Pandit Deendayal Upadhyay Government Medical College, Rajkot	Gujarat Biotechnology Research Centre	Labdhi Pandya, Afzal Ansari, Nikha Trivedi, Gauravi Dhruv, Arti Trivedi, Apurvasinh Puvar, Janvi Raval, Zarna Patel, Monika Gandhi, Pinal Trivedi, Maharshi Pandya, Nidhi Patel, Nitin Savaliya, Raghawendra Kumar, Dinesh Kumar, Zuber Saiyed, Komal Patel, R D Dixit, A M Kadri, Harsh Bakshi, Chaitanya Joshi, Madhvi Joshi
EPI_ISL_483839	Pandit Deendayal Upadhyay Government Medical College, Rajkot	Gujarat Biotechnology Research Centre	Afzal Ansari, Nikha Trivedi, Gauravi Dhruv, Arti Trivedi, Apurvasinh Puvar, Janvi Raval, Zarna Patel, Monika Gandhi, Pinal Trivedi, Maharshi Pandya, Nidhi Patel, Nitin Savaliya, Raghawendra Kumar, Dinesh Kumar, Zuber Saiyed, Komal Patel, Labdhi Pandya, R D Dixit, A M Kadri, Harsh Bakshi, Chaitanya Joshi, Madhvi Joshi
EPI_ISL_483840	Pandit Deendayal Upadhyay Government Medical College, Rajkot	Gujarat Biotechnology Research Centre	Nikha Trivedi, Gauravi Dhruv, Arti Trivedi, Apurvasinh Puvar, Janvi Raval, Zarna Patel, Monika Gandhi, Pinal Trivedi, Maharshi Pandya, Nidhi Patel, Nitin Savaliya, Raghawendra Kumar, Dinesh Kumar, Zuber Saiyed, Komal Patel, Labdhi Pandya, Afzal Ansari, R D Dixit, A M Kadri, Harsh Bakshi, Chaitanya Joshi, Madhvi Joshi
EPI_ISL_483841	Pandit Deendayal Upadhyay Government Medical College, Rajkot	Gujarat Biotechnology Research Centre	Gauravi Dhruv, Arti Trivedi, Apurvasinh Puvar, Janvi Raval, Zarna Patel, Monika Gandhi, Pinal Trivedi, Maharshi Pandya, Nidhi Patel, Nitin Savaliya, Raghawendra Kumar, Dinesh Kumar, Zuber Saiyed, Komal Patel, Labdhi Pandya, Afzal Ansari, Nikha Trivedi, R D Dixit, A M Kadri, Harsh Bakshi, Chaitanya Joshi, Madhvi Joshi
EPI_ISL_483842	Pandit Deendayal Upadhyay Government Medical College, Rajkot	Gujarat Biotechnology Research Centre	Arti Trivedi, Apurvasinh Puvar, Janvi Raval, Zarna Patel, Monika Gandhi, Pinal Trivedi, Maharshi Pandya, Nidhi Patel, Nitin Savaliya, Raghawendra Kumar, Dinesh Kumar, Zuber Saiyed, Komal Patel, Labdhi Pandya, Afzal Ansari, Nikha Trivedi, Gauravi Dhruv, R D Dixit, A M Kadri, Harsh Bakshi, Chaitanya Joshi, Madhvi Joshi
EPI_ISL_483843	Pandit Deendayal Upadhyay Government Medical College, Rajkot	Gujarat Biotechnology Research Centre	Apurvasinh Puvar, Janvi Raval, Zarna Patel, Monika Gandhi, Pinal Trivedi, Maharshi Pandya, Nidhi Patel, Nitin Savaliya, Raghawendra Kumar, Dinesh Kumar, Zuber Saiyed, Komal Patel, Labdhi Pandya, Afzal Ansari, Nikha Trivedi, Gauravi Dhruv, Arti Trivedi, R D Dixit, A M Kadri, Harsh Bakshi, Chaitanya Joshi, Madhvi Joshi
EPI_ISL_483844	Pandit Deendayal Upadhyay Government Medical College, Rajkot	Gujarat Biotechnology Research Centre	Janvi Raval, Zarna Patel, Monika Gandhi, Pinal Trivedi, Maharshi Pandya, Nidhi Patel, Nitin Savaliya, Raghawendra Kumar, Dinesh Kumar, Zuber Saiyed, Komal Patel, Labdhi Pandya, Afzal Ansari, Nikha Trivedi, Gauravi Dhruv, Arti Trivedi, Apurvasinh Puvar, R D Dixit, A M Kadri, Harsh Bakshi, Chaitanya Joshi, Madhvi Joshi
EPI_ISL_483845	Pandit Deendayal Upadhyay Government Medical College, Rajkot	Gujarat Biotechnology Research Centre	Zarna Patel, Monika Gandhi, Pinal Trivedi, Maharshi Pandya, Nidhi Patel, Nitin Savaliya, Raghawendra Kumar, Dinesh Kumar, Zuber Saiyed, Komal Patel, Labdhi Pandya, Afzal Ansari, Nikha Trivedi, Gauravi Dhruv, Arti Trivedi, Apurvasinh Puvar, Janvi Raval, R D Dixit, A M Kadri, Harsh Bakshi, Chaitanya Joshi, Madhvi Joshi
EPI_ISL_483846	Pandit Deendayal Upadhyay Government Medical College, Rajkot	Gujarat Biotechnology Research Centre	Monika Gandhi, Pinal Trivedi, Maharshi Pandya, Nidhi Patel, Nitin Savaliya, Raghawendra Kumar, Dinesh Kumar, Zuber Saiyed, Komal Patel, Labdhi Pandya, Afzal Ansari, Nikha Trivedi, Gauravi Dhruv, Arti Trivedi, Apurvasinh Puvar, Janvi Raval, Zarna Patel, R D Dixit, A M Kadri, Harsh Bakshi, Chaitanya Joshi, Madhvi Joshi
EPI_ISL_483847	Pandit Deendayal Upadhyay Government Medical College, Rajkot	Gujarat Biotechnology Research Centre	Pinal Trivedi, Maharshi Pandya, Nidhi Patel, Nitin Savaliya, Raghawendra Kumar, Dinesh Kumar, Zuber Saiyed, Komal Patel, Labdhi Pandya, Afzal Ansari, Nikha Trivedi, Gauravi Dhruv, Arti Trivedi, Apurvasinh Puvar, Janvi Raval, Zarna Patel, Monika Gandhi, R D Dixit, A M Kadri, Harsh Bakshi, Chaitanya Joshi, Madhvi Joshi
EPI_ISL_483848	Pandit Deendayal Upadhyay Government Medical College, Rajkot	Gujarat Biotechnology Research Centre	Maharshi Pandya, Nidhi Patel, Nitin Savaliya, Raghawendra Kumar, Dinesh Kumar, Zuber Saiyed, Komal Patel, Labdhi Pandya, Afzal Ansari, Nikha Trivedi, Gauravi Dhruv, Arti Trivedi, Apurvasinh Puvar, Janvi Raval, Zarna Patel, Monika Gandhi, Pinal Trivedi, R D Dixit, A M Kadri, Harsh Bakshi, Chaitanya Joshi, Madhvi Joshi

EPI_ISL_483849	Pandit Deendayal Upadhyay Government Medical College, Rajkot	Gujarat Biotechnology Research Centre	Nidhi Patel, Nitin Savaliya, Raghawendra Kumar, Dinesh Kumar, Zuber Saiyed, Komal Patel, Labdhi Pandya, Afzal Ansari, Nikha Trivedi, Gauravi Dhruv, Arti Trivedi, Apurvasinh Puvar, Janvi Raval, Zarna Patel, Monika Gandhi, Pinal Trivedi, Maharshi Pandya, R D Dixit, A M Kadri, Harsh Bakshi, Chaitanya Joshi, Madhvi Joshi
EPI_ISL_483850	Department of Microbiology, Government Medical College, Surat	Gujarat Biotechnology Research Centre	Nitin Savaliya, Raghawendra Kumar, Dinesh Kumar, Zuber Saiyed, Komal Patel, Labdhi Pandya, Afzal Ansari, Nikha Trivedi, Naresh Chauhan, Summaiya Mullan, Amit gamit, Apurvasinh Puvar, Janvi Raval, Zarna Patel, Monika Gandhi, Pinal Trivedi, Maharshi Pandya, Nidhi Patel, R D Dixit, A M Kadri, Harsh Bakshi, Chaitanya Joshi, Madhvi Joshi
EPI_ISL_483851	Department of Microbiology, Government Medical College, Surat	Gujarat Biotechnology Research Centre	Raghawendra Kumar, Dinesh Kumar, Zuber Saiyed, Komal Patel, Labdhi Pandya, Afzal Ansari, Nikha Trivedi, Naresh Chauhan, Summaiya Mullan, Amit gamit, Apurvasinh Puvar, Janvi Raval, Zarna Patel, Monika Gandhi, Pinal Trivedi, Maharshi Pandya, Nidhi Patel, Nitin Savaliya, R D Dixit, A M Kadri, Harsh Bakshi, Chaitanya Joshi, Madhvi Joshi
EPI_ISL_483852	Department of Microbiology, Government Medical College, Surat	Gujarat Biotechnology Research Centre	Dinesh Kumar, Zuber Saiyed, Komal Patel, Labdhi Pandya, Afzal Ansari, Nikha Trivedi, Naresh Chauhan, Summaiya Mullan, Amit gamit, Apurvasinh Puvar, Janvi Raval, Zarna Patel, Monika Gandhi, Pinal Trivedi, Maharshi Pandya, Nidhi Patel, Nitin Savaliya, Raghawendra Kumar, R D Dixit, A M Kadri, Harsh Bakshi, Chaitanya Joshi, Madhvi Joshi
EPI_ISL_483853	Department of Microbiology, Government Medical College, Surat	Gujarat Biotechnology Research Centre	Zuber Saiyed, Komal Patel, Labdhi Pandya, Afzal Ansari, Nikha Trivedi, Naresh Chauhan, Summaiya Mullan, Amit gamit, Apurvasinh Puvar, Janvi Raval, Zarna Patel, Monika Gandhi, Pinal Trivedi, Maharshi Pandya, Nidhi Patel, Nitin Savaliya, Raghawendra Kumar, Dinesh Kumar, R D Dixit, A M Kadri, Harsh Bakshi, Chaitanya Joshi, Madhvi Joshi
EPI_ISL_483854	Department of Microbiology, Government Medical College, Surat	Gujarat Biotechnology Research Centre	Komal Patel, Labdhi Pandya, Afzal Ansari, Nikha Trivedi, Naresh Chauhan, Summaiya Mullan, Amit gamit, Apurvasinh Puvar, Janvi Raval, Zarna Patel, Monika Gandhi, Pinal Trivedi, Maharshi Pandya, Nidhi Patel, Nitin Savaliya, Raghawendra Kumar, Dinesh Kumar, Zuber Saiyed, R D Dixit, A M Kadri, Harsh Bakshi, Chaitanya Joshi, Madhvi Joshi
EPI_ISL_483855	Department of Microbiology, Government Medical College, Surat	Gujarat Biotechnology Research Centre	Labdhi Pandya, Afzal Ansari, Nikha Trivedi, Naresh Chauhan, Summaiya Mullan, Amit gamit, Apurvasinh Puvar, Janvi Raval, Zarna Patel, Monika Gandhi, Pinal Trivedi, Maharshi Pandya, Nidhi Patel, Nitin Savaliya, Raghawendra Kumar, Dinesh Kumar, Zuber Saiyed, Komal Patel, R D Dixit, A M Kadri, Harsh Bakshi, Chaitanya Joshi, Madhvi Joshi
EPI_ISL_483856	Department of Microbiology, Government Medical College, Surat	Gujarat Biotechnology Research Centre	Afzal Ansari, Nikha Trivedi, Naresh Chauhan, Summaiya Mullan, Amit gamit, Apurvasinh Puvar, Janvi Raval, Zarna Patel, Monika Gandhi, Pinal Trivedi, Maharshi Pandya, Nidhi Patel, Nitin Savaliya, Raghawendra Kumar, Dinesh Kumar, Zuber Saiyed, Komal Patel, Labdhi Pandya, R D Dixit, A M Kadri, Harsh Bakshi, Chaitanya Joshi, Madhvi Joshi
EPI_ISL_483857	Department of Microbiology, Government Medical College, Surat	Gujarat Biotechnology Research Centre	Nikha Trivedi, Naresh Chauhan, Summaiya Mullan, Amit gamit, Apurvasinh Puvar, Janvi Raval, Zarna Patel, Monika Gandhi, Pinal Trivedi, Maharshi Pandya, Nidhi Patel, Nitin Savaliya, Raghawendra Kumar, Dinesh Kumar, Zuber Saiyed, Komal Patel, Labdhi Pandya, Afzal Ansari, R D Dixit, A M Kadri, Harsh Bakshi, Chaitanya Joshi, Madhvi Joshi
EPI_ISL_483858	Department of Microbiology, Government Medical College, Surat	Gujarat Biotechnology Research Centre	Naresh Chauhan, Summaiya Mullan, Amit gamit, Apurvasinh Puvar, Janvi Raval, Zarna Patel, Monika Gandhi, Pinal Trivedi, Maharshi Pandya, Nidhi Patel, Nitin Savaliya, Raghawendra Kumar, Dinesh Kumar, Zuber Saiyed, Komal Patel, Labdhi Pandya, Afzal Ansari, Nikha Trivedi, R D Dixit, A M Kadri, Harsh Bakshi, Chaitanya Joshi, Madhvi Joshi
EPI_ISL_483859	Department of Microbiology, Government Medical College, Surat	Gujarat Biotechnology Research Centre	Summaiya Mullan, Amit gamit, Apurvasinh Puvar, Janvi Raval, Zarna Patel, Monika Gandhi, Pinal Trivedi, Maharshi Pandya, Nidhi Patel, Nitin Savaliya, Raghawendra Kumar, Dinesh Kumar, Zuber Saiyed, Komal Patel, Labdhi Pandya, Afzal Ansari, Nikha Trivedi, Naresh Chauhan, R D Dixit, A M Kadri, Harsh Bakshi, Chaitanya Joshi, Madhvi Joshi
EPI_ISL_483860	Department of Microbiology, Government Medical College, Surat	Gujarat Biotechnology Research Centre	Amit gamit, Apurvasinh Puvar, Janvi Raval, Zarna Patel, Monika Gandhi, Pinal Trivedi, Maharshi Pandya, Nidhi Patel, Nitin Savaliya, Raghawendra Kumar, Dinesh Kumar, Zuber Saiyed, Komal Patel, Labdhi Pandya, Afzal Ansari, Nikha Trivedi, Naresh Chauhan, Summaiya Mullan, R D Dixit, A M Kadri, Harsh Bakshi, Chaitanya Joshi, Madhvi Joshi
EPI_ISL_483861	Department of Microbiology, Government Medical College, Surat	Gujarat Biotechnology Research Centre	Apurvasinh Puvar, Janvi Raval, Zarna Patel, Monika Gandhi, Pinal Trivedi, Maharshi Pandya, Nidhi Patel, Nitin Savaliya, Raghawendra Kumar, Dinesh Kumar, Zuber Saiyed, Komal Patel, Labdhi Pandya, Afzal Ansari, Nikha Trivedi, Naresh Chauhan, Summaiya Mullan, Amit gamit, R D Dixit, A M Kadri, Harsh Bakshi, Chaitanya Joshi, Madhvi Joshi
EPI_ISL_483862	Department of Microbiology, Government Medical College, Surat	Gujarat Biotechnology Research Centre	Janvi Raval, Zarna Patel, Monika Gandhi, Pinal Trivedi, Maharshi Pandya, Nidhi Patel, Nitin Savaliya, Raghawendra Kumar, Dinesh Kumar, Zuber Saiyed, Komal Patel, Labdhi Pandya, Afzal Ansari, Nikha Trivedi, Naresh Chauhan, Summaiya Mullan, Amit gamit, Apurvasinh Puvar, R D Dixit, A M Kadri, Harsh Bakshi, Chaitanya Joshi, Madhvi Joshi
EPI_ISL_483863	Department of Microbiology, Government Medical College, Surat	Gujarat Biotechnology Research Centre	Zarna Patel, Monika Gandhi, Pinal Trivedi, Maharshi Pandya, Nidhi Patel, Nitin Savaliya, Raghawendra Kumar, Dinesh Kumar, Zuber Saiyed, Komal Patel, Labdhi Pandya, Afzal Ansari, Nikha Trivedi, Naresh Chauhan, Summaiya Mullan, Amit gamit, Apurvasinh Puvar, Janvi Raval, R D Dixit, A M Kadri, Harsh Bakshi, Chaitanya Joshi, Madhvi Joshi
EPI_ISL_483864	Department of Microbiology, Government Medical College, Surat	Gujarat Biotechnology Research Centre	Monika Gandhi, Pinal Trivedi, Maharshi Pandya, Nidhi Patel, Nitin Savaliya, Raghawendra Kumar, Dinesh Kumar, Zuber Saiyed, Komal Patel, Labdhi Pandya, Afzal Ansari, Nikha Trivedi, Naresh Chauhan, Summaiya Mullan, Amit gamit, Apurvasinh Puvar, Janvi Raval, Zarna Patel, R D Dixit, A M Kadri, Harsh Bakshi, Chaitanya Joshi, Madhvi Joshi
EPI_ISL_483865	Department of Microbiology, Government Medical College, Surat	Gujarat Biotechnology Research Centre	Pinal Trivedi, Maharshi Pandya, Nidhi Patel, Nitin Savaliya, Raghawendra Kumar, Dinesh Kumar, Zuber Saiyed, Komal Patel, Labdhi Pandya, Afzal Ansari, Nikha Trivedi, Naresh Chauhan, Summaiya Mullan, Amit gamit, Apurvasinh Puvar, Janvi Raval, Zarna Patel, Monika Gandhi, R D Dixit, A M Kadri, Harsh Bakshi, Chaitanya Joshi, Madhvi Joshi
EPI_ISL_483866	Department of Microbiology, Government Medical College, Surat	Gujarat Biotechnology Research Centre	Maharshi Pandya, Nidhi Patel, Nitin Savaliya, Raghawendra Kumar, Dinesh Kumar, Zuber Saiyed, Komal Patel, Labdhi Pandya, Afzal Ansari, Nikha Trivedi, Naresh Chauhan, Summaiya Mullan, Amit gamit, Apurvasinh Puvar, Janvi Raval, Zarna Patel, Monika Gandhi, Pinal Trivedi, R D Dixit, A M Kadri, Harsh Bakshi, Chaitanya Joshi, Madhvi Joshi
EPI_ISL_483867	Department of Microbiology, Government Medical College, Surat	Gujarat Biotechnology Research Centre	Nidhi Patel, Nitin Savaliya, Raghawendra Kumar, Dinesh Kumar, Zuber Saiyed, Komal Patel, Labdhi Pandya, Afzal Ansari, Nikha Trivedi, Naresh Chauhan, Summaiya Mullan, Amit gamit, Apurvasinh Puvar, Janvi Raval, Zarna Patel, Monika Gandhi, Pinal Trivedi, Maharshi Pandya, R D Dixit, A M Kadri, Harsh Bakshi, Chaitanya Joshi, Madhvi Joshi
EPI_ISL_483868	Department of Microbiology, Government Medical College, Surat	Gujarat Biotechnology Research Centre	Nitin Savaliya, Raghawendra Kumar, Dinesh Kumar, Zuber Saiyed, Komal Patel, Labdhi Pandya, Afzal Ansari, Nikha Trivedi, Naresh Chauhan, Summaiya Mullan, Amit gamit, Apurvasinh Puvar, Janvi Raval, Zarna Patel, Monika Gandhi, Pinal Trivedi, Maharshi Pandya, Nidhi Patel, R D Dixit, A M Kadri, Harsh Bakshi, Chaitanya Joshi, Madhvi Joshi
EPI_ISL_483869	Department of Microbiology, Government Medical College, Surat	Gujarat Biotechnology Research Centre	Raghawendra Kumar, Dinesh Kumar, Zuber Saiyed, Komal Patel, Labdhi Pandya, Afzal Ansari, Nikha Trivedi, Naresh Chauhan, Summaiya Mullan, Amit gamit, Apurvasinh Puvar, Janvi Raval, Zarna Patel, Monika Gandhi, Pinal Trivedi, Maharshi Pandya, Nidhi Patel, Nitin Savaliya, R D Dixit, A M Kadri, Harsh Bakshi, Chaitanya Joshi, Madhvi Joshi
EPI_ISL_483870	Department of Microbiology, Government Medical College, Surat	Gujarat Biotechnology Research Centre	Dinesh Kumar, Zuber Saiyed, Komal Patel, Labdhi Pandya, Afzal Ansari, Nikha Trivedi, Naresh Chauhan, Summaiya Mullan, Amit gamit, Apurvasinh Puvar, Janvi Raval, Zarna Patel, Monika Gandhi, Pinal Trivedi, Maharshi Pandya, Nidhi Patel, Nitin Savaliya, Raghawendra Kumar, R D Dixit, A M Kadri, Harsh Bakshi, Chaitanya Joshi, Madhvi Joshi
EPI_ISL_483871	Department of Microbiology, Government Medical College, Surat	Gujarat Biotechnology Research Centre	Zuber Saiyed, Komal Patel, Labdhi Pandya, Afzal Ansari, Nikha Trivedi, Naresh Chauhan, Summaiya Mullan, Amit gamit, Apurvasinh Puvar, Janvi Raval, Zarna Patel, Monika Gandhi, Pinal Trivedi, Maharshi Pandya, Nidhi Patel, Nitin Savaliya, Raghawendra Kumar, Dinesh Kumar, R D Dixit, A M Kadri, Harsh Bakshi, Chaitanya Joshi, Madhvi Joshi
EPI_ISL_483872	Department of Microbiology, Government Medical College, Surat	Gujarat Biotechnology Research Centre	Komal Patel, Labdhi Pandya, Afzal Ansari, Nikha Trivedi, Naresh Chauhan, Summaiya Mullan, Amit gamit, Apurvasinh Puvar, Janvi Raval, Zarna Patel, Monika Gandhi, Pinal Trivedi, Maharshi Pandya, Nidhi Patel, Nitin Savaliya, Raghawendra Kumar, Dinesh Kumar, Zuber Saiyed, R D Dixit, A M Kadri, Harsh Bakshi, Chaitanya Joshi, Madhvi Joshi
EPI_ISL_483873	Department of Microbiology, Government Medical College, Surat	Gujarat Biotechnology Research Centre	Labdhi Pandya, Afzal Ansari, Nikha Trivedi, Naresh Chauhan, Summaiya Mullan, Amit gamit, Apurvasinh Puvar, Janvi Raval, Zarna Patel, Monika Gandhi, Pinal Trivedi, Maharshi Pandya, Nidhi Patel, Nitin Savaliya, Raghawendra Kumar, Dinesh Kumar, Zuber Saiyed, Komal Patel, R D Dixit, A M Kadri, Harsh Bakshi, Chaitanya Joshi, Madhvi Joshi

EPI_ISL_483874	Department of Microbiology, Government Medical College, Surat	Gujarat Biotechnology Research Centre	Afzal Ansari, Nikha Trivedi, Naresh Chauhan, Summaiya Mullan, Amit gamit, Apurvasinh Puvar, Janvi Raval, Zarna Patel, Monika Gandhi, Pinal Trivedi, Maharshi Pandya, Nidhi Patel, Nitin Savaliya, Raghawendra Kumar, Dinesh Kumar, Zuber Saiyed, Komal Patel, Labdhi Pandya, R D Dixit, A M Kadri, Harsh Bakshi, Chaitanya Joshi, Madhvi Joshi
EPI_ISL_483875	Department of Microbiology, Government Medical College, Surat	Gujarat Biotechnology Research Centre	Nikha Trivedi, Naresh Chauhan, Summaiya Mullan, Amit gamit, Apurvasinh Puvar, Janvi Raval, Zarna Patel, Monika Gandhi, Pinal Trivedi, Maharshi Pandya, Nidhi Patel, Nitin Savaliya, Raghawendra Kumar, Dinesh Kumar, Zuber Saiyed, Komal Patel, Labdhi Pandya, Afzal Ansari, R D Dixit, A M Kadri, Harsh Bakshi, Chaitanya Joshi, Madhvi Joshi
EPI_ISL_483876	Department of Microbiology, Government Medical College, Surat	Gujarat Biotechnology Research Centre	Naresh Chauhan, Summaiya Mullan, Amit gamit, Apurvasinh Puvar, Janvi Raval, Zarna Patel, Monika Gandhi, Pinal Trivedi, Maharshi Pandya, Nidhi Patel, Nitin Savaliya, Raghawendra Kumar, Dinesh Kumar, Zuber Saiyed, Komal Patel, Labdhi Pandya, Afzal Ansari, Nikha Trivedi, R D Dixit, A M Kadri, Harsh Bakshi, Chaitanya Joshi, Madhvi Joshi
EPI_ISL_483877	Department of Microbiology, Government Medical College, Surat	Gujarat Biotechnology Research Centre	Summaiya Mullan, Amit gamit, Apurvasinh Puvar, Janvi Raval, Zarna Patel, Monika Gandhi, Pinal Trivedi, Maharshi Pandya, Nidhi Patel, Nitin Savaliya, Raghawendra Kumar, Dinesh Kumar, Zuber Saiyed, Komal Patel, Labdhi Pandya, Afzal Ansari, Nikha Trivedi, Naresh Chauhan, R D Dixit, A M Kadri, Harsh Bakshi, Chaitanya Joshi, Madhvi Joshi
EPI_ISL_483878	Department of Microbiology, Government Medical College, Surat	Gujarat Biotechnology Research Centre	Amit gamit, Apurvasinh Puvar, Janvi Raval, Zarna Patel, Monika Gandhi, Pinal Trivedi, Maharshi Pandya, Nidhi Patel, Nitin Savaliya, Raghawendra Kumar, Dinesh Kumar, Zuber Saiyed, Komal Patel, Labdhi Pandya, Afzal Ansari, Nikha Trivedi, Naresh Chauhan, Summaiya Mullan, R D Dixit, A M Kadri, Harsh Bakshi, Chaitanya Joshi, Madhvi Joshi
EPI_ISL_483879	Department of Microbiology, Government Medical College, Surat	Gujarat Biotechnology Research Centre	Apurvasinh Puvar, Janvi Raval, Zarna Patel, Monika Gandhi, Pinal Trivedi, Maharshi Pandya, Nidhi Patel, Nitin Savaliya, Raghawendra Kumar, Dinesh Kumar, Zuber Saiyed, Komal Patel, Labdhi Pandya, Afzal Ansari, Nikha Trivedi, Naresh Chauhan, Summaiya Mullan, Amit gamit, R D Dixit, A M Kadri, Harsh Bakshi, Chaitanya Joshi, Madhvi Joshi
EPI_ISL_485603	Division of Infectious Disease	Steininger Lab	Jakob Thannesberger, Ingeborg Klymiuk, Nicolas Rascovan, Lorenz Schubert, Oliver Robak, Christoph Steininger
EPI_ISL_485635, EPI_ISL_485708, EPI_ISL_485710, EPI_ISL_485711	Institut Pasteur Dakar	Institut Pasteur de Dakar	Ndongo Dia, Moussa Moise Diagne, Mamadou diop, Marie Henriette Dior Ndione, Mamadou Malado Jallow, Safietou Sanke, Ousmane Faye, Amadou Alpha Sall.
EPI_ISL_485712	Institut Pasteur	Institut Pasteur de Dakar	Ndongo Dia, Moussa Moise Diagne, Mamadou diop, Marie Henriette Dior Ndione, Mamadou Malado Jallow, Safietou Sanke, Ousmane Faye, Amadou Alpha Sall.
EPI_ISL_485713, EPI_ISL_485715, EPI_ISL_485716, EPI_ISL_485717	Institut Pasteur Dakar	Institut Pasteur de Dakar	Ndongo Dia, Moussa Moise Diagne, Mamadou diop, Marie Henriette Dior Ndione, Mamadou Malado Jallow, Safietou Sanke, Ousmane Faye, Amadou Alpha Sall.
EPI_ISL_486382	District Surveillence Unit	Department of Neurovirology, National Institute of Mental Health and Neuroscience (NIMHANS)	Chitra Pattabiraman, Vijayalakshmi Reddy, Harsha PK, Risha Rasheed, Shafeeq S Hameed, Manjunatha Venkataswamy, Anita Desai, Ravi Vasanthapuram
EPI_ISL_486383	CV Raman Hospital	Department of Neurovirology, National Institute of Mental Health and Neuroscience (NIMHANS)	Chitra Pattabiraman, Vijayalakshmi Reddy, Harsha PK, Risha Rasheed, Shafeeq S Hameed, Manjunatha Venkataswamy, Anita Desai, Ravi Vasanthapuram
EPI_ISL_486384	DH	Department of Neurovirology, National Institute of Mental Health and Neuroscience (NIMHANS)	Chitra Pattabiraman, Vijayalakshmi Reddy, Harsha PK, Risha Rasheed, Shafeeq S Hameed, Manjunatha Venkataswamy, Anita Desai, Ravi Vasanthapuram
EPI_ISL_486385, EPI_ISL_486386	Victoria Hospital	Department of Neurovirology, National Institute of Mental Health and Neuroscience (NIMHANS)	Chitra Pattabiraman, Vijayalakshmi Reddy, Harsha PK, Risha Rasheed, Shafeeq S Hameed, Manjunatha Venkataswamy, Anita Desai, Ravi Vasanthapuram
EPI_ISL_486387, EPI_ISL_486388, EPI_ISL_486389	DH	Department of Neurovirology, National Institute of Mental Health and Neuroscience (NIMHANS)	Chitra Pattabiraman, Vijayalakshmi Reddy, Harsha PK, Risha Rasheed, Shafeeq S Hameed, Manjunatha Venkataswamy, Anita Desai, Ravi Vasanthapuram
EPI_ISL_486392	Victoria Hospital	Department of Neurovirology, National Institute of Mental Health and Neuroscience (NIMHANS)	Chitra Pattabiraman, Vijayalakshmi Reddy, Harsha PK, Risha Rasheed, Shafeeq S Hameed, Manjunatha Venkataswamy, Anita Desai, Ravi Vasanthapuram
EPI_ISL_486393	SJMCH	Department of Neurovirology, National Institute of Mental Health and Neuroscience (NIMHANS)	Chitra Pattabiraman, Vijayalakshmi Reddy, Harsha PK, Risha Rasheed, Shafeeq S Hameed, Manjunatha Venkataswamy, Anita Desai, Ravi Vasanthapuram
EPI_ISL_486394	MIMS	Department of Neurovirology, National Institute of Mental Health and Neuroscience (NIMHANS)	Chitra Pattabiraman, Vijayalakshmi Reddy, Harsha PK, Risha Rasheed, Shafeeq S Hameed, Manjunatha Venkataswamy, Anita Desai, Ravi Vasanthapuram
EPI_ISL_486395	BIMS	Department of Neurovirology, National Institute of Mental Health and Neuroscience (NIMHANS)	Chitra Pattabiraman, Vijayalakshmi Reddy, Harsha PK, Risha Rasheed, Shafeeq S Hameed, Manjunatha Venkataswamy, Anita Desai, Ravi Vasanthapuram
EPI_ISL_486396	Jayanagar General Hospital to Victoria Hospital	Department of Neurovirology, National Institute of Mental Health and Neuroscience (NIMHANS)	Chitra Pattabiraman, Vijayalakshmi Reddy, Harsha PK, Risha Rasheed, Shafeeq S Hameed, Manjunatha Venkataswamy, Anita Desai, Ravi Vasanthapuram
EPI_ISL_486397	KC General Hospital	Department of Neurovirology, National Institute of Mental Health and Neuroscience (NIMHANS)	Chitra Pattabiraman, Vijayalakshmi Reddy, Harsha PK, Risha Rasheed, Shafeeq S Hameed, Manjunatha Venkataswamy, Anita Desai, Ravi Vasanthapuram
EPI_ISL_486398, EPI_ISL_486399	MIMS	Department of Neurovirology, National Institute of Mental Health and Neuroscience (NIMHANS)	Chitra Pattabiraman, Vijayalakshmi Reddy, Harsha PK, Risha Rasheed, Shafeeq S Hameed, Manjunatha Venkataswamy, Anita Desai, Ravi Vasanthapuram
EPI_ISL_486400	Victoria Hospital	Department of Neurovirology, National Institute of Mental Health and Neuroscience (NIMHANS)	Chitra Pattabiraman, Vijayalakshmi Reddy, Harsha PK, Risha Rasheed, Shafeeq S Hameed, Manjunatha Venkataswamy, Anita Desai, Ravi Vasanthapuram
EPI_ISL_486401, EPI_ISL_486402, EPI_ISL_486403	DH	Department of Neurovirology, National Institute of Mental Health and Neuroscience (NIMHANS)	Chitra Pattabiraman, Vijayalakshmi Reddy, Harsha PK, Risha Rasheed, Shafeeq S Hameed, Manjunatha Venkataswamy, Anita Desai, Ravi Vasanthapuram
EPI_ISL_486404	Victoria Hospital	Department of Neurovirology, National Institute of Mental Health and Neuroscience (NIMHANS)	Chitra Pattabiraman, Vijayalakshmi Reddy, Harsha PK, Risha Rasheed, Shafeeq S Hameed, Manjunatha Venkataswamy, Anita Desai, Ravi Vasanthapuram
EPI_ISL_486405, EPI_ISL_486406, EPI_ISL_486407, EPI_ISL_486408, EPI_ISL_486409	DH	Department of Neurovirology, National Institute of Mental Health and Neuroscience (NIMHANS)	Chitra Pattabiraman, Vijayalakshmi Reddy, Harsha PK, Risha Rasheed, Shafeeq S Hameed, Manjunatha Venkataswamy, Anita Desai, Ravi Vasanthapuram
EPI_ISL_486645	Renmin Hospital of Wuhan University Hubei General Hospital	State Key Laboratory of Agriculture Microbiology, Huazhong Agric	Zhong Zou
EPI_ISL_486815, EPI_ISL_486816, EPI_ISL_486817, EPI_ISL_486818, EPI_ISL_486819, EPI_ISL_486820, EPI_ISL_486821, EPI_ISL_486822, EPI_ISL_486823, EPI_ISL_486824, EPI_ISL_486825, EPI_ISL_486826, EPI_ISL_486827, EPI_ISL_486828, EPI_ISL_486829			
see above	Molecular diagnostic laboratory of Federal Budget Institution of Science "Central Research Institute of Epidemiology" of The Federal Service on Customers' Rights Protection and Human Well-being Surveillance	Group of Genomics and Postgenomic Technologies of Central Research Institute of Epidemiology	Speranskaya AS, Kaptelova VV, Valdokhina AV, Bulanenko VP, Samoilov AE, Korneenko EV, Tivanova EV, Shipulina OY, Akimkin VG
EPI_ISL_486834	Suceava County Energency Hospital "Sf. Ioan cel Nou"	SMU Metagenomics lab	Lobiuc Andrei, Antoniadis Panagiotis
EPI_ISL_486842, EPI_ISL_486843, EPI_ISL_486844	Institute of Microbiology, Universidad San Francisco de Quito	Institute of Microbiology, Universidad San Francisco de Quito	Belén Prado-Vivar, Sully Márquez, Juan José Guadalupe, Monica Becerra-Wong, Carla Torres, Bernardo Gutiérrez, Fausto Maldonado, Geovanny Carzola, Verónica Barragán, Patricio Rojas-Silva, Gabriel Trueba, Michelle Grunauer, Paúl Cárdenas
EPI_ISL_486845, EPI_ISL_486846, EPI_ISL_486847, EPI_ISL_486848, EPI_ISL_486849, EPI_ISL_486850, EPI_ISL_486851	Institute of Microbiology, Universidad San Francisco de Quito	Institute of Microbiology, Universidad San Francisco de Quito	Belén Prado-Vivar, Sully Márquez, Juan José Guadalupe, Monica Becerra-Wong, Carla Torres, Bernardo Gutiérrez, Jonathan Araujo, Verónica Barragán, Patricio Rojas-Silva, Gabriel Trueba, Michelle Grunauer, Paúl Cárdenas

EPI_ISL_486852	CDRI/SGPGI	CSIR-CDRI/SGPGI	Saumya Sarkar, Dharam Veer Singh, Rahul Vishvkarma, Ujjala Ghoshal, Uday Ghoshal, Ravishankar Ramachandran, Tapas Kumar Kundu, Rajender Singh
EPI_ISL_486853	CSIR-CDRI/SGPGI	CSIR-CDRI/SGPGI	Saumya Sarkar, Dharam Veer Singh, Rahul Vishvkarma, Ujjala Ghoshal, Uday Ghoshal, Ravishankar Ramachandran, Tapas Kumar Kundu, Rajender Singh
EPI_ISL_486854	Emergency County Hospital Suceava	Stefan cel Mare, University Metagenomics lab	Lobiuc Andrei et al.
EPI_ISL_486855	Emergency county Hospital Suceava	"Stefan cel Mare" University Metagenomics Lab	Lobiuc Andrei et al.
EPI_ISL_486856	Emergency County Hospital	Stefan cel Mare, University Metagenomics lab	Lobiuc Andrei et al.
EPI_ISL_486857, EPI_ISL_486859, EPI_ISL_486860, EPI_ISL_486861, EPI_ISL_486862, EPI_ISL_486863, EPI_ISL_486864, EPI_ISL_486865, EPI_ISL_486866, EPI_ISL_486867, EPI_ISL_486868, EPI_ISL_486869, EPI_ISL_486870, EPI_ISL_486871, EPI_ISL_486872, EPI_ISL_486873			
see above	Institut Pasteur Dakar	Institut Pasteur de Dakar	Ndongo Dia, Moussa Moise Diagne, Mamadou Diop, Marie Henriette Dior Ndione, Mamadou Malado Jallow, Safietou Sanke, Ousmane Faye, Amadou Alpha Sall.
EPI_ISL_486876	Clinical Microbiology Laboratory- Basurto University Hospital	Biocruces-Bizkaia	Mikel J. Urrutikoetxea-Gutierrez, Ana Belén Belén de la Hoz, Matxalen Vidal-García, Mº Carmen Nieto Toboso, Estibaliz Ugalde-Zarraga, José Luis Díaz de Tuesta del Arco
EPI_ISL_486881	CV Raman Hospital	Department of Neurovirology, National Institute of Mental Health and Neuroscience (NIMHANS)	Chitra Pattabiraman, Vijayalakshmi Reddy, Harsha PK, Risha Rasheed, Shafeeq S Hameed, Manjunatha Venkataswamy, Anita Desai, Ravi Vasanthapuram
EPI_ISL_487087, EPI_ISL_487090, EPI_ISL_487095, EPI_ISL_487097, EPI_ISL_487098, EPI_ISL_487107	Nigeria Centre for Disease Control (NCDC)	African Centre of Excellence for Genomics of Infectious Diseases (ACEGID), Redeemer's University, Ede, Osun State, Nigeria	Oluniyi P.E., Ajogbasile F.V., Kayode A., Oguzie J., Olawoye I., Uwanibe J., Olumade T., Folarin O.A., Ihekweazu C., Happi C.T.
EPI_ISL_487277, EPI_ISL_487278, EPI_ISL_487279, EPI_ISL_487280, EPI_ISL_487281, EPI_ISL_487282, EPI_ISL_487283, EPI_ISL_487284, EPI_ISL_487285, EPI_ISL_487286, EPI_ISL_487287, EPI_ISL_487288, EPI_ISL_487289, EPI_ISL_487290, EPI_ISL_487291, EPI_ISL_487292, EPI_ISL_487293, EPI_ISL_487294, EPI_ISL_487295, EPI_ISL_487296, EPI_ISL_487297, EPI_ISL_487298, EPI_ISL_487299, EPI_ISL_487300, EPI_ISL_487301, EPI_ISL_487302, EPI_ISL_487303, EPI_ISL_487304, EPI_ISL_487305, EPI_ISL_487306, EPI_ISL_487307, EPI_ISL_487308, EPI_ISL_487309, EPI_ISL_487310, EPI_ISL_487311, EPI_ISL_487312, EPI_ISL_487313, EPI_ISL_487314, EPI_ISL_487315, EPI_ISL_487316, EPI_ISL_487317, EPI_ISL_487318, EPI_ISL_487319, EPI_ISL_487320, EPI_ISL_487321, EPI_ISL_487322, EPI_ISL_487323, EPI_ISL_487324, EPI_ISL_487325, EPI_ISL_487326, EPI_ISL_487327, EPI_ISL_487328			
see above	NHLS-IALCH	KRISP, KZN Research Innovation and Sequencing Platform	Giandhari J, Pillay S, Lessells R, Chimukangara B, Mdlalose K, York D, Khan S, Tegally H, Wilkinson E, de Oliveira T
EPI_ISL_487329, EPI_ISL_487330, EPI_ISL_487331, EPI_ISL_487332, EPI_ISL_487333, EPI_ISL_487334, EPI_ISL_487335, EPI_ISL_487336, EPI_ISL_487337, EPI_ISL_487338, EPI_ISL_487339, EPI_ISL_487340, EPI_ISL_487341			
see above	Molecular Diagnostics Services (MDS)	KRISP, KZN Research Innovation and Sequencing Platform	Giandhari J, Pillay S, Lessells R, Chimukangara B, Mdlalose K, York D, Khan S, Tegally H, Wilkinson E, de Oliveira T
EPI_ISL_487348	NHLS-IALCH	KRISP, KZN Research Innovation and Sequencing Platform	Giandhari J, Pillay S, Lessells R, Chimukangara B, Mdlalose K, York D, Khan S, Tegally H, Wilkinson E, de Oliveira T
EPI_ISL_487370, EPI_ISL_487377, EPI_ISL_487379, EPI_ISL_487381	Hellenic Pasteur Institute, National Influenza Reference laboratory of Southern Greece & Unit of Bioinformatics and Applied Genomics	Hellenic Pasteur Institute, National Influenza Reference laboratory of Southern Greece & Unit of Bioinformatics and Applied Genomics	Vasiliki Pogka, Timokratis Karamitros, Athanasios Kossyvakis, Antonios Kalliaropoulos, Horefti Elina, Evangelidou Maria, Androniki Voulgari-Kokota, Aspasia Kontou, Andreas Mentis
EPI_ISL_487432, EPI_ISL_487433, EPI_ISL_487434, EPI_ISL_487435, EPI_ISL_487436	Queen Astrid Military Hospital	Institute of Tropical Medicine	Philippe Selhorst, Colin Anthony
EPI_ISL_489708, EPI_ISL_489709	The National Institute of Public Health	The National Institute of Public Health and State Veterinary Institute Prague	Nagy,A;Jirincova,H;Novakova,L;Trnka,D;Vecerova,J
EPI_ISL_489833, EPI_ISL_489834, EPI_ISL_489835	Clinical Microbiology Laboratory- Basurto University Hospital	Biocruces-Bizkaia	Mikel J. Urrutikoetxea-Gutierrez, Ana Belén Belén de la Hoz, Matxalen Vidal-García, Mº Carmen Nieto Toboso, Estibaliz Ugalde-Zarraga, José Luis Díaz de Tuesta del Arco
EPI_ISL_489995	CSIR-CDRI/SGPGI, Lucknow	CSIR-CDRI/SGPGI, Lucknow	Saumya Sarkar, Dharam Veer Singh, Rahul Vishvkarma, Ujjala Ghoshal, Uday Ghoshal, Ravishankar Ramachandran, Tapas Kumar Kundu, Rajender Singh
EPI_ISL_490013	CSIR-CDRI/SGPGI, Lucknow	CSIR-CDRI, Lucknow	Saumya Sarkar, Dharam Veer Singh, Rahul Vishvkarma, Ujjala Ghoshal, Uday Ghoshal, Ravishankar Ramachandran, Tapas Kumar Kundu, Rajender Singh
EPI_ISL_490101, EPI_ISL_490102, EPI_ISL_490103	Institute for Medical Research, Infectious Disease Research Centre, National Institutes of Health, Ministry of Health Malaysia	Institute for Medical Research, Infectious Disease Research Centre, National Institutes of Health, Ministry of Health Malaysia	Suppiah J, Mohd-Zawawi Z, Kamel K, Kalyanasundram J, Thayan R
EPI_ISL_490104, EPI_ISL_490106	CSIR-CDRI/SGPGI, Lucknow	CSIR-CDRI/SGPGI, Lucknow	Saumya Sarkar, Dharam Veer Singh, Rahul Vishvkarma, Ujjala Ghoshal, Uday Ghoshal, Ravishankar Ramachandran, Tapas Kumar Kundu, Rajender Singh
EPI_ISL_490112	The National Institute of Public Health	The National Institute of Public Health and State Veterinary Institute Prague	Nagy,A;Jirincova,H;Novakova,L;Trnka,D;Vecerova,J
EPI_ISL_490202, EPI_ISL_490203, EPI_ISL_490204, EPI_ISL_490977	Clinical Microbiology Laboratory- Basurto University Hospital	Biocruces-Bizkaia	Mikel J. Urrutikoetxea-Gutierrez, Ana Belén Belén de la Hoz, Matxalen Vidal-García, Mº Carmen Nieto Toboso, Estibaliz Ugalde-Zarraga, José Luis Díaz de Tuesta del Arco
EPI_ISL_491092	The National Institute of Public Health	State Veterinary Institute Prague	Nagy,A;Jirincova,H;Novakova,L;Trnka,D;Vecerova,J
EPI_ISL_491093, EPI_ISL_491094, EPI_ISL_491095	The National Institute of Public Health	The National Institute of Public Health and State Veterinary Institute Prague	Nagy,A; Jirincova,H; Novakova,L; Trnka,D; Vecerova,J
EPI_ISL_491096, EPI_ISL_491113, EPI_ISL_491114	CSIR-CDRI/SGPGI, Lucknow	CSIR-CDRI/SGPGI, Lucknow	Saumya Sarkar, Dharam Veer Singh, Rahul Vishvkarma, Ujjala Ghoshal, Uday Ghoshal, Ravishankar Ramachandran, Tapas Kumar Kundu, Rajender Singh
EPI_ISL_491115	Cicin-Sain Lab	Cicin-Sain Lab	M. Zeeshan Chaudhry, Kathrin Eschke, Yeonsu Kim, Luka Cicin-Sain
EPI_ISL_491117, EPI_ISL_491118	The National Institute of Public Health	The National Institute of Public Health and State Veterinary Institute Prague	Nagy,A;Jirincova,H;Novakova,L;Trnka,D;Vecerova,J
EPI_ISL_491436	Laboratorio de Referencia Nacional de Virus Respiratorio. Instituto Nacional de Salud Perú	Laboratorio de Referencia Nacional de Biotecnología y Biología Molecular. Instituto Nacional de Salud Perú	Carlos Padilla Rojas, Karolyn Vega Chozo, Priscila Lope Pari, Omar Caceres Rey, Marco Galarza Perez, Maribel Huaringa Nuñez, Johanna Balbuena Torres, Henri Bailon Calderon, Nancy Rojas Serrano
EPI_ISL_491437	Area de Salud Escazu (Coopesana)	Inciensa, Instituto Costarricense de Investigación y Enseñanza en Nutrición y Salud	Francisco Duarte, Hebleen Brenes, Claudio Soto-Garita, Estela Cordero, Adriana Godinez & Melany Calderon
EPI_ISL_491438	Hospital San Rafael de Alajuela	Inciensa, Instituto Costarricense de Investigación y Enseñanza en Nutrición y Salud	Francisco Duarte, Hebleen Brenes, Claudio Soto-Garita, Estela Cordero, Adriana Godinez & Melany Calderon
EPI_ISL_491439	Hospital Calderon Guardia	Inciensa, Instituto Costarricense de Investigación y Enseñanza en Nutrición y Salud	Francisco Duarte, Hebleen Brenes, Claudio Soto-Garita, Estela Cordero, Adriana Godinez & Melany Calderon
EPI_ISL_491440	Hospital San Rafael de Alajuela	Inciensa, Instituto Costarricense de Investigación y Enseñanza en Nutrición y Salud	Francisco Duarte, Hebleen Brenes, Claudio Soto-Garita, Estela Cordero, Adriana Godinez & Melany Calderon
EPI_ISL_491441, EPI_ISL_491442	Hospital Clinica Biblica	Inciensa, Instituto Costarricense de Investigación y Enseñanza en Nutrición y Salud	Francisco Duarte, Hebleen Brenes, Claudio Soto-Garita, Estela Cordero, Adriana Godinez & Melany Calderon
EPI_ISL_491443	Hospital Fernando Escalante Pradilla	Inciensa, Instituto Costarricense de Investigación y Enseñanza en Nutrición y Salud	Francisco Duarte, Hebleen Brenes, Claudio Soto-Garita, Estela Cordero, Adriana Godinez & Melany Calderon

EPI_ISL_491444	Area de Salud Escazu (Coopesana)	Inciensa, Instituto Costarricense de Investigación y Enseñanza en Nutrición y Salud	Francisco Duarte, Hebleen Brenes, Claudio Soto-Garita, Estela Cordero, Adriana Godinez & Melany Calderon
EPI_ISL_491445	Area de Salud Mata Redonda	Inciensa, Instituto Costarricense de Investigación y Enseñanza en Nutrición y Salud	Francisco Duarte, Hebleen Brenes, Claudio Soto-Garita, Estela Cordero, Adriana Godinez & Melany Calderon
EPI_ISL_491446	Area de Salud Alajuela Central	Inciensa, Instituto Costarricense de Investigación y Enseñanza en Nutrición y Salud	Francisco Duarte, Hebleen Brenes, Claudio Soto-Garita, Estela Cordero, Adriana Godinez & Melany Calderon
EPI_ISL_491447	Hospital Fernando Escalante Pradilla	Inciensa, Instituto Costarricense de Investigación y Enseñanza en Nutrición y Salud	Francisco Duarte, Hebleen Brenes, Claudio Soto-Garita, Estela Cordero, Adriana Godinez & Melany Calderon
EPI_ISL_491448	Hospital San Rafael de Alajuela	Inciensa, Instituto Costarricense de Investigación y Enseñanza en Nutrición y Salud	Francisco Duarte, Hebleen Brenes, Claudio Soto-Garita, Estela Cordero, Adriana Godinez & Melany Calderon
EPI_ISL_491449	Area de Salud Alajuela Sur	Inciensa, Instituto Costarricense de Investigación y Enseñanza en Nutrición y Salud	Francisco Duarte, Hebleen Brenes, Claudio Soto-Garita, Estela Cordero, Adriana Godinez & Melany Calderon
EPI_ISL_491450	Hospital San Juan de Dios	Inciensa, Instituto Costarricense de Investigación y Enseñanza en Nutrición y Salud	Francisco Duarte, Hebleen Brenes, Claudio Soto-Garita, Estela Cordero, Adriana Godinez & Melany Calderon
EPI_ISL_491451	Hospital México	Inciensa, Instituto Costarricense de Investigación y Enseñanza en Nutrición y Salud	Francisco Duarte, Hebleen Brenes, Claudio Soto-Garita, Estela Cordero, Adriana Godinez & Melany Calderon
EPI_ISL_491452	Hospital San Rafael de Alajuela	Inciensa, Instituto Costarricense de Investigación y Enseñanza en Nutrición y Salud	Francisco Duarte, Hebleen Brenes, Claudio Soto-Garita, Estela Cordero, Adriana Godinez & Melany Calderon
EPI_ISL_491453	Hospital México	Inciensa, Instituto Costarricense de Investigación y Enseñanza en Nutrición y Salud	Francisco Duarte, Hebleen Brenes, Claudio Soto-Garita, Estela Cordero, Adriana Godinez & Melany Calderon
EPI_ISL_491454	Hospital San Juan de Dios	Inciensa, Instituto Costarricense de Investigación y Enseñanza en Nutrición y Salud	Francisco Duarte, Hebleen Brenes, Claudio Soto-Garita, Estela Cordero, Adriana Godinez & Melany Calderon
EPI_ISL_491455	Hospital Clinica Biblica	Inciensa, Instituto Costarricense de Investigación y Enseñanza en Nutrición y Salud	Francisco Duarte, Hebleen Brenes, Claudio Soto-Garita, Estela Cordero, Adriana Godinez & Melany Calderon
EPI_ISL_491456	Hospital San Juan de Dios	Inciensa, Instituto Costarricense de Investigación y Enseñanza en Nutrición y Salud	Francisco Duarte, Hebleen Brenes, Claudio Soto-Garita, Estela Cordero, Adriana Godinez & Melany Calderon
EPI_ISL_491457	Area de Salud Los Santos	Inciensa, Instituto Costarricense de Investigación y Enseñanza en Nutrición y Salud	Francisco Duarte, Hebleen Brenes, Claudio Soto-Garita, Estela Cordero, Adriana Godinez & Melany Calderon
EPI_ISL_491463	Laboratorio de Referencia Nacional de Virus Respiratorio. Instituto Nacional de Salud Perú	Laboratorio de Referencia Nacional de Biotecnología y Biología Molecular. Instituto Nacional de Salud Perú	Carlos Padilla Rojas, Karolyn Vega Chozo, Priscila Lope Pari, Omar Caceres Rey, Marco Galarza Perez, Maribel Huaringa Nuñez, Johanna Balbuena Torres, Henri Bailon Calderon, Nancy Rojas Serrano
EPI_ISL_491465, EPI_ISL_491466	San Lazaro Hospital	Research Institute for Tropical Medicine	Ma. Angelica Tujan, Othoniel Jan Onza, Francisco Gerardo Polotan, Inez Andrea Medado, Criselda Bautista, Kirstyn Brunker, Edelwisa Mercado, Daria Manalo, Catalino Demetria
EPI_ISL_491467, EPI_ISL_491468	Research Institute for Tropical Medicine	Research Institute for Tropical Medicine	Ma. Angelica Tujan, Othoniel Jan Onza, Francisco Gerardo Polotan, Inez Andrea Medado, Criselda Bautista, Kirstyn Brunker, Edelwisa Mercado, Daria Manalo, Catalino Demetria
EPI_ISL_491470	Cardinal Santos Medical Center	Research Institute for Tropical Medicine	Ma. Angelica Tujan, Othoniel Jan Onza, Francisco Gerardo Polotan, Inez Andrea Medado, Criselda Bautista, Kirstyn Brunker, Edelwisa Mercado, Daria Manalo, Catalino Demetria
EPI_ISL_491471	San Lazaro Hospital	Research Institute for Tropical Medicine	Ma. Angelica Tujan, Othoniel Jan Onza, Francisco Gerardo Polotan, Inez Andrea Medado, Criselda Bautista, Kirstyn Brunker, Edelwisa Mercado, Daria Manalo, Catalino Demetria
EPI_ISL_491472	Cardinal Santos Medical Center	Research Institute for Tropical Medicine	Ma. Angelica Tujan, Othoniel Jan Onza, Francisco Gerardo Polotan, Inez Andrea Medado, Criselda Bautista, Kirstyn Brunker, Edelwisa Mercado, Daria Manalo, Catalino Demetria
EPI_ISL_491473	The Medical City	Research Institute for Tropical Medicine	Ma. Angelica Tujan, Othoniel Jan Onza, Francisco Gerardo Polotan, Inez Andrea Medado, Criselda Bautista, Kirstyn Brunker, Edelwisa Mercado, Daria Manalo, Catalino Demetria
EPI_ISL_491474	Research Institute for Tropical Medicine	Research Institute for Tropical Medicine	Ma. Angelica Tujan, Othoniel Jan Onza, Francisco Gerardo Polotan, Inez Andrea Medado, Criselda Bautista, Kirstyn Brunker, Edelwisa Mercado, Daria Manalo, Catalino Demetria
EPI_ISL_491476	BSL3 Lab, Pendik Veterinary Control Enstitue	Genomic Laboratory (GLAB), Istanbul Technical University	Mustafa HASOKSUZ, Fahriye SARAC, Osman ERGANIS, Serdar UZAR, Hakan ENUL, Cumhur ADIAY, Ahmet SAIT, Orbay SAYI, Kadir YESILBAG, Oguz KARABEY
EPI_ISL_491477, EPI_ISL_491478, EPI_ISL_491479, EPI_ISL_491480	CSIR-CDRI/SGPGI, Lucknow	CSIR-CDRI/SGPGI, Lucknow	Saumya Sarkar, Dharam Veer Singh, Rahul Vishvkarma, Ujjala Ghoshal, Uday Ghoshal, Ravishankar Ramachandran, Tapas Kumar Kundu, Rajender Singh
EPI_ISL_491968, EPI_ISL_491969, EPI_ISL_491970, EPI_ISL_491973, EPI_ISL_491974, EPI_ISL_491976, EPI_ISL_491978, EPI_ISL_491979, EPI_ISL_491980, EPI_ISL_491981, EPI_ISL_491982, EPI_ISL_491983, EPI_ISL_491984, EPI_ISL_491985, EPI_ISL_491986, EPI_ISL_491988, EPI_ISL_491989, EPI_ISL_491990, EPI_ISL_491991, EPI_ISL_491992, EPI_ISL_491993, EPI_ISL_491994, EPI_ISL_491995, EPI_ISL_491996, EPI_ISL_491997, EPI_ISL_491998, EPI_ISL_491999, EPI_ISL_492000, EPI_ISL_492001, EPI_ISL_492002, EPI_ISL_492003, EPI_ISL_492005, EPI_ISL_492006, EPI_ISL_492007, EPI_ISL_492008, EPI_ISL_492009, EPI_ISL_492010, EPI_ISL_492011, EPI_ISL_492012, EPI_ISL_492014, EPI_ISL_492016, EPI_ISL_492017, EPI_ISL_492019, EPI_ISL_492020, EPI_ISL_492021, EPI_ISL_492022, EPI_ISL_492023, EPI_ISL_492024, EPI_ISL_492025, EPI_ISL_492026			
see above	Oman-NIC	Department of Microbiology and Immunology-SQUH	Fahad Zadjali, Samira Al-Maruqi, Amina Al Jardani, Khulood Al-Mammary, Hanan Al-kindi, Fatma BaAlawi, Hamida AL Barwani, Zeyana AL-Dahmani, Intisar Al-Shukri, Aisha Al-Busaidi, Aisha Al-Amri, Ahlam Al-Amri, Mohammed Al-Tobi, Samiha Al Kharusi, Abdulla Balkhair
EPI_ISL_492033	Instituto de Biologia do Exército	Laboratório Metabolismo Macromolecular FirminoTorres de Castro, Instituto de Biofísica Carlos Chagas Filho, Universidade Federal do Rio de Janeiro	Bianca Catarina Azevedo Cabral, Aline Rosa Vianna de Souza, Caleb GM Santos, Marcos Dornelas-Ribeiro, Tatiana LS Nogueira, Nádia Vaez Gonçalves da Cruz, Elizabeth Valentin, Marcio da Costa Cipitelli, Virginia Sara Grancieri do Amaral, Rodrigo Soares de Moura Neto, Clarissa Damaso, Rosane Silva
EPI_ISL_492034	Instituto de Biologia do Exército	Laboratório Metabolismo Macromolecular FirminoTorres de Castro, Instituto de Biofísica Carlos Chagas Filho, Universidade Federal do Rio de Janeiro	Bianca Catarina Azevedo Cabral, Aline Rosa Vianna de Souza, Nádia Vaez Gonçalves da Cruz, Caleb GM Santos, Marcos Dornelas-Ribeiro, Tatiana LS Nogueira, Elizabeth Valentin, Marcio da Costa Cipitelli, Virginia Sara Grancieri do Amaral, Rodrigo Soares de Moura Neto, Clarissa Damaso, Rosane Silva
EPI_ISL_492035	Instituto de Biologia do Exército	Laboratório Metabolismo Macromolecular FirminoTorres de Castro, Instituto de Biofísica Carlos Chagas Filho, Universidade Federal do Rio de Janeiro	Bianca Catarina Azevedo Cabral, Aline Rosa Vianna de Souza, Tatiana LS Nogueira, Nádia Vaez Gonçalves da Cruz, Caleb GM Santos, Marcos Dornelas-Ribeiro, Elizabeth Valentin, Marcio da Costa Cipitelli, Virginia Sara Grancieri do Amaral, Rodrigo Soares de Moura Neto, Clarissa Damaso, Rosane Silva
EPI_ISL_492036	Instituto de Biologia do Exército	Laboratório Metabolismo Macromolecular FirminoTorres de Castro, Instituto de Biofísica Carlos Chagas Filho, Universidade Federal do Rio de Janeiro	Bianca Catarina Azevedo Cabral, Aline Rosa Vianna de Souza , Marcos Dornelas-Ribeiro, Tatiana LS Nogueira, Nádia Vaez Gonçalves da Cruz, Caleb GM Santos, Elizabeth Valentin, Marcio da Costa Cipitelli, Virginia Sara Grancieri do Amaral, Rodrigo Soares de Moura Neto, Clarissa Damaso, Rosane Silva
EPI_ISL_492038	Instituto de Biologia do Exército	Laboratório Metabolismo Macromolecular FirminoTorres de Castro, Instituto de Biofísica Carlos Chagas Filho, Universidade Federal do Rio de Janeiro	Bianca Catarina Azevedo Cabral, Aline Rosa Vianna de Souza, Nádia Vaez Gonçalves da Cruz, Caleb GM Santos, Marcos Dornelas-Ribeiro, Tatiana LS Nogueira, Elizabeth Valentin, Marcio da Costa Cipitelli, Virginia Sara Grancieri do Amaral, Rodrigo Soares de Moura Neto, Clarissa Damaso, Rosane Silva
EPI_ISL_492039	Instituto de Biologia do Exército	Laboratório Metabolismo Macromolecular FirminoTorres de Castro, Instituto de Biofísica Carlos Chagas Filho, Universidade Federal do Rio de Janeiro	Bianca Catarina Azevedo Cabral, Aline Rosa Vianna de Souza, Tatiana LS Nogueira, Nádia Vaez Gonçalves da Cruz, Caleb GM Santos, Marcos Dornelas-Ribeiro, Elizabeth Valentin, Marcio da Costa Cipitelli, Virginia Sara Grancieri do Amaral, Rodrigo Soares de Moura Neto, Clarissa Damaso, Rosane Silva
EPI_ISL_492041	Instituto de Biologia do Exército	Laboratório Metabolismo Macromolecular FirminoTorres de	Bianca Catarina Azevedo Cabral, Aline Rosa Vianna de Souza, Caleb GM Santos, Marcos Dornelas-Ribeiro, Tatiana LS Nogueira, Nádia Vaez Gonçalves

		Castro, Instituto de Biofísica Carlos Chagas Filho, Universidade Federal do Rio de Janeiro	da Cruz, Elizabeth Valentin, Marcio da Costa Cipitelli, Virginia Sara Grancieri do Amaral, Rodrigo Soares de Moura Neto, Clarissa Damaso, Rosane Silva
EPI_ISL_492042	Instituto de Biologia do Exército	Laboratório Metabolismo Macromolecular FirminoTorres de Castro, Instituto de Biofísica Carlos Chagas Filho, Universidade Federal do Rio de Janeiro	Bianca Catarina Azevedo Cabral, Aline Rosa Vianna de Souza, Nádia Vaez Gonçalves da Cruz, Caleb GM Santos, Marcos Dornelas-Ribeiro, Tatiana LS Nogueira, Elizabeth Valentin, Marcio da Costa Cipitelli, Virginia Sara Grancieri do Amaral, Rodrigo Soares de Moura Neto, Clarissa Damaso, Rosane Silva
EPI_ISL_492043	Instituto de Biologia do Exército	Laboratório Metabolismo Macromolecular FirminoTorres de Castro, Instituto de Biofísica Carlos Chagas Filho, Universidade Federal do Rio de Janeiro	Bianca Catarina Azevedo Cabral, Aline Rosa Vianna de Souza, Tatiana LS Nogueira, Nádia Vaez Gonçalves da Cruz, Caleb GM Santos, Marcos Dornelas-Ribeiro, Elizabeth Valentin, Marcio da Costa Cipitelli, Virginia Sara Grancieri do Amaral, Rodrigo Soares de Moura Neto, Clarissa Damaso, Rosane Silva
EPI_ISL_492044	Instituto de Biologia do Exército	Laboratório Metabolismo Macromolecular FirminoTorres de Castro, Instituto de Biofísica Carlos Chagas Filho, Universidade Federal do Rio de Janeiro	Bianca Catarina Azevedo Cabral, Aline Rosa Vianna de Souza , Marcos Dornelas-Ribeiro, Tatiana LS Nogueira, Nádia Vaez Gonçalves da Cruz, Caleb GM Santos, Elizabeth Valentin, Marcio da Costa Cipitelli, Virginia Sara Grancieri do Amaral, Rodrigo Soares de Moura Neto, Clarissa Damaso, Rosane Silva
EPI_ISL_492045	Instituto de Biologia do Exército	Laboratório Metabolismo Macromolecular FirminoTorres de Castro, Instituto de Biofísica Carlos Chagas Filho, Universidade Federal do Rio de Janeiro	Bianca Catarina Azevedo Cabral, Aline Rosa Vianna de Souza, Caleb GM Santos, Marcos Dornelas-Ribeiro, Tatiana LS Nogueira, Nádia Vaez Gonçalves da Cruz, Elizabeth Valentin, Marcio da Costa Cipitelli, Virginia Sara Grancieri do Amaral, Rodrigo Soares de Moura Neto, Clarissa Damaso, Rosane Silva
EPI_ISL_492048	Instituto de Biologia do Exército	Laboratório Metabolismo Macromolecular FirminoTorres de Castro, Instituto de Biofísica Carlos Chagas Filho, Universidade Federal do Rio de Janeiro	Bianca Catarina Azevedo Cabral, Aline Rosa Vianna de Souza , Marcos Dornelas-Ribeiro, Tatiana LS Nogueira, Nádia Vaez Gonçalves da Cruz, Caleb GM Santos, Elizabeth Valentin, Marcio da Costa Cipitelli, Virginia Sara Grancieri do Amaral, Rodrigo Soares de Moura Neto, Clarissa Damaso, Rosane Silva
EPI_ISL_492184	INT Fondazione Pascale	INT Fondazione Pascale	Pascale
EPI_ISL_492978, EPI_ISL_492979	Department of Laboratory Medicine Tan Tock Seng Hospital	Department of Laboratory Medicine Tan Tock Seng Hospital	Chen YYC, Zair X, Li C, Tang WY, Maurer-Stroh S, Barkham TMS, Nagarajan N, Sessions OM
EPI_ISL_492982, EPI_ISL_492983, EPI_ISL_492984, EPI_ISL_492985, EPI_ISL_492986, EPI_ISL_492987	IRCCS Sacro Cuore Don Calabria Hospital, Department of Infectious, Tropical Diseases & Microbiology	University of Verona, Department of Biotechnology	Antonio Mori, Michela Deiana, Elena Pomari, Chiara Piubelli; Giulia Lopatriello, Luca Marcolungo, Cristina Beltrami, Chiara Degli Esposti, Emanuela Cosentino, Massimo Delledonne
EPI_ISL_493197	INT Fondazione Pascale	INT Fondazione Pascale	Pascale
EPI_ISL_493198, EPI_ISL_493199, EPI_ISL_493200, EPI_ISL_493201, EPI_ISL_493202, EPI_ISL_493203, EPI_ISL_493204, EPI_ISL_493205, EPI_ISL_493206, EPI_ISL_493207	Virology Lab,Department of Pathology, National Cheng Kung University Hospital	Virology Lab,Department of Pathology, National Cheng Kung University Hospital	Huey-Pin Tsai, et al
EPI_ISL_493208	Virology Lab,Department of Pathology, National Cheng Kung University Hospital	Virology Lab,Department of Pathology, National Cheng Kung University Hospital	Huey-Pin Tsai et al
EPI_ISL_493213	INT Fondazione Pascale	INT Fondazione Pascale	Pascale
EPI_ISL_493332	Istituto Zooprofilattico Sperimentale del Mezzogiorno	INMI Lazzaro Spallanzani IRCCS	Cesare E.M. Gruber, Martina Rueca, Barbara Bartolini, Francesco Messina, Antonino Di Caro, Giovanna Fusco, Maurizio Viscardi, Giorgia Borriello, Maria R. Capobianchi
EPI_ISL_493333	Istituto Zooprofilattico Sperimentale del Mezzogiorno	INMI Lazzaro Spallanzani IRCCS	Barbara Bartolini, Martina Rueca, Cesare E.M. Gruber, Francesco Messina, Antonino Di Caro, Giovanna Fusco, Maurizio Viscardi, Giorgia Borriello, Maria R. Capobianchi
EPI_ISL_493334, EPI_ISL_493335, EPI_ISL_493336, EPI_ISL_493337, EPI_ISL_493338	Instituto de Diagnostico y Referencia Epidemiologicos (INDRE)	Instituto de Diagnostico y Referencia Epidemiologicos (INDRE)	Gisela Barrera-Badillo , Abril Rodriguez-Maldonado, Claudia Wong-Arambula , Natividad Cruz-Ortiz, Tatiana Nunez-Garcia, Dayanira Arellano-Suarez, Fabiola Garces-Ayala, Edgar Mendieta-Condado, Lucia Hernandez-Rivas, Irma Lopez-Martinez, Ernesto Ramirez-Gonzalez.
EPI_ISL_493339, EPI_ISL_493340, EPI_ISL_493341	Instituto de Diagnostico y Referencia Epidemiologicos (INDRE)	Instituto de Diagnostico y Referencia Epidemiologicos (INDRE)	Gisela Barrera-Badillo , Abril Rodriguez-Maldonado, Claudia Wong-Arambula , Natividad Cruz-Ortiz, Tatiana Nunez-Garcia, Dayanira Arellano-Suarez, Adnan Araiza-Rodriguez, Edgar Mendieta-Condado, Lucia Hernandez-Rivas, Irma Lopez-Martinez, Ernesto Ramirez-Gonzalez.
EPI_ISL_493342, EPI_ISL_493343	Instituto de Diagnostico y Referencia Epidemiologicos (INDRE)	Instituto de Diagnostico y Referencia Epidemiologicos (INDRE)	Ernesto Ramirez-Gonzalez, Abril Rodriguez-Maldonado, Claudia Wong-Arambula , Natividad Cruz-Ortiz, Tatiana Nunez-Garcia, Dayanira Arellano-Suarez, Adnan Araiza-Rodriguez, Edgar Mendieta-Condado, Lucia Hernandez-Rivas, Irma Lopez-Martinez, Gisela Barrera-Badillo.
EPI_ISL_493344, EPI_ISL_493345, EPI_ISL_493346, EPI_ISL_493347, EPI_ISL_493348, EPI_ISL_493349	Instituto de Diagnostico y Referencia Epidemiologicos (INDRE)	Instituto de Diagnostico y Referencia Epidemiologicos (INDRE)	Ernesto Ramirez-Gonzalez, Abril Rodriguez-Maldonado, Claudia Wong-Arambula , Natividad Cruz-Ortiz, Tatiana Nunez-Garcia, Dayanira Arellano-Suarez, Adnan Araiza-Rodriguez, Fabiola Garces-Ayala, Lucia Hernandez-Rivas, Irma Lopez-Martinez, Gisela Barrera-Badillo.
EPI_ISL_494747	Virology Lab,Department of Pathology, National Cheng Kung University Hospital	Virology Lab,Department of Pathology, National Cheng Kung University Hospital	Huey-Pin Tsai, et al
EPI_ISL_494756, EPI_ISL_494757, EPI_ISL_494759, EPI_ISL_494761, EPI_ISL_494762, EPI_ISL_494763, EPI_ISL_494771, EPI_ISL_494774, EPI_ISL_494775, EPI_ISL_494776	INT Fondazione Pascale	INT Fondazione Pascale	INT Fondazione Pascale
EPI_ISL_495014	B.J. Medical College and Civil hospital	Gujarat Biotechnology Research Centre	Janvi Raval, Zarna Patel, Monika Gandhi, Pinal Trivedi, Maharshi Pandya, Nidhi Patel, Nitin Savaliya, Raghawendra Kumar, Dinesh Kumar, Zuber Saiyed, Komal Patel, Labdhi Pandya, Afzal Ansari, Nikha Trivedi, Pranay Shah, Kamlesh J Upadhyay, Sanjay Kapadia, Apurvasinh Puvar, R D Dixit, A M Kadri, Harsh Bakshi, Chaitanya Joshi, Madhvi Joshi
EPI_ISL_495015	B.J. Medical College and Civil hospital	Gujarat Biotechnology Research Centre	Zarna Patel, Monika Gandhi, Pinal Trivedi, Maharshi Pandya, Nidhi Patel, Nitin Savaliya, Raghawendra Kumar, Dinesh Kumar, Zuber Saiyed, Komal Patel, Labdhi Pandya, Afzal Ansari, Nikha Trivedi, Pranay Shah, Kamlesh J Upadhyay, Sanjay Kapadia, Apurvasinh Puvar, Janvi Raval, R D Dixit, A M Kadri, Harsh Bakshi, Chaitanya Joshi, Madhvi Joshi
EPI_ISL_495016	B.J. Medical College and Civil hospital	Gujarat Biotechnology Research Centre	Monika Gandhi, Pinal Trivedi, Maharshi Pandya, Nidhi Patel, Nitin Savaliya, Raghawendra Kumar, Dinesh Kumar, Zuber Saiyed, Komal Patel, Labdhi Pandya, Afzal Ansari, Nikha Trivedi, Pranay Shah, Kamlesh J Upadhyay, Sanjay Kapadia, Apurvasinh Puvar, Janvi Raval, Zarna Patel, R D Dixit, A M Kadri, Harsh Bakshi, Chaitanya Joshi, Madhvi Joshi
EPI_ISL_495017	B.J. Medical College and Civil hospital	Gujarat Biotechnology Research Centre	Pinal Trivedi, Maharshi Pandya, Nidhi Patel, Nitin Savaliya, Raghawendra Kumar, Dinesh Kumar, Zuber Saiyed, Komal Patel, Labdhi Pandya, Afzal Ansari, Nikha Trivedi, Pranay Shah, Kamlesh J Upadhyay, Sanjay Kapadia, Apurvasinh Puvar, Janvi Raval, Zarna Patel, Monika Gandhi, R D Dixit, A M Kadri, Harsh Bakshi, Chaitanya Joshi, Madhvi Joshi
EPI_ISL_495018	B.J. Medical College and Civil hospital	Gujarat Biotechnology Research Centre	Maharshi Pandya, Nidhi Patel, Nitin Savaliya, Raghawendra Kumar, Dinesh Kumar, Zuber Saiyed, Komal Patel, Labdhi Pandya, Afzal Ansari, Nikha Trivedi, Pranay Shah, Kamlesh J Upadhyay, Sanjay Kapadia, Apurvasinh Puvar, Janvi Raval, Zarna Patel, Monika Gandhi, Pinal Trivedi, R D Dixit, A M Kadri, Harsh Bakshi, Chaitanya Joshi, Madhvi Joshi
EPI_ISL_495019	B.J. Medical College and Civil hospital	Gujarat Biotechnology Research Centre	Nidhi Patel, Nitin Savaliya, Raghawendra Kumar, Dinesh Kumar, Zuber Saiyed, Komal Patel, Labdhi Pandya, Afzal Ansari, Nikha Trivedi, Pranay Shah, Kamlesh J Upadhyay, Sanjay Kapadia, Apurvasinh Puvar, Janvi Raval, Zarna Patel, Monika Gandhi, Pinal Trivedi, Maharshi Pandya, R D Dixit, A M Kadri, Harsh Bakshi, Chaitanya Joshi, Madhvi Joshi
EPI_ISL_495020	Government Medical College, Bhavnagar	Gujarat Biotechnology Research Centre	Kairavi Desai, Saklin Malek, Shirish Patel, Nitin Savaliya, Raghawendra Kumar, Dinesh Kumar, Zuber Saiyed, Komal Patel, Labdhi Pandya, Afzal Ansari, Nikha Trivedi, Apurvasinh Puvar, Janvi Raval, Zarna Patel, Monika Gandhi, Pinal Trivedi, Maharshi Pandya, Nidhi Patel, R D Dixit, A M Kadri, Harsh Bakshi, Chaitanya Joshi, Madhvi Joshi

EPI_ISL_495021	Government Medical College, Bhavnagar	Gujarat Biotechnology Research Centre	Saklin Malek, Shirish Patel, Kairavi Desai, Raghawendra Kumar, Dinesh Kumar, Zuber Saiyed, Komal Patel, Labdhi Pandya, Afzal Ansari, Nikha Trivedi, Apurvasinh Puvar, Janvi Raval, Zarna Patel, Monika Gandhi, Pinal Trivedi, Maharshi Pandya, Nidhi Patel, Nitin Savaliya, R D Dixit, A M Kadri, Harsh Bakshi, Chaitanya Joshi, Madhvi Joshi
EPI_ISL_495022	Government Medical College, Bhavnagar	Gujarat Biotechnology Research Centre	Shirish Patel, Kairavi Desai, Saklin Malek, Dinesh Kumar, Zuber Saiyed, Komal Patel, Labdhi Pandya, Afzal Ansari, Nikha Trivedi, Apurvasinh Puvar, Janvi Raval, Zarna Patel, Monika Gandhi, Pinal Trivedi, Maharshi Pandya, Nidhi Patel, Nitin Savaliya, Raghawendra Kumar, R D Dixit, A M Kadri, Harsh Bakshi, Chaitanya Joshi, Madhvi Joshi
EPI_ISL_495023	Government Medical College, Bhavnagar	Gujarat Biotechnology Research Centre	Nitin Savaliya, Raghawendra Kumar, Dinesh Kumar, Zuber Saiyed, Komal Patel, Labdhi Pandya, Afzal Ansari, Nikha Trivedi, Kairavi Desai, Saklin Malek, Shirish Patel, Apurvasinh Puvar, Janvi Raval, Zarna Patel, Monika Gandhi, Pinal Trivedi, Maharshi Pandya, Nidhi Patel, R D Dixit, A M Kadri, Harsh Bakshi, Chaitanya Joshi, Madhvi Joshi
EPI_ISL_495024	Government Medical College, Bhavnagar	Gujarat Biotechnology Research Centre	Raghawendra Kumar, Dinesh Kumar, Zuber Saiyed, Komal Patel, Labdhi Pandya, Afzal Ansari, Nikha Trivedi, Kairavi Desai, Saklin Malek, Shirish Patel, Apurvasinh Puvar, Janvi Raval, Zarna Patel, Monika Gandhi, Pinal Trivedi, Maharshi Pandya, Nidhi Patel, Nitin Savaliya, R D Dixit, A M Kadri, Harsh Bakshi, Chaitanya Joshi, Madhvi Joshi
EPI_ISL_495025	Government Medical College, Bhavnagar	Gujarat Biotechnology Research Centre	Dinesh Kumar, Zuber Saiyed, Komal Patel, Labdhi Pandya, Afzal Ansari, Nikha Trivedi, Kairavi Desai, Saklin Malek, Shirish Patel, Apurvasinh Puvar, Janvi Raval, Zarna Patel, Monika Gandhi, Pinal Trivedi, Maharshi Pandya, Nidhi Patel, Nitin Savaliya, Raghawendra Kumar, R D Dixit, A M Kadri, Harsh Bakshi, Chaitanya Joshi, Madhvi Joshi
EPI_ISL_495026	GMERS Medical College & Hospital, Gotri, Vadodara	Gujarat Biotechnology Research Centre	Komal Patel, Labdhi Pandya, Afzal Ansari, Nikha Trivedi, Meenakshi Shah, Neena Doshi, Varsha Godbole, Apurvasinh Puvar, Janvi Raval, Zarna Patel, Monika Gandhi, Pinal Trivedi, Maharshi Pandya, Nidhi Patel, Nitin Savaliya, Raghawendra Kumar, Dinesh Kumar, Zuber Saiyed, R D Dixit, A M Kadri, Harsh Bakshi, Chaitanya Joshi, Madhvi Joshi
EPI_ISL_495027	GMERS Medical College & Hospital, Gotri, Vadodara	Gujarat Biotechnology Research Centre	Labdhi Pandya, Afzal Ansari, Nikha Trivedi, Meenakshi Shah, Neena Doshi, Varsha Godbole, Apurvasinh Puvar, Janvi Raval, Zarna Patel, Monika Gandhi, Pinal Trivedi, Maharshi Pandya, Nidhi Patel, Nitin Savaliya, Raghawendra Kumar, Dinesh Kumar, Zuber Saiyed, Komal Patel, R D Dixit, A M Kadri, Harsh Bakshi, Chaitanya Joshi, Madhvi Joshi
EPI_ISL_495028	GMERS Medical College & Hospital, Gotri, Vadodara	Gujarat Biotechnology Research Centre	Afzal Ansari, Nikha Trivedi, Meenakshi Shah, Neena Doshi, Varsha Godbole, Apurvasinh Puvar, Janvi Raval, Zarna Patel, Monika Gandhi, Pinal Trivedi, Maharshi Pandya, Nidhi Patel, Nitin Savaliya, Raghawendra Kumar, Dinesh Kumar, Zuber Saiyed, Komal Patel, Labdhi Pandya, R D Dixit, A M Kadri, Harsh Bakshi, Chaitanya Joshi, Madhvi Joshi
EPI_ISL_495029	GMERS Medical College & Hospital, Gotri, Vadodara	Gujarat Biotechnology Research Centre	Nikha Trivedi, Meenakshi Shah, Neena Doshi, Varsha Godbole, Apurvasinh Puvar, Janvi Raval, Zarna Patel, Monika Gandhi, Pinal Trivedi, Maharshi Pandya, Nidhi Patel, Nitin Savaliya, Raghawendra Kumar, Dinesh Kumar, Zuber Saiyed, Komal Patel, Labdhi Pandya, Afzal Ansari, R D Dixit, A M Kadri, Harsh Bakshi, Chaitanya Joshi, Madhvi Joshi
EPI_ISL_495030	GMERS Medical College & Hospital, Gotri, Vadodara	Gujarat Biotechnology Research Centre	Meenakshi Shah, Neena Doshi, Varsha Godbole, Apurvasinh Puvar, Janvi Raval, Zarna Patel, Monika Gandhi, Pinal Trivedi, Maharshi Pandya, Nidhi Patel, Nitin Savaliya, Raghawendra Kumar, Dinesh Kumar, Zuber Saiyed, Komal Patel, Labdhi Pandya, Afzal Ansari, Nikha Trivedi, R D Dixit, A M Kadri, Harsh Bakshi, Chaitanya Joshi, Madhvi Joshi
EPI_ISL_495031	GMERS Medical College & Hospital, Gotri, Vadodara	Gujarat Biotechnology Research Centre	Neena Doshi, Varsha Godbole, Apurvasinh Puvar, Janvi Raval, Zarna Patel, Monika Gandhi, Pinal Trivedi, Maharshi Pandya, Nidhi Patel, Nitin Savaliya, Raghawendra Kumar, Dinesh Kumar, Zuber Saiyed, Komal Patel, Labdhi Pandya, Afzal Ansari, Nikha Trivedi, Meenakshi Shah, R D Dixit, A M Kadri, Harsh Bakshi, Chaitanya Joshi, Madhvi Joshi
EPI_ISL_495032	GMERS Medical College & Hospital, Gotri, Vadodara	Gujarat Biotechnology Research Centre	Varsha Godbole, Apurvasinh Puvar, Janvi Raval, Zarna Patel, Monika Gandhi, Pinal Trivedi, Maharshi Pandya, Nidhi Patel, Nitin Savaliya, Raghawendra Kumar, Dinesh Kumar, Zuber Saiyed, Komal Patel, Labdhi Pandya, Afzal Ansari, Nikha Trivedi, Meenakshi Shah, Neena Doshi, R D Dixit, A M Kadri, Harsh Bakshi, Chaitanya Joshi, Madhvi Joshi
EPI_ISL_495033	GMERS Medical College & Hospital, Gotri, Vadodara	Gujarat Biotechnology Research Centre	Apurvasinh Puvar, Janvi Raval, Zarna Patel, Monika Gandhi, Pinal Trivedi, Maharshi Pandya, Nidhi Patel, Nitin Savaliya, Raghawendra Kumar, Dinesh Kumar, Zuber Saiyed, Komal Patel, Labdhi Pandya, Afzal Ansari, Nikha Trivedi, Meenakshi Shah, Neena Doshi, Varsha Godbole, R D Dixit, A M Kadri, Harsh Bakshi, Chaitanya Joshi, Madhvi Joshi
EPI_ISL_495034	GMERS Medical College & Hospital, Gotri, Vadodara	Gujarat Biotechnology Research Centre	Janvi Raval, Zarna Patel, Monika Gandhi, Pinal Trivedi, Maharshi Pandya, Nidhi Patel, Nitin Savaliya, Raghawendra Kumar, Dinesh Kumar, Zuber Saiyed, Komal Patel, Labdhi Pandya, Afzal Ansari, Nikha Trivedi, Meenakshi Shah, Neena Doshi, Varsha Godbole, Apurvasinh Puvar, R D Dixit, A M Kadri, Harsh Bakshi, Chaitanya Joshi, Madhvi Joshi
EPI_ISL_495035	GMERS Medical College & Hospital, Gotri, Vadodara	Gujarat Biotechnology Research Centre	Zarna Patel, Monika Gandhi, Pinal Trivedi, Maharshi Pandya, Nidhi Patel, Nitin Savaliya, Raghawendra Kumar, Dinesh Kumar, Zuber Saiyed, Komal Patel, Labdhi Pandya, Afzal Ansari, Nikha Trivedi, Meenakshi Shah, Neena Doshi, Varsha Godbole, Apurvasinh Puvar, Janvi Raval, R D Dixit, A M Kadri, Harsh Bakshi, Chaitanya Joshi, Madhvi Joshi
EPI_ISL_495038	GMERS Medical College & Hospital, Gotri, Vadodara	Gujarat Biotechnology Research Centre	Maharshi Pandya, Nidhi Patel, Nitin Savaliya, Raghawendra Kumar, Dinesh Kumar, Zuber Saiyed, Komal Patel, Labdhi Pandya, Afzal Ansari, Nikha Trivedi, Meenakshi Shah, Neena Doshi, Varsha Godbole, Apurvasinh Puvar, Janvi Raval, Zarna Patel, Monika Gandhi, Pinal Trivedi, R D Dixit, A M Kadri, Harsh Bakshi, Chaitanya Joshi, Madhvi Joshi
EPI_ISL_495039	GMERS Medical College & Hospital, Gotri, Vadodara	Gujarat Biotechnology Research Centre	Nidhi Patel, Nitin Savaliya, Raghawendra Kumar, Dinesh Kumar, Zuber Saiyed, Komal Patel, Labdhi Pandya, Afzal Ansari, Nikha Trivedi, Meenakshi Shah, Neena Doshi, Varsha Godbole, Apurvasinh Puvar, Janvi Raval, Zarna Patel, Monika Gandhi, Pinal Trivedi, Maharshi Pandya, R D Dixit, A M Kadri, Harsh Bakshi, Chaitanya Joshi, Madhvi Joshi
EPI_ISL_495042	GMERS Medical College & Hospital, Gotri, Vadodara	Gujarat Biotechnology Research Centre	Dinesh Kumar, Zuber Saiyed, Komal Patel, Labdhi Pandya, Afzal Ansari, Nikha Trivedi, Meenakshi Shah, Neena Doshi, Varsha Godbole, Apurvasinh Puvar, Janvi Raval, Zarna Patel, Monika Gandhi, Pinal Trivedi, Maharshi Pandya, Nidhi Patel, Nitin Savaliya, Raghawendra Kumar, R D Dixit, A M Kadri, Harsh Bakshi, Chaitanya Joshi, Madhvi Joshi
EPI_ISL_495043	GMERS Medical College & Hospital, Gotri, Vadodara	Gujarat Biotechnology Research Centre	Zuber Saiyed, Komal Patel, Labdhi Pandya, Afzal Ansari, Nikha Trivedi, Meenakshi Shah, Neena Doshi, Varsha Godbole, Apurvasinh Puvar, Janvi Raval, Zarna Patel, Monika Gandhi, Pinal Trivedi, Maharshi Pandya, Nidhi Patel, Nitin Savaliya, Raghawendra Kumar, Dinesh Kumar, R D Dixit, A M Kadri, Harsh Bakshi, Chaitanya Joshi, Madhvi Joshi
EPI_ISL_495044	GMERS Medical College & Hospital, Gotri, Vadodara	Gujarat Biotechnology Research Centre	Komal Patel, Labdhi Pandya, Afzal Ansari, Nikha Trivedi, Meenakshi Shah, Neena Doshi, Varsha Godbole, Apurvasinh Puvar, Janvi Raval, Zarna Patel, Monika Gandhi, Pinal Trivedi, Maharshi Pandya, Nidhi Patel, Nitin Savaliya, Raghawendra Kumar, Dinesh Kumar, Zuber Saiyed, R D Dixit, A M Kadri, Harsh Bakshi, Chaitanya Joshi, Madhvi Joshi
EPI_ISL_495045	GMERS Medical College & Hospital, Gotri, Vadodara	Gujarat Biotechnology Research Centre	Labdhi Pandya, Afzal Ansari, Nikha Trivedi, Meenakshi Shah, Neena Doshi, Varsha Godbole, Apurvasinh Puvar, Janvi Raval, Zarna Patel, Monika Gandhi, Pinal Trivedi, Maharshi Pandya, Nidhi Patel, Nitin Savaliya, Raghawendra Kumar, Dinesh Kumar, Zuber Saiyed, Komal Patel, R D Dixit, A M Kadri, Harsh Bakshi, Chaitanya Joshi, Madhvi Joshi
EPI_ISL_495046	GMERS Medical College & Hospital, Gotri, Vadodara	Gujarat Biotechnology Research Centre	Afzal Ansari, Nikha Trivedi, Meenakshi Shah, Neena Doshi, Varsha Godbole, Apurvasinh Puvar, Janvi Raval, Zarna Patel, Monika Gandhi, Pinal Trivedi, Maharshi Pandya, Nidhi Patel, Nitin Savaliya, Raghawendra Kumar, Dinesh Kumar, Zuber Saiyed, Komal Patel, Labdhi Pandya, R D Dixit, A M Kadri, Harsh Bakshi, Chaitanya Joshi, Madhvi Joshi
EPI_ISL_495047	GMERS Medical College & Hospital, Gotri, Vadodara	Gujarat Biotechnology Research Centre	Nikha Trivedi, Meenakshi Shah, Neena Doshi, Varsha Godbole, Apurvasinh Puvar, Janvi Raval, Zarna Patel, Monika Gandhi, Pinal Trivedi, Maharshi Pandya, Nidhi Patel, Nitin Savaliya, Raghawendra Kumar, Dinesh Kumar, Zuber Saiyed, Komal Patel, Labdhi Pandya, Afzal Ansari, R D Dixit, A M Kadri, Harsh Bakshi, Chaitanya Joshi, Madhvi Joshi
EPI_ISL_495048	GMERS Medical College & Hospital, Gotri, Vadodara	Gujarat Biotechnology Research Centre	Meenakshi Shah, Neena Doshi, Varsha Godbole, Apurvasinh Puvar, Janvi Raval, Zarna Patel, Monika Gandhi, Pinal Trivedi, Maharshi Pandya, Nidhi Patel, Nitin Savaliya, Raghawendra Kumar, Dinesh Kumar, Zuber Saiyed, Komal Patel, Labdhi Pandya, Afzal Ansari, Nikha Trivedi, R D Dixit, A M Kadri, Harsh Bakshi, Chaitanya Joshi, Madhvi Joshi
EPI_ISL_495049	GMERS Medical College & Hospital, Gotri, Vadodara	Gujarat Biotechnology Research Centre	Neena Doshi, Varsha Godbole, Apurvasinh Puvar, Janvi Raval, Zarna Patel, Monika Gandhi, Pinal Trivedi, Maharshi Pandya, Nidhi Patel, Nitin Savaliya, Raghawendra Kumar, Dinesh Kumar, Zuber Saiyed, Komal Patel, Labdhi Pandya, Afzal Ansari, Nikha Trivedi, Meenakshi Shah, R D Dixit, A M Kadri, Harsh Bakshi, Chaitanya Joshi, Madhvi Joshi

EPI_ISL_495050	GMERS Medical College & Hospital, Gotri, Vadodara	Gujarat Biotechnology Research Centre	Varsha Godbole, Apurvasinh Puvar, Janvi Raval, Zarna Patel, Monika Gandhi, Pinal Trivedi, Maharshi Pandya, Nidhi Patel, Nitin Savaliya, Raghawendra Kumar, Dinesh Kumar, Zuber Saiyed, Komal Patel, Labdhi Pandya, Afzal Ansari, Nikha Trivedi, Meenakshi Shah, Neena Doshi, R D Dixit, A M Kadri, Harsh Bakshi, Chaitanya Joshi, Madhvi Joshi
EPI_ISL_495051	GMERS Medical College & Hospital, Gotri, Vadodara	Gujarat Biotechnology Research Centre	Apurvasinh Puvar, Janvi Raval, Zarna Patel, Monika Gandhi, Pinal Trivedi, Maharshi Pandya, Nidhi Patel, Nitin Savaliya, Raghawendra Kumar, Dinesh Kumar, Zuber Saiyed, Komal Patel, Labdhi Pandya, Afzal Ansari, Nikha Trivedi, Meenakshi Shah, Neena Doshi, Varsha Godbole, R D Dixit, A M Kadri, Harsh Bakshi, Chaitanya Joshi, Madhvi Joshi
EPI_ISL_495052	GMERS Medical College & Hospital, Gotri, Vadodara	Gujarat Biotechnology Research Centre	Janvi Raval, Zarna Patel, Monika Gandhi, Pinal Trivedi, Maharshi Pandya, Nidhi Patel, Nitin Savaliya, Raghawendra Kumar, Dinesh Kumar, Zuber Saiyed, Komal Patel, Labdhi Pandya, Afzal Ansari, Nikha Trivedi, Meenakshi Shah, Neena Doshi, Varsha Godbole, Apurvasinh Puvar, R D Dixit, A M Kadri, Harsh Bakshi, Chaitanya Joshi, Madhvi Joshi
EPI_ISL_495053	GMERS Medical College & Hospital, Gotri, Vadodara	Gujarat Biotechnology Research Centre	Zarna Patel, Monika Gandhi, Pinal Trivedi, Maharshi Pandya, Nidhi Patel, Nitin Savaliya, Raghawendra Kumar, Dinesh Kumar, Zuber Saiyed, Komal Patel, Labdhi Pandya, Afzal Ansari, Nikha Trivedi, Meenakshi Shah, Neena Doshi, Varsha Godbole, Apurvasinh Puvar, Janvi Raval, R D Dixit, A M Kadri, Harsh Bakshi, Chaitanya Joshi, Madhvi Joshi
EPI_ISL_495054	GMERS Medical College & Hospital, Gotri, Vadodara	Gujarat Biotechnology Research Centre	Monika Gandhi, Pinal Trivedi, Maharshi Pandya, Nidhi Patel, Nitin Savaliya, Raghawendra Kumar, Dinesh Kumar, Zuber Saiyed, Komal Patel, Labdhi Pandya, Afzal Ansari, Nikha Trivedi, Meenakshi Shah, Neena Doshi, Varsha Godbole, Apurvasinh Puvar, Janvi Raval, Zarna Patel, R D Dixit, A M Kadri, Harsh Bakshi, Chaitanya Joshi, Madhvi Joshi
EPI_ISL_495055	GMERS Medical College & Hospital, Gotri, Vadodara	Gujarat Biotechnology Research Centre	Pinal Trivedi, Maharshi Pandya, Nidhi Patel, Nitin Savaliya, Raghawendra Kumar, Dinesh Kumar, Zuber Saiyed, Komal Patel, Labdhi Pandya, Afzal Ansari, Nikha Trivedi, Meenakshi Shah, Neena Doshi, Varsha Godbole, Apurvasinh Puvar, Janvi Raval, Zarna Patel, Monika Gandhi, R D Dixit, A M Kadri, Harsh Bakshi, Chaitanya Joshi, Madhvi Joshi
EPI_ISL_495056	GMERS Medical College & Hospital, Gotri, Vadodara	Gujarat Biotechnology Research Centre	Maharshi Pandya, Nidhi Patel, Nitin Savaliya, Raghawendra Kumar, Dinesh Kumar, Zuber Saiyed, Komal Patel, Labdhi Pandya, Afzal Ansari, Nikha Trivedi, Meenakshi Shah, Neena Doshi, Varsha Godbole, Apurvasinh Puvar, Janvi Raval, Zarna Patel, Monika Gandhi, Pinal Trivedi, R D Dixit, A M Kadri, Harsh Bakshi, Chaitanya Joshi, Madhvi Joshi
EPI_ISL_495057	GMERS Medical College & Hospital, Gotri, Vadodara	Gujarat Biotechnology Research Centre	Nidhi Patel, Nitin Savaliya, Raghawendra Kumar, Dinesh Kumar, Zuber Saiyed, Komal Patel, Labdhi Pandya, Afzal Ansari, Nikha Trivedi, Meenakshi Shah, Neena Doshi, Varsha Godbole, Apurvasinh Puvar, Janvi Raval, Zarna Patel, Monika Gandhi, Pinal Trivedi, Maharshi Pandya, R D Dixit, A M Kadri, Harsh Bakshi, Chaitanya Joshi, Madhvi Joshi
EPI_ISL_495058	GMERS Medical College & Hospital, Gotri, Vadodara	Gujarat Biotechnology Research Centre	Nitin Savaliya, Raghawendra Kumar, Dinesh Kumar, Zuber Saiyed, Komal Patel, Labdhi Pandya, Afzal Ansari, Nikha Trivedi, Meenakshi Shah, Neena Doshi, Varsha Godbole, Apurvasinh Puvar, Janvi Raval, Zarna Patel, Monika Gandhi, Pinal Trivedi, Maharshi Pandya, Nidhi Patel, R D Dixit, A M Kadri, Harsh Bakshi, Chaitanya Joshi, Madhvi Joshi
EPI_ISL_495059	GMERS Medical College & Hospital, Gotri, Vadodara	Gujarat Biotechnology Research Centre	Raghawendra Kumar, Dinesh Kumar, Zuber Saiyed, Komal Patel, Labdhi Pandya, Afzal Ansari, Nikha Trivedi, Meenakshi Shah, Neena Doshi, Varsha Godbole, Apurvasinh Puvar, Janvi Raval, Zarna Patel, Monika Gandhi, Pinal Trivedi, Maharshi Pandya, Nidhi Patel, Nitin Savaliya, R D Dixit, A M Kadri, Harsh Bakshi, Chaitanya Joshi, Madhvi Joshi
EPI_ISL_495060	GMERS Medical College & Hospital, Gotri, Vadodara	Gujarat Biotechnology Research Centre	Dinesh Kumar, Zuber Saiyed, Komal Patel, Labdhi Pandya, Afzal Ansari, Nikha Trivedi, Meenakshi Shah, Neena Doshi, Varsha Godbole, Apurvasinh Puvar, Janvi Raval, Zarna Patel, Monika Gandhi, Pinal Trivedi, Maharshi Pandya, Nidhi Patel, Nitin Savaliya, Raghawendra Kumar, R D Dixit, A M Kadri, Harsh Bakshi, Chaitanya Joshi, Madhvi Joshi
EPI_ISL_495061	GMERS Medical College & Hospital, Gotri, Vadodara	Gujarat Biotechnology Research Centre	Zuber Saiyed, Komal Patel, Labdhi Pandya, Afzal Ansari, Nikha Trivedi, Meenakshi Shah, Neena Doshi, Varsha Godbole, Apurvasinh Puvar, Janvi Raval, Zarna Patel, Monika Gandhi, Pinal Trivedi, Maharshi Pandya, Nidhi Patel, Nitin Savaliya, Raghawendra Kumar, Dinesh Kumar, R D Dixit, A M Kadri, Harsh Bakshi, Chaitanya Joshi, Madhvi Joshi
EPI_ISL_495062	GMERS Medical College & Hospital, Gotri, Vadodara	Gujarat Biotechnology Research Centre	Komal Patel, Labdhi Pandya, Afzal Ansari, Nikha Trivedi, Meenakshi Shah, Neena Doshi, Varsha Godbole, Apurvasinh Puvar, Janvi Raval, Zarna Patel, Monika Gandhi, Pinal Trivedi, Maharshi Pandya, Nidhi Patel, Nitin Savaliya, Raghawendra Kumar, Dinesh Kumar, Zuber Saiyed, R D Dixit, A M Kadri, Harsh Bakshi, Chaitanya Joshi, Madhvi Joshi
EPI_ISL_495063	GMERS Medical College & Hospital, Himmatnagar	Gujarat Biotechnology Research Centre	Labdhi Pandya, Afzal Ansari, Nikha Trivedi, Himanshu Khatri, Mayur Gandhi, Apurvasinh Puvar, Janvi Raval, Zarna Patel, Monika Gandhi, Pinal Trivedi, Maharshi Pandya, Nidhi Patel, Nitin Savaliya, Raghawendra Kumar, Dinesh Kumar, Zuber Saiyed, Komal Patel, R D Dixit, A M Kadri, Harsh Bakshi, Chaitanya Joshi, Madhvi Joshi,
EPI_ISL_495064	GMERS Medical College & Hospital, Himmatnagar	Gujarat Biotechnology Research Centre	Afzal Ansari, Nikha Trivedi, Himanshu Khatri, Mayur Gandhi, Apurvasinh Puvar, Janvi Raval, Zarna Patel, Monika Gandhi, Pinal Trivedi, Maharshi Pandya, Nidhi Patel, Nitin Savaliya, Raghawendra Kumar, Dinesh Kumar, Zuber Saiyed, Komal Patel, Labdhi Pandya, R D Dixit, A M Kadri, Harsh Bakshi, Chaitanya Joshi, Madhvi Joshi,
EPI_ISL_495065	GAIMS & G K General Hospital	Gujarat Biotechnology Research Centre	Nikha Trivedi, Babulal Babhoria, Hitesh Assudani, Apurvasinh Puvar, Janvi Raval, Zarna Patel, Monika Gandhi, Pinal Trivedi, Maharshi Pandya, Nidhi Patel, Nitin Savaliya, Raghawendra Kumar, Dinesh Kumar, Zuber Saiyed, Komal Patel, Labdhi Pandya, Afzal Ansari, R D Dixit, A M Kadri, Harsh Bakshi, Chaitanya Joshi, Madhvi Joshi,
EPI_ISL_495066	GAIMS & G K General Hospital	Gujarat Biotechnology Research Centre	Babulal Babhoria, Hitesh Assudani, Apurvasinh Puvar, Janvi Raval, Zarna Patel, Monika Gandhi, Pinal Trivedi, Maharshi Pandya, Nidhi Patel, Nitin Savaliya, Raghawendra Kumar, Dinesh Kumar, Zuber Saiyed, Komal Patel, Labdhi Pandya, Afzal Ansari, Nikha Trivedi, R D Dixit, A M Kadri, Harsh Bakshi, Chaitanya Joshi, Madhvi Joshi,
EPI_ISL_495067	Dr. N. D. Desai Medical College & Hospital	Gujarat Biotechnology Research Centre	J G Buch, Jigar Gusani, Supreet Prabhu, Apurvasinh Puvar, Janvi Raval, Zarna Patel, Monika Gandhi, Pinal Trivedi, Maharshi Pandya, Nidhi Patel, Nitin Savaliya, Raghawendra Kumar, Dinesh Kumar, Zuber Saiyed, Komal Patel, Labdhi Pandya, Afzal Ansari, Nikha Trivedi, R D Dixit, A M Kadri, Harsh Bakshi, Chaitanya Joshi, Madhvi Joshi
EPI_ISL_495068	Dr. N. D. Desai Medical College & Hospital	Gujarat Biotechnology Research Centre	Jigar Gusani, Supreet Prabhu, Apurvasinh Puvar, Janvi Raval, Zarna Patel, Monika Gandhi, Pinal Trivedi, Maharshi Pandya, Nidhi Patel, Nitin Savaliya, Raghawendra Kumar, Dinesh Kumar, Zuber Saiyed, Komal Patel, Labdhi Pandya, Afzal Ansari, Nikha Trivedi, J G Buch, R D Dixit, A M Kadri, Harsh Bakshi, Chaitanya Joshi, Madhvi Joshi
EPI_ISL_495069	Dr. N. D. Desai Medical College & Hospital	Gujarat Biotechnology Research Centre	Supreet Prabhu, Apurvasinh Puvar, Janvi Raval, Zarna Patel, Monika Gandhi, Pinal Trivedi, Maharshi Pandya, Nidhi Patel, Nitin Savaliya, Raghawendra Kumar, Dinesh Kumar, Zuber Saiyed, Komal Patel, Labdhi Pandya, Afzal Ansari, Nikha Trivedi, J G Buch, Jigar Gusani, R D Dixit, A M Kadri, Harsh Bakshi, Chaitanya Joshi, Madhvi Joshi
EPI_ISL_495070	Department of MicroBiology, Government Medical College, Surat	Gujarat Biotechnology Research Centre	Apurvasinh Puvar, Janvi Raval, Zarna Patel, Monika Gandhi, Pinal Trivedi, Maharshi Pandya, Nidhi Patel, Nitin Savaliya, Raghawendra Kumar, Dinesh Kumar, Zuber Saiyed, Komal Patel, Labdhi Pandya, Afzal Ansari, Nikha Trivedi, Naresh Chauhan, Summaiya Mullan, Amit gamit, R D Dixit, A M Kadri, Harsh Bakshi, Chaitanya Joshi, Madhvi Joshi
EPI_ISL_495071	Department of MicroBiology, Government Medical College, Surat	Gujarat Biotechnology Research Centre	Janvi Raval, Zarna Patel, Monika Gandhi, Pinal Trivedi, Maharshi Pandya, Nidhi Patel, Nitin Savaliya, Raghawendra Kumar, Dinesh Kumar, Zuber Saiyed, Komal Patel, Labdhi Pandya, Afzal Ansari, Nikha Trivedi, Naresh Chauhan, Summaiya Mullan, Amit gamit, Apurvasinh Puvar, R D Dixit, A M Kadri, Harsh Bakshi, Chaitanya Joshi, Madhvi Joshi
EPI_ISL_495072	Department of MicroBiology, Government Medical College, Surat	Gujarat Biotechnology Research Centre	Zarna Patel, Monika Gandhi, Pinal Trivedi, Maharshi Pandya, Nidhi Patel, Nitin Savaliya, Raghawendra Kumar, Dinesh Kumar, Zuber Saiyed, Komal Patel, Labdhi Pandya, Afzal Ansari, Nikha Trivedi, Naresh Chauhan, Summaiya Mullan, Amit gamit, Apurvasinh Puvar, Janvi Raval, R D Dixit, A M Kadri, Harsh Bakshi, Chaitanya Joshi, Madhvi Joshi
EPI_ISL_495073	Department of MicroBiology, Government Medical College, Surat	Gujarat Biotechnology Research Centre	Monika Gandhi, Pinal Trivedi, Maharshi Pandya, Nidhi Patel, Nitin Savaliya, Raghawendra Kumar, Dinesh Kumar, Zuber Saiyed, Komal Patel, Labdhi Pandya, Afzal Ansari, Nikha Trivedi, Naresh Chauhan, Summaiya Mullan, Amit gamit, Apurvasinh Puvar, Janvi Raval, Zarna Patel, R D Dixit, A M Kadri, Harsh Bakshi, Chaitanya Joshi, Madhvi Joshi
EPI_ISL_495074	Department of MicroBiology, Government Medical College, Surat	Gujarat Biotechnology Research Centre	Pinal Trivedi, Maharshi Pandya, Nidhi Patel, Nitin Savaliya, Raghawendra Kumar, Dinesh Kumar, Zuber Saiyed, Komal Patel, Labdhi Pandya, Afzal Ansari, Nikha Trivedi, Naresh Chauhan, Summaiya Mullan, Amit gamit, Apurvasinh Puvar, Janvi Raval, Zarna Patel, Monika Gandhi, R D Dixit, A M Kadri, Harsh Bakshi, Chaitanya Joshi, Madhvi Joshi

EPI_ISL_495075	Department of MicroBiology, Government Medical College, Surat	Gujarat Biotechnology Research Centre	Nidhi Patel, Nitin Savaliya, Raghawendra Kumar, Dinesh Kumar, Zuber Saiyed, Komal Patel, Labdhi Pandya, Afzal Ansari, Nikha Trivedi, Naresh Chauhan, Summaiya Mullan, Amit gamit, Apurvasinh Puvar, Janvi Raval, Zarna Patel, Monika Gandhi, Pinal Trivedi, Maharshi Pandya, R D Dixit, A M Kadri, Harsh Bakshi, Chaitanya Joshi, Madhvi Joshi
EPI_ISL_495076	Department of MicroBiology, Government Medical College, Surat	Gujarat Biotechnology Research Centre	Nitin Savaliya, Raghawendra Kumar, Dinesh Kumar, Zuber Saiyed, Komal Patel, Labdhi Pandya, Afzal Ansari, Nikha Trivedi, Naresh Chauhan, Summaiya Mullan, Amit gamit, Apurvasinh Puvar, Janvi Raval, Zarna Patel, Monika Gandhi, Pinal Trivedi, Maharshi Pandya, Nidhi Patel, R D Dixit, A M Kadri, Harsh Bakshi, Chaitanya Joshi, Madhvi Joshi
EPI_ISL_495077	Department of MicroBiology, Government Medical College, Surat	Gujarat Biotechnology Research Centre	Raghawendra Kumar, Dinesh Kumar, Zuber Saiyed, Komal Patel, Labdhi Pandya, Afzal Ansari, Nikha Trivedi, Naresh Chauhan, Summaiya Mullan, Amit gamit, Apurvasinh Puvar, Janvi Raval, Zarna Patel, Monika Gandhi, Pinal Trivedi, Maharshi Pandya, Nidhi Patel, Nitin Savaliya, R D Dixit, A M Kadri, Harsh Bakshi, Chaitanya Joshi, Madhvi Joshi
EPI_ISL_495078	Department of MicroBiology, Government Medical College, Surat	Gujarat Biotechnology Research Centre	Dinesh Kumar, Zuber Saiyed, Komal Patel, Labdhi Pandya, Afzal Ansari, Nikha Trivedi, Naresh Chauhan, Summaiya Mullan, Amit gamit, Apurvasinh Puvar, Janvi Raval, Zarna Patel, Monika Gandhi, Pinal Trivedi, Maharshi Pandya, Nidhi Patel, Nitin Savaliya, Raghawendra Kumar, R D Dixit, A M Kadri, Harsh Bakshi, Chaitanya Joshi, Madhvi Joshi
EPI_ISL_495079	Department of MicroBiology, Government Medical College, Surat	Gujarat Biotechnology Research Centre	Afzal Ansari, Nikha Trivedi, Naresh Chauhan, Summaiya Mullan, Amit gamit, Apurvasinh Puvar, Janvi Raval, Zarna Patel, Monika Gandhi, Pinal Trivedi, Maharshi Pandya, Nidhi Patel, Nitin Savaliya, Raghawendra Kumar, Dinesh Kumar, Zuber Saiyed, Komal Patel, Labdhi Pandya, R D Dixit, A M Kadri, Harsh Bakshi, Chaitanya Joshi, Madhvi Joshi
EPI_ISL_495080	Department of MicroBiology, Government Medical College, Surat	Gujarat Biotechnology Research Centre	Nikha Trivedi, Naresh Chauhan, Summaiya Mullan, Amit gamit, Apurvasinh Puvar, Janvi Raval, Zarna Patel, Monika Gandhi, Pinal Trivedi, Maharshi Pandya, Nidhi Patel, Nitin Savaliya, Raghawendra Kumar, Dinesh Kumar, Zuber Saiyed, Komal Patel, Labdhi Pandya, Afzal Ansari, R D Dixit, A M Kadri, Harsh Bakshi, Chaitanya Joshi, Madhvi Joshi
EPI_ISL_495411, EPI_ISL_495412, EPI_ISL_495414, EPI_ISL_495415, EPI_ISL_495416, EPI_ISL_495417, EPI_ISL_495418, EPI_ISL_495419, EPI_ISL_495420, EPI_ISL_495421, EPI_ISL_495422, EPI_ISL_495423, EPI_ISL_495424, EPI_ISL_495425, EPI_ISL_495426, EPI_ISL_495427, EPI_ISL_495428, EPI_ISL_495429, EPI_ISL_495431, EPI_ISL_495432, EPI_ISL_495433, EPI_ISL_495434, EPI_ISL_495435, EPI_ISL_495436, EPI_ISL_495437, EPI_ISL_495438, EPI_ISL_495439, EPI_ISL_495440, EPI_ISL_495442, EPI_ISL_495443, EPI_ISL_495444, EPI_ISL_495445, EPI_ISL_495446, EPI_ISL_495447, EPI_ISL_495448, EPI_ISL_495449, EPI_ISL_495450, EPI_ISL_495451, EPI_ISL_495452, EPI_ISL_495453, EPI_ISL_495454, EPI_ISL_495455, EPI_ISL_495456, EPI_ISL_495457			
see above	Kafkas University, Faculty of Medicine, Department of Medical Microbiology	Kafkas University, Faculty of Medicine, Department of Medical Microbiology	Murat Karamese, Didem Ozgur, E. Ediz Tutuncu
EPI_ISL_495459	Centers for Disease Control and Prevention of Lishui	Department of InspectionCenters for Disease Control and Prevention of Lishui	Wang Xiaoguang,Ji Qiaoying,Ji Jiansong,Ye Bifeng,Ye Ling
EPI_ISL_495516, EPI_ISL_495517, EPI_ISL_495518, EPI_ISL_495519, EPI_ISL_495520, EPI_ISL_495521, EPI_ISL_495522, EPI_ISL_495523, EPI_ISL_495524, EPI_ISL_495525, EPI_ISL_495526, EPI_ISL_495527, EPI_ISL_495528, EPI_ISL_495529, EPI_ISL_495530, EPI_ISL_495531, EPI_ISL_495532, EPI_ISL_495533, EPI_ISL_495534			
see above	NHLS-IALCH	KRISP, KZN Research Innovation and Sequencing Platform	Giandhari J, Pillay S, Lessells R, Chimukangara B, Mdlalose K, York D, Khan S, Tegally H, Wilkinson E, de Oliveira T
EPI_ISL_495535, EPI_ISL_495536, EPI_ISL_495537, EPI_ISL_495538, EPI_ISL_495539, EPI_ISL_495540, EPI_ISL_495541, EPI_ISL_495542	Medical Disagnostics Services (MDS)	KRISP, KZN Research Innovation and Sequencing Platform	Giandhari J, Pillay S, Lessells R, Chimukangara B, Mdlalose K, York D, Khan S, Tegally H, Wilkinson E, de Oliveira T
EPI_ISL_495543, EPI_ISL_495544, EPI_ISL_495545, EPI_ISL_495546, EPI_ISL_495547, EPI_ISL_495548, EPI_ISL_495549, EPI_ISL_495550, EPI_ISL_495551, EPI_ISL_495552, EPI_ISL_495553, EPI_ISL_495554, EPI_ISL_495555, EPI_ISL_495556, EPI_ISL_495557, EPI_ISL_495558, EPI_ISL_495559, EPI_ISL_495560, EPI_ISL_495561, EPI_ISL_495562			
see above	NHLS-IALCH	KRISP, KZN Research Innovation and Sequencing Platform	Giandhari J, Pillay S, Lessells R, Chimukangara B, Mdlalose K, York D, Khan S, Tegally H, Wilkinson E, de Oliveira T
EPI_ISL_496520, EPI_ISL_496529, EPI_ISL_496533	Armed Forces Medical College	National Centre For Cell Science	Dhiraj Paul, Kunal Jani, Radha Chauhan, Janesh Kumar, Vasudevan Seshadri, Girdhari Lal, Rajesh Karyakarte, Suvarna Joshi, Murlidhar Tambe, Sourav Sen, Santosh Karade, Kavita Bala Anand, Shelinder Pal Singh Shergill, Rajiv Mohan Gupta, Manoj Kumar Bhat, Arvind Sahu, Maharashtra COVID-19 Study Group, DBT's PAN-INDIA 1000 SARS-CoV2 RNA genome sequencing consortium, Yogesh S Shouche
EPI_ISL_496534	B.J. Govt. Medical College	National Centre For Cell Science	Dhiraj Paul, Kunal Jani, Radha Chauhan, Janesh Kumar, Vasudevan Seshadri, Girdhari Lal, Rajesh Karyakarte, Suvarna Joshi, Murlidhar Tambe, Sourav Sen, Santosh Karade, Kavita Bala Anand, Shelinder Pal Singh Shergill, Rajiv Mohan Gupta, Manoj Kumar Bhat, Arvind Sahu, Maharashtra COVID-19 Study Group, DBT's PAN-INDIA 1000 SARS-CoV2 RNA genome sequencing consortium, Yogesh S Shouche
EPI_ISL_496537, EPI_ISL_496540, EPI_ISL_496541, EPI_ISL_496542, EPI_ISL_496543, EPI_ISL_496544, EPI_ISL_496545	Armed Forces Medical College	National Centre For Cell Science	Dhiraj Paul, Kunal Jani, Radha Chauhan, Janesh Kumar, Vasudevan Seshadri, Girdhari Lal, Rajesh Karyakarte, Suvarna Joshi, Murlidhar Tambe, Sourav Sen, Santosh Karade, Kavita Bala Anand, Shelinder Pal Singh Shergill, Rajiv Mohan Gupta, Manoj Kumar Bhat, Arvind Sahu, Maharashtra COVID-19 Study Group, DBT's PAN-INDIA 1000 SARS-CoV2 RNA genome sequencing consortium, Yogesh S Shouche
EPI_ISL_496548, EPI_ISL_496549, EPI_ISL_496551	B.J. Govt. Medical College	National Centre For Cell Science	Dhiraj Paul, Kunal Jani, Radha Chauhan, Janesh Kumar, Vasudevan Seshadri, Girdhari Lal, Rajesh Karyakarte, Suvarna Joshi, Murlidhar Tambe, Sourav Sen, Santosh Karade, Kavita Bala Anand, Shelinder Pal Singh Shergill, Rajiv Mohan Gupta, Manoj Kumar Bhat, Arvind Sahu, Maharashtra COVID-19 Study Group, DBT's PAN-INDIA 1000 SARS-CoV2 RNA genome sequencing consortium, Yogesh S Shouche
EPI_ISL_496555, EPI_ISL_496556, EPI_ISL_496557, EPI_ISL_496559, EPI_ISL_496560, EPI_ISL_496571, EPI_ISL_496581, EPI_ISL_496587	National Centre For Cell Science	National Centre For Cell Science	Dhiraj Paul, Kunal Jani, Radha Chauhan, Janesh Kumar, Vasudevan Seshadri, Girdhari Lal, Rajesh Karyakarte, Suvarna Joshi, Murlidhar Tambe, Sourav Sen, Santosh Karade, Kavita Bala Anand, Shelinder Pal Singh Shergill, Rajiv Mohan Gupta, Manoj Kumar Bhat, Arvind Sahu, Maharashtra COVID-19 Study Group, DBT's PAN-INDIA 1000 SARS-CoV2 RNA genome sequencing consortium, Yogesh S Shouche
EPI_ISL_496603, EPI_ISL_496604, EPI_ISL_496605, EPI_ISL_496606, EPI_ISL_496607, EPI_ISL_496608, EPI_ISL_496609, EPI_ISL_496610, EPI_ISL_496611, EPI_ISL_496612, EPI_ISL_496613, EPI_ISL_496614, EPI_ISL_496615, EPI_ISL_496616, EPI_ISL_496617, EPI_ISL_496618, EPI_ISL_496619, EPI_ISL_496621, EPI_ISL_496624, EPI_ISL_496625, EPI_ISL_496626, EPI_ISL_496627, EPI_ISL_496629, EPI_ISL_496630, EPI_ISL_496631, EPI_ISL_496632, EPI_ISL_496633, EPI_ISL_496634, EPI_ISL_496636, EPI_ISL_496637, EPI_ISL_496638, EPI_ISL_496640, EPI_ISL_496641, EPI_ISL_496642, EPI_ISL_496643, EPI_ISL_496644, EPI_ISL_496645, EPI_ISL_496646, EPI_ISL_496647, EPI_ISL_496648, EPI_ISL_496649, EPI_ISL_496650, EPI_ISL_496651, EPI_ISL_496652, EPI_ISL_496653, EPI_ISL_496654, EPI_ISL_496655, EPI_ISL_496657, EPI_ISL_496658, EPI_ISL_496659, EPI_ISL_496660, EPI_ISL_496663, EPI_ISL_496664, EPI_ISL_496665, EPI_ISL_496666, EPI_ISL_496669, EPI_ISL_496670, EPI_ISL_496671, EPI_ISL_496675, EPI_ISL_496676, EPI_ISL_496677, EPI_ISL_496678, EPI_ISL_496679, EPI_ISL_496680, EPI_ISL_496681, EPI_ISL_496682, EPI_ISL_496683, EPI_ISL_496684, EPI_ISL_496685, EPI_ISL_496687, EPI_ISL_496688, EPI_ISL_496689, EPI_ISL_496692, EPI_ISL_496693, EPI_ISL_496694, EPI_ISL_496695, EPI_ISL_496699, EPI_ISL_496700, EPI_ISL_496701, EPI_ISL_496702, EPI_ISL_496703, EPI_ISL_496705, EPI_ISL_496706, EPI_ISL_496708, EPI_ISL_496709, EPI_ISL_496710, EPI_ISL_496711, EPI_ISL_496712, EPI_ISL_496713, EPI_ISL_496714, EPI_ISL_496715, EPI_ISL_496716, EPI_ISL_496718, EPI_ISL_496719, EPI_ISL_496721, EPI_ISL_496722, EPI_ISL_496723, EPI_ISL_496724, EPI_ISL_496725, EPI_ISL_496726, EPI_ISL_496727, EPI_ISL_496728, EPI_ISL_496730, EPI_ISL_496731, EPI_ISL_496732, EPI_ISL_496733, EPI_ISL_496734, EPI_ISL_496735, EPI_ISL_496736, EPI_ISL_496737, EPI_ISL_496740, EPI_ISL_496741, EPI_ISL_496742, EPI_ISL_496743, EPI_ISL_496744, EPI_ISL_496745, EPI_ISL_496746, EPI_ISL_496748, EPI_ISL_496749, EPI_ISL_496750, EPI_ISL_496751, EPI_ISL_496753, EPI_ISL_496756, EPI_ISL_496758, EPI_ISL_496762, EPI_ISL_496763, EPI_ISL_496764, EPI_ISL_496766, EPI_ISL_496767, EPI_ISL_496768, EPI_ISL_496770, EPI_ISL_496771, EPI_ISL_496772, EPI_ISL_496773, EPI_ISL_496774, EPI_ISL_496775, EPI_ISL_496776, EPI_ISL_496777, EPI_ISL_496778, EPI_ISL_496780, EPI_ISL_496781, EPI_ISL_496782, EPI_ISL_496783, EPI_ISL_496784, EPI_ISL_496785, EPI_ISL_496786, EPI_ISL_496787, EPI_ISL_496788, EPI_ISL_496789, EPI_ISL_496790, EPI_ISL_496791, EPI_ISL_496792, EPI_ISL_496793, EPI_ISL_496794, EPI_ISL_496795, EPI_ISL_496796, EPI_ISL_496797, EPI_ISL_496798, EPI_ISL_496799, EPI_ISL_496800, EPI_ISL_496801, EPI_ISL_496802, EPI_ISL_496803, EPI_ISL_496804, EPI_ISL_496805, EPI_ISL_496806, EPI_ISL_496809, EPI_ISL_496810, EPI_ISL_496811, EPI_ISL_496813, EPI_ISL_496814, EPI_ISL_496815, EPI_ISL_496817, EPI_ISL_496818, EPI_ISL_496819, EPI_ISL_496821, EPI_ISL_496822, EPI_ISL_496823, EPI_ISL_496824, EPI_ISL_496825, EPI_ISL_496826, EPI_ISL_496827, EPI_ISL_496828, EPI_ISL_496829, EPI_ISL_496830, EPI_ISL_496831, EPI_ISL_496832, EPI_ISL_496833, EPI_ISL_496834, EPI_ISL_496835, EPI_ISL_496836, EPI_ISL_496837, EPI_ISL_496838, EPI_ISL_496840, EPI_ISL_496841, EPI_ISL_496842, EPI_ISL_496843, EPI_ISL_496844, EPI_ISL_496846, EPI_ISL_496847, EPI_ISL_496848, EPI_ISL_496850, EPI_ISL_496851, EPI_ISL_496852, EPI_ISL_496853, EPI_ISL_496854, EPI_ISL_496855, EPI_ISL_496856, EPI_ISL_496857, EPI_ISL_496859, EPI_ISL_496860, EPI_ISL_496861, EPI_ISL_496862, EPI_ISL_496863, EPI_ISL_496865, EPI_ISL_496867, EPI_ISL_496868, EPI_ISL_496870, EPI_ISL_496871, EPI_ISL_496873, EPI_ISL_496874, EPI_ISL_496875, EPI_ISL_496876, EPI_ISL_496877, EPI_ISL_496878, EPI_ISL_496879, EPI_ISL_496880, EPI_ISL_496881, EPI_ISL_496884, EPI_ISL_496885, EPI_ISL_496886, EPI_ISL_496887, EPI_ISL_496888, EPI_ISL_496889, EPI_ISL_496890, EPI_ISL_496891, EPI_ISL_496892, EPI_ISL_496893, EPI_ISL_496894, EPI_ISL_496895, EPI_ISL_496896, EPI_ISL_496897, EPI_ISL_496898, EPI_ISL_496899, EPI_ISL_496900, EPI_ISL_496901, EPI_ISL_496903, EPI_ISL_496904, EPI_ISL_496906, EPI_ISL_496907, EPI_ISL_496908, EPI_ISL_496909, EPI_ISL_496911, EPI_ISL_496914, EPI_ISL_496915			
see above	Gorgas Memorial Laboratory of Health Studies	Gorgas Memorial Laboratory of Health Studies	Danilo Franco, Claudia Gonzalez Sandra Lopez-Verges, Alexander A Martinez
EPI_ISL_497736, EPI_ISL_497738, EPI_ISL_497744, EPI_ISL_497745	Instituto Nacional de Salud, Bogotá, Colombia	Instituto Nacional de Salud, Bogotá, Colombia	Katherine Laiton-Donato, Diego A. Álvarez-Díaz, Carlos Franco-Muñoz, Jonathan Reales, Diego Andrés Prada, Jose A. Usme-Ciro, Nicolas D. Franco-Sierra, Zulma M. Cucunubá, Christian Julian VillabonaArenas, Liz Villabona-Arenas, Sussy Echeverria, Astrid C. Flórez, Carolina Ferro, Diana Marcela Walteros-Acero, Franklin Prieto, Carlos Andrés Durán, Martha Lucia Ospina Martinez, Marcela Mercado-Reyes
EPI_ISL_497758, EPI_ISL_497760	CSIR-CDRI/SGPGI, Lucknow	CSIr-CDRI/SGPGI, Lucknow	Saumya Sarkar, Dharam Veer Singh, Rahul Vishvkarma, Ujjala Ghoshal, Uday Ghoshal, Ravishankar Ramachandran, Tapas Kumar Kundu, Rajender Singh
EPI_ISL_497762, EPI_ISL_497763	CSIR-CDRI/SGPGI, Lucknow	CSIR-CDRI/SGPGI, Lucknow	Saumya Sarkar, Dharam Veer Singh, Rahul Vishvkarma, Ujjala Ghoshal, Uday Ghoshal, Ravishankar Ramachandran, Tapas Kumar Kundu, Rajender Singh

EPI_ISL_497764	CSIR-CDRi/SGPGI, Lucknow	CSIR-CDRI/SGPGI, Lucknow	Saumya Sarkar, Dharam Veer Singh, Rahul Vishvkarma, Ujjala Ghoshal, Uday Ghoshal, Ravishankar Ramachandran, Tapas Kumar Kundu, Rajender Singh
EPI_ISL_497765	CSIR-CDRI/SGPGI, Lucknow	CSIR-CDRI/SGPGI, Lucknow	Saumya Sarkar, Dharam Veer Singh, Rahul Vishvkarma, Ujjala Ghoshal, Uday Ghoshal, Ravishankar Ramachandran, Tapas Kumar Kundu, Rajender Singh
EPI_ISL_497766	CSIR-CDRI/SGPGI, Lucknow	CSIR-CDRi/SGPGI, Lucknow	Saumya Sarkar, Dharam Veer Singh, Rahul Vishvkarma, Ujjala Ghoshal, Uday Ghoshal, Ravishankar Ramachandran, Tapas Kumar Kundu, Rajender Singh
EPI_ISL_497767	CSIR-CDRI/SGPGI, Lucknow	CSIR-CDRI/SGPGI, Lucknow	Saumya Sarkar, Dharam Veer Singh, Rahul Vishvkarma, Ujjala Ghoshal, Uday Ghoshal, Ravishankar Ramachandran, Tapas Kumar Kundu, Rajender Singh
EPI_ISL_497875, EPI_ISL_497876, EPI_ISL_497878, EPI_ISL_497879	National Centre For Cell Science	National Centre For Cell Science	Dhiraj Paul, Kunal Jani, Radha Chauhan, Janesh Kumar, Vasudevan Seshadri, Girdhari Lal, Rajesh Karyakarte, Suvarna Joshi, Murlidhar Tambe, Sourav Sen, Santosh Karade, Kavita Bala Anand, Shelinder Pal Singh Shergill, Rajiv Mohan Gupta, Manoj Kumar Bhat, Arvind Sahu, Maharashtra COVID-19 Study Group, DBT's PAN-INDIA 1000 SARS-CoV2 RNA genome sequencing consortium, Yogesh S Shouche
EPI_ISL_497880, EPI_ISL_497881, EPI_ISL_497882, EPI_ISL_497883, EPI_ISL_497884, EPI_ISL_497885, EPI_ISL_497886, EPI_ISL_497887	Armed Forces Medical College	National Centre For Cell Science	Dhiraj Paul, Kunal Jani, Radha Chauhan, Janesh Kumar, Vasudevan Seshadri, Girdhari Lal, Rajesh Karyakarte, Suvarna Joshi, Murlidhar Tambe, Sourav Sen, Santosh Karade, Kavita Bala Anand, Shelinder Pal Singh Shergill, Rajiv Mohan Gupta, Manoj Kumar Bhat, Arvind Sahu, Maharashtra COVID-19 Study Group, DBT's PAN-INDIA 1000 SARS-CoV2 RNA genome sequencing consortium, Yogesh S Shouche
EPI_ISL_497888, EPI_ISL_497891	B.J. Govt. Medical College	National Centre For Cell Science	Dhiraj Paul, Kunal Jani, Radha Chauhan, Janesh Kumar, Vasudevan Seshadri, Girdhari Lal, Rajesh Karyakarte, Suvarna Joshi, Murlidhar Tambe, Sourav Sen, Santosh Karade, Kavita Bala Anand, Shelinder Pal Singh Shergill, Rajiv Mohan Gupta, Manoj Kumar Bhat, Arvind Sahu, Maharashtra COVID-19 Study Group, DBT's PAN-INDIA 1000 SARS-CoV2 RNA genome sequencing consortium, Yogesh S Shouche
EPI_ISL_497950	Shaoxing CDC	Zhejiang Provincial Center for Disease Control and Prevention	Yin Chen, Yanjun Zhang, Haiyan Mao, Junhang Pan, Xiuyu Lou, Yi Sun, Hao Yan, Zhen Li, Wen Shi
EPI_ISL_498054, EPI_ISL_498055, EPI_ISL_498056, EPI_ISL_498057, EPI_ISL_498058, EPI_ISL_498059, EPI_ISL_498060, EPI_ISL_498061, EPI_ISL_498062, EPI_ISL_498063, EPI_ISL_498064, EPI_ISL_498065, EPI_ISL_498066, EPI_ISL_498067, EPI_ISL_498068, EPI_ISL_498069, EPI_ISL_498070, EPI_ISL_498071, EPI_ISL_498072, EPI_ISL_498073, EPI_ISL_498074, EPI_ISL_498075, EPI_ISL_498076, EPI_ISL_498077, EPI_ISL_498078, EPI_ISL_498079, EPI_ISL_498080, EPI_ISL_498081, EPI_ISL_498082, EPI_ISL_498083, EPI_ISL_498084, EPI_ISL_498085, EPI_ISL_498086, EPI_ISL_498087, EPI_ISL_498088, EPI_ISL_498089, EPI_ISL_498090, EPI_ISL_498091, EPI_ISL_498092, EPI_ISL_498093, EPI_ISL_498094, EPI_ISL_498095, EPI_ISL_498096, EPI_ISL_498097, EPI_ISL_498098, EPI_ISL_498099, EPI_ISL_498100, EPI_ISL_498101, EPI_ISL_498102, EPI_ISL_498103, EPI_ISL_498104, EPI_ISL_498105, EPI_ISL_498106, EPI_ISL_498107, EPI_ISL_498108, EPI_ISL_498109, EPI_ISL_498110, EPI_ISL_498111, EPI_ISL_498112, EPI_ISL_498113, EPI_ISL_498114, EPI_ISL_498115, EPI_ISL_498116, EPI_ISL_498117, EPI_ISL_498118, EPI_ISL_498119, EPI_ISL_498120, EPI_ISL_498121, EPI_ISL_498122, EPI_ISL_498123, EPI_ISL_498124, EPI_ISL_498125, EPI_ISL_498126			
see above	NHLS-IALCH	KRISP, KZN Research Innovation and Sequencing Platform	Giandhari J, Pillay S, Lessells R, Chimukangara B, Mdlalose K, York D, Khan S, Tegally H, Wilkinson E, de Oliveira T
EPI_ISL_498152, EPI_ISL_498153, EPI_ISL_498154, EPI_ISL_498155, EPI_ISL_498156, EPI_ISL_498157, EPI_ISL_498158, EPI_ISL_498159, EPI_ISL_498160, EPI_ISL_498161, EPI_ISL_498162, EPI_ISL_498163, EPI_ISL_498164, EPI_ISL_498165, EPI_ISL_498166, EPI_ISL_498167, EPI_ISL_498168, EPI_ISL_498169, EPI_ISL_498170			
see above	Instituto Nacional de Salud, Bogotá, Colombia	Instituto Nacional de Salud, Bogotá, Colombia	Katherine Laiton-Donato, Diego A. Álvarez-Díaz, Carlos Franco-Muñoz, Jonathan Reales, Diego Andrés Prada, Jose A. Usme-Ciro, Nicolas D. Franco-Sierra, Zulma M. Cucunubá, Christian Julian VillabonaArenas, Liz Villabona-Arenas, Sussy Echeverria, Astrid C. Flórez, Carolina Ferro, Diana Marcela Walteros-Acero, Franklin Prieto, Carlos Andrés Durán, Martha Lucia Ospina Martinez, Marcela Mercado-Reyes
EPI_ISL_498171, EPI_ISL_498172, EPI_ISL_498173, EPI_ISL_498174, EPI_ISL_498175, EPI_ISL_498176, EPI_ISL_498177, EPI_ISL_498178, EPI_ISL_498179, EPI_ISL_498180, EPI_ISL_498181, EPI_ISL_498182, EPI_ISL_498183, EPI_ISL_498184, EPI_ISL_498185, EPI_ISL_498186, EPI_ISL_498187, EPI_ISL_498188, EPI_ISL_498189, EPI_ISL_498190, EPI_ISL_498191, EPI_ISL_498192			
see above	OUCRU	OUCRU	Nguyen Van Vinh Chau, Nguyen Thi Thu Hong, Nguyen Thi Han Ny, Le Nguyen Truc Nhu, Nghiem My Ngoc, Vo Thanh Lam, Nguyen Thanh Dung, Lam Minh Yen, Ngo Ngoc Quang Minh, Le Manh Hung, Nguyen Tri Dung, Dinh Nguyen Huy Man, Lam Anh Nguyet, Tran Chanh Xuan, Tran Tinh Hien, Nguyen Thanh Phong, Tran Nguyen Hoang Tu, Tran Tan Thanh, Nguyen Thanh Truong, Nguyen Tan Binh, Tang Chi Thuong, Guy Thwaites, and Le Van Tan, for OUCRU COVID-19 research group*
EPI_ISL_498229, EPI_ISL_498230, EPI_ISL_498231, EPI_ISL_498232, EPI_ISL_498233, EPI_ISL_498234, EPI_ISL_498235, EPI_ISL_498236, EPI_ISL_498237, EPI_ISL_498238, EPI_ISL_498239, EPI_ISL_498240, EPI_ISL_498241, EPI_ISL_498242, EPI_ISL_498243, EPI_ISL_498244, EPI_ISL_498245, EPI_ISL_498246, EPI_ISL_498247, EPI_ISL_498248, EPI_ISL_498249, EPI_ISL_498250, EPI_ISL_498251, EPI_ISL_498252			
see above	Institut Pasteur de Dakar	Institut Pasteur de Dakar	Ndongo Dia, Moussa Moise Diagne, Mamadou Diop, Marie Henriette Dior Ndione, Mamadou Malado Jallow, Safietou Sankhe Mbengue, Ousmane Faye, Amadou Alpha Sall.
EPI_ISL_498551, EPI_ISL_498552, EPI_ISL_498554, EPI_ISL_498556	Lebanese American University	Lebanese American University	Abi Habib,W., Abdallah,J., El Shesheny,R., Mokhbat,J., Webby,R.J., Goldstein,J. and Kayali,G.
EPI_ISL_498558, EPI_ISL_498559, EPI_ISL_498560, EPI_ISL_498561, EPI_ISL_498562, EPI_ISL_498563	Laboratory of Molecular Virology International Center for Genetic Engineering and Biotechnology (ICGEB)	ARGO Open Lab Platform for Genome Sequencing	Licastro D, Rajasekharan S, Dal Monego S, Segat L, D'Agaro P, Marcello A
EPI_ISL_499083	Instituto de Virologia "Dr. J. M. Vanella", Facultad de Ciencias Medicas, Universidad Nacional de Cordoba. Laboratorio Central de la Provincia de Cordoba, Argentina. Ministerio de Salud de la provincia de Cordoba, Argentina.	Laboratorio de Virología, Hospital de Niños Ricardo Gutiérrez, CABA, Argentina.	Sandra Gallego, Brenda Konigheim, Sebastian Blanco, Lorena Spinsanti, Javier Aguilar, Adrian Diaz, Gonzalo Castro, Gabriela Barbas, Mercedes Nabaes, Stephanie Goya, Monica Natale, Silvina Lusso, Mariana Viegas.
EPI_ISL_500539, EPI_ISL_500540, EPI_ISL_500541, EPI_ISL_500542, EPI_ISL_500543, EPI_ISL_500544, EPI_ISL_500545, EPI_ISL_500546, EPI_ISL_500547, EPI_ISL_500548, EPI_ISL_500549, EPI_ISL_500550, EPI_ISL_500551, EPI_ISL_500552, EPI_ISL_500553, EPI_ISL_500554, EPI_ISL_500555, EPI_ISL_500556, EPI_ISL_500557, EPI_ISL_500558, EPI_ISL_500559, EPI_ISL_500560, EPI_ISL_500561, EPI_ISL_500562, EPI_ISL_500563, EPI_ISL_500564, EPI_ISL_500565, EPI_ISL_500566, EPI_ISL_500567, EPI_ISL_500568, EPI_ISL_500569, EPI_ISL_500570, EPI_ISL_500571, EPI_ISL_500572			
see above	Singapore General Hospital	Department of Microbiology	Nurdyana Abdul Rahman, Kun Lee Lim, Chenhao Li, Kian Sing Chan, Lynette Oon, Kern Rei Chng, Niranjan Nagarajan, Karrie Ko
EPI_ISL_500716	BSL3 Lab Pendik Veterinary Control Institute	Department of Medicinal Genetics, Bursa Uluda University, Faculty of medicine By Sehime Gülsün Temel, Adem Alemdar, Kadir Yeilba	Mustafa HASOKSUZ, Fahriye SARAC, Osman ERGANIS, Serdar UZAR, Hakan ENUL, Cumhur ADIAY, Ahmet SAIT, Orbay SAYI, Kadir YESILBAG, Oguz KARABEY
EPI_ISL_500864	Instituto de Pesquisas Biomédicas, Hospital Naval Marcílio Dias	Bioinformatics Laboratory / LNCC	Andressa Rangel de Oliveira Lima, Luiz Gonzaga Paula de Almeida, Alexandra Lehmkuhl Gerber, Marlon Daniel Lima Tonin, Mônica Gomes Monteiro da R. Santos, Claudio Alberto Mule Monteiro, Átila Duque Rossi, Isabel de Medeiros Magalhaes de As, Luana Ferreira Martins de Toledo, Amilcar Tanuri, Albina Luciana da Silva Freitas, Fernanda Conceição Silva do Amorim, Shana Priscila Coutinho Barroso, Cynthia Chester Cardoso, Carolina Moreira Voloch, Ana Tereza Vasconcelos
EPI_ISL_500946	GMERS Medical College & Hospital, Gotri, Vadodara	Gujarat Biotechnology Research Centre	Zuber Saiyed, Komal Patel, Labdhi Pandya, Afzal Ansari, Nikha Trivedi, Meenakshi Shah, Neena Doshi, Varsha Godbole, Apurvasinh Puvar, Janvi Raval, Zarna Patel, Monika Gandhi, Pinal Trivedi, Maharshi Pandya, Nidhi Patel, Nitin Savaliya, Raghawendra Kumar, Dinesh Kumar, R D Dixit, A M Kadri, Harsh Bakshi, Chaitanya Joshi, Madhvi Joshi
EPI_ISL_500947	Department of MicroBiology, Government Medical College, Surat	Gujarat Biotechnology Research Centre	Maharshi Pandya, Nidhi Patel, Nitin Savaliya, Raghawendra Kumar, Dinesh Kumar, Zuber Saiyed, Komal Patel, Labdhi Pandya, Afzal Ansari, Nikha Trivedi, Naresh Chauhan, Summaiya Mullan, Amit gamit, Apurvasinh Puvar, Janvi Raval, Zarna Patel, Monika Gandhi, Pinal Trivedi, R D Dixit, A M Kadri, Harsh Bakshi, Chaitanya Joshi, Madhvi Joshi
EPI_ISL_500948	Department of MicroBiology, Government Medical College, Surat	Gujarat Biotechnology Research Centre	Zuber Saiyed, Komal Patel, Labdhi Pandya, Afzal Ansari, Nikha Trivedi, Naresh Chauhan, Summaiya Mullan, Amit gamit, Apurvasinh Puvar, Janvi Raval, Zarna Patel, Monika Gandhi, Pinal Trivedi, Maharshi Pandya, Nidhi Patel, Nitin Savaliya, Raghawendra Kumar, Dinesh Kumar, R D Dixit, A M Kadri, Harsh Bakshi, Chaitanya Joshi, Madhvi Joshi
EPI_ISL_500949	Department of MicroBiology, Government Medical College, Surat	Gujarat Biotechnology Research Centre	Komal Patel, Labdhi Pandya, Afzal Ansari, Nikha Trivedi, Naresh Chauhan, Summaiya Mullan, Amit gamit, Apurvasinh Puvar, Janvi Raval, Zarna Patel, Monika Gandhi, Pinal Trivedi, Maharshi Pandya, Nidhi Patel, Nitin Savaliya, Raghawendra Kumar, Dinesh Kumar, Zuber Saiyed, R D Dixit, A M Kadri, Harsh Bakshi, Chaitanya Joshi, Madhvi Joshi
EPI_ISL_500950	Department of MicroBiology, Government Medical College, Surat	Gujarat Biotechnology Research Centre	Labdhi Pandya, Afzal Ansari, Nikha Trivedi, Naresh Chauhan, Summaiya Mullan, Amit gamit, Apurvasinh Puvar, Janvi Raval, Zarna Patel, Monika Gandhi, Pinal Trivedi, Maharshi Pandya, Nidhi Patel, Nitin Savaliya, Raghawendra Kumar, Dinesh Kumar, Zuber Saiyed, Komal Patel, R D Dixit, A M Kadri, Harsh

			Bakshi, Chaitanya Joshi, Madhvi Joshi
EPI_ISL_501167, EPI_ISL_501168, EPI_ISL_501169, EPI_ISL_501170, EPI_ISL_501171, EPI_ISL_501172, EPI_ISL_501173, EPI_ISL_501174	Baylor College of Medicine	Baylor College of Medicine: HGSC	Vasanthi Avadhanula, Erin Nicholson, David Henke, Pedro Piedra, Harsha Doddapaneni, Donna Muzny, Qingchang Meng, Hsu Chao, Zeineen Momin, Hua Shen, George Weissenberger, Kavya Kottapalli, Yimiti Meiheerguli, Sejal Salvi, Ginger Metcalf, Vipin Menon, Sara J.J. Cregeen, Matthew C. Ross, Tulin Ayvaz, Richard Sucgang, Kristi L. Hoffman, Matthew Wong, Joseph F. Petrosino
EPI_ISL_501176, EPI_ISL_501177, EPI_ISL_501178, EPI_ISL_501179, EPI_ISL_501180, EPI_ISL_501181, EPI_ISL_501182, EPI_ISL_501183, EPI_ISL_501184, EPI_ISL_501185, EPI_ISL_501186, EPI_ISL_501187, EPI_ISL_501188, EPI_ISL_501189, EPI_ISL_501190, EPI_ISL_501191, EPI_ISL_501192, EPI_ISL_501193, EPI_ISL_501194, EPI_ISL_501195, EPI_ISL_501196, EPI_ISL_501197, EPI_ISL_501198, EPI_ISL_501199, EPI_ISL_501200, EPI_ISL_501201, EPI_ISL_501202, EPI_ISL_501203, EPI_ISL_501204, EPI_ISL_501205, EPI_ISL_501206, EPI_ISL_501207, EPI_ISL_501208, EPI_ISL_501209, EPI_ISL_501210, EPI_ISL_501211, EPI_ISL_501212, EPI_ISL_501213, EPI_ISL_501214, EPI_ISL_501215, EPI_ISL_501216, EPI_ISL_501217, EPI_ISL_501218, EPI_ISL_501219, EPI_ISL_501220, EPI_ISL_501221, EPI_ISL_501222, EPI_ISL_501223, EPI_ISL_501224, EPI_ISL_501225, EPI_ISL_501226, EPI_ISL_501227, EPI_ISL_501228			
see above	Department of Medical Microbiology, University Malaya Medical Centre	Department of Medical Microbiology, Faculty of Medicine, University of Malaya	Yoong Min CHONG, Jennifer Chong, I-Ching SAM, Yoke Fun CHAN, University Malaya Medical Centre COVID Team
EPI_ISL_501230	Hellenic Pasteur Institute, Public Health Laboratories	Hellenic Pasteur Institute, National Influenza Reference laboratory of Southern Greece & Unit of Bioinformatics and Applied Genomics	Vasiliki Pogka, Timokratis Karamitros, Athanasios Kossyvakis, Antonios Kalliaropoulos, Horefti Elina, Evangelidou Maria, Androniki Voulgari-Kokota, Aspasia Kontou, Andreas Mentis
EPI_ISL_501231, EPI_ISL_501232, EPI_ISL_501233, EPI_ISL_501234, EPI_ISL_501235, EPI_ISL_501236, EPI_ISL_501237, EPI_ISL_501238, EPI_ISL_501241, EPI_ISL_501246, EPI_ISL_501248, EPI_ISL_501249, EPI_ISL_501250, EPI_ISL_501251			
see above	Hellenic Pasteur Institute, National Influenza Reference laboratory of Southern Greece & Unit of Bioinformatics and Applied Genomics	Hellenic Pasteur Institute, National Influenza Reference laboratory of Southern Greece & Unit of Bioinformatics and Applied Genomics	Vasiliki Pogka, Timokratis Karamitros, Athanasios Kossyvakis, Antonios Kalliaropoulos, Horefti Elina, Evangelidou Maria, Androniki Voulgari-Kokota, Aspasia Kontou, Andreas Mentis
EPI_ISL_505003	Biology Dpt	Microbiology and Infections Diseases	Emmanuelle Billon-Denis, Audrey Ferrier-Rembert, Annabelle Garnier, Laurence Cheutin, Clarisse Vigne, Emilie Tessier, Jessica Denis, Olivier Gorgé, Flora Nolent, Isabelle Drouet, Noémie Verguet, Olivier Ferraris, Jean-Nicolas Tournier
EPI_ISL_506041	Biology Dpt, HIA Percy	Microbiology and Infectious Diseases Dpt	Emmanuelle Billon-Denis, Audrey Ferrier-Rembert, Annabelle Garnier, Laurence Cheutin, Clarisse Vigne, Emilie Tessier, Jessica Denis, Olivier Gorgé, Flora Nolent, Isabelle Drouet, Noémie Verguet, Olivier Ferraris, Jean-Nicolas Tournier
EPI_ISL_506996, EPI_ISL_506997, EPI_ISL_506998, EPI_ISL_506999, EPI_ISL_507000	Department of Medical Microbiology, University Malaya Medical Centre	Department of Medical Microbiology, Faculty of Medicine, University of Malaya	Yoong Min CHONG, Jennifer Chong, I-Ching SAM, Yoke Fun CHAN, University Malaya Medical Centre COVID Team
EPI_ISL_507002, EPI_ISL_507003, EPI_ISL_507004, EPI_ISL_507005, EPI_ISL_507006	Department of Laboratory Medicine, Tan Tock Seng Hospital	Department of Laboratory Medicine, Tan Tock Seng Hospital	Chen YYC, Zair X, Li C, Tang WY, Maurer-Stroh S, Barkham TMS, Nagarajan N, Sessions OM
EPI_ISL_507008	National Veterinary Institute	National Veterinary Institute	Siamak Zohari
EPI_ISL_507206, EPI_ISL_507207, EPI_ISL_507208, EPI_ISL_507209, EPI_ISL_507210, EPI_ISL_507211, EPI_ISL_507212, EPI_ISL_507213, EPI_ISL_507214, EPI_ISL_507215	Department of Experimental Modeling and Pathogenesis of Infectious Diseases	WHO National Influenza Centre Russian Federation	Andrey Komissarov, Artem Fadeev, Mariia Sergeeva, Anna Ivanova, Daria Danilenko
EPI_ISL_507225, EPI_ISL_507226, EPI_ISL_507261, EPI_ISL_507262, EPI_ISL_507263, EPI_ISL_507264, EPI_ISL_507274, EPI_ISL_507279, EPI_ISL_507291, EPI_ISL_507292, EPI_ISL_507293, EPI_ISL_507294			
see above	WHO National Influenza Centre Russian Federation	WHO National Influenza Centre Russian Federation	Andrey Komissarov, Artem Fadeev, Mariia Sergeeva, Anna Ivanova, Daria Danilenko
EPI_ISL_508862, EPI_ISL_508863	Virology Unit, Institut Pasteur de Madagascar	Virology Unit, Institut Pasteur de Madagascar	Christian Ranaivoson, Cara Brook, Norosoa Razanajatovo, Vida Ahyong, Tsiry Randriambolamanantsoa, Michelle Tan, Vololoniaina Raharinosy, Helisoa Razafimanjato, Cristina M. Tato, Joseph L. DeRisi, Soa Fy Andriamandimby, Jean-Michel Heraud
EPI_ISL_509223, EPI_ISL_509224, EPI_ISL_509225, EPI_ISL_509226, EPI_ISL_509227, EPI_ISL_509228, EPI_ISL_509229, EPI_ISL_509230, EPI_ISL_509231, EPI_ISL_509232, EPI_ISL_509233, EPI_ISL_509234, EPI_ISL_509235, EPI_ISL_509236, EPI_ISL_509237, EPI_ISL_509238, EPI_ISL_509239, EPI_ISL_509240, EPI_ISL_509241, EPI_ISL_509242, EPI_ISL_509243, EPI_ISL_509244, EPI_ISL_509245, EPI_ISL_509246, EPI_ISL_509247, EPI_ISL_509248, EPI_ISL_509249, EPI_ISL_509250, EPI_ISL_509251, EPI_ISL_509252, EPI_ISL_509253, EPI_ISL_509254, EPI_ISL_509255, EPI_ISL_509256, EPI_ISL_509257, EPI_ISL_509258, EPI_ISL_509259, EPI_ISL_509260, EPI_ISL_509261, EPI_ISL_509262, EPI_ISL_509263, EPI_ISL_509264, EPI_ISL_509265, EPI_ISL_509266, EPI_ISL_509267, EPI_ISL_509268, EPI_ISL_509269, EPI_ISL_509270, EPI_ISL_509271, EPI_ISL_509272, EPI_ISL_509273, EPI_ISL_509274, EPI_ISL_509275, EPI_ISL_509276, EPI_ISL_509277, EPI_ISL_509278, EPI_ISL_509279, EPI_ISL_509280, EPI_ISL_509281, EPI_ISL_509282, EPI_ISL_509283, EPI_ISL_509284, EPI_ISL_509285, EPI_ISL_509286, EPI_ISL_509287, EPI_ISL_509288, EPI_ISL_509289, EPI_ISL_509290, EPI_ISL_509291, EPI_ISL_509292, EPI_ISL_509293, EPI_ISL_509294, EPI_ISL_509295, EPI_ISL_509296, EPI_ISL_509297, EPI_ISL_509298, EPI_ISL_509299, EPI_ISL_509300, EPI_ISL_509301, EPI_ISL_509302, EPI_ISL_509303, EPI_ISL_509304, EPI_ISL_509305, EPI_ISL_509306, EPI_ISL_509307, EPI_ISL_509308, EPI_ISL_509309, EPI_ISL_509310, EPI_ISL_509311, EPI_ISL_509312, EPI_ISL_509313, EPI_ISL_509314, EPI_ISL_509315, EPI_ISL_509316, EPI_ISL_509317, EPI_ISL_509318, EPI_ISL_509319, EPI_ISL_509320, EPI_ISL_509321, EPI_ISL_509322, EPI_ISL_509323, EPI_ISL_509324, EPI_ISL_509325, EPI_ISL_509326, EPI_ISL_509327, EPI_ISL_509328, EPI_ISL_509329, EPI_ISL_509330, EPI_ISL_509331, EPI_ISL_509332, EPI_ISL_509333, EPI_ISL_509334, EPI_ISL_509335, EPI_ISL_509336, EPI_ISL_509337, EPI_ISL_509338, EPI_ISL_509339, EPI_ISL_509340, EPI_ISL_509341, EPI_ISL_509342, EPI_ISL_509343, EPI_ISL_509344, EPI_ISL_509345, EPI_ISL_509346, EPI_ISL_509347, EPI_ISL_509348, EPI_ISL_509349, EPI_ISL_509350, EPI_ISL_509351, EPI_ISL_509352, EPI_ISL_509353, EPI_ISL_509354, EPI_ISL_509355, EPI_ISL_509356, EPI_ISL_509357, EPI_ISL_509358, EPI_ISL_509359, EPI_ISL_509360, EPI_ISL_509361, EPI_ISL_509362, EPI_ISL_509363, EPI_ISL_509364, EPI_ISL_509365, EPI_ISL_509366, EPI_ISL_509367, EPI_ISL_509368, EPI_ISL_509369, EPI_ISL_509370, EPI_ISL_509371			
see above	NHLS-IALCH	KRISP, KZN Research Innovation and Sequencing Platform	Giandhari J, Pillay S, Lessells R, Mdlalose K, York D, Tegally H, Wilkinson E, de Oliveira T
EPI_ISL_509372, EPI_ISL_509373, EPI_ISL_509374, EPI_ISL_509375, EPI_ISL_509376, EPI_ISL_509377, EPI_ISL_509378, EPI_ISL_509379, EPI_ISL_509380, EPI_ISL_509381, EPI_ISL_509382, EPI_ISL_509383, EPI_ISL_509384, EPI_ISL_509385, EPI_ISL_509386, EPI_ISL_509387, EPI_ISL_509388, EPI_ISL_509389, EPI_ISL_509390, EPI_ISL_509391, EPI_ISL_509392, EPI_ISL_509393, EPI_ISL_509394, EPI_ISL_509395			
see above	Singapore General Hospital	Department of Microbiology	Nurdyana Abdul Rahman, Kun Lee Lim, Chenhao Li, Kian Sing Chan, Lynette Oon, Kern Rei Chng, Niranjan Nagarajan, Karrie Ko
EPI_ISL_509412, EPI_ISL_509413, EPI_ISL_509414, EPI_ISL_509415, EPI_ISL_509416, EPI_ISL_509417	Acibadem Labcell Cellular Therapy Laboratory	Acibadem Mehmet Ali Aydinlar University School of Medicine, Medical Genetics Department	Ozden Hatirnaz Ng, Sezer Akyoney, Ilayda Sahin, Gunseli Bayram Akcapinar, Ozkan Ozdemir, Derya Dilek Kancagi, Gozde Sir Karakus, Bulut Yurtsever, Cihan Tastan, Ercument Ovali, Ugur Ozbek
EPI_ISL_510689	Hospital Universitari Germans Trias i Pujol(HUGTiP)/Fundació Lluita contra la SIDA (FLSida)/IRTA-CReSA	IrsiCaixa AIDS Research Lab	Pilar Armengol, Marc Noguera-Julian, Jordi Rodón, Julia Vergara, Lidia Ruiz, Nuria Izquierdo, Jorge Carrillo, Roger Paredes, Albert Bensaid, Julia Blanco, Joaquim Segalés, Bonaventura Clotet
EPI_ISL_511878	Santosa Hospital Bandung Kopo	Laboratorium Kesehatan Provinsi Jawa Barat; Molecular Genetics Laboratory-Faculty of Medicine-Universitas Padjadjaran; School of Life Sciences and Technology & School of Pharmacy-Institut Teknologi Bandung	Ema Rahmawati, Marselina Irasonia Tan, Yunia Sribudiani, Catur Riani, Azzania Fibriani, Husna Nugrahapraja, Tarwadi, Hesti Lina Wiraswati, Lia Faridah, Savira Ekawardhani, Ryan Bayusantika Ristandi, Rifky Waluyajati Rachman, Cut Nur Cinthia Alamanda, Hammam Riza, Soni Solistia Wirawan, Agung Eru Wibowo
EPI_ISL_511879	Laboratorium Kesehatan Provinsi Jawa Barat	Molecular Genetics Laboratory-Faculty of Medicine-Universitas Padjadjaran; School of Life Sciences and Technology & School of Pharmacy-Institut Teknologi Bandung; Laboratorium Kesehatan Provinsi Jawa Barat	Marselina Irasonia Tan, Yunia Sribudiani, Catur Riani, Azzania Fibriani, Husna Nugrahapraja, Tarwadi, Ema Rahmawati, Hesti Lina Wiraswati, Lia Faridah, Savira Ekawardhani, Ryan Bayusantika Ristandi, Rifky Waluyajati Rachman, Cut Nur Cinthia Alamanda, Hammam Riza, Soni Solistia Wirawan, Agung Eru Wibowo
EPI_ISL_511891, EPI_ISL_511892, EPI_ISL_511893, EPI_ISL_511894, EPI_ISL_511895, EPI_ISL_511896, EPI_ISL_511897, EPI_ISL_511898	National Hospital of Tropical Diseases	Oxford University Clinical Research Unit, Hanoi, Vietnam	Nguyen Thi Tam, Van Dinh Trang, Nguyen Thi Hong Thuong, Vu Thi Ngoc Bich, Nguyen Thu Trang, Nguyen Thi Ngoc Diep, Le Nguyen Minh Hoa, Pham Ngoc Thach, H. Rogier van Doorn, on behalf of the OUCRU COVID-19 research group
EPI_ISL_512058	B.J. Medical College and Civil hospital, Ahmedabad	Gujarat Biotechnology Research Centre	Monika Gandhi, Pinal Trivedi, Maharshi Pandya, Nidhi Patel, Nitin Savaliya, Raghawendra Kumar, Dinesh Kumar, Zuber Saiyed, Komal Patel, Labdhi Pandya, Afzal Ansari, Nikha Trivedi, Pranay Shah, Kamlesh J Upadhyay, Sanjay Kapadia, Apurvasinh Puvar, Janvi Raval, Zarna Patel, R D Dixit, A M Kadri, Harsh Bakshi, Chaitanya Joshi, Madhvi Joshi
EPI_ISL_512059	B.J. Medical College and Civil hospital, Ahmedabad	Gujarat Biotechnology Research Centre	Pinal Trivedi, Maharshi Pandya, Nidhi Patel, Nitin Savaliya, Raghawendra Kumar, Dinesh Kumar, Zuber Saiyed, Komal Patel, Labdhi Pandya, Afzal Ansari, Nikha Trivedi, Pranay Shah, Kamlesh J Upadhyay, Sanjay Kapadia, Apurvasinh Puvar, Janvi Raval, Zarna Patel, Monika Gandhi, R D Dixit, A M Kadri, Harsh Bakshi, Chaitanya Joshi, Madhvi Joshi
EPI_ISL_512060	B.J. Medical College and Civil hospital, Ahmedabad	Gujarat Biotechnology Research Centre	Maharshi Pandya, Nidhi Patel, Nitin Savaliya, Raghawendra Kumar, Dinesh Kumar, Zuber Saiyed, Komal Patel, Labdhi Pandya, Afzal Ansari, Nikha Trivedi,

			Pranay Shah, Kamlesh J Upadhyay, Sanjay Kapadia, Apurvasinh Puvar, Janvi Raval, Zarna Patel, Monika Gandhi, Pinal Trivedi, R D Dixit, A M Kadri, Harsh Bakshi, Chaitanya Joshi, Madhvi Joshi
EPI_ISL_512061	B.J. Medical College and Civil hospital, Ahmedabad	Gujarat Biotechnology Research Centre	Nidhi Patel, Nitin Savaliya, Raghawendra Kumar, Dinesh Kumar, Zuber Saiyed, Komal Patel, Labdhi Pandya, Afzal Ansari, Nikha Trivedi, Pranay Shah, Kamlesh J Upadhyay, Sanjay Kapadia, Apurvasinh Puvar, Janvi Raval, Zarna Patel, Monika Gandhi, Pinal Trivedi, Maharshi Pandya, R D Dixit, A M Kadri, Harsh Bakshi, Chaitanya Joshi, Madhvi Joshi
EPI_ISL_512062	B.J. Medical College and Civil hospital, Ahmedabad	Gujarat Biotechnology Research Centre	Nitin Savaliya, Raghawendra Kumar, Dinesh Kumar, Zuber Saiyed, Komal Patel, Labdhi Pandya, Afzal Ansari, Nikha Trivedi, Pranay Shah, Kamlesh J Upadhyay, Sanjay Kapadia, Apurvasinh Puvar, Janvi Raval, Zarna Patel, Monika Gandhi, Pinal Trivedi, Maharshi Pandya, Nidhi Patel, R D Dixit, A M Kadri, Harsh Bakshi, Chaitanya Joshi, Madhvi Joshi
EPI_ISL_512063	B.J. Medical College and Civil hospital, Ahmedabad	Gujarat Biotechnology Research Centre	Raghawendra Kumar, Dinesh Kumar, Zuber Saiyed, Komal Patel, Labdhi Pandya, Afzal Ansari, Nikha Trivedi, Pranay Shah, Kamlesh J Upadhyay, Sanjay Kapadia, Apurvasinh Puvar, Janvi Raval, Zarna Patel, Monika Gandhi, Pinal Trivedi, Maharshi Pandya, Nidhi Patel, Nitin Savaliya, R D Dixit, A M Kadri, Harsh Bakshi, Chaitanya Joshi, Madhvi Joshi
EPI_ISL_512064	B.J. Medical College and Civil hospital, Ahmedabad	Gujarat Biotechnology Research Centre	Dinesh Kumar, Zuber Saiyed, Komal Patel, Labdhi Pandya, Afzal Ansari, Nikha Trivedi, Pranay Shah, Kamlesh J Upadhyay, Sanjay Kapadia, Apurvasinh Puvar, Janvi Raval, Zarna Patel, Monika Gandhi, Pinal Trivedi, Maharshi Pandya, Nidhi Patel, Nitin Savaliya, Raghawendra Kumar, R D Dixit, A M Kadri, Harsh Bakshi, Chaitanya Joshi, Madhvi Joshi
EPI_ISL_512065	B.J. Medical College and Civil hospital, Ahmedabad	Gujarat Biotechnology Research Centre	Zuber Saiyed, Komal Patel, Labdhi Pandya, Afzal Ansari, Nikha Trivedi, Pranay Shah, Kamlesh J Upadhyay, Sanjay Kapadia, Apurvasinh Puvar, Janvi Raval, Zarna Patel, Monika Gandhi, Pinal Trivedi, Maharshi Pandya, Nidhi Patel, Nitin Savaliya, Raghawendra Kumar, Dinesh Kumar, R D Dixit, A M Kadri, Harsh Bakshi, Chaitanya Joshi, Madhvi Joshi
EPI_ISL_512066	Sardar Vallabhbhai Patel Institute of Medical Sciences & Research	Gujarat Biotechnology Research Centre	Komal Patel, Labdhi Pandya, Afzal Ansari, Nikha Trivedi, Pranay Shah, Kamlesh J Upadhyay, Sanjay Kapadia, Apurvasinh Puvar, Janvi Raval, Zarna Patel, Monika Gandhi, Pinal Trivedi, Maharshi Pandya, Nidhi Patel, Nitin Savaliya, Raghawendra Kumar, Dinesh Kumar, Zuber Saiyed, R D Dixit, A M Kadri, Harsh Bakshi, Chaitanya Joshi, Madhvi Joshi
EPI_ISL_512067	Sardar Vallabhbhai Patel Institute of Medical Sciences & Research	Gujarat Biotechnology Research Centre	Labdhi Pandya, Afzal Ansari, Nikha Trivedi, Pranay Shah, Kamlesh J Upadhyay, Sanjay Kapadia, Apurvasinh Puvar, Janvi Raval, Zarna Patel, Monika Gandhi, Pinal Trivedi, Maharshi Pandya, Nidhi Patel, Nitin Savaliya, Raghawendra Kumar, Dinesh Kumar, Zuber Saiyed, Komal Patel, R D Dixit, A M Kadri, Harsh Bakshi, Chaitanya Joshi, Madhvi Joshi
EPI_ISL_512068	Sardar Vallabhbhai Patel Institute of Medical Sciences & Research	Gujarat Biotechnology Research Centre	Afzal Ansari, Nikha Trivedi, Pranay Shah, Kamlesh J Upadhyay, Sanjay Kapadia, Apurvasinh Puvar, Janvi Raval, Zarna Patel, Monika Gandhi, Pinal Trivedi, Maharshi Pandya, Nidhi Patel, Nitin Savaliya, Raghawendra Kumar, Dinesh Kumar, Zuber Saiyed, Komal Patel, Labdhi Pandya, R D Dixit, A M Kadri, Harsh Bakshi, Chaitanya Joshi, Madhvi Joshi
EPI_ISL_512069	Sardar Vallabhbhai Patel Institute of Medical Sciences & Research	Gujarat Biotechnology Research Centre	Nikha Trivedi, Pranay Shah, Kamlesh J Upadhyay, Sanjay Kapadia, Apurvasinh Puvar, Janvi Raval, Zarna Patel, Monika Gandhi, Pinal Trivedi, Maharshi Pandya, Nidhi Patel, Nitin Savaliya, Raghawendra Kumar, Dinesh Kumar, Zuber Saiyed, Komal Patel, Labdhi Pandya, Afzal Ansari, R D Dixit, A M Kadri, Harsh Bakshi, Chaitanya Joshi, Madhvi Joshi
EPI_ISL_512070	Department of MicroBiology, Government Medical College, Surat	Gujarat Biotechnology Research Centre	Naresh Chauhan, Summaiya Mullan, Amit gamit, Apurvasinh Puvar, Janvi Raval, Zarna Patel, Monika Gandhi, Pinal Trivedi, Maharshi Pandya, Nidhi Patel, Nitin Savaliya, Raghawendra Kumar, Dinesh Kumar, Zuber Saiyed, Komal Patel, Labdhi Pandya, Afzal Ansari, Nikha Trivedi, R D Dixit, A M Kadri, Harsh Bakshi, Chaitanya Joshi, Madhvi Joshi
EPI_ISL_512071	Department of MicroBiology, Government Medical College, Surat	Gujarat Biotechnology Research Centre	Summaiya Mullan, Amit gamit, Apurvasinh Puvar, Janvi Raval, Zarna Patel, Monika Gandhi, Pinal Trivedi, Maharshi Pandya, Nidhi Patel, Nitin Savaliya, Raghawendra Kumar, Dinesh Kumar, Zuber Saiyed, Komal Patel, Labdhi Pandya, Afzal Ansari, Nikha Trivedi, Naresh Chauhan, R D Dixit, A M Kadri, Harsh Bakshi, Chaitanya Joshi, Madhvi Joshi
EPI_ISL_512072	Saikrishna Hospital, Mehsana	Gujarat Biotechnology Research Centre	Harshadbhai Parmar, Apurvasinh Puvar, Janvi Raval, Zarna Patel, Monika Gandhi, Pinal Trivedi, Maharshi Pandya, Nidhi Patel, Nitin Savaliya, Raghawendra Kumar, Dinesh Kumar, Zuber Saiyed, Komal Patel, Labdhi Pandya, Afzal Ansari, Nikha Trivedi, R D Dixit, A M Kadri, Harsh Bakshi, Chaitanya Joshi, Madhvi Joshi
EPI_ISL_512073	Saikrishna Hospital, Mehsana	Gujarat Biotechnology Research Centre	Apurvasinh Puvar, Janvi Raval, Zarna Patel, Monika Gandhi, Pinal Trivedi, Maharshi Pandya, Nidhi Patel, Nitin Savaliya, Raghawendra Kumar, Dinesh Kumar, Zuber Saiyed, Komal Patel, Labdhi Pandya, Afzal Ansari, Nikha Trivedi, Harshadbhai Parmar, R D Dixit, A M Kadri, Harsh Bakshi, Chaitanya Joshi, Madhvi Joshi
EPI_ISL_512074	Saikrishna Hospital, Mehsana	Gujarat Biotechnology Research Centre	Janvi Raval, Zarna Patel, Monika Gandhi, Pinal Trivedi, Maharshi Pandya, Nidhi Patel, Nitin Savaliya, Raghawendra Kumar, Dinesh Kumar, Zuber Saiyed, Komal Patel, Labdhi Pandya, Afzal Ansari, Nikha Trivedi, Harshadbhai Parmar, Apurvasinh Puvar, R D Dixit, A M Kadri, Harsh Bakshi, Chaitanya Joshi, Madhvi Joshi
EPI_ISL_512075	Dr. RSS Hospital, Modasa	Gujarat Biotechnology Research Centre	Monika Gandhi, Pinal Trivedi, Maharshi Pandya, Nidhi Patel, Nitin Savaliya, Raghawendra Kumar, Dinesh Kumar, Zuber Saiyed, Komal Patel, Labdhi Pandya, Afzal Ansari, Nikha Trivedi, Harsh Chaudhari, Apurvasinh Puvar, Janvi Raval, Zarna Patel, R D Dixit, A M Kadri, Harsh Bakshi, Chaitanya Joshi, Madhvi Joshi
EPI_ISL_512076	Dr. RSS Hospital, Modasa	Gujarat Biotechnology Research Centre	Pinal Trivedi, Maharshi Pandya, Nidhi Patel, Nitin Savaliya, Raghawendra Kumar, Dinesh Kumar, Zuber Saiyed, Komal Patel, Labdhi Pandya, Afzal Ansari, Nikha Trivedi, Harsh Chaudhari, Apurvasinh Puvar, Janvi Raval, Zarna Patel, Monika Gandhi, R D Dixit, A M Kadri, Harsh Bakshi, Chaitanya Joshi, Madhvi Joshi
EPI_ISL_512077	Dr. RSS Hospital, Modasa	Gujarat Biotechnology Research Centre	Maharshi Pandya, Nidhi Patel, Nitin Savaliya, Raghawendra Kumar, Dinesh Kumar, Zuber Saiyed, Komal Patel, Labdhi Pandya, Afzal Ansari, Nikha Trivedi, Harsh Chaudhari, Apurvasinh Puvar, Janvi Raval, Zarna Patel, Monika Gandhi, Pinal Trivedi, R D Dixit, A M Kadri, Harsh Bakshi, Chaitanya Joshi, Madhvi Joshi
EPI_ISL_512653	Area De Salud Desamparados 1 - Clinica Dr. Marcial Fallas [Grifo Alto/Desampara	Inciensa, Instituto Costarricense de Investigación y Enseñanza en Nutrición y Salud	Francisco Duarte, Hebleen Porras, Claudio Soto-Garita, Estela Cordero, Adriana Godinez & Melany Calderon
EPI_ISL_512670	Centro Nacional De Rehabilitacion Humberto Araya Rojas (Cenare)	Inciensa, Instituto Costarricense de Investigación y Enseñanza en Nutrición y Salud	Francisco Duarte, Hebleen Porras, Claudio Soto-Garita, Estela Cordero, Adriana Godinez & Melany Calderon
EPI_ISL_512844	Department of Medical Research	DMR_Myanmar	Myat Htut Nyunt, Hnin Ohnmar Soe, Kay Thi Aye, Wah Wah Aung,Yi Yi Kyaw, Aung Kyaw Kyaw, Theingi Win Myat, Phyu Win Ei, Aung Zaw Latt, Nan Aye Thida Oo, Lai Lai San, Su Mon Win, Ni Ni Zaw, Htin Lin, Hlaing Myat Thu, Zaw Than Htun
EPI_ISL_512846	O.I.J. MORGUE JUDICIAL	Inciensa, Instituto Costarricense de Investigación y Enseñanza en Nutrición y Salud	Francisco Duarte, Hebleen Porras, Claudio Soto-Garita, Estela Cordero, Adriana Godinez & Melany Calderon
EPI_ISL_512873	Centre Pasteur of Cameroun	Virology Service, Centre Pasteur of Cameroun	Richard Njouom and Serge Alain SADEUH-Mba
EPI_ISL_512874, EPI_ISL_512875, EPI_ISL_512876, EPI_ISL_512877, EPI_ISL_512878, EPI_ISL_512879, EPI_ISL_512880, EPI_ISL_512881, EPI_ISL_512882, EPI_ISL_512883, EPI_ISL_512884, EPI_ISL_512885, EPI_ISL_512886, EPI_ISL_512887, EPI_ISL_512888, EPI_ISL_512889, EPI_ISL_512890, EPI_ISL_512891, EPI_ISL_512892, EPI_ISL_512893, EPI_ISL_512894, EPI_ISL_512895, EPI_ISL_512896, EPI_ISL_512897, EPI_ISL_512898, EPI_ISL_512899, EPI_ISL_512900, EPI_ISL_512901, EPI_ISL_512902, EPI_ISL_512903			
see above	Pathogen Genomics Lab King Abdullah University of Science and Technology(KAUST)	Pathogen Genomics Lab King Abdullah University of Science and Technology(KAUST)	Raeece Naeem, Rahul P Salunke, Sharif Hala, Sara Mfarrej, Amit Kumar Subudhi, Fadwa Alofi, Fathia Ben Rached, Afrah Alsomali, Asim Khogeer, Ahmad Bakur Mahmoud, Anwar Hashem, Naif Almontashiri, Arnab Pain
EPI_ISL_512904, EPI_ISL_512905, EPI_ISL_512906, EPI_ISL_512907	Pathogen Genomics Lab King Abdullah University of Science and Technology(KAUST)	Pathogen Genomics Lab King Abdullah University of Science and Technology(KAUST)	Fathia Ben Rached, Raeece Naeem, Sharif Hala, Fadwa Alofi, Rahul P Salunke, Sara Mfarrej, Amit Kumar Subudhi, Afrah Alsomali, Asim Khogeer, Ahmad Bakur Mahmoud, Anwar Hashem, Naif Almontashiri, Arnab Pain
EPI_ISL_512908, EPI_ISL_512909, EPI_ISL_512910, EPI_ISL_512911, EPI_ISL_512912, EPI_ISL_512913, EPI_ISL_512914, EPI_ISL_512915, EPI_ISL_512916, EPI_ISL_512917	Pathogen Genomics Lab King Abdullah University of Science and Technology(KAUST)	Pathogen Genomics Lab King Abdullah University of Science and Technology(KAUST)	Sharif Hala, Fadwa Alofi, Sara Mfarrej, Amit Kumar Subudhi, Rahul P Salunke, Fathia Ben Rached, Amanda Ooi, Luke Esau, Afrah Alsomali, Asim Khogeer, Jumana Taha, Abdulaziz Alahmadi, Kahled Algithami, Raeece Naeem, Anwar Hashem, Naif Almontashiri, Arnab Pain
EPI_ISL_512918, EPI_ISL_512919, EPI_ISL_512920, EPI_ISL_512921, EPI_ISL_512922, EPI_ISL_512923, EPI_ISL_512924, EPI_ISL_512925, EPI_ISL_512926, EPI_ISL_512927, EPI_ISL_512928, EPI_ISL_512929, EPI_ISL_512930, EPI_ISL_512931, EPI_ISL_512932, EPI_ISL_512933, EPI_ISL_512934, EPI_ISL_512935,			

EPI_ISL_512936, EPI_ISL_512937, EPI_ISL_512938, EPI_ISL_512939, EPI_ISL_512940, EPI_ISL_512941, EPI_ISL_512942, EPI_ISL_512943, EPI_ISL_512944, EPI_ISL_512945			
see above	Pathogen Genomics Lab King Abdullah University of Science and Technology(KAUST)	Pathogen Genomics Lab King Abdullah University of Science and Technology(KAUST)	Fadwa Alofi, Sharif Hala, Rahul P Salunke, Sara Mfarrej, Amit Kumar Subudhi, Fathia Ben Rached, Amanda, Luke, Afrah Alsomali, Asim Khogeer, Jumana Taha, Abdulaziz Alahmadi, Kahled Algithami, Raeece Naeem, Anwar Hashem, Naif Almontashiri, Arnab Pain
EPI_ISL_512946, EPI_ISL_512947, EPI_ISL_512948, EPI_ISL_512949, EPI_ISL_512950, EPI_ISL_512951, EPI_ISL_512952, EPI_ISL_512953, EPI_ISL_512954, EPI_ISL_512955, EPI_ISL_512956, EPI_ISL_512957, EPI_ISL_512958, EPI_ISL_512959, EPI_ISL_512960, EPI_ISL_512961, EPI_ISL_512962, EPI_ISL_512963, EPI_ISL_512964, EPI_ISL_512965, EPI_ISL_512966, EPI_ISL_512967, EPI_ISL_512968, EPI_ISL_512969, EPI_ISL_512970, EPI_ISL_512971, EPI_ISL_512972, EPI_ISL_512973, EPI_ISL_512974, EPI_ISL_512975, EPI_ISL_512976, EPI_ISL_512977, EPI_ISL_512978, EPI_ISL_512979, EPI_ISL_512980, EPI_ISL_512981, EPI_ISL_512982, EPI_ISL_512983, EPI_ISL_512984, EPI_ISL_512985, EPI_ISL_512986, EPI_ISL_512987, EPI_ISL_512988			
see above	Pathogen Genomics Lab King Abdullah University of Science and Technology(KAUST)	Pathogen Genomics Lab King Abdullah University of Science and Technology(KAUST)	Sara Mfarrej, Raeece Naeem, Rahul P Salunke, Sharif Hala, Fadwa Alofi, Amit Kumar Subudhi, Fathia Ben Rached, Afrah Alsomali, Jumana Taha, Abdulaziz Alahmadi, Asim Khogeer, Nashwa Al-khotani, Anwar Hashem, Naif Almontashiri, Arnab Pain
EPI_ISL_512989, EPI_ISL_512990, EPI_ISL_512991, EPI_ISL_512992, EPI_ISL_512993, EPI_ISL_512994, EPI_ISL_512995, EPI_ISL_512996, EPI_ISL_512997, EPI_ISL_512998, EPI_ISL_512999, EPI_ISL_513000, EPI_ISL_513001			
see above	Pathogen Genomics Lab King Abdullah University of Science and Technology(KAUST)	Pathogen Genomics Lab King Abdullah University of Science and Technology(KAUST)	Amit Kumar Subudhi, Rahul P Salunke, Sara Mfarrej, Sharif Hala, Fadwa Alofi, Fathia Ben Rached, Afrah Alsomali, Asim Khogeer, Nashwa Al-khotani, Raeece Naeem, Anwar Hashem, Naif Almontashiri, Arnab Pain
EPI_ISL_513002, EPI_ISL_513003, EPI_ISL_513004, EPI_ISL_513005, EPI_ISL_513006, EPI_ISL_513007, EPI_ISL_513008, EPI_ISL_513009, EPI_ISL_513010, EPI_ISL_513011, EPI_ISL_513012, EPI_ISL_513013, EPI_ISL_513014, EPI_ISL_513015, EPI_ISL_513016, EPI_ISL_513017, EPI_ISL_513018, EPI_ISL_513019, EPI_ISL_513020, EPI_ISL_513021, EPI_ISL_513022, EPI_ISL_513023, EPI_ISL_513024, EPI_ISL_513025, EPI_ISL_513026			
see above	Pathogen Genomics Lab King Abdullah University of Science and Technology(KAUST)	Pathogen Genomics Lab King Abdullah University of Science and Technology(KAUST)	Afrah Alsomali, Fathia Ben Rached, Raeece Naeem, Sharif Hala,Rahul P Salunke, Amanda Ooi, Luke Esau, Sara Mfarrej, Amit Kumar Subudhi, Fadwa Alofi, Asim Khogeer, Kahled Algithami, Anwar Hashem, Naif Almontashiri, Arnab Pain
EPI_ISL_513027, EPI_ISL_513028, EPI_ISL_513029, EPI_ISL_513030, EPI_ISL_513031, EPI_ISL_513032, EPI_ISL_513033, EPI_ISL_513034, EPI_ISL_513035, EPI_ISL_513036, EPI_ISL_513037, EPI_ISL_513038, EPI_ISL_513039, EPI_ISL_513040, EPI_ISL_513041, EPI_ISL_513042, EPI_ISL_513043, EPI_ISL_513044, EPI_ISL_513045, EPI_ISL_513046, EPI_ISL_513047, EPI_ISL_513048, EPI_ISL_513049, EPI_ISL_513050, EPI_ISL_513051, EPI_ISL_513052, EPI_ISL_513053, EPI_ISL_513054, EPI_ISL_513055, EPI_ISL_513056, EPI_ISL_513057, EPI_ISL_513058, EPI_ISL_513059, EPI_ISL_513060, EPI_ISL_513061, EPI_ISL_513062, EPI_ISL_513063			
see above	Pathogen Genomics Lab King Abdullah University of Science and Technology(KAUST)	Pathogen Genomics Lab King Abdullah University of Science and Technology(KAUST)	Rahul P Salunke, Sharif Hala, Raeece Naeem, Sara Mfarrej, Amit Kumar Subudhi, Amanda Ooi, Luke Esau, Fadwa Alofi, Fathia Ben Rached, Afrah Alsomali, Asim Khogeer, Ahmad Bakur Mahmoud, Anwar Hashem, Naif Almontashiri, Arnab Pain
EPI_ISL_513064, EPI_ISL_513065, EPI_ISL_513066, EPI_ISL_513067, EPI_ISL_513068, EPI_ISL_513069, EPI_ISL_513070, EPI_ISL_513071, EPI_ISL_513072, EPI_ISL_513073, EPI_ISL_513074, EPI_ISL_513075, EPI_ISL_513076			
see above	Pathogen Genomics Lab King Abdullah University of Science and Technology(KAUST)	Pathogen Genomics Lab King Abdullah University of Science and Technology(KAUST)	Raeece Naeem, Rahul P Salunke, Sharif Hala, Sara Mfarrej, Amit Kumar Subudhi, Fadwa Alofi, Fathia Ben Rached, Afrah Alsomali, Asim Khogeer, Ahmad Bakur Mahmoud, Anwar Hashem, Naif Almontashiri, Arnab Pain
EPI_ISL_513078, EPI_ISL_513079, EPI_ISL_513081, EPI_ISL_513082, EPI_ISL_513083, EPI_ISL_513084, EPI_ISL_513086, EPI_ISL_513087, EPI_ISL_513089, EPI_ISL_513090, EPI_ISL_513092, EPI_ISL_513093, EPI_ISL_513094, EPI_ISL_513095, EPI_ISL_513096, EPI_ISL_513097, EPI_ISL_513098, EPI_ISL_513099, EPI_ISL_513100, EPI_ISL_513101, EPI_ISL_513102, EPI_ISL_513103, EPI_ISL_513104, EPI_ISL_513105, EPI_ISL_513106, EPI_ISL_513107, EPI_ISL_513108, EPI_ISL_513109, EPI_ISL_513110, EPI_ISL_513111, EPI_ISL_513112, EPI_ISL_513113, EPI_ISL_513114, EPI_ISL_513115, EPI_ISL_513116, EPI_ISL_513117, EPI_ISL_513118, EPI_ISL_513119, EPI_ISL_513120			
see above	Pathogen Genomics Lab King Abdullah University of Science and Technology(KAUST)	Pathogen Genomics Lab King Abdullah University of Science and Technology(KAUST)	Fathia Ben Rached, Raeece Naeem, Sharif Hala, Fadwa Alofi, Rahul P Salunke, Sara Mfarrej, Amit Kumar Subudhi, Afrah Alsomali, Asim Khogeer, Ahmad Bakur Mahmoud, Anwar Hashem, Naif Almontashiri, Arnab Pain
EPI_ISL_513122, EPI_ISL_513123, EPI_ISL_513124, EPI_ISL_513125, EPI_ISL_513126, EPI_ISL_513127, EPI_ISL_513128, EPI_ISL_513129, EPI_ISL_513130, EPI_ISL_513131, EPI_ISL_513132, EPI_ISL_513133, EPI_ISL_513134, EPI_ISL_513135, EPI_ISL_513137, EPI_ISL_513138, EPI_ISL_513139, EPI_ISL_513140, EPI_ISL_513141, EPI_ISL_513142, EPI_ISL_513143, EPI_ISL_513144, EPI_ISL_513145, EPI_ISL_513146			
see above	Pathogen Genomics Lab King Abdullah University of Science and Technology(KAUST)	Pathogen Genomics Lab King Abdullah University of Science and Technology(KAUST)	Sharif Hala, Fadwa Alofi, Sara Mfarrej, Amit Kumar Subudhi, Rahul P Salunke, Fathia Ben Rached, Amanda Ooi, Luke Esau, Afrah Alsomali, Asim Khogeer, Jumana Taha, Abdulaziz Alahmadi, Kahled Algithami, Raeece Naeem, Anwar Hashem, Naif Almontashiri, Arnab Pain
EPI_ISL_513147, EPI_ISL_513148, EPI_ISL_513149, EPI_ISL_513150, EPI_ISL_513151, EPI_ISL_513152, EPI_ISL_513153, EPI_ISL_513154, EPI_ISL_513155, EPI_ISL_513156, EPI_ISL_513157, EPI_ISL_513158, EPI_ISL_513159, EPI_ISL_513160, EPI_ISL_513161, EPI_ISL_513162, EPI_ISL_513163, EPI_ISL_513164, EPI_ISL_513165, EPI_ISL_513166, EPI_ISL_513167			
see above	Pathogen Genomics Lab King Abdullah University of Science and Technology(KAUST)	Pathogen Genomics Lab King Abdullah University of Science and Technology(KAUST)	Fadwa Alofi, Sharif Hala, Rahul P Salunke, Sara Mfarrej, Amit Kumar Subudhi, Fathia Ben Rached, Amanda, Luke, Afrah Alsomali, Asim Khogeer, Jumana Taha, Abdulaziz Alahmadi, Kahled Algithami, Raeece Naeem, Anwar Hashem, Naif Almontashiri, Arnab Pain
EPI_ISL_513168, EPI_ISL_513169, EPI_ISL_513170, EPI_ISL_513171, EPI_ISL_513172, EPI_ISL_513173, EPI_ISL_513174	Pathogen Genomics Lab King Abdullah University of Science and Technology(KAUST)	Pathogen Genomics Lab King Abdullah University of Science and Technology(KAUST)	Sara Mfarrej, Raeece Naeem, Rahul P Salunke, Sharif Hala, Fadwa Alofi, Amit Kumar Subudhi, Fathia Ben Rached, Afrah Alsomali, Jumana Taha, Abdulaziz Alahmadi, Asim Khogeer, Nashwa Al-khotani, Anwar Hashem, Naif Almontashiri, Arnab Pain
EPI_ISL_513175, EPI_ISL_513176, EPI_ISL_513177, EPI_ISL_513178, EPI_ISL_513179, EPI_ISL_513180, EPI_ISL_513181, EPI_ISL_513182, EPI_ISL_513183, EPI_ISL_513184, EPI_ISL_513185, EPI_ISL_513186, EPI_ISL_513187, EPI_ISL_513188, EPI_ISL_513189			
see above	Pathogen Genomics Lab King Abdullah University of Science and Technology(KAUST)	Pathogen Genomics Lab King Abdullah University of Science and Technology(KAUST)	Amit Kumar Subudhi, Rahul P Salunke, Sara Mfarrej, Sharif Hala, Fadwa Alofi, Fathia Ben Rached, Afrah Alsomali, Asim Khogeer, Nashwa Al-khotani, Raeece Naeem, Anwar Hashem, Naif Almontashiri, Arnab Pain
EPI_ISL_513190, EPI_ISL_513191, EPI_ISL_513192, EPI_ISL_513193, EPI_ISL_513194, EPI_ISL_513195, EPI_ISL_513196, EPI_ISL_513197	Pathogen Genomics Lab King Abdullah University of Science and Technology(KAUST)	Pathogen Genomics Lab King Abdullah University of Science and Technology(KAUST)	Afrah Alsomali, Fathia Ben Rached, Raeece Naeem, Sharif Hala,Rahul P Salunke, Amanda Ooi, Luke Esau, Sara Mfarrej, Amit Kumar Subudhi, Fadwa Alofi, Asim Khogeer, Kahled Algithami, Anwar Hashem, Naif Almontashiri, Arnab Pain
EPI_ISL_513198, EPI_ISL_513199, EPI_ISL_513200, EPI_ISL_513201, EPI_ISL_513202, EPI_ISL_513203, EPI_ISL_513204, EPI_ISL_513205, EPI_ISL_513206, EPI_ISL_513207	Pathogen Genomics Lab King Abdullah University of Science and Technology(KAUST)	Pathogen Genomics Lab King Abdullah University of Science and Technology(KAUST)	Rahul P Salunke, Sharif Hala, Raeece Naeem, Sara Mfarrej, Amit Kumar Subudhi, Amanda Ooi, Luke Esau, Fadwa Alofi, Fathia Ben Rached, Afrah Alsomali, Asim Khogeer, Ahmad Bakur Mahmoud, Anwar Hashem, Naif Almontashiri, Arnab Pain
EPI_ISL_513208, EPI_ISL_513209, EPI_ISL_513210, EPI_ISL_513211, EPI_ISL_513212, EPI_ISL_513213	Pathogen Genomics Lab King Abdullah University of Science and Technology(KAUST)	Pathogen Genomics Lab King Abdullah University of Science and Technology(KAUST)	Raeece Naeem, Rahul P Salunke, Sharif Hala, Sara Mfarrej, Amit Kumar Subudhi, Fadwa Alofi, Fathia Ben Rached, Afrah Alsomali, Asim Khogeer, Ahmad Bakur Mahmoud, Anwar Hashem, Naif Almontashiri, Arnab Pain
EPI_ISL_513214, EPI_ISL_513215, EPI_ISL_513216, EPI_ISL_513217, EPI_ISL_513218, EPI_ISL_513219, EPI_ISL_513220, EPI_ISL_513221, EPI_ISL_513222, EPI_ISL_513223, EPI_ISL_513224, EPI_ISL_513225, EPI_ISL_513226			
see above	Pathogen Genomics Lab King Abdullah University of Science and Technology(KAUST)	Pathogen Genomics Lab King Abdullah University of Science and Technology(KAUST)	Sharif Hala, Fadwa Alofi, Sara Mfarrej, Amit Kumar Subudhi, Rahul P Salunke, Fathia Ben Rached, Amanda Ooi, Luke Esau, Afrah Alsomali, Asim Khogeer, Jumana Taha, Abdulaziz Alahmadi, Kahled Algithami, Raeece Naeem, Anwar Hashem, Naif Almontashiri, Arnab Pain
EPI_ISL_513227, EPI_ISL_513228, EPI_ISL_513229, EPI_ISL_513230, EPI_ISL_513231, EPI_ISL_513232, EPI_ISL_513233, EPI_ISL_513234, EPI_ISL_513235, EPI_ISL_513236, EPI_ISL_513237, EPI_ISL_513238, EPI_ISL_513240, EPI_ISL_513241, EPI_ISL_513242, EPI_ISL_513243, EPI_ISL_513244, EPI_ISL_513245, EPI_ISL_513246, EPI_ISL_513247, EPI_ISL_513248			
see above	Pathogen Genomics Lab King Abdullah University of Science and Technology(KAUST)	Pathogen Genomics Lab King Abdullah University of Science and Technology(KAUST)	Amit Kumar Subudhi, Rahul P Salunke, Sara Mfarrej, Sharif Hala, Fadwa Alofi, Fathia Ben Rached, Afrah Alsomali, Asim Khogeer, Nashwa Al-khotani, Raeece Naeem, Anwar Hashem, Naif Almontashiri, Arnab Pain
EPI_ISL_513249, EPI_ISL_513250, EPI_ISL_513251, EPI_ISL_513252, EPI_ISL_513253, EPI_ISL_513254, EPI_ISL_513255, EPI_ISL_513256, EPI_ISL_513257, EPI_ISL_513258, EPI_ISL_513259, EPI_ISL_513260, EPI_ISL_513261, EPI_ISL_513262, EPI_ISL_513263, EPI_ISL_513264			
see above	Pathogen Genomics Lab King Abdullah University of Science and Technology(KAUST)	Pathogen Genomics Lab King Abdullah University of Science and Technology(KAUST)	Afrah Alsomali, Fathia Ben Rached, Raeece Naeem, Sharif Hala,Rahul P Salunke, Amanda Ooi, Luke Esau, Sara Mfarrej, Amit Kumar Subudhi, Fadwa Alofi, Asim Khogeer, Kahled Algithami, Anwar Hashem, Naif Almontashiri, Arnab Pain
EPI_ISL_513294	Baylor College of Medicine	Baylor College of Medicine: HGSC	Vasanthi Avadhanula, Erin Nicholson, David Henke, Pedro Piedra, Harsha Doddapaneni, Donna Muzny, Qingchang Meng, Hsu Chao, Zeineen Momin, Hua Shen, George Weissenberger, Kavya Kottapalli, Yimiti Meiheerguli, Sejal Salvi, Ginger Metcalf, Vipin Menon, Sara J.J. Cregeen, Matthew C. Ross, Tulin Ayvaz, Richard Sucgang, Kristi L. Hoffman, Matthew Wong, Joseph F. Petrosino
EPI_ISL_513314, EPI_ISL_513511, EPI_ISL_513512	The National Institute of Public Health	The National Institute of Public Health and State Veterinary Institute Prague	Nagy,A;Jirincova,H;Novakova,L;Trnka,D;Vecerova,J
EPI_ISL_513513, EPI_ISL_513514, EPI_ISL_513515, EPI_ISL_513516, EPI_ISL_513517, EPI_ISL_513518, EPI_ISL_513519, EPI_ISL_513520, EPI_ISL_513521, EPI_ISL_513522, EPI_ISL_513523, EPI_ISL_513524, EPI_ISL_513525, EPI_ISL_513526, EPI_ISL_513527, EPI_ISL_513528, EPI_ISL_513529, EPI_ISL_513530, EPI_ISL_513531, EPI_ISL_513532, EPI_ISL_513533, EPI_ISL_513534, EPI_ISL_513535, EPI_ISL_513536, EPI_ISL_513537, EPI_ISL_513538, EPI_ISL_513539, EPI_ISL_513540, EPI_ISL_513541, EPI_ISL_513542, EPI_ISL_513543, EPI_ISL_513544, EPI_ISL_513545, EPI_ISL_513546, EPI_ISL_513547, EPI_ISL_513548,			

EPI_ISL_513549, EPI_ISL_513550, EPI_ISL_513551, EPI_ISL_513552, EPI_ISL_513553, EPI_ISL_513554, EPI_ISL_513555, EPI_ISL_513556, EPI_ISL_513557, EPI_ISL_513558, EPI_ISL_513559, EPI_ISL_513560, EPI_ISL_513561, EPI_ISL_513562, EPI_ISL_513563, EPI_ISL_513564, EPI_ISL_513565, EPI_ISL_513566, EPI_ISL_513567, EPI_ISL_513568, EPI_ISL_513569, EPI_ISL_513570, EPI_ISL_513571, EPI_ISL_513572, EPI_ISL_513573, EPI_ISL_513574, EPI_ISL_513575, EPI_ISL_513576, EPI_ISL_513577, EPI_ISL_513578, EPI_ISL_513579, EPI_ISL_513580, EPI_ISL_513581, EPI_ISL_513582, EPI_ISL_513583			
see above	Programa de Oncovirologia, Instituto Nacional de Câncer	Programa de Oncovirologia, Instituto Nacional de Câncer	Juliana D. Siqueira, Livia R. Goes, Brunna M. Alves, Claudia Cicala,James Arthos, João P.B. Viola, Andreia C. de Melo, Marcelo A. Soares
EPI_ISL_514131	Rondônia Central Public Health Laboratory (LACEN/RO), vinculated to State Health Secretariat of Rondônia (SESAU/RO)	Molecular Virology Laboratory of Oswaldo Cruz Foundation of Rondônia	Luan Felipo Botelho-Souza, Felipe Souza Nogueira-Lima, Tárcio Peixoto Roca, Alcione de Oliveira dos Santos, Felipe Gomes Naveca, Adriana Cristina Salvador Maia, Cicileia Correia da Silva, Aline Linhares Ferreira de Melo Mendonça, Celina Aparecida Bertoni Lugtenburg, Camila Flávia Gomes Azzi, Juliana Loca Furtado, Suelen Cavalcante, Rita de Cássia Pontello Rampazzo, Caio Henrique Nemeth Santos, Alice Paula Di Sabatino Guimarães, Jansen Fernandes de Medeiros, Fernando Rodrigues Máximo, Juan Miguel Vilallobos-Salcedo and Deusilene Souza Vieira1
EPI_ISL_514132	Rondônia Central Public Health Laboratory (LACEN/RO), vinculated to State Health Secretariat of Rondônia (SESAU/RO)	Molecular Virology Laboratory of Oswaldo Cruz Foundation of Rondônia	Luan Felipo Botelho-Souza, Felipe Souza Nogueira-Lima, Tárcio Peixoto Roca, Alcione de Oliveira dos Santos, Felipe Gomes Naveca, Adriana Cristina Salvador Maia, Cicileia Correia da Silva, Aline Linhares Ferreira de Melo Mendonça, Celina Aparecida Bertoni Lugtenburg, Camila Flávia Gomes Azzi, Juliana Loca Furtado, Suelen Cavalcante, Rita de Cássia Pontello Rampazzo, Caio Henrique Nemeth Santos, Alice Paula Di Sabatino Guimarães, Jansen Fernandes de Medeiros, Fernando Rodrigues Máximo, Juan Miguel Vilallobos-Salcedo and Deusilene Souza Vieira.
EPI_ISL_514133, EPI_ISL_514134, EPI_ISL_514135, EPI_ISL_514136, EPI_ISL_514137, EPI_ISL_514138	Rondônia Central Public Health Laboratory (LACEN/RO), vinculated to State Health Secretariat of Rondônia (SESAU/RO)	Molecular Virology Laboratory of Oswaldo Cruz Foundation of Rondônia	Luan Felipo Botelho-Souza, Felipe Souza Nogueira-Lima, Tárcio Peixoto Roca, Alcione de Oliveira dos Santos, Felipe Gomes Naveca, Adriana Cristina Salvador Maia, Cicileia Correia da Silva, Aline Linhares Ferreira de Melo Mendonça, Celina Aparecida Bertoni Lugtenburg, Camila Flávia Gomes Azzi, Juliana Loca Furtado, Suelen Cavalcante, Rita de Cássia Pontello Rampazzo, Caio Henrique Nemeth Santos, Alice Paula Di Sabatino Guimarães, Jansen Fernandes de Medeiros, Fernando Rodrigues Máximo, Juan Miguel Vilallobos-Salcedo and Deusilene Souza Vieira
EPI_ISL_514434	NSTU COVID-19 Diagnostic Center,	NSU Genome Research Institute (NGRI), North South University	Dr. Muhammad Maqsud Hossain, Aura Rahman, Prof. Firoz Ahmed, Tahrima Huq, Abdus Sadique, Jahidul Alam, Md Aminul Islam, Prof. Md. Didar-Ul-Alam, Prof. Kazi Nadim Hasan, Prof. Abdul Khaleque, Prof, Hasan Mahmud Reza
EPI_ISL_514435	Dr. RSS Hospital, Modasa	Gujarat Biotechnology Research Centre	Harsh Chaudhari, Zarna Patel, Monika Gandhi, Pinal Trivedi, Maharshi Pandya, Nidhi Patel, Nitin Savaliya, Raghawendra Kumar, Dinesh Kumar, Zuber Saiyed, Komal Patel, Labdhi Pandya, Afzal Ansari, Nikha Trivedi, Apurvasinh Puvar, Janvi Raval, R D Dixit, A M Kadri, Harsh Bakshi, Chaitanya Joshi, Madhvi Joshi
EPI_ISL_514436	Dr. RSS Hospital, Modasa	Gujarat Biotechnology Research Centre	Zarna Patel, Monika Gandhi, Pinal Trivedi, Maharshi Pandya, Nidhi Patel, Nitin Savaliya, Raghawendra Kumar, Dinesh Kumar, Zuber Saiyed, Komal Patel, Labdhi Pandya, Afzal Ansari, Nikha Trivedi, Harsh Chaudhari, Apurvasinh Puvar, Janvi Raval, R D Dixit, A M Kadri, Harsh Bakshi, Chaitanya Joshi, Madhvi Joshi
EPI_ISL_514437	Dr. RSS Hospital, Modasa	Gujarat Biotechnology Research Centre	Nidhi Patel, Nitin Savaliya, Raghawendra Kumar, Dinesh Kumar, Zuber Saiyed, Komal Patel, Labdhi Pandya, Afzal Ansari, Nikha Trivedi, Harsh Chaudhari, Apurvasinh Puvar, Janvi Raval, Zarna Patel, Monika Gandhi, Pinal Trivedi, Maharshi Pandya, R D Dixit, A M Kadri, Harsh Bakshi, Chaitanya Joshi, Madhvi Joshi
EPI_ISL_514440	NSTU COVID-19 Diagnostic Center	NSU Genome Research Institute (NGRI), North South University	Dr. Muhammad Maqsud Hossain, Aura Rahman, Prof. Firoz Ahmed, Tahrima Huq, Abdus Sadique, Tamanna Afroze, Jahidul Alam, Md Aminul Islam, Prof. Md. Didar-Ul-Alam, Prof. Kazi Nadim Hasan, Prof. Abdul Khaleque, Prof, Hasan Mahmud Reza
EPI_ISL_514441	NSTU COVID-19 Diagnostic Center	NSU Genome Research Institute (NGRI), North South University	Dr. Muhammad Maqsud Hossain, Aura Rahman, Prof. Firoz Ahmed, Tahrima Huq, Abdus Sadique, Jahidul Alam, Md Aminul Islam, Prof. Md. Didar-Ul-Alam, Prof. Kazi Nadim Hasan, Prof. Abdul Khaleque, Prof, Hasan Mahmud Reza
EPI_ISL_514580	NSTU COVID-19 Diagnostic Center	NSU Genome Research Institute (NGRI), North South University	Dr. Muhammad Maqsud Hossain, Aura Rahman, Prof. Firoz Ahmed, Tahrima Huq, Abdus Sadique, Jahidul Alam, Tamanna Afroze, Md Aminul Islam, Prof. Md. Didar-Ul-Alam, Prof. Kazi Nadim Hasan, Prof. Abdul Khaleque, Prof, Hasan Mahmud Reza
EPI_ISL_514581	B.J. Medical College and Civil hospital, Ahmedabad	Gujarat Biotechnology Research Centre	Nitin Savaliya, Raghawendra Kumar, Dinesh Kumar, Zuber Saiyed, Komal Patel, Labdhi Pandya, Afzal Ansari, Nikha Trivedi, Pranay Shah, Kamlesh J Upadhyay, Sanjay Kapadia, Apurvasinh Puvar, Janvi Raval, Zarna Patel, Monika Gandhi, Pinal Trivedi, Maharshi Pandya, Nidhi Patel, R D Dixit, A M Kadri, Harsh Bakshi, Chaitanya Joshi, Madhvi Joshi
EPI_ISL_514582	B.J. Medical College and Civil hospital, Ahmedabad	Gujarat Biotechnology Research Centre	Raghawendra Kumar, Dinesh Kumar, Zuber Saiyed, Komal Patel, Labdhi Pandya, Afzal Ansari, Nikha Trivedi, Pranay Shah, Kamlesh J Upadhyay, Sanjay Kapadia, Apurvasinh Puvar, Janvi Raval, Zarna Patel, Monika Gandhi, Pinal Trivedi, Maharshi Pandya, Nidhi Patel, Nitin Savaliya, R D Dixit, A M Kadri, Harsh Bakshi, Chaitanya Joshi, Madhvi Joshi
EPI_ISL_514583	B.J. Medical College and Civil hospital, Ahmedabad	Gujarat Biotechnology Research Centre	Dinesh Kumar, Zuber Saiyed, Komal Patel, Labdhi Pandya, Afzal Ansari, Nikha Trivedi, Pranay Shah, Kamlesh J Upadhyay, Sanjay Kapadia, Apurvasinh Puvar, Janvi Raval, Zarna Patel, Monika Gandhi, Pinal Trivedi, Maharshi Pandya, Nidhi Patel, Nitin Savaliya, Raghawendra Kumar, R D Dixit, A M Kadri, Harsh Bakshi, Chaitanya Joshi, Madhvi Joshi
EPI_ISL_514584	B.J. Medical College and Civil hospital, Ahmedabad	Gujarat Biotechnology Research Centre	Zuber Saiyed, Komal Patel, Labdhi Pandya, Afzal Ansari, Nikha Trivedi, Pranay Shah, Kamlesh J Upadhyay, Sanjay Kapadia, Apurvasinh Puvar, Janvi Raval, Zarna Patel, Monika Gandhi, Pinal Trivedi, Maharshi Pandya, Nidhi Patel, Nitin Savaliya, Raghawendra Kumar, Dinesh Kumar, R D Dixit, A M Kadri, Harsh Bakshi, Chaitanya Joshi, Madhvi Joshi
EPI_ISL_514585	B.J. Medical College and Civil hospital, Ahmedabad	Gujarat Biotechnology Research Centre	Komal Patel, Labdhi Pandya, Afzal Ansari, Nikha Trivedi, Pranay Shah, Kamlesh J Upadhyay, Sanjay Kapadia, Apurvasinh Puvar, Janvi Raval, Zarna Patel, Monika Gandhi, Pinal Trivedi, Maharshi Pandya, Nidhi Patel, Nitin Savaliya, Raghawendra Kumar, Dinesh Kumar, Zuber Saiyed, R D Dixit, A M Kadri, Harsh Bakshi, Chaitanya Joshi, Madhvi Joshi
EPI_ISL_514586	B.J. Medical College and Civil hospital, Ahmedabad	Gujarat Biotechnology Research Centre	Labdhi Pandya, Afzal Ansari, Nikha Trivedi, Pranay Shah, Kamlesh J Upadhyay, Sanjay Kapadia, Apurvasinh Puvar, Janvi Raval, Zarna Patel, Monika Gandhi, Pinal Trivedi, Maharshi Pandya, Nidhi Patel, Nitin Savaliya, Raghawendra Kumar, Dinesh Kumar, Zuber Saiyed, Komal Patel, R D Dixit, A M Kadri, Harsh Bakshi, Chaitanya Joshi, Madhvi Joshi
EPI_ISL_514587	B.J. Medical College and Civil hospital, Ahmedabad	Gujarat Biotechnology Research Centre	Afzal Ansari, Nikha Trivedi, Pranay Shah, Kamlesh J Upadhyay, Sanjay Kapadia, Apurvasinh Puvar, Janvi Raval, Zarna Patel, Monika Gandhi, Pinal Trivedi, Maharshi Pandya, Nidhi Patel, Nitin Savaliya, Raghawendra Kumar, Dinesh Kumar, Zuber Saiyed, Komal Patel, Labdhi Pandya, R D Dixit, A M Kadri, Harsh Bakshi, Chaitanya Joshi, Madhvi Joshi
EPI_ISL_514588	B.J. Medical College and Civil hospital, Ahmedabad	Gujarat Biotechnology Research Centre	Nikha Trivedi, Pranay Shah, Kamlesh J Upadhyay, Sanjay Kapadia, Apurvasinh Puvar, Janvi Raval, Zarna Patel, Monika Gandhi, Pinal Trivedi, Maharshi Pandya, Nidhi Patel, Nitin Savaliya, Raghawendra Kumar, Dinesh Kumar, Zuber Saiyed, Komal Patel, Labdhi Pandya, Afzal Ansari, R D Dixit, A M Kadri, Harsh Bakshi, Chaitanya Joshi, Madhvi Joshi
EPI_ISL_514589	B.J. Medical College and Civil hospital, Ahmedabad	Gujarat Biotechnology Research Centre	Pranay Shah, Kamlesh J Upadhyay, Sanjay Kapadia, Apurvasinh Puvar, Janvi Raval, Zarna Patel, Monika Gandhi, Pinal Trivedi, Maharshi Pandya, Nidhi Patel, Nitin Savaliya, Raghawendra Kumar, Dinesh Kumar, Zuber Saiyed, Komal Patel, Labdhi Pandya, Afzal Ansari, Nikha Trivedi, R D Dixit, A M Kadri, Harsh Bakshi, Chaitanya Joshi, Madhvi Joshi
EPI_ISL_514590	B.J. Medical College and Civil hospital, Ahmedabad	Gujarat Biotechnology Research Centre	Kamlesh J Upadhyay, Sanjay Kapadia, Apurvasinh Puvar, Janvi Raval, Zarna Patel, Monika Gandhi, Pinal Trivedi, Maharshi Pandya, Nidhi Patel, Nitin Savaliya, Raghawendra Kumar, Dinesh Kumar, Zuber Saiyed, Komal Patel, Labdhi Pandya, Afzal Ansari, Nikha Trivedi, Pranay Shah, R D Dixit, A M Kadri, Harsh Bakshi, Chaitanya Joshi, Madhvi Joshi
EPI_ISL_514591	B.J. Medical College and Civil hospital, Ahmedabad	Gujarat Biotechnology Research Centre	Sanjay Kapadia,Apurvasinh Puvar,Janvi Raval,Zarna Patel, Monika Gandhi, Pinal Trivedi, Maharshi Pandya, Nidhi Patel, Nitin Savaliya, Raghawendra Kumar, Dinesh Kumar, Zuber Saiyed, Komal Patel, Labdhi Pandya, Afzal Ansari, Nikha Trivedi, Pranay Shah, Kamlesh J Upadhyay, R D Dixit, A M Kadri, Harsh Bakshi, Chaitanya Joshi, Madhvi Joshi
EPI_ISL_514592	B.J. Medical College and Civil hospital, Ahmedabad	Gujarat Biotechnology Research Centre	Apurvasinh Puvar, Janvi Raval, Zarna Patel, Monika Gandhi, Pinal Trivedi, Maharshi Pandya, Nidhi Patel, Nitin Savaliya, Raghawendra Kumar, Dinesh Kumar, Zuber Saiyed, Komal Patel, Labdhi Pandya, Afzal Ansari, Nikha Trivedi, Pranay Shah, Kamlesh J Upadhyay, Sanjay Kapadia, R D Dixit, A M Kadri, Harsh Bakshi, Chaitanya Joshi, Madhvi Joshi
EPI_ISL_514593	B.J. Medical College and Civil hospital, Ahmedabad	Gujarat Biotechnology Research Centre	Janvi Raval, Zarna Patel, Monika Gandhi, Pinal Trivedi, Maharshi Pandya, Nidhi Patel, Nitin Savaliya, Raghawendra Kumar, Dinesh Kumar, Zuber Saiyed, Komal Patel, Labdhi Pandya, Afzal Ansari, Nikha Trivedi, Pranay Shah, Kamlesh J Upadhyay, Sanjay Kapadia, Apurvasinh Puvar, R D Dixit, A M Kadri, Harsh Bakshi, Chaitanya Joshi, Madhvi Joshi
EPI_ISL_514594	B.J. Medical College and Civil hospital, Ahmedabad	Gujarat Biotechnology Research Centre	Zarna Patel, Monika Gandhi, Pinal Trivedi, Maharshi Pandya, Nidhi Patel, Nitin Savaliya, Raghawendra Kumar, Dinesh Kumar, Zuber Saiyed, Komal Patel, Labdhi Pandya, Afzal Ansari, Nikha Trivedi, Pranay Shah, Kamlesh J Upadhyay, Sanjay Kapadia, Apurvasinh Puvar, Janvi Raval, R D Dixit, A M Kadri, Harsh Bakshi, Chaitanya Joshi, Madhvi Joshi

EPI_ISL_514595	B.J. Medical College and Civil hospital, Ahmedabad	Gujarat Biotechnology Research Centre	Monika Gandhi, Pinal Trivedi, Maharshi Pandya, Nidhi Patel, Nitin Savaliya, Raghawendra Kumar, Dinesh Kumar, Zuber Saiyed, Komal Patel, Labdhi Pandya, Afzal Ansari, Nikha Trivedi, Pranay Shah, Kamlesh J Upadhyay, Sanjay Kapadia, Apurvasinh Puvar, Janvi Raval, Zarna Patel, R D Dixit, A M Kadri, Harsh Bakshi, Chaitanya Joshi, Madhvi Joshi
EPI_ISL_514596	B.J. Medical College and Civil hospital, Ahmedabad	Gujarat Biotechnology Research Centre	Pinal Trivedi, Maharshi Pandya, Nidhi Patel, Nitin Savaliya, Raghawendra Kumar, Dinesh Kumar, Zuber Saiyed, Komal Patel, Labdhi Pandya, Afzal Ansari, Nikha Trivedi, Pranay Shah, Kamlesh J Upadhyay, Sanjay Kapadia, Apurvasinh Puvar, Janvi Raval, Zarna Patel, Monika Gandhi, R D Dixit, A M Kadri, Harsh Bakshi, Chaitanya Joshi, Madhvi Joshi
EPI_ISL_514597	B.J. Medical College and Civil hospital, Ahmedabad	Gujarat Biotechnology Research Centre	Maharshi Pandya, Nidhi Patel, Nitin Savaliya, Raghawendra Kumar, Dinesh Kumar, Zuber Saiyed, Komal Patel, Labdhi Pandya, Afzal Ansari, Nikha Trivedi, Pranay Shah, Kamlesh J Upadhyay, Sanjay Kapadia, Apurvasinh Puvar, Janvi Raval, Zarna Patel, Monika Gandhi, Pinal Trivedi, R D Dixit, A M Kadri, Harsh Bakshi, Chaitanya Joshi, Madhvi Joshi
EPI_ISL_514598	B.J. Medical College and Civil hospital, Ahmedabad	Gujarat Biotechnology Research Centre	Nidhi Patel, Nitin Savaliya, Raghawendra Kumar, Dinesh Kumar, Zuber Saiyed, Komal Patel, Labdhi Pandya, Afzal Ansari, Nikha Trivedi, Pranay Shah, Kamlesh J Upadhyay, Sanjay Kapadia, Apurvasinh Puvar, Janvi Raval, Zarna Patel, Monika Gandhi, Pinal Trivedi, Maharshi Pandya, R D Dixit, A M Kadri, Harsh Bakshi, Chaitanya Joshi, Madhvi Joshi
EPI_ISL_514599	B.J. Medical College and Civil hospital, Ahmedabad	Gujarat Biotechnology Research Centre	Nitin Savaliya, Raghawendra Kumar, Dinesh Kumar, Zuber Saiyed, Komal Patel, Labdhi Pandya, Afzal Ansari, Nikha Trivedi, Pranay Shah, Kamlesh J Upadhyay, Sanjay Kapadia, Apurvasinh Puvar, Janvi Raval, Zarna Patel, Monika Gandhi, Pinal Trivedi, Maharshi Pandya, Nidhi Patel, R D Dixit, A M Kadri, Harsh Bakshi, Chaitanya Joshi, Madhvi Joshi
EPI_ISL_514600	B.J. Medical College and Civil hospital, Ahmedabad	Gujarat Biotechnology Research Centre	Raghawendra Kumar, Dinesh Kumar, Zuber Saiyed, Komal Patel, Labdhi Pandya, Afzal Ansari, Nikha Trivedi, Pranay Shah, Kamlesh J Upadhyay, Sanjay Kapadia, Apurvasinh Puvar, Janvi Raval, Zarna Patel, Monika Gandhi, Pinal Trivedi, Maharshi Pandya, Nidhi Patel, Nitin Savaliya, R D Dixit, A M Kadri, Harsh Bakshi, Chaitanya Joshi, Madhvi Joshi
EPI_ISL_514601	B.J. Medical College and Civil hospital, Ahmedabad	Gujarat Biotechnology Research Centre	Dinesh Kumar, Zuber Saiyed, Komal Patel, Labdhi Pandya, Afzal Ansari, Nikha Trivedi, Pranay Shah, Kamlesh J Upadhyay, Sanjay Kapadia, Apurvasinh Puvar, Janvi Raval, Zarna Patel, Monika Gandhi, Pinal Trivedi, Maharshi Pandya, Nidhi Patel, Nitin Savaliya, Raghawendra Kumar, R D Dixit, A M Kadri, Harsh Bakshi, Chaitanya Joshi, Madhvi Joshi
EPI_ISL_514602	B.J. Medical College and Civil hospital, Ahmedabad	Gujarat Biotechnology Research Centre	Zuber Saiyed, Komal Patel, Labdhi Pandya, Afzal Ansari, Nikha Trivedi, Pranay Shah, Kamlesh J Upadhyay, Sanjay Kapadia, Apurvasinh Puvar, Janvi Raval, Zarna Patel, Monika Gandhi, Pinal Trivedi, Maharshi Pandya, Nidhi Patel, Nitin Savaliya, Raghawendra Kumar, Dinesh Kumar, R D Dixit, A M Kadri, Harsh Bakshi, Chaitanya Joshi, Madhvi Joshi
EPI_ISL_514603	B.J. Medical College and Civil hospital, Ahmedabad	Gujarat Biotechnology Research Centre	Komal Patel, Labdhi Pandya, Afzal Ansari, Nikha Trivedi, Pranay Shah, Kamlesh J Upadhyay, Sanjay Kapadia, Apurvasinh Puvar, Janvi Raval, Zarna Patel, Monika Gandhi, Pinal Trivedi, Maharshi Pandya, Nidhi Patel, Nitin Savaliya, Raghawendra Kumar, Dinesh Kumar, Zuber Saiyed, R D Dixit, A M Kadri, Harsh Bakshi, Chaitanya Joshi, Madhvi Joshi
EPI_ISL_514604	B.J. Medical College and Civil hospital, Ahmedabad	Gujarat Biotechnology Research Centre	Labdhi Pandya, Afzal Ansari, Nikha Trivedi, Pranay Shah, Kamlesh J Upadhyay, Sanjay Kapadia, Apurvasinh Puvar, Janvi Raval, Zarna Patel, Monika Gandhi, Pinal Trivedi, Maharshi Pandya, Nidhi Patel, Nitin Savaliya, Raghawendra Kumar, Dinesh Kumar, Zuber Saiyed, Komal Patel, R D Dixit, A M Kadri, Harsh Bakshi, Chaitanya Joshi, Madhvi Joshi
EPI_ISL_514605	B.J. Medical College and Civil hospital, Ahmedabad	Gujarat Biotechnology Research Centre	Afzal Ansari, Nikha Trivedi, Pranay Shah, Kamlesh J Upadhyay, Sanjay Kapadia, Apurvasinh Puvar, Janvi Raval, Zarna Patel, Monika Gandhi, Pinal Trivedi, Maharshi Pandya, Nidhi Patel, Nitin Savaliya, Raghawendra Kumar, Dinesh Kumar, Zuber Saiyed, Komal Patel, Labdhi Pandya, R D Dixit, A M Kadri, Harsh Bakshi, Chaitanya Joshi, Madhvi Joshi
EPI_ISL_514606	B.J. Medical College and Civil hospital, Ahmedabad	Gujarat Biotechnology Research Centre	Nikha Trivedi, Pranay Shah, Kamlesh J Upadhyay, Sanjay Kapadia, Apurvasinh Puvar, Janvi Raval, Zarna Patel, Monika Gandhi, Pinal Trivedi, Maharshi Pandya, Nidhi Patel, Nitin Savaliya, Raghawendra Kumar, Dinesh Kumar, Zuber Saiyed, Komal Patel, Labdhi Pandya, Afzal Ansari, R D Dixit, A M Kadri, Harsh Bakshi, Chaitanya Joshi, Madhvi Joshi
EPI_ISL_514607	B.J. Medical College and Civil hospital, Ahmedabad	Gujarat Biotechnology Research Centre	Pranay Shah, Kamlesh J Upadhyay, Sanjay Kapadia, Apurvasinh Puvar, Janvi Raval, Zarna Patel, Monika Gandhi, Pinal Trivedi, Maharshi Pandya, Nidhi Patel, Nitin Savaliya, Raghawendra Kumar, Dinesh Kumar, Zuber Saiyed, Komal Patel, Labdhi Pandya, Afzal Ansari, Nikha Trivedi, R D Dixit, A M Kadri, Harsh Bakshi, Chaitanya Joshi, Madhvi Joshi
EPI_ISL_514608	B.J. Medical College and Civil hospital, Ahmedabad	Gujarat Biotechnology Research Centre	Kamlesh J Upadhyay, Sanjay Kapadia, Apurvasinh Puvar, Janvi Raval, Zarna Patel, Monika Gandhi, Pinal Trivedi, Maharshi Pandya, Nidhi Patel, Nitin Savaliya, Raghawendra Kumar, Dinesh Kumar, Zuber Saiyed, Komal Patel, Labdhi Pandya, Afzal Ansari, Nikha Trivedi, Pranay Shah, R D Dixit, A M Kadri, Harsh Bakshi, Chaitanya Joshi, Madhvi Joshi
EPI_ISL_514609	B.J. Medical College and Civil hospital, Ahmedabad	Gujarat Biotechnology Research Centre	Sanjay Kapadia, Apurvasinh Puvar, Janvi Raval, Zarna Patel, Monika Gandhi, Pinal Trivedi, Maharshi Pandya, Nidhi Patel, Nitin Savaliya, Raghawendra Kumar, Dinesh Kumar, Zuber Saiyed, Komal Patel, Labdhi Pandya, Afzal Ansari, Nikha Trivedi, Pranay Shah, Kamlesh J Upadhyay, R D Dixit, A M Kadri, Harsh Bakshi, Chaitanya Joshi, Madhvi Joshi
EPI_ISL_514610	GMERS Medical College & Hospital, Gotri, Vadodara	Gujarat Biotechnology Research Centre	Meenakshi Shah, Neena Doshi, Varsha Godbole, Apurvasinh Puvar, Janvi Raval, Zarna Patel, Monika Gandhi, Pinal Trivedi, Maharshi Pandya, Nidhi Patel, Nitin Savaliya, Raghawendra Kumar, Dinesh Kumar, Zuber Saiyed, Komal Patel, Labdhi Pandya, Afzal Ansari, Nikha Trivedi, Pranay Shah, Kamlesh J Upadhyay, Sanjay Kapadia, R D Dixit, A M Kadri, Harsh Bakshi, Chaitanya Joshi, Madhvi Joshi,
EPI_ISL_514611	GMERS Medical College and Hospital, Gandhinagar	Gujarat Biotechnology Research Centre	Seema Bhatt, Gaurishankar Shrimali, Bhavesh Modi, Bharti Rajani, Apurvasinh Puvar, Janvi Raval, Zarna Patel, Monika Gandhi, Pinal Trivedi, Maharshi Pandya, Nidhi Patel, Nitin Savaliya, Raghawendra Kumar, Dinesh Kumar, Zuber Saiyed, Komal Patel, Labdhi Pandya, Afzal Ansari, Nikha Trivedi, Pranay Shah, Kamlesh J Upadhyay, Sanjay Kapadia, R D Dixit, A M Kadri, Harsh Bakshi, Chaitanya Joshi, Madhvi Joshi
EPI_ISL_514612	GMERS Medical College and Hospital, Gandhinagar	Gujarat Biotechnology Research Centre	Gaurishankar Shrimali, Bhavesh Modi, Bharti Rajani, Apurvasinh Puvar, Janvi Raval, Zarna Patel, Monika Gandhi, Pinal Trivedi, Maharshi Pandya, Nidhi Patel, Nitin Savaliya, Raghawendra Kumar, Dinesh Kumar, Zuber Saiyed, Komal Patel, Labdhi Pandya, Afzal Ansari, Nikha Trivedi, Pranay Shah, Kamlesh J Upadhyay, Sanjay Kapadia, Seema Bhatt, R D Dixit, A M Kadri, Harsh Bakshi, Chaitanya Joshi, Madhvi Joshi
EPI_ISL_514613, EPI_ISL_514614, EPI_ISL_514615	NSTU COVID-19 Diagnostic Center	NSU Genome Research Institute (NGRI), North South University	Dr. Muhammad Maqsud Hossain, Aura Rahman, Prof. Firoz Ahmed, Tahrima Huq, Abdus Sadique, Jahidul Alam, Tamanna Afroze, Md Aminul Islam, Prof. Md. Didar-Ul-Alam, Prof. Kazi Nadim Hasan, Prof. Abdul Khaleque, Prof, Hasan Mahmud Reza
EPI_ISL_515466	Discovery DNA	Discovery DNA	Dustin Hittel, Marina Kerr, Leo Dimnik, Desmond Koo, Alice Li, Aneal Khan
EPI_ISL_515520	Hospital Municipal do Tatuape Carmino Caricchio	Instituto Adolfo Lutz, Interdiciplinary Procedures Center, Strategic Laboratory	Claudio Tavares Sacchi, Claudia Regina Gonçalves, Erica Valessa Ramos Gomes
EPI_ISL_515521	Hospital Municipal Dr Waldemar Tebaldi	Instituto Adolfo Lutz, Interdiciplinary Procedures Center, Strategic Laboratory	Claudio Tavares Sacchi, Claudia Regina Gonçalves, Erica Valessa Ramos Gomes
EPI_ISL_515522	UPA 24HS de Itatiba	Instituto Adolfo Lutz, Interdiciplinary Procedures Center, Strategic Laboratory	Claudio Tavares Sacchi, Claudia Regina Gonçalves, Erica Valessa Ramos Gomes
EPI_ISL_515523, EPI_ISL_515524	PS Municipal Dr Lauro Ribas Braga	Instituto Adolfo Lutz, Interdiciplinary Procedures Center, Strategic Laboratory	Claudio Tavares Sacchi, Claudia Regina Gonçalves, Erica Valessa Ramos Gomes
EPI_ISL_515525	National Influenza Center - Instituto Adolfo Lutz	Instituto Adolfo Lutz, Interdiciplinary Procedures Center, Strategic Laboratory	Claudio Tavares Sacchi, Claudia Regina Gonçalves, Erica Valessa Ramos Gomes
EPI_ISL_515526	Hospital Municipal do Tatuape Carmino Caricchio	Instituto Adolfo Lutz, Interdiciplinary Procedures Center, Strategic Laboratory	Claudio Tavares Sacchi, Claudia Regina Gonçalves, Erica Valessa Ramos Gomes
EPI_ISL_515527	Hospital Santa Clara	Instituto Adolfo Lutz, Interdiciplinary Procedures Center, Strategic Laboratory	Claudio Tavares Sacchi, Claudia Regina Gonçalves, Erica Valessa Ramos Gomes
EPI_ISL_515528	Hospital Sao Paulo de Ensino da Unifesp	Instituto Adolfo Lutz, Interdiciplinary Procedures Center, Strategic Laboratory	Claudio Tavares Sacchi, Claudia Regina Gonçalves, Erica Valessa Ramos Gomes

EPI_ISL_515529	Pronto Socorro Municipal Julio Tupy	Instituto Adolfo Lutz, Interdiciplinary Procedures Center, Strategic Laboratory	Claudio Tavares Sacchi, Claudia Regina Gonçalves, Erica Valessa Ramos Gomes
EPI_ISL_515541	Hospital Montemagno	Instituto Adolfo Lutz, Interdiciplinary Procedures Center, Strategic Laboratory	Claudio Tavares Sacchi, Claudia Regina Gonçalves, Erica Valessa Ramos Gomes
EPI_ISL_515542	Vigilância Epidemiológica de Leme	Instituto Adolfo Lutz, Interdiciplinary Procedures Center, Strategic Laboratory	Claudio Tavares Sacchi, Claudia Regina Gonçalves, Erica Valessa Ramos Gomes
EPI_ISL_515543	Serviço de Vigilância Sanitária e Epidemiológica	Instituto Adolfo Lutz, Interdiciplinary Procedures Center, Strategic Laboratory	Claudio Tavares Sacchi, Claudia Regina Gonçalves, Erica Valessa Ramos Gomes
EPI_ISL_515544	Ama Dr Jose Soares Hungria	Instituto Adolfo Lutz, Interdiciplinary Procedures Center, Strategic Laboratory	Claudio Tavares Sacchi, Claudia Regina Gonçalves, Erica Valessa Ramos Gomes
EPI_ISL_515545	Hospital Sao Paulo de Ensino da Unifesp	Instituto Adolfo Lutz, Interdiciplinary Procedures Center, Strategic Laboratory	Claudio Tavares Sacchi, Claudia Regina Gonçalves, Erica Valessa Ramos Gomes
EPI_ISL_515546	Hospital Municipal do Tatuape Carmino Caricchio	Instituto Adolfo Lutz, Interdiciplinary Procedures Center, Strategic Laboratory	Claudio Tavares Sacchi, Claudia Regina Gonçalves, Erica Valessa Ramos Gomes
EPI_ISL_515547	Centro Medico da Policia Militar do Estado de Sao Paulo	Instituto Adolfo Lutz, Interdiciplinary Procedures Center, Strategic Laboratory	Claudio Tavares Sacchi, Claudia Regina Gonçalves, Erica Valessa Ramos Gomes
EPI_ISL_515548	Hospital Municipal Dr. Jose Soares Hungria	Instituto Adolfo Lutz, Interdiciplinary Procedures Center, Strategic Laboratory	Claudio Tavares Sacchi, Claudia Regina Gonçalves, Erica Valessa Ramos Gomes
EPI_ISL_515550	UPA Vila Santa Catarina	Instituto Adolfo Lutz, Interdiciplinary Procedures Center, Strategic Laboratory	Claudio Tavares Sacchi, Claudia Regina Gonçalves, Erica Valessa Ramos Gomes
EPI_ISL_515551, EPI_ISL_515552	Hospital Municipal do Tatuape Carmino Caricchio	Instituto Adolfo Lutz, Interdiciplinary Procedures Center, Strategic Laboratory	Claudio Tavares Sacchi, Claudia Regina Gonçalves, Erica Valessa Ramos Gomes
EPI_ISL_515553	Hospital Municipal Dr. Ignacio Proença de Gouvea	Instituto Adolfo Lutz, Interdiciplinary Procedures Center, Strategic Laboratory	Claudio Tavares Sacchi, Claudia Regina Gonçalves, Erica Valessa Ramos Gomes
EPI_ISL_515554	Pronto Socorro Municipal de Perus	Instituto Adolfo Lutz, Interdiciplinary Procedures Center, Strategic Laboratory	Claudio Tavares Sacchi, Claudia Regina Gonçalves, Erica Valessa Ramos Gomes
EPI_ISL_515555	Hospital Geral de Vila Nova Cachoeirinha	Instituto Adolfo Lutz, Interdiciplinary Procedures Center, Strategic Laboratory	Claudio Tavares Sacchi, Claudia Regina Gonçalves, Erica Valessa Ramos Gomes
EPI_ISL_515557	Hospital Municipal Dr. Moysés Deutsch	Instituto Adolfo Lutz, Interdiciplinary Procedures Center, Strategic Laboratory	Claudio Tavares Sacchi, Claudia Regina Gonçalves, Erica Valessa Ramos Gomes
EPI_ISL_515559, EPI_ISL_515560	Hospital Sao Paulo de Ensino da Unifesp	Instituto Adolfo Lutz, Interdiciplinary Procedures Center, Strategic Laboratory	Claudio Tavares Sacchi, Claudia Regina Gonçalves, Erica Valessa Ramos Gomes
EPI_ISL_515561	Hospital Montemagno	Instituto Adolfo Lutz, Interdiciplinary Procedures Center, Strategic Laboratory	Claudio Tavares Sacchi, Claudia Regina Gonçalves, Erica Valessa Ramos Gomes
EPI_ISL_515562	Hospital Municipal Doutor Alexandre Zaio	Instituto Adolfo Lutz, Interdiciplinary Procedures Center, Strategic Laboratory	Claudio Tavares Sacchi, Claudia Regina Gonçalves, Erica Valessa Ramos Gomes
EPI_ISL_515563	Hospital Municipal Dr. Jose Soares Hungria	Instituto Adolfo Lutz, Interdiciplinary Procedures Center, Strategic Laboratory	Claudio Tavares Sacchi, Claudia Regina Gonçalves, Erica Valessa Ramos Gomes
EPI_ISL_515564	Hosp. Municipal Prof. Dr. Alípio Corrêa Netto	Instituto Adolfo Lutz, Interdiciplinary Procedures Center, Strategic Laboratory	Claudio Tavares Sacchi, Claudia Regina Gonçalves, Erica Valessa Ramos Gomes
EPI_ISL_515565	Hospital do Servidor Público Estadual Francisco Morato de Oliveira	Instituto Adolfo Lutz, Interdiciplinary Procedures Center, Strategic Laboratory	Claudio Tavares Sacchi, Claudia Regina Gonçalves, Erica Valessa Ramos Gomes
EPI_ISL_515566	PS Municipal Dr Lauro Ribas Braga	Instituto Adolfo Lutz, Interdiciplinary Procedures Center, Strategic Laboratory	Claudio Tavares Sacchi, Claudia Regina Gonçalves, Erica Valessa Ramos Gomes
EPI_ISL_515568, EPI_ISL_515569, EPI_ISL_515570, EPI_ISL_515571, EPI_ISL_515572, EPI_ISL_515573, EPI_ISL_515574, EPI_ISL_515575, EPI_ISL_515576, EPI_ISL_515577, EPI_ISL_515578, EPI_ISL_515579, EPI_ISL_515580, EPI_ISL_515581, EPI_ISL_515582, EPI_ISL_515583, EPI_ISL_515584, EPI_ISL_515585, EPI_ISL_515586, EPI_ISL_515587, EPI_ISL_515588, EPI_ISL_515589, EPI_ISL_515590, EPI_ISL_515591, EPI_ISL_515592, EPI_ISL_515593, EPI_ISL_515594, EPI_ISL_515595, EPI_ISL_515596, EPI_ISL_515597, EPI_ISL_515598, EPI_ISL_515599, EPI_ISL_515600, EPI_ISL_515601, EPI_ISL_515602, EPI_ISL_515603, EPI_ISL_515604, EPI_ISL_515605, EPI_ISL_515606, EPI_ISL_515607, EPI_ISL_515608, EPI_ISL_515609, EPI_ISL_515610, EPI_ISL_515611, EPI_ISL_515612, EPI_ISL_515613, EPI_ISL_515614, EPI_ISL_515615, EPI_ISL_515616, EPI_ISL_515617, EPI_ISL_515618, EPI_ISL_515619, EPI_ISL_515620, EPI_ISL_515621, EPI_ISL_515622, EPI_ISL_515623, EPI_ISL_515624, EPI_ISL_515625, EPI_ISL_515626, EPI_ISL_515627, EPI_ISL_515628, EPI_ISL_515629, EPI_ISL_515630, EPI_ISL_515631, EPI_ISL_515632, EPI_ISL_515633, EPI_ISL_515634, EPI_ISL_515635, EPI_ISL_515636, EPI_ISL_515637, EPI_ISL_515638, EPI_ISL_515639, EPI_ISL_515640, EPI_ISL_515641, EPI_ISL_515642, EPI_ISL_515643, EPI_ISL_515644, EPI_ISL_515645, EPI_ISL_515646, EPI_ISL_515647, EPI_ISL_515648, EPI_ISL_515649, EPI_ISL_515650, EPI_ISL_515651, EPI_ISL_515652, EPI_ISL_515653, EPI_ISL_515654, EPI_ISL_515655, EPI_ISL_515656, EPI_ISL_515657, EPI_ISL_515658, EPI_ISL_515659, EPI_ISL_515660, EPI_ISL_515661, EPI_ISL_515662, EPI_ISL_515663, EPI_ISL_515664, EPI_ISL_515665, EPI_ISL_515666, EPI_ISL_515667, EPI_ISL_515668, EPI_ISL_515669, EPI_ISL_515670, EPI_ISL_515671, EPI_ISL_515672, EPI_ISL_515673, EPI_ISL_515674, EPI_ISL_515675, EPI_ISL_515676, EPI_ISL_515677, EPI_ISL_515678, EPI_ISL_515679, EPI_ISL_515680, EPI_ISL_515681, EPI_ISL_515682, EPI_ISL_515683, EPI_ISL_515684, EPI_ISL_515685, EPI_ISL_515686, EPI_ISL_515687, EPI_ISL_515688, EPI_ISL_515689, EPI_ISL_515690, EPI_ISL_515691, EPI_ISL_515692, EPI_ISL_515693, EPI_ISL_515694, EPI_ISL_515695, EPI_ISL_515696, EPI_ISL_515697, EPI_ISL_515698, EPI_ISL_515699, EPI_ISL_515700, EPI_ISL_515701, EPI_ISL_515702, EPI_ISL_515703, EPI_ISL_515704, EPI_ISL_515705, EPI_ISL_515706, EPI_ISL_515707, EPI_ISL_515708, EPI_ISL_515709, EPI_ISL_515710, EPI_ISL_515711, EPI_ISL_515712, EPI_ISL_515713, EPI_ISL_515714, EPI_ISL_515715, EPI_ISL_515716, EPI_ISL_515717, EPI_ISL_515718, EPI_ISL_515719, EPI_ISL_515720, EPI_ISL_515721, EPI_ISL_515722, EPI_ISL_515723, EPI_ISL_515724, EPI_ISL_515725, EPI_ISL_515726, EPI_ISL_515727, EPI_ISL_515728, EPI_ISL_515729, EPI_ISL_515730, EPI_ISL_515731, EPI_ISL_515732, EPI_ISL_515733, EPI_ISL_515734, EPI_ISL_515735, EPI_ISL_515736, EPI_ISL_515737, EPI_ISL_515738, EPI_ISL_515739, EPI_ISL_515740, EPI_ISL_515741, EPI_ISL_515742, EPI_ISL_515743, EPI_ISL_515744, EPI_ISL_515745, EPI_ISL_515746, EPI_ISL_515747, EPI_ISL_515748, EPI_ISL_515749, EPI_ISL_515750, EPI_ISL_515751, EPI_ISL_515752, EPI_ISL_515753, EPI_ISL_515754, EPI_ISL_515755, EPI_ISL_515756, EPI_ISL_515757, EPI_ISL_515758, EPI_ISL_515759, EPI_ISL_515760, EPI_ISL_515761, EPI_ISL_515762, EPI_ISL_515763, EPI_ISL_515764, EPI_ISL_515765, EPI_ISL_515766, EPI_ISL_515767, EPI_ISL_515768, EPI_ISL_515769, EPI_ISL_515770, EPI_ISL_515771, EPI_ISL_515772, EPI_ISL_515773, EPI_ISL_515774, EPI_ISL_515775, EPI_ISL_515776, EPI_ISL_515777, EPI_ISL_515778, EPI_ISL_515779, EPI_ISL_515780, EPI_ISL_515781, EPI_ISL_515782, EPI_ISL_515783, EPI_ISL_515784, EPI_ISL_515785, EPI_ISL_515786, EPI_ISL_515787, EPI_ISL_515788, EPI_ISL_515789, EPI_ISL_515790, EPI_ISL_515791, EPI_ISL_515792, EPI_ISL_515793, EPI_ISL_515794, EPI_ISL_515795, EPI_ISL_515796, EPI_ISL_515797, EPI_ISL_515798, EPI_ISL_515799			
see above	NHLS-IALCH	KRISP, KZN Research Innovation and Sequencing Platform	Giandhari J, Pillay S, Lessells R, Mdlalose K, York D, Khan S, Tegally H, Wilkinson E, de Oliveira T
EPI_ISL_515800, EPI_ISL_515801, EPI_ISL_515802, EPI_ISL_515803, EPI_ISL_515804, EPI_ISL_515805, EPI_ISL_515806, EPI_ISL_515807, EPI_ISL_515808, EPI_ISL_515809, EPI_ISL_515810, EPI_ISL_515811, EPI_ISL_515812, EPI_ISL_515813, EPI_ISL_515814, EPI_ISL_515815, EPI_ISL_515816, EPI_ISL_515817, EPI_ISL_515818, EPI_ISL_515819, EPI_ISL_515820, EPI_ISL_515821, EPI_ISL_515822, EPI_ISL_515823, EPI_ISL_515824, EPI_ISL_515825, EPI_ISL_515826, EPI_ISL_515827, EPI_ISL_515828, EPI_ISL_515829, EPI_ISL_515830, EPI_ISL_515831, EPI_ISL_515832, EPI_ISL_515833, EPI_ISL_515834, EPI_ISL_515835, EPI_ISL_515836, EPI_ISL_515837, EPI_ISL_515838, EPI_ISL_515839, EPI_ISL_515840, EPI_ISL_515841, EPI_ISL_515842, EPI_ISL_515843, EPI_ISL_515844, EPI_ISL_515845, EPI_ISL_515846, EPI_ISL_515847, EPI_ISL_515848, EPI_ISL_515849, EPI_ISL_515850, EPI_ISL_515851, EPI_ISL_515852, EPI_ISL_515853, EPI_ISL_515854, EPI_ISL_515855, EPI_ISL_515856, EPI_ISL_515857, EPI_ISL_515858, EPI_ISL_515859, EPI_ISL_515860, EPI_ISL_515861, EPI_ISL_515862, EPI_ISL_515863, EPI_ISL_515864, EPI_ISL_515865, EPI_ISL_515866, EPI_ISL_515867, EPI_ISL_515868, EPI_ISL_515869, EPI_ISL_515870, EPI_ISL_515871, EPI_ISL_515872, EPI_ISL_515873, EPI_ISL_515874, EPI_ISL_515875, EPI_ISL_515876, EPI_ISL_515877, EPI_ISL_515878, EPI_ISL_515879, EPI_ISL_515880, EPI_ISL_515881, EPI_ISL_515882, EPI_ISL_515883, EPI_ISL_515884, EPI_ISL_515885, EPI_ISL_515886, EPI_ISL_515887, EPI_ISL_515888, EPI_ISL_515889, EPI_ISL_515890, EPI_ISL_515891, EPI_ISL_515892, EPI_ISL_515893			
see above	Medical Disagnostics Services (MDS)	KRISP, KZN Research Innovation and Sequencing Platform	Giandhari J, Pillay S, Lessells R, ChimukangaraB, Mdlalose K, York D, Khan S, Tegally H, Wilkinson E, de Oliveira T
EPI_ISL_515932	DH	Department of Neurovirology, National Institute of Mental Health and Neuroscience (NIMHANS)	Chitra Pattabiraman,Vijayalakshmi Reddy, Harsha PK, Risha Rasheed, Pramada Prasad, Shafeeq S Hameed, Manjunatha Venkataswamy, Anita Desai, Ravi Vasanthapuram
EPI_ISL_515933	BIMS	Department of Neurovirology, National Institute of Mental Health and Neuroscience (NIMHANS)	Chitra Pattabiraman,Vijayalakshmi Reddy, Harsha PK, Risha Rasheed, Pramada Prasad, Shafeeq S Hameed, Manjunatha Venkataswamy, Anita Desai, Ravi Vasanthapuram
EPI_ISL_515934, EPI_ISL_515935, EPI_ISL_515936	DH	Department of Neurovirology, National Institute of Mental Health and Neuroscience (NIMHANS)	Chitra Pattabiraman,Vijayalakshmi Reddy, Harsha PK, Risha Rasheed, Pramada Prasad, Shafeeq S Hameed, Manjunatha Venkataswamy, Anita Desai, Ravi Vasanthapuram
EPI_ISL_515937	BIMS	Department of Neurovirology, National Institute of Mental Health and Neuroscience (NIMHANS)	Chitra Pattabiraman,Vijayalakshmi Reddy, Harsha PK, Risha Rasheed, Pramada Prasad, Shafeeq S Hameed, Manjunatha Venkataswamy, Anita Desai, Ravi Vasanthapuram

EPI_ISL_515938, EPI_ISL_515939, EPI_ISL_515941, EPI_ISL_515942	CV RAMAN HOSPITAL	Department of Neurovirology, National Institute of Mental Health and Neuroscience (NIMHANS)	Chitra Pattabiraman,Vijayalakshmi Reddy, Harsha PK, Risha Rasheed, Pramada Prasad, Shafeeq S Hameed, Manjunatha Venkataswamy, Anita Desai, Ravi Vasanthapuram
EPI_ISL_515943, EPI_ISL_515944	ESIC	Department of Neurovirology, National Institute of Mental Health and Neuroscience (NIMHANS)	Chitra Pattabiraman,Vijayalakshmi Reddy, Harsha PK, Risha Rasheed, Pramada Prasad, Shafeeq S Hameed, Manjunatha Venkataswamy, Anita Desai, Ravi Vasanthapuram
EPI_ISL_515945, EPI_ISL_515946, EPI_ISL_515947, EPI_ISL_515948, EPI_ISL_515949	DH	Department of Neurovirology, National Institute of Mental Health and Neuroscience (NIMHANS)	Chitra Pattabiraman,Vijayalakshmi Reddy, Harsha PK, Risha Rasheed, Pramada Prasad, Shafeeq S Hameed, Manjunatha Venkataswamy, Anita Desai, Ravi Vasanthapuram
EPI_ISL_515950, EPI_ISL_515951, EPI_ISL_515952, EPI_ISL_515953	VICTORIA HOSPITAL	Department of Neurovirology, National Institute of Mental Health and Neuroscience (NIMHANS)	Chitra Pattabiraman,Vijayalakshmi Reddy, Harsha PK, Risha Rasheed, Pramada Prasad, Shafeeq S Hameed, Manjunatha Venkataswamy, Anita Desai, Ravi Vasanthapuram
EPI_ISL_515954, EPI_ISL_515955	DH	Department of Neurovirology, National Institute of Mental Health and Neuroscience (NIMHANS)	Chitra Pattabiraman,Vijayalakshmi Reddy, Harsha PK, Risha Rasheed, Pramada Prasad, Shafeeq S Hameed, Manjunatha Venkataswamy, Anita Desai, Ravi Vasanthapuram
EPI_ISL_515956	SHEKAR HOSPITAL	Department of Neurovirology, National Institute of Mental Health and Neuroscience (NIMHANS)	Chitra Pattabiraman,Vijayalakshmi Reddy, Harsha PK, Risha Rasheed, Pramada Prasad, Shafeeq S Hameed, Manjunatha Venkataswamy, Anita Desai, Ravi Vasanthapuram
EPI_ISL_515957, EPI_ISL_515958, EPI_ISL_515959	DH	Department of Neurovirology, National Institute of Mental Health and Neuroscience (NIMHANS)	Chitra Pattabiraman,Vijayalakshmi Reddy, Harsha PK, Risha Rasheed, Pramada Prasad, Shafeeq S Hameed, Manjunatha Venkataswamy, Anita Desai, Ravi Vasanthapuram
EPI_ISL_515960	JGH	Department of Neurovirology, National Institute of Mental Health and Neuroscience (NIMHANS)	Chitra Pattabiraman,Vijayalakshmi Reddy, Harsha PK, Risha Rasheed, Pramada Prasad, Shafeeq S Hameed, Manjunatha Venkataswamy, Anita Desai, Ravi Vasanthapuram
EPI_ISL_515961, EPI_ISL_515962	DH	Department of Neurovirology, National Institute of Mental Health and Neuroscience (NIMHANS)	Chitra Pattabiraman,Vijayalakshmi Reddy, Harsha PK, Risha Rasheed, Pramada Prasad, Shafeeq S Hameed, Manjunatha Venkataswamy, Anita Desai, Ravi Vasanthapuram
EPI_ISL_515963, EPI_ISL_515964	VICTORIA HOSPITAL	Department of Neurovirology, National Institute of Mental Health and Neuroscience (NIMHANS)	Chitra Pattabiraman,Vijayalakshmi Reddy, Harsha PK, Risha Rasheed, Pramada Prasad, Shafeeq S Hameed, Manjunatha Venkataswamy, Anita Desai, Ravi Vasanthapuram
EPI_ISL_515965	DH	Department of Neurovirology, National Institute of Mental Health and Neuroscience (NIMHANS)	Chitra Pattabiraman,Vijayalakshmi Reddy, Harsha PK, Risha Rasheed, Pramada Prasad, Shafeeq S Hameed, Manjunatha Venkataswamy, Anita Desai, Ravi Vasanthapuram
EPI_ISL_515966, EPI_ISL_515967	VICTORIA HOSPITAL	Department of Neurovirology, National Institute of Mental Health and Neuroscience (NIMHANS)	Chitra Pattabiraman,Vijayalakshmi Reddy, Harsha PK, Risha Rasheed, Pramada Prasad, Shafeeq S Hameed, Manjunatha Venkataswamy, Anita Desai, Ravi Vasanthapuram
EPI_ISL_515968, EPI_ISL_515969, EPI_ISL_515970, EPI_ISL_515971, EPI_ISL_515972, EPI_ISL_515973	MIMS	Department of Neurovirology, National Institute of Mental Health and Neuroscience (NIMHANS)	Chitra Pattabiraman,Vijayalakshmi Reddy, Harsha PK, Risha Rasheed, Pramada Prasad, Shafeeq S Hameed, Manjunatha Venkataswamy, Anita Desai, Ravi Vasanthapuram
EPI_ISL_516075	BIMS	Department of Neurovirology, National Institute of Mental Health and Neuroscience (NIMHANS)	Chitra Pattabiraman,Vijayalakshmi Reddy, Harsha PK, Risha Rasheed, Pramada Prasad, Shafeeq S Hameed, Manjunatha Venkataswamy, Anita Desai, Ravi Vasanthapuram
EPI_ISL_516076, EPI_ISL_516077, EPI_ISL_516078	VICTORIA HOSPITAL	Department of Neurovirology, National Institute of Mental Health and Neuroscience (NIMHANS)	Chitra Pattabiraman,Vijayalakshmi Reddy, Harsha PK, Risha Rasheed, Pramada Prasad, Shafeeq S Hameed, Manjunatha Venkataswamy, Anita Desai, Ravi Vasanthapuram
EPI_ISL_516193	Hospital Universitari Germans Trias i Pujol.	IrsiCaixa AIDS Research Lab	Marc Noguera-Julian, Mariona Parera, Maria Pilar Armengol, Marta Massanella, Ester Ballana, Lidia Ruiz, Nuria Izquierdo, Jorge Carrillo, Roger Paredes, Julia Blanco, Joaquim Segalés, Bonaventura Clotet
EPI_ISL_516608	Instituto de Diagnostico y Referencia Epidemiologicos (INDRE)	Instituto de Diagnostico y Referencia Epidemiologicos (INDRE)	Ernesto Ramirez-Gonzalez, Abril Rodriguez-Maldonado, Claudia Wong-Arambula , Natividad Cruz-Ortiz, Tatiana Nunez-Garcia, Dayanira Arellano-Suarez, Adnan Araiza-Rodriguez, Edgar Mendieta-Condado, Lucia Hernandez-Rivas, Irma Lopez-Martinez, Gisela Barrera-Badillo.
EPI_ISL_516609, EPI_ISL_516610	Instituto de Diagnostico y Referencia Epidemiologicos (INDRE)	Instituto de Diagnostico y Referencia Epidemiologicos (INDRE)	Ernesto Ramirez-Gonzalez, Abril Rodriguez-Maldonado, Claudia Wong-Arambula , Natividad Cruz-Ortiz, Tatiana Nunez-Garcia, Dayanira Arellano-Suarez, Adnan Araiza-Rodriguez, Fabiola Garces-Ayala, Lucia Hernandez-Rivas, Irma Lopez-Martinez, Gisela Barrera-Badillo.
EPI_ISL_516611	Instituto de Diagnostico y Referencia Epidemiologicos (INDRE)	Instituto de Diagnostico y Referencia Epidemiologicos (INDRE)	Gisela Barrera-Badillo , Abril Rodriguez-Maldonado, Claudia Wong-Arambula , Natividad Cruz-Ortiz, Tatiana Nunez-Garcia, Dayanira Arellano-Suarez, Fabiola Garces-Ayala, Edgar Mendieta-Condado, Lucia Hernandez-Rivas, Irma Lopez-Martinez, Ernesto Ramirez-Gonzalez.
EPI_ISL_516612	Instituto de Diagnostico y Referencia Epidemiologicos (INDRE)	Instituto de Diagnostico y Referencia Epidemiologicos (INDRE)	Ernesto Ramirez-Gonzalez, Abril Rodriguez-Maldonado, Claudia Wong-Arambula , Natividad Cruz-Ortiz, Tatiana Nunez-Garcia, Dayanira Arellano-Suarez, Adnan Araiza-Rodriguez, Fabiola Garces-Ayala, Lucia Hernandez-Rivas, Irma Lopez-Martinez, Gisela Barrera-Badillo.
EPI_ISL_516613	Instituto de Diagnostico y Referencia Epidemiologicos (INDRE)	Instituto de Diagnostico y Referencia Epidemiologicos (INDRE)	Ernesto Ramirez-Gonzalez, Abril Rodriguez-Maldonado, Claudia Wong-Arambula , Natividad Cruz-Ortiz, Tatiana Nunez-Garcia, Dayanira Arellano-Suarez, Adnan Araiza-Rodriguez, Edgar Mendieta-Condado, Lucia Hernandez-Rivas, Irma Lopez-Martinez, Gisela Barrera-Badillo.
EPI_ISL_516614, EPI_ISL_516615, EPI_ISL_516616, EPI_ISL_516617	Instituto de Diagnostico y Referencia Epidemiologicos (INDRE)	Instituto de Diagnostico y Referencia Epidemiologicos (INDRE)	Ernesto Ramirez-Gonzalez, Abril Rodriguez-Maldonado, Claudia Wong-Arambula , Natividad Cruz-Ortiz, Tatiana Nunez-Garcia, Dayanira Arellano-Suarez, Adnan Araiza-Rodriguez, Fabiola Garces-Ayala, Lucia Hernandez-Rivas, Irma Lopez-Martinez, Gisela Barrera-Badillo.
EPI_ISL_516618, EPI_ISL_516619	Instituto de Diagnostico y Referencia Epidemiologicos (INDRE)	Instituto de Diagnostico y Referencia Epidemiologicos (INDRE)	Gisela Barrera-Badillo , Abril Rodriguez-Maldonado, Claudia Wong-Arambula , Natividad Cruz-Ortiz, Tatiana Nunez-Garcia, Dayanira Arellano-Suarez, Fabiola Garces-Ayala, Edgar Mendieta-Condado, Lucia Hernandez-Rivas, Irma Lopez-Martinez, Ernesto Ramirez-Gonzalez.
EPI_ISL_516620, EPI_ISL_516621	Instituto de Diagnostico y Referencia Epidemiologicos (INDRE)	Instituto de Diagnostico y Referencia Epidemiologicos (INDRE)	Gisela Barrera-Badillo , Abril Rodriguez-Maldonado, Claudia Wong-Arambula , Natividad Cruz-Ortiz, Tatiana Nunez-Garcia, Dayanira Arellano-Suarez, Adnan Araiza-Rodriguez, Edgar Mendieta-Condado, Lucia Hernandez-Rivas, Irma Lopez-Martinez, Ernesto Ramirez-Gonzalez.
EPI_ISL_516622	Instituto de Diagnostico y Referencia Epidemiologicos (INDRE)	Instituto de Diagnostico y Referencia Epidemiologicos (INDRE)	Gisela Barrera-Badillo , Abril Rodriguez-Maldonado, Claudia Wong-Arambula , Natividad Cruz-Ortiz, Tatiana Nunez-Garcia, Dayanira Arellano-Suarez, Fabiola Garces-Ayala, Edgar Mendieta-Condado, Lucia Hernandez-Rivas, Irma Lopez-Martinez, Ernesto Ramirez-Gonzalez.
EPI_ISL_516623	Instituto de Diagnostico y Referencia Epidemiologicos (INDRE)	Instituto de Diagnostico y Referencia Epidemiologicos (INDRE)	Gisela Barrera-Badillo , Abril Rodriguez-Maldonado, Claudia Wong-Arambula , Natividad Cruz-Ortiz, Tatiana Nunez-Garcia, Dayanira Arellano-Suarez, Adnan Araiza-Rodriguez, Edgar Mendieta-Condado, Lucia Hernandez-Rivas, Irma Lopez-Martinez, Ernesto Ramirez-Gonzalez.
EPI_ISL_516624, EPI_ISL_516625	Instituto de Diagnostico y Referencia Epidemiologicos (INDRE)	Instituto de Diagnostico y Referencia Epidemiologicos (INDRE)	Gisela Barrera-Badillo , Abril Rodriguez-Maldonado, Claudia Wong-Arambula , Natividad Cruz-Ortiz, Tatiana Nunez-Garcia, Dayanira Arellano-Suarez, Fabiola Garces-Ayala, Edgar Mendieta-Condado, Lucia Hernandez-Rivas, Irma Lopez-Martinez, Ernesto Ramirez-Gonzalez.
EPI_ISL_516748, EPI_ISL_516749, EPI_ISL_516750, EPI_ISL_516751, EPI_ISL_516752, EPI_ISL_516753, EPI_ISL_516754, EPI_ISL_516755, EPI_ISL_516756, EPI_ISL_516757, EPI_ISL_516758, EPI_ISL_516759, EPI_ISL_516760, EPI_ISL_516761, EPI_ISL_516762, EPI_ISL_516763, EPI_ISL_516764, EPI_ISL_516765, EPI_ISL_516766, EPI_ISL_516767, EPI_ISL_516768, EPI_ISL_516769, EPI_ISL_516770, EPI_ISL_516771, EPI_ISL_516772, EPI_ISL_516773, EPI_ISL_516774, EPI_ISL_516775, EPI_ISL_516776, EPI_ISL_516777, EPI_ISL_516778, EPI_ISL_516779, EPI_ISL_516780, EPI_ISL_516781, EPI_ISL_516782			
see above	van Bakel Laboratory, Genetics and Genomics Sciences, Icahn School of Medicine at Mount Sinai	van Bakel Laboratory, Genetics and Genomics Sciences, Icahn School of Medicine at Mount Sinai	Andrew G. Letizia, Irene Ramos, Ajay Obla, Carl Goforth, Dawn Weir, Yongchao Ge, Marcas M. Bamman, Jayeeta Dutta, Ethan Ellis, Luis Estrella, Mary-Catherine George, Ana S. Gonzalez-Reiche, Darnell Graham, Adriana van de Guchte, Ramiro Gutierrez, Franca Jones, Aspasia Kalomoiri, Rhonda Lizewski, Stephen Lizewski, Jan Marayag, Nada Marjanovic, Eugene V. Millar, Venugopalan Nair, German Nudelman, Edgar Nunez, Brian Pike, James Regeimbal, Stas Rirak , Ernesto Santa Ana, Rachel S. Gelernter Sealfon, Robert Sebra, Mark Simons, Alessandra Soares-Schanoski, Michael Termini, Sindhu Vangeti, Carlos Williams, Harm van Bakel, Stuart C. Sealfon
EPI_ISL_516800	Rumah Sakit Akademik Universitas Gadjah Mada	Genetics Working Group (Pokja Genetik) Faculty of Medicine, Public Health and Nursing Universitas Gadjah Mada (FK-KMK UGM); Disease Investigation Center Wates Ministry of Agriculture Indonesia; Department of Microbiology FK-KMK UGM; Laboratorium Diagnostik Yayasan Tahija World Mosquito Program (WMP) Yogyakarta Center for Tropical Medicine FK-KMK UGM; Integrated Research center FK-KMK UGM; Department of Computer Science and Electronics FMIPA UGM	Gunadi, Hendra Wibawa, . Marcellus, Mohamad S. Hakim, Edwin W. Daniwijaya, Ludhang P. Rizki, Endah Supriyati, Eggi Arguni, Titik Nuryastuti, Tri Wibawa, Dwi AA Nugrahaningsih, Afiahayati, Siswanto, Alvin S. Kalim, Desyifa Mursalin
EPI_ISL_516801, EPI_ISL_516802,	Department of Laboratory Medicine, Tan Tock Seng Hospital	Department of Laboratory Medicine, Tan Tock Seng Hospital	Chen YYC, Zair X, Li C, Tang WY, Maurer-Stroh S, Barkham TMS, Nagarajan N, Sessions OM

EPI_ISL_516803, EPI_ISL_516804, EPI_ISL_516805			
EPI_ISL_516806	Rumah Sakit PKU Gamping	Genetics Working Group (Pokja Genetik) Faculty of Medicine, Public Health and Nursing Universitas Gadjah Mada (FK-KMK UGM); Disease Investigation Center Wates Ministry of Agriculture Indonesia; Department of Microbiology FK-KMK UGM; Laboratorium Diagnostik Yayasan Tahija World Mosquito Program (WMP) Yogyakarta Center for Tropical Medicine FK-KMK UGM; Integrated Research center FK-KMK UGM; Department of Computer Science and Electronics FMIPA UGM	Gunadi, Hendra Wibawa, . Marcellus, Mohamad S. Hakim, Edwin W. Daniwijaya, Ludhang P. Rizki, Endah Supriyati, Eggi Arguni, Titik Nuryastuti, Tri Wibawa, Dwi AA Nugrahaningsih, . Afiahayati , . Siswanto, Ardorisye Saptaty Fornia, Kemala Athollah
EPI_ISL_516829	RSUD Nyi Ageng Serang	Genetics Working Group (Pokja Genetik) Faculty of Medicine, Public Health and Nursing Universitas Gadjah Mada (FK-KMK UGM), Disease Investigation Center Wates Ministry of Agriculture Indonesia, Department of Microbiology FK-KMK UGM, Laboratorium Diagnostik Yayasan Tahija World Mosquito Program (WMP) Yogyakarta Center for Tropical Medicine FK-KMK UGM, Integrated Research Center FK-KMK UGM, Department of Computer Science and Electronics FMIPA UGM	Gunadi, Hendra Wibawa, . Marcellus, Mohamad S. Hakim, Edwin W. Daniwijaya, Ludhang P. Rizki, Endah Supriyati, Eggi Arguni, Titik Nuryastuti, Tri Wibawa, Dwi AA Nugrahaningsih, Afiahayati , . Siswanto, Beby Dewi Sartika, Dyah Ayu Puspitarani
EPI_ISL_516934, EPI_ISL_516936, EPI_ISL_516938	Nicolae Testemitanu State University of Medicine and Pharmacy	International Centre for Genetic Engineering and Biotechnology (ICGEB) and ARGO Open Lab Platform for Genome Sequencing	Ulinici M, Licastro D, Dal Monego S, Rajasekharan S, Marcello A
EPI_ISL_516940, EPI_ISL_516941, EPI_ISL_516942, EPI_ISL_516943, EPI_ISL_516944, EPI_ISL_516945, EPI_ISL_516946, EPI_ISL_516947, EPI_ISL_516948, EPI_ISL_516949, EPI_ISL_516950, EPI_ISL_516951, EPI_ISL_516952, EPI_ISL_516953, EPI_ISL_516954, EPI_ISL_516955, EPI_ISL_516956, EPI_ISL_516957, EPI_ISL_516958, EPI_ISL_516959, EPI_ISL_516960, EPI_ISL_516961, EPI_ISL_516962, EPI_ISL_516963, EPI_ISL_516964, EPI_ISL_516965, EPI_ISL_516966, EPI_ISL_516967, EPI_ISL_516968, EPI_ISL_516969, EPI_ISL_516970, EPI_ISL_516971, EPI_ISL_516972, EPI_ISL_516973, EPI_ISL_516974, EPI_ISL_516975, EPI_ISL_516976, EPI_ISL_516977, EPI_ISL_516978, EPI_ISL_516979, EPI_ISL_516980, EPI_ISL_516981, EPI_ISL_516982, EPI_ISL_516983, EPI_ISL_516984, EPI_ISL_516985, EPI_ISL_516986			
see above	King Georges Medical University	CSIR-National Botanical Research Institute	Priti Prasad, Shantanu Prakash, Kishan Sahu, Babita Singh, Suruchi Shukla, Hricha Mishra, Danish Nasar Khan , Om Prakash, MLB Bhatt, SK Barik, Mehar H.Asif,Samir V. Sawant,Amita Jain, Sumit Kr. Bag
EPI_ISL_517996, EPI_ISL_517997, EPI_ISL_517998, EPI_ISL_517999, EPI_ISL_518000, EPI_ISL_518001, EPI_ISL_518002, EPI_ISL_518003, EPI_ISL_518004, EPI_ISL_518005, EPI_ISL_518006, EPI_ISL_518007, EPI_ISL_518008, EPI_ISL_518009, EPI_ISL_518010, EPI_ISL_518011, EPI_ISL_518012, EPI_ISL_518013, EPI_ISL_518014, EPI_ISL_518015, EPI_ISL_518016, EPI_ISL_518017			
see above	Singapore General Hospital	Department of Microbiology	Nurdyana Abdul Rahman, Kun Lee Lim, Chenhao Li, Kian Sing Chan, Lynette Oon, Kern Rei Chng, Niranjan Nagarajan, Karrie Ko
EPI_ISL_518033, EPI_ISL_518034, EPI_ISL_518035, EPI_ISL_518036, EPI_ISL_518037, EPI_ISL_518038, EPI_ISL_518039, EPI_ISL_518040, EPI_ISL_518041, EPI_ISL_518042, EPI_ISL_518043, EPI_ISL_518044, EPI_ISL_518045, EPI_ISL_518046, EPI_ISL_518047, EPI_ISL_518048, EPI_ISL_518049, EPI_ISL_518050, EPI_ISL_518051, EPI_ISL_518052, EPI_ISL_518053			
see above	NHLS-IALCH	KRISP, KZN Research Innovation and Sequencing Platform	Giandhari J, Pillay S, Lessells R, Mdlalose K, York D, Khan S, Tegally H, Wilkinson E, de Oliveira T
EPI_ISL_518819	Qadr Hospital, Tangerang, Banten	Biosafety Level-3 Laboratory, Indonesian Institute of Sciences (LIPI)	Anik Budhi Dharmayanthi, Syam Budi Iryanto, Andri Wardiana, Anggia Prasetyoputri, Isa Nuryana, Ade Andriani, Ario Betha Juanssilfero, Asep M Ridwanuloh, Ahmad Fathoni, Rifki Sadikin, Ratih Asmana Ningrum, Wien Kusharyoto, Puspita Lisdiyanti
EPI_ISL_522350	KU Leuven, Rega Institute, Clinical and Epidemiological Virology	KU Leuven, Rega Institute, Clinical and Epidemiological Virology	Tony Wawina-Bokalanga, Joan Marti-Carerras, Bert Vanmechelen, Piet Maes
EPI_ISL_522406	Universidad Iberoamericana (UNIBE)	International Centre for Genetic Engineering and Biotechnology (ICGEB)and ARGO Open Lab Platform	Robert Paulino-Ramirez, Eileen Riego, Alejandro Vallejo, Victor Calderon, Leandro Tapia, Danilo Licastro, Simeone Dal Monego, Sreejith Rajasekharan, and Alessandro Marcello.
EPI_ISL_522547, EPI_ISL_522548	Platforme CYROI	UMR PIMIT Université de La Réunion	David Wilkinson, Camille Lebarbenchon, Patrick Mavingui
EPI_ISL_522872	Instituto Nacional de Medicina Genómica	Instituto Nacional de Medicina Genómica	Hidalgo-Miranda A, Mendoza-Vargas A, Reyes-Grajeda JP, Cisneros-Villanueva M, Cedro-Tanda A, Hurtado-Cordova E, Peñaloza-Figueroa F, Herrera-Montalvo LA
EPI_ISL_522873, EPI_ISL_522874, EPI_ISL_522875, EPI_ISL_522876, EPI_ISL_522877	Instituto Nacional de Medicina Genomica	Instituto Nacional de Medicina Genomica	Hidalgo-Miranda A, Mendoza-Vargas A, Reyes-Grajeda JP, Cisneros-Villanueva M, Cedro-Tanda A, Hurtado-Cordova E, Peñaloza-Figueroa F, Herrera-Montalvo LA
EPI_ISL_522878	Instituto Nacional de Medicina Genomica	Instituto Nacional de Medicina Genomica	Hidalgo-Miranda A, Mendoza-Vargas A, Reyes-Grajeda JP, Cisneros-Villanueva M, Hurtado-Cordova E, Cedro-Tanda A, Peñaloza-Figueroa F, Herrera-Montalvo LA
EPI_ISL_522879, EPI_ISL_522880, EPI_ISL_522940, EPI_ISL_522941, EPI_ISL_522942, EPI_ISL_522978, EPI_ISL_522979, EPI_ISL_522980, EPI_ISL_522981, EPI_ISL_522982, EPI_ISL_522983, EPI_ISL_522984, EPI_ISL_522985, EPI_ISL_522986, EPI_ISL_523500			
see above	Instituto Nacional de Medicina Genómica	Instituto Nacional de Medicina Genómica	Hidalgo-Miranda A, Mendoza-Vargas A, Reyes-Grajeda JP, Cisneros-Villanueva M, Cedro-Tanda A, Hurtado-Cordova E, Peñaloza-Figueroa F, Herrera-Montalvo LA
EPI_ISL_523811, EPI_ISL_523812	Universidad Iberoamericana, Instituto de Medicina Tropical & Salud Global	International Centre for Genetic Engineering and Biotechnology (ICGEB) and ARGO Open Lab Platform	Robert Paulino-Ramirez, Eileen Riego, Alejandro Vallejo Degaudenzi, Victor Virgilio Calderon, Leandro Tapia, Danilo Licastro, Simeone Dal Monego, Sreejith Rajasekharan and Alessandro Marcello.
EPI_ISL_523955	Hospital Municipal do Tatuape Carmino Caricchio	Instituto Adolfo Lutz, Interdiciplinary Procedures Center, Strategic Laboratory	Claudio Tavares Sacchi, Claudia Regina Gonçalves, Erica Valessa Ramos Gomes
EPI_ISL_523956	Hospital Regional de Assis	Instituto Adolfo Lutz, Interdiciplinary Procedures Center, Strategic Laboratory	Claudio Tavares Sacchi, Claudia Regina Gonçalves, Erica Valessa Ramos Gomes
EPI_ISL_523957	Hospital Itamaraty	Instituto Adolfo Lutz, Interdiciplinary Procedures Center, Strategic Laboratory	Claudio Tavares Sacchi, Claudia Regina Gonçalves, Erica Valessa Ramos Gomes
EPI_ISL_523958, EPI_ISL_523959	Pronto Socorro Municipal de Perus	Instituto Adolfo Lutz, Interdiciplinary Procedures Center, Strategic Laboratory	Claudio Tavares Sacchi, Claudia Regina Gonçalves, Erica Valessa Ramos Gomes
EPI_ISL_523963	UBS Vila Silvia	Instituto Adolfo Lutz, Interdiciplinary Procedures Center, Strategic Laboratory	Claudio Tavares Sacchi, Claudia Regina Gonçalves, Erica Valessa Ramos Gomes
EPI_ISL_523965	Hospital do Servidor Público Estadual Francisco Morato de Oliveira	Instituto Adolfo Lutz, Interdiciplinary Procedures Center, Strategic Laboratory	Claudio Tavares Sacchi, Claudia Regina Gonçalves, Erica Valessa Ramos Gomes
EPI_ISL_523967	Hospital Sancta Maggiore	Instituto Adolfo Lutz, Interdiciplinary Procedures Center, Strategic Laboratory	Claudio Tavares Sacchi, Claudia Regina Gonçalves, Erica Valessa Ramos Gomes
EPI_ISL_523969	Hospital Sao Paulo de Ensino da Unifesp	Instituto Adolfo Lutz, Interdiciplinary Procedures Center, Strategic Laboratory	Claudio Tavares Sacchi, Claudia Regina Gonçalves, Erica Valessa Ramos Gomes
EPI_ISL_523970	Conjunto Hospitalar do Mandaqui	Instituto Adolfo Lutz, Interdiciplinary Procedures Center, Strategic Laboratory	Claudio Tavares Sacchi, Claudia Regina Gonçalves, Erica Valessa Ramos Gomes
EPI_ISL_523971	Hospital Geral Santa Marcelina	Instituto Adolfo Lutz, Interdiciplinary Procedures Center, Strategic Laboratory	Claudio Tavares Sacchi, Claudia Regina Gonçalves, Erica Valessa Ramos Gomes

EPI_ISL_523972	Hospital do Servidor Público Estadual Francisco Morato de Oliveira	Instituto Adolfo Lutz, Interdiciplinary Procedures Center, Strategic Laboratory	Claudio Tavares Sacchi, Claudia Regina Gonçalves, Erica Valessa Ramos Gomes
EPI_ISL_523973	PS Municipal Dona Maria Antonieta Ferreira de Barros	Instituto Adolfo Lutz, Interdiciplinary Procedures Center, Strategic Laboratory	Claudio Tavares Sacchi, Claudia Regina Gonçalves, Erica Valessa Ramos Gomes
EPI_ISL_523974	Hospital Municipal do Tatuape Carmino Caricchio	Instituto Adolfo Lutz, Interdiciplinary Procedures Center, Strategic Laboratory	Claudio Tavares Sacchi, Claudia Regina Gonçalves, Erica Valessa Ramos Gomes
EPI_ISL_523975	UPA Tito Lopes	Instituto Adolfo Lutz, Interdiciplinary Procedures Center, Strategic Laboratory	Claudio Tavares Sacchi, Claudia Regina Gonçalves, Erica Valessa Ramos Gomes
EPI_ISL_523976	Hospital Municipal do Tatuape Carmino Caricchio	Instituto Adolfo Lutz, Interdiciplinary Procedures Center, Strategic Laboratory	Claudio Tavares Sacchi, Claudia Regina Gonçalves, Erica Valessa Ramos Gomes
EPI_ISL_523977	Hosp. Municipal Prof. Dr. Alípio Corrêa Netto	Instituto Adolfo Lutz, Interdiciplinary Procedures Center, Strategic Laboratory	Claudio Tavares Sacchi, Claudia Regina Gonçalves, Erica Valessa Ramos Gomes
EPI_ISL_523978	Hospital do Servidor Público Estadual Francisco Morato de Oliveira	Instituto Adolfo Lutz, Interdiciplinary Procedures Center, Strategic Laboratory	Claudio Tavares Sacchi, Claudia Regina Gonçalves, Erica Valessa Ramos Gomes
EPI_ISL_523980	UPA Tito Lopes	Instituto Adolfo Lutz, Interdiciplinary Procedures Center, Strategic Laboratory	Claudio Tavares Sacchi, Claudia Regina Gonçalves, Erica Valessa Ramos Gomes
EPI_ISL_523981	Hospital Sao Paulo de Ensino da Unifesp	Instituto Adolfo Lutz, Interdiciplinary Procedures Center, Strategic Laboratory	Claudio Tavares Sacchi, Claudia Regina Gonçalves, Erica Valessa Ramos Gomes
EPI_ISL_523982	Hospital do Servidor Público Estadual Francisco Morato de Oliveira	Instituto Adolfo Lutz, Interdiciplinary Procedures Center, Strategic Laboratory	Claudio Tavares Sacchi, Claudia Regina Gonçalves, Erica Valessa Ramos Gomes
EPI_ISL_523983	UPA Campo Limpo	Instituto Adolfo Lutz, Interdiciplinary Procedures Center, Strategic Laboratory	Claudio Tavares Sacchi, Claudia Regina Gonçalves, Erica Valessa Ramos Gomes
EPI_ISL_523984	Ama Dr Jose Soares Hungria	Instituto Adolfo Lutz, Interdiciplinary Procedures Center, Strategic Laboratory	Claudio Tavares Sacchi, Claudia Regina Gonçalves, Erica Valessa Ramos Gomes
EPI_ISL_523985	Hospital Municipal Dr. Benedicto Montenegro	Instituto Adolfo Lutz, Interdiciplinary Procedures Center, Strategic Laboratory	Claudio Tavares Sacchi, Claudia Regina Gonçalves, Erica Valessa Ramos Gomes
EPI_ISL_523986	Ama Dr Jose Soares Hungria	Instituto Adolfo Lutz, Interdiciplinary Procedures Center, Strategic Laboratory	Claudio Tavares Sacchi, Claudia Regina Gonçalves, Erica Valessa Ramos Gomes
EPI_ISL_523988	Hospital Sao Paulo de Ensino da Unifesp	Instituto Adolfo Lutz, Interdiciplinary Procedures Center, Strategic Laboratory	Claudio Tavares Sacchi, Claudia Regina Gonçalves, Erica Valessa Ramos Gomes
EPI_ISL_523989	AMA Jardim Joamar	Instituto Adolfo Lutz, Interdiciplinary Procedures Center, Strategic Laboratory	Claudio Tavares Sacchi, Claudia Regina Gonçalves, Erica Valessa Ramos Gomes
EPI_ISL_523990	AMA Jardim Peri	Instituto Adolfo Lutz, Interdiciplinary Procedures Center, Strategic Laboratory	Claudio Tavares Sacchi, Claudia Regina Gonçalves, Erica Valessa Ramos Gomes
EPI_ISL_523991, EPI_ISL_523992	Hospital Municipal Carmen Prudente	Instituto Adolfo Lutz, Interdiciplinary Procedures Center, Strategic Laboratory	Claudio Tavares Sacchi, Claudia Regina Gonçalves, Erica Valessa Ramos Gomes
EPI_ISL_523993	UPA Campo Limpo	Instituto Adolfo Lutz, Interdiciplinary Procedures Center, Strategic Laboratory	Claudio Tavares Sacchi, Claudia Regina Gonçalves, Erica Valessa Ramos Gomes
EPI_ISL_524445, EPI_ISL_524446, EPI_ISL_524447, EPI_ISL_524448, EPI_ISL_524449	Singapore General Hospital	Department of Microbiology	Nurdyana Abdul Rahman, Kun Lee Lim, Chenhao Li, Kian Sing Chan, Lynette Oon, Kern Rei Chng, Niranjan Nagarajan, Karrie Ko
EPI_ISL_524462	Hospital Metropolitano	Instituto Adolfo Lutz, Interdiciplinary Procedures Center, Strategic Laboratory	Claudio Tavares Sacchi, Claudia Regina Gonçalves, Erica Valessa Ramos Gomes
EPI_ISL_524463	Hospital Regional de Cotia	Instituto Adolfo Lutz, Interdiciplinary Procedures Center, Strategic Laboratory	Claudio Tavares Sacchi, Claudia Regina Gonçalves, Erica Valessa Ramos Gomes
EPI_ISL_524464	Santa Casa de Santa Isabel	Instituto Adolfo Lutz, Interdiciplinary Procedures Center, Strategic Laboratory	Claudio Tavares Sacchi, Claudia Regina Gonçalves, Erica Valessa Ramos Gomes
EPI_ISL_524465	PS Municipal Dr. Caetano Virgílio Neto	Instituto Adolfo Lutz, Interdiciplinary Procedures Center, Strategic Laboratory	Claudio Tavares Sacchi, Claudia Regina Gonçalves, Erica Valessa Ramos Gomes
EPI_ISL_524466	PS Municipal Dr Lauro Ribas Braga	Instituto Adolfo Lutz, Interdiciplinary Procedures Center, Strategic Laboratory	Claudio Tavares Sacchi, Claudia Regina Gonçalves, Erica Valessa Ramos Gomes
EPI_ISL_524467	Hospital Municipal Dr. Moysés Deutsch	Instituto Adolfo Lutz, Interdiciplinary Procedures Center, Strategic Laboratory	Claudio Tavares Sacchi, Claudia Regina Gonçalves, Erica Valessa Ramos Gomes
EPI_ISL_524468	Hospital Municipal Vereador Jose Storopolli	Instituto Adolfo Lutz, Interdiciplinary Procedures Center, Strategic Laboratory	Claudio Tavares Sacchi, Claudia Regina Gonçalves, Erica Valessa Ramos Gomes
EPI_ISL_524469	Santa Casa de Misericordia de Sao Paulo	Instituto Adolfo Lutz, Interdiciplinary Procedures Center, Strategic Laboratory	Claudio Tavares Sacchi, Claudia Regina Gonçalves, Erica Valessa Ramos Gomes
EPI_ISL_524470	Hospital do Servidor Público Estadual Francisco Morato de Oliveira	Instituto Adolfo Lutz, Interdiciplinary Procedures Center, Strategic Laboratory	Claudio Tavares Sacchi, Claudia Regina Gonçalves, Erica Valessa Ramos Gomes
EPI_ISL_524713	B.J. Medical College and Civil hospital, Ahmedabad	Gujarat Biotechnology Research Centre	Apurvasinh Puvar, Janvi Raval, Zarna Patel, Monika Gandhi, Pinal Trivedi, Maharshi Pandya, Nidhi Patel, Nitin Savaliya, Raghawendra Kumar, Dinesh Kumar, Zuber Saiyed, Komal Patel, Labdhi Pandya, Afzal Ansari, Nikha Trivedi, Pranay Shah, Kamlesh J Upadhyay, Sanjay Kapadia, R D Dixit, A M Kadri, Harsh Bakshi, Chaitanya Joshi, Madhvi Joshi
EPI_ISL_524714	B.J. Medical College and Civil hospital, Ahmedabad	Gujarat Biotechnology Research Centre	Janvi Raval, Zarna Patel, Monika Gandhi, Pinal Trivedi, Maharshi Pandya, Nidhi Patel, Nitin Savaliya, Raghawendra Kumar, Dinesh Kumar, Zuber Saiyed, Komal Patel, Labdhi Pandya, Afzal Ansari, Nikha Trivedi, Pranay Shah, Kamlesh J Upadhyay, Sanjay Kapadia, Apurvasinh Puvar, R D Dixit, A M Kadri, Harsh Bakshi, Chaitanya Joshi, Madhvi Joshi
EPI_ISL_524715	B.J. Medical College and Civil hospital, Ahmedabad	Gujarat Biotechnology Research Centre	Zarna Patel, Monika Gandhi, Pinal Trivedi, Maharshi Pandya, Nidhi Patel, Nitin Savaliya, Raghawendra Kumar, Dinesh Kumar, Zuber Saiyed, Komal Patel, Labdhi Pandya, Afzal Ansari, Nikha Trivedi, Pranay Shah, Kamlesh J Upadhyay, Sanjay Kapadia, Apurvasinh Puvar, Janvi Raval, R D Dixit, A M Kadri, Harsh Bakshi, Chaitanya Joshi, Madhvi Joshi
EPI_ISL_524716	B.J. Medical College and Civil hospital, Ahmedabad	Gujarat Biotechnology Research Centre	Monika Gandhi, Pinal Trivedi, Maharshi Pandya, Nidhi Patel, Nitin Savaliya, Raghawendra Kumar, Dinesh Kumar, Zuber Saiyed, Komal Patel, Labdhi Pandya, Afzal Ansari, Nikha Trivedi, Pranay Shah, Kamlesh J Upadhyay, Sanjay Kapadia, Apurvasinh Puvar, Janvi Raval, Zarna Patel, R D Dixit, A M Kadri, Harsh Bakshi, Chaitanya Joshi, Madhvi Joshi

EPI_ISL_524717	B.J. Medical College and Civil hospital, Ahmedabad	Gujarat Biotechnology Research Centre	Pinal Trivedi, Maharshi Pandya, Nidhi Patel, Nitin Savaliya, Raghawendra Kumar, Dinesh Kumar, Zuber Saiyed, Komal Patel, Labdhi Pandya, Afzal Ansari, Nikha Trivedi, Pranay Shah, Kamlesh J Upadhyay, Sanjay Kapadia, Apurvasinh Puvar, Janvi Raval, Zarna Patel, Monika Gandhi, R D Dixit, A M Kadri, Harsh Bakshi, Chaitanya Joshi, Madhvi Joshi
EPI_ISL_524718	B.J. Medical College and Civil hospital, Ahmedabad	Gujarat Biotechnology Research Centre	Maharshi Pandya, Nidhi Patel, Nitin Savaliya, Raghawendra Kumar, Dinesh Kumar, Zuber Saiyed, Komal Patel, Labdhi Pandya, Afzal Ansari, Nikha Trivedi, Pranay Shah, Kamlesh J Upadhyay, Sanjay Kapadia, Apurvasinh Puvar, Janvi Raval, Zarna Patel, Monika Gandhi, Pinal Trivedi, R D Dixit, A M Kadri, Harsh Bakshi, Chaitanya Joshi, Madhvi Joshi
EPI_ISL_524719	B.J. Medical College and Civil hospital, Ahmedabad	Gujarat Biotechnology Research Centre	Nidhi Patel, Nitin Savaliya, Raghawendra Kumar, Dinesh Kumar, Zuber Saiyed, Komal Patel, Labdhi Pandya, Afzal Ansari, Nikha Trivedi, Pranay Shah, Kamlesh J Upadhyay, Sanjay Kapadia, Apurvasinh Puvar, Janvi Raval, Zarna Patel, Monika Gandhi, Pinal Trivedi, Maharshi Pandya, R D Dixit, A M Kadri, Harsh Bakshi, Chaitanya Joshi, Madhvi Joshi
EPI_ISL_524720	B.J. Medical College and Civil hospital, Ahmedabad	Gujarat Biotechnology Research Centre	Nitin Savaliya, Raghawendra Kumar, Dinesh Kumar, Zuber Saiyed, Komal Patel, Labdhi Pandya, Afzal Ansari, Nikha Trivedi, Pranay Shah, Kamlesh J Upadhyay, Sanjay Kapadia, Apurvasinh Puvar, Janvi Raval, Zarna Patel, Monika Gandhi, Pinal Trivedi, Maharshi Pandya, Nidhi Patel, R D Dixit, A M Kadri, Harsh Bakshi, Chaitanya Joshi, Madhvi Joshi
EPI_ISL_524721	B.J. Medical College and Civil hospital, Ahmedabad	Gujarat Biotechnology Research Centre	Raghawendra Kumar, Dinesh Kumar, Zuber Saiyed, Komal Patel, Labdhi Pandya, Afzal Ansari, Nikha Trivedi, Pranay Shah, Kamlesh J Upadhyay, Sanjay Kapadia, Apurvasinh Puvar, Janvi Raval, Zarna Patel, Monika Gandhi, Pinal Trivedi, Maharshi Pandya, Nidhi Patel, Nitin Savaliya, R D Dixit, A M Kadri, Harsh Bakshi, Chaitanya Joshi, Madhvi Joshi
EPI_ISL_524722	B.J. Medical College and Civil hospital, Ahmedabad	Gujarat Biotechnology Research Centre	Dinesh Kumar, Zuber Saiyed, Komal Patel, Labdhi Pandya, Afzal Ansari, Nikha Trivedi, Pranay Shah, Kamlesh J Upadhyay, Sanjay Kapadia, Apurvasinh Puvar, Janvi Raval, Zarna Patel, Monika Gandhi, Pinal Trivedi, Maharshi Pandya, Nidhi Patel, Nitin Savaliya, Raghawendra Kumar, R D Dixit, A M Kadri, Harsh Bakshi, Chaitanya Joshi, Madhvi Joshi
EPI_ISL_524723	B.J. Medical College and Civil hospital, Ahmedabad	Gujarat Biotechnology Research Centre	Zuber Saiyed, Komal Patel, Labdhi Pandya, Afzal Ansari, Nikha Trivedi, Pranay Shah, Kamlesh J Upadhyay, Sanjay Kapadia, Apurvasinh Puvar, Janvi Raval, Zarna Patel, Monika Gandhi, Pinal Trivedi, Maharshi Pandya, Nidhi Patel, Nitin Savaliya, Raghawendra Kumar, Dinesh Kumar, R D Dixit, A M Kadri, Harsh Bakshi, Chaitanya Joshi, Madhvi Joshi
EPI_ISL_524724	B.J. Medical College and Civil hospital, Ahmedabad	Gujarat Biotechnology Research Centre	Komal Patel, Labdhi Pandya, Afzal Ansari, Nikha Trivedi, Pranay Shah, Kamlesh J Upadhyay, Sanjay Kapadia, Apurvasinh Puvar, Janvi Raval, Zarna Patel, Monika Gandhi, Pinal Trivedi, Maharshi Pandya, Nidhi Patel, Nitin Savaliya, Raghawendra Kumar, Dinesh Kumar, Zuber Saiyed, R D Dixit, A M Kadri, Harsh Bakshi, Chaitanya Joshi, Madhvi Joshi
EPI_ISL_524725	B.J. Medical College and Civil hospital, Ahmedabad	Gujarat Biotechnology Research Centre	Labdhi Pandya, Afzal Ansari, Nikha Trivedi, Pranay Shah, Kamlesh J Upadhyay, Sanjay Kapadia, Apurvasinh Puvar, Janvi Raval, Zarna Patel, Monika Gandhi, Pinal Trivedi, Maharshi Pandya, Nidhi Patel, Nitin Savaliya, Raghawendra Kumar, Dinesh Kumar, Zuber Saiyed, Komal Patel, R D Dixit, A M Kadri, Harsh Bakshi, Chaitanya Joshi, Madhvi Joshi
EPI_ISL_524726	B.J. Medical College and Civil hospital, Ahmedabad	Gujarat Biotechnology Research Centre	Afzal Ansari, Nikha Trivedi, Pranay Shah, Kamlesh J Upadhyay, Sanjay Kapadia, Apurvasinh Puvar, Janvi Raval, Zarna Patel, Monika Gandhi, Pinal Trivedi, Maharshi Pandya, Nidhi Patel, Nitin Savaliya, Raghawendra Kumar, Dinesh Kumar, Zuber Saiyed, Komal Patel, Labdhi Pandya, R D Dixit, A M Kadri, Harsh Bakshi, Chaitanya Joshi, Madhvi Joshi
EPI_ISL_524727	B.J. Medical College and Civil hospital, Ahmedabad	Gujarat Biotechnology Research Centre	Nikha Trivedi, Pranay Shah, Kamlesh J Upadhyay, Sanjay Kapadia, Apurvasinh Puvar, Janvi Raval, Zarna Patel, Monika Gandhi, Pinal Trivedi, Maharshi Pandya, Nidhi Patel, Nitin Savaliya, Raghawendra Kumar, Dinesh Kumar, Zuber Saiyed, Komal Patel, Labdhi Pandya, Afzal Ansari, R D Dixit, A M Kadri, Harsh Bakshi, Chaitanya Joshi, Madhvi Joshi
EPI_ISL_524728	B.J. Medical College and Civil hospital, Ahmedabad	Gujarat Biotechnology Research Centre	Pranay Shah, Kamlesh J Upadhyay, Sanjay Kapadia, Apurvasinh Puvar, Janvi Raval, Zarna Patel, Monika Gandhi, Pinal Trivedi, Maharshi Pandya, Nidhi Patel, Nitin Savaliya, Raghawendra Kumar, Dinesh Kumar, Zuber Saiyed, Komal Patel, Labdhi Pandya, Afzal Ansari, Nikha Trivedi, R D Dixit, A M Kadri, Harsh Bakshi, Chaitanya Joshi, Madhvi Joshi
EPI_ISL_524729	B.J. Medical College and Civil hospital, Ahmedabad	Gujarat Biotechnology Research Centre	Kamlesh J Upadhyay, Sanjay Kapadia, Apurvasinh Puvar, Janvi Raval, Zarna Patel, Monika Gandhi, Pinal Trivedi, Maharshi Pandya, Nidhi Patel, Nitin Savaliya, Raghawendra Kumar, Dinesh Kumar, Zuber Saiyed, Komal Patel, Labdhi Pandya, Afzal Ansari, Nikha Trivedi, Pranay Shah, R D Dixit, A M Kadri, Harsh Bakshi, Chaitanya Joshi, Madhvi Joshi
EPI_ISL_524730	GMERS Medical College & Hospital, Gotri, Vadodara	Gujarat Biotechnology Research Centre	Meenakshi Shah, Neena Doshi, Varsha Godbole, Apurvasinh Puvar, Janvi Raval, Zarna Patel, Monika Gandhi, Pinal Trivedi, Maharshi Pandya, Nidhi Patel, Nitin Savaliya, Raghawendra Kumar, Dinesh Kumar, Zuber Saiyed, Komal Patel, Labdhi Pandya, Afzal Ansari, Nikha Trivedi, R D Dixit, A M Kadri, Harsh Bakshi, Chaitanya Joshi, Madhvi Joshi
EPI_ISL_524731	GMERS Medical College & Hospital, Gotri, Vadodara	Gujarat Biotechnology Research Centre	Neena Doshi, Varsha Godbole, Apurvasinh Puvar, Janvi Raval, Zarna Patel, Monika Gandhi, Pinal Trivedi, Maharshi Pandya, Nidhi Patel, Nitin Savaliya, Raghawendra Kumar, Dinesh Kumar, Zuber Saiyed, Komal Patel, Labdhi Pandya, Afzal Ansari, Nikha Trivedi, Meenakshi Shah, R D Dixit, A M Kadri, Harsh Bakshi, Chaitanya Joshi, Madhvi Joshi
EPI_ISL_524732	Dr. RSS Hospital, Modasa	Gujarat Biotechnology Research Centre	Harsh Chaudhari, Apurvasinh Puvar, Janvi Raval, Zarna Patel, Monika Gandhi, Pinal Trivedi, Maharshi Pandya, Nidhi Patel, Nitin Savaliya, Raghawendra Kumar, Dinesh Kumar, Zuber Saiyed, Komal Patel, Labdhi Pandya, Afzal Ansari, Nikha Trivedi, R D Dixit, A M Kadri, Harsh Bakshi, Chaitanya Joshi, Madhvi Joshi, Neena Doshi, Varsha Godbole
EPI_ISL_524733	GMERS Medical College and Hospital, Dharpur, Patan	Gujarat Biotechnology Research Centre	A N Parmar, Apurvasinh Puvar, Janvi Raval, Zarna Patel, Monika Gandhi, Pinal Trivedi, Maharshi Pandya, Nidhi Patel, Nitin Savaliya, Raghawendra Kumar, Dinesh Kumar, Zuber Saiyed, Komal Patel, Labdhi Pandya, Afzal Ansari, Nikha Trivedi, R D Dixit, A M Kadri, Harsh Bakshi, Chaitanya Joshi, Madhvi Joshi
EPI_ISL_524734	GMERS Medical College and Hospital, Dharpur, Patan	Gujarat Biotechnology Research Centre	Apurvasinh Puvar, Janvi Raval, Zarna Patel, Monika Gandhi, Pinal Trivedi, Maharshi Pandya, Nidhi Patel, Nitin Savaliya, Raghawendra Kumar, Dinesh Kumar, Zuber Saiyed, Komal Patel, Labdhi Pandya, Afzal Ansari, Nikha Trivedi, A N Parmar, R D Dixit, A M Kadri, Harsh Bakshi, Chaitanya Joshi, Madhvi Joshi
EPI_ISL_524735	Government General Hospital, Jam Khambhaliya, Devbhoomi Dwarka	Gujarat Biotechnology Research Centre	Harish Matani, Janvi Raval, Zarna Patel, Monika Gandhi, Pinal Trivedi, Maharshi Pandya, Nidhi Patel, Nitin Savaliya, Raghawendra Kumar, Dinesh Kumar, Zuber Saiyed, Komal Patel, Labdhi Pandya, Afzal Ansari, Nikha Trivedi, Apurvasinh Puvar, R D Dixit, A M Kadri, Harsh Bakshi, Chaitanya Joshi, Madhvi Joshi
EPI_ISL_524736	Government General Hospital, Jam Khambhaliya, Devbhoomi Dwarka	Gujarat Biotechnology Research Centre	Janvi Raval, Zarna Patel, Monika Gandhi, Pinal Trivedi, Maharshi Pandya, Nidhi Patel, Nitin Savaliya, Raghawendra Kumar, Dinesh Kumar, Zuber Saiyed, Komal Patel, Labdhi Pandya, Afzal Ansari, Nikha Trivedi, Harish Matani, Apurvasinh Puvar, R D Dixit, A M Kadri, Harsh Bakshi, Chaitanya Joshi, Madhvi Joshi
EPI_ISL_524737	GMERS Medical College and Hospital, Gandhinagar	Gujarat Biotechnology Research Centre	Zarna Patel, Monika Gandhi, Pinal Trivedi, Maharshi Pandya, Nidhi Patel, Nitin Savaliya, Raghawendra Kumar, Dinesh Kumar, Zuber Saiyed, Komal Patel, Labdhi Pandya, Afzal Ansari, Nikha Trivedi, Seema Bhatt, Gaurishankar Shrimali, Bhavesh Modi, Bharti Rajani, Apurvasinh Puvar, Janvi Raval, R D Dixit, A M Kadri, Harsh Bakshi, Chaitanya Joshi, Madhvi Joshi
EPI_ISL_524738	GMERS Medical College and Hospital, Gandhinagar	Gujarat Biotechnology Research Centre	Monika Gandhi, Pinal Trivedi, Maharshi Pandya, Nidhi Patel, Nitin Savaliya, Raghawendra Kumar, Dinesh Kumar, Zuber Saiyed, Komal Patel, Labdhi Pandya, Afzal Ansari, Nikha Trivedi, Seema Bhatt, Gaurishankar Shrimali, Bhavesh Modi, Bharti Rajani, Apurvasinh Puvar, Janvi Raval, Zarna Patel, R D Dixit, A M Kadri, Harsh Bakshi, Chaitanya Joshi, Madhvi Joshi
EPI_ISL_524739	GMERS Medical College and Hospital, Gandhinagar	Gujarat Biotechnology Research Centre	Pinal Trivedi, Maharshi Pandya, Nidhi Patel, Nitin Savaliya, Raghawendra Kumar, Dinesh Kumar, Zuber Saiyed, Komal Patel, Labdhi Pandya, Afzal Ansari, Nikha Trivedi, Seema Bhatt, Gaurishankar Shrimali, Bhavesh Modi, Bharti Rajani, Apurvasinh Puvar, Janvi Raval, Zarna Patel, Monika Gandhi, R D Dixit, A M Kadri, Harsh Bakshi, Chaitanya Joshi, Madhvi Joshi
EPI_ISL_524740	GMERS Medical College and Hospital, Gandhinagar	Gujarat Biotechnology Research Centre	Maharshi Pandya, Nidhi Patel, Nitin Savaliya, Raghawendra Kumar, Dinesh Kumar, Zuber Saiyed, Komal Patel, Labdhi Pandya, Afzal Ansari, Nikha Trivedi, Seema Bhatt, Gaurishankar Shrimali, Bhavesh Modi, Bharti Rajani, Apurvasinh Puvar, Janvi Raval, Zarna Patel, Monika Gandhi, Pinal Trivedi, R D Dixit, A M Kadri, Harsh Bakshi, Chaitanya Joshi, Madhvi Joshi
EPI_ISL_524741	GMERS Medical College and Hospital, Gandhinagar	Gujarat Biotechnology Research Centre	Nidhi Patel, Nitin Savaliya, Raghawendra Kumar, Dinesh Kumar, Zuber Saiyed, Komal Patel, Labdhi Pandya, Afzal Ansari, Nikha Trivedi, Seema Bhatt, Gaurishankar Shrimali, Bhavesh Modi, Bharti Rajani, Apurvasinh Puvar, Janvi Raval, Zarna Patel, Monika Gandhi, Pinal Trivedi, Maharshi Pandya, R D Dixit, A M Kadri, Harsh Bakshi, Chaitanya Joshi, Madhvi Joshi

EPI_ISL_524742	GMERS Medical College and Hospital, Gandhinagar	Gujarat Biotechnology Research Centre	Dinesh Kumar, Zuber Saiyed, Komal Patel, Labdhi Pandya, Afzal Ansari, Nikha Trivedi, Seema Bhatt, Gaurishankar Shrimali, Bhavesh Modi, Bharti Rajani, Apurvasinh Puvar, Janvi Raval, Zarna Patel, Monika Gandhi, Pinal Trivedi, Maharshi Pandya, Nidhi Patel, Nitin Savaliya, Raghawendra Kumar, R D Dixit, A M Kadri, Harsh Bakshi, Chaitanya Joshi, Madhvi Joshi
EPI_ISL_524743	GMERS Medical College and Hospital, Gandhinagar	Gujarat Biotechnology Research Centre	Zuber Saiyed, Komal Patel, Labdhi Pandya, Afzal Ansari, Nikha Trivedi, Seema Bhatt, Gaurishankar Shrimali, Bhavesh Modi, Bharti Rajani, Apurvasinh Puvar, Janvi Raval, Zarna Patel, Monika Gandhi, Pinal Trivedi, Maharshi Pandya, Nidhi Patel, Nitin Savaliya, Raghawendra Kumar, Dinesh Kumar, R D Dixit, A M Kadri, Harsh Bakshi, Chaitanya Joshi, Madhvi Joshi
EPI_ISL_524744	GMERS Medical College and Hospital, Gandhinagar	Gujarat Biotechnology Research Centre	Komal Patel, Labdhi Pandya, Afzal Ansari, Nikha Trivedi, Seema Bhatt, Gaurishankar Shrimali, Bhavesh Modi, Bharti Rajani, Apurvasinh Puvar, Janvi Raval, Zarna Patel, Monika Gandhi, Pinal Trivedi, Maharshi Pandya, Nidhi Patel, Nitin Savaliya, Raghawendra Kumar, Dinesh Kumar, Zuber Saiyed, R D Dixit, A M Kadri, Harsh Bakshi, Chaitanya Joshi, Madhvi Joshi
EPI_ISL_524745	GMERS Medical College and Hospital, Gandhinagar	Gujarat Biotechnology Research Centre	Labdhi Pandya, Afzal Ansari, Nikha Trivedi, Seema Bhatt, Gaurishankar Shrimali, Bhavesh Modi, Bharti Rajani, Apurvasinh Puvar, Janvi Raval, Zarna Patel, Monika Gandhi, Pinal Trivedi, Maharshi Pandya, Nidhi Patel, Nitin Savaliya, Raghawendra Kumar, Dinesh Kumar, Zuber Saiyed, Komal Patel, R D Dixit, A M Kadri, Harsh Bakshi, Chaitanya Joshi, Madhvi Joshi
EPI_ISL_524746	Sir M P Shah Government Medical College, Jamnagar	Gujarat Biotechnology Research Centre	Binita Aring, Afzal Ansari, Nikha Trivedi, Apurvasinh Puvar, Janvi Raval, Zarna Patel, Monika Gandhi, Pinal Trivedi, Maharshi Pandya, Nidhi Patel, Nitin Savaliya, Raghawendra Kumar, Dinesh Kumar, Zuber Saiyed, Komal Patel, Labdhi Pandya, R D Dixit, A M Kadri, Harsh Bakshi, Chaitanya Joshi, Madhvi Joshi
EPI_ISL_524747	Sir M P Shah Government Medical College, Jamnagar	Gujarat Biotechnology Research Centre	Afzal Ansari, Nikha Trivedi, Binita Aring, Apurvasinh Puvar, Janvi Raval, Zarna Patel, Monika Gandhi, Pinal Trivedi, Maharshi Pandya, Nidhi Patel, Nitin Savaliya, Raghawendra Kumar, Dinesh Kumar, Zuber Saiyed, Komal Patel, Labdhi Pandya, R D Dixit, A M Kadri, Harsh Bakshi, Chaitanya Joshi, Madhvi Joshi
EPI_ISL_524748	Sir M P Shah Government Medical College, Jamnagar	Gujarat Biotechnology Research Centre	Nikha Trivedi, Apurvasinh Puvar, Janvi Raval, Binita Aring, Zarna Patel, Monika Gandhi, Pinal Trivedi, Maharshi Pandya, Nidhi Patel, Nitin Savaliya, Raghawendra Kumar, Dinesh Kumar, Zuber Saiyed, Komal Patel, Labdhi Pandya, Afzal Ansari, R D Dixit, A M Kadri, Harsh Bakshi, Chaitanya Joshi, Madhvi Joshi
EPI_ISL_524749	Sir M P Shah Government Medical College, Jamnagar	Gujarat Biotechnology Research Centre	Apurvasinh Puvar, Janvi Raval, Zarna Patel, Monika Gandhi, Pinal Trivedi, Maharshi Pandya, Nidhi Patel, Nitin Savaliya, Raghawendra Kumar, Dinesh Kumar, Zuber Saiyed, Komal Patel, Labdhi Pandya, Afzal Ansari, Nikha Trivedi, Binita Aring, R D Dixit, A M Kadri, Harsh Bakshi, Chaitanya Joshi, Madhvi Joshi
EPI_ISL_524750	Sir M P Shah Government Medical College, Jamnagar	Gujarat Biotechnology Research Centre	Janvi Raval, Zarna Patel, Monika Gandhi, Pinal Trivedi, Maharshi Pandya, Nidhi Patel, Nitin Savaliya, Raghawendra Kumar, Dinesh Kumar, Zuber Saiyed, Komal Patel, Labdhi Pandya, Afzal Ansari, Nikha Trivedi, Binita Aring, Apurvasinh Puvar, R D Dixit, A M Kadri, Harsh Bakshi, Chaitanya Joshi, Madhvi Joshi
EPI_ISL_524751	Sir M P Shah Government Medical College, Jamnagar	Gujarat Biotechnology Research Centre	Zarna Patel, Monika Gandhi, Pinal Trivedi, Maharshi Pandya, Nidhi Patel, Nitin Savaliya, Raghawendra Kumar, Dinesh Kumar, Zuber Saiyed, Komal Patel, Labdhi Pandya, Afzal Ansari, Nikha Trivedi, Binita Aring, Apurvasinh Puvar, Janvi Raval, R D Dixit, A M Kadri, Harsh Bakshi, Chaitanya Joshi, Madhvi Joshi
EPI_ISL_524752	GMERS Medical College and Hospital, Dharpur, Patan	Gujarat Biotechnology Research Centre	Monika Gandhi, Pinal Trivedi, Maharshi Pandya, Nidhi Patel, Nitin Savaliya, Raghawendra Kumar, Dinesh Kumar, Zuber Saiyed, Komal Patel, Labdhi Pandya, Afzal Ansari, Nikha Trivedi, A N Parmar, Apurvasinh Puvar, Janvi Raval, Zarna Patel, R D Dixit, A M Kadri, Harsh Bakshi, Chaitanya Joshi, Madhvi Joshi
EPI_ISL_524753	GMERS Medical College and Hospital, Dharpur, Patan	Gujarat Biotechnology Research Centre	Pinal Trivedi, Maharshi Pandya, Nidhi Patel, Nitin Savaliya, Raghawendra Kumar, Dinesh Kumar, Zuber Saiyed, Komal Patel, Labdhi Pandya, Afzal Ansari, Nikha Trivedi, A N Parmar, Apurvasinh Puvar, Janvi Raval, Zarna Patel, Monika Gandhi, R D Dixit, A M Kadri, Harsh Bakshi, Chaitanya Joshi, Madhvi Joshi
EPI_ISL_524754	GMERS Medical College and Hospital, Dharpur, Patan	Gujarat Biotechnology Research Centre	Maharshi Pandya, Nidhi Patel, Nitin Savaliya, Raghawendra Kumar, Dinesh Kumar, Zuber Saiyed, Komal Patel, Labdhi Pandya, Afzal Ansari, Nikha Trivedi, A N Parmar, Apurvasinh Puvar, Janvi Raval, Zarna Patel, Monika Gandhi, Pinal Trivedi, R D Dixit, A M Kadri, Harsh Bakshi, Chaitanya Joshi, Madhvi Joshi
EPI_ISL_524755	GMERS Medical College and Hospital, Dharpur, Patan	Gujarat Biotechnology Research Centre	Nidhi Patel, Nitin Savaliya, Raghawendra Kumar, Dinesh Kumar, Zuber Saiyed, Komal Patel, Labdhi Pandya, Afzal Ansari, Nikha Trivedi, A N Parmar, Apurvasinh Puvar, Janvi Raval, Zarna Patel, Monika Gandhi, Pinal Trivedi, Maharshi Pandya, R D Dixit, A M Kadri, Harsh Bakshi, Chaitanya Joshi, Madhvi Joshi
EPI_ISL_524756	GMERS Medical College and Hospital, Dharpur, Patan	Gujarat Biotechnology Research Centre	Nitin Savaliya, Raghawendra Kumar, Dinesh Kumar, Zuber Saiyed, Komal Patel, Labdhi Pandya, Afzal Ansari, Nikha Trivedi, A N Parmar, Apurvasinh Puvar, Janvi Raval, Zarna Patel, Monika Gandhi, Pinal Trivedi, Maharshi Pandya, Nidhi Patel, R D Dixit, A M Kadri, Harsh Bakshi, Chaitanya Joshi, Madhvi Joshi
EPI_ISL_524757	GMERS Medical College and Hospital, Dharpur, Patan	Gujarat Biotechnology Research Centre	Raghawendra Kumar, Dinesh Kumar, Zuber Saiyed, Komal Patel, Labdhi Pandya, Afzal Ansari, Nikha Trivedi, A N Parmar, Apurvasinh Puvar, Janvi Raval, Zarna Patel, Monika Gandhi, Pinal Trivedi, Maharshi Pandya, Nidhi Patel, Nitin Savaliya, R D Dixit, A M Kadri, Harsh Bakshi, Chaitanya Joshi, Madhvi Joshi
EPI_ISL_524758	GMERS Medical College and Hospital, Dharpur, Patan	Gujarat Biotechnology Research Centre	Dinesh Kumar, Zuber Saiyed, Komal Patel, Labdhi Pandya, Afzal Ansari, Nikha Trivedi, A N Parmar, Apurvasinh Puvar, Janvi Raval, Zarna Patel, Monika Gandhi, Pinal Trivedi, Maharshi Pandya, Nidhi Patel, Nitin Savaliya, Raghawendra Kumar, R D Dixit, A M Kadri, Harsh Bakshi, Chaitanya Joshi, Madhvi Joshi
EPI_ISL_524759	GMERS Medical College and Hospital, Dharpur, Patan	Gujarat Biotechnology Research Centre	Zuber Saiyed, Komal Patel, Labdhi Pandya, Afzal Ansari, Nikha Trivedi, A N Parmar, Apurvasinh Puvar, Janvi Raval, Zarna Patel, Monika Gandhi, Pinal Trivedi, Maharshi Pandya, Nidhi Patel, Nitin Savaliya, Raghawendra Kumar, Dinesh Kumar, R D Dixit, A M Kadri, Harsh Bakshi, Chaitanya Joshi, Madhvi Joshi
EPI_ISL_524760	GMERS Medical College and Hospital, Dharpur, Patan	Gujarat Biotechnology Research Centre	Komal Patel, Labdhi Pandya, Afzal Ansari, Nikha Trivedi, A N Parmar, Apurvasinh Puvar, Janvi Raval, Zarna Patel, Monika Gandhi, Pinal Trivedi, Maharshi Pandya, Nidhi Patel, Nitin Savaliya, Raghawendra Kumar, Dinesh Kumar, Zuber Saiyed, R D Dixit, A M Kadri, Harsh Bakshi, Chaitanya Joshi, Madhvi Joshi
EPI_ISL_524761	GMERS Medical College and Hospital, Dharpur, Patan	Gujarat Biotechnology Research Centre	Labdhi Pandya, Afzal Ansari, Nikha Trivedi, A N Parmar, Apurvasinh Puvar, Janvi Raval, Zarna Patel, Monika Gandhi, Pinal Trivedi, Maharshi Pandya, Nidhi Patel, Nitin Savaliya, Raghawendra Kumar, Dinesh Kumar, Zuber Saiyed, Komal Patel, R D Dixit, A M Kadri, Harsh Bakshi, Chaitanya Joshi, Madhvi Joshi
EPI_ISL_524762	GMERS Medical College and Hospital, Dharpur, Patan	Gujarat Biotechnology Research Centre	Afzal Ansari, Nikha Trivedi, A N Parmar, Apurvasinh Puvar, Janvi Raval, Zarna Patel, Monika Gandhi, Pinal Trivedi, Maharshi Pandya, Nidhi Patel, Nitin Savaliya, Raghawendra Kumar, Dinesh Kumar, Zuber Saiyed, Komal Patel, Labdhi Pandya, R D Dixit, A M Kadri, Harsh Bakshi, Chaitanya Joshi, Madhvi Joshi
EPI_ISL_524763	GMERS Medical College and Hospital, Dharpur, Patan	Gujarat Biotechnology Research Centre	Nikha Trivedi, A N Parmar, Apurvasinh Puvar, Janvi Raval, Zarna Patel, Monika Gandhi, Pinal Trivedi, Maharshi Pandya, Nidhi Patel, Nitin Savaliya, Raghawendra Kumar, Dinesh Kumar, Zuber Saiyed, Komal Patel, Labdhi Pandya, Afzal Ansari, R D Dixit, A M Kadri, Harsh Bakshi, Chaitanya Joshi, Madhvi Joshi
EPI_ISL_524764	GMERS Medical College and Hospital, Dharpur, Patan	Gujarat Biotechnology Research Centre	A N Parmar, Apurvasinh Puvar, Janvi Raval, Zarna Patel, Monika Gandhi, Pinal Trivedi, Maharshi Pandya, Nidhi Patel, Nitin Savaliya, Raghawendra Kumar, Dinesh Kumar, Zuber Saiyed, Komal Patel, Labdhi Pandya, Afzal Ansari, Nikha Trivedi, R D Dixit, A M Kadri, Harsh Bakshi, Chaitanya Joshi, Madhvi Joshi
EPI_ISL_524765	GMERS Medical College and Hospital, Dharpur, Patan	Gujarat Biotechnology Research Centre	Apurvasinh Puvar, Janvi Raval, Zarna Patel, Monika Gandhi, Pinal Trivedi, Maharshi Pandya, Nidhi Patel, Nitin Savaliya, Raghawendra Kumar, Dinesh Kumar, Zuber Saiyed, Komal Patel, Labdhi Pandya, Afzal Ansari, Nikha Trivedi, A N Parmar, R D Dixit, A M Kadri, Harsh Bakshi, Chaitanya Joshi, Madhvi Joshi
EPI_ISL_524766	GMERS Medical College and Hospital, Dharpur, Patan	Gujarat Biotechnology Research Centre	Janvi Raval, Zarna Patel, Monika Gandhi, Pinal Trivedi, Maharshi Pandya, Nidhi Patel, Nitin Savaliya, Raghawendra Kumar, Dinesh Kumar, Zuber Saiyed, Komal Patel, Labdhi Pandya, Afzal Ansari, Nikha Trivedi, Apurvasinh Puvar, A N Parmar, R D Dixit, A M Kadri, Harsh Bakshi, Chaitanya Joshi, Madhvi Joshi
EPI_ISL_524800	Evandro Chagas Institute	Evandro Chagas Institute	Santos, M.C.; Silva, A.M.; Junior, W.D.C.; Barbagelata, L.S.; Ferreira, J.A.; Sousa, E.M.A.; da Silva, P.S.; Resque, H.R; Martins, L.C.; Sousa Junior, E.C.;Viana, G.M.R
EPI_ISL_525419	GMERS Medical College and Hospital, Gandhinagar	Gujarat Biotechnology Research Centre	Nitin Savaliya, Raghawendra Kumar, Dinesh Kumar, Zuber Saiyed, Komal Patel, Labdhi Pandya, Afzal Ansari, Nikha Trivedi, Seema Bhatt, Gaurishankar Shrimali, Bhavesh Modi, Bharti Rajani, Apurvasinh Puvar, Janvi Raval, Zarna Patel, Monika Gandhi, Pinal Trivedi, Maharshi Pandya, Nidhi Patel, R D Dixit,

			A M Kadri, Harsh Bakshi, Chaitanya Joshi, Madhvi Joshi
EPI_ISL_525420	GMERS Medical College and Hospital, Gandhinagar	Gujarat Biotechnology Research Centre	Raghawendra Kumar, Dinesh Kumar, Zuber Saiyed, Komal Patel, Labdhi Pandya, Afzal Ansari, Nikha Trivedi, Seema Bhatt, Gaurishankar Shrimali, Bhavesh Modi, Bharti Rajani, Apurvasinh Puvar, Janvi Raval, Zarna Patel, Monika Gandhi, Pinal Trivedi, Maharshi Pandya, Nidhi Patel, Nitin Savaliya, R D Dixit, A M Kadri, Harsh Bakshi, Chaitanya Joshi, Madhvi Joshi
EPI_ISL_525421	B.J. Medical College and Civil hospital, Ahmedabad	Gujarat Biotechnology Research Centre	Zarna Patel, Monika Gandhi, Pinal Trivedi, Maharshi Pandya, Nidhi Patel, Nitin Savaliya, Raghawendra Kumar, Dinesh Kumar, Zuber Saiyed, Komal Patel, Labdhi Pandya, Afzal Ansari, Nikha Trivedi, Pranay Shah, Kamlesh J Upadhyay, Sanjay Kapadia, Apurvasinh Puvar, Janvi Raval, R D Dixit, A M Kadri, Harsh Bakshi, Chaitanya Joshi, Madhvi Joshi
EPI_ISL_525422	B.J. Medical College and Civil hospital, Ahmedabad	Gujarat Biotechnology Research Centre	Monika Gandhi, Pinal Trivedi, Maharshi Pandya, Nidhi Patel, Nitin Savaliya, Raghawendra Kumar, Dinesh Kumar, Zuber Saiyed, Komal Patel, Labdhi Pandya, Afzal Ansari, Nikha Trivedi, Pranay Shah, Kamlesh J Upadhyay, Sanjay Kapadia, Apurvasinh Puvar, Janvi Raval, Zarna Patel, R D Dixit, A M Kadri, Harsh Bakshi, Chaitanya Joshi, Madhvi Joshi
EPI_ISL_525430	Institute of Microbiology, Universidad San Francisco de Quito	Institute of Microbiology, Universidad San Francisco de Quito	Juan José Guadalupe, Monica Becerra-Wong, Belén Prado-Vivar, Sully Márquez, Bernardo Gutiérrez, Verónica Barragán, Patricio Rojas-Silva, Gabriel Trueba, Michelle Grunauer, Paúl Cárdenas
EPI_ISL_525467	Universidad Iberoamericana	International Centre for Genetic Engineering and Biotechnology (ICGEB) and ARGO Open Lab Platform	Robert Paulino-Ramirez, Eileen Riego, Alejandro Vallejo Degaudenzi, Victor Virgilio Calderon, Leandro Tapia, Patricia Leon, Danilo Licastro, Simeone Dal Monego, Sreejith Rajasekharan and Alessandro Marcello.
EPI_ISL_525468	Universidad Iberoamericana	International Centre for Genetic Engineering and Biotechnology (ICGEB) and ARGO Open Lab Platform	Robert Paulino-Ramirez, Eileen Riego, Alejandro Vallejo Degaudenzi, Victor Virgilio Calderon, Leandro Tapia,Patricia Leon,Danilo Licastro, Simeone Dal Monego, Sreejith Rajasekharan and Alessandro Marcello.
EPI_ISL_525469, EPI_ISL_525470, EPI_ISL_525471	Universidad Iberoamericana	International Centre for Genetic Engineering and Biotechnology (ICGEB) and ARGO Open Lab Platform	Robert Paulino-Ramirez, Eileen Riego, Alejandro Vallejo Degaudenzi, Victor Virgilio Calderon, Leandro Tapia, Patricia Leon, Danilo Licastro, Simeone Dal Monego, Sreejith Rajasekharan and Alessandro Marcello.
EPI_ISL_525474	Centre for Dengue Research	Centre for Dengue Research, USJ, SL	Chandima Jeewandara, Deshni Jayathilaka, Dinuka Ariyaratne, Laksiri Gomes, Diyanath Ranasinghe, Dinuka Guruge, Ruwan Wijayamuni, Gathsaurie Neelika Malavige
EPI_ISL_525476	Centre for Dengue Research	Centre for Dengue Research	Chandima Jeewandara, Deshni Jayathilaka, Dinuka Ariyaratne, Laksiri Gomes, Diyanath Ranasinghe, Dinuka Guruge, Ruwan Wijayamuni, Gathsaurie Neelika Malavige
EPI_ISL_525478, EPI_ISL_525479, EPI_ISL_525481	Centre for Dengue Research	Centre for Dengue Research	Chandima Jeewandara, Deshni Jayathilaka, Dinuka Ariyaratne, Laksiri Gomes, Diyanath Ranasinghe, Ananda Wijewickrama, Eranga Narangoda, Damayanthi Idampitiya, Gathsaurie Neelika Malavige
EPI_ISL_525486, EPI_ISL_525488, EPI_ISL_525489	Centre for Dengue Research	Centre for Dengue Research	Chandima Jeewandara, Deshni Jayathilaka, Dinuka Ariyaratne, Laksiri Gomes, Diyanath Ranasinghe, Ananda Wijewickrama, Malika Karunaratne, Eranga Narangoda, Damayanthi Idampitiya, Gathsaurie Neelika Malavige
EPI_ISL_525492	RSUP dr. SOERADJI TIRTONEGORO	Genetics Working Group (Pokja Genetik) Faculty of Medicine, Public Health and Nursing Universitas Gadjah Mada (FK-KMK UGM), Disease Investigation Center Wates Ministry of Agriculture Indonesia, Department of Microbiology FK-KMK UGM, Laboratorium Diagnostik Yayasan Tahija World Mosquito Program (WMP) Yogyakarta Center for Tropical Medicine FK-KMK UGM, Integrated Research Center FK-KMK UGM, Department of Computer Science and Electronics FMIPA UGM	Gunadi, Hendra Wibawa, . Marcellus, Mohamad S. Hakim, Edwin W. Daniwijaya, Ludhang P. Rizki, Endah Supriyati, Eggi Arguni, Titik Nuryastuti, Tri Wibawa, Dwi AA Nugrahaningsih, Afiahayati , . Siswanto, Kurniyanto, Indah Juliana, Alvin S. Kalim, Dwiki Afandy
EPI_ISL_525495, EPI_ISL_525496	Laboratory of Molecular Virology of the International Centre for Genetic Engineering and Biotechnology (ICGEB)	ARGO Open Lab Platform for Genome Sequencing	Licastro D, Rajasekharan S, Dal Monego S, Segat L, D'Agaro P, Marcello A
EPI_ISL_525553, EPI_ISL_525556, EPI_ISL_525557, EPI_ISL_525570, EPI_ISL_525571, EPI_ISL_525572	Istituto Zooprofilattico Sperimentale Puglia e Basilicata; Dipartimento di Bioscienze, Biotecnologie e Biofarmaceutica dell'Università degli Studi di Bari "A.Moro"; Istituto di Biomembrane. Bioenergetica e Biotecnologie Molecolari del Consiglio Nazionale delle Ricerche di Bari	Beaconlab (Bioinformatics, Evolution and Comparative Genomics lab), Dept of Biosciences, University on Milan	Parisi A.,Pesole G., Manzari C., Chiara M
EPI_ISL_525578, EPI_ISL_525621, EPI_ISL_525627, EPI_ISL_525631, EPI_ISL_525638, EPI_ISL_525639, EPI_ISL_525640, EPI_ISL_525641, EPI_ISL_525642, EPI_ISL_525646, EPI_ISL_525647, EPI_ISL_525648, EPI_ISL_525649, EPI_ISL_525650, EPI_ISL_525654, EPI_ISL_525658, EPI_ISL_525659, EPI_ISL_525676, EPI_ISL_525677, EPI_ISL_525678, EPI_ISL_525679, EPI_ISL_525680, EPI_ISL_525681, EPI_ISL_525682, EPI_ISL_525683, EPI_ISL_525684, EPI_ISL_525685, EPI_ISL_525686			
see above	Wadsworth Center, New York State Department of Health	Wadsworth Center, New York State Department of Health	Kirsten St. George, Daryl M. Lamson, Sara Griesemer, Jonathan Plitnick, Navjot Singh, Matthew D. Shudt, Erica Lasek-Nesselquist
EPI_ISL_526215, EPI_ISL_526216, EPI_ISL_526217, EPI_ISL_526218, EPI_ISL_526219, EPI_ISL_526220, EPI_ISL_526221, EPI_ISL_526222, EPI_ISL_526223, EPI_ISL_526224, EPI_ISL_526225, EPI_ISL_526226, EPI_ISL_526227, EPI_ISL_526228, EPI_ISL_526229, EPI_ISL_526230, EPI_ISL_526231, EPI_ISL_526232, EPI_ISL_526233, EPI_ISL_526234, EPI_ISL_526235, EPI_ISL_526236, EPI_ISL_526237, EPI_ISL_526238			
see above	Hungarian Defence Forces Military Medical Centre	National Laboratory of Virology, Szentágothai Research Centre	Endre Gábor Tóth, Balázs Somogyi, Bálint Eszenyi, Ferenc Jakab, Gábor Kemenesi
EPI_ISL_526686, EPI_ISL_526687, EPI_ISL_526688	Faith Laboratory, Immunology Institute, Icahn School of Medicine at Mount Sinai	van Bakel Laboratory, Genetics and Genomics Sciences, Icahn School of Medicine at Mount Sinai	Graham J. Britton, Alice Chen-Liaw, Francesca Cossarini, Alexandra Livanos, Matthew P. Spindler, Tamar Plitt, Joseph Eggers, Ilaria Mogno, Ana S. Gonzalez-Reiche, Sophia Sui, Michael Tankelevich, Lauren Tal Grinspan, Rebekah E. Dixon, Divya Jha, Gustavo Martinez-Delgado, Fatima Amanat, Daisy Hoagland, Benjamin R. tenOever, Marla C. Dubinsky, Miriam Merad, Harm Van Bakel, Florian Krammer, Gerold Bongers, Saurabh Mehandru and Jeremiah J. Faith
EPI_ISL_526932, EPI_ISL_526933, EPI_ISL_526934	Instituto Nacional de Salud, Bogotá, Colombia	Instituto Nacional de Salud, Bogotá, Colombia	Katherine Laiton-Donato, Diego A. Álvarez-Díaz, Carlos Franco-Muñoz, Mauricio Pacheco-Montealegre, Jonathan Reales, Diego Andrés Prada, Jose A. Usme-Ciro, Zulma M. Cucunubá, Christian Julian VillabonaArenas, Liz Villabona-Arenas, Sussy Echeverria, Astrid C. Flórez, Carolina Ferro, Diana Marcela Walteros-Acero, Franklin Prieto, Carlos Andrés Durán, Martha Lucia Ospina Martinez, Marcela Mercado-Reyes
EPI_ISL_526935, EPI_ISL_526936, EPI_ISL_526937, EPI_ISL_526938, EPI_ISL_526939, EPI_ISL_526940, EPI_ISL_526941, EPI_ISL_526942, EPI_ISL_526943, EPI_ISL_526944, EPI_ISL_526945, EPI_ISL_526946, EPI_ISL_526947, EPI_ISL_526948			
see above	Faroese National Reference Laboratory for Fish and Animal Diseases	Faroese National Reference Laboratory for Fish and Animal Diseases	Maria Marjunardóttir Dahl, Petra Elisabeth Petersen, Debes Hammershaimb Christiansen
EPI_ISL_526949, EPI_ISL_526950, EPI_ISL_526951, EPI_ISL_526953, EPI_ISL_526954, EPI_ISL_526955, EPI_ISL_526956, EPI_ISL_526957, EPI_ISL_526958, EPI_ISL_526959, EPI_ISL_526960, EPI_ISL_526961			
see above	Instituto Nacional de Salud, Bogotá, Colombia	Instituto Nacional de Salud, Bogotá, Colombia	Katherine Laiton-Donato, Diego A. Álvarez-Díaz, Carlos Franco-Muñoz, Mauricio Pacheco-Montealegre, Jonathan Reales, Diego Andrés Prada, Jose A. Usme-Ciro, Zulma M. Cucunubá, Christian Julian VillabonaArenas, Liz Villabona-Arenas, Sussy Echeverria, Astrid C. Flórez, Carolina Ferro, Diana Marcela Walteros-Acero, Franklin Prieto, Carlos Andrés Durán, Martha Lucia Ospina Martinez, Marcela Mercado-Reyes
EPI_ISL_526962, EPI_ISL_526963, EPI_ISL_526964, EPI_ISL_526965	Instituto Nacional de Salud, Bogotá, Colombia	Instituto Nacional de Salud, Bogotá, Colombia	Katherine Laiton-Donato, Diego A. Álvarez-Díaz, Carlos Franco-Muñoz, Jonathan Reales, Diego Andrés Prada, Jeadran Malagón-Rojas, Felix Betzler, Wendy K. Jo, Edmilson F. de Oliveira-Filho, Carolina Ferro, Diana Marcela Walteros-Acero, Franklin Prieto, Carlos Andrés Durán, Martha Lucia Ospina Martinez, Marcela Mercado-Reyes
EPI_ISL_526967, EPI_ISL_526968, EPI_ISL_526969, EPI_ISL_526970, EPI_ISL_526971, EPI_ISL_526973, EPI_ISL_526974	Instituto Nacional de Salud, Bogotá, Colombia	Instituto Nacional de Salud, Bogotá, Colombia	Katherine Laiton-Donato, Diego A. Álvarez-Díaz, Carlos Franco-Muñoz, Mauricio Pacheco-Montealegre, Jonathan Reales, Diego Andrés Prada, Jose A. Usme-Ciro, Zulma M. Cucunubá, Christian Julian VillabonaArenas, Liz Villabona-Arenas, Sussy Echeverria, Astrid C. Flórez, Carolina Ferro, Diana Marcela Walteros-Acero, Franklin Prieto, Carlos Andrés Durán, Martha Lucia Ospina Martinez, Marcela Mercado-Reyes
EPI_ISL_527742	Centro Nacional De Rehabilitacion Humberto Araya Rojas (Cenare)	Inciensa, Instituto Costarricense de Investigación y Enseñanza en Nutrición y Salud	Francisco Duarte, Hebleen Porras, Claudio Soto-Garita, Estela Cordero, Adriana Godinez & Melany Calderon
EPI_ISL_527753	Hospital San Vicente De Paul	Inciensa, Instituto Costarricense de Investigación y	Francisco Duarte, Hebleen Porras, Claudio Soto-Garita, Estela Cordero, Adriana Godinez & Melany Calderon

		Enseñanza en Nutrición y Salud	
EPI_ISL_527818, EPI_ISL_527819	Centro de Investigaciones, Universidad de Especialidades Espíritu Santo	Institute of Microbiology, Universidad San Francisco de Quito	Derly Andrade, Juan Carlos Fernandez, Belén Prado-Vivar, Sully Márquez, Juan José Guadalupe, Monica Becerra-Wong, Bernardo Gutiérrez, Gabriel Morey, Ruben Armas, Jose Pedro Barberan, Fernando Espinoza, Edith Lopez, Verónica Barragán, Patricio Rojas-Silva, Gabriel Trueba, Michelle Grunauer, Paúl Cárdenas
EPI_ISL_527856	Hospital Municipal Prof. Waldomiro de Paula	Instituto Adolfo Lutz, Interdiciplinary Procedures Center, Strategic Laboratory	Claudio Tavares Sacchi, Claudia Regina Gonçalves, Erica Valessa Ramos Gomes
EPI_ISL_527857	Hospital Regional Vale do Ribeira	Instituto Adolfo Lutz, Interdiciplinary Procedures Center, Strategic Laboratory	Claudio Tavares Sacchi, Claudia Regina Gonçalves, Erica Valessa Ramos Gomes
EPI_ISL_527858	Pronto Atendimento Sancta Maggiore Jardim Paulista	Instituto Adolfo Lutz, Interdiciplinary Procedures Center, Strategic Laboratory	Claudio Tavares Sacchi, Claudia Regina Gonçalves, Erica Valessa Ramos Gomes
EPI_ISL_527859	Hospital Municipal Vereador Jose Storopolli	Instituto Adolfo Lutz, Interdiciplinary Procedures Center, Strategic Laboratory	Claudio Tavares Sacchi, Claudia Regina Gonçalves, Erica Valessa Ramos Gomes
EPI_ISL_527860	Hospital Municipal de Parelheiros Josanias Castanha Braga	Instituto Adolfo Lutz, Interdiciplinary Procedures Center, Strategic Laboratory	Claudio Tavares Sacchi, Claudia Regina Gonçalves, Erica Valessa Ramos Gomes
EPI_ISL_527861	Hospital e Maternidade Celso Pierro	Instituto Adolfo Lutz, Interdiciplinary Procedures Center, Strategic Laboratory	Av. Dr. Arnaldo, 355 - Brazil, Cerqueira Cesar, São Paulo - SP, 01246-1301
EPI_ISL_527862	Hospital Municipal de Urgência	Instituto Adolfo Lutz, Interdiciplinary Procedures Center, Strategic Laboratory	Claudio Tavares Sacchi, Claudia Regina Gonçalves, Erica Valessa Ramos Gomes
EPI_ISL_527863	Hospital Municipal do Tatuape Carmino Caricchio	Instituto Adolfo Lutz, Interdiciplinary Procedures Center, Strategic Laboratory	Claudio Tavares Sacchi, Claudia Regina Gonçalves, Erica Valessa Ramos Gomes
EPI_ISL_527864	Hospital e Pronto Socorro Comunitário Vila Iolanda	Instituto Adolfo Lutz, Interdiciplinary Procedures Center, Strategic Laboratory	Claudio Tavares Sacchi, Claudia Regina Gonçalves, Erica Valessa Ramos Gomes
EPI_ISL_527865	Hospital e Maternidade São Cristóvão	Instituto Adolfo Lutz, Interdiciplinary Procedures Center, Strategic Laboratory	Claudio Tavares Sacchi, Claudia Regina Gonçalves, Erica Valessa Ramos Gomes
EPI_ISL_527866	PS Municipal Dr Lauro Ribas Braga	Instituto Adolfo Lutz, Interdiciplinary Procedures Center, Strategic Laboratory	Av. Dr. Arnaldo, 355 - Brazil, Cerqueira Cesar, São Paulo - SP, 01246-1301
EPI_ISL_527867	Pronto Socorro Municipal - Balneario São José	Instituto Adolfo Lutz, Interdiciplinary Procedures Center, Strategic Laboratory	Claudio Tavares Sacchi, Claudia Regina Gonçalves, Erica Valessa Ramos Gomes
EPI_ISL_527868	Hospital e Maternidade do Braz	Instituto Adolfo Lutz, Interdiciplinary Procedures Center, Strategic Laboratory	Claudio Tavares Sacchi, Claudia Regina Gonçalves, Erica Valessa Ramos Gomes
EPI_ISL_527869	Hospital Municipal Carmen Prudente	Instituto Adolfo Lutz, Interdiciplinary Procedures Center, Strategic Laboratory	Claudio Tavares Sacchi, Claudia Regina Gonçalves, Erica Valessa Ramos Gomes
EPI_ISL_527870	Hospital Municipal Mário Gatti	Instituto Adolfo Lutz, Interdiciplinary Procedures Center, Strategic Laboratory	Claudio Tavares Sacchi, Claudia Regina Gonçalves, Erica Valessa Ramos Gomes
EPI_ISL_528382, EPI_ISL_528383, EPI_ISL_528384, EPI_ISL_528385	Translational Health Science and Technology Institute -ESIC medical college and hospital, Faridabad	THSTI Bioassay laboratory	Saurabh Kumar, Jigme Wangchuk, Anil Kumar Pandey, Asim Das, Guruprasad R. Medigeshi
EPI_ISL_528538	Alsafar	Alsafar	Andreas Henschel, Gihan Elsir Ahmed Daw Elbait, Samuel Feng, Rifat, Ernesto Damiani, Guan Tay, Habiba Alsafar
EPI_ISL_528539	LVM/UFRJ	LNCC	Gustavo M. Romário M. de Souza; Bruno B. Bezerra; Lucio A. Caldas; Fabio Limonte; Elena Cobos; Sharton V. A. Coelho; Luiz Almeida; Luiza Higga; Isadora A. Correa; Diana Marianni; Luciana B. Arruda; Marcelo Bozza; Orlando Ferreira; Wanderley de Souza; Ana Teresa R. Vasconcelos; Terezinha M. Castineiras; Amilcar Tanuri; Luciana J. Costa.
EPI_ISL_528637, EPI_ISL_528638	LVM/UFRJ	Bioinformatics Laboratory / LNCC	Gustavo D. P. Silva; M. Romário M. de Souza; Bruno B. Bezerra; Lucio A. Caldas; Fabio Limonte; Elena Cobos; Sharton V. A. Coelho; Luiz Almeida; Luiza Higga; Isadora A. Correa; Diana Marianni; Luciana B. Arruda; Marcelo Bozza; Orlando Ferreira; Wanderley de Souza; Ana Teresa R. Vasconcelos; Terezinha M. Castineiras; Amilcar Tanuri; Luciana J. Costa
EPI_ISL_528687, EPI_ISL_528689, EPI_ISL_528691, EPI_ISL_528692, EPI_ISL_528693, EPI_ISL_528694, EPI_ISL_528695, EPI_ISL_528696, EPI_ISL_528698, EPI_ISL_528699, EPI_ISL_528700, EPI_ISL_528701, EPI_ISL_528702, EPI_ISL_528703, EPI_ISL_528704, EPI_ISL_528705, EPI_ISL_528706, EPI_ISL_528707, EPI_ISL_528708, EPI_ISL_528709, EPI_ISL_528710, EPI_ISL_528711, EPI_ISL_528712, EPI_ISL_528713, EPI_ISL_528714, EPI_ISL_528715, EPI_ISL_528716, EPI_ISL_528717, EPI_ISL_528718, EPI_ISL_528719, EPI_ISL_528720, EPI_ISL_528721			
see above	Alsafar - Khalifa University Abu Dhabi	Alsafar - Khalifa University Abu Dhabi	Andreas Henschel, Gihan Daw Elbait, Samuel Feng, Rifat Hamoudi, Ernesto Damiani, Guan Tay, Habiba Alsafar
EPI_ISL_528738, EPI_ISL_528739, EPI_ISL_528740, EPI_ISL_528741	Malaysia Genome Institute	Malaysia Genome Institute	Mohd Noor Mat Isa, Irni Suhayu Sapian, Yusuf Muhammad Noor, Nurhezreen Md Iqbal, Mohd Faizal Abu Bakar, Enizza Kasim, Shamsidar Sopie, Siti Noraini Othman, Azrin Ahmad, Nor Azfa Johari, Shahrul Hisham Zainal Ariffin
EPI_ISL_528742	Malaysia Genome Institute	Malaysia Genome Institute	Mohd Noor Mat Isa, Irni Suhayu Sapian, Gan Han Ming, Yusuf Muhammad Noor, Tan Ju Lin, Nurhezreen Md Iqbal, Mohd Faizal Abu Bakar, Enizza Kasim, Shamsidar Sopie, Siti Noraini Othman, Azrin Ahmad, Nor Azfa Johari, Shahrul Hisham Zainal Ariffin
EPI_ISL_528743, EPI_ISL_528744	Malaysia Genome Institute	Malaysia Genome Institute	Mohd Noor Mat Isa, Irni Suhayu Sapian, Yusuf Muhammad Noor, Nurhezreen Md Iqbal, Mohd Faizal Abu Bakar, Enizza Kasim, Shamsidar Sopie, Siti Noraini Othman, Azrin Ahmad, Nor Azfa Johari, Shahrul Hisham Zainal Ariffin
EPI_ISL_528745	Laboratorium Kesehatan Provinsi Jawa Barat	School of Life Sciences and Technology & School of Pharmacy-Institut Teknologi Bandung; Molecular Genetics Laboratory-Faculty of Medicine-Universitas Padjadjaran; Laboratorium Kesehatan Provinsi Jawa Barat	Marselina Irasonia Tan, Yunia Sribudiani, Catur Riani, Azzania Fibriani, Husna Nugrahapraja, Tarwadi, Ema Rahmawati, Savira Ekawardhani, Hesti Lina Wiraswati, Ryan Bayusantika Ristandi, Rifky Waluyajati Rachman, Cut Nur Cinthia Alamanda, Lia Faridah, Tri Hanggono Achmad, Mas Rizky A.A. Syamsunarno, Fensi Amalina, Hammam Riza, Sony Solistia Wirawan, Agung Eru Wibowo, Irvan Faizal
EPI_ISL_528746	Immanuel Hospital	Molecular Genetics Laboratory-Faculty of Medicine-Universitas Padjadjaran; School of Life Sciences and Technology & School of Pharmacy-Institut Teknologi Bandung; Laboratorium Kesehatan Provinsi Jawa Barat	Yunia Sribudiani,Tri Hanggono Achmad, Mas Rizky A.A. Syamsunarno, Fensi Amalina, Catur Riani, Azzania Fibriani, Husna Nugrahapraja, Marselina Irasonia Tan, Tarwadi, Ema Rahmawati, Savira Ekawardhani, Hesti Lina Wiraswati, Ryan Bayusantika Ristandi, Rifky Waluyajati Rachman, Cut Nur Cinthia Alamanda, Lia Faridah, Hammam Riza, Sony Solistia Wirawan, Agung Eru Wibowo, Irvan Faizal
EPI_ISL_528747	Santo Borromeus Hospital	School of Pharmacy & School of Life Sciences and Technology - Institut Teknologi Bandung; Molecular Genetics Laboratory-Faculty of Medicine-Universitas Padjadjaran; Laboratorium Kesehatan Provinsi Jawa Barat	Catur Riani, Marselina Irasonia Tan, Yunia Sribudiani, Azzania Fibriani, Husna Nugrahapraja, Tarwadi, Ema Rahmawati, Savira Ekawardhani, Hesti Lina Wiraswati, Ryan Bayusantika Ristandi, Rifky Waluyajati Rachman, Cut Nur Cinthia Alamanda, Lia Faridah, Miftahul Faridl, Karimatu Khoirunnisa, Hammam Riza, Sony Solistia Wirawan, Agung Eru Wibowo, Irvan Faizal
EPI_ISL_528748	Dinkes Provinsi Jawa Barat	School of Life Sciences and Technology & School of Pharmacy-Institut Teknologi Bandung; Molecular Genetics Laboratory-Faculty of Medicine-Universitas Padjadjaran; Laboratorium Kesehatan Provinsi Jawa Barat	Azzania Fibriani, Catur Riani, Marselina Irasonia Tan, Yunia Sribudiani, Husna Nugrahapraja, Tarwadi, Ema Rahmawati, Savira Ekawardhani, Hesti Lina Wiraswati, Ryan Bayusantika Ristandi, Rifky Waluyajati Rachman, Cut Nur Cinthia Alamanda, Lia Faridah, Gusti Ayu Prani Pradani, Adelina Khristiani Rahayu, Hammam Riza, Sony Solistia Wirawan, Agung Eru Wibowo, Irvan Faizal
EPI_ISL_528749	Santosa Hospital Bandung Central	School of Life Sciences and Technology & School of Pharmacy-Institut Teknologi Bandung; Molecular Genetics Laboratory-Faculty of Medicine-Universitas Padjadjaran; Laboratorium Kesehatan Provinsi Jawa Barat	Husna Nugrahapraja, Azzania Fibriani, Catur Riani, Marselina Irasonia Tan, Yunia Sribudiani, Tarwadi, Ema Rahmawati, Savira Ekawardhani, Hesti Lina Wiraswati, Ryan Bayusantika Ristandi, Rifky Waluyajati Rachman, Cut Nur Cinthia Alamanda, Lia Faridah, Davin H. E. Setiamarga, Rizki Mardian, Hammam Riza, Sony Solistia Wirawan, Agung Eru Wibowo, Irvan Faizal

EPI_ISL_528750	Santo Borromeus Hospital	School of Life Sciences and Technology & School of Pharmacy-Institut Teknologi Bandung; Molecular Genetics Laboratory-Faculty of Medicine-Universitas Padjadjaran; Laboratorium Kesehatan Provinsi Jawa Barat	Marselina Irasonia Tan, Yunia Sribudiani, Catur Riani, Azzania Fibriani, Husna Nugrahapraja, Tarwadi, Ema Rahmawati, Savira Ekawardhani, Hesti Lina Wiraswati, Ryan Bayusantika Ristandi, Rifky Waluyajati Rachman, Cut Nur Cinthia Alamanda, Lia Faridah, Miftahul Faridl, Karimatu Khoirunnisa, Hammam Riza, Sony Solistia Wirawan, Agung Eru Wibowo, Irvan Faizal
EPI_ISL_528751	Santo Borromeus Hospital	Molecular Genetics Laboratory-Faculty of Medicine-Universitas Padjadjaran; School of Life Sciences and Technology & School of Pharmacy-Institut Teknologi Bandung; Laboratorium Kesehatan Provinsi Jawa Barat	Yunia Sribudiani, Tri Hanggono Achmad, Mas Rizky A.A. Syamsunarno, Fensi Amalina, Catur Riani, Azzania Fibriani, Husna Nugrahapraja, Marselina Irasonia Tan, Tarwadi, Ema Rahmawati, Savira Ekawardhani, Hesti Lina Wiraswati, Ryan Bayusantika Ristandi, Rifky Waluyajati Rachman, Cut Nur Cinthia Alamanda, Lia Faridah, Hammam Riza, Sony Solistia Wirawan, Agung Eru Wibowo, Irvan Faizal
EPI_ISL_528752	Dr. H. A. Rotinsulu Lung Hospital	School of Pharmacy & School of Life Sciences and Technology - Institut Teknologi Bandung; Molecular Genetics Laboratory-Faculty of Medicine-Universitas Padjadjaran; Laboratorium Kesehatan Provinsi Jawa Barat	Catur Riani, Marselina Irasonia Tan, Yunia Sribudiani, Azzania Fibriani, Husna Nugrahapraja, Tarwadi, Ema Rahmawati, Savira Ekawardhani, Hesti Lina Wiraswati, Ryan Bayusantika Ristandi, Rifky Waluyajati Rachman, Cut Nur Cinthia Alamanda, Lia Faridah, Gusti Ayu Prani Pradani, Adelina Khristiani Rahayu, Hammam Riza, Sony Solistia Wirawan, Agung Eru Wibowo, Irvan Faizal
EPI_ISL_528753	Dinkes Kota Bogor	School of Life Sciences and Technology & School of Pharmacy-Institut Teknologi Bandung; Molecular Genetics Laboratory-Faculty of Medicine-Universitas Padjadjaran; Laboratorium Kesehatan Provinsi Jawa Barat	Azzania Fibriani, Catur Riani, Marselina Irasonia Tan, Yunia Sribudiani, Husna Nugrahapraja, Tarwadi, Ema Rahmawati, Savira Ekawardhani, Hesti Lina Wiraswati, Ryan Bayusantika Ristandi, Rifky Waluyajati Rachman, Cut Nur Cinthia Alamanda, Lia Faridah, Davin H. E. Setiamarga, Rizki Mardian , Hammam Riza, Sony Solistia Wirawan, Agung Eru Wibowo, Irvan Faizal
EPI_ISL_528759	Santo Borromeus Hospital	School of Life Sciences and Technology & School of Pharmacy-Institut Teknologi Bandung; Molecular Genetics Laboratory-Faculty of Medicine-Universitas Padjadjaran; Laboratorium Kesehatan Provinsi Jawa Barat	Husna Nugrahapraja, Azzania Fibriani, Catur Riani, Marselina Irasonia Tan, Yunia Sribudiani, Tarwadi, Ema Rahmawati, Savira Ekawardhani, Hesti Lina Wiraswati, Ryan Bayusantika Ristandi, Rifky Waluyajati Rachman, Cut Nur Cinthia Alamanda, Lia Faridah, Tri Hanggono Achmad, Mas Rizky A.A. Syamsunarno, Fensi Amalina, Hammam Riza, Sony Solistia Wirawan, Agung Eru Wibowo, Irvan Faizal
EPI_ISL_528809	Department of Medicine, Gandhi hospital, Hyderabad	CSIR-Centre for Cellular and Molecular Biology	Rajarao Mesipogu , Thrilok Chander Bingi ,Vinayasekhar Aedula,Tulasi Nagabandi, Namami Gaur, Sakshi Shambhavi, Lamuk Zaveri, Shagufta Khan, Nikhil Hajirnis, M Soujanya Reddy, Pratheusa Maccha, Purushotham Vodnala, Payel Mukherjee, Sofia Banu, Priya Singh, Onkar Kulkarni, Dhiviya Vedagiri, Divya Gupta, Vishal Sah, Santosh Kumar Kuncha, Krishnan Harinivas Harshan, Archana Bharadwaj Siva, Karthik Bharadwaj Tallapaka,G. Aditya Kumar, Koushick Sivakumar, Pooja Ramesh Gupta, Rajan Kumar Jha, Shraddha Vijay Lahoti, Rakesh K Mishra, Divya Tej Sowpati
EPI_ISL_528810, EPI_ISL_528811, EPI_ISL_528812	Department of Medicine, Gandhi hospital, Hyderabad	CSIR-Centre for Cellular and Molecular Biology	Thrilok Chander Bingi,Rajarao Mesipogu ,Vinayasekhar Aedula,Lamuk Zaveri, Shagufta Khan, Namami Gaur, Sakshi Shambhavi, Nikhil Hajirnis, M Soujanya Reddy, Pratheusa Maccha, Tulasi Nagabandi, Purushotham Vodnala, Payel Mukherjee, Sofia Banu, Priya Singh, Onkar Kulkarni, Dhiviya Vedagiri, Divya Gupta, Vishal Sah, Santosh Kumar Kuncha, Krishnan Harinivas Harshan, Archana Bharadwaj Siva, Karthik Bharadwaj Tallapaka, Renu Sudhakar, Somesh Gorde, Gangumala Srinivas Reddy, Sujoy Deb, Swati Bayyana, Rakesh K Mishra, Divya Tej Sowpati
EPI_ISL_528813	Department of Medicine, Gandhi hospital, Hyderabad	CSIR-Centre for Cellular and Molecular Biology	Vinayasekhar Aedula,Thrilok Chander Bingi, Rajarao Mesipogu, Shagufta Khan, Lamuk Zaveri, Namami Gaur, Sakshi Shambhavi, Nikhil Hajirnis, M Soujanya Reddy, Pratheusa Maccha,Tulasi Nagabandi, Purushotham Vodnala, Payel Mukherjee, Sofia Banu, Priya Singh, Onkar Kulkarni, Dhiviya Vedagiri, Divya Gupta, Vishal Sah, Santosh Kumar Kuncha, Krishnan Harinivas Harshan, Archana Bharadwaj Siva, Karthik Bharadwaj Tallapaka,Umesh Kumar, Unis Ahmad Bhat, Ajay Sarawagi, Priyanka Pant, Rajkanwar Nathawat, Rakesh K Mishra, Divya Tej Sowpati
EPI_ISL_528814	Department of Medicine, Gandhi hospital, Hyderabad	CSIR-Centre for Cellular and Molecular Biology	Rajarao Mesipogu , Thrilok Chander Bingi ,Vinayasekhar Aedula,Tulasi Nagabandi, Namami Gaur, Sakshi Shambhavi, Lamuk Zaveri, Shagufta Khan, Nikhil Hajirnis, M Soujanya Reddy, Pratheusa Maccha, Purushotham Vodnala, Payel Mukherjee, Sofia Banu, Priya Singh, Onkar Kulkarni, Dhiviya Vedagiri, Divya Gupta, Vishal Sah, Santosh Kumar Kuncha, Krishnan Harinivas Harshan, Archana Bharadwaj Siva, Karthik Bharadwaj Tallapaka,G. Aditya Kumar, Koushick Sivakumar, Pooja Ramesh Gupta, Rajan Kumar Jha, Shraddha Vijay Lahoti, Rakesh K Mishra, Divya Tej Sowpati
EPI_ISL_528815	Department of Medicine, Gandhi hospital, Hyderabad	CSIR-Centre for Cellular and Molecular Biology	Vinayasekhar Aedula,Thrilok Chander Bingi, Rajarao Mesipogu, Shagufta Khan, Lamuk Zaveri, Namami Gaur, Sakshi Shambhavi, Nikhil Hajirnis, M Soujanya Reddy, Pratheusa Maccha,Tulasi Nagabandi, Purushotham Vodnala, Payel Mukherjee, Sofia Banu, Priya Singh, Onkar Kulkarni, Dhiviya Vedagiri, Divya Gupta, Vishal Sah, Santosh Kumar Kuncha, Krishnan Harinivas Harshan, Archana Bharadwaj Siva, Karthik Bharadwaj Tallapaka,Umesh Kumar, Unis Ahmad Bhat, Ajay Sarawagi, Priyanka Pant, Rajkanwar Nathawat, Rakesh K Mishra, Divya Tej Sowpati
EPI_ISL_528816	Department of Medicine, Gandhi hospital, Hyderabad	CSIR-Centre for Cellular and Molecular Biology	Rajarao Mesipogu , Thrilok Chander Bingi ,Vinayasekhar Aedula,Tulasi Nagabandi, Namami Gaur, Sakshi Shambhavi, Lamuk Zaveri, Shagufta Khan, Nikhil Hajirnis, M Soujanya Reddy, Pratheusa Maccha, Purushotham Vodnala, Payel Mukherjee, Sofia Banu, Priya Singh, Onkar Kulkarni, Dhiviya Vedagiri, Divya Gupta, Vishal Sah, Santosh Kumar Kuncha, Krishnan Harinivas Harshan, Archana Bharadwaj Siva, Karthik Bharadwaj Tallapaka,G. Aditya Kumar, Koushick Sivakumar, Pooja Ramesh Gupta, Rajan Kumar Jha, Shraddha Vijay Lahoti, Rakesh K Mishra, Divya Tej Sowpati
EPI_ISL_528817	Department of Medicine, Gandhi hospital, Hyderabad	CSIR-Centre for Cellular and Molecular Biology	Thrilok Chander Bingi,Rajarao Mesipogu ,Vinayasekhar Aedula,Lamuk Zaveri, Shagufta Khan, Namami Gaur, Sakshi Shambhavi, Nikhil Hajirnis, M Soujanya Reddy, Pratheusa Maccha, Tulasi Nagabandi, Purushotham Vodnala, Payel Mukherjee, Sofia Banu, Priya Singh, Onkar Kulkarni, Dhiviya Vedagiri, Divya Gupta, Vishal Sah, Santosh Kumar Kuncha, Krishnan Harinivas Harshan, Archana Bharadwaj Siva, Karthik Bharadwaj Tallapaka, Renu Sudhakar, Somesh Gorde, Gangumala Srinivas Reddy, Sujoy Deb, Swati Bayyana, Rakesh K Mishra, Divya Tej Sowpati
EPI_ISL_528818	Department of Medicine, Gandhi hospital, Hyderabad	CSIR-Centre for Cellular and Molecular Biology	Vinayasekhar Aedula,Thrilok Chander Bingi, Rajarao Mesipogu, Shagufta Khan, Lamuk Zaveri, Namami Gaur, Sakshi Shambhavi, Nikhil Hajirnis, M Soujanya Reddy, Pratheusa Maccha,Tulasi Nagabandi, Purushotham Vodnala, Payel Mukherjee, Sofia Banu, Priya Singh, Onkar Kulkarni, Dhiviya Vedagiri, Divya Gupta, Vishal Sah, Santosh Kumar Kuncha, Krishnan Harinivas Harshan, Archana Bharadwaj Siva, Karthik Bharadwaj Tallapaka,Umesh Kumar, Unis Ahmad Bhat, Ajay Sarawagi, Priyanka Pant, Rajkanwar Nathawat, Rakesh K Mishra, Divya Tej Sowpati
EPI_ISL_528819, EPI_ISL_528820	Department of Medicine, Gandhi hospital, Hyderabad	CSIR-Centre for Cellular and Molecular Biology	Rajarao Mesipogu , Thrilok Chander Bingi ,Vinayasekhar Aedula,Tulasi Nagabandi, Namami Gaur, Sakshi Shambhavi, Lamuk Zaveri, Shagufta Khan, Nikhil Hajirnis, M Soujanya Reddy, Pratheusa Maccha, Purushotham Vodnala, Payel Mukherjee, Sofia Banu, Priya Singh, Onkar Kulkarni, Dhiviya Vedagiri, Divya Gupta, Vishal Sah, Santosh Kumar Kuncha, Krishnan Harinivas Harshan, Archana Bharadwaj Siva, Karthik Bharadwaj Tallapaka,G. Aditya Kumar, Koushick Sivakumar, Pooja Ramesh Gupta, Rajan Kumar Jha, Shraddha Vijay Lahoti, Rakesh K Mishra, Divya Tej Sowpati
EPI_ISL_528821	Department of Medicine, Gandhi hospital, Hyderabad	CSIR-Centre for Cellular and Molecular Biology	Thrilok Chander Bingi,Rajarao Mesipogu ,Vinayasekhar Aedula,Lamuk Zaveri, Shagufta Khan, Namami Gaur, Sakshi Shambhavi, Nikhil Hajirnis, M Soujanya Reddy, Pratheusa Maccha, Tulasi Nagabandi, Purushotham Vodnala, Payel Mukherjee, Sofia Banu, Priya Singh, Onkar Kulkarni, Dhiviya Vedagiri, Divya Gupta, Vishal Sah, Santosh Kumar Kuncha, Krishnan Harinivas Harshan, Archana Bharadwaj Siva, Karthik Bharadwaj Tallapaka, Renu Sudhakar, Somesh Gorde, Gangumala Srinivas Reddy, Sujoy Deb, Swati Bayyana, Rakesh K Mishra, Divya Tej Sowpati
EPI_ISL_528822	Department of Medicine, Gandhi hospital, Hyderabad	CSIR-Centre for Cellular and Molecular Biology	Vinayasekhar Aedula,Thrilok Chander Bingi, Rajarao Mesipogu, Shagufta Khan, Lamuk Zaveri, Namami Gaur, Sakshi Shambhavi, Nikhil Hajirnis, M Soujanya Reddy, Pratheusa Maccha,Tulasi Nagabandi, Purushotham Vodnala, Payel Mukherjee, Sofia Banu, Priya Singh, Onkar Kulkarni, Dhiviya Vedagiri, Divya Gupta, Vishal Sah, Santosh Kumar Kuncha, Krishnan Harinivas Harshan, Archana Bharadwaj Siva, Karthik Bharadwaj Tallapaka,Umesh Kumar, Unis Ahmad Bhat, Ajay Sarawagi, Priyanka Pant, Rajkanwar Nathawat, Rakesh K Mishra, Divya Tej Sowpati
EPI_ISL_528919	Ospedale Civile S. Liberatore-Atri	Istituto Zooprofilattico Sperimentale dell'Abruzzo e Molise "G.Caporale"	Lorusso A, Marcacci M, Di Domenico M, Curini V, Ancora M, Cammà C, Rinaldi A, Mangone I, Di Pasquale A, Puglia I, Savini G.
EPI_ISL_528920, EPI_ISL_528921	Presidio Ospedaliero "Santo Spirito"-Pescara	Istituto Zooprofilattico Sperimentale dell'Abruzzo e Molise "G.Caporale"	Lorusso A, Marcacci M, Di Domenico M, Curini V, Ancora M, Cammà C, Rinaldi A, Mangone I, Di Pasquale A, Puglia I, Savini G.
EPI_ISL_528922	Ospedale "Giuseppe Mazzini"-Teramo	Istituto Zooprofilattico Sperimentale dell'Abruzzo e Molise "G.Caporale"	Lorusso A, Marcacci M, Di Domenico M, Curini V, Ancora M, Cammà C, Rinaldi A, Mangone I, Di Pasquale A, Puglia I, Savini G.
EPI_ISL_528923	Ospedale Civile S. Liberatore-Atri	Istituto Zooprofilattico Sperimentale dell'Abruzzo e Molise "G.Caporale"	Lorusso A, Marcacci M, Di Domenico M, Curini V, Ancora M, Cammà C, Rinaldi A, Mangone I, Di Pasquale A, Puglia I, Savini G.
EPI_ISL_528924	Ospedale "Giuseppe Mazzini"-Teramo	Istituto Zooprofilattico Sperimentale dell'Abruzzo e Molise "G.Caporale"	Lorusso A, Marcacci M, Di Domenico M, Curini V, Ancora M, Cammà C, Rinaldi A, Mangone I, Di Pasquale A, Puglia I, Savini G.
EPI_ISL_528925	Ospedale Regionale San Salvatore-L'Aquila	Istituto Zooprofilattico Sperimentale dell'Abruzzo e Molise "G.Caporale"	Lorusso A, Marcacci M, Di Domenico M, Curini V, Ancora M, Cammà C, Rinaldi A, Mangone I, Di Pasquale A, Puglia I, Savini G.

EPI_ISL_528926, EPI_ISL_528927, EPI_ISL_528928	Ospedale "Giuseppe Mazzini"-Teramo	Istituto Zooprofilattico Sperimentale dell'Abruzzo e Molise "G.Caporale"	Lorusso A, Marcacci M, Di Domenico M, Curini V, Ancora M, Cammà C, Rinaldi A, Mangone I, Di Pasquale A, Puglia I, Savini G.
EPI_ISL_528929	Ospedale Civile S. Liberatore-Atri	Istituto Zooprofilattico Sperimentale dell'Abruzzo e Molise "G.Caporale"	Lorusso A, Marcacci M, Di Domenico M, Curini V, Ancora M, Cammà C, Rinaldi A, Mangone I, Di Pasquale A, Puglia I, Savini G.
EPI_ISL_528934, EPI_ISL_528935, EPI_ISL_528936, EPI_ISL_528937, EPI_ISL_528938, EPI_ISL_528939, EPI_ISL_528940, EPI_ISL_528941, EPI_ISL_528942, EPI_ISL_528943, EPI_ISL_528944, EPI_ISL_528945, EPI_ISL_528946, EPI_ISL_528947, EPI_ISL_528948, EPI_ISL_528949			
see above	Agenzia di Tutela della Salute di Bergamo	Istituto Zooprofilattico Sperimentale dell'Abruzzo e Molise "G.Caporale"	Lorusso A, Marcacci M, Di Domenico M, Curini V, Ancora M, Cammà C, Rinaldi A, Mangone I, Di Pasquale A, Puglia I, Savini G.
EPI_ISL_528990	Ospedale Civile Maria SS. dello Splendore	Istituto Zooprofilattico Sperimentale dell'Abruzzo e Molise "G.Caporale"	Lorusso A, Marcacci M, Di Domenico M, Curini V, Ancora M, Cammà C, Rinaldi A, Mangone I, Di Pasquale A, Puglia I, Savini G.
EPI_ISL_528991, EPI_ISL_528992	Ospedale SS Annunziata-Sulmona	Istituto Zooprofilattico Sperimentale dell'Abruzzo e Molise "G.Caporale"	Lorusso A, Marcacci M, Di Domenico M, Curini V, Ancora M, Cammà C, Rinaldi A, Mangone I, Di Pasquale A, Puglia I, Savini G.
EPI_ISL_528993	Ospedale Civile S. Liberatore-Atri	Istituto Zooprofilattico Sperimentale dell'Abruzzo e Molise "G.Caporale"	Lorusso A, Marcacci M, Di Domenico M, Curini V, Ancora M, Cammà C, Rinaldi A, Mangone I, Di Pasquale A, Puglia I, Savini G.
EPI_ISL_528994, EPI_ISL_528995, EPI_ISL_528996, EPI_ISL_528997, EPI_ISL_528998, EPI_ISL_528999, EPI_ISL_529000, EPI_ISL_529001, EPI_ISL_529002, EPI_ISL_529003, EPI_ISL_529004, EPI_ISL_529005			
see above	Servizio di igiene epidemiologia e sanità pubblica (SIESP)-Chieti	Istituto Zooprofilattico Sperimentale dell'Abruzzo e Molise "G.Caporale"	Lorusso A, Marcacci M, Di Domenico M, Curini V, Ancora M, Cammà C, Rinaldi A, Mangone I, Di Pasquale A, Puglia I, Savini G.
EPI_ISL_529006	Servizio di igiene e sanità pubblica (SIESP)-Teramo	Istituto Zooprofilattico Sperimentale dell'Abruzzo e Molise "G.Caporale"	Lorusso A, Marcacci M, Di Domenico M, Curini V, Ancora M, Cammà C, Rinaldi A, Mangone I, Di Pasquale A, Puglia I, Savini G.
EPI_ISL_529007	Ospedale Civile S. Liberatore-Atri	Istituto Zooprofilattico Sperimentale dell'Abruzzo e Molise "G.Caporale"	Lorusso A, Marcacci M, Di Domenico M, Curini V, Ancora M, Cammà C, Rinaldi A, Mangone I, Di Pasquale A, Puglia I, Savini G.
EPI_ISL_529008	Servizio di igiene e sanità pubblica (SIESP)-Teramo	Istituto Zooprofilattico Sperimentale dell'Abruzzo e Molise "G.Caporale"	Lorusso A, Marcacci M, Di Domenico M, Curini V, Ancora M, Cammà C, Rinaldi A, Mangone I, Di Pasquale A, Puglia I, Savini G.
EPI_ISL_529009	Ospedale Civile S. Liberatore-Atri	Istituto Zooprofilattico Sperimentale dell'Abruzzo e Molise "G.Caporale"	Lorusso A, Marcacci M, Di Domenico M, Curini V, Ancora M, Cammà C, Rinaldi A, Mangone I, Di Pasquale A, Puglia I, Savini G.
EPI_ISL_529010, EPI_ISL_529011, EPI_ISL_529012	Servizio Igiene Epidemiologia e Sanità Pubblica (SIESP)-L'Aquila	Istituto Zooprofilattico Sperimentale dell'Abruzzo e Molise "G.Caporale"	Lorusso A, Marcacci M, Di Domenico M, Curini V, Ancora M, Cammà C, Rinaldi A, Mangone I, Di Pasquale A, Puglia I, Savini G.
EPI_ISL_529013	Presidio Ospedaliero "S.Filippo e Nicola"-Avezzano	Istituto Zooprofilattico Sperimentale dell'Abruzzo e Molise "G.Caporale"	Lorusso A, Marcacci M, Di Domenico M, Curini V, Ancora M, Cammà C, Rinaldi A, Mangone I, Di Pasquale A, Puglia I, Savini G.
EPI_ISL_529014, EPI_ISL_529015	Ospedale "Ss. Annunziata"	Istituto Zooprofilattico Sperimentale dell'Abruzzo e Molise "G.Caporale"	Lorusso A, Marcacci M, Di Domenico M, Curini V, Ancora M, Cammà C, Rinaldi A, Mangone I, Di Pasquale A, Puglia I, Savini G.
EPI_ISL_529016	Ospedale SS Annunziata-Sulmona	Istituto Zooprofilattico Sperimentale dell'Abruzzo e Molise "G.Caporale"	Lorusso A, Marcacci M, Di Domenico M, Curini V, Ancora M, Cammà C, Rinaldi A, Mangone I, Di Pasquale A, Puglia I, Savini G.
EPI_ISL_529017	RSA San Raffaele Sulmona	Istituto Zooprofilattico Sperimentale dell'Abruzzo e Molise "G.Caporale"	Lorusso A, Marcacci M, Di Domenico M, Curini V, Ancora M, Cammà C, Rinaldi A, Mangone I, Di Pasquale A, Puglia I, Savini G.
EPI_ISL_529018	Ospedale "Giuseppe Mazzini"-Teramo	Istituto Zooprofilattico Sperimentale dell'Abruzzo e Molise "G.Caporale"	Lorusso A, Marcacci M, Di Domenico M, Curini V, Ancora M, Cammà C, Rinaldi A, Mangone I, Di Pasquale A, Puglia I, Savini G.
EPI_ISL_529019	RSA/RP Villa San Giovanni - Gruppo Edos	Istituto Zooprofilattico Sperimentale dell'Abruzzo e Molise "G.Caporale"	Lorusso A, Marcacci M, Di Domenico M, Curini V, Ancora M, Cammà C, Rinaldi A, Mangone I, Di Pasquale A, Puglia I, Savini G.
EPI_ISL_529020, EPI_ISL_529021	Ospedale Civile S. Liberatore-Atri	Istituto Zooprofilattico Sperimentale dell'Abruzzo e Molise "G.Caporale"	Lorusso A, Marcacci M, Di Domenico M, Curini V, Ancora M, Cammà C, Rinaldi A, Mangone I, Di Pasquale A, Puglia I, Savini G.
EPI_ISL_529022	Ospedale "Ss. Annunziata"	Istituto Zooprofilattico Sperimentale dell'Abruzzo e Molise "G.Caporale"	Lorusso A, Marcacci M, Di Domenico M, Curini V, Ancora M, Cammà C, Rinaldi A, Mangone I, Di Pasquale A, Puglia I, Savini G.
EPI_ISL_529023, EPI_ISL_529024, EPI_ISL_529025	Servizio di igiene epidemiologia e sanità pubblica (SIESP)-Chieti	Istituto Zooprofilattico Sperimentale dell'Abruzzo e Molise "G.Caporale"	Lorusso A, Marcacci M, Di Domenico M, Curini V, Ancora M, Cammà C, Rinaldi A, Mangone I, Di Pasquale A, Puglia I, Savini G.
EPI_ISL_529026	Ospedale "Giuseppe Mazzini"-Teramo	Istituto Zooprofilattico Sperimentale dell'Abruzzo e Molise "G.Caporale"	Lorusso A, Marcacci M, Di Domenico M, Curini V, Ancora M, Cammà C, Rinaldi A, Mangone I, Di Pasquale A, Puglia I, Savini G.
EPI_ISL_529030	Kingston Health Sciences Center	Queen's Genomics Lab at Ongwanada (Q-GLO)	Sjaarda CP, Rustom N, Huang D, Perez-Patrigeon S, Hudson ML, Wong H,Guan H, Ayub M, Soares CN, Colautti R, Evans GA, Sheth P
EPI_ISL_529031	Central Molecular Microbiology Laboratory, Clinical and Chemical Pathology Department, Faculty of Medicine, CAIRO UNIVERSITY	Next Generation Sequencing Reference Laboratory, Faculty of Medicine, Cairo University and The Center for Genome and Microbiome Research, Faculty of Pharmacy, CAIRO UNIVERSITY	May Sherif Soliman, May Abdelfattah, Ramy Karam Aziz
EPI_ISL_529032	Central Molecular Microbiology Laboratory and Next Generation Sequencing Reference Laboratory, Clinical and Chemical Pathology Department, Faculty of Medicine, CAIRO UNIVERSITY	Next Generation Sequencing Reference Laboratory, Faculty of Medicine, CAIRO UNIVERSITY and The Center for Genome and Microbiome Research, Faculty of Pharmacy, CAIRO UNIVERSITY	May Sherif Soliman, May Abdelfattah, Ramy Karam Aziz
EPI_ISL_529035, EPI_ISL_529036	Wadsworth Center, New York State Department of Health	Wadsworth Center, New York State Department of Health	Kirsten St. George, Daryl M. Lamson, Sara Griesemer, Jonathan Plitnick, Navjot Singh, Matthew D. Shudt, Erica Lasek-Nesselquist
EPI_ISL_529138	RSAL Dr. Ramelan Surabaya	Institute of Tropical Disease, Universitas Airlangga	Jezzy R Dewantari, Rima R Prasetya, Krisnoadi Rahardjo, Aldise M Nastri, Radito Soesanto, Gatot Soegiarto, Laksmi Wulandari, Retno A Setyoningrum, Resti Yudhawati, Yohko K Shimizu, Mitsuhiro Nishimura, Yasuko Mori, Soetjipto, Kazufumi Shimizu, Maria I Lusida
EPI_ISL_529718	Santo Borromeus Hospital	Laboratorium Kesehatan Provinsi Jawa Barat; School of Life Sciences and Technology & School of Pharmacy-Institut Teknologi Bandung; Molecular Genetics Laboratory-Faculty of Medicine-Universitas Padjadjaran	Ema Rahmawati, Marselina Irasonia Tan, Yunia Sribudiani, Catur Riani, Azzania Fibriani, Husna Nugrahapraja, Tarwadi, Savira Ekawardhani, Hesti Lina Wiraswati, Ryan Bayusantika Ristandi, Rifky Waluyajati Rachman, Cut Nur Cinthia Alamanda, Lia Faridah, Gusti Ayu Prani Pradani, Adelina Khristiani Rahayu, Hammam Riza, Sony Solistia Wirawan, Agung Eru Wibowo, Irvan Faizal
EPI_ISL_529719	Sumedang Regional General Hospital	Laboratorium Kesehatan Provinsi Jawa Barat; School of Life Sciences and Technology & School of Pharmacy-Institut Teknologi Bandung; Molecular Genetics Laboratory-Faculty of Medicine-Universitas Padjadjaran	Ema Rahmawati, Marselina Irasonia Tan, Yunia Sribudiani, Catur Riani, Azzania Fibriani, Husna Nugrahapraja, Tarwadi, Savira Ekawardhani, Hesti Lina Wiraswati, Ryan Bayusantika Ristandi, Rifky Waluyajati Rachman, Cut Nur Cinthia Alamanda, Lia Faridah, Gusti Ayu Prani Pradani, Adelina Khristiani Rahayu, Hammam Riza, Sony Solistia Wirawan, Agung Eru Wibowo, Irvan Faizal
EPI_ISL_529720, EPI_ISL_529721, EPI_ISL_529722, EPI_ISL_529723, EPI_ISL_529724, EPI_ISL_529725, EPI_ISL_529726, EPI_ISL_529727, EPI_ISL_529728, EPI_ISL_529729, EPI_ISL_529730, EPI_ISL_529731, EPI_ISL_529732, EPI_ISL_529733, EPI_ISL_529734, EPI_ISL_529735, EPI_ISL_529736, EPI_ISL_529737, EPI_ISL_529738, EPI_ISL_529739, EPI_ISL_529740, EPI_ISL_529741, EPI_ISL_529742, EPI_ISL_529743, EPI_ISL_529744, EPI_ISL_529745, EPI_ISL_529746, EPI_ISL_529747, EPI_ISL_529748, EPI_ISL_529749, EPI_ISL_529750, EPI_ISL_529751, EPI_ISL_529752, EPI_ISL_529753, EPI_ISL_529754, EPI_ISL_529755, EPI_ISL_529756, EPI_ISL_529757, EPI_ISL_529758, EPI_ISL_529759, EPI_ISL_529760, EPI_ISL_529761, EPI_ISL_529762, EPI_ISL_529763, EPI_ISL_529764, EPI_ISL_529765, EPI_ISL_529766, EPI_ISL_529767, EPI_ISL_529768, EPI_ISL_529769, EPI_ISL_529770, EPI_ISL_529771, EPI_ISL_529772, EPI_ISL_529773, EPI_ISL_529774, EPI_ISL_529775, EPI_ISL_529776, EPI_ISL_529777, EPI_ISL_529778, EPI_ISL_529779, EPI_ISL_529780, EPI_ISL_529781, EPI_ISL_529782, EPI_ISL_529783, EPI_ISL_529784, EPI_ISL_529785, EPI_ISL_529786, EPI_ISL_529787, EPI_ISL_529788, EPI_ISL_529789, EPI_ISL_529790, EPI_ISL_529791, EPI_ISL_529792, EPI_ISL_529793, EPI_ISL_529794, EPI_ISL_529795, EPI_ISL_529796, EPI_ISL_529797, EPI_ISL_529798, EPI_ISL_529799, EPI_ISL_529800, EPI_ISL_529801, EPI_ISL_529802, EPI_ISL_529803			
see above	NHLS-IALCH	KRISP, KZN Research Innovation and Sequencing Platform	Giandhari J, Pillay S, Lessells R, Mdlalose K, York D, Khan S, Tegally H, Wilkinson E, de Oliveira T

EPI_ISL_529961	Husada Utama Hospital	Institute of Tropical Disease, Universitas Airlangga	Krisnoadi Rahardjo, Aldise M Nastri, Jezzy R Dewantari, Rima R Prasetya, Didi Dewanto, Gatot Soegiarto, Laksmi Wulandari, Retno A Setyoningrum, Resti Yudhawati, Yohko K Shimizu, Mitsuhiro Nishimura, Yasuko Mori, Soetjipto, Kazufumi Shimizu, Maria I Lusida
EPI_ISL_529962	Universitas Airlangga Hospital	Institute of Tropical Disease, Universitas Airlangga	Jezzy R Dewantari, Rima R Prasetya, Krisnoadi Rahardjo, Aldise M Nastri, Nasronudin, Gatot Soegiarto, Laksmi Wulandari, Retno A Setyoningrum, Resti Yudhawati, Yohko K Shimizu, Mitsuhiro Nishimura, Yasuko Mori, Soetjipto, Kazufumi Shimizu, Maria I Lusida
EPI_ISL_529963	Universitas Airlangga Hospital	Institute of Tropical Disease, Universitas Airlangga	Rima R Prasetya, Krisnoadi Rahardjo, Aldise M Nastri, Jezzy R Dewantari, Nasronudin, Gatot Soegiarto, Laksmi Wulandari, Retno A Setyoningrum, Resti Yudhawati, Yohko K Shimizu, Mitsuhiro Nishimura, Yasuko Mori, Soetjipto, Kazufumi Shimizu, Maria I Lusida
EPI_ISL_529964	RSUD Bangil Pasuruan	Institute of Tropical Disease, Universitas Airlangga	Aldise M Nastri, Jezzy R Dewantari, Rima R Prasetya, Krisnoadi Rahardjo, Arma Roosalina, Gatot Soegiarto, Laksmi Wulandari, Retno A Setyoningrum, Resti Yudhawati, Yohko K Shimizu, Mitsuhiro Nishimura, Yasuko Mori, Soetjipto, Kazufumi Shimizu, Maria I Lusida
EPI_ISL_529965	RSUD Dr. Soetomo	Institute of Tropical Disease, Universitas Airlangga	Krisnoadi Rahardjo, Aldise M Nastri, Jezzy R Dewantari, Rima R Prasetya, Joni Wahyuhadi, Gatot Soegiarto, Laksmi Wulandari, Retno A Setyoningrum, Resti Yudhawati, Yohko K Shimizu, Mitsuhiro Nishimura, Yasuko Mori, Soetjipto, Kazufumi Shimizu, Maria I Lusida
EPI_ISL_529966	RSUD Sidoarjo	Institute of Tropical Disease, Universitas Airlangga	Kazufumi Shimizu, Krisnoadi Rahardjo, Aldise M Nastri, Jezzy R Dewantari, Rima R Prasetya, Atok Irawan, Gatot Soegiarto, Laksmi Wulandari, Retno A Setyoningrum, Resti Yudhawati, Yohko K Shimizu, Mitsuhiro Nishimura, Yasuko Mori, Soetjipto, Maria I Lusida
EPI_ISL_529967	RSUD Sidoarjo	Institute of Tropical Disease, Universitas Airlangga	Aldise M Nastri, Jezzy R Dewantari, Rima R Prasetya, Krisnoadi Rahardjo, Atok Irawan, Gatot Soegiarto, Laksmi Wulandari, Retno A Setyoningrum, Resti Yudhawati, Yohko K Shimizu, Mitsuhiro Nishimura, Yasuko Mori, Soetjipto, Kazufumi Shimizu, Maria I Lusida
EPI_ISL_530347, EPI_ISL_530348, EPI_ISL_530349, EPI_ISL_530350	The National Institute of Public Health	State Veterinary Institute Prague	Nagy,A;Jirincova,H;Novakova,L;Trnka,D;Vecerova,J
EPI_ISL_534311	UPA III 26 de Agosto	Instituto Adolfo Lutz, Interdiciplinary Procedures Center, Strategic Laboratory	Claudio Tavares Sacchi, Claudia Regina Gonçalves, Erica Valessa Ramos Gomes
EPI_ISL_534312	Distrito Sanitario Sul	Instituto Adolfo Lutz, Interdiciplinary Procedures Center, Strategic Laboratory	Claudio Tavares Sacchi, Claudia Regina Gonçalves, Erica Valessa Ramos Gomes
EPI_ISL_534313	Hospital da Sta Casa de Sto Amaro	Instituto Adolfo Lutz, Interdiciplinary Procedures Center, Strategic Laboratory	Claudio Tavares Sacchi, Claudia Regina Gonçalves, Erica Valessa Ramos Gomes
EPI_ISL_534314	Hospital Universitario da USP de SP	Instituto Adolfo Lutz, Interdiciplinary Procedures Center, Strategic Laboratory	Claudio Tavares Sacchi, Claudia Regina Gonçalves, Erica Valessa Ramos Gomes
EPI_ISL_534315	Serviço de Verificação de Óbitos SVO Guaruulhos	Instituto Adolfo Lutz, Interdiciplinary Procedures Center, Strategic Laboratory	Claudio Tavares Sacchi, Claudia Regina Gonçalves, Erica Valessa Ramos Gomes
EPI_ISL_534316	OS Mun Santana Lauro Ribas Braga	Instituto Adolfo Lutz, Interdiciplinary Procedures Center, Strategic Laboratory	Claudio Tavares Sacchi, Claudia Regina Gonçalves, Erica Valessa Ramos Gomes
EPI_ISL_534317	Hospital Geral de Itapevi	Instituto Adolfo Lutz, Interdiciplinary Procedures Center, Strategic Laboratory	Claudio Tavares Sacchi, Claudia Regina Gonçalves, Erica Valessa Ramos Gomes
EPI_ISL_534318	Hospital Municipal Antonio Giglio	Instituto Adolfo Lutz, Interdiciplinary Procedures Center, Strategic Laboratory	Claudio Tavares Sacchi, Claudia Regina Gonçalves, Erica Valessa Ramos Gomes
EPI_ISL_534319, EPI_ISL_534320	Hospital do Serv Pub ESTAFCO Morato de Oliveira	Instituto Adolfo Lutz, Interdiciplinary Procedures Center, Strategic Laboratory	Claudio Tavares Sacchi, Claudia Regina Gonçalves, Erica Valessa Ramos Gomes
EPI_ISL_534321	PS e Maternidade Nair Fonseca Leitao Arantes	Instituto Adolfo Lutz, Interdiciplinary Procedures Center, Strategic Laboratory	Claudio Tavares Sacchi, Claudia Regina Gonçalves, Erica Valessa Ramos Gomes
EPI_ISL_534322	PS Mun Julio Tupy	Instituto Adolfo Lutz, Interdiciplinary Procedures Center, Strategic Laboratory	Claudio Tavares Sacchi, Claudia Regina Gonçalves, Erica Valessa Ramos Gomes
EPI_ISL_534323	Hospital e Pronto Socorro Comunitario Vila Yolanda	Instituto Adolfo Lutz, Interdiciplinary Procedures Center, Strategic Laboratory	Claudio Tavares Sacchi, Claudia Regina Gonçalves, Erica Valessa Ramos Gomes
EPI_ISL_534324	Hospital Mun Ver Jose Storopolli	Instituto Adolfo Lutz, Interdiciplinary Procedures Center, Strategic Laboratory	Claudio Tavares Sacchi, Claudia Regina Gonçalves, Erica Valessa Ramos Gomes
EPI_ISL_534325	Unidade de Vigilancia em Saude de Guarulhos	Instituto Adolfo Lutz, Interdiciplinary Procedures Center, Strategic Laboratory	Claudio Tavares Sacchi, Claudia Regina Gonçalves, Erica Valessa Ramos Gomes
EPI_ISL_534326	Notre Dame Intermedica Saude AS	Instituto Adolfo Lutz, Interdiciplinary Procedures Center, Strategic Laboratory	Claudio Tavares Sacchi, Claudia Regina Gonçalves, Erica Valessa Ramos Gomes
EPI_ISL_534335	LabPLUS	Institute of Environmental Science and Research (ESR)	Matt Storey, Xiaoyun Ren, Anja Werno, Antje van der Linden, Arlo Upton, Chris Mansell, David Hammer, Dragana Drinkovic, Gary McAuliffe, Hana Sofia Andersson, James Ussher, Jill Sherwood, Josh Freeman, Julia Howard, Juliet Elvy, Mary DeAlmeida, Matt Blakiston, Matthew Rogers, Max Bloomfield, Michael Addidle, Michelle Balm, Sally Roberts, Sarah Jefferies, Sharmini Muttaiyah, Susan Morpeth, Susan Taylor, Timothy Blackmore, Vani Sathyendran, Veronica Playle, Virginia Hope, Erasmus Smit, Lauren Jelly, Joep de Ligt
EPI_ISL_534336	Department of Laboratory Medicine, National Taiwan University Hospital	Microbial Genomics Core Lab, National Taiwan University Centers of Genomic and Precision Medicine	Shiou-Hwei Yeh, You-Yu Lin, Ya-Yun Lai, Chiao-Ling Li, Shan-Chwen Chang, Pei-Jer Chen, Sui-Yuan Chang
EPI_ISL_534337, EPI_ISL_534338, EPI_ISL_534339, EPI_ISL_534340, EPI_ISL_534341, EPI_ISL_534342, EPI_ISL_534343, EPI_ISL_534344, EPI_ISL_534345	Molecular diagnostic laboratory of Federal Budget Institution of Science "Central Research Institute of Epidemiology" of The Federal Service on Customers' Rights Protection and Human Well-being Surveillance	Group of Genomics and Postgenomic Technologies of Central Research Institute of Epidemilology	Speranskaya AS, Kaptelova VV, Samoilov AE, Valdokhina AV, Bulanenko VP, Bukharina A.Y., Tivanova EV, Shipulina OY, Akimkin VG
EPI_ISL_534346, EPI_ISL_534347, EPI_ISL_534348, EPI_ISL_534349	Molecular diagnostic laboratory of Federal Budget Institution of Science "Central Research Institute of Epidemiology" of The Federal Service on Customers' Rights Protection and Human Well-being Surveillance	Group of Genomics and Postgenomic Technologies of Central Research Institute of Epidemilology	Speranskaya AS, Kaptelova VV, Valdokhina AV, Bulanenko VP, Samoilov AE, Korneenko EV, Tivanova EV, Shipulina OY, Akimkin VG
EPI_ISL_535390, EPI_ISL_535391, EPI_ISL_535392, EPI_ISL_535393, EPI_ISL_535394, EPI_ISL_535395, EPI_ISL_535396, EPI_ISL_535397, EPI_ISL_535398, EPI_ISL_535399, EPI_ISL_535400, EPI_ISL_535401, EPI_ISL_535402, EPI_ISL_535403, EPI_ISL_535404, EPI_ISL_535405, EPI_ISL_535406, EPI_ISL_535407, EPI_ISL_535408, EPI_ISL_535409, EPI_ISL_535410, EPI_ISL_535411, EPI_ISL_535412, EPI_ISL_535413, EPI_ISL_535414, EPI_ISL_535415, EPI_ISL_535416, EPI_ISL_535417, EPI_ISL_535418, EPI_ISL_535419, EPI_ISL_535420, EPI_ISL_535421, EPI_ISL_535422, EPI_ISL_535423, EPI_ISL_535424, EPI_ISL_535425, EPI_ISL_535426, EPI_ISL_535427, EPI_ISL_535428, EPI_ISL_535429, EPI_ISL_535430, EPI_ISL_535431, EPI_ISL_535432, EPI_ISL_535433, EPI_ISL_535434, EPI_ISL_535435, EPI_ISL_535436, EPI_ISL_535437, EPI_ISL_535438, EPI_ISL_535439, EPI_ISL_535440, EPI_ISL_535441, EPI_ISL_535442, EPI_ISL_535443, EPI_ISL_535444, EPI_ISL_535445, EPI_ISL_535446, EPI_ISL_535447, EPI_ISL_535448, EPI_ISL_535449, EPI_ISL_535450, EPI_ISL_535451, EPI_ISL_535452, EPI_ISL_535453, EPI_ISL_535454, EPI_ISL_535455, EPI_ISL_535456, EPI_ISL_535457, EPI_ISL_535458, EPI_ISL_535459, EPI_ISL_535460, EPI_ISL_535461, EPI_ISL_535462, EPI_ISL_535463, EPI_ISL_535464, EPI_ISL_535465, EPI_ISL_535466, EPI_ISL_535467, EPI_ISL_535468, EPI_ISL_535469, EPI_ISL_535470, EPI_ISL_535471, EPI_ISL_535472, EPI_ISL_535473, EPI_ISL_535474, EPI_ISL_535475, EPI_ISL_535476, EPI_ISL_535477, EPI_ISL_535478, EPI_ISL_535479, EPI_ISL_535480, EPI_ISL_535481, EPI_ISL_535482, EPI_ISL_535483, EPI_ISL_535484, EPI_ISL_535485, EPI_ISL_535486, EPI_ISL_535487, EPI_ISL_535488, EPI_ISL_535496, EPI_ISL_535497, EPI_ISL_535498, EPI_ISL_535499, EPI_ISL_535500, EPI_ISL_535501, EPI_ISL_535502, EPI_ISL_535503, EPI_ISL_535504, EPI_ISL_535505, EPI_ISL_535506, EPI_ISL_535507, EPI_ISL_535508, EPI_ISL_535509, EPI_ISL_535510, EPI_ISL_535511, EPI_ISL_535512, EPI_ISL_535513, EPI_ISL_535514, EPI_ISL_535515, EPI_ISL_535516, EPI_ISL_535517, EPI_ISL_535518, EPI_ISL_535519, EPI_ISL_535520, EPI_ISL_535521, EPI_ISL_535522, EPI_ISL_535523, EPI_ISL_535524, EPI_ISL_535525, EPI_ISL_535526, EPI_ISL_535527, EPI_ISL_535528, EPI_ISL_535529, EPI_ISL_535530, EPI_ISL_535531, EPI_ISL_535532, EPI_ISL_535533, EPI_ISL_535534, EPI_ISL_535535, EPI_ISL_535536, EPI_ISL_535537, EPI_ISL_535538, EPI_ISL_535539, EPI_ISL_535540, EPI_ISL_535541, EPI_ISL_535542, EPI_ISL_535543, EPI_ISL_535544, EPI_ISL_535545, EPI_ISL_535546, EPI_ISL_535547, EPI_ISL_535548, EPI_ISL_535549, EPI_ISL_535550, EPI_ISL_535551, EPI_ISL_535552, EPI_ISL_535553, EPI_ISL_535554, EPI_ISL_535555, EPI_ISL_535556, EPI_ISL_535557, EPI_ISL_535558, EPI_ISL_535559, EPI_ISL_535560, EPI_ISL_535561, EPI_ISL_535562, EPI_ISL_535563, EPI_ISL_535564, EPI_ISL_535565, EPI_ISL_535566, EPI_ISL_535567, EPI_ISL_535568, EPI_ISL_535569, EPI_ISL_535570, EPI_ISL_535571, EPI_ISL_535572			
see above	NHLS-IALCH	KRISP, KZN Research Innovation and Sequencing Platform	Giandhari J, Pillay S, Lessells R, Mdlalose K, York D, Khan S, Tegally H, Wilkinson E, de Oliveira T
EPI_ISL_535575	Hospital Universitari Germans Trias i Pujol(HUGTiP)/Fundació	IrsiCaixa AIDS Research Lab	Marc Noguera-Julian, Mariona Parera, Maria Pilar Armengol, Marta Massanella, Ester Ballana, Lidia Ruiz, Nuria Izquierdo, Jorge Carrillo, Roger Paredes,

	Lluita contra la SIDA (FLSida)		Julia Blanco, Joaquim Segalés, Bonaventura Clotet
EPI_ISL_536399	Laboratory of Immunovirology. Universidad de Antioquia	Instituto Nacional de Salud - Unidad de Secuenciación y Genómica	Wbeimar Aguilar-Jimenez, Lizdany Flórez, Francisco J. Díaz, Katherine Laiton-Donato, Carlos Franco-Muñoz, Diego Álvarez-Diaz and Marcela Mercado-Reyes
EPI_ISL_536411, EPI_ISL_536793	Medtimes Molecular Laboratory	Medtimes Molecular Laboratory	Eric Chan, Winsome Wong, Jacqueline Tam, Isaac Chow
EPI_ISL_538435, EPI_ISL_538436, EPI_ISL_538437, EPI_ISL_538438, EPI_ISL_538439, EPI_ISL_538440, EPI_ISL_538441, EPI_ISL_538442, EPI_ISL_538443, EPI_ISL_538444, EPI_ISL_538445, EPI_ISL_538446, EPI_ISL_538447, EPI_ISL_538448, EPI_ISL_538449, EPI_ISL_538450, EPI_ISL_538451, EPI_ISL_538452, EPI_ISL_538453, EPI_ISL_538454, EPI_ISL_538455, EPI_ISL_538456, EPI_ISL_538457, EPI_ISL_538458, EPI_ISL_538459, EPI_ISL_538460, EPI_ISL_538461, EPI_ISL_538462, EPI_ISL_538463, EPI_ISL_538464, EPI_ISL_538465, EPI_ISL_538466, EPI_ISL_538467, EPI_ISL_538468, EPI_ISL_538469, EPI_ISL_538470, EPI_ISL_538471, EPI_ISL_538472, EPI_ISL_538473, EPI_ISL_538474, EPI_ISL_538475, EPI_ISL_538476, EPI_ISL_538477, EPI_ISL_538478, EPI_ISL_538479, EPI_ISL_538480, EPI_ISL_538481, EPI_ISL_538482, EPI_ISL_538483, EPI_ISL_538484, EPI_ISL_538485, EPI_ISL_538486, EPI_ISL_538487, EPI_ISL_538488, EPI_ISL_538489, EPI_ISL_538490, EPI_ISL_538491, EPI_ISL_538492, EPI_ISL_538493, EPI_ISL_538494, EPI_ISL_538495, EPI_ISL_538496, EPI_ISL_538497			
see above	Department of Laboratory Medicine, Tan Tock Seng Hospital	Department of Laboratory Medicine, Tan Tock Seng Hospital	Chen YYC, Zair X, Lim JX, Li C, Tang WY, Maurer-Stroh S, Barkham TMS, Nagarajan N, Sessions OM
EPI_ISL_538500, EPI_ISL_538501	RS Prima Husada Cipta Medan North Sumatra	National Institute of Health Research and Development	Pawestri, HA; Subangkit; Puspa, KD; Nugraha, AA; Ikawati, HD; Pangesti, KNA; Soekarso, T; Paisal; Setiawaty,V.
EPI_ISL_538502	RSUD Sultan Imanudin Pangkalan Bun Center Kalimantan	National Institute of Health Research and Development	Pawestri, HA; Subangkit; Puspa, KD; Nugraha, AA; Ikawati, HD; Pangesti, KNA; Soekarso, T; Paisal; Setiawaty,V.
EPI_ISL_538552	Hospital Universitari Germans Trias i Pujol(HUGTiP)/Fundació Lluita contra la SIDA (FLSida)	IrsiCaixa AIDS Research Lab	Marc Noguera-Julian, Mariona Parera, Maria Pilar Armengol, Marta Massanella, Ester Ballana, Lidia Ruiz, Nuria Izquierdo, Jorge Carrillo, Roger Paredes, Julia Blanco, Joaquim Segalés, Bonaventura Clotet
EPI_ISL_539299	Molecular diagnostic laboratory of Federal Budget Institution of Science "Central Research Institute of Epidemiology" of The Federal Service on Customers' Rights Protection and Human Well-being Surveillance	Group of Genomics and Postgenomic Technologies of Central Research Institute of Epidemilology	Speranskaya AS, Kaptelova VV, Valdokhina AV, Bulanenko VP, Samoilov AE, Korneenko EV, Tivanova EV, Shipulina OY, Akimkin VG
EPI_ISL_539493, EPI_ISL_539494	The National Institute of Public Health	State Veterinary Institute Prague	Nagy,A;Jirincova,H;Novakova,L;Trnka,D;Vecerova,J
EPI_ISL_539495	Centers for Disease Control and Prevention, Dengue Branch	Centers for Disease Control and Prevention, Dengue Branch	Gilberto A. Santiago, Glenda Gonzalez, Betzabel Flores, Keyla Charriez, Jorge L. Munoz-Jordan, Gabriela Paz-Bailey, Janice Perez, Vanessa Rivera-Amill, Diego Sainz de la Peña, Jorge Bertran
EPI_ISL_539496	Hospital Nostra Senyora de Meritxell	Instituto de Salud Carlos III	Iglesias-Caballero, M. Molinero Calamita, M. González-Esguevillas, M. Camarero, S. Pozo, F. Casas, I. Jiménez, P. Jiménez, M. Zaballos, A. Monzón, S. Varona, S. Juliá, M. Cuesta, I, F. Fernández
EPI_ISL_539497, EPI_ISL_539498	Hospital Virgen de las Nieves	Instituto de Salud Carlos III	Iglesias-Caballero, M. Molinero Calamita, M. González-Esguevillas, M. Camarero, S. Pozo, F. Casas, I. Jiménez, P. Jiménez, M. Zaballos, A. Monzón, S. Varona, S. Juliá, M. Cuesta, I, J. Lepe
EPI_ISL_539499	Hospital Costa del Sol	Instituto de Salud Carlos III	Iglesias-Caballero, M. Molinero Calamita, M. González-Esguevillas, M. Camarero, S. Pozo, F. Casas, I. Jiménez, P. Jiménez, M. Zaballos, A. Monzón, S. Varona, S. Juliá, M. Cuesta, I, F. Fernández
EPI_ISL_539500, EPI_ISL_539501, EPI_ISL_539502	Hospital Clínico Universitario Lozano Blesa	Instituto de Salud Carlos III	Iglesias-Caballero, M. Molinero Calamita, M. González-Esguevillas, M. Camarero, S. Pozo, F. Casas, I. Jiménez, P. Jiménez, M. Zaballos, A. Monzón, S. Varona, S. Juliá, M. Cuesta, I, R. Benito
EPI_ISL_539503	Hospital Universitario Miguel Servet	Instituto de Salud Carlos III	Iglesias-Caballero, M. Molinero Calamita, M. González-Esguevillas, M. Camarero, S. Pozo, F. Casas, I. Jiménez, P. Jiménez, M. Zaballos, A. Monzón, S. Varona, S. Juliá, M. Cuesta, I, A. Rezusta
EPI_ISL_539504	Hospital Universitario Miguel Servet	Instituto de Salud Carlos III	Iglesias-Caballero, M. Molinero Calamita, M. González-Esguevillas, M. Camarero, S. Pozo, F. Casas, I. Jiménez, P. Jiménez, M. Zaballos, A. Monzón, S. Varona, S. Juliá, M. Cuesta, I, R. Benito
EPI_ISL_539505, EPI_ISL_539506, EPI_ISL_539507, EPI_ISL_539508	Hospital Universitario Miguel Servet	Instituto de Salud Carlos III	Iglesias-Caballero, M. Molinero Calamita, M. González-Esguevillas, M. Camarero, S. Pozo, F. Casas, I. Jiménez, P. Jiménez, M. Zaballos, A. Monzón, S. Varona, S. Juliá, M. Cuesta, I, A. Rezusta
EPI_ISL_539509, EPI_ISL_539510, EPI_ISL_539511, EPI_ISL_539512	Hospital Clínico Universitario Lozano Blesa	Instituto de Salud Carlos III	Iglesias-Caballero, M. Molinero Calamita, M. González-Esguevillas, M. Camarero, S. Pozo, F. Casas, I. Jiménez, P. Jiménez, M. Zaballos, A. Monzón, S. Varona, S. Juliá, M. Cuesta, I, R. Benito
EPI_ISL_539513, EPI_ISL_539514, EPI_ISL_539515, EPI_ISL_539516, EPI_ISL_539517, EPI_ISL_539518, EPI_ISL_539519	Hospital Universitario Miguel Servet	Instituto de Salud Carlos III	Iglesias-Caballero, M. Molinero Calamita, M. González-Esguevillas, M. Camarero, S. Pozo, F. Casas, I. Jiménez, P. Jiménez, M. Zaballos, A. Monzón, S. Varona, S. Juliá, M. Cuesta, I, A. Rezusta
EPI_ISL_539520, EPI_ISL_539521	Hospital Universitario de Ceuta	Instituto de Salud Carlos III	Iglesias-Caballero, M. Molinero Calamita, M. González-Esguevillas, M. Camarero, S. Pozo, F. Casas, I. Jiménez, P. Jiménez, M. Zaballos, A. Monzón, S. Varona, S. Juliá, M. Cuesta, I, J. López
EPI_ISL_539522	Hospital Universitario de Ceuta	Instituto de Salud Carlos III	Iglesias-Caballero, M. Molinero Calamita, M. González-Esguevillas, M. Camarero, S. Pozo, F. Casas, I. Jiménez, P. Jiménez, M. Zaballos, A. Monzón, S. Varona, S. Juliá, M. Cuesta, I, G. Sánchez
EPI_ISL_539523	Hospital General de Segovia	Instituto de Salud Carlos III	Iglesias-Caballero, M. Molinero Calamita, M. González-Esguevillas, M. Camarero, S. Pozo, F. Casas, I. Jiménez, P. Jiménez, M. Zaballos, A. Monzón, S. Varona, S. Juliá, M. Cuesta, I, S. Hernando
EPI_ISL_539524	Gerencia de Asistencia Sanitaria de Soria	Instituto de Salud Carlos III	Iglesias-Caballero, M. Molinero Calamita, M. González-Esguevillas, M. Camarero, S. Pozo, F. Casas, I. Jiménez, P. Jiménez, M. Zaballos, A. Monzón, S. Varona, S. Juliá, M. Cuesta, I, C. Aldea
EPI_ISL_539525	Hospital Nuestra Señora de Sonsoles	Instituto de Salud Carlos III	Iglesias-Caballero, M. Molinero Calamita, M. González-Esguevillas, M. Camarero, S. Pozo, F. Casas, I. Jiménez, P. Jiménez, M. Zaballos, A. Monzón, S. Varona, S. Juliá, M. Cuesta, I, A. San Pedro
EPI_ISL_539526, EPI_ISL_539527, EPI_ISL_539528, EPI_ISL_539529, EPI_ISL_539530	Consejeria de Sanidad y Asuntos Sociales	Instituto de Salud Carlos III	Iglesias-Caballero, M. Molinero Calamita, M. González-Esguevillas, M. Camarero, S. Pozo, F. Casas, I. Jiménez, P. Jiménez, M. Zaballos, A. Monzón, S. Varona, S. Juliá, M. Cuesta, I, G. Gutiérrez
EPI_ISL_539531	C.H.U Nuestra Señora de Candelaria	Instituto de Salud Carlos III	Iglesias-Caballero, M. Molinero Calamita, M. González-Esguevillas, M. Camarero, S. Pozo, F. Casas, I. Jiménez, P. Jiménez, M. Zaballos, A. Monzón, S. Varona, S. Juliá, M. Cuesta, I, O. Díez
EPI_ISL_539532	Hospital Universitario de Canarias	Instituto de Salud Carlos III	Iglesias-Caballero, M. Molinero Calamita, M. González-Esguevillas, M. Camarero, S. Pozo, F. Casas, I. Jiménez, P. Jiménez, M. Zaballos, A. Monzón, S. Varona, S. Juliá, M. Cuesta, I, B. Castro
EPI_ISL_539533, EPI_ISL_539534, EPI_ISL_539535, EPI_ISL_539536, EPI_ISL_539537, EPI_ISL_539538, EPI_ISL_539539, EPI_ISL_539540, EPI_ISL_539541, EPI_ISL_539542, EPI_ISL_539543, EPI_ISL_539544, EPI_ISL_539545, EPI_ISL_539546, EPI_ISL_539547, EPI_ISL_539548, EPI_ISL_539549, EPI_ISL_539550, EPI_ISL_539551, EPI_ISL_539552, EPI_ISL_539553, EPI_ISL_539554, EPI_ISL_539555, EPI_ISL_539556			
see above	Hospital Clínic	Instituto de Salud Carlos III	Iglesias-Caballero, M. Molinero Calamita, M. González-Esguevillas, M. Camarero, S. Pozo, F. Casas, I. Jiménez, P. Jiménez, M. Zaballos, A. Monzón, S. Varona, S. Juliá, M. Cuesta, I, M.A Marcos
EPI_ISL_539557	Gerencia del área de salud de Badajoz, Llerena y Zafra	Instituto de Salud Carlos III	Iglesias-Caballero, M. Molinero Calamita, M. González-Esguevillas, M. Camarero, S. Pozo, F. Casas, I. Jiménez, P. Jiménez, M. Zaballos, A. Monzón, S. Varona, S. Juliá, M. Cuesta, I, C. Pazos
EPI_ISL_539558	Hospital San Pedro de Alcántara	Instituto de Salud Carlos III	Iglesias-Caballero, M. Molinero Calamita, M. González-Esguevillas, M. Camarero, S. Pozo, F. Casas, I. Jiménez, P. Jiménez, M. Zaballos, A. Monzón, S. Varona, S. Juliá, M. Cuesta, I, E. Cerro
EPI_ISL_539559	Hospital Campo Arañuelo	Instituto de Salud Carlos III	Iglesias-Caballero, M. Molinero Calamita, M. González-Esguevillas, M. Camarero, S. Pozo, F. Casas, I. Jiménez, P. Jiménez, M. Zaballos, A. Monzón, S. Varona, S. Juliá, M. Cuesta, I, J. López
EPI_ISL_539560	Hospital Campo Arañuelo	Instituto de Salud Carlos III	Iglesias-Caballero, M. Molinero Calamita, M. González-Esguevillas, M. Camarero, S. Pozo, F. Casas, I. Jiménez, P. Jiménez, M. Zaballos, A. Monzón, S. Varona, S. Juliá, M. Cuesta, I, G. Rodríguez
EPI_ISL_539561	Hospital San Pedro de Alcántara	Instituto de Salud Carlos III	Iglesias-Caballero, M. Molinero Calamita, M. González-Esguevillas, M. Camarero, S. Pozo, F. Casas, I. Jiménez, P. Jiménez, M. Zaballos, A. Monzón, S. Varona, S. Juliá, M. Cuesta, I, J. López

EPI_ISL_539562	Hospital San Pedro de Alcántara	Instituto de Salud Carlos III	Iglesias-Caballero, M. Molinero Calamita, M. González-Esguevillas, M. Camarero, S. Pozo, F. Casas, I. Jiménez, P. Jiménez, M. Zaballos, A. Monzón, S. Varona, S. Juliá, M. Cuesta, I, M.A Cañizares
EPI_ISL_539563	Complejo Hospitalario Universitario La Coruna	Instituto de Salud Carlos III	Iglesias-Caballero, M. Molinero Calamita, M. González-Esguevillas, M. Camarero, S. Pozo, F. Casas, I. Jiménez, P. Jiménez, M. Zaballos, A. Monzón, S. Varona, S. Juliá, M. Cuesta, I, J. López
EPI_ISL_539564	Complejo Hospitalario Universitario La Coruna	Instituto de Salud Carlos III	Iglesias-Caballero, M. Molinero Calamita, M. González-Esguevillas, M. Camarero, S. Pozo, F. Casas, I. Jiménez, P. Jiménez, M. Zaballos, A. Monzón, S. Varona, S. Juliá, M. Cuesta, I, M.I Paz
EPI_ISL_539565	Xerencia de Xestión Integrada de Pontevedra e o Salnés	Instituto de Salud Carlos III	Iglesias-Caballero, M. Molinero Calamita, M. González-Esguevillas, M. Camarero, S. Pozo, F. Casas, I. Jiménez, P. Jiménez, M. Zaballos, A. Monzón, S. Varona, S. Juliá, M. Cuesta, I, J. López
EPI_ISL_539566	Xerencia de Xestión Integrada de Pontevedra e o Salnés	Instituto de Salud Carlos III	Iglesias-Caballero, M. Molinero Calamita, M. González-Esguevillas, M. Camarero, S. Pozo, F. Casas, I. Jiménez, P. Jiménez, M. Zaballos, A. Monzón, S. Varona, S. Juliá, M. Cuesta, I, I. Pérez
EPI_ISL_539567	Hospital Comarcal de Melilla	Instituto de Salud Carlos III	Iglesias-Caballero, M. Molinero Calamita, M. González-Esguevillas, M. Camarero, S. Pozo, F. Casas, I. Jiménez, P. Jiménez, M. Zaballos, A. Monzón, S. Varona, S. Juliá, M. Cuesta, I, J. López
EPI_ISL_539568	Hospital Comarcal de Melilla	Instituto de Salud Carlos III	Iglesias-Caballero, M. Molinero Calamita, M. González-Esguevillas, M. Camarero, S. Pozo, F. Casas, I. Jiménez, P. Jiménez, M. Zaballos, A. Monzón, S. Varona, S. Juliá, M. Cuesta, I, C. Ezpeleta
EPI_ISL_539569, EPI_ISL_539570, EPI_ISL_539571, EPI_ISL_539572	Complejo Hospitalario de Navarra	Instituto de Salud Carlos III	Iglesias-Caballero, M. Molinero Calamita, M. González-Esguevillas, M. Camarero, S. Pozo, F. Casas, I. Jiménez, P. Jiménez, M. Zaballos, A. Monzón, S. Varona, S. Juliá, M. Cuesta, I, J. López
EPI_ISL_539776	National Institute of Public Health (Czech Republic)	State Veterinary Institute Prague	Nagy, A; Jirincova, H; Novakova, L; Trnka, D; Vecerova, J.
EPI_ISL_539777	The National Institute of Public Health	State Veterinary Institute Prague	Nagy,A;Jirincova,H;Novakova,L;Trnka,D;Vecerova,J
EPI_ISL_539778, EPI_ISL_539779, EPI_ISL_539780, EPI_ISL_539781, EPI_ISL_539782	National Institute of Public Health (Czech Republic)	State Veterinary Institute Prague	Nagy, A; Jirincova, H; Novakova, L; Trnka, D; Vecerova, J.
EPI_ISL_539803	Queen Mary Hospital	Hong Kong Department of Health	Alan K.L. Tsang, Peter C.W. Yip, Edman T.K. Lam, Rickjason C.W. Chan, Dominic N.C. Tsang
EPI_ISL_539804	Yan Chai Hospital	Hong Kong Department of Health	Alan K.L. Tsang, Peter C.W. Yip, Edman T.K. Lam, Rickjason C.W. Chan, Dominic N.C. Tsang
EPI_ISL_539805	Kwong Wah Hospital	Hong Kong Department of Health	Alan K.L. Tsang, Peter C.W. Yip, Edman T.K. Lam, Rickjason C.W. Chan, Dominic N.C. Tsang
EPI_ISL_539806, EPI_ISL_539807, EPI_ISL_539808	Princess Margaret Hospital	Hong Kong Department of Health	Alan K.L. Tsang, Peter C.W. Yip, Edman T.K. Lam, Rickjason C.W. Chan, Dominic N.C. Tsang
EPI_ISL_539809, EPI_ISL_539811, EPI_ISL_539812	Asiaworld Expo Command Post	Hong Kong Department of Health	Alan K.L. Tsang, Peter C.W. Yip, Edman T.K. Lam, Rickjason C.W. Chan, Dominic N.C. Tsang
EPI_ISL_539813	Prince of Wales Hospital	Hong Kong Department of Health	Alan K.L. Tsang, Peter C.W. Yip, Edman T.K. Lam, Rickjason C.W. Chan, Dominic N.C. Tsang
EPI_ISL_539814	Tuen Mun Hospital	Hong Kong Department of Health	Alan K.L. Tsang, Peter C.W. Yip, Edman T.K. Lam, Rickjason C.W. Chan, Dominic N.C. Tsang
EPI_ISL_539815	Queen Elizabeth Hospital	Hong Kong Department of Health	Alan K.L. Tsang, Peter C.W. Yip, Edman T.K. Lam, Rickjason C.W. Chan, Dominic N.C. Tsang
EPI_ISL_539816, EPI_ISL_539817	Queen Mary Hospital	Hong Kong Department of Health	Alan K.L. Tsang, Peter C.W. Yip, Edman T.K. Lam, Rickjason C.W. Chan, Dominic N.C. Tsang
EPI_ISL_539818, EPI_ISL_539819	Tuen Mun Hospital	Hong Kong Department of Health	Alan K.L. Tsang, Peter C.W. Yip, Edman T.K. Lam, Rickjason C.W. Chan, Dominic N.C. Tsang
EPI_ISL_539820	Queen Mary Hospital	Hong Kong Department of Health	Alan K.L. Tsang, Peter C.W. Yip, Edman T.K. Lam, Rickjason C.W. Chan, Dominic N.C. Tsang
EPI_ISL_539821	Asiaworld Expo Command Post	Hong Kong Department of Health	Alan K.L. Tsang, Peter C.W. Yip, Edman T.K. Lam, Rickjason C.W. Chan, Dominic N.C. Tsang
EPI_ISL_539822, EPI_ISL_539823	Communicable Disease Branch	Hong Kong Department of Health	Alan K.L. Tsang, Peter C.W. Yip, Edman T.K. Lam, Rickjason C.W. Chan, Dominic N.C. Tsang
EPI_ISL_539850, EPI_ISL_539851	Pok Oi Hospital	Hong Kong Department of Health	Alan K.L. Tsang, Peter C.W. Yip, Edman T.K. Lam, Rickjason C.W. Chan, Dominic N.C. Tsang
EPI_ISL_540993, EPI_ISL_540994, EPI_ISL_540995, EPI_ISL_540996, EPI_ISL_540997, EPI_ISL_540998, EPI_ISL_540999, EPI_ISL_541000, EPI_ISL_541001, EPI_ISL_541002, EPI_ISL_541003, EPI_ISL_541004, EPI_ISL_541005, EPI_ISL_541006, EPI_ISL_541007			
see above	Health and Environmental Research Institute of Gwangju Metropolitan city	Health and Environmental Research Institute of Gwangju Metropolitan city	Min Ji Kim, Ji-eun Lee
EPI_ISL_541082	The National Institute of Public Health	State Veterinary Institute Prague	Nagy,A;Jirincova,H;Novakova,L;Trnka,D;Vecerova,J
EPI_ISL_541138, EPI_ISL_541332, EPI_ISL_541333, EPI_ISL_541334	The National Institute of Public Health	State Veterinary Institute Prague	Nagy,A; Jirincova,H; Novakova,L; Trnka,D; Vecerova,J
EPI_ISL_541335	The National Institute of Public Health	Sídlištní 136/24 165 03, Prague Czech Republic	Nagy,A; Jirincova,H; Novakova,L; Trnka,D; Vecerova,J
EPI_ISL_541336, EPI_ISL_541337	The National Institute of Public Health	State Veterinary Institute Prague	Nagy,A; Jirincova,H; Novakova,L; Trnka,D; Vecerova,J
EPI_ISL_541352	Laboratory of Respiratory Viruses and Measles, Oswaldo Cruz Institute, FIOCRUZ	Laboratory of Respiratory Viruses and Measles, Oswaldo Cruz Institute, FIOCRUZ	Paola Resende, Luciana Appolinario, Fernando Motta, Anna Carolina Paixão, Ana Carolina Mendonça, Jonathan Lopes, Marilda Siqueira
EPI_ISL_541372, EPI_ISL_541374, EPI_ISL_541375, EPI_ISL_541376, EPI_ISL_541379, EPI_ISL_541383, EPI_ISL_541385	LACEN/SE	Laboratory of Respiratory Viruses and Measles, Oswaldo Cruz Institute, FIOCRUZ	Paola Resende, Luciana Appolinario, Fernando Motta, Anna Carolina Paixão, Ana Carolina Mendonça, Jonathan Lopes, Clioma Santos, Marilda Siqueira
EPI_ISL_541942	Health and Environmental Research Institute of Gwangju	Health & Environment Institute of Gwangju	Min Ji Kim, Ji-eun Lee
EPI_ISL_541970	Influenza Centre, University of Bergen	Norwegian Institute of Public Health, Department of Virology	Fan Zhou, Rebecca J Cox, Karl A Brokstad, Bjørn Blomberg, Kathrine Stene-Johansen, Kamilla Heddeland Instefjord, Hilde Elshaug, Rasmus Riis Kopperud, Hilde Synnøve Vollan, Karoline Bragstad, Olav Hungnes
EPI_ISL_542099, EPI_ISL_542101, EPI_ISL_542104, EPI_ISL_542106, EPI_ISL_542107, EPI_ISL_542108, EPI_ISL_542110, EPI_ISL_542112, EPI_ISL_542114, EPI_ISL_542116, EPI_ISL_542118, EPI_ISL_542119, EPI_ISL_542120, EPI_ISL_542121, EPI_ISL_542122, EPI_ISL_542124, EPI_ISL_542128, EPI_ISL_542130, EPI_ISL_542131, EPI_ISL_542133, EPI_ISL_542145, EPI_ISL_542146, EPI_ISL_542152, EPI_ISL_542153, EPI_ISL_542154, EPI_ISL_542155, EPI_ISL_542156, EPI_ISL_542158, EPI_ISL_542159, EPI_ISL_542162, EPI_ISL_542165, EPI_ISL_542166, EPI_ISL_542169, EPI_ISL_542170, EPI_ISL_542172, EPI_ISL_542173, EPI_ISL_542174, EPI_ISL_542175, EPI_ISL_542178, EPI_ISL_542180, EPI_ISL_542181, EPI_ISL_542183, EPI_ISL_542192, EPI_ISL_542193, EPI_ISL_542194, EPI_ISL_542195, EPI_ISL_542196, EPI_ISL_542197, EPI_ISL_542198, EPI_ISL_542199, EPI_ISL_542200, EPI_ISL_542201, EPI_ISL_542202, EPI_ISL_542203, EPI_ISL_542204, EPI_ISL_542205, EPI_ISL_542206, EPI_ISL_542207, EPI_ISL_542208, EPI_ISL_542209, EPI_ISL_542210, EPI_ISL_542211, EPI_ISL_542212, EPI_ISL_542213, EPI_ISL_542214, EPI_ISL_542215, EPI_ISL_542216, EPI_ISL_542217, EPI_ISL_542218, EPI_ISL_542219, EPI_ISL_542220, EPI_ISL_542221, EPI_ISL_542222, EPI_ISL_542223, EPI_ISL_542224, EPI_ISL_542225, EPI_ISL_542226, EPI_ISL_542227, EPI_ISL_542228, EPI_ISL_542229, EPI_ISL_542230, EPI_ISL_542231, EPI_ISL_542232, EPI_ISL_542233, EPI_ISL_542234, EPI_ISL_542236, EPI_ISL_542237, EPI_ISL_542238, EPI_ISL_542239, EPI_ISL_542240, EPI_ISL_542241, EPI_ISL_542242, EPI_ISL_542243, EPI_ISL_542248, EPI_ISL_542249, EPI_ISL_542252, EPI_ISL_542253, EPI_ISL_542254, EPI_ISL_542255, EPI_ISL_542256, EPI_ISL_542257, EPI_ISL_542258, EPI_ISL_542260, EPI_ISL_542261, EPI_ISL_542262, EPI_ISL_542263, EPI_ISL_542264, EPI_ISL_542274, EPI_ISL_542275, EPI_ISL_542276, EPI_ISL_542277			
see above	ASST GOM Niguarda	Dep. Of Oncology and Hemato-Oncology University of Milan	Claudia Alteri, Valeria Cento, Antonio Piralla, Valentino Costabile, Monica Tallarita, Luna Colagrossi, Silvia Renica, Federica Giardina, Federica Novazzi, Stefano Gaiarsa, Elisa Matarazzo, Maria Antonello, Chiara Vismara, Roberto Fumagalli, Oscar Massimiliano Epis, Massimo Puoti, Carlo Federico Perno, Fausto Baldanti
EPI_ISL_542278, EPI_ISL_542279, EPI_ISL_542280, EPI_ISL_542281, EPI_ISL_542282, EPI_ISL_542283, EPI_ISL_542284, EPI_ISL_542285, EPI_ISL_542286, EPI_ISL_542287, EPI_ISL_542288, EPI_ISL_542289, EPI_ISL_542290, EPI_ISL_542291, EPI_ISL_542292, EPI_ISL_542294, EPI_ISL_542297, EPI_ISL_542299, EPI_ISL_542302, EPI_ISL_542303, EPI_ISL_542304, EPI_ISL_542305, EPI_ISL_542306, EPI_ISL_542307, EPI_ISL_542308, EPI_ISL_542309, EPI_ISL_542310, EPI_ISL_542311, EPI_ISL_542312, EPI_ISL_542313, EPI_ISL_542314, EPI_ISL_542315, EPI_ISL_542316, EPI_ISL_542317, EPI_ISL_542318, EPI_ISL_542319, EPI_ISL_542320, EPI_ISL_542321, EPI_ISL_542322, EPI_ISL_542323, EPI_ISL_542324, EPI_ISL_542325, EPI_ISL_542326, EPI_ISL_542327, EPI_ISL_542328, EPI_ISL_542329, EPI_ISL_542330, EPI_ISL_542331, EPI_ISL_542332, EPI_ISL_542333, EPI_ISL_542334, EPI_ISL_542335, EPI_ISL_542336, EPI_ISL_542337,			

EPI_ISL_542338, EPI_ISL_542339, EPI_ISL_542340, EPI_ISL_542341, EPI_ISL_542342, EPI_ISL_542343, EPI_ISL_542344, EPI_ISL_542345, EPI_ISL_542346, EPI_ISL_542347, EPI_ISL_542348, EPI_ISL_542349, EPI_ISL_542350, EPI_ISL_542351, EPI_ISL_542352, EPI_ISL_542353, EPI_ISL_542354, EPI_ISL_542355, EPI_ISL_542357, EPI_ISL_542358, EPI_ISL_542359, EPI_ISL_542360, EPI_ISL_542361, EPI_ISL_542362, EPI_ISL_542363, EPI_ISL_542364, EPI_ISL_542365, EPI_ISL_542366, EPI_ISL_542367, EPI_ISL_542369, EPI_ISL_542370, EPI_ISL_542371, EPI_ISL_542372, EPI_ISL_542373, EPI_ISL_542374, EPI_ISL_542375, EPI_ISL_542376, EPI_ISL_542377, EPI_ISL_542378, EPI_ISL_542379, EPI_ISL_542381, EPI_ISL_542382, EPI_ISL_542383, EPI_ISL_542384, EPI_ISL_542385, EPI_ISL_542386, EPI_ISL_542387, EPI_ISL_542388, EPI_ISL_542389, EPI_ISL_542390, EPI_ISL_542391, EPI_ISL_542392			
see above	San Matteo Hospital Pavia	Dep. Of Oncology and Hemato-Oncology University of Milan	Claudia Alteri, Valeria Cento, Antonio Piralla, Valentino Costabile, Monica Tallarita, Luna Colagrossi, Silvia Renica, Federica Giardina, Federica Novazzi, Stefano Gaiarsa, Elisa Matarazzo, Maria Antonello, Chiara Vismara, Roberto Fumagalli, Oscar Massimiliano Epis, Massimo Puoti, Carlo Federico Perno, Fausto Baldanti
EPI_ISL_542413, EPI_ISL_542415, EPI_ISL_542419, EPI_ISL_542420, EPI_ISL_542421, EPI_ISL_542422, EPI_ISL_542423, EPI_ISL_542424, EPI_ISL_542425, EPI_ISL_542426, EPI_ISL_542427, EPI_ISL_542428, EPI_ISL_542429, EPI_ISL_542431, EPI_ISL_542432, EPI_ISL_542433, EPI_ISL_542435, EPI_ISL_542440, EPI_ISL_542442, EPI_ISL_542443			
see above	ASST GOM Niguarda	Dep. Of Oncology and Hemato-Oncology University of Milan	Claudia Alteri, Valeria Cento, Antonio Piralla, Valentino Costabile, Monica Tallarita, Luna Colagrossi, Silvia Renica, Federica Giardina, Federica Novazzi, Stefano Gaiarsa, Elisa Matarazzo, Maria Antonello, Chiara Vismara, Roberto Fumagalli, Oscar Massimiliano Epis, Massimo Puoti, Carlo Federico Perno, Fausto Baldanti
EPI_ISL_545580, EPI_ISL_545954, EPI_ISL_545956	The National Institute of Public Health	State Veterinary Institute Prague	Nagy,A;Jirincova,H;Novakova,L;Trnka,D;Vecerova,J
EPI_ISL_545957, EPI_ISL_546436, EPI_ISL_546935, EPI_ISL_546936	The National Institute of Public Health	State Veterinary Institute Prague	Nagy,A; Jirincova,H; Novakova,L; Trnka,D; Vecerova,J
EPI_ISL_547570	PS Municipal Dr Augusto Gomes de Mattos	Instituto Adolfo Lutz, Interdiciplinary Procedures Center, Strategic Laboratory	Claudio Tavares Sacchi, Claudia Regina Gonçalves, Erica Valessa Ramos Gomes, Karoline Rodrigues Campos
EPI_ISL_547571	Hospital Municipal Antônio Giglio	Instituto Adolfo Lutz, Interdiciplinary Procedures Center, Strategic Laboratory	Claudio Tavares Sacchi, Claudia Regina Gonçalves, Erica Valessa Ramos Gomes, Karoline Rodrigues Campos
EPI_ISL_547573	Vigilância em Saúde de Cajamar	Instituto Adolfo Lutz, Interdiciplinary Procedures Center, Strategic Laboratory	Claudio Tavares Sacchi, Claudia Regina Gonçalves, Erica Valessa Ramos Gomes, Karoline Rodrigues Campos
EPI_ISL_547574	Hospital Universitario da USP	Instituto Adolfo Lutz, Interdiciplinary Procedures Center, Strategic Laboratory	Claudio Tavares Sacchi, Claudia Regina Gonçalves, Erica Valessa Ramos Gomes, Karoline Rodrigues Campos
EPI_ISL_547575	SVO Jundiaí	Instituto Adolfo Lutz, Interdiciplinary Procedures Center, Strategic Laboratory	Claudio Tavares Sacchi, Claudia Regina Gonçalves, Erica Valessa Ramos Gomes, Karoline Rodrigues Campos
EPI_ISL_547576	Secretaria Municipal de Saúde	Instituto Adolfo Lutz, Interdiciplinary Procedures Center, Strategic Laboratory	Claudio Tavares Sacchi, Claudia Regina Gonçalves, Erica Valessa Ramos Gomes, Karoline Rodrigues Campos
EPI_ISL_547577	Hospital e Maternidade Nossa Senhora das Graças	Instituto Adolfo Lutz, Interdiciplinary Procedures Center, Strategic Laboratory	Claudio Tavares Sacchi, Claudia Regina Gonçalves, Erica Valessa Ramos Gomes, Karoline Rodrigues Campos
EPI_ISL_547578	Hospital Doutor Domingos Leonardo Cerávolo	Instituto Adolfo Lutz, Interdiciplinary Procedures Center, Strategic Laboratory	Claudio Tavares Sacchi, Claudia Regina Gonçalves, Erica Valessa Ramos Gomes, Karoline Rodrigues Campos
EPI_ISL_547579	Santa Casa de Misericórdia de Araçatuba	Instituto Adolfo Lutz, Interdiciplinary Procedures Center, Strategic Laboratory	Claudio Tavares Sacchi, Claudia Regina Gonçalves, Erica Valessa Ramos Gomes, Karoline Rodrigues Campos
EPI_ISL_547580	Santa Casa da Misericórdia de Presidente Prudente	Instituto Adolfo Lutz, Interdiciplinary Procedures Center, Strategic Laboratory	Claudio Tavares Sacchi, Claudia Regina Gonçalves, Erica Valessa Ramos Gomes, Karoline Rodrigues Campos
EPI_ISL_547965	Laboratorio Biologia Molecolare SarsCov2 UOC LAboratorio Analisi SErvizio Medicina di Laboratorio Ospedale San Francesco ATS-ASSL Nuoro	Laboratorio Specialistico UOC Ematologia Ospedale San Francesco - ATS ASSL NUORO	Piras Giovanna, Asproni Rosanna, Monne Maria Itria, Fancello Tatiana,Fiamma Maura,Toja Alessandro, Sanna Filomena, Floris Anna Rita, Sulis Vincenzo, Palmas Angelo Domenico, Casu Gavino, Lo Maglio Iana, Mameli Giuseppe.
EPI_ISL_547966, EPI_ISL_547967, EPI_ISL_547968	The National Institute of Public Health	State Veterinary Institute Prague	Nagy,A;Jirincova,H;Novakova,L;Trnka,D;Vecerova,J
EPI_ISL_548208, EPI_ISL_548214	Laboratoire de Virologie, HUG	Swiss National Reference Centre for Influenza	LAUBSCHER F.
EPI_ISL_548243, EPI_ISL_548244	Faith Laboratory, Immunology Institute, Icahn School of Medicine at Mount Sinai	van Bakel Laboratory, Genetics and Genomics Sciences, Icahn School of Medicine at Mount Sinai	Graham J. Britton, Alice Chen-Liaw, Francesca Cossarini, Alexandra Livanos, Matthew P. Spindler, Tamar Plitt, Joseph Eggers, Ilaria Mogno, Ana S. Gonzalez-Reiche, Sophia Sui, Michael Tankelevich, Lauren Tal Grinspan, Rebekah E. Dixon, Divya Jha, Gustavo Martinez-Delgado, Fatima Amanat, Daisy Hoagland, Benjamin R. tenOever, Marla C. Dubinsky, Miriam Merad, Harm Van Bakel, Florian Krammer, Gerold Bongers, Saurabh Mehandru and Jeremiah J. Faith
EPI_ISL_548966, EPI_ISL_548967, EPI_ISL_548968, EPI_ISL_548969, EPI_ISL_548970, EPI_ISL_548971	Expo2020 Emergency Center	Agiomix	Walaa Allam, Cherif Ben Hamada, Cengiz Yakicier, Walid Dridi, Rashid Mohammed, Tamer Degheidy
EPI_ISL_560407	Istituto Zooprofilattico Sperimentale del Mezzogiorno	INMI Lazzaro Spallanzani IRCCS	Barbara Bartolini, Cesare E.M. Gruber, Martina Rueca, Francesco Messina, Antonino Di Caro, Giovanna Fusco, Maurizio Viscardi, Giorgia Borriello, Sergio Brandi, Maria R. Capobianchi
EPI_ISL_560408, EPI_ISL_560409, EPI_ISL_560410, EPI_ISL_560411, EPI_ISL_560412	The National Institute of Public Health	State Veterinary Institute Prague	Nagy, A; Jirincova, H; Novakova, L; Trnka, D; Vecerova, J
EPI_ISL_560568, EPI_ISL_560569, EPI_ISL_560570, EPI_ISL_560571, EPI_ISL_560572, EPI_ISL_560573, EPI_ISL_560574, EPI_ISL_560575, EPI_ISL_560576, EPI_ISL_560577, EPI_ISL_560578, EPI_ISL_560579, EPI_ISL_560580			
see above	hôpital	National Reference Center for Viruses of Respiratory Infections, Institut Pasteur, Paris	Sylvie Behillil, Fabiana Gambaro, Etienne Simon-Lorière, Vincent Enouf, Maud Vanpeene, Sylvie van der Werf
EPI_ISL_560581, EPI_ISL_560582, EPI_ISL_560583, EPI_ISL_560584, EPI_ISL_560585, EPI_ISL_560586, EPI_ISL_560587, EPI_ISL_560588, EPI_ISL_560589, EPI_ISL_560590	Hopital	National Reference Center for Viruses of Respiratory Infections, Institut Pasteur, Paris	Sylvie Behillil, Fabiana Gambaro, Etienne Simon-Lorière, Vincent Enouf, Maud Vanpeene, Sylvie van der Werf
EPI_ISL_560591, EPI_ISL_560592, EPI_ISL_560593, EPI_ISL_560594, EPI_ISL_560595, EPI_ISL_560596, EPI_ISL_560597	hopital	National Reference Center for Viruses of Respiratory Infections, Institut Pasteur, Paris	Sylvie Behillil, Fabiana Gambaro, Etienne Simon-Lorière, Vincent Enouf, Maud Vanpeene, Sylvie van der Werf
EPI_ISL_560598, EPI_ISL_560599, EPI_ISL_560600, EPI_ISL_560601, EPI_ISL_560602, EPI_ISL_560603, EPI_ISL_560604, EPI_ISL_560605, EPI_ISL_560606, EPI_ISL_560607, EPI_ISL_560608, EPI_ISL_560609, EPI_ISL_560610, EPI_ISL_560611, EPI_ISL_560612, EPI_ISL_560613, EPI_ISL_560614, EPI_ISL_560615, EPI_ISL_560616, EPI_ISL_560617, EPI_ISL_560618, EPI_ISL_560619, EPI_ISL_560620, EPI_ISL_560621, EPI_ISL_560622, EPI_ISL_560623, EPI_ISL_560624, EPI_ISL_560625, EPI_ISL_560626, EPI_ISL_560627, EPI_ISL_560628, EPI_ISL_560629, EPI_ISL_560630, EPI_ISL_560631, EPI_ISL_560632, EPI_ISL_560633, EPI_ISL_560634, EPI_ISL_560635, EPI_ISL_560636			
see above	Hospital	National Reference Center for Viruses of Respiratory Infections, Institut Pasteur, Paris	Sylvie Behillil, Fabiana Gambaro, Etienne Simon-Lorière, Vincent Enouf, Maud Vanpeene, Sylvie van der Werf
EPI_ISL_560637, EPI_ISL_560638, EPI_ISL_560639, EPI_ISL_560640, EPI_ISL_560641, EPI_ISL_560642	Labo Analyses Med	National Reference Center for Viruses of Respiratory Infections, Institut Pasteur, Paris	Sylvie Behillil, Fabiana Gambaro, Etienne Simon-Lorière, Vincent Enouf, Maud Vanpeene, Sylvie van der Werf

EPI_ISL_560643, EPI_ISL_560644, EPI_ISL_560645, EPI_ISL_560646	Hospital	National Reference Center for Viruses of Respiratory Infections, Institut Pasteur, Paris	Sylvie Behillil, Fabiana Gambaro, Etienne Simon-Lorière, Vincent Enouf, Maud Vanpeene, Sylvie van der Werf
EPI_ISL_560991	RSUD Dr. Soetomo	Institute of Tropical Disease, Universitas Airlangga	Rima R Prasetya, Krisnoadi Rahardjo, Aldise M Nastri, Jezzy R Dewantari, Joni Wahyuhadi, Gatot Soegiarto, Laksmi Wulandari, Retno A Setyoningrum, Resti Yudhawati, Yohko K Shimizu, Mitsuhiro Nishimura, Yasuko Mori, Soetjipto, Kazufumi Shimizu, Maria I Lusida
EPI_ISL_566077, EPI_ISL_566078, EPI_ISL_566079, EPI_ISL_566080, EPI_ISL_566081, EPI_ISL_566082, EPI_ISL_566083	Pathogenic Microorganisms Variability Laboratory	WHO National Influenza Centre Russian Federation	Andrey Komissarov, Artem Fadeev, Anna Ivanova, Kseniya Komissarova, Daria Danilenko, Dmitry Lioznov, Nadezhda Kuznetsova, Elena Shidlovskaya, Elizaveta Divisenko, Ekaterina Milashenko, Kirill Krasnoslobotsev, Evgeniya Mukasheva, Anna Ignatieva, Svetlana Trushakova, Alexey Shchetinin, Maria Nikiforova, Andrey Pochtovyy, Valeria Bacalin, Evgeny Usachev, Olga Burgasova, Ludmila Kolobukhina, Svetlana Smetanina, Elena Burtseva, Artem Tkachuk, Vladimir Gushchin, Alexander Gintsburg
EPI_ISL_568577	The National Institute of Public Health	State Veterinary Institute Prague	Nagy, A; Jirincova, H; Novakova, L; Trnka,D; Vecerova, J
EPI_ISL_568579	Virus Molecular Laboratory of the Microbiology and Virology Department	INMI Lazzaro Spallanzani IRCCS	Cesare E.M. Gruber, Martina Rueca, Barbara Bartolini, Francesco Messina, Silvia Meschi, Francesca Colavita, Concetta Castilletti, Elena Percivalle, Irene Cassaniti, Edoardo Vecchio Nepita, Fausto Baldanti, Maria R. Capobianchi, Antonino Di Caro
EPI_ISL_568686	RSUD Prof. DR. Margono Soekarjo	Eijkman Institute for Molecular Biology, Ministry of Research and Technology/National Agency for Research and Innovation	Frilasita A Yudhaputri, Edison Johar, Hidayat Trimarsanto, Iskandar A Adnan, Willy Agustine, David H Muljono, Safarina G Malik, Herawati Sudoyo, Khin Saw Myint, Amin Soebandrio
EPI_ISL_568687	RSUP Fatmawati	Eijkman Institute for Molecular Biology, Ministry of Research and Technology/National Agency for Research and Innovation	Frilasita A Yudhaputri, Edison Johar, Hidayat Trimarsanto, Iskandar A Adnan, Willy Agustine, David H Muljono, Safarina G Malik, Herawati Sudoyo, Khin Saw Myint, Amin Soebandrio
EPI_ISL_568688	RS Pelni	Eijkman Institute for Molecular Biology, Ministry of Research and Technology/National Agency for Research and Innovation	Frilasita A Yudhaputri, Edison Johar, Hidayat Trimarsanto, Iskandar A Adnan, Willy Agustine, David H Muljono, Safarina G Malik, Herawati Sudoyo, Khin Saw Myint, Amin Soebandrio
EPI_ISL_568689	RS Pondok Indah Puri Indah	Eijkman Institute for Molecular Biology, Ministry of Research and Technology/National Agency for Research and Innovation	Frilasita A Yudhaputri, Edison Johar, Hidayat Trimarsanto, Iskandar A Adnan, Willy Agustine, David H Muljono, Safarina G Malik, Herawati Sudoyo, Khin Saw Myint, Amin Soebandrio
EPI_ISL_568691	RSUP Fatmawati	Eijkman Institute for Molecular Biology, Ministry of Research and Technology/National Agency for Research and Innovation	Frilasita A Yudhaputri, Edison Johar, Hidayat Trimarsanto, Iskandar A Adnan, Willy Agustine, David H Muljono, Safarina G Malik, Herawati Sudoyo, Khin Saw Myint, Amin Soebandrio
EPI_ISL_568692	RS Kramat 128	Eijkman Institute for Molecular Biology, Ministry of Research and Technology/National Agency for Research and Innovation	Frilasita A Yudhaputri, Edison Johar, Hidayat Trimarsanto, Iskandar A Adnan, Willy Agustine, David H Muljono, Safarina G Malik, Herawati Sudoyo, Khin Saw Myint, Amin Soebandrio
EPI_ISL_568693	RS Freeport Tembagapura	Eijkman Institute for Molecular Biology, Ministry of Research and Technology/National Agency for Research and Innovation	Frilasita A Yudhaputri, Edison Johar, Hidayat Trimarsanto, Iskandar A Adnan, Willy Agustine, David H Muljono, Safarina G Malik, Herawati Sudoyo, Khin Saw Myint, Amin Soebandrio
EPI_ISL_568694	RSUP Prof. Dr. R. Kandou Manado	Eijkman Institute for Molecular Biology, Ministry of Research and Technology/National Agency for Research and Innovation	Frilasita A Yudhaputri, Edison Johar, Hidayat Trimarsanto, Iskandar A Adnan, Willy Agustine, David H Muljono, Safarina G Malik, Herawati Sudoyo, Khin Saw Myint, Amin Soebandrio
EPI_ISL_568873, EPI_ISL_568874, EPI_ISL_568875	Malaysia Genome Institute	Malaysia Genome Institute	Mohd Noor Mat Isa, Irni Suhayu Sapian, Yusuf Muhammad Noor, Nurhezreen Md Iqbal, Mohd Faizal Abu Bakar, Enizza Kasim, Shamsidar Sopie, Siti Noraini Othman, Azrin Ahmad, Nor Azfa Johari, Shahrul Hisham Zainal Ariffin
EPI_ISL_569865, EPI_ISL_569866, EPI_ISL_569867, EPI_ISL_569868, EPI_ISL_569869, EPI_ISL_569870, EPI_ISL_569871, EPI_ISL_569872, EPI_ISL_569873, EPI_ISL_569875, EPI_ISL_569876, EPI_ISL_569877, EPI_ISL_569878, EPI_ISL_569879, EPI_ISL_569880, EPI_ISL_569881, EPI_ISL_569882, EPI_ISL_569883, EPI_ISL_569884, EPI_ISL_569885, EPI_ISL_569886			
see above	Amedeo di savoia	Crosetto lab, Karolinska Institutet, SciLifeLab	Michele Simonetti, Maria Grazia Milia, Luuk Harbers, Ning Zhang, Anna Sapino, Valeria Ghisetti, Nicola Crosetto
EPI_ISL_572330, EPI_ISL_572331, EPI_ISL_572333	Institute for Virology, University Hospital Duesseldorf, Medical Faculty, Heinrich-Heine-University Duesseldorf	Institute for Virology, University Hospital Duesseldorf, Medical Faculty, Heinrich-Heine-University Duesseldorf	Maximilian Damagnez, Verena Keitel, Björn Jensen, Nadine Lübke, Lisa Müller, Philipp Ostermann, Tina Senff, Ortwin Adams, Philipp Albrecht, Gerald Antoch, Johannes Bode, Edwin Bölke, Saskia Elben, Torsten Feldt, Johannes C. Fischer, , Anselm Kunstein, Caroline Klindt, Alexander Killer, Tom Lüdde, Annemarie Mohring, Jennifer Neubert, Heiner Schaal, Ansgar Schulz, Jörg Timm, Andreas Walker
EPI_ISL_572338, EPI_ISL_572339, EPI_ISL_572340, EPI_ISL_572342, EPI_ISL_572345, EPI_ISL_572346, EPI_ISL_572347, EPI_ISL_572348, EPI_ISL_572351, EPI_ISL_572352, EPI_ISL_572354, EPI_ISL_572359, EPI_ISL_572368, EPI_ISL_572385, EPI_ISL_572386, EPI_ISL_572387, EPI_ISL_572388, EPI_ISL_572390, EPI_ISL_572391, EPI_ISL_572392, EPI_ISL_572394			
see above	LACEN/PE	WallauLab, Aggeu Magalhaes Institute	Marcelo Henrique Santos Paiva, Duschinka Ribeiro Duarte Guedes, Cássia Docena, Matheus Filgueira Bezerra, Filipe Zimmer Dezordi, Laís Ceschini Machado, Larissa Krokovsky, Elisama Helvecio, Alexandre Freitas da Silva, Luydson Richardson Silva Vasconcelos, Antonio Mauro Rezende, Severino Jefferson Ribeiro da Silva, Kamila Gaudêncio da Silva Sales, Bruna Santos Lima Figueiredo de Sá, Derciliano Lopes da Cruz, Claudio Eduardo Cavalcanti, Armando de Menezes Neto, Caroline Targino Alves da Silva, Renata Pessôa Germano Mendes, Maria Almerice Lopes da Silva, Tiago Gräf, Paola Cristina Resende, Gonzalo Bello0, Michelle da Silva Barros, Wheverton Ricardo Correia do Nascimento,, Rodrigo Moraes Loyo Arcoverde, Luciane Caroline Albuquerque Bezerra, Sinval Pinto Brandão Filho, Constância Flávia Junqueira Ayres, Gabriel Luz Wallau
EPI_ISL_572397	Institute for Virology, University Hospital Duesseldorf, Medical Faculty, Heinrich-Heine-University Duesseldorf	Institute for Virology, University Hospital Duesseldorf, Medical Faculty, Heinrich-Heine-University Duesseldorf	Maximilian Damagnez, Verena Keitel, Björn Jensen, Nadine Lübke, Lisa Müller, Philipp Ostermann, Tina Senff, Ortwin Adams, Philipp Albrecht, Gerald Antoch, Johannes Bode, Edwin Bölke, Saskia Elben, Torsten Feldt, Johannes C. Fischer, , Anselm Kunstein, Caroline Klindt, Alexander Killer, Tom Lüdde, Annemarie Mohring, Jennifer Neubert, Heiner Schaal, Ansgar Schulz, Jörg Timm, Andreas Walker
EPI_ISL_572398	Laboratory of Biology and Identification of Arboviruses	Pathogenic Microorganisms Variability Laboratory	Alexey Shchetinin, Maria Nikiforova, Andrei Siniavin, Victor Larichev, Alina Kozlova, Muhammad Saifullin, Alexey Prilipov, Vladimir Gushchin, Alexander Gintsburg
EPI_ISL_573152, EPI_ISL_574259	Institute for Virology, University Hospital Duesseldorf, Medical Faculty, Heinrich-Heine-University Duesseldorf	Institute for Virology, University Hospital Duesseldorf, Medical Faculty, Heinrich-Heine-University Duesseldorf	Maximilian Damagnez, Verena Keitel, Björn Jensen, Nadine Lübke, Lisa Müller, Philipp Ostermann, Tina Senff, Ortwin Adams, Philipp Albrecht, Gerald Antoch, Johannes Bode, Edwin Bölke, Saskia Elben, Torsten Feldt, Johannes C. Fischer, , Anselm Kunstein, Caroline Klindt, Alexander Killer, Tom Lüdde, Annemarie Mohring, Jennifer Neubert, Heiner Schaal, Ansgar Schulz, Jörg Timm, Andreas Walker
EPI_ISL_574292	Instituto Nacional de Investigación en Salud Pública	Instituto Nacional de Investigación en Salud Pública	Leandro Patino Patino, Domenica de Mora Coloma, Denisse Portugal, Alfredo Bruno Caicedo, Andrés Carrasco, Orson Mestanza, Manuel Gonzalez, Alberto Orlando Narvaez.
EPI_ISL_574293	Instituto Nacional de Investigación en Salud Pública	Instituto Nacional de Investigación en Salud Publica	Leandro Patino Patino, Doménica de Mora Coloma, Denisse Portugal, Alfredo Bruno Caicedo,Andrés Carrasco, Orson Mestanza, Manuel Gonzalez, Alberto Orlando Narvaez.
EPI_ISL_574294	Instituto Nacional de Investigacion en Salud Pública	Instituto Nacional de Investigación en Salud Pública	Leandro Patino Patino, Doménica de Mora Coloma, Denisse Portugal, Alfredo Bruno,Andrés Carrasco , Orson Mestanza , Manuel Gonzalez, Alberto Orlando Narvaez
EPI_ISL_574431	Hospital IESS Babahoyo	Institute of Microbiology, Universidad San Francisco de Quito	Belén Prado-Vivar, Sully Márquez, Juan José Guadalupe, Monica Becerra-Wong, Fernanda Zurita, Bernardo Gutiérrez, Francisco Cordova, Ninfa Henriquez, Killen Briones-Zamora, Killen Briones-Claudette, Verónica Barragán, Patricio Rojas-Silva, Gabriel Trueba, Michelle Grunauer, Paúl Cárdenas
EPI_ISL_574577	Hospital Municipal Dr. Ignacio Proença de Gouvea	Instituto Adolfo Lutz, Interdiciplinary Procedures Center, Strategic Laboratory	Claudio Tavares Sacchi, Claudia Regina Gonçalves, Erica Valessa Ramos Gomes, Karoline Rodrigues Campos
EPI_ISL_574578	Hospital Municipal Mário Gatti	Instituto Adolfo Lutz, Interdiciplinary Procedures Center, Strategic Laboratory	Claudio Tavares Sacchi, Claudia Regina Gonçalves, Erica Valessa Ramos Gomes, Karoline Rodrigues Campos
EPI_ISL_574579	Hospital Municipal Dr. Ignacio Proença de Gouvea	Instituto Adolfo Lutz, Interdiciplinary Procedures Center, Strategic Laboratory	Claudio Tavares Sacchi, Claudia Regina Gonçalves, Erica Valessa Ramos Gomes, Karoline Rodrigues Campos
EPI_ISL_574580	Hospital Cidade Tiradentes Carmen Prudente	Instituto Adolfo Lutz, Interdiciplinary Procedures Center, Strategic Laboratory	Claudio Tavares Sacchi, Claudia Regina Gonçalves, Erica Valessa Ramos Gomes, Karoline Rodrigues Campos
EPI_ISL_574582	Hospital Municipal Dr. Jose Soares Hungria	Instituto Adolfo Lutz, Interdiciplinary Procedures Center, Strategic Laboratory	Claudio Tavares Sacchi, Claudia Regina Gonçalves, Erica Valessa Ramos Gomes, Karoline Rodrigues Campos
EPI_ISL_574583	Secretaria Municipal de Saude de Jandira	Instituto Adolfo Lutz, Interdiciplinary Procedures Center, Strategic Laboratory	Claudio Tavares Sacchi, Claudia Regina Gonçalves, Erica Valessa Ramos Gomes, Karoline Rodrigues Campos
EPI_ISL_574588	Hospital Estadual Sumare	Instituto Adolfo Lutz, Interdiciplinary Procedures Center,	Claudio Tavares Sacchi, Claudia Regina Gonçalves, Erica Valessa Ramos Gomes, Karoline Rodrigues Campos

		Strategic Laboratory	
EPI_ISL_574589	Hospital Municipal Dr. Jose Soares Hungria	Instituto Adolfo Lutz, Interdiciplinary Procedures Center, Strategic Laboratory	Claudio Tavares Sacchi, Claudia Regina Gonçalves, Erica Valessa Ramos Gomes, Karoline Rodrigues Campos
EPI_ISL_574590	Unidade de Printo Atendimento UPA I Santa Isabel	Instituto Adolfo Lutz, Interdiciplinary Procedures Center, Strategic Laboratory	Claudio Tavares Sacchi, Claudia Regina Gonçalves, Erica Valessa Ramos Gomes, Karoline Rodrigues Campos
EPI_ISL_574591, EPI_ISL_574592	Hospital Domingos Leonardo Ceravolo Presidente Prudente	Instituto Adolfo Lutz, Interdiciplinary Procedures Center, Strategic Laboratory	Claudio Tavares Sacchi, Claudia Regina Gonçalves, Erica Valessa Ramos Gomes, Karoline Rodrigues Campos
EPI_ISL_574593	CS II Dr. Antonio Vicoso Moreira de Rezende Sumare	Instituto Adolfo Lutz, Interdiciplinary Procedures Center, Strategic Laboratory	Claudio Tavares Sacchi, Claudia Regina Gonçalves, Erica Valessa Ramos Gomes, Karoline Rodrigues Campos
EPI_ISL_574594	Hospital Escola da Universidade de Taubate	Instituto Adolfo Lutz, Interdiciplinary Procedures Center, Strategic Laboratory	Claudio Tavares Sacchi, Claudia Regina Gonçalves, Erica Valessa Ramos Gomes, Karoline Rodrigues Campos
EPI_ISL_574595	Hospital Geral de Vila Penteado Dr. Jose Pamgella	Instituto Adolfo Lutz, Interdiciplinary Procedures Center, Strategic Laboratory	Claudio Tavares Sacchi, Claudia Regina Gonçalves, Erica Valessa Ramos Gomes, Karoline Rodrigues Campos
EPI_ISL_574596	CS II Dr. Antonio Vicoso Moreira de Rezende Sumare	Instituto Adolfo Lutz, Interdiciplinary Procedures Center, Strategic Laboratory	Claudio Tavares Sacchi, Claudia Regina Gonçalves, Erica Valessa Ramos Gomes, Karoline Rodrigues Campos
EPI_ISL_574597	Secretaria Municipal de Saude de Jarinu	Instituto Adolfo Lutz, Interdiciplinary Procedures Center, Strategic Laboratory	Claudio Tavares Sacchi, Claudia Regina Gonçalves, Erica Valessa Ramos Gomes, Karoline Rodrigues Campos
EPI_ISL_574598	Servico de Verificacao de Obito SVO	Instituto Adolfo Lutz, Interdiciplinary Procedures Center, Strategic Laboratory	Claudio Tavares Sacchi, Claudia Regina Gonçalves, Erica Valessa Ramos Gomes, Karoline Rodrigues Campos
EPI_ISL_574603	RS Freeport Tembagapura	Eijkman Institute for Molecular Biology, Ministry of Research and Technology/National Agency for Research and Innovation	Frilasita A Yudhaputri, Edison Johar, Hidayat Trimarsanto, Iskandar A Adnan, Willy Agustine, David H Muljono, Safarina G Malik, Herawati Sudoyo, Khin Saw Myint, Amin Soebandrio
EPI_ISL_574604	RSUP Prof. Dr. R. Kandou Manado	Eijkman Institute for Molecular Biology, Ministry of Research and Technology/National Agency for Research and Innovation	Frilasita A Yudhaputri, Edison Johar, Hidayat Trimarsanto, Iskandar A Adnan, Willy Agustine, David H Muljono, Safarina G Malik, Herawati Sudoyo, Khin Saw Myint, Amin Soebandrio
EPI_ISL_574605	RSUP dr. Sardjito	Eijkman Institute for Molecular Biology, Ministry of Research and Technology/National Agency for Research and Innovation	Frilasita A Yudhaputri, Edison Johar, Hidayat Trimarsanto, Iskandar A Adnan, Willy Agustine, David H Muljono, Safarina G Malik, Herawati Sudoyo, Khin Saw Myint, Amin Soebandrio
EPI_ISL_574606	RS Siloam Purwakarta	Eijkman Institute for Molecular Biology, Ministry of Research and Technology/National Agency for Research and Innovation	Frilasita A Yudhaputri, Edison Johar, Hidayat Trimarsanto, Iskandar A Adnan, Willy Agustine, David H Muljono, Safarina G Malik, Herawati Sudoyo, Khin Saw Myint, Amin Soebandrio
EPI_ISL_574607	RS Husada	Eijkman Institute for Molecular Biology, Ministry of Research and Technology/National Agency for Research and Innovation	Frilasita A Yudhaputri, Edison Johar, Hidayat Trimarsanto, Iskandar A Adnan, Willy Agustine, David H Muljono, Safarina G Malik, Herawati Sudoyo, Khin Saw Myint, Amin Soebandrio
EPI_ISL_574608	RS Mitra Keluarga Kelapa Gading	Eijkman Institute for Molecular Biology, Ministry of Research and Technology/National Agency for Research and Innovation	Frilasita A Yudhaputri, Edison Johar, Hidayat Trimarsanto, Iskandar A Adnan, Willy Agustine, David H Muljono, Safarina G Malik, Herawati Sudoyo, Khin Saw Myint, Amin Soebandrio
EPI_ISL_574609	RSJPD Harapan Kita	Eijkman Institute for Molecular Biology, Ministry of Research and Technology/National Agency for Research and Innovation	Frilasita A Yudhaputri, Edison Johar, Hidayat Trimarsanto, Iskandar A Adnan, Willy Agustine, David H Muljono, Safarina G Malik, Herawati Sudoyo, Khin Saw Myint, Amin Soebandrio
EPI_ISL_574610	Puskesmas Tambora	Eijkman Institute for Molecular Biology, Ministry of Research and Technology/National Agency for Research and Innovation	Frilasita A Yudhaputri, Edison Johar, Hidayat Trimarsanto, Iskandar A Adnan, Willy Agustine, David H Muljono, Safarina G Malik, Herawati Sudoyo, Khin Saw Myint, Amin Soebandrio
EPI_ISL_574611	RS Premier Bintaro	Eijkman Institute for Molecular Biology, Ministry of Research and Technology/National Agency for Research and Innovation	Frilasita A Yudhaputri, Edison Johar, Hidayat Trimarsanto, Iskandar A Adnan, Willy Agustine, David H Muljono, Safarina G Malik, Herawati Sudoyo, Khin Saw Myint, Amin Soebandrio
EPI_ISL_574612	RS Hermina Mekarsari	Eijkman Institute for Molecular Biology, Ministry of Research and Technology/National Agency for Research and Innovation	Frilasita A Yudhaputri, Edison Johar, Hidayat Trimarsanto, Iskandar A Adnan, Willy Agustine, David H Muljono, Safarina G Malik, Herawati Sudoyo, Khin Saw Myint, Amin Soebandrio
EPI_ISL_574614	RS Kramat 128	Eijkman Institute for Molecular Biology, Ministry of Research and Technology/National Agency for Research and Innovation	Frilasita A Yudhaputri, Edison Johar, Hidayat Trimarsanto, Iskandar A Adnan, Willy Agustine, David H Muljono, Safarina G Malik, Herawati Sudoyo, Khin Saw Myint, Amin Soebandrio
EPI_ISL_574615	RS Harapan Bunda	Eijkman Institute for Molecular Biology, Ministry of Research and Technology/National Agency for Research and Innovation	Frilasita A Yudhaputri, Edison Johar, Hidayat Trimarsanto, Iskandar A Adnan, Willy Agustine, David H Muljono, Safarina G Malik, Herawati Sudoyo, Khin Saw Myint, Amin Soebandrio
EPI_ISL_574616, EPI_ISL_574617	RS Kramat 128	Eijkman Institute for Molecular Biology, Ministry of Research and Technology/National Agency for Research and Innovation	Frilasita A Yudhaputri, Edison Johar, Hidayat Trimarsanto, Iskandar A Adnan, Willy Agustine, David H Muljono, Safarina G Malik, Herawati Sudoyo, Khin Saw Myint, Amin Soebandrio
EPI_ISL_574618	RS Kramat 128	Eijkman Institute for Molecular Biology, Ministry of Research and Technology/National Agency for Research and Innovation	Hidayat Trimarsanto, Frilasita A Yudhaputri, Edison Johar, Iskandar A Adnan, Willy Agustine, David H Muljono, Safarina G Malik, Herawati Sudoyo, Khin Saw Myint, Amin Soebandrio
EPI_ISL_574619, EPI_ISL_574620, EPI_ISL_574621, EPI_ISL_574622, EPI_ISL_574623	BTKLPP Kelas I Manado	Eijkman Institute for Molecular Biology, Ministry of Research and Technology/National Agency for Research and Innovation	Hidayat Trimarsanto, Frilasita A Yudhaputri, Edison Johar, Iskandar A Adnan, Willy Agustine, David H Muljono, Safarina G Malik, Herawati Sudoyo, Khin Saw Myint, Amin Soebandrio
EPI_ISL_575329, EPI_ISL_575330	National Institute for Viral Disease Control and Prevention, China CDC	National Institute for Viral Disease Control and Prevention, China CDC	Rongbao Gao, Kang Xiao, Qinqin Song, ZhiQiang Xia, Dong Xia, Juan Song, Haijun Du, Yuan He, Shuai Pang, Xuancheng Lu, Guizhen Wu, Geogia Fu Gao, Jun Han
EPI_ISL_575331	RSUD Wates	Genetics Working Group (Pokja Genetik) Faculty of Medicine, Public Health and Nursing Universitas Gadjah Mada (FK-KMK UGM); Disease Investigation Center Wates Ministry of Agriculture Indonesia; Department of Microbiology FK-KMK UGM; Laboratorium Diagnostik Yayasan Tahija World Mosquito Program (WMP) Yogyakarta Center for Tropical Medicine FK-KMK UGM; Integrated Research Center FK-KMK UGM; Department of Computer Science and Electronics FMIPA UGM	Gunadi, Hendra Wibawa, . Marcellus, Mohamad S. Hakim, Edwin W. Daniwijaya, Ludhang P. Rizki, Endah Supriyati, Eggi Arguni, Titik Nuryastuti, Tri Wibawa, Dwi AA Nugrahaningsih, Afiahayati, Siswanto, Kristy Iskandar, Nungki Anggorowati, Bastianto Kusumajaya, Zumrati Ahmad, Alvin S. Kalim, Susan Simanjaya
EPI_ISL_576113	RSUP Dr. Sardjito	Genetics Working Group (Pokja Genetik) Faculty of Medicine, Public Health and Nursing Universitas Gadjah Mada (FK-KMK UGM); Disease Investigation Center Wates Ministry of Agriculture Indonesia; Department of Microbiology FK-KMK UGM; Laboratorium Diagnostik Yayasan Tahija World Mosquito Program (WMP) Yogyakarta Center for Tropical Medicine FK-KMK UGM; Integrated Research Center FK-KMK UGM; Department of Computer Science and Electronics FMIPA UGM	Gunadi, Hendra Wibawa, . Marcellus, Mohamad S. Hakim, Edwin W. Daniwijaya, Ludhang P. Rizki, Endah Supriyati, Eggi Arguni, Titik Nuryastuti, Tri Wibawa, Dwi AA Nugrahaningsih, Afiahayati, Siswanto, Kristy Iskandar, Nungki Anggorowati, Ika Trisnawati, Riat El Khair, William Widitjiarso, Fadil Fahri
EPI_ISL_576114	RSUP Dr. Sardjito	Genetics Working Group (Pokja Genetik) Faculty of Medicine, Public Health and Nursing Universitas Gadjah Mada (FK-KMK UGM); Disease Investigation Center Wates Ministry of	Gunadi, Hendra Wibawa, . Marcellus, Mohamad S. Hakim, Edwin W. Daniwijaya, Ludhang P. Rizki, Endah Supriyati, Eggi Arguni, Titik Nuryastuti, Tri Wibawa, Dwi AA Nugrahaningsih, Afiahayati, Siswanto, Kristy Iskandar, Nungki Anggorowati, Ika Trisnawati, Riat El Khair, Audric Kenny Tedja, Aditya Rifqi Fauzi

		Agriculture Indonesia; Department of Microbiology FK-KMK UGM; Laboratorium Diagnostik Yayasan Tahija World Mosquito Program (WMP) Yogyakarta Center for Tropical Medicine FK-KMK UGM; Integrated Research Center FK-KMK UGM; Department of Computer Science and Electronics FMIPA UGM	
EPI_ISL_576115	RSUP Dr Sardjito	Genetics Working Group (Pokja Genetik) Faculty of Medicine, Public Health and Nursing Universitas Gadjah Mada (FK-KMK UGM); Disease Investigation Center Wates Ministry of Agriculture Indonesia; Department of Microbiology FK-KMK UGM; Laboratorium Diagnostik Yayasan Tahija World Mosquito Program (WMP) Yogyakarta Center for Tropical Medicine FK-KMK UGM; Integrated Research Center FK-KMK UGM; Department of Computer Science and Electronics FMIPA UGM	Gunadi, Hendra Wibawa, . Marcellus, Mohamad S. Hakim, Edwin W. Daniwijaya, Ludhang P. Rizki, Endah Supriyati, Eggi Arguni, Titik Nuryastuti, Tri Wibawa, Dwi AA Nugrahaningsih, Afiahayati, Siswanto, Kristy Iskandar, Nungki Anggorowati, Ika Trisnawati, Riat El Khair, Yunika Puspadewi, Osman Sianipar, Umi Solekhah Intansari, Elizabeth Henny Herningtiyas, Ira Puspitawati, Nur Imma Fatimah Harahap, Untung Wirawan, Maria Patricia Inggriani
EPI_ISL_576116	RSUP Dr. Sardjito	Genetics Working Group (Pokja Genetik) Faculty of Medicine, Public Health and Nursing Universitas Gadjah Mada (FK-KMK UGM); Disease Investigation Center Wates Ministry of Agriculture Indonesia; Department of Microbiology FK-KMK UGM; Laboratorium Diagnostik Yayasan Tahija World Mosquito Program (WMP) Yogyakarta Center for Tropical Medicine FK-KMK UGM; Integrated Research Center FK-KMK UGM; Department of Computer Science and Electronics FMIPA UGM	Gunadi, Hendra Wibawa, . Marcellus, Mohamad S. Hakim, Edwin W. Daniwijaya, Ludhang P. Rizki, Endah Supriyati, Eggi Arguni, Titik Nuryastuti, Tri Wibawa, Dwi AA Nugrahaningsih, Afiahayati, Siswanto, Kristy Iskandar, Nungki Anggorowati, Ika Trisnawati, Sumardi, Eko Budiono, Bambang Sigit Riyanto, Heni Retnowulan, Munawar Gani, Satria Maulana, Nur Rahmi Ananda, Riat El Khair, Yunika Puspadewi, Osman Sianipar, Umi Solekhah Intansari, Elizabeth Henny Herningtiyas, Ira Puspitawati, Nur Imma Fatimah Harahap, Ririn Enggy Yuliyanti, Alvin Santoso Kalim
EPI_ISL_576128	RSUP Dr. Sardjito	Genetics Working Group (Pokja Genetik) Faculty of Medicine, Public Health and Nursing Universitas Gadjah Mada (FK-KMK UGM); Disease Investigation Center Wates Ministry of Agriculture Indonesia; Department of Microbiology FK-KMK UGM; Laboratorium Diagnostik Yayasan Tahija World Mosquito Program (WMP) Yogyakarta Center for Tropical Medicine FK-KMK UGM; Integrated Research Center FK-KMK UGM; Department of Computer Science and Electronics FMIPA UGM	Gunadi, Hendra Wibawa, Marcellus, Mohamad S. Hakim, Edwin W. Daniwijaya, Ludhang P. Rizki, Endah Supriyati, Eggi Arguni, Titik Nuryastuti, Tri Wibawa, Dwi AA Nugrahaningsih, Afiahayati, Siswanto, Kristy Iskandar, Nungki Anggorowati, Ika Trisnawati, Sumardi, Eko Budiono, Bambang Sigit Riyanto, Heni Retnowulan, Munawar Gani, Satria Maulana, Nur Rahmi Ananda, Riat El Khair, Yunika Puspadewi, Osman Sianipar, Umi Solekhah Intansari, Elizabeth Henny Herningtiyas, Ira Puspitawati, Nur Imma Fatimah Harahap, Dyah Ayu Puspitarani, Kemala Athollah
EPI_ISL_576130	RSUP Dr. Sardjito	Genetics Working Group (Pokja Genetik) Faculty of Medicine, Public Health and Nursing Universitas Gadjah Mada (FK-KMK UGM); Disease Investigation Center Wates Ministry of Agriculture Indonesia; Department of Microbiology FK-KMK UGM; Laboratorium Diagnostik Yayasan Tahija World Mosquito Program (WMP) Yogyakarta Center for Tropical Medicine FK-KMK UGM; Integrated Research Center FK-KMK UGM; Department of Computer Science and Electronics FMIPA UGM	Gunadi, Hendra Wibawa, Marcellus, Mohamad S. Hakim, Edwin W. Daniwijaya, Ludhang P. Rizki, Endah Supriyati, Eggi Arguni, Titik Nuryastuti, Tri Wibawa, Dwi AA Nugrahaningsih, Afiahayati, Siswanto, Kristy Iskandar, Nungki Anggorowati, Rina Triasih, Amalia Setyati, Dwikisworo Setyowireni, Ida Safitri Laksanawati, Cahya Dewi Satria, Titis Widowati, Elisabeth Siti Herini, Riat El Khair, Yunika Puspadewi, Osman Sianipar, Umi Solekhah Intansari, Elizabeth Henny Herningtiyas, Ira Puspitawati, Nur Imma Fatimah Harahap, Alvin S. Kalim, Susan Simanjaya, Desyifa Mursalin
EPI_ISL_576145	RSA Universitas Gadjah Mada	Genetics Working Group (Pokja Genetik) Faculty of Medicine, Public Health and Nursing Universitas Gadjah Mada (FK-KMK UGM); Disease Investigation Center Wates Ministry of Agriculture Indonesia; Department of Microbiology FK-KMK UGM; Laboratorium Diagnostik Yayasan Tahija World Mosquito Program (WMP) Yogyakarta Center for Tropical Medicine FK-KMK UGM; Integrated Research Center FK-KMK UGM; Department of Computer Science and Electronics FMIPA UGM	Gunadi, Hendra Wibawa, Marcellus, Mohamad S. Hakim, Edwin W. Daniwijaya, Ludhang P. Rizki, Endah Supriyati, Eggi Arguni, Titik Nuryastuti, Tri Wibawa, Dwi AA Nugrahaningsih, Afiahayati, Siswanto, Kristy Iskandar, Nungki Anggorowati, William Widitjiarso, Fadil Fahri
EPI_ISL_576146, EPI_ISL_576147, EPI_ISL_576148	Department of Respiratory & Other Viral Infections of L.V. Gromashevsky Institute of Epidemiology & Infectious Diseases NAMS of Ukraine	Department of Respiratory & Other Viral Infections of L.V. Gromashevsky Institute of Epidemiology & Infectious Diseases NAMS of Ukraine, JSC "Farmak"	Alla Mironenko, Ihor Kravchuk, Liudmyla Bolotova, Larysa Radchenko, Nataliia Teteriuk
EPI_ISL_576149	Department of Respiratory & Other Viral Infections of L.V. Gromashevsky Institute of Epidemiology & Infectious Diseases NAMS of Ukraine	Department of Respiratory & Other Viral Infections of L.V. Gromashevsky Institute of Epidemiology & Infectious Diseases NAMS of Ukraine, JSC "Farmak"	Alla Mironenko, Andriy Goy, Ihor Kravchuk, Liudmyla Bolotova, Larysa Radchenko, Nataliia Teteriuk
EPI_ISL_576258, EPI_ISL_576259, EPI_ISL_576260	Instituto de Diagnostico y Referencia Epidemiologicos (INDRE)	Instituto de Diagnostico y Referencia Epidemiologicos (INDRE)	Gisela Barrera-Badillo , Abril Rodriguez-Maldonado, Claudia Wong-Arambula , Natividad Cruz-Ortiz, Tatiana Nunez-Garcia, Dayanira Arellano-Suarez, Fabiola Garces-Ayala, Edgar Mendieta-Condado, Lucia Hernandez-Rivas, Irma Lopez-Martinez, Ernesto Ramirez-Gonzalez.
EPI_ISL_576261, EPI_ISL_576262, EPI_ISL_576263	Instituto de Diagnostico y Referencia Epidemiologicos (INDRE)	Instituto de Diagnostico y Referencia Epidemiologicos (INDRE)	Gisela Barrera-Badillo , Abril Rodriguez-Maldonado, Claudia Wong-Arambula , Natividad Cruz-Ortiz, Tatiana Nunez-Garcia, Dayanira Arellano-Suarez, Adnan Araiza-Rodriguez, Edgar Mendieta-Condado, Lucia Hernandez-Rivas, Irma Lopez-Martinez, Ernesto Ramirez-Gonzalez.
EPI_ISL_576264, EPI_ISL_576265	Instituto de Diagnostico y Referencia Epidemiologicos (INDRE)	Instituto de Diagnostico y Referencia Epidemiologicos (INDRE)	Ernesto Ramirez-Gonzalez, Abril Rodriguez-Maldonado, Claudia Wong-Arambula , Natividad Cruz-Ortiz, Tatiana Nunez-Garcia, Dayanira Arellano-Suarez, Adnan Araiza-Rodriguez, Edgar Mendieta-Condado, Lucia Hernandez-Rivas, Irma Lopez-Martinez, Gisela Barrera-Badillo.
EPI_ISL_576266, EPI_ISL_576267, EPI_ISL_576268, EPI_ISL_576269, EPI_ISL_576270, EPI_ISL_576271	Instituto de Diagnostico y Referencia Epidemiologicos (INDRE)	Instituto de Diagnostico y Referencia Epidemiologicos (INDRE)	Ernesto Ramirez-Gonzalez, Abril Rodriguez-Maldonado, Claudia Wong-Arambula , Natividad Cruz-Ortiz, Tatiana Nunez-Garcia, Dayanira Arellano-Suarez, Adnan Araiza-Rodriguez, Fabiola Garces-Ayala, Lucia Hernandez-Rivas, Irma Lopez-Martinez, Gisela Barrera-Badillo.
EPI_ISL_576272	Instituto de Diagnostico y Referencia Epidemiologicos (INDRE)	Instituto de Diagnostico y Referencia Epidemiologicos (INDRE)	Gisela Barrera-Badillo , Abril Rodriguez-Maldonado, Claudia Wong-Arambula , Natividad Cruz-Ortiz, Tatiana Nunez-Garcia, Dayanira Arellano-Suarez, Fabiola Garces-Ayala, Edgar Mendieta-Condado, Lucia Hernandez-Rivas, Irma Lopez-Martinez, Ernesto Ramirez-Gonzalez.
EPI_ISL_576273	Instituto de Diagnostico y Referencia Epidemiologicos (INDRE)	Instituto de Diagnostico y Referencia Epidemiologicos (INDRE)	Ernesto Ramirez-Gonzalez, Abril Rodriguez-Maldonado, Claudia Wong-Arambula , Natividad Cruz-Ortiz, Tatiana Nunez-Garcia, Dayanira Arellano-Suarez, Adnan Araiza-Rodriguez, Fabiola Garces-Ayala, Lucia Hernandez-Rivas, Irma Lopez-Martinez, Gisela Barrera-Badillo.
EPI_ISL_576274, EPI_ISL_576275, EPI_ISL_576276	Instituto de Diagnostico y Referencia Epidemiologicos (INDRE)	Instituto de Diagnostico y Referencia Epidemiologicos (INDRE)	Gisela Barrera-Badillo , Abril Rodriguez-Maldonado, Claudia Wong-Arambula , Natividad Cruz-Ortiz, Tatiana Nunez-Garcia, Dayanira Arellano-Suarez, Adnan Araiza-Rodriguez, Edgar Mendieta-Condado, Lucia Hernandez-Rivas, Irma Lopez-Martinez, Ernesto Ramirez-Gonzalez.
EPI_ISL_576277, EPI_ISL_576278	Instituto de Diagnostico y Referencia Epidemiologicos (INDRE)	Instituto de Diagnostico y Referencia Epidemiologicos (INDRE)	Ernesto Ramirez-Gonzalez, Abril Rodriguez-Maldonado, Claudia Wong-Arambula , Natividad Cruz-Ortiz, Tatiana Nunez-Garcia, Dayanira Arellano-Suarez, Adnan Araiza-Rodriguez, Edgar Mendieta-Condado, Lucia Hernandez-Rivas, Irma Lopez-Martinez, Gisela Barrera-Badillo.
EPI_ISL_576279	Instituto de Diagnostico y Referencia Epidemiologicos (INDRE)	Instituto de Diagnostico y Referencia Epidemiologicos (INDRE)	Ernesto Ramirez-Gonzalez, Abril Rodriguez-Maldonado, Claudia Wong-Arambula , Natividad Cruz-Ortiz, Tatiana Nunez-Garcia, Dayanira Arellano-Suarez, Adnan Araiza-Rodriguez, Fabiola Garces-Ayala, Lucia Hernandez-Rivas, Irma Lopez-Martinez, Gisela Barrera-Badillo.
EPI_ISL_576383	RSUD Budi Rahayu Kota Magelang	Genetics Working Group (Pokja Genetik) Faculty of Medicine, Public Health and Nursing Universitas Gadjah Mada (FK-KMK UGM); Disease Investigation Center Wates Ministry of	Gunadi, Hendra Wibawa, Marcellus, Mohamad S. Hakim, Edwin W. Daniwijaya, Ludhang P. Rizki, Endah Supriyati, Eggi Arguni, Titik Nuryastuti, Tri Wibawa, Dwi AA Nugrahaningsih, Afiahayati, Siswanto, Kristy Iskandar, Nungki Anggorowati, Irene, Indaryati, Havid Setyawan, Ari Meliyanti, Merliana Sari Situmeang, Audric Kenny Tedja, Aditya Rifqi Fauzi

		Agriculture Indonesia; Department of Microbiology FK-KMK UGM; Laboratorium Diagnostik Yayasan Tahija World Mosquito Program (WMP) Yogyakarta Center for Tropical Medicine FK-KMK UGM; Integrated Research Center FK-KMK UGM; Department of Computer Science and Electronics FMIPA UGM; Balai Besar Teknik Kesehatan Lingkungan dan Pengendalian Penyakit (BBTKLPP) Yogyakarta	
EPI_ISL_577624, EPI_ISL_577625, EPI_ISL_577626, EPI_ISL_577627, EPI_ISL_577628, EPI_ISL_577629, EPI_ISL_577630, EPI_ISL_577631, EPI_ISL_577632, EPI_ISL_577633, EPI_ISL_577634, EPI_ISL_577635, EPI_ISL_577636, EPI_ISL_577637, EPI_ISL_577638			
see above	The National Institute of Public Health	State Veterinary Institute Prague	Nagy,A;Jirincova,H;Novakova,L;Trnka,D;Vecerova,J
EPI_ISL_578186	Hospital General Juan Ramón Jiménez	Instituto de Salud Carlos III	Iglesias-Caballero, M. Molinero Calamita, M. González-Esguevillas, M. Camarero, S. Pozo, F. Casas, I. Jiménez, P. Jiménez, M. Zaballos, A. Monzón, S. Varona, S. Juliá, M. Cuesta, I, J. Saavedra
EPI_ISL_578187, EPI_ISL_578188	Hospital Virgen de las Nieves	Instituto de Salud Carlos III	Iglesias-Caballero, M. Molinero Calamita, M. González-Esguevillas, M. Camarero, S. Pozo, F. Casas, I. Jiménez, P. Jiménez, M. Zaballos, A. Monzón, S. Varona, S. Juliá, M. Cuesta, I, J.M Navarro
EPI_ISL_578189	Hospital Universitario Miguel Servet	Instituto de Salud Carlos III	Iglesias-Caballero, M. Molinero Calamita, M. González-Esguevillas, M. Camarero, S. Pozo, F. Casas, I. Jiménez, P. Jiménez, M. Zaballos, A. Monzón, S. Varona, S. Juliá, M. Cuesta, I, A. Rezusta
EPI_ISL_578190, EPI_ISL_578191	Hospital Clínico Universitario Lozano Blesa	Instituto de Salud Carlos III	Iglesias-Caballero, M. Molinero Calamita, M. González-Esguevillas, M. Camarero, S. Pozo, F. Casas, I. Jiménez, P. Jiménez, M. Zaballos, A. Monzón, S. Varona, S. Juliá, M. Cuesta, I, R. Benito
EPI_ISL_578192, EPI_ISL_578193	Hospital Universitario Miguel Servet	Instituto de Salud Carlos III	Iglesias-Caballero, M. Molinero Calamita, M. González-Esguevillas, M. Camarero, S. Pozo, F. Casas, I. Jiménez, P. Jiménez, M. Zaballos, A. Monzón, S. Varona, S. Juliá, M. Cuesta, I, A. Rezusta
EPI_ISL_578194	Complejo Hospitalario Universitario La Coruna	Instituto de Salud Carlos III	Iglesias-Caballero, M. Molinero Calamita, M. González-Esguevillas, M. Camarero, S. Pozo, F. Casas, I. Jiménez, P. Jiménez, M. Zaballos, A. Monzón, S. Varona, S. Juliá, M. Cuesta, I, J. López
EPI_ISL_578195	Complejo Hospitalario de Orense	Instituto de Salud Carlos III	Iglesias-Caballero, M. Molinero Calamita, M. González-Esguevillas, M. Camarero, S. Pozo, F. Casas, I. Jiménez, P. Jiménez, M. Zaballos, A. Monzón, S. Varona, S. Juliá, M. Cuesta, I, M. García
EPI_ISL_578196	Hospital San Pedro	Instituto de Salud Carlos III	Iglesias-Caballero, M. Molinero Calamita, M. González-Esguevillas, M. Camarero, S. Pozo, F. Casas, I. Jiménez, P. Jiménez, M. Zaballos, A. Monzón, S. Varona, S. Juliá, M. Cuesta, I, J.M Azcona
EPI_ISL_578197	Hospital San Pedro	Instituto de Salud Carlos III	Iglesias-Caballero, M. Molinero Calamita, M. González-Esguevillas, M. Camarero, S. Pozo, F. Casas, I. Jiménez, P. Jiménez, M. Zaballos, A. Monzón, S. Varona, S. Juliá, M. Cuesta, I, C. Alonso
EPI_ISL_578198, EPI_ISL_578199	Hospital San Pedro	Instituto de Salud Carlos III	Iglesias-Caballero, M. Molinero Calamita, M. González-Esguevillas, M. Camarero, S. Pozo, F. Casas, I. Jiménez, P. Jiménez, M. Zaballos, A. Monzón, S. Varona, S. Juliá, M. Cuesta, I, M. Blasco
EPI_ISL_578200, EPI_ISL_578201	Hospital San Pedro	Instituto de Salud Carlos III	Iglesias-Caballero, M. Molinero Calamita, M. González-Esguevillas, M. Camarero, S. Pozo, F. Casas, I. Jiménez, P. Jiménez, M. Zaballos, A. Monzón, S. Varona, S. Juliá, M. Cuesta, I, C. Alonso
EPI_ISL_578697	Clinical Virology Laboratory, Institute of Liver and Biliary Sciences	ILBS - IGIB	Ekta Gupta, Sheetalnath Rooge, Abhishek Padhi, Reshu Agarwal, Jaswinder Singh Maras, Shridhar Sivasubbu, Vinod Scaria, Shvetank Sharma
EPI_ISL_582029, EPI_ISL_582030	Biology Department, College of Science, Al-Muthanna University	International Centre for Genetic Engineering and Biotechnology (ICGEB) and ARGO Open Lab Platform	Nihad Al-Rashedi, Danilo Licastro, Sreejith Rajasekharan, Simeone Dal Monego, Alessandro Marcello
EPI_ISL_582033	Department of Pathology, School of Medicine, Imam Khomeini Hospital, Tehran University of Medical Sciences	Genetics Research Center. University Of Social Welfare And Rehabilitation Sciences	Zohreh Fattahi, Marzieh Mohseni, Khadijeh Jalalvand, Azam Ghaziasadi, Seyedeh elham Mortazavi, Ali Jafarpour, Azar Hadadi, Alireza Abdollahi, Ali Jafarpour, Azam Ghaziasad, Seyedeh elham Mortazavi, Saber Soltani, Reza Najafipour , Kimia Kahrizi, Seyed Mohammad Jazayeri, Hossein Najmabadi
EPI_ISL_582124	Malaysia Genome Institute	Malaysia Genome Institute	Mohd Noor Mat Isa, Irni Suhayu Sapian, Yusuf Muhammad Noor, Nurhezreen Md Iqbal, Mohd Faizal Abu Bakar, Enizza Kasim, Shamsidar Sopie, Siti Noraini Othman, Azrin Ahmad, Nor Azfa Johari, Shahrul Hisham Zainal Ariffin
EPI_ISL_582125, EPI_ISL_582126	Sheikh Khalifa Medical City	Molecular Surveillance lab Sheikh Khalifa Medical City	Amirtharaj Francis, Sajeed Abdul, Hala Imambaccus, Sahar Almarzooqi, Hiba Saud, Stefan Weber
EPI_ISL_582127, EPI_ISL_582128, EPI_ISL_582129, EPI_ISL_582130, EPI_ISL_582131, EPI_ISL_582132	Antwerp University Hospital	Institute of Tropical Medicine	Philippe Selhorst, Colin Anthony
EPI_ISL_582509	Department of Respiratory and other Viral Infections of L.V.Gromashevsky Institute of Epidemiology & Infectious Diseases NAMS of Ukraine	Department of Respiratory and other Viral Infections of L.V.Gromashevsky Institute of Epidemiology & Infectious Diseases NAMS of Ukraine, JSC "Farmak"	Alla Mironenko, Andriy Goy, Ihor Kravchuk, Ludmyla Bolotova, Larysa Radchenko, Nataliia Teteriuk
EPI_ISL_582510	Department of Respiratory and other Viral Infections of L.V.Gromashevsky Institute of Epidemiology & Infectious Diseases NAMS of Ukraine	Department of Respiratory and other Viral Infections of L.V.Gromashevsky Institute of Epidemiology & Infectious Diseases NAMS of Ukrain, JSC "Farmak"	Alla Mironenko, Andriy Goy, Ihor Kravchuk, Ludmyla Bolotova, Larysa Radchenko, Nataliia Teteriuk
EPI_ISL_582511, EPI_ISL_582512, EPI_ISL_582513	Department of Respiratory and other Viral Infections of L.V.Gromashevsky Institute of Epidemiology & Infectious Diseases NAMS of Ukrain	Department of Respiratory and other Viral Infections of L.V.Gromashevsky Institute of Epidemiology & Infectious Diseases NAMS of Ukrain, JSC "Farmak"	Alla Mironenko, Andriy Goy, Ihor Kravchuk, Ludmyla Bolotova, Larysa Radchenko, Nataliia Teteriuk
EPI_ISL_582608, EPI_ISL_582609, EPI_ISL_582610, EPI_ISL_582611, EPI_ISL_582612, EPI_ISL_582613, EPI_ISL_582614, EPI_ISL_582615, EPI_ISL_582616, EPI_ISL_582617, EPI_ISL_582618, EPI_ISL_582619, EPI_ISL_582620, EPI_ISL_582621, EPI_ISL_582622, EPI_ISL_582623, EPI_ISL_582624, EPI_ISL_582625, EPI_ISL_582626, EPI_ISL_582627, EPI_ISL_582628, EPI_ISL_582629, EPI_ISL_582630, EPI_ISL_582631, EPI_ISL_582632, EPI_ISL_582633, EPI_ISL_582634, EPI_ISL_582635, EPI_ISL_582636, EPI_ISL_582637, EPI_ISL_582638, EPI_ISL_582639, EPI_ISL_582640, EPI_ISL_582641, EPI_ISL_582642, EPI_ISL_582643, EPI_ISL_582644, EPI_ISL_582645, EPI_ISL_582646, EPI_ISL_582647, EPI_ISL_582648, EPI_ISL_582649, EPI_ISL_582650, EPI_ISL_582651, EPI_ISL_582652, EPI_ISL_582653, EPI_ISL_582654, EPI_ISL_582655, EPI_ISL_582656, EPI_ISL_582657, EPI_ISL_582658, EPI_ISL_582659, EPI_ISL_582660, EPI_ISL_582661, EPI_ISL_582662, EPI_ISL_582663, EPI_ISL_582664, EPI_ISL_582665, EPI_ISL_582666, EPI_ISL_582667, EPI_ISL_582668, EPI_ISL_582669, EPI_ISL_582670, EPI_ISL_582671, EPI_ISL_582672, EPI_ISL_582673, EPI_ISL_582674, EPI_ISL_582675, EPI_ISL_582676, EPI_ISL_582677, EPI_ISL_582678, EPI_ISL_582679, EPI_ISL_582680, EPI_ISL_582681, EPI_ISL_582682, EPI_ISL_582683, EPI_ISL_582684, EPI_ISL_582685, EPI_ISL_582686, EPI_ISL_582687, EPI_ISL_582688, EPI_ISL_582689, EPI_ISL_582690, EPI_ISL_582691			
see above	Sheikh Khalifa Medical City	Molecular/Surveillance lab Sheikh Khalifa Medical City	Amirtharaj Francis, Sajeed Abdul, Hala Imambaccus, Sahar Almarzooqi, Hiba Saud, Stefan Weber
EPI_ISL_583426	Garcia-Sastre Laboratory, Department of Microbiology, Icahn School of Medicine at Mount Sinai	van Bakel Laboratory, Genetics and Genomics Sciences, Icahn School of Medicine at Mount Sinai	Teresa Aydillo, Ana S. Gonzalez-Reiche, Sadaf Aslam, Adriana van de Guchte, Zenab Khan, Ajay Obla, Jayeeta Dutta, Harm van Bakel, Judith Aberg, Adolfo García-Sastre, Gunjan Shah, Tobias Hohl, Genovefa Papanicolaou, Miguel-Angel Perales, Kent Sepkowitz, Ngolela Esther Babady, and Mini Kamboj
EPI_ISL_583427, EPI_ISL_583428	Memorial Sloan Kettering Cancer Center	van Bakel Laboratory, Genetics and Genomics Sciences, Icahn School of Medicine at Mount Sinai	Teresa Aydillo, Ana S. Gonzalez-Reiche, Sadaf Aslam, Adriana van de Guchte, Zenab Khan, Ajay Obla, Jayeeta Dutta, Harm van Bakel, Judith Aberg, Adolfo García-Sastre, Gunjan Shah, Tobias Hohl, Genovefa Papanicolaou, Miguel-Angel Perales, Kent Sepkowitz, Ngolela Esther Babady, and Mini Kamboj
EPI_ISL_583429	Garcia-Sastre Laboratory, Department of Microbiology, Icahn School of Medicine at Mount Sinai	van Bakel Laboratory, Genetics and Genomics Sciences, Icahn School of Medicine at Mount Sinai	Teresa Aydillo, Ana S. Gonzalez-Reiche, Sadaf Aslam, Adriana van de Guchte, Zenab Khan, Ajay Obla, Jayeeta Dutta, Harm van Bakel, Judith Aberg, Adolfo García-Sastre, Gunjan Shah, Tobias Hohl, Genovefa Papanicolaou, Miguel-Angel Perales, Kent Sepkowitz, Ngolela Esther Babady, and Mini Kamboj
EPI_ISL_583430, EPI_ISL_583431, EPI_ISL_583432, EPI_ISL_583433	Memorial Sloan Kettering Cancer Center	van Bakel Laboratory, Genetics and Genomics Sciences, Icahn School of Medicine at Mount Sinai	Teresa Aydillo, Ana S. Gonzalez-Reiche, Sadaf Aslam, Adriana van de Guchte, Zenab Khan, Ajay Obla, Jayeeta Dutta, Harm van Bakel, Judith Aberg, Adolfo García-Sastre, Gunjan Shah, Tobias Hohl, Genovefa Papanicolaou, Miguel-Angel Perales, Kent Sepkowitz, Ngolela Esther Babady, and Mini Kamboj
EPI_ISL_583434, EPI_ISL_583435, EPI_ISL_583436	Garcia-Sastre Laboratory, Department of Microbiology, Icahn School of Medicine at Mount Sinai	van Bakel Laboratory, Genetics and Genomics Sciences, Icahn School of Medicine at Mount Sinai	Teresa Aydillo, Ana S. Gonzalez-Reiche, Sadaf Aslam, Adriana van de Guchte, Zenab Khan, Ajay Obla, Jayeeta Dutta, Harm van Bakel, Judith Aberg, Adolfo García-Sastre, Gunjan Shah, Tobias Hohl, Genovefa Papanicolaou, Miguel-Angel Perales, Kent Sepkowitz, Ngolela Esther Babady, and Mini

			Kamboj
EPI_ISL_583437, EPI_ISL_583438, EPI_ISL_583439, EPI_ISL_583440	Memorial Sloan Kettering Cancer Center	van Bakel Laboratory, Genetics and Genomics Sciences, Icahn School of Medicine at Mount Sinai	Teresa Aydillo, Ana S. Gonzalez-Reiche, Sadaf Aslam, Adriana van de Guchte, Zenab Khan, Ajay Obla, Jayeeta Dutta, Harm van Bakel, Judith Aberg, Adolfo García-Sastre, Gunjan Shah, Tobias Hohl, Genovefa Papanicolaou, Miguel-Angel Perales, Kent Sepkowitz, Ngolela Esther Babady, and Mini Kamboj
EPI_ISL_583441, EPI_ISL_583442	Garcia-Sastre Laboratory, Department of Microbiology, Icahn School of Medicine at Mount Sinai	van Bakel Laboratory, Genetics and Genomics Sciences, Icahn School of Medicine at Mount Sinai	Teresa Aydillo, Ana S. Gonzalez-Reiche, Sadaf Aslam, Adriana van de Guchte, Zenab Khan, Ajay Obla, Jayeeta Dutta, Harm van Bakel, Judith Aberg, Adolfo García-Sastre, Gunjan Shah, Tobias Hohl, Genovefa Papanicolaou, Miguel-Angel Perales, Kent Sepkowitz, Ngolela Esther Babady, and Mini Kamboj
EPI_ISL_583443, EPI_ISL_583444, EPI_ISL_583445, EPI_ISL_583446, EPI_ISL_583447, EPI_ISL_583448, EPI_ISL_583449, EPI_ISL_583450, EPI_ISL_583451	Memorial Sloan Kettering Cancer Center	van Bakel Laboratory, Genetics and Genomics Sciences, Icahn School of Medicine at Mount Sinai	Teresa Aydillo, Ana S. Gonzalez-Reiche, Sadaf Aslam, Adriana van de Guchte, Zenab Khan, Ajay Obla, Jayeeta Dutta, Harm van Bakel, Judith Aberg, Adolfo García-Sastre, Gunjan Shah, Tobias Hohl, Genovefa Papanicolaou, Miguel-Angel Perales, Kent Sepkowitz, Ngolela Esther Babady, and Mini Kamboj
EPI_ISL_583452, EPI_ISL_583453, EPI_ISL_583454	Garcia-Sastre Laboratory, Department of Microbiology, Icahn School of Medicine at Mount Sinai	van Bakel Laboratory, Genetics and Genomics Sciences, Icahn School of Medicine at Mount Sinai	Teresa Aydillo, Ana S. Gonzalez-Reiche, Sadaf Aslam, Adriana van de Guchte, Zenab Khan, Ajay Obla, Jayeeta Dutta, Harm van Bakel, Judith Aberg, Adolfo García-Sastre, Gunjan Shah, Tobias Hohl, Genovefa Papanicolaou, Miguel-Angel Perales, Kent Sepkowitz, Ngolela Esther Babady, and Mini Kamboj
EPI_ISL_583455, EPI_ISL_583456, EPI_ISL_583457	Memorial Sloan Kettering Cancer Center	van Bakel Laboratory, Genetics and Genomics Sciences, Icahn School of Medicine at Mount Sinai	Teresa Aydillo, Ana S. Gonzalez-Reiche, Sadaf Aslam, Adriana van de Guchte, Zenab Khan, Ajay Obla, Jayeeta Dutta, Harm van Bakel, Judith Aberg, Adolfo García-Sastre, Gunjan Shah, Tobias Hohl, Genovefa Papanicolaou, Miguel-Angel Perales, Kent Sepkowitz, Ngolela Esther Babady, and Mini Kamboj
EPI_ISL_583458, EPI_ISL_583459	Garcia-Sastre Laboratory, Department of Microbiology, Icahn School of Medicine at Mount Sinai	van Bakel Laboratory, Genetics and Genomics Sciences, Icahn School of Medicine at Mount Sinai	Teresa Aydillo, Ana S. Gonzalez-Reiche, Sadaf Aslam, Adriana van de Guchte, Zenab Khan, Ajay Obla, Jayeeta Dutta, Harm van Bakel, Judith Aberg, Adolfo García-Sastre, Gunjan Shah, Tobias Hohl, Genovefa Papanicolaou, Miguel-Angel Perales, Kent Sepkowitz, Ngolela Esther Babady, and Mini Kamboj
EPI_ISL_583460, EPI_ISL_583461, EPI_ISL_583462	Memorial Sloan Kettering Cancer Center	van Bakel Laboratory, Genetics and Genomics Sciences, Icahn School of Medicine at Mount Sinai	Teresa Aydillo, Ana S. Gonzalez-Reiche, Sadaf Aslam, Adriana van de Guchte, Zenab Khan, Ajay Obla, Jayeeta Dutta, Harm van Bakel, Judith Aberg, Adolfo García-Sastre, Gunjan Shah, Tobias Hohl, Genovefa Papanicolaou, Miguel-Angel Perales, Kent Sepkowitz, Ngolela Esther Babady, and Mini Kamboj
EPI_ISL_583463, EPI_ISL_583464	Garcia-Sastre Laboratory, Department of Microbiology, Icahn School of Medicine at Mount Sinai	van Bakel Laboratory, Genetics and Genomics Sciences, Icahn School of Medicine at Mount Sinai	Teresa Aydillo, Ana S. Gonzalez-Reiche, Sadaf Aslam, Adriana van de Guchte, Zenab Khan, Ajay Obla, Jayeeta Dutta, Harm van Bakel, Judith Aberg, Adolfo García-Sastre, Gunjan Shah, Tobias Hohl, Genovefa Papanicolaou, Miguel-Angel Perales, Kent Sepkowitz, Ngolela Esther Babady, and Mini Kamboj
EPI_ISL_583465, EPI_ISL_583466	Memorial Sloan Kettering Cancer Center	van Bakel Laboratory, Genetics and Genomics Sciences, Icahn School of Medicine at Mount Sinai	Teresa Aydillo, Ana S. Gonzalez-Reiche, Sadaf Aslam, Adriana van de Guchte, Zenab Khan, Ajay Obla, Jayeeta Dutta, Harm van Bakel, Judith Aberg, Adolfo García-Sastre, Gunjan Shah, Tobias Hohl, Genovefa Papanicolaou, Miguel-Angel Perales, Kent Sepkowitz, Ngolela Esther Babady, and Mini Kamboj
EPI_ISL_583467	Garcia-Sastre Laboratory, Department of Microbiology, Icahn School of Medicine at Mount Sinai	van Bakel Laboratory, Genetics and Genomics Sciences, Icahn School of Medicine at Mount Sinai	Teresa Aydillo, Ana S. Gonzalez-Reiche, Sadaf Aslam, Adriana van de Guchte, Zenab Khan, Ajay Obla, Jayeeta Dutta, Harm van Bakel, Judith Aberg, Adolfo García-Sastre, Gunjan Shah, Tobias Hohl, Genovefa Papanicolaou, Miguel-Angel Perales, Kent Sepkowitz, Ngolela Esther Babady, and Mini Kamboj
EPI_ISL_583468, EPI_ISL_583469, EPI_ISL_583470	Memorial Sloan Kettering Cancer Center	van Bakel Laboratory, Genetics and Genomics Sciences, Icahn School of Medicine at Mount Sinai	Teresa Aydillo, Ana S. Gonzalez-Reiche, Sadaf Aslam, Adriana van de Guchte, Zenab Khan, Ajay Obla, Jayeeta Dutta, Harm van Bakel, Judith Aberg, Adolfo García-Sastre, Gunjan Shah, Tobias Hohl, Genovefa Papanicolaou, Miguel-Angel Perales, Kent Sepkowitz, Ngolela Esther Babady, and Mini Kamboj
EPI_ISL_583471	Garcia-Sastre Laboratory, Department of Microbiology, Icahn School of Medicine at Mount Sinai	van Bakel Laboratory, Genetics and Genomics Sciences, Icahn School of Medicine at Mount Sinai	Teresa Aydillo, Ana S. Gonzalez-Reiche, Sadaf Aslam, Adriana van de Guchte, Zenab Khan, Ajay Obla, Jayeeta Dutta, Harm van Bakel, Judith Aberg, Adolfo García-Sastre, Gunjan Shah, Tobias Hohl, Genovefa Papanicolaou, Miguel-Angel Perales, Kent Sepkowitz, Ngolela Esther Babady, and Mini Kamboj
EPI_ISL_583472	Memorial Sloan Kettering Cancer Center	van Bakel Laboratory, Genetics and Genomics Sciences, Icahn School of Medicine at Mount Sinai	Teresa Aydillo, Ana S. Gonzalez-Reiche, Sadaf Aslam, Adriana van de Guchte, Zenab Khan, Ajay Obla, Jayeeta Dutta, Harm van Bakel, Judith Aberg, Adolfo García-Sastre, Gunjan Shah, Tobias Hohl, Genovefa Papanicolaou, Miguel-Angel Perales, Kent Sepkowitz, Ngolela Esther Babady, and Mini Kamboj
EPI_ISL_583473	Garcia-Sastre Laboratory, Department of Microbiology, Icahn School of Medicine at Mount Sinai	van Bakel Laboratory, Genetics and Genomics Sciences, Icahn School of Medicine at Mount Sinai	Teresa Aydillo, Ana S. Gonzalez-Reiche, Sadaf Aslam, Adriana van de Guchte, Zenab Khan, Ajay Obla, Jayeeta Dutta, Harm van Bakel, Judith Aberg, Adolfo García-Sastre, Gunjan Shah, Tobias Hohl, Genovefa Papanicolaou, Miguel-Angel Perales, Kent Sepkowitz, Ngolela Esther Babady, and Mini Kamboj
EPI_ISL_583474, EPI_ISL_583475, EPI_ISL_583476	Memorial Sloan Kettering Cancer Center	van Bakel Laboratory, Genetics and Genomics Sciences, Icahn School of Medicine at Mount Sinai	Teresa Aydillo, Ana S. Gonzalez-Reiche, Sadaf Aslam, Adriana van de Guchte, Zenab Khan, Ajay Obla, Jayeeta Dutta, Harm van Bakel, Judith Aberg, Adolfo García-Sastre, Gunjan Shah, Tobias Hohl, Genovefa Papanicolaou, Miguel-Angel Perales, Kent Sepkowitz, Ngolela Esther Babady, and Mini Kamboj
EPI_ISL_583477	Garcia-Sastre Laboratory, Department of Microbiology, Icahn School of Medicine at Mount Sinai	van Bakel Laboratory, Genetics and Genomics Sciences, Icahn School of Medicine at Mount Sinai	Teresa Aydillo, Ana S. Gonzalez-Reiche, Sadaf Aslam, Adriana van de Guchte, Zenab Khan, Ajay Obla, Jayeeta Dutta, Harm van Bakel, Judith Aberg, Adolfo García-Sastre, Gunjan Shah, Tobias Hohl, Genovefa Papanicolaou, Miguel-Angel Perales, Kent Sepkowitz, Ngolela Esther Babady, and Mini Kamboj
EPI_ISL_583478, EPI_ISL_583479, EPI_ISL_583480	Memorial Sloan Kettering Cancer Center	van Bakel Laboratory, Genetics and Genomics Sciences, Icahn School of Medicine at Mount Sinai	Teresa Aydillo, Ana S. Gonzalez-Reiche, Sadaf Aslam, Adriana van de Guchte, Zenab Khan, Ajay Obla, Jayeeta Dutta, Harm van Bakel, Judith Aberg, Adolfo García-Sastre, Gunjan Shah, Tobias Hohl, Genovefa Papanicolaou, Miguel-Angel Perales, Kent Sepkowitz, Ngolela Esther Babady, and Mini Kamboj
EPI_ISL_583490	Hospital Estadual Sumare	Instituto Adolfo Lutz, Interdiciplinary Procedures Center, Strategic Laboratory	Claudio Tavares Sacchi, Claudia Regina Gonçalves, Erica Valessa Ramos Gomes, Karoline Rodrigues Campos
EPI_ISL_583491	Centro de Saude Esf IV Zona Rual Domingos de SJ Rio Pardo	Instituto Adolfo Lutz, Interdiciplinary Procedures Center, Strategic Laboratory	Claudio Tavares Sacchi, Claudia Regina Gonçalves, Erica Valessa Ramos Gomes, Karoline Rodrigues Campos
EPI_ISL_583492	Santa Casa Anna Cintra	Instituto Adolfo Lutz, Interdiciplinary Procedures Center, Strategic Laboratory	Claudio Tavares Sacchi, Claudia Regina Gonçalves, Erica Valessa Ramos Gomes, Karoline Rodrigues Campos
EPI_ISL_583493	Vigilância em Saúde de Cajamar	Instituto Adolfo Lutz, Interdiciplinary Procedures Center, Strategic Laboratory	Claudio Tavares Sacchi, Claudia Regina Gonçalves, Erica Valessa Ramos Gomes, Karoline Rodrigues Campos
EPI_ISL_583494	CS II Dr. Antonio Vicoso Moreira de Rezende Sumare	Instituto Adolfo Lutz, Interdiciplinary Procedures Center, Strategic Laboratory	Claudio Tavares Sacchi, Claudia Regina Gonçalves, Erica Valessa Ramos Gomes, Karoline Rodrigues Campos
EPI_ISL_583495	Serviço de Verificação de Óbitos SVO Guaruulhos	Instituto Adolfo Lutz, Interdiciplinary Procedures Center, Strategic Laboratory	Claudio Tavares Sacchi, Claudia Regina Gonçalves, Erica Valessa Ramos Gomes, Karoline Rodrigues Campos
EPI_ISL_583496	UPA Jandira	Instituto Adolfo Lutz, Interdiciplinary Procedures Center, Strategic Laboratory	Claudio Tavares Sacchi, Claudia Regina Gonçalves, Erica Valessa Ramos Gomes, Karoline Rodrigues Campos
EPI_ISL_583497	Complexo Hospitalar Ouro Verde de Campinas	Instituto Adolfo Lutz, Interdiciplinary Procedures Center, Strategic Laboratory	Claudio Tavares Sacchi, Claudia Regina Gonçalves, Erica Valessa Ramos Gomes, Karoline Rodrigues Campos
EPI_ISL_583498	Hospital Municipal Dr. Waldemar Tebaldi	Instituto Adolfo Lutz, Interdiciplinary Procedures Center, Strategic Laboratory	Claudio Tavares Sacchi, Claudia Regina Gonçalves, Erica Valessa Ramos Gomes, Karoline Rodrigues Campos
EPI_ISL_583499	Distrito Sanitario Sul Campinas	Instituto Adolfo Lutz, Interdiciplinary Procedures Center, Strategic Laboratory	Claudio Tavares Sacchi, Claudia Regina Gonçalves, Erica Valessa Ramos Gomes, Karoline Rodrigues Campos

EPI_ISL_583500	Centro de Saude I Tacito Leite de Carvalho e Silva	Instituto Adolfo Lutz, Interdiciplinary Procedures Center, Strategic Laboratory	Claudio Tavares Sacchi, Claudia Regina Gonçalves, Erica Valessa Ramos Gomes, Karoline Rodrigues Campos
EPI_ISL_583501	Hospital Estadual de CampanhaCOVID 19 Barradas	Instituto Adolfo Lutz, Interdiciplinary Procedures Center, Strategic Laboratory	Claudio Tavares Sacchi, Claudia Regina Gonçalves, Erica Valessa Ramos Gomes, Karoline Rodrigues Campos
EPI_ISL_583502	Serv de Vig Sanitaria Epidemio e CTRL de Zoonoses Guaruja	Instituto Adolfo Lutz, Interdiciplinary Procedures Center, Strategic Laboratory	Claudio Tavares Sacchi, Claudia Regina Gonçalves, Erica Valessa Ramos Gomes, Karoline Rodrigues Campos
EPI_ISL_583503	CTA Centro de Testagem e Aconselhamento	Instituto Adolfo Lutz, Interdiciplinary Procedures Center, Strategic Laboratory	Claudio Tavares Sacchi, Claudia Regina Gonçalves, Erica Valessa Ramos Gomes, Karoline Rodrigues Campos
EPI_ISL_583504, EPI_ISL_583505	Casa de Saude Stella Maris	Instituto Adolfo Lutz, Interdiciplinary Procedures Center, Strategic Laboratory	Claudio Tavares Sacchi, Claudia Regina Gonçalves, Erica Valessa Ramos Gomes, Karoline Rodrigues Campos
EPI_ISL_583893, EPI_ISL_583894, EPI_ISL_583895, EPI_ISL_583896, EPI_ISL_583897, EPI_ISL_583898, EPI_ISL_583899, EPI_ISL_583900	Singapore General Hospital	Department of Microbiology	Nurdyana Abdul Rahman, Kun Lee Lim, Chenhao Li, Sui Sin Goh, Kenneth Xin Long Chan, Kian Sing Chan, Lynette Oon, Kern Rei Chng, Niranjan Nagarajan, Karrie Ko
EPI_ISL_583914	DH	Department of Neurovirology, National Institute of Mental Health and Neuroscience (NIMHANS)	Chitra Pattabiraman,Vijayalakshmi Reddy, Harsha PK, Risha Rasheed, Pramada Prasad, Shafeeq S Hameed, Manjunatha Venkataswamy, Anita Desai, Ravi Vasanthapuram
EPI_ISL_583915	Victoria Hospital	Department of Neurovirology, National Institute of Mental Health and Neuroscience (NIMHANS)	Chitra Pattabiraman,Vijayalakshmi Reddy, Harsha PK, Risha Rasheed, Pramada Prasad, Shafeeq S Hameed, Manjunatha Venkataswamy, Anita Desai, Ravi Vasanthapuram
EPI_ISL_584048	Laboratory of Molecular Virology, Department of Biomedical, Surgical and Dental Sciences University of Milano	Laboratory of Molecular Virology, Department of Biomedical, Surgical and Dental Sciences University of Milano	Delbue,S., Modenese,A., Bianchi,M., Fattori,M., D'Alessandro,S., Pariani,E., Basilico,N., Galli,C. and Ferrante,P.
EPI_ISL_584049	Laboratory of Molecular Virology, Department of Biomedical, Surgical and Dental Sciences University of Milano	Laboratory of Molecular Virology, Department of Biomedical, Surgical and Dental Sciences University of Milano	Delbue,S., Modenese,A., Bianchi,M., Fattori,M., D'Alessandro,S.,Pariani,E., Basilico,N., Galli,C. and Ferrante,P.
EPI_ISL_584052	Laboratory of Molecular Virology, Department of Biomedical, Surgical and Dental Sciences University of Milano	Laboratory of Molecular Virology, Department of Biomedical, Surgical and Dental Sciences University of Milano	Delbue,S., D'Alessandro,S., Modenese,A., Signorini,L., Parapini,S.,Dolci,M., Binda,S., Primache,V., Taramelli,D., Incorvaia,B. and Ferrante,P.
EPI_ISL_584073, EPI_ISL_584074, EPI_ISL_584075, EPI_ISL_584076, EPI_ISL_584077, EPI_ISL_584078, EPI_ISL_584079, EPI_ISL_584080, EPI_ISL_584082	The National Institute of Public Health	State Veterinary Institute Prague	Nagy,A;Jirincova,H;Novakova,L;Trnka,D;Vecerova,J
EPI_ISL_586513	B.J. Medical College and Civil hospital, Ahmedabad	Gujarat Biotechnology Research Centre	Pinal Trivedi, Maharshi Pandya, Nidhi Patel, Nitin Savaliya, Raghawendra Kumar, Dinesh Kumar, Zuber Saiyed, Komal Patel, Labdhi Pandya, Afzal Ansari, Nikha Trivedi, Pranay Shah, Kamlesh J Upadhyay, Sanjay Kapadia, Apurvasinh Puvar, Janvi Raval, Zarna Patel, Monika Gandhi, R D Dixit, A M Kadri, Harsh Bakshi, Chaitanya Joshi, Madhvi Joshi
EPI_ISL_586514	B.J. Medical College and Civil hospital, Ahmedabad	Gujarat Biotechnology Research Centre	Maharshi Pandya, Nidhi Patel, Nitin Savaliya, Raghawendra Kumar, Dinesh Kumar, Zuber Saiyed, Komal Patel, Labdhi Pandya, Afzal Ansari, Nikha Trivedi, Pranay Shah, Kamlesh J Upadhyay, Sanjay Kapadia, Apurvasinh Puvar, Janvi Raval, Zarna Patel, Monika Gandhi, Pinal Trivedi, R D Dixit, A M Kadri, Harsh Bakshi, Chaitanya Joshi, Madhvi Joshi
EPI_ISL_586515	B.J. Medical College and Civil hospital, Ahmedabad	Gujarat Biotechnology Research Centre	Nidhi Patel, Nitin Savaliya, Raghawendra Kumar, Dinesh Kumar, Zuber Saiyed, Komal Patel, Labdhi Pandya, Afzal Ansari, Nikha Trivedi, Pranay Shah, Kamlesh J Upadhyay, Sanjay Kapadia, Apurvasinh Puvar, Janvi Raval, Zarna Patel, Monika Gandhi, Pinal Trivedi, Maharshi Pandya, R D Dixit, A M Kadri, Harsh Bakshi, Chaitanya Joshi, Madhvi Joshi
EPI_ISL_586516	B.J. Medical College and Civil hospital, Ahmedabad	Gujarat Biotechnology Research Centre	Nitin Savaliya, Raghawendra Kumar, Dinesh Kumar, Zuber Saiyed, Komal Patel, Labdhi Pandya, Afzal Ansari, Nikha Trivedi, Pranay Shah, Kamlesh J Upadhyay, Sanjay Kapadia, Apurvasinh Puvar, Janvi Raval, Zarna Patel, Monika Gandhi, Pinal Trivedi, Maharshi Pandya, Nidhi Patel, R D Dixit, A M Kadri, Harsh Bakshi, Chaitanya Joshi, Madhvi Joshi
EPI_ISL_586517	B.J. Medical College and Civil hospital, Ahmedabad	Gujarat Biotechnology Research Centre	Raghawendra Kumar, Dinesh Kumar, Zuber Saiyed, Komal Patel, Labdhi Pandya, Afzal Ansari, Nikha Trivedi, Pranay Shah, Kamlesh J Upadhyay, Sanjay Kapadia, Apurvasinh Puvar, Janvi Raval, Zarna Patel, Monika Gandhi, Pinal Trivedi, Maharshi Pandya, Nidhi Patel, Nitin Savaliya, R D Dixit, A M Kadri, Harsh Bakshi, Chaitanya Joshi, Madhvi Joshi
EPI_ISL_586518	B.J. Medical College and Civil hospital, Ahmedabad	Gujarat Biotechnology Research Centre	Dinesh Kumar, Zuber Saiyed, Komal Patel, Labdhi Pandya, Afzal Ansari, Nikha Trivedi, Pranay Shah, Kamlesh J Upadhyay, Sanjay Kapadia, Apurvasinh Puvar, Janvi Raval, Zarna Patel, Monika Gandhi, Pinal Trivedi, Maharshi Pandya, Nidhi Patel, Nitin Savaliya, Raghawendra Kumar, R D Dixit, A M Kadri, Harsh Bakshi, Chaitanya Joshi, Madhvi Joshi
EPI_ISL_586519	B.J. Medical College and Civil hospital, Ahmedabad	Gujarat Biotechnology Research Centre	Zuber Saiyed, Komal Patel, Labdhi Pandya, Afzal Ansari, Nikha Trivedi, Pranay Shah, Kamlesh J Upadhyay, Sanjay Kapadia, Apurvasinh Puvar, Janvi Raval, Zarna Patel, Monika Gandhi, Pinal Trivedi, Maharshi Pandya, Nidhi Patel, Nitin Savaliya, Raghawendra Kumar, Dinesh Kumar, R D Dixit, A M Kadri, Harsh Bakshi, Chaitanya Joshi, Madhvi Joshi
EPI_ISL_586520	B.J. Medical College and Civil hospital, Ahmedabad	Gujarat Biotechnology Research Centre	Komal Patel, Labdhi Pandya, Afzal Ansari, Nikha Trivedi, Pranay Shah, Kamlesh J Upadhyay, Sanjay Kapadia, Apurvasinh Puvar, Janvi Raval, Zarna Patel, Monika Gandhi, Pinal Trivedi, Maharshi Pandya, Nidhi Patel, Nitin Savaliya, Raghawendra Kumar, Dinesh Kumar, Zuber Saiyed, R D Dixit, A M Kadri, Harsh Bakshi, Chaitanya Joshi, Madhvi Joshi
EPI_ISL_586521	B.J. Medical College and Civil hospital, Ahmedabad	Gujarat Biotechnology Research Centre	Labdhi Pandya, Afzal Ansari, Nikha Trivedi, Pranay Shah, Kamlesh J Upadhyay, Sanjay Kapadia, Apurvasinh Puvar, Janvi Raval, Zarna Patel, Monika Gandhi, Pinal Trivedi, Maharshi Pandya, Nidhi Patel, Nitin Savaliya, Raghawendra Kumar, Dinesh Kumar, Zuber Saiyed, Komal Patel, R D Dixit, A M Kadri, Harsh Bakshi, Chaitanya Joshi, Madhvi Joshi
EPI_ISL_586522	B.J. Medical College and Civil hospital, Ahmedabad	Gujarat Biotechnology Research Centre	Afzal Ansari, Nikha Trivedi, Pranay Shah, Kamlesh J Upadhyay, Sanjay Kapadia, Apurvasinh Puvar, Janvi Raval, Zarna Patel, Monika Gandhi, Pinal Trivedi, Maharshi Pandya, Nidhi Patel, Nitin Savaliya, Raghawendra Kumar, Dinesh Kumar, Zuber Saiyed, Komal Patel, Labdhi Pandya, R D Dixit, A M Kadri, Harsh Bakshi, Chaitanya Joshi, Madhvi Joshi
EPI_ISL_586523	B.J. Medical College and Civil hospital, Ahmedabad	Gujarat Biotechnology Research Centre	Nikha Trivedi, Pranay Shah, Kamlesh J Upadhyay, Sanjay Kapadia, Apurvasinh Puvar, Janvi Raval, Zarna Patel, Monika Gandhi, Pinal Trivedi, Maharshi Pandya, Nidhi Patel, Nitin Savaliya, Raghawendra Kumar, Dinesh Kumar, Zuber Saiyed, Komal Patel, Labdhi Pandya, Afzal Ansari, R D Dixit, A M Kadri, Harsh Bakshi, Chaitanya Joshi, Madhvi Joshi
EPI_ISL_586524	B.J. Medical College and Civil hospital, Ahmedabad	Gujarat Biotechnology Research Centre	Pranay Shah, Kamlesh J Upadhyay, Sanjay Kapadia, Apurvasinh Puvar, Janvi Raval, Zarna Patel, Monika Gandhi, Pinal Trivedi, Maharshi Pandya, Nidhi Patel, Nitin Savaliya, Raghawendra Kumar, Dinesh Kumar, Zuber Saiyed, Komal Patel, Labdhi Pandya, Afzal Ansari, Nikha Trivedi, R D Dixit, A M Kadri, Harsh Bakshi, Chaitanya Joshi, Madhvi Joshi
EPI_ISL_586525	B.J. Medical College and Civil hospital, Ahmedabad	Gujarat Biotechnology Research Centre	Kamlesh J Upadhyay, Sanjay Kapadia, Apurvasinh Puvar, Janvi Raval, Zarna Patel, Monika Gandhi, Pinal Trivedi, Maharshi Pandya, Nidhi Patel, Nitin Savaliya, Raghawendra Kumar, Dinesh Kumar, Zuber Saiyed, Komal Patel, Labdhi Pandya, Afzal Ansari, Nikha Trivedi, Pranay Shah, R D Dixit, A M Kadri, Harsh Bakshi, Chaitanya Joshi, Madhvi Joshi
EPI_ISL_586526	B.J. Medical College and Civil hospital, Ahmedabad	Gujarat Biotechnology Research Centre	Sanjay Kapadia, Apurvasinh Puvar, Janvi Raval, Zarna Patel, Monika Gandhi, Pinal Trivedi, Maharshi Pandya, Nidhi Patel, Nitin Savaliya, Raghawendra Kumar, Dinesh Kumar, Zuber Saiyed, Komal Patel, Labdhi Pandya, Afzal Ansari, Nikha Trivedi, Pranay Shah, Kamlesh J Upadhyay, R D Dixit, A M Kadri, Harsh Bakshi, Chaitanya Joshi, Madhvi Joshi
EPI_ISL_586527	B.J. Medical College and Civil hospital, Ahmedabad	Gujarat Biotechnology Research Centre	Apurvasinh Puvar, Janvi Raval, Zarna Patel, Monika Gandhi, Pinal Trivedi, Maharshi Pandya, Nidhi Patel, Nitin Savaliya, Raghawendra Kumar, Dinesh Kumar, Zuber Saiyed, Komal Patel, Labdhi Pandya, Afzal Ansari, Nikha Trivedi, Pranay Shah, Kamlesh J Upadhyay, Sanjay Kapadia, R D Dixit, A M Kadri, Harsh Bakshi, Chaitanya Joshi, Madhvi Joshi

EPI_ISL_586528	B.J. Medical College and Civil hospital, Ahmedabad	Gujarat Biotechnology Research Centre	Zarna Patel, Monika Gandhi, Pinal Trivedi, Maharshi Pandya, Nidhi Patel, Nitin Savaliya, Raghawendra Kumar, Dinesh Kumar, Zuber Saiyed, Komal Patel, Labdhi Pandya, Afzal Ansari, Nikha Trivedi, Pranay Shah, Kamlesh J Upadhyay, Sanjay Kapadia, Apurvasinh Puvar, Janvi Raval, R D Dixit, A M Kadri, Harsh Bakshi, Chaitanya Joshi, Madhvi Joshi
EPI_ISL_586529	B.J. Medical College and Civil hospital, Ahmedabad	Gujarat Biotechnology Research Centre	Monika Gandhi, Pinal Trivedi, Maharshi Pandya, Nidhi Patel, Nitin Savaliya, Raghawendra Kumar, Dinesh Kumar, Zuber Saiyed, Komal Patel, Labdhi Pandya, Afzal Ansari, Nikha Trivedi, Pranay Shah, Kamlesh J Upadhyay, Sanjay Kapadia, Apurvasinh Puvar, Janvi Raval, Zarna Patel, R D Dixit, A M Kadri, Harsh Bakshi, Chaitanya Joshi, Madhvi Joshi
EPI_ISL_586530	B.J. Medical College and Civil hospital, Ahmedabad	Gujarat Biotechnology Research Centre	Pinal Trivedi, Maharshi Pandya, Nidhi Patel, Nitin Savaliya, Raghawendra Kumar, Dinesh Kumar, Zuber Saiyed, Komal Patel, Labdhi Pandya, Afzal Ansari, Nikha Trivedi, Pranay Shah, Kamlesh J Upadhyay, Sanjay Kapadia, Apurvasinh Puvar, Janvi Raval, Zarna Patel, Monika Gandhi, R D Dixit, A M Kadri, Harsh Bakshi, Chaitanya Joshi, Madhvi Joshi
EPI_ISL_586531	General Hospital,Chhotaudepur	Gujarat Biotechnology Research Centre	Yogesh Parmar, Arti Thakur, Dharak Pandya, Maharshi Pandya, Nidhi Patel, Nitin Savaliya, Raghawendra Kumar, Dinesh Kumar, Zuber Saiyed, Komal Patel, Labdhi Pandya, Afzal Ansari, Nikha Trivedi, Apurvasinh Puvar, Janvi Raval, Zarna Patel, Monika Gandhi, Pinal Trivedi, R D Dixit, A M Kadri, Harsh Bakshi, Chaitanya Joshi, Madhvi Joshi
EPI_ISL_586532	General Hospital,Chhotaudepur	Gujarat Biotechnology Research Centre	Arti Thakur, Dharak Pandya, Maharshi Pandya, Nidhi Patel, Nitin Savaliya, Raghawendra Kumar, Dinesh Kumar, Zuber Saiyed, Komal Patel, Labdhi Pandya, Afzal Ansari, Nikha Trivedi, Apurvasinh Puvar, Janvi Raval, Zarna Patel, Monika Gandhi, Pinal Trivedi, Yogesh Parmar, R D Dixit, A M Kadri, Harsh Bakshi, Chaitanya Joshi, Madhvi Joshi
EPI_ISL_586533	General Hospital,Chhotaudepur	Gujarat Biotechnology Research Centre	Dharak Pandya, Maharshi Pandya, Nidhi Patel, Nitin Savaliya, Raghawendra Kumar, Dinesh Kumar, Zuber Saiyed, Komal Patel, Labdhi Pandya, Afzal Ansari, Nikha Trivedi, Apurvasinh Puvar, Janvi Raval, Zarna Patel, Monika Gandhi, Pinal Trivedi, Yogesh Parmar, Arti Thakur, R D Dixit, A M Kadri, Harsh Bakshi, Chaitanya Joshi, Madhvi Joshi
EPI_ISL_586534	General Hospital,Chhotaudepur	Gujarat Biotechnology Research Centre	Maharshi Pandya, Nidhi Patel, Nitin Savaliya, Raghawendra Kumar, Dinesh Kumar, Zuber Saiyed, Komal Patel, Labdhi Pandya, Afzal Ansari, Nikha Trivedi, Apurvasinh Puvar, Janvi Raval, Zarna Patel, Monika Gandhi, Pinal Trivedi, Yogesh Parmar, Arti Thakur, Dharak Pandya, R D Dixit, A M Kadri, Harsh Bakshi, Chaitanya Joshi, Madhvi Joshi
EPI_ISL_586535	General Hospital,Chhotaudepur	Gujarat Biotechnology Research Centre	Nidhi Patel, Nitin Savaliya, Raghawendra Kumar, Dinesh Kumar, Zuber Saiyed, Komal Patel, Labdhi Pandya, Afzal Ansari, Nikha Trivedi, Apurvasinh Puvar, Janvi Raval, Zarna Patel, Monika Gandhi, Pinal Trivedi, Yogesh Parmar, Arti Thakur, Dharak Pandya, Maharshi Pandya, R D Dixit, A M Kadri, Harsh Bakshi, Chaitanya Joshi, Madhvi Joshi
EPI_ISL_586536	Zydus Medical Hospital,Dahod	Gujarat Biotechnology Research Centre	Kalpesh Labana, Mohit Desai, Nitin Savaliya, Raghawendra Kumar, Dinesh Kumar, Zuber Saiyed, Komal Patel, Labdhi Pandya, Afzal Ansari, Nikha Trivedi, Apurvasinh Puvar, Janvi Raval, Zarna Patel, Monika Gandhi, Pinal Trivedi, Maharshi Pandya, Nidhi Patel, R D Dixit, A M Kadri, Harsh Bakshi, Chaitanya Joshi, Madhvi Joshi
EPI_ISL_586537	Zydus Medical Hospital,Dahod	Gujarat Biotechnology Research Centre	Mohit Desai, Nitin Savaliya, Raghawendra Kumar, Dinesh Kumar, Zuber Saiyed, Komal Patel, Labdhi Pandya, Afzal Ansari, Nikha Trivedi, Apurvasinh Puvar, Janvi Raval, Zarna Patel, Monika Gandhi, Pinal Trivedi, Maharshi Pandya, Nidhi Patel, Kalpesh Labana, R D Dixit, A M Kadri, Harsh Bakshi, Chaitanya Joshi, Madhvi Joshi
EPI_ISL_586538	Zydus Medical Hospital,Dahod	Gujarat Biotechnology Research Centre	Nitin Savaliya, Raghawendra Kumar, Dinesh Kumar, Zuber Saiyed, Komal Patel, Labdhi Pandya, Afzal Ansari, Nikha Trivedi, Apurvasinh Puvar, Janvi Raval, Zarna Patel, Monika Gandhi, Pinal Trivedi, Maharshi Pandya, Nidhi Patel, Kalpesh Labana, Mohit Desai, R D Dixit, A M Kadri, Harsh Bakshi, Chaitanya Joshi, Madhvi Joshi
EPI_ISL_586539	Zydus Medical Hospital,Dahod	Gujarat Biotechnology Research Centre	Raghawendra Kumar, Dinesh Kumar, Zuber Saiyed, Komal Patel, Labdhi Pandya, Afzal Ansari, Nikha Trivedi, Apurvasinh Puvar, Janvi Raval, Zarna Patel, Monika Gandhi, Pinal Trivedi, Maharshi Pandya, Nidhi Patel, Kalpesh Labana, Mohit Desai, Nitin Savaliya, R D Dixit, A M Kadri, Harsh Bakshi, Chaitanya Joshi, Madhvi Joshi
EPI_ISL_586540	Zydus Medical Hospital,Dahod	Gujarat Biotechnology Research Centre	Dinesh Kumar, Zuber Saiyed, Komal Patel, Labdhi Pandya, Afzal Ansari, Nikha Trivedi, Apurvasinh Puvar, Janvi Raval, Zarna Patel, Monika Gandhi, Pinal Trivedi, Maharshi Pandya, Nidhi Patel, Kalpesh Labana, Mohit Desai, Nitin Savaliya, Raghawendra Kumar, R D Dixit, A M Kadri, Harsh Bakshi, Chaitanya Joshi, Madhvi Joshi
EPI_ISL_586541	Zydus Medical Hospital,Dahod	Gujarat Biotechnology Research Centre	Zuber Saiyed, Komal Patel, Labdhi Pandya, Afzal Ansari, Nikha Trivedi, Apurvasinh Puvar, Janvi Raval, Zarna Patel, Monika Gandhi, Pinal Trivedi, Maharshi Pandya, Nidhi Patel, Kalpesh Labana, Mohit Desai, Nitin Savaliya, Raghawendra Kumar, Dinesh Kumar, R D Dixit, A M Kadri, Harsh Bakshi, Chaitanya Joshi, Madhvi Joshi
EPI_ISL_586542	Government General Hospital, Jam Khambhaliya, Devbhoomi Dwarka	Gujarat Biotechnology Research Centre	Komal Patel, Labdhi Pandya, Afzal Ansari, Nikha Trivedi, Harish Matani, Apurvasinh Puvar, Janvi Raval, Zarna Patel, Monika Gandhi, Pinal Trivedi, Maharshi Pandya, Nidhi Patel, Nitin Savaliya, Raghawendra Kumar, Dinesh Kumar, Zuber Saiyed, R D Dixit, A M Kadri, Harsh Bakshi, Chaitanya Joshi, Madhvi Joshi
EPI_ISL_586543	Government General Hospital, Jam Khambhaliya, Devbhoomi Dwarka	Gujarat Biotechnology Research Centre	Labdhi Pandya, Afzal Ansari, Nikha Trivedi, Harish Matani, Apurvasinh Puvar, Janvi Raval, Zarna Patel, Monika Gandhi, Pinal Trivedi, Maharshi Pandya, Nidhi Patel, Nitin Savaliya, Raghawendra Kumar, Dinesh Kumar, Zuber Saiyed, Komal Patel, R D Dixit, A M Kadri, Harsh Bakshi, Chaitanya Joshi, Madhvi Joshi
EPI_ISL_586544	Government General Hospital, Jam Khambhaliya, Devbhoomi Dwarka	Gujarat Biotechnology Research Centre	Afzal Ansari, Nikha Trivedi, Harish Matani, Apurvasinh Puvar, Janvi Raval, Zarna Patel, Monika Gandhi, Pinal Trivedi, Maharshi Pandya, Nidhi Patel, Nitin Savaliya, Raghawendra Kumar, Dinesh Kumar, Zuber Saiyed, Komal Patel, Labdhi Pandya, R D Dixit, A M Kadri, Harsh Bakshi, Chaitanya Joshi, Madhvi Joshi
EPI_ISL_586545	Government General Hospital, Jam Khambhaliya, Devbhoomi Dwarka	Gujarat Biotechnology Research Centre	Nikha Trivedi, Harish Matani, Apurvasinh Puvar, Janvi Raval, Zarna Patel, Monika Gandhi, Pinal Trivedi, Maharshi Pandya, Nidhi Patel, Nitin Savaliya, Raghawendra Kumar, Dinesh Kumar, Zuber Saiyed, Komal Patel, Labdhi Pandya, Afzal Ansari, R D Dixit, A M Kadri, Harsh Bakshi, Chaitanya Joshi, Madhvi Joshi
EPI_ISL_586546	General Hospital,Godhra	Gujarat Biotechnology Research Centre	Neela Katara, Pinal Gandhi, Sangeeta Kumar, Apurvasinh Puvar, Janvi Raval, Zarna Patel, Monika Gandhi, Pinal Trivedi, Maharshi Pandya, Nidhi Patel, Nitin Savaliya, Raghawendra Kumar, Dinesh Kumar, Zuber Saiyed, Komal Patel, Labdhi Pandya, Afzal Ansari, Nikha Trivedi, R D Dixit, A M Kadri, Harsh Bakshi, Chaitanya Joshi, Madhvi Joshi
EPI_ISL_586547	General Hospital,Godhra	Gujarat Biotechnology Research Centre	Pinal Gandhi, Sangeeta Kumar, Apurvasinh Puvar, Janvi Raval, Zarna Patel, Monika Gandhi, Pinal Trivedi, Maharshi Pandya, Nidhi Patel, Nitin Savaliya, Raghawendra Kumar, Dinesh Kumar, Zuber Saiyed, Komal Patel, Labdhi Pandya, Afzal Ansari, Nikha Trivedi, Neela Katara, R D Dixit, A M Kadri, Harsh Bakshi, Chaitanya Joshi, Madhvi Joshi
EPI_ISL_586548	General Hospital,Godhra	Gujarat Biotechnology Research Centre	Sangeeta Kumar, Apurvasinh Puvar, Janvi Raval, Zarna Patel, Monika Gandhi, Pinal Trivedi, Maharshi Pandya, Nidhi Patel, Nitin Savaliya, Raghawendra Kumar, Dinesh Kumar, Zuber Saiyed, Komal Patel, Labdhi Pandya, Afzal Ansari, Nikha Trivedi, Neela Katara, Pinal Gandhi, R D Dixit, A M Kadri, Harsh Bakshi, Chaitanya Joshi, Madhvi Joshi
EPI_ISL_586549	CCC,Veraval	Gujarat Biotechnology Research Centre	Jignesh Parmar, Jitendra Bamrotia, Apurvasinh Puvar, Janvi Raval, Zarna Patel, Monika Gandhi, Pinal Trivedi, Maharshi Pandya, Nidhi Patel, Nitin Savaliya, Raghawendra Kumar, Dinesh Kumar, Zuber Saiyed, Komal Patel, Labdhi Pandya, Afzal Ansari, Nikha Trivedi, R D Dixit, A M Kadri, Harsh Bakshi, Chaitanya Joshi, Madhvi Joshi
EPI_ISL_586550	CCC,Veraval	Gujarat Biotechnology Research Centre	Jitendra Bamrotia, Apurvasinh Puvar, Janvi Raval, Zarna Patel, Monika Gandhi, Pinal Trivedi, Maharshi Pandya, Nidhi Patel, Nitin Savaliya, Raghawendra Kumar, Dinesh Kumar, Zuber Saiyed, Komal Patel, Labdhi Pandya, Afzal Ansari, Nikha Trivedi, Jignesh Parmar, R D Dixit, A M Kadri, Harsh Bakshi, Chaitanya Joshi, Madhvi Joshi
EPI_ISL_586551	Government Hospital,Veraval	Gujarat Biotechnology Research Centre	Monika Gandhi, Pinal Trivedi, Maharshi Pandya, Nidhi Patel, Nitin Savaliya, Raghawendra Kumar, Dinesh Kumar, Zuber Saiyed, Komal Patel, Labdhi Pandya, Afzal Ansari, Nikha Trivedi, Jignesh Parmar, Jitendra Bamrotia, Apurvasinh Puvar, Janvi Raval, Zarna Patel, R D Dixit, A M Kadri, Harsh Bakshi, Chaitanya Joshi, Madhvi Joshi
EPI_ISL_586552	Government Hospital,Veraval	Gujarat Biotechnology Research Centre	Pinal Trivedi, Maharshi Pandya, Nidhi Patel, Nitin Savaliya, Raghawendra Kumar, Dinesh Kumar, Zuber Saiyed, Komal Patel, Labdhi Pandya, Afzal Ansari, Nikha Trivedi, Jignesh Parmar, Jitendra Bamrotia, Apurvasinh Puvar, Janvi Raval, Zarna Patel, Monika Gandhi, R D Dixit, A M Kadri, Harsh Bakshi, Chaitanya Joshi, Madhvi Joshi

EPI_ISL_586553	Government Hospital,Veraval	Gujarat Biotechnology Research Centre	Maharshi Pandya, Nidhi Patel, Nitin Savaliya, Raghawendra Kumar, Dinesh Kumar, Zuber Saiyed, Komal Patel, Labdhi Pandya, Afzal Ansari, Nikha Trivedi, Jignesh Parmar, Jitendra Bamrotia, Apurvasinh Puvar, Janvi Raval, Zarna Patel, Monika Gandhi, Pinal Trivedi, R D Dixit, A M Kadri, Harsh Bakshi, Chaitanya Joshi, Madhvi Joshi
EPI_ISL_586554	Government Hospital,Veraval	Gujarat Biotechnology Research Centre	Nidhi Patel, Nitin Savaliya, Raghawendra Kumar, Dinesh Kumar, Zuber Saiyed, Komal Patel, Labdhi Pandya, Afzal Ansari, Nikha Trivedi, Jignesh Parmar, Jitendra Bamrotia, Apurvasinh Puvar, Janvi Raval, Zarna Patel, Monika Gandhi, Pinal Trivedi, Maharshi Pandya, R D Dixit, A M Kadri, Harsh Bakshi, Chaitanya Joshi, Madhvi Joshi
EPI_ISL_586555	CCC,Veraval	Gujarat Biotechnology Research Centre	Nitin Savaliya, Raghawendra Kumar, Dinesh Kumar, Zuber Saiyed, Komal Patel, Labdhi Pandya, Afzal Ansari, Nikha Trivedi, Jignesh Parmar, Jitendra Bamrotia, Apurvasinh Puvar, Janvi Raval, Zarna Patel, Monika Gandhi, Pinal Trivedi, Maharshi Pandya, Nidhi Patel, R D Dixit, A M Kadri, Harsh Bakshi, Chaitanya Joshi, Madhvi Joshi
EPI_ISL_586556	CCC,Veraval	Gujarat Biotechnology Research Centre	Raghawendra Kumar, Dinesh Kumar, Zuber Saiyed, Komal Patel, Labdhi Pandya, Afzal Ansari, Nikha Trivedi, Jignesh Parmar, Jitendra Bamrotia, Apurvasinh Puvar, Janvi Raval, Zarna Patel, Monika Gandhi, Pinal Trivedi, Maharshi Pandya, Nidhi Patel, Nitin Savaliya, R D Dixit, A M Kadri, Harsh Bakshi, Chaitanya Joshi, Madhvi Joshi
EPI_ISL_586557	CCC,Veraval	Gujarat Biotechnology Research Centre	Dinesh Kumar, Zuber Saiyed, Komal Patel, Labdhi Pandya, Afzal Ansari, Nikha Trivedi, Jignesh Parmar, Jitendra Bamrotia, Apurvasinh Puvar, Janvi Raval, Zarna Patel, Monika Gandhi, Pinal Trivedi, Maharshi Pandya, Nidhi Patel, Nitin Savaliya, Raghawendra Kumar, R D Dixit, A M Kadri, Harsh Bakshi, Chaitanya Joshi, Madhvi Joshi
EPI_ISL_586558	CCC,Veraval	Gujarat Biotechnology Research Centre	Zuber Saiyed, Komal Patel, Labdhi Pandya, Afzal Ansari, Nikha Trivedi, Jignesh Parmar, Jitendra Bamrotia, Apurvasinh Puvar, Janvi Raval, Zarna Patel, Monika Gandhi, Pinal Trivedi, Maharshi Pandya, Nidhi Patel, Nitin Savaliya, Raghawendra Kumar, Dinesh Kumar, R D Dixit, A M Kadri, Harsh Bakshi, Chaitanya Joshi, Madhvi Joshi
EPI_ISL_586559	CCC,Veraval	Gujarat Biotechnology Research Centre	Komal Patel, Labdhi Pandya, Afzal Ansari, Nikha Trivedi, Jignesh Parmar, Jitendra Bamrotia, Apurvasinh Puvar, Janvi Raval, Zarna Patel, Monika Gandhi, Pinal Trivedi, Maharshi Pandya, Nidhi Patel, Nitin Savaliya, Raghawendra Kumar, Dinesh Kumar, Zuber Saiyed, R D Dixit, A M Kadri, Harsh Bakshi, Chaitanya Joshi, Madhvi Joshi
EPI_ISL_586560	CCC,Veraval	Gujarat Biotechnology Research Centre	Labdhi Pandya, Afzal Ansari, Nikha Trivedi, Jignesh Parmar, Jitendra Bamrotia, Apurvasinh Puvar, Janvi Raval, Zarna Patel, Monika Gandhi, Pinal Trivedi, Maharshi Pandya, Nidhi Patel, Nitin Savaliya, Raghawendra Kumar, Dinesh Kumar, Zuber Saiyed, Komal Patel, R D Dixit, A M Kadri, Harsh Bakshi, Chaitanya Joshi, Madhvi Joshi
EPI_ISL_586561	CCC,Veraval	Gujarat Biotechnology Research Centre	Afzal Ansari, Nikha Trivedi, Jignesh Parmar, Jitendra Bamrotia, Apurvasinh Puvar, Janvi Raval, Zarna Patel, Monika Gandhi, Pinal Trivedi, Maharshi Pandya, Nidhi Patel, Nitin Savaliya, Raghawendra Kumar, Dinesh Kumar, Zuber Saiyed, Komal Patel, Labdhi Pandya, R D Dixit, A M Kadri, Harsh Bakshi, Chaitanya Joshi, Madhvi Joshi
EPI_ISL_586562	GMERS GMERS Medical College and Hospital, Junagadh	Gujarat Biotechnology Research Centre	Nikha Trivedi, Bhavesh Bagda , Ravi Dedaniya, Apurvasinh Puvar, Janvi Raval, Zarna Patel, Monika Gandhi, Pinal Trivedi, Maharshi Pandya, Nidhi Patel, Nitin Savaliya, Raghawendra Kumar, Dinesh Kumar, Zuber Saiyed, Komal Patel, Labdhi Pandya, Afzal Ansari, R D Dixit, A M Kadri, Harsh Bakshi, Chaitanya Joshi, Madhvi Joshi
EPI_ISL_586563	GMERS GMERS Medical College and Hospital, Junagadh	Gujarat Biotechnology Research Centre	Bhavesh Bagda , Ravi Dedaniya, Apurvasinh Puvar, Janvi Raval, Zarna Patel, Monika Gandhi, Pinal Trivedi, Maharshi Pandya, Nidhi Patel, Nitin Savaliya, Raghawendra Kumar, Dinesh Kumar, Zuber Saiyed, Komal Patel, Labdhi Pandya, Afzal Ansari, Nikha Trivedi, R D Dixit, A M Kadri, Harsh Bakshi, Chaitanya Joshi, Madhvi Joshi
EPI_ISL_586564	GMERS GMERS Medical College and Hospital, Junagadh	Gujarat Biotechnology Research Centre	Ravi Dedaniya, Apurvasinh Puvar, Janvi Raval, Zarna Patel, Monika Gandhi, Pinal Trivedi, Maharshi Pandya, Nidhi Patel, Nitin Savaliya, Raghawendra Kumar, Dinesh Kumar, Zuber Saiyed, Komal Patel, Labdhi Pandya, Afzal Ansari, Nikha Trivedi, Bhavesh Bagda , R D Dixit, A M Kadri, Harsh Bakshi, Chaitanya Joshi, Madhvi Joshi
EPI_ISL_586565	GMERS GMERS Medical College and Hospital, Junagadh	Gujarat Biotechnology Research Centre	Apurvasinh Puvar, Janvi Raval, Zarna Patel, Monika Gandhi, Pinal Trivedi, Maharshi Pandya, Nidhi Patel, Nitin Savaliya, Raghawendra Kumar, Dinesh Kumar, Zuber Saiyed, Komal Patel, Labdhi Pandya, Afzal Ansari, Nikha Trivedi, Bhavesh Bagda , Ravi Dedaniya, R D Dixit, A M Kadri, Harsh Bakshi, Chaitanya Joshi, Madhvi Joshi
EPI_ISL_586566	GMERS GMERS Medical College and Hospital, Junagadh	Gujarat Biotechnology Research Centre	Janvi Raval, Zarna Patel, Monika Gandhi, Pinal Trivedi, Maharshi Pandya, Nidhi Patel, Nitin Savaliya, Raghawendra Kumar, Dinesh Kumar, Zuber Saiyed, Komal Patel, Labdhi Pandya, Afzal Ansari, Nikha Trivedi, Bhavesh Bagda , Ravi Dedaniya, Apurvasinh Puvar, R D Dixit, A M Kadri, Harsh Bakshi, Chaitanya Joshi, Madhvi Joshi
EPI_ISL_586567, EPI_ISL_586568	Saikrishna Hospital,Mehsana	Gujarat Biotechnology Research Centre	Zarna Patel, Monika Gandhi, Pinal Trivedi, Maharshi Pandya, Nidhi Patel, Nitin Savaliya, Raghawendra Kumar, Dinesh Kumar, Zuber Saiyed, Komal Patel, Labdhi Pandya, Afzal Ansari, Nikha Trivedi, Harshadbhai Parmar, Apurvasinh Puvar, Janvi Raval, R D Dixit, A M Kadri, Harsh Bakshi, Chaitanya Joshi, Madhvi Joshi
EPI_ISL_586569	GCRI,Ahmedabad	Gujarat Biotechnology Research Centre	Shashank Pandya, Harsha Panchal, Apurvasinh Puvar, Janvi Raval, Zarna Patel, Monika Gandhi, Pinal Trivedi, Maharshi Pandya, Nidhi Patel, Nitin Savaliya, Raghawendra Kumar, Dinesh Kumar, Zuber Saiyed, Komal Patel, Labdhi Pandya, Afzal Ansari, Nikha Trivedi, R D Dixit, A M Kadri, Harsh Bakshi, Chaitanya Joshi, Madhvi Joshi
EPI_ISL_586570	GCRI,Ahmedabad	Gujarat Biotechnology Research Centre	Harsha Panchal, Apurvasinh Puvar, Janvi Raval, Zarna Patel, Monika Gandhi, Pinal Trivedi, Maharshi Pandya, Nidhi Patel, Nitin Savaliya, Raghawendra Kumar, Dinesh Kumar, Zuber Saiyed, Komal Patel, Labdhi Pandya, Afzal Ansari, Nikha Trivedi, Shashank Pandya, R D Dixit, A M Kadri, Harsh Bakshi, Chaitanya Joshi, Madhvi Joshi
EPI_ISL_590689	B.J. Medical College and Civil hospital, Ahmedabad	Gujarat Biotechnology Research Centre	Janvi Raval, Zarna Patel, Monika Gandhi, Pinal Trivedi, Maharshi Pandya, Nidhi Patel, Nitin Savaliya, Raghawendra Kumar, Dinesh Kumar, Zuber Saiyed, Komal Patel, Labdhi Pandya, Afzal Ansari, Nikha Trivedi, Pranay Shah, Kamlesh J Upadhyay, Sanjay Kapadia, Apurvasinh Puvar, R D Dixit, A M Kadri, Harsh Bakshi, Chaitanya Joshi, Madhvi Joshi
EPI_ISL_590690	CCC,Veraval	Gujarat Biotechnology Research Centre	Apurvasinh Puvar, Janvi Raval, Zarna Patel, Monika Gandhi, Pinal Trivedi, Maharshi Pandya, Nidhi Patel, Nitin Savaliya, Raghawendra Kumar, Dinesh Kumar, Zuber Saiyed, Komal Patel, Labdhi Pandya, Afzal Ansari, Nikha Trivedi, Jignesh Parmar, Jitendra Bamrotia, R D Dixit, A M Kadri, Harsh Bakshi, Chaitanya Joshi, Madhvi Joshi
EPI_ISL_590691	Government Hospital,Veraval	Gujarat Biotechnology Research Centre	Janvi Raval, Zarna Patel, Monika Gandhi, Pinal Trivedi, Maharshi Pandya, Nidhi Patel, Nitin Savaliya, Raghawendra Kumar, Dinesh Kumar, Zuber Saiyed, Komal Patel, Labdhi Pandya, Afzal Ansari, Nikha Trivedi, Jignesh Parmar, Jitendra Bamrotia, Apurvasinh Puvar, R D Dixit, A M Kadri, Harsh Bakshi, Chaitanya Joshi, Madhvi Joshi
EPI_ISL_590692	Government Hospital,Veraval	Gujarat Biotechnology Research Centre	Zarna Patel, Monika Gandhi, Pinal Trivedi, Maharshi Pandya, Nidhi Patel, Nitin Savaliya, Raghawendra Kumar, Dinesh Kumar, Zuber Saiyed, Komal Patel, Labdhi Pandya, Afzal Ansari, Nikha Trivedi, Jignesh Parmar, Jitendra Bamrotia, Apurvasinh Puvar, Janvi Raval, R D Dixit, A M Kadri, Harsh Bakshi, Chaitanya Joshi, Madhvi Joshi
EPI_ISL_590693	INMI Lazzaro Spallanzani IRCCS	INMI Lazzaro Spallanzani IRCCS	Martina Rueca, Barbara Bartolini, Cesare E.M. Gruber, Francesco Messina, Emanuela Giombini, Beatrice Valli, Eleonora Lalle, Simone Lanini, Francesco Vairo, Maria R. Capobianchi, Antonino Di Caro
EPI_ISL_590694	INMI Lazzaro Spallanzani IRCCS	INMI Lazzaro Spallanzani IRCCS	Barbara Bartolini, Martina Rueca, Francesco Messina, Cesare E.M. Gruber, Emanuela Giombini, Beatrice Valli, Eleonora Lalle, Simone Lanini, Francesco Vairo, Maria R. Capobianchi, Antonino Di Caro
EPI_ISL_590695	INMI Lazzaro Spallanzani IRCCS	INMI Lazzaro Spallanzani IRCCS	Cesare E.M. Gruber, Francesco Messina, Barbara Bartolini, Martina Rueca, Emanuela Giombini, Beatrice Valli, Eleonora Lalle, Simone Lanini, Francesco Vairo, Antonino Di Caro, Maria R. Capobianchi
EPI_ISL_590696	INMI Lazzaro Spallanzani IRCCS	INMI Lazzaro Spallanzani IRCCS	Cesare E.M. Gruber, Barbara Bartolini, Francesco Messina, Martina Rueca, Emanuela Giombini, Beatrice Valli, Eleonora Lalle, Simone Lanini, Francesco Vairo, Antonino Di Caro, Maria R. Capobianchi
EPI_ISL_590697	INMI Lazzaro Spallanzani IRCCS	INMI Lazzaro Spallanzani IRCCS	Martina Rueca, Cesare E.M. Gruber, Barbara Bartolini, Francesco Messina, Emanuela Giombini, Beatrice Valli, Eleonora Lalle, Simone Lanini, Francesco Vairo, Antonino Di Caro, Maria R. Capobianchi
EPI_ISL_590698	INMI Lazzaro Spallanzani IRCCS	INMI Lazzaro Spallanzani IRCCS	Barbara Bartolini, Francesco Messina, Cesare E.M. Gruber, Martina Rueca, Emanuela Giombini, Beatrice Valli, Eleonora Lalle, Simone Lanini, Francesco

			Vairo, Maria R. Capobianchi, Antonino Di Caro
EPI_ISL_591272, EPI_ISL_591273, EPI_ISL_591274, EPI_ISL_591275, EPI_ISL_591276, EPI_ISL_591277, EPI_ISL_591278, EPI_ISL_591279, EPI_ISL_591280	National Institute for Viral Disease Control and Prevention, China CDC	National Institute for Viral Disease Control and Prevention, China CDC	Huilai Ma, Zhaoguo Wang, Xiang Zhao, Jun Han, Yong Zhang, Hong Wang, Cao Chen, Ji Wang, Jingdong Song, Yao Meng, Yuchao Wu, Zhixiao Chen, Dayan Wang, Ruqin Gao, George F.Gao, Wenbo Xu
EPI_ISL_591471, EPI_ISL_591472, EPI_ISL_591473, EPI_ISL_591474, EPI_ISL_591475, EPI_ISL_591476, EPI_ISL_591477, EPI_ISL_591478, EPI_ISL_591479, EPI_ISL_591480, EPI_ISL_591481, EPI_ISL_591482, EPI_ISL_591483			
see above	Pathogen Genomics Center, National Institute of Infectious Diseases	Pathogen Genomics Center, National Institute of Infectious Diseases	Tsuyoshi Sekizuka, Kentaro Itokawa, Rina Tanaka, Masanori Hashino, Hajime Kamiya, Tomoe Shimada, Makoto Kuroda
EPI_ISL_591540	St.Johns Hospital	Department of Neurovirology, National Institute of Mental Health and Neuroscience (NIMHANS)	Chitra Pattabiraman,Vijayalakshmi Reddy, Harsha PK, Risha Rasheed, Pramada Prasad, Shafeeq S Hameed, Manjunatha Venkataswamy, Anita Desai, Ravi Vasanthapuram
EPI_ISL_593478, EPI_ISL_593479, EPI_ISL_593480, EPI_ISL_593553, EPI_ISL_593554, EPI_ISL_593555, EPI_ISL_593556, EPI_ISL_593557, EPI_ISL_593558	Brigham and Women's Hospital	Jonathan Li Laboratory	Manish C. Choudhary, James Regan, Jonathan Z. Li
EPI_ISL_593596	Swedish Medical Center, Seattle, WA, USA	Institute for Systems Biology	Jason D. Goldman, Kai Wang, Katharina Röltgen, Sandra C. A. Nielsen, Jared C. Roach, Samia N. Naccache, Fan Yang, Oliver F. Wirz, Kathryn E. Yost, Ji-Yeun Lee, Kelly Chun, Terri Wrin,, Christos J. Petropoulos, Inyoul Lee, Shannon Fallen, Paula M. Manner, Julie A. Wallick, Heather A. Algren, RN, Kim M. Murray, Yapeng Su, Jennifer Hadlock, Joshua Jeharajah, William R. Berrington, George P. Pappas, Sonam T. Nyatsatsang, Alexander L. Greninger, Ansuman T. Satpathy, John S. Pauk, Scott D. Boyd, James R. Heath
EPI_ISL_593902	Sentinelles, Limay	National Reference Center for Viruses of Respiratory Infections, Institut Pasteur, Paris	Sylvie Behillil, Fabiana Gambaro, Etienne Simon-Lorière, Vincent Enouf, Maud Vanpeene, Sylvie van der Werf
EPI_ISL_593903	Hospital, Argenteuil	National Reference Center for Viruses of Respiratory Infections, Institut Pasteur, Paris	Sylvie Behillil, Fabiana Gambaro, Etienne Simon-Lorière, Vincent Enouf, Maud Vanpeene, Sylvie van der Werf
EPI_ISL_593904, EPI_ISL_593905, EPI_ISL_593906, EPI_ISL_593907, EPI_ISL_593908, EPI_ISL_593909	Labo Analyses Med, Sarcelles	National Reference Center for Viruses of Respiratory Infections, Institut Pasteur, Paris	Sylvie Behillil, Fabiana Gambaro, Etienne Simon-Lorière, Vincent Enouf, Maud Vanpeene, Sylvie van der Werf
EPI_ISL_593910	Hospital, Boulogne-Billancourt	National Reference Center for Viruses of Respiratory Infections, Institut Pasteur, Paris	Sylvie Behillil, Fabiana Gambaro, Etienne Simon-Lorière, Vincent Enouf, Maud Vanpeene, Sylvie van der Werf
EPI_ISL_593911	Hospital, Le Chesnay Cédex	National Reference Center for Viruses of Respiratory Infections, Institut Pasteur, Paris	Sylvie Behillil, Fabiana Gambaro, Etienne Simon-Lorière, Vincent Enouf, Maud Vanpeene, Sylvie van der Werf
EPI_ISL_593912	Hospital, Marange Silvange	National Reference Center for Viruses of Respiratory Infections, Institut Pasteur, Paris	Sylvie Behillil, Fabiana Gambaro, Etienne Simon-Lorière, Vincent Enouf, Maud Vanpeene, Sylvie van der Werf
EPI_ISL_593913	Hospital, Talange	National Reference Center for Viruses of Respiratory Infections, Institut Pasteur, Paris	Sylvie Behillil, Fabiana Gambaro, Etienne Simon-Lorière, Vincent Enouf, Maud Vanpeene, Sylvie van der Werf
EPI_ISL_593914	Hospital, Metz	National Reference Center for Viruses of Respiratory Infections, Institut Pasteur, Paris	Sylvie Behillil, Fabiana Gambaro, Etienne Simon-Lorière, Vincent Enouf, Maud Vanpeene, Sylvie van der Werf
EPI_ISL_593915	Hospital, Amneville	National Reference Center for Viruses of Respiratory Infections, Institut Pasteur, Paris	Sylvie Behillil, Fabiana Gambaro, Etienne Simon-Lorière, Vincent Enouf, Maud Vanpeene, Sylvie van der Werf
EPI_ISL_593916	Hospital, Sarreguemines	National Reference Center for Viruses of Respiratory Infections, Institut Pasteur, Paris	Sylvie Behillil, Fabiana Gambaro, Etienne Simon-Lorière, Vincent Enouf, Maud Vanpeene, Sylvie van der Werf
EPI_ISL_593917	Hospital, Montigny les Metz	National Reference Center for Viruses of Respiratory Infections, Institut Pasteur, Paris	Sylvie Behillil, Fabiana Gambaro, Etienne Simon-Lorière, Vincent Enouf, Maud Vanpeene, Sylvie van der Werf
EPI_ISL_593918	Hospital, Joeuf	National Reference Center for Viruses of Respiratory Infections, Institut Pasteur, Paris	Sylvie Behillil, Fabiana Gambaro, Etienne Simon-Lorière, Vincent Enouf, Maud Vanpeene, Sylvie van der Werf
EPI_ISL_593919	Hospital, Ottonville	National Reference Center for Viruses of Respiratory Infections, Institut Pasteur, Paris	Sylvie Behillil, Fabiana Gambaro, Etienne Simon-Lorière, Vincent Enouf, Maud Vanpeene, Sylvie van der Werf
EPI_ISL_593920	Hospital, Metz	National Reference Center for Viruses of Respiratory Infections, Institut Pasteur, Paris	Sylvie Behillil, Fabiana Gambaro, Etienne Simon-Lorière, Vincent Enouf, Maud Vanpeene, Sylvie van der Werf
EPI_ISL_593921	Hospital, Fameck	National Reference Center for Viruses of Respiratory Infections, Institut Pasteur, Paris	Sylvie Behillil, Fabiana Gambaro, Etienne Simon-Lorière, Vincent Enouf, Maud Vanpeene, Sylvie van der Werf
EPI_ISL_593922, EPI_ISL_593923	Hospital, Maizières les Metz	National Reference Center for Viruses of Respiratory Infections, Institut Pasteur, Paris	Sylvie Behillil, Fabiana Gambaro, Etienne Simon-Lorière, Vincent Enouf, Maud Vanpeene, Sylvie van der Werf
EPI_ISL_593924, EPI_ISL_593925, EPI_ISL_593926, EPI_ISL_593927, EPI_ISL_593928, EPI_ISL_593929, EPI_ISL_593930, EPI_ISL_593931, EPI_ISL_593932, EPI_ISL_593933	Labo Analyses Med, Puteaux	National Reference Center for Viruses of Respiratory Infections, Institut Pasteur, Paris	Sylvie Behillil, Fabiana Gambaro, Etienne Simon-Lorière, Vincent Enouf, Maud Vanpeene, Sylvie van der Werf
EPI_ISL_593934	Sentinelles, Chanteloup-En-Brie	National Reference Center for Viruses of Respiratory Infections, Institut Pasteur, Paris	Sylvie Behillil, Fabiana Gambaro, Etienne Simon-Lorière, Vincent Enouf, Maud Vanpeene, Sylvie van der Werf
EPI_ISL_593935	Sentinelles, Plessis-Trevise	National Reference Center for Viruses of Respiratory Infections, Institut Pasteur, Paris	Sylvie Behillil, Fabiana Gambaro, Etienne Simon-Lorière, Vincent Enouf, Maud Vanpeene, Sylvie van der Werf
EPI_ISL_593936	Sentinelles, Fondettes	National Reference Center for Viruses of Respiratory Infections, Institut Pasteur, Paris	Sylvie Behillil, Fabiana Gambaro, Etienne Simon-Lorière, Vincent Enouf, Maud Vanpeene, Sylvie van der Werf
EPI_ISL_594118	Centro de Investigaciones, Universidad de Especialidades Espíritu Santo	Institute of Microbiology, Universidad San Francisco de Quito	Derly Andrade, Juan Carlos Fernandez, Belén Prado-Vivar, Sully Márquez, Juan José Guadalupe, Monica Becerra-Wong, Bernardo Gutiérrez, Gabriel Morey, Ruben Armas, Jose Pedro Barberan, Fernando Espinoza, Edith Lopez, Verónica Barragán, Patricio Rojas-Silva, Gabriel Trueba, Michelle Grunauer, Paúl Cárdenas
EPI_ISL_594163, EPI_ISL_594164, EPI_ISL_594165	hopital	National Reference Center for Viruses of Respiratory Infections, Institut Pasteur, Paris	Sylvie Behillil, Fabiana Gambaro, Etienne Simon-Lorière, Vincent Enouf, Maud Vanpeene, Sylvie van der Werf
EPI_ISL_594185, EPI_ISL_594186, EPI_ISL_594187, EPI_ISL_594188	Department of Pathology, School of Medicine, Imam Khomeini Hospital, Tehran University of Medical Sciences	Genetics Research Center, University of Social Welfare and Rehabilitation Sciences	Zohreh Fattahi, Marzieh Mohseni, Khadijeh Jalalvand, Azam Ghaziasadi, Seyedeh elham Mortazavi, Ali Jafarpour, Azar Hadadi, Alireza Abdollahi, Ali Jafarpour, Azam Ghaziasad, Seyedeh elham Mortazavi, Saber Soltani, Reza Najafipour, Kimia Kahrizi, Seyed Mohammad Jazayeri, Hossein Najmabadi
EPI_ISL_596228	WHO National Influenza Centre Russian Federation	WHO National Influenza Centre Russian Federation	Andrey Komissarov, Artem Fadeev, Anna Ivanova, Kseniya Komissarova, Dmitry Bazhenov, Daria Danilenko, Ksenia Safina, Elena Nabieva, Georgii Bazykin, Dmitry Lioznov

EPI_ISL_596233, EPI_ISL_596234	WHO National Influenza Centre Russian Federation	WHO National Influenza Centre Russian Federation	Andrey Komissarov, Artem Fadeev, Anna Ivanova, Kseniya Komissarova, Dmitry Bazhenov, Daria Danilenko
EPI_ISL_596264, EPI_ISL_596265	WHO National Influenza Centre Russian Federation	WHO National Influenza Centre Russian Federation	Andrey Komissarov, Artem Fadeev, Anna Ivanova, Mariia Sergeeva, Kseniya Komissarova, Dmitry Bazhenov, Daria Danilenko
EPI_ISL_596266, EPI_ISL_596267	WHO National Influenza Centre Russian Federation	WHO National Influenza Centre Russian Federation	Andrey Komissarov, Artem Fadeev, Anna Ivanova, Kseniya Komissarova, Dmitry Bazhenov, Daria Danilenko
EPI_ISL_596449	Institute for Medical Research, Infectious Disease Research Centre, National Institutes of Health, Ministry of Health Malaysia	Institute for Medical Research, Infectious Disease Research Centre, National Institutes of Health, Ministry of Health Malaysia	Suppiah J, Kamel K, Mohd-Zawawi Z, Thayan R
EPI_ISL_596451, EPI_ISL_596452	Department of Pathology, School of Medicine, Imam Khomeini Hospital, Tehran University of Medical Sciences	Genetics Research Center, University of Social Welfare and Rehabilitation Sciences	Zohreh Fattahi, Marzieh Mohseni, Khadijeh Jalalvand, Azam Ghaziasadi, Seyedeh elham Mortazavi, Ali Jafarpour, Azar Hadadi, Alireza Abdollahi, Ali Jafarpour, Azam Ghaziasad, Seyedeh elham Mortazavi, Saber Soltani, Reza Najafipour, Kimia Kahrizi, Seyed Mohammad Jazayeri, Hossein Najmabadi
EPI_ISL_596453	Booali laboratory, Qom, Iran. Department of Virology, School of Public Health, Tehran University of Medical Sciences, Tehran, Iran.	Genetics Research Center, University of Social Welfare and Rehabilitation Sciences	Zohreh Fattahi, Marzieh Mohseni, Khadijeh Jalalvand, Azam Ghaziasadi, Seyedeh elham Mortazavi, Ali Jafarpour, Mohammad Khazeni, Seyed Amir Momeni, Kimia Kahrizi, Seyed Mohammad Jazayeri, Hossein Najmbadi
EPI_ISL_596454	Infectious Disease and Tropical Medicine Research Center, Resistant Tuberculosis Institute, Zahedan University of Medical Sciences, Zahedan, Iran.	Genetics Research Center, University of Social Welfare and Rehabilitation Sciences	Zohreh Fattahi, Marzieh Mohseni, Khadijeh Jalalvand, Azam Ghaziasadi, Seyedeh elham Mortazavi, Ali Jafarpour, Ebrahim Kord, Seyed Mohammad Hashemi-Shahri, Kimia Kahrizi, Seyed Mohammad Jazayeri, Hossein Najmabadi
EPI_ISL_596455	Department of Pathology, School of Medicine, Imam Khomeini Hospital, Tehran University of Medical Sciences	Genetics Research Center, University of Social Welfare and Rehabilitation Sciences	Zohreh Fattahi, Marzieh Mohseni, Khadijeh Jalalvand, Azam Ghaziasadi, Seyedeh elham Mortazavi, Ali Jafarpour, Azar Hadadi, Alireza Abdollahi, Ali Jafarpour, Azam Ghaziasad, Seyedeh elham Mortazavi, Saber Soltani, Reza Najafipour, Kimia Kahrizi, Seyed Mohammad Jazayeri, Hossein Najmabadi
EPI_ISL_600429	Institute of Epidemiology Disease Control And Research	Institute for Developing Science and Health Initiatives	Lauren Cowley, Mokibul Hassan Afrad, Sadia Isfat Ara Rahman, Md. Mahfuz-Al-mamun, Firadausi Qadri, Tahmina Shirin
EPI_ISL_602412, EPI_ISL_602413, EPI_ISL_602414, EPI_ISL_602415, EPI_ISL_602416	WHO National Influenza Centre Russian Federation	WHO National Influenza Centre Russian Federation	Andrey Komissarov, Artem Fadeev, Kseniya Komissarova, Anna Ivanova, Dmitry Bazhenov, Daria Danilenko
EPI_ISL_602564	Centre for Dengue Research, Department of Immunology and Molecular Medicine	Centre for Dengue Research	Chandima Jeewandara, Deshni Jayathilaka, Dinuka Ariyaratne, Diyanath Ranasinghe, Laksiri Gomes, Ananda Wijewickrama, Malika Karunaratne, Gathsaurie Neelika Malavige
EPI_ISL_602565	Centre for Dengue Research, Department of Immunology and Molecular Medicine	Centre for Dengue Research, Department of Immunology and Molecular Medicine,	Chandima Jeewandara, Deshni Jayathilaka, Dinuka Ariyaratne, Diyanath Ranasinghe, Laksiri Gomes, Dinuka Guruge, Ruwan Wiajayamuni, Gathsaurie Neelika Malavige
EPI_ISL_602566, EPI_ISL_602567, EPI_ISL_602568, EPI_ISL_602569	Centre for Dengue Research, Department of Immunology and Molecular Medicine	Centre for Dengue Research, Department of Immunology and Molecular Medicine	Chandima Jeewandara, Deshni Jayathilaka, Dinuka Ariyaratne, Laksiri Gomes, Diyanath Ranasinghe, Gathsaurie Neelika Malavige
EPI_ISL_602570, EPI_ISL_602571	Centre for Dengue Research, Department of Immunology and Molecular Medicine	Centre for Dengue Research, Department of Immunology and Molecular Medicine	Chandima Jeewandara, Deshni Jayathilaka, Dinuka Ariyaratne, Laksiri Gomes, Diyanath Ranasinghe, Ananda Wijewickrama, Malika Karunaratne, Gathsaurie Neelika Malavige
EPI_ISL_602572, EPI_ISL_602573	Centre for Dengue Research, Department of Immunology and Molecular Medicine	Centre for Dengue Research, Department of Immunology and Molecular Medicine	Chandima Jeewandara, Deshni Jayathilaka, Dinuka Ariyaratne, Diyanath Ranasinghe, Laksiri Gomes, Dinuka Guruge, Ruwan Wiajayamuni, Gathsaurie Neelika Malavige
EPI_ISL_602575, EPI_ISL_602576	Centre for Dengue Research, Department of Immunology and Molecular Medicine	Centre for Dengue Research, Department of Immunology and Molecular Medicine	Chandima Jeewandara, Deshni Jayathilaka, Dinuka Ariyaratne, Laksiri Gomes, Diyanath Ranasinghe, Gathsaurie Neelika Malavige
EPI_ISL_602622	AHRI-Sigal	KRISP, KZN Research Innovation and Sequencing Platform	Gazy I, Sigla, Karim F, Cele S, Giandhari J, Pillay S, Tegally H, Wilkinson E, de Oliveira T
EPI_ISL_602623, EPI_ISL_602624, EPI_ISL_602625, EPI_ISL_602626, EPI_ISL_602627, EPI_ISL_602628, EPI_ISL_602629, EPI_ISL_602630, EPI_ISL_602631	AHRI-Sigal	KRISP, KZN Research Innovation and Sequencing Platform	Gazy I, Sigl A, Karim F, Cele S, Giandhari J, Pillay S, Tegally H, Wilkinson E, de Oliveira T
EPI_ISL_602632, EPI_ISL_602633, EPI_ISL_602634, EPI_ISL_602635, EPI_ISL_602636, EPI_ISL_602637, EPI_ISL_602638, EPI_ISL_602639, EPI_ISL_602640, EPI_ISL_602641, EPI_ISL_602642, EPI_ISL_602643, EPI_ISL_602644, EPI_ISL_602645, EPI_ISL_602646, EPI_ISL_602647, EPI_ISL_602648, EPI_ISL_602649, EPI_ISL_602650, EPI_ISL_602651, EPI_ISL_602652, EPI_ISL_602653, EPI_ISL_602654, EPI_ISL_602655, EPI_ISL_602656, EPI_ISL_602657, EPI_ISL_602658, EPI_ISL_602659, EPI_ISL_602660, EPI_ISL_602661, EPI_ISL_602662, EPI_ISL_602663, EPI_ISL_602664, EPI_ISL_602665, EPI_ISL_602666, EPI_ISL_602667, EPI_ISL_602668, EPI_ISL_602669, EPI_ISL_602670, EPI_ISL_602671, EPI_ISL_602672, EPI_ISL_602673, EPI_ISL_602674, EPI_ISL_602675, EPI_ISL_602676, EPI_ISL_602677, EPI_ISL_602678, EPI_ISL_602679, EPI_ISL_602680, EPI_ISL_602681, EPI_ISL_602682, EPI_ISL_602683, EPI_ISL_602684, EPI_ISL_602685, EPI_ISL_602686, EPI_ISL_602687, EPI_ISL_602688, EPI_ISL_602689, EPI_ISL_602690, EPI_ISL_602691, EPI_ISL_602692, EPI_ISL_602693, EPI_ISL_602694, EPI_ISL_602695, EPI_ISL_602696, EPI_ISL_602697, EPI_ISL_602698, EPI_ISL_602699, EPI_ISL_602700, EPI_ISL_602701, EPI_ISL_602702, EPI_ISL_602703, EPI_ISL_602704, EPI_ISL_602705, EPI_ISL_602706, EPI_ISL_602707, EPI_ISL_602708, EPI_ISL_602709, EPI_ISL_602710, EPI_ISL_602711, EPI_ISL_602712, EPI_ISL_602713, EPI_ISL_602714, EPI_ISL_602715, EPI_ISL_602716, EPI_ISL_602717, EPI_ISL_602718, EPI_ISL_602719, EPI_ISL_602720, EPI_ISL_602721, EPI_ISL_602722, EPI_ISL_602723, EPI_ISL_602724, EPI_ISL_602725, EPI_ISL_602726, EPI_ISL_602727, EPI_ISL_602728, EPI_ISL_602729, EPI_ISL_602730, EPI_ISL_602731, EPI_ISL_602732, EPI_ISL_602733, EPI_ISL_602734, EPI_ISL_602735, EPI_ISL_602736, EPI_ISL_602737, EPI_ISL_602738, EPI_ISL_602739, EPI_ISL_602740, EPI_ISL_602741, EPI_ISL_602742, EPI_ISL_602743, EPI_ISL_602744, EPI_ISL_602745, EPI_ISL_602746, EPI_ISL_602747, EPI_ISL_602748, EPI_ISL_602749, EPI_ISL_602750, EPI_ISL_602751, EPI_ISL_602752, EPI_ISL_602753, EPI_ISL_602754, EPI_ISL_602755, EPI_ISL_602756, EPI_ISL_602757, EPI_ISL_602758, EPI_ISL_602759, EPI_ISL_602760, EPI_ISL_602761, EPI_ISL_602762, EPI_ISL_602763, EPI_ISL_602764, EPI_ISL_602765, EPI_ISL_602766, EPI_ISL_602767, EPI_ISL_602768, EPI_ISL_602769, EPI_ISL_602770, EPI_ISL_602771, EPI_ISL_602772, EPI_ISL_602773, EPI_ISL_602774, EPI_ISL_602775, EPI_ISL_602776, EPI_ISL_602777, EPI_ISL_602778, EPI_ISL_602779, EPI_ISL_602780, EPI_ISL_602781, EPI_ISL_602782, EPI_ISL_602783, EPI_ISL_602784, EPI_ISL_602785, EPI_ISL_602786, EPI_ISL_602787, EPI_ISL_602788, EPI_ISL_602789, EPI_ISL_602790, EPI_ISL_602791, EPI_ISL_602792, EPI_ISL_602793, EPI_ISL_602794, EPI_ISL_602795, EPI_ISL_602796, EPI_ISL_602797, EPI_ISL_602798, EPI_ISL_602799, EPI_ISL_602800, EPI_ISL_602801, EPI_ISL_602802, EPI_ISL_602803, EPI_ISL_602804, EPI_ISL_602805, EPI_ISL_602806, EPI_ISL_602807, EPI_ISL_602808, EPI_ISL_602809, EPI_ISL_602810, EPI_ISL_602811, EPI_ISL_602812, EPI_ISL_602813, EPI_ISL_602814, EPI_ISL_602815, EPI_ISL_602816, EPI_ISL_602817, EPI_ISL_602818, EPI_ISL_602819, EPI_ISL_602820, EPI_ISL_602821, EPI_ISL_602822, EPI_ISL_602823, EPI_ISL_602824, EPI_ISL_602825, EPI_ISL_602826, EPI_ISL_602827, EPI_ISL_602828, EPI_ISL_602829, EPI_ISL_602830, EPI_ISL_602831, EPI_ISL_602832, EPI_ISL_602833, EPI_ISL_602834, EPI_ISL_602835, EPI_ISL_602836, EPI_ISL_602837, EPI_ISL_602838, EPI_ISL_602839, EPI_ISL_602840, EPI_ISL_602841, EPI_ISL_602842, EPI_ISL_602843, EPI_ISL_602844, EPI_ISL_602845, EPI_ISL_602846, EPI_ISL_602847, EPI_ISL_602848, EPI_ISL_602849, EPI_ISL_602850, EPI_ISL_602851, EPI_ISL_602852, EPI_ISL_602853, EPI_ISL_602854, EPI_ISL_602855, EPI_ISL_602856, EPI_ISL_602857, EPI_ISL_602858, EPI_ISL_602859, EPI_ISL_602860, EPI_ISL_602861, EPI_ISL_602862, EPI_ISL_602863, EPI_ISL_602864, EPI_ISL_602865, EPI_ISL_602866, EPI_ISL_602867, EPI_ISL_602868, EPI_ISL_602869, EPI_ISL_602870, EPI_ISL_602871, EPI_ISL_602872, EPI_ISL_602873, EPI_ISL_602874, EPI_ISL_602875, EPI_ISL_602876, EPI_ISL_602877, EPI_ISL_602878, EPI_ISL_602879, EPI_ISL_602880, EPI_ISL_602881, EPI_ISL_602882, EPI_ISL_602883, EPI_ISL_602884, EPI_ISL_602885, EPI_ISL_602886, EPI_ISL_602887, EPI_ISL_602888, EPI_ISL_602889, EPI_ISL_602890, EPI_ISL_602891, EPI_ISL_602892, EPI_ISL_602893, EPI_ISL_602894, EPI_ISL_602895, EPI_ISL_602896, EPI_ISL_602897, EPI_ISL_602898, EPI_ISL_602899, EPI_ISL_602900, EPI_ISL_602901, EPI_ISL_602902, EPI_ISL_602903, EPI_ISL_602904, EPI_ISL_602905, EPI_ISL_602906, EPI_ISL_602907, EPI_ISL_602908, EPI_ISL_602909, EPI_ISL_602910, EPI_ISL_602911, EPI_ISL_602912, EPI_ISL_602913, EPI_ISL_602914, EPI_ISL_602915, EPI_ISL_602916, EPI_ISL_602917, EPI_ISL_602918, EPI_ISL_602919, EPI_ISL_602920, EPI_ISL_602921, EPI_ISL_602922, EPI_ISL_602923, EPI_ISL_602924, EPI_ISL_602925, EPI_ISL_602926, EPI_ISL_602927, EPI_ISL_602928, EPI_ISL_602929, EPI_ISL_602930			
see above	NHLS-IALCH	KRISP, KZN Research Innovation and Sequencing Platform	Giandhari J, Pillay S, Lessells R, Mdlalose K, York D, Khan S, Tegally H, Wilkinson E, de Oliveira T
EPI_ISL_603021	Pronto Socorro Dr. Conrado Cesarino Nuvolini	Instituto Adolfo Lutz, Interdiciplinary Procedures Center, Strategic Laboratory	Claudio Tavares Sacchi, Claudia Regina Gonçalves, Erica Valessa Ramos Gomes, Karoline Rodrigues Campos
EPI_ISL_603022	Departamento de Vigilância à Saúde	Instituto Adolfo Lutz, Interdiciplinary Procedures Center, Strategic Laboratory	Claudio Tavares Sacchi, Claudia Regina Gonçalves, Erica Valessa Ramos Gomes, Karoline Rodrigues Campos
EPI_ISL_603023	Vigilância em Saúde Visa Sul	Instituto Adolfo Lutz, Interdiciplinary Procedures Center, Strategic Laboratory	Claudio Tavares Sacchi, Claudia Regina Gonçalves, Erica Valessa Ramos Gomes, Karoline Rodrigues Campos
EPI_ISL_603024	Santa Casa de Misericordia de Araçatuba	Instituto Adolfo Lutz, Interdiciplinary Procedures Center, Strategic Laboratory	Claudio Tavares Sacchi, Claudia Regina Gonçalves, Erica Valessa Ramos Gomes, Karoline Rodrigues Campos
EPI_ISL_603025	UPA Central de Caraguatatuba	Instituto Adolfo Lutz, Interdiciplinary Procedures Center, Strategic Laboratory	Claudio Tavares Sacchi, Claudia Regina Gonçalves, Erica Valessa Ramos Gomes, Karoline Rodrigues Campos
EPI_ISL_603026	Santa Casa da Misericórdia de Presidente Prudente	Instituto Adolfo Lutz, Interdiciplinary Procedures Center, Strategic Laboratory	Claudio Tavares Sacchi, Claudia Regina Gonçalves, Erica Valessa Ramos Gomes, Karoline Rodrigues Campos
EPI_ISL_603027	Santa Casa de Misericordia de Araçatuba	Instituto Adolfo Lutz, Interdiciplinary Procedures Center, Strategic Laboratory	Claudio Tavares Sacchi, Claudia Regina Gonçalves, Erica Valessa Ramos Gomes, Karoline Rodrigues Campos
EPI_ISL_603028	Hospital Municipal Santa Ana	Instituto Adolfo Lutz, Interdiciplinary Procedures Center, Strategic Laboratory	Claudio Tavares Sacchi, Claudia Regina Gonçalves, Erica Valessa Ramos Gomes, Karoline Rodrigues Campos
EPI_ISL_603029	Hospital Municipal Mário Gatti	Instituto Adolfo Lutz, Interdiciplinary Procedures Center,	Claudio Tavares Sacchi, Claudia Regina Gonçalves, Erica Valessa Ramos Gomes, Karoline Rodrigues Campos

		Strategic Laboratory	
EPI_ISL_603030	Hospital Domingos Leonardo Ceravolo Presidente Prudente	Instituto Adolfo Lutz, Interdiciplinary Procedures Center, Strategic Laboratory	Claudio Tavares Sacchi, Claudia Regina Gonçalves, Erica Valessa Ramos Gomes, Karoline Rodrigues Campos
EPI_ISL_603031	Santa Casa de Presidente Epitácio	Instituto Adolfo Lutz, Interdiciplinary Procedures Center, Strategic Laboratory	Claudio Tavares Sacchi, Claudia Regina Gonçalves, Erica Valessa Ramos Gomes, Karoline Rodrigues Campos
EPI_ISL_603032	Santa Casa da Misericórdia de Presidente Prudente	Instituto Adolfo Lutz, Interdiciplinary Procedures Center, Strategic Laboratory	Claudio Tavares Sacchi, Claudia Regina Gonçalves, Erica Valessa Ramos Gomes, Karoline Rodrigues Campos
EPI_ISL_603033	Vigilancia Epidemiologica de São Bernardo do Campo	Instituto Adolfo Lutz, Interdiciplinary Procedures Center, Strategic Laboratory	Claudio Tavares Sacchi, Claudia Regina Gonçalves, Erica Valessa Ramos Gomes, Karoline Rodrigues Campos
EPI_ISL_603034	Departamento de Vigilância à Saúde	Instituto Adolfo Lutz, Interdiciplinary Procedures Center, Strategic Laboratory	Claudio Tavares Sacchi, Claudia Regina Gonçalves, Erica Valessa Ramos Gomes, Karoline Rodrigues Campos
EPI_ISL_603035	Secretaria Municipal de Saúde	Instituto Adolfo Lutz, Interdiciplinary Procedures Center, Strategic Laboratory	Claudio Tavares Sacchi, Claudia Regina Gonçalves, Erica Valessa Ramos Gomes, Karoline Rodrigues Campos
EPI_ISL_603036	Hospital Santa Ana	Instituto Adolfo Lutz, Interdiciplinary Procedures Center, Strategic Laboratory	Claudio Tavares Sacchi, Claudia Regina Gonçalves, Erica Valessa Ramos Gomes, Karoline Rodrigues Campos
EPI_ISL_603037	Hospital Geral de Pedreira	Instituto Adolfo Lutz, Interdiciplinary Procedures Center, Strategic Laboratory	Claudio Tavares Sacchi, Claudia Regina Gonçalves, Erica Valessa Ramos Gomes, Karoline Rodrigues Campos
EPI_ISL_603038	Santa Casa de Misericordia de Araçatuba	Instituto Adolfo Lutz, Interdiciplinary Procedures Center, Strategic Laboratory	Claudio Tavares Sacchi, Claudia Regina Gonçalves, Erica Valessa Ramos Gomes, Karoline Rodrigues Campos
EPI_ISL_603039	Hospital Municipal Mário Gatti	Instituto Adolfo Lutz, Interdiciplinary Procedures Center, Strategic Laboratory	Claudio Tavares Sacchi, Claudia Regina Gonçalves, Erica Valessa Ramos Gomes, Karoline Rodrigues Campos
EPI_ISL_603137	INMI Lazzaro Spallanzani IRCCS	INMI Lazzaro Spallanzani IRCCS	Cesare E.M. Gruber, Martina Rueca, Barbara Bartolini, Francesco Messina, Emanuela Giombini, Simone Lanini, Antonino Di Caro, Maria R. Capobianchi
EPI_ISL_603138	INMI Lazzaro Spallanzani IRCCS	INMI Lazzaro Spallanzani IRCCS	Martina Rueca, Francesco Messina, Barbara Bartolini, Cesare E.M. Gruber, Emanuela Giombini, Simone Lanini, Antonino Di Caro, Maria R. Capobianchi
EPI_ISL_603139	INMI Lazzaro Spallanzani IRCCS	INMI Lazzaro Spallanzani IRCCS	Martina Rueca, Cesare E.M. Gruber, Barbara Bartolini, Francesco Messina, Emanuela Giombini, Simone Lanini, Antonino Di Caro, Maria R. Capobianchi
EPI_ISL_603140	INMI Lazzaro Spallanzani IRCCS	INMI Lazzaro Spallanzani IRCCS	Martina Rueca, Cesare E.M. Gruber, Francesco Messina, Barbara Bartolini, Emanuela Giombini, Simone Lanini, Antonino Di Caro, Maria R. Capobianchi
EPI_ISL_603141	INMI Lazzaro Spallanzani IRCCS	INMI Lazzaro Spallanzani IRCCS	Francesco Messina, Cesare E.M. Gruber, Martina Rueca, Barbara Bartolini, Emanuela Giombini, Simone Lanini, Maria R. Capobianchi, Antonino Di Caro
EPI_ISL_603142	INMI Lazzaro Spallanzani IRCCS	INMI Lazzaro Spallanzani IRCCS	Barbara Bartolini, Cesare E.M. Gruber, Francesco Messina, Martina Rueca, Simone Lanini, Emanuela Giombini, Maria R. Capobianchi, Antonino Di Caro
EPI_ISL_603143	INMI Lazzaro Spallanzani IRCCS	INMI Lazzaro Spallanzani IRCCS	Martina Rueca, Francesco Messina, Cesare E.M. Gruber, Barbara Bartolini, Emanuela Giombini, Simone Lanini, Antonino Di Caro, Maria R. Capobianchi
EPI_ISL_603144	INMI Lazzaro Spallanzani IRCCS	INMI Lazzaro Spallanzani IRCCS	Cesare E.M. Gruber, Martina Rueca, Francesco Messina, Barbara Bartolini, Emanuela Giombini, Simone Lanini, Maria R. Capobianchi, Antonino Di Caro
EPI_ISL_603145	INMI Lazzaro Spallanzani IRCCS	INMI Lazzaro Spallanzani IRCCS	Francesco Messina, Cesare E.M. Gruber, Martina Rueca, Barbara Bartolini, Emanuela Giombini, Simone Lanini, Antonino Di Caro, Maria R. Capobianchi
EPI_ISL_603146	INMI Lazzaro Spallanzani IRCCS	INMI Lazzaro Spallanzani IRCCS	Cesare E.M. Gruber, Martina Rueca, Barbara Bartolini, Emanuela Giombini, Francesco Messina, Simone Lanini, Maria R. Capobianchi, Antonino Di Caro
EPI_ISL_603147	INMI Lazzaro Spallanzani IRCCS	INMI Lazzaro Spallanzani IRCCS	Cesare E.M. Gruber, Barbara Bartolini, Francesco Messina, Martina Rueca, Simone Lanini, Emanuela Giombini, Antonino Di Caro, Maria R. Capobianchi
EPI_ISL_603148	INMI Lazzaro Spallanzani IRCCS	INMI Lazzaro Spallanzani IRCCS	Cesare E.M. Gruber, Francesco Messina, Martina Rueca, Simone Lanini, Emanuela Giombini, Barbara Bartolini, Maria R. Capobianchi, Antonino Di Caro
EPI_ISL_603149	INMI Lazzaro Spallanzani IRCCS	INMI Lazzaro Spallanzani IRCCS	Francesco Messina, Barbara Bartolini, Cesare E.M. Gruber, Martina Rueca, Simone Lanini, Emanuela Giombini, Patrizia Massarelli, Antonino Di Caro, Maria R. Capobianchi
EPI_ISL_603150	INMI Lazzaro Spallanzani IRCCS	INMI Lazzaro Spallanzani IRCCS	Barbara Bartolini, Francesco Messina, Martina Rueca, Cesare E.M. Gruber, Simone Lanini, Emanuela Giombini, Patrizia Massarelli, Maria R. Capobianchi, Antonino Di Caro
EPI_ISL_603151	INMI Lazzaro Spallanzani IRCCS	INMI Lazzaro Spallanzani IRCCS	Francesco Messina, Barbara Bartolini, Cesare E.M. Gruber, Martina Rueca, Emanuela Giombini, Simone Lanini, Patrizia Massarelli, Maria R. Capobianchi, Antonino Di Caro
EPI_ISL_603152	INMI Lazzaro Spallanzani IRCCS	INMI Lazzaro Spallanzani IRCCS	Barbara Bartolini, Cesare E.M. Gruber, Francesco Messina, Martina Rueca, Emanuela Giombini, Simone Lanini, Patrizia Massarelli, Antonino Di Caro, Maria R. Capobianchi
EPI_ISL_603153	INMI Lazzaro Spallanzani IRCCS	INMI Lazzaro Spallanzani IRCCS	Francesco Messina, Cesare E.M. Gruber, Barbara Bartolini, Simone Lanini, Emanuela Giombini, Martina Rueca, Marcello Meledandri, Maria Letizia Schiavone, Annunziata Tamburro, Antonino Di Caro, Maria R. Capobianchi
EPI_ISL_603154	INMI Lazzaro Spallanzani IRCCS	INMI Lazzaro Spallanzani IRCCS	Martina Rueca, Francesco Messina, Cesare E.M. Gruber, Barbara Bartolini, Emanuela Giombini, Simone Lanini, Antonino Di Caro, Maria R. Capobianchi
EPI_ISL_603155	INMI Lazzaro Spallanzani IRCCS	INMI Lazzaro Spallanzani IRCCS	Martina Rueca, Francesco Messina, Barbara Bartolini, Cesare E.M. Gruber, Simone Lanini, Emanuela Giombini, Maria R. Capobianchi, Antonino Di Caro
EPI_ISL_603156	INMI Lazzaro Spallanzani IRCCS	INMI Lazzaro Spallanzani IRCCS	Barbara Bartolini, Francesco Messina, Cesare E.M. Gruber, Martina Rueca, Emanuela Giombini, Simone Lanini, Maria R. Capobianchi, Antonino Di Caro
EPI_ISL_603157	INMI Lazzaro Spallanzani IRCCS	INMI Lazzaro Spallanzani IRCCS	Francesco Messina, Martina Rueca, Barbara Bartolini, Cesare E.M. Gruber, Simone Lanini, Emanuela Giombini, Fulvia Pimpinelli, Antonino Di Caro, Maria R. Capobianchi
EPI_ISL_603158	INMI Lazzaro Spallanzani IRCCS	INMI Lazzaro Spallanzani IRCCS	Barbara Bartolini, Cesare E.M. Gruber, Francesco Messina, Martina Rueca, Emanuela Giombini, Simone Lanini, Fulvia Pimpinelli, Maria R. Capobianchi, Antonino Di Caro
EPI_ISL_603159	INMI Lazzaro Spallanzani IRCCS	INMI Lazzaro Spallanzani IRCCS	Barbara Bartolini, Francesco Messina, Cesare E.M. Gruber, Martina Rueca, Simone Lanini, Emanuela Giombini, Fulvia Pimpinelli, Maria R. Capobianchi, Antonino Di Caro
EPI_ISL_603160	INMI Lazzaro Spallanzani IRCCS	INMI Lazzaro Spallanzani IRCCS	Barbara Bartolini, Simone Lanini, Emanuela Giombini, Francesco Messina, Cesare E.M. Gruber, Martina Rueca, Fulvia Pimpinelli, Maria R. Capobianchi, Antonino Di Caro
EPI_ISL_603161	INMI Lazzaro Spallanzani IRCCS	INMI Lazzaro Spallanzani IRCCS	Cesare E.M. Gruber, Barbara Bartolini, Francesco Messina, Martina Rueca, Emanuela Giombini, Simone Lanini, Fulvia Pimpinelli, Antonino Di Caro, Maria R. Capobianchi
EPI_ISL_603162	INMI Lazzaro Spallanzani IRCCS	INMI Lazzaro Spallanzani IRCCS	Cesare E.M. Gruber, Francesco Messina, Martina Rueca, Barbara Bartolini, Emanuela Giombini, Simone Lanini, Fulvia Pimpinelli, Antonino Di Caro, Maria R. Capobianchi
EPI_ISL_603163	INMI Lazzaro Spallanzani IRCCS	INMI Lazzaro Spallanzani IRCCS	Cesare E.M. Gruber, Martina Rueca, Emanuela Giombini, Simone Lanini, Francesco Messina, Barbara Bartolini, Fulvia Pimpinelli, Maria R. Capobianchi, Antonino Di Caro
EPI_ISL_603164	INMI Lazzaro Spallanzani IRCCS	INMI Lazzaro Spallanzani IRCCS	Cesare E.M. Gruber, Francesco Messina, Martina Rueca, Barbara Bartolini, Emanuela Giombini, Simone Lanini, Fulvia Pimpinelli, Antonino Di Caro, Maria R. Capobianchi
EPI_ISL_603165	INMI Lazzaro Spallanzani IRCCS	INMI Lazzaro Spallanzani IRCCS	Martina Rueca, Francesco Messina, Barbara Bartolini, Cesare E.M. Gruber, Emanuela Giombini, Simone Lanini, Fulvia Pimpinelli, Maria R. Capobianchi, Antonino Di Caro
EPI_ISL_603166	INMI Lazzaro Spallanzani IRCCS	INMI Lazzaro Spallanzani IRCCS	Martina Rueca, Cesare E.M. Gruber, Barbara Bartolini, Francesco Messina, Simone Lanini, Emanuela Giombini, Fulvia Pimpinelli, Maria R. Capobianchi, Antonino Di Caro
EPI_ISL_603167	INMI Lazzaro Spallanzani IRCCS	INMI Lazzaro Spallanzani IRCCS	Martina Rueca, Cesare E.M. Gruber, Francesco Messina, Barbara Bartolini, Simone Lanini, Emanuela Giombini, Fulvia Pimpinelli, Maria R. Capobianchi,

			Antonino Di Caro
EPI_ISL_603168	INMI Lazzaro Spallanzani IRCCS	INMI Lazzaro Spallanzani IRCCS	Martina Rueca, Barbara Bartolini, Cesare E.M. Gruber, Simone Lanini, Emanuela Giombini, Francesco Messina, Fulvia Pimpinelli, Antonino Di Caro, Maria R. Capobianchi
EPI_ISL_603169	INMI Lazzaro Spallanzani IRCCS	INMI Lazzaro Spallanzani IRCCS	Francesco Messina, Barbara Bartolini, Cesare E.M. Gruber, Martina Rueca, Simone Lanini, Emanuela Giombini, Fulvia Pimpinelli, Maria R. Capobianchi, Antonino Di Caro
EPI_ISL_603170	INMI Lazzaro Spallanzani IRCCS	INMI Lazzaro Spallanzani IRCCS	Martina Rueca, Barbara Bartolini, Cesare E.M. Gruber, Francesco Messina, Simone Lanini, Emanuela Giombini, Maria R. Capobianchi, Antonino Di Caro
EPI_ISL_603171	INMI Lazzaro Spallanzani IRCCS	INMI Lazzaro Spallanzani IRCCS	Barbara Bartolini, Cesare E.M. Gruber, Francesco Messina, Martina Rueca, Emanuela Giombini, Simone Lanini, Mariarosa Gaudio, Antonino Di Caro, Maria R. Capobianchi
EPI_ISL_603172	INMI Lazzaro Spallanzani IRCCS	INMI Lazzaro Spallanzani IRCCS	Cesare E.M. Gruber, Martina Rueca, Barbara Bartolini, Simone Lanini, Francesco Messina, Emanuela Giombini, Mariarosa Gaudio, Antonino Di Caro, Maria R. Capobianchi
EPI_ISL_603173	INMI Lazzaro Spallanzani IRCCS	INMI Lazzaro Spallanzani IRCCS	Barbara Bartolini, Cesare E.M. Gruber, Martina Rueca, Francesco Messina, Simone Lanini, Emanuela Giombini, Giuseppina Cappiello, Maria R. Capobianchi, Antonino Di Caro
EPI_ISL_603174	INMI Lazzaro Spallanzani IRCCS	INMI Lazzaro Spallanzani IRCCS	Francesco Messina, Barbara Bartolini, Martina Rueca, Emanuela Giombini, Simone Lanini, Cesare E.M. Gruber, Mariarosa Gaudio, Maria R. Capobianchi, Antonino Di Caro
EPI_ISL_603175	INMI Lazzaro Spallanzani IRCCS	INMI Lazzaro Spallanzani IRCCS	Barbara Bartolini, Cesare E.M. Gruber, Simone Lanini, Emanuela Giombini, Francesco Messina, Martina Rueca, Mariarosa Gaudio, Antonino Di Caro, Maria R. Capobianchi
EPI_ISL_603176	INMI Lazzaro Spallanzani IRCCS	INMI Lazzaro Spallanzani IRCCS	Cesare E.M. Gruber, Barbara Bartolini, Francesco Messina, Martina Rueca, Simone Lanini, Emanuela Giombini, Antonino Di Caro, Maria R. Capobianchi
EPI_ISL_603221, EPI_ISL_603222	National Institute of Laboratory Medicine and Referral Center	Genomic Research Lab, BCSIR	Abu Sayeed Mohammad Mahmud, Mohammad Samir Uzzaman, Eshrar Osman, Md. Ahashan Habib, Shahina Akter, Tanjina Akhter Banu, Md. Murshed Hasan Sarkar, Barna Goswami, Iffat Jahan, Md. Saddam Hossain, Tasnim Nafisa, Md. Maruf Ahmed Molla, Mahmuda Yeasmin, Asish Kumar Ghosh, A. K. M. Shamsuzzaman, Monira Parveen, Md. Masum Hossain Arif, Md. Salim Khan
EPI_ISL_603223	National Institute of Laboratory Medicine and Referral Center	Genomic Research Lab, BCSIR	Md. Murshed Hasan Sarkar, Abu Sayeed Mohammad Mahmud, Mohammad Samir Uzzaman, Eshrar Osman, Md. Ahashan Habib, Shahina Akter, Tanjina Akhter Banu, Barna Goswami, Iffat Jahan, Md. Saddam Hossain, Tasnim Nafisa, Md. Maruf Ahmed Molla, Mahmuda Yeasmin, Asish Kumar Ghosh, A. K. M. Shamsuzzaman, Monira Parveen, Md. Masum Hossain Arif, Md. Salim Khan
EPI_ISL_603238, EPI_ISL_603239	National Institute of Laboratory Medicine and Referral Center	Genomic Research Lab, BCSIR	Shahina Akter, Abu Sayeed Mohammad Mahmud, Mohammad Samir Uzzaman, Eshrar Osman, Md. Ahashan Habib, Tanjina Akhter Banu, Md. Murshed Hasan Sarkar, Barna Goswami, Iffat Jahan, Md. Saddam Hossain, Tasnim Nafisa, Md. Maruf Ahmed Molla, Mahmuda Yeasmin, Asish Kumar Ghosh, A. K. M. Shamsuzzaman, Monira Parveen, Md. Masum Hossain Arif, Md. Salim Khan
EPI_ISL_603240, EPI_ISL_603241	National Institute of Laboratory Medicine and Referral Center	Genomic Research Lab, BCSIR	Tanjina Akhter Banu, Abu Sayeed Mohammad Mahmud, Mohammad Samir Uzzaman, Eshrar Osman, Md. Ahashan Habib, Shahina Akter, Md. Murshed Hasan Sarkar, Barna Goswami, Iffat Jahan, Md. Saddam Hossain, Tasnim Nafisa, Md. Maruf Ahmed Molla, Mahmuda Yeasmin, Asish Kumar Ghosh, A. K. M. Shamsuzzaman, Monira Parveen, Md. Masum Hossain Arif, Md. Salim Khan
EPI_ISL_605784	AHRI-Sigal	KRISP, KZN Research Innovation and Sequencing Platform	Gazy I, Sigal A, Karim F, Cele S, Giandhari J, Pillay S, Tegally H, Wilkinson E, de Oliveira T
EPI_ISL_605785, EPI_ISL_605786, EPI_ISL_605787, EPI_ISL_605788, EPI_ISL_605789, EPI_ISL_605790	NHLS-IALCH	KRISP, KZN Research Innovation and Sequencing Platform	Giandhari J, Pillay S, Lessells R, Mdlalose K, York D, Khan S, Tegally H, Wilkinson E, de Oliveira T
EPI_ISL_605799, EPI_ISL_605800, EPI_ISL_605801, EPI_ISL_605802, EPI_ISL_605803, EPI_ISL_605804, EPI_ISL_605805, EPI_ISL_605806, EPI_ISL_605807, EPI_ISL_605808, EPI_ISL_605809, EPI_ISL_605810, EPI_ISL_605811, EPI_ISL_605812, EPI_ISL_605813, EPI_ISL_605814, EPI_ISL_605815, EPI_ISL_605816			
see above	Clinical Virology Laboratory, Institute of Liver and Biliary Sciences	ILBS - IGIB	Ekta Gupta, Sheetalnath Rooge, Abhishek Padhi, Reshu Agarwal, Jaswinder Singh Maras, Shridhar Sivasubbu, Vinod Scaria, Shvetank Sharma
EPI_ISL_609994	INMI Lazzaro Spallanzani IRCCS	INMI Lazzaro Spallanzani IRCCS	C.E.M Gruber, F Messina, M Rueca, B Bartolini, E Giombini, MR Capobianchi, A Di Caro
EPI_ISL_609995	INMI Lazzaro Spallanzani IRCCS	INMI Lazzaro Spallanzani IRCCS	E Giombini, C.E.M Gruber, M Rueca, B Bartolini, F Messina, A Di Caro, MR Capobianchi
EPI_ISL_609996	INMI Lazzaro Spallanzani IRCCS	INMI Lazzaro Spallanzani IRCCS	F Messina, M Rueca, B Bartolini, C.E.M Gruber, E Giombini, MR Capobianchi, A Di Caro
EPI_ISL_609997	INMI Lazzaro Spallanzani IRCCS	INMI Lazzaro Spallanzani IRCCS	M Rueca, B Bartolini, C.E.M Gruber, F Messina, E Giombini, A Di Caro, MR Capobianchi
EPI_ISL_610152, EPI_ISL_610153, EPI_ISL_610154	Singapore General Hospital	Department of Microbiology	Nurdyana Abdul Rahman, Kun Lee Lim, Chenhao Li, Sui Sin Goh, Kenneth Xin Long Chan, Kian Sing Chan, Lynette Oon, Kern Rei Chng, Niranjan Nagarajan, Karrie Ko
EPI_ISL_610155	Puskesmas Cangkrep	Genetics Working Group (Pokja Genetik) Faculty of Medicine, Public Health and Nursing Universitas Gadjah Mada (FK-KMK UGM); Disease Investigation Center Wates Ministry of Agriculture Indonesia; Department of Microbiology FK-KMK UGM; Laboratorium Diagnostik Yayasan Tahija World Mosquito Program (WMP) Yogyakarta Center for Tropical Medicine FK-KMK UGM; Integrated Research Center FK-KMK UGM; Department of Computer Science and Electronics FMIPA UGM; Balai Besar Teknik Kesehatan Lingkungan dan Pengendalian Penyakit (BBTKLPP) Yogyakarta	Gunadi, Hendra Wibawa, Marcellus, Mohamad S. Hakim, Edwin W. Daniwijaya, Ludhang P. Rizki, Endah Supriyati, Eggi Arguni, Titik Nuryastuti, Tri Wibawa, Dwi AA Nugrahaningsih, Afiahayati, Siswanto, Kristy Iskandar, Nungki Anggorowati, Irene, Indaryati, Havid Setyawan, Nawang Sukestiningsih, Fadil Fahri, Ririn Enggy Yuliyanti
EPI_ISL_610158	RS Panti Rini	Genetics Working Group (Pokja Genetik) Faculty of Medicine, Public Health and Nursing Universitas Gadjah Mada (FK-KMK UGM); Disease Investigation Center Wates Ministry of Agriculture Indonesia; Department of Microbiology FK-KMK UGM; Laboratorium Diagnostik Yayasan Tahija World Mosquito Program (WMP) Yogyakarta Center for Tropical Medicine FK-KMK UGM; Integrated Research Center FK-KMK UGM; Department of Computer Science and Electronics FMIPA UGM	Gunadi, Hendra Wibawa, Marcellus, Mohamad S. Hakim, Edwin W. Daniwijaya, Ludhang P. Rizki, Endah Supriyati, Eggi Arguni, Titik Nuryastuti, Tri Wibawa, Dwi AA Nugrahaningsih, Afiahayati, Siswanto, Kristy Iskandar, Nungki Anggorowati, V. Noegroho Isti Donodjati, Wawan Triharjanto, Dwiki Afandy, Dyah Ayu Puspitarani
EPI_ISL_610161	RSUD Dr. Tjitrowardojo	Genetics Working Group (Pokja Genetik) Faculty of Medicine, Public Health and Nursing Universitas Gadjah Mada (FK-KMK UGM); Disease Investigation Center Wates Ministry of Agriculture Indonesia; Department of Microbiology FK-KMK UGM; Laboratorium Diagnostik Yayasan Tahija World Mosquito Program (WMP) Yogyakarta Center for Tropical Medicine FK-KMK UGM; Integrated Research Center FK-KMK UGM; Department of Computer Science and Electronics FMIPA UGM; Balai Besar Teknik Kesehatan Lingkungan dan Pengendalian Penyakit (BBTKLPP) Yogyakarta	Gunadi, Hendra Wibawa, Marcellus, Mohamad S. Hakim, Edwin W. Daniwijaya, Ludhang P. Rizki, Endah Supriyati, Eggi Arguni, Titik Nuryastuti, Tri Wibawa, Dwi AA Nugrahaningsih, Afiahayati, Siswanto, Kristy Iskandar, Nungki Anggorowati, Irene, Indaryati, Havid Setyawan, Wuryanto, Kemala Athollah, Desyifa Mursalin
EPI_ISL_610162	RSUD Dr. Tjitrowardojo	Genetics Working Group (Pokja Genetik) Faculty of Medicine,	Gunadi, Hendra Wibawa, Marcellus, Mohamad S. Hakim, Edwin W. Daniwijaya, Ludhang P. Rizki, Endah Supriyati, Eggi Arguni, Titik Nuryastuti, Tri

		Public Health and Nursing Universitas Gadjah Mada (FK-KMK UGM); Disease Investigation Center Wates Ministry of Agriculture Indonesia; Department of Microbiology FK-KMK UGM; Laboratorium Diagnostik Yayasan Tahija World Mosquito Program (WMP) Yogyakarta Center for Tropical Medicine FK-KMK UGM; Integrated Research Center FK-KMK UGM; Department of Computer Science and Electronics FMIPA UGM; Balai Besar Teknik Kesehatan Lingkungan dan Pengendalian Penyakit (BBTKLPP) Yogyakarta	Wibawa, Dwi AA Nugrahaningsih, Afiahayati, Siswanto, Kristy Iskandar, Nungki Anggorowati, Irene, Indaryati, Havid Setyawan, Wuryanto, Susan Simanjaya, Alvin Santoso Kalim
EPI_ISL_610227, EPI_ISL_610228, EPI_ISL_610229, EPI_ISL_610230, EPI_ISL_610231, EPI_ISL_610232, EPI_ISL_610233, EPI_ISL_610234, EPI_ISL_610235, EPI_ISL_610236	Molecular diagnostic laboratory of Federal Budget Institution of Science "Central Research Institute of Epidemiology" of The Federal Service on Customers' Rights Protection and Human Well-being Surveillance	Group of Genomics and Postgenomic Technologies of Central Research Institute of Epidemilology	Samoilov AE, Kaptelova VV, Valdokhina AV, Bulanenko VP, Speranskaya AS, Tivanova EV, Shipulina OY, Akimkin VG
EPI_ISL_610237, EPI_ISL_610238	Molecular diagnostic laboratory of Federal Budget Institution of Science "Central Research Institute of Epidemiology" of The Federal Service on Customers' Rights Protection and Human Well-being Surveillance	Group of Genomics and Postgenomic Technologies of Central Research Institute of Epidemilology	Samoilov AE, Kaptelova VV, Bukharina AY, Speranskaya AS, Tivanova EV, Shipulina OY, Akimkin VG
EPI_ISL_610239, EPI_ISL_610240, EPI_ISL_610241, EPI_ISL_610242, EPI_ISL_610243, EPI_ISL_610244	Molecular diagnostic laboratory of Federal Budget Institution of Science "Central Research Institute of Epidemiology" of The Federal Service on Customers' Rights Protection and Human Well-being Surveillance	Group of Genomics and Postgenomic Technologies of Central Research Institute of Epidemilology	Samoilov AE, Kaptelova VV, Valdokhina AV, Bulanenko VP, Speranskaya AS, Tivanova EV, Shipulina OY, Akimkin VG
EPI_ISL_610249	Virologisches Institut, Universitätsklinikum Erlangen	Virologisches Institut, Universitätsklinikum Erlangen	Armin Ensser, Klaus Korn, Klaus Überla
EPI_ISL_613419, EPI_ISL_613420, EPI_ISL_613421, EPI_ISL_613422, EPI_ISL_613423, EPI_ISL_613424, EPI_ISL_613425, EPI_ISL_613426, EPI_ISL_613429, EPI_ISL_613431, EPI_ISL_613432, EPI_ISL_613433, EPI_ISL_613434, EPI_ISL_613435, EPI_ISL_613436, EPI_ISL_613437, EPI_ISL_613439, EPI_ISL_613440, EPI_ISL_613445, EPI_ISL_613446			
see above	Institut Pasteur de la Guadeloupe	Institut Pasteur de la Guadeloupe	Marion Barbet, Sylvie Behillil, Méline Bizard, Angela Brisebarre, Camille Capel, Etienne Simon-Lorière, Vincent Enouf, Maud Vanpeene, Sylvie van der Werf, Stéphanie Guyomard, Sébastien Breurec, Antoine Talarmin
EPI_ISL_613447, EPI_ISL_613448, EPI_ISL_613449, EPI_ISL_613450, EPI_ISL_613451, EPI_ISL_613452, EPI_ISL_613453, EPI_ISL_613454, EPI_ISL_613455, EPI_ISL_613456	Institut Pasteur de la Guadeloupe	Institut Pasteur de la Guadeloupe	Marion Barbet, Sylvie Behillil, Méline Bizard, Angela Brisebarre, Camille Capel, Etienne Simon-Lorière, Vincent Enouf, Maud Vanpeene, Sylvie van der Werf, Stéphanie Guyomard, Sébastien Breurec, Radjin Steingrover, Cherina Fleming, Antoine Talarmin
EPI_ISL_613457	QUARANTINE CAMP	Hong Kong Department of Health	Alan K.L. Tsang, Peter C.W. Yip, Edman T.K. Lam, Rickjason C.W. Chan, Dominic N.C. Tsang
EPI_ISL_613545, EPI_ISL_613546, EPI_ISL_613547, EPI_ISL_613548, EPI_ISL_613549, EPI_ISL_613550, EPI_ISL_613551, EPI_ISL_613552, EPI_ISL_613553, EPI_ISL_613554, EPI_ISL_613555, EPI_ISL_613556, EPI_ISL_613557, EPI_ISL_613558, EPI_ISL_613559			
see above	CHRU Pontchaillou - Laboratoire de Virologie 2, rue Henri Le Guilloux	National Reference Center for Viruses of Respiratory Infections, Institut Pasteur, Paris	Marion Barbet, Sylvie Behillil, Méline Bizard, Angela Brisebarre, Camille Capel, Etienne Simon-Lorière, Vincent Enouf, Maud Vanpeene, Sylvie van der Werf, Gisèle Lagathu
EPI_ISL_613560	Laboratorio Biologia Molecolare Sars Cov2 - UOC Laboratorio Analisi - Servizio Medicina di Laboratorio, Ospedale "San Francesco" - ATS-ASSL Nuoro Via Mannironi 1, 08100 Nuoro	Laboratorio specialistico UOC Ematologia - Ospedale "San Francesco" - ATS-ASSL Nuoro Nuoro	Piras Giovanna, Fancello Tatiana, Asproni Rosanna, Fiamma Maura, Monne Maria Itria, Toja Alessandro, Sanna Filomena, Floris Anna Rita, Sulis Vincenzo, Palmas Angelo Domenico, Casu Gavino, Lo Maglio Iana, Mameli Giuseppe
EPI_ISL_613563, EPI_ISL_613564, EPI_ISL_613707, EPI_ISL_613708	Laboratory of Molecular Biology, Blood Center of Ribeirão Preto	Laboratory of Molecular Biology, Blood Center of Ribeirão Preto, Faculty of Medicine of Ribeirão Preto, University of São Paulo	Svetoslav N Slavov, Marta Giovanetti, Vagner Fonseca, Elaine V Santos, Evandra S Rodrigues, Talita Adelino, Joilson Xavier, Glauco de Carvalho Pereira, Aparecida Y Yamamoto, Diego Villa Clé, Rodrigo T Calado; Dimas T Covas, Luiz CJ Alcantara, Simone Kashima
EPI_ISL_613709	Laboratory of Molecular Biology, Blood Center of Ribeirão Preto, Faculty of Medicine of Ribeirão Preto, University of São Paulo	Laboratory of Molecular Biology, Blood Center of Ribeirão Preto, Faculty of Medicine of Ribeirão Preto, University of São Paulo	Svetoslav N Slavov, Marta Giovanetti, Vagner Fonseca, Elaine V Santos, Evandra S Rodrigues, Talita Adelino, Joilson Xavier, Glauco de Carvalho Pereira, Aparecida Y Yamamoto, Diego Villa Clé, Rodrigo T Calado; Dimas T Covas, Luiz CJ Alcantara, Simone Kashima
EPI_ISL_613710	Laboratorio Biologia Molecolare Sars Cov2 - UOC Laboratorio Analisi - Servizio Medicina di Laboratorio, Ospedale "San Francesco" - ATS-ASSL Nuoro	Laboratorio specialistico UOC Ematologia - Ospedale "San Francesco" - ATS-ASSL Nuoro	Piras Giovanna, Fancello Tatiana, Asproni Rosanna, Fiamma Maura, Monne Maria Itria, Toja Alessandro, Sanna Filomena, Floris Anna Rita, Sulis Vincenzo, Palmas Angelo Domenico, Casu Gavino, Lo Maglio Iana, Mameli Giuseppe.
EPI_ISL_613711, EPI_ISL_613951, EPI_ISL_613952, EPI_ISL_613954	Laboratory of Molecular Biology, Blood Center of Ribeirão Preto, Faculty of Medicine of Ribeirão Preto, University of São Paulo	Laboratory of Molecular Biology, Blood Center of Ribeirão Preto, Faculty of Medicine of Ribeirão Preto, University of São Paulo	Svetoslav N Slavov, Marta Giovanetti, Vagner Fonseca, Elaine V Santos, Evandra S Rodrigues, Talita Adelino, Joilson Xavier, Glauco de Carvalho Pereira, Aparecida Y Yamamoto, Diego Villa Clé, Rodrigo T Calado; Dimas T Covas, Luiz CJ Alcantara, Simone Kashima
EPI_ISL_613955	Laboratorio Biologia Molecolare Sars Cov2 - UOC Laboratorio Analisi - Servizio Medicina di Laboratorio, Ospedale "San Francesco" - ATS-ASSL Nuoro	Laboratorio specialistico UOC Ematologia - Ospedale "San Francesco" - ATS-ASSL Nuoro	Piras Giovanna, Fancello Tatiana, Asproni Rosanna, Fiamma Maura, Monne Maria Itria, Toja Alessandro, Sanna Filomena, Floris Anna Rita, Sulis Vincenzo, Palmas Angelo Domenico, Casu Gavino, Lo Maglio Iana, Mameli Giuseppe
EPI_ISL_613961, EPI_ISL_613962, EPI_ISL_613963, EPI_ISL_613964, EPI_ISL_613965, EPI_ISL_614011, EPI_ISL_614155, EPI_ISL_614156	Laboratory of Molecular Biology, Blood Center of Ribeirão Preto, Faculty of Medicine of Ribeirão Preto, University of São Paulo	Laboratory of Molecular Biology, Blood Center of Ribeirão Preto, Faculty of Medicine of Ribeirão Preto, University of São Paulo	Svetoslav N Slavov, Marta Giovanetti, Vagner Fonseca, Elaine V Santos, Evandra S Rodrigues, Talita Adelino, Joilson Xavier, Glauco de Carvalho Pereira, Aparecida Y Yamamoto, Diego Villa Clé, Rodrigo T Calado; Dimas T Covas, Luiz CJ Alcantara, Simone Kashima
EPI_ISL_614265, EPI_ISL_614266, EPI_ISL_614267, EPI_ISL_614268, EPI_ISL_614269, EPI_ISL_614270, EPI_ISL_614271, EPI_ISL_614272, EPI_ISL_614273, EPI_ISL_614274, EPI_ISL_614275, EPI_ISL_614276, EPI_ISL_614277, EPI_ISL_614278, EPI_ISL_614279, EPI_ISL_614280			
see above	Eurofins	National Reference Center for Viruses of Respiratory Infections, Institut Pasteur, Paris	Marion Barbet, Sylvie Behillil, Méline Bizard, Angela Brisebarre, Camille Capel, Etienne Simon-Lorière, Vincent Enouf, Maud Vanpeene, Sylvie van der Werf
EPI_ISL_614281	CHRU Pontchaillou - Laboratoire de Virologie 2, rue Henri Le Guilloux	National Reference Center for Viruses of Respiratory Infections, Institut Pasteur, Paris	Marion Barbet, Sylvie Behillil, Méline Bizard, Angela Brisebarre, Camille Capel, Etienne Simon-Lorière, Vincent Enouf, Maud Vanpeene, Sylvie van der Werf, Gisèle Lagathu
EPI_ISL_614282, EPI_ISL_614283, EPI_ISL_614284, EPI_ISL_614285, EPI_ISL_614286, EPI_ISL_614287, EPI_ISL_614288, EPI_ISL_614289, EPI_ISL_614290, EPI_ISL_614291, EPI_ISL_614292, EPI_ISL_614293			
see above	General practitioner	National Reference Center for Viruses of Respiratory Infections, Institut Pasteur, Paris	Marion Barbet, Sylvie Behillil, Méline Bizard, Angela Brisebarre, Camille Capel, Etienne Simon-Lorière, Vincent Enouf, Maud Vanpeene, Sylvie van der Werf
EPI_ISL_614295, EPI_ISL_614296, EPI_ISL_614297, EPI_ISL_614298, EPI_ISL_614299, EPI_ISL_614300, EPI_ISL_614301, EPI_ISL_614302, EPI_ISL_614303, EPI_ISL_614304, EPI_ISL_614305, EPI_ISL_614306, EPI_ISL_614307, EPI_ISL_614308, EPI_ISL_614309, EPI_ISL_614310, EPI_ISL_614311, EPI_ISL_614312, EPI_ISL_614313, EPI_ISL_614314, EPI_ISL_614315, EPI_ISL_614316, EPI_ISL_614317			
see above	Faroese National Reference Laboratory for Fish and Animal Diseases	Faroese National Reference Laboratory for Fish and Animal Diseases	Maria Marjunardóttir Dahl, Petra Elisabeth Petersen, Debes Hammershaimb Christiansen
EPI_ISL_614396, EPI_ISL_614397, EPI_ISL_614398	Laboratorio Biologia Molecolare Sars Cov2 - UOC Laboratorio Analisi - Servizio Medicina di Laboratorio, Ospedale "San Francesco" - ATS-ASSL Nuoro	Laboratorio specialistico UOC Ematologia - Ospedale "San Francesco" - ATS-ASSL Nuoro	Piras Giovanna, Fancello Tatiana, Asproni Rosanna, Fiamma Maura, Monne Maria Itria, Toja Alessandro, Sanna Filomena, Floris Anna Rita, Sulis Vincenzo, Palmas Angelo Domenico, Casu Gavino, Lo Maglio Iana, Mameli Giuseppe

EPI_ISL_614763	Rwanda National Reference Laboratory	Rwanda National Reference Laboratory	Mukantwari Enatha,Stefan Rooke,Aine O'Toole,Umuringa Jeanne d'Arc
EPI_ISL_614889	Laboratorio Biologia Molecolare Sars Cov2 - UOC Laboratorio Analisi - Servizio Medicina di Laboratorio, Ospedale "San Francesco" - ATS-ASSL Nuoro	Laboratorio specialistico UOC Ematologia - Ospedale "San Francesco" - ATS-ASSL Nuoro	Piras Giovanna, Fancello Tatiana, Asproni Rosanna, Fiamma Maura, Monne Maria Itria, Toja Alessandro, Sanna Filomena, Floris Anna Rita, Sulis Vincenzo, Palmas Angelo Domenico, Casu Gavino, Lo Maglio Iana, Mameli Giuseppe
EPI_ISL_614891	Rwanda National Reference Laboratory	Rwanda National Reference Laboratory	Mukantwari Enatha,Stefan Rooke,Aine O'Toole ,Umuringa Jeanne d'Arc
EPI_ISL_614892	Rwanda National Reference Laboratory	Rwanda National Reference Laboratory	Mukantwari Enatha,Stefan Rooke,Aine O'Toole,Umuringa Jeanne d'Arc
EPI_ISL_614980	Rwanda National Reference Laboratory	Rwanda National Reference Laboratory	MUkantwari Enatha,Stefan Rook,Aine O'Toole,UMuringa Jeanne d'Arc
EPI_ISL_615063, EPI_ISL_615064, EPI_ISL_615066, EPI_ISL_615067	Rwanda National Reference Laboratory	Rwanda National Reference Laboratory	Mukantwari Enatha,Stefan Rooke,Aine O'Toole,UMuringa Jeanne d'Arc
EPI_ISL_615069	Rwanda National Reference Laboratory	Rwanda National Laboratory	Mukantwari Enatha,Stefan Rooke,Aine O'Toole,Umuringa Jeanne d'Arc
EPI_ISL_615071	Rwanda National Reference Laboratory	Rwanda National Reference Laboratory	MUkanntwari Eanatha,Stefan Rook,AIne O'Toole,Umuringa Jeanne d'Arc
EPI_ISL_615074	Rwanda National Reference Laboratory	Rwanda National Reference Laboratory	Mukantwari Enatha,Stefan Rooke,AIne O'Toole ,Umuringa Jeanne d'Arc
EPI_ISL_615075	Rwanda National Reference Laboratory	Rwanda National Reference Laboratory	MUkantwari Enatha,Stefan Rooke,Aine O'Toole ,Umuringa Jeanne d'Arc
EPI_ISL_623096, EPI_ISL_623097, EPI_ISL_623103	General practitioner	National Reference Center for Viruses of Respiratory Infections, Institut Pasteur, Paris	Marion Barbet, Sylvie Behillil, Méline Bizard, Angela Brisebarre, Camille Capel, Etienne Simon-Lorière, Vincent Enouf, Maud Vanpeene, Sylvie van der Werf
EPI_ISL_623104, EPI_ISL_623105	Simile Medicina Diagnóstica	Bioinformatics Laboratory / LNCC	Carolina M Voloch, Ronaldo S Francisco Jr, Luiz G P de Almeida, Otavio J. Brustolini, Cynthia C Cardoso, Alexandra L Gerber, Ana Paula de C Guimarães, Diana Mariani, Covid19-UFRJ Workgroup, Luís Cristóvão Pôrto, Renato S Aguiar, Terezinha M P P Castiñeiras, Orlando C. Ferreira, Amilcar Tanuri, Ana Tereza R de Vasconcelos
EPI_ISL_623106, EPI_ISL_623107, EPI_ISL_623108, EPI_ISL_623109, EPI_ISL_623110, EPI_ISL_623111, EPI_ISL_623112, EPI_ISL_623113, EPI_ISL_623114, EPI_ISL_623115, EPI_ISL_623116, EPI_ISL_623117, EPI_ISL_623118, EPI_ISL_623119, EPI_ISL_623120, EPI_ISL_623121, EPI_ISL_623122, EPI_ISL_623123, EPI_ISL_623124, EPI_ISL_623125, EPI_ISL_623126, EPI_ISL_623127, EPI_ISL_623128, EPI_ISL_623129, EPI_ISL_623130, EPI_ISL_623131, EPI_ISL_623132, EPI_ISL_623133, EPI_ISL_623134, EPI_ISL_623135, EPI_ISL_623136, EPI_ISL_623137, EPI_ISL_623138, EPI_ISL_623139, EPI_ISL_623140, EPI_ISL_623141, EPI_ISL_623142, EPI_ISL_623143, EPI_ISL_623144, EPI_ISL_623145, EPI_ISL_623146, EPI_ISL_623147, EPI_ISL_623148, EPI_ISL_623149, EPI_ISL_623150, EPI_ISL_623151, EPI_ISL_623152, EPI_ISL_623153, EPI_ISL_623154, EPI_ISL_623155, EPI_ISL_623156, EPI_ISL_623157, EPI_ISL_623158, EPI_ISL_623159, EPI_ISL_623160, EPI_ISL_623161, EPI_ISL_623162, EPI_ISL_623163, EPI_ISL_623164, EPI_ISL_623165, EPI_ISL_623166, EPI_ISL_623167, EPI_ISL_623168, EPI_ISL_623169			
see above	Laboratorio de Virologia Molecular / UFRJ	Bioinformatics Laboratory / LNCC	Carolina M Voloch, Ronaldo S Francisco Jr, Luiz G P de Almeida, Otavio J. Brustolini, Cynthia C Cardoso, Alexandra L Gerber, Ana Paula de C Guimarães, Diana Mariani, Covid19-UFRJ Workgroup, Luís Cristóvão Pôrto, Renato S Aguiar, Terezinha M P P Castiñeiras, Orlando C. Ferreira, Amilcar Tanuri, Ana Tereza R de Vasconcelos
EPI_ISL_625456	Virology Unit, Institut Pasteur de Madagascar	Virology Unit, Institut Pasteur de Madagascar	Christian Ranaivoson, Cara Brook, Norosoa Razanajatovo, Vida Ahyong, Tsiry Randriambolamanantsoa, Michelle Tan, Vololoniaina Raharinosy, Helisoa Razafimanjato, Cristina M. Tato, Joseph L. DeRisi, Soa Fy Andriamandimby, Jean-Michel Heraud, Philippe Dussart
EPI_ISL_625683	National Reference Laboratory for COVID-19, Pasteur Institute of Iran	National Reference Laboratory for COVID-19, Pasteur Institute of Iran	Zahra Ahmadi, Zahra Fereydouni, Tahmineh Jalali, Mohammad Hassan Pouriayevali, Mahsa Tavakkoli, Marzieh Sajjadi, Setareh Kashanian, Sanam Azad-Manjiri, Heasam Nemati, Tahereh Mohammadi, Kayhan Azadmanesh, Zabihollah Shoja, Sana Eybpoosh, Ahmad Ghasemi, Parastoo Yekta, Sepideh Gerdooei, Farideh Niknam, Mostafa Salehi-Vaziri
EPI_ISL_626566	National Centre for Communication Disease (NCCD) National Influenza Center	National Centre for Communication Disease (NCCD) National Influenza Center	Naranzul Ts,Darmaa B,Bayasgalan N,Ankhbayar S,Tsogtbaatar B,Erden-Ochir Ts,Nymadawa P,Chang-Seon Song
EPI_ISL_626567, EPI_ISL_626568, EPI_ISL_626569	National Centre for Communicable Disease (NCCD) National Influenza Center	National Centre for Communicable Disease (NCCD) National Influenza Center	Naranzul Ts,Darmaa B,Bayasgalan N,Ankhbayar S, Nyamkhuu D, Erden-Ochir Ts,Nymadawa P Chang-Seon Song
EPI_ISL_626570, EPI_ISL_626571, EPI_ISL_626572, EPI_ISL_626573, EPI_ISL_626575, EPI_ISL_626577, EPI_ISL_626578, EPI_ISL_626579, EPI_ISL_626580, EPI_ISL_626581, EPI_ISL_626582, EPI_ISL_626583, EPI_ISL_626585, EPI_ISL_626586, EPI_ISL_626587, EPI_ISL_626588, EPI_ISL_626590, EPI_ISL_626591, EPI_ISL_626592, EPI_ISL_626593, EPI_ISL_626594, EPI_ISL_626595, EPI_ISL_626596, EPI_ISL_626597, EPI_ISL_626598, EPI_ISL_626599, EPI_ISL_626600, EPI_ISL_626601, EPI_ISL_626602, EPI_ISL_626603, EPI_ISL_626604, EPI_ISL_626605, EPI_ISL_626606, EPI_ISL_626608, EPI_ISL_626609, EPI_ISL_626612, EPI_ISL_626613, EPI_ISL_626614, EPI_ISL_626615, EPI_ISL_626616, EPI_ISL_626617, EPI_ISL_626618, EPI_ISL_626619, EPI_ISL_626620, EPI_ISL_626621, EPI_ISL_626623, EPI_ISL_626624, EPI_ISL_626625			
see above	The National Institute of Public Health	State Veterinary Institute Prague	Nagy,A;Jirincova,H;Novakova,L;Trnka,D;Vecerova,J
EPI_ISL_626626	National Centre for Communicable Disease (NCCD) National Influenza Center	National Centre for Communicable Disease (NCCD) National Influenza Center	Naranzul Ts,Darmaa B,Bayasgalan N,Ankhbayar S, Nyamkhuu D, Erden-Ochir Ts,Nymadawa P Chang-Seon Song
EPI_ISL_628750, EPI_ISL_628751, EPI_ISL_628752, EPI_ISL_628753, EPI_ISL_628754, EPI_ISL_628755, EPI_ISL_628756, EPI_ISL_628757, EPI_ISL_628759, EPI_ISL_628760, EPI_ISL_628761			
see above	UHAS COVID-19 Lab	UHAS COVID-19 Lab	Kwabena O. Duedu, Jones Gyamfi, Reuben Ayivor-Djanie, John O. Gyapong and the UHAS COVID-19 Lab Team
EPI_ISL_631504	Wyckoff Heights Medical Center	New York City Public Health Laboratory	Jade Wang, et al.
EPI_ISL_631510, EPI_ISL_631511	New York Presbyterian/ Weill Cornell Medical Center	New York City Public Health Laboratory	Jade Wang, et al.
EPI_ISL_631513	Montefiore Medical Center	New York City Public Health Laboratory	Jade Wang, et al.
EPI_ISL_631538	Maimonides Medical Center	New York City Public Health Laboratory	Jade Wang, et al.
EPI_ISL_631542	New York Presbyterian Lower Manhattan	New York City Public Health Laboratory	Jade Wang, et al.
EPI_ISL_631546	Maimonides Medical Center	New York City Public Health Laboratory	Jade Wang, et al.
EPI_ISL_631554	Jamaica Hospital Medical Center	New York City Public Health Laboratory	Jade Wang, et al.
EPI_ISL_631556	NYC HH Jacobi Medical Center	New York City Public Health Laboratory	Jade Wang, et al.
EPI_ISL_631558	NYU Langone Health	New York City Public Health Laboratory	Jade Wang, et al.
EPI_ISL_631561	Montefiore Medical Center	New York City Public Health Laboratory	Jade Wang, et al.
EPI_ISL_631562	Brookdale University Hospital Medical Center	New York City Public Health Laboratory	Jade Wang, et al.
EPI_ISL_631568	New York Presbyterian Queens	New York City Public Health Laboratory	Jade Wang, et al.
EPI_ISL_631570	OCME Office Of Chief Medical Examiner	New York City Public Health Laboratory	Jade Wang, et al.
EPI_ISL_631573	Brookdale University Hospital Medical Center	New York City Public Health Laboratory	Jade Wang, et al.
EPI_ISL_631574	Jamaica Hospital Medical Center	New York City Public Health Laboratory	Jade Wang, et al.
EPI_ISL_631577, EPI_ISL_631584, EPI_ISL_631587	NYC HH Lincoln Medical And Mental Health Center	New York City Public Health Laboratory	Jade Wang, et al.
EPI_ISL_631589	Flushing Hospital Medical Center	New York City Public Health Laboratory	Jade Wang, et al.
EPI_ISL_631591	OCME Office Of Chief Medical Examiner	New York City Public Health Laboratory	Jade Wang, et al.
EPI_ISL_631592, EPI_ISL_631593	Jamaica Hospital Medical Center	New York City Public Health Laboratory	Jade Wang, et al.
EPI_ISL_631594	OCME Office Of Chief Medical Examiner	New York City Public Health Laboratory	Jade Wang, et al.
EPI_ISL_631595	Jamaica Hospital Medical Center	New York City Public Health Laboratory	Jade Wang, et al.
EPI_ISL_631596	James J. Peters VA Medical Center	New York City Public Health Laboratory	Jade Wang, et al.

EPI_ISL_631597	Jamaica Hospital Medical Center	New York City Public Health Laboratory	Jade Wang, et al.
EPI_ISL_631598	OCME Office Of Chief Medical Examiner	New York City Public Health Laboratory	Jade Wang, et al.
EPI_ISL_631602	Flushing Hospital Medical Center	New York City Public Health Laboratory	Jade Wang, et al.
EPI_ISL_631603	NYC HH Lincoln Medical And Mental Health Center	New York City Public Health Laboratory	Jade Wang, et al.
EPI_ISL_631605	OCME Office Of Chief Medical Examiner	New York City Public Health Laboratory	Jade Wang, et al.
EPI_ISL_631606	Flushing Hospital Medical Center	New York City Public Health Laboratory	Jade Wang, et al.
EPI_ISL_631607	NYC HH Lincoln Medical And Mental Health Center	New York City Public Health Laboratory	Jade Wang, et al.
EPI_ISL_631610	Jamaica Hospital Medical Center	New York City Public Health Laboratory	Jade Wang, et al.
EPI_ISL_631611	OCME Office Of Chief Medical Examiner	New York City Public Health Laboratory	Jade Wang, et al.
EPI_ISL_631613, EPI_ISL_631615	Jamaica Hospital Medical Center	New York City Public Health Laboratory	Jade Wang, et al.
EPI_ISL_631616	Flushing Hospital Medical Center	New York City Public Health Laboratory	Jade Wang, et al.
EPI_ISL_631617, EPI_ISL_631618, EPI_ISL_631619	OCME Office Of Chief Medical Examiner	New York City Public Health Laboratory	Jade Wang, et al.
EPI_ISL_631624, EPI_ISL_631625, EPI_ISL_631628, EPI_ISL_631629, EPI_ISL_631633	Richmond University Medical Center	New York City Public Health Laboratory	Jade Wang, et al.
EPI_ISL_631635	OCME Office Of Chief Medical Examiner	New York City Public Health Laboratory	Jade Wang, et al.
EPI_ISL_631637	Jamaica Hospital Medical Center	New York City Public Health Laboratory	Jade Wang, et al.
EPI_ISL_631639	OCME Office Of Chief Medical Examiner	New York City Public Health Laboratory	Jade Wang, et al.
EPI_ISL_631686	NYC HH Lincoln Medical And Mental Health Center	New York City Public Health Laboratory	Jade Wang, et al.
EPI_ISL_631687	OCME Office Of Chief Medical Examiner	New York City Public Health Laboratory	Jade Wang, et al.
EPI_ISL_631689, EPI_ISL_631690	Lincoln Medical Center	New York City Public Health Laboratory	Jade Wang, et al.
EPI_ISL_631691	NYC HH Lincoln Medical And Mental Health Center	New York City Public Health Laboratory	Jade Wang, et al.
EPI_ISL_631694, EPI_ISL_631695	OCME Office Of Chief Medical Examiner	New York City Public Health Laboratory	Jade Wang, et al.
EPI_ISL_631696	Flushing Hospital Medical Center	New York City Public Health Laboratory	Jade Wang, et al.
EPI_ISL_631697, EPI_ISL_631698	Jamaica Hospital Medical Center	New York City Public Health Laboratory	Jade Wang, et al.
EPI_ISL_631699, EPI_ISL_631700, EPI_ISL_631701	Flushing Hospital Medical Center	New York City Public Health Laboratory	Jade Wang, et al.
EPI_ISL_631703, EPI_ISL_631704, EPI_ISL_631705, EPI_ISL_631706	Jamaica Hospital Medical Center	New York City Public Health Laboratory	Jade Wang, et al.
EPI_ISL_631708, EPI_ISL_631710	Flushing Hospital Medical Center	New York City Public Health Laboratory	Jade Wang, et al.
EPI_ISL_631711, EPI_ISL_631712	Jamaica Hospital Medical Center	New York City Public Health Laboratory	Jade Wang, et al.
EPI_ISL_631715	Flushing Hospital Medical Center	New York City Public Health Laboratory	Jade Wang, et al.
EPI_ISL_631716, EPI_ISL_631719, EPI_ISL_631720, EPI_ISL_631723	Jamaica Hospital Medical Center	New York City Public Health Laboratory	Jade Wang, et al.
EPI_ISL_631724	Flushing Hospital Medical Center	New York City Public Health Laboratory	Jade Wang, et al.
EPI_ISL_631726, EPI_ISL_631728, EPI_ISL_631729	Jamaica Hospital Medical Center	New York City Public Health Laboratory	Jade Wang, et al.
EPI_ISL_631732	Flushing Hospital Medical Center	New York City Public Health Laboratory	Jade Wang, et al.
EPI_ISL_631733, EPI_ISL_631734, EPI_ISL_631735, EPI_ISL_631736, EPI_ISL_631740	Jamaica Hospital Medical Center	New York City Public Health Laboratory	Jade Wang, et al.
EPI_ISL_631754	NYC HH Lincoln Medical And Mental Health Center	New York City Public Health Laboratory	Jade Wang, et al.
EPI_ISL_631755, EPI_ISL_631757	Richmond University Medical Center	New York City Public Health Laboratory	Jade Wang, et al.
EPI_ISL_631758, EPI_ISL_631759, EPI_ISL_631760, EPI_ISL_631761, EPI_ISL_631762	OCME Office Of Chief Medical Examiner	New York City Public Health Laboratory	Jade Wang, et al.
EPI_ISL_631767, EPI_ISL_631768	Jamaica Hospital Medical Center	New York City Public Health Laboratory	Jade Wang, et al.
EPI_ISL_631769, EPI_ISL_631770	Flushing Hospital Medical Center	New York City Public Health Laboratory	Jade Wang, et al.
EPI_ISL_631772	Jamaica Hospital Medical Center	New York City Public Health Laboratory	Jade Wang, et al.
EPI_ISL_631773, EPI_ISL_631774	Flushing Hospital Medical Center	New York City Public Health Laboratory	Jade Wang, et al.
EPI_ISL_631775, EPI_ISL_631776, EPI_ISL_631777	Jamaica Hospital Medical Center	New York City Public Health Laboratory	Jade Wang, et al.
EPI_ISL_631778, EPI_ISL_631781, EPI_ISL_631782	St Barnabas Hospital	New York City Public Health Laboratory	Jade Wang, et al.
EPI_ISL_631790	Jamaica Hospital Medical Center	New York City Public Health Laboratory	Jade Wang, et al.
EPI_ISL_631797	OCME Office Of Chief Medical Examiner	New York City Public Health Laboratory	Jade Wang, et al.
EPI_ISL_631798	Jamaica Hospital Medical Center	New York City Public Health Laboratory	Jade Wang, et al.
EPI_ISL_631801	Richmond University Medical Center	New York City Public Health Laboratory	Jade Wang, et al.
EPI_ISL_631803, EPI_ISL_631806	Brookdale University Hospital Medical Center	New York City Public Health Laboratory	Jade Wang, et al.
EPI_ISL_631808, EPI_ISL_631811	Wyckoff Heights Medical Center	New York City Public Health Laboratory	Jade Wang, et al.
EPI_ISL_631812, EPI_ISL_631813	Jamaica Hospital Medical Center	New York City Public Health Laboratory	Jade Wang, et al.
EPI_ISL_631814, EPI_ISL_631815,	Richmond University Medical Center	New York City Public Health Laboratory	Jade Wang, et al.

EPI_ISL_631821			
EPI_ISL_631829	Brookdale University Hospital Medical Center	New York City Public Health Laboratory	Jade Wang, et al.
EPI_ISL_631831	VA NY Harbor Healthcare System	New York City Public Health Laboratory	Jade Wang, et al.
EPI_ISL_631835, EPI_ISL_631836	Jamaica Hospital Medical Center	New York City Public Health Laboratory	Jade Wang, et al.
EPI_ISL_631840, EPI_ISL_631841, EPI_ISL_631845	OCME Office Of Chief Medical Examiner	New York City Public Health Laboratory	Jade Wang, et al.
EPI_ISL_631850	Jamaica Hospital Medical Center	New York City Public Health Laboratory	Jade Wang, et al.
EPI_ISL_631856	St Barnabas Hospital	New York City Public Health Laboratory	Jade Wang, et al.
EPI_ISL_631866, EPI_ISL_631867	Jamaica Hospital Medical Center	New York City Public Health Laboratory	Jade Wang, et al.
EPI_ISL_631868	Richmond University Medical Center	New York City Public Health Laboratory	Jade Wang, et al.
EPI_ISL_632310	1-Laboratory of Microbiology, National Reference Lab, Charles Nicolle Hospital; 2-University of Tunis ElManar, Faculty of Medicine of Tunis, LR99ES09, Tunis, Tunisia	1-Clinical and Experimental Pharmacology Lab, LR16SP02, National Center of Pharmacovigilance, University of Tunis El Manar, Tunis, Tunisia. 2-Neurodegenerative diseases and psychiatric troubles, LR18SP03, Razi Hospital, University of Tunis El Manar, Tunis, Tunisia. 3- Ministry of Health, National Observatory of New and Emerging Diseases, 1006, Tunis, Tunisia	Ilhem Boutiba-Ben Boubaker, Sameh Trabelsi, Nissaf Ben Alaya, Maher Kharrat, Alia Ben Kahla, Jalila Ben Khelil, Salma Abid, Sana Ferjani, Mouna Ben Sassi, Mouna Safer, Imen Mkada, Imen Kacem, Gaies Emna, Soumaya Rammeh, Riadh Daghfous, Riadh Gouider.
EPI_ISL_632312	NU-sjukvården	Clinical microbiology, Sahlgrenska University Hospital	Johan Ringlander, Josefin Olausson, Hedvig Engström Jakobsson, Magnus Lindh
EPI_ISL_632313	NU-sjukvården	Clinical Microbiology, Sahlgrenska University Hospital,	Johan Ringlander, Josefin Olausson, Hedvig Engström Jakobsson, Magnus Lindh
EPI_ISL_632934	Department of Acute Infectious Diseases Control and Prevention,Yunnan Provincial Center for Disease Control and Prevention	Department of Acute Infectious Diseases Control and Prevention,Yunnan Provincial Center for Disease Control and Prevention	Meiling Zhang,Jienan Zhou,Senquan Jia,Xiaonan Zhao,Xiaoqing Fu
EPI_ISL_632935	Department of Acute Infectious Diseases Control and Prevention, Yunnan Provincial Center for Disease Control and Prevention	Department of Acute Infectious Diseases Control and Prevention, Yunnan Provincial Center for Disease Control and Prevention	Meiling Zhang, Jienan Zhou, Senquan Jia, Xiaonan Zhao, Xiaoqing Fu
EPI_ISL_632936	Puskesmas Mlati 1 Sleman	Genetics Working Group (Pokja Genetik) Faculty of Medicine, Public Health and Nursing Universitas Gadjah Mada (FK-KMK UGM); Disease Investigation Center Wates Ministry of Agriculture Indonesia; Department of Microbiology FK-KMK UGM; Laboratorium Diagnostik Yayasan Tahija World Mosquito Program (WMP) Yogyakarta Center for Tropical Medicine FK-KMK UGM; Integrated Research Center FK-KMK UGM; Department of Computer Science and Electronics FMIPA UGM; RSUP Dr. Sardjito	Gunadi, Hendra Wibawa, Marcellus, Mohamad S. Hakim, Edwin W. Daniwijaya, Ludhang P. Rizki, Endah Supriyati, Eggi Arguni, Titik Nuryastuti, Tri Wibawa, Dwi AA Nugrahaningsih, Afiahayati, Siswanto, Kristy Iskandar, Nungki Anggorowati, Ika Trisnawati, Sumardi, Eko Budiono, Bambang Sigit Riyanto, Heni Retnowulan, Munawar Gani, Satria Maulana, Nur Rahmi Ananda, Riat El Khair, Yunika Puspadewi, Osman Sianipar, Umi Solekhah Intansari, Elizabeth Henny Herningtiyas, Ira Puspitawati, Nur Imma Fatimah Harahap, Ernawati, Untung Wirawan , William Widitjiarso
EPI_ISL_632937	RSUD Saptosari Gunung Kidul	Genetics Working Group (Pokja Genetik) Faculty of Medicine, Public Health and Nursing Universitas Gadjah Mada (FK-KMK UGM); Disease Investigation Center Wates Ministry of Agriculture Indonesia; Department of Microbiology FK-KMK UGM; Laboratorium Diagnostik Yayasan Tahija World Mosquito Program (WMP) Yogyakarta Center for Tropical Medicine FK-KMK UGM; Integrated Research Center FK-KMK UGM; Department of Computer Science and Electronics FMIPA UGM; Balai Besar Teknik Kesehatan Lingkungan dan Pengendalian Penyakit (BBTKLPP) Yogyakarta	Gunadi, Hendra Wibawa, Marcellus, Mohamad S. Hakim, Edwin W. Daniwijaya, Ludhang P. Rizki, Endah Supriyati, Eggi Arguni, Titik Nuryastuti, Tri Wibawa, Dwi AA Nugrahaningsih, Afiahayati, Siswanto, Kristy Iskandar, Nungki Anggorowati, Irene, Indaryati, Havid Setyawan, Eko Darmawan, Maria Patricia Inggriani, Audric Kenny Tedja
EPI_ISL_634977	1-Laboratory of Microbiology, National Reference Lab, Charles Nicolle Hospital; 2-University of Tunis ElManar, Faculty of Medicine of Tunis, LR99ES09, Tunis, Tunisia	1-Clinical and Experimental Pharmacology Lab, LR16SP02, National Center of Pharmacovigilance, University of Tunis El Manar, Tunis, Tunisia. 2-Neurodegenerative diseases and psychiatric troubles, LR18SP03, Razi Hospital, University of Tunis El Manar, Tunis, Tunisia. 3- Ministry of Health, National Observatory of New and Emerging Diseases, 1006, Tunis, Tunisia	Ilhem Boutiba-Ben Boubaker, Sameh Trabelsi, Nissaf Ben Alaya, Maher Kharrat, Alia Ben Kahla, Jalila Ben Khelil, Salma Abid, Sana Ferjani, Mouna Ben Sassi, Mouna Safer, Imen Mkada, Imen Kacem, Gaies Emna, Soumaya Rammeh, Riadh Daghfous, Riadh Gouider.
EPI_ISL_634978, EPI_ISL_634979, EPI_ISL_634980, EPI_ISL_634981, EPI_ISL_634982, EPI_ISL_634983, EPI_ISL_634984, EPI_ISL_634985, EPI_ISL_634986, EPI_ISL_634987, EPI_ISL_634988, EPI_ISL_634989, EPI_ISL_634990, EPI_ISL_634991, EPI_ISL_634992, EPI_ISL_634993, EPI_ISL_634994, EPI_ISL_634995, EPI_ISL_634996, EPI_ISL_634997, EPI_ISL_634998, EPI_ISL_634999, EPI_ISL_635000, EPI_ISL_635001, EPI_ISL_635002, EPI_ISL_635003, EPI_ISL_635004, EPI_ISL_635005, EPI_ISL_635006, EPI_ISL_635007, EPI_ISL_635008, EPI_ISL_635009, EPI_ISL_635010, EPI_ISL_635011, EPI_ISL_635012, EPI_ISL_635013, EPI_ISL_635014, EPI_ISL_635015, EPI_ISL_635016, EPI_ISL_635017, EPI_ISL_635018, EPI_ISL_635019, EPI_ISL_635020, EPI_ISL_635021, EPI_ISL_635022, EPI_ISL_635023, EPI_ISL_635024, EPI_ISL_635025, EPI_ISL_635026, EPI_ISL_635027, EPI_ISL_635028, EPI_ISL_635029, EPI_ISL_635030, EPI_ISL_635031, EPI_ISL_635032, EPI_ISL_635033, EPI_ISL_635034, EPI_ISL_635035, EPI_ISL_635036, EPI_ISL_635037, EPI_ISL_635038, EPI_ISL_635039, EPI_ISL_635040, EPI_ISL_635041, EPI_ISL_635042, EPI_ISL_635043, EPI_ISL_635044, EPI_ISL_635045, EPI_ISL_635046, EPI_ISL_635047, EPI_ISL_635048, EPI_ISL_635049, EPI_ISL_635050, EPI_ISL_635051, EPI_ISL_635052, EPI_ISL_635053, EPI_ISL_635054, EPI_ISL_635055, EPI_ISL_635056, EPI_ISL_635057, EPI_ISL_635058			
see above	National Health Laboratory Service - Inkosi Albert Luthuli Central Hospital (NHLS-IALCH)	KRISP, KZN Research Innovation and Sequencing Platform	Giandhari J, Pillay S, Lessells R, Mdlalose K, York D, Khan S, Tegally H, Wilkinson E, de Oliveira T
EPI_ISL_635059, EPI_ISL_635060, EPI_ISL_635061, EPI_ISL_635062	1-Laboratory of Microbiology, National Reference Lab, Charles Nicolle Hospital; 2-University of Tunis ElManar, Faculty of Medicine of Tunis, LR99ES09, Tunis, Tunisia	1-Clinical and Experimental Pharmacology Lab, LR16SP02, National Center of Pharmacovigilance, University of Tunis El Manar, Tunis, Tunisia. 2-Neurodegenerative diseases and psychiatric troubles, LR18SP03, Razi Hospital, University of Tunis El Manar, Tunis, Tunisia. 3- Ministry of Health, National Observatory of New and Emerging Diseases, 1006, Tunis, Tunisia	Ilhem Boutiba-Ben Boubaker, Sameh Trabelsi, Nissaf Ben Alaya, Maher Kharrat, Alia Ben Kahla, Jalila Ben Khelil, Salma Abid, Sana Ferjani, Mouna Ben Sassi, Mouna Safer, Imen Mkada, Imen Kacem, Gaies Emna, Soumaya Rammeh, Riadh Daghfous, Riadh Gouider.
EPI_ISL_636476, EPI_ISL_636477, EPI_ISL_636485	CNR Virus des Infections Respiratoires - France SUD	CNR Virus des Infections Respiratoires - France SUD	Antonin Bal, Gregory Destras, Gwendolyne Burfin, Hadrien Règue, Alexandre Gaymard, Maude Bouscambert-Duchamp, Florence Morfin-Sherpa, Martine Valette, Bruno Lina, Laurence Josset
EPI_ISL_636737, EPI_ISL_636834, EPI_ISL_636835, EPI_ISL_636836, EPI_ISL_636837, EPI_ISL_636838	Laboratório de Imunofarmacologia - Instituto Oswaldo Cruz	Laboratório de Imunofarmacologia - Instituto Oswaldo Cruz	Souza,T.M., Fintelman-Rodrigues,N., De Paula,A.D., Saraiva,F.B., Ferreira,M.A. and Sacramento,C.Q.
EPI_ISL_636960	Pathogen Genomics Lab King Abdullah University of Science and Technology(KAUST)	Pathogen Genomics Lab King Abdullah University of Science and Technology(KAUST)	Sharif Hala, Fadwa Alofi, Sara Mfarrej, Amit Kumar Subudhi, Rahul P Salunke, Fathia Ben Rached, Amanda Ooi, Luke Esau, Afrah Alsomali, Asim Khogeer, Jumana Taha, Abdulaziz Alahmadi, Kahled Algithami, Raeece Naeem, Anwar Hashem, Naif Almontashiri, Arnab Pain
EPI_ISL_636961	Pathogen Genomics Lab King Abdullah University of Science and Technology(KAUST)	Pathogen Genomics Lab King Abdullah University of Science and Technology(KAUST)	Fadwa Alofi, Sharif Hala, Rahul P Salunke, Sara Mfarrej, Amit Kumar Subudhi, Fathia Ben Rached, Amanda, Luke, Afrah Alsomali, Asim Khogeer, Jumana Taha, Abdulaziz Alahmadi, Kahled Algithami, Raeece Naeem, Anwar Hashem, Naif Almontashiri, Arnab Pain

EPI_ISL_636962, EPI_ISL_636963, EPI_ISL_636964	Pathogen Genomics Lab King Abdullah University of Science and Technology(KAUST)	Pathogen Genomics Lab King Abdullah University of Science and Technology(KAUST)	Rahul P Salunke, Sharif Hala, Raeece Naeem, Sara Mfarrej, Amit Kumar Subudhi, Amanda Ooi, Luke Esau, Fadwa Alofi, Fathia Ben Rached, Afrah Alsomali, Asim Khogeer, Ahmad Bakur Mahmoud, Anwar Hashem, Naif Almontashiri, Arnab Pain
EPI_ISL_636965	Pathogen Genomics Lab King Abdullah University of Science and Technology(KAUST)	Pathogen Genomics Lab King Abdullah University of Science and Technology(KAUST)	Raeece Naeem, Rahul P Salunke, Sharif Hala, Sara Mfarrej, Amit Kumar Subudhi, Fadwa Alofi, Fathia Ben Rached, Afrah Alsomali, Asim Khogeer, Ahmad Bakur Mahmoud, Anwar Hashem, Naif Almontashiri, Arnab Pain
EPI_ISL_636966, EPI_ISL_636967, EPI_ISL_636968	Pathogen Genomics Lab King Abdullah University of Science and Technology(KAUST)	Pathogen Genomics Lab King Abdullah University of Science and Technology(KAUST)	Fathia Ben Rached, Raeece Naeem, Sharif Hala, Fadwa Alofi, Rahul P Salunke, Sara Mfarrej, Amit Kumar Subudhi, Afrah Alsomali, Asim Khogeer, Ahmad Bakur Mahmoud, Anwar Hashem, Naif Almontashiri, Arnab Pain
EPI_ISL_636969, EPI_ISL_636970, EPI_ISL_636971	Etlik Veterinary Control Central Research Institute	Etlik Veterinary Control Central Research Institute	Sabri Hacioglu, Ahu Pakdemirli, Dilek Dulger, Erdem Danyer, Ummu Sena Sari, Cevdet Yarali, Ozcan Yildirim
EPI_ISL_636972	Pathogen Genomics Lab King Abdullah University of Science and Technology (KAUST)	Pathogen Genomics Lab King Abdullah University of Science and Technology (KAUST)	Afrah Alsomali, Fathia Ben Rached, Raeece Naeem, Sharif Hala,Rahul P Salunke, Amanda Ooi, Luke Esau, Sara Mfarrej, Amit Kumar Subudhi, Fadwa Alofi, Asim Khogeer, Kahled Algithami, Anwar Hashem, Naif Almontashiri, Arnab Pain
EPI_ISL_636973	Public Health Lab	Public Health Lab	Alwasti, H
EPI_ISL_636975, EPI_ISL_636976, EPI_ISL_636977, EPI_ISL_636978, EPI_ISL_636979	HP Pemba	KRISP, KZN Research Innovation and Sequencing Platform	Ismael N, Giandhari J, Pillay S, Tegally H, Wilkinson E, de Oliveira T, Nadia Sitoe, Paulo Arnaldo, Nedio Mabunda
EPI_ISL_636980	CS Xai Xai	KRISP, KZN Research Innovation and Sequencing Platform	Ismael N, Giandhari J, Pillay S, Tegally H, Wilkinson E, de Oliveira T, Nadia Sitoe, Paulo Arnaldo, Nedio Mabunda
EPI_ISL_636981	City of Chimoio	KRISP, KZN Research Innovation and Sequencing Platform	Ismael N, Giandhari J, Pillay S, Tegally H, Wilkinson E, de Oliveira T, Nadia Sitoe, Paulo Arnaldo, Nedio Mabunda
EPI_ISL_637108, EPI_ISL_637109	Laboratorio Biologia Molecolare Sars Cov2 - UOC Laboratorio Analisi - Servizio Medicina di Laboratorio, Ospedale "San Francesco" - ATS-ASSL Nuoro	Laboratorio specialistico UOC Ematologia - Ospedale "San Francesco" - ATS-ASSL Nuoro	Piras Giovanna, Fancello Tatiana, Asproni Rosanna, Fiamma Maura, Monne Maria Itria, Toja Alessandro, Sanna Filomena, Floris Anna Rita, Sulis Vincenzo, Palmas Angelo Domenico, Casu Gavino, Lo Maglio Iana, Mameli Giuseppe
EPI_ISL_637110, EPI_ISL_637111, EPI_ISL_637112, EPI_ISL_637113	Rafik Hariri University Hospital	Microbial Pathogenomics Lab	Georgi Merhi, Tamara Salloum, Rita Feghali, Sima Tokajian
EPI_ISL_641320, EPI_ISL_641321, EPI_ISL_641322, EPI_ISL_641323, EPI_ISL_641324, EPI_ISL_641325, EPI_ISL_641326, EPI_ISL_641327, EPI_ISL_641328, EPI_ISL_641329, EPI_ISL_641330, EPI_ISL_641331, EPI_ISL_641332, EPI_ISL_641333, EPI_ISL_641334, EPI_ISL_641335, EPI_ISL_641336, EPI_ISL_641337, EPI_ISL_641338, EPI_ISL_641339, EPI_ISL_641340, EPI_ISL_641341, EPI_ISL_641342, EPI_ISL_641343, EPI_ISL_641344, EPI_ISL_641345, EPI_ISL_641346, EPI_ISL_641347, EPI_ISL_641348, EPI_ISL_641349, EPI_ISL_641350, EPI_ISL_641351, EPI_ISL_641352, EPI_ISL_641353, EPI_ISL_641354, EPI_ISL_641355, EPI_ISL_641356, EPI_ISL_641357, EPI_ISL_641358, EPI_ISL_641359, EPI_ISL_641360, EPI_ISL_641361, EPI_ISL_641362, EPI_ISL_641363, EPI_ISL_641364, EPI_ISL_641365, EPI_ISL_641366, EPI_ISL_641367, EPI_ISL_641368, EPI_ISL_641369, EPI_ISL_641370, EPI_ISL_641371, EPI_ISL_641372, EPI_ISL_641373, EPI_ISL_641374, EPI_ISL_641375, EPI_ISL_641376			
see above	Devki Devi Foundation, a unit of Max Healthcare	CSIR Institute of Genomics and Integrative Biology (CSIR-IGIB) / Max	Rajesh Pandey#, Samreen Siddiqui, Pooja Sharma, Bansidhar Tarai, Vivekanand A, Bharathram Uppili, Saruchi Wadhwa, Nishu Tyagi, Mitali Mukerji, Poonam Das, Sujeet Jha, Mohammed Faruq, Vinitaa Jha, Anurag Agrawal
EPI_ISL_644671, EPI_ISL_644672	Institute for Medical Research, Infectious Disease Research Centre, National Institutes of Health, Ministry of Health Malaysia	Institute for Medical Research, Infectious Disease Research Centre, National Institutes of Health, Ministry of Health Malaysia	Suppiah J, Kamel K, Mohd-Zawawi Z, Thayan R
EPI_ISL_644823	Madigan Army Medical Center	U.S. Air Force School of Aerospace Medicine	Emily Parsons, Matthew Timlin, Clarise Starr, Anthony Fries, Ronald Wells, Mattew Studer, Rebecca Sainato
EPI_ISL_648043, EPI_ISL_648044, EPI_ISL_648045, EPI_ISL_648046, EPI_ISL_648047, EPI_ISL_648048, EPI_ISL_648049, EPI_ISL_648050, EPI_ISL_648051, EPI_ISL_648052, EPI_ISL_648053, EPI_ISL_648054, EPI_ISL_648055, EPI_ISL_648056, EPI_ISL_648057, EPI_ISL_648058, EPI_ISL_648059, EPI_ISL_648060, EPI_ISL_648061, EPI_ISL_648062, EPI_ISL_648063, EPI_ISL_648064, EPI_ISL_648065, EPI_ISL_648066, EPI_ISL_648067, EPI_ISL_648068, EPI_ISL_648069, EPI_ISL_648070, EPI_ISL_648071, EPI_ISL_648072, EPI_ISL_648073, EPI_ISL_648074, EPI_ISL_648075, EPI_ISL_648076, EPI_ISL_648077, EPI_ISL_648078, EPI_ISL_648079, EPI_ISL_648080, EPI_ISL_648081, EPI_ISL_648082, EPI_ISL_648083, EPI_ISL_648084, EPI_ISL_648085, EPI_ISL_648086, EPI_ISL_648087, EPI_ISL_648088, EPI_ISL_648089, EPI_ISL_648090, EPI_ISL_648091, EPI_ISL_648092, EPI_ISL_648093, EPI_ISL_648094, EPI_ISL_648095, EPI_ISL_648096, EPI_ISL_648097, EPI_ISL_648098, EPI_ISL_648099, EPI_ISL_648100, EPI_ISL_648101, EPI_ISL_648102, EPI_ISL_648103, EPI_ISL_648104, EPI_ISL_648105, EPI_ISL_648106, EPI_ISL_648107, EPI_ISL_648108, EPI_ISL_648109, EPI_ISL_648110, EPI_ISL_648111, EPI_ISL_648112, EPI_ISL_648113, EPI_ISL_648114, EPI_ISL_648115, EPI_ISL_648116, EPI_ISL_648117, EPI_ISL_648118, EPI_ISL_648119, EPI_ISL_648120, EPI_ISL_648121			
see above	Department of Laboratory Medicine, Tan Tock Seng Hospital	Department of Laboratory Medicine, Tan Tock Seng Hospital	Chen YYC, Zair X, Lim JX, Li C, Tang WY, Maurer-Stroh S, Barkham TMS, Nagarajan N, Sessions OM
EPI_ISL_648124, EPI_ISL_648125, EPI_ISL_648126, EPI_ISL_648127	UHAS COVID-19 Lab	UHAS COVID-19 Lab	Kwabena O. Duedu, Jones Gyamfi, Reuben Ayivor-Djanie, John O. Gyapong and the UHAS COVID-19 Lab Team
EPI_ISL_648217	SILAB	Laboratorio Mixto de Biotecnología Acuática (LMBA)	Joaquín Ezpeleta, Ignacio García Labari, Victoria Posner, Vanina Villanova, Pablo Casal, Sofía Lavista Llanos, Federico Remes Lenicov, Ana Paletta, Flavio Spetale, Agustina Cerri, Silvana Spinelli, Elisa Bolatti, Diego Chouhy, María Re, Gastón Viarengo, Ana Cavatorta, Julian Acosta, Javier Murillo, Laura Angelone, Leandro Ciappina, Pilar Bulacio, Adriana Giri, Silvia Arranz, Elizabeth Tapia
EPI_ISL_648265, EPI_ISL_648267, EPI_ISL_648270	Brigham and Women's Hospital	Jonathan Li laboratory	Jonathan Z. Li, Manish C. Choudhary, Upasana D. Adhikari, George Eng, Douglas S Kwon
EPI_ISL_648610, EPI_ISL_648611, EPI_ISL_648612, EPI_ISL_648613, EPI_ISL_648614, EPI_ISL_648615, EPI_ISL_648616, EPI_ISL_648617, EPI_ISL_648618, EPI_ISL_648619, EPI_ISL_648620, EPI_ISL_648621, EPI_ISL_648622, EPI_ISL_648623, EPI_ISL_648624, EPI_ISL_648625, EPI_ISL_648626, EPI_ISL_648627, EPI_ISL_648628, EPI_ISL_648629, EPI_ISL_648630, EPI_ISL_648631, EPI_ISL_648632, EPI_ISL_648633, EPI_ISL_648634, EPI_ISL_648635, EPI_ISL_648636, EPI_ISL_648637, EPI_ISL_648638, EPI_ISL_648639, EPI_ISL_648640, EPI_ISL_648641, EPI_ISL_648642, EPI_ISL_648643, EPI_ISL_648644, EPI_ISL_648645, EPI_ISL_648646, EPI_ISL_648647, EPI_ISL_648648, EPI_ISL_648649, EPI_ISL_648650, EPI_ISL_648651, EPI_ISL_648652, EPI_ISL_648653, EPI_ISL_648654, EPI_ISL_648655, EPI_ISL_648656, EPI_ISL_648657, EPI_ISL_648658, EPI_ISL_648659, EPI_ISL_648660, EPI_ISL_648661, EPI_ISL_648662, EPI_ISL_648663, EPI_ISL_648664, EPI_ISL_648665, EPI_ISL_648666, EPI_ISL_648667, EPI_ISL_648668, EPI_ISL_648669, EPI_ISL_648670, EPI_ISL_648671, EPI_ISL_648672, EPI_ISL_648673, EPI_ISL_648674, EPI_ISL_648675, EPI_ISL_648676, EPI_ISL_648678, EPI_ISL_648679, EPI_ISL_648680, EPI_ISL_648681, EPI_ISL_648682, EPI_ISL_648683, EPI_ISL_648684, EPI_ISL_648685, EPI_ISL_648686, EPI_ISL_648687, EPI_ISL_648688, EPI_ISL_648689, EPI_ISL_648690, EPI_ISL_648691, EPI_ISL_648692, EPI_ISL_648693, EPI_ISL_648694, EPI_ISL_648695, EPI_ISL_648696, EPI_ISL_648697, EPI_ISL_648698, EPI_ISL_648699, EPI_ISL_648700, EPI_ISL_648701, EPI_ISL_648702, EPI_ISL_648703, EPI_ISL_648704, EPI_ISL_648705, EPI_ISL_648706, EPI_ISL_648707, EPI_ISL_648708, EPI_ISL_648709, EPI_ISL_648710, EPI_ISL_648711, EPI_ISL_648712, EPI_ISL_648713, EPI_ISL_648714, EPI_ISL_648715, EPI_ISL_648716, EPI_ISL_648717, EPI_ISL_648718, EPI_ISL_648719, EPI_ISL_648720, EPI_ISL_648721, EPI_ISL_648722, EPI_ISL_648723, EPI_ISL_648724, EPI_ISL_648725, EPI_ISL_648726, EPI_ISL_648727, EPI_ISL_648728, EPI_ISL_648729, EPI_ISL_648730, EPI_ISL_648731, EPI_ISL_648732, EPI_ISL_648733, EPI_ISL_648734, EPI_ISL_648735, EPI_ISL_648736, EPI_ISL_648737, EPI_ISL_648738, EPI_ISL_648739, EPI_ISL_648740, EPI_ISL_648741, EPI_ISL_648742, EPI_ISL_648743, EPI_ISL_648744, EPI_ISL_648745, EPI_ISL_648746, EPI_ISL_648747, EPI_ISL_648748, EPI_ISL_648749, EPI_ISL_648750, EPI_ISL_648751, EPI_ISL_648752, EPI_ISL_648753, EPI_ISL_648754, EPI_ISL_648755, EPI_ISL_648756, EPI_ISL_648757, EPI_ISL_648758, EPI_ISL_648759, EPI_ISL_648760, EPI_ISL_648761, EPI_ISL_648762, EPI_ISL_648763, EPI_ISL_648764, EPI_ISL_648765, EPI_ISL_648766, EPI_ISL_648767, EPI_ISL_648768, EPI_ISL_648769, EPI_ISL_648770, EPI_ISL_648771, EPI_ISL_648772, EPI_ISL_648773, EPI_ISL_648774, EPI_ISL_648775, EPI_ISL_648776, EPI_ISL_648777, EPI_ISL_648778, EPI_ISL_648779, EPI_ISL_648780, EPI_ISL_648781, EPI_ISL_648782, EPI_ISL_648783, EPI_ISL_648784, EPI_ISL_648785, EPI_ISL_648786, EPI_ISL_648787, EPI_ISL_648788, EPI_ISL_648789, EPI_ISL_648790, EPI_ISL_648791, EPI_ISL_648792, EPI_ISL_648793, EPI_ISL_648794, EPI_ISL_648795, EPI_ISL_648796, EPI_ISL_648797, EPI_ISL_648798, EPI_ISL_648799, EPI_ISL_648800, EPI_ISL_648801, EPI_ISL_648802, EPI_ISL_648803, EPI_ISL_648804, EPI_ISL_648805, EPI_ISL_648806, EPI_ISL_648807, EPI_ISL_648808, EPI_ISL_648809, EPI_ISL_648810, EPI_ISL_648811, EPI_ISL_648812, EPI_ISL_648813, EPI_ISL_648814, EPI_ISL_648815, EPI_ISL_648816, EPI_ISL_648817, EPI_ISL_648818, EPI_ISL_648819, EPI_ISL_648820			
see above	Department of Laboratory Medicine, Tan Tock Seng Hospital	Department of Laboratory Medicine, Tan Tock Seng Hospital	Chen YYC, Zair X, Lim JX, Li C, Tang WY, Maurer-Stroh S, Barkham TMS, Nagarajan N, Sessions OM
EPI_ISL_649154	Queen Astrid Military Hospital	Institute of Tropical Medicine	Philippe Selhorst, Colin Anthony
EPI_ISL_649189, EPI_ISL_649190	Istituto Zooprofilattico Sperimentale della Puglia e della Basilicata	Istituto Zooprofilattico Sperimentale della Puglia e della Basilicata	Parisi A., Bianco A., Capozzi L., Del Sambro L., Manzulli V, Rondinone V., Pace L., Galante D., Cipolletta D.
EPI_ISL_649191	Istituto Zooprofilattico Sperimentale della Puglia e della Basilicata	Istituto Zooprofilattico Sperimentale della Puglia e della Basilicata	Parisi A., Bianco A., Capozzi L., Del Sambro L., Manzulli V, Rondinone V., Pace L., Cipolletta D., Galante D.
EPI_ISL_649938	I.R.C.C.S. "S. De Bellis" - Ente Ospedaliero	Istituto Zooprofilattico Sperimentale della Puglia e della Basilicata	Parisi A., Bianco A., Capozzi L., Del Sambro L., Lippolis A., Notarnicola M., Cipolletta D., Galante D.
EPI_ISL_649939	I.R.C.C.S. "S. De Bellis" - Ente Ospedaliero	Istituto Zooprofilattico Sperimentale della Puglia e della Basilicata	Parisi A., Bianco A., Capozzi L., Del Sambro L., Lippolis A., Notarnicola M., Manzulli V, Rondinone V., Pace L.
EPI_ISL_649940	Istituto Zooprofilattico Sperimentale della Puglia e della Basilicata	Istituto Zooprofilattico Sperimentale della Puglia e della Basilicata	Parisi A., Bianco A., Capozzi L., Del Sambro L., Manzulli V, Rondinone V., Pace L., Cipolletta D., Galante D.
EPI_ISL_653745, EPI_ISL_653746, EPI_ISL_653747, EPI_ISL_653748, EPI_ISL_653749, EPI_ISL_653754, EPI_ISL_653755, EPI_ISL_653756, EPI_ISL_653757, EPI_ISL_653758, EPI_ISL_653759, EPI_ISL_653760, EPI_ISL_653761, EPI_ISL_653762			
see above	Instituto Nacional de Salud, Bogotá, Colombia	Instituto Nacional de Salud, Bogotá, Colombia	Katherine Laiton-Donato, Diego A. Álvarez-Díaz, Carlos Franco-Muñoz, Mauricio Pacheco-Montealegre, Jonathan Reales, Diego Andrés Prada, Jose A. Usme-Ciro, Zulma M. Cucunubá, Christian Julian VillabonaArenas, Liz Villabona-Arenas, Sussy Echeverria, Astrid C. Flórez, Carolina Ferro, Diana Marcela Walteros-Acero, Franklin Prieto, Carlos Andrés Durán, Martha Lucia Ospina Martinez, Marcela Mercado-Reyes
EPI_ISL_653763, EPI_ISL_653764, EPI_ISL_653765, EPI_ISL_653766, EPI_ISL_653767, EPI_ISL_653768, EPI_ISL_653769, EPI_ISL_653770, EPI_ISL_653771, EPI_ISL_653772, EPI_ISL_653773, EPI_ISL_653774, EPI_ISL_653775, EPI_ISL_653776, EPI_ISL_653777, EPI_ISL_653778, EPI_ISL_653779, EPI_ISL_653780,			

EPI_ISL_653781, EPI_ISL_653782, EPI_ISL_653783

see above	I.R.C.C.S. "S. De Bellis" - Ente Ospedaliero	Istituto Zooprofilattico Sperimentale della Puglia e della Basilicata	Parisi A., Bianco A., Capozzi L., Del Sambro L., Lippolis A., Notarnicola M., Manzulli V, Rondinone V., Pace L.
EPI_ISL_653784, EPI_ISL_653785, EPI_ISL_653786	Istituto Zooprofilattico Sperimentale della Puglia e della Basilicata	Istituto Zooprofilattico Sperimentale della Puglia e della Basilicata	Parisi A., Bianco A., Capozzi L., Del Sambro L., Manzulli V, Rondinone V., Pace L., Cipolletta D., Galante D.
EPI_ISL_653787, EPI_ISL_653788, EPI_ISL_653789, EPI_ISL_653790, EPI_ISL_653791, EPI_ISL_653792, EPI_ISL_653793	I.R.C.C.S. "S. De Bellis" - Ente Ospedaliero	Istituto Zooprofilattico Sperimentale della Puglia e della Basilicata	Parisi A., Bianco A., Capozzi L., Del Sambro L., Lippolis A., Notarnicola M., Manzulli V, Rondinone V., Pace L.

EPI_ISL_653794, EPI_ISL_653795, EPI_ISL_653796, EPI_ISL_653797, EPI_ISL_653798, EPI_ISL_653799, EPI_ISL_653800, EPI_ISL_653801, EPI_ISL_653802, EPI_ISL_653803, EPI_ISL_653804, EPI_ISL_653805, EPI_ISL_653806, EPI_ISL_653807, EPI_ISL_653808, EPI_ISL_653809, EPI_ISL_653810, EPI_ISL_653811, EPI_ISL_653812

see above	I.R.C.C.S. "S. De Bellis" - Ente Ospedaliero	Istituto Zooprofilattico Sperimentale della Puglia e della Basilicata	Parisi A., Bianco A., Capozzi L., Del Sambro L., Lippolis A., Notarnicola M., Cipolletta D., Galante D.
EPI_ISL_653813	Istituto Zooprofilattico Sperimentale della Puglia e della Basilicata	Istituto Zooprofilattico Sperimentale della Puglia e della Basilicata	Parisi A., Bianco A., Capozzi L., Del Sambro L., Manzulli V, Rondinone V., Pace L., Cipolletta D., Galante D.
EPI_ISL_653814, EPI_ISL_653815, EPI_ISL_653816, EPI_ISL_653817, EPI_ISL_653818, EPI_ISL_653819, EPI_ISL_653820, EPI_ISL_653821, EPI_ISL_653822, EPI_ISL_653823	I.R.C.C.S. "S. De Bellis" - Ente Ospedaliero	Istituto Zooprofilattico Sperimentale della Puglia e della Basilicata	Parisi A., Bianco A., Capozzi L., Del Sambro L., Lippolis A., Notarnicola M., Cipolletta D., Galante D.
EPI_ISL_653824, EPI_ISL_653825	Instituto Nacional de Salud, Bogotá, Colombia	Instituto Nacional de Salud, Bogotá, Colombia	Katherine Laiton-Donato, Diego A. Álvarez-Díaz, Carlos Franco-Muñoz, Mauricio Pacheco-Montealegre, Jonathan Reales, Diego Andrés Prada, Jose A. Usme-Ciro, Zulma M. Cucunubá, Christian Julian VillabonaArenas, Liz Villabona-Arenas, Sussy Echeverria, Astrid C. Flórez, Carolina Ferro, Diana Marcela Walteros-Acero, Franklin Prieto, Carlos Andrés Durán, Martha Lucia Ospina Martinez, Marcela Mercado-Reyes
EPI_ISL_653916	Diagnostic- and Research Institute of Pathology, Medical University of Graz	Diagnostic- and Research Institute of Pathology, Medical University of Graz	Karl Kashofer, Peter Regitnig, Martin Zacharias, Gregor Gorkiewicz

EPI_ISL_653922, EPI_ISL_653923, EPI_ISL_653924, EPI_ISL_653925, EPI_ISL_653926, EPI_ISL_653927, EPI_ISL_653928, EPI_ISL_653929, EPI_ISL_653930, EPI_ISL_653931, EPI_ISL_653932

see above	Molecular diagnostic laboratory of Federal Budget Institution of Science "Central Research Institute of Epidemiology" of The Federal Service on Customers' Rights Protection and Human Well-being Surveillance	Group of Genomics and Postgenomic Technologies of Central Research Institute of Epidemilology	Samoilov AE, Kaptelova VV, Dudorova A.V., Speranskaya AS, Tivanova EV, Shipulina OY, Akimkin VG
EPI_ISL_654016, EPI_ISL_654017, EPI_ISL_654018, EPI_ISL_654019, EPI_ISL_654020	Laboratory of Microbiology, National Reference Lab, Charles Nicolle Hospital; 2-University of Tunis ElManar, Faculty of Medicine of Tunis, LR99ES09, Tunis, Tunisia	1-Clinical and Experimental Pharmacology Lab, LR16SP02, National Center of Pharmacovigilance, University of Tunis El Manar, Tunis, Tunisia. 2-Neurodegenerative diseases and psychiatric troubles, LR18SP03, Razi Hospital, University of Tunis El Manar, Tunis, Tunisia. 3- Ministry of Health, National Observatory of New and Emerging Diseases, 1006, Tunis, Tunisia	Ilhem Boutiba-Ben Boubaker, Sameh Trabelsi, Nissaf Ben Alaya, Maher Kharrat, Alia Ben Kahla, Jalila Ben Khelil, Salma Abid, Sana Ferjani, Mouna Ben Sassi, Mouna Safer, Imen Mkada, Imen Kacem, Gaies Emna, Soumaya Rammeh, Riadh Daghfous, Riadh Gouider.
EPI_ISL_654793	Instituto Nacional de Salud, Bogotá, Colombia	Instituto Nacional de Salud, Bogotá, Colombia	Katherine Laiton-Donato, Diego A. Álvarez-Díaz, Carlos Franco-Muñoz, Mauricio Pacheco-Montealegre, Jonathan Reales, Diego Andrés Prada, Jose A. Usme-Ciro, Zulma M. Cucunubá, Christian Julian VillabonaArenas, Liz Villabona-Arenas, Sussy Echeverria, Astrid C. Flórez, Carolina Ferro, Diana Marcela Walteros-Acero, Franklin Prieto, Carlos Andrés Durán, Martha Lucia Ospina Martinez, Marcela Mercado-Reyes
EPI_ISL_654818	Department of Respiratory and other Viral Infections of L.V.Gromashevsky Institute of Epidemiology & Infectious Diseases NAMS of Ukrain	Department of Respiratory and other Viral Infections of L.V.Gromashevsky Institute of Epidemiology & Infectious Diseases NAMS of Ukrain, JSC "Farmak"	Alla Mironenko, Andriy Goy, Ihor Kravchuk, Ludmyla Bolotova, Larysa Radchenko, Nataliia Teteriuk
EPI_ISL_654819, EPI_ISL_654820	Department of Respiratory and other Viral Infections of L.V.Gromashevsky Institute of Epidemiology & Infectious Diseases NAMS of Ukrain	Department of Respiratory and other Viral Infections of L.V.Gromashevsky Institute of Epidemiology & Infectious Diseases NAMS of Ukrain, JSC "Farmak"	Alla Mironenko, Andriy Goy, Ihor Kravchuk, Ludmyla Bolotova, Larysa Radchenko, Nataliia Teteriuk
EPI_ISL_654867	Pasteur Institute in Ho Chi Minh city	Department of Microbiology and Immunology - Pasteur Institute in Ho Chi Minh city	Hoàng Minh, Cao Minh Thng, Phm Th Thu Hng, V Phm Hng Nhung, ào Huy Mnh, Hunh Th Kim Loan, Nguyn Hoàng Quân, Hunh Phng Tho, Hoàng Nh ào, Nguyn Trung Hiu, Nguyn Hoàng Anh, Nguyn Thu Ngc, Phm Th Nhung, La Hoàng Huy, ng Thanh Giang, Nguyn Th Thanh Thng, Trn Th Hng Kim, Nguyn Thanh Long, Phm Duy Quang, Lng Chn Quang, Hoàng Quc Cng, Phan Trng Lân, Nguyn V Thng
EPI_ISL_654868	Pasteur Institute in Ho Chi Minh city	Department of Microbiology and Immunology - Pasteur Institute in Ho Chi Minh city	ào Huy Mnh, Phm Th Thu Hng, V Phm Hng Nhung, Cao Minh Thng, Hunh Th Kim Loan, Nguyn Hoàng Quân, Hunh Phng Tho, Hoàng Nh ào, Nguyn Trung Hiu, Nguyn Hoàng Anh, Nguyn Thu Ngc, Phm Th Nhung, Nguyn Th Ngc Tho, Hà Bích Ngc, Nguyn Th Thanh Thng, Hoàng Minh, Trn Th Hng Kim, Nguyn Thanh Long, Phm Duy Quang, Lng Chn Quang, Hoàng Quc Cng, Nguyn V Thng, Phan Trng Lân
EPI_ISL_654869	Pasteur Institute in Ho Chi Minh city	Department of Microbiology and Immunology - Pasteur Institute in Ho Chi Minh city	Phm Th Nhung, ào Huy Mnh, Phm Th Thu Hng, V Phm Hng Nhung, Cao Minh Thng, Hunh Th Kim Loan, Nguyn Hoàng Quân, Hunh Phng Tho, Hoàng Nh ào, Nguyn Trung Hiu, Nguyn Hoàng Anh, Nguyn Thu Ngc, Nguyn Th Ngc Tho, ng Thanh Giang, Võ Th Trang ài, Hoàng Minh, Trn Th Hng Kim, Nguyn Thanh Long, Phm Duy Quang, Lng Chn Quang, Hoàng Quc Cng, Phan Trng Lân, Nguyn V Thng
EPI_ISL_654870	Pasteur Institute in Ho Chi Minh city	Department of Microbiology and Immunology - Pasteur Institute in Ho Chi Minh city	Trn Th Hng Kim, Phm Th Thu Hng, ào Huy Mnh, V Phm Hng Nhung, Cao Minh Thng, Hunh Th Kim Loan, Nguyn Hoàng Quân, Hunh Phng Tho, Hoàng Nh ào, Nguyn Trung Hiu, Nguyn Hoàng Anh, Nguyn Thu Ngc, Phm Th Nhung, Nguyn Th Ngc Tho, ng Thanh Giang, Nguyn Th Thanh Thng, Hoàng Kim Loan, Nguyn Thanh Long, Phm Duy Quang, Lng Chn Quang, Hoàng Quc Cng, Nguyn V Thng, Phan Trng Lân
EPI_ISL_654872	Pasteur Institute in Ho Chi Minh city	Department of Microbiology and Immunology - Pasteur Institute in Ho Chi Minh city	Phm Th Thu Hng, ào Huy Mnh, V Phm Hng Nhung, Cao Minh Thng, Hunh Th Kim Loan, Nguyn Hoàng Quân, Hunh Phng Tho, Hoàng Nh ào, Nguyn Trung Hiu, Nguyn Hoàng Anh, Nguyn Thu Ngc, Phm Th Nhung, Nguyn Th Ngc Tho, ng Thanh Giang, Nguyn Th Thanh Thng, Hoàng Minh, Nguyn Hoàng V, Nguyn Thanh Long, Phm Duy Quang, Lng Chn Quang, Hoàng Quc Cng, Phan Trng Lân, Nguyn V Thng
EPI_ISL_654873	Pasteur Institute in Ho Chi Minh city	Department of Microbiology and Immunology - Pasteur Institute in Ho Chi Minh city	Phm Th Thu Hng, ào Huy Mnh, V Phm Hng Nhung, Cao Minh Thng, Hunh Th Kim Loan, Nguyn Hoàng Quân, Hunh Phng Tho, Hoàng Nh ào, Nguyn Trung Hiu, Nguyn Th Thanh Tho, Nguyn Thu Ngc, Phm Th Nhung, Nguyn Th Ngc Tho, ng Thanh Giang, Nguyn Th Thanh Thng, Hoàng Minh, Trn Th Hng Kim, Nguyn Thanh Long, Phm Duy Quang, Lng Chn Quang, Hoàng Quc Cng, Nguyn V Thng, Phan Trng Lân
EPI_ISL_654874	Pasteur Institute in Ho Chi Minh city	Department of Microbiology and Immunology - Pasteur Institute in Ho Chi Minh city	Nguyn Th Ngc Tho, V Phm Hng Nhung, ào Huy Mnh, Phm Th Thu Hng, Cao Minh Thng, Hunh Th Kim Loan, Nguyn Hoàng Quân, Hunh Phng Tho, Hoàng Nh ào, Trn Tôn, Nguyn Hoàng Anh, Nguyn Thu Ngc, Phm Th Nhung, ng Thanh Giang, Nguyn Th Thanh Thng, Hoàng Minh, Trn Th Hng Kim, Nguyn Thanh Long, Phm Duy Quang, Lng Chn Quang, Hoàng Quc Cng, Phan Trng Lân, Nguyn V Thng
EPI_ISL_654875	Pasteur Institute in Ho Chi Minh city	Department of Microbiology and Immunology - Pasteur Institute in Ho Chi Minh city	V Phm Hng Nhung, ào Huy Mnh, Phm Th Thu Hng, Cao Minh Thng, Hunh Th Kim Loan, Nguyn Hoàng Quân, Hunh Phng Tho, Hoàng Nh ào, Nguyn Trung Hiu, Nguyn Hoàng Anh, Nguyn Vit Thinh, Phm Th Nhung, Nguyn Th Ngc Tho, ng Thanh Giang, Nguyn Th Thanh Thng, Hoàng Minh, Trn Th Hng Kim, Nguyn Thanh Long, Phm Duy Quang, Lng Chn Quang, Hoàng Quc Cng, Nguyn V Thng, Phan Trng Lân
EPI_ISL_654876	Pasteur Institute in Ho Chi Minh city	Department of Microbiology and Immunology - Pasteur Institute in Ho Chi Minh city	V Phm Hng Nhung, ào Huy Mnh, Phm Th Thu Hng, Cao Minh Thng, Hunh Th Kim Loan, Nguyn Hoàng Quân, Hunh Phng Tho, Hoàng Nh ào, Nguyn Trung Hiu, Nguyn Hoàng Anh, Nguyn Thu Ngc, Trng Công Hiu, Nguyn Th Ngc Tho, ng Thanh Giang, Nguyn Th Thanh Thng, Hoàng Minh, Trn Th Hng Kim, Nguyn Thanh Long, Phm Duy Quang, Lng Chn Quang, Hoàng Quc Cng, Phan Trng Lân, Nguyn V Thng
EPI_ISL_654877	Pasteur Institute in Ho Chi Minh city	Department of Microbiology and Immunology - Pasteur Institute in Ho Chi Minh city	ng Thanh Giang, Phm Duy Quang, ào Huy Mnh, Phm Th Thu Hng, V Phm Hng Nhung, Cao Minh Thng, Hunh Th Kim Loan, Nguyn Hoàng Quân, Hunh Phng Tho, Hoàng Nh ào, Nguyn Trung Hiu, Nguyn Hoàng Anh, Nguyn Thu Ngc, Phm Th Nhung, Trng Thùy Dung, Nguyn Th Thanh Thng, Hoàng Minh, Trn

			Th Hng Kim, Nguyn Thanh Long, Lng Chn Quang, Hoàng Quc Cng, Nguyn V Thng, Phan Trng Lân
EPI_ISL_654878	Pasteur Institute in Ho Chi Minh city	Department of Microbiology and Immunology - Pasteur Institute in Ho Chi Minh city	Phm Duy Quang, ào Huy Mnh, Phm Th Thu Hng, V Phm Hng Nhung, Cao Minh Thng, Hunh Th Kim Loan, Nguyn Hoàng Quân, Hunh Phng Tho, Hoàng Nh ào, Nguyn Trung Hiu, Nguyn Hoàng Anh, Nguyn Thu Ngc, Phm Th Nhung, Nguyn Th Ngc Tho, Viên ng Khánh Linh, Nguyn Th Thanh Thng, Hoàng Minh, Trn Th Hng Kim, Nguyn Thanh Long, Lng Chn Quang, Hoàng Quc Cng, Phan Trng Lân, Nguyn V Thng
EPI_ISL_654881	Pasteur Institute in Ho Chi Minh city	Department of Microbiology and Immunology - Pasteur Institute in Ho Chi Minh city	Lng Chn Quang, ào Huy Mnh, Phm Th Thu Hng, V Phm Hng Nhung, Cao Minh Thng, Hunh Th Kim Loan, Nguyn Hoàng Quân, Hunh Phng Tho, Hoàng Nh ào, Nguyn Trung Hiu, Nguyn Hoàng Anh, Nguyn Thu Ngc, Phm Th Nhung, Nguyn Th Ngc Tho, ng Thanh Giang, Nguyn Th Thanh Thng, Hoàng Minh, Nguyn Th Phng Thúy, Nguyn Thanh Long, Phm Duy Quang, Hoàng Quc Cng, Nguyn V Thng, Phan Trng Lân
EPI_ISL_654882	Pasteur Institute in Ho Chi Minh city	Department of Microbiology and Immunology - Pasteur Institute in Ho Chi Minh city	Lng Chn Quang, ào Huy Mnh, Phm Th Thu Hng, V Phm Hng Nhung, Cao Minh Thng, Hunh Th Kim Loan, Nguyn Hoàng Quân, Hunh Phng Tho, Hoàng Nh ào, Nguyn Trung Hiu, Nguyn Hoàng Anh, Nguyn Ngc Anh Tun, Phm Th Nhung, Nguyn Th Ngc Tho, ng Thanh Giang, Nguyn Th Thanh Thng, Hoàng Minh, Trn Th Hng Kim, Nguyn Thanh Long, Phm Duy Quang, Hoàng Quc Cng, Phan Trng Lân, Nguyn V Thng
EPI_ISL_658863, EPI_ISL_658865, EPI_ISL_658868, EPI_ISL_658870	Instituto de Diagnostico y Referencia Epidemiologicos (INDRE)	Instituto de Diagnostico y Referencia Epidemiologicos (INDRE)	Gisela Barrera-Badillo , Abril Rodriguez-Maldonado, Claudia Wong-Arambula , Natividad Cruz-Ortiz, Tatiana Nunez-Garcia, Dayanira Arellano-Suarez, Fabiola Garces-Ayala, Lucia Hernandez-Rivas, Irma Lopez-Martinez, Ernesto Ramirez-Gonzalez.
EPI_ISL_658873, EPI_ISL_658875	Instituto de Diagnostico y Referencia Epidemiologicos (INDRE)	Instituto de Diagnostico y Referencia Epidemiologicos (INDRE)	Gisela Barrera-Badillo , Abril Rodriguez-Maldonado, Claudia Wong-Arambula , Natividad Cruz-Ortiz, Tatiana Nunez-Garcia, Dayanira Arellano-Suarez, Fabiola Garces-Ayala, Lucia Hernandez-Rivas, Irma Lopez-Martinez, Ernesto Ramirez-Gonzalez.
EPI_ISL_658878, EPI_ISL_658880, EPI_ISL_658883, EPI_ISL_658886, EPI_ISL_658888, EPI_ISL_658891, EPI_ISL_658893, EPI_ISL_658896	Instituto de Diagnostico y Referencia Epidemiologicos (INDRE)	Instituto de Diagnostico y Referencia Epidemiologicos (INDRE)	Ernesto Ramirez-Gonzalez, Abril Rodriguez-Maldonado, Claudia Wong-Arambula , Natividad Cruz-Ortiz, Tatiana Nunez-Garcia, Dayanira Arellano-Suarez, Fabiola Garces-Ayala, Lucia Hernandez-Rivas, Irma Lopez-Martinez, Gisela Barrera-Badillo.
EPI_ISL_658899	Instituto de Diagnostico y Referencia Epidemiologicos (INDRE)	Instituto de Diagnostico y Referencia Epidemiologicos (INDRE)	Gisela Barrera-Badillo , Abril Rodriguez-Maldonado, Claudia Wong-Arambula , Natividad Cruz-Ortiz, Tatiana Nunez-Garcia, Dayanira Arellano-Suarez, Fabiola Garces-Ayala, Lucia Hernandez-Rivas, Irma Lopez-Martinez, Ernesto Ramirez-Gonzalez.
EPI_ISL_658901	Instituto de Diagnostico y Referencia Epidemiologicos (INDRE)	Instituto de Diagnostico y Referencia Epidemiologicos (INDRE)	Ernesto Ramirez-Gonzalez, Abril Rodriguez-Maldonado, Claudia Wong-Arambula , Natividad Cruz-Ortiz, Tatiana Nunez-Garcia, Dayanira Arellano-Suarez, Fabiola Garces-Ayala, Lucia Hernandez-Rivas, Irma Lopez-Martinez, Gisela Barrera-Badillo.
EPI_ISL_658904	Instituto de Diagnostico y Referencia Epidemiologicos (INDRE)	Instituto de Diagnostico y Referencia Epidemiologicos (INDRE)	Ernesto Ramirez-Gonzalez, Abril Rodriguez-Maldonado, Claudia Wong-Arambula , Natividad Cruz-Ortiz, Tatiana Nunez-Garcia, Dayanira Arellano-Suarez, Fabiola Garces-Ayala, Lucia Hernandez-Rivas, Irma Lopez-Martinez, Gisela Barrera-Badillo, Marisol Galindo-Galindo, Linda Andarade-Sanchez, Octavio Ruiz-Muñiz, Pamela Ramirez-Medina, Roman Canul-Aguilar, Gaudalupe Herrera-Ramirez, Jazmin Galicia-Hernandez.
EPI_ISL_660068	Instituto de Diagnostico y Referencia Epidemiologicos (INDRE)	Instituto de diagnóstico y Referencia Epidemiologicos (INDRE) Departamento de Virología	Gisela Barrera-Badillo , Abril Rodriguez-Maldonado, Claudia Wong-Arambula , Natividad Cruz-Ortiz, Tatiana Nunez-Garcia, Dayanira Arellano-Suarez, Fabiola Garces-Ayala, Lucia Hernandez-Rivas, Irma Lopez-Martinez, Ernesto Ramirez-Gonzalez.
EPI_ISL_660069, EPI_ISL_660070	Zurita & Zurita Laboratorios	Zurita & Zurita Laboratorios	Gabriela Sevillano Camilo Zurita-Salinas Karen Loaiza David Ortega-Paredes Jeannete Zurita
EPI_ISL_660109, EPI_ISL_660110	Division of Infectious Diseases, Department of Internal Medicine, Yonsei University College of Medicine	Department of biotechnology, Yonsei University	Minwoo Kim, Youn-Jung Lee, Jae sun Yoon, Jin Young Ahn, Jung Ho Kim, Jun Yong Choi*, and Jong-Won Oh*
EPI_ISL_660121, EPI_ISL_660122, EPI_ISL_660123, EPI_ISL_660124, EPI_ISL_660125, EPI_ISL_660126, EPI_ISL_660127, EPI_ISL_660128, EPI_ISL_660129	Ampath	National Health Laboratory Service (NHLS), Tygerberg	Susan Engelbrecht, Draper C, Davis M-A, Siegfried N, Williamson C, Hsiao M, Kayla Delaney, Bronwyn Kleinhans, Houriiyah Tegally, Eduan Wilkindon, Gert van Zyl, Wolfgang Preiser, Tulio de Oliveira
EPI_ISL_660130, EPI_ISL_660131, EPI_ISL_660132, EPI_ISL_660133, EPI_ISL_660134, EPI_ISL_660135, EPI_ISL_660136	Lancet	National Health Laboratory Service (NHLS), Tygerberg	Susan Engelbrecht, Draper C, Davis M-A, Siegfried N, Williamson C, Hsiao M, Kayla Delaney, Bronwyn Kleinhans, Houriiyah Tegally, Eduan Wilkindon, Gert van Zyl, Wolfgang Preiser, Tulio de Oliveira
EPI_ISL_660137, EPI_ISL_660138, EPI_ISL_660139, EPI_ISL_660141, EPI_ISL_660142	Hamadi	National Health Laboratory Service (NHLS), Tygerberg	Susan Engelbrecht, Draper C, Davis M-A, Siegfried N, Williamson C, Hsiao M, Kayla Delaney, Bronwyn Kleinhans, Houriiyah Tegally, Eduan Wilkindon, Gert van Zyl, Wolfgang Preiser, Tulio de Oliveira
EPI_ISL_660143, EPI_ISL_660144, EPI_ISL_660145, EPI_ISL_660146, EPI_ISL_660147, EPI_ISL_660148, EPI_ISL_660149, EPI_ISL_660150, EPI_ISL_660151, EPI_ISL_660152, EPI_ISL_660153, EPI_ISL_660154, EPI_ISL_660155, EPI_ISL_660156, EPI_ISL_660157, EPI_ISL_660158			
see above	PathCare	National Health Laboratory Service (NHLS), Tygerberg	Susan Engelbrecht, Draper C, Davis M-A, Siegfried N, Williamson C, Hsiao M, Kayla Delaney, Bronwyn Kleinhans, Houriiyah Tegally, Eduan Wilkindon, Gert van Zyl, Wolfgang Preiser, Tulio de Oliveira
EPI_ISL_660159, EPI_ISL_660160, EPI_ISL_660161, EPI_ISL_660162, EPI_ISL_660163, EPI_ISL_660164	NHLS-IALCH	KRISP, KZN Research Innovation and Sequencing Platform	Giandhari J, Pillay S, Lessells R, Mdlalose K, York D, Khan S, Tegally H, Wilkinson E, de Oliveira T
EPI_ISL_660165, EPI_ISL_660166, EPI_ISL_660167, EPI_ISL_660168, EPI_ISL_660169, EPI_ISL_660170, EPI_ISL_660171, EPI_ISL_660172, EPI_ISL_660173, EPI_ISL_660174, EPI_ISL_660175, EPI_ISL_660176, EPI_ISL_660177, EPI_ISL_660178, EPI_ISL_660179, EPI_ISL_660180, EPI_ISL_660181, EPI_ISL_660182, EPI_ISL_660183, EPI_ISL_660184, EPI_ISL_660185			
see above	NHLS-IALCH	KRISP, KZN Research Innovation and Sequencing Platform	Gazy I, Sigal A, Karim F, Cele S, Giandhari J, Pillay S, Tegally H, Wilkinson E, de Oliveira T
EPI_ISL_660186, EPI_ISL_660187, EPI_ISL_660188, EPI_ISL_660189, EPI_ISL_660190, EPI_ISL_660191, EPI_ISL_660192, EPI_ISL_660193, EPI_ISL_660194, EPI_ISL_660195, EPI_ISL_660196, EPI_ISL_660197, EPI_ISL_660198, EPI_ISL_660199, EPI_ISL_660200, EPI_ISL_660201, EPI_ISL_660202, EPI_ISL_660203, EPI_ISL_660204, EPI_ISL_660205, EPI_ISL_660206, EPI_ISL_660207, EPI_ISL_660208, EPI_ISL_660209, EPI_ISL_660210, EPI_ISL_660211, EPI_ISL_660212, EPI_ISL_660213, EPI_ISL_660214, EPI_ISL_660215, EPI_ISL_660216, EPI_ISL_660217, EPI_ISL_660218, EPI_ISL_660219, EPI_ISL_660220			
see above	NHLS-IALCH	KRISP, KZN Research Innovation and Sequencing Platform	Giandhari J, Pillay S, Lessells R, Mdlalose K, York D, Khan S, Tegally H, Wilkinson E, de Oliveira T
EPI_ISL_660221, EPI_ISL_660222	KRISP, KZN Research Innovation and Sequencing Platform	KRISP, KZN Research Innovation and Sequencing Platform	Giandhari J, Pillay S, Lessells R, Mdlalose K, York D, Khan S, Tegally H, Wilkinson E, de Oliveira T
EPI_ISL_660223, EPI_ISL_660224, EPI_ISL_660225, EPI_ISL_660226, EPI_ISL_660227	NHLS-IALCH	KRISP, KZN Research Innovation and Sequencing Platform	Giandhari J, Pillay S, Lessells R, Mdlalose K, York D, Khan S, Tegally H, Wilkinson E, de Oliveira T
EPI_ISL_660228, EPI_ISL_660229, EPI_ISL_660230, EPI_ISL_660231, EPI_ISL_660232, EPI_ISL_660233	KRISP, KZN Research Innovation and Sequencing Platform	KRISP, KZN Research Innovation and Sequencing Platform	Giandhari J, Pillay S, Lessells R, Mdlalose K, York D, Khan S, Tegally H, Wilkinson E, de Oliveira T
EPI_ISL_660234, EPI_ISL_660235, EPI_ISL_660236, EPI_ISL_660237, EPI_ISL_660238, EPI_ISL_660239, EPI_ISL_660240, EPI_ISL_660241, EPI_ISL_660242, EPI_ISL_660243, EPI_ISL_660244, EPI_ISL_660245, EPI_ISL_660246, EPI_ISL_660247, EPI_ISL_660248, EPI_ISL_660249, EPI_ISL_660250, EPI_ISL_660251, EPI_ISL_660252, EPI_ISL_660253, EPI_ISL_660254, EPI_ISL_660255			
see above	NHLS-IALCH	KRISP, KZN Research Innovation and Sequencing Platform	Giandhari J, Pillay S, Lessells R, Mdlalose K, York D, Khan S, Tegally H, Wilkinson E, de Oliveira T
EPI_ISL_660256, EPI_ISL_660257, EPI_ISL_660258	KRISP, KZN Research Innovation and Sequencing Platform	KRISP, KZN Research Innovation and Sequencing Platform	Giandhari J, Pillay S, Lessells R, Mdlalose K, York D, Khan S, Tegally H, Wilkinson E, de Oliveira T
EPI_ISL_660259, EPI_ISL_660260, EPI_ISL_660261, EPI_ISL_660262, EPI_ISL_660263	Molecualr Diagnostic Services (MDS)	KRISP, KZN Research Innovation and Sequencing Platform	Giandhari J, Pillay S, Lessells R, Mdlalose K, York D, Khan S, Tegally H, Wilkinson E, de Oliveira T
EPI_ISL_660323	Hospital	National Reference Center for Viruses of Respiratory Infections, Institut Pasteur, Paris	Marion Barbet, Sylvie Behillil, Méline Bizard, Angela Brisebarre, Camille Capel, Etienne Simon-Lorière, Vincent Enouf, Maud Vanpeene, Sylvie van der Werf, Alexandra Ducancelle
EPI_ISL_660324, EPI_ISL_660325	General practitioner	National Reference Center for Viruses of Respiratory	Marion Barbet, Sylvie Behillil, Méline Bizard, Angela Brisebarre, Camille Capel, Etienne Simon-Lorière, Vincent Enouf, Maud Vanpeene, Sylvie van der

		Infections, Institut Pasteur, Paris	Werf
EPI_ISL_660433, EPI_ISL_660434, EPI_ISL_660435, EPI_ISL_660436, EPI_ISL_660437, EPI_ISL_660438	Molecular diagnostic laboratory of Federal Budget Institution of Science "Central Research Institute of Epidemiology" of The Federal Service on Customers' Rights Protection and Human Well-being Surveillance	Group of Genomics and Postgenomic Technologies of Central Research Institute of Epidemilology	Samoilov AE, Kaptelova VV, Valdokhina AV, Bulanenko VP, Speranskaya AS, Tivanova EV, Shipulina OY, Akimkin VG
EPI_ISL_660553, EPI_ISL_660554, EPI_ISL_660555, EPI_ISL_660556, EPI_ISL_660557, EPI_ISL_660558, EPI_ISL_660559, EPI_ISL_660560, EPI_ISL_660561, EPI_ISL_660562, EPI_ISL_660563, EPI_ISL_660564, EPI_ISL_660565, EPI_ISL_660566, EPI_ISL_660567, EPI_ISL_660568, EPI_ISL_660569, EPI_ISL_660570, EPI_ISL_660571, EPI_ISL_660572, EPI_ISL_660573			
see above	The National Institute of Public Health	State Veterinary Institute Prague	Nagy,A;Jirincova,H;Novakova,L;Trnka,D;Vecerova,J
EPI_ISL_660605, EPI_ISL_660606, EPI_ISL_660607, EPI_ISL_660608, EPI_ISL_660609, EPI_ISL_660610, EPI_ISL_660611, EPI_ISL_660612, EPI_ISL_660613, EPI_ISL_660614, EPI_ISL_660615, EPI_ISL_660616, EPI_ISL_660617, EPI_ISL_660618, EPI_ISL_660619, EPI_ISL_660620, EPI_ISL_660621, EPI_ISL_660622, EPI_ISL_660623, EPI_ISL_660624, EPI_ISL_660625, EPI_ISL_660626, EPI_ISL_660627, EPI_ISL_660628, EPI_ISL_660629, EPI_ISL_660630, EPI_ISL_660631, EPI_ISL_660632, EPI_ISL_660633, EPI_ISL_660634, EPI_ISL_660635, EPI_ISL_660636, EPI_ISL_660637, EPI_ISL_660638, EPI_ISL_660639, EPI_ISL_660640, EPI_ISL_660641, EPI_ISL_660642, EPI_ISL_660643, EPI_ISL_660644, EPI_ISL_660645, EPI_ISL_660646, EPI_ISL_660647, EPI_ISL_660648, EPI_ISL_660649, EPI_ISL_660650, EPI_ISL_660651, EPI_ISL_660652, EPI_ISL_660653, EPI_ISL_660654, EPI_ISL_660655, EPI_ISL_660656, EPI_ISL_660657, EPI_ISL_660658, EPI_ISL_660659, EPI_ISL_660660, EPI_ISL_660661, EPI_ISL_660662, EPI_ISL_660663			
see above	NHLS-IALCH	KRISP, KZN Research Innovation and Sequencing Platform	Giandhari J, Pillay S, Lessells R, Mdlalose K, York D, Khan S, Tegally H, Wilkinson E, de Oliveira T
EPI_ISL_660664	KRISP, KZN Research Innovation and Sequencing Platform	KRISP, KZN Research Innovation and Sequencing Platform	Giandhari J, Pillay S, Lessells R, Mdlalose K, York D, Khan S, Tegally H, Wilkinson E, de Oliveira T
EPI_ISL_666870	Laboratoire de Virologie, CHU de Caen, Normandie, France.	GRAM2.0, Université de Caen Normandie	Meriadeg LE GOUIL, Januel VOUTEAU, Juliette ECHE, Estelle LEPERCHOIS, Astrid VABRET
EPI_ISL_668447, EPI_ISL_668448, EPI_ISL_668449, EPI_ISL_668450, EPI_ISL_668451, EPI_ISL_668452, EPI_ISL_668453, EPI_ISL_668454, EPI_ISL_668455	Centre for Dengue Research, Department of Immunology and Molecular Medicine	Centre for Dengue Research, Department of Immunology and Molecular Medicine	Chandima Jeewandara, Deshni Jayathilaka, Dinuka Ariyaratne, Diyanath Ranasinghe, Laksiri Gomes, Gathsaurie Neelika Malavige
EPI_ISL_671414, EPI_ISL_671415, EPI_ISL_671416, EPI_ISL_671417, EPI_ISL_671418	National Laboratory of Virology, Szentágothai Research Centre	National Laboratory of Virology, Szentágothai Research Centre	Endre Gábor Tóth, Balázs Somogyi, Brigitta, Ferenc Jakab, Gábor Kemenesi
EPI_ISL_671419, EPI_ISL_671420, EPI_ISL_671421, EPI_ISL_671422, EPI_ISL_671423, EPI_ISL_671424, EPI_ISL_671425, EPI_ISL_671426, EPI_ISL_671427, EPI_ISL_671428, EPI_ISL_671429, EPI_ISL_671430, EPI_ISL_671431, EPI_ISL_671432, EPI_ISL_671433, EPI_ISL_671434, EPI_ISL_671435, EPI_ISL_671436, EPI_ISL_671437, EPI_ISL_671438, EPI_ISL_671439, EPI_ISL_671440, EPI_ISL_671441, EPI_ISL_671442, EPI_ISL_671443, EPI_ISL_671444, EPI_ISL_671445, EPI_ISL_671446, EPI_ISL_671447, EPI_ISL_671448, EPI_ISL_671449, EPI_ISL_671450, EPI_ISL_671451, EPI_ISL_671452, EPI_ISL_671453, EPI_ISL_671454, EPI_ISL_671455, EPI_ISL_671456, EPI_ISL_671457, EPI_ISL_671458, EPI_ISL_671459, EPI_ISL_671460, EPI_ISL_671461, EPI_ISL_671462, EPI_ISL_671463, EPI_ISL_671464, EPI_ISL_671465, EPI_ISL_671466, EPI_ISL_671467, EPI_ISL_671468, EPI_ISL_671469, EPI_ISL_671470, EPI_ISL_671471, EPI_ISL_671472, EPI_ISL_671473, EPI_ISL_671474, EPI_ISL_671475, EPI_ISL_671476, EPI_ISL_671477, EPI_ISL_671478, EPI_ISL_671479, EPI_ISL_671480, EPI_ISL_671481, EPI_ISL_671482, EPI_ISL_671483, EPI_ISL_671484, EPI_ISL_671485, EPI_ISL_671486, EPI_ISL_671487, EPI_ISL_671488, EPI_ISL_671489, EPI_ISL_671490, EPI_ISL_671491, EPI_ISL_671492, EPI_ISL_671493, EPI_ISL_671494, EPI_ISL_671495			
see above	University of Debrecen, Department of Medical Microbiology	National Laboratory of Virology, Szentágothai Research Centre	Endre Gábor Tóth, Balázs Somogyi, Brigitta Zana, Eszter Csoma, Ferenc Jakab, Gábor Kemenesi
EPI_ISL_672669, EPI_ISL_672687, EPI_ISL_672688, EPI_ISL_672689, EPI_ISL_672690, EPI_ISL_672691, EPI_ISL_672693, EPI_ISL_672694, EPI_ISL_672696, EPI_ISL_672697, EPI_ISL_672698, EPI_ISL_672699, EPI_ISL_672700, EPI_ISL_672723, EPI_ISL_672725, EPI_ISL_672726, EPI_ISL_672727, EPI_ISL_672728, EPI_ISL_672729, EPI_ISL_672730, EPI_ISL_672731, EPI_ISL_672732, EPI_ISL_672733, EPI_ISL_672734, EPI_ISL_672735, EPI_ISL_672737, EPI_ISL_672738, EPI_ISL_672739, EPI_ISL_672740, EPI_ISL_672741, EPI_ISL_672742, EPI_ISL_672743, EPI_ISL_672745			
see above	Hospital das Clínicas da Faculdade de Medicina da Universidade de São Paulo (HC-FMUSP)	Laboratório de Parasitologia Médica - Instituto de Medicina Tropical - Universidade de São Paulo	Brazil-UK Centre for Arbovirus Discovery Diagnosis Genomics and Epidemiology (CADDE) Genomic Network - Instituto de Medicina Tropical
EPI_ISL_677673	Wadsworth Center, New York State Department.of Health	Wadsworth Center, New York State Department.of Health	Kirsten St. George, Daryl M. Lamson, Alexis Russel, Jonathan Plitnick, Navjot Singh, John Kelly, Sara Griesemer, Erasmus Schneider, Erica Lasek-Nesselquist
EPI_ISL_677678, EPI_ISL_677679, EPI_ISL_677680, EPI_ISL_677681, EPI_ISL_677682, EPI_ISL_677683, EPI_ISL_677684, EPI_ISL_677685, EPI_ISL_677686, EPI_ISL_677687, EPI_ISL_677688, EPI_ISL_677689, EPI_ISL_677692, EPI_ISL_677695, EPI_ISL_677696, EPI_ISL_677699			
see above	Vanda Pharmaceuticals Clinical Site	Vanda Pharmaceuticals	Vanda Pharmaceuticals
EPI_ISL_677727, EPI_ISL_677728, EPI_ISL_677729, EPI_ISL_677730, EPI_ISL_677731, EPI_ISL_677732, EPI_ISL_677733, EPI_ISL_677734, EPI_ISL_677735, EPI_ISL_677736, EPI_ISL_677737, EPI_ISL_677738, EPI_ISL_677739, EPI_ISL_677740, EPI_ISL_677741, EPI_ISL_677742, EPI_ISL_677743, EPI_ISL_677744, EPI_ISL_677745, EPI_ISL_677746, EPI_ISL_677747, EPI_ISL_677748, EPI_ISL_677749, EPI_ISL_677750, EPI_ISL_677751, EPI_ISL_677752, EPI_ISL_677753, EPI_ISL_677754, EPI_ISL_677755, EPI_ISL_677756, EPI_ISL_677757, EPI_ISL_677758, EPI_ISL_677759, EPI_ISL_677760, EPI_ISL_677761, EPI_ISL_677762, EPI_ISL_677763, EPI_ISL_677764, EPI_ISL_677765, EPI_ISL_677766, EPI_ISL_677767, EPI_ISL_677768, EPI_ISL_677769, EPI_ISL_677770, EPI_ISL_677771, EPI_ISL_677772, EPI_ISL_677773, EPI_ISL_677774, EPI_ISL_677775, EPI_ISL_677776, EPI_ISL_677777, EPI_ISL_677778, EPI_ISL_677779, EPI_ISL_677780, EPI_ISL_677781, EPI_ISL_677782, EPI_ISL_677783, EPI_ISL_677784, EPI_ISL_677785, EPI_ISL_677786, EPI_ISL_677787, EPI_ISL_677788, EPI_ISL_677789, EPI_ISL_677790, EPI_ISL_677791, EPI_ISL_677792, EPI_ISL_677793, EPI_ISL_677794, EPI_ISL_677795, EPI_ISL_677796, EPI_ISL_677797, EPI_ISL_677798, EPI_ISL_677799, EPI_ISL_677800, EPI_ISL_677801, EPI_ISL_677802, EPI_ISL_677803, EPI_ISL_677804, EPI_ISL_677805, EPI_ISL_677806, EPI_ISL_677807, EPI_ISL_677808, EPI_ISL_677809, EPI_ISL_677810, EPI_ISL_677811, EPI_ISL_677812, EPI_ISL_677813, EPI_ISL_677814, EPI_ISL_677815, EPI_ISL_677816, EPI_ISL_677817, EPI_ISL_677818, EPI_ISL_677819, EPI_ISL_677820, EPI_ISL_678290, EPI_ISL_678291			
see above	University of Szeged, Institute of Clinical Microbiology	National Laboratory of Virology, Szentágothai Research Centre	Endre Gábor Tóth, Balázs Somogyi, Brigitta, Gabriella Terhes, Ferenc Jakab, Gábor Kemenesi
EPI_ISL_678387, EPI_ISL_678388, EPI_ISL_678389, EPI_ISL_678390, EPI_ISL_678391, EPI_ISL_678392, EPI_ISL_678393, EPI_ISL_678394, EPI_ISL_678395, EPI_ISL_678396	University of Debrecen, Department of Medical Microbiology	National Laboratory of Virology, Szentágothai Research Centre	Endre Gábor Tóth, Balázs Somogyi, Brigitta Zana, Eszter Csoma, Ferenc Jakab, Gábor Kemenesi
EPI_ISL_681702, EPI_ISL_681703	Zurita & Zurita Laboratorios	Zurita & Zurita Laboratorios	Gabriela Sevillano, Camilo Zurita-Salinas, Jeannete Zurita
EPI_ISL_682242	HOSPITAL SAN VICENTE DE PAUL	Inciensa, Instituto Costarricense de Investigación y Enseñanza en Nutrición y Salud	Francisco Duarte, Hebleen Porras, Claudio Soto-Garita, Estela Cordero, Adriana Godinez, Melany Calderon & Mariel López
EPI_ISL_682250, EPI_ISL_682251, EPI_ISL_682252	AREA DE SALUD ESCAZU (COOPESANA)	Inciensa, Instituto Costarricense de Investigación y Enseñanza en Nutrición y Salud	Francisco Duarte, Hebleen Porras, Claudio Soto-Garita, Estela Cordero, Adriana Godinez & Melany Calderon
EPI_ISL_682255	HOSPITAL SAN VICENTE DE PAUL	Inciensa, Instituto Costarricense de Investigación y Enseñanza en Nutrición y Salud	Francisco Duarte, Hebleen Porras, Claudio Soto-Garita, Estela Cordero, Adriana Godinez & Melany Calderon
EPI_ISL_682257	HOSPITAL SAN JUAN DE DIOS	Inciensa, Instituto Costarricense de Investigación y Enseñanza en Nutrición y Salud	Francisco Duarte, Hebleen Porras, Claudio Soto-Garita, Estela Cordero, Adriana Godinez & Melany Calderon
EPI_ISL_682261	TAMIZAJE COMUNITARIO- PASO CANOAS	Inciensa, Instituto Costarricense de Investigación y Enseñanza en Nutrición y Salud	Francisco Duarte, Hebleen Porras, Claudio Soto-Garita, Estela Cordero, Adriana Godinez & Melany Calderon
EPI_ISL_682262, EPI_ISL_682264, EPI_ISL_682266, EPI_ISL_682273	HOSPITAL SAN JUAN DE DIOS	Inciensa, Instituto Costarricense de Investigación y Enseñanza en Nutrición y Salud	Francisco Duarte, Hebleen Porras, Claudio Soto-Garita, Estela Cordero, Adriana Godinez & Melany Calderon

We gratefully acknowledge the following Authors from the Originating laboratories responsible for obtaining the specimens, as well as the Submitting laboratories where the genome data were generated and shared via GISAID, on which this research is based.

All Submitters of data may be contacted directly via www.gisaid.org

Authors are sorted alphabetically.

Accession ID	Originating Laboratory	Submitting Laboratory	Authors
EPI_ISL_684108, EPI_ISL_684109	Tokyo Metropolitan Institute of Public Health	Pathogen Genomics Center, National Institute of Infectious Diseases	Tsuyoshi Sekizuka, Kentaro Itokawa, Rina Tanaka, Masanori Hashino, Makoto Kuroda
EPI_ISL_684110, EPI_ISL_684111, EPI_ISL_684112, EPI_ISL_684113, EPI_ISL_684114, EPI_ISL_684115, EPI_ISL_684116, EPI_ISL_684117, EPI_ISL_684118, EPI_ISL_684119, EPI_ISL_684120, EPI_ISL_684121, EPI_ISL_684122, EPI_ISL_684123, EPI_ISL_684124, EPI_ISL_684125, EPI_ISL_684126			
see above	Pathogen Genomics Center, National Institute of Infectious Diseases	Pathogen Genomics Center, National Institute of Infectious Diseases	Tsuyoshi Sekizuka, Kentaro Itokawa, Rina Tanaka, Masanori Hashino, Makoto Kuroda
EPI_ISL_684127	Gunma Prefectural Institute of Public Health and Environmental Sciences	Pathogen Genomics Center, National Institute of Infectious Diseases	Tsuyoshi Sekizuka, Kentaro Itokawa, Rina Tanaka, Masanori Hashino, Makoto Kuroda
EPI_ISL_684128, EPI_ISL_684129, EPI_ISL_684130, EPI_ISL_684131, EPI_ISL_684132, EPI_ISL_684133, EPI_ISL_684134, EPI_ISL_684135	Pathogen Genomics Center, National Institute of Infectious Diseases	Pathogen Genomics Center, National Institute of Infectious Diseases	Tsuyoshi Sekizuka, Kentaro Itokawa, Rina Tanaka, Masanori Hashino, Makoto Kuroda
EPI_ISL_684136, EPI_ISL_684137, EPI_ISL_684138	Tokyo Metropolitan Institute of Public Health	Pathogen Genomics Center, National Institute of Infectious Diseases	Tsuyoshi Sekizuka, Kentaro Itokawa, Rina Tanaka, Masanori Hashino, Makoto Kuroda
EPI_ISL_684139, EPI_ISL_684140, EPI_ISL_684141, EPI_ISL_684142, EPI_ISL_684143, EPI_ISL_684144, EPI_ISL_684145, EPI_ISL_684146	Pathogen Genomics Center, National Institute of Infectious Diseases	Pathogen Genomics Center, National Institute of Infectious Diseases	Tsuyoshi Sekizuka, Kentaro Itokawa, Rina Tanaka, Masanori Hashino, Makoto Kuroda
EPI_ISL_684147	Sakai City Institute of Public Health	Pathogen Genomics Center, National Institute of Infectious Diseases	Tsuyoshi Sekizuka, Kentaro Itokawa, Rina Tanaka, Masanori Hashino, Makoto Kuroda
EPI_ISL_684148, EPI_ISL_684149	Saitama Prefectural Institute of Public Health	Pathogen Genomics Center, National Institute of Infectious Diseases	Tsuyoshi Sekizuka, Kentaro Itokawa, Rina Tanaka, Masanori Hashino, Makoto Kuroda
EPI_ISL_684150, EPI_ISL_684151, EPI_ISL_684152, EPI_ISL_684153	Pathogen Genomics Center, National Institute of Infectious Diseases	Pathogen Genomics Center, National Institute of Infectious Diseases	Tsuyoshi Sekizuka, Kentaro Itokawa, Rina Tanaka, Masanori Hashino, Makoto Kuroda
EPI_ISL_684154, EPI_ISL_684155	Tokyo Metropolitan Institute of Public Health	Pathogen Genomics Center, National Institute of Infectious Diseases	Tsuyoshi Sekizuka, Kentaro Itokawa, Rina Tanaka, Masanori Hashino, Makoto Kuroda
EPI_ISL_684156, EPI_ISL_684157, EPI_ISL_684158	Pathogen Genomics Center, National Institute of Infectious Diseases	Pathogen Genomics Center, National Institute of Infectious Diseases	Tsuyoshi Sekizuka, Kentaro Itokawa, Rina Tanaka, Masanori Hashino, Makoto Kuroda
EPI_ISL_684159, EPI_ISL_684160, EPI_ISL_684161	Chiba Prefectural Institute of Public Health	Pathogen Genomics Center, National Institute of Infectious Diseases	Tsuyoshi Sekizuka, Kentaro Itokawa, Rina Tanaka, Masanori Hashino, Makoto Kuroda
EPI_ISL_684162, EPI_ISL_684163, EPI_ISL_684164, EPI_ISL_684165, EPI_ISL_684166, EPI_ISL_684167, EPI_ISL_684168, EPI_ISL_684169, EPI_ISL_684170, EPI_ISL_684171, EPI_ISL_684172, EPI_ISL_684173, EPI_ISL_684174, EPI_ISL_684175, EPI_ISL_684176, EPI_ISL_684177, EPI_ISL_684178, EPI_ISL_684179, EPI_ISL_684180, EPI_ISL_684181, EPI_ISL_684182, EPI_ISL_684183, EPI_ISL_684184, EPI_ISL_684185, EPI_ISL_684186, EPI_ISL_684187, EPI_ISL_684188, EPI_ISL_684189, EPI_ISL_684190, EPI_ISL_684191, EPI_ISL_684192, EPI_ISL_684193, EPI_ISL_684194, EPI_ISL_684195, EPI_ISL_684196, EPI_ISL_684197, EPI_ISL_684198, EPI_ISL_684199, EPI_ISL_684200, EPI_ISL_684201, EPI_ISL_684202, EPI_ISL_684203, EPI_ISL_684204, EPI_ISL_684205, EPI_ISL_684206, EPI_ISL_684207, EPI_ISL_684208, EPI_ISL_684209, EPI_ISL_684210, EPI_ISL_684211, EPI_ISL_684212, EPI_ISL_684213, EPI_ISL_684214, EPI_ISL_684215, EPI_ISL_684216, EPI_ISL_684217, EPI_ISL_684218, EPI_ISL_684219			
see above	Pathogen Genomics Center, National Institute of Infectious Diseases	Pathogen Genomics Center, National Institute of Infectious Diseases	Tsuyoshi Sekizuka, Kentaro Itokawa, Rina Tanaka, Masanori Hashino, Makoto Kuroda
EPI_ISL_684220, EPI_ISL_684221, EPI_ISL_684222, EPI_ISL_684223, EPI_ISL_684224, EPI_ISL_684225, EPI_ISL_684226, EPI_ISL_684227, EPI_ISL_684228, EPI_ISL_684229, EPI_ISL_684230, EPI_ISL_684231, EPI_ISL_684232, EPI_ISL_684233, EPI_ISL_684234, EPI_ISL_684235, EPI_ISL_684236, EPI_ISL_684237, EPI_ISL_684238, EPI_ISL_684239, EPI_ISL_684240, EPI_ISL_684241, EPI_ISL_684242, EPI_ISL_684243, EPI_ISL_684244, EPI_ISL_684245, EPI_ISL_684246, EPI_ISL_684247, EPI_ISL_684248, EPI_ISL_684249, EPI_ISL_684250, EPI_ISL_684251, EPI_ISL_684252, EPI_ISL_684253, EPI_ISL_684254, EPI_ISL_684255, EPI_ISL_684256, EPI_ISL_684257, EPI_ISL_684258, EPI_ISL_684259, EPI_ISL_684260, EPI_ISL_684261, EPI_ISL_684262, EPI_ISL_684263, EPI_ISL_684264, EPI_ISL_684265, EPI_ISL_684266, EPI_ISL_684267, EPI_ISL_684268, EPI_ISL_684269, EPI_ISL_684270, EPI_ISL_684271, EPI_ISL_684272, EPI_ISL_684273, EPI_ISL_684274, EPI_ISL_684275, EPI_ISL_684276, EPI_ISL_684277, EPI_ISL_684278, EPI_ISL_684279, EPI_ISL_684280, EPI_ISL_684281, EPI_ISL_684282, EPI_ISL_684283, EPI_ISL_684284, EPI_ISL_684285, EPI_ISL_684286, EPI_ISL_684287, EPI_ISL_684288			
see above	Gunma Prefectural Institute of Public Health and Environmental Sciences	Pathogen Genomics Center, National Institute of Infectious Diseases	Tsuyoshi Sekizuka, Kentaro Itokawa, Rina Tanaka, Masanori Hashino, Makoto Kuroda
EPI_ISL_684289, EPI_ISL_684290, EPI_ISL_684291, EPI_ISL_684292, EPI_ISL_684293, EPI_ISL_684294, EPI_ISL_684295, EPI_ISL_684296, EPI_ISL_684297, EPI_ISL_684298, EPI_ISL_684299, EPI_ISL_684300, EPI_ISL_684301, EPI_ISL_684302, EPI_ISL_684303, EPI_ISL_684304, EPI_ISL_684305, EPI_ISL_684306, EPI_ISL_684307, EPI_ISL_684308, EPI_ISL_684309, EPI_ISL_684310, EPI_ISL_684311, EPI_ISL_684312, EPI_ISL_684313, EPI_ISL_684314, EPI_ISL_684315, EPI_ISL_684316, EPI_ISL_684317, EPI_ISL_684318, EPI_ISL_684319, EPI_ISL_684320, EPI_ISL_684321, EPI_ISL_684322, EPI_ISL_684323, EPI_ISL_684324			
see above	Pathogen Genomics Center, National Institute of Infectious Diseases	Pathogen Genomics Center, National Institute of Infectious Diseases	Tsuyoshi Sekizuka, Kentaro Itokawa, Rina Tanaka, Masanori Hashino, Makoto Kuroda
EPI_ISL_684325, EPI_ISL_684326, EPI_ISL_684327, EPI_ISL_684328, EPI_ISL_684329, EPI_ISL_684330, EPI_ISL_684331, EPI_ISL_684332, EPI_ISL_684333, EPI_ISL_684334, EPI_ISL_684335, EPI_ISL_684336, EPI_ISL_684337, EPI_ISL_684338			
see above	Gunma Prefectural Institute of Public Health and Environmental Sciences	Pathogen Genomics Center, National Institute of Infectious Diseases	Tsuyoshi Sekizuka, Kentaro Itokawa, Rina Tanaka, Masanori Hashino, Makoto Kuroda
EPI_ISL_684339, EPI_ISL_684340, EPI_ISL_684341, EPI_ISL_684342, EPI_ISL_684343, EPI_ISL_684344, EPI_ISL_684345, EPI_ISL_684346, EPI_ISL_684347, EPI_ISL_684348, EPI_ISL_684349, EPI_ISL_684350, EPI_ISL_684351, EPI_ISL_684352, EPI_ISL_684353, EPI_ISL_684354, EPI_ISL_684355, EPI_ISL_684356, EPI_ISL_684357, EPI_ISL_684358, EPI_ISL_684359, EPI_ISL_684360, EPI_ISL_684361, EPI_ISL_684362, EPI_ISL_684363, EPI_ISL_684364, EPI_ISL_684365, EPI_ISL_684366, EPI_ISL_684367, EPI_ISL_684368, EPI_ISL_684369, EPI_ISL_684370, EPI_ISL_684371, EPI_ISL_684372, EPI_ISL_684373, EPI_ISL_684374, EPI_ISL_684375, EPI_ISL_684376, EPI_ISL_684377, EPI_ISL_684378, EPI_ISL_684379, EPI_ISL_684380, EPI_ISL_684381, EPI_ISL_684382, EPI_ISL_684383, EPI_ISL_684384, EPI_ISL_684385, EPI_ISL_684386, EPI_ISL_684387, EPI_ISL_684388, EPI_ISL_684389, EPI_ISL_684390, EPI_ISL_684391, EPI_ISL_684392, EPI_ISL_684393, EPI_ISL_684394, EPI_ISL_684395, EPI_ISL_684396, EPI_ISL_684397, EPI_ISL_684398, EPI_ISL_684399, EPI_ISL_684400, EPI_ISL_684401, EPI_ISL_684402, EPI_ISL_684403, EPI_ISL_684404, EPI_ISL_684405, EPI_ISL_684406, EPI_ISL_684407			
see above	Pathogen Genomics Center, National Institute of Infectious Diseases	Pathogen Genomics Center, National Institute of Infectious Diseases	Tsuyoshi Sekizuka, Kentaro Itokawa, Rina Tanaka, Masanori Hashino, Makoto Kuroda
EPI_ISL_684408, EPI_ISL_684409, EPI_ISL_684410, EPI_ISL_684411, EPI_ISL_684412, EPI_ISL_684413, EPI_ISL_684414, EPI_ISL_684415, EPI_ISL_684416, EPI_ISL_684417, EPI_ISL_684418, EPI_ISL_684419, EPI_ISL_684420, EPI_ISL_684421, EPI_ISL_684422, EPI_ISL_684423, EPI_ISL_684424, EPI_ISL_684425, EPI_ISL_684426, EPI_ISL_684427, EPI_ISL_684428, EPI_ISL_684429, EPI_ISL_684430, EPI_ISL_684431, EPI_ISL_684432, EPI_ISL_684433, EPI_ISL_684434, EPI_ISL_684435, EPI_ISL_684436, EPI_ISL_684437, EPI_ISL_684438, EPI_ISL_684439, EPI_ISL_684440, EPI_ISL_684441, EPI_ISL_684442, EPI_ISL_684443, EPI_ISL_684444, EPI_ISL_684445, EPI_ISL_684446, EPI_ISL_684447, EPI_ISL_684448, EPI_ISL_684449, EPI_ISL_684450, EPI_ISL_684451, EPI_ISL_684452, EPI_ISL_684453, EPI_ISL_684454, EPI_ISL_684455, EPI_ISL_684456, EPI_ISL_684457, EPI_ISL_684458, EPI_ISL_684459, EPI_ISL_684460, EPI_ISL_684461, EPI_ISL_684462, EPI_ISL_684463, EPI_ISL_684464, EPI_ISL_684465, EPI_ISL_684466, EPI_ISL_684467, EPI_ISL_684468, EPI_ISL_684469, EPI_ISL_684470, EPI_ISL_684471, EPI_ISL_684472, EPI_ISL_684473, EPI_ISL_684474, EPI_ISL_684475, EPI_ISL_684476, EPI_ISL_684477, EPI_ISL_684478, EPI_ISL_684479, EPI_ISL_684480, EPI_ISL_684481, EPI_ISL_684482, EPI_ISL_684483			
see above	Fukuoka Institute of Health and Environmental Sciences	Pathogen Genomics Center, National Institute of Infectious Diseases	Tsuyoshi Sekizuka, Kentaro Itokawa, Rina Tanaka, Masanori Hashino, Makoto Kuroda
EPI_ISL_684484, EPI_ISL_684485, EPI_ISL_684486, EPI_ISL_684487, EPI_ISL_684488, EPI_ISL_684489, EPI_ISL_684490, EPI_ISL_684491, EPI_ISL_684492, EPI_ISL_684493, EPI_ISL_684494, EPI_ISL_684495, EPI_ISL_684496, EPI_ISL_684497, EPI_ISL_684498, EPI_ISL_684499, EPI_ISL_684500, EPI_ISL_684501, EPI_ISL_684502, EPI_ISL_684503, EPI_ISL_684504, EPI_ISL_684505			

see above	Pathogen Genomics Center, National Institute of Infectious Diseases	Pathogen Genomics Center, National Institute of Infectious Diseases	Tsuyoshi Sekizuka, Kentaro Itokawa, Rina Tanaka, Masanori Hashino, Makoto Kuroda
EPI_ISL_684506, EPI_ISL_684507, EPI_ISL_684508, EPI_ISL_684509, EPI_ISL_684510, EPI_ISL_684511, EPI_ISL_684512, EPI_ISL_684513, EPI_ISL_684514, EPI_ISL_684515, EPI_ISL_684516, EPI_ISL_684517, EPI_ISL_684518, EPI_ISL_684519, EPI_ISL_684520, EPI_ISL_684521, EPI_ISL_684522			
see above	Tokyo Metropolitan Institute of Public Health	Pathogen Genomics Center, National Institute of Infectious Diseases	Tsuyoshi Sekizuka, Kentaro Itokawa, Rina Tanaka, Masanori Hashino, Makoto Kuroda
EPI_ISL_684523, EPI_ISL_684524, EPI_ISL_684525, EPI_ISL_684526, EPI_ISL_684527, EPI_ISL_684528, EPI_ISL_684529, EPI_ISL_684530, EPI_ISL_684531, EPI_ISL_684532, EPI_ISL_684533, EPI_ISL_684534, EPI_ISL_684535, EPI_ISL_684536, EPI_ISL_684537, EPI_ISL_684538, EPI_ISL_684539, EPI_ISL_684540, EPI_ISL_684541, EPI_ISL_684542, EPI_ISL_684543, EPI_ISL_684544, EPI_ISL_684545, EPI_ISL_684546, EPI_ISL_684547, EPI_ISL_684548, EPI_ISL_684549, EPI_ISL_684550, EPI_ISL_684551, EPI_ISL_684552, EPI_ISL_684553, EPI_ISL_684554, EPI_ISL_684555			
see above	Pathogen Genomics Center, National Institute of Infectious Diseases	Pathogen Genomics Center, National Institute of Infectious Diseases	Tsuyoshi Sekizuka, Kentaro Itokawa, Rina Tanaka, Masanori Hashino, Makoto Kuroda
EPI_ISL_684556, EPI_ISL_684557	Tokyo Metropolitan Institute of Public Health	Pathogen Genomics Center, National Institute of Infectious Diseases	Tsuyoshi Sekizuka, Kentaro Itokawa, Rina Tanaka, Masanori Hashino, Makoto Kuroda
EPI_ISL_684558, EPI_ISL_684559, EPI_ISL_684560, EPI_ISL_684561, EPI_ISL_684562, EPI_ISL_684563, EPI_ISL_684564, EPI_ISL_684565	Pathogen Genomics Center, National Institute of Infectious Diseases	Pathogen Genomics Center, National Institute of Infectious Diseases	Tsuyoshi Sekizuka, Kentaro Itokawa, Rina Tanaka, Masanori Hashino, Makoto Kuroda
EPI_ISL_684566, EPI_ISL_684567, EPI_ISL_684568, EPI_ISL_684569, EPI_ISL_684570, EPI_ISL_684571, EPI_ISL_684572, EPI_ISL_684573, EPI_ISL_684574, EPI_ISL_684575, EPI_ISL_684576, EPI_ISL_684577			
see above	Gunma Prefectural Institute of Public Health and Environmental Sciences	Pathogen Genomics Center, National Institute of Infectious Diseases	Tsuyoshi Sekizuka, Kentaro Itokawa, Rina Tanaka, Masanori Hashino, Makoto Kuroda
EPI_ISL_684578, EPI_ISL_684579, EPI_ISL_684580, EPI_ISL_684581, EPI_ISL_684582, EPI_ISL_684583, EPI_ISL_684584, EPI_ISL_684585, EPI_ISL_684586, EPI_ISL_684587, EPI_ISL_684588, EPI_ISL_684589, EPI_ISL_684590, EPI_ISL_684591, EPI_ISL_684592, EPI_ISL_684593, EPI_ISL_684594, EPI_ISL_684595, EPI_ISL_684596, EPI_ISL_684597, EPI_ISL_684598, EPI_ISL_684599, EPI_ISL_684600, EPI_ISL_684601, EPI_ISL_684602, EPI_ISL_684603, EPI_ISL_684604, EPI_ISL_684605, EPI_ISL_684606, EPI_ISL_684607, EPI_ISL_684608, EPI_ISL_684609, EPI_ISL_684610, EPI_ISL_684611, EPI_ISL_684612, EPI_ISL_684613, EPI_ISL_684614			
see above	Pathogen Genomics Center, National Institute of Infectious Diseases	Pathogen Genomics Center, National Institute of Infectious Diseases	Tsuyoshi Sekizuka, Kentaro Itokawa, Rina Tanaka, Masanori Hashino, Makoto Kuroda
EPI_ISL_684615, EPI_ISL_684616, EPI_ISL_684617, EPI_ISL_684618, EPI_ISL_684619, EPI_ISL_684620, EPI_ISL_684621, EPI_ISL_684622, EPI_ISL_684623, EPI_ISL_684624, EPI_ISL_684625, EPI_ISL_684626, EPI_ISL_684627, EPI_ISL_684628, EPI_ISL_684629, EPI_ISL_684630, EPI_ISL_684631, EPI_ISL_684632, EPI_ISL_684633			
see above	Tokyo Metropolitan Institute of Public Health	Pathogen Genomics Center, National Institute of Infectious Diseases	Tsuyoshi Sekizuka, Kentaro Itokawa, Rina Tanaka, Masanori Hashino, Makoto Kuroda
EPI_ISL_684634, EPI_ISL_684635, EPI_ISL_684636, EPI_ISL_684637, EPI_ISL_684638, EPI_ISL_684639, EPI_ISL_684640, EPI_ISL_684641, EPI_ISL_684642, EPI_ISL_684643, EPI_ISL_684644, EPI_ISL_684645, EPI_ISL_684646, EPI_ISL_684647, EPI_ISL_684648, EPI_ISL_684649, EPI_ISL_684650, EPI_ISL_684651, EPI_ISL_684652, EPI_ISL_684653, EPI_ISL_684654, EPI_ISL_684655, EPI_ISL_684656, EPI_ISL_684657, EPI_ISL_684658, EPI_ISL_684659, EPI_ISL_684660, EPI_ISL_684661, EPI_ISL_684662, EPI_ISL_684663, EPI_ISL_684664, EPI_ISL_684665, EPI_ISL_684666, EPI_ISL_684667, EPI_ISL_684668, EPI_ISL_684669, EPI_ISL_684670, EPI_ISL_684671, EPI_ISL_684672, EPI_ISL_684673, EPI_ISL_684674, EPI_ISL_684675, EPI_ISL_684676, EPI_ISL_684677, EPI_ISL_684678, EPI_ISL_684679, EPI_ISL_684680, EPI_ISL_684681, EPI_ISL_684682, EPI_ISL_684683, EPI_ISL_684684, EPI_ISL_684685, EPI_ISL_684686, EPI_ISL_684687, EPI_ISL_684688, EPI_ISL_684689, EPI_ISL_684690, EPI_ISL_684691, EPI_ISL_684692			
see above	Fukuoka Institute of Health and Environmental Sciences	Pathogen Genomics Center, National Institute of Infectious Diseases	Tsuyoshi Sekizuka, Kentaro Itokawa, Rina Tanaka, Masanori Hashino, Makoto Kuroda
EPI_ISL_684693, EPI_ISL_684694, EPI_ISL_684695, EPI_ISL_684696, EPI_ISL_684697, EPI_ISL_684698, EPI_ISL_684699, EPI_ISL_684700, EPI_ISL_684701, EPI_ISL_684702, EPI_ISL_684703, EPI_ISL_684704, EPI_ISL_684705, EPI_ISL_684706, EPI_ISL_684707, EPI_ISL_684708, EPI_ISL_684709, EPI_ISL_684710, EPI_ISL_684711, EPI_ISL_684712, EPI_ISL_684713, EPI_ISL_684714, EPI_ISL_684715, EPI_ISL_684716, EPI_ISL_684717, EPI_ISL_684718, EPI_ISL_684719, EPI_ISL_684720, EPI_ISL_684721, EPI_ISL_684722			
see above	Pathogen Genomics Center, National Institute of Infectious Diseases	Pathogen Genomics Center, National Institute of Infectious Diseases	Tsuyoshi Sekizuka, Kentaro Itokawa, Rina Tanaka, Masanori Hashino, Makoto Kuroda
EPI_ISL_684723, EPI_ISL_684724, EPI_ISL_684725, EPI_ISL_684726, EPI_ISL_684727	Fukuoka Institute of Health and Environmental Sciences	Pathogen Genomics Center, National Institute of Infectious Diseases	Tsuyoshi Sekizuka, Kentaro Itokawa, Rina Tanaka, Masanori Hashino, Makoto Kuroda
EPI_ISL_684728, EPI_ISL_684729, EPI_ISL_684730, EPI_ISL_684731, EPI_ISL_684732, EPI_ISL_684733, EPI_ISL_684734, EPI_ISL_684735	Tokyo Metropolitan Institute of Public Health	Pathogen Genomics Center, National Institute of Infectious Diseases	Tsuyoshi Sekizuka, Kentaro Itokawa, Rina Tanaka, Masanori Hashino, Makoto Kuroda
EPI_ISL_684736, EPI_ISL_684737, EPI_ISL_684738, EPI_ISL_684739, EPI_ISL_684740, EPI_ISL_684741, EPI_ISL_684742, EPI_ISL_684743, EPI_ISL_684744, EPI_ISL_684745, EPI_ISL_684746, EPI_ISL_684747, EPI_ISL_684748, EPI_ISL_684749, EPI_ISL_684750, EPI_ISL_684751, EPI_ISL_684752, EPI_ISL_684753, EPI_ISL_684754, EPI_ISL_684755, EPI_ISL_684756, EPI_ISL_684757, EPI_ISL_684758, EPI_ISL_684759, EPI_ISL_684760, EPI_ISL_684761, EPI_ISL_684762, EPI_ISL_684763, EPI_ISL_684764, EPI_ISL_684765, EPI_ISL_684766, EPI_ISL_684767, EPI_ISL_684768, EPI_ISL_684769, EPI_ISL_684770, EPI_ISL_684771, EPI_ISL_684772, EPI_ISL_684773, EPI_ISL_684774, EPI_ISL_684775, EPI_ISL_684776, EPI_ISL_684777, EPI_ISL_684778, EPI_ISL_684779, EPI_ISL_684780, EPI_ISL_684781, EPI_ISL_684782, EPI_ISL_684783, EPI_ISL_684784, EPI_ISL_684785, EPI_ISL_684786, EPI_ISL_684787, EPI_ISL_684788, EPI_ISL_684789, EPI_ISL_684790, EPI_ISL_684791, EPI_ISL_684792, EPI_ISL_684793, EPI_ISL_684794, EPI_ISL_684795, EPI_ISL_684796, EPI_ISL_684797			
see above	Pathogen Genomics Center, National Institute of Infectious Diseases	Pathogen Genomics Center, National Institute of Infectious Diseases	Tsuyoshi Sekizuka, Kentaro Itokawa, Rina Tanaka, Masanori Hashino, Makoto Kuroda
EPI_ISL_684798, EPI_ISL_684799, EPI_ISL_684800, EPI_ISL_684801, EPI_ISL_684802, EPI_ISL_684803, EPI_ISL_684804, EPI_ISL_684805, EPI_ISL_684806, EPI_ISL_684807, EPI_ISL_684808, EPI_ISL_684809, EPI_ISL_684810, EPI_ISL_684811, EPI_ISL_684812, EPI_ISL_684813, EPI_ISL_684814, EPI_ISL_684815, EPI_ISL_684816, EPI_ISL_684817, EPI_ISL_684818, EPI_ISL_684819, EPI_ISL_684820, EPI_ISL_684821, EPI_ISL_684822, EPI_ISL_684823, EPI_ISL_684824, EPI_ISL_684825, EPI_ISL_684826, EPI_ISL_684827, EPI_ISL_684828, EPI_ISL_684829, EPI_ISL_684830, EPI_ISL_684831, EPI_ISL_684832, EPI_ISL_684833, EPI_ISL_684834, EPI_ISL_684835, EPI_ISL_684836, EPI_ISL_684837, EPI_ISL_684838, EPI_ISL_684839, EPI_ISL_684840, EPI_ISL_684841, EPI_ISL_684842, EPI_ISL_684843, EPI_ISL_684844, EPI_ISL_684845, EPI_ISL_684846, EPI_ISL_684847, EPI_ISL_684848, EPI_ISL_684849, EPI_ISL_684850, EPI_ISL_684851, EPI_ISL_684852, EPI_ISL_684853, EPI_ISL_684854, EPI_ISL_684855, EPI_ISL_684856			
see above	Yamagata Prefectural Institute of Public Health	Pathogen Genomics Center, National Institute of Infectious Diseases	Tsuyoshi Sekizuka, Kentaro Itokawa, Rina Tanaka, Masanori Hashino, Makoto Kuroda
EPI_ISL_684857, EPI_ISL_684858, EPI_ISL_684859, EPI_ISL_684860, EPI_ISL_684861, EPI_ISL_684862, EPI_ISL_684863, EPI_ISL_684864, EPI_ISL_684865, EPI_ISL_684866, EPI_ISL_684867, EPI_ISL_684868, EPI_ISL_684869, EPI_ISL_684870, EPI_ISL_684871, EPI_ISL_684872, EPI_ISL_684873, EPI_ISL_684874, EPI_ISL_684875, EPI_ISL_684876, EPI_ISL_684877, EPI_ISL_684878, EPI_ISL_684879, EPI_ISL_684880			
see above	Pathogen Genomics Center, National Institute of Infectious Diseases	Pathogen Genomics Center, National Institute of Infectious Diseases	Tsuyoshi Sekizuka, Kentaro Itokawa, Rina Tanaka, Masanori Hashino, Makoto Kuroda
EPI_ISL_684881, EPI_ISL_684882, EPI_ISL_684883, EPI_ISL_684884, EPI_ISL_684885, EPI_ISL_684886, EPI_ISL_684887, EPI_ISL_684888, EPI_ISL_684889, EPI_ISL_684890, EPI_ISL_684891, EPI_ISL_684892, EPI_ISL_684893, EPI_ISL_684894, EPI_ISL_684895, EPI_ISL_684896, EPI_ISL_684897, EPI_ISL_684898, EPI_ISL_684899, EPI_ISL_684900, EPI_ISL_684901, EPI_ISL_684902, EPI_ISL_684903, EPI_ISL_684904, EPI_ISL_684905, EPI_ISL_684906, EPI_ISL_684907, EPI_ISL_684908, EPI_ISL_684909, EPI_ISL_684910, EPI_ISL_684911, EPI_ISL_684912, EPI_ISL_684913, EPI_ISL_684914, EPI_ISL_684915, EPI_ISL_684916, EPI_ISL_684917, EPI_ISL_684918, EPI_ISL_684919, EPI_ISL_684920, EPI_ISL_684921, EPI_ISL_684922, EPI_ISL_684923, EPI_ISL_684924, EPI_ISL_684925, EPI_ISL_684926, EPI_ISL_684927, EPI_ISL_684928, EPI_ISL_684929, EPI_ISL_684930, EPI_ISL_684931, EPI_ISL_684932, EPI_ISL_684933, EPI_ISL_684934, EPI_ISL_684935, EPI_ISL_684936, EPI_ISL_684937, EPI_ISL_684938, EPI_ISL_684939, EPI_ISL_684940, EPI_ISL_684941, EPI_ISL_684942, EPI_ISL_684943, EPI_ISL_684944, EPI_ISL_684945, EPI_ISL_684946, EPI_ISL_684947, EPI_ISL_684948, EPI_ISL_684949, EPI_ISL_684950, EPI_ISL_684951, EPI_ISL_684952, EPI_ISL_684953, EPI_ISL_684954, EPI_ISL_684955, EPI_ISL_684956, EPI_ISL_684957, EPI_ISL_684958, EPI_ISL_684959, EPI_ISL_684960, EPI_ISL_684961, EPI_ISL_684962, EPI_ISL_684963, EPI_ISL_684964, EPI_ISL_684965, EPI_ISL_684966, EPI_ISL_684967, EPI_ISL_684968, EPI_ISL_684969, EPI_ISL_684970, EPI_ISL_684971, EPI_ISL_684972, EPI_ISL_684973, EPI_ISL_684974, EPI_ISL_684975, EPI_ISL_684976, EPI_ISL_684977, EPI_ISL_684978, EPI_ISL_684979, EPI_ISL_684980, EPI_ISL_684981, EPI_ISL_684982, EPI_ISL_684983, EPI_ISL_684984, EPI_ISL_684985, EPI_ISL_684986, EPI_ISL_684987, EPI_ISL_684988, EPI_ISL_684989, EPI_ISL_684990, EPI_ISL_684991, EPI_ISL_684992, EPI_ISL_684993, EPI_ISL_684994, EPI_ISL_684995, EPI_ISL_684996, EPI_ISL_684997, EPI_ISL_684998, EPI_ISL_684999, EPI_ISL_685000, EPI_ISL_685001, EPI_ISL_685002, EPI_ISL_685003, EPI_ISL_685004, EPI_ISL_685005, EPI_ISL_685006, EPI_ISL_685007, EPI_ISL_685008, EPI_ISL_685009, EPI_ISL_685010, EPI_ISL_685011, EPI_ISL_685012, EPI_ISL_685013, EPI_ISL_685014, EPI_ISL_685015, EPI_ISL_685016, EPI_ISL_685017, EPI_ISL_685018, EPI_ISL_685019, EPI_ISL_685020, EPI_ISL_685021, EPI_ISL_685022, EPI_ISL_685023, EPI_ISL_685024, EPI_ISL_685025, EPI_ISL_685026, EPI_ISL_685027, EPI_ISL_685028, EPI_ISL_685029, EPI_ISL_685030, EPI_ISL_685031, EPI_ISL_685032, EPI_ISL_685033, EPI_ISL_685034, EPI_ISL_685035, EPI_ISL_685036, EPI_ISL_685037, EPI_ISL_685038, EPI_ISL_685039, EPI_ISL_685040, EPI_ISL_685041, EPI_ISL_685042, EPI_ISL_685043, EPI_ISL_685044, EPI_ISL_685045, EPI_ISL_685046, EPI_ISL_685047, EPI_ISL_685048, EPI_ISL_685049, EPI_ISL_685050, EPI_ISL_685051, EPI_ISL_685052, EPI_ISL_685053, EPI_ISL_685054, EPI_ISL_685055, EPI_ISL_685056, EPI_ISL_685057, EPI_ISL_685058, EPI_ISL_685059, EPI_ISL_685060, EPI_ISL_685061, EPI_ISL_685062, EPI_ISL_685063, EPI_ISL_685064, EPI_ISL_685065, EPI_ISL_685066, EPI_ISL_685067, EPI_ISL_685068, EPI_ISL_685069, EPI_ISL_685070, EPI_ISL_685071, EPI_ISL_685072, EPI_ISL_685073, EPI_ISL_685074, EPI_ISL_685075, EPI_ISL_685076, EPI_ISL_685077, EPI_ISL_685078, EPI_ISL_685079, EPI_ISL_685080, EPI_ISL_685081, EPI_ISL_685082, EPI_ISL_685083, EPI_ISL_685084, EPI_ISL_685085, EPI_ISL_685086, EPI_ISL_685087, EPI_ISL_685088, EPI_ISL_685089, EPI_ISL_685090, EPI_ISL_685091, EPI_ISL_685092, EPI_ISL_685093, EPI_ISL_685094, EPI_ISL_685095, EPI_ISL_685096, EPI_ISL_685097, EPI_ISL_685098, EPI_ISL_685099, EPI_ISL_685100, EPI_ISL_685101, EPI_ISL_685102, EPI_ISL_685103, EPI_ISL_685104, EPI_ISL_685105, EPI_ISL_685106, EPI_ISL_685107, EPI_ISL_685108, EPI_ISL_685109, EPI_ISL_685110, EPI_ISL_685111, EPI_ISL_685112, EPI_ISL_685113, EPI_ISL_685114, EPI_ISL_685115, EPI_ISL_685116, EPI_ISL_685117, EPI_ISL_685118, EPI_ISL_685119, EPI_ISL_685120, EPI_ISL_685121, EPI_ISL_685122, EPI_ISL_685123, EPI_ISL_685124, EPI_ISL_685125, EPI_ISL_685126, EPI_ISL_685127, EPI_ISL_685128, EPI_ISL_685129, EPI_ISL_685130, EPI_ISL_685131, EPI_ISL_685132			

see above	Saitama Prefectural Institute of Public Health	Pathogen Genomics Center, National Institute of Infectious Diseases	Tsuyoshi Sekizuka, Kentaro Itokawa, Rina Tanaka, Masanori Hashino, Makoto Kuroda
EPI_ISL_685133, EPI_ISL_685134, EPI_ISL_685135, EPI_ISL_685136, EPI_ISL_685137, EPI_ISL_685138, EPI_ISL_685139, EPI_ISL_685140, EPI_ISL_685141, EPI_ISL_685142, EPI_ISL_685143, EPI_ISL_685144, EPI_ISL_685145, EPI_ISL_685146, EPI_ISL_685147, EPI_ISL_685148, EPI_ISL_685149, EPI_ISL_685150, EPI_ISL_685151, EPI_ISL_685152, EPI_ISL_685153, EPI_ISL_685154, EPI_ISL_685155, EPI_ISL_685156, EPI_ISL_685157, EPI_ISL_685158, EPI_ISL_685159, EPI_ISL_685160, EPI_ISL_685161, EPI_ISL_685162, EPI_ISL_685163, EPI_ISL_685164, EPI_ISL_685165, EPI_ISL_685166, EPI_ISL_685167, EPI_ISL_685168, EPI_ISL_685169, EPI_ISL_685170, EPI_ISL_685171, EPI_ISL_685172, EPI_ISL_685173, EPI_ISL_685174, EPI_ISL_685175, EPI_ISL_685176, EPI_ISL_685177, EPI_ISL_685178, EPI_ISL_685179, EPI_ISL_685180, EPI_ISL_685181, EPI_ISL_685182, EPI_ISL_685183, EPI_ISL_685184, EPI_ISL_685185, EPI_ISL_685186, EPI_ISL_685187, EPI_ISL_685188, EPI_ISL_685189, EPI_ISL_685190, EPI_ISL_685191, EPI_ISL_685192, EPI_ISL_685193, EPI_ISL_685194, EPI_ISL_685195, EPI_ISL_685196, EPI_ISL_685197, EPI_ISL_685198, EPI_ISL_685199, EPI_ISL_685200, EPI_ISL_685201, EPI_ISL_685202, EPI_ISL_685203, EPI_ISL_685204, EPI_ISL_685205, EPI_ISL_685206, EPI_ISL_685207, EPI_ISL_685208, EPI_ISL_685209, EPI_ISL_685210, EPI_ISL_685211, EPI_ISL_685212, EPI_ISL_685213, EPI_ISL_685214, EPI_ISL_685215, EPI_ISL_685216, EPI_ISL_685217, EPI_ISL_685218, EPI_ISL_685219			
see above	Pathogen Genomics Center, National Institute of Infectious Diseases	Pathogen Genomics Center, National Institute of Infectious Diseases	Tsuyoshi Sekizuka, Kentaro Itokawa, Rina Tanaka, Masanori Hashino, Makoto Kuroda
EPI_ISL_685220, EPI_ISL_685221, EPI_ISL_685222, EPI_ISL_685223, EPI_ISL_685224, EPI_ISL_685225, EPI_ISL_685226, EPI_ISL_685227, EPI_ISL_685228, EPI_ISL_685229, EPI_ISL_685230, EPI_ISL_685231, EPI_ISL_685232, EPI_ISL_685233, EPI_ISL_685234, EPI_ISL_685235, EPI_ISL_685236, EPI_ISL_685237			
see above	Tokyo Metropolitan Institute of Public Health	Pathogen Genomics Center, National Institute of Infectious Diseases	Tsuyoshi Sekizuka, Kentaro Itokawa, Rina Tanaka, Masanori Hashino, Makoto Kuroda
EPI_ISL_685238, EPI_ISL_685239, EPI_ISL_685240, EPI_ISL_685241, EPI_ISL_685242, EPI_ISL_685243, EPI_ISL_685244, EPI_ISL_685245, EPI_ISL_685246, EPI_ISL_685247, EPI_ISL_685248, EPI_ISL_685249, EPI_ISL_685250, EPI_ISL_685251, EPI_ISL_685252, EPI_ISL_685253, EPI_ISL_685254, EPI_ISL_685255, EPI_ISL_685256, EPI_ISL_685257, EPI_ISL_685258, EPI_ISL_685259, EPI_ISL_685260, EPI_ISL_685261, EPI_ISL_685262, EPI_ISL_685263, EPI_ISL_685264, EPI_ISL_685265, EPI_ISL_685266, EPI_ISL_685267, EPI_ISL_685268, EPI_ISL_685269, EPI_ISL_685270, EPI_ISL_685271, EPI_ISL_685272, EPI_ISL_685273, EPI_ISL_685274, EPI_ISL_685275, EPI_ISL_685276, EPI_ISL_685277, EPI_ISL_685278			
see above	Pathogen Genomics Center, National Institute of Infectious Diseases	Pathogen Genomics Center, National Institute of Infectious Diseases	Tsuyoshi Sekizuka, Kentaro Itokawa, Rina Tanaka, Masanori Hashino, Makoto Kuroda
EPI_ISL_685279, EPI_ISL_685280, EPI_ISL_685281	Gunma Prefectural Institute of Public Health and Environmental Sciences	Pathogen Genomics Center, National Institute of Infectious Diseases	Tsuyoshi Sekizuka, Kentaro Itokawa, Rina Tanaka, Masanori Hashino, Makoto Kuroda
EPI_ISL_685282, EPI_ISL_685283, EPI_ISL_685284, EPI_ISL_685285, EPI_ISL_685286, EPI_ISL_685287, EPI_ISL_685288, EPI_ISL_685289, EPI_ISL_685290, EPI_ISL_685291, EPI_ISL_685292, EPI_ISL_685293, EPI_ISL_685294, EPI_ISL_685295, EPI_ISL_685296			
see above	Pathogen Genomics Center, National Institute of Infectious Diseases	Pathogen Genomics Center, National Institute of Infectious Diseases	Tsuyoshi Sekizuka, Kentaro Itokawa, Rina Tanaka, Masanori Hashino, Makoto Kuroda
EPI_ISL_685297, EPI_ISL_685298, EPI_ISL_685299, EPI_ISL_685300, EPI_ISL_685301, EPI_ISL_685302, EPI_ISL_685303, EPI_ISL_685304, EPI_ISL_685305, EPI_ISL_685306, EPI_ISL_685307, EPI_ISL_685308, EPI_ISL_685309, EPI_ISL_685310			
see above	Niigata Prefectural Institute of Public Health and Environmental Sciences	Pathogen Genomics Center, National Institute of Infectious Diseases	Tsuyoshi Sekizuka, Kentaro Itokawa, Rina Tanaka, Masanori Hashino, Makoto Kuroda
EPI_ISL_685311, EPI_ISL_685312, EPI_ISL_685313, EPI_ISL_685314, EPI_ISL_685315, EPI_ISL_685316, EPI_ISL_685317, EPI_ISL_685318, EPI_ISL_685319, EPI_ISL_685320, EPI_ISL_685321, EPI_ISL_685322, EPI_ISL_685323, EPI_ISL_685324, EPI_ISL_685325, EPI_ISL_685326, EPI_ISL_685327, EPI_ISL_685328, EPI_ISL_685329, EPI_ISL_685330, EPI_ISL_685331, EPI_ISL_685332, EPI_ISL_685333, EPI_ISL_685334, EPI_ISL_685335, EPI_ISL_685336, EPI_ISL_685337, EPI_ISL_685338, EPI_ISL_685339, EPI_ISL_685340, EPI_ISL_685341, EPI_ISL_685342, EPI_ISL_685343, EPI_ISL_685344, EPI_ISL_685345, EPI_ISL_685346, EPI_ISL_685347, EPI_ISL_685348, EPI_ISL_685349, EPI_ISL_685350, EPI_ISL_685351, EPI_ISL_685352, EPI_ISL_685353, EPI_ISL_685354, EPI_ISL_685355, EPI_ISL_685356, EPI_ISL_685357, EPI_ISL_685358, EPI_ISL_685359, EPI_ISL_685360, EPI_ISL_685361, EPI_ISL_685362, EPI_ISL_685363, EPI_ISL_685364, EPI_ISL_685365, EPI_ISL_685366, EPI_ISL_685367, EPI_ISL_685368, EPI_ISL_685369, EPI_ISL_685370, EPI_ISL_685371, EPI_ISL_685372, EPI_ISL_685373, EPI_ISL_685374, EPI_ISL_685375, EPI_ISL_685376, EPI_ISL_685377, EPI_ISL_685378, EPI_ISL_685379, EPI_ISL_685380, EPI_ISL_685381, EPI_ISL_685382, EPI_ISL_685383, EPI_ISL_685384, EPI_ISL_685385, EPI_ISL_685386, EPI_ISL_685387, EPI_ISL_685388, EPI_ISL_685389, EPI_ISL_685390, EPI_ISL_685391, EPI_ISL_685392, EPI_ISL_685393, EPI_ISL_685394, EPI_ISL_685395, EPI_ISL_685396, EPI_ISL_685397, EPI_ISL_685398, EPI_ISL_685399, EPI_ISL_685400, EPI_ISL_685401, EPI_ISL_685402, EPI_ISL_685403, EPI_ISL_685404, EPI_ISL_685405, EPI_ISL_685406			
see above	Pathogen Genomics Center, National Institute of Infectious Diseases	Pathogen Genomics Center, National Institute of Infectious Diseases	Tsuyoshi Sekizuka, Kentaro Itokawa, Rina Tanaka, Masanori Hashino, Makoto Kuroda
EPI_ISL_685407, EPI_ISL_685408, EPI_ISL_685409, EPI_ISL_685410, EPI_ISL_685411, EPI_ISL_685412, EPI_ISL_685413, EPI_ISL_685414, EPI_ISL_685415, EPI_ISL_685416, EPI_ISL_685417, EPI_ISL_685418, EPI_ISL_685419, EPI_ISL_685420, EPI_ISL_685421, EPI_ISL_685422, EPI_ISL_685423, EPI_ISL_685424, EPI_ISL_685425, EPI_ISL_685426, EPI_ISL_685427, EPI_ISL_685428, EPI_ISL_685429, EPI_ISL_685430, EPI_ISL_685431, EPI_ISL_685432, EPI_ISL_685433, EPI_ISL_685434, EPI_ISL_685435, EPI_ISL_685436, EPI_ISL_685437, EPI_ISL_685438, EPI_ISL_685439, EPI_ISL_685440, EPI_ISL_685441, EPI_ISL_685442, EPI_ISL_685443, EPI_ISL_685444, EPI_ISL_685445, EPI_ISL_685446, EPI_ISL_685447, EPI_ISL_685448, EPI_ISL_685449, EPI_ISL_685450, EPI_ISL_685451, EPI_ISL_685452, EPI_ISL_685453, EPI_ISL_685454, EPI_ISL_685455, EPI_ISL_685456, EPI_ISL_685457, EPI_ISL_685458, EPI_ISL_685459, EPI_ISL_685460, EPI_ISL_685461, EPI_ISL_685462, EPI_ISL_685463, EPI_ISL_685464, EPI_ISL_685465, EPI_ISL_685466, EPI_ISL_685467, EPI_ISL_685468, EPI_ISL_685469, EPI_ISL_685470, EPI_ISL_685471, EPI_ISL_685472, EPI_ISL_685473, EPI_ISL_685474, EPI_ISL_685475, EPI_ISL_685476, EPI_ISL_685477, EPI_ISL_685478, EPI_ISL_685479, EPI_ISL_685480, EPI_ISL_685481, EPI_ISL_685482, EPI_ISL_685483, EPI_ISL_685484, EPI_ISL_685485, EPI_ISL_685486, EPI_ISL_685487, EPI_ISL_685488, EPI_ISL_685489, EPI_ISL_685490, EPI_ISL_685491, EPI_ISL_685492, EPI_ISL_685493, EPI_ISL_685494, EPI_ISL_685495, EPI_ISL_685496, EPI_ISL_685497			
see above	Tokyo Metropolitan Institute of Public Health	Pathogen Genomics Center, National Institute of Infectious Diseases	Tsuyoshi Sekizuka, Kentaro Itokawa, Rina Tanaka, Masanori Hashino, Makoto Kuroda
EPI_ISL_685498, EPI_ISL_685499, EPI_ISL_685500, EPI_ISL_685501, EPI_ISL_685502, EPI_ISL_685503, EPI_ISL_685504	Yamagata Prefectural Institute of Public Health	Pathogen Genomics Center, National Institute of Infectious Diseases	Tsuyoshi Sekizuka, Kentaro Itokawa, Rina Tanaka, Masanori Hashino, Makoto Kuroda
EPI_ISL_685505, EPI_ISL_685506, EPI_ISL_685507, EPI_ISL_685508, EPI_ISL_685509, EPI_ISL_685510, EPI_ISL_685511, EPI_ISL_685512, EPI_ISL_685513, EPI_ISL_685514, EPI_ISL_685515, EPI_ISL_685516, EPI_ISL_685517, EPI_ISL_685518, EPI_ISL_685519, EPI_ISL_685520, EPI_ISL_685521, EPI_ISL_685522, EPI_ISL_685523, EPI_ISL_685524, EPI_ISL_685525, EPI_ISL_685526, EPI_ISL_685527, EPI_ISL_685528, EPI_ISL_685529, EPI_ISL_685530, EPI_ISL_685531, EPI_ISL_685532, EPI_ISL_685533, EPI_ISL_685534, EPI_ISL_685535, EPI_ISL_685536, EPI_ISL_685537, EPI_ISL_685538, EPI_ISL_685539, EPI_ISL_685540, EPI_ISL_685541, EPI_ISL_685542, EPI_ISL_685543, EPI_ISL_685544, EPI_ISL_685545, EPI_ISL_685546, EPI_ISL_685547, EPI_ISL_685548, EPI_ISL_685549, EPI_ISL_685550, EPI_ISL_685551, EPI_ISL_685552, EPI_ISL_685553, EPI_ISL_685554, EPI_ISL_685555, EPI_ISL_685556, EPI_ISL_685557, EPI_ISL_685558, EPI_ISL_685559, EPI_ISL_685560, EPI_ISL_685561, EPI_ISL_685562, EPI_ISL_685563, EPI_ISL_685564, EPI_ISL_685565, EPI_ISL_685566, EPI_ISL_685567, EPI_ISL_685568, EPI_ISL_685569, EPI_ISL_685570, EPI_ISL_685571, EPI_ISL_685572, EPI_ISL_685573, EPI_ISL_685574, EPI_ISL_685575, EPI_ISL_685576, EPI_ISL_685577, EPI_ISL_685578, EPI_ISL_685579, EPI_ISL_685580, EPI_ISL_685581, EPI_ISL_685582, EPI_ISL_685583, EPI_ISL_685584, EPI_ISL_685585, EPI_ISL_685586, EPI_ISL_685587, EPI_ISL_685588, EPI_ISL_685589, EPI_ISL_685590, EPI_ISL_685591, EPI_ISL_685592, EPI_ISL_685593, EPI_ISL_685594, EPI_ISL_685595, EPI_ISL_685596, EPI_ISL_685597, EPI_ISL_685598, EPI_ISL_685599, EPI_ISL_685600, EPI_ISL_685601, EPI_ISL_685602, EPI_ISL_685603, EPI_ISL_685604, EPI_ISL_685605			
see above	Pathogen Genomics Center, National Institute of Infectious Diseases	Pathogen Genomics Center, National Institute of Infectious Diseases	Tsuyoshi Sekizuka, Kentaro Itokawa, Rina Tanaka, Masanori Hashino, Makoto Kuroda
EPI_ISL_685606, EPI_ISL_685607, EPI_ISL_685608, EPI_ISL_685609, EPI_ISL_685610, EPI_ISL_685611, EPI_ISL_685612, EPI_ISL_685613, EPI_ISL_685614, EPI_ISL_685615, EPI_ISL_685616			
see above	Tokyo Metropolitan Institute of Public Health	Pathogen Genomics Center, National Institute of Infectious Diseases	Tsuyoshi Sekizuka, Kentaro Itokawa, Rina Tanaka, Masanori Hashino, Makoto Kuroda
EPI_ISL_685617, EPI_ISL_685618, EPI_ISL_685619, EPI_ISL_685620, EPI_ISL_685621, EPI_ISL_685622, EPI_ISL_685623, EPI_ISL_685624, EPI_ISL_685625, EPI_ISL_685626, EPI_ISL_685627, EPI_ISL_685628, EPI_ISL_685629, EPI_ISL_685630, EPI_ISL_685631, EPI_ISL_685632, EPI_ISL_685633, EPI_ISL_685634, EPI_ISL_685635, EPI_ISL_685636, EPI_ISL_685637, EPI_ISL_685638, EPI_ISL_685639, EPI_ISL_685640, EPI_ISL_685641, EPI_ISL_685642, EPI_ISL_685643, EPI_ISL_685644, EPI_ISL_685645, EPI_ISL_685646, EPI_ISL_685647, EPI_ISL_685648, EPI_ISL_685649, EPI_ISL_685650, EPI_ISL_685651, EPI_ISL_685652, EPI_ISL_685653, EPI_ISL_685654, EPI_ISL_685655, EPI_ISL_685656, EPI_ISL_685657, EPI_ISL_685658, EPI_ISL_685659, EPI_ISL_685660, EPI_ISL_685661, EPI_ISL_685662, EPI_ISL_685663, EPI_ISL_685664, EPI_ISL_685665			
see above	Pathogen Genomics Center, National Institute of Infectious Diseases	Pathogen Genomics Center, National Institute of Infectious Diseases	Tsuyoshi Sekizuka, Kentaro Itokawa, Rina Tanaka, Masanori Hashino, Makoto Kuroda
EPI_ISL_685666, EPI_ISL_685667, EPI_ISL_685668, EPI_ISL_685669, EPI_ISL_685670, EPI_ISL_685671, EPI_ISL_685672, EPI_ISL_685673, EPI_ISL_685674, EPI_ISL_685675, EPI_ISL_685676, EPI_ISL_685677, EPI_ISL_685678, EPI_ISL_685679, EPI_ISL_685680, EPI_ISL_685681, EPI_ISL_685682, EPI_ISL_685683, EPI_ISL_685684, EPI_ISL_685685, EPI_ISL_685686, EPI_ISL_685687, EPI_ISL_685688, EPI_ISL_685689, EPI_ISL_685690, EPI_ISL_685691, EPI_ISL_685692, EPI_ISL_685693, EPI_ISL_685694			
see above	Tokyo Metropolitan Institute of Public Health	Pathogen Genomics Center, National Institute of Infectious Diseases	Tsuyoshi Sekizuka, Kentaro Itokawa, Rina Tanaka, Masanori Hashino, Makoto Kuroda
EPI_ISL_685695, EPI_ISL_685696, EPI_ISL_685697, EPI_ISL_685698, EPI_ISL_685699, EPI_ISL_685700, EPI_ISL_685701, EPI_ISL_685702, EPI_ISL_685703, EPI_ISL_685704, EPI_ISL_685705, EPI_ISL_685706, EPI_ISL_685707, EPI_ISL_685708, EPI_ISL_685709, EPI_ISL_685710, EPI_ISL_685711, EPI_ISL_685712, EPI_ISL_685713, EPI_ISL_685714, EPI_ISL_685715, EPI_ISL_685716, EPI_ISL_685717, EPI_ISL_685718, EPI_ISL_685719, EPI_ISL_685720, EPI_ISL_685721, EPI_ISL_685722, EPI_ISL_685723, EPI_ISL_685724, EPI_ISL_685725, EPI_ISL_685726, EPI_ISL_685727, EPI_ISL_685728, EPI_ISL_685729, EPI_ISL_685730, EPI_ISL_685731, EPI_ISL_685732, EPI_ISL_685733, EPI_ISL_685734, EPI_ISL_685735, EPI_ISL_685736, EPI_ISL_685737, EPI_ISL_685738, EPI_ISL_685739, EPI_ISL_685740, EPI_ISL_685741, EPI_ISL_685742, EPI_ISL_685743, EPI_ISL_685744, EPI_ISL_685745, EPI_ISL_685746, EPI_ISL_685747, EPI_ISL_685748, EPI_ISL_685749, EPI_ISL_685750, EPI_ISL_685751, EPI_ISL_685752, EPI_ISL_685753, EPI_ISL_685754, EPI_ISL_685755, EPI_ISL_685756, EPI_ISL_685757, EPI_ISL_685758, EPI_ISL_685759, EPI_ISL_685760, EPI_ISL_685761, EPI_ISL_685762, EPI_ISL_685763, EPI_ISL_685764, EPI_ISL_685765, EPI_ISL_685766, EPI_ISL_685767, EPI_ISL_685768, EPI_ISL_685769, EPI_ISL_685770, EPI_ISL_685771, EPI_ISL_685772, EPI_ISL_685773, EPI_ISL_685774, EPI_ISL_685775, EPI_ISL_685776, EPI_ISL_685777, EPI_ISL_685778, EPI_ISL_685779, EPI_ISL_685780, EPI_ISL_685781, EPI_ISL_685782, EPI_ISL_685783, EPI_ISL_685784, EPI_ISL_685785, EPI_ISL_685786, EPI_ISL_685787, EPI_ISL_685788, EPI_ISL_685789, EPI_ISL_685790, EPI_ISL_685791, EPI_ISL_685792, EPI_ISL_685793, EPI_ISL_685794, EPI_ISL_685795, EPI_ISL_685796, EPI_ISL_685797, EPI_ISL_685798, EPI_ISL_685799, EPI_ISL_685800, EPI_ISL_685801, EPI_ISL_685802, EPI_ISL_685803, EPI_ISL_685804, EPI_ISL_685805, EPI_ISL_685806, EPI_ISL_685807, EPI_ISL_685808, EPI_ISL_685809, EPI_ISL_685810, EPI_ISL_685811, EPI_ISL_685812, EPI_ISL_685813, EPI_ISL_685814, EPI_ISL_685815, EPI_ISL_685816, EPI_ISL_685817, EPI_ISL_685818, EPI_ISL_685819, EPI_ISL_685820, EPI_ISL_685821, EPI_ISL_685822, EPI_ISL_685823, EPI_ISL_685824, EPI_ISL_685825, EPI_ISL_685826, EPI_ISL_685827, EPI_ISL_685828, EPI_ISL_685829, EPI_ISL_685830, EPI_ISL_685831, EPI_ISL_685832, EPI_ISL_685833, EPI_ISL_685834, EPI_ISL_685835, EPI_ISL_685836, EPI_ISL_685837, EPI_ISL_685838, EPI_ISL_685839, EPI_ISL_685840, EPI_ISL_685841, EPI_ISL_685842, EPI_ISL_685843, EPI_ISL_685844, EPI_ISL_685845, EPI_ISL_685846, EPI_ISL_685847, EPI_ISL_685848, EPI_ISL_685849, EPI_ISL_685850, EPI_ISL_685851, EPI_ISL_685852, EPI_ISL_685853, EPI_ISL_685854, EPI_ISL_685855, EPI_ISL_685856, EPI_ISL_685857, EPI_ISL_685858, EPI_ISL_685859, EPI_ISL_685860, EPI_ISL_685861, EPI_ISL_685862, EPI_ISL_685863, EPI_ISL_685864, EPI_ISL_685865, EPI_ISL_685866, EPI_ISL_685867, EPI_ISL_685868, EPI_ISL_685869, EPI_ISL_685870, EPI_ISL_685871, EPI_ISL_685872, EPI_ISL_685873, EPI_ISL_685874,			

EPI_ISL_685875, EPI_ISL_685876, EPI_ISL_685877, EPI_ISL_685878, EPI_ISL_685879, EPI_ISL_685880, EPI_ISL_685881, EPI_ISL_685882, EPI_ISL_685883, EPI_ISL_685884, EPI_ISL_685885, EPI_ISL_685886, EPI_ISL_685887, EPI_ISL_685888, EPI_ISL_685889, EPI_ISL_685890, EPI_ISL_685891, EPI_ISL_685892, EPI_ISL_685893, EPI_ISL_685894, EPI_ISL_685895, EPI_ISL_685896, EPI_ISL_685897, EPI_ISL_685898, EPI_ISL_685899, EPI_ISL_685900, EPI_ISL_685901, EPI_ISL_685902, EPI_ISL_685903, EPI_ISL_685904, EPI_ISL_685905, EPI_ISL_685906, EPI_ISL_685907, EPI_ISL_685908, EPI_ISL_685909, EPI_ISL_685910, EPI_ISL_685911, EPI_ISL_685912, EPI_ISL_685913, EPI_ISL_685914, EPI_ISL_685915, EPI_ISL_685916, EPI_ISL_685917

see above	Pathogen Genomics Center, National Institute of Infectious Diseases	Pathogen Genomics Center, National Institute of Infectious Diseases	Tsuyoshi Sekizuka, Kentaro Itokawa, Rina Tanaka, Masanori Hashino, Makoto Kuroda
EPI_ISL_685918, EPI_ISL_685919, EPI_ISL_685920, EPI_ISL_685921, EPI_ISL_685922, EPI_ISL_685923, EPI_ISL_685924	Fukuoka Institute of Health and Environmental Sciences	Pathogen Genomics Center, National Institute of Infectious Diseases	Tsuyoshi Sekizuka, Kentaro Itokawa, Rina Tanaka, Masanori Hashino, Makoto Kuroda

EPI_ISL_685925, EPI_ISL_685926, EPI_ISL_685927, EPI_ISL_685928, EPI_ISL_685929, EPI_ISL_685930, EPI_ISL_685931, EPI_ISL_685932, EPI_ISL_685933, EPI_ISL_685934, EPI_ISL_685935, EPI_ISL_685936, EPI_ISL_685937, EPI_ISL_685938, EPI_ISL_685939, EPI_ISL_685940, EPI_ISL_685941, EPI_ISL_685942, EPI_ISL_685943, EPI_ISL_685944, EPI_ISL_685945, EPI_ISL_685946, EPI_ISL_685947, EPI_ISL_685948, EPI_ISL_685949, EPI_ISL_685950, EPI_ISL_685951, EPI_ISL_685952, EPI_ISL_685953, EPI_ISL_685954, EPI_ISL_685955, EPI_ISL_685956, EPI_ISL_685957, EPI_ISL_685958, EPI_ISL_685959, EPI_ISL_685960, EPI_ISL_685961, EPI_ISL_685962

see above	Pathogen Genomics Center, National Institute of Infectious Diseases	Pathogen Genomics Center, National Institute of Infectious Diseases	Tsuyoshi Sekizuka, Kentaro Itokawa, Rina Tanaka, Masanori Hashino, Makoto Kuroda

EPI_ISL_685963, EPI_ISL_685964, EPI_ISL_685965, EPI_ISL_685966, EPI_ISL_685967, EPI_ISL_685968, EPI_ISL_685969, EPI_ISL_685970, EPI_ISL_685971, EPI_ISL_685972, EPI_ISL_685973, EPI_ISL_685974, EPI_ISL_685975, EPI_ISL_685976, EPI_ISL_685977, EPI_ISL_685978, EPI_ISL_685979, EPI_ISL_685980, EPI_ISL_685981, EPI_ISL_685982, EPI_ISL_685983, EPI_ISL_685984, EPI_ISL_685985, EPI_ISL_685986, EPI_ISL_685987

see above	Tokyo Metropolitan Institute of Public Health	Pathogen Genomics Center, National Institute of Infectious Diseases	Tsuyoshi Sekizuka, Kentaro Itokawa, Rina Tanaka, Masanori Hashino, Makoto Kuroda

EPI_ISL_685988, EPI_ISL_685989, EPI_ISL_685990, EPI_ISL_685991, EPI_ISL_685992, EPI_ISL_685993, EPI_ISL_685994, EPI_ISL_685995, EPI_ISL_685996, EPI_ISL_685997, EPI_ISL_685998, EPI_ISL_685999, EPI_ISL_686000, EPI_ISL_686001, EPI_ISL_686002, EPI_ISL_686003, EPI_ISL_686004, EPI_ISL_686005, EPI_ISL_686006, EPI_ISL_686007, EPI_ISL_686008, EPI_ISL_686009, EPI_ISL_686010, EPI_ISL_686011, EPI_ISL_686012, EPI_ISL_686013, EPI_ISL_686014, EPI_ISL_686015, EPI_ISL_686016, EPI_ISL_686017, EPI_ISL_686018, EPI_ISL_686019, EPI_ISL_686020, EPI_ISL_686021, EPI_ISL_686022, EPI_ISL_686023, EPI_ISL_686024, EPI_ISL_686025, EPI_ISL_686026, EPI_ISL_686027, EPI_ISL_686028, EPI_ISL_686029, EPI_ISL_686030, EPI_ISL_686031, EPI_ISL_686032, EPI_ISL_686033, EPI_ISL_686034, EPI_ISL_686035, EPI_ISL_686036, EPI_ISL_686037, EPI_ISL_686038, EPI_ISL_686039, EPI_ISL_686040, EPI_ISL_686041, EPI_ISL_686042, EPI_ISL_686043, EPI_ISL_686044, EPI_ISL_686045, EPI_ISL_686046, EPI_ISL_686047, EPI_ISL_686048, EPI_ISL_686049, EPI_ISL_686050, EPI_ISL_686051, EPI_ISL_686052, EPI_ISL_686053, EPI_ISL_686054, EPI_ISL_686055, EPI_ISL_686056, EPI_ISL_686057, EPI_ISL_686058, EPI_ISL_686059, EPI_ISL_686060, EPI_ISL_686061, EPI_ISL_686062, EPI_ISL_686063, EPI_ISL_686064, EPI_ISL_686065, EPI_ISL_686066, EPI_ISL_686067, EPI_ISL_686068, EPI_ISL_686069, EPI_ISL_686070, EPI_ISL_686071, EPI_ISL_686072, EPI_ISL_686073, EPI_ISL_686074, EPI_ISL_686075, EPI_ISL_686076, EPI_ISL_686077, EPI_ISL_686078, EPI_ISL_686079, EPI_ISL_686080, EPI_ISL_686081, EPI_ISL_686082

see above	Pathogen Genomics Center, National Institute of Infectious Diseases	Pathogen Genomics Center, National Institute of Infectious Diseases	Tsuyoshi Sekizuka, Kentaro Itokawa, Rina Tanaka, Masanori Hashino, Makoto Kuroda

EPI_ISL_686083, EPI_ISL_686084, EPI_ISL_686085, EPI_ISL_686086, EPI_ISL_686087, EPI_ISL_686088, EPI_ISL_686089, EPI_ISL_686090, EPI_ISL_686091, EPI_ISL_686092, EPI_ISL_686093, EPI_ISL_686094, EPI_ISL_686095, EPI_ISL_686096, EPI_ISL_686097, EPI_ISL_686098, EPI_ISL_686099, EPI_ISL_686100, EPI_ISL_686101, EPI_ISL_686102, EPI_ISL_686103, EPI_ISL_686104, EPI_ISL_686105, EPI_ISL_686106, EPI_ISL_686107

see above	Saitama Prefectural Institute of Public Health	Pathogen Genomics Center, National Institute of Infectious Diseases	Tsuyoshi Sekizuka, Kentaro Itokawa, Rina Tanaka, Masanori Hashino, Makoto Kuroda

EPI_ISL_686109, EPI_ISL_686110, EPI_ISL_686111, EPI_ISL_686112, EPI_ISL_686113, EPI_ISL_686114, EPI_ISL_686115, EPI_ISL_686116, EPI_ISL_686117, EPI_ISL_686118, EPI_ISL_686119, EPI_ISL_686120, EPI_ISL_686121, EPI_ISL_686122, EPI_ISL_686123, EPI_ISL_686124, EPI_ISL_686125, EPI_ISL_686126, EPI_ISL_686127, EPI_ISL_686128, EPI_ISL_686129, EPI_ISL_686130, EPI_ISL_686131, EPI_ISL_686132, EPI_ISL_686133, EPI_ISL_686134, EPI_ISL_686135, EPI_ISL_686136, EPI_ISL_686137, EPI_ISL_686138, EPI_ISL_686139, EPI_ISL_686140, EPI_ISL_686141, EPI_ISL_686142, EPI_ISL_686143, EPI_ISL_686144, EPI_ISL_686145, EPI_ISL_686146, EPI_ISL_686147, EPI_ISL_686148, EPI_ISL_686149, EPI_ISL_686150, EPI_ISL_686151, EPI_ISL_686152, EPI_ISL_686153, EPI_ISL_686154, EPI_ISL_686155, EPI_ISL_686156, EPI_ISL_686157, EPI_ISL_686158, EPI_ISL_686159, EPI_ISL_686160, EPI_ISL_686161, EPI_ISL_686162, EPI_ISL_686163, EPI_ISL_686164, EPI_ISL_686165, EPI_ISL_686166, EPI_ISL_686167, EPI_ISL_686168, EPI_ISL_686169, EPI_ISL_686170, EPI_ISL_686171, EPI_ISL_686172, EPI_ISL_686173, EPI_ISL_686174, EPI_ISL_686175, EPI_ISL_686176, EPI_ISL_686177, EPI_ISL_686178, EPI_ISL_686179, EPI_ISL_686180, EPI_ISL_686181, EPI_ISL_686182, EPI_ISL_686183, EPI_ISL_686184, EPI_ISL_686185, EPI_ISL_686186, EPI_ISL_686187, EPI_ISL_686188, EPI_ISL_686189, EPI_ISL_686190, EPI_ISL_686191, EPI_ISL_686192, EPI_ISL_686193, EPI_ISL_686194, EPI_ISL_686195, EPI_ISL_686196, EPI_ISL_686197, EPI_ISL_686198, EPI_ISL_686199, EPI_ISL_686200, EPI_ISL_686201, EPI_ISL_686202, EPI_ISL_686203, EPI_ISL_686204, EPI_ISL_686205, EPI_ISL_686206, EPI_ISL_686207, EPI_ISL_686208, EPI_ISL_686209, EPI_ISL_686210, EPI_ISL_686211, EPI_ISL_686212, EPI_ISL_686213, EPI_ISL_686214, EPI_ISL_686215, EPI_ISL_686216, EPI_ISL_686217, EPI_ISL_686218, EPI_ISL_686219, EPI_ISL_686220, EPI_ISL_686221, EPI_ISL_686222, EPI_ISL_686223, EPI_ISL_686224, EPI_ISL_686225, EPI_ISL_686226, EPI_ISL_686227, EPI_ISL_686228, EPI_ISL_686229, EPI_ISL_686230, EPI_ISL_686231, EPI_ISL_686232, EPI_ISL_686233, EPI_ISL_686234, EPI_ISL_686235, EPI_ISL_686236, EPI_ISL_686237, EPI_ISL_686238, EPI_ISL_686239, EPI_ISL_686240, EPI_ISL_686241, EPI_ISL_686242, EPI_ISL_686243, EPI_ISL_686244, EPI_ISL_686245, EPI_ISL_686246, EPI_ISL_686247, EPI_ISL_686248, EPI_ISL_686249, EPI_ISL_686250, EPI_ISL_686251, EPI_ISL_686252, EPI_ISL_686253, EPI_ISL_686254, EPI_ISL_686255, EPI_ISL_686256, EPI_ISL_686257, EPI_ISL_686258, EPI_ISL_686259, EPI_ISL_686260, EPI_ISL_686261, EPI_ISL_686262, EPI_ISL_686263, EPI_ISL_686264, EPI_ISL_686265, EPI_ISL_686266, EPI_ISL_686267, EPI_ISL_686268, EPI_ISL_686269, EPI_ISL_686270, EPI_ISL_686271, EPI_ISL_686272, EPI_ISL_686273, EPI_ISL_686274, EPI_ISL_686275, EPI_ISL_686276, EPI_ISL_686277, EPI_ISL_686278, EPI_ISL_686279, EPI_ISL_686280, EPI_ISL_686281, EPI_ISL_686282, EPI_ISL_686283, EPI_ISL_686284, EPI_ISL_686285, EPI_ISL_686286, EPI_ISL_686287, EPI_ISL_686288, EPI_ISL_686289, EPI_ISL_686290, EPI_ISL_686291, EPI_ISL_686292, EPI_ISL_686293, EPI_ISL_686294, EPI_ISL_686295, EPI_ISL_686296, EPI_ISL_686297, EPI_ISL_686298, EPI_ISL_686299, EPI_ISL_686300, EPI_ISL_686301, EPI_ISL_686302, EPI_ISL_686303, EPI_ISL_686304, EPI_ISL_686305, EPI_ISL_686306, EPI_ISL_686307, EPI_ISL_686308, EPI_ISL_686309, EPI_ISL_686310, EPI_ISL_686311, EPI_ISL_686312, EPI_ISL_686313, EPI_ISL_686314, EPI_ISL_686315, EPI_ISL_686316, EPI_ISL_686317, EPI_ISL_686318, EPI_ISL_686319, EPI_ISL_686320, EPI_ISL_686321, EPI_ISL_686322, EPI_ISL_686323, EPI_ISL_686324, EPI_ISL_686325, EPI_ISL_686326, EPI_ISL_686327, EPI_ISL_686328, EPI_ISL_686329, EPI_ISL_686330, EPI_ISL_686331, EPI_ISL_686332, EPI_ISL_686333, EPI_ISL_686334, EPI_ISL_686335, EPI_ISL_686336, EPI_ISL_686337, EPI_ISL_686338, EPI_ISL_686339, EPI_ISL_686340, EPI_ISL_686341, EPI_ISL_686342, EPI_ISL_686343, EPI_ISL_686344, EPI_ISL_686345, EPI_ISL_686346, EPI_ISL_686347, EPI_ISL_686348, EPI_ISL_686349, EPI_ISL_686350, EPI_ISL_686351, EPI_ISL_686352, EPI_ISL_686353, EPI_ISL_686354, EPI_ISL_686355, EPI_ISL_686356, EPI_ISL_686357, EPI_ISL_686358, EPI_ISL_686359, EPI_ISL_686360, EPI_ISL_686361, EPI_ISL_686362, EPI_ISL_686363, EPI_ISL_686364, EPI_ISL_686365, EPI_ISL_686366, EPI_ISL_686367, EPI_ISL_686368, EPI_ISL_686369, EPI_ISL_686370, EPI_ISL_686371, EPI_ISL_686372, EPI_ISL_686373, EPI_ISL_686374, EPI_ISL_686375, EPI_ISL_686376, EPI_ISL_686377, EPI_ISL_686378, EPI_ISL_686379, EPI_ISL_686380, EPI_ISL_686381, EPI_ISL_686382, EPI_ISL_686383, EPI_ISL_686384, EPI_ISL_686385, EPI_ISL_686386, EPI_ISL_686387, EPI_ISL_686388, EPI_ISL_686389, EPI_ISL_686390, EPI_ISL_686391, EPI_ISL_686392, EPI_ISL_686393, EPI_ISL_686394, EPI_ISL_686395, EPI_ISL_686396, EPI_ISL_686397, EPI_ISL_686398, EPI_ISL_686399, EPI_ISL_686400, EPI_ISL_686401, EPI_ISL_686402, EPI_ISL_686403, EPI_ISL_686404, EPI_ISL_686405, EPI_ISL_686406, EPI_ISL_686407, EPI_ISL_686408, EPI_ISL_686409, EPI_ISL_686410, EPI_ISL_686411, EPI_ISL_686412, EPI_ISL_686413, EPI_ISL_686414, EPI_ISL_686415, EPI_ISL_686416, EPI_ISL_686417, EPI_ISL_686418, EPI_ISL_686419, EPI_ISL_686420, EPI_ISL_686421, EPI_ISL_686422, EPI_ISL_686423, EPI_ISL_686424, EPI_ISL_686425, EPI_ISL_686426, EPI_ISL_686427, EPI_ISL_686428, EPI_ISL_686429, EPI_ISL_686430, EPI_ISL_686431, EPI_ISL_686432, EPI_ISL_686433, EPI_ISL_686434, EPI_ISL_686435, EPI_ISL_686436, EPI_ISL_686437, EPI_ISL_686438, EPI_ISL_686439, EPI_ISL_686440, EPI_ISL_686441, EPI_ISL_686442, EPI_ISL_686443, EPI_ISL_686444, EPI_ISL_686445, EPI_ISL_686446, EPI_ISL_686447, EPI_ISL_686448, EPI_ISL_686449, EPI_ISL_686450, EPI_ISL_686451, EPI_ISL_686452, EPI_ISL_686453, EPI_ISL_686454, EPI_ISL_686455, EPI_ISL_686456, EPI_ISL_686457, EPI_ISL_686458, EPI_ISL_686459, EPI_ISL_686460, EPI_ISL_686461, EPI_ISL_686462, EPI_ISL_686463, EPI_ISL_686464, EPI_ISL_686465, EPI_ISL_686466, EPI_ISL_686467, EPI_ISL_686468, EPI_ISL_686469, EPI_ISL_686470, EPI_ISL_686471, EPI_ISL_686472, EPI_ISL_686473, EPI_ISL_686474, EPI_ISL_686475, EPI_ISL_686476, EPI_ISL_686477, EPI_ISL_686478, EPI_ISL_686479, EPI_ISL_686480, EPI_ISL_686481, EPI_ISL_686482, EPI_ISL_686483, EPI_ISL_686484, EPI_ISL_686485, EPI_ISL_686486, EPI_ISL_686487, EPI_ISL_686488, EPI_ISL_686489, EPI_ISL_686490, EPI_ISL_686491, EPI_ISL_686492, EPI_ISL_686493, EPI_ISL_686494, EPI_ISL_686495, EPI_ISL_686496, EPI_ISL_686497, EPI_ISL_686498, EPI_ISL_686499, EPI_ISL_686500, EPI_ISL_686501, EPI_ISL_686502, EPI_ISL_686503, EPI_ISL_686504, EPI_ISL_686505, EPI_ISL_686506, EPI_ISL_686507, EPI_ISL_686508, EPI_ISL_686509, EPI_ISL_686510, EPI_ISL_686511, EPI_ISL_686512, EPI_ISL_686513, EPI_ISL_686514, EPI_ISL_686515, EPI_ISL_686516, EPI_ISL_686517, EPI_ISL_686518, EPI_ISL_686519, EPI_ISL_686520

see above	Pathogen Genomics Center, National Institute of Infectious Diseases	Pathogen Genomics Center, National Institute of Infectious Diseases	Tsuyoshi Sekizuka, Kentaro Itokawa, Rina Tanaka, Masanori Hashino, Makoto Kuroda

EPI_ISL_686521, EPI_ISL_686522, EPI_ISL_686523, EPI_ISL_686524, EPI_ISL_686525, EPI_ISL_686526, EPI_ISL_686527, EPI_ISL_686528, EPI_ISL_686529, EPI_ISL_686530, EPI_ISL_686531, EPI_ISL_686532, EPI_ISL_686533, EPI_ISL_686534

see above	Sakai City Institute of Public Health	Pathogen Genomics Center, National Institute of Infectious Diseases	Tsuyoshi Sekizuka, Kentaro Itokawa, Rina Tanaka, Masanori Hashino, Makoto Kuroda

EPI_ISL_686612, EPI_ISL_686613, EPI_ISL_686614, EPI_ISL_686615, EPI_ISL_686616, EPI_ISL_686617, EPI_ISL_686618, EPI_ISL_686619, EPI_ISL_686620, EPI_ISL_686621, EPI_ISL_686622, EPI_ISL_686623, EPI_ISL_686624, EPI_ISL_686625, EPI_ISL_686626, EPI_ISL_686627, EPI_ISL_686628, EPI_ISL_686629, EPI_ISL_686630, EPI_ISL_686631, EPI_ISL_686632, EPI_ISL_686633, EPI_ISL_686634, EPI_ISL_686635, EPI_ISL_686636, EPI_ISL_686637, EPI_ISL_686638, EPI_ISL_686639, EPI_ISL_686640, EPI_ISL_686641, EPI_ISL_686642, EPI_ISL_686643, EPI_ISL_686644, EPI_ISL_686645, EPI_ISL_686646, EPI_ISL_686647, EPI_ISL_686648, EPI_ISL_686649, EPI_ISL_686650, EPI_ISL_686651, EPI_ISL_686652, EPI_ISL_686653, EPI_ISL_686654, EPI_ISL_686655, EPI_ISL_686656, EPI_ISL_686657, EPI_ISL_686658

see above	Pathogen Genomics Center, National Institute of Infectious Diseases	Pathogen Genomics Center, National Institute of Infectious Diseases	Tsuyoshi Sekizuka, Kentaro Itokawa, Rina Tanaka, Masanori Hashino, Makoto Kuroda

EPI_ISL_686659, EPI_ISL_686660, EPI_ISL_686661, EPI_ISL_686662, EPI_ISL_686663, EPI_ISL_686664, EPI_ISL_686665, EPI_ISL_686666, EPI_ISL_686667, EPI_ISL_686668, EPI_ISL_686669, EPI_ISL_686670, EPI_ISL_686671, EPI_ISL_686672, EPI_ISL_686673, EPI_ISL_686674, EPI_ISL_686675, EPI_ISL_686676, EPI_ISL_686677, EPI_ISL_686678, EPI_ISL_686679, EPI_ISL_686680, EPI_ISL_686681, EPI_ISL_686682, EPI_ISL_686683, EPI_ISL_686684, EPI_ISL_686685, EPI_ISL_686686, EPI_ISL_686687, EPI_ISL_686688, EPI_ISL_686689, EPI_ISL_686690, EPI_ISL_686691, EPI_ISL_686692, EPI_ISL_686693, EPI_ISL_686694, EPI_ISL_686695, EPI_ISL_686696, EPI_ISL_686697, EPI_ISL_686698, EPI_ISL_686699, EPI_ISL_686700, EPI_ISL_686701, EPI_ISL_686702, EPI_ISL_686703, EPI_ISL_686704, EPI_ISL_686705, EPI_ISL_686706, EPI_ISL_686707, EPI_ISL_686708, EPI_ISL_686709, EPI_ISL_686710, EPI_ISL_686711, EPI_ISL_686712, EPI_ISL_686713, EPI_ISL_686714, EPI_ISL_686715, EPI_ISL_686716, EPI_ISL_686717, EPI_ISL_686718, EPI_ISL_686719, EPI_ISL_686720, EPI_ISL_686721, EPI_ISL_686722, EPI_ISL_686723, EPI_ISL_686724, EPI_ISL_686725, EPI_ISL_686726, EPI_ISL_686727, EPI_ISL_686728, EPI_ISL_686729, EPI_ISL_686730, EPI_ISL_686731, EPI_ISL_686732, EPI_ISL_686733, EPI_ISL_686734, EPI_ISL_686735, EPI_ISL_686736, EPI_ISL_686737, EPI_ISL_686738, EPI_ISL_686739, EPI_ISL_686740, EPI_ISL_686741, EPI_ISL_686742, EPI_ISL_686743, EPI_ISL_686744, EPI_ISL_686745, EPI_ISL_686746, EPI_ISL_686747, EPI_ISL_686748, EPI_ISL_686749, EPI_ISL_686750, EPI_ISL_686751, EPI_ISL_686752, EPI_ISL_686753, EPI_ISL_686754, EPI_ISL_686755, EPI_ISL_686756, EPI_ISL_686757, EPI_ISL_686758, EPI_ISL_686759, EPI_ISL_686760, EPI_ISL_686761, EPI_ISL_686762, EPI_ISL_686763, EPI_ISL_686764, EPI_ISL_686765, EPI_ISL_686766, EPI_ISL_686767, EPI_ISL_686768, EPI_ISL_686769, EPI_ISL_686770, EPI_ISL_686771, EPI_ISL_686772, EPI_ISL_686773, EPI_ISL_686774, EPI_ISL_686775, EPI_ISL_686776, EPI_ISL_686777, EPI_ISL_686778, EPI_ISL_686779, EPI_ISL_686780, EPI_ISL_686781, EPI_ISL_686782, EPI_ISL_686783, EPI_ISL_686784, EPI_ISL_686785, EPI_ISL_686786, EPI_ISL_686787, EPI_ISL_686788, EPI_ISL_686789, EPI_ISL_686790, EPI_ISL_686791, EPI_ISL_686792, EPI_ISL_686793, EPI_ISL_686794, EPI_ISL_686795, EPI_ISL_686796, EPI_ISL_686797, EPI_ISL_686798, EPI_ISL_686799, EPI_ISL_686800, EPI_ISL_686801, EPI_ISL_686802, EPI_ISL_686803, EPI_ISL_686804, EPI_ISL_686805, EPI_ISL_686806, EPI_ISL_686807, EPI_ISL_686808, EPI_ISL_686809, EPI_ISL_686810, EPI_ISL_686811, EPI_ISL_686812, EPI_ISL_686813, EPI_ISL_686814, EPI_ISL_686815, EPI_ISL_686816, EPI_ISL_686817, EPI_ISL_686818, EPI_ISL_686819, EPI_ISL_686820, EPI_ISL_686821, EPI_ISL_686822, EPI_ISL_686823, EPI_ISL_686824, EPI_ISL_686825, EPI_ISL_686826, EPI_ISL_686827, EPI_ISL_686828, EPI_ISL_686829, EPI_ISL_686830, EPI_ISL_686831, EPI_ISL_686832, EPI_ISL_686833, EPI_ISL_686834, EPI_ISL_686835, EPI_ISL_686836, EPI_ISL_686837, EPI_ISL_686838, EPI_ISL_686839, EPI_ISL_686840, EPI_ISL_686841, EPI_ISL_686842, EPI_ISL_686843, EPI_ISL_686844

see above	Chiba Prefectural Institute of Public Health	Pathogen Genomics Center, National Institute of Infectious Diseases	Tsuyoshi Sekizuka, Kentaro Itokawa, Rina Tanaka, Masanori Hashino, Makoto Kuroda

EPI_ISL_686845, EPI_ISL_686846, EPI_ISL_686847, EPI_ISL_686848, EPI_ISL_686849, EPI_ISL_686850, EPI_ISL_686851, EPI_ISL_686852, EPI_ISL_686853, EPI_ISL_686854, EPI_ISL_686855, EPI_ISL_686856, EPI_ISL_686857, EPI_ISL_686858, EPI_ISL_686859, EPI_ISL_686860, EPI_ISL_686861, EPI_ISL_686862, EPI_ISL_686863, EPI_ISL_686864, EPI_ISL_686865, EPI_ISL_686866, EPI_ISL_686867, EPI_ISL_686868, EPI_ISL_686869, EPI_ISL_686870, EPI_ISL_686871, EPI_ISL_686872, EPI_ISL_686873, EPI_ISL_686874, EPI_ISL_686875, EPI_ISL_686876, EPI_ISL_686877, EPI_ISL_686878, EPI_ISL_686879, EPI_ISL_686880,

EPI_ISL_686881, EPI_ISL_686882, EPI_ISL_686883, EPI_ISL_686884, EPI_ISL_686885, EPI_ISL_686886, EPI_ISL_686887, EPI_ISL_686888, EPI_ISL_686889, EPI_ISL_686890, EPI_ISL_686891, EPI_ISL_686892, EPI_ISL_686893, EPI_ISL_686894, EPI_ISL_686895, EPI_ISL_686896, EPI_ISL_686897, EPI_ISL_686898, EPI_ISL_686899, EPI_ISL_686900, EPI_ISL_686901, EPI_ISL_686902, EPI_ISL_686903, EPI_ISL_686904, EPI_ISL_686905, EPI_ISL_686906, EPI_ISL_686907, EPI_ISL_686908, EPI_ISL_686909, EPI_ISL_686910, EPI_ISL_686911, EPI_ISL_686912, EPI_ISL_686913, EPI_ISL_686914, EPI_ISL_686915, EPI_ISL_686916, EPI_ISL_686917, EPI_ISL_686918, EPI_ISL_686919, EPI_ISL_686920, EPI_ISL_686921, EPI_ISL_686922, EPI_ISL_686923, EPI_ISL_686924, EPI_ISL_686925, EPI_ISL_686926, EPI_ISL_686927, EPI_ISL_686928, EPI_ISL_686929, EPI_ISL_686930, EPI_ISL_686931, EPI_ISL_686932, EPI_ISL_686933, EPI_ISL_686934, EPI_ISL_686935, EPI_ISL_686936, EPI_ISL_686937, EPI_ISL_686938, EPI_ISL_686939, EPI_ISL_686940, EPI_ISL_686941, EPI_ISL_686942, EPI_ISL_686943, EPI_ISL_686944, EPI_ISL_686945, EPI_ISL_686946, EPI_ISL_686947, EPI_ISL_686948, EPI_ISL_686949, EPI_ISL_686950, EPI_ISL_686951, EPI_ISL_686952, EPI_ISL_686953, EPI_ISL_686954, EPI_ISL_686955, EPI_ISL_686956, EPI_ISL_686957, EPI_ISL_686958, EPI_ISL_686959, EPI_ISL_686960, EPI_ISL_686961, EPI_ISL_686962, EPI_ISL_686963, EPI_ISL_686964, EPI_ISL_686965, EPI_ISL_686966, EPI_ISL_686967, EPI_ISL_686968, EPI_ISL_686969, EPI_ISL_686970, EPI_ISL_686971, EPI_ISL_686972, EPI_ISL_686973, EPI_ISL_686974, EPI_ISL_686975, EPI_ISL_686976, EPI_ISL_686977, EPI_ISL_686978, EPI_ISL_686979, EPI_ISL_686980, EPI_ISL_686981, EPI_ISL_686982, EPI_ISL_686983, EPI_ISL_686984, EPI_ISL_686985, EPI_ISL_686986, EPI_ISL_686987, EPI_ISL_686988, EPI_ISL_686989, EPI_ISL_686990, EPI_ISL_686991, EPI_ISL_686992, EPI_ISL_686993, EPI_ISL_686994, EPI_ISL_686995, EPI_ISL_686996, EPI_ISL_686997, EPI_ISL_686998, EPI_ISL_686999, EPI_ISL_687000, EPI_ISL_687001, EPI_ISL_687002, EPI_ISL_687003, EPI_ISL_687004, EPI_ISL_687005, EPI_ISL_687006, EPI_ISL_687007, EPI_ISL_687008, EPI_ISL_687009, EPI_ISL_687010, EPI_ISL_687011, EPI_ISL_687012, EPI_ISL_687013, EPI_ISL_687014, EPI_ISL_687015, EPI_ISL_687016, EPI_ISL_687017, EPI_ISL_687018, EPI_ISL_687019, EPI_ISL_687020, EPI_ISL_687021, EPI_ISL_687022, EPI_ISL_687023, EPI_ISL_687024, EPI_ISL_687025, EPI_ISL_687026, EPI_ISL_687027, EPI_ISL_687028, EPI_ISL_687029, EPI_ISL_687030, EPI_ISL_687031, EPI_ISL_687032, EPI_ISL_687033, EPI_ISL_687034, EPI_ISL_687035, EPI_ISL_687036, EPI_ISL_687037, EPI_ISL_687038, EPI_ISL_687039, EPI_ISL_687040, EPI_ISL_687041, EPI_ISL_687042

see above | Pathogen Genomics Center, National Institute of Infectious Diseases | Pathogen Genomics Center, National Institute of Infectious Diseases | Tsuyoshi Sekizuka, Kentaro Itokawa, Rina Tanaka, Masanori Hashino, Makoto Kuroda

EPI_ISL_687043, EPI_ISL_687044, EPI_ISL_687045, EPI_ISL_687046, EPI_ISL_687047, EPI_ISL_687048, EPI_ISL_687049, EPI_ISL_687050, EPI_ISL_687051, EPI_ISL_687052, EPI_ISL_687053, EPI_ISL_687054, EPI_ISL_687055, EPI_ISL_687056, EPI_ISL_687057, EPI_ISL_687058, EPI_ISL_687059, EPI_ISL_687060, EPI_ISL_687061, EPI_ISL_687062, EPI_ISL_687063, EPI_ISL_687064, EPI_ISL_687065, EPI_ISL_687066, EPI_ISL_687067, EPI_ISL_687068, EPI_ISL_687069, EPI_ISL_687070, EPI_ISL_687071, EPI_ISL_687072, EPI_ISL_687073, EPI_ISL_687074, EPI_ISL_687075, EPI_ISL_687076, EPI_ISL_687077, EPI_ISL_687078

see above | Kanagawa Prefectural Institute of Public Health | Pathogen Genomics Center, National Institute of Infectious Diseases | Tsuyoshi Sekizuka, Kentaro Itokawa, Rina Tanaka, Masanori Hashino, Makoto Kuroda

EPI_ISL_687079, EPI_ISL_687080, EPI_ISL_687081, EPI_ISL_687082, EPI_ISL_687083, EPI_ISL_687084, EPI_ISL_687085, EPI_ISL_687086, EPI_ISL_687087, EPI_ISL_687088, EPI_ISL_687089, EPI_ISL_687090, EPI_ISL_687091, EPI_ISL_687092, EPI_ISL_687093, EPI_ISL_687094, EPI_ISL_687095, EPI_ISL_687096, EPI_ISL_687097, EPI_ISL_687098, EPI_ISL_687099, EPI_ISL_687100, EPI_ISL_687101, EPI_ISL_687102, EPI_ISL_687103, EPI_ISL_687104, EPI_ISL_687105, EPI_ISL_687106, EPI_ISL_687107, EPI_ISL_687108, EPI_ISL_687109, EPI_ISL_687110, EPI_ISL_687111, EPI_ISL_687112, EPI_ISL_687113, EPI_ISL_687114, EPI_ISL_687115, EPI_ISL_687116, EPI_ISL_687117, EPI_ISL_687118, EPI_ISL_687119, EPI_ISL_687120, EPI_ISL_687121, EPI_ISL_687122, EPI_ISL_687123, EPI_ISL_687124, EPI_ISL_687125, EPI_ISL_687126, EPI_ISL_687127, EPI_ISL_687128, EPI_ISL_687129, EPI_ISL_687130, EPI_ISL_687131, EPI_ISL_687132, EPI_ISL_687133, EPI_ISL_687134, EPI_ISL_687135, EPI_ISL_687136, EPI_ISL_687137, EPI_ISL_687138, EPI_ISL_687139, EPI_ISL_687140, EPI_ISL_687141, EPI_ISL_687142, EPI_ISL_687143, EPI_ISL_687144, EPI_ISL_687145, EPI_ISL_687146, EPI_ISL_687147, EPI_ISL_687148, EPI_ISL_687149, EPI_ISL_687150, EPI_ISL_687151, EPI_ISL_687152, EPI_ISL_687153, EPI_ISL_687154, EPI_ISL_687155, EPI_ISL_687156, EPI_ISL_687157, EPI_ISL_687158, EPI_ISL_687159, EPI_ISL_687160, EPI_ISL_687161, EPI_ISL_687162, EPI_ISL_687163, EPI_ISL_687164, EPI_ISL_687165, EPI_ISL_687166, EPI_ISL_687167, EPI_ISL_687168, EPI_ISL_687169, EPI_ISL_687170, EPI_ISL_687171, EPI_ISL_687172, EPI_ISL_687173, EPI_ISL_687174, EPI_ISL_687175, EPI_ISL_687176, EPI_ISL_687177, EPI_ISL_687178, EPI_ISL_687179, EPI_ISL_687180, EPI_ISL_687181, EPI_ISL_687182, EPI_ISL_687183, EPI_ISL_687184, EPI_ISL_687185, EPI_ISL_687186, EPI_ISL_687187, EPI_ISL_687188, EPI_ISL_687189, EPI_ISL_687190, EPI_ISL_687191, EPI_ISL_687192, EPI_ISL_687193, EPI_ISL_687194, EPI_ISL_687195, EPI_ISL_687196, EPI_ISL_687197, EPI_ISL_687198, EPI_ISL_687199, EPI_ISL_687200, EPI_ISL_687201, EPI_ISL_687202, EPI_ISL_687203, EPI_ISL_687204, EPI_ISL_687205, EPI_ISL_687206, EPI_ISL_687207, EPI_ISL_687208, EPI_ISL_687209, EPI_ISL_687210, EPI_ISL_687211, EPI_ISL_687212, EPI_ISL_687213, EPI_ISL_687214, EPI_ISL_687215, EPI_ISL_687216, EPI_ISL_687217, EPI_ISL_687218, EPI_ISL_687219, EPI_ISL_687220, EPI_ISL_687221, EPI_ISL_687222, EPI_ISL_687223, EPI_ISL_687224, EPI_ISL_687225, EPI_ISL_687226, EPI_ISL_687227, EPI_ISL_687228, EPI_ISL_687229, EPI_ISL_687230, EPI_ISL_687231, EPI_ISL_687232, EPI_ISL_687233, EPI_ISL_687234, EPI_ISL_687235, EPI_ISL_687236, EPI_ISL_687237, EPI_ISL_687238, EPI_ISL_687239, EPI_ISL_687240, EPI_ISL_687241, EPI_ISL_687242, EPI_ISL_687243, EPI_ISL_687244, EPI_ISL_687245, EPI_ISL_687246, EPI_ISL_687247, EPI_ISL_687248, EPI_ISL_687249, EPI_ISL_687250, EPI_ISL_687251, EPI_ISL_687252, EPI_ISL_687253, EPI_ISL_687254, EPI_ISL_687255, EPI_ISL_687256, EPI_ISL_687257, EPI_ISL_687258, EPI_ISL_687259, EPI_ISL_687260, EPI_ISL_687261, EPI_ISL_687262, EPI_ISL_687263, EPI_ISL_687264, EPI_ISL_687265, EPI_ISL_687266, EPI_ISL_687267, EPI_ISL_687268, EPI_ISL_687269, EPI_ISL_687270, EPI_ISL_687271, EPI_ISL_687272, EPI_ISL_687273, EPI_ISL_687274, EPI_ISL_687275, EPI_ISL_687276, EPI_ISL_687277, EPI_ISL_687278, EPI_ISL_687279, EPI_ISL_687280, EPI_ISL_687281, EPI_ISL_687282, EPI_ISL_687283, EPI_ISL_687284, EPI_ISL_687285, EPI_ISL_687286, EPI_ISL_687287, EPI_ISL_687288, EPI_ISL_687289, EPI_ISL_687290, EPI_ISL_687291, EPI_ISL_687292, EPI_ISL_687293, EPI_ISL_687294, EPI_ISL_687295, EPI_ISL_687296, EPI_ISL_687297, EPI_ISL_687298, EPI_ISL_687299, EPI_ISL_687300, EPI_ISL_687301, EPI_ISL_687302, EPI_ISL_687303, EPI_ISL_687304, EPI_ISL_687305, EPI_ISL_687306, EPI_ISL_687307, EPI_ISL_687308, EPI_ISL_687309, EPI_ISL_687310, EPI_ISL_687311, EPI_ISL_687312, EPI_ISL_687313, EPI_ISL_687314, EPI_ISL_687315, EPI_ISL_687316, EPI_ISL_687317, EPI_ISL_687318, EPI_ISL_687319, EPI_ISL_687320, EPI_ISL_687321, EPI_ISL_687322, EPI_ISL_687323, EPI_ISL_687324, EPI_ISL_687325, EPI_ISL_687326, EPI_ISL_687327, EPI_ISL_687328, EPI_ISL_687329, EPI_ISL_687330, EPI_ISL_687331, EPI_ISL_687332, EPI_ISL_687333, EPI_ISL_687334, EPI_ISL_687335, EPI_ISL_687336, EPI_ISL_687337, EPI_ISL_687338, EPI_ISL_687339, EPI_ISL_687340, EPI_ISL_687341, EPI_ISL_687342, EPI_ISL_687343, EPI_ISL_687344, EPI_ISL_687345, EPI_ISL_687346, EPI_ISL_687347, EPI_ISL_687348, EPI_ISL_687349, EPI_ISL_687350, EPI_ISL_687351, EPI_ISL_687352, EPI_ISL_687353, EPI_ISL_687354, EPI_ISL_687355, EPI_ISL_687356, EPI_ISL_687357, EPI_ISL_687358, EPI_ISL_687359, EPI_ISL_687360, EPI_ISL_687361, EPI_ISL_687362, EPI_ISL_687363, EPI_ISL_687364, EPI_ISL_687365, EPI_ISL_687366, EPI_ISL_687367, EPI_ISL_687368, EPI_ISL_687369, EPI_ISL_687370, EPI_ISL_687371, EPI_ISL_687372, EPI_ISL_687373, EPI_ISL_687374, EPI_ISL_687375, EPI_ISL_687376, EPI_ISL_687377, EPI_ISL_687378, EPI_ISL_687379, EPI_ISL_687380, EPI_ISL_687381, EPI_ISL_687382, EPI_ISL_687383, EPI_ISL_687384, EPI_ISL_687385, EPI_ISL_687386, EPI_ISL_687387, EPI_ISL_687388, EPI_ISL_687389, EPI_ISL_687390, EPI_ISL_687391, EPI_ISL_687392, EPI_ISL_687393, EPI_ISL_687394, EPI_ISL_687395, EPI_ISL_687396, EPI_ISL_687397, EPI_ISL_687398, EPI_ISL_687399, EPI_ISL_687400, EPI_ISL_687401, EPI_ISL_687402, EPI_ISL_687403, EPI_ISL_687404, EPI_ISL_687405, EPI_ISL_687406, EPI_ISL_687407, EPI_ISL_687408, EPI_ISL_687409, EPI_ISL_687410, EPI_ISL_687411, EPI_ISL_687412, EPI_ISL_687413, EPI_ISL_687414, EPI_ISL_687415, EPI_ISL_687416, EPI_ISL_687417, EPI_ISL_687418, EPI_ISL_687419, EPI_ISL_687420, EPI_ISL_687421, EPI_ISL_687422, EPI_ISL_687423, EPI_ISL_687424, EPI_ISL_687425, EPI_ISL_687426, EPI_ISL_687427, EPI_ISL_687428, EPI_ISL_687429, EPI_ISL_687430, EPI_ISL_687431, EPI_ISL_687432, EPI_ISL_687433, EPI_ISL_687434, EPI_ISL_687435, EPI_ISL_687436, EPI_ISL_687437, EPI_ISL_687438, EPI_ISL_687439, EPI_ISL_687440, EPI_ISL_687441, EPI_ISL_687442, EPI_ISL_687443, EPI_ISL_687444, EPI_ISL_687445, EPI_ISL_687446, EPI_ISL_687447, EPI_ISL_687448, EPI_ISL_687449, EPI_ISL_687450, EPI_ISL_687451, EPI_ISL_687452, EPI_ISL_687453, EPI_ISL_687454, EPI_ISL_687455, EPI_ISL_687456, EPI_ISL_687457, EPI_ISL_687458, EPI_ISL_687459, EPI_ISL_687460, EPI_ISL_687461, EPI_ISL_687462, EPI_ISL_687463, EPI_ISL_687464, EPI_ISL_687465, EPI_ISL_687466, EPI_ISL_687467, EPI_ISL_687468, EPI_ISL_687469, EPI_ISL_687470, EPI_ISL_687471, EPI_ISL_687472, EPI_ISL_687473, EPI_ISL_687474, EPI_ISL_687475, EPI_ISL_687476, EPI_ISL_687477, EPI_ISL_687478, EPI_ISL_687479, EPI_ISL_687480, EPI_ISL_687481, EPI_ISL_687482, EPI_ISL_687483, EPI_ISL_687484, EPI_ISL_687485, EPI_ISL_687486, EPI_ISL_687487, EPI_ISL_687488, EPI_ISL_687489, EPI_ISL_687490, EPI_ISL_687491, EPI_ISL_687492, EPI_ISL_687493, EPI_ISL_687494, EPI_ISL_687495, EPI_ISL_687496, EPI_ISL_687497, EPI_ISL_687498, EPI_ISL_687499, EPI_ISL_687500, EPI_ISL_687501, EPI_ISL_687502, EPI_ISL_687503, EPI_ISL_687504, EPI_ISL_687505, EPI_ISL_687506, EPI_ISL_687507, EPI_ISL_687508, EPI_ISL_687509, EPI_ISL_687510, EPI_ISL_687511, EPI_ISL_687512, EPI_ISL_687513, EPI_ISL_687514, EPI_ISL_687515, EPI_ISL_687516, EPI_ISL_687517, EPI_ISL_687518, EPI_ISL_687519, EPI_ISL_687520, EPI_ISL_687521, EPI_ISL_687522, EPI_ISL_687523, EPI_ISL_687524, EPI_ISL_687525, EPI_ISL_687526, EPI_ISL_687527, EPI_ISL_687528, EPI_ISL_687529, EPI_ISL_687530, EPI_ISL_687531, EPI_ISL_687532

see above | Pathogen Genomics Center, National Institute of Infectious Diseases | Pathogen Genomics Center, National Institute of Infectious Diseases | Tsuyoshi Sekizuka, Kentaro Itokawa, Rina Tanaka, Masanori Hashino, Makoto Kuroda

EPI_ISL_687533, EPI_ISL_687534, EPI_ISL_687535, EPI_ISL_687536, EPI_ISL_687537, EPI_ISL_687538, EPI_ISL_687539, EPI_ISL_687540, EPI_ISL_687541, EPI_ISL_687542, EPI_ISL_687543, EPI_ISL_687544, EPI_ISL_687545, EPI_ISL_687546, EPI_ISL_687547, EPI_ISL_687548, EPI_ISL_687549, EPI_ISL_687550, EPI_ISL_687551, EPI_ISL_687552, EPI_ISL_687553, EPI_ISL_687554, EPI_ISL_687555, EPI_ISL_687556, EPI_ISL_687557, EPI_ISL_687558, EPI_ISL_687559, EPI_ISL_687560

see above | Fukuoka Institute of Health and Environmental Sciences | Pathogen Genomics Center, National Institute of Infectious Diseases | Tsuyoshi Sekizuka, Kentaro Itokawa, Rina Tanaka, Masanori Hashino, Makoto Kuroda

EPI_ISL_687561, EPI_ISL_687562, EPI_ISL_687563, EPI_ISL_687564, EPI_ISL_687565, EPI_ISL_687566, EPI_ISL_687567, EPI_ISL_687568, EPI_ISL_687569, EPI_ISL_687570, EPI_ISL_687571, EPI_ISL_687572, EPI_ISL_687573, EPI_ISL_687574, EPI_ISL_687575, EPI_ISL_687576, EPI_ISL_687577, EPI_ISL_687578, EPI_ISL_687579, EPI_ISL_687580, EPI_ISL_687581, EPI_ISL_687582, EPI_ISL_687583, EPI_ISL_687584, EPI_ISL_687585, EPI_ISL_687586, EPI_ISL_687587, EPI_ISL_687588, EPI_ISL_687589, EPI_ISL_687590, EPI_ISL_687591, EPI_ISL_687592, EPI_ISL_687593, EPI_ISL_687594, EPI_ISL_687595, EPI_ISL_687596, EPI_ISL_687597, EPI_ISL_687598, EPI_ISL_687599, EPI_ISL_687600, EPI_ISL_687601, EPI_ISL_687602, EPI_ISL_687603, EPI_ISL_687604, EPI_ISL_687605, EPI_ISL_687606, EPI_ISL_687607, EPI_ISL_687608, EPI_ISL_687609, EPI_ISL_687610, EPI_ISL_687611, EPI_ISL_687612, EPI_ISL_687613, EPI_ISL_687614, EPI_ISL_687615, EPI_ISL_687616, EPI_ISL_687617, EPI_ISL_687618

see above | Pathogen Genomics Center, National Institute of Infectious Diseases | Pathogen Genomics Center, National Institute of Infectious Diseases | Tsuyoshi Sekizuka, Kentaro Itokawa, Rina Tanaka, Masanori Hashino, Makoto Kuroda

EPI_ISL_687619, EPI_ISL_687620 | Saitama Prefectural Institute of Public Health | Pathogen Genomics Center, National Institute of Infectious Diseases | Tsuyoshi Sekizuka, Kentaro Itokawa, Rina Tanaka, Masanori Hashino, Makoto Kuroda

EPI_ISL_687621, EPI_ISL_687622, EPI_ISL_687623, EPI_ISL_687624, EPI_ISL_687625, EPI_ISL_687626, EPI_ISL_687627, EPI_ISL_687628, EPI_ISL_687629, EPI_ISL_687630, EPI_ISL_687631, EPI_ISL_687632, EPI_ISL_687633, EPI_ISL_687634, EPI_ISL_687635, EPI_ISL_687636, EPI_ISL_687637, EPI_ISL_687638, EPI_ISL_687639, EPI_ISL_687640, EPI_ISL_687641, EPI_ISL_687642, EPI_ISL_687643, EPI_ISL_687644, EPI_ISL_687645, EPI_ISL_687646, EPI_ISL_687647, EPI_ISL_687648, EPI_ISL_687649, EPI_ISL_687650, EPI_ISL_687651, EPI_ISL_687652, EPI_ISL_687653, EPI_ISL_687654, EPI_ISL_687655, EPI_ISL_687656, EPI_ISL_687657, EPI_ISL_687658, EPI_ISL_687659, EPI_ISL_687660, EPI_ISL_687661, EPI_ISL_687662, EPI_ISL_687663, EPI_ISL_687664, EPI_ISL_687665, EPI_ISL_687666, EPI_ISL_687667, EPI_ISL_687668, EPI_ISL_687669, EPI_ISL_687670, EPI_ISL_687671, EPI_ISL_687672, EPI_ISL_687673, EPI_ISL_687674, EPI_ISL_687675, EPI_ISL_687676, EPI_ISL_687677, EPI_ISL_687678, EPI_ISL_687679, EPI_ISL_687680, EPI_ISL_687681, EPI_ISL_687682, EPI_ISL_687683, EPI_ISL_687684, EPI_ISL_687685, EPI_ISL_687686, EPI_ISL_687687, EPI_ISL_687688, EPI_ISL_687689, EPI_ISL_687690, EPI_ISL_687691, EPI_ISL_687692, EPI_ISL_687693, EPI_ISL_687694, EPI_ISL_687695, EPI_ISL_687696, EPI_ISL_687697, EPI_ISL_687698

see above | Pathogen Genomics Center, National Institute of Infectious Diseases | Pathogen Genomics Center, National Institute of Infectious Diseases | Tsuyoshi Sekizuka, Kentaro Itokawa, Rina Tanaka, Masanori Hashino, Makoto Kuroda

EPI_ISL_687699, EPI_ISL_687700, EPI_ISL_687701, EPI_ISL_687702, EPI_ISL_687703, EPI_ISL_687704, EPI_ISL_687705, EPI_ISL_687706, EPI_ISL_687707, EPI_ISL_687708, EPI_ISL_687709, EPI_ISL_687710, EPI_ISL_687711, EPI_ISL_687712, EPI_ISL_687713, EPI_ISL_687714, EPI_ISL_687715, EPI_ISL_687716, EPI_ISL_687717

see above | Niigata Prefectural Institute of Public Health and Environmental Sciences | Pathogen Genomics Center, National Institute of Infectious Diseases | Tsuyoshi Sekizuka, Kentaro Itokawa, Rina Tanaka, Masanori Hashino, Makoto Kuroda

EPI_ISL_687718, EPI_ISL_687719, EPI_ISL_687720, EPI_ISL_687721, EPI_ISL_687722, EPI_ISL_687723, EPI_ISL_687724, EPI_ISL_687725, EPI_ISL_687726, EPI_ISL_687727, EPI_ISL_687728, EPI_ISL_687729, EPI_ISL_687730, EPI_ISL_687731, EPI_ISL_687732, EPI_ISL_687733, EPI_ISL_687734, EPI_ISL_687735, EPI_ISL_687736, EPI_ISL_687737, EPI_ISL_687738, EPI_ISL_687739, EPI_ISL_687740, EPI_ISL_687741, EPI_ISL_687742, EPI_ISL_687743, EPI_ISL_687744, EPI_ISL_687745, EPI_ISL_687746, EPI_ISL_687747, EPI_ISL_687748, EPI_ISL_687749, EPI_ISL_687750, EPI_ISL_687751, EPI_ISL_687752, EPI_ISL_687753, EPI_ISL_687754, EPI_ISL_687755, EPI_ISL_687756, EPI_ISL_687757, EPI_ISL_687758, EPI_ISL_687759, EPI_ISL_687760, EPI_ISL_687761, EPI_ISL_687762, EPI_ISL_687763, EPI_ISL_687764, EPI_ISL_687765, EPI_ISL_687766, EPI_ISL_687767, EPI_ISL_687768, EPI_ISL_687769, EPI_ISL_687770, EPI_ISL_687771, EPI_ISL_687772, EPI_ISL_687773, EPI_ISL_687774, EPI_ISL_687775, EPI_ISL_687776, EPI_ISL_687777, EPI_ISL_687778, EPI_ISL_687779, EPI_ISL_687780, EPI_ISL_687781, EPI_ISL_687782, EPI_ISL_687783, EPI_ISL_687784, EPI_ISL_687785, EPI_ISL_687786, EPI_ISL_687787, EPI_ISL_687788, EPI_ISL_687789, EPI_ISL_687790, EPI_ISL_687791, EPI_ISL_687792, EPI_ISL_687793, EPI_ISL_687794, EPI_ISL_687795, EPI_ISL_687796, EPI_ISL_687797, EPI_ISL_687798, EPI_ISL_687799, EPI_ISL_687800, EPI_ISL_687801, EPI_ISL_687802, EPI_ISL_687803, EPI_ISL_687804, EPI_ISL_687805, EPI_ISL_687806, EPI_ISL_687807, EPI_ISL_687808, EPI_ISL_687809, EPI_ISL_687810, EPI_ISL_687811, EPI_ISL_687812, EPI_ISL_687813, EPI_ISL_687814, EPI_ISL_687815, EPI_ISL_687816, EPI_ISL_687817, EPI_ISL_687818, EPI_ISL_687819, EPI_ISL_687820, EPI_ISL_687821, EPI_ISL_687822, EPI_ISL_687823, EPI_ISL_687824, EPI_ISL_687825, EPI_ISL_687826, EPI_ISL_687827, EPI_ISL_687828, EPI_ISL_687829, EPI_ISL_687830, EPI_ISL_687831, EPI_ISL_687832, EPI_ISL_687833, EPI_ISL_687834, EPI_ISL_687835, EPI_ISL_687836, EPI_ISL_687837, EPI_ISL_687838, EPI_ISL_687839, EPI_ISL_687840, EPI_ISL_687841, EPI_ISL_687842, EPI_ISL_687843, EPI_ISL_687844, EPI_ISL_687845, EPI_ISL_687846, EPI_ISL_687847, EPI_ISL_687848, EPI_ISL_687849, EPI_ISL_687850, EPI_ISL_687851, EPI_ISL_687852, EPI_ISL_687853, EPI_ISL_687854, EPI_ISL_687855, EPI_ISL_687856, EPI_ISL_687857, EPI_ISL_687858, EPI_ISL_687859, EPI_ISL_687860, EPI_ISL_687861, EPI_ISL_687862, EPI_ISL_687863, EPI_ISL_687864, EPI_ISL_687865, EPI_ISL_687866, EPI_ISL_687867, EPI_ISL_687868, EPI_ISL_687869, EPI_ISL_687870, EPI_ISL_687871, EPI_ISL_687872, EPI_ISL_687873, EPI_ISL_687874, EPI_ISL_687875, EPI_ISL_687876, EPI_ISL_687877, EPI_ISL_687878, EPI_ISL_687879, EPI_ISL_687880, EPI_ISL_687881, EPI_ISL_687882, EPI_ISL_687883, EPI_ISL_687884, EPI_ISL_687885, EPI_ISL_687886, EPI_ISL_687887, EPI_ISL_687888, EPI_ISL_687889, EPI_ISL_687890, EPI_ISL_687891, EPI_ISL_687892, EPI_ISL_687893, EPI_ISL_687894, EPI_ISL_687895, EPI_ISL_687896, EPI_ISL_687897, EPI_ISL_687898, EPI_ISL_687899, EPI_ISL_687900, EPI_ISL_687901, EPI_ISL_687902, EPI_ISL_687903, EPI_ISL_687904, EPI_ISL_687905, EPI_ISL_687906, EPI_ISL_687907, EPI_ISL_687908, EPI_ISL_687909, EPI_ISL_687910, EPI_ISL_687911, EPI_ISL_687912, EPI_ISL_687913, EPI_ISL_687914, EPI_ISL_687915, EPI_ISL_687916, EPI_ISL_687917, EPI_ISL_687918, EPI_ISL_687919, EPI_ISL_687920, EPI_ISL_687921, EPI_ISL_687922, EPI_ISL_687923, EPI_ISL_687924, EPI_ISL_687925, EPI_ISL_687926, EPI_ISL_687927, EPI_ISL_687928, EPI_ISL_687929, EPI_ISL_687930, EPI_ISL_687931, EPI_ISL_687932, EPI_ISL_687933, EPI_ISL_687934, EPI_ISL_687935, EPI_ISL_687936, EPI_ISL_687937, EPI_ISL_687938, EPI_ISL_687939, EPI_ISL_687940, EPI_ISL_687941, EPI_ISL_687942, EPI_ISL_687943, EPI_ISL_687944, EPI_ISL_687945, EPI_ISL_687946, EPI_ISL_687947, EPI_ISL_687948, EPI_ISL_687949, EPI_ISL_687950, EPI_ISL_687951, EPI_ISL_687952, EPI_ISL_687953, EPI_ISL_687954, EPI_ISL_687955, EPI_ISL_687956, EPI_ISL_687957, EPI_ISL_687958, EPI_ISL_687959, EPI_ISL_687960, EPI_ISL_687961, EPI_ISL_687962, EPI_ISL_687963, EPI_ISL_687964, EPI_ISL_687965, EPI_ISL_687966, EPI_ISL_687967, EPI_ISL_687968, EPI_ISL_687969,

EPI_ISL_687970, EPI_ISL_687971, EPI_ISL_687972, EPI_ISL_687973, EPI_ISL_687974, EPI_ISL_687975, EPI_ISL_687976, EPI_ISL_687977, EPI_ISL_687978, EPI_ISL_687979, EPI_ISL_687980, EPI_ISL_687981, EPI_ISL_687982, EPI_ISL_687983, EPI_ISL_687984, EPI_ISL_687985, EPI_ISL_687986, EPI_ISL_687987, EPI_ISL_687988, EPI_ISL_687989, EPI_ISL_687990, EPI_ISL_687991, EPI_ISL_687992, EPI_ISL_687993, EPI_ISL_687994, EPI_ISL_687995, EPI_ISL_687996, EPI_ISL_687997, EPI_ISL_687998, EPI_ISL_687999, EPI_ISL_688000, EPI_ISL_688001, EPI_ISL_688002, EPI_ISL_688003, EPI_ISL_688004, EPI_ISL_688005, EPI_ISL_688006, EPI_ISL_688007, EPI_ISL_688008, EPI_ISL_688009, EPI_ISL_688010, EPI_ISL_688011, EPI_ISL_688012, EPI_ISL_688013, EPI_ISL_688014, EPI_ISL_688015, EPI_ISL_688016, EPI_ISL_688017, EPI_ISL_688018, EPI_ISL_688019, EPI_ISL_688020, EPI_ISL_688021, EPI_ISL_688022, EPI_ISL_688023, EPI_ISL_688024, EPI_ISL_688025, EPI_ISL_688026, EPI_ISL_688027, EPI_ISL_688028, EPI_ISL_688029, EPI_ISL_688030, EPI_ISL_688031, EPI_ISL_688032, EPI_ISL_688033, EPI_ISL_688034, EPI_ISL_688035, EPI_ISL_688036, EPI_ISL_688037, EPI_ISL_688038, EPI_ISL_688039, EPI_ISL_688040, EPI_ISL_688041, EPI_ISL_688042, EPI_ISL_688043, EPI_ISL_688044, EPI_ISL_688045, EPI_ISL_688046, EPI_ISL_688047, EPI_ISL_688048, EPI_ISL_688049, EPI_ISL_688050, EPI_ISL_688051, EPI_ISL_688052, EPI_ISL_688053, EPI_ISL_688054, EPI_ISL_688055, EPI_ISL_688056, EPI_ISL_688057, EPI_ISL_688058, EPI_ISL_688059, EPI_ISL_688060, EPI_ISL_688061, EPI_ISL_688062, EPI_ISL_688063, EPI_ISL_688064, EPI_ISL_688065, EPI_ISL_688066, EPI_ISL_688067, EPI_ISL_688068, EPI_ISL_688069, EPI_ISL_688070, EPI_ISL_688071, EPI_ISL_688072, EPI_ISL_688073, EPI_ISL_688074, EPI_ISL_688075, EPI_ISL_688076, EPI_ISL_688077, EPI_ISL_688078, EPI_ISL_688079, EPI_ISL_688080, EPI_ISL_688081, EPI_ISL_688082, EPI_ISL_688083, EPI_ISL_688084, EPI_ISL_688085, EPI_ISL_688086, EPI_ISL_688087, EPI_ISL_688088, EPI_ISL_688089, EPI_ISL_688090, EPI_ISL_688091, EPI_ISL_688092, EPI_ISL_688093, EPI_ISL_688094, EPI_ISL_688095, EPI_ISL_688096, EPI_ISL_688097, EPI_ISL_688098, EPI_ISL_688099, EPI_ISL_688100, EPI_ISL_688101, EPI_ISL_688102, EPI_ISL_688103, EPI_ISL_688104, EPI_ISL_688105, EPI_ISL_688106, EPI_ISL_688107, EPI_ISL_688108, EPI_ISL_688109, EPI_ISL_688110, EPI_ISL_688111, EPI_ISL_688112, EPI_ISL_688113, EPI_ISL_688114, EPI_ISL_688115, EPI_ISL_688116, EPI_ISL_688117, EPI_ISL_688118, EPI_ISL_688119, EPI_ISL_688120, EPI_ISL_688121, EPI_ISL_688122, EPI_ISL_688123, EPI_ISL_688124, EPI_ISL_688125, EPI_ISL_688126, EPI_ISL_688127, EPI_ISL_688128, EPI_ISL_688129, EPI_ISL_688130, EPI_ISL_688131, EPI_ISL_688132, EPI_ISL_688133, EPI_ISL_688134, EPI_ISL_688135, EPI_ISL_688136, EPI_ISL_688137, EPI_ISL_688138, EPI_ISL_688139, EPI_ISL_688140, EPI_ISL_688141, EPI_ISL_688142, EPI_ISL_688143, EPI_ISL_688144, EPI_ISL_688145, EPI_ISL_688146, EPI_ISL_688147, EPI_ISL_688148, EPI_ISL_688149, EPI_ISL_688150, EPI_ISL_688151, EPI_ISL_688152, EPI_ISL_688153, EPI_ISL_688154, EPI_ISL_688155, EPI_ISL_688156, EPI_ISL_688157, EPI_ISL_688158, EPI_ISL_688159, EPI_ISL_688160, EPI_ISL_688161, EPI_ISL_688162, EPI_ISL_688163, EPI_ISL_688164, EPI_ISL_688165, EPI_ISL_688166, EPI_ISL_688167, EPI_ISL_688168, EPI_ISL_688169, EPI_ISL_688170, EPI_ISL_688171, EPI_ISL_688172, EPI_ISL_688173, EPI_ISL_688174, EPI_ISL_688175, EPI_ISL_688176, EPI_ISL_688177, EPI_ISL_688178, EPI_ISL_688179, EPI_ISL_688180, EPI_ISL_688181, EPI_ISL_688182, EPI_ISL_688183, EPI_ISL_688184, EPI_ISL_688185, EPI_ISL_688186, EPI_ISL_688187, EPI_ISL_688188, EPI_ISL_688189, EPI_ISL_688190, EPI_ISL_688191, EPI_ISL_688192, EPI_ISL_688193, EPI_ISL_688194, EPI_ISL_688195, EPI_ISL_688196, EPI_ISL_688197, EPI_ISL_688198, EPI_ISL_688199, EPI_ISL_688200, EPI_ISL_688201, EPI_ISL_688202, EPI_ISL_688203, EPI_ISL_688204, EPI_ISL_688205, EPI_ISL_688206, EPI_ISL_688207, EPI_ISL_688208, EPI_ISL_688209, EPI_ISL_688210, EPI_ISL_688211, EPI_ISL_688212, EPI_ISL_688213, EPI_ISL_688214, EPI_ISL_688215, EPI_ISL_688216, EPI_ISL_688217, EPI_ISL_688218, EPI_ISL_688219, EPI_ISL_688220, EPI_ISL_688221, EPI_ISL_688222, EPI_ISL_688223, EPI_ISL_688224, EPI_ISL_688225, EPI_ISL_688226, EPI_ISL_688227, EPI_ISL_688228, EPI_ISL_688229, EPI_ISL_688230, EPI_ISL_688231, EPI_ISL_688232, EPI_ISL_688233, EPI_ISL_688234, EPI_ISL_688235, EPI_ISL_688236, EPI_ISL_688237, EPI_ISL_688238, EPI_ISL_688239, EPI_ISL_688240, EPI_ISL_688241, EPI_ISL_688242, EPI_ISL_688243, EPI_ISL_688244, EPI_ISL_688245, EPI_ISL_688246, EPI_ISL_688247, EPI_ISL_688248, EPI_ISL_688249, EPI_ISL_688250, EPI_ISL_688251, EPI_ISL_688252, EPI_ISL_688253, EPI_ISL_688254, EPI_ISL_688255, EPI_ISL_688256, EPI_ISL_688257, EPI_ISL_688258, EPI_ISL_688259, EPI_ISL_688260, EPI_ISL_688261, EPI_ISL_688262, EPI_ISL_688263, EPI_ISL_688264, EPI_ISL_688265, EPI_ISL_688266, EPI_ISL_688267, EPI_ISL_688268, EPI_ISL_688269, EPI_ISL_688270, EPI_ISL_688271, EPI_ISL_688272, EPI_ISL_688273, EPI_ISL_688274, EPI_ISL_688275, EPI_ISL_688276, EPI_ISL_688277, EPI_ISL_688278, EPI_ISL_688279, EPI_ISL_688280, EPI_ISL_688281, EPI_ISL_688282, EPI_ISL_688283			
see above	Pathogen Genomics Center, National Institute of Infectious Diseases	Pathogen Genomics Center, National Institute of Infectious Diseases	Tsuyoshi Sekizuka, Kentaro Itokawa, Rina Tanaka, Masanori Hashino, Makoto Kuroda
EPI_ISL_688284, EPI_ISL_688285, EPI_ISL_688286, EPI_ISL_688287, EPI_ISL_688288, EPI_ISL_688289, EPI_ISL_688290, EPI_ISL_688291, EPI_ISL_688292, EPI_ISL_688293, EPI_ISL_688294, EPI_ISL_688295, EPI_ISL_688296, EPI_ISL_688297, EPI_ISL_688298, EPI_ISL_688299, EPI_ISL_688300, EPI_ISL_688301, EPI_ISL_688302, EPI_ISL_688303, EPI_ISL_688304, EPI_ISL_688305, EPI_ISL_688306, EPI_ISL_688307, EPI_ISL_688308, EPI_ISL_688309, EPI_ISL_688310, EPI_ISL_688311, EPI_ISL_688312, EPI_ISL_688313, EPI_ISL_688314, EPI_ISL_688315, EPI_ISL_688316, EPI_ISL_688317, EPI_ISL_688318, EPI_ISL_688319, EPI_ISL_688320, EPI_ISL_688321, EPI_ISL_688322, EPI_ISL_688323, EPI_ISL_688324, EPI_ISL_688325, EPI_ISL_688326, EPI_ISL_688327, EPI_ISL_688328, EPI_ISL_688329, EPI_ISL_688330, EPI_ISL_688331, EPI_ISL_688332, EPI_ISL_688333, EPI_ISL_688334, EPI_ISL_688335, EPI_ISL_688336, EPI_ISL_688337, EPI_ISL_688338, EPI_ISL_688339, EPI_ISL_688340, EPI_ISL_688341, EPI_ISL_688342, EPI_ISL_688343, EPI_ISL_688344, EPI_ISL_688345, EPI_ISL_688346, EPI_ISL_688347, EPI_ISL_688348, EPI_ISL_688349, EPI_ISL_688350, EPI_ISL_688351, EPI_ISL_688352, EPI_ISL_688353, EPI_ISL_688354, EPI_ISL_688355, EPI_ISL_688356, EPI_ISL_688357, EPI_ISL_688358, EPI_ISL_688359, EPI_ISL_688360, EPI_ISL_688361, EPI_ISL_688362, EPI_ISL_688363, EPI_ISL_688364, EPI_ISL_688365, EPI_ISL_688366, EPI_ISL_688367, EPI_ISL_688368, EPI_ISL_688369, EPI_ISL_688370, EPI_ISL_688371, EPI_ISL_688372, EPI_ISL_688373, EPI_ISL_688374, EPI_ISL_688375, EPI_ISL_688376, EPI_ISL_688377, EPI_ISL_688378, EPI_ISL_688379, EPI_ISL_688380, EPI_ISL_688381, EPI_ISL_688382, EPI_ISL_688383, EPI_ISL_688384, EPI_ISL_688385, EPI_ISL_688386, EPI_ISL_688387, EPI_ISL_688388, EPI_ISL_688389, EPI_ISL_688390, EPI_ISL_688391, EPI_ISL_688392, EPI_ISL_688393, EPI_ISL_688394, EPI_ISL_688395, EPI_ISL_688396, EPI_ISL_688397, EPI_ISL_688398, EPI_ISL_688399, EPI_ISL_688400, EPI_ISL_688401			
see above	Saitama Prefectural Institute of Public Health	Pathogen Genomics Center, National Institute of Infectious Diseases	Tsuyoshi Sekizuka, Kentaro Itokawa, Rina Tanaka, Masanori Hashino, Makoto Kuroda
EPI_ISL_688402, EPI_ISL_688403, EPI_ISL_688404, EPI_ISL_688405, EPI_ISL_688406, EPI_ISL_688407, EPI_ISL_688408, EPI_ISL_688409, EPI_ISL_688410, EPI_ISL_688411, EPI_ISL_688412, EPI_ISL_688413, EPI_ISL_688414, EPI_ISL_688415, EPI_ISL_688416, EPI_ISL_688417, EPI_ISL_688418, EPI_ISL_688419, EPI_ISL_688420, EPI_ISL_688421, EPI_ISL_688422, EPI_ISL_688423, EPI_ISL_688424, EPI_ISL_688425, EPI_ISL_688426, EPI_ISL_688427, EPI_ISL_688428, EPI_ISL_688429, EPI_ISL_688430, EPI_ISL_688431, EPI_ISL_688432, EPI_ISL_688433, EPI_ISL_688434, EPI_ISL_688435, EPI_ISL_688436, EPI_ISL_688437, EPI_ISL_688438, EPI_ISL_688439, EPI_ISL_688440, EPI_ISL_688441, EPI_ISL_688442, EPI_ISL_688443, EPI_ISL_688444, EPI_ISL_688445, EPI_ISL_688446, EPI_ISL_688447, EPI_ISL_688448, EPI_ISL_688449, EPI_ISL_688450, EPI_ISL_688451, EPI_ISL_688452, EPI_ISL_688453, EPI_ISL_688454, EPI_ISL_688455, EPI_ISL_688456, EPI_ISL_688457, EPI_ISL_688458, EPI_ISL_688459, EPI_ISL_688460, EPI_ISL_688461, EPI_ISL_688462, EPI_ISL_688463, EPI_ISL_688464, EPI_ISL_688465, EPI_ISL_688466, EPI_ISL_688467, EPI_ISL_688468, EPI_ISL_688469, EPI_ISL_688470, EPI_ISL_688471, EPI_ISL_688472, EPI_ISL_688473, EPI_ISL_688474, EPI_ISL_688475, EPI_ISL_688476, EPI_ISL_688477, EPI_ISL_688478, EPI_ISL_688479, EPI_ISL_688480, EPI_ISL_688481, EPI_ISL_688482, EPI_ISL_688483, EPI_ISL_688484, EPI_ISL_688485, EPI_ISL_688486, EPI_ISL_688487, EPI_ISL_688488, EPI_ISL_688489, EPI_ISL_688490, EPI_ISL_688491, EPI_ISL_688492, EPI_ISL_688493, EPI_ISL_688494, EPI_ISL_688495, EPI_ISL_688496, EPI_ISL_688497, EPI_ISL_688498, EPI_ISL_688499, EPI_ISL_688500, EPI_ISL_688501, EPI_ISL_688502, EPI_ISL_688503, EPI_ISL_688504, EPI_ISL_688505, EPI_ISL_688506, EPI_ISL_688507, EPI_ISL_688508, EPI_ISL_688509, EPI_ISL_688510, EPI_ISL_688511, EPI_ISL_688512, EPI_ISL_688513, EPI_ISL_688514, EPI_ISL_688515, EPI_ISL_688516, EPI_ISL_688517, EPI_ISL_688518, EPI_ISL_688519, EPI_ISL_688520, EPI_ISL_688521, EPI_ISL_688522, EPI_ISL_688523, EPI_ISL_688524, EPI_ISL_688525, EPI_ISL_688526, EPI_ISL_688527, EPI_ISL_688528, EPI_ISL_688529, EPI_ISL_688530, EPI_ISL_688531, EPI_ISL_688532, EPI_ISL_688533, EPI_ISL_688534, EPI_ISL_688535, EPI_ISL_688536, EPI_ISL_688537, EPI_ISL_688538, EPI_ISL_688539, EPI_ISL_688540, EPI_ISL_688541, EPI_ISL_688542, EPI_ISL_688543, EPI_ISL_688544, EPI_ISL_688545, EPI_ISL_688546, EPI_ISL_688547, EPI_ISL_688548, EPI_ISL_688549, EPI_ISL_688550, EPI_ISL_688551, EPI_ISL_688552, EPI_ISL_688553, EPI_ISL_688554, EPI_ISL_688555, EPI_ISL_688556, EPI_ISL_688557, EPI_ISL_688558, EPI_ISL_688559, EPI_ISL_688560, EPI_ISL_688561, EPI_ISL_688562, EPI_ISL_688563, EPI_ISL_688564, EPI_ISL_688565, EPI_ISL_688566, EPI_ISL_688567, EPI_ISL_688568, EPI_ISL_688569, EPI_ISL_688570, EPI_ISL_688571, EPI_ISL_688572, EPI_ISL_688573, EPI_ISL_688574, EPI_ISL_688575, EPI_ISL_688576, EPI_ISL_688577, EPI_ISL_688578, EPI_ISL_688579, EPI_ISL_688580, EPI_ISL_688581, EPI_ISL_688582			
see above	Pathogen Genomics Center, National Institute of Infectious Diseases	Pathogen Genomics Center, National Institute of Infectious Diseases	Tsuyoshi Sekizuka, Kentaro Itokawa, Rina Tanaka, Masanori Hashino, Makoto Kuroda
EPI_ISL_688583, EPI_ISL_688584, EPI_ISL_688585, EPI_ISL_688586, EPI_ISL_688587, EPI_ISL_688588, EPI_ISL_688589, EPI_ISL_688590, EPI_ISL_688591, EPI_ISL_688592, EPI_ISL_688593, EPI_ISL_688594, EPI_ISL_688595, EPI_ISL_688596, EPI_ISL_688597, EPI_ISL_688598, EPI_ISL_688599, EPI_ISL_688600, EPI_ISL_688601, EPI_ISL_688602, EPI_ISL_688603, EPI_ISL_688604			
see above	Tokyo Metropolitan Institute of Public Health	Pathogen Genomics Center, National Institute of Infectious Diseases	Tsuyoshi Sekizuka, Kentaro Itokawa, Rina Tanaka, Masanori Hashino, Makoto Kuroda
EPI_ISL_688605, EPI_ISL_688606, EPI_ISL_688607, EPI_ISL_688608, EPI_ISL_688609, EPI_ISL_688610, EPI_ISL_688611	Pathogen Genomics Center, National Institute of Infectious Diseases	Pathogen Genomics Center, National Institute of Infectious Diseases	Tsuyoshi Sekizuka, Kentaro Itokawa, Rina Tanaka, Masanori Hashino, Makoto Kuroda
EPI_ISL_688612	Fukuoka Institute of Health and Environmental Sciences	Pathogen Genomics Center, National Institute of Infectious Diseases	Tsuyoshi Sekizuka, Kentaro Itokawa, Rina Tanaka, Masanori Hashino, Makoto Kuroda
EPI_ISL_688613, EPI_ISL_688614, EPI_ISL_688615, EPI_ISL_688616, EPI_ISL_688617, EPI_ISL_688618	Pathogen Genomics Center, National Institute of Infectious Diseases	Pathogen Genomics Center, National Institute of Infectious Diseases	Tsuyoshi Sekizuka, Kentaro Itokawa, Rina Tanaka, Masanori Hashino, Makoto Kuroda
EPI_ISL_688619, EPI_ISL_688620, EPI_ISL_688621, EPI_ISL_688622, EPI_ISL_688623	Fukuoka Institute of Health and Environmental Sciences	Pathogen Genomics Center, National Institute of Infectious Diseases	Tsuyoshi Sekizuka, Kentaro Itokawa, Rina Tanaka, Masanori Hashino, Makoto Kuroda
EPI_ISL_688624, EPI_ISL_688625, EPI_ISL_688626, EPI_ISL_688627, EPI_ISL_688628, EPI_ISL_688629, EPI_ISL_688630, EPI_ISL_688631, EPI_ISL_688632, EPI_ISL_688633	Pathogen Genomics Center, National Institute of Infectious Diseases	Pathogen Genomics Center, National Institute of Infectious Diseases	Tsuyoshi Sekizuka, Kentaro Itokawa, Rina Tanaka, Masanori Hashino, Makoto Kuroda
EPI_ISL_688634	Fukuoka Institute of Health and Environmental Sciences	Pathogen Genomics Center, National Institute of Infectious Diseases	Tsuyoshi Sekizuka, Kentaro Itokawa, Rina Tanaka, Masanori Hashino, Makoto Kuroda
EPI_ISL_688635, EPI_ISL_688636, EPI_ISL_688637, EPI_ISL_688638, EPI_ISL_688639, EPI_ISL_688640, EPI_ISL_688641, EPI_ISL_688642, EPI_ISL_688643, EPI_ISL_688644, EPI_ISL_688645, EPI_ISL_688646, EPI_ISL_688647, EPI_ISL_688648, EPI_ISL_688649, EPI_ISL_688650, EPI_ISL_688651, EPI_ISL_688652, EPI_ISL_688653, EPI_ISL_688654, EPI_ISL_688655, EPI_ISL_688656, EPI_ISL_688657, EPI_ISL_688658, EPI_ISL_688659, EPI_ISL_688660, EPI_ISL_688661, EPI_ISL_688662, EPI_ISL_688663, EPI_ISL_688664, EPI_ISL_688665, EPI_ISL_688666, EPI_ISL_688667, EPI_ISL_688668, EPI_ISL_688669, EPI_ISL_688670, EPI_ISL_688671, EPI_ISL_688672, EPI_ISL_688673, EPI_ISL_688674, EPI_ISL_688675, EPI_ISL_688676, EPI_ISL_688677, EPI_ISL_688678, EPI_ISL_688679, EPI_ISL_688680, EPI_ISL_688681, EPI_ISL_688682, EPI_ISL_688683, EPI_ISL_688684, EPI_ISL_688685, EPI_ISL_688686, EPI_ISL_688687, EPI_ISL_688688, EPI_ISL_688689, EPI_ISL_688690, EPI_ISL_688691, EPI_ISL_688692, EPI_ISL_688693, EPI_ISL_688694, EPI_ISL_688695, EPI_ISL_688696, EPI_ISL_688697, EPI_ISL_688698, EPI_ISL_688699			
see above	Pathogen Genomics Center, National Institute of Infectious Diseases	Pathogen Genomics Center, National Institute of Infectious Diseases	Tsuyoshi Sekizuka, Kentaro Itokawa, Rina Tanaka, Masanori Hashino, Makoto Kuroda
EPI_ISL_688700, EPI_ISL_688701, EPI_ISL_688702, EPI_ISL_688703, EPI_ISL_688704, EPI_ISL_688705, EPI_ISL_688706, EPI_ISL_688707, EPI_ISL_688708, EPI_ISL_688709	Sakai City Institute of Public Health	Pathogen Genomics Center, National Institute of Infectious Diseases	Tsuyoshi Sekizuka, Kentaro Itokawa, Rina Tanaka, Masanori Hashino, Makoto Kuroda
EPI_ISL_688710, EPI_ISL_688711, EPI_ISL_688712, EPI_ISL_688713, EPI_ISL_688714, EPI_ISL_688715, EPI_ISL_688716, EPI_ISL_688717, EPI_ISL_688718, EPI_ISL_688719, EPI_ISL_688720, EPI_ISL_688721, EPI_ISL_688722, EPI_ISL_688723, EPI_ISL_688724, EPI_ISL_688725, EPI_ISL_688726, EPI_ISL_688727, EPI_ISL_688728, EPI_ISL_688729, EPI_ISL_688730, EPI_ISL_688731, EPI_ISL_688732, EPI_ISL_688733, EPI_ISL_688734, EPI_ISL_688735, EPI_ISL_688736, EPI_ISL_688737, EPI_ISL_688738, EPI_ISL_688739, EPI_ISL_688740, EPI_ISL_688741, EPI_ISL_688742, EPI_ISL_688743, EPI_ISL_688744, EPI_ISL_688745, EPI_ISL_688746, EPI_ISL_688747, EPI_ISL_688748, EPI_ISL_688749, EPI_ISL_688750, EPI_ISL_688751, EPI_ISL_688752, EPI_ISL_688753, EPI_ISL_688754, EPI_ISL_688755, EPI_ISL_688756, EPI_ISL_688757, EPI_ISL_688758, EPI_ISL_688759, EPI_ISL_688760, EPI_ISL_688761, EPI_ISL_688762, EPI_ISL_688763,			

EPI_ISL_688764, EPI_ISL_688765, EPI_ISL_688766, EPI_ISL_688767, EPI_ISL_688768, EPI_ISL_688769, EPI_ISL_688770, EPI_ISL_688771, EPI_ISL_688772, EPI_ISL_688773, EPI_ISL_688774, EPI_ISL_688775, EPI_ISL_688776, EPI_ISL_688777, EPI_ISL_688778, EPI_ISL_688779, EPI_ISL_688780, EPI_ISL_688781, EPI_ISL_688782, EPI_ISL_688783, EPI_ISL_688784, EPI_ISL_688785, EPI_ISL_688786, EPI_ISL_688787, EPI_ISL_688788, EPI_ISL_688789, EPI_ISL_688790, EPI_ISL_688791, EPI_ISL_688792, EPI_ISL_688793, EPI_ISL_688794, EPI_ISL_688795, EPI_ISL_688796, EPI_ISL_688797, EPI_ISL_688798, EPI_ISL_688799, EPI_ISL_688800, EPI_ISL_688801, EPI_ISL_688802, EPI_ISL_688803, EPI_ISL_688804, EPI_ISL_688805, EPI_ISL_688806, EPI_ISL_688807, EPI_ISL_688808, EPI_ISL_688809, EPI_ISL_688810, EPI_ISL_688811, EPI_ISL_688812, EPI_ISL_688813, EPI_ISL_688814, EPI_ISL_688815, EPI_ISL_688816, EPI_ISL_688817, EPI_ISL_688818, EPI_ISL_688819, EPI_ISL_688820, EPI_ISL_688821, EPI_ISL_688822, EPI_ISL_688823, EPI_ISL_688824, EPI_ISL_688825, EPI_ISL_688826, EPI_ISL_688827, EPI_ISL_688828, EPI_ISL_688829, EPI_ISL_688830, EPI_ISL_688831, EPI_ISL_688832, EPI_ISL_688833, EPI_ISL_688834, EPI_ISL_688835, EPI_ISL_688836, EPI_ISL_688837, EPI_ISL_688838, EPI_ISL_688839, EPI_ISL_688840, EPI_ISL_688841, EPI_ISL_688842, EPI_ISL_688843, EPI_ISL_688844, EPI_ISL_688845, EPI_ISL_688846, EPI_ISL_688847, EPI_ISL_688848, EPI_ISL_688849, EPI_ISL_688850, EPI_ISL_688851, EPI_ISL_688852, EPI_ISL_688853, EPI_ISL_688854, EPI_ISL_688855, EPI_ISL_688856, EPI_ISL_688857, EPI_ISL_688858, EPI_ISL_688859, EPI_ISL_688860, EPI_ISL_688861, EPI_ISL_688862, EPI_ISL_688863, EPI_ISL_688864, EPI_ISL_688865, EPI_ISL_688866, EPI_ISL_688867, EPI_ISL_688868, EPI_ISL_688869, EPI_ISL_688870, EPI_ISL_688871, EPI_ISL_688872, EPI_ISL_688873, EPI_ISL_688874, EPI_ISL_688875, EPI_ISL_688876, EPI_ISL_688877, EPI_ISL_688878, EPI_ISL_688879, EPI_ISL_688880, EPI_ISL_688881, EPI_ISL_688882, EPI_ISL_688883, EPI_ISL_688884, EPI_ISL_688885, EPI_ISL_688886, EPI_ISL_688887, EPI_ISL_688888, EPI_ISL_688889, EPI_ISL_688890, EPI_ISL_688891, EPI_ISL_688892, EPI_ISL_688893, EPI_ISL_688894, EPI_ISL_688895, EPI_ISL_688896

see above	Pathogen Genomics Center, National Institute of Infectious Diseases	Pathogen Genomics Center, National Institute of Infectious Diseases	Tsuyoshi Sekizuka, Kentaro Itokawa, Rina Tanaka, Masanori Hashino, Makoto Kuroda

EPI_ISL_688897, EPI_ISL_688898, EPI_ISL_688899, EPI_ISL_688900, EPI_ISL_688901, EPI_ISL_688902, EPI_ISL_688903, EPI_ISL_688904, EPI_ISL_688905, EPI_ISL_688906, EPI_ISL_688907, EPI_ISL_688908, EPI_ISL_688909, EPI_ISL_688910, EPI_ISL_688911, EPI_ISL_688912, EPI_ISL_688913, EPI_ISL_688914, EPI_ISL_688915, EPI_ISL_688916, EPI_ISL_688917, EPI_ISL_688918, EPI_ISL_688919, EPI_ISL_688920, EPI_ISL_688921, EPI_ISL_688922, EPI_ISL_688923, EPI_ISL_688924, EPI_ISL_688925, EPI_ISL_688926, EPI_ISL_688927, EPI_ISL_688928, EPI_ISL_688929, EPI_ISL_688930, EPI_ISL_688931, EPI_ISL_688932, EPI_ISL_688933, EPI_ISL_688934, EPI_ISL_688935, EPI_ISL_688936, EPI_ISL_688937, EPI_ISL_688938, EPI_ISL_688939, EPI_ISL_688940, EPI_ISL_688941, EPI_ISL_688942, EPI_ISL_688943, EPI_ISL_688944, EPI_ISL_688945, EPI_ISL_688946, EPI_ISL_688947, EPI_ISL_688948, EPI_ISL_688949, EPI_ISL_688950, EPI_ISL_688951, EPI_ISL_688952, EPI_ISL_688953, EPI_ISL_688954, EPI_ISL_688955, EPI_ISL_688956, EPI_ISL_688957, EPI_ISL_688958, EPI_ISL_688959, EPI_ISL_688960, EPI_ISL_688961, EPI_ISL_688962, EPI_ISL_688963, EPI_ISL_688964, EPI_ISL_688965, EPI_ISL_688966, EPI_ISL_688967, EPI_ISL_688968

see above	Fukuoka Institute of Health and Environmental Sciences	Pathogen Genomics Center, National Institute of Infectious Diseases	Tsuyoshi Sekizuka, Kentaro Itokawa, Rina Tanaka, Masanori Hashino, Makoto Kuroda

EPI_ISL_688969, EPI_ISL_688970, EPI_ISL_688971, EPI_ISL_688972, EPI_ISL_688973, EPI_ISL_688974, EPI_ISL_688975, EPI_ISL_688976, EPI_ISL_688977, EPI_ISL_688978, EPI_ISL_688979, EPI_ISL_688980, EPI_ISL_688981, EPI_ISL_688982, EPI_ISL_688983, EPI_ISL_688984, EPI_ISL_688985, EPI_ISL_688986, EPI_ISL_688987, EPI_ISL_688988, EPI_ISL_688989, EPI_ISL_688990, EPI_ISL_688991, EPI_ISL_688992, EPI_ISL_688993, EPI_ISL_688994, EPI_ISL_688995, EPI_ISL_688996, EPI_ISL_688997, EPI_ISL_688998, EPI_ISL_688999, EPI_ISL_689000, EPI_ISL_689001, EPI_ISL_689002, EPI_ISL_689003, EPI_ISL_689004, EPI_ISL_689005, EPI_ISL_689006, EPI_ISL_689007, EPI_ISL_689008, EPI_ISL_689009, EPI_ISL_689010, EPI_ISL_689011, EPI_ISL_689012, EPI_ISL_689013, EPI_ISL_689014, EPI_ISL_689015, EPI_ISL_689016, EPI_ISL_689017, EPI_ISL_689018, EPI_ISL_689019, EPI_ISL_689020, EPI_ISL_689021, EPI_ISL_689022, EPI_ISL_689023, EPI_ISL_689024, EPI_ISL_689025, EPI_ISL_689026, EPI_ISL_689027, EPI_ISL_689028, EPI_ISL_689029, EPI_ISL_689030, EPI_ISL_689031, EPI_ISL_689032, EPI_ISL_689033, EPI_ISL_689034, EPI_ISL_689035, EPI_ISL_689036, EPI_ISL_689037, EPI_ISL_689038, EPI_ISL_689039, EPI_ISL_689040, EPI_ISL_689041, EPI_ISL_689042, EPI_ISL_689043, EPI_ISL_689044, EPI_ISL_689045, EPI_ISL_689046

see above	Pathogen Genomics Center, National Institute of Infectious Diseases	Pathogen Genomics Center, National Institute of Infectious Diseases	Tsuyoshi Sekizuka, Kentaro Itokawa, Rina Tanaka, Masanori Hashino, Makoto Kuroda

EPI_ISL_689047, EPI_ISL_689048, EPI_ISL_689049, EPI_ISL_689050, EPI_ISL_689051, EPI_ISL_689052, EPI_ISL_689053, EPI_ISL_689054, EPI_ISL_689055, EPI_ISL_689056, EPI_ISL_689057, EPI_ISL_689058, EPI_ISL_689059, EPI_ISL_689060, EPI_ISL_689061, EPI_ISL_689062, EPI_ISL_689063, EPI_ISL_689064, EPI_ISL_689065, EPI_ISL_689066, EPI_ISL_689067, EPI_ISL_689068, EPI_ISL_689069, EPI_ISL_689070, EPI_ISL_689071, EPI_ISL_689072, EPI_ISL_689073, EPI_ISL_689074, EPI_ISL_689075, EPI_ISL_689076

see above	Gunma Prefectural Institute of Public Health and Environmental Sciences	Pathogen Genomics Center, National Institute of Infectious Diseases	Tsuyoshi Sekizuka, Kentaro Itokawa, Rina Tanaka, Masanori Hashino, Makoto Kuroda

EPI_ISL_689077, EPI_ISL_689078, EPI_ISL_689079, EPI_ISL_689080, EPI_ISL_689081, EPI_ISL_689082, EPI_ISL_689083, EPI_ISL_689084, EPI_ISL_689085, EPI_ISL_689086, EPI_ISL_689087, EPI_ISL_689088

see above	Tokyo Metropolitan Institute of Public Health	Pathogen Genomics Center, National Institute of Infectious Diseases	Tsuyoshi Sekizuka, Kentaro Itokawa, Rina Tanaka, Masanori Hashino, Makoto Kuroda

EPI_ISL_689089, EPI_ISL_689090, EPI_ISL_689091, EPI_ISL_689092, EPI_ISL_689093, EPI_ISL_689094, EPI_ISL_689095, EPI_ISL_689096, EPI_ISL_689097, EPI_ISL_689098, EPI_ISL_689099, EPI_ISL_689100, EPI_ISL_689101, EPI_ISL_689102, EPI_ISL_689103, EPI_ISL_689104, EPI_ISL_689105, EPI_ISL_689106, EPI_ISL_689107, EPI_ISL_689108, EPI_ISL_689109, EPI_ISL_689110, EPI_ISL_689111, EPI_ISL_689112, EPI_ISL_689113, EPI_ISL_689114, EPI_ISL_689115, EPI_ISL_689116, EPI_ISL_689117, EPI_ISL_689118, EPI_ISL_689119, EPI_ISL_689120, EPI_ISL_689121, EPI_ISL_689122, EPI_ISL_689123, EPI_ISL_689124, EPI_ISL_689125, EPI_ISL_689126, EPI_ISL_689127, EPI_ISL_689128, EPI_ISL_689129, EPI_ISL_689130, EPI_ISL_689131, EPI_ISL_689132, EPI_ISL_689133, EPI_ISL_689134, EPI_ISL_689135, EPI_ISL_689136, EPI_ISL_689137, EPI_ISL_689138, EPI_ISL_689139, EPI_ISL_689140, EPI_ISL_689141, EPI_ISL_689142, EPI_ISL_689143, EPI_ISL_689144, EPI_ISL_689145, EPI_ISL_689146, EPI_ISL_689147, EPI_ISL_689148, EPI_ISL_689149, EPI_ISL_689150, EPI_ISL_689151, EPI_ISL_689152, EPI_ISL_689153, EPI_ISL_689154, EPI_ISL_689155, EPI_ISL_689156, EPI_ISL_689157, EPI_ISL_689158, EPI_ISL_689159, EPI_ISL_689160, EPI_ISL_689161, EPI_ISL_689162, EPI_ISL_689163, EPI_ISL_689164, EPI_ISL_689165, EPI_ISL_689166, EPI_ISL_689167, EPI_ISL_689168, EPI_ISL_689169, EPI_ISL_689170, EPI_ISL_689171, EPI_ISL_689172, EPI_ISL_689173, EPI_ISL_689174, EPI_ISL_689175, EPI_ISL_689176, EPI_ISL_689177, EPI_ISL_689178, EPI_ISL_689179, EPI_ISL_689180, EPI_ISL_689181, EPI_ISL_689182, EPI_ISL_689183, EPI_ISL_689184, EPI_ISL_689185, EPI_ISL_689186, EPI_ISL_689187, EPI_ISL_689188, EPI_ISL_689189, EPI_ISL_689190, EPI_ISL_689191, EPI_ISL_689192, EPI_ISL_689193, EPI_ISL_689194, EPI_ISL_689195, EPI_ISL_689196, EPI_ISL_689197, EPI_ISL_689198, EPI_ISL_689199, EPI_ISL_689200, EPI_ISL_689201, EPI_ISL_689202, EPI_ISL_689203, EPI_ISL_689204, EPI_ISL_689205, EPI_ISL_689206, EPI_ISL_689207, EPI_ISL_689208, EPI_ISL_689209, EPI_ISL_689210, EPI_ISL_689211, EPI_ISL_689212, EPI_ISL_689213, EPI_ISL_689214, EPI_ISL_689215, EPI_ISL_689216, EPI_ISL_689217, EPI_ISL_689218, EPI_ISL_689219, EPI_ISL_689220, EPI_ISL_689221, EPI_ISL_689222, EPI_ISL_689223, EPI_ISL_689224, EPI_ISL_689225, EPI_ISL_689226, EPI_ISL_689227, EPI_ISL_689228, EPI_ISL_689229, EPI_ISL_689230, EPI_ISL_689231, EPI_ISL_689232, EPI_ISL_689233, EPI_ISL_689234, EPI_ISL_689235, EPI_ISL_689236, EPI_ISL_689237, EPI_ISL_689238, EPI_ISL_689239, EPI_ISL_689240, EPI_ISL_689241, EPI_ISL_689242, EPI_ISL_689243, EPI_ISL_689244, EPI_ISL_689245, EPI_ISL_689246, EPI_ISL_689247, EPI_ISL_689248, EPI_ISL_689249, EPI_ISL_689250, EPI_ISL_689251, EPI_ISL_689252, EPI_ISL_689253, EPI_ISL_689254, EPI_ISL_689255, EPI_ISL_689256, EPI_ISL_689257, EPI_ISL_689258, EPI_ISL_689259, EPI_ISL_689260, EPI_ISL_689261, EPI_ISL_689262, EPI_ISL_689263, EPI_ISL_689264, EPI_ISL_689265, EPI_ISL_689266, EPI_ISL_689267, EPI_ISL_689268, EPI_ISL_689269, EPI_ISL_689270, EPI_ISL_689271, EPI_ISL_689272, EPI_ISL_689273, EPI_ISL_689274, EPI_ISL_689275, EPI_ISL_689276, EPI_ISL_689277, EPI_ISL_689278, EPI_ISL_689279, EPI_ISL_689280, EPI_ISL_689281, EPI_ISL_689282, EPI_ISL_689283, EPI_ISL_689284, EPI_ISL_689285, EPI_ISL_689286, EPI_ISL_689287, EPI_ISL_689288, EPI_ISL_689289, EPI_ISL_689290, EPI_ISL_689291, EPI_ISL_689292, EPI_ISL_689293, EPI_ISL_689294, EPI_ISL_689295, EPI_ISL_689296, EPI_ISL_689297, EPI_ISL_689298, EPI_ISL_689299, EPI_ISL_689300, EPI_ISL_689301, EPI_ISL_689302, EPI_ISL_689303, EPI_ISL_689304, EPI_ISL_689305, EPI_ISL_689306, EPI_ISL_689307, EPI_ISL_689308, EPI_ISL_689309, EPI_ISL_689310, EPI_ISL_689311, EPI_ISL_689312, EPI_ISL_689313, EPI_ISL_689314, EPI_ISL_689315, EPI_ISL_689316, EPI_ISL_689317, EPI_ISL_689318, EPI_ISL_689319, EPI_ISL_689320, EPI_ISL_689321, EPI_ISL_689322, EPI_ISL_689323, EPI_ISL_689324, EPI_ISL_689325, EPI_ISL_689326, EPI_ISL_689327, EPI_ISL_689328, EPI_ISL_689329, EPI_ISL_689330, EPI_ISL_689331, EPI_ISL_689332, EPI_ISL_689333, EPI_ISL_689334, EPI_ISL_689335, EPI_ISL_689336, EPI_ISL_689337, EPI_ISL_689338, EPI_ISL_689339, EPI_ISL_689340, EPI_ISL_689341, EPI_ISL_689342, EPI_ISL_689343, EPI_ISL_689344, EPI_ISL_689345, EPI_ISL_689346, EPI_ISL_689347, EPI_ISL_689348, EPI_ISL_689349, EPI_ISL_689350, EPI_ISL_689351, EPI_ISL_689352, EPI_ISL_689353, EPI_ISL_689354, EPI_ISL_689355, EPI_ISL_689356, EPI_ISL_689357, EPI_ISL_689358, EPI_ISL_689359, EPI_ISL_689360, EPI_ISL_689361, EPI_ISL_689362, EPI_ISL_689363, EPI_ISL_689364, EPI_ISL_689365, EPI_ISL_689366, EPI_ISL_689367, EPI_ISL_689368, EPI_ISL_689369, EPI_ISL_689370, EPI_ISL_689371, EPI_ISL_689372, EPI_ISL_689373, EPI_ISL_689374, EPI_ISL_689375, EPI_ISL_689376, EPI_ISL_689377, EPI_ISL_689378, EPI_ISL_689379, EPI_ISL_689380, EPI_ISL_689381, EPI_ISL_689382, EPI_ISL_689383, EPI_ISL_689384, EPI_ISL_689385, EPI_ISL_689386, EPI_ISL_689387, EPI_ISL_689388, EPI_ISL_689389, EPI_ISL_689390, EPI_ISL_689391, EPI_ISL_689392, EPI_ISL_689393, EPI_ISL_689394, EPI_ISL_689395, EPI_ISL_689396, EPI_ISL_689397, EPI_ISL_689398, EPI_ISL_689399, EPI_ISL_689400, EPI_ISL_689401, EPI_ISL_689402, EPI_ISL_689403, EPI_ISL_689404, EPI_ISL_689405, EPI_ISL_689406, EPI_ISL_689407, EPI_ISL_689408, EPI_ISL_689409, EPI_ISL_689410, EPI_ISL_689411, EPI_ISL_689412, EPI_ISL_689413, EPI_ISL_689414, EPI_ISL_689415, EPI_ISL_689416, EPI_ISL_689417, EPI_ISL_689418, EPI_ISL_689419, EPI_ISL_689420, EPI_ISL_689421, EPI_ISL_689422, EPI_ISL_689423, EPI_ISL_689424, EPI_ISL_689425, EPI_ISL_689426, EPI_ISL_689427, EPI_ISL_689428, EPI_ISL_689429, EPI_ISL_689430, EPI_ISL_689431, EPI_ISL_689432, EPI_ISL_689433, EPI_ISL_689434, EPI_ISL_689435, EPI_ISL_689436, EPI_ISL_689437, EPI_ISL_689438, EPI_ISL_689439, EPI_ISL_689440, EPI_ISL_689441, EPI_ISL_689442, EPI_ISL_689443, EPI_ISL_689444, EPI_ISL_689445, EPI_ISL_689446, EPI_ISL_689447, EPI_ISL_689448, EPI_ISL_689449, EPI_ISL_689450, EPI_ISL_689451, EPI_ISL_689452, EPI_ISL_689453, EPI_ISL_689454, EPI_ISL_689455, EPI_ISL_689456, EPI_ISL_689457, EPI_ISL_689458, EPI_ISL_689459

see above	Pathogen Genomics Center, National Institute of Infectious Diseases	Pathogen Genomics Center, National Institute of Infectious Diseases	Tsuyoshi Sekizuka, Kentaro Itokawa, Rina Tanaka, Masanori Hashino, Makoto Kuroda

EPI_ISL_689460, EPI_ISL_689461, EPI_ISL_689462, EPI_ISL_689463, EPI_ISL_689464, EPI_ISL_689465, EPI_ISL_689466, EPI_ISL_689467, EPI_ISL_689468, EPI_ISL_689469, EPI_ISL_689470, EPI_ISL_689471, EPI_ISL_689472, EPI_ISL_689473, EPI_ISL_689474, EPI_ISL_689475, EPI_ISL_689476, EPI_ISL_689477, EPI_ISL_689478, EPI_ISL_689479, EPI_ISL_689480, EPI_ISL_689481, EPI_ISL_689482, EPI_ISL_689483, EPI_ISL_689484, EPI_ISL_689485, EPI_ISL_689486, EPI_ISL_689487, EPI_ISL_689488, EPI_ISL_689489, EPI_ISL_689490, EPI_ISL_689491, EPI_ISL_689492, EPI_ISL_689493, EPI_ISL_689494, EPI_ISL_689495, EPI_ISL_689496

see above	Fukuoka Institute of Health and Environmental Sciences	Pathogen Genomics Center, National Institute of Infectious Diseases	Tsuyoshi Sekizuka, Kentaro Itokawa, Rina Tanaka, Masanori Hashino, Makoto Kuroda

EPI_ISL_689497, EPI_ISL_689498, EPI_ISL_689499, EPI_ISL_689500, EPI_ISL_689501, EPI_ISL_689502, EPI_ISL_689503, EPI_ISL_689504, EPI_ISL_689505, EPI_ISL_689506, EPI_ISL_689507, EPI_ISL_689508, EPI_ISL_689509, EPI_ISL_689510, EPI_ISL_689511, EPI_ISL_689512, EPI_ISL_689513, EPI_ISL_689514, EPI_ISL_689515, EPI_ISL_689516, EPI_ISL_689517, EPI_ISL_689518, EPI_ISL_689519, EPI_ISL_689520, EPI_ISL_689521, EPI_ISL_689522, EPI_ISL_689523, EPI_ISL_689524, EPI_ISL_689525, EPI_ISL_689526, EPI_ISL_689527, EPI_ISL_689528, EPI_ISL_689529, EPI_ISL_689530, EPI_ISL_689531, EPI_ISL_689532, EPI_ISL_689533, EPI_ISL_689534, EPI_ISL_689535, EPI_ISL_689536, EPI_ISL_689537, EPI_ISL_689538, EPI_ISL_689539, EPI_ISL_689540, EPI_ISL_689541, EPI_ISL_689542, EPI_ISL_689543, EPI_ISL_689544, EPI_ISL_689545, EPI_ISL_689546, EPI_ISL_689547, EPI_ISL_689548, EPI_ISL_689549, EPI_ISL_689550, EPI_ISL_689551, EPI_ISL_689552, EPI_ISL_689553, EPI_ISL_689554, EPI_ISL_689555, EPI_ISL_689556, EPI_ISL_689557, EPI_ISL_689558, EPI_ISL_689559, EPI_ISL_689560, EPI_ISL_689561, EPI_ISL_689562, EPI_ISL_689563, EPI_ISL_689564, EPI_ISL_689565, EPI_ISL_689566, EPI_ISL_689567, EPI_ISL_689568, EPI_ISL_689569, EPI_ISL_689570, EPI_ISL_689571, EPI_ISL_689572, EPI_ISL_689573, EPI_ISL_689574, EPI_ISL_689575, EPI_ISL_689576, EPI_ISL_689577, EPI_ISL_689578, EPI_ISL_689579, EPI_ISL_689580, EPI_ISL_689581, EPI_ISL_689582, EPI_ISL_689583, EPI_ISL_689584, EPI_ISL_689585, EPI_ISL_689586, EPI_ISL_689587, EPI_ISL_689588, EPI_ISL_689589, EPI_ISL_689590, EPI_ISL_689591, EPI_ISL_689592, EPI_ISL_689593, EPI_ISL_689594, EPI_ISL_689595, EPI_ISL_689596, EPI_ISL_689597, EPI_ISL_689598, EPI_ISL_689599, EPI_ISL_689600, EPI_ISL_689601, EPI_ISL_689602, EPI_ISL_689603, EPI_ISL_689604, EPI_ISL_689605, EPI_ISL_689606, EPI_ISL_689607, EPI_ISL_689608, EPI_ISL_689609, EPI_ISL_689610, EPI_ISL_689611

see above	Pathogen Genomics Center, National Institute of Infectious Diseases	Pathogen Genomics Center, National Institute of Infectious Diseases	Tsuyoshi Sekizuka, Kentaro Itokawa, Rina Tanaka, Masanori Hashino, Makoto Kuroda
EPI_ISL_689612	Gunma Prefectural Institute of Public Health and Environmental Sciences	Pathogen Genomics Center, National Institute of Infectious Diseases	Tsuyoshi Sekizuka, Kentaro Itokawa, Rina Tanaka, Masanori Hashino, Makoto Kuroda
EPI_ISL_689613, EPI_ISL_689614, EPI_ISL_689615, EPI_ISL_689616	Pathogen Genomics Center, National Institute of Infectious Diseases	Pathogen Genomics Center, National Institute of Infectious Diseases	Tsuyoshi Sekizuka, Kentaro Itokawa, Rina Tanaka, Masanori Hashino, Makoto Kuroda
EPI_ISL_689617	Gunma Prefectural Institute of Public Health and Environmental Sciences	Pathogen Genomics Center, National Institute of Infectious Diseases	Tsuyoshi Sekizuka, Kentaro Itokawa, Rina Tanaka, Masanori Hashino, Makoto Kuroda

EPI_ISL_689618, EPI_ISL_689619, EPI_ISL_689620, EPI_ISL_689621, EPI_ISL_689622, EPI_ISL_689623, EPI_ISL_689624, EPI_ISL_689625, EPI_ISL_689626, EPI_ISL_689627, EPI_ISL_689628, EPI_ISL_689629, EPI_ISL_689630, EPI_ISL_689631, EPI_ISL_689632, EPI_ISL_689633, EPI_ISL_689634, EPI_ISL_689635, EPI_ISL_689636, EPI_ISL_689637, EPI_ISL_689638, EPI_ISL_689639, EPI_ISL_689640, EPI_ISL_689641, EPI_ISL_689642, EPI_ISL_689643, EPI_ISL_689644, EPI_ISL_689645

see above	Pathogen Genomics Center, National Institute of Infectious Diseases	Pathogen Genomics Center, National Institute of Infectious Diseases	Tsuyoshi Sekizuka, Kentaro Itokawa, Rina Tanaka, Masanori Hashino, Makoto Kuroda

EPI_ISL_689646	Fukuoka Institute of Health and Environmental Sciences	Pathogen Genomics Center, National Institute of Infectious Diseases	Tsuyoshi Sekizuka, Kentaro Itokawa, Rina Tanaka, Masanori Hashino, Makoto Kuroda
EPI_ISL_689647, EPI_ISL_689648, EPI_ISL_689649, EPI_ISL_689650, EPI_ISL_689651, EPI_ISL_689652, EPI_ISL_689653, EPI_ISL_689654, EPI_ISL_689655, EPI_ISL_689656, EPI_ISL_689657, EPI_ISL_689658, EPI_ISL_689659, EPI_ISL_689660, EPI_ISL_689661, EPI_ISL_689662, EPI_ISL_689663, EPI_ISL_689664, EPI_ISL_689665, EPI_ISL_689666, EPI_ISL_689667, EPI_ISL_689668, EPI_ISL_689669, EPI_ISL_689670, EPI_ISL_689671, EPI_ISL_689672, EPI_ISL_689673, EPI_ISL_689674, EPI_ISL_689675, EPI_ISL_689676, EPI_ISL_689677, EPI_ISL_689678, EPI_ISL_689679, EPI_ISL_689680, EPI_ISL_689681, EPI_ISL_689682, EPI_ISL_689683, EPI_ISL_689684, EPI_ISL_689685			
see above	Pathogen Genomics Center, National Institute of Infectious Diseases	Pathogen Genomics Center, National Institute of Infectious Diseases	Tsuyoshi Sekizuka, Kentaro Itokawa, Rina Tanaka, Masanori Hashino, Makoto Kuroda
EPI_ISL_689686, EPI_ISL_689687, EPI_ISL_689688, EPI_ISL_689689, EPI_ISL_689690, EPI_ISL_689691	Gunma Prefectural Institute of Public Health and Environmental Sciences	Pathogen Genomics Center, National Institute of Infectious Diseases	Tsuyoshi Sekizuka, Kentaro Itokawa, Rina Tanaka, Masanori Hashino, Makoto Kuroda
EPI_ISL_689692, EPI_ISL_689693, EPI_ISL_689694, EPI_ISL_689695, EPI_ISL_689696, EPI_ISL_689697	Fukuoka Institute of Health and Environmental Sciences	Pathogen Genomics Center, National Institute of Infectious Diseases	Tsuyoshi Sekizuka, Kentaro Itokawa, Rina Tanaka, Masanori Hashino, Makoto Kuroda
EPI_ISL_689698, EPI_ISL_689699, EPI_ISL_689700, EPI_ISL_689701, EPI_ISL_689702, EPI_ISL_689703, EPI_ISL_689704, EPI_ISL_689705, EPI_ISL_689706, EPI_ISL_689707, EPI_ISL_689708, EPI_ISL_689709, EPI_ISL_689710, EPI_ISL_689711, EPI_ISL_689712, EPI_ISL_689713, EPI_ISL_689714, EPI_ISL_689715, EPI_ISL_689716, EPI_ISL_689717, EPI_ISL_689718, EPI_ISL_689719, EPI_ISL_689720, EPI_ISL_689721, EPI_ISL_689722, EPI_ISL_689723, EPI_ISL_689724, EPI_ISL_689725, EPI_ISL_689726, EPI_ISL_689727, EPI_ISL_689728, EPI_ISL_689729, EPI_ISL_689730, EPI_ISL_689731, EPI_ISL_689732, EPI_ISL_689733, EPI_ISL_689734, EPI_ISL_689735, EPI_ISL_689736, EPI_ISL_689737, EPI_ISL_689738, EPI_ISL_689739, EPI_ISL_689740, EPI_ISL_689741, EPI_ISL_689742, EPI_ISL_689743, EPI_ISL_689744, EPI_ISL_689745, EPI_ISL_689746, EPI_ISL_689747, EPI_ISL_689748, EPI_ISL_689749, EPI_ISL_689750, EPI_ISL_689751, EPI_ISL_689752, EPI_ISL_689753, EPI_ISL_689754, EPI_ISL_689755, EPI_ISL_689756, EPI_ISL_689757, EPI_ISL_689758			
see above	Pathogen Genomics Center, National Institute of Infectious Diseases	Pathogen Genomics Center, National Institute of Infectious Diseases	Tsuyoshi Sekizuka, Kentaro Itokawa, Rina Tanaka, Masanori Hashino, Makoto Kuroda
EPI_ISL_689759, EPI_ISL_689760, EPI_ISL_689761, EPI_ISL_689762, EPI_ISL_689763, EPI_ISL_689764, EPI_ISL_689765, EPI_ISL_689766, EPI_ISL_689767, EPI_ISL_689768, EPI_ISL_689769, EPI_ISL_689770, EPI_ISL_689771, EPI_ISL_689772, EPI_ISL_689773, EPI_ISL_689774, EPI_ISL_689775, EPI_ISL_689776, EPI_ISL_689777, EPI_ISL_689778, EPI_ISL_689779, EPI_ISL_689780, EPI_ISL_689781, EPI_ISL_689782, EPI_ISL_689783, EPI_ISL_689784, EPI_ISL_689785, EPI_ISL_689786, EPI_ISL_689787, EPI_ISL_689788, EPI_ISL_689789, EPI_ISL_689790, EPI_ISL_689791, EPI_ISL_689792, EPI_ISL_689793, EPI_ISL_689794, EPI_ISL_689795, EPI_ISL_689796, EPI_ISL_689797, EPI_ISL_689798, EPI_ISL_689799, EPI_ISL_689800, EPI_ISL_689801, EPI_ISL_689802, EPI_ISL_689803, EPI_ISL_689804, EPI_ISL_689805, EPI_ISL_689806, EPI_ISL_689807, EPI_ISL_689808, EPI_ISL_689809, EPI_ISL_689810, EPI_ISL_689811, EPI_ISL_689812, EPI_ISL_689813, EPI_ISL_689814, EPI_ISL_689815, EPI_ISL_689816, EPI_ISL_689817, EPI_ISL_689818, EPI_ISL_689819, EPI_ISL_689820, EPI_ISL_689821, EPI_ISL_689822, EPI_ISL_689823, EPI_ISL_689824, EPI_ISL_689825			
see above	Fukuoka Institute of Health and Environmental Sciences	Pathogen Genomics Center, National Institute of Infectious Diseases	Tsuyoshi Sekizuka, Kentaro Itokawa, Rina Tanaka, Masanori Hashino, Makoto Kuroda
EPI_ISL_689826, EPI_ISL_689827, EPI_ISL_689828, EPI_ISL_689829, EPI_ISL_689830, EPI_ISL_689831, EPI_ISL_689832, EPI_ISL_689833, EPI_ISL_689834, EPI_ISL_689835, EPI_ISL_689836, EPI_ISL_689837, EPI_ISL_689838, EPI_ISL_689839, EPI_ISL_689840, EPI_ISL_689841, EPI_ISL_689842, EPI_ISL_689843, EPI_ISL_689844, EPI_ISL_689845, EPI_ISL_689846, EPI_ISL_689847, EPI_ISL_689848, EPI_ISL_689849, EPI_ISL_689850, EPI_ISL_689851, EPI_ISL_689852, EPI_ISL_689853, EPI_ISL_689854, EPI_ISL_689855, EPI_ISL_689856, EPI_ISL_689857, EPI_ISL_689858, EPI_ISL_689859, EPI_ISL_689860, EPI_ISL_689861, EPI_ISL_689862, EPI_ISL_689863, EPI_ISL_689864, EPI_ISL_689865, EPI_ISL_689866, EPI_ISL_689867, EPI_ISL_689868, EPI_ISL_689869, EPI_ISL_689870, EPI_ISL_689871, EPI_ISL_689872, EPI_ISL_689873, EPI_ISL_689874, EPI_ISL_689875, EPI_ISL_689876, EPI_ISL_689877, EPI_ISL_689878, EPI_ISL_689879, EPI_ISL_689880, EPI_ISL_689881, EPI_ISL_689882, EPI_ISL_689883, EPI_ISL_689884, EPI_ISL_689885, EPI_ISL_689886, EPI_ISL_689887, EPI_ISL_689888, EPI_ISL_689889, EPI_ISL_689890, EPI_ISL_689891, EPI_ISL_689892, EPI_ISL_689893, EPI_ISL_689894, EPI_ISL_689895, EPI_ISL_689896, EPI_ISL_689897, EPI_ISL_689898, EPI_ISL_689899, EPI_ISL_689900, EPI_ISL_689901, EPI_ISL_689902, EPI_ISL_689903, EPI_ISL_689904, EPI_ISL_689905, EPI_ISL_689906, EPI_ISL_689907, EPI_ISL_689908, EPI_ISL_689909, EPI_ISL_689910, EPI_ISL_689911, EPI_ISL_689912, EPI_ISL_689913, EPI_ISL_689914, EPI_ISL_689915, EPI_ISL_689916, EPI_ISL_689917, EPI_ISL_689918, EPI_ISL_689919, EPI_ISL_689920, EPI_ISL_689921, EPI_ISL_689922, EPI_ISL_689923, EPI_ISL_689924, EPI_ISL_689925, EPI_ISL_689926, EPI_ISL_689927, EPI_ISL_689928, EPI_ISL_689929, EPI_ISL_689930, EPI_ISL_689931, EPI_ISL_689932, EPI_ISL_689933, EPI_ISL_689934, EPI_ISL_689935, EPI_ISL_689936, EPI_ISL_689937, EPI_ISL_689938, EPI_ISL_689939, EPI_ISL_689940, EPI_ISL_689941, EPI_ISL_689942, EPI_ISL_689943, EPI_ISL_689944, EPI_ISL_689945, EPI_ISL_689946, EPI_ISL_689947, EPI_ISL_689948, EPI_ISL_689949, EPI_ISL_689950, EPI_ISL_689951, EPI_ISL_689952, EPI_ISL_689953, EPI_ISL_689954, EPI_ISL_689955, EPI_ISL_689956, EPI_ISL_689957, EPI_ISL_689958, EPI_ISL_689959, EPI_ISL_689960, EPI_ISL_689961, EPI_ISL_689962, EPI_ISL_689963, EPI_ISL_689964, EPI_ISL_689965, EPI_ISL_689966, EPI_ISL_689967, EPI_ISL_689968, EPI_ISL_689969, EPI_ISL_689970, EPI_ISL_689971, EPI_ISL_689972, EPI_ISL_689973, EPI_ISL_689974, EPI_ISL_689975, EPI_ISL_689976, EPI_ISL_689977, EPI_ISL_689978, EPI_ISL_689979, EPI_ISL_689980, EPI_ISL_689981, EPI_ISL_689982, EPI_ISL_689983, EPI_ISL_689984, EPI_ISL_689985, EPI_ISL_689986, EPI_ISL_689987, EPI_ISL_689988, EPI_ISL_689989, EPI_ISL_689990, EPI_ISL_689991, EPI_ISL_689992, EPI_ISL_689993, EPI_ISL_689994, EPI_ISL_689995, EPI_ISL_689996, EPI_ISL_689997, EPI_ISL_689998, EPI_ISL_689999, EPI_ISL_690000, EPI_ISL_690001, EPI_ISL_690002, EPI_ISL_690003, EPI_ISL_690004, EPI_ISL_690005, EPI_ISL_690006, EPI_ISL_690007, EPI_ISL_690008, EPI_ISL_690009, EPI_ISL_690010			
see above	Pathogen Genomics Center, National Institute of Infectious Diseases	Pathogen Genomics Center, National Institute of Infectious Diseases	Tsuyoshi Sekizuka, Kentaro Itokawa, Rina Tanaka, Masanori Hashino, Makoto Kuroda
EPI_ISL_690011, EPI_ISL_690012, EPI_ISL_690013, EPI_ISL_690014, EPI_ISL_690015, EPI_ISL_690016, EPI_ISL_690017, EPI_ISL_690018, EPI_ISL_690019, EPI_ISL_690020, EPI_ISL_690021, EPI_ISL_690022, EPI_ISL_690023, EPI_ISL_690024, EPI_ISL_690025, EPI_ISL_690026, EPI_ISL_690027, EPI_ISL_690028			
see above	Sakai City Institute of Public Health	Pathogen Genomics Center, National Institute of Infectious Diseases	Tsuyoshi Sekizuka, Kentaro Itokawa, Rina Tanaka, Masanori Hashino, Makoto Kuroda
EPI_ISL_690029, EPI_ISL_690030, EPI_ISL_690031, EPI_ISL_690032, EPI_ISL_690033, EPI_ISL_690034, EPI_ISL_690035, EPI_ISL_690036, EPI_ISL_690037, EPI_ISL_690038, EPI_ISL_690039, EPI_ISL_690040, EPI_ISL_690041, EPI_ISL_690042, EPI_ISL_690043, EPI_ISL_690044, EPI_ISL_690045, EPI_ISL_690046, EPI_ISL_690047, EPI_ISL_690048, EPI_ISL_690049, EPI_ISL_690050, EPI_ISL_690051, EPI_ISL_690052, EPI_ISL_690053, EPI_ISL_690054, EPI_ISL_690055, EPI_ISL_690056, EPI_ISL_690057, EPI_ISL_690058, EPI_ISL_690059, EPI_ISL_690060, EPI_ISL_690061, EPI_ISL_690062, EPI_ISL_690063, EPI_ISL_690064, EPI_ISL_690065, EPI_ISL_690066, EPI_ISL_690067, EPI_ISL_690068, EPI_ISL_690069, EPI_ISL_690070, EPI_ISL_690071, EPI_ISL_690072, EPI_ISL_690073, EPI_ISL_690074, EPI_ISL_690075, EPI_ISL_690076, EPI_ISL_690077, EPI_ISL_690078, EPI_ISL_690079, EPI_ISL_690080, EPI_ISL_690081, EPI_ISL_690082, EPI_ISL_690083, EPI_ISL_690084, EPI_ISL_690085, EPI_ISL_690086, EPI_ISL_690087, EPI_ISL_690088, EPI_ISL_690089, EPI_ISL_690090, EPI_ISL_690091, EPI_ISL_690092, EPI_ISL_690093, EPI_ISL_690094, EPI_ISL_690095, EPI_ISL_690096, EPI_ISL_690097, EPI_ISL_690098, EPI_ISL_690099, EPI_ISL_690100, EPI_ISL_690101, EPI_ISL_690102, EPI_ISL_690103, EPI_ISL_690104, EPI_ISL_690105, EPI_ISL_690106, EPI_ISL_690107, EPI_ISL_690108, EPI_ISL_690109, EPI_ISL_690110, EPI_ISL_690111, EPI_ISL_690112, EPI_ISL_690113, EPI_ISL_690114, EPI_ISL_690115, EPI_ISL_690116, EPI_ISL_690117, EPI_ISL_690118, EPI_ISL_690119, EPI_ISL_690120, EPI_ISL_690121, EPI_ISL_690122, EPI_ISL_690123, EPI_ISL_690124, EPI_ISL_690125, EPI_ISL_690126, EPI_ISL_690127, EPI_ISL_690128, EPI_ISL_690129, EPI_ISL_690130, EPI_ISL_690131, EPI_ISL_690132, EPI_ISL_690133, EPI_ISL_690134, EPI_ISL_690135, EPI_ISL_690136, EPI_ISL_690137, EPI_ISL_690138, EPI_ISL_690139, EPI_ISL_690140, EPI_ISL_690141, EPI_ISL_690142, EPI_ISL_690143, EPI_ISL_690144, EPI_ISL_690145, EPI_ISL_690146, EPI_ISL_690147, EPI_ISL_690148, EPI_ISL_690149, EPI_ISL_690150, EPI_ISL_690151, EPI_ISL_690152, EPI_ISL_690153, EPI_ISL_690154, EPI_ISL_690155, EPI_ISL_690156, EPI_ISL_690157, EPI_ISL_690158, EPI_ISL_690159, EPI_ISL_690160, EPI_ISL_690161, EPI_ISL_690162, EPI_ISL_690163, EPI_ISL_690164, EPI_ISL_690165, EPI_ISL_690166, EPI_ISL_690167, EPI_ISL_690168, EPI_ISL_690169, EPI_ISL_690170, EPI_ISL_690171, EPI_ISL_690172, EPI_ISL_690173, EPI_ISL_690174, EPI_ISL_690175, EPI_ISL_690176, EPI_ISL_690177, EPI_ISL_690178, EPI_ISL_690179, EPI_ISL_690180, EPI_ISL_690181, EPI_ISL_690182, EPI_ISL_690183, EPI_ISL_690184, EPI_ISL_690185, EPI_ISL_690186, EPI_ISL_690187, EPI_ISL_690188, EPI_ISL_690189, EPI_ISL_690190, EPI_ISL_690191, EPI_ISL_690192, EPI_ISL_690193, EPI_ISL_690194, EPI_ISL_690195, EPI_ISL_690196, EPI_ISL_690197, EPI_ISL_690198, EPI_ISL_690199, EPI_ISL_690200, EPI_ISL_690201, EPI_ISL_690202, EPI_ISL_690203, EPI_ISL_690204, EPI_ISL_690205, EPI_ISL_690206, EPI_ISL_690207, EPI_ISL_690208, EPI_ISL_690209			
see above	Pathogen Genomics Center, National Institute of Infectious Diseases	Pathogen Genomics Center, National Institute of Infectious Diseases	Tsuyoshi Sekizuka, Kentaro Itokawa, Rina Tanaka, Masanori Hashino, Makoto Kuroda
EPI_ISL_690210, EPI_ISL_690211, EPI_ISL_690212, EPI_ISL_690213, EPI_ISL_690214, EPI_ISL_690215, EPI_ISL_690216, EPI_ISL_690217, EPI_ISL_690218, EPI_ISL_690219, EPI_ISL_690220, EPI_ISL_690221, EPI_ISL_690222, EPI_ISL_690223, EPI_ISL_690224, EPI_ISL_690225, EPI_ISL_690226, EPI_ISL_690227, EPI_ISL_690228, EPI_ISL_690229, EPI_ISL_690230, EPI_ISL_690231, EPI_ISL_690232, EPI_ISL_690233, EPI_ISL_690234, EPI_ISL_690235, EPI_ISL_690236, EPI_ISL_690237, EPI_ISL_690238, EPI_ISL_690239, EPI_ISL_690240, EPI_ISL_690241, EPI_ISL_690242, EPI_ISL_690243, EPI_ISL_690244, EPI_ISL_690245, EPI_ISL_690246, EPI_ISL_690247, EPI_ISL_690248, EPI_ISL_690249, EPI_ISL_690250, EPI_ISL_690251, EPI_ISL_690252, EPI_ISL_690253			
see above	Gunma Prefectural Institute of Public Health and Environmental Sciences	Pathogen Genomics Center, National Institute of Infectious Diseases	Tsuyoshi Sekizuka, Kentaro Itokawa, Rina Tanaka, Masanori Hashino, Makoto Kuroda
EPI_ISL_690254, EPI_ISL_690255, EPI_ISL_690256, EPI_ISL_690257, EPI_ISL_690258, EPI_ISL_690259, EPI_ISL_690260, EPI_ISL_690261, EPI_ISL_690262, EPI_ISL_690263, EPI_ISL_690264, EPI_ISL_690265, EPI_ISL_690266, EPI_ISL_690267, EPI_ISL_690268, EPI_ISL_690269, EPI_ISL_690270, EPI_ISL_690271, EPI_ISL_690272, EPI_ISL_690273, EPI_ISL_690274, EPI_ISL_690275, EPI_ISL_690276, EPI_ISL_690277, EPI_ISL_690278, EPI_ISL_690279, EPI_ISL_690280, EPI_ISL_690281, EPI_ISL_690282, EPI_ISL_690283, EPI_ISL_690284, EPI_ISL_690285, EPI_ISL_690286, EPI_ISL_690287, EPI_ISL_690288, EPI_ISL_690289, EPI_ISL_690290, EPI_ISL_690291, EPI_ISL_690292, EPI_ISL_690293, EPI_ISL_690294, EPI_ISL_690295, EPI_ISL_690296, EPI_ISL_690297, EPI_ISL_690298, EPI_ISL_690299, EPI_ISL_690300, EPI_ISL_690301, EPI_ISL_690302, EPI_ISL_690303, EPI_ISL_690304, EPI_ISL_690305, EPI_ISL_690306, EPI_ISL_690307, EPI_ISL_690308, EPI_ISL_690309, EPI_ISL_690310, EPI_ISL_690311, EPI_ISL_690312, EPI_ISL_690313, EPI_ISL_690314, EPI_ISL_690315, EPI_ISL_690316, EPI_ISL_690317, EPI_ISL_690318, EPI_ISL_690319, EPI_ISL_690320, EPI_ISL_690321, EPI_ISL_690322, EPI_ISL_690323, EPI_ISL_690324, EPI_ISL_690325, EPI_ISL_690326, EPI_ISL_690327, EPI_ISL_690328, EPI_ISL_690329, EPI_ISL_690330, EPI_ISL_690331, EPI_ISL_690332, EPI_ISL_690333, EPI_ISL_690334, EPI_ISL_690335, EPI_ISL_690336, EPI_ISL_690337, EPI_ISL_690338, EPI_ISL_690339, EPI_ISL_690340, EPI_ISL_690341, EPI_ISL_690342, EPI_ISL_690343, EPI_ISL_690344, EPI_ISL_690345, EPI_ISL_690346, EPI_ISL_690347, EPI_ISL_690348, EPI_ISL_690349, EPI_ISL_690350, EPI_ISL_690351, EPI_ISL_690352, EPI_ISL_690353, EPI_ISL_690354, EPI_ISL_690355, EPI_ISL_690356, EPI_ISL_690357, EPI_ISL_690358, EPI_ISL_690359, EPI_ISL_690360, EPI_ISL_690361, EPI_ISL_690362, EPI_ISL_690363, EPI_ISL_690364, EPI_ISL_690365, EPI_ISL_690366, EPI_ISL_690367, EPI_ISL_690368, EPI_ISL_690369, EPI_ISL_690370, EPI_ISL_690371, EPI_ISL_690372, EPI_ISL_690373, EPI_ISL_690374, EPI_ISL_690375, EPI_ISL_690376, EPI_ISL_690377, EPI_ISL_690378, EPI_ISL_690379, EPI_ISL_690380, EPI_ISL_690381, EPI_ISL_690382, EPI_ISL_690383, EPI_ISL_690384, EPI_ISL_690385, EPI_ISL_690386, EPI_ISL_690387, EPI_ISL_690388, EPI_ISL_690389, EPI_ISL_690390, EPI_ISL_690391, EPI_ISL_690392, EPI_ISL_690393, EPI_ISL_690394, EPI_ISL_690395, EPI_ISL_690396, EPI_ISL_690397, EPI_ISL_690398, EPI_ISL_690399, EPI_ISL_690400, EPI_ISL_690401, EPI_ISL_690402, EPI_ISL_690403, EPI_ISL_690404, EPI_ISL_690405, EPI_ISL_690406, EPI_ISL_690407, EPI_ISL_690408, EPI_ISL_690409, EPI_ISL_690410, EPI_ISL_690411, EPI_ISL_690412, EPI_ISL_690413, EPI_ISL_690414, EPI_ISL_690415, EPI_ISL_690416, EPI_ISL_690417, EPI_ISL_690418, EPI_ISL_690419, EPI_ISL_690420, EPI_ISL_690421, EPI_ISL_690422, EPI_ISL_690423, EPI_ISL_690424, EPI_ISL_690425, EPI_ISL_690426, EPI_ISL_690427, EPI_ISL_690428, EPI_ISL_690429, EPI_ISL_690430, EPI_ISL_690431			
see above	Pathogen Genomics Center, National Institute of Infectious Diseases	Pathogen Genomics Center, National Institute of Infectious Diseases	Tsuyoshi Sekizuka, Kentaro Itokawa, Rina Tanaka, Masanori Hashino, Makoto Kuroda
EPI_ISL_690432, EPI_ISL_690433, EPI_ISL_690434, EPI_ISL_690435, EPI_ISL_690436, EPI_ISL_690437, EPI_ISL_690438, EPI_ISL_690439, EPI_ISL_690440, EPI_ISL_690441, EPI_ISL_690442, EPI_ISL_690443, EPI_ISL_690444, EPI_ISL_690445, EPI_ISL_690446, EPI_ISL_690447, EPI_ISL_690448, EPI_ISL_690449, EPI_ISL_690450, EPI_ISL_690451, EPI_ISL_690452, EPI_ISL_690453, EPI_ISL_690454, EPI_ISL_690455, EPI_ISL_690456, EPI_ISL_690457, EPI_ISL_690458, EPI_ISL_690459, EPI_ISL_690460, EPI_ISL_690461, EPI_ISL_690462, EPI_ISL_690463, EPI_ISL_690464, EPI_ISL_690465, EPI_ISL_690466, EPI_ISL_690467, EPI_ISL_690468, EPI_ISL_690469, EPI_ISL_690470, EPI_ISL_690471, EPI_ISL_690472, EPI_ISL_690473, EPI_ISL_690474, EPI_ISL_690475, EPI_ISL_690476, EPI_ISL_690477, EPI_ISL_690478, EPI_ISL_690479, EPI_ISL_690480, EPI_ISL_690481, EPI_ISL_690482, EPI_ISL_690483, EPI_ISL_690484, EPI_ISL_690485, EPI_ISL_690486, EPI_ISL_690487, EPI_ISL_690488, EPI_ISL_690489, EPI_ISL_690490, EPI_ISL_690491, EPI_ISL_690492, EPI_ISL_690493, EPI_ISL_690494, EPI_ISL_690495, EPI_ISL_690496, EPI_ISL_690497, EPI_ISL_690498, EPI_ISL_690499, EPI_ISL_690500, EPI_ISL_690501, EPI_ISL_690502, EPI_ISL_690503, EPI_ISL_690504, EPI_ISL_690505, EPI_ISL_690506, EPI_ISL_690507, EPI_ISL_690508, EPI_ISL_690509, EPI_ISL_690510, EPI_ISL_690511, EPI_ISL_690512, EPI_ISL_690513, EPI_ISL_690514, EPI_ISL_690515, EPI_ISL_690516, EPI_ISL_690517, EPI_ISL_690518, EPI_ISL_690519, EPI_ISL_690520, EPI_ISL_690521, EPI_ISL_690522, EPI_ISL_690523, EPI_ISL_690524, EPI_ISL_690525, EPI_ISL_690526			

see above	Fukuoka Institute of Health and Environmental Sciences	Pathogen Genomics Center, National Institute of Infectious Diseases	Tsuyoshi Sekizuka, Kentaro Itokawa, Rina Tanaka, Masanori Hashino, Makoto Kuroda
EPI_ISL_690527, EPI_ISL_690528, EPI_ISL_690529, EPI_ISL_690530, EPI_ISL_690531, EPI_ISL_690532, EPI_ISL_690533, EPI_ISL_690534, EPI_ISL_690535, EPI_ISL_690536, EPI_ISL_690537, EPI_ISL_690538, EPI_ISL_690539, EPI_ISL_690540, EPI_ISL_690541, EPI_ISL_690542, EPI_ISL_690543, EPI_ISL_690544, EPI_ISL_690545, EPI_ISL_690546, EPI_ISL_690547, EPI_ISL_690548, EPI_ISL_690549, EPI_ISL_690550, EPI_ISL_690551, EPI_ISL_690552, EPI_ISL_690553, EPI_ISL_690554, EPI_ISL_690555, EPI_ISL_690556, EPI_ISL_690557, EPI_ISL_690558, EPI_ISL_690559, EPI_ISL_690560, EPI_ISL_690561, EPI_ISL_690562, EPI_ISL_690563, EPI_ISL_690564, EPI_ISL_690565, EPI_ISL_690566, EPI_ISL_690567, EPI_ISL_690568, EPI_ISL_690569, EPI_ISL_690570, EPI_ISL_690571, EPI_ISL_690572, EPI_ISL_690573, EPI_ISL_690574, EPI_ISL_690575, EPI_ISL_690576, EPI_ISL_690577, EPI_ISL_690578, EPI_ISL_690579, EPI_ISL_690580, EPI_ISL_690581, EPI_ISL_690582, EPI_ISL_690583, EPI_ISL_690584, EPI_ISL_690585, EPI_ISL_690586, EPI_ISL_690587, EPI_ISL_690588, EPI_ISL_690589, EPI_ISL_690590, EPI_ISL_690591, EPI_ISL_690592, EPI_ISL_690593, EPI_ISL_690594, EPI_ISL_690595, EPI_ISL_690596, EPI_ISL_690597, EPI_ISL_690598, EPI_ISL_690599, EPI_ISL_690600, EPI_ISL_690601, EPI_ISL_690602, EPI_ISL_690603, EPI_ISL_690604, EPI_ISL_690605, EPI_ISL_690606, EPI_ISL_690607, EPI_ISL_690608, EPI_ISL_690609, EPI_ISL_690610, EPI_ISL_690611			
see above	Pathogen Genomics Center, National Institute of Infectious Diseases	Pathogen Genomics Center, National Institute of Infectious Diseases	Tsuyoshi Sekizuka, Kentaro Itokawa, Rina Tanaka, Masanori Hashino, Makoto Kuroda
EPI_ISL_690612, EPI_ISL_690613	Tokyo Metropolitan Institute of Public Health	Pathogen Genomics Center, National Institute of Infectious Diseases	Tsuyoshi Sekizuka, Kentaro Itokawa, Rina Tanaka, Masanori Hashino, Makoto Kuroda
EPI_ISL_690614, EPI_ISL_690615, EPI_ISL_690616, EPI_ISL_690617, EPI_ISL_690618	Pathogen Genomics Center, National Institute of Infectious Diseases	Pathogen Genomics Center, National Institute of Infectious Diseases	Tsuyoshi Sekizuka, Kentaro Itokawa, Rina Tanaka, Masanori Hashino, Makoto Kuroda
EPI_ISL_690619	Saitama Prefectural Institute of Public Health	Pathogen Genomics Center, National Institute of Infectious Diseases	Tsuyoshi Sekizuka, Kentaro Itokawa, Rina Tanaka, Masanori Hashino, Makoto Kuroda
EPI_ISL_690620, EPI_ISL_690621, EPI_ISL_690622, EPI_ISL_690623, EPI_ISL_690624, EPI_ISL_690625	Pathogen Genomics Center, National Institute of Infectious Diseases	Pathogen Genomics Center, National Institute of Infectious Diseases	Tsuyoshi Sekizuka, Kentaro Itokawa, Rina Tanaka, Masanori Hashino, Makoto Kuroda
EPI_ISL_690626, EPI_ISL_690627, EPI_ISL_690628	Sakai City Institute of Public Health	Pathogen Genomics Center, National Institute of Infectious Diseases	Tsuyoshi Sekizuka, Kentaro Itokawa, Rina Tanaka, Masanori Hashino, Makoto Kuroda
EPI_ISL_690629	Pathogen Genomics Center, National Institute of Infectious Diseases	Pathogen Genomics Center, National Institute of Infectious Diseases	Tsuyoshi Sekizuka, Kentaro Itokawa, Rina Tanaka, Masanori Hashino, Makoto Kuroda
EPI_ISL_690630, EPI_ISL_690631	Saitama Prefectural Institute of Public Health	Pathogen Genomics Center, National Institute of Infectious Diseases	Tsuyoshi Sekizuka, Kentaro Itokawa, Rina Tanaka, Masanori Hashino, Makoto Kuroda
EPI_ISL_690632, EPI_ISL_690633, EPI_ISL_690634, EPI_ISL_690635, EPI_ISL_690636, EPI_ISL_690637	Pathogen Genomics Center, National Institute of Infectious Diseases	Pathogen Genomics Center, National Institute of Infectious Diseases	Tsuyoshi Sekizuka, Kentaro Itokawa, Rina Tanaka, Masanori Hashino, Makoto Kuroda
EPI_ISL_690638, EPI_ISL_690639, EPI_ISL_690640	Tokyo Metropolitan Institute of Public Health	Pathogen Genomics Center, National Institute of Infectious Diseases	Tsuyoshi Sekizuka, Kentaro Itokawa, Rina Tanaka, Masanori Hashino, Makoto Kuroda
EPI_ISL_690641, EPI_ISL_690642, EPI_ISL_690643, EPI_ISL_690644, EPI_ISL_690645, EPI_ISL_690646, EPI_ISL_690647, EPI_ISL_690648, EPI_ISL_690649, EPI_ISL_690650, EPI_ISL_690651, EPI_ISL_690652, EPI_ISL_690653, EPI_ISL_690654, EPI_ISL_690655, EPI_ISL_690656, EPI_ISL_690657, EPI_ISL_690658, EPI_ISL_690659, EPI_ISL_690660, EPI_ISL_690661, EPI_ISL_690662, EPI_ISL_690663, EPI_ISL_690664, EPI_ISL_690665, EPI_ISL_690666, EPI_ISL_690667, EPI_ISL_690668, EPI_ISL_690669, EPI_ISL_690670, EPI_ISL_690671, EPI_ISL_690672, EPI_ISL_690673, EPI_ISL_690674, EPI_ISL_690675, EPI_ISL_690676, EPI_ISL_690677, EPI_ISL_690678, EPI_ISL_690679, EPI_ISL_690680, EPI_ISL_690681, EPI_ISL_690682, EPI_ISL_690683			
see above	Saitama Prefectural Institute of Public Health	Pathogen Genomics Center, National Institute of Infectious Diseases	Tsuyoshi Sekizuka, Kentaro Itokawa, Rina Tanaka, Masanori Hashino, Makoto Kuroda
EPI_ISL_690684, EPI_ISL_690685, EPI_ISL_690686, EPI_ISL_690687, EPI_ISL_690688, EPI_ISL_690689, EPI_ISL_690690, EPI_ISL_690691, EPI_ISL_690692, EPI_ISL_690693, EPI_ISL_690694, EPI_ISL_690695, EPI_ISL_690696, EPI_ISL_690697, EPI_ISL_690698, EPI_ISL_690699, EPI_ISL_690700, EPI_ISL_690701, EPI_ISL_690702, EPI_ISL_690703, EPI_ISL_690704, EPI_ISL_690705, EPI_ISL_690706, EPI_ISL_690707, EPI_ISL_690708, EPI_ISL_690709, EPI_ISL_690710, EPI_ISL_690711, EPI_ISL_690712, EPI_ISL_690713, EPI_ISL_690714, EPI_ISL_690715, EPI_ISL_690716, EPI_ISL_690717, EPI_ISL_690718, EPI_ISL_690719, EPI_ISL_690720, EPI_ISL_690721, EPI_ISL_690722, EPI_ISL_690723, EPI_ISL_690724, EPI_ISL_690725, EPI_ISL_690726, EPI_ISL_690727, EPI_ISL_690728, EPI_ISL_690729, EPI_ISL_690730, EPI_ISL_690731, EPI_ISL_690732, EPI_ISL_690733, EPI_ISL_690734, EPI_ISL_690735, EPI_ISL_690736, EPI_ISL_690737, EPI_ISL_690738, EPI_ISL_690739, EPI_ISL_690740, EPI_ISL_690741, EPI_ISL_690742, EPI_ISL_690743, EPI_ISL_690744, EPI_ISL_690745, EPI_ISL_690746, EPI_ISL_690747, EPI_ISL_690748, EPI_ISL_690749, EPI_ISL_690750, EPI_ISL_690751, EPI_ISL_690752, EPI_ISL_690753, EPI_ISL_690754, EPI_ISL_690755, EPI_ISL_690756, EPI_ISL_690757, EPI_ISL_690758, EPI_ISL_690759, EPI_ISL_690760, EPI_ISL_690761, EPI_ISL_690762, EPI_ISL_690763, EPI_ISL_690764, EPI_ISL_690765, EPI_ISL_690766, EPI_ISL_690767, EPI_ISL_690768, EPI_ISL_690769, EPI_ISL_690770, EPI_ISL_690771, EPI_ISL_690772, EPI_ISL_690773, EPI_ISL_690774, EPI_ISL_690775, EPI_ISL_690776, EPI_ISL_690777, EPI_ISL_690778, EPI_ISL_690779, EPI_ISL_690780, EPI_ISL_690781, EPI_ISL_690782, EPI_ISL_690783, EPI_ISL_690784, EPI_ISL_690785, EPI_ISL_690786, EPI_ISL_690787, EPI_ISL_690788, EPI_ISL_690789, EPI_ISL_690790, EPI_ISL_690791			
see above	Pathogen Genomics Center, National Institute of Infectious Diseases	Pathogen Genomics Center, National Institute of Infectious Diseases	Tsuyoshi Sekizuka, Kentaro Itokawa, Rina Tanaka, Masanori Hashino, Makoto Kuroda
EPI_ISL_690792, EPI_ISL_690793, EPI_ISL_690794, EPI_ISL_690795, EPI_ISL_690796, EPI_ISL_690797, EPI_ISL_690798, EPI_ISL_690799, EPI_ISL_690800, EPI_ISL_690801, EPI_ISL_690802, EPI_ISL_690803, EPI_ISL_690804, EPI_ISL_690805, EPI_ISL_690806, EPI_ISL_690807, EPI_ISL_690808, EPI_ISL_690809, EPI_ISL_690810, EPI_ISL_690811, EPI_ISL_690812, EPI_ISL_690813, EPI_ISL_690814, EPI_ISL_690815, EPI_ISL_690816, EPI_ISL_690817			
see above	Tokyo Metropolitan Institute of Public Health	Pathogen Genomics Center, National Institute of Infectious Diseases	Tsuyoshi Sekizuka, Kentaro Itokawa, Rina Tanaka, Masanori Hashino, Makoto Kuroda
EPI_ISL_690818, EPI_ISL_690819, EPI_ISL_690820, EPI_ISL_690821, EPI_ISL_690822, EPI_ISL_690823, EPI_ISL_690824, EPI_ISL_690825, EPI_ISL_690826, EPI_ISL_690827, EPI_ISL_690828, EPI_ISL_690829, EPI_ISL_690830, EPI_ISL_690831, EPI_ISL_690832, EPI_ISL_690833, EPI_ISL_690834, EPI_ISL_690835, EPI_ISL_690836			
see above	Kanagawa Prefectural Institute of Public Health	Pathogen Genomics Center, National Institute of Infectious Diseases	Tsuyoshi Sekizuka, Kentaro Itokawa, Rina Tanaka, Masanori Hashino, Makoto Kuroda
EPI_ISL_690837, EPI_ISL_690838, EPI_ISL_690839, EPI_ISL_690840, EPI_ISL_690841, EPI_ISL_690842, EPI_ISL_690843, EPI_ISL_690844	Sakai City Institute of Public Health	Pathogen Genomics Center, National Institute of Infectious Diseases	Tsuyoshi Sekizuka, Kentaro Itokawa, Rina Tanaka, Masanori Hashino, Makoto Kuroda
EPI_ISL_690845, EPI_ISL_690846, EPI_ISL_690847, EPI_ISL_690848, EPI_ISL_690849, EPI_ISL_690850, EPI_ISL_690851, EPI_ISL_690852, EPI_ISL_690853, EPI_ISL_690854, EPI_ISL_690855, EPI_ISL_690856, EPI_ISL_690857, EPI_ISL_690858, EPI_ISL_690859, EPI_ISL_690860, EPI_ISL_690861, EPI_ISL_690862, EPI_ISL_690863, EPI_ISL_690864, EPI_ISL_690865, EPI_ISL_690866, EPI_ISL_690867, EPI_ISL_690868, EPI_ISL_690869, EPI_ISL_690870, EPI_ISL_690871, EPI_ISL_690872, EPI_ISL_690873, EPI_ISL_690874, EPI_ISL_690875, EPI_ISL_690876, EPI_ISL_690877, EPI_ISL_690878, EPI_ISL_690879, EPI_ISL_690880, EPI_ISL_690881, EPI_ISL_690882, EPI_ISL_690883, EPI_ISL_690884, EPI_ISL_690885, EPI_ISL_690886, EPI_ISL_690887, EPI_ISL_690888, EPI_ISL_690889, EPI_ISL_690890, EPI_ISL_690891, EPI_ISL_690892, EPI_ISL_690893, EPI_ISL_690894, EPI_ISL_690895, EPI_ISL_690896, EPI_ISL_690897, EPI_ISL_690898, EPI_ISL_690899, EPI_ISL_690900, EPI_ISL_690901			
see above	Pathogen Genomics Center, National Institute of Infectious Diseases	Pathogen Genomics Center, National Institute of Infectious Diseases	Tsuyoshi Sekizuka, Kentaro Itokawa, Rina Tanaka, Masanori Hashino, Makoto Kuroda
EPI_ISL_690902, EPI_ISL_690903, EPI_ISL_690904, EPI_ISL_690905, EPI_ISL_690906, EPI_ISL_690907, EPI_ISL_690908	Tokyo Metropolitan Institute of Public Health	Pathogen Genomics Center, National Institute of Infectious Diseases	Tsuyoshi Sekizuka, Kentaro Itokawa, Rina Tanaka, Masanori Hashino, Makoto Kuroda
EPI_ISL_690909, EPI_ISL_690910, EPI_ISL_690911, EPI_ISL_690912, EPI_ISL_690913, EPI_ISL_690914, EPI_ISL_690915, EPI_ISL_690916, EPI_ISL_690917, EPI_ISL_690918, EPI_ISL_690919, EPI_ISL_690920, EPI_ISL_690921, EPI_ISL_690922, EPI_ISL_690923, EPI_ISL_690924, EPI_ISL_690925, EPI_ISL_690926, EPI_ISL_690927, EPI_ISL_690928, EPI_ISL_690929, EPI_ISL_690930, EPI_ISL_690931, EPI_ISL_690932, EPI_ISL_690933, EPI_ISL_690934, EPI_ISL_690935, EPI_ISL_690936, EPI_ISL_690937, EPI_ISL_690938, EPI_ISL_690939, EPI_ISL_690940, EPI_ISL_690941, EPI_ISL_690942, EPI_ISL_690943, EPI_ISL_690944, EPI_ISL_690945, EPI_ISL_690946, EPI_ISL_690947, EPI_ISL_690948, EPI_ISL_690949, EPI_ISL_690950, EPI_ISL_690951, EPI_ISL_690952, EPI_ISL_690953, EPI_ISL_690954, EPI_ISL_690955, EPI_ISL_690956, EPI_ISL_690957, EPI_ISL_690958, EPI_ISL_690959, EPI_ISL_690960, EPI_ISL_690961, EPI_ISL_690962, EPI_ISL_690963, EPI_ISL_690964, EPI_ISL_690965, EPI_ISL_690966, EPI_ISL_690967, EPI_ISL_690968, EPI_ISL_690969, EPI_ISL_690970, EPI_ISL_690971, EPI_ISL_690972, EPI_ISL_690973, EPI_ISL_690974, EPI_ISL_690975, EPI_ISL_690976, EPI_ISL_690977, EPI_ISL_690978, EPI_ISL_690979, EPI_ISL_690980, EPI_ISL_690981			
see above	Pathogen Genomics Center, National Institute of Infectious Diseases	Pathogen Genomics Center, National Institute of Infectious Diseases	Tsuyoshi Sekizuka, Kentaro Itokawa, Rina Tanaka, Masanori Hashino, Makoto Kuroda
EPI_ISL_690982, EPI_ISL_690983, EPI_ISL_690984, EPI_ISL_690985,	Fukuoka Institute of Health and Environmental Sciences	Pathogen Genomics Center, National Institute of Infectious Diseases	Tsuyoshi Sekizuka, Kentaro Itokawa, Rina Tanaka, Masanori Hashino, Makoto Kuroda

EPI_ISL_690986			
EPI_ISL_690987, EPI_ISL_690988, EPI_ISL_690989, EPI_ISL_690990, EPI_ISL_690991, EPI_ISL_690992, EPI_ISL_690993, EPI_ISL_690994, EPI_ISL_690995, EPI_ISL_690996, EPI_ISL_690997, EPI_ISL_690998, EPI_ISL_690999, EPI_ISL_691000, EPI_ISL_691001, EPI_ISL_691002, EPI_ISL_691003, EPI_ISL_691004, EPI_ISL_691005, EPI_ISL_691006, EPI_ISL_691007, EPI_ISL_691008, EPI_ISL_691009, EPI_ISL_691010, EPI_ISL_691011, EPI_ISL_691012, EPI_ISL_691013, EPI_ISL_691014, EPI_ISL_691015, EPI_ISL_691016, EPI_ISL_691017, EPI_ISL_691018, EPI_ISL_691019, EPI_ISL_691020, EPI_ISL_691021, EPI_ISL_691022, EPI_ISL_691023, EPI_ISL_691024, EPI_ISL_691025, EPI_ISL_691026, EPI_ISL_691027, EPI_ISL_691028, EPI_ISL_691029, EPI_ISL_691030, EPI_ISL_691031, EPI_ISL_691032, EPI_ISL_691033, EPI_ISL_691034, EPI_ISL_691035, EPI_ISL_691036, EPI_ISL_691037, EPI_ISL_691038, EPI_ISL_691039, EPI_ISL_691040, EPI_ISL_691041, EPI_ISL_691042, EPI_ISL_691043, EPI_ISL_691044, EPI_ISL_691045, EPI_ISL_691046, EPI_ISL_691047, EPI_ISL_691048, EPI_ISL_691049, EPI_ISL_691050, EPI_ISL_691051, EPI_ISL_691052, EPI_ISL_691053, EPI_ISL_691054, EPI_ISL_691055, EPI_ISL_691056, EPI_ISL_691057, EPI_ISL_691058, EPI_ISL_691059, EPI_ISL_691060, EPI_ISL_691061, EPI_ISL_691062, EPI_ISL_691063, EPI_ISL_691064, EPI_ISL_691065, EPI_ISL_691066, EPI_ISL_691067, EPI_ISL_691068, EPI_ISL_691069, EPI_ISL_691070, EPI_ISL_691071, EPI_ISL_691072, EPI_ISL_691073, EPI_ISL_691074, EPI_ISL_691075, EPI_ISL_691076, EPI_ISL_691077, EPI_ISL_691078, EPI_ISL_691079, EPI_ISL_691080, EPI_ISL_691081, EPI_ISL_691082, EPI_ISL_691083, EPI_ISL_691084, EPI_ISL_691085, EPI_ISL_691086, EPI_ISL_691087, EPI_ISL_691088, EPI_ISL_691089, EPI_ISL_691090, EPI_ISL_691091, EPI_ISL_691092, EPI_ISL_691093, EPI_ISL_691094, EPI_ISL_691095, EPI_ISL_691096, EPI_ISL_691097, EPI_ISL_691098, EPI_ISL_691099, EPI_ISL_691100, EPI_ISL_691101, EPI_ISL_691102, EPI_ISL_691103, EPI_ISL_691104, EPI_ISL_691105, EPI_ISL_691106, EPI_ISL_691107, EPI_ISL_691108, EPI_ISL_691109, EPI_ISL_691110, EPI_ISL_691111, EPI_ISL_691112, EPI_ISL_691113, EPI_ISL_691114, EPI_ISL_691115, EPI_ISL_691116, EPI_ISL_691117, EPI_ISL_691118, EPI_ISL_691119, EPI_ISL_691120, EPI_ISL_691121, EPI_ISL_691122, EPI_ISL_691123, EPI_ISL_691124, EPI_ISL_691125, EPI_ISL_691126, EPI_ISL_691127, EPI_ISL_691128, EPI_ISL_691129, EPI_ISL_691130, EPI_ISL_691131, EPI_ISL_691132, EPI_ISL_691133, EPI_ISL_691134, EPI_ISL_691135, EPI_ISL_691136, EPI_ISL_691137, EPI_ISL_691138, EPI_ISL_691139, EPI_ISL_691140, EPI_ISL_691141, EPI_ISL_691142, EPI_ISL_691143, EPI_ISL_691144, EPI_ISL_691145, EPI_ISL_691146, EPI_ISL_691147, EPI_ISL_691148, EPI_ISL_691149, EPI_ISL_691150, EPI_ISL_691151, EPI_ISL_691152, EPI_ISL_691153, EPI_ISL_691154, EPI_ISL_691155, EPI_ISL_691156, EPI_ISL_691157, EPI_ISL_691158, EPI_ISL_691159, EPI_ISL_691160, EPI_ISL_691161, EPI_ISL_691162, EPI_ISL_691163, EPI_ISL_691164, EPI_ISL_691165, EPI_ISL_691166, EPI_ISL_691167, EPI_ISL_691168, EPI_ISL_691169, EPI_ISL_691170, EPI_ISL_691171, EPI_ISL_691172, EPI_ISL_691173, EPI_ISL_691174, EPI_ISL_691175, EPI_ISL_691176, EPI_ISL_691177, EPI_ISL_691178, EPI_ISL_691179, EPI_ISL_691180, EPI_ISL_691181, EPI_ISL_691182, EPI_ISL_691183, EPI_ISL_691184, EPI_ISL_691185, EPI_ISL_691186, EPI_ISL_691187, EPI_ISL_691188, EPI_ISL_691189, EPI_ISL_691190, EPI_ISL_691191, EPI_ISL_691192, EPI_ISL_691193, EPI_ISL_691194, EPI_ISL_691195, EPI_ISL_691196, EPI_ISL_691197, EPI_ISL_691198, EPI_ISL_691199, EPI_ISL_691200, EPI_ISL_691201, EPI_ISL_691202, EPI_ISL_691203, EPI_ISL_691204, EPI_ISL_691205, EPI_ISL_691206, EPI_ISL_691207, EPI_ISL_691208, EPI_ISL_691209, EPI_ISL_691210, EPI_ISL_691211, EPI_ISL_691212, EPI_ISL_691213, EPI_ISL_691214, EPI_ISL_691215, EPI_ISL_691216, EPI_ISL_691217, EPI_ISL_691218, EPI_ISL_691219, EPI_ISL_691220, EPI_ISL_691221, EPI_ISL_691222, EPI_ISL_691223, EPI_ISL_691224, EPI_ISL_691225, EPI_ISL_691226, EPI_ISL_691227, EPI_ISL_691228, EPI_ISL_691229, EPI_ISL_691230, EPI_ISL_691231, EPI_ISL_691232, EPI_ISL_691233, EPI_ISL_691234, EPI_ISL_691235, EPI_ISL_691236, EPI_ISL_691237, EPI_ISL_691238, EPI_ISL_691239, EPI_ISL_691240, EPI_ISL_691241, EPI_ISL_691242, EPI_ISL_691243, EPI_ISL_691244, EPI_ISL_691245, EPI_ISL_691246, EPI_ISL_691247, EPI_ISL_691248, EPI_ISL_691249, EPI_ISL_691250, EPI_ISL_691251, EPI_ISL_691252, EPI_ISL_691253, EPI_ISL_691254, EPI_ISL_691255, EPI_ISL_691256, EPI_ISL_691257, EPI_ISL_691258, EPI_ISL_691259, EPI_ISL_691260, EPI_ISL_691261, EPI_ISL_691262, EPI_ISL_691263, EPI_ISL_691264, EPI_ISL_691265, EPI_ISL_691266, EPI_ISL_691267, EPI_ISL_691268, EPI_ISL_691269, EPI_ISL_691270, EPI_ISL_691271, EPI_ISL_691272, EPI_ISL_691273, EPI_ISL_691274, EPI_ISL_691275, EPI_ISL_691276, EPI_ISL_691277, EPI_ISL_691278, EPI_ISL_691279, EPI_ISL_691280, EPI_ISL_691281, EPI_ISL_691282, EPI_ISL_691283			
see above	Pathogen Genomics Center, National Institute of Infectious Diseases	Pathogen Genomics Center, National Institute of Infectious Diseases	Tsuyoshi Sekizuka, Kentaro Itokawa, Rina Tanaka, Masanori Hashino, Makoto Kuroda
EPI_ISL_691284, EPI_ISL_691285, EPI_ISL_691286, EPI_ISL_691287, EPI_ISL_691288	Chiba Prefectural Institute of Public Health	Pathogen Genomics Center, National Institute of Infectious Diseases	Tsuyoshi Sekizuka, Kentaro Itokawa, Rina Tanaka, Masanori Hashino, Makoto Kuroda
EPI_ISL_691289, EPI_ISL_691290, EPI_ISL_691291, EPI_ISL_691292, EPI_ISL_691293, EPI_ISL_691294, EPI_ISL_691295, EPI_ISL_691296, EPI_ISL_691297, EPI_ISL_691298, EPI_ISL_691299, EPI_ISL_691300, EPI_ISL_691301, EPI_ISL_691302, EPI_ISL_691303, EPI_ISL_691304, EPI_ISL_691305, EPI_ISL_691306, EPI_ISL_691307, EPI_ISL_691308, EPI_ISL_691309, EPI_ISL_691310, EPI_ISL_691311, EPI_ISL_691312, EPI_ISL_691313, EPI_ISL_691314, EPI_ISL_691315, EPI_ISL_691316, EPI_ISL_691317, EPI_ISL_691318, EPI_ISL_691319, EPI_ISL_691320, EPI_ISL_691321, EPI_ISL_691322, EPI_ISL_691323, EPI_ISL_691324, EPI_ISL_691325, EPI_ISL_691326, EPI_ISL_691327, EPI_ISL_691328, EPI_ISL_691329, EPI_ISL_691330, EPI_ISL_691331, EPI_ISL_691332, EPI_ISL_691333, EPI_ISL_691334, EPI_ISL_691335, EPI_ISL_691336, EPI_ISL_691337, EPI_ISL_691338, EPI_ISL_691339, EPI_ISL_691340, EPI_ISL_691341, EPI_ISL_691342, EPI_ISL_691343, EPI_ISL_691344, EPI_ISL_691345, EPI_ISL_691346, EPI_ISL_691347, EPI_ISL_691348, EPI_ISL_691349, EPI_ISL_691350, EPI_ISL_691351, EPI_ISL_691352, EPI_ISL_691353, EPI_ISL_691354, EPI_ISL_691355, EPI_ISL_691356, EPI_ISL_691357, EPI_ISL_691358, EPI_ISL_691359, EPI_ISL_691360, EPI_ISL_691361, EPI_ISL_691362, EPI_ISL_691363			
see above	Pathogen Genomics Center, National Institute of Infectious Diseases	Pathogen Genomics Center, National Institute of Infectious Diseases	Tsuyoshi Sekizuka, Kentaro Itokawa, Rina Tanaka, Masanori Hashino, Makoto Kuroda
EPI_ISL_691364, EPI_ISL_691365, EPI_ISL_691366, EPI_ISL_691367, EPI_ISL_691368, EPI_ISL_691369, EPI_ISL_691370, EPI_ISL_691371, EPI_ISL_691372, EPI_ISL_691373, EPI_ISL_691374, EPI_ISL_691375, EPI_ISL_691376, EPI_ISL_691377, EPI_ISL_691378			
see above	Saitama Prefectural Institute of Public Health	Pathogen Genomics Center, National Institute of Infectious Diseases	Tsuyoshi Sekizuka, Kentaro Itokawa, Rina Tanaka, Masanori Hashino, Makoto Kuroda
EPI_ISL_691379, EPI_ISL_691380, EPI_ISL_691381, EPI_ISL_691382, EPI_ISL_691383, EPI_ISL_691384, EPI_ISL_691385, EPI_ISL_691386, EPI_ISL_691387, EPI_ISL_691388, EPI_ISL_691389, EPI_ISL_691390, EPI_ISL_691391, EPI_ISL_691392, EPI_ISL_691393, EPI_ISL_691394, EPI_ISL_691395, EPI_ISL_691396, EPI_ISL_691397, EPI_ISL_691398, EPI_ISL_691399, EPI_ISL_691400, EPI_ISL_691401, EPI_ISL_691402, EPI_ISL_691403, EPI_ISL_691404, EPI_ISL_691405, EPI_ISL_691406, EPI_ISL_691407, EPI_ISL_691408, EPI_ISL_691409, EPI_ISL_691410, EPI_ISL_691411, EPI_ISL_691412, EPI_ISL_691413, EPI_ISL_691414, EPI_ISL_691415, EPI_ISL_691416, EPI_ISL_691417, EPI_ISL_691418, EPI_ISL_691419, EPI_ISL_691420, EPI_ISL_691421, EPI_ISL_691422, EPI_ISL_691423, EPI_ISL_691424, EPI_ISL_691425, EPI_ISL_691426, EPI_ISL_691427, EPI_ISL_691428, EPI_ISL_691429, EPI_ISL_691430, EPI_ISL_691431, EPI_ISL_691432, EPI_ISL_691433, EPI_ISL_691434, EPI_ISL_691435, EPI_ISL_691436, EPI_ISL_691437, EPI_ISL_691438, EPI_ISL_691439, EPI_ISL_691440, EPI_ISL_691441, EPI_ISL_691442, EPI_ISL_691443, EPI_ISL_691444, EPI_ISL_691445, EPI_ISL_691446, EPI_ISL_691447, EPI_ISL_691448, EPI_ISL_691449, EPI_ISL_691450, EPI_ISL_691451, EPI_ISL_691452, EPI_ISL_691453, EPI_ISL_691454, EPI_ISL_691455, EPI_ISL_691456, EPI_ISL_691457, EPI_ISL_691458, EPI_ISL_691459, EPI_ISL_691460, EPI_ISL_691461, EPI_ISL_691462, EPI_ISL_691463, EPI_ISL_691464, EPI_ISL_691465, EPI_ISL_691466, EPI_ISL_691467, EPI_ISL_691468, EPI_ISL_691469, EPI_ISL_691470, EPI_ISL_691471, EPI_ISL_691472, EPI_ISL_691473, EPI_ISL_691474, EPI_ISL_691475, EPI_ISL_691476, EPI_ISL_691477, EPI_ISL_691478, EPI_ISL_691479, EPI_ISL_691480, EPI_ISL_691481, EPI_ISL_691482, EPI_ISL_691483, EPI_ISL_691484, EPI_ISL_691485, EPI_ISL_691486, EPI_ISL_691487, EPI_ISL_691488, EPI_ISL_691489, EPI_ISL_691490, EPI_ISL_691491, EPI_ISL_691492, EPI_ISL_691493, EPI_ISL_691494, EPI_ISL_691495, EPI_ISL_691496, EPI_ISL_691497, EPI_ISL_691498, EPI_ISL_691499, EPI_ISL_691500, EPI_ISL_691501, EPI_ISL_691502, EPI_ISL_691503, EPI_ISL_691504, EPI_ISL_691505, EPI_ISL_691506, EPI_ISL_691507, EPI_ISL_691508			
see above	Pathogen Genomics Center, National Institute of Infectious Diseases	Pathogen Genomics Center, National Institute of Infectious Diseases	Tsuyoshi Sekizuka, Kentaro Itokawa, Rina Tanaka, Masanori Hashino, Makoto Kuroda
EPI_ISL_691509, EPI_ISL_691510, EPI_ISL_691511, EPI_ISL_691512, EPI_ISL_691513, EPI_ISL_691514, EPI_ISL_691515, EPI_ISL_691516, EPI_ISL_691517, EPI_ISL_691518, EPI_ISL_691519, EPI_ISL_691520, EPI_ISL_691521, EPI_ISL_691522, EPI_ISL_691523, EPI_ISL_691524, EPI_ISL_691525, EPI_ISL_691526, EPI_ISL_691527, EPI_ISL_691528, EPI_ISL_691529, EPI_ISL_691530, EPI_ISL_691531, EPI_ISL_691532, EPI_ISL_691533, EPI_ISL_691534, EPI_ISL_691535, EPI_ISL_691536, EPI_ISL_691537, EPI_ISL_691538, EPI_ISL_691539, EPI_ISL_691540, EPI_ISL_691541, EPI_ISL_691542, EPI_ISL_691543, EPI_ISL_691544, EPI_ISL_691545			
see above	Sakai City Institute of Public Health	Pathogen Genomics Center, National Institute of Infectious Diseases	Tsuyoshi Sekizuka, Kentaro Itokawa, Rina Tanaka, Masanori Hashino, Makoto Kuroda
EPI_ISL_691546, EPI_ISL_691547, EPI_ISL_691548, EPI_ISL_691549	Pathogen Genomics Center, National Institute of Infectious Diseases	Pathogen Genomics Center, National Institute of Infectious Diseases	Tsuyoshi Sekizuka, Kentaro Itokawa, Rina Tanaka, Masanori Hashino, Makoto Kuroda
EPI_ISL_691550, EPI_ISL_691551	Fukuoka Institute of Health and Environmental Sciences	Pathogen Genomics Center, National Institute of Infectious Diseases	Tsuyoshi Sekizuka, Kentaro Itokawa, Rina Tanaka, Masanori Hashino, Makoto Kuroda
EPI_ISL_691552, EPI_ISL_691553, EPI_ISL_691554, EPI_ISL_691555, EPI_ISL_691556, EPI_ISL_691557, EPI_ISL_691558, EPI_ISL_691559, EPI_ISL_691560, EPI_ISL_691561, EPI_ISL_691562, EPI_ISL_691563, EPI_ISL_691564, EPI_ISL_691565, EPI_ISL_691566, EPI_ISL_691567, EPI_ISL_691568, EPI_ISL_691569, EPI_ISL_691570, EPI_ISL_691571, EPI_ISL_691572, EPI_ISL_691573, EPI_ISL_691574, EPI_ISL_691575, EPI_ISL_691576, EPI_ISL_691577, EPI_ISL_691578, EPI_ISL_691579, EPI_ISL_691580, EPI_ISL_691581, EPI_ISL_691582, EPI_ISL_691583, EPI_ISL_691584, EPI_ISL_691585, EPI_ISL_691586, EPI_ISL_691587, EPI_ISL_691588, EPI_ISL_691589, EPI_ISL_691590, EPI_ISL_691591, EPI_ISL_691592, EPI_ISL_691593, EPI_ISL_691594, EPI_ISL_691595, EPI_ISL_691596, EPI_ISL_691597, EPI_ISL_691598, EPI_ISL_691599, EPI_ISL_691600, EPI_ISL_691601, EPI_ISL_691602, EPI_ISL_691603, EPI_ISL_691604, EPI_ISL_691605, EPI_ISL_691606, EPI_ISL_691607, EPI_ISL_691608, EPI_ISL_691609, EPI_ISL_691610, EPI_ISL_691611			
see above	Pathogen Genomics Center, National Institute of Infectious Diseases	Pathogen Genomics Center, National Institute of Infectious Diseases	Tsuyoshi Sekizuka, Kentaro Itokawa, Rina Tanaka, Masanori Hashino, Makoto Kuroda
EPI_ISL_691681	Consejeria de Sanidad y Asuntos Sociales	Instituto de Salud Carlos III	Iglesias-Caballero, M. Camarero, S. Molinero Calamita, M. González-Esguevillas, M. Pozo, F. Casas, I. Jiménez, P. Jiménez, M. Zaballos, A. Monzón, S. Varona, S. Juliá, M. Cuesta, I. Gutiérrez, G.
EPI_ISL_691682	Hospital General Universitario de Ciudad Real	Instituto de Salud Carlos III	Iglesias-Caballero, M. Camarero, S. Molinero Calamita, M. González-Esguevillas, M. Pozo, F. Casas, I. Jiménez, P. Jiménez, M. Zaballos, A. Monzón, S. Varona, S. Juliá, M. Cuesta, I. Jarilla, M.
EPI_ISL_691683	Hospital General Universitario de Ciudad Real	Instituto de Salud Carlos III	Iglesias-Caballero, M. Camarero, S. Molinero Calamita, M. González-Esguevillas, M. Pozo, F. Casas, I. Jiménez, P. Jiménez, M. Zaballos, A. Monzón, S. Varona, S. Juliá, M. Cuesta, I. Madrigal, M.
EPI_ISL_691684	Complejo Hospitalario Universitario de Albacete	Instituto de Salud Carlos III	Iglesias-Caballero, M. Camarero, S. Molinero Calamita, M. González-Esguevillas, M. Pozo, F. Casas, I. Jiménez, P. Jiménez, M. Zaballos, A. Monzón, S. Varona, S. Juliá, M. Cuesta, I. Martínez, E.
EPI_ISL_691685	Hospital de Leon	Instituto de Salud Carlos III	Iglesias-Caballero, M. Camarero, S. Molinero Calamita, M. González-Esguevillas, M. Pozo, F. Casas, I. Jiménez, P. Jiménez, M. Zaballos, A. Monzón, S. Varona, S. Juliá, M. Cuesta, I. Vidan, J.
EPI_ISL_691686, EPI_ISL_691687, EPI_ISL_691688, EPI_ISL_691689, EPI_ISL_691690, EPI_ISL_691691, EPI_ISL_691692, EPI_ISL_691693, EPI_ISL_691694, EPI_ISL_691695, EPI_ISL_691696, EPI_ISL_691697, EPI_ISL_691698, EPI_ISL_691699			
see above	Hospital Universitario de Ceuta	Instituto de Salud Carlos III	Iglesias-Caballero, M. Camarero, S. Molinero Calamita, M. González-Esguevillas, M. Pozo, F. Casas, I. Jiménez, P. Jiménez, M. Zaballos, A. Monzón, S. Varona, S. Juliá, M. Cuesta, I. Hijano, S.
EPI_ISL_691700, EPI_ISL_691701, EPI_ISL_691702, EPI_ISL_691703, EPI_ISL_691704, EPI_ISL_691705, EPI_ISL_691706, EPI_ISL_691707, EPI_ISL_691708, EPI_ISL_691709, EPI_ISL_691710, EPI_ISL_691711, EPI_ISL_691712, EPI_ISL_691713, EPI_ISL_691714, EPI_ISL_691715, EPI_ISL_691716, EPI_ISL_691717, EPI_ISL_691718, EPI_ISL_691719, EPI_ISL_691720, EPI_ISL_691721, EPI_ISL_691722, EPI_ISL_691723, EPI_ISL_691724, EPI_ISL_691725, EPI_ISL_691726, EPI_ISL_691727, EPI_ISL_691728			
see above	Hospital Clínico San Carlos	Instituto de Salud Carlos III	Iglesias-Caballero, M. Camarero, S. Molinero Calamita, M. González-Esguevillas, M. Pozo, F. Casas, I. Jiménez, P. Jiménez, M. Zaballos, A. Monzón, S. Varona, S. Juliá, M. Cuesta, I. Rodríguez, I.

EPI_ISL_691729, EPI_ISL_691730	Hospital Universitario Severo Ochoa	Instituto de Salud Carlos III	Iglesias-Caballero, M. Camarero, S. Molinero Calamita, M. González-Esguevillas, M. Pozo, F. Casas, I. Jiménez, P. Jiménez, M. Zaballos, A. Monzón, S. Varona, S. Juliá, M. Cuesta, I. García, M.
EPI_ISL_691731	Hospital Universitario Puerta de Hierro	Instituto de Salud Carlos III	Iglesias-Caballero, M. Camarero, S. Molinero Calamita, M. González-Esguevillas, M. Pozo, F. Casas, I. Jiménez, P. Jiménez, M. Zaballos, A. Monzón, S. Varona, S. Juliá, M. Cuesta, I. Velasco, A.
EPI_ISL_691732	Hospital de Madrid	Instituto de Salud Carlos III	Iglesias-Caballero, M. Camarero, S. Molinero Calamita, M. González-Esguevillas, M. Pozo, F. Casas, I. Jiménez, P. Jiménez, M. Zaballos, A. Monzón, S. Varona, S. Juliá, M. Cuesta, I. Alhambra, A.
EPI_ISL_691733, EPI_ISL_691734	Pathogen Genomics Center, National Institute of Infectious Diseases	Pathogen Genomics Center, National Institute of Infectious Diseases	Tsuyoshi Sekizuka, Kentaro Itokawa, Rina Tanaka, Masanori Hashino, Makoto Kuroda
EPI_ISL_691735	Fukuoka Institute of Health and Environmental Sciences	Pathogen Genomics Center, National Institute of Infectious Diseases	Tsuyoshi Sekizuka, Kentaro Itokawa, Rina Tanaka, Masanori Hashino, Makoto Kuroda
EPI_ISL_691736, EPI_ISL_691737, EPI_ISL_691738, EPI_ISL_691739, EPI_ISL_691740, EPI_ISL_691741, EPI_ISL_691742, EPI_ISL_691743, EPI_ISL_691744, EPI_ISL_691745, EPI_ISL_691746, EPI_ISL_691747, EPI_ISL_691748, EPI_ISL_691749, EPI_ISL_691750, EPI_ISL_691751, EPI_ISL_691752			
see above	Pathogen Genomics Center, National Institute of Infectious Diseases	Pathogen Genomics Center, National Institute of Infectious Diseases	Tsuyoshi Sekizuka, Kentaro Itokawa, Rina Tanaka, Masanori Hashino, Makoto Kuroda
EPI_ISL_691753	Sakai City Institute of Public Health	Pathogen Genomics Center, National Institute of Infectious Diseases	Tsuyoshi Sekizuka, Kentaro Itokawa, Rina Tanaka, Masanori Hashino, Makoto Kuroda
EPI_ISL_691754, EPI_ISL_691755	Pathogen Genomics Center, National Institute of Infectious Diseases	Pathogen Genomics Center, National Institute of Infectious Diseases	Tsuyoshi Sekizuka, Kentaro Itokawa, Rina Tanaka, Masanori Hashino, Makoto Kuroda
EPI_ISL_691756	Fukuoka Institute of Health and Environmental Sciences	Pathogen Genomics Center, National Institute of Infectious Diseases	Tsuyoshi Sekizuka, Kentaro Itokawa, Rina Tanaka, Masanori Hashino, Makoto Kuroda
EPI_ISL_691757, EPI_ISL_691758, EPI_ISL_691759, EPI_ISL_691760, EPI_ISL_691761, EPI_ISL_691762, EPI_ISL_691763	Pathogen Genomics Center, National Institute of Infectious Diseases	Pathogen Genomics Center, National Institute of Infectious Diseases	Tsuyoshi Sekizuka, Kentaro Itokawa, Rina Tanaka, Masanori Hashino, Makoto Kuroda
EPI_ISL_691764, EPI_ISL_691765	Fukuoka Institute of Health and Environmental Sciences	Pathogen Genomics Center, National Institute of Infectious Diseases	Tsuyoshi Sekizuka, Kentaro Itokawa, Rina Tanaka, Masanori Hashino, Makoto Kuroda
EPI_ISL_691766	Saitama Prefectural Institute of Public Health	Pathogen Genomics Center, National Institute of Infectious Diseases	Tsuyoshi Sekizuka, Kentaro Itokawa, Rina Tanaka, Masanori Hashino, Makoto Kuroda
EPI_ISL_691767, EPI_ISL_691768, EPI_ISL_691769, EPI_ISL_691770, EPI_ISL_691771, EPI_ISL_691772, EPI_ISL_691773, EPI_ISL_691774, EPI_ISL_691775, EPI_ISL_691776, EPI_ISL_691777, EPI_ISL_691778, EPI_ISL_691779, EPI_ISL_691780, EPI_ISL_691781, EPI_ISL_691782, EPI_ISL_691783, EPI_ISL_691784, EPI_ISL_691785, EPI_ISL_691786, EPI_ISL_691787, EPI_ISL_691788, EPI_ISL_691789, EPI_ISL_691790, EPI_ISL_691791, EPI_ISL_691792, EPI_ISL_691793, EPI_ISL_691794, EPI_ISL_691795, EPI_ISL_691796, EPI_ISL_691797, EPI_ISL_691798, EPI_ISL_691799, EPI_ISL_691800, EPI_ISL_691801, EPI_ISL_691802, EPI_ISL_691803, EPI_ISL_691804, EPI_ISL_691805, EPI_ISL_691806, EPI_ISL_691807, EPI_ISL_691808, EPI_ISL_691809, EPI_ISL_691810, EPI_ISL_691811, EPI_ISL_691812, EPI_ISL_691813, EPI_ISL_691814, EPI_ISL_691815, EPI_ISL_691816, EPI_ISL_691817, EPI_ISL_691818, EPI_ISL_691819, EPI_ISL_691820, EPI_ISL_691821, EPI_ISL_691822, EPI_ISL_691823, EPI_ISL_691824, EPI_ISL_691825, EPI_ISL_691826, EPI_ISL_691827, EPI_ISL_691828, EPI_ISL_691829, EPI_ISL_691830, EPI_ISL_691831, EPI_ISL_691832, EPI_ISL_691833, EPI_ISL_691834, EPI_ISL_691835, EPI_ISL_691836, EPI_ISL_691837, EPI_ISL_691838, EPI_ISL_691839, EPI_ISL_691840, EPI_ISL_691841, EPI_ISL_691842, EPI_ISL_691843, EPI_ISL_691844, EPI_ISL_691845, EPI_ISL_691846, EPI_ISL_691847, EPI_ISL_691848, EPI_ISL_691849, EPI_ISL_691850, EPI_ISL_691851, EPI_ISL_691852, EPI_ISL_691853, EPI_ISL_691854, EPI_ISL_691855, EPI_ISL_691856, EPI_ISL_691857, EPI_ISL_691858, EPI_ISL_691859, EPI_ISL_691860, EPI_ISL_691861, EPI_ISL_691862, EPI_ISL_691863, EPI_ISL_691864, EPI_ISL_691865, EPI_ISL_691866, EPI_ISL_691867, EPI_ISL_691868, EPI_ISL_691869, EPI_ISL_691870, EPI_ISL_691871, EPI_ISL_691872, EPI_ISL_691873, EPI_ISL_691874, EPI_ISL_691875, EPI_ISL_691876, EPI_ISL_691877, EPI_ISL_691878, EPI_ISL_691879, EPI_ISL_691880, EPI_ISL_691881, EPI_ISL_691882, EPI_ISL_691883, EPI_ISL_691884, EPI_ISL_691885, EPI_ISL_691886, EPI_ISL_691887, EPI_ISL_691888, EPI_ISL_691889, EPI_ISL_691890, EPI_ISL_691891, EPI_ISL_691892, EPI_ISL_691893, EPI_ISL_691894, EPI_ISL_691895, EPI_ISL_691896, EPI_ISL_691897, EPI_ISL_691898, EPI_ISL_691899, EPI_ISL_691900, EPI_ISL_691901, EPI_ISL_691902, EPI_ISL_691903, EPI_ISL_691904, EPI_ISL_691905, EPI_ISL_691906, EPI_ISL_691907, EPI_ISL_691908, EPI_ISL_691909, EPI_ISL_691910, EPI_ISL_691911, EPI_ISL_691912, EPI_ISL_691913, EPI_ISL_691914, EPI_ISL_691915, EPI_ISL_691916, EPI_ISL_691917, EPI_ISL_691918, EPI_ISL_691919, EPI_ISL_691920, EPI_ISL_691921, EPI_ISL_691922, EPI_ISL_691923, EPI_ISL_691924, EPI_ISL_691925, EPI_ISL_691926, EPI_ISL_691927, EPI_ISL_691928, EPI_ISL_691929, EPI_ISL_691930, EPI_ISL_691931, EPI_ISL_691932, EPI_ISL_691933, EPI_ISL_691934, EPI_ISL_691935, EPI_ISL_691936, EPI_ISL_691937, EPI_ISL_691938, EPI_ISL_691939, EPI_ISL_691940, EPI_ISL_691941, EPI_ISL_691942, EPI_ISL_691943, EPI_ISL_691944, EPI_ISL_691945, EPI_ISL_691946, EPI_ISL_691947, EPI_ISL_691948, EPI_ISL_691949, EPI_ISL_691950, EPI_ISL_691951, EPI_ISL_691952, EPI_ISL_691953, EPI_ISL_691954, EPI_ISL_691955, EPI_ISL_691956, EPI_ISL_691957, EPI_ISL_691958, EPI_ISL_691959, EPI_ISL_691960, EPI_ISL_691961, EPI_ISL_691962, EPI_ISL_691963, EPI_ISL_691964, EPI_ISL_691965, EPI_ISL_691966, EPI_ISL_691967, EPI_ISL_691968, EPI_ISL_691969, EPI_ISL_691970, EPI_ISL_691971, EPI_ISL_691972, EPI_ISL_691973, EPI_ISL_691974, EPI_ISL_691975, EPI_ISL_691976, EPI_ISL_691977, EPI_ISL_691978, EPI_ISL_691979, EPI_ISL_691980, EPI_ISL_691981, EPI_ISL_691982, EPI_ISL_691983, EPI_ISL_691984, EPI_ISL_691985, EPI_ISL_691986, EPI_ISL_691987, EPI_ISL_691988, EPI_ISL_691989, EPI_ISL_691990, EPI_ISL_691991, EPI_ISL_691992, EPI_ISL_691993, EPI_ISL_691994, EPI_ISL_691995, EPI_ISL_691996, EPI_ISL_691997, EPI_ISL_691998, EPI_ISL_691999, EPI_ISL_692000, EPI_ISL_692001, EPI_ISL_692002, EPI_ISL_692003, EPI_ISL_692004, EPI_ISL_692005, EPI_ISL_692006, EPI_ISL_692007, EPI_ISL_692008, EPI_ISL_692009, EPI_ISL_692010, EPI_ISL_692011, EPI_ISL_692012, EPI_ISL_692013, EPI_ISL_692014, EPI_ISL_692015, EPI_ISL_692016, EPI_ISL_692017, EPI_ISL_692018, EPI_ISL_692019, EPI_ISL_692020, EPI_ISL_692021, EPI_ISL_692022, EPI_ISL_692023, EPI_ISL_692024, EPI_ISL_692025, EPI_ISL_692026, EPI_ISL_692027, EPI_ISL_692028, EPI_ISL_692029, EPI_ISL_692030, EPI_ISL_692031, EPI_ISL_692032, EPI_ISL_692033, EPI_ISL_692034, EPI_ISL_692035, EPI_ISL_692036, EPI_ISL_692037, EPI_ISL_692038, EPI_ISL_692039, EPI_ISL_692040, EPI_ISL_692041, EPI_ISL_692042, EPI_ISL_692043, EPI_ISL_692044, EPI_ISL_692045, EPI_ISL_692046			
see above	Pathogen Genomics Center, National Institute of Infectious Diseases	Pathogen Genomics Center, National Institute of Infectious Diseases	Tsuyoshi Sekizuka, Kentaro Itokawa, Rina Tanaka, Masanori Hashino, Makoto Kuroda
EPI_ISL_692047	Gunma Prefectural Institute of Public Health and Environmental Sciences	Pathogen Genomics Center, National Institute of Infectious Diseases	Tsuyoshi Sekizuka, Kentaro Itokawa, Rina Tanaka, Masanori Hashino, Makoto Kuroda
EPI_ISL_692048, EPI_ISL_692049, EPI_ISL_692050	Fukuoka Institute of Health and Environmental Sciences	Pathogen Genomics Center, National Institute of Infectious Diseases	Tsuyoshi Sekizuka, Kentaro Itokawa, Rina Tanaka, Masanori Hashino, Makoto Kuroda
EPI_ISL_692051, EPI_ISL_692052	Niigata Prefectural Institute of Public Health and Environmental Sciences	Pathogen Genomics Center, National Institute of Infectious Diseases	Tsuyoshi Sekizuka, Kentaro Itokawa, Rina Tanaka, Masanori Hashino, Makoto Kuroda
EPI_ISL_692053, EPI_ISL_692054, EPI_ISL_692055, EPI_ISL_692056, EPI_ISL_692057, EPI_ISL_692058	Sakai City Institute of Public Health	Pathogen Genomics Center, National Institute of Infectious Diseases	Tsuyoshi Sekizuka, Kentaro Itokawa, Rina Tanaka, Masanori Hashino, Makoto Kuroda
EPI_ISL_692059, EPI_ISL_692060, EPI_ISL_692061, EPI_ISL_692062, EPI_ISL_692063, EPI_ISL_692064, EPI_ISL_692065, EPI_ISL_692066, EPI_ISL_692067, EPI_ISL_692068, EPI_ISL_692069, EPI_ISL_692070, EPI_ISL_692071, EPI_ISL_692072, EPI_ISL_692073, EPI_ISL_692074, EPI_ISL_692075, EPI_ISL_692076, EPI_ISL_692077, EPI_ISL_692078, EPI_ISL_692079, EPI_ISL_692080, EPI_ISL_692081, EPI_ISL_692082, EPI_ISL_692083, EPI_ISL_692084, EPI_ISL_692085, EPI_ISL_692086, EPI_ISL_692087, EPI_ISL_692088, EPI_ISL_692089, EPI_ISL_692090, EPI_ISL_692091, EPI_ISL_692092, EPI_ISL_692093			
see above	Pathogen Genomics Center, National Institute of Infectious Diseases	Pathogen Genomics Center, National Institute of Infectious Diseases	Tsuyoshi Sekizuka, Kentaro Itokawa, Rina Tanaka, Masanori Hashino, Makoto Kuroda
EPI_ISL_692094, EPI_ISL_692095, EPI_ISL_692096, EPI_ISL_692097	Yamagata Prefectural Institute of Public Health	Pathogen Genomics Center, National Institute of Infectious Diseases	Tsuyoshi Sekizuka, Kentaro Itokawa, Rina Tanaka, Masanori Hashino, Makoto Kuroda
EPI_ISL_692098, EPI_ISL_692099, EPI_ISL_692100, EPI_ISL_692101, EPI_ISL_692102, EPI_ISL_692103, EPI_ISL_692104, EPI_ISL_692105, EPI_ISL_692106, EPI_ISL_692107, EPI_ISL_692108, EPI_ISL_692109, EPI_ISL_692110, EPI_ISL_692111, EPI_ISL_692112, EPI_ISL_692113, EPI_ISL_692114, EPI_ISL_692115, EPI_ISL_692116, EPI_ISL_692117, EPI_ISL_692118			
see above	Pathogen Genomics Center, National Institute of Infectious Diseases	Pathogen Genomics Center, National Institute of Infectious Diseases	Tsuyoshi Sekizuka, Kentaro Itokawa, Rina Tanaka, Masanori Hashino, Makoto Kuroda
EPI_ISL_692119, EPI_ISL_692120, EPI_ISL_692121, EPI_ISL_692122, EPI_ISL_692123, EPI_ISL_692124, EPI_ISL_692125, EPI_ISL_692126, EPI_ISL_692127, EPI_ISL_692128	Tokyo Metropolitan Institute of Public Health	Pathogen Genomics Center, National Institute of Infectious Diseases	Tsuyoshi Sekizuka, Kentaro Itokawa, Rina Tanaka, Masanori Hashino, Makoto Kuroda
EPI_ISL_692129, EPI_ISL_692130, EPI_ISL_692131, EPI_ISL_692132, EPI_ISL_692133, EPI_ISL_692134, EPI_ISL_692135, EPI_ISL_692136, EPI_ISL_692137, EPI_ISL_692138, EPI_ISL_692139, EPI_ISL_692140, EPI_ISL_692141, EPI_ISL_692142, EPI_ISL_692143, EPI_ISL_692144, EPI_ISL_692145, EPI_ISL_692146, EPI_ISL_692147, EPI_ISL_692148, EPI_ISL_692149, EPI_ISL_692150, EPI_ISL_692151, EPI_ISL_692152, EPI_ISL_692153, EPI_ISL_692154, EPI_ISL_692155, EPI_ISL_692156, EPI_ISL_692157, EPI_ISL_692158, EPI_ISL_692159, EPI_ISL_692160, EPI_ISL_692161			
see above	Pathogen Genomics Center, National Institute of Infectious Diseases	Pathogen Genomics Center, National Institute of Infectious Diseases	Tsuyoshi Sekizuka, Kentaro Itokawa, Rina Tanaka, Masanori Hashino, Makoto Kuroda
EPI_ISL_692162, EPI_ISL_692163	Tokyo Metropolitan Institute of Public Health	Pathogen Genomics Center, National Institute of Infectious	Tsuyoshi Sekizuka, Kentaro Itokawa, Rina Tanaka, Masanori Hashino, Makoto Kuroda

		Diseases	
EPI_ISL_692164, EPI_ISL_692165, EPI_ISL_692166, EPI_ISL_692167, EPI_ISL_692168, EPI_ISL_692169, EPI_ISL_692170, EPI_ISL_692171, EPI_ISL_692172, EPI_ISL_692173, EPI_ISL_692174, EPI_ISL_692175, EPI_ISL_692176, EPI_ISL_692177, EPI_ISL_692178, EPI_ISL_692179, EPI_ISL_692180, EPI_ISL_692181, EPI_ISL_692182, EPI_ISL_692183, EPI_ISL_692184, EPI_ISL_692185, EPI_ISL_692186, EPI_ISL_692187, EPI_ISL_692188, EPI_ISL_692189, EPI_ISL_692190, EPI_ISL_692191, EPI_ISL_692192, EPI_ISL_692193, EPI_ISL_692194, EPI_ISL_692195, EPI_ISL_692196, EPI_ISL_692197, EPI_ISL_692198, EPI_ISL_692199, EPI_ISL_692200, EPI_ISL_692201, EPI_ISL_692202, EPI_ISL_692203, EPI_ISL_692204, EPI_ISL_692205, EPI_ISL_692206, EPI_ISL_692207, EPI_ISL_692208, EPI_ISL_692209, EPI_ISL_692210, EPI_ISL_692211, EPI_ISL_692212, EPI_ISL_692213, EPI_ISL_692214, EPI_ISL_692215, EPI_ISL_692216, EPI_ISL_692217, EPI_ISL_692218			
see above	Pathogen Genomics Center, National Institute of Infectious Diseases	Pathogen Genomics Center, National Institute of Infectious Diseases	Tsuyoshi Sekizuka, Kentaro Itokawa, Rina Tanaka, Masanori Hashino, Makoto Kuroda
EPI_ISL_692219, EPI_ISL_692220, EPI_ISL_692221, EPI_ISL_692222, EPI_ISL_692223, EPI_ISL_692224, EPI_ISL_692225, EPI_ISL_692226, EPI_ISL_692227, EPI_ISL_692228, EPI_ISL_692229			
see above	Fukuoka Institute of Health and Environmental Sciences	Pathogen Genomics Center, National Institute of Infectious Diseases	Tsuyoshi Sekizuka, Kentaro Itokawa, Rina Tanaka, Masanori Hashino, Makoto Kuroda
EPI_ISL_692230, EPI_ISL_692231, EPI_ISL_692232, EPI_ISL_692233, EPI_ISL_692234, EPI_ISL_692235, EPI_ISL_692236, EPI_ISL_692237, EPI_ISL_692238, EPI_ISL_692239, EPI_ISL_692240, EPI_ISL_692241, EPI_ISL_692242, EPI_ISL_692243, EPI_ISL_692244, EPI_ISL_692245, EPI_ISL_692246, EPI_ISL_692247, EPI_ISL_692248, EPI_ISL_692249, EPI_ISL_692250, EPI_ISL_692251, EPI_ISL_692252, EPI_ISL_692253, EPI_ISL_692254, EPI_ISL_692255, EPI_ISL_692256, EPI_ISL_692257, EPI_ISL_692258, EPI_ISL_692259, EPI_ISL_692260, EPI_ISL_692261, EPI_ISL_692262, EPI_ISL_692263, EPI_ISL_692264, EPI_ISL_692265, EPI_ISL_692266, EPI_ISL_692267, EPI_ISL_692268, EPI_ISL_692269, EPI_ISL_692270, EPI_ISL_692271, EPI_ISL_692272, EPI_ISL_692273, EPI_ISL_692274, EPI_ISL_692275, EPI_ISL_692276, EPI_ISL_692277, EPI_ISL_692278, EPI_ISL_692279, EPI_ISL_692280, EPI_ISL_692281, EPI_ISL_692282, EPI_ISL_692283			
see above	Pathogen Genomics Center, National Institute of Infectious Diseases	Pathogen Genomics Center, National Institute of Infectious Diseases	Tsuyoshi Sekizuka, Kentaro Itokawa, Rina Tanaka, Masanori Hashino, Makoto Kuroda
EPI_ISL_692284, EPI_ISL_692285, EPI_ISL_692286, EPI_ISL_692287, EPI_ISL_692288, EPI_ISL_692289, EPI_ISL_692290, EPI_ISL_692291, EPI_ISL_692292, EPI_ISL_692293, EPI_ISL_692294, EPI_ISL_692295, EPI_ISL_692296, EPI_ISL_692297, EPI_ISL_692298, EPI_ISL_692299, EPI_ISL_692300, EPI_ISL_692301, EPI_ISL_692302, EPI_ISL_692303, EPI_ISL_692304, EPI_ISL_692305, EPI_ISL_692306, EPI_ISL_692307, EPI_ISL_692308, EPI_ISL_692309, EPI_ISL_692310, EPI_ISL_692311, EPI_ISL_692312, EPI_ISL_692313, EPI_ISL_692314, EPI_ISL_692315			
see above	Sakai City Institute of Public Health	Pathogen Genomics Center, National Institute of Infectious Diseases	Tsuyoshi Sekizuka, Kentaro Itokawa, Rina Tanaka, Masanori Hashino, Makoto Kuroda
EPI_ISL_692316, EPI_ISL_692317, EPI_ISL_692318, EPI_ISL_692319, EPI_ISL_692320, EPI_ISL_692321, EPI_ISL_692322, EPI_ISL_692323, EPI_ISL_692324, EPI_ISL_692325, EPI_ISL_692326, EPI_ISL_692327			
see above	Gunma Prefectural Institute of Public Health and Environmental Sciences	Pathogen Genomics Center, National Institute of Infectious Diseases	Tsuyoshi Sekizuka, Kentaro Itokawa, Rina Tanaka, Masanori Hashino, Makoto Kuroda
EPI_ISL_692328, EPI_ISL_692329, EPI_ISL_692330, EPI_ISL_692331, EPI_ISL_692332, EPI_ISL_692333, EPI_ISL_692334, EPI_ISL_692335, EPI_ISL_692336, EPI_ISL_692337, EPI_ISL_692338, EPI_ISL_692339, EPI_ISL_692340, EPI_ISL_692341, EPI_ISL_692342, EPI_ISL_692343, EPI_ISL_692344, EPI_ISL_692345, EPI_ISL_692346, EPI_ISL_692347, EPI_ISL_692348, EPI_ISL_692349, EPI_ISL_692350, EPI_ISL_692351, EPI_ISL_692352, EPI_ISL_692353, EPI_ISL_692354, EPI_ISL_692355, EPI_ISL_692356, EPI_ISL_692357, EPI_ISL_692358, EPI_ISL_692359, EPI_ISL_692360, EPI_ISL_692361, EPI_ISL_692362, EPI_ISL_692363, EPI_ISL_692364, EPI_ISL_692365, EPI_ISL_692366, EPI_ISL_692367, EPI_ISL_692368, EPI_ISL_692369, EPI_ISL_692370, EPI_ISL_692371, EPI_ISL_692372, EPI_ISL_692373, EPI_ISL_692374, EPI_ISL_692375, EPI_ISL_692376, EPI_ISL_692377, EPI_ISL_692378, EPI_ISL_692379, EPI_ISL_692380, EPI_ISL_692381, EPI_ISL_692382, EPI_ISL_692383, EPI_ISL_692384, EPI_ISL_692385, EPI_ISL_692386, EPI_ISL_692387, EPI_ISL_692388, EPI_ISL_692389, EPI_ISL_692390, EPI_ISL_692391, EPI_ISL_692392, EPI_ISL_692393, EPI_ISL_692394, EPI_ISL_692395, EPI_ISL_692396, EPI_ISL_692397, EPI_ISL_692398, EPI_ISL_692399, EPI_ISL_692400, EPI_ISL_692401, EPI_ISL_692402, EPI_ISL_692403, EPI_ISL_692404, EPI_ISL_692405, EPI_ISL_692406, EPI_ISL_692407, EPI_ISL_692408, EPI_ISL_692409, EPI_ISL_692410, EPI_ISL_692411, EPI_ISL_692412, EPI_ISL_692413, EPI_ISL_692414, EPI_ISL_692415, EPI_ISL_692416, EPI_ISL_692417, EPI_ISL_692418, EPI_ISL_692419, EPI_ISL_692420, EPI_ISL_692421, EPI_ISL_692422, EPI_ISL_692423, EPI_ISL_692424, EPI_ISL_692425, EPI_ISL_692426, EPI_ISL_692427, EPI_ISL_692428, EPI_ISL_692429, EPI_ISL_692430, EPI_ISL_692431, EPI_ISL_692432, EPI_ISL_692433, EPI_ISL_692434, EPI_ISL_692435, EPI_ISL_692436, EPI_ISL_692437, EPI_ISL_692438, EPI_ISL_692439, EPI_ISL_692440, EPI_ISL_692441			
see above	Pathogen Genomics Center, National Institute of Infectious Diseases	Pathogen Genomics Center, National Institute of Infectious Diseases	Tsuyoshi Sekizuka, Kentaro Itokawa, Rina Tanaka, Masanori Hashino, Makoto Kuroda
EPI_ISL_692442, EPI_ISL_692443, EPI_ISL_692444, EPI_ISL_692445, EPI_ISL_692446, EPI_ISL_692447, EPI_ISL_692448, EPI_ISL_692449, EPI_ISL_692450, EPI_ISL_692451, EPI_ISL_692452, EPI_ISL_692453, EPI_ISL_692454, EPI_ISL_692455, EPI_ISL_692456, EPI_ISL_692457, EPI_ISL_692458, EPI_ISL_692459, EPI_ISL_692460, EPI_ISL_692461, EPI_ISL_692462, EPI_ISL_692463, EPI_ISL_692464, EPI_ISL_692465, EPI_ISL_692466, EPI_ISL_692467, EPI_ISL_692468, EPI_ISL_692469, EPI_ISL_692470, EPI_ISL_692471, EPI_ISL_692472, EPI_ISL_692473, EPI_ISL_692474, EPI_ISL_692475, EPI_ISL_692476, EPI_ISL_692477, EPI_ISL_692478, EPI_ISL_692479, EPI_ISL_692480, EPI_ISL_692481, EPI_ISL_692482, EPI_ISL_692483, EPI_ISL_692484, EPI_ISL_692485, EPI_ISL_692486, EPI_ISL_692487, EPI_ISL_692488, EPI_ISL_692489, EPI_ISL_692490, EPI_ISL_692491, EPI_ISL_692492, EPI_ISL_692493, EPI_ISL_692494, EPI_ISL_692495, EPI_ISL_692496, EPI_ISL_692497			
see above	Fukuoka Institute of Health and Environmental Sciences	Pathogen Genomics Center, National Institute of Infectious Diseases	Tsuyoshi Sekizuka, Kentaro Itokawa, Rina Tanaka, Masanori Hashino, Makoto Kuroda
EPI_ISL_692498, EPI_ISL_692499, EPI_ISL_692500, EPI_ISL_692501, EPI_ISL_692502, EPI_ISL_692503, EPI_ISL_692504, EPI_ISL_692505, EPI_ISL_692506, EPI_ISL_692507, EPI_ISL_692508, EPI_ISL_692509, EPI_ISL_692510, EPI_ISL_692511, EPI_ISL_692512, EPI_ISL_692513, EPI_ISL_692514, EPI_ISL_692515, EPI_ISL_692516, EPI_ISL_692517, EPI_ISL_692518, EPI_ISL_692519, EPI_ISL_692520, EPI_ISL_692521, EPI_ISL_692522, EPI_ISL_692523, EPI_ISL_692524, EPI_ISL_692525, EPI_ISL_692526, EPI_ISL_692527, EPI_ISL_692528, EPI_ISL_692529, EPI_ISL_692530, EPI_ISL_692531, EPI_ISL_692532, EPI_ISL_692533, EPI_ISL_692534, EPI_ISL_692535, EPI_ISL_692536, EPI_ISL_692537, EPI_ISL_692538, EPI_ISL_692539, EPI_ISL_692540, EPI_ISL_692541, EPI_ISL_692542, EPI_ISL_692543, EPI_ISL_692544, EPI_ISL_692545, EPI_ISL_692546, EPI_ISL_692547, EPI_ISL_692548, EPI_ISL_692549, EPI_ISL_692550, EPI_ISL_692551, EPI_ISL_692552, EPI_ISL_692553, EPI_ISL_692554, EPI_ISL_692555, EPI_ISL_692556, EPI_ISL_692557, EPI_ISL_692558, EPI_ISL_692559, EPI_ISL_692560, EPI_ISL_692561, EPI_ISL_692562, EPI_ISL_692563, EPI_ISL_692564, EPI_ISL_692565, EPI_ISL_692566, EPI_ISL_692567, EPI_ISL_692568, EPI_ISL_692569, EPI_ISL_692570, EPI_ISL_692571, EPI_ISL_692572, EPI_ISL_692573, EPI_ISL_692574, EPI_ISL_692575, EPI_ISL_692576, EPI_ISL_692577, EPI_ISL_692578, EPI_ISL_692579, EPI_ISL_692580, EPI_ISL_692581, EPI_ISL_692582, EPI_ISL_692583, EPI_ISL_692584, EPI_ISL_692585, EPI_ISL_692586, EPI_ISL_692587, EPI_ISL_692588, EPI_ISL_692589, EPI_ISL_692590, EPI_ISL_692591, EPI_ISL_692592, EPI_ISL_692593, EPI_ISL_692594, EPI_ISL_692595, EPI_ISL_692596, EPI_ISL_692597, EPI_ISL_692598, EPI_ISL_692599, EPI_ISL_692600, EPI_ISL_692601, EPI_ISL_692602, EPI_ISL_692603, EPI_ISL_692604, EPI_ISL_692605, EPI_ISL_692606, EPI_ISL_692607, EPI_ISL_692608, EPI_ISL_692609, EPI_ISL_692610, EPI_ISL_692611, EPI_ISL_692612, EPI_ISL_692613, EPI_ISL_692614, EPI_ISL_692615, EPI_ISL_692616, EPI_ISL_692617, EPI_ISL_692618, EPI_ISL_692619, EPI_ISL_692620, EPI_ISL_692621, EPI_ISL_692622, EPI_ISL_692623, EPI_ISL_692624, EPI_ISL_692625, EPI_ISL_692626, EPI_ISL_692627, EPI_ISL_692628, EPI_ISL_692629, EPI_ISL_692630, EPI_ISL_692631, EPI_ISL_692632, EPI_ISL_692633, EPI_ISL_692634, EPI_ISL_692635, EPI_ISL_692636, EPI_ISL_692637, EPI_ISL_692638, EPI_ISL_692639, EPI_ISL_692640, EPI_ISL_692641, EPI_ISL_692642, EPI_ISL_692643, EPI_ISL_692644, EPI_ISL_692645, EPI_ISL_692646, EPI_ISL_692647, EPI_ISL_692648, EPI_ISL_692649, EPI_ISL_692650, EPI_ISL_692651, EPI_ISL_692652			
see above	Pathogen Genomics Center, National Institute of Infectious Diseases	Pathogen Genomics Center, National Institute of Infectious Diseases	Tsuyoshi Sekizuka, Kentaro Itokawa, Rina Tanaka, Masanori Hashino, Makoto Kuroda
EPI_ISL_692653, EPI_ISL_692654, EPI_ISL_692655, EPI_ISL_692656, EPI_ISL_692657, EPI_ISL_692658, EPI_ISL_692659, EPI_ISL_692660, EPI_ISL_692661, EPI_ISL_692662, EPI_ISL_692663, EPI_ISL_692664, EPI_ISL_692665, EPI_ISL_692666, EPI_ISL_692667, EPI_ISL_692668, EPI_ISL_692669, EPI_ISL_692670, EPI_ISL_692671, EPI_ISL_692672, EPI_ISL_692673, EPI_ISL_692674, EPI_ISL_692675, EPI_ISL_692676, EPI_ISL_692677, EPI_ISL_692678, EPI_ISL_692679			
see above	Sakai City Institute of Public Health	Pathogen Genomics Center, National Institute of Infectious Diseases	Tsuyoshi Sekizuka, Kentaro Itokawa, Rina Tanaka, Masanori Hashino, Makoto Kuroda
EPI_ISL_692680, EPI_ISL_692681, EPI_ISL_692682, EPI_ISL_692683, EPI_ISL_692684, EPI_ISL_692685, EPI_ISL_692686, EPI_ISL_692687, EPI_ISL_692688, EPI_ISL_692689, EPI_ISL_692690, EPI_ISL_692691, EPI_ISL_692692, EPI_ISL_692693, EPI_ISL_692694, EPI_ISL_692695, EPI_ISL_692696, EPI_ISL_692697, EPI_ISL_692698, EPI_ISL_692699, EPI_ISL_692700, EPI_ISL_692701, EPI_ISL_692702, EPI_ISL_692703, EPI_ISL_692704, EPI_ISL_692705, EPI_ISL_692706, EPI_ISL_692707, EPI_ISL_692708, EPI_ISL_692709, EPI_ISL_692710, EPI_ISL_692711, EPI_ISL_692712, EPI_ISL_692713, EPI_ISL_692714, EPI_ISL_692715, EPI_ISL_692716, EPI_ISL_692717, EPI_ISL_692718, EPI_ISL_692719, EPI_ISL_692720, EPI_ISL_692721, EPI_ISL_692722, EPI_ISL_692723, EPI_ISL_692724, EPI_ISL_692725, EPI_ISL_692726, EPI_ISL_692727, EPI_ISL_692728, EPI_ISL_692729, EPI_ISL_692730, EPI_ISL_692731, EPI_ISL_692732			
see above	Pathogen Genomics Center, National Institute of Infectious Diseases	Pathogen Genomics Center, National Institute of Infectious Diseases	Tsuyoshi Sekizuka, Kentaro Itokawa, Rina Tanaka, Masanori Hashino, Makoto Kuroda
EPI_ISL_693195	Hospital e Pronto Socorro Portinari	Instituto Adolfo Lutz, Interdiciplinary Procedures Center, Strategic Laboratory	Claudio Tavares Sacchi, Claudia Regina Gonçalves, Erica Valessa Ramos Gomes, Karoline Rodrigues Campos
EPI_ISL_693196	Hospital Santa Clara	Instituto Adolfo Lutz, Interdiciplinary Procedures Center, Strategic Laboratory	Claudio Tavares Sacchi, Claudia Regina Gonçalves, Erica Valessa Ramos Gomes, Karoline Rodrigues Campos
EPI_ISL_693197	Central de Rede de Frio Municipal	Instituto Adolfo Lutz, Interdiciplinary Procedures Center, Strategic Laboratory	Claudio Tavares Sacchi, Claudia Regina Gonçalves, Erica Valessa Ramos Gomes, Karoline Rodrigues Campos
EPI_ISL_693198	Santa Casa de Misericordia de Sao Paulo - Hospital Central	Instituto Adolfo Lutz, Interdiciplinary Procedures Center, Strategic Laboratory	Claudio Tavares Sacchi, Claudia Regina Gonçalves, Erica Valessa Ramos Gomes, Karoline Rodrigues Campos
EPI_ISL_693199	Hospital do Servidor Publico Estadual Francisco Morato de Oliveira	Instituto Adolfo Lutz, Interdiciplinary Procedures Center, Strategic Laboratory	Claudio Tavares Sacchi, Claudia Regina Gonçalves, Erica Valessa Ramos Gomes, Karoline Rodrigues Campos
EPI_ISL_693200	Hospital e Maternidade Mairipora	Instituto Adolfo Lutz, Interdiciplinary Procedures Center, Strategic Laboratory	Claudio Tavares Sacchi, Claudia Regina Gonçalves, Erica Valessa Ramos Gomes, Karoline Rodrigues Campos
EPI_ISL_693201	Hospital Sao Paulo de Ensino da Unifesp	Instituto Adolfo Lutz, Interdiciplinary Procedures Center, Strategic Laboratory	Claudio Tavares Sacchi, Claudia Regina Gonçalves, Erica Valessa Ramos Gomes, Karoline Rodrigues Campos
EPI_ISL_693202	Pronto Socorro Municipal Prof. Joao Catarin Mezomo	Instituto Adolfo Lutz, Interdiciplinary Procedures Center, Strategic Laboratory	Claudio Tavares Sacchi, Claudia Regina Gonçalves, Erica Valessa Ramos Gomes, Karoline Rodrigues Campos

EPI_ISL_693203	Hospital Municipal Doutor Arthur Ribeiro de Saboya	Instituto Adolfo Lutz, Interdiciplinary Procedures Center, Strategic Laboratory	Claudio Tavares Sacchi, Claudia Regina Gonçalves, Erica Valessa Ramos Gomes, Karoline Rodrigues Campos
EPI_ISL_693204	Pronto Socorro Dr. Conrado Cesarino Nuvolini	Instituto Adolfo Lutz, Interdiciplinary Procedures Center, Strategic Laboratory	Claudio Tavares Sacchi, Claudia Regina Gonçalves, Erica Valessa Ramos Gomes, Karoline Rodrigues Campos
EPI_ISL_693205	Hospital de Campanha Covid-19 Assis	Instituto Adolfo Lutz, Interdiciplinary Procedures Center, Strategic Laboratory	Claudio Tavares Sacchi, Claudia Regina Gonçalves, Erica Valessa Ramos Gomes, Karoline Rodrigues Campos
EPI_ISL_693206	Hospital Municipal Mario Gatti	Instituto Adolfo Lutz, Interdiciplinary Procedures Center, Strategic Laboratory	Claudio Tavares Sacchi, Claudia Regina Gonçalves, Erica Valessa Ramos Gomes, Karoline Rodrigues Campos
EPI_ISL_693207	Cs II Doutor Antonio Vicoso Moreira de Rezende	Instituto Adolfo Lutz, Interdiciplinary Procedures Center, Strategic Laboratory	Claudio Tavares Sacchi, Claudia Regina Gonçalves, Erica Valessa Ramos Gomes, Karoline Rodrigues Campos
EPI_ISL_693208, EPI_ISL_693209	Hospital Municipal Antonio Giglio	Instituto Adolfo Lutz, Interdiciplinary Procedures Center, Strategic Laboratory	Claudio Tavares Sacchi, Claudia Regina Gonçalves, Erica Valessa Ramos Gomes, Karoline Rodrigues Campos
EPI_ISL_693210	Pronto-Socorro Dr. Osmar Mesquita	Instituto Adolfo Lutz, Interdiciplinary Procedures Center, Strategic Laboratory	Claudio Tavares Sacchi, Claudia Regina Gonçalves, Erica Valessa Ramos Gomes, Karoline Rodrigues Campos
EPI_ISL_693211	Santa Casa de Misericordia e Maternidade	Instituto Adolfo Lutz, Interdiciplinary Procedures Center, Strategic Laboratory	Claudio Tavares Sacchi, Claudia Regina Gonçalves, Erica Valessa Ramos Gomes, Karoline Rodrigues Campos
EPI_ISL_693212	Santa Casa de Misericordia de Braganca Paulista	Instituto Adolfo Lutz, Interdiciplinary Procedures Center, Strategic Laboratory	Claudio Tavares Sacchi, Claudia Regina Gonçalves, Erica Valessa Ramos Gomes, Karoline Rodrigues Campos
EPI_ISL_693213	Hospital E Maternidade Municipal Governador Mario Covas	Instituto Adolfo Lutz, Interdiciplinary Procedures Center, Strategic Laboratory	Claudio Tavares Sacchi, Claudia Regina Gonçalves, Erica Valessa Ramos Gomes, Karoline Rodrigues Campos
EPI_ISL_693214	Unidade de Pronto Atendimento Central de Caraguatatuba	Instituto Adolfo Lutz, Interdiciplinary Procedures Center, Strategic Laboratory	Claudio Tavares Sacchi, Claudia Regina Gonçalves, Erica Valessa Ramos Gomes, Karoline Rodrigues Campos
EPI_ISL_693215	Secretaria Municipal de Saúde de Iracemapolis	Instituto Adolfo Lutz, Interdiciplinary Procedures Center, Strategic Laboratory	Claudio Tavares Sacchi, Claudia Regina Gonçalves, Erica Valessa Ramos Gomes, Karoline Rodrigues Campos
EPI_ISL_693216, EPI_ISL_693217	Unidade de Vigilância Epiudemiológica de Araras	Instituto Adolfo Lutz, Interdiciplinary Procedures Center, Strategic Laboratory	Claudio Tavares Sacchi, Claudia Regina Gonçalves, Erica Valessa Ramos Gomes, Karoline Rodrigues Campos
EPI_ISL_693218	Hospital Domingos Leonardo Ceravolo Presidente Prudente	Instituto Adolfo Lutz, Interdiciplinary Procedures Center, Strategic Laboratory	Claudio Tavares Sacchi, Claudia Regina Gonçalves, Erica Valessa Ramos Gomes, Karoline Rodrigues Campos
EPI_ISL_693219	Santa Casa da Misericórdia de Presidente Prudente	Instituto Adolfo Lutz, Interdiciplinary Procedures Center, Strategic Laboratory	Claudio Tavares Sacchi, Claudia Regina Gonçalves, Erica Valessa Ramos Gomes, Karoline Rodrigues Campos
EPI_ISL_693220	Laboratório Municipal de Piracicaba	Instituto Adolfo Lutz, Interdiciplinary Procedures Center, Strategic Laboratory	Claudio Tavares Sacchi, Claudia Regina Gonçalves, Erica Valessa Ramos Gomes, Karoline Rodrigues Campos
EPI_ISL_693221, EPI_ISL_693222	Secretaria Municipal de Saúde de Birigui	Instituto Adolfo Lutz, Interdiciplinary Procedures Center, Strategic Laboratory	Claudio Tavares Sacchi, Claudia Regina Gonçalves, Erica Valessa Ramos Gomes, Karoline Rodrigues Campos
EPI_ISL_693223, EPI_ISL_693224	Laboratório Municipal de Piracicaba	Instituto Adolfo Lutz, Interdiciplinary Procedures Center, Strategic Laboratory	Claudio Tavares Sacchi, Claudia Regina Gonçalves, Erica Valessa Ramos Gomes, Karoline Rodrigues Campos
EPI_ISL_693225	Ubs Vila Rosa - Olímpia Gomes De Almeida	Instituto Adolfo Lutz, Interdiciplinary Procedures Center, Strategic Laboratory	Claudio Tavares Sacchi, Claudia Regina Gonçalves, Erica Valessa Ramos Gomes, Karoline Rodrigues Campos
EPI_ISL_693226	Unidade de Pronto Atendimento Sao José	Instituto Adolfo Lutz, Interdiciplinary Procedures Center, Strategic Laboratory	Claudio Tavares Sacchi, Claudia Regina Gonçalves, Erica Valessa Ramos Gomes, Karoline Rodrigues Campos
EPI_ISL_693227	UBS Vila Marchi	Instituto Adolfo Lutz, Interdiciplinary Procedures Center, Strategic Laboratory	Claudio Tavares Sacchi, Claudia Regina Gonçalves, Erica Valessa Ramos Gomes, Karoline Rodrigues Campos
EPI_ISL_693228	Secretaria Municipal de Sorocaba	Instituto Adolfo Lutz, Interdiciplinary Procedures Center, Strategic Laboratory	Claudio Tavares Sacchi, Claudia Regina Gonçalves, Erica Valessa Ramos Gomes, Karoline Rodrigues Campos
EPI_ISL_693229	Hospital 8 de Maio	Instituto Adolfo Lutz, Interdiciplinary Procedures Center, Strategic Laboratory	Claudio Tavares Sacchi, Claudia Regina Gonçalves, Erica Valessa Ramos Gomes, Karoline Rodrigues Campos
EPI_ISL_693230	Hospital e Pronto Socorro Portinari	Instituto Adolfo Lutz, Interdiciplinary Procedures Center, Strategic Laboratory	Claudio Tavares Sacchi, Claudia Regina Gonçalves, Erica Valessa Ramos Gomes, Karoline Rodrigues Campos
EPI_ISL_693231	Pronto Socorro Municipal de Santa Branca	Instituto Adolfo Lutz, Interdiciplinary Procedures Center, Strategic Laboratory	Claudio Tavares Sacchi, Claudia Regina Gonçalves, Erica Valessa Ramos Gomes, Karoline Rodrigues Campos
EPI_ISL_693232	Hospital e Pronto Socorro Portinari	Instituto Adolfo Lutz, Interdiciplinary Procedures Center, Strategic Laboratory	Claudio Tavares Sacchi, Claudia Regina Gonçalves, Erica Valessa Ramos Gomes, Karoline Rodrigues Campos
EPI_ISL_693233	Hospital Santa Cruz	Instituto Adolfo Lutz, Interdiciplinary Procedures Center, Strategic Laboratory	Claudio Tavares Sacchi, Claudia Regina Gonçalves, Erica Valessa Ramos Gomes, Karoline Rodrigues Campos
EPI_ISL_693234	Upa Vereador Jose Da Rocha Goncalves	Instituto Adolfo Lutz, Interdiciplinary Procedures Center, Strategic Laboratory	Claudio Tavares Sacchi, Claudia Regina Gonçalves, Erica Valessa Ramos Gomes, Karoline Rodrigues Campos
EPI_ISL_693235	Casmi Centro Atendimento Saude da Mulher e Infancia	Instituto Adolfo Lutz, Interdiciplinary Procedures Center, Strategic Laboratory	Claudio Tavares Sacchi, Claudia Regina Gonçalves, Erica Valessa Ramos Gomes, Karoline Rodrigues Campos
EPI_ISL_693236	Hospital Santa Marcelina Sao Paulo	Instituto Adolfo Lutz, Interdiciplinary Procedures Center, Strategic Laboratory	Claudio Tavares Sacchi, Claudia Regina Gonçalves, Erica Valessa Ramos Gomes, Karoline Rodrigues Campos
EPI_ISL_693237	UPA Santa Isabel	Instituto Adolfo Lutz, Interdiciplinary Procedures Center, Strategic Laboratory	Claudio Tavares Sacchi, Claudia Regina Gonçalves, Erica Valessa Ramos Gomes, Karoline Rodrigues Campos
EPI_ISL_693238, EPI_ISL_693239	Secao Centro de Diagnostico Secedi	Instituto Adolfo Lutz, Interdiciplinary Procedures Center, Strategic Laboratory	Claudio Tavares Sacchi, Claudia Regina Gonçalves, Erica Valessa Ramos Gomes, Karoline Rodrigues Campos
EPI_ISL_693240	Centro de Vigilância a Saude de Diadema	Instituto Adolfo Lutz, Interdiciplinary Procedures Center, Strategic Laboratory	Claudio Tavares Sacchi, Claudia Regina Gonçalves, Erica Valessa Ramos Gomes, Karoline Rodrigues Campos
EPI_ISL_693241	Hospital e Maternidade Sao Lucas	Instituto Adolfo Lutz, Interdiciplinary Procedures Center, Strategic Laboratory	Claudio Tavares Sacchi, Claudia Regina Gonçalves, Erica Valessa Ramos Gomes, Karoline Rodrigues Campos
EPI_ISL_693242	Centro de Vigilância a Saude de Diadema	Instituto Adolfo Lutz, Interdiciplinary Procedures Center, Strategic Laboratory	Claudio Tavares Sacchi, Claudia Regina Gonçalves, Erica Valessa Ramos Gomes, Karoline Rodrigues Campos

EPI_ISL_693243	Laboratório Municipal de Piracicaba	Instituto Adolfo Lutz, Interdiciplinary Procedures Center, Strategic Laboratory	Claudio Tavares Sacchi, Claudia Regina Gonçalves, Erica Valessa Ramos Gomes, Karoline Rodrigues Campos
EPI_ISL_693244	Centro Médico da Polícia Militar do Estado de Sao Paulo	Instituto Adolfo Lutz, Interdiciplinary Procedures Center, Strategic Laboratory	Claudio Tavares Sacchi, Claudia Regina Gonçalves, Erica Valessa Ramos Gomes, Karoline Rodrigues Campos
EPI_ISL_693245	UPA Santa Isabel	Instituto Adolfo Lutz, Interdiciplinary Procedures Center, Strategic Laboratory	Claudio Tavares Sacchi, Claudia Regina Gonçalves, Erica Valessa Ramos Gomes, Karoline Rodrigues Campos
EPI_ISL_693246	Laboratorio Municipal de Rio Grande da Serra	Instituto Adolfo Lutz, Interdiciplinary Procedures Center, Strategic Laboratory	Claudio Tavares Sacchi, Claudia Regina Gonçalves, Erica Valessa Ramos Gomes, Karoline Rodrigues Campos
EPI_ISL_693247	Secao Centro de Diagnostico Secedi	Instituto Adolfo Lutz, Interdiciplinary Procedures Center, Strategic Laboratory	Claudio Tavares Sacchi, Claudia Regina Gonçalves, Erica Valessa Ramos Gomes, Karoline Rodrigues Campos
EPI_ISL_693248	Centro Municipal de Epidemiologia e Imunizações	Instituto Adolfo Lutz, Interdiciplinary Procedures Center, Strategic Laboratory	Claudio Tavares Sacchi, Claudia Regina Gonçalves, Erica Valessa Ramos Gomes, Karoline Rodrigues Campos
EPI_ISL_693302, EPI_ISL_693303, EPI_ISL_693304, EPI_ISL_693305, EPI_ISL_693306	Department of Laboratory Medicine, National Taiwan University Hospital	Microbial Genomics Core Lab, National Taiwan University Centers of Genomic and Precision Medicine	Shiou-Hwei Yeh, You-Yu Lin, Ya-Yun Lai, Chiao-Ling Li, Shan-Chwen Chang, Pei-Jer Chen, Sui-Yuan Chang
EPI_ISL_693660, EPI_ISL_693661, EPI_ISL_693662, EPI_ISL_693663, EPI_ISL_693665, EPI_ISL_693666, EPI_ISL_693667, EPI_ISL_693669, EPI_ISL_693672, EPI_ISL_693674, EPI_ISL_693675, EPI_ISL_693677, EPI_ISL_693678, EPI_ISL_693679, EPI_ISL_693681, EPI_ISL_693682, EPI_ISL_693683, EPI_ISL_693684, EPI_ISL_693685, EPI_ISL_693687			
see above	The National Institute of Public Health	State Veterinary Institute Prague	Nagy,A;Jirincova,H;Trnka,D;Vecerova,J
EPI_ISL_693758, EPI_ISL_693759, EPI_ISL_693760, EPI_ISL_693761, EPI_ISL_693762, EPI_ISL_693763, EPI_ISL_693764	Hospital	National Reference Center for Viruses of Respiratory Infections, Institut Pasteur, Paris	Marion Barbet, Sylvie Behillil, Méline Bizard, Angela Brisebarre, Camille Capel, Etienne Simon-Lorière, Vincent Enouf, Maud Vanpeene, Sylvie van der Werf, Gisèle Lagathu
EPI_ISL_693765	hospital	National Reference Center for Viruses of Respiratory Infections, Institut Pasteur, Paris	Marion Barbet, Sylvie Behillil, Méline Bizard, Angela Brisebarre, Camille Capel, Etienne Simon-Lorière, Vincent Enouf, Maud Vanpeene, Sylvie van der Werf, Gisèle Lagathu
EPI_ISL_693766, EPI_ISL_693767, EPI_ISL_693768, EPI_ISL_693769, EPI_ISL_693770, EPI_ISL_693771, EPI_ISL_693772, EPI_ISL_693773, EPI_ISL_693774	General practitioner	National Reference Center for Viruses of Respiratory Infections, Institut Pasteur, Paris	Marion Barbet, Sylvie Behillil, Méline Bizard, Angela Brisebarre, Camille Capel, Etienne Simon-Lorière, Vincent Enouf, Maud Vanpeene, Sylvie van der Werf
EPI_ISL_699655, EPI_ISL_699656, EPI_ISL_699657	1-Laboratory of Microbiology, National Reference Lab, Charles Nicolle Hospital; 2-University of Tunis ElManar, Faculty of Medicine of Tunis, LR99ES09, Tunis, Tunisia	1-Clinical and Experimental Pharmacology Lab, LR16SP02, National Center of Pharmacovigilance, University of Tunis El Manar, Tunis, Tunisia. 2-Neurodegenerative diseases and psychiatric troubles, LR18SP03, Razi Hospital, University of Tunis El Manar, Tunis, Tunisia. 3- Ministry of Health, National Observatory of New and Emerging Diseases, 1006, Tunis, Tunisia	Ilhem Boutiba-Ben Boubaker, Sameh Trabelsi, Nissaf Ben Alaya, Maher Kharrat, Alia Ben Kahla, Jalila Ben Khelil, Salma Abid, Sana Ferjani, Asma Ferjani, Mouna Ben Sassi, Mouna Safer, Guedi Berrabeh,Salwa Mrabet, Hanen ElJebari, Gaies Emna, Riadh Daghfous, Riadh Gouider.
EPI_ISL_699658, EPI_ISL_699659, EPI_ISL_699660, EPI_ISL_699661, EPI_ISL_699662, EPI_ISL_699663, EPI_ISL_699664, EPI_ISL_699665, EPI_ISL_699666, EPI_ISL_699667, EPI_ISL_699668, EPI_ISL_699669, EPI_ISL_699670, EPI_ISL_699671, EPI_ISL_699672, EPI_ISL_699674, EPI_ISL_699675, EPI_ISL_699676, EPI_ISL_699677, EPI_ISL_699678, EPI_ISL_699680, EPI_ISL_699681, EPI_ISL_699682, EPI_ISL_699683, EPI_ISL_699684, EPI_ISL_699685, EPI_ISL_699686, EPI_ISL_699687, EPI_ISL_699688, EPI_ISL_699691, EPI_ISL_699692, EPI_ISL_699693, EPI_ISL_699694, EPI_ISL_699695, EPI_ISL_699696, EPI_ISL_699697, EPI_ISL_699698, EPI_ISL_699699, EPI_ISL_699700, EPI_ISL_699701, EPI_ISL_699702, EPI_ISL_699703, EPI_ISL_699704, EPI_ISL_699705, EPI_ISL_699706, EPI_ISL_699709, EPI_ISL_699710, EPI_ISL_699711, EPI_ISL_699712, EPI_ISL_699713, EPI_ISL_699714, EPI_ISL_699715, EPI_ISL_699716, EPI_ISL_699717, EPI_ISL_699718, EPI_ISL_699719, EPI_ISL_699720, EPI_ISL_699721, EPI_ISL_699722, EPI_ISL_699723, EPI_ISL_699724, EPI_ISL_699725, EPI_ISL_699726, EPI_ISL_699727, EPI_ISL_699728, EPI_ISL_699729, EPI_ISL_699730, EPI_ISL_699731, EPI_ISL_699732, EPI_ISL_699733, EPI_ISL_699735, EPI_ISL_699736, EPI_ISL_699737, EPI_ISL_699738, EPI_ISL_699739, EPI_ISL_699740, EPI_ISL_699741, EPI_ISL_699742, EPI_ISL_699743, EPI_ISL_699744, EPI_ISL_699745, EPI_ISL_699746, EPI_ISL_699747, EPI_ISL_699748, EPI_ISL_699749, EPI_ISL_699750, EPI_ISL_699751, EPI_ISL_699752, EPI_ISL_699753, EPI_ISL_699754, EPI_ISL_699759, EPI_ISL_699760, EPI_ISL_699761, EPI_ISL_699763, EPI_ISL_699764, EPI_ISL_699765, EPI_ISL_699766, EPI_ISL_699767, EPI_ISL_699769, EPI_ISL_699770, EPI_ISL_699771, EPI_ISL_699772, EPI_ISL_699773, EPI_ISL_699774, EPI_ISL_699775, EPI_ISL_699776, EPI_ISL_699777, EPI_ISL_699778, EPI_ISL_699779, EPI_ISL_699780, EPI_ISL_699782, EPI_ISL_699783, EPI_ISL_699785, EPI_ISL_699786, EPI_ISL_699787, EPI_ISL_699788, EPI_ISL_699790, EPI_ISL_699791, EPI_ISL_699792, EPI_ISL_699793, EPI_ISL_699794, EPI_ISL_699795, EPI_ISL_699796, EPI_ISL_699797, EPI_ISL_699798, EPI_ISL_699799, EPI_ISL_699802, EPI_ISL_699803, EPI_ISL_699804, EPI_ISL_699805, EPI_ISL_699806, EPI_ISL_699807, EPI_ISL_699808, EPI_ISL_699809, EPI_ISL_699810, EPI_ISL_699811, EPI_ISL_699813, EPI_ISL_699814, EPI_ISL_699815, EPI_ISL_699816, EPI_ISL_699817, EPI_ISL_699818, EPI_ISL_699820, EPI_ISL_699821, EPI_ISL_699822, EPI_ISL_699823, EPI_ISL_699825, EPI_ISL_699826, EPI_ISL_699827, EPI_ISL_699828, EPI_ISL_699829, EPI_ISL_699830, EPI_ISL_699831, EPI_ISL_699832, EPI_ISL_699833, EPI_ISL_699834, EPI_ISL_699835, EPI_ISL_699836, EPI_ISL_699837, EPI_ISL_699838, EPI_ISL_699839, EPI_ISL_699840, EPI_ISL_699841, EPI_ISL_699842, EPI_ISL_699843, EPI_ISL_699844, EPI_ISL_699845, EPI_ISL_699846, EPI_ISL_699847, EPI_ISL_699851, EPI_ISL_699852, EPI_ISL_699853, EPI_ISL_699854, EPI_ISL_699855, EPI_ISL_699856, EPI_ISL_699857, EPI_ISL_699858, EPI_ISL_699859, EPI_ISL_699860, EPI_ISL_699861, EPI_ISL_699862, EPI_ISL_699863, EPI_ISL_699864, EPI_ISL_699865, EPI_ISL_699866, EPI_ISL_699867, EPI_ISL_699868, EPI_ISL_699869, EPI_ISL_699870, EPI_ISL_699871, EPI_ISL_699872, EPI_ISL_699873, EPI_ISL_699874, EPI_ISL_699878, EPI_ISL_699879, EPI_ISL_699880, EPI_ISL_699882, EPI_ISL_699883, EPI_ISL_699884, EPI_ISL_699886, EPI_ISL_699887, EPI_ISL_699888, EPI_ISL_699889, EPI_ISL_699890, EPI_ISL_699891, EPI_ISL_699893, EPI_ISL_699894, EPI_ISL_699897, EPI_ISL_699898, EPI_ISL_699899, EPI_ISL_699902, EPI_ISL_699904, EPI_ISL_699906, EPI_ISL_699907, EPI_ISL_699909, EPI_ISL_699910, EPI_ISL_699911, EPI_ISL_699912, EPI_ISL_699914, EPI_ISL_699915, EPI_ISL_699916, EPI_ISL_699917, EPI_ISL_699918, EPI_ISL_699919, EPI_ISL_699920, EPI_ISL_699921, EPI_ISL_699922, EPI_ISL_699923, EPI_ISL_699924, EPI_ISL_699925, EPI_ISL_699926, EPI_ISL_699927, EPI_ISL_699928, EPI_ISL_699930, EPI_ISL_699931, EPI_ISL_699932, EPI_ISL_699933, EPI_ISL_699934, EPI_ISL_699935, EPI_ISL_699936, EPI_ISL_699937, EPI_ISL_699938, EPI_ISL_699939, EPI_ISL_699940, EPI_ISL_699941, EPI_ISL_699942, EPI_ISL_699943, EPI_ISL_699944, EPI_ISL_699945, EPI_ISL_699946, EPI_ISL_699947, EPI_ISL_699948, EPI_ISL_699949, EPI_ISL_699950, EPI_ISL_699951, EPI_ISL_699952, EPI_ISL_699953, EPI_ISL_699954, EPI_ISL_699955, EPI_ISL_699956, EPI_ISL_699957, EPI_ISL_699958, EPI_ISL_699961, EPI_ISL_699962, EPI_ISL_699963, EPI_ISL_699964, EPI_ISL_699966, EPI_ISL_699967, EPI_ISL_699968, EPI_ISL_699970, EPI_ISL_699971, EPI_ISL_699972, EPI_ISL_699973, EPI_ISL_699974, EPI_ISL_699975, EPI_ISL_699979, EPI_ISL_699980, EPI_ISL_699981, EPI_ISL_699983, EPI_ISL_699984, EPI_ISL_699985, EPI_ISL_699986, EPI_ISL_699987, EPI_ISL_699988, EPI_ISL_699989, EPI_ISL_699990, EPI_ISL_699991, EPI_ISL_699992, EPI_ISL_699993, EPI_ISL_699994, EPI_ISL_699996, EPI_ISL_699997, EPI_ISL_699998, EPI_ISL_699999, EPI_ISL_700000, EPI_ISL_700002, EPI_ISL_700003, EPI_ISL_700004, EPI_ISL_700005, EPI_ISL_700006, EPI_ISL_700009, EPI_ISL_700010, EPI_ISL_700011, EPI_ISL_700012, EPI_ISL_700013, EPI_ISL_700015, EPI_ISL_700016, EPI_ISL_700017, EPI_ISL_700018, EPI_ISL_700020, EPI_ISL_700021, EPI_ISL_700024, EPI_ISL_700025, EPI_ISL_700027, EPI_ISL_700028, EPI_ISL_700030, EPI_ISL_700031, EPI_ISL_700033, EPI_ISL_700034, EPI_ISL_700036, EPI_ISL_700037, EPI_ISL_700038, EPI_ISL_700039, EPI_ISL_700040, EPI_ISL_700041, EPI_ISL_700043, EPI_ISL_700044, EPI_ISL_700046, EPI_ISL_700047, EPI_ISL_700048, EPI_ISL_700049, EPI_ISL_700050, EPI_ISL_700051, EPI_ISL_700052, EPI_ISL_700053, EPI_ISL_700054, EPI_ISL_700055, EPI_ISL_700056, EPI_ISL_700057, EPI_ISL_700058, EPI_ISL_700059, EPI_ISL_700061, EPI_ISL_700064, EPI_ISL_700065, EPI_ISL_700066, EPI_ISL_700067, EPI_ISL_700068, EPI_ISL_700069, EPI_ISL_700070, EPI_ISL_700071, EPI_ISL_700072, EPI_ISL_700073, EPI_ISL_700074, EPI_ISL_700076, EPI_ISL_700077, EPI_ISL_700078, EPI_ISL_700079, EPI_ISL_700080, EPI_ISL_700081, EPI_ISL_700082, EPI_ISL_700083, EPI_ISL_700084, EPI_ISL_700085, EPI_ISL_700086, EPI_ISL_700088, EPI_ISL_700089, EPI_ISL_700091, EPI_ISL_700092, EPI_ISL_700094, EPI_ISL_700096, EPI_ISL_700097, EPI_ISL_700098, EPI_ISL_700099, EPI_ISL_700100, EPI_ISL_700101, EPI_ISL_700102, EPI_ISL_700103, EPI_ISL_700104, EPI_ISL_700107, EPI_ISL_700108, EPI_ISL_700109, EPI_ISL_700110, EPI_ISL_700111, EPI_ISL_700112, EPI_ISL_700113, EPI_ISL_700114, EPI_ISL_700115, EPI_ISL_700116, EPI_ISL_700118, EPI_ISL_700120, EPI_ISL_700123, EPI_ISL_700124, EPI_ISL_700125, EPI_ISL_700127, EPI_ISL_700128, EPI_ISL_700129, EPI_ISL_700130, EPI_ISL_700131, EPI_ISL_700132, EPI_ISL_700136, EPI_ISL_700138, EPI_ISL_700140, EPI_ISL_700143, EPI_ISL_700144, EPI_ISL_700145, EPI_ISL_700146, EPI_ISL_700147, EPI_ISL_700149, EPI_ISL_700150, EPI_ISL_700151, EPI_ISL_700154, EPI_ISL_700155, EPI_ISL_700156, EPI_ISL_700157, EPI_ISL_700158, EPI_ISL_700160, EPI_ISL_700161, EPI_ISL_700162, EPI_ISL_700164, EPI_ISL_700166, EPI_ISL_700167, EPI_ISL_700168, EPI_ISL_700169, EPI_ISL_700172, EPI_ISL_700174, EPI_ISL_700176, EPI_ISL_700177, EPI_ISL_700179, EPI_ISL_700180, EPI_ISL_700181, EPI_ISL_700183, EPI_ISL_700184, EPI_ISL_700185, EPI_ISL_700187, EPI_ISL_700188, EPI_ISL_700189, EPI_ISL_700190, EPI_ISL_700191, EPI_ISL_700192, EPI_ISL_700193, EPI_ISL_700194, EPI_ISL_700195, EPI_ISL_700196, EPI_ISL_700197, EPI_ISL_700198, EPI_ISL_700199, EPI_ISL_700200, EPI_ISL_700201, EPI_ISL_700202, EPI_ISL_700203, EPI_ISL_700204, EPI_ISL_700205, EPI_ISL_700206, EPI_ISL_700208, EPI_ISL_700209, EPI_ISL_700210, EPI_ISL_700211, EPI_ISL_700212, EPI_ISL_700213, EPI_ISL_700214, EPI_ISL_700215, EPI_ISL_700216, EPI_ISL_700217, EPI_ISL_700219, EPI_ISL_700221, EPI_ISL_700224, EPI_ISL_700227, EPI_ISL_700229, EPI_ISL_700230, EPI_ISL_700231, EPI_ISL_700232, EPI_ISL_700234, EPI_ISL_700237, EPI_ISL_700238, EPI_ISL_700240, EPI_ISL_700241, EPI_ISL_700242, EPI_ISL_700245, EPI_ISL_700246, EPI_ISL_700247, EPI_ISL_700248, EPI_ISL_700249, EPI_ISL_700250, EPI_ISL_700251, EPI_ISL_700252, EPI_ISL_700253, EPI_ISL_700254, EPI_ISL_700255, EPI_ISL_700256, EPI_ISL_700258, EPI_ISL_700260, EPI_ISL_700262, EPI_ISL_700263, EPI_ISL_700265, EPI_ISL_700266, EPI_ISL_700267, EPI_ISL_700268, EPI_ISL_700269, EPI_ISL_700270, EPI_ISL_700271, EPI_ISL_700273, EPI_ISL_700274, EPI_ISL_700275, EPI_ISL_700276, EPI_ISL_700277, EPI_ISL_700278, EPI_ISL_700279, EPI_ISL_700280, EPI_ISL_700281, EPI_ISL_700282, EPI_ISL_700283, EPI_ISL_700284, EPI_ISL_700285, EPI_ISL_700286, EPI_ISL_700287, EPI_ISL_700288, EPI_ISL_700289, EPI_ISL_700290, EPI_ISL_700291, EPI_ISL_700293, EPI_ISL_700294, EPI_ISL_700295, EPI_ISL_700296, EPI_ISL_700297, EPI_ISL_700298, EPI_ISL_700299, EPI_ISL_700302, EPI_ISL_700303, EPI_ISL_700304, EPI_ISL_700305, EPI_ISL_700306, EPI_ISL_700307, EPI_ISL_700308, EPI_ISL_700309, EPI_ISL_700310, EPI_ISL_700311, EPI_ISL_700312, EPI_ISL_700313, EPI_ISL_700314, EPI_ISL_700315, EPI_ISL_700316, EPI_ISL_700317, EPI_ISL_700318, EPI_ISL_700320, EPI_ISL_700321, EPI_ISL_700322, EPI_ISL_700323, EPI_ISL_700324, EPI_ISL_700325			
see above	Hematopathology Laboratory, ACTREC, TMC	Hematopathology Laboratory, ACTREC, TMC	Hematopathology Laboratory, ACTREC
EPI_ISL_700371	CHU Nantes	CNR Virus des Infections Respiratoires - France SUD	Antonin Bal, Louise Castain, Gregory Destras, Gwendolyne Burfin, Hadrien Règue, Quentin Semanas, Martine Valette, Bruno Lina, Virginie Ferré, Celine Bressollette, Laurence Josset
EPI_ISL_707696	Medical Research Center, Faculty of Medicine, Syarif Hidayatullah State Islamic University Jakarta	Medical Research Center, Faculty of Medicine, Syarif Hidayatullah State Islamic University Jakarta	Laifa Hendarmin, Chris Adhiyanto, Erike Suwarsono, Zeti Harriyati, Rini Puspitaningrum, Ferania Mela, Dennis Nurjadi
EPI_ISL_707697, EPI_ISL_707698, EPI_ISL_707699, EPI_ISL_707700	1-Laboratory of Microbiology, National Reference Lab, Charles Nicolle Hospital; 2-University of Tunis ElManar, Faculty of Medicine of Tunis, LR99ES09, Tunis, Tunisia	1-Clinical and Experimental Pharmacology Lab, LR16SP02, National Center of Pharmacovigilance, University of Tunis El Manar, Tunis, Tunisia. 2-Neurodegenerative diseases and psychiatric troubles, LR18SP03, Razi Hospital, University of Tunis El Manar, Tunis, Tunisia. 3- Ministry of Health, National Observatory of New and Emerging Diseases, 1006, Tunis, Tunisia	Ilhem Boutiba-Ben Boubaker, Sameh Trabelsi, Nissaf Ben Alaya, Maher Kharrat, Alia Ben Kahla, Jalila Ben Khelil, Salma Abid, Sana Ferjani, Mouna Ben Sassi, Mouna Safer, Zaineb Hamzaoui, Habiba Ben Romdhane, Souissi Amira, Rouaa Ben Othman, Hanen El Jebari, Asma Ferjani, Gaies Emna, Riadh Daghfous, Riadh Gouider.

EPI_ISL_707711, EPI_ISL_707712, EPI_ISL_707713, EPI_ISL_707771, EPI_ISL_707772, EPI_ISL_707773, EPI_ISL_707774, EPI_ISL_707776, EPI_ISL_707777	Rwanda National Reference Laboratory	Rwanda National Reference Laboratory	Enatha Mukantwari,Jeanne d'Arc Umuringa
EPI_ISL_707778	Medical Research Center, Faculty of Medicine, Syarif Hidayatullah State Islamic University Jakarta	Medical Research Center, Faculty of Medicine, Syarif Hidayatullah State Islamic University Jakarta	Erike A Suwarsono, Chris Adhiyanto, Laifa Hendarmin, Zeti Harriyati, Rini Puspitaningrum, Ferania Mela, Dennis Nurjadi
EPI_ISL_707779	Rwanda National Reference Laboratory	Rwanda National Reference Laboratory	Enatha Mukantwari,Jeanne d'Arc UMuringa
EPI_ISL_707780	Rwanda National Reference Laboratory	Rwanda National Reference Laboratory	Enatha Mukantwari,Jeanne d'Arc Umuringa
EPI_ISL_707783	Rwanda National Reference Laboratory	Rwanda National Reference Laboratory	ENatha MUkantwari,Jeanne d'Arc Mukantwari
EPI_ISL_707787, EPI_ISL_707788	Rwanda National Reference Laboratory	Rwanda National Reference Laboratory	Enatha Mukantwari,Jeanne d'Arc Umuringa
EPI_ISL_707789	Rwanda National Reference Laboratory	Rwanda National Reference Laboratory	Enatha Mukantwari,Jeanne d'Arc
EPI_ISL_707790	Rwanda National Reference Laboratory	Rwanda National Reference Laboratory	Enatha Mukantwari,Jeanne d'Arc Umuringa
EPI_ISL_707791	1-Laboratory of Microbiology, National Reference Lab, Charles Nicolle Hospital; 2-University of Tunis ElManar, Faculty of Medicine of Tunis, LR99ES09, Tunis, Tunisia	1-Clinical and Experimental Pharmacology Lab, LR16SP02, National Center of Pharmacovigilance, University of Tunis El Manar, Tunis, Tunisia. 2-Neurodegenerative diseases and psychiatric troubles, LR18SP03, Razi Hospital, University of Tunis El Manar, Tunis, Tunisia. 3- Ministry of Health, National Observatory of New and Emerging Diseases, 1006, Tunis, Tunisia	Ilhem Boutiba-Ben Boubaker, Sameh Trabelsi, Nissaf Ben Alaya, Maher Kharrat, Alia Ben Kahla, Jalila Ben Khelil, Salma Abid, Sana Ferjani, Mouna Ben Sassi, Mouna Safer, Awatef El MOussi, Habiba Ben Romdhane, Souissi Amira, Ines Mdini, Hanen El Jebari, Asma Ferjani, Gaies Emna, Riadh Daghfous, Riadh Gouider.
EPI_ISL_707890	Medical Research Center, Faculty of Medicine, Syarif Hidayatullah State Islamic University Jakarta	Medical Research Center, Faculty of Medicine, Syarif Hidayatullah State Islamic University Jakarta	Zeti Harriyati, Chris Adhiyanto, Laifa Hendarmin, Erike A Suwarsono, Dennis Nurjadi
EPI_ISL_707928	Fujita Health University Hospital	Fujita Health University School of Medicine, Department of Microbiology	Masahiro Suzuki, Aki Sakurai, Yohei Doi
EPI_ISL_707936, EPI_ISL_707937, EPI_ISL_707938, EPI_ISL_707939, EPI_ISL_708184, EPI_ISL_708185, EPI_ISL_708186, EPI_ISL_708188, EPI_ISL_708189, EPI_ISL_708190, EPI_ISL_708191, EPI_ISL_708192, EPI_ISL_708193, EPI_ISL_708194, EPI_ISL_708195			
see above	Pamukkale University Hospital	Pamukkale University Department of Medical Genetics	Onur TOKGUN et al.
EPI_ISL_708529	Secretária Municipal de Saude de Fernandópolis	Instituto Adolfo Lutz, Interdiciplinary Procedures Center, Strategic Laboratory	Claudio Tavares Sacchi, Claudia Regina Gonçalves, Erica Valessa Ramos Gomes, Carlos Henrique Camargo, Karoline Rodrigues Campos, Fernanda Modesto Tolentino Binhardi, Maricelia Navarro Pinheiro Flores, Marcia Maria Costa Nunes Soares, Janaina Other Martins Montanha
EPI_ISL_708530	Secretaria Municipal de Saude de Fernandópolis	Instituto Adolfo Lutz, Interdiciplinary Procedures Center, Strategic Laboratory	Claudio Tavares Sacchi, Claudia Regina Gonçalves, Erica Valessa Ramos Gomes, Carlos Henrique Camargo, Karoline Rodrigues Campos, Fernanda Modesto Tolentino Binhardi, Maricelia Navarro Pinheiro Flores, Marcia Maria Costa Nunes Soares, Janaina Other Martins Montanha
EPI_ISL_708652	Medical Research Center, Faculty of Medicine, Syarif Hidayatullah State Islamic University Jakarta	Medical Research Center, Faculty of Medicine, Syarif Hidayatullah State Islamic University Jakarta	Chris Adhiyanto, Laifa Hendarmin, Erike A Suwarsono, Zeti Harriyati, Endah Wulandari, Flori R Sari, Hari Hendarto
EPI_ISL_708839, EPI_ISL_708840, EPI_ISL_709044, EPI_ISL_709542	National Institute of Blood Diseases (NIBD), Molecular Biology Lab	Genomics Lab NIBD	Samina Naz Mukry, Sayed Ali Raza, Shariq Ahmed, Aneeta Shahni, Gul Sufaida, Arshi Naz , Tahir Sultan Shamsi
EPI_ISL_710483, EPI_ISL_710484	Department of Medical Laboratory Sciences, Arab American University	Department of Medical Laboratory Sciences, Arab American University	Al-Jawabreh,A., Nasereddin,A., Dumaidi,K., Al-Jawabreh,H., Ereqat,S.
EPI_ISL_710503	Laboratorio specialistico UOC Ematologia - Ospedale "San Francesco" - ATS-ASSL Nuoro	Laboratorio specialistico UOC Ematologia - Ospedale "San Francesco" - ATS-ASSL Nuoro	Giovanna Piras
EPI_ISL_710534, EPI_ISL_710537, EPI_ISL_710540, EPI_ISL_710541, EPI_ISL_710575, EPI_ISL_711057, EPI_ISL_712060	Hôpital Fattouma-Bourguiba de Monastir	Laboratoire des Procédés de Criblage Moléculaire et Cellulaire-Centre de Biotechnologie de Sfax	Souissi,A., Abid,N., Ben Ayed,I., Gargouri,S., Abdelmoulah,F.,Elargoubi,A., Smeti,I., Bensaid,M., Stambouli,N., Kharat,N., Ajili,F., Fki-berrajah,L., Mhalla,S., Chtourou,A., Gaaloul,I., Nabli,A., Turki,M., Aouni,M., Hammami,A., Mastouri,M., Karray Hakim,H., Kamoun,S., Rebai,A. and Masmoudi,S.
EPI_ISL_712068, EPI_ISL_712069	Laboratoire de Microbiologie- CHU Habib Bourguiba - Sfax adresse	Laboratoire des Procédés de Criblage Moléculaire et Cellulaire-Centre de Biotechnologie de Sfax	Souissi,A., Abid,N., Ben Ayed,I., Gargouri,S., Abdelmoulah,F.,Elargoubi,A., Smeti,I., Bensaid,M., Stambouli,N., Kharat,N., Ajili,F., Fki-berrajah,L., Mhalla,S., Chtourou,A., Gaaloul,I., Nabli,A., Turki,M., Aouni,M., Hammami,A., Mastouri,M., Karray Hakim,H., Kamoun,S., Rebai,A. and Masmoudi,S.
EPI_ISL_717965, EPI_ISL_717966, EPI_ISL_717967, EPI_ISL_717968, EPI_ISL_717969, EPI_ISL_717970, EPI_ISL_717971, EPI_ISL_717972, EPI_ISL_717973, EPI_ISL_717974	Molecular diagnostic laboratory of Federal Budget Institution of Science "Central Research Institute of Epidemiology" of The Federal Service on Customers' Rights Protection and Human Well-being Surveillance	Group of Genomics and Postgenomic Technologies of Central Research Institute of Epidemilology	Samoilov AE, Kaptelova VV, Dudorova AV, Korneenko EV, Speranskaya AS, Tivanova EV, Shipulina OY, Akimkin VG
EPI_ISL_718132, EPI_ISL_718133, EPI_ISL_718134, EPI_ISL_718135, EPI_ISL_718136, EPI_ISL_718137, EPI_ISL_718138, EPI_ISL_718139, EPI_ISL_718140, EPI_ISL_718141, EPI_ISL_718142, EPI_ISL_718143, EPI_ISL_718144, EPI_ISL_718145, EPI_ISL_718146, EPI_ISL_718147, EPI_ISL_718148, EPI_ISL_718149, EPI_ISL_718150, EPI_ISL_718151, EPI_ISL_718152, EPI_ISL_718153, EPI_ISL_718154, EPI_ISL_718155, EPI_ISL_718156, EPI_ISL_718157, EPI_ISL_718158, EPI_ISL_718159, EPI_ISL_718160, EPI_ISL_718161, EPI_ISL_718162, EPI_ISL_718163, EPI_ISL_718164, EPI_ISL_718165, EPI_ISL_718166			
see above	Ministry of Health Hospitals	Institute of Health and Community Medicine	David Perera, Ooi Mong How, Chua Hock Hin, Tonnii Sia Loong Loong, Wong Jyn Shan, Wong Kiing Aik, Chan Chia Jui
EPI_ISL_718167	Borneo Medical Centre	Institute of Health and Community Medicine	David Perera, Ooi Mong How, Chua Hock Hin, Tonnii Sia Loong Loong, Wong Jyn Shan, Wong Kiing Aik, Chan Chia Jui
EPI_ISL_718168, EPI_ISL_718169, EPI_ISL_718170, EPI_ISL_718171, EPI_ISL_718172, EPI_ISL_718173	Ministry of Health Hospitals	Institute of Health and Community Medicine	David Perera, Ooi Mong How, Chua Hock Hin, Tonnii Sia Loong Loong, Wong Jyn Shan, Wong Kiing Aik, Chan Chia Jui
EPI_ISL_718174	Borneo Medical Centre	Institute of Health and Community Medicine	David Perera, Ooi Mong How, Chua Hock Hin, Tonnii Sia Loong Loong, Wong Jyn Shan, Wong Kiing Aik, Chan Chia Jui
EPI_ISL_718175	Ministry of Health Hospitals	Institute of Health and Community Medicine	David Perera, Ooi Mong How, Chua Hock Hin, Tonnii Sia Loong Loong, Wong Jyn Shan, Wong Kiing Aik, Chan Chia Jui
EPI_ISL_718176	Borneo Medical Centre	Institute of Health and Community Medicine	David Perera, Ooi Mong How, Chua Hock Hin, Tonnii Sia Loong Loong, Wong Jyn Shan, Wong Kiing Aik, Chan Chia Jui
EPI_ISL_718177	Ministry of Health Hospitals	Institute of Health and Community Medicine	David Perera, Ooi Mong How, Chua Hock Hin, Tonnii Sia Loong Loong, Wong Jyn Shan, Wong Kiing Aik, Chan Chia Jui
EPI_ISL_718178	Borneo Medical Centre	Institute of Health and Community Medicine	David Perera, Ooi Mong How, Chua Hock Hin, Tonnii Sia Loong Loong, Wong Jyn Shan, Wong Kiing Aik, Chan Chia Jui
EPI_ISL_718179, EPI_ISL_718180, EPI_ISL_718181	Ministry of Health Hospitals	Institute of Health and Community Medicine	David Perera, Ooi Mong How, Chua Hock Hin, Tonnii Sia Loong Loong, Wong Jyn Shan, Wong Kiing Aik, Chan Chia Jui
EPI_ISL_718182	Borneo Medical Centre	Institute of Health and Community Medicine	David Perera, Ooi Mong How, Chua Hock Hin, Tonnii Sia Loong Loong, Wong Jyn Shan, Wong Kiing Aik, Chan Chia Jui
EPI_ISL_718183, EPI_ISL_718184	Ministry of Health Hospitals	Institute of Health and Community Medicine	David Perera, Ooi Mong How, Chua Hock Hin, Tonnii Sia Loong Loong, Wong Jyn Shan, Wong Kiing Aik, Chan Chia Jui
EPI_ISL_718185	Borneo Medical Centre	Institute of Health and Community Medicine	David Perera, Ooi Mong How, Chua Hock Hin, Tonnii Sia Loong Loong, Wong Jyn Shan, Wong Kiing Aik, Chan Chia Jui
EPI_ISL_718186, EPI_ISL_718187, EPI_ISL_718188, EPI_ISL_718189, EPI_ISL_718190	Ministry of Health Hospitals	Institute of Health and Community Medicine	David Perera, Ooi Mong How, Chua Hock Hin, Tonnii Sia Loong Loong, Wong Jyn Shan, Wong Kiing Aik, Chan Chia Jui

EPI_ISL_718191, EPI_ISL_718192	Borneo Medical Centre	Institute of Health and Community Medicine	David Perera, Ooi Mong How, Chua Hock Hin, Tonnii Sia Loong Loong, Wong Jyn Shan, Wong Kiing Aik, Chan Chia Jui
EPI_ISL_718193, EPI_ISL_718194, EPI_ISL_718195, EPI_ISL_718196	Ministry of Health Hospitals	Institute of Health and Community Medicine	David Perera, Ooi Mong How, Chua Hock Hin, Tonnii Sia Loong Loong, Wong Jyn Shan, Wong Kiing Aik, Chan Chia Jui
EPI_ISL_718197, EPI_ISL_718198	Borneo Medical Centre	Institute of Health and Community Medicine	David Perera, Ooi Mong How, Chua Hock Hin, Tonnii Sia Loong Loong, Wong Jyn Shan, Wong Kiing Aik, Chan Chia Jui
EPI_ISL_718199, EPI_ISL_718200, EPI_ISL_718201, EPI_ISL_718202	Ministry of Health Hospitals	Institute of Health and Community Medicine	David Perera, Ooi Mong How, Chua Hock Hin, Tonnii Sia Loong Loong, Wong Jyn Shan, Wong Kiing Aik, Chan Chia Jui
EPI_ISL_718203, EPI_ISL_718204, EPI_ISL_718205	Borneo Medical Centre	Institute of Health and Community Medicine	David Perera, Ooi Mong How, Chua Hock Hin, Tonnii Sia Loong Loong, Wong Jyn Shan, Wong Kiing Aik, Chan Chia Jui
EPI_ISL_718206, EPI_ISL_718207, EPI_ISL_718208, EPI_ISL_718209, EPI_ISL_718210, EPI_ISL_718211, EPI_ISL_718212, EPI_ISL_718213, EPI_ISL_718214, EPI_ISL_718215	Ministry of Health Hospitals	Institute of Health and Community Medicine	David Perera, Ooi Mong How, Chua Hock Hin, Tonnii Sia Loong Loong, Wong Jyn Shan, Wong Kiing Aik, Chan Chia Jui
EPI_ISL_718216	Borneo Medical Centre	Institute of Health and Community Medicine	David Perera, Ooi Mong How, Chua Hock Hin, Tonnii Sia Loong Loong, Wong Jyn Shan, Wong Kiing Aik, Chan Chia Jui
EPI_ISL_718217	Ministry of Health Hospitals	Institute of Health and Community Medicine	David Perera, Ooi Mong How, Chua Hock Hin, Tonnii Sia Loong Loong, Wong Jyn Shan, Wong Kiing Aik, Chan Chia Jui
EPI_ISL_718218	Borneo Medical Centre	Institute of Health and Community Medicine	David Perera, Ooi Mong How, Chua Hock Hin, Tonnii Sia Loong Loong, Wong Jyn Shan, Wong Kiing Aik, Chan Chia Jui
EPI_ISL_718219, EPI_ISL_718220, EPI_ISL_718221	Ministry of Health Hospitals	Institute of Health and Community Medicine	David Perera, Ooi Mong How, Chua Hock Hin, Tonnii Sia Loong Loong, Wong Jyn Shan, Wong Kiing Aik, Chan Chia Jui
EPI_ISL_718222	Borneo Medical Centre	Institute of Health and Community Medicine	David Perera, Ooi Mong How, Chua Hock Hin, Tonnii Sia Loong Loong, Wong Jyn Shan, Wong Kiing Aik, Chan Chia Jui
EPI_ISL_718226, EPI_ISL_718227, EPI_ISL_718228, EPI_ISL_718229, EPI_ISL_718230, EPI_ISL_718231, EPI_ISL_718232, EPI_ISL_718233, EPI_ISL_718234, EPI_ISL_718235, EPI_ISL_718236, EPI_ISL_718237, EPI_ISL_718238, EPI_ISL_718239, EPI_ISL_718240, EPI_ISL_718241, EPI_ISL_718242, EPI_ISL_718243, EPI_ISL_718244, EPI_ISL_718245, EPI_ISL_718246, EPI_ISL_718247, EPI_ISL_718248, EPI_ISL_718249			
see above	Hospital	National Reference Center for Viruses of Respiratory Infections, Institut Pasteur, Paris	Marion Barbet, Sylvie Behillil, Méline Bizard, Angela Brisebarre, Camille Capel, Etienne Simon-Lorière, Vincent Enouf, Maud Vanpeene, Sylvie van der Werf, Gisèle Lagathu
EPI_ISL_718267, EPI_ISL_718268, EPI_ISL_718271, EPI_ISL_718272	Institute for Medical Research, Infectious Disease Research Centre, National Institutes of Health, Ministry of Health Malaysia	Institute for Medical Research, Infectious Disease Research Centre, National Institutes of Health, Ministry of Health Malaysia	Suppiah J, Kamel K, Mohd-Zawawi Z, Thayan R
EPI_ISL_718273	Fujita Health University Okazaki Medical Center	Department of Virology and Parasitology, Fujita Health University School of Medicine	Takayuki Murata, Aki Sakurai, Satoshi Komoto, Tomihiko Ide,Takuma Ishihara, Masahiro suzuki, Yohei Doi
EPI_ISL_718274, EPI_ISL_718275, EPI_ISL_718277, EPI_ISL_718278, EPI_ISL_718279	Institute for Medical Research, Infectious Disease Research Centre, National Institutes of Health, Ministry of Health Malaysia	Institute for Medical Research, Infectious Disease Research Centre, National Institutes of Health, Ministry of Health Malaysia	Suppiah J, Kamel K, Mohd-Zawawi Z, Thayan R
EPI_ISL_721995, EPI_ISL_721998, EPI_ISL_721999, EPI_ISL_722005, EPI_ISL_722006, EPI_ISL_722008, EPI_ISL_722010, EPI_ISL_722011, EPI_ISL_722016, EPI_ISL_722017, EPI_ISL_722018, EPI_ISL_722019, EPI_ISL_722022, EPI_ISL_722039, EPI_ISL_722040, EPI_ISL_722045, EPI_ISL_722051, EPI_ISL_722061, EPI_ISL_722065, EPI_ISL_722066, EPI_ISL_722071, EPI_ISL_722076, EPI_ISL_722089, EPI_ISL_722091, EPI_ISL_722093, EPI_ISL_722094, EPI_ISL_722098, EPI_ISL_722110, EPI_ISL_722117, EPI_ISL_722118, EPI_ISL_722128			
see above	Hospital das Clínicas Universidade de São Paulo Medical School	Laboratório de Parasitologia Médica - Instituto de Medicina Tropical - Universidade de São Paulo	Brazil-UK Centre for Arbovirus Discovery Diagnosis Genomics and Epidemiology (CADDE) Genomic Network - Instituto de Medicina Tropical
EPI_ISL_722851	I.R.C.C.S. "S. De Bellis" - Ente Ospedaliero	Istituto Zooprofilattico Sperimentale della Puglia e della Basilicata	Parisi A., Bianco A., Capozzi L., Del Sambro L., Lippolis A., Notarnicola M., Manzulli V, Rondinone V., Pace L.
EPI_ISL_722852	I.R.C.C.S. "S. De Bellis" - Ente Ospedaliero	Istituto Zooprofilattico Sperimentale della Puglia e della Basilicata	Parisi A., Bianco A., Capozzi L., Del Sambro L., Lippolis A., Notarnicola M., Cipolletta D., Galante D.
EPI_ISL_722853	I.R.C.C.S. "S. De Bellis" - Ente Ospedaliero	Istituto Zooprofilattico Sperimentale della Puglia e della Basilicata	Parisi A., Bianco A., Capozzi L., Del Sambro L., Lippolis A., Notarnicola M., Manzulli V, Rondinone V., Pace L.
EPI_ISL_722854	I.R.C.C.S. "S. De Bellis" - Ente Ospedaliero	Istituto Zooprofilattico Sperimentale della Puglia e della Basilicata	Parisi A., Bianco A., Capozzi L., Del Sambro L., Lippolis A., Notarnicola M., Cipolletta D., Galante D.
EPI_ISL_722855, EPI_ISL_722856, EPI_ISL_722857, EPI_ISL_722858	Dipartimento di Scienze Biomediche e Oncologia Umana - Azienda Ospedaliero Universitaria Consorziale Policlinico	Istituto Zooprofilattico Sperimentale della Puglia e della Basilicata	Parisi A., Bianco A., Capozzi L., Del Sambro L., Chironna M., Loconsole D.
EPI_ISL_722859, EPI_ISL_722860	I.R.C.C.S. "S. De Bellis" - Ente Ospedaliero	Istituto Zooprofilattico Sperimentale della Puglia e della Basilicata	Parisi A., Bianco A., Capozzi L., Del Sambro L., Lippolis A., Notarnicola M., Manzulli V, Rondinone V., Pace L.
EPI_ISL_722861	I.R.C.C.S. "S. De Bellis" - Ente Ospedaliero	Istituto Zooprofilattico Sperimentale della Puglia e della Basilicata	Parisi A., Bianco A., Capozzi L., Del Sambro L., Lippolis A., Notarnicola M., Cipolletta D., Galante D.
EPI_ISL_722862	I.R.C.C.S. "S. De Bellis" - Ente Ospedaliero	Istituto Zooprofilattico Sperimentale della Puglia e della Basilicata	Parisi A., Bianco A., Capozzi L., Del Sambro L., Lippolis A., Notarnicola M., Manzulli V, Rondinone V., Pace L.
EPI_ISL_722863	I.R.C.C.S. "S. De Bellis" - Ente Ospedaliero	Istituto Zooprofilattico Sperimentale della Puglia e della Basilicata	Parisi A., Bianco A., Capozzi L., Del Sambro L., Lippolis A., Notarnicola M., Cipolletta D., Galante D.
EPI_ISL_722864, EPI_ISL_722865	I.R.C.C.S. "S. De Bellis" - Ente Ospedaliero	Istituto Zooprofilattico Sperimentale della Puglia e della Basilicata	Parisi A., Bianco A., Capozzi L., Del Sambro L., Lippolis A., Notarnicola M., Manzulli V, Rondinone V., Pace L.
EPI_ISL_722866	I.R.C.C.S. "S. De Bellis" - Ente Ospedaliero	Istituto Zooprofilattico Sperimentale della Puglia e della Basilicata	Parisi A., Bianco A., Capozzi L., Del Sambro L., Lippolis A., Notarnicola M., Cipolletta D., Galante D.
EPI_ISL_722867, EPI_ISL_722868	I.R.C.C.S. "S. De Bellis" - Ente Ospedaliero	Istituto Zooprofilattico Sperimentale della Puglia e della Basilicata	Parisi A., Bianco A., Capozzi L., Del Sambro L., Lippolis A., Notarnicola M., Manzulli V, Rondinone V., Pace L.
EPI_ISL_722869	I.R.C.C.S. "S. De Bellis" - Ente Ospedaliero	Istituto Zooprofilattico Sperimentale della Puglia e della Basilicata	Parisi A., Bianco A., Capozzi L., Del Sambro L., Lippolis A., Notarnicola M., Cipolletta D., Galante D.
EPI_ISL_722870	I.R.C.C.S. "S. De Bellis" - Ente Ospedaliero	Istituto Zooprofilattico Sperimentale della Puglia e della Basilicata	Parisi A., Bianco A., Capozzi L., Del Sambro L., Lippolis A., Notarnicola M., Manzulli V, Rondinone V., Pace L.
EPI_ISL_722871	I.R.C.C.S. "S. De Bellis" - Ente Ospedaliero	Istituto Zooprofilattico Sperimentale della Puglia e della Basilicata	Parisi A., Bianco A., Capozzi L., Del Sambro L., Lippolis A., Notarnicola M., Cipolletta D., Galante D.
EPI_ISL_722872	Dipartimento di Scienze Biomediche e Oncologia Umana - Azienda Ospedaliero Universitaria Consorziale Policlinico	Istituto Zooprofilattico Sperimentale della Puglia e della Basilicata	Parisi A., Bianco A., Capozzi L., Del Sambro L., Chironna M., Loconsole D.
EPI_ISL_722873, EPI_ISL_722874, EPI_ISL_722875, EPI_ISL_722876, EPI_ISL_722877, EPI_ISL_722878, EPI_ISL_722879, EPI_ISL_722880, EPI_ISL_722881, EPI_ISL_722882, EPI_ISL_722883, EPI_ISL_722884, EPI_ISL_722885, EPI_ISL_722886, EPI_ISL_722887, EPI_ISL_722888, EPI_ISL_722889, EPI_ISL_722890, EPI_ISL_722891, EPI_ISL_722892, EPI_ISL_722893, EPI_ISL_722894			
see above	Istituto Zooprofilattico Sperimentale della Puglia e della	Istituto Zooprofilattico Sperimentale della Puglia e della	Parisi A., Bianco A., Capozzi L., Del Sambro L., Manzulli V, Rondinone V., Pace L., Cipolletta D., Galante D.

	Basilicata	Basilicata	
EPI_ISL_722895	I.R.C.C.S. "S. De Bellis" - Ente Ospedaliero	Istituto Zooprofilattico Sperimentale della Puglia e della Basilicata	Parisi A., Bianco A., Capozzi L., Del Sambro L., Lippolis A., Notarnicola M., Cipolletta D., Galante D.
EPI_ISL_722896, EPI_ISL_722897	Dipartimento di Scienze Biomediche e Oncologia Umana - Azienda Ospedaliero Universitaria Consorziale Policlinico	Istituto Zooprofilattico Sperimentale della Puglia e della Basilicata	Parisi A., Bianco A., Capozzi L., Del Sambro L., Chironna M., Loconsole D.
EPI_ISL_722898	I.R.C.C.S. "S. De Bellis" - Ente Ospedaliero	Istituto Zooprofilattico Sperimentale della Puglia e della Basilicata	Parisi A., Bianco A., Capozzi L., Del Sambro L., Lippolis A., Notarnicola M., Cipolletta D., Galante D.
EPI_ISL_722899	Dipartimento di Scienze Biomediche e Oncologia Umana - Azienda Ospedaliero Universitaria Consorziale Policlinico	Istituto Zooprofilattico Sperimentale della Puglia e della Basilicata	Parisi A., Bianco A., Capozzi L., Del Sambro L., Chironna M., Loconsole D.
EPI_ISL_722900	I.R.C.C.S. "S. De Bellis" - Ente Ospedaliero	Istituto Zooprofilattico Sperimentale della Puglia e della Basilicata	Parisi A., Bianco A., Capozzi L., Del Sambro L., Lippolis A., Notarnicola M., Cipolletta D., Galante D.
EPI_ISL_722901, EPI_ISL_722902, EPI_ISL_722903, EPI_ISL_722904, EPI_ISL_722905, EPI_ISL_722906, EPI_ISL_722907, EPI_ISL_722908, EPI_ISL_722909, EPI_ISL_722910, EPI_ISL_722911			
see above	Istituto Zooprofilattico Sperimentale della Puglia e della Basilicata	Istituto Zooprofilattico Sperimentale della Puglia e della Basilicata	Parisi A., Bianco A., Capozzi L., Del Sambro L., Manzulli V, Rondinone V., Pace L., Cipolletta D., Galante D.
EPI_ISL_723047, EPI_ISL_723048, EPI_ISL_723049, EPI_ISL_723050, EPI_ISL_723051, EPI_ISL_723052, EPI_ISL_723053, EPI_ISL_723054, EPI_ISL_723055, EPI_ISL_723056, EPI_ISL_723057, EPI_ISL_723058, EPI_ISL_723059, EPI_ISL_723060, EPI_ISL_723061, EPI_ISL_723062, EPI_ISL_723063, EPI_ISL_723064, EPI_ISL_723065, EPI_ISL_723066, EPI_ISL_723067, EPI_ISL_723070, EPI_ISL_723071, EPI_ISL_723073, EPI_ISL_723074, EPI_ISL_723075, EPI_ISL_723076			
see above	Hematopathology Laboratory, ACTREC, TMC	Hematopathology Laboratory, ACTREC, TMC	Hematopathology Laboratory, ACTREC
EPI_ISL_727998, EPI_ISL_727999	Fujita Health University Okazaki Medical Center	Department of Virology and Parasitology, Fujita Health University School of Medicine	Takayuki Murata, Yohei Doi, Takuma Ishihara, Masahiro suzuki, Aki Sakurai, Satoshi Komoto, Tomihiko Ide
EPI_ISL_728000	Fujita Health University Okazaki Medical Center	Department of Virology and Parasitology, Fujita Health University School of Medicin	Takayuki Murata, Yohei Doi, Takuma Ishihara, Masahiro suzuki, Aki Sakurai, Satoshi Komoto, Tomihiko Ide
EPI_ISL_728153, EPI_ISL_728154, EPI_ISL_728155	Fujita Health University Okazaki Medical Center	Department of Virology and Parasitology, Fujita Health University School of Medicine	Takayuki Murata, Yohei Doi, Takuma Ishihara, Masahiro suzuki, Aki Sakurai, Satoshi Komoto, Tomihiko Ide
EPI_ISL_728156, EPI_ISL_728159	Fujita Health University Okazaki Medical Center	Department of Virology and Parasitology, Fujita Health University School of Medicine	Takayuki Murata, Aki Sakurai, Satoshi Komoto, Tomihiko Ide,Takuma Ishihara, Masahiro suzuki, Yohei Doi
EPI_ISL_728206, EPI_ISL_728207, EPI_ISL_728208	Fujita Health University Okazaki Medical Center	Fujita health University, Department of Microbiology	Takayuki Murata, Aki Sakurai, Satoshi Komoto, Tomihiko Ide,Takuma Ishihara, Masahiro suzuki, Yohei Doi
EPI_ISL_729797, EPI_ISL_729798, EPI_ISL_729800, EPI_ISL_729848, EPI_ISL_729849	Laboratorio Central de Saude Publica do Estado do Rio Grande do Sul (LACEN-RS)	Laboratory of Respiratory Viruses and Measles, Oswaldo Cruz Institute, FIOCRUZ	Paola Resende, Luciana Appolinario, Fernando Motta, Anna Carolina Paixão, Ana Carolina Mendonça, Tatiana Schaffer Gregianini, Marilda Tereza Mar da Rosa, Marilda Siqueira
EPI_ISL_730653	University of Bari, Valenzano, Italy	Istituto Zooprofilattico Sperimentale dell'Abruzzo e del Molise "G. Caporale".	N. Decaro, E. Lorusso, G. Elia, C. Desario, D. Buonavoglia, V., Martella, C. Buonavoglia, A. Lorusso, C. Cammà, V. Curini
EPI_ISL_732531, EPI_ISL_732532, EPI_ISL_732533, EPI_ISL_732534, EPI_ISL_732535, EPI_ISL_732536, EPI_ISL_732537, EPI_ISL_732538, EPI_ISL_732539, EPI_ISL_732540, EPI_ISL_732556, EPI_ISL_732557, EPI_ISL_732558, EPI_ISL_732559, EPI_ISL_732560			
see above	Bundeswehr Institute of Microbiology	Bundeswehr Institute of Microbiology	Elham Khatamzas, Markus Antwerpen, Mathias Walter, Alexandra Rehn, Sabine Zange, Enrico Georgi, Michael von Bergwelt-Baildon, Roman Wölfel
EPI_ISL_732563	Bundeswehr Institute of Microbiology	Bundeswehr Institute of Microbiology	Markus Antwerpen, Alexandra Rehn, Mathias Walter, Malena Bestehorn-Willmann, Sabine Zange, Enrico Georgi, Roman Wölfel
EPI_ISL_732658	Bundeswehr Institute of Microbiology	Bundeswehr Institute of Microbiology	Elham Khatamzas, Markus Antwerpen, Mathias Walter, Alexandra Rehn, Sabine Zange, Enrico Georgi, Michael von Bergwelt-Baildon, Roman Wölfel
EPI_ISL_732659, EPI_ISL_732660, EPI_ISL_732661, EPI_ISL_732662, EPI_ISL_732663, EPI_ISL_732664, EPI_ISL_732665, EPI_ISL_732666, EPI_ISL_732667, EPI_ISL_732668, EPI_ISL_732669, EPI_ISL_732670, EPI_ISL_732671, EPI_ISL_732672, EPI_ISL_732673, EPI_ISL_732674, EPI_ISL_732675, EPI_ISL_732676			
see above	Molecular diagnostic laboratory of Federal Budget Institution of Science "Central Research Institute of Epidemiology" of The Federal Service on Customers' Rights Protection and Human Well-being Surveillance	Group of Genomics and Postgenomic Technologies of Central Research Institute of Epidemilology	Samoilov AE, Kaptelova VV, Dudorova AV, Korneenko EV, Saenko SS, Speranskaya AS, Tivanova EV, Shipulina OY, Akimkin VG
EPI_ISL_732948, EPI_ISL_732949, EPI_ISL_732950, EPI_ISL_732951, EPI_ISL_732952, EPI_ISL_732953, EPI_ISL_732954, EPI_ISL_732955, EPI_ISL_732956, EPI_ISL_732957, EPI_ISL_732958, EPI_ISL_732959			
see above	Department of Tropical Parasitology	Laboratory of Recombinant Vaccines	Lukasz Rabalski, Maciej Kosinski, Teemu Smura, Kirsi Aaltonen, Ravi Kant, Tarja Sironen, Boguslaw Szewczyk, Maciej Grzybek
EPI_ISL_732971, EPI_ISL_732975, EPI_ISL_732976, EPI_ISL_732977, EPI_ISL_732978, EPI_ISL_732979, EPI_ISL_732980, EPI_ISL_732981, EPI_ISL_732982, EPI_ISL_732983, EPI_ISL_732984, EPI_ISL_732985			
see above	WHO National Influenza Centre Russian Federation	WHO National Influenza Centre Russian Federation	Andrey Komissarov, Artem Fadeev, Anna Ivanova, Kseniya Komissarova, Dmitry Bazhenov, Daria Danilenko, Ksenia Safina, Elena Nabieva, Georgii Bazykin, Dmitry Lioznov
EPI_ISL_733568	Centre for Health Protection	Hong Kong Department of Health	Alan K.L. Tsang, Peter C.W. Yip, Edman T.K. Lam, Rickjason C.W. Chan, Dominic N.C. Tsang
EPI_ISL_733569	CHAI WAN FAMILIES CLINIC	Hong Kong Department of Health	Alan K.L. Tsang, Peter C.W. Yip, Edman T.K. Lam, Rickjason C.W. Chan, Dominic N.C. Tsang
EPI_ISL_733570	POK OI HOSPITAL	Hong Kong Department of Health	Alan K.L. Tsang, Peter C.W. Yip, Edman T.K. Lam, Rickjason C.W. Chan, Dominic N.C. Tsang
EPI_ISL_733571	Queen Mary Hospital	Hong Kong Department of Health	Alan K.L. Tsang, Peter C.W. Yip, Edman T.K. Lam, Rickjason C.W. Chan, Dominic N.C. Tsang
EPI_ISL_733572, EPI_ISL_733573, EPI_ISL_733574, EPI_ISL_733575, EPI_ISL_733576, EPI_ISL_733577, EPI_ISL_733578, EPI_ISL_733579	Temporary Specimen Collection Centre	Hong Kong Department of Health	Alan K.L. Tsang, Peter C.W. Yip, Edman T.K. Lam, Rickjason C.W. Chan, Dominic N.C. Tsang
EPI_ISL_734167, EPI_ISL_734168, EPI_ISL_734169	CHRU Pontchaillou - Laboratoire de Virologie	National Reference Center for Viruses of Respiratory Infections, Institut Pasteur, Paris	Marion Barbet, Sylvie Behillil, Méline Bizard, Angela Brisebarre, Camille Capel, Etienne Simon-Lorière, Vincent Enouf, Maud Vanpeene, Sylvie van der Werf, Gisèle Lagathu
EPI_ISL_735259, EPI_ISL_735260, EPI_ISL_735261, EPI_ISL_735262, EPI_ISL_735263, EPI_ISL_735264, EPI_ISL_735265, EPI_ISL_735266, EPI_ISL_735267, EPI_ISL_735268, EPI_ISL_735269, EPI_ISL_735270, EPI_ISL_735271, EPI_ISL_735272, EPI_ISL_735273, EPI_ISL_735274, EPI_ISL_735275, EPI_ISL_735276, EPI_ISL_735277, EPI_ISL_735278, EPI_ISL_735279, EPI_ISL_735280, EPI_ISL_735281, EPI_ISL_735282, EPI_ISL_735283, EPI_ISL_735284, EPI_ISL_735285, EPI_ISL_735286, EPI_ISL_735287, EPI_ISL_735288, EPI_ISL_735289, EPI_ISL_735290, EPI_ISL_735291, EPI_ISL_735292, EPI_ISL_735293, EPI_ISL_735294, EPI_ISL_735295, EPI_ISL_735296, EPI_ISL_735297, EPI_ISL_735298, EPI_ISL_735299, EPI_ISL_735300, EPI_ISL_735301, EPI_ISL_735302, EPI_ISL_735303, EPI_ISL_735304, EPI_ISL_735305, EPI_ISL_735306, EPI_ISL_735307, EPI_ISL_735308, EPI_ISL_735309, EPI_ISL_735310, EPI_ISL_735311, EPI_ISL_735312, EPI_ISL_735313, EPI_ISL_735314, EPI_ISL_735315, EPI_ISL_735316, EPI_ISL_735317, EPI_ISL_735318, EPI_ISL_735319, EPI_ISL_735320, EPI_ISL_735321, EPI_ISL_735322, EPI_ISL_735323, EPI_ISL_735324, EPI_ISL_735325, EPI_ISL_735326, EPI_ISL_735327, EPI_ISL_735328, EPI_ISL_735329, EPI_ISL_735330, EPI_ISL_735331, EPI_ISL_735332, EPI_ISL_735333, EPI_ISL_735334, EPI_ISL_735335, EPI_ISL_735336, EPI_ISL_735337, EPI_ISL_735338, EPI_ISL_735339, EPI_ISL_735340, EPI_ISL_735341, EPI_ISL_735342, EPI_ISL_735343, EPI_ISL_735344, EPI_ISL_735345, EPI_ISL_735346, EPI_ISL_735347, EPI_ISL_735348, EPI_ISL_735349, EPI_ISL_735350, EPI_ISL_735351, EPI_ISL_735352, EPI_ISL_735353, EPI_ISL_735354, EPI_ISL_735355, EPI_ISL_735356, EPI_ISL_735357, EPI_ISL_735358, EPI_ISL_735359, EPI_ISL_735360, EPI_ISL_735361, EPI_ISL_735362, EPI_ISL_735363, EPI_ISL_735364, EPI_ISL_735365			
see above	Genomic Laboratory (GLAB) (Conjoint lab of Health Directorate of Istanbul and Istanbul Technical University)	Genomic Laboratory (GLAB), Istanbul Technical University	Ilker Karacan, Tugba Kizilboga Akgun, Nihat Bugra Agaoglu, Payam Zolfagharian, Mehtap Aydin, Gizem Alkurt, Jale Yildiz, Betsi Kose, Nisan Denizce Can, Ayse Serra Ozel, Nilsun Altunal, Arzu Irvem, Yasemin Kendir Demirkol, Ozlem Akgun Dogan, Levent Doganay, Gizem Dinler Doganay
EPI_ISL_735396	Hospital de Camplanha COVID 19 SER	Instituto Adolfo Lutz, Interdiciplinary Procedures Center, Strategic Laboratory	Claudio Tavares Sacchi, Claudia Regina Gonçalves, Erica Valessa Ramos Gomes, Karoline Rodrigues Campos
EPI_ISL_735397	Unidade Respiratória Nova Hortolandia	Instituto Adolfo Lutz, Interdiciplinary Procedures Center, Strategic Laboratory	Claudio Tavares Sacchi, Claudia Regina Gonçalves, Erica Valessa Ramos Gomes, Karoline Rodrigues Campos
EPI_ISL_735398	Laboratorio Fleury	Instituto Adolfo Lutz, Interdiciplinary Procedures Center,	Claudio Tavares Sacchi, Claudia Regina Gonçalves, Erica Valessa Ramos Gomes, Karoline Rodrigues Campos

		Strategic Laboratory	
EPI_ISL_735399	Hospital Municiplal Dr Ignacio de gouvea	Instituto Adolfo Lutz, Interdiciplinary Procedures Center, Strategic Laboratory	Claudio Tavares Sacchi, Claudia Regina Gonçalves, Erica Valessa Ramos Gomes, Karoline Rodrigues Campos
EPI_ISL_735400	Instituto Adolfo Lutz - Regional de Santos	Instituto Adolfo Lutz, Interdiciplinary Procedures Center, Strategic Laboratory	Claudio Tavares Sacchi, Claudia Regina Gonçalves, Erica Valessa Ramos Gomes, Karoline Rodrigues Campos
EPI_ISL_735401, EPI_ISL_735402, EPI_ISL_735403, EPI_ISL_735404	Instituto Adolfo Lutz - Regional de Rio Claro	Instituto Adolfo Lutz, Interdiciplinary Procedures Center, Strategic Laboratory	Claudio Tavares Sacchi, Claudia Regina Gonçalves, Erica Valessa Ramos Gomes, Karoline Rodrigues Campos
EPI_ISL_735405	Secretaria Minucipal de Saude de Birigui	Instituto Adolfo Lutz, Interdiciplinary Procedures Center, Strategic Laboratory	Claudio Tavares Sacchi, Claudia Regina Gonçalves, Erica Valessa Ramos Gomes, Karoline Rodrigues Campos
EPI_ISL_735406	Unidade de Pronto Atendimento UPA I Sta Isabel	Instituto Adolfo Lutz, Interdiciplinary Procedures Center, Strategic Laboratory	Claudio Tavares Sacchi, Claudia Regina Gonçalves, Erica Valessa Ramos Gomes, Karoline Rodrigues Campos
EPI_ISL_735407	Santa Casa de Marilia	Instituto Adolfo Lutz, Interdiciplinary Procedures Center, Strategic Laboratory	Claudio Tavares Sacchi, Claudia Regina Gonçalves, Erica Valessa Ramos Gomes, Karoline Rodrigues Campos
EPI_ISL_735408	COVID 19 Centro de Combate ao Coronavirus CCC Jandira	Instituto Adolfo Lutz, Interdiciplinary Procedures Center, Strategic Laboratory	Claudio Tavares Sacchi, Claudia Regina Gonçalves, Erica Valessa Ramos Gomes, Karoline Rodrigues Campos
EPI_ISL_735409	Unidade de Pronto Atendimento Carlos Lourenco	Instituto Adolfo Lutz, Interdiciplinary Procedures Center, Strategic Laboratory	Claudio Tavares Sacchi, Claudia Regina Gonçalves, Erica Valessa Ramos Gomes, Karoline Rodrigues Campos
EPI_ISL_735410	Instituto Adolfo Lutz - Regional de Rio Claro	Instituto Adolfo Lutz, Interdiciplinary Procedures Center, Strategic Laboratory	Claudio Tavares Sacchi, Claudia Regina Gonçalves, Erica Valessa Ramos Gomes, Karoline Rodrigues Campos
EPI_ISL_735411	Centro de Vigilancia a Saude de Diadema	Instituto Adolfo Lutz, Interdiciplinary Procedures Center, Strategic Laboratory	Claudio Tavares Sacchi, Claudia Regina Gonçalves, Erica Valessa Ramos Gomes, Karoline Rodrigues Campos
EPI_ISL_735412	Hospital e Pronto Socorro Portinari	Instituto Adolfo Lutz, Interdiciplinary Procedures Center, Strategic Laboratory	Claudio Tavares Sacchi, Claudia Regina Gonçalves, Erica Valessa Ramos Gomes, Karoline Rodrigues Campos
EPI_ISL_735413	Militello Centro de Diagnosticos e Biopesquisa Clinica	Instituto Adolfo Lutz, Interdiciplinary Procedures Center, Strategic Laboratory	Claudio Tavares Sacchi, Claudia Regina Gonçalves, Erica Valessa Ramos Gomes, Karoline Rodrigues Campos
EPI_ISL_735414, EPI_ISL_735415	Unidade de Pronto Atendimento de Agenor de Campos	Instituto Adolfo Lutz, Interdiciplinary Procedures Center, Strategic Laboratory	Claudio Tavares Sacchi, Claudia Regina Gonçalves, Erica Valessa Ramos Gomes, Karoline Rodrigues Campos
EPI_ISL_735416	Centro de Saude II Dr Jose Paione Mococa	Instituto Adolfo Lutz, Interdiciplinary Procedures Center, Strategic Laboratory	Claudio Tavares Sacchi, Claudia Regina Gonçalves, Erica Valessa Ramos Gomes, Karoline Rodrigues Campos
EPI_ISL_735417	Unidade de Pronto Atendimento de Agenor de Campos	Instituto Adolfo Lutz, Interdiciplinary Procedures Center, Strategic Laboratory	Claudio Tavares Sacchi, Claudia Regina Gonçalves, Erica Valessa Ramos Gomes, Karoline Rodrigues Campos
EPI_ISL_735418	Hospital Regional do Vale do Paraiba	Instituto Adolfo Lutz, Interdiciplinary Procedures Center, Strategic Laboratory	Claudio Tavares Sacchi, Claudia Regina Gonçalves, Erica Valessa Ramos Gomes, Karoline Rodrigues Campos
EPI_ISL_735419	UBS Alvarenga	Instituto Adolfo Lutz, Interdiciplinary Procedures Center, Strategic Laboratory	Claudio Tavares Sacchi, Claudia Regina Gonçalves, Erica Valessa Ramos Gomes, Karoline Rodrigues Campos
EPI_ISL_735420	UBS Riacho Grande	Instituto Adolfo Lutz, Interdiciplinary Procedures Center, Strategic Laboratory	Claudio Tavares Sacchi, Claudia Regina Gonçalves, Erica Valessa Ramos Gomes, Karoline Rodrigues Campos
EPI_ISL_735421	UBS Sta Terezinha	Instituto Adolfo Lutz, Interdiciplinary Procedures Center, Strategic Laboratory	Claudio Tavares Sacchi, Claudia Regina Gonçalves, Erica Valessa Ramos Gomes, Karoline Rodrigues Campos
EPI_ISL_735422	UBS Dematchi	Instituto Adolfo Lutz, Interdiciplinary Procedures Center, Strategic Laboratory	Claudio Tavares Sacchi, Claudia Regina Gonçalves, Erica Valessa Ramos Gomes, Karoline Rodrigues Campos
EPI_ISL_735423, EPI_ISL_735424	Centro de Vigilancia a Saude de Diadema	Instituto Adolfo Lutz, Interdiciplinary Procedures Center, Strategic Laboratory	Claudio Tavares Sacchi, Claudia Regina Gonçalves, Erica Valessa Ramos Gomes, Karoline Rodrigues Campos
EPI_ISL_735425	Hospital e Maternidade Sao Lucas	Instituto Adolfo Lutz, Interdiciplinary Procedures Center, Strategic Laboratory	Claudio Tavares Sacchi, Claudia Regina Gonçalves, Erica Valessa Ramos Gomes, Karoline Rodrigues Campos
EPI_ISL_735426	Centro de Vigilancia a Saude de Diadema	Instituto Adolfo Lutz, Interdiciplinary Procedures Center, Strategic Laboratory	Claudio Tavares Sacchi, Claudia Regina Gonçalves, Erica Valessa Ramos Gomes, Karoline Rodrigues Campos
EPI_ISL_735427	Instituto Adolfo Lutz - Regional de Santos	Instituto Adolfo Lutz, Interdiciplinary Procedures Center, Strategic Laboratory	Claudio Tavares Sacchi, Claudia Regina Gonçalves, Erica Valessa Ramos Gomes, Karoline Rodrigues Campos
EPI_ISL_735428, EPI_ISL_735429	Hospital Nipo Brasileiro	Instituto Adolfo Lutz, Interdiciplinary Procedures Center, Strategic Laboratory	Claudio Tavares Sacchi, Claudia Regina Gonçalves, Erica Valessa Ramos Gomes, Karoline Rodrigues Campos
EPI_ISL_735430	Instituto Adolfo Lutz - Regional de Santos	Instituto Adolfo Lutz, Interdiciplinary Procedures Center, Strategic Laboratory	Claudio Tavares Sacchi, Claudia Regina Gonçalves, Erica Valessa Ramos Gomes, Karoline Rodrigues Campos
EPI_ISL_735431, EPI_ISL_735432	Hospital Nipo Brasileiro	Instituto Adolfo Lutz, Interdiciplinary Procedures Center, Strategic Laboratory	Claudio Tavares Sacchi, Claudia Regina Gonçalves, Erica Valessa Ramos Gomes, Karoline Rodrigues Campos
EPI_ISL_735433	Posto de Atendimento Saude Cidade Pasc Cajati	Instituto Adolfo Lutz, Interdiciplinary Procedures Center, Strategic Laboratory	Claudio Tavares Sacchi, Claudia Regina Gonçalves, Erica Valessa Ramos Gomes, Karoline Rodrigues Campos
EPI_ISL_735434	Genomic Laboratory (GLAB) (Conjoint lab of Health Directorate of Istanbul and Istanbul Technical University)	Genomic Laboratory (GLAB), Istanbul Technical University	Ilker Karacan, Tugba Kizilboga Akgun, Nihat Bugra Agaoglu, Payam Zolfagharian, Mehtap Aydin, Gizem Alkurt, Jale Yildiz, Betsi Kose, Nisan Denizce Can, Ayse Serra Ozel, Nilsun Altunal, Arzu Irvem, Yasemin Kendir Demirkol, Ozlem Akgun Dogan, Levent Doganay, Gizem Dinler Doganay
EPI_ISL_735436	Nucleic Acid Testing - Rwanda National Reference Laboratory	GIGA Medical Genomics	Yvan Butera,Keith Durkin, Maria Artesi, Bouchra Boujemla, Robert Rutayisire, Patrick Tuyisenge, Esperence Umumararungu, Sébastien Bontems, Marie-Pierre Hayette, Swaibu Gatare, Jacob Souopgui, Sabin Nsanzimana, Vincent Bours, Léon Mutesa
EPI_ISL_735437	Nucleic Acid Testing - Rwanda National Reference Laboratory	GIGA Medical Genomics	Yvan Butera, Keith Durkin, Maria Artesi, Bouchra Boujemla, Robert Rutayisire, Patrick Tuyisenge, Esperence Umumararungu, Sébastien Bontems, Marie-Pierre Hayette, Swaibu Gatare, Jacob Souopgui, Sabin Nsanzimana, Vincent Bours, Léon Mutesa
EPI_ISL_735438	Nuclei Acid Testing - Rwanda National Reference Laboratory	GIGA Medical Genomics	Yvan Butera, Keith Durkin, Maria Artesi, Bouchra Boujemla, Robert Rutayisire, Patrick Tuyisenge, Esperence Umumararungu, Sébastien Bontems, Marie-Pierre Hayette, Swaibu Gatare, Jacob Souopgui, Sabin Nsanzimana, Vincent Bours, Léon Mutesa
EPI_ISL_735444	Nucleic Acid Testing - Rwanda National Reference Laboratory	GIGA Medical Genomics	Yvan Butera, Keith Durkin, Maria Artesi, Bouchra Boujemla, Robert Rutayisire, Patrick Tuyisenge, Esperence Umumararungu, Sébastien Bontems, Marie-Pierre Hayette, Swaibu Gatare, Jacob Souopgui, Sabin Nsanzimana, Vincent Bours, Léon Mutesa
EPI_ISL_735445	Rwanda National Reference Laboratory	GIGA Medical Genomics	Yvan Butera, Keith Durkin, Maria Artesi, Bouchra Boujemla, Robert Rutayisire, Patrick Tuyisenge, Esperence Umumararungu, Sébastien Bontems, Marie-Pierre Hayette, Swaibu Gatare, Jacob Souopgui, Sabin Nsanzimana, Vincent Bours, Léon Mutesa
EPI_ISL_735446, EPI_ISL_735447,	Nucleic Acid Testing - Rwanda National Reference Laboratory	GIGA Medical Genomics	Yvan Butera, Keith Durkin, Maria Artesi, Bouchra Boujemla, Robert Rutayisire, Patrick Tuyisenge, Esperence Umumararungu, Sébastien Bontems,

EPI_ISL_735448			Marie-Pierre Hayette, Swaibu Gatare, Jacob Souopgui, Sabin Nsanzimana, Vincent Bours, Léon Mutesa
EPI_ISL_735504	University of Bari Biomedical Sciences and Human Oncology	University of Bari Biomedical Sciences and Human Oncology	Maria Chironna, Anna Sallustio, Daniela Loconsole
EPI_ISL_735505, EPI_ISL_735506	University of Bari Biomedical Sciences and Human Oncology	University of Bari Biomedical Sciences and Human Oncology	Maria Chironna, Anna Sallustio, Daniela Loconsole, Marisa Accogli
EPI_ISL_736996	Istituto Zooprofilattico Sperimentale del Mezzogiorno	National Institute for Infectious Diseases, INMI, "L. Spallanzani" IRCCS	E. Giombini, C.E.M Gruber, F. Messina, M. Rueca, B. Bartolini, F. Carletti, P. Cerino, B. Pierri, C. Buonerba, D. Di Concilio, M.C. Cuomo, A. Di Caro, M.R Capobianchi
EPI_ISL_736997	Istituto Zooprofilattico Sperimentale del Mezzogiorno	National Institute for Infectious Diseases, INMI, "L. Spallanzani" IRCCS	F. Messina, C.E.M Gruber, M. Rueca, B. Bartolini, E. Giombini, F. Carletti, P. Cerino, B. Pierri, C. Buonerba, D. Di Concilio, M.C. Cuomo, M.R. Capobianchi, A. Di Caro
EPI_ISL_736998, EPI_ISL_737002, EPI_ISL_737003, EPI_ISL_737008, EPI_ISL_737009, EPI_ISL_737010, EPI_ISL_737011, EPI_ISL_737012, EPI_ISL_737013, EPI_ISL_737014, EPI_ISL_737015, EPI_ISL_737016, EPI_ISL_737017, EPI_ISL_737018, EPI_ISL_737019, EPI_ISL_737021, EPI_ISL_737022, EPI_ISL_737023, EPI_ISL_737024, EPI_ISL_737025, EPI_ISL_737026, EPI_ISL_737027, EPI_ISL_737028, EPI_ISL_737029, EPI_ISL_737031, EPI_ISL_737032, EPI_ISL_737033			
see above	The National Institute of Public Health	State Veterinary Institute Prague	Nagy,A;Jirincova,H;Trnka,D;Vecerova,J
EPI_ISL_737200	National Reference Laboratory, Nigeria Centre for Disease Control.	National Reference Laboratory, Nigeria Centre for Disease Control, Gaduwa,Abuja, Nigeria	Dr Ndodo Nnaemeka, Olusola Akanbi, Chimaobi Chukwu, Dr Adesuyi Omoare, Nwando Mba, Shirlee Wohl, Dr Chikwe Ihekweazu
EPI_ISL_737201	National Reference Laboratory, Nigeria Centre for Disease Control.	National Reference Laboratory, Nigeria Centre for Disease Control, Gaduwa,Abuja, Nigeria	Dr Ndodo Nnaemeka, Olusola Akanbi, Chimaobi Chukwu, Dr Adesuyi Omoare, oluwaseyi Ajegbe, Grace Esebanmen, Nwando Mba, Shirlee Wohl, Anthony Ahumibe, Celestina Obiekea, Catherine Okoi, Akinpelu Afolabi, Kingsley Njoku, Dr Sikiru Badaru, Dr Chinwe Ochu, Dr Chikwe Ihekweazu
EPI_ISL_737205	National Reference Laboratory, Nigeria Centre for Disease Control.	National Reference Laboratory, Nigeria Centre for Disease Control, Gaduwa,Abuja, Nigeria	Dr Ndodo Nnaemeka, Olusola Akanbi, Chimaobi Chukwu, Dr Adesuyi Omoare, Nwando Mba, Shirlee Wohl, (JHU), Anthony Ahumibe, Celestina Obiekea, Catherine Okoi, Peter Van Heusden (SANBI), Dr Chikwe Ihekweazu
EPI_ISL_737206	National Reference Laboratory, Nigeria Centre for Disease Control.	National Reference Laboratory, Nigeria Centre for Disease Control, Gaduwa,Abuja, Nigeria	Dr Ndodo Nnaemeka, Olusola Akanbi, Chimaobi Chukwu, Dr Adesuyi Omoare, Grace Esebanmen, Nwando Mba, Shirlee Wohl, Anthony Ahumibe, Celestina Obiekea, Catherine Okoi, Dr Chinwe Ochu, Dr Chikwe Ihekweazu.
EPI_ISL_737207	National Reference Laboratory, Nigeria Centre for Disease Control.	National Reference Laboratory, Nigeria Centre for Disease Control, Gaduwa,Abuja, Nigeria	Dr Ndodo Nnaemeka, Olusola Akanbi, Chimaobi Chukwu, Dr Adesuyi Omoare, Shirlee Wohl, Anthony Ahumibe, Abdulmajid Musa, Nneamaka Uba, Bamidele Olorunfemi, Kingsley Madubuike, Dr Sikiru Badaru, Adama Ahmad, Michael Popoola, Dr Chikwe Ihekweazu
EPI_ISL_738044	SIESP CHIETI - DRIVE IN LANCIANO	Istituto Zooprofilattico Sperimentale dell'Abruzzo e Molise "G. Caporale"	Lorusso A, Marcacci M, Di Domenico M, Ancora M, Curini V, Mangone I, Rinaldi A, Di Pasquale A, Cammà C, Puglia I, Savini G
EPI_ISL_738045	SIESP DIPARTIMENTO DI PREVENZIONE CHIETI	Istituto Zooprofilattico Sperimentale dell'Abruzzo e Molise "G. Caporale"	Lorusso A, Marcacci M, Di Domenico M, Ancora M, Curini V, Mangone I, Rinaldi A, Di Pasquale A, Cammà C, Puglia I, Savini G
EPI_ISL_738046, EPI_ISL_738047, EPI_ISL_738048	SIESP CHIETI - DRIVE IN ORTONA	Istituto Zooprofilattico Sperimentale dell'Abruzzo e Molise "G. Caporale"	Lorusso A, Marcacci M, Di Domenico M, Ancora M, Curini V, Mangone I, Rinaldi A, Di Pasquale A, Cammà C, Puglia I, Savini G
EPI_ISL_738064, EPI_ISL_738065	Department of Laboratory Medicine, National Taiwan University Hospital	Microbial Genomics Core Lab, National Taiwan University Centers of Genomic and Precision Medicine	Shiou-Hwei Yeh, You-Yu Lin, Ya-Yun Lai, Chiao-Ling Li, Shan-Chwen Chang, Pei-Jer Chen, Sui-Yuan Chang
EPI_ISL_738121, EPI_ISL_738122, EPI_ISL_738123, EPI_ISL_738124, EPI_ISL_738125, EPI_ISL_738126, EPI_ISL_738127, EPI_ISL_738128, EPI_ISL_738129, EPI_ISL_738130	IZSM-U.O.C. Virologia	Istituto Zooprofilattico Sperimentale del Mezzogiorno	Maurizio Viscardi, Lorena Cardillo, Giovanna Fusco
EPI_ISL_738144	University of Bari Biomedical Sciences and Human Oncology	University of Bari Biomedical Sciences and Human Oncology	Maria Chironna, Anna Sallustio, Daniela Loconsole, Marisa Accogli
EPI_ISL_738147, EPI_ISL_738194, EPI_ISL_738243	Microbiology and Virology Unit, Florence Careggi University Hospital	Microbiology and Virology Unit, Florence Careggi University Hospital	Vincenzo Di Pilato, Marco Coppi, Alberto Antonelli, Simona Pollini, Gian Maria Rossolini
EPI_ISL_739659, EPI_ISL_739660, EPI_ISL_739661	Al-Quds Nutrition and Health Research Institute, Al-Quds University	Al-Quds Nutrition and Health Research Institute, Al-Quds University	Nasereddin,A., Ereqat,S., Al-Jawabreh,A., Rishmawi,C.

Supplementary Table 2: Sequence metadata

Virus name	Accession ID	Collection date	Location	Host	Additional location information	Gender	Patient age	Patient status	Passage	Specimen	Additional host information	Lineage	GISAID haplogroup	Country	Patient status category (Pre-symptomatic / asymptomatic VS Symptomatic)	Included in the dataset?
hCoV-19/Shenzhen/SZTH-001/2020	EPI_ISL_406592	13/01/2020	Asia / China / Guangdong / Shenzhen	Human		female	65	hospitalized	Original	Alveolar lavage fluid		A	O	China	Undertermined	No (couldn't determine the patient status as either pre-symptomatic / asymptomatic or symptomatic conclusively)
hCoV-19/Shenzhen/SZTH-002/2020	EPI_ISL_406593	13/01/2020	Asia / China / Guangdong / Shenzhen	Human		male	66	hospitalized	Original	Alveolar lavage fluid		A	S	China	Undertermined	No (couldn't determine the patient status as either pre-symptomatic / asymptomatic or symptomatic conclusively)
hCoV-19/Shenzhen/SZTH-003/2020	EPI_ISL_406594	16/01/2020	Asia / China / Guangdong / Shenzhen	Human		male	62	hospitalized	Original	Alveolar lavage fluid		B	L	China	Undertermined	No (couldn't determine the patient status as either pre-symptomatic / asymptomatic or symptomatic conclusively)
hCoV-19/Shenzhen/SZTH-004/2020	EPI_ISL_406595	16/01/2020	Asia / China / Guangdong / Shenzhen	Human		male	63	hospitalized	Original	Alveolar lavage fluid		B	L	China	Undertermined	No (couldn't determine the patient status as either pre-symptomatic / asymptomatic or symptomatic conclusively)
hCoV-19/Australia/NSW01/2020	EPI_ISL_407893	24/01/2020	Oceania / Australia / New South Wales / Sydney	Human		Male	43	Recovering	Original	Nasopharyngeal swab		A	S	Australia	Undertermined	No (couldn't determine the patient status as either pre-symptomatic / asymptomatic or symptomatic conclusively)
hCoV-19/Australia/QLD02/2020	EPI_ISL_407896	30/01/2020	Oceania / Australia / Queensland / Gold Coast	Human		Female	42	Unkown	Original	Aspirate		A	S	Australia	Undertermined	No (couldn't determine the patient status as either pre-symptomatic / asymptomatic or symptomatic conclusively)
hCoV-19/India/MH-1-27/2020	EPI_ISL_413522	27/01/2020	Asia / India / Maharashtra	Human	Travel history to Wuhan, China	Female	20	Recovered	Original	Throat swab		B	L	India	Undertermined	No (couldn't determine the patient status as either pre-symptomatic / asymptomatic or symptomatic conclusively)
hCoV-19/India/MH-1-31/2020	EPI_ISL_413523	31/01/2020	Asia / India / Maharashtra	Human	Travel history to China	Male	23	Recovered	Original	Throat Swab		A	S	India	Undertermined	No (couldn't determine the patient status as either pre-symptomatic / asymptomatic or symptomatic conclusively)
hCoV-19/Nigeria/Lagos01/2020	EPI_ISL_413550	27/02/2020	Africa / Nigeria / Lagos	Human		Male	44-year old	Hospitalized	Original	Sputum	Patient infected in Italy	B.1.1	GR	Nigeria	Undertermined	No (couldn't determine the patient status as either pre-symptomatic / asymptomatic or symptomatic conclusively)
hCoV-19/Australia/NSW08/2020	EPI_ISL_413594	28/02/2020	Oceania / Australia / New South Wales / Sydney	Human		Male	53	Recovering	Original	Oro-pharyngeal swab		B	O	Australia	Undertermined	No (couldn't determine the patient status as either pre-symptomatic / asymptomatic or symptomatic conclusively)
hCoV-19/Australia/NSW09/2020	EPI_ISL_413595	28/02/2020	Oceania / Australia / New South Wales / Sydney	Human		Female	60	Recovering	Original	Nasopharyngeal aspirate		B.4	O	Australia	Undertermined	No (couldn't determine the patient status as either pre-symptomatic / asymptomatic or symptomatic conclusively)
hCoV-19/Australia/NSW10/2020	EPI_ISL_413596	28/02/2020	Oceania / Australia / New South Wales / Sydney	Human		Female	62	Recovering	Original	Nasopharyngeal aspirate		B	O	Australia	Undertermined	No (couldn't determine the patient status as either pre-symptomatic / asymptomatic or symptomatic conclusively)
hCoV-19/Australia/NSW11/2020	EPI_ISL_413597	02/03/2020	Oceania / Australia / New South Wales / Sydney	Human		Male	38	Recovering	Original	Oro-pharyngeal swab		B.4	O	Australia	Undertermined	No (couldn't determine the patient status as either pre-symptomatic / asymptomatic or symptomatic conclusively)
hCoV-19/Australia/NSW12/2020	EPI_ISL_413598	04/03/2020	Oceania / Australia / New South Wales / Sydney	Human		Male	31	Recovering	Original	Oro-pharyngeal swab		B.4	O	Australia	Undertermined	No (couldn't determine the patient status as either pre-symptomatic / asymptomatic or symptomatic conclusively)
hCoV-19/Australia/NSW13/2020	EPI_ISL_413599	04/03/2020	Oceania / Australia / New South Wales / Sydney	Human		Male	81	Recovering	Original	Oro-pharyngeal swab		B.4	O	Australia	Undertermined	No (couldn't determine the patient status as either pre-symptomatic / asymptomatic or symptomatic conclusively)
hCoV-19/Australia/NSW14/2020	EPI_ISL_413600	03/03/2020	Oceania / Australia / New South Wales / Sydney	Human		Female	41	Recovering	Original	Sputum		B.4	O	Australia	Undertermined	No (couldn't determine the patient status as either pre-symptomatic / asymptomatic or symptomatic conclusively)
hCoV-19/Portugal/CV62/2020	EPI_ISL_413647	01/03/2020	Europe / Portugal	Human		Male	60	Hospitalized	Original	Naso-Oropharyngeal swab		B.1.1	GR	Portugal	Undertermined	No (couldn't determine the patient status as either pre-symptomatic / asymptomatic or symptomatic conclusively)
hCoV-19/Portugal/CV63/2020	EPI_ISL_413648	01/03/2020	Europe / Portugal	Human		Male	33	Hospitalized	Original	Naso-Oropharyngeal swab		B.1.74	G	Portugal	Undertermined	No (couldn't determine the patient status as either pre-symptomatic / asymptomatic or symptomatic conclusively)
hCoV-19/Hong Kong/HKPU1_2101/2020	EPI_ISL_417176	21/01/2020	Asia / Hong Kong	Human		Male	39	Discharged	Original			A	S	Hong Kong	Undertermined	No (couldn't determine the patient status as either pre-symptomatic / asymptomatic or symptomatic conclusively)
hCoV-19/Hong Kong/HKPU2_1801/2020	EPI_ISL_417177	23/01/2020	Asia / Hong Kong	Human		Male	56	Discharged	Original			B.2	O	Hong Kong	Undertermined	No (couldn't determine the patient status as either pre-symptomatic / asymptomatic or symptomatic conclusively)
hCoV-19/Hong Kong/HKPU12_2201/2020	EPI_ISL_417179	30/01/2020	Asia / Hong Kong	Human		Male	75	Hospitalized; Stable	Original			B.2	O	Hong Kong	Undertermined	No (couldn't determine the patient status as either pre-symptomatic / asymptomatic or symptomatic conclusively)
hCoV-19/Hong Kong/HKPU19_0402/2020	EPI_ISL_417181	05/02/2020	Asia / Hong Kong	Human		Female	28	Discharged	Original			B.2	O	Hong Kong	Undertermined	No (couldn't determine the patient status as either pre-symptomatic / asymptomatic or symptomatic conclusively)
hCoV-19/Hong Kong/HKPU20_3001/2020	EPI_ISL_417182	05/02/2020	Asia / Hong Kong	Human		Female	56	Discharged	Original			B.2	O	Hong Kong	Undertermined	No (couldn't determine the patient status as either pre-symptomatic / asymptomatic or symptomatic conclusively)
hCoV-19/Hong Kong/HKPU23_2601/2020	EPI_ISL_417183	30/01/2020	Asia / Hong Kong	Human		Female	63	Discharged	Original			B.2.8	O	Hong Kong	Undertermined	No (couldn't determine the patient status as either pre-symptomatic / asymptomatic or symptomatic conclusively)
hCoV-19/Hong Kong/HKPU27_3001/2020	EPI_ISL_417184	08/02/2020	Asia / Hong Kong	Human		Male	24	Discharged	Original			B.2.8	O	Hong Kong	Undertermined	No (couldn't determine the patient status as either pre-symptomatic / asymptomatic or symptomatic conclusively)
hCoV-19/Hong Kong/HKPU28_3001/2020	EPI_ISL_417185	09/02/2020	Asia / Hong Kong	Human		Male	70	Discharged	Original			B.2	O	Hong Kong	Undertermined	No (couldn't determine the patient status as either pre-symptomatic / asymptomatic or symptomatic conclusively)
hCoV-19/South Africa/R03006/2020	EPI_ISL_417186	07/03/2020	Africa / South Africa / KZN	Human		Male	38	Alive	Original	Combined nasopharyngeal and oropharyngeal swab		B.1	G	South Africa	Undertermined	No (couldn't determine the patient status as either pre-symptomatic / asymptomatic or symptomatic conclusively)
hCoV-19/Hong Kong/HKPU29_0102/2020	EPI_ISL_417187	08/02/2020	Asia / Hong Kong	Human		Female	91	Hospitalized; Stable	Original			B.2.8	O	Hong Kong	Undertermined	No (couldn't determine the patient status as either pre-symptomatic / asymptomatic or symptomatic conclusively)
hCoV-19/Hong Kong/HKPU30_2901/2020	EPI_ISL_417188	08/02/2020	Asia / Hong Kong	Human		Male	68	Discharged	Original			B.2.8	O	Hong Kong	Undertermined	No (couldn't determine the patient status as either pre-symptomatic / asymptomatic or symptomatic conclusively)
hCoV-19/Hong Kong/HKPU_2801/2020	EPI_ISL_417190	08/02/2020	Asia / Hong Kong	Human		Female	57	Discharged	Original			B.2.8	O	Hong Kong	Undertermined	No (couldn't determine the patient status as either pre-symptomatic / asymptomatic or symptomatic conclusively)
hCoV-19/Hong Kong/HKPU32_0402/2020	EPI_ISL_417193	09/02/2020	Asia / Hong Kong	Human		Male	22	Discharged	Original			B.2.8	O	Hong Kong	Undertermined	No (couldn't determine the patient status as either pre-symptomatic / asymptomatic or symptomatic conclusively)
hCoV-19/Hong Kong/HKPU33_0202/2020	EPI_ISL_417195	09/02/2020	Asia / Hong Kong	Human		Female	50	Discharged	Original			B.2.8	O	Hong Kong	Undertermined	No (couldn't determine the patient status as either pre-symptomatic / asymptomatic or symptomatic conclusively)
hCoV-19/France/HF1645/2020	EPI_ISL_418220	28/02/2020	Europe / France / Hauts de France / CompiÃ¨gne	Human		Male	54	Hospitalized	Original	Oropharyngeal swab		B.1	GH	France	Undertermined	No (couldn't determine the patient status as either pre-symptomatic / asymptomatic or symptomatic conclusively)
hCoV-19/France/HF1813/2020	EPI_ISL_418221	02/03/2020	Europe / France / Hauts de France / CompiÃ¨gne	Human		Male	86	Hospitalized	Original	Oropharyngeal swab		B.1	GH	France	Undertermined	No (couldn't determine the patient status as either pre-symptomatic / asymptomatic or symptomatic conclusively)
hCoV-19/France/CVL2000/2020	EPI_ISL_418222	04/03/2020	Europe / France / Centre-Val de Loire / Tours	Human		Female	73	Hospitalized	Original	Oropharyngeal swab		B.1	G	France	Undertermined	No (couldn't determine the patient status as either pre-symptomatic / asymptomatic or symptomatic conclusively)
hCoV-19/France/HF2060/2020	EPI_ISL_418223	05/03/2020	Europe / France / Hauts de France / CompiÃ¨gne	Human		Female	87	Hospitalized	Original	Oropharyngeal swab		B.1	GH	France	Undertermined	No (couldn't determine the patient status as either pre-symptomatic / asymptomatic or symptomatic conclusively)
hCoV-19/France/HF2150/2020	EPI_ISL_418224	08/03/2020	Europe / France / Hauts de France / CompiÃ¨gne	Human		Male	54	Hospitalized	Original	Oropharyngeal swab		B.1	GH	France	Undertermined	No (couldn't determine the patient status as either pre-symptomatic / asymptomatic or symptomatic conclusively)
hCoV-19/France/HF2155/2020	EPI_ISL_418225	08/03/2020	Europe / France / Hauts de France / CompiÃ¨gne	Human		Male	89	Hospitalized	Original	Oropharyngeal swab		B.1	GH	France	Undertermined	No (couldn't determine the patient status as either pre-symptomatic / asymptomatic or symptomatic conclusively)
hCoV-19/France/HF2381/2020	EPI_ISL_418226	09/03/2020	Europe / France / Hauts de France / Crouy en Thelle	Human		Male	91	EHPAD	Original	Oropharyngeal swab		B.1	GH	France	Undertermined	No (couldn't determine the patient status as either pre-symptomatic / asymptomatic or symptomatic conclusively)
hCoV-19/France/HF2393/2020	EPI_ISL_418227	12/03/2020	Europe / France / Hauts de France / CompiÃ¨gne	Human		Male	91	Hospitalized	Original	sputum		B.1	GH	France	Undertermined	No (couldn't determine the patient status as either pre-symptomatic / asymptomatic or symptomatic conclusively)
hCoV-19/France/HF2405/2020	EPI_ISL_418228	12/03/2020	Europe / France / Hauts de France / CompiÃ¨gne	Human		Female	63	Hospitalized	Original	Oropharyngeal swab		B.1	GH	France	Undertermined	No (couldn't determine the patient status as either pre-symptomatic / asymptomatic or symptomatic conclusively)
hCoV-19/France/IDF2410/2020	EPI_ISL_418229	12/03/2020	Europe / France / Ile de France / Levallois-Perret	Human		Female	54	Hospitalized	Original	Oropharyngeal swab		B.1	GH	France	Undertermined	No (couldn't determine the patient status as either pre-symptomatic / asymptomatic or symptomatic conclusively)
hCoV-19/France/IDF2420/2020	EPI_ISL_418230	13/03/2020	Europe / France / Ile de France / Paris	Human		Female	92	Hospitalized	Original	Oropharyngeal swab		B.1	G	France	Undertermined	No (couldn't determine the patient status as either pre-symptomatic / asymptomatic or symptomatic conclusively)
hCoV-19/France/HF2496/2020	EPI_ISL_418231	15/03/2020	Europe / France / Hauts de France / CompiÃ¨gne	Human		Female	79	Hospitalized	Original	Oropharyngeal swab		B.1	G	France	Undertermined	No (couldn't determine the patient status as either pre-symptomatic / asymptomatic or symptomatic conclusively)
hCoV-19/France/IDF2532/2020	EPI_ISL_418232	15/03/2020	Europe / France / Ile de France / Meudon la ForÃªt	Human		Male	68	Hospitalized	Original	Oropharyngeal swab		B.1	GH	France	Undertermined	No (couldn't determine the patient status as either pre-symptomatic / asymptomatic or symptomatic conclusively)
hCoV-19/France/IDF2533/2020	EPI_ISL_418233	15/03/2020	Europe / France / Ile de France / Meudon la ForÃªt	Human		Female	87	Hospitalized	Original	Oropharyngeal swab		B.1	GH	France	Undertermined	No (couldn't determine the patient status as either pre-symptomatic / asymptomatic or symptomatic conclusively)
hCoV-19/France/IDF2534/2020	EPI_ISL_418234	14/03/2020	Europe / France / Ile de France / Longjumeau	Human		Female	31	Hospitalized	Original	Oropharyngeal swab		B.1	G	France	Undertermined	No (couldn't determine the patient status as either pre-symptomatic / asymptomatic or symptomatic conclusively)
hCoV-19/France/IDF2561/2020	EPI_ISL_418235	16/03/2020	Europe / France / Ile de France / Vanves	Human		Female	7 months	Physician network	Original	Oropharyngeal swab		B.1.1	GR	France	Undertermined	No (couldn't determine the patient status as either pre-symptomatic / asymptomatic or symptomatic conclusively)
hCoV-19/France/HF2586/2020	EPI_ISL_418236	16/03/2020	Europe / France / Hauts de France / CompiÃ¨gne	Human		Female	88	Hospitalized	Original	Bronchoalveolar fluid		B.1	GH	France	Undertermined	No (couldn't determine the patient status as either pre-symptomatic / asymptomatic or symptomatic conclusively)
hCoV-19/France/HF2595/2020	EPI_ISL_418237	16/03/2020	Europe / France / Hauts de France / CompiÃ¨gne	Human		Male	69	Hospitalized	Original	Oropharyngeal swab		B.1	GH	France	Undertermined	No (couldn't determine the patient status as either pre-symptomatic / asymptomatic or symptomatic conclusively)
hCoV-19/France/HF2597/2020	EPI_ISL_418238	16/03/2020	Europe / France / Hauts de France / CompiÃ¨gne	Human		Female	72	Hospitalized	Original	Oropharyngeal swab		B.1	GH	France	Undertermined	No (couldn't determine the patient status as either pre-symptomatic / asymptomatic or symptomatic conclusively)
hCoV-19/France/HF2601/2020	EPI_ISL_418239	16/03/2020	Europe / France / Hauts de France / CompiÃ¨gne	Human		Male	68	Hospitalized	Original	Oropharyngeal swab		B.1	GH	France	Undertermined	No (couldn't determine the patient status as either pre-symptomatic / asymptomatic or symptomatic conclusively)
hCoV-19/France/IDF2684/2020	EPI_ISL_418240	16/03/2020	Europe / France / Ile de France / Longjumeau	Human		Male	83	Hospitalized	Original	Oropharyngeal swab		B.1	G	France	Undertermined	No (couldn't determine the patient status as either pre-symptomatic / asymptomatic or symptomatic conclusively)
hCoV-19/Algeria/G0638_2264/2020	EPI_ISL_418241	02/03/2020	Africa / Algeria / Boufarik	Human		Female	28	Hospitalized	Original	unknown		B.1	GH	Algeria	Undertermined	No (couldn't determine the patient status as either pre-symptomatic / asymptomatic or symptomatic conclusively)
hCoV-19/Algeria/G0640_2265/2020	EPI_ISL_418242	08/03/2020	Africa / Algeria / Blida	Human		Male	87	Hospitalized	Original	unknown		B.1	GH	Algeria	Undertermined	No (couldn't determine the patient status as either pre-symptomatic / asymptomatic or symptomatic conclusively)
hCoV-19/Spain/AN-ISCIII-201272/2020	EPI_ISL_418243	28/02/2020	Europe / Spain / Andalucia	Human		Male	64	Hospitalized	Original	Oropharyngeal swab		B.2	V	Spain	Undertermined	No (couldn't determine the patient status as either pre-symptomatic / asymptomatic or symptomatic conclusively)
hCoV-19/Spain/AN-ISCIII-201373/2020	EPI_ISL_418244	02/03/2020	Europe / Spain / Andalucia	Human		Male	74	Hospitalized	Original	Oropharyngeal swab		B	O	Spain	Undertermined	No (couldn't determine the patient status as either pre-symptomatic / asymptomatic or symptomatic conclusively)
hCoV-19/Spain/CM-ISCIII-201328/2020	EPI_ISL_418245	01/03/2020	Europe / Spain / Castilla La Mancha	Human		Male	76	Hospitalized	Original	Oropharyngeal swab		A.5	S	Spain	Undertermined	No (couldn't determine the patient status as either pre-symptomatic / asymptomatic or symptomatic conclusively)
hCoV-19/Spain/CM-ISCIII-201329/2020	EPI_ISL_418246	01/03/2020	Europe / Spain / Castilla La Mancha	Human		Male	76	Hospitalized	Original	Oropharyngeal swab		A	S	Spain	Undertermined	No (couldn't determine the patient status as either pre-symptomatic / asymptomatic or symptomatic conclusively)
hCoV-19/Spain/CL-ISCIII-201061/2020	EPI_ISL_418247	26/02/2020	Europe / Spain / Castilla y Leon	Human		Male	18	Hospitalized	Original	Oropharyngeal swab		B.1	G	Spain	Undertermined	No (couldn't determine the patient status as either pre-symptomatic / asymptomatic or symptomatic conclusively)
hCoV-19/Spain/CL-ISCIII-201323/2020	EPI_ISL_418248	01/03/2020	Europe / Spain / Castilla y Leon	Human		Male	52	Hospitalized	Original	Oropharyngeal swab		A.2	S	Spain	Undertermined	No (couldn't determine the patient status as either pre-symptomatic / asymptomatic or symptomatic conclusively)
hCoV-19/Spain/CL-ISCIII-201372/2020	EPI_ISL_418249	03/03/2020	Europe / Spain / Castilla y Leon	Human		Female	25	Hospitalized	Original	Oropharyngeal swab		A.2	S	Spain	Undertermined	No (couldn't determine the patient status as either pre-symptomatic / asymptomatic or symptomatic conclusively)
hCoV-19/Spain/CT-ISCIII-201396/2020	EPI_ISL_418250	12/07/1905	Europe / Spain / Catalunya	Human		Male	23	Hospitalized	Original	Oropharyngeal swab		B.1.1	GR	Spain	Undertermined	No (couldn't determine the patient status as either pre-symptomatic / asymptomatic or symptomatic conclusively)
hCoV-19/Spain/MD-ISCIII-201105/2020	EPI_ISL_418251	25/02/2020	Europe / Spain / Madrid	Human		Male	24	Hospitalized	Original	Oropharyngeal swab		B.1	G	Spain	Undertermined	No (couldn't determine the patient status as either pre-symptomatic / asymptomatic or symptomatic conclusively)
hCoV-19/Spain/MD-ISCIII-201449/2020	EPI_ISL_418252	04/03/2020	Europe / Spain / Madrid	Human		Male	79	Hospitalized	Original	Oropharyngeal swab		A.5	S	Spain	Undertermined	No (couldn't determine the patient status as either pre-symptomatic / asymptomatic or symptomatic conclusively)
hCoV-19/Spain/PV-ISCIII-201382/2020	EPI_ISL_418253	02/03/2020	Europe / Spain / Basque Country	Human		Male	91	Hospitalized	Original	Oropharyngeal swab		A.2	S	Spain	Undertermined	No (couldn't determine the patient status as either pre-symptomatic / asymptomatic or symptomatic conclusively)
hCoV-19/Italy/ABR-IZSGC-TE4880/2020	EPI_ISL_418256	14/03/2020	Europe / Italy / Abruzzo	Human		Male	80	Hospitalized	Original	Oropharyngeal swab		B.1.1	GR	Italy	Undertermined	No (couldn't determine the patient status as either pre-symptomatic / asymptomatic or symptomatic conclusively)
hCoV-19/Italy/ABR-IZSGC-TE5056/2020	EPI_ISL_418257	17/03/2020	Europe / Italy / Abruzzo	Human		Female	75	Screening	Original	Oropharyngeal swab		B.1.1	GR	Italy	Undertermined	No (couldn't determine the patient status as either pre-symptomatic / asymptomatic or symptomatic conclusively)
hCoV-19/Italy/ABR-IZSGC-TE4959/2020	EPI_ISL_418259	14/03/2020	Europe / Italy / Abruzzo	Human		Male	76	unkown	Original			B.1.1	GR	Italy	Undertermined	No (couldn't determine the patient status as either pre-symptomatic / asymptomatic or symptomatic conclusively)
hCoV-19/Colombia/DC-INS-78390/2020	EPI_ISL_418262	06/03/2020	South America / Colombia / Bogota	Human		Female	44105	Live	Original	Swab		B.1.1	GR	Colombia	Undertermined	No (couldn't determine the patient status as either pre-symptomatic / asymptomatic or symptomatic conclusively)
hCoV-19/Spain/CT-IrsiCaixa-04/2020	EPI_ISL_418268	13/03/2020	Europe / Spain / Catalunya / Barcelona / Badalona	Human	Hospital Universitari Germans Trias i Pujol	Male	89	Hospitalized	Original	Oropharyngeal swab		B.1.5	O	Spain	Undertermined	No (couldn't determine the patient status as either pre-symptomatic / asymptomatic or symptomatic conclusively)
hCoV-19/Wales/PHWC-24D2D/2020	EPI_ISL_419503	20/03/2020	Europe / United Kingdom / Wales	Human		Female	96	unknown	Original	Dry swab		B.3	L	United Kingdom	Undertermined	No (couldn't determine the patient status as either pre-symptomatic / asymptomatic or symptomatic conclusively)
hCoV-19/Spain/CT-ISCIII-201397/2020	EPI_ISL_419707	12/07/1905	Europe / Spain / Catalunya	Human		Female	unknown	Hospitalized	Original	Oropharyngeal swab		B.1.5	G	Spain	Undertermined	No (couldn't determine the patient status as either pre-symptomatic / asymptomatic or symptomatic conclusively)
hCoV-19/Spain/PV-ISCIII-201602/2020	EPI_ISL_419709	04/03/2020	Europe / Spain / Basque Country	Human		Male	89	Hospitalized	Original	Oropharyngeal swab		A.2	S	Spain	Undertermined	No (couldn't determine the patient status as either pre-symptomatic / asymptomatic or symptomatic conclusively)
hCoV-19/Russia/StPetersburg-RII4705S/2020	EPI_ISL_430067	07/04/2020	Europe / Russia / Saint Petersburg	Human		Female	64	Hospitalized	Original	Nasopharyngeal swab		B.1.1	GR	Russia	Undertermined	No (couldn't determine the patient status as either pre-symptomatic / asymptomatic or symptomatic conclusively)
hCoV-19/Russia/StPetersburg-RII4780S/2020	EPI_ISL_430069	07/04/2020	Europe / Russia / Saint Petersburg	Human		Female	40	Hospitalized	Original	Nasopharyngeal swab		B.1.1	GR	Russia	Undertermined	No (couldn't determine the patient status as either pre-symptomatic / asymptomatic or symptomatic conclusively)
hCoV-19/Russia/StPetersburg-RII4917S/2020	EPI_ISL_430070	07/04/2020	Europe / Russia / Saint Petersburg	Human		Male	55	Hospitalized	Original	Nasopharyngeal swab		B.1.1	GR	Russia	Undertermined	No (couldn't determine the patient status as either pre-symptomatic / asymptomatic or symptomatic conclusively)
hCoV-19/Russia/StPetersburg-RII4936S/2020	EPI_ISL_430071	08/04/2020	Europe / Russia / Saint Petersburg	Human		Male	47	Hospitalized	Original	Nasopharyngeal swab		B.1	G	Russia	Undertermined	No (couldn't determine the patient status as either pre-symptomatic / asymptomatic or symptomatic conclusively)
hCoV-19/Russia/StPetersburg-RII4937S/2020	EPI_ISL_430072	08/04/2020	Europe / Russia / Saint Petersburg	Human		Female	52	Hospitalized	Original	Nasopharyngeal swab		B.1.5	G	Russia	Undertermined	No (couldn't determine the patient status as either pre-symptomatic / asymptomatic or symptomatic conclusively)
hCoV-19/Russia/StPetersburg-RII4938S/2020	EPI_ISL_430073	08/04/2020	Europe / Russia / Saint Petersburg	Human		Female	47	Hospitalized	Original	Nasopharyngeal swab		B.1.1	GR	Russia	Undertermined	No (couldn't determine the patient status as either pre-symptomatic / asymptomatic or symptomatic conclusively)
hCoV-19/Russia/StPetersburg-RII4944S/2020	EPI_ISL_430074	08/04/2020	Europe / Russia / Saint Petersburg	Human		Male	25	Hospitalized	Original	Nasopharyngeal swab		B.1.5	G	Russia	Undertermined	No (couldn't determine the patient status as either pre-symptomatic / asymptomatic or symptomatic conclusively)
hCoV-19/Russia/StPetersburg-RII4949S/2020	EPI_ISL_430075	09/04/2020	Europe / Russia / Saint Petersburg	Human		Female	64	Hospitalized	Original	Nasopharyngeal swab		B.1.5	G	Russia	Undertermined	No (couldn't determine the patient status as either pre-symptomatic / asymptomatic or symptomatic conclusively)
hCoV-19/Russia/StPetersburg-RII4969S/2020	EPI_ISL_430076	10/04/2020	Europe / Russia / Saint Petersburg	Human		Female	71	Hospitalized	Original	Nasopharyngeal swab		B.1.1	GR	Russia	Undertermined	No (couldn't determine the patient status as either pre-symptomatic / asymptomatic or symptomatic conclusively)
hCoV-19/Russia/StPetersburg-RII4970S/2020	EPI_ISL_430077	10/04/2020	Europe / Russia / Saint Petersburg	Human		Female	unknown	Hospitalized	Original	Nasopharyngeal swab		B.1.1	GR	Russia	Undertermined	No (couldn't determine the patient status as either pre-symptomatic / asymptomatic or symptomatic conclusively)
hCoV-19/Russia/StPetersburg-RII4972S/2020	EPI_ISL_430078	10/04/2020	Europe / Russia / Saint Petersburg	Human		Female	72	Hospitalized	Original	Nasopharyngeal swab		B.1.1	GR	Russia	Undertermined	No (couldn't determine the patient status as either pre-symptomatic / asymptomatic or symptomatic conclusively)
hCoV-19/Russia/StPetersburg-RII4980S/2020	EPI_ISL_430079	10/04/2020	Europe / Russia / Saint Petersburg	Human		Female	50	Hospitalized	Original	Nasopharyngeal swab		B.1.1	GR	Russia	Undertermined	No (couldn't determine the patient status as either pre-symptomatic / asymptomatic or symptomatic conclusively)
hCoV-19/Russia/StPetersburg-RII4983S/2020	EPI_ISL_430080	10/04/2020	Europe / Russia / Saint Petersburg	Human		Female	64	Hospitalized	Original	Nasopharyngeal swab		B.1.1	GR	Russia	Undertermined	No (couldn't determine the patient status as either pre-symptomatic / asymptomatic or symptomatic conclusively)
hCoV-19/Russia/StPetersburg-RII4984S/2020	EPI_ISL_430081	10/04/2020	Europe / Russia / Saint Petersburg	Human		Male	33	Hospitalized	Original	Nasopharyngeal swab		B.1.1	GR	Russia	Undertermined	No (couldn't determine the patient status as either pre-symptomatic / asymptomatic or symptomatic conclusively)
hCoV-19/Russia/StPetersburg-RII4985S/2020	EPI_ISL_430082	10/04/2020	Europe / Russia / Saint Petersburg	Human		Female	71	Hospitalized	Original	Nasopharyngeal swab		B.1.1	GR	Russia	Undertermined	No (couldn't determine the patient status as either pre-symptomatic / asymptomatic or symptomatic conclusively)
hCoV-19/Russia/StPetersburg-RII4988S/2020	EPI_ISL_430083	10/04/2020	Europe / Russia / Saint Petersburg	Human		Female	49	Hospitalized	Original	Nasopharyngeal swab		B.1.1	GR	Russia	Undertermined	No (couldn't determine the patient status as either pre-symptomatic / asymptomatic or symptomatic conclusively)
hCoV-19/Russia/StPetersburg-RII5029S/2020	EPI_ISL_430085	10/04/2020	Europe / Russia / Saint Petersburg	Human		Female	41	Hospitalized	Original	Nasopharyngeal swab		B.1.1	GR	Russia	Undertermined	No (couldn't determine the patient status as either pre-symptomatic / asymptomatic or symptomatic conclusively)
hCoV-19/Russia/StPetersburg-RII5033S/2020	EPI_ISL_430086	10/04/2020	Europe / Russia / Saint Petersburg	Human		Female	50	Hospitalized	Original	Nasopharyngeal swab		B.1.1	GR	Russia	Undertermined	No (couldn't determine the patient status as either pre-symptomatic / asymptomatic or symptomatic conclusively)
hCoV-19/Russia/StPetersburg-RII5047S/2020	EPI_ISL_430087	10/04/2020	Europe / Russia / Saint Petersburg	Human		Female	50	Hospitalized	Original	Nasopharyngeal swab		B.1.1	GR	Russia	Undertermined	No (couldn't determine the patient status as either pre-symptomatic / asymptomatic or symptomatic conclusively)
hCoV-19/Russia/StPetersburg-RII5169S/2020	EPI_ISL_430088	09/04/2020	Europe / Russia / Saint Petersburg	Human		Female	55	Hospitalized	Original	Nasopharyngeal swab		B.1.1	GR	Russia	Undertermined	No (couldn't determine the patient status as either pre-symptomatic / asymptomatic or symptomatic conclusively)
hCoV-19/Russia/StPetersburg-RII5170S/2020	EPI_ISL_430089	09/04/2020	Europe / Russia / Saint Petersburg	Human		unknown	45	Hospitalized	Original	Nasopharyngeal swab		B.1.1	GR	Russia	Undertermined	No (couldn't determine the patient status as either pre-symptomatic / asymptomatic or symptomatic conclusively)
hCoV-19/Russia/StPetersburg-RII5244S/2020	EPI_ISL_430090	10/04/2020	Europe / Russia / Saint Petersburg	Human		unknown	59	Hospitalized	Original	Nasopharyngeal swab		B.1.5	G	Russia	Undertermined	No (couldn't determine the patient status as either pre-symptomatic / asymptomatic or symptomatic conclusively)
hCoV-19/Russia/StPetersburg-RII6027S/2020	EPI_ISL_430100	14/04/2020	Europe / Russia / Saint Petersburg	Human		Female	33	Hospitalized	Original	Nasopharyngeal swab		B.1.5	G	Russia	Undertermined	No (couldn't determine the patient status as either pre-symptomatic / asymptomatic or symptomatic conclusively)
hCoV-19/Russia/StPetersburg-RII6031S/2020	EPI_ISL_430101	14/04/2020	Europe / Russia / Saint Petersburg	Human		unknown	54	Hospitalized	Original	Nasopharyngeal swab		B.1.5	G	Russia	Undertermined	No (couldn't determine the patient status as either pre-symptomatic / asymptomatic or symptomatic conclusively)
hCoV-19/Russia/StPetersburg-RII6039S/2020	EPI_ISL_430102	14/04/2020	Europe / Russia / Saint Petersburg	Human		Female	78	Hospitalized	Original	Nasopharyngeal swab		B.1.1	GR	Russia	Undertermined	No (couldn't determine the patient status as either pre-symptomatic / asymptomatic or symptomatic conclusively)
hCoV-19/Russia/StPetersburg-RII6043S/2020	EPI_ISL_430103	14/04/2020	Europe / Russia / Saint Petersburg	Human		Female	30	Hospitalized	Original	Nasopharyngeal swab		B.1.1	GR	Russia	Undertermined	No (couldn't determine the patient status as either pre-symptomatic / asymptomatic or symptomatic conclusively)
hCoV-19/Russia/StPetersburg-RII6053S/2020	EPI_ISL_430104	15/04/2020	Europe / Russia / Saint Petersburg	Human		Female	60	Hospitalized	Original	Nasopharyngeal swab		B.1.5	G	Russia	Undertermined	No (couldn't determine the patient status as either pre-symptomatic / asymptomatic or symptomatic conclusively)
hCoV-19/Russia/StPetersburg-RII6055S/2020	EPI_ISL_430105	15/04/2020	Europe / Russia / Saint Petersburg	Human		Female	57	Hospitalized	Original	Nasopharyngeal swab		B.1	G	Russia	Undertermined	No (couldn't determine the patient status as either pre-symptomatic / asymptomatic or symptomatic conclusively)
hCoV-19/Russia/StPetersburg-RII6056S/2020	EPI_ISL_430106	15/04/2020	Europe / Russia / Saint Petersburg	Human		Female	71	Hospitalized	Original	Nasopharyngeal swab		B.1.1	GR	Russia	Undertermined	No (couldn't determine the patient status as either pre-symptomatic / asymptomatic or symptomatic conclusively)
hCoV-19/Russia/StPetersburg-RII6060S/2020	EPI_ISL_430107	15/04/2020	Europe / Russia / Saint Petersburg	Human		unknown	24	Hospitalized	Original	Nasopharyngeal swab		B.1.1	GR	Russia	Undertermined	No (couldn't determine the patient status as either pre-symptomatic / asymptomatic or symptomatic conclusively)
hCoV-19/Russia/StPetersburg-RII6061S/2020	EPI_ISL_430108	15/04/2020	Europe / Russia / Saint Petersburg	Human		Male	25	Hospitalized	Original	Nasopharyngeal swab		B.1.5	G	Russia	Undertermined	No (couldn't determine the patient status as either pre-symptomatic / asymptomatic or symptomatic conclusively)
hCoV-19/Russia/StPetersburg-RII6063S/2020	EPI_ISL_430109	15/04/2020	Europe / Russia / Saint Petersburg	Human		Female	54	Hospitalized	Original	Nasopharyngeal swab		B.1.1	GR	Russia	Undertermined	No (couldn't determine the patient status as either pre-symptomatic / asymptomatic or symptomatic conclusively)
hCoV-19/Russia/StPetersburg-RII6065S/2020	EPI_ISL_430110	15/04/2020	Europe / Russia / Saint Petersburg	Human		Female	47	Hospitalized	Original	Nasopharyngeal swab		B.1.1	GR	Russia	Undertermined	No (couldn't determine the patient status as either pre-symptomatic / asymptomatic or symptomatic conclusively)
hCoV-19/Russia/StPetersburg-RII6086S/2020	EPI_ISL_430111	15/04/2020	Europe / Russia / Saint Petersburg	Human		Female	67	Hospitalized	Original	Nasopharyngeal swab		B.1.1	GR	Russia	Undertermined	No (couldn't determine the patient status as either pre-symptomatic / asymptomatic or symptomatic conclusively)
hCoV-19/South Africa/R02827/2020	EPI_ISL_430297	06/03/2020	Africa / South Africa / GP	Human		Female	39	Live	Original	Combined nasopharyngeal and oropharyngeal swab		B	L	South Africa	Undertermined	No (couldn't determine the patient status as either pre-symptomatic / asymptomatic or symptomatic conclusively)
hCoV-19/Kazakhstan/NCB-1/2020	EPI_ISL_435045	22/03/2020	Asia / Kazakhstan / Nur-Sultan	Human		unknown	unknown	Hospitalized	Original	Oro-pharyngeal swab		B.4	O	Kazakhstan	Undertermined	No (couldn't determine the patient status as either pre-symptomatic / asymptomatic or symptomatic conclusively)
hCoV-19/India/GJ-GBRC2/2020	EPI_ISL_435049	13/04/2020	Asia / India / Gujarat / Ahmedabad	Human		Male	45	Released, Live	Original	Oro-pharyngeal swab		B.1	G	India	Undertermined	No (couldn't determine the patient status as either pre-symptomatic / asymptomatic or symptomatic conclusively)
hCoV-19/India/GJ-GBRC3/2020	EPI_ISL_435050	13/04/2020	Asia / India / Gujarat / Ahmedabad	Human		Female	42	Released, Live	Original	Oro-pharyngeal swab		B.1	G	India	Undertermined	No (couldn't determine the patient status as either pre-symptomatic / asymptomatic or symptomatic conclusively)
hCoV-19/India/GJ-GBRC4/2020	EPI_ISL_435051	13/04/2020	Asia / India / Gujarat / Ahmedabad	Human		Male	66	Deceased	Original	Oro-pharyngeal swab		B.1	GH	India	Symptomatic	Yes
hCoV-19/India/GJ-GBRC5/2020	EPI_ISL_435052	13/04/2020	Asia / India / Gujarat / Ahmedabad	Human		Female	66	Released, Live	Original	Oro-pharyngeal swab		B.1	GH	India	Undertermined	No (couldn't determine the patient status as either pre-symptomatic / asymptomatic or symptomatic conclusively)
hCoV-19/India/GJ-GBRC6/2020	EPI_ISL_435053	07/04/2020	Asia / India / Gujarat / Ahmedabad	Human		Female	60	Released	Original	Oro-pharyngeal swab		B.1	G	India	Undertermined	No (couldn't determine the patient status as either pre-symptomatic / asymptomatic or symptomatic conclusively)
hCoV-19/India/GJ-GBRC8/2020	EPI_ISL_435055	22/04/2020	Asia / India / Gujarat / Gandhinagar	Human		Male	33	Released	Original	Oro-pharyngeal swab		B.1.113	GH	India	Undertermined	No (couldn't determine the patient status as either pre-symptomatic / asymptomatic or symptomatic conclusively)
hCoV-19/India/GJ-GBRC9/2020	EPI_ISL_435056	21/04/2020	Asia / India / Gujarat / Mansa	Human		Male	65	Released	Original	Oro-pharyngeal swab		B.1	G	India	Undertermined	No (couldn't determine the patient status as either pre-symptomatic / asymptomatic or symptomatic conclusively)
hCoV-19/South Africa/R02606/2020	EPI_ISL_435058	11/03/2020	Africa / South Africa / KZN	Human		Male	38	Live	Original	Combined nasopharyngeal and oropharyngeal swab		B.1	G	South Africa	Undertermined	No (couldn't determine the patient status as either pre-symptomatic / asymptomatic or symptomatic conclusively)
hCoV-19/India/TG-GMC-KP1125/2020	EPI_ISL_437626	24/03/2020	Asia / India / Telangana / Hyderabad	Human	Travel history from USA Via Dubai to India	Male	70	Hospitalized/Released	Original	Oro-pharyngeal Swab		B.1	GH	India	Undertermined	No (couldn't determine the patient status as either pre-symptomatic / asymptomatic or symptomatic conclusively)
hCoV-19/Saudi Arabia/KAUST-Makkah124/2020	EPI_ISL_437691	24/03/2020	Asia / Saudi Arabia / Makkah	Human		Female	55	Hospitalized	Original			B.1.36	GH	Saudi Arabia	Undertermined	No (couldn't determine the patient status as either pre-symptomatic / asymptomatic or symptomatic conclusively)
hCoV-19/Saudi Arabia/KAUST-Makkah126/2020	EPI_ISL_437692	24/03/2020	Asia / Saudi Arabia / Makkah	Human		Male	32	Hospitalized	Original			B.1.36	GH	Saudi Arabia	Undertermined	No (couldn't determine the patient status as either pre-symptomatic / asymptomatic or symptomatic conclusively)
hCoV-19/Saudi Arabia/KAUST-Makkah138/2020	EPI_ISL_437693	24/03/2020	Asia / Saudi Arabia / Makkah	Human		Female	75	Hospitalized	Original			B.1.36	GH	Saudi Arabia	Undertermined	No (couldn't determine the patient status as either pre-symptomatic / asymptomatic or symptomatic conclusively)
hCoV-19/Saudi Arabia/KAUST-Makkah139/2020	EPI_ISL_437694	24/03/2020	Asia / Saudi Arabia / Makkah	Human		Male	45	Hospitalized	Original			B.1	GH	Saudi Arabia	Undertermined	No (couldn't determine the patient status as either pre-symptomatic / asymptomatic or symptomatic conclusively)
hCoV-19/Saudi Arabia/KAUST-Makkah142/2020	EPI_ISL_437695	24/03/2020	Asia / Saudi Arabia / Makkah	Human		Male	50	Hospitalized	Original			B.1.36	GH	Saudi Arabia	Undertermined	No (couldn't determine the patient status as either pre-symptomatic / asymptomatic or symptomatic conclusively)
hCoV-19/Saudi Arabia/KAUST-Makkah156/2020	EPI_ISL_437696	06/04/2020	Asia / Saudi Arabia / Makkah	Human		Male	8	Hospitalized	Original			B.1.36	GH	Saudi Arabia	Undertermined	No (couldn't determine the patient status as either pre-symptomatic / asymptomatic or symptomatic conclusively)
hCoV-19/Saudi Arabia/KAUST-Makkah163/2020	EPI_ISL_437697	06/04/2020	Asia / Saudi Arabia / Makkah	Human		Male	33	Hospitalized	Original			B.1	GH	Saudi Arabia	Undertermined	No (couldn't determine the patient status as either pre-symptomatic / asymptomatic or symptomatic conclusively)
hCoV-19/Saudi Arabia/KAUST-Makkah166/2020	EPI_ISL_437698	06/04/2020	Asia / Saudi Arabia / Makkah	Human		Male	27	Hospitalized	Original			A	S	Saudi Arabia	Undertermined	No (couldn't determine the patient status as either pre-symptomatic / asymptomatic or symptomatic conclusively)
hCoV-19/Saudi Arabia/KAUST-Makkah173/2020	EPI_ISL_437699	06/04/2020	Asia / Saudi Arabia / Makkah	Human		Male	50	Hospitalized	Original			B.1	GH	Saudi Arabia	Undertermined	No (couldn't determine the patient status as either pre-symptomatic / asymptomatic or symptomatic conclusively)
hCoV-19/Iran/KHGRC-2-2162/2020	EPI_ISL_442044	26/03/2020	Asia / Iran / Tehran	Human		unknown	unknown	Hospitalized	Original	Throat swab		B.4	L	Iran	Undertermined	No (couldn't determine the patient status as either pre-symptomatic / asymptomatic or symptomatic conclusively)
hCoV-19/India/GJ-GBRC56a/2020	EPI_ISL_447030	03/05/2020	Asia / India / Gujarat / Ahmedabad	Human		Female	65	Released	Original			B.1	G	India	Undertermined	No (couldn't determine the patient status as either pre-symptomatic / asymptomatic or symptomatic conclusively)
hCoV-19/India/GJ-GBRC56b/2020	EPI_ISL_447031	03/05/2020	Asia / India / Gujarat / Ahmedabad	Human		Female	65	Released	Original			B.1	G	India	Undertermined	No (couldn't determine the patient status as either pre-symptomatic / asymptomatic or symptomatic conclusively)
hCoV-19/India/GJ-GBRC58/2020	EPI_ISL_447033	03/05/2020	Asia / India / Gujarat / Ahmedabad	Human		Male	40	Released	Original			B.1.113	GH	India	Undertermined	No (couldn't determine the patient status as either pre-symptomatic / asymptomatic or symptomatic conclusively)
hCoV-19/India/GJ-GBRC59/2020	EPI_ISL_447034	03/05/2020	Asia / India / Gujarat / Ahmedabad	Human		Male	78	Released, Live	Original			B.1.113	GH	India	Undertermined	No (couldn't determine the patient status as either pre-symptomatic / asymptomatic or symptomatic conclusively)
hCoV-19/India/GJ-GBRC60/2020	EPI_ISL_447035	03/05/2020	Asia / India / Gujarat / Ahmedabad	Human		Female	78	Deceased	Original			B.1.113	GH	India	Symptomatic	Yes
hCoV-19/India/GJ-GBRC63/2020	EPI_ISL_447038	03/05/2020	Asia / India / Gujarat / Ahmedabad	Human		Female	34	Released	Original			B.1	G	India	Undertermined	No (couldn't determine the patient status as either pre-symptomatic / asymptomatic or symptomatic conclusively)

hCoV-19/India/GJ-GBRC64/2020	EPI_ISL_447039	03/05/2020	Asia / India / Gujarat / Ahmedabad	Human		Female	52	Released	Original			B.1.113	GH	India	Undertermined	No (couldn't determine the patient status as either pre-symptomatic / asymptomatic or symptomatic conclusively)
hCoV-19/India/GJ-GBRC66/2020	EPI_ISL_447042	03/05/2020	Asia / India / Gujarat / Ahmedabad	Human		Male	24	Released	Original			B.1.36	GH	India	Undertermined	No (couldn't determine the patient status as either pre-symptomatic / asymptomatic or symptomatic conclusively)
hCoV-19/India/GJ-GBRC67a/2020	EPI_ISL_447043	03/05/2020	Asia / India / Gujarat / Ahmedabad	Human		Female	42	Released	Original			B.1.113	GH	India	Undertermined	No (couldn't determine the patient status as either pre-symptomatic / asymptomatic or symptomatic conclusively)
hCoV-19/India/GJ-GBRC67b/2020	EPI_ISL_447044	03/05/2020	Asia / India / Gujarat / Ahmedabad	Human		Female	42	Released	Original			B.1.113	GH	India	Undertermined	No (couldn't determine the patient status as either pre-symptomatic / asymptomatic or symptomatic conclusively)
hCoV-19/India/GJ-GBRC68/2020	EPI_ISL_447045	03/05/2020	Asia / India / Gujarat / Ahmedabad	Human		Male	28	Released	Original			B.1.113	GH	India	Undertermined	No (couldn't determine the patient status as either pre-symptomatic / asymptomatic or symptomatic conclusively)
hCoV-19/India/GJ-GBRC71/2020	EPI_ISL_447048	27/04/2020	Asia / India / Gujarat / Gandhinagar	Human		Female	70	Released	Original			B.1.113	GH	India	Undertermined	No (couldn't determine the patient status as either pre-symptomatic / asymptomatic or symptomatic conclusively)
hCoV-19/India/GJ-GBRC75/2020	EPI_ISL_447053	29/04/2020	Asia / India / Gujarat / Gandhinagar	Human		Male	66	Deceased	Original			B.1.36	GH	India	Symptomatic	Yes
hCoV-19/Romania/279068/2020	EPI_ISL_447054	30/04/2020	Europe / Romania / Constanta	Human		Female	87	Live	Original	Nasopharyngeal swab		B.1	GH	Romania	Undertermined	No (couldn't determine the patient status as either pre-symptomatic / asymptomatic or symptomatic conclusively)
hCoV-19/Bangladesh/BARJ-CVASU-CTG-502/2020	EPI_ISL_450340	13/05/2020	Asia / Bangladesh / Chittagong	Human		Female	43	Live	Original	Nasopharyngeal swab		A	S	Bangladesh	Undertermined	No (couldn't determine the patient status as either pre-symptomatic / asymptomatic or symptomatic conclusively)
hCoV-19/Bangladesh/BARJ-CVASU-CTG-503/2020	EPI_ISL_450341	13/05/2020	Asia / Bangladesh / Chittagong	Human		Female	85	Released	Original	Nasopharyngeal swab		A	S	Bangladesh	Undertermined	No (couldn't determine the patient status as either pre-symptomatic / asymptomatic or symptomatic conclusively)
hCoV-19/Bangladesh/BARJ-CVASU-CTG-506/2020	EPI_ISL_450342	08/05/2020	Asia / Bangladesh / Chittagong / Chittagong / Satkania Upazila	Human		Male	42	Released	Original	Nasopharyngeal swab		A	S	Bangladesh	Undertermined	No (couldn't determine the patient status as either pre-symptomatic / asymptomatic or symptomatic conclusively)
hCoV-19/Bangladesh/BARJ-CVASU-CTG-511/2020	EPI_ISL_450343	09/05/2020	Asia / Bangladesh / Chittagong	Human		Male	32	Live	Original	Nasopharyngeal swab		B.1.113	G	Bangladesh	Undertermined	No (couldn't determine the patient status as either pre-symptomatic / asymptomatic or symptomatic conclusively)
hCoV-19/Lebanon/S1_758/2020	EPI_ISL_450508	04/03/2020	Asia / Lebanon / Beirut	Human		Male	56	Deceased	Original	Sputum/PBS	Patient infected while traveling in Egypt	B.1	GH	Lebanon	Symptomatic	Yes
hCoV-19/Lebanon/S2_759/2020	EPI_ISL_450509	15/03/2020	Asia / Lebanon / Beirut	Human		Male	63	Released	Original	Nasopharyngeal VTM		B.1	GH	Lebanon	Undertermined	No (couldn't determine the patient status as either pre-symptomatic / asymptomatic or symptomatic conclusively)
hCoV-19/Lebanon/S3_760/2020	EPI_ISL_450510	04/03/2020	Asia / Lebanon / Beirut	Human		Male	19	Released	Original	Nasopharyngeal VTM	Patient infected while traveling in Iran	B.1	O	Lebanon	Undertermined	No (couldn't determine the patient status as either pre-symptomatic / asymptomatic or symptomatic conclusively)
hCoV-19/Lebanon/S4_761/2020	EPI_ISL_450511	04/03/2020	Asia / Lebanon / Beirut	Human		Female	33	Released	Original	Nasopharyngeal VTM	Patient infected while traveling in United Kingdom	B.1.1	GR	Lebanon	Undertermined	No (couldn't determine the patient status as either pre-symptomatic / asymptomatic or symptomatic conclusively)
hCoV-19/Lebanon/S5_762/2020	EPI_ISL_450512	27/02/2020	Asia / Lebanon / Beirut	Human		Male	42	Released	Original	Nasopharyngeal VTM	Patient infected while traveling in Iran	B.4	O	Lebanon	Undertermined	No (couldn't determine the patient status as either pre-symptomatic / asymptomatic or symptomatic conclusively)
hCoV-19/Lebanon/S6_766/2020	EPI_ISL_450513	09/03/2020	Asia / Lebanon / Beirut	Human		Male	unknown	Released	Original	Nasopharyngeal VTM	Patient infected while traveling in France	B.1.1	GR	Lebanon	Undertermined	No (couldn't determine the patient status as either pre-symptomatic / asymptomatic or symptomatic conclusively)
hCoV-19/Lebanon/S8_767/2020	EPI_ISL_450514	13/03/2020	Asia / Lebanon / Beirut	Human		Female	74	Released	Original	Nasopharyngeal VTM		B.1	GH	Lebanon	Undertermined	No (couldn't determine the patient status as either pre-symptomatic / asymptomatic or symptomatic conclusively)
hCoV-19/Lebanon/S9_764/2020	EPI_ISL_450515	11/03/2020	Asia / Lebanon / Beirut	Human		Female	25	Released	Original	Nasopharyngeal VTM	Patient infected while traveling in United Kingdom	B.4	O	Lebanon	Undertermined	No (couldn't determine the patient status as either pre-symptomatic / asymptomatic or symptomatic conclusively)
hCoV-19/Lebanon/S10_768/2020	EPI_ISL_450516	15/03/2020	Asia / Lebanon / Beirut	Human		Male	36	Released	Original	Sputum/PBS		B.1	GH	Lebanon	Undertermined	No (couldn't determine the patient status as either pre-symptomatic / asymptomatic or symptomatic conclusively)
hCoV-19/Lebanon/S11_765/2020	EPI_ISL_450517	09/03/2020	Asia / Lebanon / Beirut	Human		Male	22	Released	Original	Nasopharyngeal VTM	Patient infected while traveling in Italy	B.1	GH	Lebanon	Undertermined	No (couldn't determine the patient status as either pre-symptomatic / asymptomatic or symptomatic conclusively)
hCoV-19/Poland/Sla8/2020	EPI_ISL_451662	05/05/2020	Europe / Poland / Slask	Human		Male	unknown	Quarantine	Original		Active miner	B.1.1	GR	Poland	Undertermined	No (couldn't determine the patient status as either pre-symptomatic / asymptomatic or symptomatic conclusively)
hCoV-19/Poland/Sla9/2020	EPI_ISL_451663	05/05/2020	Europe / Poland / Slask	Human		Male	unknown	Quarantine	Original		Active miner	B.1.1	GR	Poland	Undertermined	No (couldn't determine the patient status as either pre-symptomatic / asymptomatic or symptomatic conclusively)
hCoV-19/Poland/Sla10/2020	EPI_ISL_451664	05/05/2020	Europe / Poland / Slask	Human		Male	unknown	Quarantine	Original		Active miner	B.1.1	GR	Poland	Undertermined	No (couldn't determine the patient status as either pre-symptomatic / asymptomatic or symptomatic conclusively)
hCoV-19/India/GJ-GBRC103/2020	EPI_ISL_451666	04/05/2020	Asia / India / Gujarat / Jamnagar	Human		Male	54	Released, Live	Original			B.6	O	India	Undertermined	No (couldn't determine the patient status as either pre-symptomatic / asymptomatic or symptomatic conclusively)
hCoV-19/Belgium/20A30/2020	EPI_ISL_451935	18/03/2020	Europe / Belgium / Brussels	Human		Male	77	Deceased	Original	Lung		B.1	G	Belgium	Symptomatic	Yes
hCoV-19/Italy/APU-UniMI-CL1/2020	EPI_ISL_451961	14/03/2020	Europe / Italy / Apulia	Human		Male	61, 11 months	Released	Original			B.1	G	Italy	Undertermined	No (couldn't determine the patient status as either pre-symptomatic / asymptomatic or symptomatic conclusively)
hCoV-19/Italy/APU-UniMI-1259/2020	EPI_ISL_451962	20/03/2020	Europe / Italy / Apulia	Human		Male	56	Released	Original	Nasopharyngeal swab		B.1	G	Italy	Undertermined	No (couldn't determine the patient status as either pre-symptomatic / asymptomatic or symptomatic conclusively)
hCoV-19/Spain/ML-ISCIII-202449/2020	EPI_ISL_455347	14/03/2020	Europe / Spain / Melilla	Human		Male	26	Hospitalized	Original	Oropharyngeal swab		B.1.5	G	Spain	Undertermined	No (couldn't determine the patient status as either pre-symptomatic / asymptomatic or symptomatic conclusively)
hCoV-19/Spain/ML-ISCIII-202452/2020	EPI_ISL_455348	14/03/2020	Europe / Spain / Melilla	Human		Female	52	Hospitalized	Original	Oropharyngeal swab		B.1.5	G	Spain	Undertermined	No (couldn't determine the patient status as either pre-symptomatic / asymptomatic or symptomatic conclusively)
hCoV-19/Spain/ML-ISCIII-202454/2020	EPI_ISL_455349	14/03/2020	Europe / Spain / Melilla	Human		Female	60	Hospitalized	Original	Oropharyngeal swab		B.1.5	G	Spain	Undertermined	No (couldn't determine the patient status as either pre-symptomatic / asymptomatic or symptomatic conclusively)
hCoV-19/Spain/PV-ISCIII-201385/2020	EPI_ISL_455350	29/02/2020	Europe / Spain / Basque Country	Human		Female	43	Hospitalized	Original	Oropharyngeal swab		A.2	S	Spain	Undertermined	No (couldn't determine the patient status as either pre-symptomatic / asymptomatic or symptomatic conclusively)
hCoV-19/Spain/PV-ISCIII-201388/2020	EPI_ISL_455351	29/02/2020	Europe / Spain / Basque Country	Human		Female	77	Hospitalized	Original	Oropharyngeal swab		A.2	S	Spain	Undertermined	No (couldn't determine the patient status as either pre-symptomatic / asymptomatic or symptomatic conclusively)
hCoV-19/Spain/PV-ISCIII-201491/2020	EPI_ISL_455352	04/03/2020	Europe / Spain / Basque Country	Human		Male	83	Hospitalized	Original	Oropharyngeal swab		B.1	O	Spain	Undertermined	No (couldn't determine the patient status as either pre-symptomatic / asymptomatic or symptomatic conclusively)
hCoV-19/Spain/PV-ISCIII-201492/2020	EPI_ISL_455353	04/03/2020	Europe / Spain / Basque Country	Human		Female	88	Hospitalized	Original	Oropharyngeal swab		B.1	G	Spain	Undertermined	No (couldn't determine the patient status as either pre-symptomatic / asymptomatic or symptomatic conclusively)
hCoV-19/Spain/PV-ISCIII-201920/2020	EPI_ISL_455354	05/03/2020	Europe / Spain / Basque Country	Human		Female	55	Hospitalized	Original	Oropharyngeal swab		B.1	O	Spain	Undertermined	No (couldn't determine the patient status as either pre-symptomatic / asymptomatic or symptomatic conclusively)
hCoV-19/Nigeria/KW017-CV24/2020	EPI_ISL_455362	10/04/2020	Africa / Nigeria / Kwara State	Human		Male	41	Isolation	Original	Saliva and Nasopharyngeal Swab		B.1	GH	Nigeria	Undertermined	No (couldn't determine the patient status as either pre-symptomatic / asymptomatic or symptomatic conclusively)
hCoV-19/Nigeria/OG007-CV22/2020	EPI_ISL_455412	29/03/2020	Africa / Nigeria / Ogun State	Human		Female	30	Not Hospitalized	Original	Throat and Nasal Swab		B.2.1	V	Nigeria	Undertermined	No (couldn't determine the patient status as either pre-symptomatic / asymptomatic or symptomatic conclusively)
hCoV-19/Nigeria/OS016-CV3/2020	EPI_ISL_455413	27/03/2020	Africa / Nigeria / Osun State	Human		Male	25	Not Hospitalized	Original	Saliva and Nasal Swab		B.2.1	V	Nigeria	Undertermined	No (couldn't determine the patient status as either pre-symptomatic / asymptomatic or symptomatic conclusively)
hCoV-19/Nigeria/OS030-CV5/2020	EPI_ISL_455415	29/03/2020	Africa / Nigeria / Osun State	Human		Male	50	Not Hospitalized	Original			A	S	Nigeria	Undertermined	No (couldn't determine the patient status as either pre-symptomatic / asymptomatic or symptomatic conclusively)
hCoV-19/Nigeria/OS055-CV8/2020	EPI_ISL_455418	29/03/2020	Africa / Nigeria / Osun State	Human		Male	7	Not Hospitalized	Original	Saliva and Nasopharyngeal swab		A	S	Nigeria	Undertermined	No (couldn't determine the patient status as either pre-symptomatic / asymptomatic or symptomatic conclusively)
hCoV-19/Nigeria/OS060-CV9/2020	EPI_ISL_455419	29/03/2020	Africa / Nigeria / Osun State	Human		Male	48	Not Hospitalized	Original	Saliva and Nasopharyngeal swab		B.1	GH	Nigeria	Undertermined	No (couldn't determine the patient status as either pre-symptomatic / asymptomatic or symptomatic conclusively)
hCoV-19/Nigeria/OS070-CV11/2020	EPI_ISL_455422	29/03/2020	Africa / Nigeria / Osun State	Human		Male	21	Not Hospitalized	Original	Saliva and Nasopharyngeal swab		A	S	Nigeria	Undertermined	No (couldn't determine the patient status as either pre-symptomatic / asymptomatic or symptomatic conclusively)
hCoV-19/Nigeria/OS075-CV12/2020	EPI_ISL_455423	29/03/2020	Africa / Nigeria / Osun State	Human		Female	45	Not Hospitalized	Original	Saliva and Nasopharyngeal swab		A	S	Nigeria	Undertermined	No (couldn't determine the patient status as either pre-symptomatic / asymptomatic or symptomatic conclusively)
hCoV-19/Nigeria/OS085-CV14/2020	EPI_ISL_455424	29/03/2020	Africa / Nigeria / Osun State	Human		Female	45	Not Hospitalized	Original	Saliva and Nasopharyngeal swab		A	S	Nigeria	Undertermined	No (couldn't determine the patient status as either pre-symptomatic / asymptomatic or symptomatic conclusively)
hCoV-19/Nigeria/OS116-CV17/2020	EPI_ISL_455425	29/03/2020	Africa / Nigeria / Osun State	Human		Male	17	Not Hospitalized	Original	Saliva and Nasopharyngeal swab		B.1	S	Nigeria	Undertermined	No (couldn't determine the patient status as either pre-symptomatic / asymptomatic or symptomatic conclusively)
hCoV-19/Nigeria/OS122-CV18/2020	EPI_ISL_455426	29/03/2020	Africa / Nigeria / Osun State	Human		Female	38	Not Hospitalized	Original	Saliva and Nasopharyngeal swab		A	S	Nigeria	Undertermined	No (couldn't determine the patient status as either pre-symptomatic / asymptomatic or symptomatic conclusively)
hCoV-19/Nigeria/OS125-CV20/2020	EPI_ISL_455427	29/03/2020	Africa / Nigeria / Osun State	Human		Male	41	Not Hospitalized	Original	Saliva and Nasopharyngeal swab		A	S	Nigeria	Undertermined	No (couldn't determine the patient status as either pre-symptomatic / asymptomatic or symptomatic conclusively)
hCoV-19/Nigeria/OY008-CV29/2020	EPI_ISL_455429	27/03/2020	Africa / Nigeria / Oyo State	Human		Male	58	Not Hospitalized	Original	Saliva and Nasal swab		B.1	GH	Nigeria	Undertermined	No (couldn't determine the patient status as either pre-symptomatic / asymptomatic or symptomatic conclusively)
hCoV-19/Nigeria/OY035-CV34/2020	EPI_ISL_455430	02/04/2020	Africa / Nigeria / Oyo State	Human		Male	62	Hospitalized	Original	Nasopharyngeal swab		B.1.5	O	Nigeria	Undertermined	No (couldn't determine the patient status as either pre-symptomatic / asymptomatic or symptomatic conclusively)
hCoV-19/Nigeria/OY045A-CV35/2020	EPI_ISL_455431	02/04/2020	Africa / Nigeria / Oyo State	Human		Male	43	Not Hospitalized	Original	Saliva and Nasal swab		B.2.1	V	Nigeria	Undertermined	No (couldn't determine the patient status as either pre-symptomatic / asymptomatic or symptomatic conclusively)
hCoV-19/Mexico/DUR-InDRE-09/2020	EPI_ISL_455432	11/03/2020	North America / Mexico / Durango	Human	Sample from Gomez Palacios	Male	74	Deceased	Original	Oropharyngeal swab		A.1	S	Mexico	Symptomatic	Yes
hCoV-19/Mexico/GRO-InDRE-10/2020	EPI_ISL_455434	13/03/2020	North America / Mexico / Guerrero	Human	Sample from Acapulco de Juarez	Male	33	Released	Original	Oropharyngeal swab	Foreign patient from Argentina	B.1	GH	Mexico	Undertermined	No (couldn't determine the patient status as either pre-symptomatic / asymptomatic or symptomatic conclusively)
hCoV-19/Mexico/JAL-InDRE-11/2020	EPI_ISL_455435	20/03/2020	North America / Mexico / Jalisco	Human		Male	56	Deceased	Original	Oropharyngeal swab	Patient from Guadalajara	B	L	Mexico	Symptomatic	Yes
hCoV-19/Mexico/CMX-InDRE_12/2020	EPI_ISL_455436	12/03/2020	North America / Mexico / Mexico City	Human		Male	32	Released	Original	Oro-pharyngeal swab		B.1.5	G	Mexico	Undertermined	No (couldn't determine the patient status as either pre-symptomatic / asymptomatic or symptomatic conclusively)
hCoV-19/Mexico/CMX-InDRE-13/2020	EPI_ISL_455437	21/03/2020	North America / Mexico / Mexico City	Human		Male	56	Deceased	Original			B.1.5	G	Mexico	Symptomatic	Yes
hCoV-19/Mexico/ROO-InDRE-14/2020	EPI_ISL_455438	14/03/2020	North America / Mexico / Quintana Roo	Human		Male	45	Released	Original	Oropharyngeal swab		B.1.5	G	Mexico	Undertermined	No (couldn't determine the patient status as either pre-symptomatic / asymptomatic or symptomatic conclusively)
hCoV-19/Mexico/CMX-InDRE_15/2020	EPI_ISL_455439	11/03/2020	North America / Mexico / Mexico City	Human		Male	39	Released	Original	Oro-pharyngeal swab		A.1	S	Mexico	Undertermined	No (couldn't determine the patient status as either pre-symptomatic / asymptomatic or symptomatic conclusively)
hCoV-19/Mexico/JAL-InDRE-16/2020	EPI_ISL_455454	18/03/2020	North America / Mexico / Jalisco	Human		Male	58	Deceased	Original	Oropharyngeal swab		B	L	Mexico	Symptomatic	Yes
hCoV-19/Mexico/PUE-InDRE-17/2020	EPI_ISL_455455	14/03/2020	North America / Mexico / Puebla	Human		Female	32	Released	Original	Oropharyngeal swab		B.1.1	GR	Mexico	Undertermined	No (couldn't determine the patient status as either pre-symptomatic / asymptomatic or symptomatic conclusively)
hCoV-19/Mexico/QUE-InDRE-18/2020	EPI_ISL_455456	12/03/2020	North America / Mexico / Queretaro	Human		Female	44	Released	Original			A.5	S	Mexico	Undertermined	No (couldn't determine the patient status as either pre-symptomatic / asymptomatic or symptomatic conclusively)
hCoV-19/Yichun/JX2325/2020	EPI_ISL_455460	09/02/2020	Asia / China / Jiangxi / Yichun	Human		Male	26	Alive	Original			A	S	China	Undertermined	No (couldn't determine the patient status as either pre-symptomatic / asymptomatic or symptomatic conclusively)
hCoV-19/Yichun/JX2391/2020	EPI_ISL_455461	06/02/2020	Asia / China / Jiangxi / Yichun	Human		Male	31	Alive	Original			A	S	China	Undertermined	No (couldn't determine the patient status as either pre-symptomatic / asymptomatic or symptomatic conclusively)
hCoV-19/Fuzhou/JX2012/2020	EPI_ISL_455462	05/02/2020	Asia / China / Jiangxi / Fuzhou	Human		Female	24	Alive	Original			A	S	China	Undertermined	No (couldn't determine the patient status as either pre-symptomatic / asymptomatic or symptomatic conclusively)
hCoV-19/Jiujiang/JX490/2020	EPI_ISL_455463	03/02/2020	Asia / China / Jiangxi / Jiujiang	Human		Male	75	Alive	Original			B	L	China	Undertermined	No (couldn't determine the patient status as either pre-symptomatic / asymptomatic or symptomatic conclusively)
hCoV-19/Fuzhou/JX2009/2020	EPI_ISL_455464	05/02/2020	Asia / China / Jiangxi / Fuzhou	Human		Female	45	Alive	Original			A	S	China	Undertermined	No (couldn't determine the patient status as either pre-symptomatic / asymptomatic or symptomatic conclusively)
hCoV-19/Fuzhou/JX2019/2020	EPI_ISL_455465	10/02/2020	Asia / China / Jiangxi / Fuzhou	Human		Male	51	Alive	Original			B.2	O	China	Undertermined	No (couldn't determine the patient status as either pre-symptomatic / asymptomatic or symptomatic conclusively)
hCoV-19/Yingtan/JX2480/2020	EPI_ISL_455466	01/02/2020	Asia / China / Jiangxi / Yingtan	Human		Male	66	Alive	Original			B	L	China	Undertermined	No (couldn't determine the patient status as either pre-symptomatic / asymptomatic or symptomatic conclusively)
hCoV-19/Yichun/JX2424/2020	EPI_ISL_455467	12/02/2020	Asia / China / Jiangxi / Yichun	Human		Female	53	Alive	Original			A	S	China	Undertermined	No (couldn't determine the patient status as either pre-symptomatic / asymptomatic or symptomatic conclusively)
hCoV-19/Romania/284213/2020	EPI_ISL_455468	13/05/2020	Europe / Romania / Bucuresti	Human		Female	75	Hospitalized	Original	Naso-pharyngeal swab		B.1	G	Romania	Undertermined	No (couldn't determine the patient status as either pre-symptomatic / asymptomatic or symptomatic conclusively)
hCoV-19/Romania/283584/2020	EPI_ISL_455469	12/05/2020	Europe / Romania / Bucuresti	Human		Female	65	Hospitalized	Original	Naso-pharyngeal swab		B.1	G	Romania	Undertermined	No (couldn't determine the patient status as either pre-symptomatic / asymptomatic or symptomatic conclusively)
hCoV-19/Romania/284489/2020	EPI_ISL_455470	14/05/2020	Europe / Romania / Bucuresti	Human		Female	23	Hospitalized	Original	Naso-pharyngeal swab		B.1.1	GR	Romania	Undertermined	No (couldn't determine the patient status as either pre-symptomatic / asymptomatic or symptomatic conclusively)
hCoV-19/Romania/284508/2020	EPI_ISL_455471	14/05/2020	Europe / Romania / Bucuresti	Human		Female	49	Hospitalized	Original			B.1	G	Romania	Undertermined	No (couldn't determine the patient status as either pre-symptomatic / asymptomatic or symptomatic conclusively)
hCoV-19/Romania/284168/2020	EPI_ISL_455472	13/05/2020	Europe / Romania / Buzau	Human		Male	46	Hospitalized	Original	Naso-pharyngeal swab		B.1.42	G	Romania	Undertermined	No (couldn't determine the patient status as either pre-symptomatic / asymptomatic or symptomatic conclusively)
hCoV-19/Romania/284056/2020	EPI_ISL_455473	14/05/2020	Europe / Romania / Arges	Human		Male	46	Hospitalized	Original			B.1.1	GR	Romania	Undertermined	No (couldn't determine the patient status as either pre-symptomatic / asymptomatic or symptomatic conclusively)
hCoV-19/Romania/284783/2020	EPI_ISL_455474	14/05/2020	Europe / Romania / Arges	Human		Female	46	Hospitalized	Original	Naso-pharyngeal swab		B.1.1	GR	Romania	Undertermined	No (couldn't determine the patient status as either pre-symptomatic / asymptomatic or symptomatic conclusively)
hCoV-19/Romania/284762/2020	EPI_ISL_455475	14/05/2020	Europe / Romania / Arges	Human		Female	40	Hospitalized	Original			B.1.1	GR	Romania	Undertermined	No (couldn't determine the patient status as either pre-symptomatic / asymptomatic or symptomatic conclusively)
hCoV-19/Romania/285388/2020	EPI_ISL_455476	17/05/2020	Europe / Romania / Bucuresti	Human		Male	82	Hospitalized	Original	Naso-pharyngeal swab		B.1	G	Romania	Undertermined	No (couldn't determine the patient status as either pre-symptomatic / asymptomatic or symptomatic conclusively)
hCoV-19/Romania/284371/2020	EPI_ISL_455477	13/05/2020	Europe / Romania / Bucuresti	Human		Female	26	Hospitalized	Original	Naso-pharyngeal swab		B.1.1	GR	Romania	Undertermined	No (couldn't determine the patient status as either pre-symptomatic / asymptomatic or symptomatic conclusively)
hCoV-19/Romania/284468/2020	EPI_ISL_455479	14/05/2020	Europe / Romania / Bucuresti	Human		Female	40	Hospitalized	Original			B.1.1	GR	Romania	Undertermined	No (couldn't determine the patient status as either pre-symptomatic / asymptomatic or symptomatic conclusively)
hCoV-19/Croatia/17-S7new/2020	EPI_ISL_455566	30/03/2020	Europe / Croatia / Istria	Human		Male	51	Live	Original	Oro-pharyngeal swab		B.1	G	Croatia	Undertermined	No (couldn't determine the patient status as either pre-symptomatic / asymptomatic or symptomatic conclusively)
hCoV-19/Croatia/7R-S8new/2020	EPI_ISL_455567	29/03/2020	Europe / Croatia / Istria	Human		Male	58	Live	Original			B.1	GH	Croatia	Undertermined	No (couldn't determine the patient status as either pre-symptomatic / asymptomatic or symptomatic conclusively)
hCoV-19/Italy/LOM-INMI-BG-5825/2020	EPI_ISL_460079	22/02/2020	Europe / Italy / Lombardia	Human		Male	83	Hospitalized	Original	Nasopharyngeal swab		B.1	G	Italy	Undertermined	No (couldn't determine the patient status as either pre-symptomatic / asymptomatic or symptomatic conclusively)
hCoV-19/Italy/LOM-INMI-BG-11639/2020	EPI_ISL_460080	28/02/2020	Europe / Italy / Lombardia	Human		Male	87	Hospitalized	Original	Nasopharyngeal swab		B.1.1	GR	Italy	Undertermined	No (couldn't determine the patient status as either pre-symptomatic / asymptomatic or symptomatic conclusively)
hCoV-19/Italy/LOM-INMI-BG-7158/2020	EPI_ISL_460081	24/02/2020	Europe / Italy / Lombardia	Human		Male	65	Hospitalized	Original	Nasopharyngeal swab		B.1.1	GR	Italy	Undertermined	No (couldn't determine the patient status as either pre-symptomatic / asymptomatic or symptomatic conclusively)
hCoV-19/Italy/LOM-INMI-BG-9209/2020	EPI_ISL_460082	26/02/2020	Europe / Italy / Lombardia	Human		Male	87	Hospitalized	Original	Nasopharyngeal swab		B.1.5	G	Italy	Undertermined	No (couldn't determine the patient status as either pre-symptomatic / asymptomatic or symptomatic conclusively)
hCoV-19/Italy/LOM-INMI-BG-9225/2020	EPI_ISL_460083	26/02/2020	Europe / Italy / Lombardia	Human		Female	73	Hospitalized	Original	Alveolar lavage fluid		B.1	G	Italy	Undertermined	No (couldn't determine the patient status as either pre-symptomatic / asymptomatic or symptomatic conclusively)
hCoV-19/Italy/LOM-INMI-BS-12256/2020	EPI_ISL_460084	29/02/2020	Europe / Italy / Lombardia	Human		Female	89	Hospitalized	Original	Nasopharyngeal swab		B.1.5	G	Italy	Undertermined	No (couldn't determine the patient status as either pre-symptomatic / asymptomatic or symptomatic conclusively)
hCoV-19/Italy/LOM-INMI-13221/2020	EPI_ISL_460085	01/03/2020	Europe / Italy / Lombardia	Human		Female	45	Hospitalized	Original	Nasopharyngeal swab		B.1	G	Italy	Undertermined	No (couldn't determine the patient status as either pre-symptomatic / asymptomatic or symptomatic conclusively)
hCoV-19/Italy/LOM-INMI-7070/2020	EPI_ISL_460086	24/02/2020	Europe / Italy / Lombardia	Human		Male	60	Hospitalized	Original	Alveolar lavage fluid		B.1	G	Italy	Undertermined	No (couldn't determine the patient status as either pre-symptomatic / asymptomatic or symptomatic conclusively)
hCoV-19/Italy/LOM-INMI-9206/2020	EPI_ISL_460087	26/02/2020	Europe / Italy / Lombardia	Human		Female	44	Hospitalized	Original	Nasopharyngeal swab		B.1	G	Italy	Undertermined	No (couldn't determine the patient status as either pre-symptomatic / asymptomatic or symptomatic conclusively)
hCoV-19/Italy/LOM-INMI-9214/2020	EPI_ISL_460088	26/02/2020	Europe / Italy / Lombardia	Human		Male	78	Hospitalized	Original	Nasopharyngeal swab		B.1	G	Italy	Undertermined	No (couldn't determine the patient status as either pre-symptomatic / asymptomatic or symptomatic conclusively)
hCoV-19/Italy/LOM-INMI-9491/2020	EPI_ISL_460089	26/02/2020	Europe / Italy / Lombardia	Human		Male	88	Hospitalized	Original	Nasopharyngeal swab		B.1.1	GR	Italy	Undertermined	No (couldn't determine the patient status as either pre-symptomatic / asymptomatic or symptomatic conclusively)
hCoV-19/Italy/LOM-INMI-5925/2020	EPI_ISL_460091	22/02/2020	Europe / Italy / Lombardia	Human		Male	37	Hospitalized	Original	Nasopharyngeal swab		B.1	G	Italy	Undertermined	No (couldn't determine the patient status as either pre-symptomatic / asymptomatic or symptomatic conclusively)
hCoV-19/Italy/LOM-INMI-11951/2020	EPI_ISL_460092	28/02/2020	Europe / Italy / Lombardia	Human		Male	80	Hospitalized	Original	Nasopharyngeal swab		B.1.5	G	Italy	Undertermined	No (couldn't determine the patient status as either pre-symptomatic / asymptomatic or symptomatic conclusively)
hCoV-19/Italy/LOM-INMI-12578/2020	EPI_ISL_460093	29/02/2020	Europe / Italy / Lombardia	Human		Male	1	Hospitalized	Original	Nasopharyngeal swab		B.1	G	Italy	Undertermined	No (couldn't determine the patient status as either pre-symptomatic / asymptomatic or symptomatic conclusively)
hCoV-19/Italy/LOM-INMI-8058/2020	EPI_ISL_460094	25/02/2020	Europe / Italy / Lombardia	Human		Male	78	Hospitalized	Original	Nasopharyngeal swab		B.1	G	Italy	Undertermined	No (couldn't determine the patient status as either pre-symptomatic / asymptomatic or symptomatic conclusively)
hCoV-19/Bosnia and Herzegovina/03_Tuzla/2020	EPI_ISL_463893	25/05/2020	Europe / Bosnia and Herzegovina / Tuzla	Human		Male	51	Rebased	Original	Oro-pharyngeal swab		B.1	G	Bosnia and Herzegovina	Undertermined	No (couldn't determine the patient status as either pre-symptomatic / asymptomatic or symptomatic conclusively)
hCoV-19/Brazil/RJ-2422/2020	EPI_ISL_467352	20/04/2020	South America / Brazil / Rio de Janeiro / Queimados	Human	unknown	Female	27	inpatient	Original	Nasopharyngeal swab	unknown	B.1.1.33	GR	Brazil	Undertermined	No (couldn't determine the patient status as either pre-symptomatic / asymptomatic or symptomatic conclusively)
hCoV-19/Brazil/RJ-2669/2020	EPI_ISL_467353	21/04/2020	South America / Brazil / Rio de Janeiro / Niteroi	Human	unknown	Female	25	outpatient	Original	Nasopharyngeal swab	unknown	B.1.1.33	GR	Brazil	Undertermined	No (couldn't determine the patient status as either pre-symptomatic / asymptomatic or symptomatic conclusively)
hCoV-19/Brazil/RJ-2822/2020	EPI_ISL_467367	27/04/2020	South America / Brazil / Rio de Janeiro / Niteroi	Human	unknown	Male	62	inpatient	Original	Nasopharyngeal swab	unknown	B.1.1.33	GR	Brazil	Undertermined	No (couldn't determine the patient status as either pre-symptomatic / asymptomatic or symptomatic conclusively)
hCoV-19/Brazil/RJ-2844/2020	EPI_ISL_467369	28/04/2020	South America / Brazil / Rio de Janeiro	Human	unknown	Female	29	outpatient	Original	Nasopharyngeal swab	unknown	B.1.1.33	GR	Brazil	Undertermined	No (couldn't determine the patient status as either pre-symptomatic / asymptomatic or symptomatic conclusively)
hCoV-19/Indonesia/KI-EIJK-05/2020	EPI_ISL_467374	18/03/2020	Asia / Indonesia / East Kalimantan / Samarinda	Human		Female	31	Hospitalized	Original	Nasopharyngeal and oropharyngeal swabs		B	L	Indonesia	Undertermined	No (couldn't determine the patient status as either pre-symptomatic / asymptomatic or symptomatic conclusively)
hCoV-19/Indonesia/SA-EIJK-06/2020	EPI_ISL_467375	23/03/2020	Asia / Indonesia / North Sulawesi / Manado	Human		Female	70	Hospitalized	Original	Nasopharyngeal and oropharyngeal swabs	Probable imported case from Indonesia/Jakarta based on patient travel history	B	L	Indonesia	Undertermined	No (couldn't determine the patient status as either pre-symptomatic / asymptomatic or symptomatic conclusively)
hCoV-19/Indonesia/JK-EIJK-07/2020	EPI_ISL_467376	24/04/2020	Asia / Indonesia / Jakarta	Human		Male	74	Hospitalized	Original	Nasopharyngeal and oropharyngeal swabs		B	L	Indonesia	Undertermined	No (couldn't determine the patient status as either pre-symptomatic / asymptomatic or symptomatic conclusively)
hCoV-19/Spain/AR-IBV-2466/2020	EPI_ISL_468765	17/03/2020	Europe / Spain / Aragon / Zaragoza	Human		Female	67	Cured	Original			B.2	V	Spain	Undertermined	No (couldn't determine the patient status as either pre-symptomatic / asymptomatic or symptomatic conclusively)
hCoV-19/Spain/AR-IBV-2468/2020	EPI_ISL_468766	27/03/2020	Europe / Spain / Aragon / Zaragoza	Human		Female	62	Cured	Original			A.5	S	Spain	Undertermined	No (couldn't determine the patient status as either pre-symptomatic / asymptomatic or symptomatic conclusively)
hCoV-19/Spain/AR-IBV-2404/2020	EPI_ISL_468767	24/03/2020	Europe / Spain / Aragon / Teruel	Human		Male	55	Cured	Original			B.1.5	G	Spain	Undertermined	No (couldn't determine the patient status as either pre-symptomatic / asymptomatic or symptomatic conclusively)
hCoV-19/Spain/AR-IBV-2416/2020	EPI_ISL_468768	21/03/2020	Europe / Spain / Aragon / Zaragoza	Human		Female	61	Cured	Original			B.2	V	Spain	Undertermined	No (couldn't determine the patient status as either pre-symptomatic / asymptomatic or symptomatic conclusively)
hCoV-19/Spain/AR-IBV-2487/2020	EPI_ISL_468771	24/03/2020	Europe / Spain / Aragon / Zaragoza	Human		Female	32	Cured	Original			B.1.5	G	Spain	Undertermined	No (couldn't determine the patient status as either pre-symptomatic / asymptomatic or symptomatic conclusively)
hCoV-19/Spain/AR-IBV-2414/2020	EPI_ISL_468772	24/03/2020	Europe / Spain / Aragon / Teruel	Human		Female	82	Cured	Original			B.1.5	G	Spain	Undertermined	No (couldn't determine the patient status as either pre-symptomatic / asymptomatic or symptomatic conclusively)
hCoV-19/Spain/AR-IBV-2454/2020	EPI_ISL_468774	24/03/2020	Europe / Spain / Aragon / Teruel	Human		Male	82	Cured	Original			B.2	V	Spain	Undertermined	No (couldn't determine the patient status as either pre-symptomatic / asymptomatic or symptomatic conclusively)
hCoV-19/Spain/AR-IBV-2475/2020	EPI_ISL_468775	24/03/2020	Europe / Spain / Aragon / Zaragoza	Human		Male	43	Cured	Original			B.1	GH	Spain	Undertermined	No (couldn't determine the patient status as either pre-symptomatic / asymptomatic or symptomatic conclusively)
hCoV-19/Spain/AR-IBV-2442/2020	EPI_ISL_468776	24/03/2020	Europe / Spain / Aragon / Zaragoza	Human		Male	74	Cured	Original			A.2	S	Spain	Undertermined	No (couldn't determine the patient status as either pre-symptomatic / asymptomatic or symptomatic conclusively)
hCoV-19/Spain/AR-IBV-2435/2020	EPI_ISL_468777	23/03/2020	Europe / Spain / Aragon / Zaragoza	Human		Female	53	Cured	Original			B.1.1	GR	Spain	Undertermined	No (couldn't determine the patient status as either pre-symptomatic / asymptomatic or symptomatic conclusively)
hCoV-19/Spain/AR-IBV-2485/2020	EPI_ISL_468780	24/03/2020	Europe / Spain / Aragon / Zaragoza	Human		Male	63	Cured	Original			B.2	V	Spain	Undertermined	No (couldn't determine the patient status as either pre-symptomatic / asymptomatic or symptomatic conclusively)
hCoV-19/Spain/AR-IBV-2409/2020	EPI_ISL_468782	27/03/2020	Europe / Spain / Aragon / Zaragoza	Human		Female	84	Cured	Original			B.1.5	G	Spain	Undertermined	No (couldn't determine the patient status as either pre-symptomatic / asymptomatic or symptomatic conclusively)
hCoV-19/Spain/AR-IBV-2488/2020	EPI_ISL_468787	24/03/2020	Europe / Spain / Aragon / Zaragoza	Human		Female	46	Cured	Original			B.1.1	GR	Spain	Undertermined	No (couldn't determine the patient status as either pre-symptomatic / asymptomatic or symptomatic conclusively)
hCoV-19/Spain/AR-IBV-2432/2020	EPI_ISL_468788	24/03/2020	Europe / Spain / Aragon / Zaragoza	Human		Female	39	Cured	Original			B.1.1	GR	Spain	Undertermined	No (couldn't determine the patient status as either pre-symptomatic / asymptomatic or symptomatic conclusively)
hCoV-19/Spain/AR-IBV-2458/2020	EPI_ISL_468791	26/03/2020	Europe / Spain / Aragon / Zaragoza	Human		Female	42	Cured	Original			B.1.5	G	Spain	Undertermined	No (couldn't determine the patient status as either pre-symptomatic / asymptomatic or symptomatic conclusively)
hCoV-19/Spain/AR-IBV-2429/2020	EPI_ISL_468793	27/03/2020	Europe / Spain / Aragon / Zaragoza	Human		Male	61	Cured	Original			B.1.5	G	Spain	Undertermined	No (couldn't determine the patient status as either pre-symptomatic / asymptomatic or symptomatic conclusively)
hCoV-19/Spain/AR-IBV-2398/2020	EPI_ISL_468795	26/03/2020	Europe / Spain / Aragon / Alcaniz	Human		Female	57	Cured	Original			B.1.5	G	Spain	Undertermined	No (couldn't determine the patient status as either pre-symptomatic / asymptomatic or symptomatic conclusively)

hCoV-19/Spain/AR-IBV-2415/2020	EPI_ISL_468798	23/03/2020	Europe / Spain / Aragon / Zaragoza	Human		Female	50	Cured	Original		A.5	S	Spain	Undertermined	No (couldn't determine the patient status as either pre-symptomatic / asymptomatic or symptomatic conclusively)
hCoV-19/Spain/AR-IBV-2390/2020	EPI_ISL_468800	27/03/2020	Europe / Spain / Aragon / Alcaniz	Human		Male	35	Cured	Original		B.1.5	G	Spain	Undertermined	No (couldn't determine the patient status as either pre-symptomatic / asymptomatic or symptomatic conclusively)
hCoV-19/Spain/AR-IBV-2467/2020	EPI_ISL_468801	18/03/2020	Europe / Spain / Aragon / Zaragoza	Human		Female	36	Cured	Original		B.1.5	G	Spain	Undertermined	No (couldn't determine the patient status as either pre-symptomatic / asymptomatic or symptomatic conclusively)
hCoV-19/Spain/AR-IBV-2486/2020	EPI_ISL_468804	24/03/2020	Europe / Spain / Aragon / Zaragoza	Human		Female	23	Cured	Original		A.5	S	Spain	Undertermined	No (couldn't determine the patient status as either pre-symptomatic / asymptomatic or symptomatic conclusively)
hCoV-19/Spain/AR-IBV-2428/2020	EPI_ISL_468805	26/03/2020	Europe / Spain / Aragon / Zaragoza	Human		Female	47	Cured	Original		B.1.5	G	Spain	Undertermined	No (couldn't determine the patient status as either pre-symptomatic / asymptomatic or symptomatic conclusively)
hCoV-19/Spain/AR-IBV-2445/2020	EPI_ISL_468808	23/03/2020	Europe / Spain / Aragon / Zaragoza	Human		Female	54	Cured	Original		B.2	V	Spain	Undertermined	No (couldn't determine the patient status as either pre-symptomatic / asymptomatic or symptomatic conclusively)
hCoV-19/Spain/AR-IBV-2441/2020	EPI_ISL_468809	28/03/2020	Europe / Spain / Aragon / Teruel	Human		Male	77	Cured	Original		B.1.5	G	Spain	Undertermined	No (couldn't determine the patient status as either pre-symptomatic / asymptomatic or symptomatic conclusively)
hCoV-19/Spain/AR-IBV-2465/2020	EPI_ISL_468810	24/03/2020	Europe / Spain / Aragon / Zaragoza	Human		Female	60	Cured	Original		B.1.5	G	Spain	Undertermined	No (couldn't determine the patient status as either pre-symptomatic / asymptomatic or symptomatic conclusively)
hCoV-19/Spain/AR-IBV-2438/2020	EPI_ISL_468812	26/03/2020	Europe / Spain / Aragon / Zaragoza	Human		Female	40	Cured	Original		B.2	V	Spain	Undertermined	No (couldn't determine the patient status as either pre-symptomatic / asymptomatic or symptomatic conclusively)
hCoV-19/Spain/AR-IBV-2464/2020	EPI_ISL_468814	24/03/2020	Europe / Spain / Aragon / Teruel	Human		Female	59	Cured	Original		B.2	V	Spain	Undertermined	No (couldn't determine the patient status as either pre-symptomatic / asymptomatic or symptomatic conclusively)
hCoV-19/Spain/AR-IBV-2422/2020	EPI_ISL_468815	24/03/2020	Europe / Spain / Aragon / Zaragoza	Human		Male	39	Cured	Original		B.1.5	G	Spain	Undertermined	No (couldn't determine the patient status as either pre-symptomatic / asymptomatic or symptomatic conclusively)
hCoV-19/Spain/AR-IBV-2425/2020	EPI_ISL_468817	23/03/2020	Europe / Spain / Aragon / Zaragoza	Human		Female	57	Cured	Original		A.5	S	Spain	Undertermined	No (couldn't determine the patient status as either pre-symptomatic / asymptomatic or symptomatic conclusively)
hCoV-19/Spain/AR-IBV-2421/2020	EPI_ISL_468820	28/03/2020	Europe / Spain / Aragon / Teruel	Human		Male	81	Cured	Original		B.2	V	Spain	Undertermined	No (couldn't determine the patient status as either pre-symptomatic / asymptomatic or symptomatic conclusively)
hCoV-19/Spain/AR-IBV-2434/2020	EPI_ISL_468829	24/03/2020	Europe / Spain / Aragon / Teruel	Human		Female	57	Cured	Original		B.1.5	G	Spain	Undertermined	No (couldn't determine the patient status as either pre-symptomatic / asymptomatic or symptomatic conclusively)
hCoV-19/Spain/AR-IBV-2407/2020	EPI_ISL_468830	17/03/2020	Europe / Spain / Aragon / Teruel	Human		Female	56	Cured	Original		B.2	V	Spain	Undertermined	No (couldn't determine the patient status as either pre-symptomatic / asymptomatic or symptomatic conclusively)
hCoV-19/Spain/AR-IBV-2408/2020	EPI_ISL_468832	26/03/2020	Europe / Spain / Aragon / Alcaniz	Human		Female	44	Cured	Original		B.1.5	G	Spain	Undertermined	No (couldn't determine the patient status as either pre-symptomatic / asymptomatic or symptomatic conclusively)
hCoV-19/Spain/AR-IBV-2397/2020	EPI_ISL_468834	17/03/2020	Europe / Spain / Aragon / Zaragoza	Human		Male	25	Cured	Original		A.5	S	Spain	Undertermined	No (couldn't determine the patient status as either pre-symptomatic / asymptomatic or symptomatic conclusively)
hCoV-19/Spain/AR-IBV-2418/2020	EPI_ISL_468835	26/03/2020	Europe / Spain / Aragon / Alcaniz	Human		Female	55	Cured	Original		B.1.5	G	Spain	Undertermined	No (couldn't determine the patient status as either pre-symptomatic / asymptomatic or symptomatic conclusively)
hCoV-19/Spain/AR-IBV-2431/2020	EPI_ISL_468839	28/03/2020	Europe / Spain / Aragon / Teruel	Human		Male	84	Cured	Original		B.1.5	G	Spain	Undertermined	No (couldn't determine the patient status as either pre-symptomatic / asymptomatic or symptomatic conclusively)
hCoV-19/Spain/AR-IBV-2426/2020	EPI_ISL_468841	21/03/2020	Europe / Spain / Aragon / Zaragoza	Human		Male	39	Cured	Original		B.1.5	G	Spain	Undertermined	No (couldn't determine the patient status as either pre-symptomatic / asymptomatic or symptomatic conclusively)
hCoV-19/Spain/AR-IBV-2443/2020	EPI_ISL_468848	24/03/2020	Europe / Spain / Aragon / Zaragoza	Human		Male	89	Cured	Original		A.5	S	Spain	Undertermined	No (couldn't determine the patient status as either pre-symptomatic / asymptomatic or symptomatic conclusively)
hCoV-19/Spain/AR-IBV-2439/2020	EPI_ISL_468849	27/03/2020	Europe / Spain / Aragon / Alcaniz	Human		Female	42	Cured	Original		B.1.5	G	Spain	Undertermined	No (couldn't determine the patient status as either pre-symptomatic / asymptomatic or symptomatic conclusively)
hCoV-19/Spain/AR-IBV-2396/2020	EPI_ISL_468851	24/03/2020	Europe / Spain / Aragon / Zaragoza	Human		Female	29	Cured	Original		B.1.5	G	Spain	Undertermined	No (couldn't determine the patient status as either pre-symptomatic / asymptomatic or symptomatic conclusively)
hCoV-19/Spain/AR-IBV-2477/2020	EPI_ISL_468855	26/03/2020	Europe / Spain / Aragon / Zaragoza	Human		Male	30	Cured	Original		A.5	S	Spain	Undertermined	No (couldn't determine the patient status as either pre-symptomatic / asymptomatic or symptomatic conclusively)
hCoV-19/Spain/AR-IBV-2482/2020	EPI_ISL_468857	23/03/2020	Europe / Spain / Aragon / Zaragoza	Human		Female	44	Cured	Original		A.2	S	Spain	Undertermined	No (couldn't determine the patient status as either pre-symptomatic / asymptomatic or symptomatic conclusively)
hCoV-19/Spain/AR-IBV-2412/2020	EPI_ISL_468858	24/03/2020	Europe / Spain / Aragon / Zaragoza	Human		Female	47	Cured	Original		B.1.5	G	Spain	Undertermined	No (couldn't determine the patient status as either pre-symptomatic / asymptomatic or symptomatic conclusively)
hCoV-19/Spain/AR-IBV-2423/2020	EPI_ISL_468859	24/03/2020	Europe / Spain / Aragon / Zaragoza	Human		Female	53	Cured	Original		B.1	GH	Spain	Undertermined	No (couldn't determine the patient status as either pre-symptomatic / asymptomatic or symptomatic conclusively)
hCoV-19/USA/CA-CSMC143/2020	EPI_ISL_475622	06/04/2020	North America / USA / California / Los Angeles	Human		Male	39	Released	Original		B.1.2	GH	USA	Undertermined	No (couldn't determine the patient status as either pre-symptomatic / asymptomatic or symptomatic conclusively)
hCoV-19/USA/CA-CSMC144/2020	EPI_ISL_475623	08/04/2020	North America / USA / California / Los Angeles	Human		Female	58	Outpatient	Original		B.1	GH	USA	Undertermined	No (couldn't determine the patient status as either pre-symptomatic / asymptomatic or symptomatic conclusively)
hCoV-19/USA/CA-CSMC15/2020	EPI_ISL_475624	28/03/2020	North America / USA / California / Los Angeles	Human		Male	45	Released	Original		B.1	GH	USA	Undertermined	No (couldn't determine the patient status as either pre-symptomatic / asymptomatic or symptomatic conclusively)
hCoV-19/USA/CA-CSMC16/2020	EPI_ISL_475625	13/04/2020	North America / USA / California / Los Angeles	Human		Female	87	Deceased	Original		B.1	GH	USA	Symptomatic	Yes
hCoV-19/USA/CA-CSMC17/2020	EPI_ISL_475626	28/03/2020	North America / USA / California / Los Angeles	Human		Male	63	Released	Original		B.1	GH	USA	Undertermined	No (couldn't determine the patient status as either pre-symptomatic / asymptomatic or symptomatic conclusively)
hCoV-19/USA/CA-CSMC18/2020	EPI_ISL_475627	25/03/2020	North America / USA / California / Los Angeles	Human		Male	45	Released	Original		B.1	GH	USA	Undertermined	No (couldn't determine the patient status as either pre-symptomatic / asymptomatic or symptomatic conclusively)
hCoV-19/USA/CA-CSMC51/2020	EPI_ISL_475664	06/04/2020	North America / USA / California / Los Angeles	Human		Female	34	Released	Original		A.1	S	USA	Undertermined	No (couldn't determine the patient status as either pre-symptomatic / asymptomatic or symptomatic conclusively)
hCoV-19/USA/CA-CSMC52/2020	EPI_ISL_475665	06/04/2020	North America / USA / California / Los Angeles	Human		Female	73	Released	Original		B.1	GH	USA	Undertermined	No (couldn't determine the patient status as either pre-symptomatic / asymptomatic or symptomatic conclusively)
hCoV-19/USA/CA-CSMC53/2020	EPI_ISL_475666	01/04/2020	North America / USA / California / Los Angeles	Human		Female	59	Released	Original		B	O	USA	Undertermined	No (couldn't determine the patient status as either pre-symptomatic / asymptomatic or symptomatic conclusively)
hCoV-19/USA/CA-CSMC54/2020	EPI_ISL_475667	07/04/2020	North America / USA / California / Los Angeles	Human		Male	98	Deceased	Original		B.1.43	GH	USA	Symptomatic	Yes
hCoV-19/USA/CA-CSMC55/2020	EPI_ISL_475668	07/04/2020	North America / USA / California / Los Angeles	Human		Female	28	Outpatient	Original		B.1.43	GH	USA	Undertermined	No (couldn't determine the patient status as either pre-symptomatic / asymptomatic or symptomatic conclusively)
hCoV-19/USA/CA-CSMC56/2020	EPI_ISL_475669	01/04/2020	North America / USA / California / Los Angeles	Human		Male	32	Outpatient	Original		B.4	O	USA	Undertermined	No (couldn't determine the patient status as either pre-symptomatic / asymptomatic or symptomatic conclusively)
hCoV-19/USA/CA-CSMC57/2020	EPI_ISL_475670	01/04/2020	North America / USA / California / Los Angeles	Human		Male	71	Still hospitalized	Original		B.1	GH	USA	Undertermined	No (couldn't determine the patient status as either pre-symptomatic / asymptomatic or symptomatic conclusively)
hCoV-19/USA/CA-CSMC58/2020	EPI_ISL_475671	01/04/2020	North America / USA / California / Los Angeles	Human		Male	67	Released	Original		B.1.1	GR	USA	Undertermined	No (couldn't determine the patient status as either pre-symptomatic / asymptomatic or symptomatic conclusively)
hCoV-19/USA/CA-CSMC59/2020	EPI_ISL_475672	13/04/2020	North America / USA / California / Los Angeles	Human		Male	31	Outpatient	Original		B.1	GH	USA	Undertermined	No (couldn't determine the patient status as either pre-symptomatic / asymptomatic or symptomatic conclusively)
hCoV-19/USA/CA-CSMC6/2020	EPI_ISL_475673	27/03/2020	North America / USA / California / Los Angeles	Human		Male	41	Released	Original		B.1	GH	USA	Undertermined	No (couldn't determine the patient status as either pre-symptomatic / asymptomatic or symptomatic conclusively)
hCoV-19/USA/CA-CSMC60/2020	EPI_ISL_475674	01/04/2020	North America / USA / California / Los Angeles	Human		Female	71	Still hospitalized	Original		B.1.43	GH	USA	Undertermined	No (couldn't determine the patient status as either pre-symptomatic / asymptomatic or symptomatic conclusively)
hCoV-19/USA/CA-CSMC61/2020	EPI_ISL_475675	01/04/2020	North America / USA / California / Los Angeles	Human		Male	92	Released	Original		B.1.1	GR	USA	Undertermined	No (couldn't determine the patient status as either pre-symptomatic / asymptomatic or symptomatic conclusively)
hCoV-19/USA/CA-CSMC62/2020	EPI_ISL_475676	01/04/2020	North America / USA / California / Los Angeles	Human		Male	47	Deceased	Original		B.1	GH	USA	Symptomatic	Yes
hCoV-19/USA/CA-CSMC63/2020	EPI_ISL_475677	28/03/2020	North America / USA / California / Los Angeles	Human		Female	48	Released	Original		B.1	GH	USA	Undertermined	No (couldn't determine the patient status as either pre-symptomatic / asymptomatic or symptomatic conclusively)
hCoV-19/USA/CA-CSMC64/2020	EPI_ISL_475678	28/03/2020	North America / USA / California / Los Angeles	Human		Male	77	Released	Original		B.1	GH	USA	Undertermined	No (couldn't determine the patient status as either pre-symptomatic / asymptomatic or symptomatic conclusively)
hCoV-19/USA/CA-CSMC65/2020	EPI_ISL_475679	28/03/2020	North America / USA / California / Los Angeles	Human		Male	33	Released	Original		B.1	GH	USA	Undertermined	No (couldn't determine the patient status as either pre-symptomatic / asymptomatic or symptomatic conclusively)
hCoV-19/USA/CA-CSMC66/2020	EPI_ISL_475680	28/03/2020	North America / USA / California / Los Angeles	Human		Female	36	Outpatient	Original		B.1.1	GR	USA	Undertermined	No (couldn't determine the patient status as either pre-symptomatic / asymptomatic or symptomatic conclusively)
hCoV-19/USA/CA-CSMC67/2020	EPI_ISL_475681	28/03/2020	North America / USA / California / Los Angeles	Human		Female	88	Released	Original		B	L	USA	Undertermined	No (couldn't determine the patient status as either pre-symptomatic / asymptomatic or symptomatic conclusively)
hCoV-19/USA/CA-CSMC68/2020	EPI_ISL_475682	28/03/2020	North America / USA / California / Los Angeles	Human		Male	73	Released	Original		B.1	G	USA	Undertermined	No (couldn't determine the patient status as either pre-symptomatic / asymptomatic or symptomatic conclusively)
hCoV-19/USA/CA-CSMC69/2020	EPI_ISL_475683	28/03/2020	North America / USA / California / Los Angeles	Human		Female	51	Outpatient	Original		B.1.43	GH	USA	Undertermined	No (couldn't determine the patient status as either pre-symptomatic / asymptomatic or symptomatic conclusively)
hCoV-19/USA/CA-CSMC7/2020	EPI_ISL_475684	27/03/2020	North America / USA / California / Los Angeles	Human		Male	66	Released	Original		B.1	GH	USA	Undertermined	No (couldn't determine the patient status as either pre-symptomatic / asymptomatic or symptomatic conclusively)
hCoV-19/USA/CA-CSMC70/2020	EPI_ISL_475685	26/03/2020	North America / USA / California / Los Angeles	Human		Male	40	Deceased	Original		B.1	GH	USA	Symptomatic	Yes
hCoV-19/USA/CA-CSMC71/2020	EPI_ISL_475686	03/04/2020	North America / USA / California / Los Angeles	Human		Male	64	Released	Original		A.3	S	USA	Undertermined	No (couldn't determine the patient status as either pre-symptomatic / asymptomatic or symptomatic conclusively)
hCoV-19/USA/CA-CSMC72/2020	EPI_ISL_475687	26/03/2020	North America / USA / California / Los Angeles	Human		Male	57	Released	Original		B.1	GH	USA	Undertermined	No (couldn't determine the patient status as either pre-symptomatic / asymptomatic or symptomatic conclusively)
hCoV-19/USA/CA-CSMC73/2020	EPI_ISL_475688	26/03/2020	North America / USA / California / Los Angeles	Human		Male	55	Released	Original		B.1	GH	USA	Undertermined	No (couldn't determine the patient status as either pre-symptomatic / asymptomatic or symptomatic conclusively)
hCoV-19/USA/CA-CSMC74/2020	EPI_ISL_475689	26/03/2020	North America / USA / California / Los Angeles	Human		Male	39	Released	Original		B.1	GH	USA	Undertermined	No (couldn't determine the patient status as either pre-symptomatic / asymptomatic or symptomatic conclusively)
hCoV-19/USA/CA-CSMC75/2020	EPI_ISL_475690	03/04/2020	North America / USA / California / Los Angeles	Human		Female	66	Deceased	Original		B.1	G	USA	Symptomatic	Yes
hCoV-19/USA/CA-CSMC76/2020	EPI_ISL_475691	03/04/2020	North America / USA / California / Los Angeles	Human		Female	84	Deceased	Original		B.1.1	GR	USA	Symptomatic	Yes
hCoV-19/USA/CA-CSMC77/2020	EPI_ISL_475692	26/03/2020	North America / USA / California / Los Angeles	Human		Female	27	Outpatient	Original		B.1	GH	USA	Undertermined	No (couldn't determine the patient status as either pre-symptomatic / asymptomatic or symptomatic conclusively)
hCoV-19/USA/CA-CSMC78/2020	EPI_ISL_475693	26/03/2020	North America / USA / California / Los Angeles	Human		Male	39	Released	Original		B.1	GH	USA	Undertermined	No (couldn't determine the patient status as either pre-symptomatic / asymptomatic or symptomatic conclusively)
hCoV-19/USA/CA-CSMC79/2020	EPI_ISL_475694	26/03/2020	North America / USA / California / Los Angeles	Human		Male	69	Released	Original		B.1.43	GH	USA	Undertermined	No (couldn't determine the patient status as either pre-symptomatic / asymptomatic or symptomatic conclusively)
hCoV-19/USA/CA-CSMC8/2020	EPI_ISL_475695	27/03/2020	North America / USA / California / Los Angeles	Human		Female	54	Released	Original		B.1.43	GH	USA	Undertermined	No (couldn't determine the patient status as either pre-symptomatic / asymptomatic or symptomatic conclusively)
hCoV-19/USA/CA-CSMC80/2020	EPI_ISL_475696	26/03/2020	North America / USA / California / Los Angeles	Human		Female	75	Released	Original		B.1	O	USA	Undertermined	No (couldn't determine the patient status as either pre-symptomatic / asymptomatic or symptomatic conclusively)
hCoV-19/USA/CA-CSMC81/2020	EPI_ISL_475697	04/04/2020	North America / USA / California / Los Angeles	Human		Female	83	Released	Original		B.1	GH	USA	Undertermined	No (couldn't determine the patient status as either pre-symptomatic / asymptomatic or symptomatic conclusively)
hCoV-19/USA/CA-CSMC82/2020	EPI_ISL_475698	07/04/2020	North America / USA / California / Los Angeles	Human		Male	43	Released	Original		B.1.43	GH	USA	Undertermined	No (couldn't determine the patient status as either pre-symptomatic / asymptomatic or symptomatic conclusively)
hCoV-19/USA/CA-CSMC83/2020	EPI_ISL_475699	26/03/2020	North America / USA / California / Los Angeles	Human		Female	27	Outpatient	Original		B.1.2	GH	USA	Undertermined	No (couldn't determine the patient status as either pre-symptomatic / asymptomatic or symptomatic conclusively)
hCoV-19/Singapore/526/2020	EPI_ISL_479482	26/04/2020	Asia / Singapore	Human		Male	35	Hospitalized	Original		B.6	O	Singapore	Undertermined	No (couldn't determine the patient status as either pre-symptomatic / asymptomatic or symptomatic conclusively)
hCoV-19/Singapore/527/2020	EPI_ISL_479483	26/04/2020	Asia / Singapore	Human		Male	34	Hospitalized	Original		B.6	O	Singapore	Undertermined	No (couldn't determine the patient status as either pre-symptomatic / asymptomatic or symptomatic conclusively)
hCoV-19/Singapore/528/2020	EPI_ISL_479484	27/04/2020	Asia / Singapore	Human		Male	49	Hospitalized	Original		B.6	O	Singapore	Undertermined	No (couldn't determine the patient status as either pre-symptomatic / asymptomatic or symptomatic conclusively)
hCoV-19/Singapore/529/2020	EPI_ISL_479485	26/04/2020	Asia / Singapore	Human		Male	37	Hospitalized	Original		B.6	O	Singapore	Undertermined	No (couldn't determine the patient status as either pre-symptomatic / asymptomatic or symptomatic conclusively)
hCoV-19/Singapore/530/2020	EPI_ISL_479486	26/04/2020	Asia / Singapore	Human		Male	56	Hospitalized	Original		B.6	O	Singapore	Undertermined	No (couldn't determine the patient status as either pre-symptomatic / asymptomatic or symptomatic conclusively)
hCoV-19/Singapore/531/2020	EPI_ISL_479487	26/04/2020	Asia / Singapore	Human		Male	35	Hospitalized	Original		B.6	O	Singapore	Undertermined	No (couldn't determine the patient status as either pre-symptomatic / asymptomatic or symptomatic conclusively)
hCoV-19/Singapore/532/2020	EPI_ISL_479488	26/04/2020	Asia / Singapore	Human		Male	33	Hospitalized	Original		B.6	O	Singapore	Undertermined	No (couldn't determine the patient status as either pre-symptomatic / asymptomatic or symptomatic conclusively)
hCoV-19/Singapore/533/2020	EPI_ISL_479489	26/04/2020	Asia / Singapore	Human		Male	27	Hospitalized	Original		B.6	O	Singapore	Undertermined	No (couldn't determine the patient status as either pre-symptomatic / asymptomatic or symptomatic conclusively)
hCoV-19/Singapore/534/2020	EPI_ISL_479490	27/04/2020	Asia / Singapore	Human		Male	25	Hospitalized	Original		B.6	O	Singapore	Undertermined	No (couldn't determine the patient status as either pre-symptomatic / asymptomatic or symptomatic conclusively)
hCoV-19/Singapore/535/2020	EPI_ISL_479491	26/04/2020	Asia / Singapore	Human		Male	28	Hospitalized	Original		B.6	O	Singapore	Undertermined	No (couldn't determine the patient status as either pre-symptomatic / asymptomatic or symptomatic conclusively)
hCoV-19/Singapore/536/2020	EPI_ISL_479492	26/04/2020	Asia / Singapore	Human		Male	34	Hospitalized	Original		B.6	O	Singapore	Undertermined	No (couldn't determine the patient status as either pre-symptomatic / asymptomatic or symptomatic conclusively)
hCoV-19/India/MH-NIV-10176/2020	EPI_ISL_479493	15/04/2020	Asia / India / Maharashtra	Human		Female	42	Asymptomatic	Original		B.1.1.32	GR	India	Pre-symptomatic / Asymptomatic	Yes
hCoV-19/India/MH-NIV-11857/2020	EPI_ISL_479495	19/04/2020	Asia / India / Maharashtra	Human		Female	58	Symptomatic	Original		B.1.80	G	India	Symptomatic	Yes
hCoV-19/India/MH-NIV-13307/2020	EPI_ISL_479498	23/04/2020	Asia / India / Maharashtra	Human		Female	50	Symptomatic	Original		B.1.1.32	GR	India	Symptomatic	Yes
hCoV-19/India/MH-NIV-14614/2020	EPI_ISL_479499	25/04/2020	Asia / India / Maharashtra	Human		Male	51	Symptomatic	Original		B.1.1.32	GR	India	Symptomatic	Yes
hCoV-19/India/MH-NIV-15351/2020	EPI_ISL_479501	26/04/2020	Asia / India / Maharashtra	Human		Male	48	Symptomatic	Original		B.1.1	GR	India	Symptomatic	Yes
hCoV-19/India/MH-NIV-15768/2020	EPI_ISL_479502	27/04/2020	Asia / India / Maharashtra	Human		Female	53	Symptomatic	Original		B.1.1	GR	India	Symptomatic	Yes
hCoV-19/India/MH-NIV-16503/2020	EPI_ISL_479505	28/04/2020	Asia / India / Maharashtra	Human		Female	61	Symptomatic	Original		B.1.1.32	GR	India	Symptomatic	Yes
hCoV-19/India/MH-NIV-17002/2020	EPI_ISL_479507	29/04/2020	Asia / India / Maharashtra	Human		Female	58	Symptomatic	Original		B.1.1.32	GR	India	Symptomatic	Yes
hCoV-19/India/MH-NIV-17570/2020	EPI_ISL_479509	30/04/2020	Asia / India / Maharashtra	Human		Male	65	Symptomatic	Original		B.1.1.32	GR	India	Symptomatic	Yes
hCoV-19/India/MH-NIV-18650/2020	EPI_ISL_479510	30/04/2020	Asia / India / Maharashtra	Human		Female	50	Symptomatic	Original		B.1.1.32	GR	India	Symptomatic	Yes
hCoV-19/India/MH-NIV-19746/2020	EPI_ISL_479512	03/05/2020	Asia / India / Maharashtra	Human		Female	58	Symptomatic	Original		B.1.1.32	GR	India	Symptomatic	Yes
hCoV-19/India/MH-NIV-20134/2020	EPI_ISL_479513	04/05/2020	Asia / India / Maharashtra	Human		Male	55	Symptomatic	Original		B.1.1.32	GR	India	Symptomatic	Yes
hCoV-19/India/MH-NIV-20608/2020	EPI_ISL_479514	04/05/2020	Asia / India / Maharashtra	Human		Male	67	Symptomatic	Original		B.1.1.32	GR	India	Symptomatic	Yes
hCoV-19/India/MH-NIV-21041/2020	EPI_ISL_479516	05/05/2020	Asia / India / Maharashtra	Human		Male	40	Symptomatic	Original		B.1	G	India	Symptomatic	Yes
hCoV-19/India/MH-NIV-21455/2020	EPI_ISL_479517	05/05/2020	Asia / India / Maharashtra	Human		Male	64	Symptomatic	Original		B.1.1.32	GR	India	Symptomatic	Yes
hCoV-19/India/MH-NIV-22552/2020	EPI_ISL_479519	07/05/2020	Asia / India / Maharashtra	Human		Male	67	Symptomatic	Original		B.1.1.32	GR	India	Symptomatic	Yes
hCoV-19/India/MH-NIV-22876/2020	EPI_ISL_479520	07/05/2020	Asia / India / Maharashtra	Human		Female	65	Asymptomatic	Original		B.6	O	India	Pre-symptomatic / Asymptomatic	Yes
hCoV-19/India/MH-NIV-23098/2020	EPI_ISL_479521	07/05/2020	Asia / India / Maharashtra	Human		Female	70	Symptomatic	Original		B.1.1.32	GR	India	Symptomatic	Yes
hCoV-19/India/MH-NIV-23721/2020	EPI_ISL_479522	08/05/2020	Asia / India / Maharashtra	Human		Male	75	Symptomatic	Original		B.1.1.32	GR	India	Symptomatic	Yes
hCoV-19/India/MH-NIV-24174/2020	EPI_ISL_479523	08/05/2020	Asia / India / Maharashtra	Human		Male	43	Symptomatic	Original		B.1.1.32	GR	India	Symptomatic	Yes
hCoV-19/India/MH-NIV-26115/2020	EPI_ISL_479525	12/05/2020	Asia / India / Maharashtra	Human		Female	68	Symptomatic	Original		B.1.1	GR	India	Symptomatic	Yes
hCoV-19/India/MH-NIV-27109/2020	EPI_ISL_479526	11/05/2020	Asia / India / Maharashtra	Human		Male	66	Symptomatic	Original		B.1.1.32	GR	India	Symptomatic	Yes
hCoV-19/India/MH-NIV-28147/2020	EPI_ISL_479527	12/05/2020	Asia / India / Maharashtra	Human		Male	58	Symptomatic	Original		B.1.80	G	India	Symptomatic	Yes
hCoV-19/India/MH-NIV-28416/2020	EPI_ISL_479528	12/05/2020	Asia / India / Maharashtra	Human		Male	38	Symptomatic	Original		B.1.1.32	GR	India	Symptomatic	Yes
hCoV-19/India/MH-NIV-28815/2020	EPI_ISL_479529	13/05/2020	Asia / India / Maharashtra	Human		Female	85	Symptomatic	Original		B.1.1.32	GR	India	Symptomatic	Yes
hCoV-19/India/MH-NIV-28900/2020	EPI_ISL_479530	13/05/2020	Asia / India / Maharashtra	Human		Male	80	Symptomatic	Original		B.1.1.32	GR	India	Symptomatic	Yes
hCoV-19/India/MH-NIV-29053/2020	EPI_ISL_479531	13/05/2020	Asia / India / Maharashtra	Human		Female	72	Symptomatic	Original		B.1.1.32	GR	India	Symptomatic	Yes
hCoV-19/India/MH-NIV-29909/2020	EPI_ISL_479532	14/05/2020	Asia / India / Maharashtra	Human		Male	65	Symptomatic	Original		B.1.1.32	GR	India	Symptomatic	Yes
hCoV-19/India/MH-NIV-31191/2020	EPI_ISL_479533	15/05/2020	Asia / India / Maharashtra	Human		Male	79	Symptomatic	Original		B.1.1.32	GR	India	Symptomatic	Yes
hCoV-19/India/MH-NIV-35868/2020	EPI_ISL_479534	19/05/2020	Asia / India / Maharashtra	Human		Female	61	Symptomatic	Original		B.1.1.32	GR	India	Symptomatic	Yes
hCoV-19/India/MH-NIV-36405/2020	EPI_ISL_479535	18/05/2020	Asia / India / Maharashtra	Human		Male	40	Symptomatic	Original		B.1.1.32	GR	India	Symptomatic	Yes
hCoV-19/India/MH-NIV-36444/2020	EPI_ISL_479536	19/05/2020	Asia / India / Maharashtra	Human		Female	60	Symptomatic	Original		B.1.1.32	GR	India	Symptomatic	Yes
hCoV-19/India/MH-NIV-36461/2020	EPI_ISL_479537	20/05/2020	Asia / India / Maharashtra	Human		Female	70	Symptomatic	Original		B.1.1	GR	India	Symptomatic	Yes
hCoV-19/India/MH-NIV-37486/2020	EPI_ISL_479538	20/05/2020	Asia / India / Maharashtra	Human		Male	61	Symptomatic	Original		B.1.1.32	GR	India	Symptomatic	Yes
hCoV-19/India/MH-NIV-37502/2020	EPI_ISL_479539	21/05/2020	Asia / India / Maharashtra	Human		Male	58	Symptomatic	Original		B.1.80	G	India	Symptomatic	Yes
hCoV-19/India/MH-NIV-37681/2020	EPI_ISL_479540	21/05/2020	Asia / India / Maharashtra	Human		Male	65	Asymptomatic	Original		B.1.1.32	GR	India	Pre-symptomatic / Asymptomatic	Yes
hCoV-19/India/MH-NIV-37985/2020	EPI_ISL_479541	21/05/2020	Asia / India / Maharashtra	Human		Male	27	Symptomatic	Original		B.1.1.32	GR	India	Symptomatic	Yes
hCoV-19/India/MH-NIV-40837/2020	EPI_ISL_479543	23/05/2020	Asia / India / Maharashtra	Human		Female	58	Symptomatic	Original		B.1.1.32	GR	India	Symptomatic	Yes
hCoV-19/India/MH-NIV-42694/2020	EPI_ISL_479544	24/05/2020	Asia / India / Maharashtra	Human		Female	38	Asymptomatic	Original		B.1.1	GR	India	Pre-symptomatic / Asymptomatic	Yes
hCoV-19/India/MH-NIV-44241/2020	EPI_ISL_479546	25/05/2020	Asia / India / Maharashtra	Human		Male	77	Symptomatic	Original		B.1.80	G	India	Symptomatic	Yes
hCoV-19/Bulgaria/41/2020	EPI_ISL_480310	10/03/2020	Europe / Bulgaria / Sofia	Human		Female	67	Deceased	Original	Nasopharyngeal swab	B.1	G	Bulgaria	Symptomatic	Yes
hCoV-19/Costa Rica/CRV-0011/2020	EPI_ISL_480315	10/03/2020	North America / Costa Rica / San Jose	Human		Male	51	Live	Original		B.1	G	Costa Rica	Undertermined	No (couldn't determine the patient status as either pre-symptomatic / asymptomatic or symptomatic conclusively)
hCoV-19/Costa Rica/CRV-0012/2020	EPI_ISL_480316	11/03/2020	North America / Costa Rica / Alajuela	Human	Travel to Florida	Male	87	Deceased	Original		B.1	G	Costa Rica	Symptomatic	Yes
hCoV-19/Costa Rica/CRV-0013/2020	EPI_ISL_480317	28/03/2020	North America / Costa Rica / San Jose	Human	Travel to Spain	Male	73	Released	Original		B.1	G	Costa Rica	Undertermined	No (couldn't determine the patient status as either pre-symptomatic / asymptomatic or symptomatic conclusively)
hCoV-19/Costa Rica/CRV-0014/2020	EPI_ISL_480318	29/03/2020	North America / Costa Rica / Alajuela	Human		Female	45	Live	Original		B.1	G	Costa Rica	Undertermined	No (couldn't determine the patient status as either pre-symptomatic / asymptomatic or symptomatic conclusively)
hCoV-19/Costa Rica/CRV-0015/2020	EPI_ISL_480319	30/03/2020	North America / Costa Rica / Heredia	Human	Contact with someone who travel to Mexico	Female	43	Live	Original		B.1	G	Costa Rica	Undertermined	No (couldn't determine the patient status as either pre-symptomatic / asymptomatic or symptomatic conclusively)
hCoV-19/Costa Rica/CRV-0016/2020	EPI_ISL_480320	31/03/2020	North America / Costa Rica / San Jose	Human		Male	33	Live	Original		B.1	G	Costa Rica	Undertermined	No (couldn't determine the patient status as either pre-symptomatic / asymptomatic or symptomatic conclusively)
hCoV-19/Costa Rica/CRV-0017/2020	EPI_ISL_480321	08/04/2020	North America / Costa Rica / San Jose	Human	Travel to New York	Female	27	Live	Original		B.1	G	Costa Rica	Undertermined	No (couldn't determine the patient status as either pre-symptomatic / asymptomatic or symptomatic conclusively)
hCoV-19/Costa Rica/CRV-0018/2020	EPI_ISL_480322	18/03/2020	North America / Costa Rica / San Jose	Human		Female	67	Live	Original		B.1	G	Costa Rica	Undertermined	No (couldn't determine the patient status as either pre-symptomatic / asymptomatic or symptomatic conclusively)
hCoV-19/Costa Rica/CRV-0019/2020	EPI_ISL_480323	18/03/2020	North America / Costa Rica / San Jose	Human		Male	27	Live	Original		B.1.5	G	Costa Rica	Undertermined	No (couldn't determine the patient status as either pre-symptomatic / asymptomatic or symptomatic conclusively)
hCoV-19/Costa Rica/CRV-0020/2020	EPI_ISL_480324	21/03/2020	North America / Costa Rica / San Jose	Human		Female	47	Live	Original		B.1	G	Costa Rica	Undertermined	No (couldn't determine the patient status as either pre-symptomatic / asymptomatic or symptomatic conclusively)
hCoV-19/Costa Rica/CRV-0021/2020	EPI_ISL_480325	26/03/2020	North America / Costa Rica / San Jose	Human		Male	23	Live	Original		B.1	G	Costa Rica	Undertermined	No (couldn't determine the patient status as either pre-symptomatic / asymptomatic or symptomatic conclusively)
hCoV-19/Costa Rica/CRV-0022/2020	EPI_ISL_480326	01/04/2020	North America / Costa Rica / Heredia	Human		Male	58	Live	Original		B.1	G	Costa Rica	Undertermined	No (couldn't determine the patient status as either pre-symptomatic / asymptomatic or symptomatic conclusively)
hCoV-19/Costa Rica/CRV-0023/2020	EPI_ISL_480327	04/04/2020	North America / Costa Rica / San Jose	Human		Male	27	Live	Original		B.1	G	Costa Rica	Undertermined	No (couldn't determine the patient status as either pre-symptomatic / asymptomatic or symptomatic conclusively)
hCoV-19/Costa Rica/CRV-0024/2020	EPI_ISL_480328	06/04/2020	North America / Costa Rica / San Jose	Human	Travel to USA	Male	26	Live	Original		B.1	G	Costa Rica	Undertermined	No (couldn't determine the patient status as either pre-symptomatic / asymptomatic or symptomatic conclusively)
hCoV-19/India/KA-nimh-0999/2020	EPI_ISL_486382	14/04/2020	Asia / India / Karnataka	Human		Male	43	Asymptomatic	Original		B.6	O	India	Pre-symptomatic / Asymptomatic	Yes
hCoV-19/India/KA-nimh-3959/2020	EPI_ISL_486383	23/04/2020	Asia / India / Karnataka	Human		Male	58	Asymptomatic	Original		B.1.80	G	India	Pre-symptomatic / Asymptomatic	Yes
hCoV-19/India/KA-nimh-8863/2020	EPI_ISL_486384	28/04/2020	Asia / India / Karnataka	Human		Male	82	Symptomatic	Original		B.1	O	India	Symptomatic	Yes
hCoV-19/India/KA-nimh-10102/2020	EPI_ISL_486385	01/05/2020	Asia / India / Karnataka	Human		Female	45	Asymptomatic	Original		B.6	O	India	Pre-symptomatic / Asymptomatic	Yes
hCoV-19/India/KA-nimh-10106/2020	EPI_ISL_486386	01/05/2020	Asia / India / Karnataka	Human		Female	24	Asymptomatic	Original		B.6	O	India	Pre-symptomatic / Asymptomatic	Yes

hCoV-19/India/KA-nimh-11122/2020	EPI_ISL_486387	04/05/2020	Asia / India / Karnataka	Human		Female	55	Asymptomatic	Original			B.6	O	India	Pre-symptomatic / Asymptomatic	Yes
hCoV-19/India/KA-nimh-14723/2020	EPI_ISL_486388	10/05/2020	Asia / India / Karnataka	Human		Male	22	Asymptomatic	Original			B.6	O	India	Pre-symptomatic / Asymptomatic	Yes
hCoV-19/India/KA-nimh-15835/2020	EPI_ISL_486389	10/05/2020	Asia / India / Karnataka	Human		Female	15	Asymptomatic	Original			B.6	O	India	Pre-symptomatic / Asymptomatic	Yes
hCoV-19/India/KA-nimh-1454/2020	EPI_ISL_486392	15/04/2020	Asia / India / Karnataka	Human		Male	28	Asymptomatic	Original			B.6	O	India	Pre-symptomatic / Asymptomatic	Yes
hCoV-19/India/KA-nimh-1553/2020	EPI_ISL_486393	16/04/2020	Asia / India / Karnataka	Human		Female	65	Symptomatic	Original			B.1	O	India	Symptomatic	Yes
hCoV-19/India/KA-nimh-3720/2020	EPI_ISL_486394	23/04/2020	Asia / India / Karnataka	Human		Male	60	Asymptomatic	Original			B.6	O	India	Pre-symptomatic / Asymptomatic	Yes
hCoV-19/India/KA-nimh-4263/2020	EPI_ISL_486395	23/04/2020	Asia / India / Karnataka	Human		Female	80	Asymptomatic	Original			B.6	O	India	Pre-symptomatic / Asymptomatic	Yes
hCoV-19/India/KA-nimh-4377/2020	EPI_ISL_486396	24/04/2020	Asia / India / Karnataka	Human		Male	90	Asymptomatic	Original			B.1.80	G	India	Pre-symptomatic / Asymptomatic	Yes
hCoV-19/India/KA-nimh-5102/2020	EPI_ISL_486397	25/04/2020	Asia / India / Karnataka	Human		Male	13	Symptomatic	Original			B.6	O	India	Symptomatic	Yes
hCoV-19/India/KA-nimh-5297/2020	EPI_ISL_486398	25/04/2020	Asia / India / Karnataka	Human		Female	22	Asymptomatic	Original			B.6	O	India	Pre-symptomatic / Asymptomatic	Yes
hCoV-19/India/KA-nimh-7817/2020	EPI_ISL_486399	29/04/2020	Asia / India / Karnataka	Human		Male	12	Asymptomatic	Original			B.6	O	India	Pre-symptomatic / Asymptomatic	Yes
hCoV-19/Czech Republic/NRL_3971/2020	EPI_ISL_491092	19/03/2020	Europe / Czech Republic / Litovel	Human		Male	19	Mild clinical signs without hospitalization	Original	Oro-pharyngeal swab	neogenetic single kindey	B.1.1	GR	Czech Republic	Symptomatic	Yes
hCoV-19/Czech Republic/NRL_4039/2020	EPI_ISL_491093	18/03/2020	Europe / Czech Republic / Zlin / FryÅ¡tÃ¡k	Human		Male	45	Mild clinical signs without hospitalization	Original	Oro-pharyngeal swab	Import Italy, Sondrio, Madesimo 29.2.-.6.3.	B.1.1	GR	Czech Republic	Symptomatic	Yes
hCoV-19/Czech Republic/NRL_4105/2020	EPI_ISL_491094	20/03/2020	Europe / Czech Republic / PlzeÅˆ / KlenÄÃ-	Human		Female	52	Mild clinical signs without hospitalization	Original	Oro-pharyngeal swab		B.1	GH	Czech Republic	Symptomatic	Yes
hCoV-19/Czech Republic/NRL_4253/2020	EPI_ISL_491095	20/03/2020	Europe / Czech Republic / ÃšstÃ- nad Labem / VÅ¡eboÅ™ice	Human		Male	32	Mild clinical signs without hospitalization	Original	Oro-pharyngeal swab		B.1.1	GR	Czech Republic	Symptomatic	Yes
hCoV-19/India/UN-11123/2020	EPI_ISL_491096	12/07/1905	Asia / India	Human		Male	26	Hospitalized	Original			B.6	L	India	Undertermined	No (couldn't determine the patient status as either pre-symptomatic / asymptomatic or symptomatic conclusively)
hCoV-19/India/UN-UN4/2020	EPI_ISL_491113	12/07/1905	Asia / India	Human		Male	38	Hospitalized	Original			B.6	O	India	Undertermined	No (couldn't determine the patient status as either pre-symptomatic / asymptomatic or symptomatic conclusively)
hCoV-19/India/UN-17074/2020	EPI_ISL_491114	12/07/1905	Asia / India	Human		Male	unknown	Alive	Original			B.1.1	GR	India	Undertermined	No (couldn't determine the patient status as either pre-symptomatic / asymptomatic or symptomatic conclusively)
hCoV-19/Czech Republic/NRL_5201/2020	EPI_ISL_491117	18/03/2020	Europe / Czech Republic / Prague	Human		Male	43	Mild clinical signs without hospitalization	Original	Oro-pharyngeal swab		B.1.1	GR	Czech Republic	Symptomatic	Yes
hCoV-19/Czech Republic/NRL_5752/2020	EPI_ISL_491118	01/04/2020	Europe / Czech Republic / Usti nad Labem	Human		Male	47	Mild clinical signs without hospitalization	Original	Oro-pharyngeal swab		B.1.1	GR	Czech Republic	Symptomatic	Yes
hCoV-19/Lebanon/LB-R8/2020	EPI_ISL_498551	19/03/2020	Asia / Lebanon / Beirut	Human		Male	36	Released	Original	Nasopharyngeal swab		B.1.5	G	Lebanon	Undertermined	No (couldn't determine the patient status as either pre-symptomatic / asymptomatic or symptomatic conclusively)
hCoV-19/Lebanon/LB-LR9/2020	EPI_ISL_498552	18/03/2020	Asia / Lebanon / Beirut	Human		Male	40	Released	Original	Nasopharyngeal swab		B.1.1	GR	Lebanon	Undertermined	No (couldn't determine the patient status as either pre-symptomatic / asymptomatic or symptomatic conclusively)
hCoV-19/Lebanon/LB-LR10/2020	EPI_ISL_498554	20/03/2020	Asia / Lebanon / Beirut	Human		Female	67	Released	Original	Nasopharyngeal swab		B.1	GH	Lebanon	Undertermined	No (couldn't determine the patient status as either pre-symptomatic / asymptomatic or symptomatic conclusively)
hCoV-19/Lebanon/LB-R12/2020	EPI_ISL_498556	20/03/2020	Asia / Lebanon / Beirut	Human		Male	54	Released	Original	Nasopharyngeal swab		B.1	GH	Lebanon	Undertermined	No (couldn't determine the patient status as either pre-symptomatic / asymptomatic or symptomatic conclusively)
hCoV-19/Italy/FVG-ICGEB-S208/2020	EPI_ISL_498558	2020-07	Europe / Italy / Friuli Venezia Giulia	Human		Male	67	Live	Original	Oropharyngeal swab		B.1	G	Italy	Undertermined	No (couldn't determine the patient status as either pre-symptomatic / asymptomatic or symptomatic conclusively)
hCoV-19/Italy/FVG-ICGEB-S211/2020	EPI_ISL_498559	01/07/2020	Europe / Italy / Friuli Venezia Giulia	Human		Female	50	Live	Original			B.1.1	GR	Italy	Undertermined	No (couldn't determine the patient status as either pre-symptomatic / asymptomatic or symptomatic conclusively)
hCoV-19/Italy/FVG-ICGEB-S212/2020	EPI_ISL_498560	02/07/2020	Europe / Italy / Friuli Venezia Giulia	Human		Male	17	Live	Original	Nasopharyngeal swab		B.1.1	GR	Italy	Undertermined	No (couldn't determine the patient status as either pre-symptomatic / asymptomatic or symptomatic conclusively)
hCoV-19/Italy/FVG-ICGEB-S214/2020	EPI_ISL_498561	01/07/2020	Europe / Italy / Friuli Venezia Giulia	Human		Male	54	Live	Original	Nasopharyngeal swab		B.1.1	GR	Italy	Undertermined	No (couldn't determine the patient status as either pre-symptomatic / asymptomatic or symptomatic conclusively)
hCoV-19/Italy/FVG-ICGEB-S217/2020	EPI_ISL_498562	07/07/2020	Europe / Italy / Friuli Venezia Giulia	Human	Returned from Kossovo 2020-07-02	Female	31	Live	Original			B.1.1	GR	Italy	Undertermined	No (couldn't determine the patient status as either pre-symptomatic / asymptomatic or symptomatic conclusively)
hCoV-19/Italy/FVG-ICGEB-S218/2020	EPI_ISL_498563	06/07/2020	Europe / Italy / Friuli Venezia Giulia	Human	Returned from Serbia 2020/06/21	Female	27	Live	Original	Nasopharyngeal swab		B.1.1.1	GR	Italy	Undertermined	No (couldn't determine the patient status as either pre-symptomatic / asymptomatic or symptomatic conclusively)
hCoV-19/Malaysia/7924/2020	EPI_ISL_507000	10/04/2020	Asia / Malaysia / Kuala Lumpur	Human		Male	56	Hospitalized	Original	Nasopharyngeal / Throat Swab		B.6	O	Malaysia	Undertermined	No (couldn't determine the patient status as either pre-symptomatic / asymptomatic or symptomatic conclusively)
hCoV-19/Singapore/31nan/2020	EPI_ISL_507002	14/03/2020	Asia / Singapore	Human		Female	27	Hospitalized	Original			B	L	Singapore	Undertermined	No (couldn't determine the patient status as either pre-symptomatic / asymptomatic or symptomatic conclusively)
hCoV-19/Singapore/758/2020	EPI_ISL_507003	14/03/2020	Asia / Singapore	Human		Male	35	Hospitalized	Original			B.6	O	Singapore	Undertermined	No (couldn't determine the patient status as either pre-symptomatic / asymptomatic or symptomatic conclusively)
hCoV-19/Singapore/759/2020	EPI_ISL_507004	14/03/2020	Asia / Singapore	Human		Male	26	Hospitalized	Original			B.1	G	Singapore	Undertermined	No (couldn't determine the patient status as either pre-symptomatic / asymptomatic or symptomatic conclusively)
hCoV-19/Singapore/760/2020	EPI_ISL_507005	26/04/2020	Asia / Singapore	Human		Male	51	Hospitalized	Original			B.6	O	Singapore	Undertermined	No (couldn't determine the patient status as either pre-symptomatic / asymptomatic or symptomatic conclusively)
hCoV-19/Singapore/761/2020	EPI_ISL_507006	27/04/2020	Asia / Singapore	Human		Male	41	Hospitalized	Original			B.6	O	Singapore	Undertermined	No (couldn't determine the patient status as either pre-symptomatic / asymptomatic or symptomatic conclusively)
hCoV-19/Sweden/SVA200327SZ-NSW/2020	EPI_ISL_507008	27/03/2020	Europe / Sweden / Uppsala	Human		Male	53	Symptoms indicative of upper respiratory infection	Original	Nasal mid-turbinate sample		B.1	GH	Sweden	Symptomatic	Yes
hCoV-19/Russia/Novosibirsk-RII27312S/2020	EPI_ISL_507206	29/04/2020	Europe / Russia / Novosibirsk	Human		Female	70	Hospitalized	Original	Nasopharyngeal swab		B.1	G	Russia	Undertermined	No (couldn't determine the patient status as either pre-symptomatic / asymptomatic or symptomatic conclusively)
hCoV-19/Russia/Novosibirsk-RII27313S/2020	EPI_ISL_507207	29/04/2020	Europe / Russia / Novosibirsk	Human		Female	68	Hospitalized	Original	Nasopharyngeal swab		B.1.1	GR	Russia	Undertermined	No (couldn't determine the patient status as either pre-symptomatic / asymptomatic or symptomatic conclusively)
hCoV-19/Russia/Novosibirsk-RII27314S/2020	EPI_ISL_507208	29/04/2020	Europe / Russia / Novosibirsk	Human		Female	47	Hospitalized	Original	Nasopharyngeal swab		B.1	G	Russia	Undertermined	No (couldn't determine the patient status as either pre-symptomatic / asymptomatic or symptomatic conclusively)
hCoV-19/Russia/Novosibirsk-RII27315S/2020	EPI_ISL_507209	29/04/2020	Europe / Russia / Novosibirsk	Human		Male	53	Hospitalized	Original	Nasopharyngeal swab		B.1	G	Russia	Undertermined	No (couldn't determine the patient status as either pre-symptomatic / asymptomatic or symptomatic conclusively)
hCoV-19/Russia/Novosibirsk-RII27316S/2020	EPI_ISL_507210	04/05/2020	Europe / Russia / Novosibirsk	Human		Male	47	Hospitalized	Original	Nasopharyngeal swab		B.1	GH	Russia	Undertermined	No (couldn't determine the patient status as either pre-symptomatic / asymptomatic or symptomatic conclusively)
hCoV-19/Russia/Novosibirsk-RII27317S/2020	EPI_ISL_507211	30/04/2020	Europe / Russia / Novosibirsk	Human		Female	66	Hospitalized	Original	Nasopharyngeal swab		B.1.1	GR	Russia	Undertermined	No (couldn't determine the patient status as either pre-symptomatic / asymptomatic or symptomatic conclusively)
hCoV-19/Russia/Novosibirsk-RII27318S/2020	EPI_ISL_507212	29/04/2020	Europe / Russia / Novosibirsk	Human		Female	59	Hospitalized	Original	Nasopharyngeal swab		B.1	G	Russia	Undertermined	No (couldn't determine the patient status as either pre-symptomatic / asymptomatic or symptomatic conclusively)
hCoV-19/Russia/Novosibirsk-RII27320S/2020	EPI_ISL_507213	07/05/2020	Europe / Russia / Novosibirsk	Human		Female	47	Hospitalized	Original	Nasopharyngeal swab		B.1	GH	Russia	Undertermined	No (couldn't determine the patient status as either pre-symptomatic / asymptomatic or symptomatic conclusively)
hCoV-19/Russia/Novosibirsk-RII27321S/2020	EPI_ISL_507214	07/05/2020	Europe / Russia / Novosibirsk	Human		Female	60	Hospitalized	Original	Nasopharyngeal swab		B.1.1	GR	Russia	Undertermined	No (couldn't determine the patient status as either pre-symptomatic / asymptomatic or symptomatic conclusively)
hCoV-19/Russia/Novosibirsk-RII27322S/2020	EPI_ISL_507215	07/05/2020	Europe / Russia / Novosibirsk	Human		Female	41	Hospitalized	Original	Nasopharyngeal swab		B.1.1	GR	Russia	Undertermined	No (couldn't determine the patient status as either pre-symptomatic / asymptomatic or symptomatic conclusively)
hCoV-19/Russia/StPetersburg-RII10128S/2020	EPI_ISL_507225	26/04/2020	Europe / Russia / Saint Petersburg	Human		Female	39	Hospitalized	Original	Nasopharyngeal swab		B.1.5	G	Russia	Undertermined	No (couldn't determine the patient status as either pre-symptomatic / asymptomatic or symptomatic conclusively)
hCoV-19/Russia/StPetersburg-RII10183S/2020	EPI_ISL_507226	26/04/2020	Europe / Russia / Saint Petersburg	Human		Female	58	Hospitalized	Original	Nasopharyngeal swab		B.1.5	G	Russia	Undertermined	No (couldn't determine the patient status as either pre-symptomatic / asymptomatic or symptomatic conclusively)
hCoV-19/Indonesia/JB-TFRIC19-R42550/2020	EPI_ISL_511878	11/07/2020	Asia / Indonesia / West Java / Bandung	Human		Male	58	Hospitalized	Original	Nasopharyngeal and Oropharyngeal swab		B.1.36	GH	Indonesia	Undertermined	No (couldn't determine the patient status as either pre-symptomatic / asymptomatic or symptomatic conclusively)
hCoV-19/Indonesia/JB-TFRIC19-L4173/2020	EPI_ISL_511879	08/07/2020	Asia / Indonesia / West Java / Bandung	Human		Female	34	Alive	Original	Nasopharyngeal and Oropharyngeal swab		B.1.36	GH	Indonesia	Undertermined	No (couldn't determine the patient status as either pre-symptomatic / asymptomatic or symptomatic conclusively)
hCoV-19/Vietnam/VNHN_0207/2020	EPI_ISL_511891	08/03/2020	Asia / Vietnam / Hanoi	Human		Male	61	Released	Original	Nasopharyngeal swab		B.1.1	GR	Vietnam	Undertermined	No (couldn't determine the patient status as either pre-symptomatic / asymptomatic or symptomatic conclusively)
hCoV-19/Vietnam/VNHN_0554/2020	EPI_ISL_511892	11/03/2020	Asia / Vietnam / Hanoi	Human		Female	43	Released	Original	Nasopharyngeal swab		B.1.1	GR	Vietnam	Undertermined	No (couldn't determine the patient status as either pre-symptomatic / asymptomatic or symptomatic conclusively)
hCoV-19/Vietnam/VNHN_0847/2020	EPI_ISL_511893	15/03/2020	Asia / Vietnam / Hanoi	Human		Male	36	Released	Original	Nasopharyngeal swab		B.3	L	Vietnam	Undertermined	No (couldn't determine the patient status as either pre-symptomatic / asymptomatic or symptomatic conclusively)
hCoV-19/Vietnam/VNHN_0897/2020	EPI_ISL_511894	16/03/2020	Asia / Vietnam / Hanoi	Human		Male	30	Released	Original	Nasopharyngeal swab		B.1	G	Vietnam	Undertermined	No (couldn't determine the patient status as either pre-symptomatic / asymptomatic or symptomatic conclusively)
hCoV-19/Vietnam/VNHN_0899/2020	EPI_ISL_511895	16/03/2020	Asia / Vietnam / Hanoi	Human		Female	26	Released	Original	Nasopharyngeal swab		B.1.1	GR	Vietnam	Undertermined	No (couldn't determine the patient status as either pre-symptomatic / asymptomatic or symptomatic conclusively)
hCoV-19/Vietnam/VNHN_1166/2020	EPI_ISL_511896	18/03/2020	Asia / Vietnam / Hanoi	Human		Male	52	Released	Original	Nasopharyngeal swab		B.1	GH	Vietnam	Undertermined	No (couldn't determine the patient status as either pre-symptomatic / asymptomatic or symptomatic conclusively)
hCoV-19/Vietnam/VNHN_1863/2020	EPI_ISL_511897	23/03/2020	Asia / Vietnam / Hanoi	Human		Female	24	Released	Original	Nasopharyngeal swab		B.2.1	V	Vietnam	Undertermined	No (couldn't determine the patient status as either pre-symptomatic / asymptomatic or symptomatic conclusively)
hCoV-19/Vietnam/VNHN_5152/2020	EPI_ISL_511898	14/04/2020	Asia / Vietnam / Hanoi	Human		Female	47	Released	Original	Nasopharyngeal swab		B.1.1	GR	Vietnam	Undertermined	No (couldn't determine the patient status as either pre-symptomatic / asymptomatic or symptomatic conclusively)
hCoV-19/SaudiArabia/KAUST-JEDDAH757/2020	EPI_ISL_513000	01/04/2020	Asia / Saudi Arabia / Jeddah	Human		Male	18	Live	Original			B.1.36	O	Saudi Arabia	Undertermined	No (couldn't determine the patient status as either pre-symptomatic / asymptomatic or symptomatic conclusively)
hCoV-19/SaudiArabia/KAUST-JEDDAH759/2020	EPI_ISL_513001	01/04/2020	Asia / Saudi Arabia / Jeddah	Human		Female	31	Live	Original			B.1.36	GH	Saudi Arabia	Undertermined	No (couldn't determine the patient status as either pre-symptomatic / asymptomatic or symptomatic conclusively)
hCoV-19/SaudiArabia/KAUST-JEDDAH826/2020	EPI_ISL_513002	24/05/2020	Asia / Saudi Arabia / Jeddah	Human		Male	76	Deceased	Original			B.1.36	O	Saudi Arabia	Symptomatic	Yes
hCoV-19/SaudiArabia/KAUST-JEDDAH828/2020	EPI_ISL_513003	24/05/2020	Asia / Saudi Arabia / Jeddah	Human		Male	61	Hospitalized	Original			B.1.1	O	Saudi Arabia	Undertermined	No (couldn't determine the patient status as either pre-symptomatic / asymptomatic or symptomatic conclusively)
hCoV-19/SaudiArabia/KAUST-JEDDAH830/2020	EPI_ISL_513004	24/05/2020	Asia / Saudi Arabia / Jeddah	Human		Female	45	Live	Original			B.1.1	O	Saudi Arabia	Undertermined	No (couldn't determine the patient status as either pre-symptomatic / asymptomatic or symptomatic conclusively)
hCoV-19/SaudiArabia/KAUST-JEDDAH831/2020	EPI_ISL_513005	24/05/2020	Asia / Saudi Arabia / Jeddah	Human		Male	65	Hospitalized	Original			B.1.36	GH	Saudi Arabia	Undertermined	No (couldn't determine the patient status as either pre-symptomatic / asymptomatic or symptomatic conclusively)
hCoV-19/SaudiArabia/KAUST-JEDDAH833/2020	EPI_ISL_513006	24/05/2020	Asia / Saudi Arabia / Jeddah	Human		Male	54	Deceased	Original			B.1.36	GH	Saudi Arabia	Symptomatic	Yes
hCoV-19/SaudiArabia/KAUST-JEDDAH834/2020	EPI_ISL_513007	24/05/2020	Asia / Saudi Arabia / Jeddah	Human		Female	71	Hospitalized	Original			B	O	Saudi Arabia	Undertermined	No (couldn't determine the patient status as either pre-symptomatic / asymptomatic or symptomatic conclusively)
hCoV-19/SaudiArabia/KAUST-JEDDAH835/2020	EPI_ISL_513008	24/05/2020	Asia / Saudi Arabia / Jeddah	Human		Male	60	Deceased	Original			B.1.1	O	Saudi Arabia	Symptomatic	Yes
hCoV-19/SaudiArabia/KAUST-JEDDAH836/2020	EPI_ISL_513009	24/05/2020	Asia / Saudi Arabia / Jeddah	Human		Male	59	Deceased	Original			B.1.36	GH	Saudi Arabia	Symptomatic	Yes
hCoV-19/SaudiArabia/KAUST-JEDDAH837/2020	EPI_ISL_513010	24/05/2020	Asia / Saudi Arabia / Jeddah	Human		Male	43	Deceased	Original			B.1.36	O	Saudi Arabia	Symptomatic	Yes
hCoV-19/SaudiArabia/KAUST-JEDDAH838/2020	EPI_ISL_513011	24/05/2020	Asia / Saudi Arabia / Jeddah	Human		Female	70	Deceased	Original			B.1.36	O	Saudi Arabia	Symptomatic	Yes
hCoV-19/SaudiArabia/KAUST-JEDDAH840/2020	EPI_ISL_513012	24/05/2020	Asia / Saudi Arabia / Jeddah	Human		Male	55	Hospitalized	Original			B.1.1	O	Saudi Arabia	Undertermined	No (couldn't determine the patient status as either pre-symptomatic / asymptomatic or symptomatic conclusively)
hCoV-19/SaudiArabia/KAUST-JEDDAH841/2020	EPI_ISL_513013	24/05/2020	Asia / Saudi Arabia / Jeddah	Human		Male	44	Deceased	Original			B.1.36	O	Saudi Arabia	Symptomatic	Yes
hCoV-19/SaudiArabia/KAUST-JEDDAH842/2020	EPI_ISL_513014	24/05/2020	Asia / Saudi Arabia / Jeddah	Human		Male	54	Deceased	Original			B.1.1	O	Saudi Arabia	Symptomatic	Yes
hCoV-19/SaudiArabia/KAUST-JEDDAH843/2020	EPI_ISL_513015	24/05/2020	Asia / Saudi Arabia / Jeddah	Human		Male	60	Hospitalized	Original			B.1.36	O	Saudi Arabia	Undertermined	No (couldn't determine the patient status as either pre-symptomatic / asymptomatic or symptomatic conclusively)
hCoV-19/SaudiArabia/KAUST-JEDDAH844/2020	EPI_ISL_513016	24/05/2020	Asia / Saudi Arabia / Jeddah	Human		Male	79	Deceased	Original			B.1.36	O	Saudi Arabia	Symptomatic	Yes
hCoV-19/SaudiArabia/KAUST-JEDDAH845/2020	EPI_ISL_513017	24/05/2020	Asia / Saudi Arabia / Jeddah	Human		Male	39	Deceased	Original			B.1.1	O	Saudi Arabia	Symptomatic	Yes
hCoV-19/SaudiArabia/KAUST-JEDDAH846/2020	EPI_ISL_513018	24/05/2020	Asia / Saudi Arabia / Jeddah	Human		Male	58	Deceased	Original			B.1.36	GH	Saudi Arabia	Symptomatic	Yes
hCoV-19/SaudiArabia/KAUST-JEDDAH847/2020	EPI_ISL_513019	24/05/2020	Asia / Saudi Arabia / Jeddah	Human		Female	83	Deceased	Original			B.1.1	O	Saudi Arabia	Symptomatic	Yes
hCoV-19/SaudiArabia/KAUST-JEDDAH866/2020	EPI_ISL_513020	24/05/2020	Asia / Saudi Arabia / Jeddah	Human		Male	60	Deceased	Original			B.1	O	Saudi Arabia	Symptomatic	Yes
hCoV-19/SaudiArabia/KAUST-JEDDAH867/2020	EPI_ISL_513021	24/05/2020	Asia / Saudi Arabia / Jeddah	Human		Female	47	Deceased	Original			B.1.1	O	Saudi Arabia	Symptomatic	Yes
hCoV-19/SaudiArabia/KAUST-JEDDAH868/2020	EPI_ISL_513022	24/05/2020	Asia / Saudi Arabia / Jeddah	Human		Male	48	Deceased	Original			B.1.1	GR	Saudi Arabia	Symptomatic	Yes
hCoV-19/SaudiArabia/KAUST-JEDDAH869/2020	EPI_ISL_513023	24/05/2020	Asia / Saudi Arabia / Jeddah	Human		Male	48	Deceased	Original			B.1.1	GR	Saudi Arabia	Symptomatic	Yes
hCoV-19/SaudiArabia/KAUST-JEDDAH870/2020	EPI_ISL_513024	24/05/2020	Asia / Saudi Arabia / Jeddah	Human		Male	73	Deceased	Original			B.1.36	GH	Saudi Arabia	Symptomatic	Yes
hCoV-19/SaudiArabia/KAUST-JEDDAH871/2020	EPI_ISL_513025	24/05/2020	Asia / Saudi Arabia / Jeddah	Human		Male	57	Deceased	Original			B.1.1	GR	Saudi Arabia	Symptomatic	Yes
hCoV-19/SaudiArabia/KAUST-JEDDAH872/2020	EPI_ISL_513026	24/05/2020	Asia / Saudi Arabia / Jeddah	Human		Male	74	Live	Original			B.1.1	O	Saudi Arabia	Undertermined	No (couldn't determine the patient status as either pre-symptomatic / asymptomatic or symptomatic conclusively)
hCoV-19/SaudiArabia/KAUST-JEDDAH873/2020	EPI_ISL_513027	24/05/2020	Asia / Saudi Arabia / Jeddah	Human		Male	51	Deceased	Original			B.1.1	O	Saudi Arabia	Symptomatic	Yes
hCoV-19/SaudiArabia/KAUST-JEDDAH875/2020	EPI_ISL_513028	24/05/2020	Asia / Saudi Arabia / Jeddah	Human		Male	55	Deceased	Original			B.1.113	O	Saudi Arabia	Symptomatic	Yes
hCoV-19/SaudiArabia/KAUST-JEDDAH877/2020	EPI_ISL_513029	24/05/2020	Asia / Saudi Arabia / Jeddah	Human		Male	30	Deceased	Original			B.1.1	GR	Saudi Arabia	Symptomatic	Yes
hCoV-19/SaudiArabia/KAUST-JEDDAH878/2020	EPI_ISL_513030	24/05/2020	Asia / Saudi Arabia / Jeddah	Human		Female	80	Deceased	Original			B.1.1	GR	Saudi Arabia	Symptomatic	Yes
hCoV-19/SaudiArabia/KAUST-JEDDAH879/2020	EPI_ISL_513031	24/05/2020	Asia / Saudi Arabia / Jeddah	Human		Male	54	Deceased	Original			B.1	GH	Saudi Arabia	Symptomatic	Yes
hCoV-19/SaudiArabia/KAUST-JEDDAH882/2020	EPI_ISL_513032	24/05/2020	Asia / Saudi Arabia / Jeddah	Human		Male	59	Deceased	Original			B.1.36	GH	Saudi Arabia	Symptomatic	Yes
hCoV-19/SaudiArabia/KAUST-JEDDAH883/2020	EPI_ISL_513033	24/05/2020	Asia / Saudi Arabia / Jeddah	Human		Male	77	Deceased	Original			B.1.113	O	Saudi Arabia	Symptomatic	Yes
hCoV-19/SaudiArabia/KAUST-JEDDAH884/2020	EPI_ISL_513034	24/05/2020	Asia / Saudi Arabia / Jeddah	Human		Male	61	Deceased	Original			B.1.113	O	Saudi Arabia	Symptomatic	Yes
hCoV-19/SaudiArabia/KAUST-JEDDAH886/2020	EPI_ISL_513035	24/05/2020	Asia / Saudi Arabia / Jeddah	Human		Male	68	Deceased	Original			B.1.1	GR	Saudi Arabia	Symptomatic	Yes
hCoV-19/SaudiArabia/KAUST-JEDDAH887/2020	EPI_ISL_513036	24/05/2020	Asia / Saudi Arabia / Jeddah	Human		Male	31	Deceased	Original			B.1.1	O	Saudi Arabia	Symptomatic	Yes
hCoV-19/SaudiArabia/KAUST-JEDDAH888/2020	EPI_ISL_513037	24/05/2020	Asia / Saudi Arabia / Jeddah	Human		Male	62	Deceased	Original			B.1.1	O	Saudi Arabia	Symptomatic	Yes
hCoV-19/SaudiArabia/KAUST-JEDDAH889/2020	EPI_ISL_513038	24/05/2020	Asia / Saudi Arabia / Jeddah	Human		Male	55	Deceased	Original			B.1.36	GH	Saudi Arabia	Symptomatic	Yes
hCoV-19/SaudiArabia/KAUST-JEDDAH890/2020	EPI_ISL_513039	24/05/2020	Asia / Saudi Arabia / Jeddah	Human		Male	53	Deceased	Original			B.1.1	O	Saudi Arabia	Symptomatic	Yes
hCoV-19/SaudiArabia/KAUST-JEDDAH891/2020	EPI_ISL_513040	24/05/2020	Asia / Saudi Arabia / Jeddah	Human		Male	52	Deceased	Original			B.1.36	O	Saudi Arabia	Symptomatic	Yes
hCoV-19/Saudi Arabia/KAUST-JEDDAH900/2020	EPI_ISL_513041	06/04/2020	Asia / Saudi Arabia / Jeddah	Human		Male	51	Live	Original			B.1.36	O	Saudi Arabia	Undertermined	No (couldn't determine the patient status as either pre-symptomatic / asymptomatic or symptomatic conclusively)
hCoV-19/SaudiArabia/KAUST-JEDDAH903/2020	EPI_ISL_513042	06/04/2020	Asia / Saudi Arabia / Jeddah	Human		Female	45	Live	Original			B.1.36	GH	Saudi Arabia	Undertermined	No (couldn't determine the patient status as either pre-symptomatic / asymptomatic or symptomatic conclusively)
hCoV-19/SaudiArabia/KAUST-JEDDAH905/2020	EPI_ISL_513043	06/04/2020	Asia / Saudi Arabia / Jeddah	Human		Male	61	Deceased	Original			B.1.1	GR	Saudi Arabia	Symptomatic	Yes
hCoV-19/SaudiArabia/KAUST-JEDDAH911/2020	EPI_ISL_513044	06/04/2020	Asia / Saudi Arabia / Jeddah	Human		Male	51	Live	Original			B.1.1	GR	Saudi Arabia	Undertermined	No (couldn't determine the patient status as either pre-symptomatic / asymptomatic or symptomatic conclusively)
hCoV-19/SaudiArabia/KAUST-JEDDAH912/2020	EPI_ISL_513045	06/04/2020	Asia / Saudi Arabia / Jeddah	Human		Male	55	Deceased	Original			B.1.1	O	Saudi Arabia	Symptomatic	Yes
hCoV-19/SaudiArabia/KAUST-JEDDAH913/2020	EPI_ISL_513046	06/04/2020	Asia / Saudi Arabia / Jeddah	Human		Male	73	Deceased	Original			B.1.1	O	Saudi Arabia	Symptomatic	Yes
hCoV-19/SaudiArabia/KAUST-JEDDAH916/2020	EPI_ISL_513047	06/04/2020	Asia / Saudi Arabia / Jeddah	Human		Male	28	Deceased	Original			B.1	G	Saudi Arabia	Symptomatic	Yes
hCoV-19/SaudiArabia/KAUST-JEDDAH917/2020	EPI_ISL_513048	06/04/2020	Asia / Saudi Arabia / Jeddah	Human		Male	54	Deceased	Original			B.1.1	GR	Saudi Arabia	Symptomatic	Yes
hCoV-19/SaudiArabia/KAUST-JEDDAH918/2020	EPI_ISL_513049	06/04/2020	Asia / Saudi Arabia / Jeddah	Human		Male	65	Deceased	Original			B.1.1	O	Saudi Arabia	Symptomatic	Yes
hCoV-19/SaudiArabia/KAUST-JEDDAH919/2020	EPI_ISL_513050	06/04/2020	Asia / Saudi Arabia / Jeddah	Human		Male	59	Deceased	Original			B.1.36	O	Saudi Arabia	Symptomatic	Yes
hCoV-19/SaudiArabia/KAUST-JEDDAH920/2020	EPI_ISL_513051	06/04/2020	Asia / Saudi Arabia / Jeddah	Human		Male	44	Deceased	Original			B.1.1	GR	Saudi Arabia	Symptomatic	Yes
hCoV-19/SaudiArabia/KAUST-JEDDAH921/2020	EPI_ISL_513052	06/04/2020	Asia / Saudi Arabia / Jeddah	Human		Male	48	Deceased	Original			B.1	O	Saudi Arabia	Symptomatic	Yes
hCoV-19/SaudiArabia/KAUST-JEDDAH922/2020	EPI_ISL_513053	06/04/2020	Asia / Saudi Arabia / Jeddah	Human		Male	69	Deceased	Original			B.1.1	GR	Saudi Arabia	Symptomatic	Yes
hCoV-19/SaudiArabia/KAUST-JEDDAH923/2020	EPI_ISL_513054	06/04/2020	Asia / Saudi Arabia / Jeddah	Human		Male	41	Deceased	Original			B.1.1	O	Saudi Arabia	Symptomatic	Yes
hCoV-19/SaudiArabia/KAUST-JEDDAH926/2020	EPI_ISL_513055	06/04/2020	Asia / Saudi Arabia / Jeddah	Human		Male	55	Deceased	Original			B.1.1	O	Saudi Arabia	Symptomatic	Yes
hCoV-19/SaudiArabia/KAUST-JEDDAH927/2020	EPI_ISL_513056	06/04/2020	Asia / Saudi Arabia / Jeddah	Human		Male	49	Deceased	Original			B.1.1	O	Saudi Arabia	Symptomatic	Yes
hCoV-19/SaudiArabia/KAUST-JEDDAH929/2020	EPI_ISL_513057	06/04/2020	Asia / Saudi Arabia / Jeddah	Human		Female	43	Deceased	Original			B.1.1	O	Saudi Arabia	Symptomatic	Yes
hCoV-19/SaudiArabia/KAUST-JEDDAH930/2020	EPI_ISL_513058	06/04/2020	Asia / Saudi Arabia / Jeddah	Human		Male	50	Deceased	Original			B.1.1	GR	Saudi Arabia	Symptomatic	Yes
hCoV-19/SaudiArabia/KAUST-JEDDAH931/2020	EPI_ISL_513059	06/04/2020	Asia / Saudi Arabia / Jeddah	Human		Male	52	Deceased	Original			B.1.1	O	Saudi Arabia	Symptomatic	Yes
hCoV-19/SaudiArabia/KAUST-JEDDAH932/2020	EPI_ISL_513060	06/04/2020	Asia / Saudi Arabia / Jeddah	Human		Male	51	Deceased	Original			B.1.1	O	Saudi Arabia	Symptomatic	Yes
hCoV-19/SaudiArabia/KAUST-JEDDAH933/2020	EPI_ISL_513061	06/04/2020	Asia / Saudi Arabia / Jeddah	Human		Male	57	Live	Original			B.1.1	GR	Saudi Arabia	Undertermined	No (couldn't determine the patient status as either pre-symptomatic / asymptomatic or symptomatic conclusively)
hCoV-19/SaudiArabia/KAUST-JEDDAH935/2020	EPI_ISL_513062	06/04/2020	Asia / Saudi Arabia / Jeddah	Human		Male	58	Deceased	Original			B.1.36	O	Saudi Arabia	Symptomatic	Yes
hCoV-19/SaudiArabia/KAUST-JEDDAH936/2020	EPI_ISL_513063	17/06/2020	Asia / Saudi Arabia / Jeddah	Human		Male	45	Deceased	Original			B.1.36	O	Saudi Arabia	Symptomatic	Yes
hCoV-19/SaudiArabia/KAUST-JEDDAH990/2020	EPI_ISL_513064	23/06/2020	Asia / Saudi Arabia / Jeddah	Human		Male	48	Live	Original			B.1.1	O	Saudi Arabia	Undertermined	No (couldn't determine the patient status as either pre-symptomatic / asymptomatic or symptomatic conclusively)
hCoV-19/SaudiArabia/KAUST-JEDDAH991/2020	EPI_ISL_513065	23/06/2020	Asia / Saudi Arabia / Jeddah	Human		Male	52	Live	Original			B.1.36	GH	Saudi Arabia	Undertermined	No (couldn't determine the patient status as either pre-symptomatic / asymptomatic or symptomatic conclusively)
hCoV-19/SaudiArabia/KAUST-JEDDAH992/2020	EPI_ISL_513066	17/06/2020	Asia / Saudi Arabia / Jeddah	Human		Male	61	Deceased	Original			B.1.1	O	Saudi Arabia	Symptomatic	Yes
hCoV-19/SaudiArabia/KAUST-JEDDAH993/2020	EPI_ISL_513067	17/06/2020	Asia / Saudi Arabia / Jeddah	Human		Female	53	Deceased	Original			B.1.1	GR	Saudi Arabia	Symptomatic	Yes
hCoV-19/SaudiArabia/KAUST-JEDDAH995/2020	EPI_ISL_513068	17/06/2020	Asia / Saudi Arabia / Jeddah	Human		Male	61	Deceased	Original			B.1.1	O	Saudi Arabia	Symptomatic	Yes
hCoV-19/SaudiArabia/KAUST-JEDDAH996/2020	EPI_ISL_513069	17/06/2020	Asia / Saudi Arabia / Jeddah	Human		Male	60	Deceased	Original			B.1.1	O	Saudi Arabia	Symptomatic	Yes
hCoV-19/SaudiArabia/KAUST-JEDDAH997/2020	EPI_ISL_513070	17/06/2020	Asia / Saudi Arabia / Jeddah	Human		Female	72	Hospitalized	Original			B.1.1	GR	Saudi Arabia	Undertermined	No (couldn't determine the patient status as either pre-symptomatic / asymptomatic or symptomatic conclusively)
hCoV-19/SaudiArabia/KAUST-JEDDAH998/2020	EPI_ISL_513071	23/06/2020	Asia / Saudi Arabia / Jeddah	Human		Male	28	Live	Original			B.1.1	O	Saudi Arabia	Undertermined	No (couldn't determine the patient status as either pre-symptomatic / asymptomatic or symptomatic conclusively)
hCoV-19/SaudiArabia/KAUST-JEDDAH999/2020	EPI_ISL_513072	23/06/2020	Asia / Saudi Arabia / Jeddah	Human		Male	40	Live	Original			B.1.1	O	Saudi Arabia	Undertermined	No (couldn't determine the patient status as either pre-symptomatic / asymptomatic or symptomatic conclusively)

hCoV-19/SaudiArabia/KAUST-MADINAH1036/2020	EPI_ISL_513073	11/06/2020	Asia / Saudi Arabia / Madinah	Human	Male	28	Live	Original		B.1.36	GH	Saudi Arabia	Undertermined	No (couldn't determine the patient status as either pre-symptomatic / asymptomatic or symptomatic conclusively)
hCoV-19/SaudiArabia/KAUST-MADINAH1038/2020	EPI_ISL_513074	13/06/2020	Asia / Saudi Arabia / Madinah	Human	Male	57	Live	Original		B.1	O	Saudi Arabia	Undertermined	No (couldn't determine the patient status as either pre-symptomatic / asymptomatic or symptomatic conclusively)
hCoV-19/SaudiArabia/KAUST-MADINAH1043/2020	EPI_ISL_513075	19/06/2020	Asia / Saudi Arabia / Madinah	Human	Female	36	Live	Original		B.1.113	O	Saudi Arabia	Undertermined	No (couldn't determine the patient status as either pre-symptomatic / asymptomatic or symptomatic conclusively)
hCoV-19/SaudiArabia/KAUST-MADINAH1044/2020	EPI_ISL_513076	13/06/2020	Asia / Saudi Arabia / Madinah	Human	Male	58	Live	Original		B.1.36	GH	Saudi Arabia	Undertermined	No (couldn't determine the patient status as either pre-symptomatic / asymptomatic or symptomatic conclusively)
hCoV-19/SaudiArabia/KAUST-MADINAH1047/2020	EPI_ISL_513078	13/06/2020	Asia / Saudi Arabia / Madinah	Human	Male	50	Live	Original		B.1.113	GH	Saudi Arabia	Undertermined	No (couldn't determine the patient status as either pre-symptomatic / asymptomatic or symptomatic conclusively)
hCoV-19/SaudiArabia/KAUST-MADINAH1048/2020	EPI_ISL_513079	13/06/2020	Asia / Saudi Arabia / Madinah	Human	Male	50	Live	Original		B.1.36	GH	Saudi Arabia	Undertermined	No (couldn't determine the patient status as either pre-symptomatic / asymptomatic or symptomatic conclusively)
hCoV-19/SaudiArabia/KAUST-MADINAH1051/2020	EPI_ISL_513081	13/06/2020	Asia / Saudi Arabia / Madinah	Human	Male	67	Deceased	Original		B.1.113	GH	Saudi Arabia	Symptomatic	Yes
hCoV-19/SaudiArabia/KAUST-MADINAH1052/2020	EPI_ISL_513082	13/06/2020	Asia / Saudi Arabia / Madinah	Human	Male	28	Live	Original		B.1.36	O	Saudi Arabia	Undertermined	No (couldn't determine the patient status as either pre-symptomatic / asymptomatic or symptomatic conclusively)
hCoV-19/SaudiArabia/KAUST-MADINAH1053/2020	EPI_ISL_513083	13/06/2020	Asia / Saudi Arabia / Madinah	Human	Female	65	Deceased	Original		B.1.113	O	Saudi Arabia	Symptomatic	Yes
hCoV-19/SaudiArabia/KAUST-MADINAH1059/2020	EPI_ISL_513084	13/06/2020	Asia / Saudi Arabia / Madinah	Human	Male	56	Live	Original		B.1.36	GH	Saudi Arabia	Undertermined	No (couldn't determine the patient status as either pre-symptomatic / asymptomatic or symptomatic conclusively)
hCoV-19/SaudiArabia/KAUST-MADINAH1061/2020	EPI_ISL_513086	13/06/2020	Asia / Saudi Arabia / Madinah	Human	Female	33	Live	Original		B.1.36	O	Saudi Arabia	Undertermined	No (couldn't determine the patient status as either pre-symptomatic / asymptomatic or symptomatic conclusively)
hCoV-19/SaudiArabia/KAUST-MADINAH1066/2020	EPI_ISL_513087	13/06/2020	Asia / Saudi Arabia / Madinah	Human	Male	33	Live	Original		B.1.36	GH	Saudi Arabia	Undertermined	No (couldn't determine the patient status as either pre-symptomatic / asymptomatic or symptomatic conclusively)
hCoV-19/SaudiArabia/KAUST-MADINAH1072/2020	EPI_ISL_513089	11/06/2020	Asia / Saudi Arabia / Madinah	Human	Male	67	Deceased	Original		B.1.36	O	Saudi Arabia	Symptomatic	Yes
hCoV-19/SaudiArabia/KAUST-MADINAH1073/2020	EPI_ISL_513090	11/06/2020	Asia / Saudi Arabia / Madinah	Human	Female	52	Live	Original		B.1.113	GH	Saudi Arabia	Undertermined	No (couldn't determine the patient status as either pre-symptomatic / asymptomatic or symptomatic conclusively)
hCoV-19/SaudiArabia/KAUST-MADINAH1075/2020	EPI_ISL_513092	11/06/2020	Asia / Saudi Arabia / Madinah	Human	Male	41	Live	Original		B.1.113	O	Saudi Arabia	Undertermined	No (couldn't determine the patient status as either pre-symptomatic / asymptomatic or symptomatic conclusively)
hCoV-19/SaudiArabia/KAUST-MADINAH1076/2020	EPI_ISL_513093	11/06/2020	Asia / Saudi Arabia / Madinah	Human	Male	34	Live	Original		B.1.113	O	Saudi Arabia	Undertermined	No (couldn't determine the patient status as either pre-symptomatic / asymptomatic or symptomatic conclusively)
hCoV-19/SaudiArabia/KAUST-MADINAH1077/2020	EPI_ISL_513094	11/06/2020	Asia / Saudi Arabia / Madinah	Human	Male	65	Deceased	Original		B.1.113	O	Saudi Arabia	Symptomatic	Yes
hCoV-19/SaudiArabia/KAUST-MADINAH1078/2020	EPI_ISL_513095	27/05/2020	Asia / Saudi Arabia / Madinah	Human	Male	52	Deceased	Original		B.1.113	GH	Saudi Arabia	Symptomatic	Yes
hCoV-19/SaudiArabia/KAUST-MADINAH1079/2020	EPI_ISL_513096	11/06/2020	Asia / Saudi Arabia / Madinah	Human	Male	39	Deceased	Original		B.1.36	GH	Saudi Arabia	Symptomatic	Yes
hCoV-19/SaudiArabia/KAUST-MADINAH1081/2020	EPI_ISL_513097	11/06/2020	Asia / Saudi Arabia / Madinah	Human	Female	59	Deceased	Original		B.1.36	GH	Saudi Arabia	Symptomatic	Yes
hCoV-19/SaudiArabia/KAUST-MADINAH1085/2020	EPI_ISL_513098	11/06/2020	Asia / Saudi Arabia / Madinah	Human	Male	66	Deceased	Original		B.1.113	O	Saudi Arabia	Symptomatic	Yes
hCoV-19/SaudiArabia/KAUST-MADINAH1086/2020	EPI_ISL_513099	13/06/2020	Asia / Saudi Arabia / Madinah	Human	Female	65	Deceased	Original		B.1.113	G	Saudi Arabia	Symptomatic	Yes
hCoV-19/Peru/LOR-INS-058/2020	EPI_ISL_514317	02/04/2020	South America / Peru / Loreto	Human	Male	66	unknown	Original	Oropharyngeal swab	B.1.1	GR	Peru	Undertermined	No (couldn't determine the patient status as either pre-symptomatic / asymptomatic or symptomatic conclusively)
hCoV-19/Brazil/SP-118/2020	EPI_ISL_515520	30/03/2020	South America / Brazil / Sao Paulo / Sao Paulo	Human	Male	74	Deceased	Original	Naso-pharyngeal swab	B.1.1.28	GR	Brazil	Symptomatic	Yes
hCoV-19/Brazil/SP-120/2020	EPI_ISL_515521	28/03/2020	South America / Brazil / Sao Paulo / Americana	Human	Male	52	Deceased	Original	Naso-pharyngeal swab	B.1.1.33	GR	Brazil	Symptomatic	Yes
hCoV-19/Brazil/SP-122/2020	EPI_ISL_515522	31/03/2020	South America / Brazil / Sao Paulo / Itatiba	Human	Male	84	Deceased	Original	Naso-pharyngeal swab	B.1.1.33	GR	Brazil	Symptomatic	Yes
hCoV-19/Brazil/SP-124/2020	EPI_ISL_515523	02/04/2020	South America / Brazil / Sao Paulo / Sao Paulo	Human	Male	55	Deceased	Original	Naso-pharyngeal swab	B.1.1.33	GR	Brazil	Symptomatic	Yes
hCoV-19/Brazil/SP-125/2020	EPI_ISL_515524	01/04/2020	South America / Brazil / Sao Paulo / Sao Paulo	Human	Male	62	Deceased	Original	Naso-pharyngeal swab	B.1.1.28	GR	Brazil	Symptomatic	Yes
hCoV-19/Brazil/SP-600/2020	EPI_ISL_515525	30/06/2020	South America / Brazil / Sao Paulo / Sao Paulo	Human	Male	60	Asymptomatic	Original	Naso-pharyngeal swab	B.1.1.33	GR	Brazil	Pre-symptomatic / Asymptomatic	Yes
hCoV-19/Brazil/SP-602/2020	EPI_ISL_515526	23/03/2020	South America / Brazil / Sao Paulo / Sao Paulo	Human	Female	72	Deceased	Original	Naso-pharyngeal swab	B.1.1.28	GR	Brazil	Symptomatic	Yes
hCoV-19/Brazil/SP-624/2020	EPI_ISL_515527	18/03/2020	South America / Brazil / Sao Paulo / Sao Paulo	Human	Female	35	Deceased	Original	Naso-pharyngeal swab	B.1.1.33	GR	Brazil	Symptomatic	Yes
hCoV-19/Brazil/SP-625/2020	EPI_ISL_515528	25/03/2020	South America / Brazil / Sao Paulo / Sao Paulo	Human	Female	53	Deceased	Original	Naso-pharyngeal swab	B.1.1.33	GR	Brazil	Symptomatic	Yes
hCoV-19/Brazil/SP-626/2020	EPI_ISL_515529	24/03/2020	South America / Brazil / Sao Paulo / Sao Paulo	Human	Male	52	Deceased	Original	Naso-pharyngeal swab	B.1.1.28	GR	Brazil	Symptomatic	Yes
hCoV-19/Brazil/SP-627/2020	EPI_ISL_515541	23/03/2020	South America / Brazil / Sao Paulo / Sao Paulo	Human	Male	46	Deceased	Original	Naso-pharyngeal swab	B.1.1.28	GR	Brazil	Symptomatic	Yes
hCoV-19/Brazil/SP-136/2020	EPI_ISL_515542	20/04/2020	South America / Brazil / Sao Paulo / Peruibe	Human	Male	77	Deceased	Original	Naso-pharyngeal swab	B.1.1.28	GR	Brazil	Symptomatic	Yes
hCoV-19/Brazil/SP-137/2020	EPI_ISL_515543	01/04/2020	South America / Brazil / Sao Paulo / GuarujÃ¡	Human	Female	70	Deceased	Original	Naso-pharyngeal swab	B.1.1.33	GR	Brazil	Symptomatic	Yes
hCoV-19/Brazil/SP-629/2020	EPI_ISL_515544	23/03/2020	South America / Brazil / Sao Paulo / Sao Paulo	Human	Male	67	Deceased	Original	Naso-pharyngeal swab	B.1.1.28	GR	Brazil	Symptomatic	Yes
hCoV-19/Brazil/SP-533/2020	EPI_ISL_515545	22/03/2020	South America / Brazil / Sao Paulo / Sao Paulo	Human	Male	69	Deceased	Original	Naso-pharyngeal swab	B.1.1.28	GR	Brazil	Symptomatic	Yes
hCoV-19/Brazil/SP-632/2020	EPI_ISL_515546	19/03/2020	South America / Brazil / Sao Paulo / Sao Paulo	Human	Female	50	Deceased	Original	Naso-pharyngeal swab	B.1.1.28	GR	Brazil	Symptomatic	Yes
hCoV-19/Brazil/SP-633/2020	EPI_ISL_515547	20/03/2020	South America / Brazil / Sao Paulo / Sao Paulo	Human	Male	65	Deceased	Original	Nasopharyngeal swab	B.1.1.28	GR	Brazil	Symptomatic	Yes
hCoV-19/Brazil/SP-634/2020	EPI_ISL_515548	20/03/2020	South America / Brazil / Sao Paulo / Sao Paulo	Human	Male	79	Deceased	Original	Naso-pharyngeal swab	B.1.1.28	GR	Brazil	Symptomatic	Yes
hCoV-19/Brazil/SP-636/2020	EPI_ISL_515550	25/03/2020	South America / Brazil / Sao Paulo / Sao Paulo	Human	Female	52	Deceased	Original	Naso-pharyngeal swab	B.1.1.33	GR	Brazil	Symptomatic	Yes
hCoV-19/Brazil/SP-544/2020	EPI_ISL_515551	22/03/2020	South America / Brazil / Sao Paulo / Sao Paulo	Human	Male	45	Deceased	Original	Naso-pharyngeal swab	B.1.1.28	GR	Brazil	Symptomatic	Yes
hCoV-19/Brazil/SP-611/2020	EPI_ISL_515552	16/04/2020	South America / Brazil / Sao Paulo / Sao Paulo	Human	Female	72	Deceased	Original	Naso-pharyngeal swab	B.1.1.28	GR	Brazil	Symptomatic	Yes
hCoV-19/Brazil/SP-612/2020	EPI_ISL_515553	16/04/2020	South America / Brazil / Sao Paulo / Sao Paulo	Human	Male	71	Deceased	Original	Naso-pharyngeal swab	B.1.1.28	GR	Brazil	Symptomatic	Yes
hCoV-19/Brazil/SP-639/2020	EPI_ISL_515554	19/03/2020	South America / Brazil / Sao Paulo / Sao Paulo	Human	Female	83	Deceased	Original	Naso-pharyngeal swab	B.1.1.28	GR	Brazil	Symptomatic	Yes
hCoV-19/Brazil/SP-154/2020	EPI_ISL_515555	24/03/2020	South America / Brazil / Sao Paulo / Sao Paulo	Human	Female	37	Deceased	Original	Naso-pharyngeal swab	B.1.1.28	GR	Brazil	Symptomatic	Yes
hCoV-19/Brazil/SP-615/2020	EPI_ISL_515557	15/04/2020	South America / Brazil / Sao Paulo / Sao Paulo	Human	Male	70	Deceased	Original	Naso-pharyngeal swab	B.1.1.33	GR	Brazil	Symptomatic	Yes
hCoV-19/Brazil/SP-155/2020	EPI_ISL_515559	18/04/2020	South America / Brazil / Sao Paulo / Sao Paulo	Human	Female	97	Deceased	Original	Naso-pharyngeal swab	B.1.1.28	GR	Brazil	Symptomatic	Yes
hCoV-19/Brazil/SP-156/2020	EPI_ISL_515560	19/04/2020	South America / Brazil / Sao Paulo / Sao Paulo	Human	Male	70	Deceased	Original	Naso-pharyngeal swab	B.1.1.28	GR	Brazil	Symptomatic	Yes
hCoV-19/Brazil/SP-617/2020	EPI_ISL_515561	16/04/2020	South America / Brazil / Sao Paulo / Sao Paulo	Human	Male	70	Deceased	Original	Naso-pharyngeal swab	B.1.1.28	GR	Brazil	Symptomatic	Yes
hCoV-19/Brazil/SP-619/2020	EPI_ISL_515562	13/04/2020	South America / Brazil / Sao Paulo / Sao Vicente	Human	Female	59	Deceased	Original	Naso-pharyngeal swab	B.1.1.28	GR	Brazil	Symptomatic	Yes
hCoV-19/Brazil/SP-159/2020	EPI_ISL_515563	16/04/2020	South America / Brazil / Sao Paulo / Sao Paulo	Human	Male	77	Deceased	Original	Nasopharyngeal swab	B.1.1.28	GR	Brazil	Symptomatic	Yes
hCoV-19/Brazil/SP-187/2020	EPI_ISL_515564	24/03/2020	South America / Brazil / Sao Paulo / Sao Paulo	Human	Female	43	Deceased	Original	Naso-pharyngeal swab	B.1.1.28	GR	Brazil	Symptomatic	Yes
hCoV-19/Brazil/SP-188/2020	EPI_ISL_515565	24/03/2020	South America / Brazil / Sao Paulo / Sao Paulo	Human	Male	71	Deceased	Original	Naso-pharyngeal swab	B.1.1.28	GR	Brazil	Symptomatic	Yes
hCoV-19/Brazil/SP-623/2020	EPI_ISL_515566	23/04/2020	South America / Brazil / Sao Paulo / Sao Paulo	Human	Male	83	Deceased	Original	Naso-pharyngeal swab	B.1.1.28	GR	Brazil	Symptomatic	Yes
hCoV-19/South Africa/KRISP-K002162/2020	EPI_ISL_515568	08/07/2020	Africa / South Africa / KZN / eThekwini	Human	Female	28	Live	Original	Oro-pharyngeal swab	B.1.1	GR	South Africa	Undertermined	No (couldn't determine the patient status as either pre-symptomatic / asymptomatic or symptomatic conclusively)
hCoV-19/South Africa/KRISP-K002163/2020	EPI_ISL_515569	22/07/2020	Africa / South Africa / KZN / eThekwini	Human	Female	24	Live	Original	Oro-pharyngeal swab	B.1.1	GR	South Africa	Undertermined	No (couldn't determine the patient status as either pre-symptomatic / asymptomatic or symptomatic conclusively)
hCoV-19/South Africa/KRISP-K002164/2020	EPI_ISL_515570	22/07/2020	Africa / South Africa / KZN / eThekwini	Human	Female	30	Live	Original	Oro-pharyngeal swab	B.1.1	GR	South Africa	Undertermined	No (couldn't determine the patient status as either pre-symptomatic / asymptomatic or symptomatic conclusively)
hCoV-19/South Africa/KRISP-K002165/2020	EPI_ISL_515571	22/07/2020	Africa / South Africa / KZN / eThekwini	Human	Female	57	Live	Original	Oro-pharyngeal swab	B.1.1	GR	South Africa	Undertermined	No (couldn't determine the patient status as either pre-symptomatic / asymptomatic or symptomatic conclusively)
hCoV-19/South Africa/KRISP-K002168/2020	EPI_ISL_515572	01/07/2020	Africa / South Africa / KZN / eThekwini	Human	Female	21	Live	Original	Oro-pharyngeal swab	B.1.1	GR	South Africa	Undertermined	No (couldn't determine the patient status as either pre-symptomatic / asymptomatic or symptomatic conclusively)
hCoV-19/South Africa/KRISP-K002171/2020	EPI_ISL_515573	08/07/2020	Africa / South Africa / KZN / eThekwini	Human	Female	45	Live	Original	Oro-pharyngeal swab	B.1.1	GR	South Africa	Undertermined	No (couldn't determine the patient status as either pre-symptomatic / asymptomatic or symptomatic conclusively)
hCoV-19/South Africa/KRISP-K002172/2020	EPI_ISL_515574	08/07/2020	Africa / South Africa / KZN / eThekwini	Human	Female	30	Live	Original	Oro-pharyngeal swab	B.1	G	South Africa	Undertermined	No (couldn't determine the patient status as either pre-symptomatic / asymptomatic or symptomatic conclusively)
hCoV-19/South Africa/KRISP-K002173/2020	EPI_ISL_515575	08/07/2020	Africa / South Africa / KZN / eThekwini	Human	Female	51	Live	Original	Oro-pharyngeal swab	B.1.1	GR	South Africa	Undertermined	No (couldn't determine the patient status as either pre-symptomatic / asymptomatic or symptomatic conclusively)
hCoV-19/South Africa/KRISP-K002174/2020	EPI_ISL_515576	09/07/2020	Africa / South Africa / KZN / eThekwini	Human	Female	34	Live	Original	Oro-pharyngeal swab	B.1.1	GR	South Africa	Undertermined	No (couldn't determine the patient status as either pre-symptomatic / asymptomatic or symptomatic conclusively)
hCoV-19/South Africa/KRISP-K002175/2020	EPI_ISL_515577	09/07/2020	Africa / South Africa / KZN / eThekwini	Human	Female	44	Live	Original	Oro-pharyngeal swab	B.1.1	GR	South Africa	Undertermined	No (couldn't determine the patient status as either pre-symptomatic / asymptomatic or symptomatic conclusively)
hCoV-19/South Africa/KRISP-K002176/2020	EPI_ISL_515578	29/06/2020	Africa / South Africa / KZN / eThekwini	Human	Female	35	Live	Original	Oro-pharyngeal swab	B.1.1	GR	South Africa	Undertermined	No (couldn't determine the patient status as either pre-symptomatic / asymptomatic or symptomatic conclusively)
hCoV-19/South Africa/KRISP-K002177/2020	EPI_ISL_515579	09/07/2020	Africa / South Africa / KZN / eThekwini	Human	Female	40	Live	Original	Oro-pharyngeal swab	B.1.1	GR	South Africa	Undertermined	No (couldn't determine the patient status as either pre-symptomatic / asymptomatic or symptomatic conclusively)
hCoV-19/South Africa/KRISP-K002179/2020	EPI_ISL_515580	08/07/2020	Africa / South Africa / KZN / eThekwini	Human	Female	40	Live	Original	Oro-pharyngeal swab	B.1.1	GR	South Africa	Undertermined	No (couldn't determine the patient status as either pre-symptomatic / asymptomatic or symptomatic conclusively)
hCoV-19/South Africa/KRISP-K002182/2020	EPI_ISL_515581	08/07/2020	Africa / South Africa / KZN / eThekwini	Human	Female	29	Live	Original	Oro-pharyngeal swab	B.1.1	GR	South Africa	Undertermined	No (couldn't determine the patient status as either pre-symptomatic / asymptomatic or symptomatic conclusively)
hCoV-19/South Africa/KRISP-K002183/2020	EPI_ISL_515582	09/07/2020	Africa / South Africa / KZN / eThekwini	Human	Female	42	Live	Original	Oro-pharyngeal swab	B.1.1	GR	South Africa	Undertermined	No (couldn't determine the patient status as either pre-symptomatic / asymptomatic or symptomatic conclusively)
hCoV-19/South Africa/KRISP-K002184/2020	EPI_ISL_515583	30/06/2020	Africa / South Africa / KZN / eThekwini	Human	Female	40	Live	Original	Oro-pharyngeal swab	B.1	G	South Africa	Undertermined	No (couldn't determine the patient status as either pre-symptomatic / asymptomatic or symptomatic conclusively)
hCoV-19/South Africa/KRISP-K002185/2020	EPI_ISL_515584	30/06/2020	Africa / South Africa / KZN / eThekwini	Human	Male	38	Live	Original	Oro-pharyngeal swab	B.1.1	GR	South Africa	Undertermined	No (couldn't determine the patient status as either pre-symptomatic / asymptomatic or symptomatic conclusively)
hCoV-19/South Africa/KRISP-K002186/2020	EPI_ISL_515585	30/06/2020	Africa / South Africa / KZN / eThekwini	Human	Female	40	Live	Original	Oro-pharyngeal swab	B.1.1	GR	South Africa	Undertermined	No (couldn't determine the patient status as either pre-symptomatic / asymptomatic or symptomatic conclusively)
hCoV-19/South Africa/KRISP-K002187/2020	EPI_ISL_515586	08/07/2020	Africa / South Africa / KZN / eThekwini	Human	Female	57	Live	Original	Oro-pharyngeal swab	B.1.1	GR	South Africa	Undertermined	No (couldn't determine the patient status as either pre-symptomatic / asymptomatic or symptomatic conclusively)
hCoV-19/South Africa/KRISP-K002188/2020	EPI_ISL_515587	08/07/2020	Africa / South Africa / KZN / eThekwini	Human	Female	34	Live	Original	Oro-pharyngeal swab	B.1	G	South Africa	Undertermined	No (couldn't determine the patient status as either pre-symptomatic / asymptomatic or symptomatic conclusively)
hCoV-19/South Africa/KRISP-K002189/2020	EPI_ISL_515588	08/07/2020	Africa / South Africa / KZN / eThekwini	Human	Female	60	Live	Original	Oro-pharyngeal swab	B.1	G	South Africa	Undertermined	No (couldn't determine the patient status as either pre-symptomatic / asymptomatic or symptomatic conclusively)
hCoV-19/South Africa/KRISP-K002190/2020	EPI_ISL_515589	14/07/2020	Africa / South Africa / KZN / eThekwini	Human	Female	37	Live	Original	Oro-pharyngeal swab	B.1.1	GR	South Africa	Undertermined	No (couldn't determine the patient status as either pre-symptomatic / asymptomatic or symptomatic conclusively)
hCoV-19/South Africa/KRISP-K002191/2020	EPI_ISL_515590	06/07/2020	Africa / South Africa / KZN / eThekwini	Human	Male	52	Live	Original	Oro-pharyngeal swab	B.1	G	South Africa	Undertermined	No (couldn't determine the patient status as either pre-symptomatic / asymptomatic or symptomatic conclusively)
hCoV-19/South Africa/KRISP-K002192/2020	EPI_ISL_515591	06/07/2020	Africa / South Africa / KZN / eThekwini	Human	Male	47	Live	Original	Oro-pharyngeal swab	B.1	G	South Africa	Undertermined	No (couldn't determine the patient status as either pre-symptomatic / asymptomatic or symptomatic conclusively)
hCoV-19/South Africa/KRISP-K002194/2020	EPI_ISL_515592	16/07/2020	Africa / South Africa / KZN / eThekwini	Human	Male	33	Live	Original	Oro-pharyngeal swab	B.1.1	GR	South Africa	Undertermined	No (couldn't determine the patient status as either pre-symptomatic / asymptomatic or symptomatic conclusively)
hCoV-19/South Africa/KRISP-K002195/2020	EPI_ISL_515593	10/07/2020	Africa / South Africa / KZN / eThekwini	Human	Female	38	Live	Original	Oro-pharyngeal swab	B.1.1.1	GR	South Africa	Undertermined	No (couldn't determine the patient status as either pre-symptomatic / asymptomatic or symptomatic conclusively)
hCoV-19/South Africa/KRISP-K002196/2020	EPI_ISL_515594	05/07/2020	Africa / South Africa / KZN / eThekwini	Human	Male	29	Live	Original	Oro-pharyngeal swab	B.1.1	GR	South Africa	Undertermined	No (couldn't determine the patient status as either pre-symptomatic / asymptomatic or symptomatic conclusively)
hCoV-19/South Africa/KRISP-K002197/2020	EPI_ISL_515595	13/07/2020	Africa / South Africa / KZN / eThekwini	Human	Female	42	Live	Original	Oro-pharyngeal swab	B.1.1	GR	South Africa	Undertermined	No (couldn't determine the patient status as either pre-symptomatic / asymptomatic or symptomatic conclusively)
hCoV-19/South Africa/KRISP-K002198/2020	EPI_ISL_515596	17/07/2020	Africa / South Africa / KZN / eThekwini	Human	Female	50	Live	Original	Oro-pharyngeal swab	B.1	G	South Africa	Undertermined	No (couldn't determine the patient status as either pre-symptomatic / asymptomatic or symptomatic conclusively)
hCoV-19/South Africa/KRISP-K002199/2020	EPI_ISL_515597	14/07/2020	Africa / South Africa / KZN / eThekwini	Human	Female	35	Live	Original	Oro-pharyngeal swab	B.1	G	South Africa	Undertermined	No (couldn't determine the patient status as either pre-symptomatic / asymptomatic or symptomatic conclusively)
hCoV-19/South Africa/KRISP-K002200/2020	EPI_ISL_515598	01/07/2020	Africa / South Africa / KZN / eThekwini	Human	Female	45	Live	Original	Oro-pharyngeal swab	B.1	G	South Africa	Undertermined	No (couldn't determine the patient status as either pre-symptomatic / asymptomatic or symptomatic conclusively)
hCoV-19/South Africa/KRISP-K002201/2020	EPI_ISL_515599	01/07/2020	Africa / South Africa / KZN / eThekwini	Human	unknown	unknown	Live	Original	Oro-pharyngeal swab	B.1	G	South Africa	Undertermined	No (couldn't determine the patient status as either pre-symptomatic / asymptomatic or symptomatic conclusively)
hCoV-19/South Africa/KRISP-K002202/2020	EPI_ISL_515600	16/07/2020	Africa / South Africa / KZN / eThekwini	Human	Female	35	Live	Original	Oro-pharyngeal swab	B.1	G	South Africa	Undertermined	No (couldn't determine the patient status as either pre-symptomatic / asymptomatic or symptomatic conclusively)
hCoV-19/South Africa/KRISP-K002203/2020	EPI_ISL_515601	09/07/2020	Africa / South Africa / KZN / eThekwini	Human	Female	40	Live	Original	Oro-pharyngeal swab	B.1	G	South Africa	Undertermined	No (couldn't determine the patient status as either pre-symptomatic / asymptomatic or symptomatic conclusively)
hCoV-19/South Africa/KRISP-K002204/2020	EPI_ISL_515602	18/07/2020	Africa / South Africa / KZN / eThekwini	Human	Female	32	Live	Original	Oro-pharyngeal swab	B.1.1	GR	South Africa	Undertermined	No (couldn't determine the patient status as either pre-symptomatic / asymptomatic or symptomatic conclusively)
hCoV-19/South Africa/KRISP-K002205/2020	EPI_ISL_515603	16/07/2020	Africa / South Africa / KZN / eThekwini	Human	Female	44	Live	Original	Oro-pharyngeal swab	B.1.106	G	South Africa	Undertermined	No (couldn't determine the patient status as either pre-symptomatic / asymptomatic or symptomatic conclusively)
hCoV-19/South Africa/KRISP-K002206/2020	EPI_ISL_515604	17/07/2020	Africa / South Africa / KZN / eThekwini	Human	Female	47	Live	Original	Oro-pharyngeal swab	B.1.1	GR	South Africa	Undertermined	No (couldn't determine the patient status as either pre-symptomatic / asymptomatic or symptomatic conclusively)
hCoV-19/South Africa/KRISP-K002208/2020	EPI_ISL_515605	24/07/2020	Africa / South Africa / KZN / eThekwini	Human	Female	60	Live	Original	Oro-pharyngeal swab	B.1.5	G	South Africa	Undertermined	No (couldn't determine the patient status as either pre-symptomatic / asymptomatic or symptomatic conclusively)
hCoV-19/South Africa/KRISP-K002209/2020	EPI_ISL_515606	24/07/2020	Africa / South Africa / KZN / eThekwini	Human	Male	60	Live	Original	Oro-pharyngeal swab	B.1	G	South Africa	Undertermined	No (couldn't determine the patient status as either pre-symptomatic / asymptomatic or symptomatic conclusively)
hCoV-19/South Africa/KRISP-K002210/2020	EPI_ISL_515607	24/07/2020	Africa / South Africa / KZN / eThekwini	Human	Male	36	Live	Original	Oro-pharyngeal swab	B.1.1	GR	South Africa	Undertermined	No (couldn't determine the patient status as either pre-symptomatic / asymptomatic or symptomatic conclusively)
hCoV-19/South Africa/KRISP-K002211/2020	EPI_ISL_515608	08/07/2020	Africa / South Africa / KZN / eThekwini	Human	Female	34	Live	Original	Oro-pharyngeal swab	B.1	G	South Africa	Undertermined	No (couldn't determine the patient status as either pre-symptomatic / asymptomatic or symptomatic conclusively)
hCoV-19/South Africa/KRISP-K002212/2020	EPI_ISL_515609	30/07/2020	Africa / South Africa / KZN / Umgungundlovu	Human	Male	13	Live	Original	Oro-pharyngeal swab	B.1.1	GR	South Africa	Undertermined	No (couldn't determine the patient status as either pre-symptomatic / asymptomatic or symptomatic conclusively)
hCoV-19/South Africa/KRISP-K002213/2020	EPI_ISL_515610	30/07/2020	Africa / South Africa / KZN / Umgungundlovu	Human	Female	49	Live	Original	Oro-pharyngeal swab	B.1.1.1	GR	South Africa	Undertermined	No (couldn't determine the patient status as either pre-symptomatic / asymptomatic or symptomatic conclusively)
hCoV-19/South Africa/KRISP-K002214/2020	EPI_ISL_515611	30/07/2020	Africa / South Africa / KZN / Amajuba	Human	Male	unknown	Live	Original	Oro-pharyngeal swab	B.1.1	GR	South Africa	Undertermined	No (couldn't determine the patient status as either pre-symptomatic / asymptomatic or symptomatic conclusively)
hCoV-19/South Africa/KRISP-K002215/2020	EPI_ISL_515612	30/07/2020	Africa / South Africa / KZN / Umgungundlovu	Human	Female	65	Live	Original	Oro-pharyngeal swab	B.1.1	GR	South Africa	Undertermined	No (couldn't determine the patient status as either pre-symptomatic / asymptomatic or symptomatic conclusively)
hCoV-19/South Africa/KRISP-K002216/2020	EPI_ISL_515613	18/07/2020	Africa / South Africa / KZN / uMkhanyakude	Human	Female	40	Live	Original	Oro-pharyngeal swab	B.1.1	GR	South Africa	Undertermined	No (couldn't determine the patient status as either pre-symptomatic / asymptomatic or symptomatic conclusively)
hCoV-19/South Africa/KRISP-K002217/2020	EPI_ISL_515614	18/07/2020	Africa / South Africa / KZN / Umgungundlovu	Human	Female	46	Live	Original	Oro-pharyngeal swab	B.1	G	South Africa	Undertermined	No (couldn't determine the patient status as either pre-symptomatic / asymptomatic or symptomatic conclusively)
hCoV-19/South Africa/KRISP-K002218/2020	EPI_ISL_515615	17/07/2020	Africa / South Africa / KZN / uMkhanyakude	Human	Female	12	Live	Original	Oro-pharyngeal swab	B.1.1	GR	South Africa	Undertermined	No (couldn't determine the patient status as either pre-symptomatic / asymptomatic or symptomatic conclusively)
hCoV-19/South Africa/KRISP-K002219/2020	EPI_ISL_515616	21/07/2020	Africa / South Africa / KZN / King Cetshwayo	Human	Female	62	Live	Original	Oro-pharyngeal swab	B.1.1	GR	South Africa	Undertermined	No (couldn't determine the patient status as either pre-symptomatic / asymptomatic or symptomatic conclusively)
hCoV-19/South Africa/KRISP-K002220/2020	EPI_ISL_515617	13/07/2020	Africa / South Africa / KZN / Umgungundlovu	Human	Female	39	Live	Original	Oro-pharyngeal swab	B.1	G	South Africa	Undertermined	No (couldn't determine the patient status as either pre-symptomatic / asymptomatic or symptomatic conclusively)
hCoV-19/South Africa/KRISP-K002221/2020	EPI_ISL_515618	20/07/2020	Africa / South Africa / KZN / King Cetshwayo	Human	Female	39	Live	Original	Oro-pharyngeal swab	B.1.1	GR	South Africa	Undertermined	No (couldn't determine the patient status as either pre-symptomatic / asymptomatic or symptomatic conclusively)
hCoV-19/South Africa/KRISP-K002222/2020	EPI_ISL_515619	21/07/2020	Africa / South Africa / KZN / Amajuba	Human	Male	57	Live	Original	Oro-pharyngeal swab	B.1.106	G	South Africa	Undertermined	No (couldn't determine the patient status as either pre-symptomatic / asymptomatic or symptomatic conclusively)
hCoV-19/South Africa/KRISP-K002223/2020	EPI_ISL_515620	21/07/2020	Africa / South Africa / KZN / Zululand	Human	Male	49	Live	Original	Oro-pharyngeal swab	B.1.1	GR	South Africa	Undertermined	No (couldn't determine the patient status as either pre-symptomatic / asymptomatic or symptomatic conclusively)
hCoV-19/South Africa/KRISP-K002225/2020	EPI_ISL_515621	21/07/2020	Africa / South Africa / KZN / Amajuba	Human	Female	18	Live	Original	Oro-pharyngeal swab	B.1.1	GR	South Africa	Undertermined	No (couldn't determine the patient status as either pre-symptomatic / asymptomatic or symptomatic conclusively)
hCoV-19/South Africa/KRISP-K002226/2020	EPI_ISL_515622	21/07/2020	Africa / South Africa / KZN / Amajuba	Human	Female	58	Live	Original	Oro-pharyngeal swab	B.1.1	GR	South Africa	Undertermined	No (couldn't determine the patient status as either pre-symptomatic / asymptomatic or symptomatic conclusively)
hCoV-19/South Africa/KRISP-K002227/2020	EPI_ISL_515623	21/07/2020	Africa / South Africa / KZN / Amajuba	Human	Female	29	Live	Original	Oro-pharyngeal swab	B.1.5	G	South Africa	Undertermined	No (couldn't determine the patient status as either pre-symptomatic / asymptomatic or symptomatic conclusively)
hCoV-19/South Africa/KRISP-K002228/2020	EPI_ISL_515624	20/07/2020	Africa / South Africa / KZN / Zululand	Human	Female	41	Live	Original	Oro-pharyngeal swab	B.1	G	South Africa	Undertermined	No (couldn't determine the patient status as either pre-symptomatic / asymptomatic or symptomatic conclusively)
hCoV-19/South Africa/KRISP-K002229/2020	EPI_ISL_515625	21/07/2020	Africa / South Africa / KZN / Amajuba	Human	Female	20	Live	Original	Oro-pharyngeal swab	B.1.1	GR	South Africa	Undertermined	No (couldn't determine the patient status as either pre-symptomatic / asymptomatic or symptomatic conclusively)
hCoV-19/South Africa/KRISP-K002230/2020	EPI_ISL_515626	21/07/2020	Africa / South Africa / KZN / Zululand	Human	Male	40	Live	Original	Oro-pharyngeal swab	B.1.1.1	GR	South Africa	Undertermined	No (couldn't determine the patient status as either pre-symptomatic / asymptomatic or symptomatic conclusively)
hCoV-19/South Africa/KRISP-K002231/2020	EPI_ISL_515627	18/07/2020	Africa / South Africa / KZN / Zululand	Human	Male	30	Live	Original	Oro-pharyngeal swab	B.1.1	GR	South Africa	Undertermined	No (couldn't determine the patient status as either pre-symptomatic / asymptomatic or symptomatic conclusively)
hCoV-19/South Africa/KRISP-K002234/2020	EPI_ISL_515628	21/07/2020	Africa / South Africa / KZN / Amajuba	Human	Female	20	Live	Original	Oro-pharyngeal swab	B.1.1.1	GR	South Africa	Undertermined	No (couldn't determine the patient status as either pre-symptomatic / asymptomatic or symptomatic conclusively)
hCoV-19/South Africa/KRISP-K002237/2020	EPI_ISL_515629	20/07/2020	Africa / South Africa / KZN / Zululand	Human	Male	32	Live	Original	Oro-pharyngeal swab	B.1.1.1	GR	South Africa	Undertermined	No (couldn't determine the patient status as either pre-symptomatic / asymptomatic or symptomatic conclusively)
hCoV-19/South Africa/KRISP-K002238/2020	EPI_ISL_515630	20/07/2020	Africa / South Africa / KZN / uMzinyathi	Human	Male	38	Live	Original	Oro-pharyngeal swab	B.1	G	South Africa	Undertermined	No (couldn't determine the patient status as either pre-symptomatic / asymptomatic or symptomatic conclusively)
hCoV-19/South Africa/KRISP-K002239/2020	EPI_ISL_515631	20/07/2020	Africa / South Africa / KZN / uThukela	Human	Male	28	Live	Original	Oro-pharyngeal swab	B.1.1	GR	South Africa	Undertermined	No (couldn't determine the patient status as either pre-symptomatic / asymptomatic or symptomatic conclusively)
hCoV-19/South Africa/KRISP-K002241/2020	EPI_ISL_515632	21/07/2020	Africa / South Africa / KZN / Amajuba	Human	Female	28	Live	Original	Oro-pharyngeal swab	B.1.1	GR	South Africa	Undertermined	No (couldn't determine the patient status as either pre-symptomatic / asymptomatic or symptomatic conclusively)
hCoV-19/South Africa/KRISP-K002242/2020	EPI_ISL_515633	21/07/2020	Africa / South Africa / KZN / Amajuba	Human	Male	35	Live	Original	Oro-pharyngeal swab	B.1.5	G	South Africa	Undertermined	No (couldn't determine the patient status as either pre-symptomatic / asymptomatic or symptomatic conclusively)
hCoV-19/South Africa/KRISP-K002243/2020	EPI_ISL_515634	21/07/2020	Africa / South Africa / KZN / Zululand	Human	Female	38	Live	Original	Oro-pharyngeal swab	B.1.1	GR	South Africa	Undertermined	No (couldn't determine the patient status as either pre-symptomatic / asymptomatic or symptomatic conclusively)
hCoV-19/South Africa/KRISP-K002244/2020	EPI_ISL_515635	20/07/2020	Africa / South Africa / KZN / King Cetshwayo	Human	Male	37	Live	Original	Oro-pharyngeal swab	B.1.106	G	South Africa	Undertermined	No (couldn't determine the patient status as either pre-symptomatic / asymptomatic or symptomatic conclusively)
hCoV-19/South Africa/KRISP-K002249/2020	EPI_ISL_515636	21/07/2020	Africa / South Africa / KZN / Amajuba	Human	Female	36	Live	Original	Oro-pharyngeal swab	B.1.1	GR	South Africa	Undertermined	No (couldn't determine the patient status as either pre-symptomatic / asymptomatic or symptomatic conclusively)
hCoV-19/South Africa/KRISP-K002250/2020	EPI_ISL_515637	20/07/2020	Africa / South Africa / KZN / King Cetshwayo	Human	Male	29	Live	Original	Oro-pharyngeal swab	B.1	G	South Africa	Undertermined	No (couldn't determine the patient status as either pre-symptomatic / asymptomatic or symptomatic conclusively)
hCoV-19/South Africa/KRISP-K002251/2020	EPI_ISL_515638	21/07/2020	Africa / South Africa / KZN / Amajuba	Human	Male	11	Live	Original	Oro-pharyngeal swab	B.1.1	GR	South Africa	Undertermined	No (couldn't determine the patient status as either pre-symptomatic / asymptomatic or symptomatic conclusively)
hCoV-19/South Africa/KRISP-K002253/2020	EPI_ISL_515639	21/07/2020	Africa / South Africa / KZN / Amajuba	Human	Female	39	Live	Original	Oro-pharyngeal swab	B.1.1	GR	South Africa	Undertermined	No (couldn't determine the patient status as either pre-symptomatic / asymptomatic or symptomatic conclusively)
hCoV-19/South Africa/KRISP-K002255/2020	EPI_ISL_515640	21/07/2020	Africa / South Africa / KZN / Amajuba	Human	Male	38	Live	Original	Oro-pharyngeal swab	B.1.1	GR	South Africa	Undertermined	No (couldn't determine the patient status as either pre-symptomatic / asymptomatic or symptomatic conclusively)
hCoV-19/South Africa/KRISP-K002256/2020	EPI_ISL_515641	21/07/2020	Africa / South Africa / KZN / Ugu	Human	Female	28	Live	Original	Oro-pharyngeal swab	B.1.1	GR	South Africa	Undertermined	No (couldn't determine the patient status as either pre-symptomatic / asymptomatic or symptomatic conclusively)
hCoV-19/South Africa/KRISP-K002257/2020	EPI_ISL_515642	19/07/2020	Africa / South Africa / KZN / uMzinyathi	Human	Female	40	Live	Original	Oro-pharyngeal swab	B.1.1	GR	South Africa	Undertermined	No (couldn't determine the patient status as either pre-symptomatic / asymptomatic or symptomatic conclusively)
hCoV-19/South Africa/KRISP-K002258/2020	EPI_ISL_515643	18/07/2020	Africa / South Africa / KZN / uMzinyathi	Human	Male	40	Live	Original	Oro-pharyngeal swab	B.1.1	GR	South Africa	Undertermined	No (couldn't determine the patient status as either pre-symptomatic / asymptomatic or symptomatic conclusively)
hCoV-19/South Africa/KRISP-K002259/2020	EPI_ISL_515644	18/07/2020	Africa / South Africa / KZN / uMzinyathi	Human	Female	13	Live	Original	Oro-pharyngeal swab	B.1.1.1	GR	South Africa	Undertermined	No (couldn't determine the patient status as either pre-symptomatic / asymptomatic or symptomatic conclusively)
hCoV-19/South Africa/KRISP-K002260/2020	EPI_ISL_515645	16/07/2020	Africa / South Africa / KZN / Umgungundlovu	Human	Female	37	Live	Original	Oro-pharyngeal swab	B.1.5	G	South Africa	Undertermined	No (couldn't determine the patient status as either pre-symptomatic / asymptomatic or symptomatic conclusively)
hCoV-19/South Africa/KRISP-K002263/2020	EPI_ISL_515646	19/07/2020	Africa / South Africa / KZN / uMzinyathi	Human	Female	46	Live	Original	Oro-pharyngeal swab	B.1.1	GR	South Africa	Undertermined	No (couldn't determine the patient status as either pre-symptomatic / asymptomatic or symptomatic conclusively)
hCoV-19/South Africa/KRISP-K002264/2020	EPI_ISL_515647	19/07/2020	Africa / South Africa / KZN / Umgungundlovu	Human	Male	1	Live	Original	Oro-pharyngeal swab	B.1.1	GR	South Africa	Undertermined	No (couldn't determine the patient status as either pre-symptomatic / asymptomatic or symptomatic conclusively)
hCoV-19/South Africa/KRISP-K002265/2020	EPI_ISL_515648	20/07/2020	Africa / South Africa / KZN / Umgungundlovu	Human	Female	25	Live	Original	Oro-pharyngeal swab	B.1.1	GR	South Africa	Undertermined	No (couldn't determine the patient status as either pre-symptomatic / asymptomatic or symptomatic conclusively)
hCoV-19/South Africa/KRISP-K002266/2020	EPI_ISL_515649	20/07/2020	Africa / South Africa / KZN / Ugu	Human	Male	26	Live	Original	Oro-pharyngeal swab	B.1.1	GR	South Africa	Undertermined	No (couldn't determine the patient status as either pre-symptomatic / asymptomatic or symptomatic conclusively)
hCoV-19/South Africa/KRISP-K002268/2020	EPI_ISL_515650	20/07/2020	Africa / South Africa / KZN / uMzinyathi	Human	Female	35	Live	Original	Oro-pharyngeal swab	B.1.1.1	GR	South Africa	Undertermined	No (couldn't determine the patient status as either pre-symptomatic / asymptomatic or symptomatic conclusively)
hCoV-19/South Africa/KRISP-K002269/2020	EPI_ISL_515651	21/07/2020	Africa / South Africa / KZN / uMzinyathi	Human	Male	39	Live	Original	Oro-pharyngeal swab	B.1.1.1	GR	South Africa	Undertermined	No (couldn't determine the patient status as either pre-symptomatic / asymptomatic or symptomatic conclusively)
hCoV-19/South Africa/KRISP-K002270/2020	EPI_ISL_515652	20/07/2020	Africa / South Africa / KZN / Umgungundlovu	Human	Female	24	Live	Original	Oro-pharyngeal swab	B.1.1	GR	South Africa	Undertermined	No (couldn't determine the patient status as either pre-symptomatic / asymptomatic or symptomatic conclusively)
hCoV-19/South Africa/KRISP-K002271/2020	EPI_ISL_515653	19/07/2020	Africa / South Africa / KZN / uMzinyathi	Human	Female	69	Live	Original	Oro-pharyngeal swab	B.1.1	GR	South Africa	Undertermined	No (couldn't determine the patient status as either pre-symptomatic / asymptomatic or symptomatic conclusively)
hCoV-19/South Africa/KRISP-K002273/2020	EPI_ISL_515654	21/07/2020	Africa / South Africa / KZN / uMzinyathi	Human	Female	54	Live	Original	Oro-pharyngeal swab	B.1.1.1	GR	South Africa	Undertermined	No (couldn't determine the patient status as either pre-symptomatic / asymptomatic or symptomatic conclusively)
hCoV-19/South Africa/KRISP-K002274/2020	EPI_ISL_515655	21/07/2020	Africa / South Africa / KZN / Amajuba	Human	Male	33	Live	Original	Oro-pharyngeal swab	B.1.1	GR	South Africa	Undertermined	No (couldn't determine the patient status as either pre-symptomatic / asymptomatic or symptomatic conclusively)
hCoV-19/South Africa/KRISP-K002276/2020	EPI_ISL_515656	19/07/2020	Africa / South Africa / KZN / uMzinyathi	Human	Female	38	Live	Original	Oro-pharyngeal swab	B.1.1	GR	South Africa	Undertermined	No (couldn't determine the patient status as either pre-symptomatic / asymptomatic or symptomatic conclusively)
hCoV-19/South Africa/KRISP-K002277/2020	EPI_ISL_515657	18/07/2020	Africa / South Africa / KZN / uMzinyathi	Human	Female	41	Live	Original	Oro-pharyngeal swab	B.1.1	GR	South Africa	Undertermined	No (couldn't determine the patient status as either pre-symptomatic / asymptomatic or symptomatic conclusively)
hCoV-19/South Africa/KRISP-K002278/2020	EPI_ISL_515658	21/07/2020	Africa / South Africa / KZN / Amajuba	Human	Male	1	Live	Original	Oro-pharyngeal swab	B.1.1	GR	South Africa	Undertermined	No (couldn't determine the patient status as either pre-symptomatic / asymptomatic or symptomatic conclusively)
hCoV-19/South Africa/KRISP-K002279/2020	EPI_ISL_515659	21/07/2020	Africa / South Africa / KZN / Amajuba	Human	Male	26	Live	Original	Oro-pharyngeal swab	B.1.1	GR	South Africa	Undertermined	No (couldn't determine the patient status as either pre-symptomatic / asymptomatic or symptomatic conclusively)
hCoV-19/South Africa/KRISP-K002280/2020	EPI_ISL_515660	21/07/2020	Africa / South Africa / KZN / Amajuba	Human	Male	50	Live	Original	Oro-pharyngeal swab	B.1.1.1	GR	South Africa	Undertermined	No (couldn't determine the patient status as either pre-symptomatic / asymptomatic or symptomatic conclusively)

hCoV-19/South Africa/KRISP-K002281/2020	EPI_ISL_515661	21/07/2020	Africa / South Africa / KZN / Amajuba	Human		Female	21	Live	Original	Oro-pharyngeal swab	B.1.1.1	GR	South Africa	Undertermined	No (couldn't determine the patient status as either pre-symptomatic / asymptomatic or symptomatic conclusively)
hCoV-19/South Africa/KRISP-K002282/2020	EPI_ISL_515662	21/07/2020	Africa / South Africa / KZN / Amajuba	Human		Female	50	Live	Original	Oro-pharyngeal swab	B.1.1.1	GR	South Africa	Undertermined	No (couldn't determine the patient status as either pre-symptomatic / asymptomatic or symptomatic conclusively)
hCoV-19/South Africa/KRISP-K002283/2020	EPI_ISL_515663	12/07/2020	Africa / South Africa / KZN / Umgungundlovu	Human		Male	17	Live	Original	Oro-pharyngeal swab	B.1.1	GR	South Africa	Undertermined	No (couldn't determine the patient status as either pre-symptomatic / asymptomatic or symptomatic conclusively)
hCoV-19/South Africa/KRISP-K002284/2020	EPI_ISL_515664	21/07/2020	Africa / South Africa / KZN / Amajuba	Human		Female	21	Live	Original	Oro-pharyngeal swab	B.1.1	G	South Africa	Undertermined	No (couldn't determine the patient status as either pre-symptomatic / asymptomatic or symptomatic conclusively)
hCoV-19/South Africa/KRISP-K002287/2020	EPI_ISL_515665	19/07/2020	Africa / South Africa / KZN / uThukela	Human		Male	1	Live	Original	Oro-pharyngeal swab	B.1.1	GR	South Africa	Undertermined	No (couldn't determine the patient status as either pre-symptomatic / asymptomatic or symptomatic conclusively)
hCoV-19/South Africa/KRISP-K002288/2020	EPI_ISL_515666	21/07/2020	Africa / South Africa / KZN / Umgungundlovu	Human		Female	38	Live	Original	Oro-pharyngeal swab	B.1.1	GR	South Africa	Undertermined	No (couldn't determine the patient status as either pre-symptomatic / asymptomatic or symptomatic conclusively)
hCoV-19/South Africa/KRISP-K002289/2020	EPI_ISL_515667	20/07/2020	Africa / South Africa / KZN / uMzinyathi	Human		Female	20	Live	Original	Oro-pharyngeal swab	B.1.1	GR	South Africa	Undertermined	No (couldn't determine the patient status as either pre-symptomatic / asymptomatic or symptomatic conclusively)
hCoV-19/South Africa/KRISP-K002290/2020	EPI_ISL_515668	23/07/2020	Africa / South Africa / KZN / King Cetshwayo	Human		Female	34	Live	Original	Oro-pharyngeal swab	B.1.1	GR	South Africa	Undertermined	No (couldn't determine the patient status as either pre-symptomatic / asymptomatic or symptomatic conclusively)
hCoV-19/South Africa/KRISP-K002291/2020	EPI_ISL_515669	22/07/2020	Africa / South Africa / KZN / uThukela	Human		Female	56	Live	Original	Oro-pharyngeal swab	B.1.1.1	GR	South Africa	Undertermined	No (couldn't determine the patient status as either pre-symptomatic / asymptomatic or symptomatic conclusively)
hCoV-19/South Africa/KRISP-K002292/2020	EPI_ISL_515670	22/07/2020	Africa / South Africa / KZN / King Cetshwayo	Human		Female	23	Live	Original	Oro-pharyngeal swab	B.1.1	GR	South Africa	Undertermined	No (couldn't determine the patient status as either pre-symptomatic / asymptomatic or symptomatic conclusively)
hCoV-19/South Africa/KRISP-K002293/2020	EPI_ISL_515671	23/07/2020	Africa / South Africa / KZN / Amajuba	Human		Female	12	Live	Original	Oro-pharyngeal swab	B.1.1.1	GR	South Africa	Undertermined	No (couldn't determine the patient status as either pre-symptomatic / asymptomatic or symptomatic conclusively)
hCoV-19/South Africa/KRISP-K002295/2020	EPI_ISL_515672	23/07/2020	Africa / South Africa / KZN / Amajuba	Human		Male	61	Live	Original	Oro-pharyngeal swab	B.1.1	GR	South Africa	Undertermined	No (couldn't determine the patient status as either pre-symptomatic / asymptomatic or symptomatic conclusively)
hCoV-19/South Africa/KRISP-K002296/2020	EPI_ISL_515673	21/07/2020	Africa / South Africa / KZN / Umgungundlovu	Human		Female	51	Live	Original	Oro-pharyngeal swab	B.1.1	GR	South Africa	Undertermined	No (couldn't determine the patient status as either pre-symptomatic / asymptomatic or symptomatic conclusively)
hCoV-19/South Africa/KRISP-K002297/2020	EPI_ISL_515674	20/07/2020	Africa / South Africa / KZN / uMzinyathi	Human		Male	22	Live	Original	Oro-pharyngeal swab	B.1.1.1	GR	South Africa	Undertermined	No (couldn't determine the patient status as either pre-symptomatic / asymptomatic or symptomatic conclusively)
hCoV-19/South Africa/KRISP-K002298/2020	EPI_ISL_515675	23/07/2020	Africa / South Africa / KZN / uMzinyathi	Human		Male	26	Live	Original	Oro-pharyngeal swab	B.1.1	GR	South Africa	Undertermined	No (couldn't determine the patient status as either pre-symptomatic / asymptomatic or symptomatic conclusively)
hCoV-19/South Africa/KRISP-K002299/2020	EPI_ISL_515676	22/07/2020	Africa / South Africa / KZN / King Cetshwayo	Human		Female	23	Live	Original	Oro-pharyngeal swab	B.1.1	GR	South Africa	Undertermined	No (couldn't determine the patient status as either pre-symptomatic / asymptomatic or symptomatic conclusively)
hCoV-19/South Africa/KRISP-K002300/2020	EPI_ISL_515677	21/07/2020	Africa / South Africa / KZN / Umgungundlovu	Human		Female	68	Live	Original	Oro-pharyngeal swab	B.1.1.1	GR	South Africa	Undertermined	No (couldn't determine the patient status as either pre-symptomatic / asymptomatic or symptomatic conclusively)
hCoV-19/South Africa/KRISP-K002301/2020	EPI_ISL_515678	22/07/2020	Africa / South Africa / KZN / uThukela	Human		Female	46	Live	Original	Oro-pharyngeal swab	B.1.1	GR	South Africa	Undertermined	No (couldn't determine the patient status as either pre-symptomatic / asymptomatic or symptomatic conclusively)
hCoV-19/South Africa/KRISP-K002302/2020	EPI_ISL_515679	23/07/2020	Africa / South Africa / KZN / King Cetshwayo	Human		Female	37	Live	Original	Oro-pharyngeal swab	B.1.1	GR	South Africa	Undertermined	No (couldn't determine the patient status as either pre-symptomatic / asymptomatic or symptomatic conclusively)
hCoV-19/South Africa/KRISP-K002303/2020	EPI_ISL_515680	23/07/2020	Africa / South Africa / KZN / Amajuba	Human		Female	69	Live	Original	Oro-pharyngeal swab	B.1.1.1	GR	South Africa	Undertermined	No (couldn't determine the patient status as either pre-symptomatic / asymptomatic or symptomatic conclusively)
hCoV-19/South Africa/KRISP-K002304/2020	EPI_ISL_515681	23/07/2020	Africa / South Africa / KZN / Amajuba	Human		Female	27	Live	Original	Oro-pharyngeal swab	B.1.1.1	GR	South Africa	Undertermined	No (couldn't determine the patient status as either pre-symptomatic / asymptomatic or symptomatic conclusively)
hCoV-19/South Africa/KRISP-K002306/2020	EPI_ISL_515682	21/07/2020	Africa / South Africa / KZN / Umgungundlovu	Human		Male	34	Live	Original	Oro-pharyngeal swab	B.1.1	GR	South Africa	Undertermined	No (couldn't determine the patient status as either pre-symptomatic / asymptomatic or symptomatic conclusively)
hCoV-19/South Africa/KRISP-K002307/2020	EPI_ISL_515683	23/07/2020	Africa / South Africa / KZN / uThukela	Human		Female	58	Live	Original	Oro-pharyngeal swab	B.1.1	GR	South Africa	Undertermined	No (couldn't determine the patient status as either pre-symptomatic / asymptomatic or symptomatic conclusively)
hCoV-19/South Africa/KRISP-K002308/2020	EPI_ISL_515684	22/07/2020	Africa / South Africa / KZN / uThukela	Human		Female	36	Live	Original	Oro-pharyngeal swab	B.1.1	GR	South Africa	Undertermined	No (couldn't determine the patient status as either pre-symptomatic / asymptomatic or symptomatic conclusively)
hCoV-19/South Africa/KRISP-K002309/2020	EPI_ISL_515685	23/07/2020	Africa / South Africa / KZN / Amajuba	Human		Male	10	Live	Original	Oro-pharyngeal swab	B.1.1	GR	South Africa	Undertermined	No (couldn't determine the patient status as either pre-symptomatic / asymptomatic or symptomatic conclusively)
hCoV-19/South Africa/KRISP-K002310/2020	EPI_ISL_515686	23/07/2020	Africa / South Africa / KZN / Amajuba	Human		Female	63	Live	Original	Oro-pharyngeal swab	B.1.1	GR	South Africa	Undertermined	No (couldn't determine the patient status as either pre-symptomatic / asymptomatic or symptomatic conclusively)
hCoV-19/South Africa/KRISP-K002311/2020	EPI_ISL_515687	22/07/2020	Africa / South Africa / KZN / Ilembe	Human		Female	55	Live	Original	Oro-pharyngeal swab	B.1.1.1	GR	South Africa	Undertermined	No (couldn't determine the patient status as either pre-symptomatic / asymptomatic or symptomatic conclusively)
hCoV-19/South Africa/KRISP-K002312/2020	EPI_ISL_515688	22/07/2020	Africa / South Africa / KZN / King Cetshwayo	Human		Female	39	Live	Original	Oro-pharyngeal swab	B.1.1	GR	South Africa	Undertermined	No (couldn't determine the patient status as either pre-symptomatic / asymptomatic or symptomatic conclusively)
hCoV-19/South Africa/KRISP-K002313/2020	EPI_ISL_515689	22/07/2020	Africa / South Africa / KZN / Umgungundlovu	Human		Female	52	Live	Original	Oro-pharyngeal swab	B.1	G	South Africa	Undertermined	No (couldn't determine the patient status as either pre-symptomatic / asymptomatic or symptomatic conclusively)
hCoV-19/South Africa/KRISP-K002314/2020	EPI_ISL_515690	22/07/2020	Africa / South Africa / KZN / Umgungundlovu	Human		Female	90	Live	Original	Oro-pharyngeal swab	B.1.5	G	South Africa	Undertermined	No (couldn't determine the patient status as either pre-symptomatic / asymptomatic or symptomatic conclusively)
hCoV-19/South Africa/KRISP-K002315/2020	EPI_ISL_515691	22/07/2020	Africa / South Africa / KZN / Umgungundlovu	Human		Female	83	Live	Original	Oro-pharyngeal swab	B.1.5	G	South Africa	Undertermined	No (couldn't determine the patient status as either pre-symptomatic / asymptomatic or symptomatic conclusively)
hCoV-19/South Africa/KRISP-K002316/2020	EPI_ISL_515692	22/07/2020	Africa / South Africa / KZN / Umgungundlovu	Human		Female	39	Live	Original	Oro-pharyngeal swab	B.1.1	GR	South Africa	Undertermined	No (couldn't determine the patient status as either pre-symptomatic / asymptomatic or symptomatic conclusively)
hCoV-19/South Africa/KRISP-K002317/2020	EPI_ISL_515693	22/07/2020	Africa / South Africa / KZN / Umgungundlovu	Human		Male	67	Live	Original	Oro-pharyngeal swab	B.1.5	G	South Africa	Undertermined	No (couldn't determine the patient status as either pre-symptomatic / asymptomatic or symptomatic conclusively)
hCoV-19/South Africa/KRISP-K002318/2020	EPI_ISL_515694	22/07/2020	Africa / South Africa / KZN / King Cetshwayo	Human		Female	31	Live	Original	Oro-pharyngeal swab	B.1.1	GR	South Africa	Undertermined	No (couldn't determine the patient status as either pre-symptomatic / asymptomatic or symptomatic conclusively)
hCoV-19/South Africa/KRISP-K002319/2020	EPI_ISL_515695	22/07/2020	Africa / South Africa / KZN / Ilembe	Human		Female	36	Live	Original	Oro-pharyngeal swab	B.1.1	GR	South Africa	Undertermined	No (couldn't determine the patient status as either pre-symptomatic / asymptomatic or symptomatic conclusively)
hCoV-19/South Africa/KRISP-K002320/2020	EPI_ISL_515696	22/07/2020	Africa / South Africa / KZN / Umgungundlovu	Human		Male	64	Live	Original	Oro-pharyngeal swab	B.1.5	G	South Africa	Undertermined	No (couldn't determine the patient status as either pre-symptomatic / asymptomatic or symptomatic conclusively)
hCoV-19/South Africa/KRISP-K002321/2020	EPI_ISL_515697	22/07/2020	Africa / South Africa / KZN / King Cetshwayo	Human		Female	21	Live	Original	Oro-pharyngeal swab	B.1.1	GR	South Africa	Undertermined	No (couldn't determine the patient status as either pre-symptomatic / asymptomatic or symptomatic conclusively)
hCoV-19/South Africa/KRISP-K002322/2020	EPI_ISL_515698	22/07/2020	Africa / South Africa / KZN / Umgungundlovu	Human		Female	80	Live	Original	Oro-pharyngeal swab	B.1	G	South Africa	Undertermined	No (couldn't determine the patient status as either pre-symptomatic / asymptomatic or symptomatic conclusively)
hCoV-19/South Africa/KRISP-K002323/2020	EPI_ISL_515699	23/07/2020	Africa / South Africa / KZN / Zululand	Human		Female	57	Live	Original	Oro-pharyngeal swab	B.1.1	GR	South Africa	Undertermined	No (couldn't determine the patient status as either pre-symptomatic / asymptomatic or symptomatic conclusively)
hCoV-19/USA/SC-20-0546-T00/2020	EPI_ISL_516748	27/05/2020	North America / USA / South Carolina	Human		Male	18	Live	Original		B.1	GH	USA	Undertermined	No (couldn't determine the patient status as either pre-symptomatic / asymptomatic or symptomatic conclusively)
hCoV-19/Indonesia/YO-202449/2020	EPI_ISL_516800	22/06/2020	Asia / Indonesia / Special Region of Yogyakarta	Human		Male	77	Live	Original	Nasopharyngeal swab	B.1.36	GH	Indonesia	Undertermined	No (couldn't determine the patient status as either pre-symptomatic / asymptomatic or symptomatic conclusively)
hCoV-19/Singapore/829/2020	EPI_ISL_516801	10/03/2020	Asia / Singapore	Human		Male	30	Hospitalized	Original		B.1	GH	Singapore	Undertermined	No (couldn't determine the patient status as either pre-symptomatic / asymptomatic or symptomatic conclusively)
hCoV-19/Singapore/830/2020	EPI_ISL_516802	12/03/2020	Asia / Singapore	Human		Male	53	Hospitalized	Original		B.6	O	Singapore	Undertermined	No (couldn't determine the patient status as either pre-symptomatic / asymptomatic or symptomatic conclusively)
hCoV-19/Singapore/831/2020	EPI_ISL_516803	22/03/2020	Asia / Singapore	Human		Male	24	Hospitalized	Original		B.1	GH	Singapore	Undertermined	No (couldn't determine the patient status as either pre-symptomatic / asymptomatic or symptomatic conclusively)
hCoV-19/Singapore/832/2020	EPI_ISL_516804	24/03/2020	Asia / Singapore	Human		Female	56	Hospitalized	Original		B.6	O	Singapore	Undertermined	No (couldn't determine the patient status as either pre-symptomatic / asymptomatic or symptomatic conclusively)
hCoV-19/Singapore/833/2020	EPI_ISL_516805	25/03/2020	Asia / Singapore	Human		Female	29	Hospitalized	Original		A.3	S	Singapore	Undertermined	No (couldn't determine the patient status as either pre-symptomatic / asymptomatic or symptomatic conclusively)
hCoV-19/Indonesia/YO-200927/2020	EPI_ISL_516806	16/05/2020	Asia / Indonesia / Special Region of Yogyakarta	Human		Male	30	Live	Original	Nasopharyngeal swab	B	L	Indonesia	Undertermined	No (couldn't determine the patient status as either pre-symptomatic / asymptomatic or symptomatic conclusively)
hCoV-19/Indonesia/YO-781481/2020	EPI_ISL_516829	10/08/2020	Asia / Indonesia / Special Region of Yogyakarta	Human		Female	83	Live	Original	Nasopharyngeal swab	B.1.36	GH	Indonesia	Undertermined	No (couldn't determine the patient status as either pre-symptomatic / asymptomatic or symptomatic conclusively)
hCoV-19/Moldova/ICGEB_MD1/2020	EPI_ISL_516934	18/06/2020	Europe / Moldova / Chisinau	Human		Female	30	Live	Original	Nasopharyngeal swab	B.1.1	GR	Moldova	Undertermined	No (couldn't determine the patient status as either pre-symptomatic / asymptomatic or symptomatic conclusively)
hCoV-19/Moldova/ICGEB_MD7/2020	EPI_ISL_516936	17/06/2020	Europe / Moldova / Straseni	Human		Female	32	Live	Original	Nasopharyngeal swab	B.1.1	GR	Moldova	Undertermined	No (couldn't determine the patient status as either pre-symptomatic / asymptomatic or symptomatic conclusively)
hCoV-19/Moldova/ICGEB_MD6/2020	EPI_ISL_516938	17/06/2020	Europe / Moldova / Rabnita	Human		Female	63	Live	Original	Nasopharyngeal swab	B.1.1	GR	Moldova	Undertermined	No (couldn't determine the patient status as either pre-symptomatic / asymptomatic or symptomatic conclusively)
hCoV-19/India/UP-NBRI-N11-TCR316/2020	EPI_ISL_516940	02/04/2020	Asia / India / Uttar Pradesh / Lucknow / Balrampur	Human		Male	48	Asymptomatic	Original		B.6	O	India	Pre-symptomatic / Asymptomatic	Yes
hCoV-19/India/UP-NBRI-N12-TCR319/2020	EPI_ISL_516941	02/04/2020	Asia / India / Uttar Pradesh / Lucknow / Balrampur	Human		Male	47	Asymptomatic	Original		B.6	O	India	Pre-symptomatic / Asymptomatic	Yes
hCoV-19/India/UP-NBRI-N13-TCR323/2020	EPI_ISL_516942	02/04/2020	Asia / India / Uttar Pradesh / Lucknow / Balrampur	Human		Male	55	Asymptomatic	Original		B.6	O	India	Pre-symptomatic / Asymptomatic	Yes
hCoV-19/India/UP-NBRI-N14-TCR324/2020	EPI_ISL_516943	02/04/2020	Asia / India / Uttar Pradesh / Lucknow / Balrampur	Human		Male	47	Asymptomatic	Original		B.6	O	India	Pre-symptomatic / Asymptomatic	Yes
hCoV-19/India/UP-NBRI-N15-TCR325/2020	EPI_ISL_516944	02/04/2020	Asia / India / Uttar Pradesh / Lucknow / Balrampur	Human		Male	32	Asymptomatic	Original		B.6	O	India	Pre-symptomatic / Asymptomatic	Yes
hCoV-19/India/UP-NBRI-N16-TCR338/2020	EPI_ISL_516945	01/04/2020	Asia / India / Uttar Pradesh / Azamgarh	Human		Male	26	Asymptomatic	Original		B	O	India	Pre-symptomatic / Asymptomatic	Yes
hCoV-19/India/UP-NBRI-N17-TCP057/2020	EPI_ISL_516946	11/03/2020	Asia / India / Uttar Pradesh / Lucknow	Human		Female	38	Symptomatic	Original		B.1.1	GR	India	Symptomatic	Yes
hCoV-19/India/UP-NBRI-N18-TDU012/2020	EPI_ISL_516947	14/05/2020	Asia / India / Uttar Pradesh / Farukhabad	Human		Male	45	Suspected Corona	Original		B.6	O	India	Undertermined	No (couldn't determine the patient status as either pre-symptomatic / asymptomatic or symptomatic conclusively)
hCoV-19/India/UP-NBRI-N19-TCP645/2020	EPI_ISL_516948	18/03/2020	Asia / India / Uttar Pradesh / Lucknow	Human		Male	19	Symptomatic	Original		B.1.11	G	India	Symptomatic	Yes
hCoV-19/India/UP-NBRI-N20-TDU302/2020	EPI_ISL_516949	14/05/2020	Asia / India / Uttar Pradesh / Lakhimpur	Human		Male	15	Suspected coronavirus infection	Original		B.1	G	India	Undertermined	No (couldn't determine the patient status as either pre-symptomatic / asymptomatic or symptomatic conclusively)
hCoV-19/India/UP-NBRI-N21-TDU019/2020	EPI_ISL_516950	14/05/2020	Asia / India / Uttar Pradesh / Farukhabad	Human		Male	26	Suspected Corona	Original		B.1.1	GR	India	Undertermined	No (couldn't determine the patient status as either pre-symptomatic / asymptomatic or symptomatic conclusively)
hCoV-19/India/UP-NBRI-N22-TDU885/2020	EPI_ISL_516951	15/05/2020	Asia / India / Uttar Pradesh / Hardoi	Human		Male	25	Suspected Corona	Original		B.1	G	India	Undertermined	No (couldn't determine the patient status as either pre-symptomatic / asymptomatic or symptomatic conclusively)
hCoV-19/India/UP-NBRI-N23-TDU543/2020	EPI_ISL_516952	15/05/2020	Asia / India / Uttar Pradesh / Lucknow	Human		Male	55	Suspected coronavirus infection	Original		B.6	O	India	Undertermined	No (couldn't determine the patient status as either pre-symptomatic / asymptomatic or symptomatic conclusively)
hCoV-19/India/UP-NBRI-N24-TDU424/2020	EPI_ISL_516953	15/05/2020	Asia / India / Uttar Pradesh / Lucknow	Human		Male	41	Suspected coronavirus infection	Original		B.1.86	G	India	Undertermined	No (couldn't determine the patient status as either pre-symptomatic / asymptomatic or symptomatic conclusively)
hCoV-19/India/UP-NBRI-N25-TDU890/2020	EPI_ISL_516954	15/05/2020	Asia / India / Uttar Pradesh / Hardoi	Human		Male	26	Suspected Corona	Original		B.1	G	India	Undertermined	No (couldn't determine the patient status as either pre-symptomatic / asymptomatic or symptomatic conclusively)
hCoV-19/India/UP-NBRI-N26-TDV142/2020	EPI_ISL_516955	15/05/2020	Asia / India / Uttar Pradesh / Kanpur	Human		Male	23	Suspected Corona	Original		B.1	G	India	Undertermined	No (couldn't determine the patient status as either pre-symptomatic / asymptomatic or symptomatic conclusively)
hCoV-19/India/UP-NBRI-N27-TDV034/2020	EPI_ISL_516956	15/05/2020	Asia / India / Uttar Pradesh / Kannauj	Human		Male	29	Suspected Corona	Original		B.1.5	G	India	Undertermined	No (couldn't determine the patient status as either pre-symptomatic / asymptomatic or symptomatic conclusively)
hCoV-19/India/UP-NBRI-N28-TDV622/2020	EPI_ISL_516957	15/05/2020	Asia / India / Uttar Pradesh / Shahjahapur	Human		Male	22	Suspected coronavirus infection	Original		B.1	G	India	Undertermined	No (couldn't determine the patient status as either pre-symptomatic / asymptomatic or symptomatic conclusively)
hCoV-19/India/UP-NBRI-N29-TDV451/2020	EPI_ISL_516958	15/05/2020	Asia / India / Uttar Pradesh / Moradabad	Human		Male	36	Suspected Corona	Original		B.1.1	GR	India	Undertermined	No (couldn't determine the patient status as either pre-symptomatic / asymptomatic or symptomatic conclusively)
hCoV-19/India/UP-NBRI-N30-TDV623/2020	EPI_ISL_516959	15/05/2020	Asia / India / Uttar Pradesh / Shahjahapur	Human		Male	23	Suspected coronavirus infection	Original		B.1	G	India	Undertermined	No (couldn't determine the patient status as either pre-symptomatic / asymptomatic or symptomatic conclusively)
hCoV-19/India/UP-NBRI-N31-TCX154/2020	EPI_ISL_516960	16/04/2020	Asia / India / Uttar Pradesh / Lucknow	Human		Male	18	Asymptomatic	Original		B.6	O	India	Pre-symptomatic / Asymptomatic	Yes
hCoV-19/India/UP-NBRI-N32-TCX152/2020	EPI_ISL_516961	16/04/2020	Asia / India / Uttar Pradesh / Lucknow	Human		Male	59	Asymptomatic	Original		B.6	O	India	Pre-symptomatic / Asymptomatic	Yes
hCoV-19/India/UP-NBRI-N33-TCX148/2020	EPI_ISL_516962	16/04/2020	Asia / India / Uttar Pradesh / Lucknow	Human		Male	29	Asymptomatic	Original		B.6	O	India	Pre-symptomatic / Asymptomatic	Yes
hCoV-19/India/UP-NBRI-N34-TDV641/2020	EPI_ISL_516963	15/05/2020	Asia / India / Uttar Pradesh / Shahjahapur	Human		Male	26	Suspected coronavirus infection	Original		B.1.1	GR	India	Undertermined	No (couldn't determine the patient status as either pre-symptomatic / asymptomatic or symptomatic conclusively)
hCoV-19/India/UP-NBRI-N35-TDV640/2020	EPI_ISL_516964	15/05/2020	Asia / India / Uttar Pradesh / Shahjahapur	Human		Male	29	Suspected coronavirus infection	Original		B.1	G	India	Undertermined	No (couldn't determine the patient status as either pre-symptomatic / asymptomatic or symptomatic conclusively)
hCoV-19/India/UP-NBRI-N36-TDV771/2020	EPI_ISL_516965	15/05/2020	Asia / India / Uttar Pradesh / Sambhal	Human		Male	27	Suspected coronavirus infection	Original		B.6	O	India	Undertermined	No (couldn't determine the patient status as either pre-symptomatic / asymptomatic or symptomatic conclusively)
hCoV-19/India/UP-NBRI-N37-TDT658/2020	EPI_ISL_516966	14/05/2020	Asia / India / Uttar Pradesh / Lucknow	Human		Female	55	Suspected coronavirus infection	Original		B.1	G	India	Undertermined	No (couldn't determine the patient status as either pre-symptomatic / asymptomatic or symptomatic conclusively)
hCoV-19/India/UP-NBRI-N38-TDT660/2020	EPI_ISL_516967	14/05/2020	Asia / India / Uttar Pradesh / Lucknow	Human		Female	86	Suspected coronavirus infection	Original		B.1.1	GR	India	Undertermined	No (couldn't determine the patient status as either pre-symptomatic / asymptomatic or symptomatic conclusively)
hCoV-19/India/UP-NBRI-N39-TDV163/2020	EPI_ISL_516968	15/05/2020	Asia / India / Uttar Pradesh / Kanpur	Human		Female	45	Suspected Corona	Original		B.6	O	India	Undertermined	No (couldn't determine the patient status as either pre-symptomatic / asymptomatic or symptomatic conclusively)
hCoV-19/India/UP-NBRI-N40-TCQ729/2020	EPI_ISL_516969	28/03/2020	Asia / India / Uttar Pradesh / Agra	Human		Male	54	Symptomatic	Original		B.1	GH	India	Symptomatic	Yes
hCoV-19/India/UP-NBRI-N41-TCX029/2020	EPI_ISL_516970	16/04/2020	Asia / India / Uttar Pradesh / Lucknow	Human		Male	36	Asymptomatic	Original		B.6	O	India	Pre-symptomatic / Asymptomatic	Yes
hCoV-19/India/UP-NBRI-N42-TCX099/2020	EPI_ISL_516971	16/04/2020	Asia / India / Uttar Pradesh / Lucknow	Human		Male	21	Asymptomatic	Original		B.6	O	India	Pre-symptomatic / Asymptomatic	Yes
hCoV-19/India/UP-NBRI-N43-TCX120/2020	EPI_ISL_516972	16/04/2020	Asia / India / Uttar Pradesh / Lucknow	Human		Male	51	Asymptomatic	Original		B.6	O	India	Pre-symptomatic / Asymptomatic	Yes
hCoV-19/India/UP-NBRI-N45-TCW721/2020	EPI_ISL_516973	15/04/2020	Asia / India / Uttar Pradesh / Agra	Human		Female	23	Suspected Corona	Original		B.1	G	India	Undertermined	No (couldn't determine the patient status as either pre-symptomatic / asymptomatic or symptomatic conclusively)
hCoV-19/India/UP-NBRI-N46-TCQ289/2020	EPI_ISL_516974	24/03/2020	Asia / India / Uttar Pradesh / Pilibhit	Human		Male	33	Asymptomatic	Original		B.1.1	GR	India	Pre-symptomatic / Asymptomatic	Yes
hCoV-19/India/UP-NBRI-N47-TCQ156/2020	EPI_ISL_516975	23/03/2020	Asia / India / Uttar Pradesh / Kanpur	Human		Male	70	Suspected Corona	Original		B.1	GH	India	Undertermined	No (couldn't determine the patient status as either pre-symptomatic / asymptomatic or symptomatic conclusively)
hCoV-19/India/UP-NBRI-N48-TEI094/2020	EPI_ISL_516976	25/05/2020	Asia / India / Uttar Pradesh / Sambhal	Human		Male	25	Suspected Corona	Original		B.1.113	GH	India	Undertermined	No (couldn't determine the patient status as either pre-symptomatic / asymptomatic or symptomatic conclusively)
hCoV-19/India/UP-NBRI-N49-TEI217/2020	EPI_ISL_516977	26/05/2020	Asia / India / Uttar Pradesh / Barabanki	Human		Male	24	Suspected Corona	Original		B.1.113	GH	India	Undertermined	No (couldn't determine the patient status as either pre-symptomatic / asymptomatic or symptomatic conclusively)
hCoV-19/India/UP-NBRI-N50-TEI229/2020	EPI_ISL_516978	26/05/2020	Asia / India / Uttar Pradesh / Barabanki	Human		Male	30	Suspected Corona	Original		B.6	O	India	Undertermined	No (couldn't determine the patient status as either pre-symptomatic / asymptomatic or symptomatic conclusively)
hCoV-19/India/UP-NBRI-N51-TEH465/2020	EPI_ISL_516979	25/05/2020	Asia / India / Uttar Pradesh / Hardoi	Human		Female	34	Suspected Corona	Original		B.1.113	GH	India	Undertermined	No (couldn't determine the patient status as either pre-symptomatic / asymptomatic or symptomatic conclusively)
hCoV-19/India/UP-NBRI-N52-TEH623/2020	EPI_ISL_516980	25/05/2020	Asia / India / Uttar Pradesh / Kannauj	Human		Male	44	Suspected Corona	Original		B.1.113	GH	India	Undertermined	No (couldn't determine the patient status as either pre-symptomatic / asymptomatic or symptomatic conclusively)
hCoV-19/India/UP-NBRI-N53-TEH628/2020	EPI_ISL_516981	25/05/2020	Asia / India / Uttar Pradesh / Kannauj	Human		Female	22	Suspected Corona	Original		B.1.113	GH	India	Undertermined	No (couldn't determine the patient status as either pre-symptomatic / asymptomatic or symptomatic conclusively)
hCoV-19/India/UP-NBRI-N54-TEH857/2020	EPI_ISL_516982	25/05/2020	Asia / India / Uttar Pradesh / Moradabad	Human		Female	8	Suspected Corona	Original		B.1.113	GH	India	Undertermined	No (couldn't determine the patient status as either pre-symptomatic / asymptomatic or symptomatic conclusively)
hCoV-19/India/UP-NBRI-N55-TEH861/2020	EPI_ISL_516983	25/05/2020	Asia / India / Uttar Pradesh / Moradabad	Human		Female	19	Suspected Corona	Original		B.1.113	GH	India	Undertermined	No (couldn't determine the patient status as either pre-symptomatic / asymptomatic or symptomatic conclusively)
hCoV-19/India/UP-NBRI-N56-TEH862/2020	EPI_ISL_516984	25/05/2020	Asia / India / Uttar Pradesh / Moradabad	Human		Female	45	Suspected Corona	Original		B.1.113	GH	India	Undertermined	No (couldn't determine the patient status as either pre-symptomatic / asymptomatic or symptomatic conclusively)
hCoV-19/India/UP-NBRI-N57-TEH921/2020	EPI_ISL_516985	25/05/2020	Asia / India / Uttar Pradesh / Moradabad	Human		Female	17	Suspected Corona	Original		B.1.1	GR	India	Undertermined	No (couldn't determine the patient status as either pre-symptomatic / asymptomatic or symptomatic conclusively)
hCoV-19/India/UP-NBRI-N58-TEG811/2020	EPI_ISL_516986	24/05/2020	Asia / India / Uttar Pradesh / Moradabad	Human		Female	2	Suspected Corona	Original		B.1.113	GH	India	Undertermined	No (couldn't determine the patient status as either pre-symptomatic / asymptomatic or symptomatic conclusively)
hCoV-19/Reunion/RUN-PIMIT1/2020	EPI_ISL_522547	24/03/2020	Africa / Reunion	Human	Patient infected while traveling in Europe	Female	48	Released	Original		B.1	G	Reunion	Undertermined	No (couldn't determine the patient status as either pre-symptomatic / asymptomatic or symptomatic conclusively)
hCoV-19/Reunion/RUN-PIMIT2/2020	EPI_ISL_522548	24/03/2020	Africa / Reunion	Human	Patient infected while traveling in Europe	Male	47	Hospitalized	Original		B.1	G	Reunion	Undertermined	No (couldn't determine the patient status as either pre-symptomatic / asymptomatic or symptomatic conclusively)
hCoV-19/Brazil/SP-128/2020	EPI_ISL_523955	02/04/2020	South America / Brazil / Sao Paulo / Sao Paulo	Human		Male	81	Deceased	Original	Naso-pharyngeal swab	B.1.1.28	GR	Brazil	Symptomatic	Yes
hCoV-19/Brazil/SP-132/2020	EPI_ISL_523956	31/03/2020	South America / Brazil / Sao Paulo / Assis	Human		Female	82	Deceased	Original	Naso-pharyngeal swab	B.1.1.33	GR	Brazil	Symptomatic	Yes
hCoV-19/Brazil/SP-133/2020	EPI_ISL_523957	04/04/2020	South America / Brazil / Sao Paulo / Sao Paulo	Human		Male	76	Deceased	Original	Naso-pharyngeal swab	B.1.1.28	GR	Brazil	Symptomatic	Yes
hCoV-19/Brazil/SP-162/2020	EPI_ISL_523958	15/04/2020	South America / Brazil / Sao Paulo / Sao Paulo	Human		Female	60	Deceased	Original	Naso-pharyngeal swab	B.1.1.28	GR	Brazil	Symptomatic	Yes
hCoV-19/Brazil/SP-163/2020	EPI_ISL_523959	15/08/2020	South America / Brazil / Sao Paulo / Sao Paulo	Human		Female	34	Deceased	Original	Naso-pharyngeal swab	B.1.1.33	GR	Brazil	Symptomatic	Yes
hCoV-19/Brazil/SP-165/2020	EPI_ISL_523963	24/03/2020	South America / Brazil / Sao Paulo / Sao Paulo	Human		Male	65	Deceased	Original	Naso-pharyngeal swab	B.1	GH	Brazil	Symptomatic	Yes
hCoV-19/Brazil/SP-166/2020	EPI_ISL_523965	19/03/2020	South America / Brazil / Sao Paulo / Sao Paulo	Human		Female	68	Deceased	Original	Naso-pharyngeal swab	B.1.1.28	GR	Brazil	Symptomatic	Yes
hCoV-19/Brazil/SP-168/2020	EPI_ISL_523967	18/04/2020	South America / Brazil / Sao Paulo / Sao Paulo	Human		Female	91	Deceased	Original	Naso-pharyngeal swab	B.1.1.28	GR	Brazil	Symptomatic	Yes
hCoV-19/Brazil/SP-170/2020	EPI_ISL_523969	31/03/2020	South America / Brazil / Sao Paulo / Sao Paulo	Human		Female	27	Deceased	Original	Naso-pharyngeal swab	B.1.1.28	GR	Brazil	Symptomatic	Yes
hCoV-19/Brazil/SP-171/2020	EPI_ISL_523970	02/04/2020	South America / Brazil / Sao Paulo / Sao Paulo	Human		Female	78	Deceased	Original	Naso-pharyngeal swab	B.1.1.28	GR	Brazil	Symptomatic	Yes
hCoV-19/Brazil/SP-174/2020	EPI_ISL_523971	25/03/2020	South America / Brazil / Sao Paulo / Sao Paulo	Human		Female	69	Deceased	Original	Naso-pharyngeal swab	B.1.1.28	GR	Brazil	Symptomatic	Yes
hCoV-19/Brazil/SP-176/2020	EPI_ISL_523972	11/04/2020	South America / Brazil / Sao Paulo / Sao Paulo	Human		Female	76	Deceased	Original	Naso-pharyngeal swab	B.1	G	Brazil	Symptomatic	Yes
hCoV-19/Brazil/SP-177/2020	EPI_ISL_523973	03/04/2020	South America / Brazil / Sao Paulo / Sao Paulo	Human		Female	30	Deceased	Original	Naso-pharyngeal swab	B.1.1.33	GR	Brazil	Symptomatic	Yes
hCoV-19/Brazil/SP-178/2020	EPI_ISL_523974	02/04/2020	South America / Brazil / Sao Paulo / Sao Paulo	Human		Male	63	Deceased	Original	Naso-pharyngeal swab	B.1.1.28	GR	Brazil	Symptomatic	Yes
hCoV-19/Brazil/SP-179/2020	EPI_ISL_523975	03/04/2020	South America / Brazil / Sao Paulo / Sao Paulo	Human		Male	33	Deceased	Original	Naso-pharyngeal swab	B.1.1.28	GR	Brazil	Symptomatic	Yes
hCoV-19/Brazil/SP-181/2020	EPI_ISL_523976	10/04/2020	South America / Brazil / Sao Paulo / Sao Paulo	Human		Male	64	Deceased	Original	Naso-pharyngeal swab	B.1.1.33	GR	Brazil	Symptomatic	Yes
hCoV-19/Brazil/SP-182/2020	EPI_ISL_523977	10/04/2020	South America / Brazil / Sao Paulo / Sao Paulo	Human		Male	40	Deceased	Original	Naso-pharyngeal swab	B.1.1.28	GR	Brazil	Symptomatic	Yes
hCoV-19/Brazil/SP-195/2020	EPI_ISL_523978	10/04/2020	South America / Brazil / Sao Paulo / Sao Paulo	Human		Male	77	Deceased	Original	Naso-pharyngeal swab	B.1.1.28	GR	Brazil	Symptomatic	Yes
hCoV-19/Brazil/SP-199/2020	EPI_ISL_523980	02/04/2020	South America / Brazil / Sao Paulo / Sao Paulo	Human		Male	45	Deceased	Original	Naso-pharyngeal swab	B.1.1.28	GR	Brazil	Symptomatic	Yes
hCoV-19/Brazil/SP-200/2020	EPI_ISL_523981	10/04/2020	South America / Brazil / Sao Paulo / Sao Paulo	Human		Male	83	Deceased	Original	Naso-pharyngeal swab	B.1.1.28	GR	Brazil	Symptomatic	Yes
hCoV-19/Brazil/SP-190/2020	EPI_ISL_523982	25/03/2020	South America / Brazil / Sao Paulo / Sao Paulo	Human		Female	72	Deceased	Original	Naso-pharyngeal swab	B.1.1.28	GR	Brazil	Symptomatic	Yes
hCoV-19/Brazil/SP-191/2020	EPI_ISL_523983	16/04/2020	South America / Brazil / Sao Paulo / Sao Paulo	Human		Female	61	Deceased	Original	Naso-pharyngeal swab	B.1.1.28	GR	Brazil	Symptomatic	Yes
hCoV-19/Brazil/SP-193/2020	EPI_ISL_523984	19/04/2020	South America / Brazil / Sao Paulo / Sao Paulo	Human		Male	83	Deceased	Original	Naso-pharyngeal swab	B.1.1.28	GR	Brazil	Symptomatic	Yes
hCoV-19/Brazil/SP-202/2020	EPI_ISL_523985	18/04/2020	South America / Brazil / Sao Paulo / Sao Paulo	Human		Male	90	Deceased	Original	Naso-pharyngeal swab	B.1.1.28	GR	Brazil	Symptomatic	Yes
hCoV-19/Brazil/SP-203/2020	EPI_ISL_523986	23/04/2020	South America / Brazil / Sao Paulo / Sao Paulo	Human		Male	80	Deceased	Original	Naso-pharyngeal swab	B.1.1.28	GR	Brazil	Symptomatic	Yes
hCoV-19/Brazil/SP-205/2020	EPI_ISL_523988	21/04/2020	South America / Brazil / Sao Paulo / Sao Paulo	Human		Male	57	Deceased	Original	Naso-pharyngeal swab	B.1.1.28	GR	Brazil	Symptomatic	Yes
hCoV-19/Brazil/SP-208/2020	EPI_ISL_523989	20/04/2020	South America / Brazil / Sao Paulo / Sao Paulo	Human		Male	67	Deceased	Original	Naso-pharyngeal swab	B.1.1.28	GR	Brazil	Symptomatic	Yes
hCoV-19/Brazil/SP-209/2020	EPI_ISL_523990	19/04/2020	South America / Brazil / Sao Paulo / Sao Paulo	Human		Female	64	Deceased	Original	Naso-pharyngeal swab	B.1.1.28	GR	Brazil	Symptomatic	Yes
hCoV-19/Brazil/SP-211/2020	EPI_ISL_523991	22/04/2020	South America / Brazil / Sao Paulo / Sao Paulo	Human		Female	55	Deceased	Original	Naso-pharyngeal swab	B.1.1.33	GR	Brazil	Symptomatic	Yes
hCoV-19/Brazil/SP-213/2020	EPI_ISL_523992	23/04/2020	South America / Brazil / Sao Paulo / Sao Paulo	Human		Male	47	Deceased	Original	Naso-pharyngeal swab	B.1.1.33	GR	Brazil	Symptomatic	Yes
hCoV-19/Brazil/SP-217/2020	EPI_ISL_523993	17/04/2020	South America / Brazil / Sao Paulo / Sao Paulo	Human		Male	72	Deceased	Original	Nasopharyngeal swab	B.1.1.28	GR	Brazil	Symptomatic	Yes
hCoV-19/Hungary/MH-597/2020	EPI_ISL_526215	10/04/2020	Europe / Hungary / Pest county	Human		Female	83	Hospitalized	Original	Oro-pharyngeal swab	B.1.1	GR	Hungary	Undertermined	No (couldn't determine the patient status as either pre-symptomatic / asymptomatic or symptomatic conclusively)
hCoV-19/Hungary/MH-642/2020	EPI_ISL_526216	14/04/2020	Europe / Hungary / Pest county	Human		Female	60	Hospitalized	Original	Oro-pharyngeal swab	B.1.1	GR	Hungary	Undertermined	No (couldn't determine the patient status as either pre-symptomatic / asymptomatic or symptomatic conclusively)

hCoV-19/Hungary/MH-732/2020	EPI_ISL_526217	15/04/2020	Europe / Hungary / Pest county	Human	Female	36	Hospitalized	Original	Oro-pharyngeal swab	B.1.1	GR	Hungary	Undertermined	No (couldn't determine the patient status as either pre-symptomatic / asymptomatic or symptomatic conclusively)
hCoV-19/Hungary/MH-1403/2020	EPI_ISL_526218	23/04/2020	Europe / Hungary / Pest county	Human	Female	74	Hospitalized	Original	Oro-pharyngeal swab	B.1.1	GR	Hungary	Undertermined	No (couldn't determine the patient status as either pre-symptomatic / asymptomatic or symptomatic conclusively)
hCoV-19/Hungary/MH-6074/2020	EPI_ISL_526219	30/04/2020	Europe / Hungary / Pest county	Human	Female	22	Hospitalized	Original	Oro-pharyngeal swab	B.1	G	Hungary	Undertermined	No (couldn't determine the patient status as either pre-symptomatic / asymptomatic or symptomatic conclusively)
hCoV-19/Hungary/MH-6633/2020	EPI_ISL_526220	11/05/2020	Europe / Hungary / Pest county	Human	Male	67	Hospitalized	Original	Oro-pharyngeal swab	B.1	G	Hungary	Undertermined	No (couldn't determine the patient status as either pre-symptomatic / asymptomatic or symptomatic conclusively)
hCoV-19/Hungary/MH-6657/2020	EPI_ISL_526221	11/05/2020	Europe / Hungary / Pest county	Human	Female	79	Hospitalized	Original	Oro-pharyngeal swab	B.1.5	G	Hungary	Undertermined	No (couldn't determine the patient status as either pre-symptomatic / asymptomatic or symptomatic conclusively)
hCoV-19/Hungary/MH-7051/2020	EPI_ISL_526222	14/05/2020	Europe / Hungary / Pest county	Human	Female	47	Hospitalized	Original	Oro-pharyngeal swab	B.1.1	GR	Hungary	Undertermined	No (couldn't determine the patient status as either pre-symptomatic / asymptomatic or symptomatic conclusively)
hCoV-19/Hungary/MH-8268/2020	EPI_ISL_526223	25/05/2020	Europe / Hungary / Pest county	Human	Male	77	Hospitalized	Original	Oro-pharyngeal swab	B.1	G	Hungary	Undertermined	No (couldn't determine the patient status as either pre-symptomatic / asymptomatic or symptomatic conclusively)
hCoV-19/Hungary/MH-9653/2020	EPI_ISL_526224	04/06/2020	Europe / Hungary / Pest county	Human	Female	25	Hospitalized	Original	Oro-pharyngeal swab	B.1	G	Hungary	Undertermined	No (couldn't determine the patient status as either pre-symptomatic / asymptomatic or symptomatic conclusively)
hCoV-19/Hungary/MH-13211/2020	EPI_ISL_526225	06/07/2020	Europe / Hungary / Pest county	Human	Male	48	Live	Original	Oro-pharyngeal swab	B.1	G	Hungary	Undertermined	No (couldn't determine the patient status as either pre-symptomatic / asymptomatic or symptomatic conclusively)
hCoV-19/Hungary/MH-1421/2020	EPI_ISL_526226	23/04/2020	Europe / Hungary / Pest county	Human	Male	42	Hospitalized	Original	Oro-pharyngeal swab	B.1.1	GR	Hungary	Undertermined	No (couldn't determine the patient status as either pre-symptomatic / asymptomatic or symptomatic conclusively)
hCoV-19/Hungary/MH-5846/2020	EPI_ISL_526227	28/04/2020	Europe / Hungary / Borsod-Abauj-Zemplen county	Human	Male	36	Hospitalized	Original	Oro-pharyngeal swab	B.1.1	GR	Hungary	Undertermined	No (couldn't determine the patient status as either pre-symptomatic / asymptomatic or symptomatic conclusively)
hCoV-19/Hungary/MH-5657/2020	EPI_ISL_526228	25/04/2020	Europe / Hungary / Pest county	Human	Female	65	Hospitalized	Original	Oro-pharyngeal swab	B.1.1	GR	Hungary	Undertermined	No (couldn't determine the patient status as either pre-symptomatic / asymptomatic or symptomatic conclusively)
hCoV-19/Hungary/MH-722/2020	EPI_ISL_526229	15/04/2020	Europe / Hungary / Pest county	Human	Male	74	Hospitalized	Original	Oro-pharyngeal swab	B.1.1	GR	Hungary	Undertermined	No (couldn't determine the patient status as either pre-symptomatic / asymptomatic or symptomatic conclusively)
hCoV-19/Hungary/MH-879/2020	EPI_ISL_526230	17/04/2020	Europe / Hungary / Pest county	Human	Female	82	Hospitalized	Original	Oro-pharyngeal swab	B.1.5	G	Hungary	Undertermined	No (couldn't determine the patient status as either pre-symptomatic / asymptomatic or symptomatic conclusively)
hCoV-19/Hungary/MH-975/2020	EPI_ISL_526231	20/04/2020	Europe / Hungary / Pest county	Human	Male	80	Hospitalized	Original	Oro-pharyngeal swab	B.1.1	GR	Hungary	Undertermined	No (couldn't determine the patient status as either pre-symptomatic / asymptomatic or symptomatic conclusively)
hCoV-19/Hungary/MH-789/2020	EPI_ISL_526232	16/04/2020	Europe / Hungary / Pest county	Human	Female	67	Hospitalized	Original	Oro-pharyngeal swab	B.1.5	G	Hungary	Undertermined	No (couldn't determine the patient status as either pre-symptomatic / asymptomatic or symptomatic conclusively)
hCoV-19/Hungary/MH-824/2020	EPI_ISL_526233	16/04/2020	Europe / Hungary / Pest county	Human	Female	53	Hospitalized	Original	Oro-pharyngeal swab	B.1.1	GR	Hungary	Undertermined	No (couldn't determine the patient status as either pre-symptomatic / asymptomatic or symptomatic conclusively)
hCoV-19/Hungary/MH-726/2020	EPI_ISL_526234	15/04/2020	Europe / Hungary / Pest county	Human	Male	87	Hospitalized	Original	Oro-pharyngeal swab	B.1.1	GR	Hungary	Undertermined	No (couldn't determine the patient status as either pre-symptomatic / asymptomatic or symptomatic conclusively)
hCoV-19/Hungary/MH-1101/2020	EPI_ISL_526235	21/04/2020	Europe / Hungary / Pest county	Human	Male	46	Hospitalized	Original	Oro-pharyngeal swab	B.1.1	GR	Hungary	Undertermined	No (couldn't determine the patient status as either pre-symptomatic / asymptomatic or symptomatic conclusively)
hCoV-19/Hungary/MH-6603/2020	EPI_ISL_526236	08/05/2020	Europe / Hungary / Pest county	Human	Female	76	Hospitalized	Original	Oro-pharyngeal swab	B.1	G	Hungary	Undertermined	No (couldn't determine the patient status as either pre-symptomatic / asymptomatic or symptomatic conclusively)
hCoV-19/Hungary/MH-5984/2020	EPI_ISL_526237	29/04/2020	Europe / Hungary / Pest county	Human	Male	85	Hospitalized	Original	Oro-pharyngeal swab	B.1.1	GR	Hungary	Undertermined	No (couldn't determine the patient status as either pre-symptomatic / asymptomatic or symptomatic conclusively)
hCoV-19/Hungary/MH-1106/2020	EPI_ISL_526238	21/04/2020	Europe / Hungary / Pest county	Human	Female	81	Hospitalized	Original	Oro-pharyngeal swab	B.1.1	GR	Hungary	Undertermined	No (couldn't determine the patient status as either pre-symptomatic / asymptomatic or symptomatic conclusively)
hCoV-19/United Arab Emirates/434/2020	EPI_ISL_528700	13/06/2020	Asia / United Arab Emirates / Abu Dhabi	Human	Male	61	Live	Original		B.1.1	GR	United Arab Emirates	Undertermined	No (couldn't determine the patient status as either pre-symptomatic / asymptomatic or symptomatic conclusively)
hCoV-19/United Arab Emirates/44/2020	EPI_ISL_528701	11/05/2020	Asia / United Arab Emirates / Abu Dhabi	Human	Male	48	Hospitalized	Original		B.1.1	GR	United Arab Emirates	Undertermined	No (couldn't determine the patient status as either pre-symptomatic / asymptomatic or symptomatic conclusively)
hCoV-19/United Arab Emirates/484/2020	EPI_ISL_528702	15/06/2020	Asia / United Arab Emirates / Dubai	Human	Female	33	Live	Original		B.1.1	GR	United Arab Emirates	Undertermined	No (couldn't determine the patient status as either pre-symptomatic / asymptomatic or symptomatic conclusively)
hCoV-19/United Arab Emirates/529/2020	EPI_ISL_528703	26/06/2020	Asia / United Arab Emirates / Sharja	Human	Male	39	Live	Original		B.1.1	GR	United Arab Emirates	Undertermined	No (couldn't determine the patient status as either pre-symptomatic / asymptomatic or symptomatic conclusively)
hCoV-19/United Arab Emirates/54/2020	EPI_ISL_528704	14/05/2020	Asia / United Arab Emirates / Abu Dhabi	Human	Male	31	Hospitalized	Original		B.1.1	GR	United Arab Emirates	Undertermined	No (couldn't determine the patient status as either pre-symptomatic / asymptomatic or symptomatic conclusively)
hCoV-19/United Arab Emirates/558/2020	EPI_ISL_528705	30/06/2020	Asia / United Arab Emirates / Abu Dhabi	Human	Female	44	Live	Original		B.1	G	United Arab Emirates	Undertermined	No (couldn't determine the patient status as either pre-symptomatic / asymptomatic or symptomatic conclusively)
hCoV-19/United Arab Emirates/56/2020	EPI_ISL_528706	14/05/2020	Asia / United Arab Emirates / Abu Dhabi	Human	Female	26	Hospitalized	Original		B.1.1	GR	United Arab Emirates	Undertermined	No (couldn't determine the patient status as either pre-symptomatic / asymptomatic or symptomatic conclusively)
hCoV-19/United Arab Emirates/581/2020	EPI_ISL_528707	01/07/2020	Asia / United Arab Emirates / Abu Dhabi	Human	Female	24	Live	Original		B.1	G	United Arab Emirates	Undertermined	No (couldn't determine the patient status as either pre-symptomatic / asymptomatic or symptomatic conclusively)
hCoV-19/United Arab Emirates/C2-109/2020	EPI_ISL_528708	03/06/2020	Asia / United Arab Emirates / Abu Dhabi	Human	Male	65	Hospitalized	Original		A	S	United Arab Emirates	Undertermined	No (couldn't determine the patient status as either pre-symptomatic / asymptomatic or symptomatic conclusively)
hCoV-19/United Arab Emirates/H1/2020	EPI_ISL_528709	30/04/2020	Asia / United Arab Emirates / Abu Dhabi	Human	Female	29	Hospitalized	Original		B.1.1	GR	United Arab Emirates	Undertermined	No (couldn't determine the patient status as either pre-symptomatic / asymptomatic or symptomatic conclusively)
hCoV-19/United Arab Emirates/H10/2020	EPI_ISL_528710	25/04/2020	Asia / United Arab Emirates / Abu Dhabi	Human	Male	55	Hospitalized	Original		B.1.1	GR	United Arab Emirates	Undertermined	No (couldn't determine the patient status as either pre-symptomatic / asymptomatic or symptomatic conclusively)
hCoV-19/United Arab Emirates/H12/2020	EPI_ISL_528711	26/04/2020	Asia / United Arab Emirates / Abu Dhabi	Human	Male	30	Hospitalized	Original		A	S	United Arab Emirates	Undertermined	No (couldn't determine the patient status as either pre-symptomatic / asymptomatic or symptomatic conclusively)
hCoV-19/United Arab Emirates/H13/2020	EPI_ISL_528712	26/04/2020	Asia / United Arab Emirates / Abu Dhabi	Human	Male	28	Hospitalized	Original		B.1.1	GR	United Arab Emirates	Undertermined	No (couldn't determine the patient status as either pre-symptomatic / asymptomatic or symptomatic conclusively)
hCoV-19/United Arab Emirates/H18/2020	EPI_ISL_528713	24/04/2020	Asia / United Arab Emirates / Abu Dhabi	Human	Male	40	Hospitalized	Original		B.1.1	GR	United Arab Emirates	Undertermined	No (couldn't determine the patient status as either pre-symptomatic / asymptomatic or symptomatic conclusively)
hCoV-19/United Arab Emirates/H2/2020	EPI_ISL_528714	26/04/2020	Asia / United Arab Emirates / Abu Dhabi	Human	Male	24	Hospitalized	Original		B.1	G	United Arab Emirates	Undertermined	No (couldn't determine the patient status as either pre-symptomatic / asymptomatic or symptomatic conclusively)
hCoV-19/United Arab Emirates/H20/2020	EPI_ISL_528715	26/04/2020	Asia / United Arab Emirates / Abu Dhabi	Human	Male	3	Hospitalized	Original		B.1	G	United Arab Emirates	Undertermined	No (couldn't determine the patient status as either pre-symptomatic / asymptomatic or symptomatic conclusively)
hCoV-19/United Arab Emirates/H21/2020	EPI_ISL_528716	25/04/2020	Asia / United Arab Emirates / Abu Dhabi	Human	Male	46	Hospitalized	Original		B.1.1	GR	United Arab Emirates	Undertermined	No (couldn't determine the patient status as either pre-symptomatic / asymptomatic or symptomatic conclusively)
hCoV-19/United Arab Emirates/H23/2020	EPI_ISL_528717	26/04/2020	Asia / United Arab Emirates / Abu Dhabi	Human	Male	31	Hospitalized	Original		B.1.1	GR	United Arab Emirates	Undertermined	No (couldn't determine the patient status as either pre-symptomatic / asymptomatic or symptomatic conclusively)
hCoV-19/United Arab Emirates/H27/2020	EPI_ISL_528718	30/04/2020	Asia / United Arab Emirates / Abu Dhabi	Human	Female	25	Hospitalized	Original		B.1.1	GR	United Arab Emirates	Undertermined	No (couldn't determine the patient status as either pre-symptomatic / asymptomatic or symptomatic conclusively)
hCoV-19/United Arab Emirates/H5/2020	EPI_ISL_528719	25/04/2020	Asia / United Arab Emirates / Abu Dhabi	Human	Male	42	Hospitalized	Original		B.1.5	G	United Arab Emirates	Undertermined	No (couldn't determine the patient status as either pre-symptomatic / asymptomatic or symptomatic conclusively)
hCoV-19/United Arab Emirates/H8/2020	EPI_ISL_528720	24/04/2020	Asia / United Arab Emirates / Abu Dhabi	Human	Male	50	Hospitalized	Original		B.1	G	United Arab Emirates	Undertermined	No (couldn't determine the patient status as either pre-symptomatic / asymptomatic or symptomatic conclusively)
hCoV-19/United Arab Emirates/H9/2020	EPI_ISL_528721	24/04/2020	Asia / United Arab Emirates / Abu Dhabi	Human	Male	33	Hospitalized	Original		B.1.1	GR	United Arab Emirates	Undertermined	No (couldn't determine the patient status as either pre-symptomatic / asymptomatic or symptomatic conclusively)
hCoV-19/India/AP-CCMB-GC41/2020	EPI_ISL_528809	29/05/2020	Asia / India / Andhra Pradesh / Kurnool	Human	Male	54	ICU	Original	Nasopharyngeal swab/Oropharyngeal swab	B.1.1.8	GR	India	Symptomatic	Yes
hCoV-19/India/TG-CCMB-GC1/2020	EPI_ISL_528810	24/05/2020	Asia / India / Telangana / Hyderabad	Human	Male	25	Ward	Original	Nasopharyngeal swab/Oropharyngeal swab	B.1	G	India	Undertermined	No (couldn't determine the patient status as either pre-symptomatic / asymptomatic or symptomatic conclusively)
hCoV-19/India/TG-CCMB-GC16/2020	EPI_ISL_528811	19/05/2020	Asia / India / Telangana / Hyderabad	Human	Male	25	Ward	Original	Nasopharyngeal swab/Oropharyngeal swab	B.1.80	G	India	Undertermined	No (couldn't determine the patient status as either pre-symptomatic / asymptomatic or symptomatic conclusively)
hCoV-19/India/TG-CCMB-GC2/2020	EPI_ISL_528812	24/05/2020	Asia / India / Telangana / Hyderabad	Human	Male	43	Ward	Original	Nasopharyngeal swab/Oropharyngeal swab	B.1.1	GR	India	Undertermined	No (couldn't determine the patient status as either pre-symptomatic / asymptomatic or symptomatic conclusively)
hCoV-19/India/TG-CCMB-GC20/2020	EPI_ISL_528813	19/07/2020	Asia / India / Telangana / Hyderabad	Human	Female	21	Ward	Original	Nasopharyngeal swab/Oropharyngeal swab	B.1.1	GR	India	Undertermined	No (couldn't determine the patient status as either pre-symptomatic / asymptomatic or symptomatic conclusively)
hCoV-19/India/TG-CCMB-GC22/2020	EPI_ISL_528814	22/05/2020	Asia / India / Telangana / Hyderabad	Human	Male	42	ICU	Original	Nasopharyngeal swab/Oropharyngeal swab	B.1.1	GR	India	Symptomatic	Yes
hCoV-19/India/TG-CCMB-GC28/2020	EPI_ISL_528815	30/05/2020	Asia / India / Telangana / Hyderabad	Human	Male	34	Ward	Original	Nasopharyngeal swab/Oropharyngeal swab	B.1.1.8	GR	India	Undertermined	No (couldn't determine the patient status as either pre-symptomatic / asymptomatic or symptomatic conclusively)
hCoV-19/India/TG-CCMB-GC3/2020	EPI_ISL_528816	23/05/2020	Asia / India / Telangana / Rangaraddy	Human	Male	23	Ward	Original	Nasopharyngeal swab/Oropharyngeal swab	B.1.1.8	GR	India	Undertermined	No (couldn't determine the patient status as either pre-symptomatic / asymptomatic or symptomatic conclusively)
hCoV-19/India/TG-CCMB-GC39/2020	EPI_ISL_528817	27/05/2020	Asia / India / Telangana / Hyderabad	Human	Male	60	Ward	Original	Nasopharyngeal swab/Oropharyngeal swab	B.1.1	GR	India	Undertermined	No (couldn't determine the patient status as either pre-symptomatic / asymptomatic or symptomatic conclusively)
hCoV-19/India/TG-CCMB-GC4/2020	EPI_ISL_528818	17/05/2020	Asia / India / Telangana / Jagtial	Human	Male	60	Ward	Original	Nasopharyngeal swab/Oropharyngeal swab	B.1.1	GR	India	Undertermined	No (couldn't determine the patient status as either pre-symptomatic / asymptomatic or symptomatic conclusively)
hCoV-19/India/TG-CCMB-GC42/2020	EPI_ISL_528819	30/05/2020	Asia / India / Telangana / Hyderabad	Human	Male	31	Ward	Original	Nasopharyngeal swab/Oropharyngeal swab	B.1.1.8	GR	India	Undertermined	No (couldn't determine the patient status as either pre-symptomatic / asymptomatic or symptomatic conclusively)
hCoV-19/India/TG-CCMB-GC43/2020	EPI_ISL_528820	30/05/2020	Asia / India / Telangana / Hyderabad	Human	Male	30	Ward	Original	Nasopharyngeal swab/Oropharyngeal swab	B.1.1.8	GR	India	Undertermined	No (couldn't determine the patient status as either pre-symptomatic / asymptomatic or symptomatic conclusively)
hCoV-19/India/TG-CCMB-GC8/2020	EPI_ISL_528821	23/05/2020	Asia / India / Telangana / Warangal	Human	Male	20	Ward	Original	Nasopharyngeal swab/Oropharyngeal swab	B.1.80	G	India	Undertermined	No (couldn't determine the patient status as either pre-symptomatic / asymptomatic or symptomatic conclusively)
hCoV-19/India/TG-CCMB-GC9/2020	EPI_ISL_528822	23/05/2020	Asia / India / Telangana / Hyderabad	Human	Male	39	Ward	Original	Nasopharyngeal swab/Oropharyngeal swab	B.1.1.32	GR	India	Undertermined	No (couldn't determine the patient status as either pre-symptomatic / asymptomatic or symptomatic conclusively)
hCoV-19/Italy/ABR-IZSGC-TE4884/2020	EPI_ISL_528919	17/03/2020	Europe / Italy / Abruzzo	Human	Male	89	Live	Original		B.1.1	GR	Italy	Undertermined	No (couldn't determine the patient status as either pre-symptomatic / asymptomatic or symptomatic conclusively)
hCoV-19/Italy/ABR-IZSGC-TE4891/2020	EPI_ISL_528920	17/03/2020	Europe / Italy / Abruzzo	Human	Male	43	Live	Original		B.1.1	GR	Italy	Undertermined	No (couldn't determine the patient status as either pre-symptomatic / asymptomatic or symptomatic conclusively)
hCoV-19/Italy/ABR-IZSGC-TE4926/2020	EPI_ISL_528921	17/03/2020	Europe / Italy / Abruzzo	Human	Female	30	Live	Original		B.1	G	Italy	Undertermined	No (couldn't determine the patient status as either pre-symptomatic / asymptomatic or symptomatic conclusively)
hCoV-19/Italy/ABR-IZSGC-TE5040/2020	EPI_ISL_528922	17/03/2020	Europe / Italy / Abruzzo	Human	Female	72	Live	Original		B.1.1	GR	Italy	Undertermined	No (couldn't determine the patient status as either pre-symptomatic / asymptomatic or symptomatic conclusively)
hCoV-19/Italy/ABR-IZSGC-TE5148/2020	EPI_ISL_528923	18/03/2020	Europe / Italy / Abruzzo	Human	Male	62	Live	Original		B.1.1	GR	Italy	Undertermined	No (couldn't determine the patient status as either pre-symptomatic / asymptomatic or symptomatic conclusively)
hCoV-19/Italy/ABR-IZSGC-TE5173/2020	EPI_ISL_528924	18/03/2020	Europe / Italy / Abruzzo	Human	Male	45	Live	Original		B.1	G	Italy	Undertermined	No (couldn't determine the patient status as either pre-symptomatic / asymptomatic or symptomatic conclusively)
hCoV-19/Italy/ABR-IZSGC-TE5268/2020	EPI_ISL_528925	18/03/2020	Europe / Italy / Abruzzo	Human	Male	35	Live	Original		B.1.1	GR	Italy	Undertermined	No (couldn't determine the patient status as either pre-symptomatic / asymptomatic or symptomatic conclusively)
hCoV-19/Italy/ABR-IZSGC-TE5487/2020	EPI_ISL_528926	19/03/2020	Europe / Italy / Abruzzo	Human	Female	49	Live	Original		B.1.1	GR	Italy	Undertermined	No (couldn't determine the patient status as either pre-symptomatic / asymptomatic or symptomatic conclusively)
hCoV-19/Italy/ABR-IZSGC-TE6202/2020	EPI_ISL_528927	23/03/2020	Europe / Italy / Abruzzo	Human	Male	83	Live	Original		B.1.1	GR	Italy	Undertermined	No (couldn't determine the patient status as either pre-symptomatic / asymptomatic or symptomatic conclusively)
hCoV-19/Italy/ABR-IZSGC-TE6218/2020	EPI_ISL_528928	23/03/2020	Europe / Italy / Abruzzo	Human	Female	38	Live	Original		B.1.1	GR	Italy	Undertermined	No (couldn't determine the patient status as either pre-symptomatic / asymptomatic or symptomatic conclusively)
hCoV-19/Italy/ABR-IZSGC-TE7097/2020	EPI_ISL_528929	26/03/2020	Europe / Italy / Abruzzo	Human	Male	67	Live	Original		B.1	G	Italy	Undertermined	No (couldn't determine the patient status as either pre-symptomatic / asymptomatic or symptomatic conclusively)
hCoV-19/Italy/LOM-IZSGC-TE30933/2020	EPI_ISL_528934	02/05/2020	Europe / Italy / Lombardia	Human	Female	45	Live	Original		B.1.1	GR	Italy	Undertermined	No (couldn't determine the patient status as either pre-symptomatic / asymptomatic or symptomatic conclusively)
hCoV-19/Italy/LOM-IZSGC-TE31054/2020	EPI_ISL_528935	02/05/2020	Europe / Italy / Lombardia	Human	Female	58	Live	Original		B.1.1	GR	Italy	Undertermined	No (couldn't determine the patient status as either pre-symptomatic / asymptomatic or symptomatic conclusively)
hCoV-19/Italy/LOM-IZSGC-TE31074/2020	EPI_ISL_528936	02/05/2020	Europe / Italy / Lombardia	Human	Female	91	Live	Original		B.1.1	GR	Italy	Undertermined	No (couldn't determine the patient status as either pre-symptomatic / asymptomatic or symptomatic conclusively)
hCoV-19/Italy/LOM-IZSGC-TE31135/2020	EPI_ISL_528937	02/05/2020	Europe / Italy / Lombardia	Human	Female	86	Live	Original		B.1.1	GR	Italy	Undertermined	No (couldn't determine the patient status as either pre-symptomatic / asymptomatic or symptomatic conclusively)
hCoV-19/Italy/LOM-IZSGC-TE31751/2020	EPI_ISL_528938	02/05/2020	Europe / Italy / Lombardia	Human	Female	60	Live	Original		B.1.1	GR	Italy	Undertermined	No (couldn't determine the patient status as either pre-symptomatic / asymptomatic or symptomatic conclusively)
hCoV-19/Italy/LOM-IZSGC-TE31811/2020	EPI_ISL_528939	02/05/2020	Europe / Italy / Lombardia	Human	Female	63	Live	Original		B.1.1	GR	Italy	Undertermined	No (couldn't determine the patient status as either pre-symptomatic / asymptomatic or symptomatic conclusively)
hCoV-19/Italy/LOM-IZSGC-TE31818/2020	EPI_ISL_528940	02/05/2020	Europe / Italy / Lombardia	Human	Male	48	Live	Original		B.1.1	GR	Italy	Undertermined	No (couldn't determine the patient status as either pre-symptomatic / asymptomatic or symptomatic conclusively)
hCoV-19/Italy/LOM-IZSGC-TE35432/2020	EPI_ISL_528941	08/05/2020	Europe / Italy / Lombardia	Human	Female	59	Live	Original		B.1.1	GR	Italy	Undertermined	No (couldn't determine the patient status as either pre-symptomatic / asymptomatic or symptomatic conclusively)
hCoV-19/Italy/LOM-IZSGC-TE35441/2020	EPI_ISL_528942	08/05/2020	Europe / Italy / Lombardia	Human	Female	38	Live	Original		B.1.1	GR	Italy	Undertermined	No (couldn't determine the patient status as either pre-symptomatic / asymptomatic or symptomatic conclusively)
hCoV-19/Italy/LOM-IZSGC-TE35526/2020	EPI_ISL_528943	08/05/2020	Europe / Italy / Lombardia	Human	Female	90	Live	Original		B.1.1	GR	Italy	Undertermined	No (couldn't determine the patient status as either pre-symptomatic / asymptomatic or symptomatic conclusively)
hCoV-19/Italy/LOM-IZSGC-TE35532/2020	EPI_ISL_528944	08/05/2020	Europe / Italy / Lombardia	Human	Female	55	Live	Original		B.1.1	GR	Italy	Undertermined	No (couldn't determine the patient status as either pre-symptomatic / asymptomatic or symptomatic conclusively)
hCoV-19/Italy/LOM-IZSGC-TE35566/2020	EPI_ISL_528945	08/05/2020	Europe / Italy / Lombardia	Human	Female	78	Live	Original		B.1.1	GR	Italy	Undertermined	No (couldn't determine the patient status as either pre-symptomatic / asymptomatic or symptomatic conclusively)
hCoV-19/Italy/LOM-IZSGC-TE35578/2020	EPI_ISL_528946	08/05/2020	Europe / Italy / Lombardia	Human	Female	94	Live	Original		B.1.1	GR	Italy	Undertermined	No (couldn't determine the patient status as either pre-symptomatic / asymptomatic or symptomatic conclusively)
hCoV-19/Italy/LOM-IZSGC-TE38811/2020	EPI_ISL_528947	10/05/2020	Europe / Italy / Lombardia	Human	Female	82	Live	Original		B.1.1	O	Italy	Undertermined	No (couldn't determine the patient status as either pre-symptomatic / asymptomatic or symptomatic conclusively)
hCoV-19/Italy/LOM-IZSGC-TE38819/2020	EPI_ISL_528948	10/05/2020	Europe / Italy / Lombardia	Human	Female	92	Live	Original		B.1.1	GR	Italy	Undertermined	No (couldn't determine the patient status as either pre-symptomatic / asymptomatic or symptomatic conclusively)
hCoV-19/Italy/LOM-IZSGC-TE38845/2020	EPI_ISL_528949	10/05/2020	Europe / Italy / Lombardia	Human	Female	100	Live	Original		B.1.1	GR	Italy	Undertermined	No (couldn't determine the patient status as either pre-symptomatic / asymptomatic or symptomatic conclusively)
hCoV-19/Italy/ABR-IZSGC-TE10002/2020	EPI_ISL_528990	01/04/2020	Europe / Italy / Abruzzo	Human	Male	90	Live	Original		B.1.1	GR	Italy	Undertermined	No (couldn't determine the patient status as either pre-symptomatic / asymptomatic or symptomatic conclusively)
hCoV-19/Italy/ABR-IZSGC-TE10007/2020	EPI_ISL_528991	02/04/2020	Europe / Italy / Abruzzo	Human	Male	52	Live	Original		B.1.1	GR	Italy	Undertermined	No (couldn't determine the patient status as either pre-symptomatic / asymptomatic or symptomatic conclusively)
hCoV-19/Italy/ABR-IZSGC-TE32707/2020	EPI_ISL_528992	29/04/2020	Europe / Italy / Abruzzo	Human	Male	62	Live	Original		B.1	G	Italy	Undertermined	No (couldn't determine the patient status as either pre-symptomatic / asymptomatic or symptomatic conclusively)
hCoV-19/Italy/ABR-IZSGC-TE34742/2020	EPI_ISL_528993	04/05/2020	Europe / Italy / Abruzzo	Human	Female	81	Live	Original		B.1.1	GR	Italy	Undertermined	No (couldn't determine the patient status as either pre-symptomatic / asymptomatic or symptomatic conclusively)
hCoV-19/Italy/ABR-IZSGC-TE46129/2020	EPI_ISL_528994	13/05/2020	Europe / Italy / Abruzzo	Human	Female	61	Live	Original		B.1	G	Italy	Undertermined	No (couldn't determine the patient status as either pre-symptomatic / asymptomatic or symptomatic conclusively)
hCoV-19/Italy/ABR-IZSGC-TE46424/2020	EPI_ISL_528995	14/05/2020	Europe / Italy / Abruzzo	Human	Female	40	Live	Original		B.1	G	Italy	Undertermined	No (couldn't determine the patient status as either pre-symptomatic / asymptomatic or symptomatic conclusively)
hCoV-19/Italy/ABR-IZSGC-TE46428/2020	EPI_ISL_528996	14/05/2020	Europe / Italy / Abruzzo	Human	Male	4	Live	Original		B.1	G	Italy	Undertermined	No (couldn't determine the patient status as either pre-symptomatic / asymptomatic or symptomatic conclusively)
hCoV-19/Italy/ABR-IZSGC-TE46429/2020	EPI_ISL_528997	14/05/2020	Europe / Italy / Abruzzo	Human	Female	2	Live	Original		B.1	G	Italy	Undertermined	No (couldn't determine the patient status as either pre-symptomatic / asymptomatic or symptomatic conclusively)
hCoV-19/Italy/ABR-IZSGC-TE46433/2020	EPI_ISL_528998	14/05/2020	Europe / Italy / Abruzzo	Human	Female	8	Live	Original		B.1	GH	Italy	Undertermined	No (couldn't determine the patient status as either pre-symptomatic / asymptomatic or symptomatic conclusively)
hCoV-19/Italy/ABR-IZSGC-TE46435/2020	EPI_ISL_528999	14/05/2020	Europe / Italy / Abruzzo	Human	Female	27	Live	Original		B.1	G	Italy	Undertermined	No (couldn't determine the patient status as either pre-symptomatic / asymptomatic or symptomatic conclusively)
hCoV-19/Spain/AR-ISCIII-201435/2020	EPI_ISL_539500	03/03/2020	Europe / Spain / Aragon	Human	Male	79	Hospitalized	Original	Oropharyngeal swab	B	O	Spain	Undertermined	No (couldn't determine the patient status as either pre-symptomatic / asymptomatic or symptomatic conclusively)
hCoV-19/Spain/AR-ISCIII-201587/2020	EPI_ISL_539501	05/03/2020	Europe / Spain / Aragon	Human	Male	82	Hospitalized	Original	Oropharyngeal swab	B.2	V	Spain	Undertermined	No (couldn't determine the patient status as either pre-symptomatic / asymptomatic or symptomatic conclusively)
hCoV-19/Spain/AR-ISCIII-201588/2020	EPI_ISL_539502	05/03/2020	Europe / Spain / Aragon	Human	Male	61	Hospitalized	Original	Oropharyngeal swab	B.2	O	Spain	Undertermined	No (couldn't determine the patient status as either pre-symptomatic / asymptomatic or symptomatic conclusively)
hCoV-19/Spain/AR-ISCIII-201596/2020	EPI_ISL_539503	06/03/2020	Europe / Spain / Aragon	Human	Male	53	Hospitalized	Original	Oropharyngeal swab	B.2	V	Spain	Undertermined	No (couldn't determine the patient status as either pre-symptomatic / asymptomatic or symptomatic conclusively)
hCoV-19/Spain/AR-ISCIII-201597/2020	EPI_ISL_539504	06/03/2020	Europe / Spain / Aragon	Human	Female	53	Hospitalized	Original	Oropharyngeal swab	B.2	V	Spain	Undertermined	No (couldn't determine the patient status as either pre-symptomatic / asymptomatic or symptomatic conclusively)
hCoV-19/Spain/AR-ISCIII-201664/2020	EPI_ISL_539505	04/03/2020	Europe / Spain / Aragon	Human	Male	83	Hospitalized	Original	Oropharyngeal swab	B.2	V	Spain	Undertermined	No (couldn't determine the patient status as either pre-symptomatic / asymptomatic or symptomatic conclusively)
hCoV-19/Spain/AR-ISCIII-201665/2020	EPI_ISL_539506	07/03/2020	Europe / Spain / Aragon	Human	Male	64	Hospitalized	Original	Oropharyngeal swab	B.2	V	Spain	Undertermined	No (couldn't determine the patient status as either pre-symptomatic / asymptomatic or symptomatic conclusively)
hCoV-19/Spain/AR-ISCIII-201672/2020	EPI_ISL_539507	07/03/2020	Europe / Spain / Aragon	Human	Female	94	Hospitalized	Original	Oropharyngeal swab	B	O	Spain	Undertermined	No (couldn't determine the patient status as either pre-symptomatic / asymptomatic or symptomatic conclusively)
hCoV-19/Spain/AR-ISCIII-201673/2020	EPI_ISL_539508	07/03/2020	Europe / Spain / Aragon	Human	Female	90	Hospitalized	Original	Oropharyngeal swab	B.2	V	Spain	Undertermined	No (couldn't determine the patient status as either pre-symptomatic / asymptomatic or symptomatic conclusively)
hCoV-19/Spain/AR-ISCIII-201687/2020	EPI_ISL_539509	06/03/2020	Europe / Spain / Aragon	Human	Female	96	Hospitalized	Original	Oropharyngeal swab	B.2	V	Spain	Undertermined	No (couldn't determine the patient status as either pre-symptomatic / asymptomatic or symptomatic conclusively)
hCoV-19/Spain/AR-ISCIII-201688/2020	EPI_ISL_539510	06/03/2020	Europe / Spain / Aragon	Human	Female	69	Hospitalized	Original	Oropharyngeal swab	B.2	V	Spain	Undertermined	No (couldn't determine the patient status as either pre-symptomatic / asymptomatic or symptomatic conclusively)
hCoV-19/Spain/AR-ISCIII-201692/2020	EPI_ISL_539511	07/03/2020	Europe / Spain / Aragon	Human	Male	66	Hospitalized	Original	Oropharyngeal swab	B.2	V	Spain	Undertermined	No (couldn't determine the patient status as either pre-symptomatic / asymptomatic or symptomatic conclusively)
hCoV-19/Spain/AR-ISCIII-201693/2020	EPI_ISL_539512	07/03/2020	Europe / Spain / Aragon	Human	Female	46	Hospitalized	Original	Oropharyngeal swab	B.2	V	Spain	Undertermined	No (couldn't determine the patient status as either pre-symptomatic / asymptomatic or symptomatic conclusively)
hCoV-19/Spain/AR-ISCIII-201779/2020	EPI_ISL_539513	07/03/2020	Europe / Spain / Aragon	Human	Male	61	Hospitalized	Original	Oropharyngeal swab	B.2	V	Spain	Undertermined	No (couldn't determine the patient status as either pre-symptomatic / asymptomatic or symptomatic conclusively)
hCoV-19/Spain/AR-ISCIII-201780/2020	EPI_ISL_539514	07/03/2020	Europe / Spain / Aragon	Human	Female	54	Hospitalized	Original	Oropharyngeal swab	B.2	V	Spain	Undertermined	No (couldn't determine the patient status as either pre-symptomatic / asymptomatic or symptomatic conclusively)
hCoV-19/Spain/AR-ISCIII-201782/2020	EPI_ISL_539515	08/03/2020	Europe / Spain / Aragon	Human	Female	83	Hospitalized	Original	Oropharyngeal swab	B.2	V	Spain	Undertermined	No (couldn't determine the patient status as either pre-symptomatic / asymptomatic or symptomatic conclusively)
hCoV-19/Spain/AR-ISCIII-201784/2020	EPI_ISL_539516	08/03/2020	Europe / Spain / Aragon	Human	Female	96	Hospitalized	Original	Oropharyngeal swab	B.2	V	Spain	Undertermined	No (couldn't determine the patient status as either pre-symptomatic / asymptomatic or symptomatic conclusively)
hCoV-19/Spain/AR-ISCIII-201848/2020	EPI_ISL_539517	08/03/2020	Europe / Spain / Aragon	Human	Male	80	Hospitalized	Original	Oropharyngeal swab	B	O	Spain	Undertermined	No (couldn't determine the patient status as either pre-symptomatic / asymptomatic or symptomatic conclusively)
hCoV-19/Spain/AR-ISCIII-201849/2020	EPI_ISL_539518	09/03/2020	Europe / Spain / Aragon	Human	Male	88	Hospitalized	Original	Oropharyngeal swab	B.2	V	Spain	Undertermined	No (couldn't determine the patient status as either pre-symptomatic / asymptomatic or symptomatic conclusively)
hCoV-19/Spain/AR-ISCIII-201850/2020	EPI_ISL_539519	09/03/2020	Europe / Spain / Aragon	Human	Male	85	Hospitalized	Original	Oropharyngeal swab	B.2	V	Spain	Undertermined	No (couldn't determine the patient status as either pre-symptomatic / asymptomatic or symptomatic conclusively)
hCoV-19/Spain/CE-ISCIII-202293/2020	EPI_ISL_539520	12/03/2020	Europe / Spain / Ceuta	Human	Male	25	Hospitalized	Original	Oropharyngeal swab	B.2	V	Spain	Undertermined	No (couldn't determine the patient status as either pre-symptomatic / asymptomatic or symptomatic conclusively)
hCoV-19/Spain/CE-ISCIII-203352/2020	EPI_ISL_539521	16/03/2020	Europe / Spain / Ceuta	Human	Female	59	Hospitalized	Original	Oropharyngeal swab	B.2	V	Spain	Undertermined	No (couldn't determine the patient status as either pre-symptomatic / asymptomatic or symptomatic conclusively)
hCoV-19/Spain/CE-ISCIII-204881/2020	EPI_ISL_539522	18/03/2020	Europe / Spain / Ceuta	Human	Male	27	Hospitalized	Original	Oropharyngeal swab	B.1	GH	Spain	Undertermined	No (couldn't determine the patient status as either pre-symptomatic / asymptomatic or symptomatic conclusively)
hCoV-19/Spain/CL-ISCIII-202362/2020	EPI_ISL_539523	13/03/2020	Europe / Spain / Castilla y Leon	Human	Male	2 months	Hospitalized	Original	Oropharyngeal swab	B.1.5	G	Spain	Undertermined	No (couldn't determine the patient status as either pre-symptomatic / asymptomatic or symptomatic conclusively)
hCoV-19/Spain/CL-ISCIII-202468/2020	EPI_ISL_539524	15/03/2020	Europe / Spain / Castilla y Leon	Human	Male	60	Hospitalized	Original	Oropharyngeal swab	B.1.5	G	Spain	Undertermined	No (couldn't determine the patient status as either pre-symptomatic / asymptomatic or symptomatic conclusively)
hCoV-19/Spain/CL-ISCIII-204996/2020	EPI_ISL_539525	19/03/2020	Europe / Spain / Castilla y Leon	Human	Female	unknown	Hospitalized	Original	Oropharyngeal swab	B.1.5	G	Spain	Undertermined	No (couldn't determine the patient status as either pre-symptomatic / asymptomatic or symptomatic conclusively)
hCoV-19/Spain/CM-ISCIII-202018188/2020	EPI_ISL_539526	21/03/2020	Europe / Spain / Castilla La Mancha	Human	Female	18	Hospitalized	Original	Oropharyngeal swab	B.1.5	G	Spain	Undertermined	No (couldn't determine the patient status as either pre-symptomatic / asymptomatic or symptomatic conclusively)
hCoV-19/Spain/CM-ISCIII-202018234/2020	EPI_ISL_539527	22/03/2020	Europe / Spain / Castilla La Mancha	Human	Male	53	Hospitalized	Original	Oropharyngeal swab	B.1.5	G	Spain	Undertermined	No (couldn't determine the patient status as either pre-symptomatic / asymptomatic or symptomatic conclusively)
hCoV-19/Spain/CM-ISCIII-202018271/2020	EPI_ISL_539528	22/03/2020	Europe / Spain / Castilla La Mancha	Human	Male	45	Hospitalized	Original	Oropharyngeal swab	B.1.5	G	Spain	Undertermined	No (couldn't determine the patient status as either pre-symptomatic / asymptomatic or symptomatic conclusively)
hCoV-19/Spain/CM-ISCIII-202018315/2020	EPI_ISL_539529	22/03/2020	Europe / Spain / Castilla La Mancha	Human	Male	51	Hospitalized	Original	Oropharyngeal swab	B.1.5	G	Spain	Undertermined	No (couldn't determine the patient status as either pre-symptomatic / asymptomatic or symptomatic conclusively)
hCoV-19/Spain/CM-ISCIII-202018318/2020	EPI_ISL_539530	22/03/2020	Europe / Spain / Castilla La Mancha	Human	Male	50	Hospitalized	Original	Oropharyngeal swab	B.1.5	G	Spain	Undertermined	No (couldn't determine the patient status as either pre-symptomatic / asymptomatic or symptomatic conclusively)
hCoV-19/Spain/CN-ISCIII-201048/2020	EPI_ISL_539531	24/02/2020	Europe / Spain / Canary Islands	Human	Male	unknown	Hospitalized	Original	Oropharyngeal swab	B.1.77	G	Spain	Undertermined	No (couldn't determine the patient status as either pre-symptomatic / asymptomatic or symptomatic conclusively)
hCoV-19/Spain/CN-ISCIII-201810/2020	EPI_ISL_539532	06/03/2020	Europe / Spain / Canary Islands	Human	Male	29	Hospitalized	Original	Oropharyngeal swab	A.2	O	Spain	Undertermined	No (couldn't determine the patient status as either pre-symptomatic / asymptomatic or symptomatic conclusively)
hCoV-19/Spain/CT-ISCIII-2012562/2020	EPI_ISL_539533	13/05/2020	Europe / Spain / Catalunya	Human	Male	91	Hospitalized	Original	Oropharyngeal swab	B.1.5	G	Spain	Undertermined	No (couldn't determine the patient status as either pre-symptomatic / asymptomatic or symptomatic conclusively)
hCoV-19/Spain/CT-ISCIII-2012563/2020	EPI_ISL_539534	13/05/2020	Europe / Spain / Catalunya	Human	Female	84	Hospitalized	Original	Oropharyngeal swab	B.1.5.3	G	Spain	Undertermined	No (couldn't determine the patient status as either pre-symptomatic / asymptomatic or symptomatic conclusively)
hCoV-19/Spain/CT-ISCIII-2012564/2020	EPI_ISL_539535	15/05/2020	Europe / Spain / Catalunya	Human	Female	85	Hospitalized	Original	Oropharyngeal swab	B.1.5.3	G	Spain	Undertermined	No (couldn't determine the patient status as either pre-symptomatic / asymptomatic or symptomatic conclusively)
hCoV-19/Spain/CT-ISCIII-2012566/2020	EPI_ISL_539536	15/05/2020	Europe / Spain / Catalunya	Human	Female	52	Hospitalized	Original	Oropharyngeal swab	B.1.5	G	Spain	Undertermined	No (couldn't determine the patient status as either pre-symptomatic / asymptomatic or symptomatic conclusively)
hCoV-19/Spain/CT-ISCIII-2012568/2020	EPI_ISL_539537	15/05/2020	Europe / Spain / Catalunya	Human	Female	24	Hospitalized	Original	Oropharyngeal swab	B.1.5	G	Spain	Undertermined	No (couldn't determine the patient status as either pre-symptomatic / asymptomatic or symptomatic conclusively)
hCoV-19/Spain/CT-ISCIII-2012571/2020	EPI_ISL_539538	20/05/2020	Europe / Spain / Catalunya	Human	Female	unknown	Hospitalized	Original	Oropharyngeal swab	B.1.5	G	Spain	Undertermined	No (couldn't determine the patient status as either pre-symptomatic / asymptomatic or symptomatic conclusively)
hCoV-19/Spain/CT-ISCIII-2012572/2020	EPI_ISL_539539	21/05/2020	Europe / Spain / Catalunya	Human	Male	84	Hospitalized	Original	Oropharyngeal swab	B.1.1	GR	Spain	Undertermined	No (couldn't determine the patient status as either pre-symptomatic / asymptomatic or symptomatic conclusively)
hCoV-19/Spain/CT-ISCIII-2012573/2020	EPI_ISL_539540	21/05/2020	Europe / Spain / Catalunya	Human	Male	70	Hospitalized	Original	Oropharyngeal swab	B.1.1	GR	Spain	Undertermined	No (couldn't determine the patient status as either pre-symptomatic / asymptomatic or symptomatic conclusively)

hCoV-19/Spain/CT-ISCIII-2013585/2020	EPI_ISL_539541	29/06/2020	Europe / Spain / Catalunya	Human		Female	82	Hospitalized	Original	Oropharyngeal swab		B.1.5	G	Spain	Undertermined	No (couldn't determine the patient status as either pre-symptomatic / asymptomatic or symptomatic conclusively)
hCoV-19/Spain/CT-ISCIII-2013586/2020	EPI_ISL_539542	29/06/2020	Europe / Spain / Catalunya	Human		Female	85	Hospitalized	Original	Oropharyngeal swab		B.1.5	G	Spain	Undertermined	No (couldn't determine the patient status as either pre-symptomatic / asymptomatic or symptomatic conclusively)
hCoV-19/Spain/CT-ISCIII-2013591/2020	EPI_ISL_539543	11/06/2020	Europe / Spain / Catalunya	Human		Female	26	Hospitalized	Original	Oropharyngeal swab		B.1.1	GR	Spain	Undertermined	No (couldn't determine the patient status as either pre-symptomatic / asymptomatic or symptomatic conclusively)
hCoV-19/Spain/CT-ISCIII-2013592/2020	EPI_ISL_539544	11/06/2020	Europe / Spain / Catalunya	Human		Male	40	Hospitalized	Original	Oropharyngeal swab		B.1.1	GR	Spain	Undertermined	No (couldn't determine the patient status as either pre-symptomatic / asymptomatic or symptomatic conclusively)
hCoV-19/Spain/CT-ISCIII-2013593/2020	EPI_ISL_539545	09/06/2020	Europe / Spain / Catalunya	Human		Male	43	Hospitalized	Original	Oropharyngeal swab		B.1.1	GR	Spain	Undertermined	No (couldn't determine the patient status as either pre-symptomatic / asymptomatic or symptomatic conclusively)
hCoV-19/Spain/CT-ISCIII-2013594/2020	EPI_ISL_539546	01/06/2020	Europe / Spain / Catalunya	Human		Male	27	Hospitalized	Original	Oropharyngeal swab		B.1.1	GR	Spain	Undertermined	No (couldn't determine the patient status as either pre-symptomatic / asymptomatic or symptomatic conclusively)
hCoV-19/Spain/CT-ISCIII-2013595/2020	EPI_ISL_539547	28/06/2020	Europe / Spain / Catalunya	Human		Male	48	Hospitalized	Original	Oropharyngeal swab		B.1.1	GR	Spain	Undertermined	No (couldn't determine the patient status as either pre-symptomatic / asymptomatic or symptomatic conclusively)
hCoV-19/Spain/CT-ISCIII-2013597/2020	EPI_ISL_539548	26/06/2020	Europe / Spain / Catalunya	Human		Female	66	Hospitalized	Original	Oropharyngeal swab		B.1	G	Spain	Undertermined	No (couldn't determine the patient status as either pre-symptomatic / asymptomatic or symptomatic conclusively)
hCoV-19/Spain/CT-ISCIII-202044/2020	EPI_ISL_539549	03/03/2020	Europe / Spain / Catalunya	Human		Female	87	Hospitalized	Original	Oropharyngeal swab		B.1	G	Spain	Undertermined	No (couldn't determine the patient status as either pre-symptomatic / asymptomatic or symptomatic conclusively)
hCoV-19/Spain/CT-ISCIII-202045/2020	EPI_ISL_539550	01/03/2020	Europe / Spain / Catalunya	Human		Male	37	Hospitalized	Original	Oropharyngeal swab		B.2	V	Spain	Undertermined	No (couldn't determine the patient status as either pre-symptomatic / asymptomatic or symptomatic conclusively)
hCoV-19/Spain/CT-ISCIII-202050/2020	EPI_ISL_539551	01/03/2020	Europe / Spain / Catalunya	Human		Female	unknown	Hospitalized	Original	Oropharyngeal swab		B.1.5	G	Spain	Undertermined	No (couldn't determine the patient status as either pre-symptomatic / asymptomatic or symptomatic conclusively)
hCoV-19/Spain/CT-ISCIII-209811/2020	EPI_ISL_539552	20/03/2020	Europe / Spain / Catalunya	Human		Female	unknown	Hospitalized	Original	Oropharyngeal swab		B.1	GH	Spain	Undertermined	No (couldn't determine the patient status as either pre-symptomatic / asymptomatic or symptomatic conclusively)
hCoV-19/Spain/CT-ISCIII-209814/2020	EPI_ISL_539553	24/03/2020	Europe / Spain / Catalunya	Human		Male	58	Hospitalized	Original	Oropharyngeal swab		B.1	GH	Spain	Undertermined	No (couldn't determine the patient status as either pre-symptomatic / asymptomatic or symptomatic conclusively)
hCoV-19/Spain/CT-ISCIII-209815/2020	EPI_ISL_539554	24/03/2020	Europe / Spain / Catalunya	Human		Male	97	Hospitalized	Original	Oropharyngeal swab		B.1.5	G	Spain	Undertermined	No (couldn't determine the patient status as either pre-symptomatic / asymptomatic or symptomatic conclusively)
hCoV-19/Spain/CT-ISCIII-209818/2020	EPI_ISL_539555	09/03/2020	Europe / Spain / Catalunya	Human		Male	80	Hospitalized	Original	Oropharyngeal swab		B.1.5	G	Spain	Undertermined	No (couldn't determine the patient status as either pre-symptomatic / asymptomatic or symptomatic conclusively)
hCoV-19/Spain/CT-ISCIII-34696/2020	EPI_ISL_539556	02/03/2020	Europe / Spain / Catalunya	Human		Male	76	Hospitalized	Original	Oropharyngeal swab		B.1.1	GR	Spain	Undertermined	No (couldn't determine the patient status as either pre-symptomatic / asymptomatic or symptomatic conclusively)
hCoV-19/Spain/EX-ISCIII-201239/2020	EPI_ISL_539557	28/02/2020	Europe / Spain / Extremadura	Human		Male	57	Hospitalized	Original	Oropharyngeal swab		B.1	G	Spain	Undertermined	No (couldn't determine the patient status as either pre-symptomatic / asymptomatic or symptomatic conclusively)
hCoV-19/Spain/EX-ISCIII-201248/2020	EPI_ISL_539558	28/02/2020	Europe / Spain / Extremadura	Human		Male	57	Hospitalized	Original	Oropharyngeal swab		A.5	O	Spain	Undertermined	No (couldn't determine the patient status as either pre-symptomatic / asymptomatic or symptomatic conclusively)
hCoV-19/Spain/EX-ISCIII-201654/2020	EPI_ISL_539559	07/03/2020	Europe / Spain / Extremadura	Human		Male	46	Hospitalized	Original	Oropharyngeal swab		A	O	Spain	Undertermined	No (couldn't determine the patient status as either pre-symptomatic / asymptomatic or symptomatic conclusively)
hCoV-19/Spain/EX-ISCIII-201655/2020	EPI_ISL_539560	07/03/2020	Europe / Spain / Extremadura	Human		Female	41	Hospitalized	Original	Oropharyngeal swab		B.2	V	Spain	Undertermined	No (couldn't determine the patient status as either pre-symptomatic / asymptomatic or symptomatic conclusively)
hCoV-19/Spain/EX-ISCIII-201899/2020	EPI_ISL_539561	09/03/2020	Europe / Spain / Extremadura	Human		Female	59	Hospitalized	Original	Oropharyngeal swab		B.1.5	G	Spain	Undertermined	No (couldn't determine the patient status as either pre-symptomatic / asymptomatic or symptomatic conclusively)
hCoV-19/Spain/EX-ISCIII-201963/2020	EPI_ISL_539562	10/03/2020	Europe / Spain / Extremadura	Human		Female	62	Hospitalized	Original	Oropharyngeal swab		B.1.5	G	Spain	Undertermined	No (couldn't determine the patient status as either pre-symptomatic / asymptomatic or symptomatic conclusively)
hCoV-19/Spain/GA-ISCIII-201794/2020	EPI_ISL_539563	09/03/2020	Europe / Spain / Galicia	Human		Female	44	Hospitalized	Original	Oropharyngeal swab		A.2	S	Spain	Undertermined	No (couldn't determine the patient status as either pre-symptomatic / asymptomatic or symptomatic conclusively)
hCoV-19/Spain/GA-ISCIII-201859/2020	EPI_ISL_539564	10/03/2020	Europe / Spain / Galicia	Human		Male	89	Hospitalized	Original	Oropharyngeal swab		A.2	S	Spain	Undertermined	No (couldn't determine the patient status as either pre-symptomatic / asymptomatic or symptomatic conclusively)
hCoV-19/Spain/GA-ISCIII-202075/2020	EPI_ISL_539565	10/03/2020	Europe / Spain / Galicia	Human		Male	82	Hospitalized	Original	Oropharyngeal swab		B.2	V	Spain	Undertermined	No (couldn't determine the patient status as either pre-symptomatic / asymptomatic or symptomatic conclusively)
hCoV-19/Spain/GA-ISCIII-202077/2020	EPI_ISL_539566	11/03/2020	Europe / Spain / Galicia	Human		Female	37	Hospitalized	Original	Oropharyngeal swab		A.2	S	Spain	Undertermined	No (couldn't determine the patient status as either pre-symptomatic / asymptomatic or symptomatic conclusively)
hCoV-19/Spain/ML-ISCIII-2013686/2020	EPI_ISL_539567	01/08/2020	Europe / Spain / Melilla	Human		Female	8	Hospitalized	Original	Oropharyngeal swab		B.1.1	GR	Spain	Undertermined	No (couldn't determine the patient status as either pre-symptomatic / asymptomatic or symptomatic conclusively)
hCoV-19/Spain/ML-ISCIII-2013688/2020	EPI_ISL_539568	01/08/2020	Europe / Spain / Melilla	Human		Male	29	Hospitalized	Original	Oropharyngeal swab		B.1.1	GR	Spain	Undertermined	No (couldn't determine the patient status as either pre-symptomatic / asymptomatic or symptomatic conclusively)
hCoV-19/Spain/NA-ISCIII-201944/2020	EPI_ISL_539569	09/03/2020	Europe / Spain / Navarra	Human		Female	53	Hospitalized	Original	Oropharyngeal swab		A.2	O	Spain	Undertermined	No (couldn't determine the patient status as either pre-symptomatic / asymptomatic or symptomatic conclusively)
hCoV-19/Spain/NA-ISCIII-201945/2020	EPI_ISL_539570	09/03/2020	Europe / Spain / Navarra	Human		Female	46	Hospitalized	Original	Oropharyngeal swab		A.2	S	Spain	Undertermined	No (couldn't determine the patient status as either pre-symptomatic / asymptomatic or symptomatic conclusively)
hCoV-19/Spain/NA-ISCIII-201951/2020	EPI_ISL_539571	09/03/2020	Europe / Spain / Navarra	Human		Female	1	Hospitalized	Original	Oropharyngeal swab		A.2	S	Spain	Undertermined	No (couldn't determine the patient status as either pre-symptomatic / asymptomatic or symptomatic conclusively)
hCoV-19/Spain/NA-ISCIII-201952/2020	EPI_ISL_539572	09/03/2020	Europe / Spain / Navarra	Human		Female	35	Hospitalized	Original	Oropharyngeal swab		A.2	O	Spain	Undertermined	No (couldn't determine the patient status as either pre-symptomatic / asymptomatic or symptomatic conclusively)
hCoV-19/Italy/SAR-ATS-246/2020	EPI_ISL_547965	25/08/2020	Europe / Italy / Sardinia	Human		Male	63	Live	Original	Nasopharyngeal swab		B.1	G	Italy	Undertermined	No (couldn't determine the patient status as either pre-symptomatic / asymptomatic or symptomatic conclusively)
hCoV-19/Czech Republic/NRL_8118-3/2020	EPI_ISL_547966	06/08/2020	Europe / Czech Republic / ÃšstÃ- nad Labem Region / LitomÄ›Å™ice	Human		Female	64	Mild clinical signs without hospitalization	Original	Nasopharyngeal swab	Wife of hCoV-19/Czech Republic/NRL_8118-4/2020	B.1.1	GR	Czech Republic	Symptomatic	Yes
hCoV-19/Czech Republic/NRL_8118-4/2020	EPI_ISL_547967	07/08/2020	Europe / Czech Republic / ÃšstÃ- nad Labem Region / LitomÄ›Å™ice	Human		Male	64	Mild clinical signs without hospitalization	Original	Nasopharyngeal swab	Husband of hCoV-19/Czech Republic/NRL_8118-3/2020	B.1.1	GR	Czech Republic	Symptomatic	Yes
hCoV-19/Czech Republic/NRL_8118-5/2020	EPI_ISL_547968	07/08/2020	Europe / Czech Republic / ÃšstÃ- nad Labem Region / Libochovice	Human		Female	45	Mild clinical signs without hospitalization	Original	Nasopharyngeal swab		B.1.1	GR	Czech Republic	Symptomatic	Yes
hCoV-19/Indonesia/EJ-ITD-3601NT/2020	EPI_ISL_560991	14/04/2020	Asia / Indonesia / East Java / Surabaya	Human		Male	56	Hospitalized	Original	Nasopharyngeal and Oropharyngeal swab		B.6	O	Indonesia	Undertermined	No (couldn't determine the patient status as either pre-symptomatic / asymptomatic or symptomatic conclusively)
hCoV-19/Thailand/61/2020	EPI_ISL_403962	08/01/2020	Asia / Thailand / Nonthaburi	Human		Female	61	Recovered	Original	Nasopharyngeal swab and Throat swab	Patient came from Wuhan, China	B	L	Thailand	Undertermined	No (couldn't determine the patient status as either pre-symptomatic / asymptomatic or symptomatic conclusively)
hCoV-19/Thailand/74/2020	EPI_ISL_403963	13/01/2020	Asia / Thailand / Nonthaburi	Human		Female	74	Live	Original	Nasopharyngeal swab and Throat swab	Patient came from Wuhan, China	B	L	Thailand	Undertermined	No (couldn't determine the patient status as either pre-symptomatic / asymptomatic or symptomatic conclusively)
hCoV-19/Zhejiang/WZ-01/2020	EPI_ISL_404227	16/01/2020	Asia / China / Zhejiang	Human		Male	46	hospitalized	Original	Sputum	Patient came from Wuhan, China	B	L	China	Undertermined	No (couldn't determine the patient status as either pre-symptomatic / asymptomatic or symptomatic conclusively)
hCoV-19/Zhejiang/WZ-02/2020	EPI_ISL_404228	17/01/2020	Asia / China / Zhejiang	Human		Male	47	hospitalized	Original	Sputum	Patient came from Wuhan, China	B	L	China	Undertermined	No (couldn't determine the patient status as either pre-symptomatic / asymptomatic or symptomatic conclusively)
hCoV-19/Australia/NSW03/2020	EPI_ISL_408977	25/01/2020	Oceania / Australia / New South Wales / Sydney	Human		Female	21	Recovered	Original	Nasopharyngeal swab		B.2	O	Australia	Undertermined	No (couldn't determine the patient status as either pre-symptomatic / asymptomatic or symptomatic conclusively)
hCoV-19/Japan/TK-20-31-3/2020	EPI_ISL_413459	20/02/2020	Asia / Japan / Tokyo	Human	Diamond Princess cruise ship	Female	84	Deceased	Original	Autopsy, Bronch and lung	Pneumonia (Chest X-ray, CT)	B.5	O	Japan	Symptomatic	Yes
hCoV-19/New Zealand/01/2020	EPI_ISL_413490	27/02/2020	Oceania / New Zealand / Auckland	Human		Female	>60	Hospitalized	Original	Sputum	Infected in Iran	B.4	O	New Zealand	Undertermined	No (couldn't determine the patient status as either pre-symptomatic / asymptomatic or symptomatic conclusively)
hCoV-19/France/GE1583-2/2020	EPI_ISL_414623	25/02/2020	Europe / France / Grand-Est / Strasbourg	Human		Male	36	Hospitalized	Original	Sputum		A.2	S	France	Undertermined	No (couldn't determine the patient status as either pre-symptomatic / asymptomatic or symptomatic conclusively)
hCoV-19/France/N1620/2020	EPI_ISL_414624	26/02/2020	Europe / France / Normandie / Rouen	Human		unknown	unknown	hospitalized	Original	Oropharyngeal swab		B.1	G	France	Undertermined	No (couldn't determine the patient status as either pre-symptomatic / asymptomatic or symptomatic conclusively)
hCoV-19/France/PL1643/2020	EPI_ISL_414625	26/02/2020	Europe / France / Pays de la Loire / Nantes	Human		Female	59	Hospitalized	Original	unknown		B.1	GH	France	Undertermined	No (couldn't determine the patient status as either pre-symptomatic / asymptomatic or symptomatic conclusively)
hCoV-19/France/HF1684/2020	EPI_ISL_414626	29/02/2020	Europe / France / Hauts de France / CrÃ©py en Valois	Human		female	88	Hospitalized	Original	Sputum		B.1	GH	France	Undertermined	No (couldn't determine the patient status as either pre-symptomatic / asymptomatic or symptomatic conclusively)
hCoV-19/France/HF1795/2020	EPI_ISL_414627	02/03/2020	Europe / France / Hauts de France / CompiÃ¨gne	Human		Male	78	Hospitalized	Original	Oropharyngeal swab		B.1	GH	France	Undertermined	No (couldn't determine the patient status as either pre-symptomatic / asymptomatic or symptomatic conclusively)
hCoV-19/France/HF1805/2020	EPI_ISL_414628	02/03/2020	Europe / France / Hauts de France / CompiÃ¨gne	Human		Male	82	Hospitalized	Original	Oropharyngeal swab		B.1	GH	France	Undertermined	No (couldn't determine the patient status as either pre-symptomatic / asymptomatic or symptomatic conclusively)
hCoV-19/France/HF1870/2020	EPI_ISL_414629	03/03/2020	Europe / France / Hauts de France / CompiÃ¨gne	Human		Male	88	Hospitalized	Original	Oropharyngeal swab		B.1	G	France	Undertermined	No (couldn't determine the patient status as either pre-symptomatic / asymptomatic or symptomatic conclusively)
hCoV-19/France/HF1871/2020	EPI_ISL_414630	03/03/2020	Europe / France / Hauts de France / CompiÃ¨gne	Human		Male	65	Hospitalized	Original	Oropharyngeal swab		B.1	GH	France	Undertermined	No (couldn't determine the patient status as either pre-symptomatic / asymptomatic or symptomatic conclusively)
hCoV-19/France/GE1973/2020	EPI_ISL_414631	04/03/2020	Europe / France / Grand-Est / Reims	Human		Male	88	Hospitalized	Original	Oropharyngeal swab		B.1	G	France	Undertermined	No (couldn't determine the patient status as either pre-symptomatic / asymptomatic or symptomatic conclusively)
hCoV-19/France/GE1977/2020	EPI_ISL_414632	04/03/2020	Europe / France / Grand-Est / Reims	Human		Male	70	Hospitalized	Original	Oropharyngeal swab		B.1	GH	France	Undertermined	No (couldn't determine the patient status as either pre-symptomatic / asymptomatic or symptomatic conclusively)
hCoV-19/France/IDF1980/2020	EPI_ISL_414633	04/03/2020	Europe / France / Ile-de-France / Pontoise	Human		Male	86	Hospitalized	Original	Oropharyngeal swab		B.1	GH	France	Undertermined	No (couldn't determine the patient status as either pre-symptomatic / asymptomatic or symptomatic conclusively)
hCoV-19/France/HF1986/2020	EPI_ISL_414634	04/03/2020	Europe / France / Hauts de France / CompiÃ¨gne	Human		Male	60	Hospitalized	Original	Sputum		B.1	GH	France	Undertermined	No (couldn't determine the patient status as either pre-symptomatic / asymptomatic or symptomatic conclusively)
hCoV-19/France/HF1988/2020	EPI_ISL_414635	04/03/2020	Europe / France / Hauts de France / CompiÃ¨gne	Human		Female	70	Hospitalized	Original	Oropharyngeal swab		B.1	GH	France	Undertermined	No (couldn't determine the patient status as either pre-symptomatic / asymptomatic or symptomatic conclusively)
hCoV-19/France/HF1989/2020	EPI_ISL_414636	04/03/2020	Europe / France / Hauts de France / CompiÃ¨gne	Human		Male	95	Hospitalized	Original	Oropharyngeal swab		B.1	GH	France	Undertermined	No (couldn't determine the patient status as either pre-symptomatic / asymptomatic or symptomatic conclusively)
hCoV-19/France/HF1993/2020	EPI_ISL_414637	04/03/2020	Europe / France / Hauts de France / CompiÃ¨gne	Human		Male	68	Hospitalized	Original	Oropharyngeal swab		B.1	GH	France	Undertermined	No (couldn't determine the patient status as either pre-symptomatic / asymptomatic or symptomatic conclusively)
hCoV-19/France/HF1995/2020	EPI_ISL_414638	04/03/2020	Europe / France / Hauts de France / CompiÃ¨gne	Human		Female	86	Hospitalized	Original	Oropharyngeal swab		B.1	GH	France	Undertermined	No (couldn't determine the patient status as either pre-symptomatic / asymptomatic or symptomatic conclusively)
hCoV-19/DRC/KN-13/2020	EPI_ISL_414647	09/03/2020	Africa / Democratic Republic of the Congo / Kinshasa	Human		Male	52	Stable in quarantine	Original	Oro-pharyngeal swab		B.1	GH	Democratic Republic of the Congo	Undertermined	No (couldn't determine the patient status as either pre-symptomatic / asymptomatic or symptomatic conclusively)
hCoV-19/Guangzhou/GZMU0016/2020	EPI_ISL_414663	25/02/2020	Asia / China / Guangdong / Guangzhou	Human		Male	72	Alive	Original	Sputum		A	S	China	Undertermined	No (couldn't determine the patient status as either pre-symptomatic / asymptomatic or symptomatic conclusively)
hCoV-19/Guangzhou/GZMU0048/2020	EPI_ISL_414691	25/02/2020	Asia / China / Guangdong / Guangzhou	Human		Male	79	Live	Original	Upper respiratory tract		A	S	China	Undertermined	No (couldn't determine the patient status as either pre-symptomatic / asymptomatic or symptomatic conclusively)
hCoV-19/Spain/GA-ISCIII-201663/2020	EPI_ISL_417007	07/03/2020	Europe / Spain / Galicia	Human		Female	36	Hospitalized	Original	Oropharyngeal swab		B.1.5	G	Spain	Undertermined	No (couldn't determine the patient status as either pre-symptomatic / asymptomatic or symptomatic conclusively)
hCoV-19/Spain/MD-ISCIII-201442/2020	EPI_ISL_417010	04/03/2020	Europe / Spain / Madrid	Human		Female	58	Hospitalized	Original	Oropharyngeal swab		B.1.5	G	Spain	Undertermined	No (couldn't determine the patient status as either pre-symptomatic / asymptomatic or symptomatic conclusively)
hCoV-19/Belgium/ULG-6457/2020	EPI_ISL_417015	13/03/2020	Europe / Belgium / LiÃ¨ge	Human		Female	27	hospitalized or to be hospitalized	Original	Nasopharynx swab		B.1.1	GR	Belgium	Undertermined	No (couldn't determine the patient status as either pre-symptomatic / asymptomatic or symptomatic conclusively)
hCoV-19/Belgium/ULG-6503/2020	EPI_ISL_417016	13/03/2020	Europe / Belgium / LiÃ¨ge	Human		Male	85	hospitalized or to be hospitalized	Original	Nasopharynx swab		B.1	G	Belgium	Undertermined	No (couldn't determine the patient status as either pre-symptomatic / asymptomatic or symptomatic conclusively)
hCoV-19/Belgium/ULG-6670/2020	EPI_ISL_417018	14/03/2020	Europe / Belgium / LiÃ¨ge	Human		Male	53	hospitalized or to be hospitalized	Original	Nasopharynx swab		B.1.9	GH	Belgium	Undertermined	No (couldn't determine the patient status as either pre-symptomatic / asymptomatic or symptomatic conclusively)
hCoV-19/Belgium/ULG-6754/2020	EPI_ISL_417019	14/03/2020	Europe / Belgium / Liege	Human		Male	71	Hospitalized or to be hospitalized	Original	Nasopharynx swab		B.1	G	Belgium	Undertermined	No (couldn't determine the patient status as either pre-symptomatic / asymptomatic or symptomatic conclusively)
hCoV-19/Australia/NSW04/2020	EPI_ISL_417030	24/01/2020	Oceania / Australia / New South Wales / Sydney	Human		Male	35	Recovered	Original	Throat swab	Travel history to China	A	S	Australia	Undertermined	No (couldn't determine the patient status as either pre-symptomatic / asymptomatic or symptomatic conclusively)
hCoV-19/Brazil/AM-02/2020	EPI_ISL_417034	16/03/2020	South America / Brazil / Amazonas / Manaus	Human		Male	56	Asymptomatic	Original	Oro-pharyngeal swab	Imported case, patient returning from Madrid, Spain	A.2	S	Brazil	Pre-symptomatic / Asymptomatic	Yes
hCoV-19/Hungary/mbl2/2020	EPI_ISL_418183	17/03/2020	Europe / Hungary / Baranya	Human	Hospital	Female	26	Hospitalized	Original	Oro-pharyngeal swab		B.1.5	G	Hungary	Undertermined	No (couldn't determine the patient status as either pre-symptomatic / asymptomatic or symptomatic conclusively)
hCoV-19/Senegal/003/2020	EPI_ISL_418206	28/02/2020	Africa / Senegal / Dakar	Human		Male	33	Released	Original	Nasopharyngeal and oropharyngeal swabs		B.1	GH	Senegal	Undertermined	No (couldn't determine the patient status as either pre-symptomatic / asymptomatic or symptomatic conclusively)
hCoV-19/Senegal/016/2020	EPI_ISL_418207	02/03/2020	Africa / Senegal / Dakar	Human		Male	80	Released	Original	Oro and naso-pharyngeal swabs		B.1	GH	Senegal	Undertermined	No (couldn't determine the patient status as either pre-symptomatic / asymptomatic or symptomatic conclusively)
hCoV-19/Senegal/020/2020	EPI_ISL_418208	04/03/2020	Africa / Senegal / Dakar	Human		Female	68	Released	Original	Oro and naso-pharyngeal swabs		B.1	GH	Senegal	Undertermined	No (couldn't determine the patient status as either pre-symptomatic / asymptomatic or symptomatic conclusively)
hCoV-19/Senegal/026/2020	EPI_ISL_418209	03/03/2020	Africa / Senegal / Dakar	Human		Female	33	Released	Original	Oro and naso-pharyngeal swabs		B.1	GH	Senegal	Undertermined	No (couldn't determine the patient status as either pre-symptomatic / asymptomatic or symptomatic conclusively)
hCoV-19/Senegal/073/2020	EPI_ISL_418210	10/03/2020	Africa / Senegal / Diourbel / Touba	Human		Male	55	Released	Original	Oro and naso-pharyngeal swabs		B.1	G	Senegal	Undertermined	No (couldn't determine the patient status as either pre-symptomatic / asymptomatic or symptomatic conclusively)
hCoV-19/Senegal/082/2020	EPI_ISL_418211	11/03/2020	Africa / Senegal / Diourbel / Touba	Human		Female	42	Released	Original	Oro and naso-pharyngeal swabs		B.1	G	Senegal	Undertermined	No (couldn't determine the patient status as either pre-symptomatic / asymptomatic or symptomatic conclusively)
hCoV-19/Senegal/087/2020	EPI_ISL_418212	11/03/2020	Africa / Senegal / Diourbel / Touba	Human		Female	50	Released	Original	Oro and naso-pharyngeal swab		B.1	G	Senegal	Undertermined	No (couldn't determine the patient status as either pre-symptomatic / asymptomatic or symptomatic conclusively)
hCoV-19/Senegal/094/2020	EPI_ISL_418213	12/03/2020	Africa / Senegal / Diourbel / Touba	Human		Male	3	Released	Original	Oro and naso-pharyngeal swabs		B.1	G	Senegal	Undertermined	No (couldn't determine the patient status as either pre-symptomatic / asymptomatic or symptomatic conclusively)
hCoV-19/Senegal/102/2020	EPI_ISL_418214	12/03/2020	Africa / Senegal / Diourbel / Touba	Human		Female	7	Released	Original	Oro and naso-pharyngeal swabs		B.1	G	Senegal	Undertermined	No (couldn't determine the patient status as either pre-symptomatic / asymptomatic or symptomatic conclusively)
hCoV-19/Senegal/119/2020	EPI_ISL_418215	12/03/2020	Africa / Senegal / Diourbel / Touba	Human		Male	66	Live	Original	Oro and naso-pharyngeal swabs		B.1	G	Senegal	Undertermined	No (couldn't determine the patient status as either pre-symptomatic / asymptomatic or symptomatic conclusively)
hCoV-19/Senegal/136/2020	EPI_ISL_418216	13/03/2020	Africa / Senegal / Dakar	Human		Male	51 years	Live	Original	Oro and naso-pharyngeal swabs		A.2	S	Senegal	Undertermined	No (couldn't determine the patient status as either pre-symptomatic / asymptomatic or symptomatic conclusively)
hCoV-19/Senegal/139/2020	EPI_ISL_418217	13/03/2020	Africa / Senegal / Thies / Mbour	Human		Female	68 years	Released	Original	Oro and naso-pharyngeal swabs		B.1	G	Senegal	Undertermined	No (couldn't determine the patient status as either pre-symptomatic / asymptomatic or symptomatic conclusively)
hCoV-19/France/HF1465/2020	EPI_ISL_418218	21/02/2020	Europe / France / Hauts de France / CompiÃ¨gne	Human		Male	56	Hospitalized	Original	Sputum		B.1	GH	France	Undertermined	No (couldn't determine the patient status as either pre-symptomatic / asymptomatic or symptomatic conclusively)
hCoV-19/France/B1623/2020	EPI_ISL_418219	26/02/2020	Europe / France / Bretagne / Brest	Human		Male	73	Hospitalized	Original	unknown		B.1	GH	France	Undertermined	No (couldn't determine the patient status as either pre-symptomatic / asymptomatic or symptomatic conclusively)
hCoV-19/Wales/PHWC-24DB4/2020	EPI_ISL_419511	20/03/2020	Europe / United Kingdom / Wales	Human		Female	51	unknown	Original	Dry swab		B.2	V	United Kingdom	Undertermined	No (couldn't determine the patient status as either pre-symptomatic / asymptomatic or symptomatic conclusively)
hCoV-19/Austria/CeMM0009/2020	EPI_ISL_419662	14/03/2020	Europe / Austria	Human	Vienna, Austria	Male	72	Deceased	Original	Sputum		B.3	L	Austria	Symptomatic	Yes
hCoV-19/Austria/CeMM0010/2020	EPI_ISL_419663	15/03/2020	Europe / Austria	Human	Vienna, Austria	Female	48	Deceased	Original	Lung secretion		B.1.1.6	GR	Austria	Symptomatic	Yes
hCoV-19/Austria/CeMM0011/2020	EPI_ISL_419664	23/03/2020	Europe / Austria	Human	Vienna, Austria	Male	77	Deceased	Original	Lung secretion		B.1.5	G	Austria	Symptomatic	Yes
hCoV-19/Poland/Pom1/2020	EPI_ISL_428209	06/04/2020	Europe / Poland / Pomerania	Human		Male	48	Hospitalized	Original	Oro-pharyngeal swab		B.2	O	Poland	Undertermined	No (couldn't determine the patient status as either pre-symptomatic / asymptomatic or symptomatic conclusively)
hCoV-19/Taiwan/TSGH-24/2020	EPI_ISL_436099	02/04/2020	Asia / Taiwan / Taipei	Human		Female	49	Released	Original			B.1	G	Taiwan	Undertermined	No (couldn't determine the patient status as either pre-symptomatic / asymptomatic or symptomatic conclusively)
hCoV-19/Taiwan/TSGH-15/2020	EPI_ISL_436100	19/03/2020	Asia / Taiwan / New Taipei City	Human		Male	29	Released	Original			B.1.5	G	Taiwan	Undertermined	No (couldn't determine the patient status as either pre-symptomatic / asymptomatic or symptomatic conclusively)
hCoV-19/Taiwan/TSGH-16/2020	EPI_ISL_436101	19/03/2020	Asia / Taiwan / New Taipei City	Human		Male	37	Released	Original			A.1	S	Taiwan	Undertermined	No (couldn't determine the patient status as either pre-symptomatic / asymptomatic or symptomatic conclusively)
hCoV-19/Taiwan/TSGH-17/2020	EPI_ISL_436102	19/03/2020	Asia / Taiwan / New Taipei City	Human		Male	73	Released	Original			B.2.4	O	Taiwan	Undertermined	No (couldn't determine the patient status as either pre-symptomatic / asymptomatic or symptomatic conclusively)
hCoV-19/Taiwan/TSGH-18/2020	EPI_ISL_436103	24/03/2020	Asia / Taiwan / New Taipei City	Human		Female	38	Released	Original			B.1	GH	Taiwan	Undertermined	No (couldn't determine the patient status as either pre-symptomatic / asymptomatic or symptomatic conclusively)
hCoV-19/Taiwan/TSGH-19/2020	EPI_ISL_436104	26/03/2020	Asia / Taiwan / New Taipei City	Human		Male	36	Released	Original			B.1.1	GR	Taiwan	Undertermined	No (couldn't determine the patient status as either pre-symptomatic / asymptomatic or symptomatic conclusively)
hCoV-19/Taiwan/TSGH-20/2020	EPI_ISL_436105	27/03/2020	Asia / Taiwan / Taipei	Human		Male	54	Released	Original			B	L	Taiwan	Undertermined	No (couldn't determine the patient status as either pre-symptomatic / asymptomatic or symptomatic conclusively)
hCoV-19/Taiwan/TSGH-21/2020	EPI_ISL_436106	30/03/2020	Asia / Taiwan / New Taipei City	Human		Male	25	Released	Original			B.1	G	Taiwan	Undertermined	No (couldn't determine the patient status as either pre-symptomatic / asymptomatic or symptomatic conclusively)
hCoV-19/Taiwan/TSGH-22/2020	EPI_ISL_436107	31/03/2020	Asia / Taiwan / New Taipei City	Human		Male	25	Released	Original			B.1.26	GH	Taiwan	Undertermined	No (couldn't determine the patient status as either pre-symptomatic / asymptomatic or symptomatic conclusively)
hCoV-19/Taiwan/TSGH-23/2020	EPI_ISL_436108	02/04/2020	Asia / Taiwan / Taipei	Human		Female	21	Released	Original			B.1	GH	Taiwan	Undertermined	No (couldn't determine the patient status as either pre-symptomatic / asymptomatic or symptomatic conclusively)
hCoV-19/India/KA-nimh-2873/2020	EPI_ISL_436137	21/04/2020	Asia / India / Karnataka	Human		Female	28	Asymptomatic	Original			B.6	O	India	Pre-symptomatic / Asymptomatic	Yes
hCoV-19/India/KA-nimh-3952/2020	EPI_ISL_436138	23/04/2020	Asia / India / Karnataka	Human		Male	38	Asymptomatic	Original			B.1.80	G	India	Pre-symptomatic / Asymptomatic	Yes
hCoV-19/India/KA-nimh-3970/2020	EPI_ISL_436139	23/04/2020	Asia / India / Karnataka	Human		Male	20	Symptomatic	Original			B.1.80	G	India	Symptomatic	Yes
hCoV-19/India/KA-nimh-4376/2020	EPI_ISL_436140	24/04/2020	Asia / India / Karnataka	Human		Female	52	Asymptomatic	Original			B.1.80	G	India	Pre-symptomatic / Asymptomatic	Yes
hCoV-19/India/KA-nimh-4378/2020	EPI_ISL_436141	24/04/2020	Asia / India / Karnataka	Human		Female	27	Asymptomatic	Original			B.1.80	G	India	Pre-symptomatic / Asymptomatic	Yes
hCoV-19/India/KA-nimh-1596/2020	EPI_ISL_436156	17/04/2020	Asia / India / Karnataka	Human		Female	55	Asymptomatic	Original			B.6	O	India	Pre-symptomatic / Asymptomatic	Yes
hCoV-19/India/KA-nimh-1598/2020	EPI_ISL_436157	17/04/2020	Asia / India / Karnataka	Human		Female	50	Asymptomatic	Original			B.6	O	India	Pre-symptomatic / Asymptomatic	Yes
hCoV-19/Austria/Graz-MUG11/2020	EPI_ISL_437300	14/04/2020	Europe / Austria / Styria	Human		Male	77	Deceased	Original	lung swab	primary patient sample	B	O	Austria	Symptomatic	Yes
hCoV-19/Austria/Graz-MUG15/2020	EPI_ISL_437301	17/04/2020	Europe / Austria / Styria	Human		Female	80	Deceased	Original	nose swab	primary patient sample	B.1	O	Austria	Symptomatic	Yes
hCoV-19/Austria/Graz-MUG16/2020	EPI_ISL_437302	17/04/2020	Europe / Austria / Styria	Human		Female	80	Deceased	Original	nose swab	primary patient sample	B.1.22	O	Austria	Symptomatic	Yes
hCoV-19/Austria/Graz-MUG17/2020	EPI_ISL_437303	17/04/2020	Europe / Austria / Styria	Human		Male	80	Deceased	Original	pharynx swab	primary patient sample	B.2	V	Austria	Symptomatic	Yes
hCoV-19/Taiwan/TSGH-25/2020	EPI_ISL_437336	22/03/2020	Asia / Taiwan / New Taipei City	Human		Female	27	Released	Original			B	O	Taiwan	Undertermined	No (couldn't determine the patient status as either pre-symptomatic / asymptomatic or symptomatic conclusively)
hCoV-19/DRC/2536/2020	EPI_ISL_437339	15/04/2020	Africa / Democratic Republic of the Congo / Kinshasa	Human		Male	48	Hospitalized	Original			B.1	G	Democratic Republic of the Congo	Undertermined	No (couldn't determine the patient status as either pre-symptomatic / asymptomatic or symptomatic conclusively)
hCoV-19/DRC/2644/2020	EPI_ISL_437342	15/04/2020	Africa / Democratic Republic of the Congo / Kinshasa	Human		Female	47	Hospitalized	Original			B.1.84	G	Democratic Republic of the Congo	Undertermined	No (couldn't determine the patient status as either pre-symptomatic / asymptomatic or symptomatic conclusively)
hCoV-19/DRC/2727/2020	EPI_ISL_437344	16/04/2020	Africa / Democratic Republic of the Congo / Kinshasa	Human		Female	74	Hospitalized	Original			B.1	G	Democratic Republic of the Congo	Undertermined	No (couldn't determine the patient status as either pre-symptomatic / asymptomatic or symptomatic conclusively)
hCoV-19/DRC/2728/2020	EPI_ISL_437345	16/04/2020	Africa / Democratic Republic of the Congo / Kinshasa	Human		Male	51	Hospitalized	Original			B.1.84	G	Democratic Republic of the Congo	Undertermined	No (couldn't determine the patient status as either pre-symptomatic / asymptomatic or symptomatic conclusively)
hCoV-19/USA/MT-BHDH-074/2020	EPI_ISL_437433	15/04/2020	North America / USA / Montana	Human		Male	56	Live	Original			A.1	S	USA	Undertermined	No (couldn't determine the patient status as either pre-symptomatic / asymptomatic or symptomatic conclusively)

hCoV-19/India/GJ-GBRC11/2020	EPI_ISL_437439	23/04/2020	Asia / India / Gujarat / Surat	Human	Male	41	Released	Original	Oro-pharyngeal swab		A	S	India	Undertermined	No (couldn't determine the patient status as either pre-symptomatic / asymptomatic or symptomatic conclusively)
hCoV-19/India/GJ-GBRC12/2020	EPI_ISL_437440	26/04/2020	Asia / India / Gujarat / Surat	Human	Male	7	Released	Original	Oro-pharyngeal swab		B.1.36	GH	India	Undertermined	No (couldn't determine the patient status as either pre-symptomatic / asymptomatic or symptomatic conclusively)
hCoV-19/India/GJ-GBRC14/2020	EPI_ISL_437442	27/04/2020	Asia / India / Gujarat / Surat	Human	Female	7	Released	Original	Oro-pharyngeal swab		B.6	O	India	Undertermined	No (couldn't determine the patient status as either pre-symptomatic / asymptomatic or symptomatic conclusively)
hCoV-19/India/GJ-GBRC17a/2020	EPI_ISL_437445	26/04/2020	Asia / India / Gujarat / Ahmedabad	Human	Female	29	Released	Original	Oro-pharyngeal swab		B.1.113	GH	India	Undertermined	No (couldn't determine the patient status as either pre-symptomatic / asymptomatic or symptomatic conclusively)
hCoV-19/India/GJ-GBRC17b/2020	EPI_ISL_437446	26/04/2020	Asia / India / Gujarat / Ahmedabad	Human	Female	29	Released	Original	Oro-pharyngeal swab		B.1.113	GH	India	Undertermined	No (couldn't determine the patient status as either pre-symptomatic / asymptomatic or symptomatic conclusively)
hCoV-19/India/GJ-GBRC18/2020	EPI_ISL_437447	26/04/2020	Asia / India / Gujarat / Ahmedabad	Human	Male	42	Released	Original	Oro-pharyngeal swab		B.1	G	India	Undertermined	No (couldn't determine the patient status as either pre-symptomatic / asymptomatic or symptomatic conclusively)
hCoV-19/India/GJ-GBRC19/2020	EPI_ISL_437448	26/04/2020	Asia / India / Gujarat / Ahmedabad	Human	Female	40	Released	Original	Oro-pharyngeal swab		B.1	G	India	Undertermined	No (couldn't determine the patient status as either pre-symptomatic / asymptomatic or symptomatic conclusively)
hCoV-19/India/GJ-GBRC20/2020	EPI_ISL_437449	26/04/2020	Asia / India / Gujarat / Ahmedabad	Human	Female	17	Released	Original	Oro-pharyngeal swab		B.1.36	GH	India	Undertermined	No (couldn't determine the patient status as either pre-symptomatic / asymptomatic or symptomatic conclusively)
hCoV-19/India/GJ-GBRC21/2020	EPI_ISL_437450	26/04/2020	Asia / India / Gujarat / Ahmedabad	Human	Female	36	Released	Original	Oro-pharyngeal swab		B.1	G	India	Undertermined	No (couldn't determine the patient status as either pre-symptomatic / asymptomatic or symptomatic conclusively)
hCoV-19/India/GJ-GBRC23/2020	EPI_ISL_437452	26/04/2020	Asia / India / Gujarat / Ahmedabad	Human	Female	12	Released	Original	Oro-pharyngeal swab		B.1.113	GH	India	Undertermined	No (couldn't determine the patient status as either pre-symptomatic / asymptomatic or symptomatic conclusively)
hCoV-19/India/GJ-GBRC24a/2020	EPI_ISL_437453	26/04/2020	Asia / India / Gujarat / Ahmedabad	Human	Female	52	Deceased	Original	Oro-pharyngeal swab		B.6	L	India	Symptomatic	Yes
hCoV-19/India/GJ-GBRC24b/2020	EPI_ISL_437454	26/04/2020	Asia / India / Gujarat / Ahmedabad	Human	Female	52	Deceased	Original	Oro-pharyngeal swab		B.6	O	India	Symptomatic	Yes
hCoV-19/Saudi Arabia/KAUST-Makkah03/2020	EPI_ISL_437459	23/03/2020	Asia / Saudi Arabia / Makkah	Human	Female	23	Hospitalized	Original			A	S	Saudi Arabia	Undertermined	No (couldn't determine the patient status as either pre-symptomatic / asymptomatic or symptomatic conclusively)
hCoV-19/Saudi Arabia/KAUST-Makkah04/2020	EPI_ISL_437460	23/03/2020	Asia / Saudi Arabia / Makkah	Human	Male	67	Hospitalized	Original			A	S	Saudi Arabia	Undertermined	No (couldn't determine the patient status as either pre-symptomatic / asymptomatic or symptomatic conclusively)
hCoV-19/Saudi Arabia/KAUST-Makkah05/2020	EPI_ISL_437461	23/03/2020	Asia / Saudi Arabia / Makkah	Human	Male	37	Hospitalized	Original			A	S	Saudi Arabia	Undertermined	No (couldn't determine the patient status as either pre-symptomatic / asymptomatic or symptomatic conclusively)
hCoV-19/Saudi Arabia/KAUST-Makkah06/2020	EPI_ISL_437462	23/03/2020	Asia / Saudi Arabia / Makkah	Human	Female	69	Hospitalized	Original			A	S	Saudi Arabia	Undertermined	No (couldn't determine the patient status as either pre-symptomatic / asymptomatic or symptomatic conclusively)
hCoV-19/Saudi Arabia/KAUST-Makkah09/2020	EPI_ISL_437463	23/03/2020	Asia / Saudi Arabia / Makkah	Human	Male	50	Hospitalized	Original			B.1.9	GH	Saudi Arabia	Undertermined	No (couldn't determine the patient status as either pre-symptomatic / asymptomatic or symptomatic conclusively)
hCoV-19/Saudi Arabia/KAUST-Makkah10/2020	EPI_ISL_437464	23/03/2020	Asia / Saudi Arabia / Makkah	Human	Male	54	Hospitalized	Original			B.1.36	GH	Saudi Arabia	Undertermined	No (couldn't determine the patient status as either pre-symptomatic / asymptomatic or symptomatic conclusively)
hCoV-19/Saudi Arabia/KAUST-Makkah12/2020	EPI_ISL_437465	23/03/2020	Asia / Saudi Arabia / Makkah	Human	Female	50	Hospitalized	Original			A	S	Saudi Arabia	Undertermined	No (couldn't determine the patient status as either pre-symptomatic / asymptomatic or symptomatic conclusively)
hCoV-19/Saudi Arabia/KAUST-Makkah13/2020	EPI_ISL_437466	23/03/2020	Asia / Saudi Arabia / Makkah	Human	Male	56	Hospitalized	Original			A	S	Saudi Arabia	Undertermined	No (couldn't determine the patient status as either pre-symptomatic / asymptomatic or symptomatic conclusively)
hCoV-19/Saudi Arabia/KAUST-Makkah18/2020	EPI_ISL_437467	23/03/2020	Asia / Saudi Arabia / Makkah	Human	Female	75	Hospitalized	Original			A	S	Saudi Arabia	Undertermined	No (couldn't determine the patient status as either pre-symptomatic / asymptomatic or symptomatic conclusively)
hCoV-19/Saudi Arabia/KAUST-Makkah20/2020	EPI_ISL_437468	22/03/2020	Asia / Saudi Arabia / Makkah	Human	Female	15	Hospitalized	Original			B.1.9	GH	Saudi Arabia	Undertermined	No (couldn't determine the patient status as either pre-symptomatic / asymptomatic or symptomatic conclusively)
hCoV-19/Saudi Arabia/KAUST-Madinah25/2020	EPI_ISL_437469	29/03/2020	Asia / Saudi Arabia / Madinah	Human	Male	27	Hospitalized	Original			B.1.36	GH	Saudi Arabia	Undertermined	No (couldn't determine the patient status as either pre-symptomatic / asymptomatic or symptomatic conclusively)
hCoV-19/Saudi Arabia/KAUST-Madinah34/2020	EPI_ISL_437470	29/03/2020	Asia / Saudi Arabia / Madinah	Human	Female	13	Hospitalized	Original			B.1.36	GH	Saudi Arabia	Undertermined	No (couldn't determine the patient status as either pre-symptomatic / asymptomatic or symptomatic conclusively)
hCoV-19/Saudi Arabia/KAUST-Madinah38/2020	EPI_ISL_437471	29/03/2020	Asia / Saudi Arabia / Madinah	Human	Male	27	Hospitalized	Original			B.1.36	GH	Saudi Arabia	Undertermined	No (couldn't determine the patient status as either pre-symptomatic / asymptomatic or symptomatic conclusively)
hCoV-19/Saudi Arabia/KAUST-Madinah41/2020	EPI_ISL_437472	29/03/2020	Asia / Saudi Arabia / Madinah	Human	Female	17	Hospitalized	Original			B.1.36	O	Saudi Arabia	Undertermined	No (couldn't determine the patient status as either pre-symptomatic / asymptomatic or symptomatic conclusively)
hCoV-19/Saudi Arabia/KAUST-Madinah44/2020	EPI_ISL_437473	29/03/2020	Asia / Saudi Arabia / Madinah	Human	Male	63	Hospitalized	Original			B.1.36	GH	Saudi Arabia	Undertermined	No (couldn't determine the patient status as either pre-symptomatic / asymptomatic or symptomatic conclusively)
hCoV-19/Saudi Arabia/KAUST-Madinah45/2020	EPI_ISL_437474	29/03/2020	Asia / Saudi Arabia / Madinah	Human	Male	41	Hospitalized	Original			B.1.36	GH	Saudi Arabia	Undertermined	No (couldn't determine the patient status as either pre-symptomatic / asymptomatic or symptomatic conclusively)
hCoV-19/Saudi Arabia/KAUST-Jeddah64/2020	EPI_ISL_437475	01/04/2020	Asia / Saudi Arabia / Jeddah	Human	Female	41	Hospitalized	Original			A	S	Saudi Arabia	Undertermined	No (couldn't determine the patient status as either pre-symptomatic / asymptomatic or symptomatic conclusively)
hCoV-19/Saudi Arabia/KAUST-Makkah155/2020	EPI_ISL_437476	06/04/2020	Asia / Saudi Arabia / Makkah	Human	Male	32	Hospitalized	Original			B.1.36	G	Saudi Arabia	Undertermined	No (couldn't determine the patient status as either pre-symptomatic / asymptomatic or symptomatic conclusively)
hCoV-19/Saudi Arabia/KAUST-Makkah159/2020	EPI_ISL_437477	06/04/2020	Asia / Saudi Arabia / Makkah	Human	Male	29	Hospitalized	Original			B.1.36	GH	Saudi Arabia	Undertermined	No (couldn't determine the patient status as either pre-symptomatic / asymptomatic or symptomatic conclusively)
hCoV-19/Saudi Arabia/KAUST-Makkah165/2020	EPI_ISL_437478	06/04/2020	Asia / Saudi Arabia / Makkah	Human	Male	45	Hospitalized	Original			B.1.36	GH	Saudi Arabia	Undertermined	No (couldn't determine the patient status as either pre-symptomatic / asymptomatic or symptomatic conclusively)
hCoV-19/Saudi Arabia/KAUST-Makkah168/2020	EPI_ISL_437479	06/04/2020	Asia / Saudi Arabia / Makkah	Human	Female	50	Hospitalized	Original			B.1.36	GH	Saudi Arabia	Undertermined	No (couldn't determine the patient status as either pre-symptomatic / asymptomatic or symptomatic conclusively)
hCoV-19/Saudi Arabia/KAUST-Makkah19/2020	EPI_ISL_437481	18/03/2020	Asia / Saudi Arabia / Makkah	Human	Male	73	Hospitalized	Original			B.1.36	GH	Saudi Arabia	Undertermined	No (couldn't determine the patient status as either pre-symptomatic / asymptomatic or symptomatic conclusively)
hCoV-19/Saudi Arabia/KAUST-Madinah23/2020	EPI_ISL_437482	20/03/2020	Asia / Saudi Arabia / Madinah	Human	Male	32	Hospitalized	Original			B.1.36	GH	Saudi Arabia	Undertermined	No (couldn't determine the patient status as either pre-symptomatic / asymptomatic or symptomatic conclusively)
hCoV-19/Saudi Arabia/KAUST-Madinah24/2020	EPI_ISL_437483	20/03/2020	Asia / Saudi Arabia / Madinah	Human	Male	60	Hospitalized	Original			B.1.36	GH	Saudi Arabia	Undertermined	No (couldn't determine the patient status as either pre-symptomatic / asymptomatic or symptomatic conclusively)
hCoV-19/Saudi Arabia/KAUST-Madinah27/2020	EPI_ISL_437484	29/03/2020	Asia / Saudi Arabia / Madinah	Human	Female	29	Hospitalized	Original			A	S	Saudi Arabia	Undertermined	No (couldn't determine the patient status as either pre-symptomatic / asymptomatic or symptomatic conclusively)
hCoV-19/Saudi Arabia/KAUST-Madinah30/2020	EPI_ISL_437485	26/03/2020	Asia / Saudi Arabia / Madinah	Human	Male	50	Hospitalized	Original			B.1.36	GH	Saudi Arabia	Undertermined	No (couldn't determine the patient status as either pre-symptomatic / asymptomatic or symptomatic conclusively)
hCoV-19/Saudi Arabia/KAUST-Madinah31/2020	EPI_ISL_437486	26/03/2020	Asia / Saudi Arabia / Madinah	Human	Male	39	Hospitalized	Original			B.1.36	GH	Saudi Arabia	Undertermined	No (couldn't determine the patient status as either pre-symptomatic / asymptomatic or symptomatic conclusively)
hCoV-19/Saudi Arabia/KAUST-Madinah47/2020	EPI_ISL_437487	26/03/2020	Asia / Saudi Arabia / Madinah	Human	Male	28	Hospitalized	Original			B.1.36	GH	Saudi Arabia	Undertermined	No (couldn't determine the patient status as either pre-symptomatic / asymptomatic or symptomatic conclusively)
hCoV-19/Saudi Arabia/KAUST-Madinah48/2020	EPI_ISL_437488	26/03/2020	Asia / Saudi Arabia / Madinah	Human	male	26	Hospitalized	Original			B.1.36	GH	Saudi Arabia	Undertermined	No (couldn't determine the patient status as either pre-symptomatic / asymptomatic or symptomatic conclusively)
hCoV-19/Saudi Arabia/KAUST-Madinah49/2020	EPI_ISL_437489	26/03/2020	Asia / Saudi Arabia / Madinah	Human	Female	59	Hospitalized	Original			B.1.36	GH	Saudi Arabia	Undertermined	No (couldn't determine the patient status as either pre-symptomatic / asymptomatic or symptomatic conclusively)
hCoV-19/Saudi Arabia/KAUST-Madinah50/2020	EPI_ISL_437490	26/03/2020	Asia / Saudi Arabia / Madinah	Human	Male	61	Hospitalized	Original			B.1.36	GH	Saudi Arabia	Undertermined	No (couldn't determine the patient status as either pre-symptomatic / asymptomatic or symptomatic conclusively)
hCoV-19/Saudi Arabia/KAUST-Madinah51/2020	EPI_ISL_437491	26/03/2020	Asia / Saudi Arabia / Madinah	Human	Male	59	Hospitalized	Original			B.1.36	O	Saudi Arabia	Undertermined	No (couldn't determine the patient status as either pre-symptomatic / asymptomatic or symptomatic conclusively)
hCoV-19/Saudi Arabia/KAUST-Madinah53/2020	EPI_ISL_437492	26/03/2020	Asia / Saudi Arabia / Madinah	Human	Male	46	Hospitalized	Original			B.1.36	GH	Saudi Arabia	Undertermined	No (couldn't determine the patient status as either pre-symptomatic / asymptomatic or symptomatic conclusively)
hCoV-19/Saudi Arabia/KAUST-Madinah54/2020	EPI_ISL_437493	26/03/2020	Asia / Saudi Arabia / Madinah	Human	Male	41	Hospitalized	Original			B.1.36	GH	Saudi Arabia	Undertermined	No (couldn't determine the patient status as either pre-symptomatic / asymptomatic or symptomatic conclusively)
hCoV-19/Saudi Arabia/KAUST-Madinah55/2020	EPI_ISL_437494	26/03/2020	Asia / Saudi Arabia / Madinah	Human	Male	50	Hospitalized	Original			B.1.36	GH	Saudi Arabia	Undertermined	No (couldn't determine the patient status as either pre-symptomatic / asymptomatic or symptomatic conclusively)
hCoV-19/Saudi Arabia/KAUST-Madinah57/2020	EPI_ISL_437495	26/03/2020	Asia / Saudi Arabia / Madinah	Human	unknown	unknown	Live	Original			A	S	Saudi Arabia	Undertermined	No (couldn't determine the patient status as either pre-symptomatic / asymptomatic or symptomatic conclusively)
hCoV-19/Saudi Arabia/KAUST-Jeddah59/2020	EPI_ISL_437496	25/03/2020	Asia / Saudi Arabia / Jeddah	Human	Male	29	Hospitalized	Original			B.1.36	GH	Saudi Arabia	Undertermined	No (couldn't determine the patient status as either pre-symptomatic / asymptomatic or symptomatic conclusively)
hCoV-19/Saudi Arabia/KAUST-Jeddah60/2020	EPI_ISL_437497	25/03/2020	Asia / Saudi Arabia / Jeddah	Human	Male	62	Hospitalized	Original			B.1.1	GR	Saudi Arabia	Undertermined	No (couldn't determine the patient status as either pre-symptomatic / asymptomatic or symptomatic conclusively)
hCoV-19/France/IDF4791/2020	EPI_ISL_443258	02/04/2020	Europe / France / Ile de France / Paris	Human	Female	95	EHPAD_IRA	Original	Naso-pharyngeal swab		B.1	GH	France	Undertermined	No (couldn't determine the patient status as either pre-symptomatic / asymptomatic or symptomatic conclusively)
hCoV-19/France/IDF4793/2020	EPI_ISL_443259	02/04/2020	Europe / France / Ile de France / Paris	Human	Female	67	EHPAD_IRA	Original	Naso-pharyngeal swab		B.1	GH	France	Undertermined	No (couldn't determine the patient status as either pre-symptomatic / asymptomatic or symptomatic conclusively)
hCoV-19/France/IDF4843/2020	EPI_ISL_443260	02/04/2020	Europe / France / Ile de France / Longjumeau	Human	Male	57	hospitalized	Original	Naso-pharyngeal swab		B.1	GH	France	Undertermined	No (couldn't determine the patient status as either pre-symptomatic / asymptomatic or symptomatic conclusively)
hCoV-19/France/BFC5011/2020	EPI_ISL_443261	03/04/2020	Europe / France / Bourgogne / Dijon	Human	Female	42	hospitalized	Original	Naso-pharyngeal swab		B.1	G	France	Undertermined	No (couldn't determine the patient status as either pre-symptomatic / asymptomatic or symptomatic conclusively)
hCoV-19/France/BFC5012/2020	EPI_ISL_443262	02/04/2020	Europe / France / Bourgogne / Dijon	Human	Male	49	hospitalized	Original	Naso-pharyngeal swab		B.1	G	France	Undertermined	No (couldn't determine the patient status as either pre-symptomatic / asymptomatic or symptomatic conclusively)
hCoV-19/France/BFC5017/2020	EPI_ISL_443263	02/04/2020	Europe / France / Bourgogne / Dijon	Human	Male	52	hospitalized	Original	Naso-pharyngeal swab		B.1	GH	France	Undertermined	No (couldn't determine the patient status as either pre-symptomatic / asymptomatic or symptomatic conclusively)
hCoV-19/France/BFC5019/2020	EPI_ISL_443264	02/04/2020	Europe / France / Bourgogne / Dijon	Human	Female	27	hospitalized	Original	Naso-pharyngeal swab		B.1	GH	France	Undertermined	No (couldn't determine the patient status as either pre-symptomatic / asymptomatic or symptomatic conclusively)
hCoV-19/France/B5310/2020	EPI_ISL_443265	03/04/2020	Europe / France / Bretagne / Brest	Human	Male	57	hospitalized	Original	Naso-pharyngeal swab		B.1	G	France	Undertermined	No (couldn't determine the patient status as either pre-symptomatic / asymptomatic or symptomatic conclusively)
hCoV-19/France/B5322/2020	EPI_ISL_443266	16/03/2020	Europe / France / Bretagne / Brest	Human	Male	63	hospitalized	Original	Naso-pharyngeal swab		B.1	GH	France	Undertermined	No (couldn't determine the patient status as either pre-symptomatic / asymptomatic or symptomatic conclusively)
hCoV-19/France/B5325/2020	EPI_ISL_443267	18/03/2020	Europe / France / Bretagne / Brest	Human	Female	25	hospitalized	Original	Naso-pharyngeal swab		B.1.1	GR	France	Undertermined	No (couldn't determine the patient status as either pre-symptomatic / asymptomatic or symptomatic conclusively)
hCoV-19/France/B5336/2020	EPI_ISL_443268	18/03/2020	Europe / France / Bretagne / Brest	Human	Male	40	hospitalized	Original	Naso-pharyngeal swab		B.1	GH	France	Undertermined	No (couldn't determine the patient status as either pre-symptomatic / asymptomatic or symptomatic conclusively)
hCoV-19/France/B5344/2020	EPI_ISL_443269	21/03/2020	Europe / France / Bretagne / Brest	Human	Male	67	hospitalized	Original	Naso-pharyngeal swab		B.1	GH	France	Undertermined	No (couldn't determine the patient status as either pre-symptomatic / asymptomatic or symptomatic conclusively)
hCoV-19/France/B5353/2020	EPI_ISL_443270	23/03/2020	Europe / France / Bretagne / Brest	Human	Female	53	hospitalized	Original	Naso-pharyngeal swab		B.1	G	France	Undertermined	No (couldn't determine the patient status as either pre-symptomatic / asymptomatic or symptomatic conclusively)
hCoV-19/France/B5355/2020	EPI_ISL_443271	24/03/2020	Europe / France / Bretagne / Brest	Human	Male	25	hospitalized	Original	Naso-pharyngeal swab		B.1.1	GR	France	Undertermined	No (couldn't determine the patient status as either pre-symptomatic / asymptomatic or symptomatic conclusively)
hCoV-19/France/B5359/2020	EPI_ISL_443272	24/03/2020	Europe / France / Bretagne / Brest	Human	Female	78	hospitalized	Original	Naso-pharyngeal swab		B.1	GH	France	Undertermined	No (couldn't determine the patient status as either pre-symptomatic / asymptomatic or symptomatic conclusively)
hCoV-19/France/B5363/2020	EPI_ISL_443273	24/03/2020	Europe / France / Bretagne / Brest	Human	Female	89	hospitalized	Original	Naso-pharyngeal swab		B.1	GH	France	Undertermined	No (couldn't determine the patient status as either pre-symptomatic / asymptomatic or symptomatic conclusively)
hCoV-19/France/B5374/2020	EPI_ISL_443274	25/03/2020	Europe / France / Bretagne / Brest	Human	Female	52	hospitalized	Original	Naso-pharyngeal swab		B.1.1	GR	France	Undertermined	No (couldn't determine the patient status as either pre-symptomatic / asymptomatic or symptomatic conclusively)
hCoV-19/France/B5397/2020	EPI_ISL_443275	27/03/2020	Europe / France / Bretagne / Brest	Human	Female	82	hospitalized	Original	Naso-pharyngeal swab		B.1.1	GR	France	Undertermined	No (couldn't determine the patient status as either pre-symptomatic / asymptomatic or symptomatic conclusively)
hCoV-19/France/B5400/2020	EPI_ISL_443276	27/03/2020	Europe / France / Bretagne / Brest	Human	Female	40	hospitalized	Original	Naso-pharyngeal swab		B.1	GH	France	Undertermined	No (couldn't determine the patient status as either pre-symptomatic / asymptomatic or symptomatic conclusively)
hCoV-19/France/B5413/2020	EPI_ISL_443277	28/03/2020	Europe / France / Bretagne / Brest	Human	Female	94	hospitalized	Original	Naso-pharyngeal swab		B.1	GH	France	Undertermined	No (couldn't determine the patient status as either pre-symptomatic / asymptomatic or symptomatic conclusively)
hCoV-19/France/B5415/2020	EPI_ISL_443278	29/03/2020	Europe / France / Bretagne / Brest	Human	Female	77	hospitalized	Original	Naso-pharyngeal swab		B.1.1	GR	France	Undertermined	No (couldn't determine the patient status as either pre-symptomatic / asymptomatic or symptomatic conclusively)
hCoV-19/France/B5434/2020	EPI_ISL_443279	01/04/2020	Europe / France / Bretagne / Brest	Human	Female	33	hospitalized	Original	Naso-pharyngeal swab		B.1	GH	France	Undertermined	No (couldn't determine the patient status as either pre-symptomatic / asymptomatic or symptomatic conclusively)
hCoV-19/France/B5447/2020	EPI_ISL_443280	01/04/2020	Europe / France / Bretagne / Brest	Human	Female	25	hospitalized	Original	Naso-pharyngeal swab		B.1.1	GR	France	Undertermined	No (couldn't determine the patient status as either pre-symptomatic / asymptomatic or symptomatic conclusively)
hCoV-19/France/B5456/2020	EPI_ISL_443281	03/04/2020	Europe / France / Bretagne / Brest	Human	Female	25	hospitalized	Original	Naso-pharyngeal swab		B.1	GH	France	Undertermined	No (couldn't determine the patient status as either pre-symptomatic / asymptomatic or symptomatic conclusively)
hCoV-19/France/B5458/2020	EPI_ISL_443282	22/03/2020	Europe / France / Bretagne / Brest	Human	Female	80	hospitalized	Original	Naso-pharyngeal swab		B.1.1	GR	France	Undertermined	No (couldn't determine the patient status as either pre-symptomatic / asymptomatic or symptomatic conclusively)
hCoV-19/France/B5465/2020	EPI_ISL_443283	23/03/2020	Europe / France / Bretagne / Brest	Human	Male	92	hospitalized	Original	Naso-pharyngeal swab		B.1	GH	France	Undertermined	No (couldn't determine the patient status as either pre-symptomatic / asymptomatic or symptomatic conclusively)
hCoV-19/France/IDF5650/2020	EPI_ISL_443284	02/04/2020	Europe / France / Hauts de France / Pontoise	Human	Female	91	hospitalized	Original	unknown		B.1	GH	France	Undertermined	No (couldn't determine the patient status as either pre-symptomatic / asymptomatic or symptomatic conclusively)
hCoV-19/France/IDF5655/2020	EPI_ISL_443285	02/04/2020	Europe / France / Hauts de France / Pontoise	Human	Female	31	hospitalized	Original	unknown		B.1	GH	France	Undertermined	No (couldn't determine the patient status as either pre-symptomatic / asymptomatic or symptomatic conclusively)
hCoV-19/France/IDF5657/2020	EPI_ISL_443286	02/04/2020	Europe / France / Hauts de France / Pontoise	Human	Male	77	hospitalized	Original	unknown		B.1	G	France	Undertermined	No (couldn't determine the patient status as either pre-symptomatic / asymptomatic or symptomatic conclusively)
hCoV-19/France/IDF5662/2020	EPI_ISL_443287	03/04/2020	Europe / France / Hauts de France / Pontoise	Human	Female	29	hospitalized	Original	unknown		B.1	GH	France	Undertermined	No (couldn't determine the patient status as either pre-symptomatic / asymptomatic or symptomatic conclusively)
hCoV-19/France/IDF5666/2020	EPI_ISL_443288	03/04/2020	Europe / France / Hauts de France / Pontoise	Human	Female	82	hospitalized	Original	unknown		B.1	G	France	Undertermined	No (couldn't determine the patient status as either pre-symptomatic / asymptomatic or symptomatic conclusively)
hCoV-19/France/B5678/2020	EPI_ISL_443289	27/03/2020	Europe / France / Bretagne / Rennes	Human	Female	35	hospitalized	Original	sputum		B.1	GH	France	Undertermined	No (couldn't determine the patient status as either pre-symptomatic / asymptomatic or symptomatic conclusively)
hCoV-19/France/B5681/2020	EPI_ISL_443290	31/03/2020	Europe / France / Bretagne / Rennes	Human	Female	84	hospitalized	Original	Naso-pharyngeal swab		B.1	GH	France	Undertermined	No (couldn't determine the patient status as either pre-symptomatic / asymptomatic or symptomatic conclusively)
hCoV-19/France/B5685/2020	EPI_ISL_443291	02/04/2020	Europe / France / Bretagne / Rennes	Human	Female	86	hospitalized	Original	Naso-pharyngeal swab		B.1	G	France	Undertermined	No (couldn't determine the patient status as either pre-symptomatic / asymptomatic or symptomatic conclusively)
hCoV-19/France/B5687/2020	EPI_ISL_443292	03/04/2020	Europe / France / Bretagne / Rennes	Human	Female	19	hospitalized	Original	Naso-pharyngeal swab		B.1	GH	France	Undertermined	No (couldn't determine the patient status as either pre-symptomatic / asymptomatic or symptomatic conclusively)
hCoV-19/France/B5688/2020	EPI_ISL_443293	03/04/2020	Europe / France / Bretagne / Rennes	Human	unknown	77	hospitalized	Original	Unknown		B.1	G	France	Undertermined	No (couldn't determine the patient status as either pre-symptomatic / asymptomatic or symptomatic conclusively)
hCoV-19/France/B5689/2020	EPI_ISL_443294	06/04/2020	Europe / France / Bretagne / Rennes	Human	Female	23	hospitalized	Original	Naso-pharyngeal swab		B.1	G	France	Undertermined	No (couldn't determine the patient status as either pre-symptomatic / asymptomatic or symptomatic conclusively)
hCoV-19/France/IDF5847/2020	EPI_ISL_443295	19/03/2020	Europe / France / Ile de France / Paris	Human	Male	0.1	hospitalized	Original	unknown		B.1	GH	France	Undertermined	No (couldn't determine the patient status as either pre-symptomatic / asymptomatic or symptomatic conclusively)
hCoV-19/France/IDF5848/2020	EPI_ISL_443296	19/03/2020	Europe / France / Ile de France / Paris	Human	Male	0.2	hospitalized	Original	unknown		B.1	GH	France	Undertermined	No (couldn't determine the patient status as either pre-symptomatic / asymptomatic or symptomatic conclusively)
hCoV-19/France/IDF5850/2020	EPI_ISL_443297	26/03/2020	Europe / France / Ile de France / Paris	Human	Female	8	hospitalized	Original	unknown		B.1	GH	France	Undertermined	No (couldn't determine the patient status as either pre-symptomatic / asymptomatic or symptomatic conclusively)
hCoV-19/France/IDF5851/2020	EPI_ISL_443298	25/03/2020	Europe / France / Ile de France / Paris	Human	Male	9	hospitalized	Original	unknown		B.1	GH	France	Undertermined	No (couldn't determine the patient status as either pre-symptomatic / asymptomatic or symptomatic conclusively)
hCoV-19/France/IDF5853/2020	EPI_ISL_443299	25/03/2020	Europe / France / Ile de France / Paris	Human	Female	14	hospitalized	Original	unknown		B.1	GH	France	Undertermined	No (couldn't determine the patient status as either pre-symptomatic / asymptomatic or symptomatic conclusively)
hCoV-19/France/IDF3081/2020	EPI_ISL_443300	17/03/2020	Europe / France / Ile de France / Pecq	Human	Male	77	Physician network	Original	Naso-pharyngeal swab		B.1	GH	France	Undertermined	No (couldn't determine the patient status as either pre-symptomatic / asymptomatic or symptomatic conclusively)
hCoV-19/France/IDF3359/2020	EPI_ISL_443301	23/03/2020	Europe / France / Ile de France / Guyancourt	Human	Female	55	Physician network	Original	Naso-pharyngeal swab		B.1	G	France	Undertermined	No (couldn't determine the patient status as either pre-symptomatic / asymptomatic or symptomatic conclusively)
hCoV-19/France/IDF3368/2020	EPI_ISL_443302	23/03/2020	Europe / France / Ile de France / Cergy	Human	Male	66	Physician network	Original	Naso-pharyngeal swab		B.1	GH	France	Undertermined	No (couldn't determine the patient status as either pre-symptomatic / asymptomatic or symptomatic conclusively)
hCoV-19/France/IDF5577/2020	EPI_ISL_443303	09/04/2020	Europe / France / Ile de France / Asnieres sur Seine	Human	Female	29	EHPAD	Original	Naso-pharyngeal swab		B.1	GH	France	Undertermined	No (couldn't determine the patient status as either pre-symptomatic / asymptomatic or symptomatic conclusively)
hCoV-19/France/IDF5778/2020	EPI_ISL_443304	01/04/2020	Europe / France / Ile de France / Colombes	Human	Female	78	EHPAD	Original	Naso-pharyngeal swab		B.1	GH	France	Undertermined	No (couldn't determine the patient status as either pre-symptomatic / asymptomatic or symptomatic conclusively)
hCoV-19/France/IDF5986/2020	EPI_ISL_443305	13/04/2020	Europe / France / Ile de France / Longjumeau	Human	Male	25	hospitalized	Original	Naso-pharyngeal swab		B.1	G	France	Undertermined	No (couldn't determine the patient status as either pre-symptomatic / asymptomatic or symptomatic conclusively)
hCoV-19/France/IDF6037/2020	EPI_ISL_443306	02/04/2020	Europe / France / Ile de France / Moussy le Neuf	Human	Male	20	Physician network	Original	Naso-pharyngeal swab		B.1	GH	France	Undertermined	No (couldn't determine the patient status as either pre-symptomatic / asymptomatic or symptomatic conclusively)
hCoV-19/France/BFC6053/2020	EPI_ISL_443307	09/04/2020	Europe / France / Bourgogne Franche comte / Chalon sur Saone	Human	Female	90	EHPAD	Original	Naso-pharyngeal swab		B.1	G	France	Undertermined	No (couldn't determine the patient status as either pre-symptomatic / asymptomatic or symptomatic conclusively)
hCoV-19/France/GE6058/2020	EPI_ISL_443308	15/04/2020	Europe / France / Grand Est / Nancy	Human	Female	95	EHPAD	Original	Naso-pharyngeal swab		B.1	G	France	Undertermined	No (couldn't determine the patient status as either pre-symptomatic / asymptomatic or symptomatic conclusively)
hCoV-19/France/HF3669/2020	EPI_ISL_443309	25/03/2020	Europe / France / Hauts de France / Compiegne	Human	Female	76	hospitalized	Original	Naso-pharyngeal swab		B.1	GH	France	Undertermined	No (couldn't determine the patient status as either pre-symptomatic / asymptomatic or symptomatic conclusively)
hCoV-19/France/GE4420/2020	EPI_ISL_443310	20/03/2020	Europe / France / Grand-Est / Ham sous Varsberg	Human	Female	76	Physician network	Original	Naso-pharyngeal swab		B.1	G	France	Undertermined	No (couldn't determine the patient status as either pre-symptomatic / asymptomatic or symptomatic conclusively)
hCoV-19/France/IDF4423/2020	EPI_ISL_443311	23/03/2020	Europe / France / Ile de France / Montigny le Bretonneux	Human	Female	62	Physician network	Original	Naso-pharyngeal swab		B.1	GH	France	Undertermined	No (couldn't determine the patient status as either pre-symptomatic / asymptomatic or symptomatic conclusively)
hCoV-19/France/CVL4430/2020	EPI_ISL_443312	24/03/2020	Europe / France / Centre - Val de Loire / Abondant	Human	Male	71	Physician network	Original	Naso-pharyngeal swab		B.1	GH	France	Undertermined	No (couldn't determine the patient status as either pre-symptomatic / asymptomatic or symptomatic conclusively)
hCoV-19/France/B6041/2020	EPI_ISL_443313	05/03/2020	Europe / France / Bretagne / Ploudalmezeau	Human	Male	57	Physician network	Original	Naso-pharyngeal swab		B.1.5	G	France	Undertermined	No (couldn't determine the patient status as either pre-symptomatic / asymptomatic or symptomatic conclusively)
hCoV-19/France/IDF6073/2020	EPI_ISL_443314	14/04/2020	Europe / France / Ile de France / Longjumeau	Human	Female	85	hospitalized	Original	Naso-pharyngeal swab		B.1	GH	France	Undertermined	No (couldn't determine the patient status as either pre-symptomatic / asymptomatic or symptomatic conclusively)
hCoV-19/France/HF6466/2020	EPI_ISL_443315	02/04/2020	Europe / France / Hauts de France / Nogent l'Artaud	Human	Male	80	EHPAD	Original	Naso-pharyngeal swab		B.1	GH	France	Undertermined	No (couldn't determine the patient status as either pre-symptomatic / asymptomatic or symptomatic conclusively)
hCoV-19/France/HF3668/2020	EPI_ISL_443316	25/03/2020	Europe / France / Hauts de France / Compiegne	Human	Female	76	hospitalized	Original	unknown		B.1	GH	France	Undertermined	No (couldn't determine the patient status as either pre-symptomatic / asymptomatic or symptomatic conclusively)
hCoV-19/France/IDF4432/2020	EPI_ISL_443317	23/03/2020	Europe / France / Ile de France / Cergy	Human	Female	34	Physician network	Original			B.1	GH	France	Undertermined	No (couldn't determine the patient status as either pre-symptomatic / asymptomatic or symptomatic conclusively)
hCoV-19/India/GJ-GBRC26/2020	EPI_ISL_444457	26/04/2020	Asia / India / Gujarat / Ahmedabad	Human	Male	40	Released	Original	Oro-pharyngeal swab		B.1.36	GH	India	Undertermined	No (couldn't determine the patient status as either pre-symptomatic / asymptomatic or symptomatic conclusively)
hCoV-19/India/GJ-GBRC27/2020	EPI_ISL_444458	26/04/2020	Asia / India / Gujarat / Ahmedabad	Human	Female	35	Released	Original	Oro-pharyngeal swab		B.1	G	India	Undertermined	No (couldn't determine the patient status as either pre-symptomatic / asymptomatic or symptomatic conclusively)
hCoV-19/India/GJ-GBRC28/2020	EPI_ISL_444459	30/04/2020	Asia / India / Gujarat / Ahmedabad	Human	Male	48	Released	Original	Oro-pharyngeal swab		B.1.113	GH	India	Undertermined	No (couldn't determine the patient status as either pre-symptomatic / asymptomatic or symptomatic conclusively)
hCoV-19/India/GJ-GBRC29/2020	EPI_ISL_444460	30/04/2020	Asia / India / Gujarat / Ahmedabad	Human	Male	37	Released	Original	Oro-pharyngeal swab		B.1	GH	India	Undertermined	No (couldn't determine the patient status as either pre-symptomatic / asymptomatic or symptomatic conclusively)
hCoV-19/India/GJ-GBRC31/2020	EPI_ISL_444462	29/04/2020	Asia / India / Gujarat / Ahmedabad	Human	Female	28	Released	Original	Oro-pharyngeal swab		B.1	G	India	Undertermined	No (couldn't determine the patient status as either pre-symptomatic / asymptomatic or symptomatic conclusively)
hCoV-19/India/GJ-GBRC34/2020	EPI_ISL_444465	29/04/2020	Asia / India / Gujarat / Ahmedabad	Human	Female	53	Released	Original	Oro-pharyngeal swab		B.1.113	GH	India	Undertermined	No (couldn't determine the patient status as either pre-symptomatic / asymptomatic or symptomatic conclusively)
hCoV-19/India/GJ-GBRC35/2020	EPI_ISL_444466	29/04/2020	Asia / India / Gujarat / Ahmedabad	Human	Male	35	Released	Original	Oro-pharyngeal swab		B.1.113	GH	India	Undertermined	No (couldn't determine the patient status as either pre-symptomatic / asymptomatic or symptomatic conclusively)
hCoV-19/India/GJ-GBRC37/2020	EPI_ISL_444468	29/04/2020	Asia / India / Gujarat / Ahmedabad	Human	Female	60	Released	Original	Oro-pharyngeal swab		B.1	G	India	Undertermined	No (couldn't determine the patient status as either pre-symptomatic / asymptomatic or symptomatic conclusively)
hCoV-19/India/GJ-GBRC38/2020	EPI_ISL_444469	29/04/2020	Asia / India / Gujarat / Ahmedabad	Human	Male	37	Released	Original	Oro-pharyngeal swab		B.1.113	GH	India	Undertermined	No (couldn't determine the patient status as either pre-symptomatic / asymptomatic or symptomatic conclusively)
hCoV-19/India/GJ-GBRC40/2020	EPI_ISL_444471	29/04/2020	Asia / India / Gujarat / Ahmedabad	Human	Female	34	Released	Original	Oro-pharyngeal swab		B.1.36	GH	India	Undertermined	No (couldn't determine the patient status as either pre-symptomatic / asymptomatic or symptomatic conclusively)
hCoV-19/India/GJ-GBRC43/2020	EPI_ISL_444474	29/04/2020	Asia / India / Gujarat / Ahmedabad	Human	Female	52	Released	Original	Oro-pharyngeal swab		B.1.36	GH	India	Undertermined	No (couldn't determine the patient status as either pre-symptomatic / asymptomatic or symptomatic conclusively)
hCoV-19/India/GJ-GBRC44/2020	EPI_ISL_444475	29/04/2020	Asia / India / Gujarat / Ahmedabad	Human	Male	20	Released	Original	Oro-pharyngeal swab		B.1	G	India	Undertermined	No (couldn't determine the patient status as either pre-symptomatic / asymptomatic or symptomatic conclusively)
hCoV-19/India/GJ-GBRC45/2020	EPI_ISL_444476	29/04/2020	Asia / India / Gujarat / Ahmedabad	Human	Female	24	Released	Original	Oro-pharyngeal swab		B.1.113	GH	India	Undertermined	No (couldn't determine the patient status as either pre-symptomatic / asymptomatic or symptomatic conclusively)
hCoV-19/India/GJ-GBRC46/2020	EPI_ISL_444477	29/04/2020	Asia / India / Gujarat / Ahmedabad	Human	Male	60	Deceased	Original	Oro-pharyngeal swab		B.1	G	India	Symptomatic	Yes
hCoV-19/India/GJ-GBRC49/2020	EPI_ISL_444480	30/04/2020	Asia / India / Gujarat / Ahmedabad	Human	Male	60	Deceased	Original	Oro-pharyngeal swab		B.1.36	GH	India	Symptomatic	Yes
hCoV-19/India/GJ-GBRC50/2020	EPI_ISL_444481	30/04/2020	Asia / India / Gujarat / Ahmedabad	Human	Female	55	Released	Original	Oro-pharyngeal swab		B.1	G	India	Undertermined	No (couldn't determine the patient status as either pre-symptomatic / asymptomatic or symptomatic conclusively)
hCoV-19/India/GJ-GBRC51/2020	EPI_ISL_444482	02/05/2020	Asia / India / Gujarat / Prantij	Human	Male	18	Released	Original	Oro-pharyngeal swab		B.1.36	GH	India	Undertermined	No (couldn't determine the patient status as either pre-symptomatic / asymptomatic or symptomatic conclusively)
hCoV-19/India/GJ-GBRC52/2020	EPI_ISL_444483	02/05/2020	Asia / India / Gujarat / Prantij	Human	Male	30	Deceased	Original	Oro-pharyngeal swab		B.1	G	India	Symptomatic	Yes
hCoV-19/India/GJ-GBRC53/2020	EPI_ISL_444484	04/05/2020	Asia / India / Gujarat / Modasa	Human	Male	28	Released	Original	Oro-pharyngeal swab		B.1.36	GH	India	Undertermined	No (couldn't determine the patient status as either pre-symptomatic / asymptomatic or symptomatic conclusively)
hCoV-19/India/GJ-GBRC55/2020	EPI_ISL_444486	04/05/2020	Asia / India / Gujarat / Dhansura	Human	Male	55	Released	Original	Oro-pharyngeal swab		B.1.113	GH	India	Undertermined	No (couldn't determine the patient status as either pre-symptomatic / asymptomatic or symptomatic conclusively)
hCoV-19/Sweden/KI-CTMR-V300056977-1H/2020	EPI_ISL_444488	2020-03	Europe / Sweden / Stockholm	Human	unknown	unkown	unkown	Original			B.1	GH	Sweden	Undertermined	No (couldn't determine the patient status as either pre-symptomatic / asymptomatic or symptomatic conclusively)
hCoV-19/Sweden/KI-CTMR-V300057201-8E/2020	EPI_ISL_444489	2020-03	Europe / Sweden / Stockholm	Human	unknown	unkown	unkown	Original			B.1	GH	Sweden	Undertermined	No (couldn't determine the patient status as either pre-symptomatic / asymptomatic or symptomatic conclusively)
hCoV-19/Sweden/KI-CTMR-V300037768-4D/2020	EPI_ISL_444490	2020-03	Europe / Sweden / Stockholm	Human	unknown	unkown	unkown	Original			B.1	GH	Sweden	Undertermined	No (couldn't determine the patient status as either pre-symptomatic / asymptomatic or symptomatic conclusively)
hCoV-19/Sweden/KI-CTMR-V300037768-8A/2020	EPI_ISL_444491	2020-03	Europe / Sweden / Stockholm	Human	unknown	unkown	unkown	Original			B.1	GH	Sweden	Undertermined	No (couldn't determine the patient status as either pre-symptomatic / asymptomatic or symptomatic conclusively)
hCoV-19/Sweden/KI-CTMR-V300056977-4A/2020	EPI_ISL_444492	2020-03	Europe / Sweden / Stockholm	Human	unknown	unkown	unkown	Original			B.1.1	GR	Sweden	Undertermined	No (couldn't determine the patient status as either pre-symptomatic / asymptomatic or symptomatic conclusively)
hCoV-19/Uruguay/Mdeo-1/2020	EPI_ISL_444493	13/03/2020	South America / Uruguay / Montevideo	Human	Female	unknown	Live	Original	Oro-pharyngeal swab		A.5	S	Uruguay	Undertermined	No (couldn't determine the patient status as either pre-symptomatic / asymptomatic or symptomatic conclusively)
hCoV-19/India/MH-CCMB-NIV1/2020	EPI_ISL_450321	04/04/2020	Asia / India / Maharashtra / Pune	Human	Male	57	Hospitalized	Original	Oro-pharyngeal swab		B.6	O	India	Undertermined	No (couldn't determine the patient status as either pre-symptomatic / asymptomatic or symptomatic conclusively)
hCoV-19/India/MH-CCMB-NIV2/2020	EPI_ISL_450322	04/04/2020	Asia / India / Maharashtra / Ahmednagar	Human	Male	35	Hospitalized	Original	Oro-pharyngeal swab		B.6	O	India	Undertermined	No (couldn't determine the patient status as either pre-symptomatic / asymptomatic or symptomatic conclusively)
hCoV-19/India/MH-CCMB-NIV3/2020	EPI_ISL_450323	27/03/2020	Asia / India / Maharashtra / Kolhapur	Human	Female	44	Hospitalized	Original	Oro-pharyngeal swab		B.1	G	India	Undertermined	No (couldn't determine the patient status as either pre-symptomatic / asymptomatic or symptomatic conclusively)
hCoV-19/India/MH-CCMB-NIV4/2020	EPI_ISL_450324	26/03/2020	Asia / India / Maharashtra / Sangli	Human	Female	65	Hospitalized	Original	Oro-pharyngeal swab		A	S	India	Undertermined	No (couldn't determine the patient status as either pre-symptomatic / asymptomatic or symptomatic conclusively)
hCoV-19/India/MH-CCMB-NIV5/2020	EPI_ISL_450325	17/03/2020	Asia / India / Maharashtra / Mumbai	Human	Male	37	Hospitalized	Original	Oro-pharyngeal swab		B.1	G	India	Undertermined	No (couldn't determine the patient status as either pre-symptomatic / asymptomatic or symptomatic conclusively)
hCoV-19/Poland/Pom3/2020	EPI_ISL_451644	02/05/2020	Europe / Poland / Pomorze	Human	Male	88	Hospitalized	Original			B.1.1.1	GR	Poland	Undertermined	No (couldn't determine the patient status as either pre-symptomatic / asymptomatic or symptomatic conclusively)
hCoV-19/Poland/Pom4/2020	EPI_ISL_451645	02/05/2020	Europe / Poland / Pomorze	Human	Male	94	Hospitalized	Original			B.1.1.1	GR	Poland	Undertermined	No (couldn't determine the patient status as either pre-symptomatic / asymptomatic or symptomatic conclusively)
hCoV-19/Poland/Pom5/2020	EPI_ISL_451646	22/04/2020	Europe / Poland / Pomorze	Human	Woman	69	Hospitalized	Original			B.1.1.1	GR	Poland	Undertermined	No (couldn't determine the patient status as either pre-symptomatic / asymptomatic or symptomatic conclusively)
hCoV-19/Poland/Pom6/2020	EPI_ISL_451647	23/04/2020	Europe / Poland / Pomorze	Human	Male	33	Quarantine	Original			B.1.1.1	GR	Poland	Undertermined	No (couldn't determine the patient status as either pre-symptomatic / asymptomatic or symptomatic conclusively)
hCoV-19/Poland/Sla1/2020	EPI_ISL_451655	05/05/2020	Europe / Poland / Slask	Human	Male	unknown	Quarantine	Original		Active miner	B.1.1	GR	Poland	Undertermined	No (couldn't determine the patient status as either pre-symptomatic / asymptomatic or symptomatic conclusively)
hCoV-19/Poland/Sla2/2020	EPI_ISL_451656	05/05/2020	Europe / Poland / Slask	Human	Male	unknown	Quarantine	Original		Active miner	B.1.1	GR	Poland	Undertermined	No (couldn't determine the patient status as either pre-symptomatic / asymptomatic or symptomatic conclusively)
hCoV-19/Poland/Sla3/2020	EPI_ISL_451657	05/05/2020	Europe / Poland / Slask	Human	Male	unknown	Quarantine	Original		Active miner	B.1.1	GR	Poland	Undertermined	No (couldn't determine the patient status as either pre-symptomatic / asymptomatic or symptomatic conclusively)
hCoV-19/Poland/Sla4/2020	EPI_ISL_451658	05/05/2020	Europe / Poland / Slask	Human	Male	unknown	Quarantine	Original		Active miner	B.1.1	GR	Poland	Undertermined	No (couldn't determine the patient status as either pre-symptomatic / asymptomatic or symptomatic conclusively)
hCoV-19/Poland/Sla5/2020	EPI_ISL_451659	05/05/2020	Europe / Poland / Slask	Human	Male	unknown	Quarantine	Original		Active miner	B.1.1	GR	Poland	Undertermined	No (couldn't determine the patient status as either pre-symptomatic / asymptomatic or symptomatic conclusively)

hCoV-19/Poland/Sla6/2020	EPI_ISL_451660	05/05/2020	Europe / Poland / Slask	Human		Male	unknown	Quarantine	Original		Active miner	B.1.1	GR	Poland	Undertermined	No (couldn't determine the patient status as either pre-symptomatic / asymptomatic or symptomatic conclusively)
hCoV-19/Poland/Sla7/2020	EPI_ISL_451661	05/05/2020	Europe / Poland / Slask	Human		Male	unknown	Quarantine	Original		Active miner	B.1.1	GR	Poland	Undertermined	No (couldn't determine the patient status as either pre-symptomatic / asymptomatic or symptomatic conclusively)
hCoV-19/India/MH-NIV-11676/2020	EPI_ISL_454522	19/04/2020	Asia / India / Maharashtra	Human		unknown	37	Mild	Original			B.1.1.32	GR	India	Symptomatic	Yes
hCoV-19/India/MH-NIV-11699/2020	EPI_ISL_454523	19/04/2020	Asia / India / Maharashtra	Human		Female	59	Mild	Original			B.1.1.32	GR	India	Symptomatic	No (unusually high diversity)
hCoV-19/India/MH-NIV-12058/2020	EPI_ISL_454524	20/04/2020	Asia / India / Maharashtra	Human		Male	37	Mild	Original			B.1.1.32	GR	India	Symptomatic	Yes
hCoV-19/India/MH-NIV-1738/2020	EPI_ISL_454525	12/03/2020	Asia / India / Maharashtra	Human		Male	41	Mild	Original			B.4	O	India	Symptomatic	Yes
hCoV-19/India/MH-NIV-1804/2020	EPI_ISL_454526	13/03/2020	Asia / India / Maharashtra	Human		Female	12	Mild	Original			B.4	O	India	Symptomatic	Yes
hCoV-19/India/MH-NIV-2387/2020	EPI_ISL_454527	13/03/2020	Asia / India / Maharashtra	Human		Female	59	Mild	Original			B.1	G	India	Symptomatic	Yes
hCoV-19/India/MH-NIV-3472/2020	EPI_ISL_454528	17/03/2020	Asia / India / Maharashtra	Human		Male	50	Mild	Original			B.4	O	India	Symptomatic	Yes
hCoV-19/India/MH-NIV-4130/2020	EPI_ISL_454529	21/03/2020	Asia / India / Maharashtra	Human		Male	17	Mild	Original			B.1.80	G	India	Symptomatic	Yes
hCoV-19/India/MH-NIV-4438/2020	EPI_ISL_454533	23/03/2020	Asia / India / Maharashtra	Human		Male	53	Mild	Original			B.1.5	G	India	Symptomatic	Yes
hCoV-19/India/MH-NIV-4622/2020	EPI_ISL_454534	24/03/2020	Asia / India / Maharashtra	Human		Female	5	Mild	Original			A.7	S	India	Symptomatic	Yes
hCoV-19/India/MH-NIV-5097/2020	EPI_ISL_454535	26/03/2020	Asia / India / Maharashtra	Human		Female	70	Mild	Original			B.2	S	India	Symptomatic	No (unusually high diversity)
hCoV-19/India/MH-NIV-5102/2020	EPI_ISL_454536	26/03/2020	Asia / India / Maharashtra	Human		Female	39	Mild	Original			A.7	S	India	Symptomatic	Yes
hCoV-19/India/MH-NIV-5104/2020	EPI_ISL_454537	26/03/2020	Asia / India / Maharashtra	Human		Male	45	Mild	Original			A.7	S	India	Symptomatic	Yes
hCoV-19/India/MH-NIV-5118/2020	EPI_ISL_454538	26/03/2020	Asia / India / Maharashtra	Human		Male	35	Mild	Original			A	S	India	Symptomatic	No (unusually high diversity)
hCoV-19/India/MH-NIV-5815-1/2020	EPI_ISL_454539	12/04/2020	Asia / India / Maharashtra	Human		Male	64	Mild	Original			B.6	O	India	Symptomatic	No (unusually high diversity)
hCoV-19/India/MH-NIV-6216/2020	EPI_ISL_454540	01/04/2020	Asia / India / Maharashtra	Human		Male	35	Mild	Original			B.6	O	India	Symptomatic	Yes
hCoV-19/India/MH-NIV-6285/2020	EPI_ISL_454541	30/03/2020	Asia / India / Maharashtra	Human		Male	39	Mild	Original			B.6	O	India	Symptomatic	No (unusually high diversity)
hCoV-19/India/MH-NIV-6315/2020	EPI_ISL_454542	30/03/2020	Asia / India / Maharashtra	Human		Male	17	Mild	Original			B.6	O	India	Symptomatic	Yes
hCoV-19/India/MH-NIV-6423/2020	EPI_ISL_454543	31/03/2020	Asia / India / Maharashtra	Human		Female	40	Mild	Original			B.6	O	India	Symptomatic	Yes
hCoV-19/India/MH-NIV-6614/2020	EPI_ISL_454544	02/04/2020	Asia / India / Maharashtra	Human		Female	35	Mild	Original			B.6	O	India	Symptomatic	Yes
hCoV-19/India/MH-NIV-6638/2020	EPI_ISL_454545	02/04/2020	Asia / India / Maharashtra	Human		Male	59	Mild	Original			B.1	O	India	Symptomatic	No (unusually high diversity)
hCoV-19/India/MH-NIV-6964/2020	EPI_ISL_454546	03/04/2020	Asia / India / Maharashtra	Human		Male	19	Mild	Original			B.6	O	India	Symptomatic	Yes
hCoV-19/India/MH-NIV-7044/2020	EPI_ISL_454547	04/04/2020	Asia / India / Maharashtra	Human		Male	23	Mild	Original			B.6	O	India	Symptomatic	Yes
hCoV-19/India/MH-NIV-7098/2020	EPI_ISL_454548	03/04/2020	Asia / India / Maharashtra	Human		Male	42	Mild	Original			B.6	O	India	Symptomatic	Yes
hCoV-19/India/MH-NIV-7558/2020	EPI_ISL_454549	05/04/2020	Asia / India / Maharashtra	Human		Male	22	Mild	Original			B.2.1	V	India	Symptomatic	Yes
hCoV-19/India/MH-NIV-7832/2020	EPI_ISL_454550	06/04/2020	Asia / India / Maharashtra	Human		Female	20	Mild	Original			B.1.1.32	GR	India	Symptomatic	Yes
hCoV-19/India/MH-NIV-7893/2020	EPI_ISL_454551	07/04/2020	Asia / India / Maharashtra	Human		Female	63	Mild	Original			B.6	O	India	Symptomatic	Yes
hCoV-19/India/MH-NIV-7966/2020	EPI_ISL_454552	06/04/2020	Asia / India / Maharashtra	Human		Male	63	Mild	Original			B.6	O	India	Symptomatic	Yes
hCoV-19/India/MH-NIV-8187/2020	EPI_ISL_454553	02/04/2020	Asia / India / Maharashtra	Human		Male	16	Mild	Original			B.6	O	India	Symptomatic	No (unusually high diversity)
hCoV-19/India/MH-NIV-8199/2020	EPI_ISL_454554	02/04/2020	Asia / India / Maharashtra	Human		Male	30	Mild	Original			B.6	O	India	Symptomatic	No (unusually high diversity)
hCoV-19/India/MH-NIV-8404/2020	EPI_ISL_454555	07/04/2020	Asia / India / Maharashtra	Human		Male	30	Mild	Original			B.6	O	India	Symptomatic	Yes
hCoV-19/India/MH-NIV-9127/2020	EPI_ISL_454556	12/04/2020	Asia / India / Maharashtra	Human		Female	25	Mild	Original			B.1.1.32	O	India	Symptomatic	Yes
hCoV-19/India/MH-NIV-9154/2020	EPI_ISL_454557	13/04/2020	Asia / India / Maharashtra	Human		Male	80	Mild	Original			B.1.1.32	GR	India	Symptomatic	Yes
hCoV-19/India/MH-NIV-9314/2020	EPI_ISL_454558	13/04/2020	Asia / India / Maharashtra	Human		Female	70	Mild	Original			B.1.1	GR	India	Symptomatic	Yes
hCoV-19/India/MH-NIV-967/2020	EPI_ISL_454559	09/03/2020	Asia / India / Maharashtra	Human		Male	51	Mild	Original			B.4	O	India	Symptomatic	No (unusually high diversity)
hCoV-19/India/MH-NIV-701-3/2020	EPI_ISL_454560	06/04/2020	Asia / India / Maharashtra	Human		Male	38	Asymptomatic	Original			B.1.80	G	India	Pre-symptomatic / Asymptomatic	Yes
hCoV-19/India/MH-NIV-729-3/2020	EPI_ISL_454561	07/04/2020	Asia / India / Maharashtra	Human		Female	24	Asymptomatic	Original			B.6	O	India	Pre-symptomatic / Asymptomatic	Yes
hCoV-19/India/MH-NIV-815-3/2020	EPI_ISL_454562	07/04/2020	Asia / India / Maharashtra	Human		Male	76	fever	Original			B.1.80	G	India	Undertermined	No (couldn't determine the patient status as either pre-symptomatic / asymptomatic or symptomatic conclusively)
hCoV-19/Croatia/OY-S1new/2020	EPI_ISL_454574	09/04/2020	Europe / Croatia / Istria	Human		Male	36	Alive	Original	Oro-pharyngeal swab		B.1	G	Croatia	Undertermined	No (couldn't determine the patient status as either pre-symptomatic / asymptomatic or symptomatic conclusively)
hCoV-19/Croatia/592_Osijek/2020	EPI_ISL_454578	13/03/2020	Europe / Croatia / Osijek	Human		Female	63	Hospitalized	Original	combined nasopharyngeal and oropharyngeal swab		B.1	G	Croatia	Undertermined	No (couldn't determine the patient status as either pre-symptomatic / asymptomatic or symptomatic conclusively)
hCoV-19/Croatia/1146_Split/2020	EPI_ISL_454581	18/03/2020	Europe / Croatia / Split	Human		Male	85	Hospitalized	Original	combined nasopharyngeal and oropharyngeal swab		B.1.1	O	Croatia	Undertermined	No (couldn't determine the patient status as either pre-symptomatic / asymptomatic or symptomatic conclusively)
hCoV-19/Croatia/1560_Split/2020	EPI_ISL_454583	18/03/2020	Europe / Croatia / Split	Human		Male	25	Hospitalized	Original	combined nasopharyngeal and oropharyngeal swab		B.1	G	Croatia	Undertermined	No (couldn't determine the patient status as either pre-symptomatic / asymptomatic or symptomatic conclusively)
hCoV-19/Croatia/1761_Dubrovnik/2020	EPI_ISL_454588	20/03/2020	Europe / Croatia / Dubrovnik	Human		Male	58	Hospitalized	Original	combined nasopharyngeal and oropharyngeal swab		B.3	O	Croatia	Undertermined	No (couldn't determine the patient status as either pre-symptomatic / asymptomatic or symptomatic conclusively)
hCoV-19/Croatia/297_Varazdin/2020	EPI_ISL_454592	05/03/2020	Europe / Croatia / Varazdin	Human		Male	61	Hospitalized	Original	combined nasopharyngeal and oropharyngeal swab		B.1	G	Croatia	Undertermined	No (couldn't determine the patient status as either pre-symptomatic / asymptomatic or symptomatic conclusively)
hCoV-19/Croatia/361_Pula/2020	EPI_ISL_454595	09/03/2020	Europe / Croatia / Pula	Human		Female	41	Hospitalized	Original	combined nasopharyngeal and oropharyngeal swab		B.1.1	GR	Croatia	Undertermined	No (couldn't determine the patient status as either pre-symptomatic / asymptomatic or symptomatic conclusively)
hCoV-19/Malaysia/IIUM316/2020	EPI_ISL_455312	09/04/2020	Asia / Malaysia / Pahang	Human	Kuantan	Female	29	Live	Original	Combined nasopharyngeal and oropharyngeal swab	Healthcare worker	B.6	O	Malaysia	Undertermined	No (couldn't determine the patient status as either pre-symptomatic / asymptomatic or symptomatic conclusively)
hCoV-19/Malaysia/IIUM91/2020	EPI_ISL_455313	02/04/2020	Asia / Malaysia / Pahang	Human	Army base camp	Male	33	Live	Original	Combined nasopharyngeal and oropharyngeal swab	Unknown	B.1.36	GH	Malaysia	Undertermined	No (couldn't determine the patient status as either pre-symptomatic / asymptomatic or symptomatic conclusively)
hCoV-19/Spain/AN-ISCIII-201257/2020	EPI_ISL_455314	28/02/2020	Europe / Spain / Andalucia	Human		Female	37	Hospitalized	Original	Oropharyngeal swab		A	S	Spain	Undertermined	No (couldn't determine the patient status as either pre-symptomatic / asymptomatic or symptomatic conclusively)
hCoV-19/Spain/AN-ISCIII-201488/2020	EPI_ISL_455315	04/03/2020	Europe / Spain / Andalucia	Human		Female	18	Hospitalized	Original	Oropharyngeal swab		B.1	GH	Spain	Undertermined	No (couldn't determine the patient status as either pre-symptomatic / asymptomatic or symptomatic conclusively)
hCoV-19/Spain/AN-ISCIII-201613/2020	EPI_ISL_455316	05/03/2020	Europe / Spain / Andalucia	Human		Male	79	Hospitalized	Original	Oropharyngeal swab		A.2	S	Spain	Undertermined	No (couldn't determine the patient status as either pre-symptomatic / asymptomatic or symptomatic conclusively)
hCoV-19/Spain/AN-ISCIII-201614/2020	EPI_ISL_455317	05/03/2020	Europe / Spain / Andalucia	Human		Male	75	Hospitalized	Original	Oropharyngeal swab		A	S	Spain	Undertermined	No (couldn't determine the patient status as either pre-symptomatic / asymptomatic or symptomatic conclusively)
hCoV-19/Spain/AN-ISCIII-201615/2020	EPI_ISL_455318	05/03/2020	Europe / Spain / Andalucia	Human		Female	unknown	Hospitalized	Original	Oropharyngeal swab		B	L	Spain	Undertermined	No (couldn't determine the patient status as either pre-symptomatic / asymptomatic or symptomatic conclusively)
hCoV-19/Spain/AN-ISCIII-201616/2020	EPI_ISL_455319	05/03/2020	Europe / Spain / Andalucia	Human		Male	73	Hospitalized	Original	Oropharyngeal swab		B	L	Spain	Undertermined	No (couldn't determine the patient status as either pre-symptomatic / asymptomatic or symptomatic conclusively)
hCoV-19/Spain/AN-ISCIII-201620/2020	EPI_ISL_455320	05/03/2020	Europe / Spain / Andalucia	Human		Male	70	Hospitalized	Original	Oropharyngeal swab		A.2	S	Spain	Undertermined	No (couldn't determine the patient status as either pre-symptomatic / asymptomatic or symptomatic conclusively)
hCoV-19/Spain/AN-ISCIII-201622/2020	EPI_ISL_455321	05/03/2020	Europe / Spain / Andalucia	Human		Male	unknown	Hospitalized	Original	Oropharyngeal swab		A.2	S	Spain	Undertermined	No (couldn't determine the patient status as either pre-symptomatic / asymptomatic or symptomatic conclusively)
hCoV-19/Spain/AN-ISCIII-201623/2020	EPI_ISL_455322	05/03/2020	Europe / Spain / Andalucia	Human		Male	unknown	Hospitalized	Original	Oropharyngeal swab		A.2	S	Spain	Undertermined	No (couldn't determine the patient status as either pre-symptomatic / asymptomatic or symptomatic conclusively)
hCoV-19/Spain/AN-ISCIII-201636/2020	EPI_ISL_455323	06/03/2020	Europe / Spain / Andalucia	Human		Female	21	Hospitalized	Original	Oropharyngeal swab		B.1	O	Spain	Undertermined	No (couldn't determine the patient status as either pre-symptomatic / asymptomatic or symptomatic conclusively)
hCoV-19/Spain/AN-ISCIII-201649/2020	EPI_ISL_455324	03/03/2020	Europe / Spain / Andalucia	Human		Female	74	Hospitalized	Original	Oropharyngeal swab		B	O	Spain	Undertermined	No (couldn't determine the patient status as either pre-symptomatic / asymptomatic or symptomatic conclusively)
hCoV-19/Spain/CN-ISCIII-201811/2020	EPI_ISL_455325	05/03/2020	Europe / Spain / Canary Islands	Human		Female	26	Hospitalized	Original	Oropharyngeal swab		A.2	O	Spain	Undertermined	No (couldn't determine the patient status as either pre-symptomatic / asymptomatic or symptomatic conclusively)
hCoV-19/Spain/CN-ISCIII-201939/2020	EPI_ISL_455326	29/02/2020	Europe / Spain / Canary Islands	Human		Female	28	Hospitalized	Original	Oropharyngeal swab		B.1.1	GR	Spain	Undertermined	No (couldn't determine the patient status as either pre-symptomatic / asymptomatic or symptomatic conclusively)
hCoV-19/Spain/CM-ISCIII-201306/2020	EPI_ISL_455327	02/03/2020	Europe / Spain / Castilla La Mancha	Human		Male	44	Hospitalized	Original	Oropharyngeal swab		B.1.74	G	Spain	Undertermined	No (couldn't determine the patient status as either pre-symptomatic / asymptomatic or symptomatic conclusively)
hCoV-19/Spain/GA-ISCIII-201792/2020	EPI_ISL_455328	09/03/2020	Europe / Spain / Galicia	Human		Female	67	Hospitalized	Original	Oropharyngeal swab		A.2	S	Spain	Undertermined	No (couldn't determine the patient status as either pre-symptomatic / asymptomatic or symptomatic conclusively)
hCoV-19/Spain/GA-ISCIII-201793/2020	EPI_ISL_455329	09/03/2020	Europe / Spain / Galicia	Human		Male	65	Hospitalized	Original	Oropharyngeal swab		A.2	S	Spain	Undertermined	No (couldn't determine the patient status as either pre-symptomatic / asymptomatic or symptomatic conclusively)
hCoV-19/Spain/GA-ISCIII-201796/2020	EPI_ISL_455330	09/03/2020	Europe / Spain / Galicia	Human		Female	70	Hospitalized	Original	Oropharyngeal swab		A.2	S	Spain	Undertermined	No (couldn't determine the patient status as either pre-symptomatic / asymptomatic or symptomatic conclusively)
hCoV-19/Spain/GA-ISCIII-201863/2020	EPI_ISL_455331	10/03/2020	Europe / Spain / Galicia	Human		Female	58	Hospitalized	Original	Oropharyngeal swab		A.2	S	Spain	Undertermined	No (couldn't determine the patient status as either pre-symptomatic / asymptomatic or symptomatic conclusively)
hCoV-19/Spain/GA-ISCIII-202076/2020	EPI_ISL_455332	10/03/2020	Europe / Spain / Galicia	Human		Female	62	Hospitalized	Original	Oropharyngeal swab		B.1.5	G	Spain	Undertermined	No (couldn't determine the patient status as either pre-symptomatic / asymptomatic or symptomatic conclusively)
hCoV-19/Spain/GA-ISCIII-202230/2020	EPI_ISL_455333	11/03/2020	Europe / Spain / Galicia	Human		Female	37	Hospitalized	Original	Oropharyngeal swab		B.1.5	G	Spain	Undertermined	No (couldn't determine the patient status as either pre-symptomatic / asymptomatic or symptomatic conclusively)
hCoV-19/Spain/GA-ISCIII-202282/2020	EPI_ISL_455334	11/03/2020	Europe / Spain / Galicia	Human		Male	39	Hospitalized	Original	Oropharyngeal swab		B.1.5	G	Spain	Undertermined	No (couldn't determine the patient status as either pre-symptomatic / asymptomatic or symptomatic conclusively)
hCoV-19/Spain/GA-ISCIII-202297/2020	EPI_ISL_455335	11/03/2020	Europe / Spain / Galicia	Human		Female	55	Hospitalized	Original	Oropharyngeal swab		B.1.5	G	Spain	Undertermined	No (couldn't determine the patient status as either pre-symptomatic / asymptomatic or symptomatic conclusively)
hCoV-19/Spain/RI-ISCIII-201277/2020	EPI_ISL_455336	29/02/2020	Europe / Spain / La Rioja	Human		Male	41	Hospitalized	Original	Oropharyngeal swab		A.2	S	Spain	Undertermined	No (couldn't determine the patient status as either pre-symptomatic / asymptomatic or symptomatic conclusively)
hCoV-19/Spain/RI-ISCIII-201334/2020	EPI_ISL_455337	01/03/2020	Europe / Spain / La Rioja	Human		Male	43	Hospitalized	Original	Oropharyngeal swab		A.2	S	Spain	Undertermined	No (couldn't determine the patient status as either pre-symptomatic / asymptomatic or symptomatic conclusively)
hCoV-19/Spain/RI-ISCIII-201563/2020	EPI_ISL_455338	04/03/2020	Europe / Spain / La Rioja	Human		Male	41	Hospitalized	Original	Oropharyngeal swab		A.2	S	Spain	Undertermined	No (couldn't determine the patient status as either pre-symptomatic / asymptomatic or symptomatic conclusively)
hCoV-19/Spain/RI-ISCIII-201565/2020	EPI_ISL_455339	04/03/2020	Europe / Spain / La Rioja	Human		Female	36	Hospitalized	Original	Oropharyngeal swab		A.2	S	Spain	Undertermined	No (couldn't determine the patient status as either pre-symptomatic / asymptomatic or symptomatic conclusively)
hCoV-19/Spain/RI-ISCIII-201568/2020	EPI_ISL_455340	05/03/2020	Europe / Spain / La Rioja	Human		Male	37	Hospitalized	Original	Oropharyngeal swab		A.2	S	Spain	Undertermined	No (couldn't determine the patient status as either pre-symptomatic / asymptomatic or symptomatic conclusively)
hCoV-19/Spain/RI-ISCIII-201570/2020	EPI_ISL_455341	03/03/2020	Europe / Spain / La Rioja	Human		Female	34	Hospitalized	Original	Oropharyngeal swab		A.2	S	Spain	Undertermined	No (couldn't determine the patient status as either pre-symptomatic / asymptomatic or symptomatic conclusively)
hCoV-19/Spain/RI-ISCIII-201572/2020	EPI_ISL_455342	03/03/2020	Europe / Spain / La Rioja	Human		Male	54	Hospitalized	Original	Oropharyngeal swab		A	O	Spain	Undertermined	No (couldn't determine the patient status as either pre-symptomatic / asymptomatic or symptomatic conclusively)
hCoV-19/Spain/RI-ISCIII-201576/2020	EPI_ISL_455343	03/03/2020	Europe / Spain / La Rioja	Human		Female	48	Hospitalized	Original	Oropharyngeal swab		A.2	S	Spain	Undertermined	No (couldn't determine the patient status as either pre-symptomatic / asymptomatic or symptomatic conclusively)
hCoV-19/Spain/ML-ISCIII-202020/2020	EPI_ISL_455344	11/03/2020	Europe / Spain / Melilla	Human		Male	54	Hospitalized	Original	Oropharyngeal swab		B.1.5	G	Spain	Undertermined	No (couldn't determine the patient status as either pre-symptomatic / asymptomatic or symptomatic conclusively)
hCoV-19/Spain/ML-ISCIII-202134/2020	EPI_ISL_455345	11/03/2020	Europe / Spain / Melilla	Human		Female	31	Hospitalized	Original	Oropharyngeal swab		B.1	GH	Spain	Undertermined	No (couldn't determine the patient status as either pre-symptomatic / asymptomatic or symptomatic conclusively)
hCoV-19/Spain/ML-ISCIII-202135/2020	EPI_ISL_455346	11/03/2020	Europe / Spain / Melilla	Human		Male	47	Hospitalized	Original	Oropharyngeal swab		B.1.5	G	Spain	Undertermined	No (couldn't determine the patient status as either pre-symptomatic / asymptomatic or symptomatic conclusively)
hCoV-19/Oman/RESP-20-6560/2020	EPI_ISL_457997	28/03/2020	Asia / Oman / Muscta	Human		Male	62	Hospitaized	Original	Oro-pharyngeal swab		B	L	Oman	Undertermined	No (couldn't determine the patient status as either pre-symptomatic / asymptomatic or symptomatic conclusively)
hCoV-19/Indonesia/JI-ITD-1238Sp/2020	EPI_ISL_458079	30/03/2020	Asia / Indonesia / East Java / Surabaya	Human		Male	71	Hospitalized	Original	Sputum		B	L	Indonesia	Undertermined	No (couldn't determine the patient status as either pre-symptomatic / asymptomatic or symptomatic conclusively)
hCoV-19/Indonesia/JI-ITD-1273NT/2020	EPI_ISL_458081	30/03/2020	Asia / Indonesia / East Java / Pasuruan	Human		Male	39	Hospitalized	Original	Nasopharyngeal and Oropharyngeal swab		B	L	Indonesia	Undertermined	No (couldn't determine the patient status as either pre-symptomatic / asymptomatic or symptomatic conclusively)
hCoV-19/Indonesia/JI-ITD-2766NT/2020	EPI_ISL_458082	09/04/2020	Asia / Indonesia / East Java / Surabaya	Human		Male	21	Hospitalized	Original	Nasopharyngeal and Oropharyngeal swab		B.1	GH	Indonesia	Undertermined	No (couldn't determine the patient status as either pre-symptomatic / asymptomatic or symptomatic conclusively)
hCoV-19/Indonesia/JI-ITD-3101NT/2020	EPI_ISL_458083	11/04/2020	Asia / Indonesia / East Java / Surabaya	Human		Male	42	Hospitalized	Original	Nasopharyngeal and Oropharyngeal swab		B	L	Indonesia	Undertermined	No (couldn't determine the patient status as either pre-symptomatic / asymptomatic or symptomatic conclusively)
hCoV-19/India/GJ-GBRC131/2020	EPI_ISL_458086	24/05/2020	Asia / India / Gujarat / Ahmedabad	Human		Female	70	Hospitalized	Original			B.1.113	GH	India	Undertermined	No (couldn't determine the patient status as either pre-symptomatic / asymptomatic or symptomatic conclusively)
hCoV-19/India/GJ-GBRC132/2020	EPI_ISL_458087	24/05/2020	Asia / India / Gujarat / Ahmedabad	Human		Male	37	Hospitalized	Original			B.1.36	GH	India	Undertermined	No (couldn't determine the patient status as either pre-symptomatic / asymptomatic or symptomatic conclusively)
hCoV-19/India/GJ-GBRC133/2020	EPI_ISL_458088	24/05/2020	Asia / India / Gujarat / Ahmedabad	Human		Male	51	Hospitalized	Original			B.1.113	GH	India	Undertermined	No (couldn't determine the patient status as either pre-symptomatic / asymptomatic or symptomatic conclusively)
hCoV-19/India/GJ-GBRC134/2020	EPI_ISL_458089	24/05/2020	Asia / India / Gujarat / Ahmedabad	Human		Female	75	Hospitalized	Original			B.1.36	GH	India	Undertermined	No (couldn't determine the patient status as either pre-symptomatic / asymptomatic or symptomatic conclusively)
hCoV-19/India/GJ-GBRC135a/2020	EPI_ISL_458090	24/05/2020	Asia / India / Gujarat / Ahmedabad	Human		Female	56	Hospitalized	Original			B.1.113	GH	India	Undertermined	No (couldn't determine the patient status as either pre-symptomatic / asymptomatic or symptomatic conclusively)
hCoV-19/India/GJ-GBRC135b/2020	EPI_ISL_458091	24/05/2020	Asia / India / Gujarat / Ahmedabad	Human		Female	56	Hospitalized	Original			B.1.113	GH	India	Undertermined	No (couldn't determine the patient status as either pre-symptomatic / asymptomatic or symptomatic conclusively)
hCoV-19/India/GJ-GBRC136a/2020	EPI_ISL_458092	24/05/2020	Asia / India / Gujarat / Ahmedabad	Human		Male	65	Hospitalized	Original			B.1.113	GH	India	Undertermined	No (couldn't determine the patient status as either pre-symptomatic / asymptomatic or symptomatic conclusively)
hCoV-19/India/GJ-GBRC136b/2020	EPI_ISL_458093	24/05/2020	Asia / India / Gujarat / Ahmedabad	Human		Male	65	Hospitalized	Original			B.1.113	G	India	Undertermined	No (couldn't determine the patient status as either pre-symptomatic / asymptomatic or symptomatic conclusively)
hCoV-19/India/GJ-GBRC137a/2020	EPI_ISL_458094	24/05/2020	Asia / India / Gujarat / Ahmedabad	Human		Male	58	Hospitalized	Original			B.1.113	GH	India	Undertermined	No (couldn't determine the patient status as either pre-symptomatic / asymptomatic or symptomatic conclusively)
hCoV-19/India/GJ-GBRC137b/2020	EPI_ISL_458095	24/05/2020	Asia / India / Gujarat / Ahmedabad	Human		Male	58	Hospitalized	Original			B.1.113	GH	India	Undertermined	No (couldn't determine the patient status as either pre-symptomatic / asymptomatic or symptomatic conclusively)
hCoV-19/India/GJ-GBRC138/2020	EPI_ISL_458096	24/05/2020	Asia / India / Gujarat / Ahmedabad	Human		Male	63	Hospitalized	Original			B.1	G	India	Undertermined	No (couldn't determine the patient status as either pre-symptomatic / asymptomatic or symptomatic conclusively)
hCoV-19/India/GJ-GBRC139a/2020	EPI_ISL_458097	24/05/2020	Asia / India / Gujarat / Ahmedabad	Human		Female	65	Hospitalized	Original			B.1.113	GH	India	Undertermined	No (couldn't determine the patient status as either pre-symptomatic / asymptomatic or symptomatic conclusively)
hCoV-19/India/GJ-GBRC139b/2020	EPI_ISL_458098	24/05/2020	Asia / India / Gujarat / Ahmedabad	Human		Female	65	Hospitalized	Original			B.1.113	GH	India	Undertermined	No (couldn't determine the patient status as either pre-symptomatic / asymptomatic or symptomatic conclusively)
hCoV-19/India/GJ-GBRC140/2020	EPI_ISL_458099	24/05/2020	Asia / India / Gujarat / Ahmedabad	Human		Female	40	Hospitalized	Original			B.1.113	GH	India	Undertermined	No (couldn't determine the patient status as either pre-symptomatic / asymptomatic or symptomatic conclusively)
hCoV-19/Spain/CT-IrsiCaixa-sp50/2020	EPI_ISL_462447	22/03/2020	Europe / Spain / Catalunya / Barcelona / Igualada	Human		Female	57	Home	Original	Nasopharyngeal swab	Health Worker	B.1.5	G	Spain	Undertermined	No (couldn't determine the patient status as either pre-symptomatic / asymptomatic or symptomatic conclusively)
hCoV-19/Spain/CT-IrsiCaixa-sp142/2020	EPI_ISL_462448	24/03/2020	Europe / Spain / Catalunya / Barcelona / MatarÃ³	Human		Male	61	Mild, at home.	Original	Nasopharyngeal swab	Health Worker	B.1.5	G	Spain	Symptomatic	Yes
hCoV-19/Spain/CT-IrsiCaixa-015D0/2020	EPI_ISL_462449	20/03/2020	Europe / Spain / Catalunya / Barcelona / Igualada	Human		Female	42	Live, mild symptoms, at home	Original	Nasopharyngeal swab	Health Worker	B.1.5	O	Spain	Symptomatic	Yes
hCoV-19/Spain/AN-ISCIII-202659/2020	EPI_ISL_462477	13/03/2020	Europe / Spain / Andalucia	Human		Male	35	Hospitalized	Original	Oropharyngeal swab		A.2	S	Spain	Undertermined	No (couldn't determine the patient status as either pre-symptomatic / asymptomatic or symptomatic conclusively)
hCoV-19/Cyprus/001/2020	EPI_ISL_463741	11/03/2020	Europe / Cyprus	Human		Female	36	Recovered	Original	Oro-pharyngeal swab		B.1.1	GR	Cyprus	Undertermined	No (couldn't determine the patient status as either pre-symptomatic / asymptomatic or symptomatic conclusively)
hCoV-19/Cyprus/002/2020	EPI_ISL_463742	22/03/2020	Europe / Cyprus	Human		Male	53	Recovered	Original	Oro-pharyngeal swab		B.1.1	GR	Cyprus	Undertermined	No (couldn't determine the patient status as either pre-symptomatic / asymptomatic or symptomatic conclusively)
hCoV-19/Cyprus/003/2020	EPI_ISL_463743	25/03/2020	Europe / Cyprus	Human		Female	40	Recovered	Original	Oro-pharyngeal swab		B.1.1	GR	Cyprus	Undertermined	No (couldn't determine the patient status as either pre-symptomatic / asymptomatic or symptomatic conclusively)
hCoV-19/Cyprus/004/2020	EPI_ISL_463744	23/03/2020	Europe / Cyprus	Human		Male	74	Recovered	Original	Oro-pharyngeal swab		B.1.1	GR	Cyprus	Undertermined	No (couldn't determine the patient status as either pre-symptomatic / asymptomatic or symptomatic conclusively)
hCoV-19/Cyprus/005/2020	EPI_ISL_463745	01/04/2020	Europe / Cyprus	Human		Female	51	Recovered	Original	Oro-pharyngeal swab		B.1.1	GR	Cyprus	Undertermined	No (couldn't determine the patient status as either pre-symptomatic / asymptomatic or symptomatic conclusively)
hCoV-19/Cyprus/006/2020	EPI_ISL_463746	11/04/2020	Europe / Cyprus	Human		Female	39	Recovered	Original	Oro-pharyngeal swab		B.1.1	GR	Cyprus	Undertermined	No (couldn't determine the patient status as either pre-symptomatic / asymptomatic or symptomatic conclusively)
hCoV-19/Cyprus/007/2020	EPI_ISL_463747	17/04/2020	Europe / Cyprus	Human		Male	33	Recovered	Original	Oro-pharyngeal swab		B.1.79	G	Cyprus	Undertermined	No (couldn't determine the patient status as either pre-symptomatic / asymptomatic or symptomatic conclusively)
hCoV-19/Cyprus/008/2020	EPI_ISL_463748	27/04/2020	Europe / Cyprus	Human		Male	61	Recovered	Original	Oro-pharyngeal swab		B.1.1	GR	Cyprus	Undertermined	No (couldn't determine the patient status as either pre-symptomatic / asymptomatic or symptomatic conclusively)
hCoV-19/Morocco/RA85/2020	EPI_ISL_467299	21/05/2020	Africa / Morocco	Human		Male	25	Live	Original	Naso-pharyngeal swab		B.1	G	Morocco	Undertermined	No (couldn't determine the patient status as either pre-symptomatic / asymptomatic or symptomatic conclusively)
hCoV-19/Bosnia and Herzegovina/04-Sarajevo/2020	EPI_ISL_467300	08/04/2020	Europe / Bosnia and Herzegovina / Sarajevo	Human		Male	1	Released	Original			B.1	G	Bosnia and Herzegovina	Undertermined	No (couldn't determine the patient status as either pre-symptomatic / asymptomatic or symptomatic conclusively)
hCoV-19/Croatia/LG-S2new/2020	EPI_ISL_468591	10/04/2020	Europe / Croatia / Istria	Human		Male	57	Live	Original			B.1	G	Croatia	Undertermined	No (couldn't determine the patient status as either pre-symptomatic / asymptomatic or symptomatic conclusively)
hCoV-19/Croatia/AU-S10new/2020	EPI_ISL_468656	09/04/2020	Europe / Croatia / Istria	Human		Female	31	Live	Original			B.1	G	Croatia	Undertermined	No (couldn't determine the patient status as either pre-symptomatic / asymptomatic or symptomatic conclusively)
hCoV-19/USA/CA-CSMC123/2020	EPI_ISL_475600	26/03/2020	North America / USA / California / Los Angeles	Human		Male	66	Still hospitalized	Original			B.1.2	GH	USA	Undertermined	No (couldn't determine the patient status as either pre-symptomatic / asymptomatic or symptomatic conclusively)
hCoV-19/USA/CA-CSMC124/2020	EPI_ISL_475601	05/04/2020	North America / USA / California / Los Angeles	Human		Male	33	Released	Original			B.1.1	GR	USA	Undertermined	No (couldn't determine the patient status as either pre-symptomatic / asymptomatic or symptomatic conclusively)
hCoV-19/USA/CA-CSMC125/2020	EPI_ISL_475602	05/04/2020	North America / USA / California / Los Angeles	Human		Female	93	Deceased	Original			B.1.1	GR	USA	Symptomatic	Yes
hCoV-19/USA/CA-CSMC126/2020	EPI_ISL_475603	09/04/2020	North America / USA / California / Los Angeles	Human		Male	77	Released	Original			B.1	GH	USA	Undertermined	No (couldn't determine the patient status as either pre-symptomatic / asymptomatic or symptomatic conclusively)
hCoV-19/USA/CA-CSMC127/2020	EPI_ISL_475604	05/04/2020	North America / USA / California / Los Angeles	Human		Female	67	Released	Original			B.1	GH	USA	Undertermined	No (couldn't determine the patient status as either pre-symptomatic / asymptomatic or symptomatic conclusively)
hCoV-19/USA/CA-CSMC128/2020	EPI_ISL_475605	09/04/2020	North America / USA / California / Los Angeles	Human		Female	87	Released	Original			B.1.3	GH	USA	Undertermined	No (couldn't determine the patient status as either pre-symptomatic / asymptomatic or symptomatic conclusively)
hCoV-19/USA/CA-CSMC129/2020	EPI_ISL_475606	05/04/2020	North America / USA / California / Los Angeles	Human		Male	48	Released	Original			B.1	GH	USA	Undertermined	No (couldn't determine the patient status as either pre-symptomatic / asymptomatic or symptomatic conclusively)
hCoV-19/USA/CA-CSMC13/2020	EPI_ISL_475607	28/03/2020	North America / USA / California / Los Angeles	Human		Female	56	Outpatient	Original			A.1	S	USA	Undertermined	No (couldn't determine the patient status as either pre-symptomatic / asymptomatic or symptomatic conclusively)
hCoV-19/USA/CA-CSMC130/2020	EPI_ISL_475608	26/03/2020	North America / USA / California / Los Angeles	Human		Male	68	Released	Original			B.1	GH	USA	Undertermined	No (couldn't determine the patient status as either pre-symptomatic / asymptomatic or symptomatic conclusively)
hCoV-19/USA/CA-CSMC131/2020	EPI_ISL_475609	26/03/2020	North America / USA / California / Los Angeles	Human		Male	28	Released	Original			B.1.43	GH	USA	Undertermined	No (couldn't determine the patient status as either pre-symptomatic / asymptomatic or symptomatic conclusively)
hCoV-19/USA/CA-CSMC132/2020	EPI_ISL_475610	26/03/2020	North America / USA / California / Los Angeles	Human		Male	29	Outpatient	Original			A	O	USA	Undertermined	No (couldn't determine the patient status as either pre-symptomatic / asymptomatic or symptomatic conclusively)
hCoV-19/USA/CA-CSMC133/2020	EPI_ISL_475611	26/03/2020	North America / USA / California / Los Angeles	Human		Male	34	Outpatient	Original			B.1	GH	USA	Undertermined	No (couldn't determine the patient status as either pre-symptomatic / asymptomatic or symptomatic conclusively)
hCoV-19/USA/CA-CSMC134/2020	EPI_ISL_475612	30/03/2020	North America / USA / California / Los Angeles	Human		Male	49	Released	Original			A.3	S	USA	Undertermined	No (couldn't determine the patient status as either pre-symptomatic / asymptomatic or symptomatic conclusively)
hCoV-19/USA/CA-CSMC135/2020	EPI_ISL_475613	26/03/2020	North America / USA / California / Los Angeles	Human		Male	59	Released	Original			A	S	USA	Undertermined	No (couldn't determine the patient status as either pre-symptomatic / asymptomatic or symptomatic conclusively)
hCoV-19/USA/CA-CSMC136/2020	EPI_ISL_475614	06/04/2020	North America / USA / California / Los Angeles	Human		Male	79	Deceased	Original			B.1	GH	USA	Symptomatic	Yes
hCoV-19/USA/CA-CSMC137/2020	EPI_ISL_475615	12/04/2020	North America / USA / California / Los Angeles	Human		Female	90	Deceased	Original			B.1	GH	USA	Symptomatic	Yes
hCoV-19/USA/CA-CSMC138/2020	EPI_ISL_475616	06/04/2020	North America / USA / California / Los Angeles	Human		Male	66	Deceased	Original			B.1	GH	USA	Symptomatic	Yes
hCoV-19/USA/CA-CSMC139/2020	EPI_ISL_475617	12/04/2020	North America / USA / California / Los Angeles	Human		Female	37	Outpatient	Original			B.1.43	GH	USA	Undertermined	No (couldn't determine the patient status as either pre-symptomatic / asymptomatic or symptomatic conclusively)
hCoV-19/USA/CA-CSMC14/2020	EPI_ISL_475618	24/03/2020	North America / USA / California / Los Angeles	Human		Male	64	Released	Original			B.1	GH	USA	Undertermined	No (couldn't determine the patient status as either pre-symptomatic / asymptomatic or symptomatic conclusively)
hCoV-19/USA/CA-CSMC140/2020	EPI_ISL_475619	12/04/2020	North America / USA / California / Los Angeles	Human		Male	92	Deceased	Original			B.1	GH	USA	Symptomatic	Yes

hCoV-19/USA/CA-CSMC141/2020	EPI_ISL_475620	08/04/2020	North America / USA / California / Los Angeles	Human	Male	61	Released	Original		B.1	GH	USA	Undetermined	No (couldn't determine the patient status as either pre-symptomatic / asymptomatic or symptomatic conclusively)
hCoV-19/USA/CA-CSMC142/2020	EPI_ISL_475621	06/04/2020	North America / USA / California / Los Angeles	Human	Female	49	Deceased	Original		B.1	GH	USA	Symptomatic	Yes
hCoV-19/USA/CA-CSMC19/2020	EPI_ISL_475628	25/03/2020	North America / USA / California / Los Angeles	Human	Female	43	Released	Original		B.1	GH	USA	Undetermined	No (couldn't determine the patient status as either pre-symptomatic / asymptomatic or symptomatic conclusively)
hCoV-19/USA/CA-CSMC2/2020	EPI_ISL_475629	27/03/2020	North America / USA / California / Los Angeles	Human	Male	87	Released	Original		B.1	GH	USA	Undetermined	No (couldn't determine the patient status as either pre-symptomatic / asymptomatic or symptomatic conclusively)
hCoV-19/USA/CA-CSMC20/2020	EPI_ISL_475630	25/03/2020	North America / USA / California / Los Angeles	Human	Female	74	Released	Original		A.1	S	USA	Undetermined	No (couldn't determine the patient status as either pre-symptomatic / asymptomatic or symptomatic conclusively)
hCoV-19/USA/CA-CSMC21/2020	EPI_ISL_475631	25/03/2020	North America / USA / California / Los Angeles	Human	Male	74	Released	Original		B.1	GH	USA	Undetermined	No (couldn't determine the patient status as either pre-symptomatic / asymptomatic or symptomatic conclusively)
hCoV-19/USA/CA-CSMC22/2020	EPI_ISL_475632	25/03/2020	North America / USA / California / Los Angeles	Human	Male	42	Outpatient	Original		B.1.1	GR	USA	Undetermined	No (couldn't determine the patient status as either pre-symptomatic / asymptomatic or symptomatic conclusively)
hCoV-19/USA/CA-CSMC23/2020	EPI_ISL_475633	25/03/2020	North America / USA / California / Los Angeles	Human	Female	27	Outpatient	Original		B.1	G	USA	Undetermined	No (couldn't determine the patient status as either pre-symptomatic / asymptomatic or symptomatic conclusively)
hCoV-19/USA/CA-CSMC24/2020	EPI_ISL_475634	25/03/2020	North America / USA / California / Los Angeles	Human	Male	55	Outpatient	Original		B.1.1	GR	USA	Undetermined	No (couldn't determine the patient status as either pre-symptomatic / asymptomatic or symptomatic conclusively)
hCoV-19/USA/CA-CSMC25/2020	EPI_ISL_475635	25/03/2020	North America / USA / California / Los Angeles	Human	Female	38	Outpatient	Original		B	L	USA	Undetermined	No (couldn't determine the patient status as either pre-symptomatic / asymptomatic or symptomatic conclusively)
hCoV-19/USA/CA-CSMC26/2020	EPI_ISL_475636	25/03/2020	North America / USA / California / Los Angeles	Human	Female	67	Released	Original		B	L	USA	Undetermined	No (couldn't determine the patient status as either pre-symptomatic / asymptomatic or symptomatic conclusively)
hCoV-19/USA/CA-CSMC27/2020	EPI_ISL_475637	26/03/2020	North America / USA / California / Los Angeles	Human	Male	69	Deceased	Original		B.1	GH	USA	Symptomatic	Yes
hCoV-19/USA/CA-CSMC28/2020	EPI_ISL_475638	25/03/2020	North America / USA / California / Los Angeles	Human	Male	19	Released	Original		B.1.43	GH	USA	Undetermined	No (couldn't determine the patient status as either pre-symptomatic / asymptomatic or symptomatic conclusively)
hCoV-19/USA/CA-CSMC29/2020	EPI_ISL_475639	25/03/2020	North America / USA / California / Los Angeles	Human	Female	77	Released	Original		B.1	GH	USA	Undetermined	No (couldn't determine the patient status as either pre-symptomatic / asymptomatic or symptomatic conclusively)
hCoV-19/USA/CA-CSMC3/2020	EPI_ISL_475640	27/03/2020	North America / USA / California / Los Angeles	Human	Female	32	Outpatient	Original		B.1	GH	USA	Undetermined	No (couldn't determine the patient status as either pre-symptomatic / asymptomatic or symptomatic conclusively)
hCoV-19/USA/CA-CSMC30/2020	EPI_ISL_475641	25/03/2020	North America / USA / California / Los Angeles	Human	Male	60	Released	Original		B.1.3	GH	USA	Undetermined	No (couldn't determine the patient status as either pre-symptomatic / asymptomatic or symptomatic conclusively)
hCoV-19/USA/CA-CSMC31/2020	EPI_ISL_475642	01/04/2020	North America / USA / California / Los Angeles	Human	Male	44	Released	Original		B.1.43	GH	USA	Undetermined	No (couldn't determine the patient status as either pre-symptomatic / asymptomatic or symptomatic conclusively)
hCoV-19/USA/CA-CSMC32/2020	EPI_ISL_475643	13/04/2020	North America / USA / California / Los Angeles	Human	Male	29	Still hospitalized	Original		B.1	GH	USA	Undetermined	No (couldn't determine the patient status as either pre-symptomatic / asymptomatic or symptomatic conclusively)
hCoV-19/USA/CA-CSMC33/2020	EPI_ISL_475644	03/04/2020	North America / USA / California / Los Angeles	Human	Female	67	Released	Original		B.1	GH	USA	Undetermined	No (couldn't determine the patient status as either pre-symptomatic / asymptomatic or symptomatic conclusively)
hCoV-19/USA/CA-CSMC34/2020	EPI_ISL_475645	03/04/2020	North America / USA / California / Los Angeles	Human	Male	44	Still hospitalized	Original		A.1	S	USA	Undetermined	No (couldn't determine the patient status as either pre-symptomatic / asymptomatic or symptomatic conclusively)
hCoV-19/USA/CA-CSMC35/2020	EPI_ISL_475646	01/04/2020	North America / USA / California / Los Angeles	Human	Male	75	Released	Original		B.1	GH	USA	Undetermined	No (couldn't determine the patient status as either pre-symptomatic / asymptomatic or symptomatic conclusively)
hCoV-19/USA/CA-CSMC36/2020	EPI_ISL_475647	03/04/2020	North America / USA / California / Los Angeles	Human	Male	56	Released	Original		B.1.2	GH	USA	Undetermined	No (couldn't determine the patient status as either pre-symptomatic / asymptomatic or symptomatic conclusively)
hCoV-19/USA/CA-CSMC37/2020	EPI_ISL_475648	03/04/2020	North America / USA / California / Los Angeles	Human	Female	97	Released	Original		B.1	GH	USA	Undetermined	No (couldn't determine the patient status as either pre-symptomatic / asymptomatic or symptomatic conclusively)
hCoV-19/USA/CA-CSMC38/2020	EPI_ISL_475649	03/04/2020	North America / USA / California / Los Angeles	Human	Female	88	Still hospitalized	Original		B.1	GH	USA	Undetermined	No (couldn't determine the patient status as either pre-symptomatic / asymptomatic or symptomatic conclusively)
hCoV-19/USA/CA-CSMC39/2020	EPI_ISL_475650	03/04/2020	North America / USA / California / Los Angeles	Human	Female	66	Released	Original		B.1.43	GH	USA	Undetermined	No (couldn't determine the patient status as either pre-symptomatic / asymptomatic or symptomatic conclusively)
hCoV-19/USA/CA-CSMC4/2020	EPI_ISL_475651	27/03/2020	North America / USA / California / Los Angeles	Human	Male	57	Released	Original		B.1.1	GR	USA	Undetermined	No (couldn't determine the patient status as either pre-symptomatic / asymptomatic or symptomatic conclusively)
hCoV-19/USA/CA-CSMC40/2020	EPI_ISL_475652	03/04/2020	North America / USA / California / Los Angeles	Human	Male	82	Deceased	Original		B.1.43	GH	USA	Symptomatic	Yes
hCoV-19/USA/CA-CSMC41/2020	EPI_ISL_475653	30/03/2020	North America / USA / California / Los Angeles	Human	Male	37	Released	Original		B.1	GH	USA	Undetermined	No (couldn't determine the patient status as either pre-symptomatic / asymptomatic or symptomatic conclusively)
hCoV-19/USA/CA-CSMC42/2020	EPI_ISL_475654	01/04/2020	North America / USA / California / Los Angeles	Human	Male	85	Released	Original		A.1	S	USA	Undetermined	No (couldn't determine the patient status as either pre-symptomatic / asymptomatic or symptomatic conclusively)
hCoV-19/USA/CA-CSMC43/2020	EPI_ISL_475655	01/04/2020	North America / USA / California / Los Angeles	Human	Female	50	Released	Original		B.1	GH	USA	Undetermined	No (couldn't determine the patient status as either pre-symptomatic / asymptomatic or symptomatic conclusively)
hCoV-19/USA/CA-CSMC44/2020	EPI_ISL_475656	31/03/2020	North America / USA / California / Los Angeles	Human	Male	49	Released	Original		B.1.43	GH	USA	Undetermined	No (couldn't determine the patient status as either pre-symptomatic / asymptomatic or symptomatic conclusively)
hCoV-19/USA/CA-CSMC45/2020	EPI_ISL_475657	31/03/2020	North America / USA / California / Los Angeles	Human	Male	59	Deceased	Original		B.1	GH	USA	Symptomatic	Yes
hCoV-19/USA/CA-CSMC46/2020	EPI_ISL_475658	09/04/2020	North America / USA / California / Los Angeles	Human	Male	56	Released	Original		B.1.43	GH	USA	Undetermined	No (couldn't determine the patient status as either pre-symptomatic / asymptomatic or symptomatic conclusively)
hCoV-19/USA/CA-CSMC47/2020	EPI_ISL_475659	09/04/2020	North America / USA / California / Los Angeles	Human	Female	40	Outpatient	Original		B.1	GH	USA	Undetermined	No (couldn't determine the patient status as either pre-symptomatic / asymptomatic or symptomatic conclusively)
hCoV-19/USA/CA-CSMC48/2020	EPI_ISL_475660	09/04/2020	North America / USA / California / Los Angeles	Human	Male	28	Outpatient	Original		B.1	GH	USA	Undetermined	No (couldn't determine the patient status as either pre-symptomatic / asymptomatic or symptomatic conclusively)
hCoV-19/USA/CA-CSMC49/2020	EPI_ISL_475661	01/04/2020	North America / USA / California / Los Angeles	Human	Male	76	Still hospitalized	Original		B.1	GH	USA	Undetermined	No (couldn't determine the patient status as either pre-symptomatic / asymptomatic or symptomatic conclusively)
hCoV-19/USA/CA-CSMC5/2020	EPI_ISL_475662	13/04/2020	North America / USA / California / Los Angeles	Human	Male	28	Outpatient	Original		B.1.43	GH	USA	Undetermined	No (couldn't determine the patient status as either pre-symptomatic / asymptomatic or symptomatic conclusively)
hCoV-19/USA/CA-CSMC50/2020	EPI_ISL_475663	06/04/2020	North America / USA / California / Los Angeles	Human	Male	90	Deceased	Original		B.1.1	GR	USA	Symptomatic	Yes
hCoV-19/USA/CA-CSMC84/2020	EPI_ISL_475700	26/03/2020	North America / USA / California / Los Angeles	Human	Female	36	Released	Original		B.1.1	GR	USA	Undetermined	No (couldn't determine the patient status as either pre-symptomatic / asymptomatic or symptomatic conclusively)
hCoV-19/USA/CA-CSMC85/2020	EPI_ISL_475701	04/04/2020	North America / USA / California / Los Angeles	Human	Female	44	Deceased	Original		B.1	GH	USA	Symptomatic	Yes
hCoV-19/USA/CA-CSMC86/2020	EPI_ISL_475702	04/04/2020	North America / USA / California / Los Angeles	Human	Female	100	Still hospitalized	Original		B.1.1	GR	USA	Undetermined	No (couldn't determine the patient status as either pre-symptomatic / asymptomatic or symptomatic conclusively)
hCoV-19/USA/CA-CSMC87/2020	EPI_ISL_475703	26/03/2020	North America / USA / California / Los Angeles	Human	Male	69	Released	Original		B.1	GH	USA	Undetermined	No (couldn't determine the patient status as either pre-symptomatic / asymptomatic or symptomatic conclusively)
hCoV-19/USA/CA-CSMC88/2020	EPI_ISL_475704	26/03/2020	North America / USA / California / Los Angeles	Human	Female	33	Outpatient	Original		A	O	USA	Undetermined	No (couldn't determine the patient status as either pre-symptomatic / asymptomatic or symptomatic conclusively)
hCoV-19/USA/CA-CSMC89/2020	EPI_ISL_475705	26/03/2020	North America / USA / California / Los Angeles	Human	Male	74	Released	Original		B.1	GH	USA	Undetermined	No (couldn't determine the patient status as either pre-symptomatic / asymptomatic or symptomatic conclusively)
hCoV-19/USA/CA-CSMC9/2020	EPI_ISL_475706	27/03/2020	North America / USA / California / Los Angeles	Human	Male	33	Released	Original		B.1	GH	USA	Undetermined	No (couldn't determine the patient status as either pre-symptomatic / asymptomatic or symptomatic conclusively)
hCoV-19/USA/CA-CSMC90/2020	EPI_ISL_475707	27/03/2020	North America / USA / California / Los Angeles	Human	Male	39	Released	Original		B.1	O	USA	Undetermined	No (couldn't determine the patient status as either pre-symptomatic / asymptomatic or symptomatic conclusively)
hCoV-19/USA/CA-CSMC91/2020	EPI_ISL_475708	27/03/2020	North America / USA / California / Los Angeles	Human	Male	52	Released	Original		A	S	USA	Undetermined	No (couldn't determine the patient status as either pre-symptomatic / asymptomatic or symptomatic conclusively)
hCoV-19/USA/CA-CSMC92/2020	EPI_ISL_475709	02/04/2020	North America / USA / California / Los Angeles	Human	Female	29	Released	Original		B.1	GH	USA	Undetermined	No (couldn't determine the patient status as either pre-symptomatic / asymptomatic or symptomatic conclusively)
hCoV-19/USA/CA-CSMC93/2020	EPI_ISL_475710	11/04/2020	North America / USA / California / Los Angeles	Human	Female	81	Deceased	Original		B.1.3	GH	USA	Symptomatic	Yes
hCoV-19/USA/CA-CSMC94/2020	EPI_ISL_475711	11/04/2020	North America / USA / California / Los Angeles	Human	Female	91	Released	Original		B.1	GH	USA	Undetermined	No (couldn't determine the patient status as either pre-symptomatic / asymptomatic or symptomatic conclusively)
hCoV-19/USA/CA-CSMC95/2020	EPI_ISL_475712	02/04/2020	North America / USA / California / Los Angeles	Human	Male	70	Outpatient	Original		B.4	O	USA	Undetermined	No (couldn't determine the patient status as either pre-symptomatic / asymptomatic or symptomatic conclusively)
hCoV-19/USA/CA-CSMC96/2020	EPI_ISL_475713	02/04/2020	North America / USA / California / Los Angeles	Human	Male	87	Deceased	Original		B.1.43	GH	USA	Symptomatic	Yes
hCoV-19/USA/CA-CSMC97/2020	EPI_ISL_475714	02/04/2020	North America / USA / California / Los Angeles	Human	Male	62	Released	Original		B.1	GH	USA	Undetermined	No (couldn't determine the patient status as either pre-symptomatic / asymptomatic or symptomatic conclusively)
hCoV-19/USA/CA-CSMC98/2020	EPI_ISL_475715	07/04/2020	North America / USA / California / Los Angeles	Human	Female	80	Deceased	Original		B.1	GH	USA	Symptomatic	Yes
hCoV-19/USA/CA-CSMC99/2020	EPI_ISL_475716	22/03/2020	North America / USA / California / Los Angeles	Human	Male	79	Released	Original		B.1	GH	USA	Undetermined	No (couldn't determine the patient status as either pre-symptomatic / asymptomatic or symptomatic conclusively)
hCoV-19/Egypt/MASRI-2/2020	EPI_ISL_475745	2020-05	Africa / Egypt	Human	Female	63	Alive	Original		B.1	GH	Egypt	Undetermined	No (couldn't determine the patient status as either pre-symptomatic / asymptomatic or symptomatic conclusively)
hCoV-19/Egypt/MASRI-3/2020	EPI_ISL_475746	2020-05	Africa / Egypt	Human	Male	41	Alive	Original		A	S	Egypt	Undetermined	No (couldn't determine the patient status as either pre-symptomatic / asymptomatic or symptomatic conclusively)
hCoV-19/Egypt/MASRI-6/2020	EPI_ISL_475747	2020-05	Africa / Egypt	Human	Male	54	Alive	Original		B.1	GH	Egypt	Undetermined	No (couldn't determine the patient status as either pre-symptomatic / asymptomatic or symptomatic conclusively)
hCoV-19/Egypt/MASRI-13/2020	EPI_ISL_475748	2020-05	Africa / Egypt	Human	Male	72	Alive	Original		B.1	GH	Egypt	Undetermined	No (couldn't determine the patient status as either pre-symptomatic / asymptomatic or symptomatic conclusively)
hCoV-19/Egypt/MASRI-14/2020	EPI_ISL_475749	2020-05	Africa / Egypt	Human	Female	35	Alive	Original		B.1	GH	Egypt	Undetermined	No (couldn't determine the patient status as either pre-symptomatic / asymptomatic or symptomatic conclusively)
hCoV-19/Egypt/MASRI-15/2020	EPI_ISL_475750	2020-05	Africa / Egypt	Human	Male	37	Alive	Original		B.1.1	GR	Egypt	Undetermined	No (couldn't determine the patient status as either pre-symptomatic / asymptomatic or symptomatic conclusively)
hCoV-19/Egypt/MASRI-9/2020	EPI_ISL_475751	2020-05	Africa / Egypt	Human	Male	50	Alive	Original		B.1.1.1	GR	Egypt	Undetermined	No (couldn't determine the patient status as either pre-symptomatic / asymptomatic or symptomatic conclusively)
hCoV-19/Egypt/MASRI-10/2020	EPI_ISL_475752	2020-05	Africa / Egypt	Human	Male	34	Alive	Original		B.1	GH	Egypt	Undetermined	No (couldn't determine the patient status as either pre-symptomatic / asymptomatic or symptomatic conclusively)
hCoV-19/Egypt/MASRI-11/2020	EPI_ISL_475753	2020-05	Africa / Egypt	Human	Female	70	Alive	Original		B.1.113	GH	Egypt	Undetermined	No (couldn't determine the patient status as either pre-symptomatic / asymptomatic or symptomatic conclusively)
hCoV-19/Italy/APU-UniMI-854/2020	EPI_ISL_477202	20/03/2020	Europe / Italy / Apulia	Human	Male	88	symptomatic	Original	Nasopharyngeal swab	B.1.1	GR	Italy	Symptomatic	Yes
hCoV-19/Japan/PG-0016/2020	EPI_ISL_479800	20/01/2020	Asia / Japan / Hokkaido	Human	unknown	unknown	Symptomatic	Original	Pharyngeal swab	B	L	Japan	Symptomatic	Yes
hCoV-19/Japan/PG-0022/2020	EPI_ISL_479801	2020-02	Asia / Japan / Hokkaido	Human	unknown	unknown	Symptomatic	Original	Pharyngeal swab	B	L	Japan	Symptomatic	Yes
hCoV-19/Japan/PG-0031/2020	EPI_ISL_479802	25/02/2020	Asia / Japan / Kanto	Human	unknown	unknown	Symptomatic	Original	Pharyngeal swab	B	L	Japan	Symptomatic	Yes
hCoV-19/Japan/PG-0033/2020	EPI_ISL_479803	28/02/2020	Asia / Japan / Kanto	Human	unknown	unknown	Symptomatic	Original	Pharyngeal swab	B	L	Japan	Symptomatic	Yes
hCoV-19/Japan/PG-0036/2020	EPI_ISL_479804	29/02/2020	Asia / Japan / Kanto	Human	unknown	unknown	Symptomatic	Original	Pharyngeal swab	B	L	Japan	Symptomatic	Yes
hCoV-19/Japan/PG-0038/2020	EPI_ISL_479805	04/03/2020	Asia / Japan / Kanto	Human	unknown	unknown	Symptomatic	Original	Pharyngeal swab	A	S	Japan	Symptomatic	Yes
hCoV-19/Japan/PG-0039/2020	EPI_ISL_479806	04/03/2020	Asia / Japan / Kanto	Human	unknown	unknown	Symptomatic	Original	Pharyngeal swab	A	S	Japan	Symptomatic	Yes
hCoV-19/Japan/PG-0040/2020	EPI_ISL_479807	05/03/2020	Asia / Japan / Kanto	Human	unknown	unknown	Symptomatic	Original	Pharyngeal swab	A	S	Japan	Symptomatic	Yes
hCoV-19/Japan/PG-0041/2020	EPI_ISL_479808	06/03/2020	Asia / Japan / Kanto	Human	unknown	unknown	Symptomatic	Original	Pharyngeal swab	B	L	Japan	Symptomatic	Yes
hCoV-19/Japan/PG-0045/2020	EPI_ISL_479809	2020-02	Asia / Japan / Kanto	Human	unknown	unknown	Symptomatic	Original	Pharyngeal swab	A	S	Japan	Symptomatic	Yes
hCoV-19/Japan/PG-0046/2020	EPI_ISL_479810	2020-02	Asia / Japan / Kanto	Human	unknown	unknown	Symptomatic	Original	Pharyngeal swab	A	S	Japan	Symptomatic	Yes
hCoV-19/Japan/PG-0048/2020	EPI_ISL_479811	2020-02	Asia / Japan / Kanto	Human	unknown	unknown	Symptomatic	Original	Pharyngeal swab	A	S	Japan	Symptomatic	Yes
hCoV-19/Japan/PG-0049/2020	EPI_ISL_479812	01/03/2020	Asia / Japan / Hokkaido	Human	unknown	unknown	Symptomatic	Original	Pharyngeal swab	B	L	Japan	Symptomatic	Yes
hCoV-19/Japan/PG-0050/2020	EPI_ISL_479813	03/03/2020	Asia / Japan / Hokkaido	Human	unknown	unknown	Symptomatic	Original	Pharyngeal swab	B	L	Japan	Symptomatic	Yes
hCoV-19/Japan/PG-0051/2020	EPI_ISL_479814	03/03/2020	Asia / Japan / Hokkaido	Human	unknown	unknown	Symptomatic	Original	Pharyngeal swab	B	L	Japan	Symptomatic	Yes
hCoV-19/Japan/PG-0052/2020	EPI_ISL_479815	05/03/2020	Asia / Japan / Hokkaido	Human	unknown	unknown	Symptomatic	Original	Pharyngeal swab	A	S	Japan	Symptomatic	Yes
hCoV-19/Japan/PG-0053/2020	EPI_ISL_479816	06/03/2020	Asia / Japan / Hokkaido	Human	unknown	unknown	Symptomatic	Original	Pharyngeal swab	B	L	Japan	Symptomatic	Yes
hCoV-19/Japan/PG-0054/2020	EPI_ISL_479817	07/03/2020	Asia / Japan / Hokkaido	Human	unknown	unknown	Symptomatic	Original	Pharyngeal swab	B	L	Japan	Symptomatic	Yes
hCoV-19/Japan/PG-0055/2020	EPI_ISL_479818	07/03/2020	Asia / Japan / Hokkaido	Human	unknown	unknown	Symptomatic	Original	Pharyngeal swab	B	L	Japan	Symptomatic	Yes
hCoV-19/Japan/PG-0056/2020	EPI_ISL_479819	08/03/2020	Asia / Japan / Hokkaido	Human	unknown	unknown	Symptomatic	Original	Pharyngeal swab	A	S	Japan	Symptomatic	Yes
hCoV-19/Japan/PG-0057/2020	EPI_ISL_479820	08/03/2020	Asia / Japan / Hokkaido	Human	unknown	unknown	Symptomatic	Original	Pharyngeal swab	A	S	Japan	Symptomatic	Yes
hCoV-19/Japan/PG-0058/2020	EPI_ISL_479821	02/03/2020	Asia / Japan / Kansai	Human	unknown	unknown	Symptomatic	Original	Pharyngeal swab	A	S	Japan	Symptomatic	Yes
hCoV-19/Japan/PG-0060/2020	EPI_ISL_479822	08/03/2020	Asia / Japan / Kansai	Human	unknown	unknown	Symptomatic	Original	Pharyngeal swab	A	S	Japan	Symptomatic	Yes
hCoV-19/Japan/PG-0063/2020	EPI_ISL_479823	01/03/2020	Asia / Japan / Chushikoku	Human	unknown	unknown	Symptomatic	Original	Pharyngeal swab	A	S	Japan	Symptomatic	Yes
hCoV-19/Japan/PG-0066/2020	EPI_ISL_479824	15/02/2020	Asia / Japan / Kyusyu	Human	unknown	unknown	Symptomatic	Original	Sputum	B	L	Japan	Symptomatic	Yes
hCoV-19/Japan/PG-0068/2020	EPI_ISL_479825	2020-02	Asia / Japan / Kanto	Human	unknown	unknown	Symptomatic	Original	Pharyngeal swab	B	O	Japan	Symptomatic	Yes
hCoV-19/Japan/PG-0071/2020	EPI_ISL_479826	26/02/2020	Asia / Japan / Hokkaido	Human	unknown	unknown	Symptomatic	Original	Pharyngeal swab	B	L	Japan	Symptomatic	Yes
hCoV-19/Japan/PG-0072/2020	EPI_ISL_479827	27/02/2020	Asia / Japan / Hokkaido	Human	unknown	unknown	Symptomatic	Original	Pharyngeal swab	B	L	Japan	Symptomatic	Yes
hCoV-19/Japan/PG-0074/2020	EPI_ISL_479828	27/02/2020	Asia / Japan / Hokkaido	Human	unknown	unknown	Symptomatic	Original	Pharyngeal swab	B	L	Japan	Symptomatic	Yes
hCoV-19/Japan/PG-0076/2020	EPI_ISL_479829	28/02/2020	Asia / Japan / Hokkaido	Human	unknown	unknown	Symptomatic	Original	Pharyngeal swab	B	L	Japan	Symptomatic	Yes
hCoV-19/Japan/PG-0077/2020	EPI_ISL_479830	28/02/2020	Asia / Japan / Hokkaido	Human	unknown	unknown	Symptomatic	Original	Pharyngeal swab	B	L	Japan	Symptomatic	Yes
hCoV-19/Japan/PG-0080/2020	EPI_ISL_479831	02/03/2020	Asia / Japan / Hokkaido	Human	unknown	unknown	Symptomatic	Original	Pharyngeal swab	B	L	Japan	Symptomatic	Yes
hCoV-19/Japan/PG-0081/2020	EPI_ISL_479832	02/03/2020	Asia / Japan / Hokkaido	Human	unknown	unknown	Symptomatic	Original	Pharyngeal swab	B	L	Japan	Symptomatic	Yes
hCoV-19/Japan/PG-0082/2020	EPI_ISL_479833	02/03/2020	Asia / Japan / Hokkaido	Human	unknown	unknown	Symptomatic	Original	Pharyngeal swab	A	S	Japan	Symptomatic	Yes
hCoV-19/Japan/PG-0084/2020	EPI_ISL_479834	04/03/2020	Asia / Japan / Hokkaido	Human	unknown	unknown	Symptomatic	Original	Pharyngeal swab	B	L	Japan	Symptomatic	Yes
hCoV-19/Japan/PG-0087/2020	EPI_ISL_479835	04/03/2020	Asia / Japan / Hokkaido	Human	unknown	unknown	Symptomatic	Original	Pharyngeal swab	A	S	Japan	Symptomatic	Yes
hCoV-19/Japan/PG-0089/2020	EPI_ISL_479836	05/03/2020	Asia / Japan / Hokkaido	Human	unknown	unknown	Symptomatic	Original	Pharyngeal swab	B	L	Japan	Symptomatic	Yes
hCoV-19/Japan/PG-0091/2020	EPI_ISL_479837	06/03/2020	Asia / Japan / Hokkaido	Human	unknown	unknown	Symptomatic	Original	Pharyngeal swab	B	L	Japan	Symptomatic	Yes
hCoV-19/Japan/PG-0092/2020	EPI_ISL_479838	06/03/2020	Asia / Japan / Hokkaido	Human	unknown	unknown	Symptomatic	Original	Pharyngeal swab	B	L	Japan	Symptomatic	Yes
hCoV-19/Japan/PG-0095/2020	EPI_ISL_479839	07/03/2020	Asia / Japan / Hokkaido	Human	unknown	unknown	Symptomatic	Original	Pharyngeal swab	B	L	Japan	Symptomatic	Yes
hCoV-19/Japan/PG-0097/2020	EPI_ISL_479840	07/03/2020	Asia / Japan / Hokkaido	Human	unknown	unknown	Symptomatic	Original	Pharyngeal swab	B	L	Japan	Symptomatic	Yes
hCoV-19/Japan/PG-0098/2020	EPI_ISL_479841	08/03/2020	Asia / Japan / Hokkaido	Human	unknown	unknown	Symptomatic	Original	Pharyngeal swab	B	O	Japan	Symptomatic	Yes
hCoV-19/Japan/PG-0099/2020	EPI_ISL_479842	08/03/2020	Asia / Japan / Hokkaido	Human	unknown	unknown	Symptomatic	Original	Pharyngeal swab	B	L	Japan	Symptomatic	Yes
hCoV-19/Japan/PG-0100/2020	EPI_ISL_479843	09/03/2020	Asia / Japan / Hokkaido	Human	unknown	unknown	Symptomatic	Original	Pharyngeal swab	B	L	Japan	Symptomatic	Yes
hCoV-19/Japan/PG-0104/2020	EPI_ISL_479844	09/03/2020	Asia / Japan / Hokkaido	Human	unknown	unknown	Symptomatic	Original	Pharyngeal swab	B	L	Japan	Symptomatic	Yes
hCoV-19/Japan/PG-0106/2020	EPI_ISL_479845	09/03/2020	Asia / Japan / Hokkaido	Human	unknown	unknown	Symptomatic	Original	Pharyngeal swab	B	L	Japan	Symptomatic	Yes
hCoV-19/Japan/PG-0108/2020	EPI_ISL_479846	09/03/2020	Asia / Japan / Hokkaido	Human	unknown	unknown	Symptomatic	Original	Pharyngeal swab	B	L	Japan	Symptomatic	Yes
hCoV-19/Japan/PG-0110/2020	EPI_ISL_479847	10/03/2020	Asia / Japan / Hokkaido	Human	unknown	unknown	Symptomatic	Original	Pharyngeal swab	B	L	Japan	Symptomatic	Yes
hCoV-19/Japan/PG-0111/2020	EPI_ISL_479848	10/03/2020	Asia / Japan / Hokkaido	Human	unknown	unknown	Symptomatic	Original	Pharyngeal swab	B	L	Japan	Symptomatic	Yes
hCoV-19/Japan/PG-0113/2020	EPI_ISL_479849	10/03/2020	Asia / Japan / Hokkaido	Human	unknown	unknown	Symptomatic	Original	Pharyngeal swab	B	L	Japan	Symptomatic	Yes
hCoV-19/Japan/PG-0114/2020	EPI_ISL_479850	13/02/2020	Asia / Japan / Kanto	Human	unknown	unknown	Symptomatic	Original	Pharyngeal swab	B	O	Japan	Symptomatic	Yes
hCoV-19/Japan/PG-0115/2020	EPI_ISL_479851	17/02/2020	Asia / Japan / Kanto	Human	unknown	unknown	Symptomatic	Original	Pharyngeal swab	B	O	Japan	Symptomatic	Yes
hCoV-19/Japan/PG-0119/2020	EPI_ISL_479852	25/02/2020	Asia / Japan / Kanto	Human	unknown	unknown	Symptomatic	Original	Pharyngeal swab	B	O	Japan	Symptomatic	Yes
hCoV-19/Japan/PG-0121/2020	EPI_ISL_479853	11/03/2020	Asia / Japan / Kanto	Human	unknown	unknown	Symptomatic	Original	Pharyngeal swab	B.1.36	GH	Japan	Symptomatic	Yes
hCoV-19/Japan/PG-0122/2020	EPI_ISL_479854	11/03/2020	Asia / Japan / Kanto	Human	unknown	unknown	Symptomatic	Original	Pharyngeal swab	B.1.36	GH	Japan	Symptomatic	Yes
hCoV-19/Japan/PG-0128/2020	EPI_ISL_479855	12/03/2020	Asia / Japan / Kansai	Human	unknown	unknown	Symptomatic	Original	Pharyngeal swab	A	S	Japan	Symptomatic	Yes
hCoV-19/Japan/PG-0130/2020	EPI_ISL_479856	12/03/2020	Asia / Japan / Kansai	Human	unknown	unknown	Symptomatic	Original	Pharyngeal swab	A	S	Japan	Symptomatic	Yes
hCoV-19/Japan/PG-0131/2020	EPI_ISL_479857	12/03/2020	Asia / Japan / Kansai	Human	unknown	unknown	Symptomatic	Original	Pharyngeal swab	A	S	Japan	Symptomatic	Yes
hCoV-19/Japan/PG-0132/2020	EPI_ISL_479858	12/03/2020	Asia / Japan / Kansai	Human	unknown	unknown	Symptomatic	Original	Pharyngeal swab	A	S	Japan	Symptomatic	Yes
hCoV-19/Japan/PG-0133/2020	EPI_ISL_479859	12/03/2020	Asia / Japan / Kansai	Human	unknown	unknown	Symptomatic	Original	Pharyngeal swab	A	S	Japan	Symptomatic	Yes
hCoV-19/Japan/PG-0136/2020	EPI_ISL_479860	14/03/2020	Asia / Japan / Kansai	Human	unknown	unknown	Symptomatic	Original	Pharyngeal swab	A	S	Japan	Symptomatic	Yes
hCoV-19/Japan/PG-0138/2020	EPI_ISL_479861	15/03/2020	Asia / Japan / Kansai	Human	unknown	unknown	Symptomatic	Original	Pharyngeal swab	B.6	O	Japan	Symptomatic	Yes
hCoV-19/Japan/PG-0139/2020	EPI_ISL_479862	12/03/2020	Asia / Japan / Kansai	Human	unknown	unknown	Symptomatic	Original	Pharyngeal swab	B	O	Japan	Symptomatic	Yes
hCoV-19/Japan/PG-0140/2020	EPI_ISL_479863	17/02/2020	Asia / Japan / Kansai	Human	unknown	unknown	Symptomatic	Original	Pharyngeal swab	B	O	Japan	Symptomatic	Yes
hCoV-19/Japan/PG-0141/2020	EPI_ISL_479864	23/02/2020	Asia / Japan / Kansai	Human	unknown	unknown	Symptomatic	Original	Pharyngeal swab	B	L	Japan	Symptomatic	Yes
hCoV-19/Japan/PG-0143/2020	EPI_ISL_479865	2020-02	Asia / Japan / Kansai	Human	unknown	unknown	Symptomatic	Original	Pharyngeal swab	B	L	Japan	Symptomatic	Yes
hCoV-19/Japan/PG-0144/2020	EPI_ISL_479866	2020-02	Asia / Japan / Kansai	Human	unknown	unknown	Symptomatic	Original	Pharyngeal swab	B	L	Japan	Symptomatic	Yes
hCoV-19/Japan/PG-0145/2020	EPI_ISL_479867	2020-02	Asia / Japan / Kansai	Human	unknown	unknown	Symptomatic	Original	Pharyngeal swab	B	L	Japan	Symptomatic	Yes
hCoV-19/Japan/PG-0153/2020	EPI_ISL_479868	16/03/2020	Asia / Japan / Kansai	Human	unknown	unknown	Symptomatic	Original	Pharyngeal swab	A	S	Japan	Symptomatic	Yes
hCoV-19/Japan/PG-0158/2020	EPI_ISL_479869	20/03/2020	Asia / Japan / Kanto	Human	unknown	unknown	Symptomatic	Original	Pharyngeal swab	A	S	Japan	Symptomatic	Yes
hCoV-19/Japan/PG-0159/2020	EPI_ISL_479870	21/02/2020	Asia / Japan / Kanto	Human	unknown	unknown	Symptomatic	Original	Pharyngeal swab	B	L	Japan	Symptomatic	Yes
hCoV-19/Japan/PG-0160/2020	EPI_ISL_479871	25/02/2020	Asia / Japan / Kanto	Human	unknown	unknown	Symptomatic	Original	Pharyngeal swab	B	L	Japan	Symptomatic	Yes
hCoV-19/Japan/PG-0162/2020	EPI_ISL_479872	11/03/2020	Asia / Japan / Hokkaido	Human	unknown	unknown	Symptomatic	Original	Pharyngeal swab	B	L	Japan	Symptomatic	Yes
hCoV-19/Japan/PG-0163/2020	EPI_ISL_479873	12/03/2020	Asia / Japan / Hokkaido	Human	unknown	unknown	Symptomatic	Original	Pharyngeal swab	B	O	Japan	Symptomatic	Yes
hCoV-19/Japan/PG-0164/2020	EPI_ISL_479874	12/03/2020	Asia / Japan / Hokkaido	Human	unknown	unknown	Symptomatic	Original	Pharyngeal swab	B	O	Japan	Symptomatic	Yes
hCoV-19/Japan/PG-0165/2020	EPI_ISL_479875	12/03/2020	Asia / Japan / Hokkaido	Human	unknown	unknown	Symptomatic	Original	Pharyngeal swab	B	L	Japan	Symptomatic	Yes
hCoV-19/Japan/PG-0166/2020	EPI_ISL_479876	12/03/2020	Asia / Japan / Hokkaido	Human	unknown	unknown	Symptomatic	Original	Pharyngeal swab	B	L	Japan	Symptomatic	Yes
hCoV-19/Japan/PG-0167/2020	EPI_ISL_479877	12/03/2020	Asia / Japan / Hokkaido	Human	unknown	unknown	Symptomatic	Original	Pharyngeal swab	B	L	Japan	Symptomatic	Yes
hCoV-19/Japan/PG-0168/2020	EPI_ISL_479878	13/03/2020	Asia / Japan / Hokkaido	Human	unknown	unknown	Symptomatic	Original	Pharyngeal swab	B	L	Japan	Symptomatic	Yes
hCoV-19/Japan/PG-0170/2020	EPI_ISL_479879	14/03/2020	Asia / Japan / Hokkaido	Human	unknown	unknown	Symptomatic	Original	Pharyngeal swab	B	L	Japan	Symptomatic	Yes
hCoV-19/Japan/PG-0171/2020	EPI_ISL_479880	14/03/2020	Asia / Japan / Hokkaido	Human	unknown	unknown	Symptomatic	Original	Pharyngeal swab	B.1.1	GR	Japan	Symptomatic	No (randomly sampled out due to data redundancy from Japan)
hCoV-19/Japan/PG-0172/2020	EPI_ISL_479881	15/03/2020	Asia / Japan / Hokkaido	Human	unknown	unknown	Symptomatic	Original	Pharyngeal swab	B	L	Japan	Symptomatic	Yes
hCoV-19/Japan/PG-0173/2020	EPI_ISL_479882	17/03/2020	Asia / Japan / Hokkaido	Human	unknown	unknown	Symptomatic	Original	Pharyngeal swab	B.1.1	GR	Japan	Symptomatic	No (randomly sampled out due to data redundancy from Japan)
hCoV-19/Japan/PG-0174/2020	EPI_ISL_479883	18/03/2020	Asia / Japan / Hokkaido	Human	unknown	unknown	Symptomatic	Original	Pharyngeal swab	B.1	GH	Japan	Symptomatic	Yes
hCoV-19/Japan/PG-0175/2020	EPI_ISL_479884	18/03/2020	Asia / Japan / Hokkaido	Human	unknown	unknown	Symptomatic	Original	Pharyngeal swab	B.1	GH	Japan	Symptomatic	Yes

hCoV-19/Japan/PG-0176/2020	EPI_ISL_479885	19/03/2020	Asia / Japan / Hokkaido	Human	unknown	unknown	Symptomatic	Original	Pharyngeal swab	B.1	GH	Japan	Symptomatic	Yes
hCoV-19/Japan/PG-0178/2020	EPI_ISL_479886	05/03/2020	Asia / Japan / Kanto	Human	unknown	unknown	Symptomatic	Original	Pharyngeal swab	B.21	L	Japan	Symptomatic	Yes
hCoV-19/Japan/PG-0179/2020	EPI_ISL_479887	05/03/2020	Asia / Japan / Kanto	Human	unknown	unknown	Symptomatic	Original	Pharyngeal swab	B.21	L	Japan	Symptomatic	Yes
hCoV-19/Japan/PG-0180/2020	EPI_ISL_479888	05/03/2020	Asia / Japan / Kanto	Human	unknown	unknown	Symptomatic	Original	Pharyngeal swab	A	S	Japan	Symptomatic	Yes
hCoV-19/Japan/PG-0181/2020	EPI_ISL_479889	06/03/2020	Asia / Japan / Kanto	Human	unknown	unknown	Symptomatic	Original	Pharyngeal swab	B	L	Japan	Symptomatic	Yes
hCoV-19/Japan/PG-0182/2020	EPI_ISL_479890	03/03/2020	Asia / Japan / Kanto	Human	unknown	unknown	Symptomatic	Original	Pharyngeal swab	A	S	Japan	Symptomatic	Yes
hCoV-19/Japan/PG-0185/2020	EPI_ISL_479891	16/03/2020	Asia / Japan / Kanto	Human	unknown	unknown	Symptomatic	Original	Pharyngeal swab	B.1.1	GR	Japan	Symptomatic	No (randomly sampled out due to data redundancy from Japan)
hCoV-19/Japan/PG-0187/2020	EPI_ISL_479892	16/03/2020	Asia / Japan / Kanto	Human	unknown	unknown	Symptomatic	Original	Pharyngeal swab	A	S	Japan	Symptomatic	Yes
hCoV-19/Japan/PG-0188/2020	EPI_ISL_479893	16/03/2020	Asia / Japan / Kanto	Human	unknown	unknown	Symptomatic	Original	Pharyngeal swab	B.1.1	GR	Japan	Symptomatic	No (randomly sampled out due to data redundancy from Japan)
hCoV-19/Japan/PG-0189/2020	EPI_ISL_479894	16/03/2020	Asia / Japan / Kanto	Human	unknown	unknown	Symptomatic	Original	Pharyngeal swab	B.1.1	GR	Japan	Symptomatic	No (randomly sampled out due to data redundancy from Japan)
hCoV-19/Japan/PG-0190/2020	EPI_ISL_479895	16/03/2020	Asia / Japan / Kanto	Human	unknown	unknown	Symptomatic	Original	Pharyngeal swab	B.1.1	GR	Japan	Symptomatic	No (randomly sampled out due to data redundancy from Japan)
hCoV-19/Japan/PG-0191/2020	EPI_ISL_479896	13/03/2020	Asia / Japan / Kanto	Human	unknown	unknown	Symptomatic	Original	Pharyngeal swab	B.1.36	GH	Japan	Symptomatic	Yes
hCoV-19/Japan/PG-0192/2020	EPI_ISL_479897	13/03/2020	Asia / Japan / Kanto	Human	unknown	unknown	Symptomatic	Original	Pharyngeal swab	B.1.36	GH	Japan	Symptomatic	Yes
hCoV-19/Japan/PG-0193/2020	EPI_ISL_479898	16/03/2020	Asia / Japan / Kanto	Human	unknown	unknown	Symptomatic	Original	Pharyngeal swab	B.1.36	GH	Japan	Symptomatic	Yes
hCoV-19/Japan/PG-0194/2020	EPI_ISL_479899	16/03/2020	Asia / Japan / Kanto	Human	unknown	unknown	Symptomatic	Original	Pharyngeal swab	B.1.36	GH	Japan	Symptomatic	Yes
hCoV-19/Japan/PG-0434/2020	EPI_ISL_480100	05/04/2020	Asia / Japan / Kansai	Human	unknown	unknown	Symptomatic	Original	Pharyngeal swab	B.1.1	GR	Japan	Symptomatic	No (randomly sampled out due to data redundancy from Japan)
hCoV-19/Japan/PG-0435/2020	EPI_ISL_480101	05/04/2020	Asia / Japan / Kansai	Human	unknown	unknown	Symptomatic	Original	Pharyngeal swab	B.1.1	GR	Japan	Symptomatic	No (randomly sampled out due to data redundancy from Japan)
hCoV-19/Japan/PG-0436/2020	EPI_ISL_480102	06/04/2020	Asia / Japan / Kansai	Human	unknown	unknown	Symptomatic	Original	Pharyngeal swab	B.1.1	GR	Japan	Symptomatic	No (randomly sampled out due to data redundancy from Japan)
hCoV-19/Japan/PG-0437/2020	EPI_ISL_480103	12/03/2020	Asia / Japan / Kanto	Human	unknown	unknown	Symptomatic	Original	Pharyngeal swab	A	S	Japan	Symptomatic	Yes
hCoV-19/Japan/PG-0439/2020	EPI_ISL_480104	13/03/2020	Asia / Japan / Kanto	Human	unknown	unknown	Symptomatic	Original	Pharyngeal swab	A	S	Japan	Symptomatic	Yes
hCoV-19/Japan/PG-0440/2020	EPI_ISL_480105	13/03/2020	Asia / Japan / Kanto	Human	unknown	unknown	Symptomatic	Original	Pharyngeal swab	A	S	Japan	Symptomatic	Yes
hCoV-19/Japan/PG-0441/2020	EPI_ISL_480106	19/03/2020	Asia / Japan / Kanto	Human	unknown	unknown	Symptomatic	Original	Pharyngeal swab	B.1	GH	Japan	Symptomatic	Yes
hCoV-19/Japan/PG-0442/2020	EPI_ISL_480107	19/03/2020	Asia / Japan / Kanto	Human	unknown	unknown	Symptomatic	Original	Pharyngeal swab	B.1	GH	Japan	Symptomatic	Yes
hCoV-19/Japan/PG-0443/2020	EPI_ISL_480108	30/03/2020	Asia / Japan / Kanto	Human	unknown	unknown	Symptomatic	Original	Pharyngeal swab	B.1.1	GR	Japan	Symptomatic	No (randomly sampled out due to data redundancy from Japan)
hCoV-19/Japan/PG-0445/2020	EPI_ISL_480109	19/03/2020	Asia / Japan / Kyusyu	Human	unknown	unknown	Symptomatic	Original	Pharyngeal swab	A	S	Japan	Symptomatic	Yes
hCoV-19/Japan/PG-0446/2020	EPI_ISL_480110	19/03/2020	Asia / Japan / Kyusyu	Human	unknown	unknown	Symptomatic	Original	Pharyngeal swab	A	S	Japan	Symptomatic	Yes
hCoV-19/Japan/PG-0448/2020	EPI_ISL_480111	21/03/2020	Asia / Japan / Kyusyu	Human	unknown	unknown	Symptomatic	Original	Pharyngeal swab	A	S	Japan	Symptomatic	Yes
hCoV-19/Japan/PG-0449/2020	EPI_ISL_480112	21/03/2020	Asia / Japan / Kyusyu	Human	unknown	unknown	Symptomatic	Original	Pharyngeal swab	A	S	Japan	Symptomatic	Yes
hCoV-19/Japan/PG-0450/2020	EPI_ISL_480113	21/03/2020	Asia / Japan / Kyusyu	Human	unknown	unknown	Symptomatic	Original	Pharyngeal swab	A	S	Japan	Symptomatic	Yes
hCoV-19/Japan/PG-0452/2020	EPI_ISL_480114	21/03/2020	Asia / Japan / Kyusyu	Human	unknown	unknown	Symptomatic	Original	Pharyngeal swab	A	S	Japan	Symptomatic	Yes
hCoV-19/Japan/PG-0453/2020	EPI_ISL_480115	21/03/2020	Asia / Japan / Kyusyu	Human	unknown	unknown	Symptomatic	Original	Pharyngeal swab	A	S	Japan	Symptomatic	Yes
hCoV-19/Japan/PG-0454/2020	EPI_ISL_480116	21/03/2020	Asia / Japan / Kyusyu	Human	unknown	unknown	Symptomatic	Original	Pharyngeal swab	A	S	Japan	Symptomatic	Yes
hCoV-19/Japan/PG-0455/2020	EPI_ISL_480117	19/03/2020	Asia / Japan / Kyusyu	Human	unknown	unknown	Symptomatic	Original	Pharyngeal swab	A	S	Japan	Symptomatic	Yes
hCoV-19/Japan/PG-0457/2020	EPI_ISL_480118	27/03/2020	Asia / Japan / Kyusyu	Human	unknown	unknown	Symptomatic	Original	Pharyngeal swab	B.6	L	Japan	Symptomatic	Yes
hCoV-19/Japan/PG-0458/2020	EPI_ISL_480119	29/03/2020	Asia / Japan / Kyusyu	Human	unknown	unknown	Symptomatic	Original	Pharyngeal swab	B.1.1	GR	Japan	Symptomatic	No (randomly sampled out due to data redundancy from Japan)
hCoV-19/Japan/PG-0459/2020	EPI_ISL_480120	30/03/2020	Asia / Japan / HokurikuShinsyu	Human	unknown	unknown	Symptomatic	Original	Pharyngeal swab	B.1.1	GR	Japan	Symptomatic	No (randomly sampled out due to data redundancy from Japan)
hCoV-19/Japan/PG-0460/2020	EPI_ISL_480121	30/03/2020	Asia / Japan / HokurikuShinsyu	Human	unknown	unknown	Symptomatic	Original	Pharyngeal swab	B.1.1	GR	Japan	Symptomatic	No (randomly sampled out due to data redundancy from Japan)
hCoV-19/Japan/PG-0461/2020	EPI_ISL_480122	30/03/2020	Asia / Japan / HokurikuShinsyu	Human	unknown	unknown	Symptomatic	Original	Pharyngeal swab	B.1.1	GR	Japan	Symptomatic	No (randomly sampled out due to data redundancy from Japan)
hCoV-19/Japan/PG-0462/2020	EPI_ISL_480123	30/03/2020	Asia / Japan / HokurikuShinsyu	Human	unknown	unknown	Symptomatic	Original	Pharyngeal swab	B.1.1	GR	Japan	Symptomatic	No (randomly sampled out due to data redundancy from Japan)
hCoV-19/Japan/PG-0463/2020	EPI_ISL_480124	30/03/2020	Asia / Japan / HokurikuShinsyu	Human	unknown	unknown	Symptomatic	Original	Pharyngeal swab	B.1.1	GR	Japan	Symptomatic	No (randomly sampled out due to data redundancy from Japan)
hCoV-19/Japan/PG-0464/2020	EPI_ISL_480125	31/03/2020	Asia / Japan / HokurikuShinsyu	Human	unknown	unknown	Symptomatic	Original	Pharyngeal swab	B.1.1	GR	Japan	Symptomatic	No (randomly sampled out due to data redundancy from Japan)
hCoV-19/Japan/PG-0465/2020	EPI_ISL_480126	07/04/2020	Asia / Japan / HokurikuShinsyu	Human	unknown	unknown	Symptomatic	Original	Pharyngeal swab	B.1.1	GR	Japan	Symptomatic	No (randomly sampled out due to data redundancy from Japan)
hCoV-19/Japan/PG-0466/2020	EPI_ISL_480127	01/04/2020	Asia / Japan / HokurikuShinsyu	Human	unknown	unknown	Symptomatic	Original	Pharyngeal swab	B.1.1	GR	Japan	Symptomatic	No (randomly sampled out due to data redundancy from Japan)
hCoV-19/Japan/PG-0468/2020	EPI_ISL_480128	31/03/2020	Asia / Japan / HokurikuShinsyu	Human	unknown	unknown	Symptomatic	Original	Pharyngeal swab	B.1.1	GR	Japan	Symptomatic	No (randomly sampled out due to data redundancy from Japan)
hCoV-19/Japan/PG-0469/2020	EPI_ISL_480129	31/03/2020	Asia / Japan / HokurikuShinsyu	Human	unknown	unknown	Symptomatic	Original	Pharyngeal swab	B.1.1	GR	Japan	Symptomatic	No (randomly sampled out due to data redundancy from Japan)
hCoV-19/Japan/PG-0470/2020	EPI_ISL_480130	01/04/2020	Asia / Japan / HokurikuShinsyu	Human	unknown	unknown	Symptomatic	Original	Pharyngeal swab	B.1.1	GR	Japan	Symptomatic	No (randomly sampled out due to data redundancy from Japan)
hCoV-19/Japan/PG-0471/2020	EPI_ISL_480131	01/04/2020	Asia / Japan / HokurikuShinsyu	Human	unknown	unknown	Symptomatic	Original	Pharyngeal swab	B.1.1	GR	Japan	Symptomatic	No (randomly sampled out due to data redundancy from Japan)
hCoV-19/Japan/PG-0472/2020	EPI_ISL_480132	01/04/2020	Asia / Japan / HokurikuShinsyu	Human	unknown	unknown	Symptomatic	Original	Pharyngeal swab	B.1.1	GR	Japan	Symptomatic	No (randomly sampled out due to data redundancy from Japan)
hCoV-19/Japan/PG-0473/2020	EPI_ISL_480133	01/04/2020	Asia / Japan / HokurikuShinsyu	Human	unknown	unknown	Symptomatic	Original	Pharyngeal swab	B.1.1	GR	Japan	Symptomatic	No (randomly sampled out due to data redundancy from Japan)
hCoV-19/Japan/PG-0474/2020	EPI_ISL_480134	01/04/2020	Asia / Japan / HokurikuShinsyu	Human	unknown	unknown	Symptomatic	Original	Pharyngeal swab	B.1.1	GR	Japan	Symptomatic	No (randomly sampled out due to data redundancy from Japan)
hCoV-19/Japan/PG-0475/2020	EPI_ISL_480135	02/04/2020	Asia / Japan / HokurikuShinsyu	Human	unknown	unknown	Symptomatic	Original	Pharyngeal swab	B.1.1	GR	Japan	Symptomatic	No (randomly sampled out due to data redundancy from Japan)
hCoV-19/Japan/PG-0476/2020	EPI_ISL_480136	02/04/2020	Asia / Japan / HokurikuShinsyu	Human	unknown	unknown	Symptomatic	Original	Pharyngeal swab	B.1.1	GR	Japan	Symptomatic	No (randomly sampled out due to data redundancy from Japan)
hCoV-19/Japan/PG-0477/2020	EPI_ISL_480137	02/04/2020	Asia / Japan / HokurikuShinsyu	Human	unknown	unknown	Symptomatic	Original	Pharyngeal swab	B.1.1	GR	Japan	Symptomatic	No (randomly sampled out due to data redundancy from Japan)
hCoV-19/Japan/PG-0478/2020	EPI_ISL_480138	02/04/2020	Asia / Japan / HokurikuShinsyu	Human	unknown	unknown	Symptomatic	Original	Pharyngeal swab	B.1.1	GR	Japan	Symptomatic	No (randomly sampled out due to data redundancy from Japan)
hCoV-19/Japan/PG-0480/2020	EPI_ISL_480139	02/04/2020	Asia / Japan / HokurikuShinsyu	Human	unknown	unknown	Symptomatic	Original	Pharyngeal swab	B.1.1	GR	Japan	Symptomatic	Yes
hCoV-19/Japan/PG-0481/2020	EPI_ISL_480140	02/04/2020	Asia / Japan / HokurikuShinsyu	Human	unknown	unknown	Symptomatic	Original	Pharyngeal swab	B.1.1	GR	Japan	Symptomatic	No (randomly sampled out due to data redundancy from Japan)
hCoV-19/Japan/PG-0482/2020	EPI_ISL_480141	03/04/2020	Asia / Japan / HokurikuShinsyu	Human	unknown	unknown	Symptomatic	Original	Pharyngeal swab	B.1.1	GR	Japan	Symptomatic	No (randomly sampled out due to data redundancy from Japan)
hCoV-19/Japan/PG-0483/2020	EPI_ISL_480142	03/04/2020	Asia / Japan / HokurikuShinsyu	Human	unknown	unknown	Symptomatic	Original	Pharyngeal swab	B.1.1	GR	Japan	Symptomatic	No (randomly sampled out due to data redundancy from Japan)
hCoV-19/Japan/PG-0484/2020	EPI_ISL_480143	03/04/2020	Asia / Japan / HokurikuShinsyu	Human	unknown	unknown	Symptomatic	Original	Pharyngeal swab	B.1.1	GR	Japan	Symptomatic	No (randomly sampled out due to data redundancy from Japan)
hCoV-19/Japan/PG-0485/2020	EPI_ISL_480144	04/04/2020	Asia / Japan / HokurikuShinsyu	Human	unknown	unknown	Symptomatic	Original	Pharyngeal swab	B.1.1	GR	Japan	Symptomatic	No (randomly sampled out due to data redundancy from Japan)
hCoV-19/Japan/PG-0486/2020	EPI_ISL_480145	04/04/2020	Asia / Japan / HokurikuShinsyu	Human	unknown	unknown	Symptomatic	Original	Pharyngeal swab	B.1.1	GR	Japan	Symptomatic	No (randomly sampled out due to data redundancy from Japan)
hCoV-19/Japan/PG-0487/2020	EPI_ISL_480146	04/04/2020	Asia / Japan / HokurikuShinsyu	Human	unknown	unknown	Symptomatic	Original	Pharyngeal swab	B.1.1	GR	Japan	Symptomatic	No (randomly sampled out due to data redundancy from Japan)
hCoV-19/Japan/PG-0488/2020	EPI_ISL_480147	06/04/2020	Asia / Japan / HokurikuShinsyu	Human	unknown	unknown	Symptomatic	Original	Pharyngeal swab	B.1.1	GR	Japan	Symptomatic	No (randomly sampled out due to data redundancy from Japan)
hCoV-19/Japan/PG-0489/2020	EPI_ISL_480148	05/04/2020	Asia / Japan / HokurikuShinsyu	Human	unknown	unknown	Symptomatic	Original	Pharyngeal swab	B.1.1	GR	Japan	Symptomatic	No (randomly sampled out due to data redundancy from Japan)
hCoV-19/Japan/PG-0490/2020	EPI_ISL_480149	05/04/2020	Asia / Japan / HokurikuShinsyu	Human	unknown	unknown	Symptomatic	Original	Pharyngeal swab	B.1.1	GR	Japan	Symptomatic	No (randomly sampled out due to data redundancy from Japan)
hCoV-19/Japan/PG-0491/2020	EPI_ISL_480150	05/04/2020	Asia / Japan / HokurikuShinsyu	Human	unknown	unknown	Symptomatic	Original	Pharyngeal swab	B.1.1	GR	Japan	Symptomatic	No (randomly sampled out due to data redundancy from Japan)
hCoV-19/Japan/PG-0492/2020	EPI_ISL_480151	06/04/2020	Asia / Japan / HokurikuShinsyu	Human	unknown	unknown	Symptomatic	Original	Pharyngeal swab	B.1.1	GR	Japan	Symptomatic	No (randomly sampled out due to data redundancy from Japan)
hCoV-19/Japan/PG-0493/2020	EPI_ISL_480152	06/04/2020	Asia / Japan / HokurikuShinsyu	Human	unknown	unknown	Symptomatic	Original	Pharyngeal swab	B.1.1	GR	Japan	Symptomatic	No (randomly sampled out due to data redundancy from Japan)
hCoV-19/Japan/PG-0494/2020	EPI_ISL_480153	07/04/2020	Asia / Japan / HokurikuShinsyu	Human	unknown	unknown	Symptomatic	Original	Pharyngeal swab	B.1.1	GR	Japan	Symptomatic	No (randomly sampled out due to data redundancy from Japan)
hCoV-19/Japan/PG-0495/2020	EPI_ISL_480154	06/04/2020	Asia / Japan / HokurikuShinsyu	Human	unknown	unknown	Symptomatic	Original	Pharyngeal swab	B.1.1	GR	Japan	Symptomatic	No (randomly sampled out due to data redundancy from Japan)
hCoV-19/Japan/PG-0496/2020	EPI_ISL_480155	06/04/2020	Asia / Japan / HokurikuShinsyu	Human	unknown	unknown	Symptomatic	Original	Pharyngeal swab	B.1.1	GR	Japan	Symptomatic	No (randomly sampled out due to data redundancy from Japan)
hCoV-19/Japan/PG-0497/2020	EPI_ISL_480156	06/04/2020	Asia / Japan / HokurikuShinsyu	Human	unknown	unknown	Symptomatic	Original	Pharyngeal swab	B.1.1	GR	Japan	Symptomatic	No (randomly sampled out due to data redundancy from Japan)
hCoV-19/Japan/PG-0498/2020	EPI_ISL_480157	07/04/2020	Asia / Japan / HokurikuShinsyu	Human	unknown	unknown	Symptomatic	Original	Pharyngeal swab	B.1.1	GR	Japan	Symptomatic	No (randomly sampled out due to data redundancy from Japan)
hCoV-19/Japan/PG-0499/2020	EPI_ISL_480158	06/04/2020	Asia / Japan / HokurikuShinsyu	Human	unknown	unknown	Symptomatic	Original	Pharyngeal swab	B.1.1	GR	Japan	Symptomatic	No (randomly sampled out due to data redundancy from Japan)
hCoV-19/Japan/PG-0500/2020	EPI_ISL_480159	03/04/2020	Asia / Japan / HokurikuShinsyu	Human	unknown	unknown	Symptomatic	Original	Pharyngeal swab	B.1.1	GR	Japan	Symptomatic	No (randomly sampled out due to data redundancy from Japan)
hCoV-19/Japan/PG-0501/2020	EPI_ISL_480160	04/04/2020	Asia / Japan / HokurikuShinsyu	Human	unknown	unknown	Symptomatic	Original	Pharyngeal swab	B.1.1	GR	Japan	Symptomatic	Yes
hCoV-19/Japan/PG-0502/2020	EPI_ISL_480161	04/04/2020	Asia / Japan / HokurikuShinsyu	Human	unknown	unknown	Symptomatic	Original	Pharyngeal swab	B.1.1	GR	Japan	Symptomatic	No (randomly sampled out due to data redundancy from Japan)
hCoV-19/Japan/PG-0503/2020	EPI_ISL_480162	04/04/2020	Asia / Japan / HokurikuShinsyu	Human	unknown	unknown	Symptomatic	Original	Pharyngeal swab	B.1.1	GR	Japan	Symptomatic	No (randomly sampled out due to data redundancy from Japan)
hCoV-19/Japan/PG-0504/2020	EPI_ISL_480163	05/04/2020	Asia / Japan / HokurikuShinsyu	Human	unknown	unknown	Symptomatic	Original	Pharyngeal swab	B.1.1	GR	Japan	Symptomatic	No (randomly sampled out due to data redundancy from Japan)
hCoV-19/Japan/PG-0505/2020	EPI_ISL_480164	07/04/2020	Asia / Japan / HokurikuShinsyu	Human	unknown	unknown	Symptomatic	Original	Pharyngeal swab	B.1.1	GR	Japan	Symptomatic	No (randomly sampled out due to data redundancy from Japan)
hCoV-19/Japan/PG-0506/2020	EPI_ISL_480165	07/04/2020	Asia / Japan / HokurikuShinsyu	Human	unknown	unknown	Symptomatic	Original	Pharyngeal swab	B.1.1	GR	Japan	Symptomatic	No (randomly sampled out due to data redundancy from Japan)
hCoV-19/Japan/PG-0507/2020	EPI_ISL_480166	07/04/2020	Asia / Japan / HokurikuShinsyu	Human	unknown	unknown	Symptomatic	Original	Pharyngeal swab	B.1.1	GR	Japan	Symptomatic	No (randomly sampled out due to data redundancy from Japan)
hCoV-19/Japan/PG-0508/2020	EPI_ISL_480167	07/04/2020	Asia / Japan / HokurikuShinsyu	Human	unknown	unknown	Symptomatic	Original	Pharyngeal swab	B.1.1	GR	Japan	Symptomatic	No (randomly sampled out due to data redundancy from Japan)
hCoV-19/Japan/PG-0509/2020	EPI_ISL_480168	07/04/2020	Asia / Japan / HokurikuShinsyu	Human	unknown	unknown	Symptomatic	Original	Pharyngeal swab	B.1.1	GR	Japan	Symptomatic	No (randomly sampled out due to data redundancy from Japan)
hCoV-19/Japan/PG-0511/2020	EPI_ISL_480169	02/04/2020	Asia / Japan / Kanto	Human	unknown	unknown	Symptomatic	Original	Pharyngeal swab	B.1.1	GR	Japan	Symptomatic	No (randomly sampled out due to data redundancy from Japan)
hCoV-19/Japan/PG-0512/2020	EPI_ISL_480170	02/04/2020	Asia / Japan / Kanto	Human	unknown	unknown	Symptomatic	Original	Pharyngeal swab	B.1.1	GR	Japan	Symptomatic	No (randomly sampled out due to data redundancy from Japan)
hCoV-19/Japan/PG-0513/2020	EPI_ISL_480171	02/04/2020	Asia / Japan / Kanto	Human	unknown	unknown	Symptomatic	Original	Pharyngeal swab	B.1.1	GR	Japan	Symptomatic	No (randomly sampled out due to data redundancy from Japan)
hCoV-19/Japan/PG-0514/2020	EPI_ISL_480172	02/04/2020	Asia / Japan / Kanto	Human	unknown	unknown	Symptomatic	Original	Pharyngeal swab	B.1.1	GR	Japan	Symptomatic	No (randomly sampled out due to data redundancy from Japan)
hCoV-19/Japan/PG-0515/2020	EPI_ISL_480173	03/04/2020	Asia / Japan / Kanto	Human	unknown	unknown	Symptomatic	Original	Pharyngeal swab	B.1.1	GR	Japan	Symptomatic	No (randomly sampled out due to data redundancy from Japan)
hCoV-19/Japan/PG-0516/2020	EPI_ISL_480174	06/04/2020	Asia / Japan / Kanto	Human	unknown	unknown	Symptomatic	Original	Pharyngeal swab	B.1.1	GR	Japan	Symptomatic	No (randomly sampled out due to data redundancy from Japan)
hCoV-19/Japan/PG-0517/2020	EPI_ISL_480175	06/04/2020	Asia / Japan / Kanto	Human	unknown	unknown	Symptomatic	Original	Pharyngeal swab	B.1.1	GR	Japan	Symptomatic	No (randomly sampled out due to data redundancy from Japan)
hCoV-19/Japan/PG-0518/2020	EPI_ISL_480176	06/04/2020	Asia / Japan / Kanto	Human	unknown	unknown	Symptomatic	Original	Pharyngeal swab	B.1.1	GR	Japan	Symptomatic	No (randomly sampled out due to data redundancy from Japan)
hCoV-19/Japan/PG-0519/2020	EPI_ISL_480177	01/04/2020	Asia / Japan / Kanto	Human	unknown	unknown	Symptomatic	Original	Pharyngeal swab	B.1.1	GR	Japan	Symptomatic	No (randomly sampled out due to data redundancy from Japan)
hCoV-19/Japan/PG-0520/2020	EPI_ISL_480178	25/03/2020	Asia / Japan / Chushikoku	Human	unknown	unknown	Symptomatic	Original	Pharyngeal swab	B.1.1	GR	Japan	Symptomatic	No (randomly sampled out due to data redundancy from Japan)
hCoV-19/Japan/PG-0521/2020	EPI_ISL_480179	28/03/2020	Asia / Japan / Chushikoku	Human	unknown	unknown	Symptomatic	Original	Pharyngeal swab	B.1.1	GR	Japan	Symptomatic	No (randomly sampled out due to data redundancy from Japan)
hCoV-19/Japan/PG-0522/2020	EPI_ISL_480180	27/03/2020	Asia / Japan / Kanto	Human	unknown	unknown	Symptomatic	Original	Pharyngeal swab	B.1	G	Japan	Symptomatic	Yes
hCoV-19/Japan/PG-0523/2020	EPI_ISL_480181	27/03/2020	Asia / Japan / Kanto	Human	unknown	unknown	Symptomatic	Original	Pharyngeal swab	B.1	G	Japan	Symptomatic	Yes
hCoV-19/Japan/PG-0524/2020	EPI_ISL_480182	01/04/2020	Asia / Japan / Kanto	Human	unknown	unknown	Symptomatic	Original	Pharyngeal swab	B.1.1	GR	Japan	Symptomatic	No (randomly sampled out due to data redundancy from Japan)
hCoV-19/Japan/PG-0525/2020	EPI_ISL_480183	03/04/2020	Asia / Japan / Kanto	Human	unknown	unknown	Symptomatic	Original	Pharyngeal swab	B.1.1	GR	Japan	Symptomatic	No (randomly sampled out due to data redundancy from Japan)
hCoV-19/Japan/PG-0526/2020	EPI_ISL_480184	04/04/2020	Asia / Japan / Kanto	Human	unknown	unknown	Symptomatic	Original	Pharyngeal swab	B.1	G	Japan	Symptomatic	Yes
hCoV-19/Japan/PG-0527/2020	EPI_ISL_480185	02/04/2020	Asia / Japan / Kanto	Human	unknown	unknown	Symptomatic	Original	Pharyngeal swab	B.1.1	GR	Japan	Symptomatic	No (randomly sampled out due to data redundancy from Japan)
hCoV-19/Japan/PG-0528/2020	EPI_ISL_480186	02/04/2020	Asia / Japan / Kanto	Human	unknown	unknown	Symptomatic	Original	Pharyngeal swab	B.1.1	GR	Japan	Symptomatic	No (randomly sampled out due to data redundancy from Japan)
hCoV-19/Japan/PG-0529/2020	EPI_ISL_480187	03/04/2020	Asia / Japan / Kanto	Human	unknown	unknown	Symptomatic	Original	Pharyngeal swab	B.1.1	GR	Japan	Symptomatic	No (randomly sampled out due to data redundancy from Japan)
hCoV-19/Japan/PG-0530/2020	EPI_ISL_480188	04/04/2020	Asia / Japan / Kanto	Human	unknown	unknown	Symptomatic	Original	Pharyngeal swab	B.1.1	GR	Japan	Symptomatic	No (randomly sampled out due to data redundancy from Japan)
hCoV-19/Japan/PG-0531/2020	EPI_ISL_480189	05/04/2020	Asia / Japan / Kanto	Human	unknown	unknown	Symptomatic	Original	Pharyngeal swab	B.1	G	Japan	Symptomatic	Yes
hCoV-19/Japan/PG-0532/2020	EPI_ISL_480190	2020-04	Asia / Japan / Kanto	Human	unknown	unknown	Symptomatic	Original	Pharyngeal swab	B.1.1	GR	Japan	Symptomatic	No (randomly sampled out due to data redundancy from Japan)
hCoV-19/Japan/PG-0533/2020	EPI_ISL_480191	2020-04	Asia / Japan / Kanto	Human	unknown	unknown	Symptomatic	Original	Pharyngeal swab	B.1.1	GR	Japan	Symptomatic	No (randomly sampled out due to data redundancy from Japan)
hCoV-19/Japan/PG-0534/2020	EPI_ISL_480192	2020-04	Asia / Japan / Kanto	Human	unknown	unknown	Symptomatic	Original	Pharyngeal swab	B.1.1	GR	Japan	Symptomatic	No (randomly sampled out due to data redundancy from Japan)
hCoV-19/Japan/PG-0536/2020	EPI_ISL_480193	2020-04	Asia / Japan / Kanto	Human	unknown	unknown	Symptomatic	Original	Pharyngeal swab	B.1.1	GR	Japan	Symptomatic	No (randomly sampled out due to data redundancy from Japan)
hCoV-19/Japan/PG-0537/2020	EPI_ISL_480194	2020-04	Asia / Japan / Kanto	Human	unknown	unknown	Symptomatic	Original	Pharyngeal swab	B.1.1	GR	Japan	Symptomatic	Yes
hCoV-19/Japan/PG-0538/2020	EPI_ISL_480195	2020-04	Asia / Japan / Kanto	Human	unknown	unknown	Symptomatic	Original	Pharyngeal swab	B.1.1	GR	Japan	Symptomatic	No (randomly sampled out due to data redundancy from Japan)
hCoV-19/Japan/PG-0539/2020	EPI_ISL_480196	30/03/2020	Asia / Japan / HokurikuShinsyu	Human	unknown	unknown	Symptomatic	Original	Pharyngeal swab	B.1.5	G	Japan	Symptomatic	Yes
hCoV-19/Japan/PG-0541/2020	EPI_ISL_480197	03/04/2020	Asia / Japan / HokurikuShinsyu	Human	unknown	unknown	Symptomatic	Original	Pharyngeal swab	B.1.5	G	Japan	Symptomatic	Yes
hCoV-19/Japan/PG-0542/2020	EPI_ISL_480198	30/03/2020	Asia / Japan / HokurikuShinsyu	Human	unknown	unknown	Symptomatic	Original	Pharyngeal swab	B.1.5	G	Japan	Symptomatic	Yes
hCoV-19/Japan/PG-0543/2020	EPI_ISL_480199	31/03/2020	Asia / Japan / HokurikuShinsyu	Human	unknown	unknown	Symptomatic	Original	Pharyngeal swab	B.1.5	G	Japan	Symptomatic	Yes
hCoV-19/Japan/PG-0544/2020	EPI_ISL_480200	31/03/2020	Asia / Japan / HokurikuShinsyu	Human	unknown	unknown	Symptomatic	Original	Pharyngeal swab	B.1.5	G	Japan	Symptomatic	Yes
hCoV-19/Japan/PG-0545/2020	EPI_ISL_480201	31/03/2020	Asia / Japan / HokurikuShinsyu	Human	unknown	unknown	Symptomatic	Original	Pharyngeal swab	B.1.1	GR	Japan	Symptomatic	No (randomly sampled out due to data redundancy from Japan)
hCoV-19/Japan/PG-0546/2020	EPI_ISL_480202	01/04/2020	Asia / Japan / HokurikuShinsyu	Human	unknown	unknown	Symptomatic	Original	Pharyngeal swab	B.1.5	G	Japan	Symptomatic	Yes
hCoV-19/Japan/PG-0547/2020	EPI_ISL_480203	02/04/2020	Asia / Japan / HokurikuShinsyu	Human	unknown	unknown	Symptomatic	Original	Pharyngeal swab	B.1.5	G	Japan	Symptomatic	Yes
hCoV-19/Japan/PG-0550/2020	EPI_ISL_480204	11/04/2020	Asia / Japan / Tohoku	Human	unknown	unknown	Symptomatic	Original	Pharyngeal swab	B.1.1	GR	Japan	Symptomatic	No (randomly sampled out due to data redundancy from Japan)
hCoV-19/Japan/PG-0552/2020	EPI_ISL_480205	07/04/2020	Asia / Japan / Kansai	Human	unknown	unknown	Symptomatic	Original	Pharyngeal swab	B.1.1	GR	Japan	Symptomatic	No (randomly sampled out due to data redundancy from Japan)
hCoV-19/Japan/PG-0555/2020	EPI_ISL_480206	07/04/2020	Asia / Japan / Kansai	Human	unknown	unknown	Symptomatic	Original	Pharyngeal swab	B.1.1	GR	Japan	Symptomatic	No (randomly sampled out due to data redundancy from Japan)
hCoV-19/Japan/PG-0556/2020	EPI_ISL_480207	07/04/2020	Asia / Japan / Kansai	Human	unknown	unknown	Symptomatic	Original	Pharyngeal swab	B.1.1	GR	Japan	Symptomatic	No (randomly sampled out due to data redundancy from Japan)
hCoV-19/Japan/PG-0557/2020	EPI_ISL_480208	07/04/2020	Asia / Japan / Kansai	Human	unknown	unknown	Symptomatic	Original	Pharyngeal swab	B.1.1	GR	Japan	Symptomatic	No (randomly sampled out due to data redundancy from Japan)
hCoV-19/Japan/PG-0559/2020	EPI_ISL_480209	07/04/2020	Asia / Japan / Kansai	Human	unknown	unknown	Symptomatic	Original	Pharyngeal swab	B.1.1	GR	Japan	Symptomatic	No (randomly sampled out due to data redundancy from Japan)
hCoV-19/Japan/PG-0560/2020	EPI_ISL_480210	08/04/2020	Asia / Japan / Kansai	Human	unknown	unknown	Symptomatic	Original	Pharyngeal swab	B.1.1	GR	Japan	Symptomatic	No (randomly sampled out due to data redundancy from Japan)
hCoV-19/Japan/PG-0561/2020	EPI_ISL_480211	08/04/2020	Asia / Japan / Kansai	Human	unknown	unknown	Symptomatic	Original	Pharyngeal swab	B.1.1	GR	Japan	Symptomatic	No (randomly sampled out due to data redundancy from Japan)
hCoV-19/Japan/PG-0563/2020	EPI_ISL_480212	08/04/2020	Asia / Japan / Kansai	Human	unknown	unknown	Symptomatic	Original	Pharyngeal swab	B.1.1	GR	Japan	Symptomatic	No (randomly sampled out due to data redundancy from Japan)
hCoV-19/Japan/PG-0564/2020	EPI_ISL_480213	08/04/2020	Asia / Japan / Kansai	Human	unknown	unknown	Symptomatic	Original	Pharyngeal swab	B.1.1	GR	Japan	Symptomatic	No (randomly sampled out due to data redundancy from Japan)
hCoV-19/Japan/PG-0566/2020	EPI_ISL_480214	09/04/2020	Asia / Japan / Kansai	Human	unknown	unknown	Symptomatic	Original	Pharyngeal swab	B.1.1	GR	Japan	Symptomatic	No (randomly sampled out due to data redundancy from Japan)
hCoV-19/Japan/PG-0572/2020	EPI_ISL_480215	09/04/2020	Asia / Japan / Kansai	Human	unknown	unknown	Symptomatic	Original	Pharyngeal swab	B.1.1	GR	Japan	Symptomatic	No (randomly sampled out due to data redundancy from Japan)
hCoV-19/Japan/PG-0573/2020	EPI_ISL_480216	09/04/2020	Asia / Japan / Kansai	Human	unknown	unknown	Symptomatic	Original	Pharyngeal swab	B.1.1	GR	Japan	Symptomatic	No (randomly sampled out due to data redundancy from Japan)
hCoV-19/Japan/PG-0576/2020	EPI_ISL_480217	06/04/2020	Asia / Japan / Kansai	Human	unknown	unknown	Symptomatic	Original	Pharyngeal swab	B.1.1	GR	Japan	Symptomatic	No (randomly sampled out due to data redundancy from Japan)
hCoV-19/Japan/PG-0579/2020	EPI_ISL_480218	11/04/2020	Asia / Japan / Kansai	Human	unknown	unknown	Symptomatic	Original	Pharyngeal swab	B.1.1	GR	Japan	Symptomatic	No (randomly sampled out due to data redundancy from Japan)
hCoV-19/Japan/PG-0582/2020	EPI_ISL_480219	11/04/2020	Asia / Japan / Kansai	Human	unknown	unknown	Symptomatic	Original	Pharyngeal swab	B.1.1	GR	Japan	Symptomatic	No (randomly sampled out due to data redundancy from Japan)
hCoV-19/Japan/PG-0587/2020	EPI_ISL_480220	12/04/2020	Asia / Japan / Kansai	Human	unknown	unknown	Symptomatic	Original	Pharyngeal swab	B.1.1	GR	Japan	Symptomatic	No (randomly sampled out due to data redundancy from Japan)
hCoV-19/Japan/PG-0589/2020	EPI_ISL_480221	01/04/2020	Asia / Japan / Kanto	Human	unknown	unknown	Symptomatic	Original	Pharyngeal swab	B	L	Japan	Symptomatic	Yes
hCoV-19/Japan/PG-0590/2020	EPI_ISL_480222	03/04/2020	Asia / Japan / Kanto	Human	unknown	unknown	Symptomatic	Original	Pharyngeal swab	B.1	GH	Japan	Symptomatic	Yes
hCoV-19/Japan/PG-0591/2020	EPI_ISL_480223	04/04/2020	Asia / Japan / Kanto	Human	unknown	unknown	Symptomatic	Original	Pharyngeal swab	B.1.1	GR	Japan	Symptomatic	No (randomly sampled out due to data redundancy from Japan)
hCoV-19/Japan/PG-0293/2020	EPI_ISL_480225	26/03/2020	Asia / Japan / HokurikuShinsyu	Human	unknown	unknown	Symptomatic	Original	Pharyngeal swab	B.1.1	GR	Japan	Symptomatic	No (randomly sampled out due to data redundancy from Japan)
hCoV-19/Japan/PG-0198/2020	EPI_ISL_480226	23/03/2020	Asia / Japan / Kanto	Human	unknown	unknown	Symptomatic	Original	Pharyngeal swab	B.6	O	Japan	Symptomatic	Yes
hCoV-19/Japan/PG-0186/2020	EPI_ISL_480227	16/03/2020	Asia / Japan / Kanto	Human	unknown	unknown	Symptomatic	Original	Pharyngeal swab	A	S	Japan	Symptomatic	Yes
hCoV-19/Turkey/GLAB-CoV003/2020	EPI_ISL_480228	13/04/2020	Europe / Turkey / Istanbul	Human	Male	46	Hospitalized	Original	Oro-pharyngeal swab	B.1.9	GH	Turkey	Undertermined	No (couldn't determine the patient status as either pre-symptomatic / asymptomatic or symptomatic conclusively)
hCoV-19/Turkey/GLAB-CoV004/2020	EPI_ISL_480229	13/04/2020	Europe / Turkey / Istanbul	Human	Male	39	Hospitalized	Original	Oro-pharyngeal swab	B.1.1	GR	Turkey	Undertermined	No (couldn't determine the patient status as either pre-symptomatic / asymptomatic or symptomatic conclusively)
hCoV-19/Turkey/GLAB-CoV006/2020	EPI_ISL_480230	13/04/2020	Europe / Turkey / Istanbul	Human	Male	48	Outpatient	Original	Oro-pharyngeal swab	B.1.1	GR	Turkey	Undertermined	No (couldn't determine the patient status as either pre-symptomatic / asymptomatic or symptomatic conclusively)
hCoV-19/Turkey/GLAB-CoV007/2020	EPI_ISL_480231	13/04/2020	Europe / Turkey / Istanbul	Human	Male	70	Hospitalized	Original	Oro-pharyngeal swab	B.1	GH	Turkey	Undertermined	No (couldn't determine the patient status as either pre-symptomatic / asymptomatic or symptomatic conclusively)
hCoV-19/Turkey/GLAB-CoV014/2020	EPI_ISL_480232	17/04/2020	Europe / Turkey / Istanbul	Human	Male	48	Asymptomatic	Original	Oro-pharyngeal swab	B.1.1	GR	Turkey	Pre-symptomatic / Asymptomatic	Yes
hCoV-19/Turkey/GLAB-CoV021/2020	EPI_ISL_480233	17/04/2020	Europe / Turkey / Istanbul	Human	Female	25	Outpatient	Original	Oro-pharyngeal swab	B.1.1	GR	Turkey	Undertermined	No (couldn't determine the patient status as either pre-symptomatic / asymptomatic or symptomatic conclusively)
hCoV-19/Turkey/GLAB-CoV030/2020	EPI_ISL_480234	18/04/2020	Europe / Turkey / Istanbul	Human	Male	15	Outpatient	Original	Oro-pharyngeal swab	B.1.9	GH	Turkey	Undertermined	No (couldn't determine the patient status as either pre-symptomatic / asymptomatic or symptomatic conclusively)

hCoV-19/Turkey/GLAB-CoV054/2020	EPI_ISL_480235	22/04/2020	Europe / Turkey / Istanbul	Human		Female	21	Outpatient	Original	Oro-pharyngeal swab		B.1.9	GH	Turkey	Undertermined	No (couldn't determine the patient status as either pre-symptomatic / asymptomatic or symptomatic conclusively)
hCoV-19/Turkey/GLAB-CoV055/2020	EPI_ISL_480236	22/04/2020	Europe / Turkey / Istanbul	Human		Male	20	Hospitalized	Original	Oro-pharyngeal swab		B.1.1	GR	Turkey	Undertermined	No (couldn't determine the patient status as either pre-symptomatic / asymptomatic or symptomatic conclusively)
hCoV-19/Turkey/GLAB-CoV059/2020	EPI_ISL_480237	22/04/2020	Europe / Turkey / Istanbul	Human		Female	24	Asymptomatic	Original	Oro-pharyngeal swab		B.1.9	GH	Turkey	Pre-symptomatic / Asymptomatic	Yes
hCoV-19/Turkey/GLAB-CoV062/2020	EPI_ISL_480238	22/04/2020	Europe / Turkey / Istanbul	Human		Male	37	Hospitalized	Original	Oro-pharyngeal swab		B.1.1	GR	Turkey	Undertermined	No (couldn't determine the patient status as either pre-symptomatic / asymptomatic or symptomatic conclusively)
hCoV-19/Turkey/GLAB-CoV063/2020	EPI_ISL_480239	22/04/2020	Europe / Turkey / Istanbul	Human		Male	54	Hospitalized	Original	Oro-pharyngeal swab		B.1.113	GH	Turkey	Undertermined	No (couldn't determine the patient status as either pre-symptomatic / asymptomatic or symptomatic conclusively)
hCoV-19/Turkey/GLAB-CoV064/2020	EPI_ISL_480240	22/04/2020	Europe / Turkey / Istanbul	Human		Male	63	Hospitalized	Original	Oro-pharyngeal swab		B.1.1	GR	Turkey	Undertermined	No (couldn't determine the patient status as either pre-symptomatic / asymptomatic or symptomatic conclusively)
hCoV-19/Turkey/GLAB-CoV086/2020	EPI_ISL_480241	23/04/2020	Europe / Turkey / Istanbul	Human		Male	54	Outpatient	Original	Oro-pharyngeal swab		B.1.9	GH	Turkey	Undertermined	No (couldn't determine the patient status as either pre-symptomatic / asymptomatic or symptomatic conclusively)
hCoV-19/Turkey/GLAB-CoV089/2020	EPI_ISL_480242	29/04/2020	Europe / Turkey / Istanbul	Human		Female	51	Hospitalized	Original	Oro-pharyngeal swab		B.1.1	GR	Turkey	Undertermined	No (couldn't determine the patient status as either pre-symptomatic / asymptomatic or symptomatic conclusively)
hCoV-19/Turkey/GLAB-CoV091/2020	EPI_ISL_480243	23/04/2020	Europe / Turkey / Istanbul	Human		Female	29	Outpatient	Original	Oro-pharyngeal swab		B.1.1	GR	Turkey	Undertermined	No (couldn't determine the patient status as either pre-symptomatic / asymptomatic or symptomatic conclusively)
hCoV-19/Turkey/GLAB-CoV092/2020	EPI_ISL_480244	23/04/2020	Europe / Turkey / Istanbul	Human		Male	82	Deceased	Original	Oro-pharyngeal swab		B.1.1	GR	Turkey	Symptomatic	Yes
hCoV-19/Turkey/GLAB-CoV094/2020	EPI_ISL_480245	26/04/2020	Europe / Turkey / Istanbul	Human		Male	31	Hospitalized	Original	Oro-pharyngeal swab		B.1	G	Turkey	Undertermined	No (couldn't determine the patient status as either pre-symptomatic / asymptomatic or symptomatic conclusively)
hCoV-19/Turkey/GLAB-CoV114/2020	EPI_ISL_480246	26/04/2020	Europe / Turkey / Istanbul	Human		Male	55	Hospitalized	Original	Oro-pharyngeal swab		B.4	O	Turkey	Undertermined	No (couldn't determine the patient status as either pre-symptomatic / asymptomatic or symptomatic conclusively)
hCoV-19/Turkey/GLAB-CoV115/2020	EPI_ISL_480247	26/04/2020	Europe / Turkey / Istanbul	Human		Male	68	Hospitalized	Original	Oro-pharyngeal swab		B.1.9	GH	Turkey	Undertermined	No (couldn't determine the patient status as either pre-symptomatic / asymptomatic or symptomatic conclusively)
hCoV-19/Turkey/GLAB-CoV126/2020	EPI_ISL_480248	26/04/2020	Europe / Turkey / Istanbul	Human		Female	66	ICU	Original	Oro-pharyngeal swab		B.1.1	GR	Turkey	Symptomatic	Yes
hCoV-19/Turkey/GLAB-CoV139/2020	EPI_ISL_480249	30/04/2020	Europe / Turkey / Istanbul	Human		Female	78	Deceased	Original	Oro-pharyngeal swab		B.1.1	GR	Turkey	Symptomatic	Yes
hCoV-19/Turkey/GLAB-CoV149/2020	EPI_ISL_480250	02/05/2020	Europe / Turkey / Istanbul	Human		Female	66	Hospitalized	Original	Oro-pharyngeal swab		B.1.1	GR	Turkey	Undertermined	No (couldn't determine the patient status as either pre-symptomatic / asymptomatic or symptomatic conclusively)
hCoV-19/Turkey/GLAB-CoV153/2020	EPI_ISL_480252	02/05/2020	Europe / Turkey / Istanbul	Human		Male	45	Outpatient	Original	Oro-pharyngeal swab		B.1.9	GH	Turkey	Undertermined	No (couldn't determine the patient status as either pre-symptomatic / asymptomatic or symptomatic conclusively)
hCoV-19/Turkey/GLAB-CoV155/2020	EPI_ISL_480253	01/05/2020	Europe / Turkey / Istanbul	Human		Male	20	Asymptomatic	Original	Oro-pharyngeal swab		B.1.1	GR	Turkey	Pre-symptomatic / Asymptomatic	Yes
hCoV-19/Turkey/GLAB-CoV156/2020	EPI_ISL_480254	02/05/2020	Europe / Turkey / Istanbul	Human		Male	42	Outpatient	Original	Oro-pharyngeal swab		B.1.9	GH	Turkey	Undertermined	No (couldn't determine the patient status as either pre-symptomatic / asymptomatic or symptomatic conclusively)
hCoV-19/Turkey/GLAB-CoV157/2020	EPI_ISL_480255	02/05/2020	Europe / Turkey / Istanbul	Human		Male	25	Outpatient	Original	Oro-pharyngeal swab		B.1.9	GH	Turkey	Undertermined	No (couldn't determine the patient status as either pre-symptomatic / asymptomatic or symptomatic conclusively)
hCoV-19/Turkey/GLAB-CoV158/2020	EPI_ISL_480256	03/05/2020	Europe / Turkey / Istanbul	Human		Male	75	Hospitalized	Original	Oro-pharyngeal swab		B.4	O	Turkey	Undertermined	No (couldn't determine the patient status as either pre-symptomatic / asymptomatic or symptomatic conclusively)
hCoV-19/Turkey/GLAB-CoV162/2020	EPI_ISL_480257	03/05/2020	Europe / Turkey / Istanbul	Human		Male	45	Hospitalized	Original	Oro-pharyngeal swab		B.1.9	GH	Turkey	Undertermined	No (couldn't determine the patient status as either pre-symptomatic / asymptomatic or symptomatic conclusively)
hCoV-19/Turkey/GLAB-CoV163/2020	EPI_ISL_480258	03/05/2020	Europe / Turkey / Istanbul	Human		Male	29	Hospitalized	Original	Oro-pharyngeal swab		B.1.1	GR	Turkey	Undertermined	No (couldn't determine the patient status as either pre-symptomatic / asymptomatic or symptomatic conclusively)
hCoV-19/Turkey/GLAB-CoV164/2020	EPI_ISL_480259	03/05/2020	Europe / Turkey / Istanbul	Human		Female	58	Hospitalized	Original	Oro-pharyngeal swab		B.1.1	GR	Turkey	Undertermined	No (couldn't determine the patient status as either pre-symptomatic / asymptomatic or symptomatic conclusively)
hCoV-19/Turkey/GLAB-CoV167/2020	EPI_ISL_480260	04/05/2020	Europe / Turkey / Istanbul	Human		Male	75	Hospitalized	Original	Oro-pharyngeal swab		B.1.1	GR	Turkey	Undertermined	No (couldn't determine the patient status as either pre-symptomatic / asymptomatic or symptomatic conclusively)
hCoV-19/Turkey/GLAB-CoV168/2020	EPI_ISL_480261	03/05/2020	Europe / Turkey / Istanbul	Human		Male	60	Hospitalized	Original	Oro-pharyngeal swab		B.1.1	GR	Turkey	Undertermined	No (couldn't determine the patient status as either pre-symptomatic / asymptomatic or symptomatic conclusively)
hCoV-19/Turkey/GLAB-CoV169/2020	EPI_ISL_480262	01/05/2020	Europe / Turkey / Istanbul	Human		Female	72	Deceased	Original	Oro-pharyngeal swab		B.1.1	GR	Turkey	Symptomatic	Yes
hCoV-19/Turkey/GLAB-CoV170/2020	EPI_ISL_480263	03/05/2020	Europe / Turkey / Istanbul	Human		Male	89	Hospitalized	Original	Oro-pharyngeal swab		B.1.1	GR	Turkey	Undertermined	No (couldn't determine the patient status as either pre-symptomatic / asymptomatic or symptomatic conclusively)
hCoV-19/Turkey/GLAB-CoV171/2020	EPI_ISL_480264	20/04/2020	Europe / Turkey / Istanbul	Human		Male	90	Deceased	Original	Oro-pharyngeal swab		B.1.1	GR	Turkey	Symptomatic	Yes
hCoV-19/Turkey/GLAB-CoV173/2020	EPI_ISL_480265	04/05/2020	Europe / Turkey / Istanbul	Human		Female	45	Hospitalized	Original	Oro-pharyngeal swab		B.1.1	GR	Turkey	Undertermined	No (couldn't determine the patient status as either pre-symptomatic / asymptomatic or symptomatic conclusively)
hCoV-19/Turkey/GLAB-CoV174/2020	EPI_ISL_480266	04/05/2020	Europe / Turkey / Istanbul	Human		Male	87	Hospitalized	Original	Oro-pharyngeal swab		B.1.1	GR	Turkey	Undertermined	No (couldn't determine the patient status as either pre-symptomatic / asymptomatic or symptomatic conclusively)
hCoV-19/Turkey/GLAB-CoV176/2020	EPI_ISL_480267	04/05/2020	Europe / Turkey / Istanbul	Human		Female	38	Hospitalized	Original	Oro-pharyngeal swab		B.1.1	GR	Turkey	Undertermined	No (couldn't determine the patient status as either pre-symptomatic / asymptomatic or symptomatic conclusively)
hCoV-19/Turkey/GLAB-CoV178/2020	EPI_ISL_480268	04/05/2020	Europe / Turkey / Istanbul	Human		Male	49	Asymptomatic	Original	Oro-pharyngeal swab		B.1.1	GR	Turkey	Pre-symptomatic / Asymptomatic	Yes
hCoV-19/Turkey/GLAB-CoV179/2020	EPI_ISL_480269	04/05/2020	Europe / Turkey / Istanbul	Human		Male	63	Hospitalized	Original	Oro-pharyngeal swab		B.1.1	GR	Turkey	Undertermined	No (couldn't determine the patient status as either pre-symptomatic / asymptomatic or symptomatic conclusively)
hCoV-19/Turkey/GLAB-CoV181/2020	EPI_ISL_480270	06/05/2020	Europe / Turkey / Istanbul	Human		Female	86	Hospitalized	Original	Oro-pharyngeal swab		B.1.1	GR	Turkey	Undertermined	No (couldn't determine the patient status as either pre-symptomatic / asymptomatic or symptomatic conclusively)
hCoV-19/Turkey/GLAB-CoV182/2020	EPI_ISL_480271	05/05/2020	Europe / Turkey / Istanbul	Human		Male	47	Hospitalized	Original	Oro-pharyngeal swab		B.1.9	GH	Turkey	Undertermined	No (couldn't determine the patient status as either pre-symptomatic / asymptomatic or symptomatic conclusively)
hCoV-19/Turkey/GLAB-CoV183/2020	EPI_ISL_480272	07/05/2020	Europe / Turkey / Istanbul	Human		Female	87	Hospitalized	Original	Oro-pharyngeal swab		B.1.1	GR	Turkey	Undertermined	No (couldn't determine the patient status as either pre-symptomatic / asymptomatic or symptomatic conclusively)
hCoV-19/Turkey/GLAB-CoV185/2020	EPI_ISL_480273	05/05/2020	Europe / Turkey / Istanbul	Human		Female	88	Hospitalized	Original	Oro-pharyngeal swab		B.1.1	GR	Turkey	Undertermined	No (couldn't determine the patient status as either pre-symptomatic / asymptomatic or symptomatic conclusively)
hCoV-19/Turkey/GLAB-CoV187/2020	EPI_ISL_480274	07/05/2020	Europe / Turkey / Istanbul	Human		Female	55	Hospitalized	Original	Oro-pharyngeal swab		B.1.9	GH	Turkey	Undertermined	No (couldn't determine the patient status as either pre-symptomatic / asymptomatic or symptomatic conclusively)
hCoV-19/Turkey/GLAB-CoV190/2020	EPI_ISL_480277	04/05/2020	Europe / Turkey / Istanbul	Human		Male	38	Hospitalized	Original	Saliva		B.1	GH	Turkey	Undertermined	No (couldn't determine the patient status as either pre-symptomatic / asymptomatic or symptomatic conclusively)
hCoV-19/Turkey/GLAB-CoV193/2020	EPI_ISL_480278	07/05/2020	Europe / Turkey / Istanbul	Human		Female	40	Hospitalized	Original	Oro-pharyngeal swab		B.1.9	GH	Turkey	Undertermined	No (couldn't determine the patient status as either pre-symptomatic / asymptomatic or symptomatic conclusively)
hCoV-19/Turkey/GLAB-CoV197/2020	EPI_ISL_480281	05/05/2020	Europe / Turkey / Istanbul	Human		Male	58	ICU	Original	Oro-pharyngeal swab		B.1.1	GR	Turkey	Symptomatic	Yes
hCoV-19/Turkey/GLAB-CoV199/2020	EPI_ISL_480282	09/05/2020	Europe / Turkey / Istanbul	Human		Female	56	ICU	Original	Oro-pharyngeal swab		B.1.1	GR	Turkey	Symptomatic	Yes
hCoV-19/Turkey/GLAB-CoV204/2020	EPI_ISL_480283	08/05/2020	Europe / Turkey / Istanbul	Human		Male	83	Hospitalized	Original	Oro-pharyngeal swab		B.1.1	GR	Turkey	Undertermined	No (couldn't determine the patient status as either pre-symptomatic / asymptomatic or symptomatic conclusively)
hCoV-19/Turkey/GLAB-CoV205/2020	EPI_ISL_480284	09/05/2020	Europe / Turkey / Istanbul	Human		Female	52	Asymptomatic	Original	Oro-pharyngeal swab		B.1.1	GR	Turkey	Pre-symptomatic / Asymptomatic	Yes
hCoV-19/Turkey/GLAB-CoV206/2020	EPI_ISL_480285	09/05/2020	Europe / Turkey / Istanbul	Human		Female	64	Hospitalized	Original	Oro-pharyngeal swab		B.1.1	GR	Turkey	Undertermined	No (couldn't determine the patient status as either pre-symptomatic / asymptomatic or symptomatic conclusively)
hCoV-19/Turkey/GLAB-CoV207/2020	EPI_ISL_480286	10/05/2020	Europe / Turkey / Istanbul	Human		Female	63	Hospitalized	Original	Oro-pharyngeal swab		B.1.1	GR	Turkey	Undertermined	No (couldn't determine the patient status as either pre-symptomatic / asymptomatic or symptomatic conclusively)
hCoV-19/Turkey/GLAB-CoV210/2020	EPI_ISL_480289	09/05/2020	Europe / Turkey / Istanbul	Human		Male	65	Deceased	Original	Oro-pharyngeal swab		B.1.113	GH	Turkey	Symptomatic	Yes
hCoV-19/Turkey/GLAB-CoV214/2020	EPI_ISL_480290	09/05/2020	Europe / Turkey / Istanbul	Human		Female	95	Hospitalized	Original	Oro-pharyngeal swab		B.1.1	GR	Turkey	Undertermined	No (couldn't determine the patient status as either pre-symptomatic / asymptomatic or symptomatic conclusively)
hCoV-19/Turkey/GLAB-CoV216/2020	EPI_ISL_480291	09/05/2020	Europe / Turkey / Istanbul	Human		Female	54	Hospitalized	Original	Oro-pharyngeal swab		B.1.9	O	Turkey	Undertermined	No (couldn't determine the patient status as either pre-symptomatic / asymptomatic or symptomatic conclusively)
hCoV-19/Turkey/GLAB-CoV217/2020	EPI_ISL_480292	10/05/2020	Europe / Turkey / Istanbul	Human		Male	51	Hospitalized	Original	Oro-pharyngeal swab		B.1.1	GR	Turkey	Undertermined	No (couldn't determine the patient status as either pre-symptomatic / asymptomatic or symptomatic conclusively)
hCoV-19/South Africa/KRISP-0191/2020	EPI_ISL_482710	04/06/2020	Africa / South Africa / KZN / eThekwini	Human		Female	45	Live	Original	Oro-pharyngeal swab	KE19	B.1.106	G	South Africa	Undertermined	No (couldn't determine the patient status as either pre-symptomatic / asymptomatic or symptomatic conclusively)
hCoV-19/South Africa/KRISP-0192/2020	EPI_ISL_482711	04/06/2020	Africa / South Africa / KZN / eThekwini	Human		Female	34	Live	Original	Oro-pharyngeal swab	KE20	B.1.106	G	South Africa	Undertermined	No (couldn't determine the patient status as either pre-symptomatic / asymptomatic or symptomatic conclusively)
hCoV-19/South Africa/KRISP-0196/2020	EPI_ISL_482712	08/06/2020	Africa / South Africa / KZN / eThekwini	Human		Female	44	Live	Original	Oro-pharyngeal swab	KE25	B.1.106	G	South Africa	Undertermined	No (couldn't determine the patient status as either pre-symptomatic / asymptomatic or symptomatic conclusively)
hCoV-19/South Africa/KRISP-0197/2020	EPI_ISL_482713	08/06/2020	Africa / South Africa / KZN / eThekwini	Human		Female	47	Live	Original	Oro-pharyngeal swab	KE26	B.1.1	GR	South Africa	Undertermined	No (couldn't determine the patient status as either pre-symptomatic / asymptomatic or symptomatic conclusively)
hCoV-19/South Africa/KRISP-0199/2020	EPI_ISL_482714	11/06/2020	Africa / South Africa / KZN	Human		Unknown	40	Live	Original	Oro-pharyngeal swab		B.1.5	O	South Africa	Undertermined	No (couldn't determine the patient status as either pre-symptomatic / asymptomatic or symptomatic conclusively)
hCoV-19/South Africa/KRISP-0200/2020	EPI_ISL_482715	11/06/2020	Africa / South Africa / KZN	Human		Unknown	38	Live	Original	Oro-pharyngeal swab		B.1	G	South Africa	Undertermined	No (couldn't determine the patient status as either pre-symptomatic / asymptomatic or symptomatic conclusively)
hCoV-19/South Africa/KRISP-0201/2020	EPI_ISL_482716	11/06/2020	Africa / South Africa / KZN	Human		Unknown	37	Live	Original	Oro-pharyngeal swab		B.1.1	GR	South Africa	Undertermined	No (couldn't determine the patient status as either pre-symptomatic / asymptomatic or symptomatic conclusively)
hCoV-19/South Africa/KRISP-0202/2020	EPI_ISL_482717	11/06/2020	Africa / South Africa / KZN	Human		Unknown	33	Live	Original	Oro-pharyngeal swab		B.1.1	GR	South Africa	Undertermined	No (couldn't determine the patient status as either pre-symptomatic / asymptomatic or symptomatic conclusively)
hCoV-19/South Africa/KRISP-0205/2020	EPI_ISL_482718	11/06/2020	Africa / South Africa / KZN	Human		Unknown	62	Live	Original	Oro-pharyngeal swab		B.1.1	GR	South Africa	Undertermined	No (couldn't determine the patient status as either pre-symptomatic / asymptomatic or symptomatic conclusively)
hCoV-19/South Africa/KRISP-0206/2020	EPI_ISL_482719	11/06/2020	Africa / South Africa / KZN	Human		Unknown	51	Live	Original	Oro-pharyngeal swab		B.1	G	South Africa	Undertermined	No (couldn't determine the patient status as either pre-symptomatic / asymptomatic or symptomatic conclusively)
hCoV-19/South Africa/KRISP-0211/2020	EPI_ISL_482720	11/06/2020	Africa / South Africa / KZN	Human		Unknown	37	Live	Original	Oro-pharyngeal swab		B.1	G	South Africa	Undertermined	No (couldn't determine the patient status as either pre-symptomatic / asymptomatic or symptomatic conclusively)
hCoV-19/South Africa/KRISP-0212/2020	EPI_ISL_482721	11/06/2020	Africa / South Africa / KZN	Human		Unknown	43	Live	Original	Oro-pharyngeal swab		B.1	G	South Africa	Undertermined	No (couldn't determine the patient status as either pre-symptomatic / asymptomatic or symptomatic conclusively)
hCoV-19/South Africa/KRISP-0213/2020	EPI_ISL_482722	11/06/2020	Africa / South Africa / KZN	Human		Unknown	35	Live	Original	Oro-pharyngeal swab		B.1	G	South Africa	Undertermined	No (couldn't determine the patient status as either pre-symptomatic / asymptomatic or symptomatic conclusively)
hCoV-19/South Africa/KRISP-0214/2020	EPI_ISL_482723	11/06/2020	Africa / South Africa / KZN	Human		Unknown	52	Live	Original	Oro-pharyngeal swab		B.1	G	South Africa	Undertermined	No (couldn't determine the patient status as either pre-symptomatic / asymptomatic or symptomatic conclusively)
hCoV-19/South Africa/KRISP-0224/2020	EPI_ISL_482724	11/06/2020	Africa / South Africa / KZN / uThukela	Human		Male	38	Live	Original	Oro-pharyngeal swab		B.1	G	South Africa	Undertermined	No (couldn't determine the patient status as either pre-symptomatic / asymptomatic or symptomatic conclusively)
hCoV-19/South Africa/KRISP-0243/2020	EPI_ISL_482725	14/06/2020	Africa / South Africa / KZN / Umgungundlovu	Human		Male	23	Live	Original	Oro-pharyngeal swab		B.1.106	G	South Africa	Undertermined	No (couldn't determine the patient status as either pre-symptomatic / asymptomatic or symptomatic conclusively)
hCoV-19/South Africa/KRISP-0245/2020	EPI_ISL_482726	14/06/2020	Africa / South Africa / KZN / uThukela	Human		Female	36	Live	Original	Oro-pharyngeal swab		B.1	G	South Africa	Undertermined	No (couldn't determine the patient status as either pre-symptomatic / asymptomatic or symptomatic conclusively)
hCoV-19/South Africa/KRISP-0254/2020	EPI_ISL_482727	14/06/2020	Africa / South Africa / KZN / Umgungundlovu	Human		Female	27	Live	Original	Oro-pharyngeal swab		B.1	G	South Africa	Undertermined	No (couldn't determine the patient status as either pre-symptomatic / asymptomatic or symptomatic conclusively)
hCoV-19/South Africa/KRISP-0255/2020	EPI_ISL_482728	14/06/2020	Africa / South Africa / KZN / uThukela	Human		Male	37	Live	Original	Oro-pharyngeal swab		B.1	G	South Africa	Undertermined	No (couldn't determine the patient status as either pre-symptomatic / asymptomatic or symptomatic conclusively)
hCoV-19/South Africa/KRISP-0259/2020	EPI_ISL_482729	15/06/2020	Africa / South Africa / KZN / eThekwini	Human		Female	47	Live	Original	Oro-pharyngeal swab		B.1	G	South Africa	Undertermined	No (couldn't determine the patient status as either pre-symptomatic / asymptomatic or symptomatic conclusively)
hCoV-19/South Africa/KRISP-0260/2020	EPI_ISL_482730	15/06/2020	Africa / South Africa / KZN / eThekwini	Human		Female	19	Live	Original	Oro-pharyngeal swab		B.1	G	South Africa	Undertermined	No (couldn't determine the patient status as either pre-symptomatic / asymptomatic or symptomatic conclusively)
hCoV-19/South Africa/KRISP-0261/2020	EPI_ISL_482731	15/06/2020	Africa / South Africa / KZN / eThekwini	Human		Male	60	Live	Original	Oro-pharyngeal swab		B.1	G	South Africa	Undertermined	No (couldn't determine the patient status as either pre-symptomatic / asymptomatic or symptomatic conclusively)
hCoV-19/Egypt/MASRI-005/2020	EPI_ISL_482759	30/04/2020	Africa / Egypt	Human	Cairo	Male	45	Alive	Original			B.1	G	Egypt	Undertermined	No (couldn't determine the patient status as either pre-symptomatic / asymptomatic or symptomatic conclusively)
hCoV-19/Egypt/MASRI-007/2020	EPI_ISL_482760	28/04/2020	Africa / Egypt	Human	Obur Hospital	Male	35	Alive	Original			B.1	G	Egypt	Undertermined	No (couldn't determine the patient status as either pre-symptomatic / asymptomatic or symptomatic conclusively)
hCoV-19/Egypt/MASRI-008/2020	EPI_ISL_482761	30/04/2020	Africa / Egypt	Human	Obur Hospital	Male	50	Alive	Original			B.1	G	Egypt	Undertermined	No (couldn't determine the patient status as either pre-symptomatic / asymptomatic or symptomatic conclusively)
hCoV-19/Egypt/MASRI-011/2020	EPI_ISL_482762	05/05/2020	Africa / Egypt	Human	Obur Hospital	Male	60	Alive	Original			B.1	G	Egypt	Undertermined	No (couldn't determine the patient status as either pre-symptomatic / asymptomatic or symptomatic conclusively)
hCoV-19/Egypt/MASRI-012/2020	EPI_ISL_482763	05/05/2020	Africa / Egypt	Human	Obur Hospital	Female	45	Alive	Original			B.1	G	Egypt	Undertermined	No (couldn't determine the patient status as either pre-symptomatic / asymptomatic or symptomatic conclusively)
hCoV-19/Egypt/MASRI-013/2020	EPI_ISL_482764	05/05/2020	Africa / Egypt	Human	Obur Hospital	Female	32	Alive	Original			B.1	G	Egypt	Undertermined	No (couldn't determine the patient status as either pre-symptomatic / asymptomatic or symptomatic conclusively)
hCoV-19/Egypt/MASRI-014/2020	EPI_ISL_482765	30/04/2020	Africa / Egypt	Human	Cairo	Female	33	Alive	Original			B.1.36	GH	Egypt	Undertermined	No (couldn't determine the patient status as either pre-symptomatic / asymptomatic or symptomatic conclusively)
hCoV-19/Egypt/MASRI-015/2020	EPI_ISL_482766	11/05/2020	Africa / Egypt	Human	Giza	Male	39	Alive	Original			B.1.113	GH	Egypt	Undertermined	No (couldn't determine the patient status as either pre-symptomatic / asymptomatic or symptomatic conclusively)
hCoV-19/Egypt/MASRI-016/2020	EPI_ISL_482767	11/05/2020	Africa / Egypt	Human	Giza	Male	51	Alive	Original			B.1	GH	Egypt	Undertermined	No (couldn't determine the patient status as either pre-symptomatic / asymptomatic or symptomatic conclusively)
hCoV-19/Egypt/MASRI-022/2020	EPI_ISL_482768	13/05/2020	Africa / Egypt	Human	Menofia	Female	50	Alive	Original			B.1	GH	Egypt	Undertermined	No (couldn't determine the patient status as either pre-symptomatic / asymptomatic or symptomatic conclusively)
hCoV-19/Egypt/MASRI-023/2020	EPI_ISL_482769	11/05/2020	Africa / Egypt	Human	Salam City	Male	39	Alive	Original			B.1	GH	Egypt	Undertermined	No (couldn't determine the patient status as either pre-symptomatic / asymptomatic or symptomatic conclusively)
hCoV-19/Egypt/MASRI-024/2020	EPI_ISL_482770	11/05/2020	Africa / Egypt	Human	New Cairo	Male	31	Alive	Original			B.1	GH	Egypt	Undertermined	No (couldn't determine the patient status as either pre-symptomatic / asymptomatic or symptomatic conclusively)
hCoV-19/Egypt/MASRI-025/2020	EPI_ISL_482771	10/05/2020	Africa / Egypt	Human	Heliopolis	Female	39	Alive	Original			B.1	GH	Egypt	Undertermined	No (couldn't determine the patient status as either pre-symptomatic / asymptomatic or symptomatic conclusively)
hCoV-19/Egypt/MASRI-026/2020	EPI_ISL_482772	10/05/2020	Africa / Egypt	Human	1St Settlement	Male	31	Alive	Original			B.1	GH	Egypt	Undertermined	No (couldn't determine the patient status as either pre-symptomatic / asymptomatic or symptomatic conclusively)
hCoV-19/Egypt/MASRI-027/2020	EPI_ISL_482773	08/05/2020	Africa / Egypt	Human	El Sharkia	Female	33	Alive	Original			B.1.1	GR	Egypt	Undertermined	No (couldn't determine the patient status as either pre-symptomatic / asymptomatic or symptomatic conclusively)
hCoV-19/Egypt/MASRI-029/2020	EPI_ISL_482774	13/05/2020	Africa / Egypt	Human	El Zawia	Female	35	Alive	Original			B.1	GH	Egypt	Undertermined	No (couldn't determine the patient status as either pre-symptomatic / asymptomatic or symptomatic conclusively)
hCoV-19/Egypt/MASRI-030/2020	EPI_ISL_482775	14/04/2020	Africa / Egypt	Human	Pediatric Hospital ASU	Female	0.5	Alive	Original			B.1.1	GR	Egypt	Undertermined	No (couldn't determine the patient status as either pre-symptomatic / asymptomatic or symptomatic conclusively)
hCoV-19/South Africa/KRISP-0193/2020	EPI_ISL_482848	03/06/2020	Africa / South Africa / KZN / Umbilo	Human		Female	56	Live	Original	Oro-pharyngeal swab	KE21	B.1.106	G	South Africa	Undertermined	No (couldn't determine the patient status as either pre-symptomatic / asymptomatic or symptomatic conclusively)
hCoV-19/South Africa/KRISP-0194/2020	EPI_ISL_482849	06/06/2020	Africa / South Africa / KZN / Umbilo	Human		Female	40	Live	Original	Oro-pharyngeal swab	KE23	B.1.106	G	South Africa	Undertermined	No (couldn't determine the patient status as either pre-symptomatic / asymptomatic or symptomatic conclusively)
hCoV-19/South Africa/KRISP-0195/2020	EPI_ISL_482850	07/06/2020	Africa / South Africa / KZN / Umbilo	Human		Female	27	Live	Original	Oro-pharyngeal swab	KE24	B.1	G	South Africa	Undertermined	No (couldn't determine the patient status as either pre-symptomatic / asymptomatic or symptomatic conclusively)
hCoV-19/South Africa/KRISP-0198/2020	EPI_ISL_482851	11/06/2020	Africa / South Africa / KZN / eThekwini	Human		unknown	46	Live	Original	Oro-pharyngeal swab		B.1.1	GR	South Africa	Undertermined	No (couldn't determine the patient status as either pre-symptomatic / asymptomatic or symptomatic conclusively)
hCoV-19/South Africa/KRISP-0203/2020	EPI_ISL_482852	11/06/2020	Africa / South Africa / KZN / eThekwini	Human		unknown	35	Live	Original	Oro-pharyngeal swab		B.1.1	GR	South Africa	Undertermined	No (couldn't determine the patient status as either pre-symptomatic / asymptomatic or symptomatic conclusively)
hCoV-19/South Africa/KRISP-0266/2020	EPI_ISL_482853	17/06/2020	Africa / South Africa / KZN / eThekwini	Human		Male	29	Live	Original	Oro-pharyngeal swab		B.1.1	GR	South Africa	Undertermined	No (couldn't determine the patient status as either pre-symptomatic / asymptomatic or symptomatic conclusively)
hCoV-19/South Africa/KRISP-0267/2020	EPI_ISL_482854	17/06/2020	Africa / South Africa / KZN / eThekwini	Human		Female	17	Live	Original	Oro-pharyngeal swab		A	S	South Africa	Undertermined	No (couldn't determine the patient status as either pre-symptomatic / asymptomatic or symptomatic conclusively)
hCoV-19/South Africa/KRISP-0268/2020	EPI_ISL_482855	17/06/2020	Africa / South Africa / KZN / eThekwini	Human		Female	24	Live	Original	Oro-pharyngeal swab		B.1.1	GR	South Africa	Undertermined	No (couldn't determine the patient status as either pre-symptomatic / asymptomatic or symptomatic conclusively)
hCoV-19/South Africa/KRISP-0269/2020	EPI_ISL_482856	17/06/2020	Africa / South Africa / KZN / eThekwini	Human		Female	25	Live	Original	Oro-pharyngeal swab		B.1	G	South Africa	Undertermined	No (couldn't determine the patient status as either pre-symptomatic / asymptomatic or symptomatic conclusively)
hCoV-19/South Africa/KRISP-0270/2020	EPI_ISL_482857	17/06/2020	Africa / South Africa / KZN / eThekwini	Human		Female	20	Live	Original	Oro-pharyngeal swab		B.1.1	GR	South Africa	Undertermined	No (couldn't determine the patient status as either pre-symptomatic / asymptomatic or symptomatic conclusively)
hCoV-19/South Africa/KRISP-0271/2020	EPI_ISL_482858	17/06/2020	Africa / South Africa / KZN / eThekwini	Human		Female	21	Live	Original	Oro-pharyngeal swab		B.1.1	GR	South Africa	Undertermined	No (couldn't determine the patient status as either pre-symptomatic / asymptomatic or symptomatic conclusively)
hCoV-19/South Africa/KRISP-0272/2020	EPI_ISL_482859	17/06/2020	Africa / South Africa / KZN / eThekwini	Human		Female	28	Live	Original	Oro-pharyngeal swab		B.1.1	GR	South Africa	Undertermined	No (couldn't determine the patient status as either pre-symptomatic / asymptomatic or symptomatic conclusively)
hCoV-19/South Africa/KRISP-0273/2020	EPI_ISL_482860	18/06/2020	Africa / South Africa / KZN / eThekwini	Human		Female	10	Live	Original	Oro-pharyngeal swab		B.1.1	GR	South Africa	Undertermined	No (couldn't determine the patient status as either pre-symptomatic / asymptomatic or symptomatic conclusively)
hCoV-19/South Africa/KRISP-0274/2020	EPI_ISL_482861	18/06/2020	Africa / South Africa / KZN / eThekwini	Human		Female	72	Live	Original	Oro-pharyngeal swab		B.1.1	GR	South Africa	Undertermined	No (couldn't determine the patient status as either pre-symptomatic / asymptomatic or symptomatic conclusively)
hCoV-19/South Africa/KRISP-0275/2020	EPI_ISL_482862	18/06/2020	Africa / South Africa / KZN / eThekwini	Human		Female	unknown	Live	Original	Oro-pharyngeal swab		B.1.1	GR	South Africa	Undertermined	No (couldn't determine the patient status as either pre-symptomatic / asymptomatic or symptomatic conclusively)
hCoV-19/South Africa/KRISP-0276/2020	EPI_ISL_482863	18/06/2020	Africa / South Africa / KZN / eThekwini	Human		Female	29	Live	Original	Oro-pharyngeal swab		B.1.1	GR	South Africa	Undertermined	No (couldn't determine the patient status as either pre-symptomatic / asymptomatic or symptomatic conclusively)
hCoV-19/South Africa/KRISP-0277/2020	EPI_ISL_482864	16/06/2020	Africa / South Africa / KZN / eThekwini	Human		Female	34	Live	Original	Oro-pharyngeal swab		B.1.1	GR	South Africa	Undertermined	No (couldn't determine the patient status as either pre-symptomatic / asymptomatic or symptomatic conclusively)
hCoV-19/South Africa/KRISP-0278/2020	EPI_ISL_482865	16/06/2020	Africa / South Africa / KZN / eThekwini	Human		Female	52	Live	Original	Oro-pharyngeal swab		B.1.1	GR	South Africa	Undertermined	No (couldn't determine the patient status as either pre-symptomatic / asymptomatic or symptomatic conclusively)
hCoV-19/South Africa/KRISP-0279/2020	EPI_ISL_482866	16/06/2020	Africa / South Africa / KZN / eThekwini	Human		Female	43	Live	Original	Oro-pharyngeal swab		B.1.1	GR	South Africa	Undertermined	No (couldn't determine the patient status as either pre-symptomatic / asymptomatic or symptomatic conclusively)
hCoV-19/South Africa/KRISP-0280/2020	EPI_ISL_482867	16/06/2020	Africa / South Africa / KZN / eThekwini	Human		Female	37	Live	Original	Oro-pharyngeal swab		B.1.1	GR	South Africa	Undertermined	No (couldn't determine the patient status as either pre-symptomatic / asymptomatic or symptomatic conclusively)
hCoV-19/South Africa/KRISP-0281/2020	EPI_ISL_482868	16/06/2020	Africa / South Africa / KZN / eThekwini	Human		Female	37	Live	Original	Oro-pharyngeal swab		B.1.1	GR	South Africa	Undertermined	No (couldn't determine the patient status as either pre-symptomatic / asymptomatic or symptomatic conclusively)
hCoV-19/South Africa/KRISP-0282/2020	EPI_ISL_482869	16/06/2020	Africa / South Africa / KZN / eThekwini	Human		Female	49	Live	Original	Oro-pharyngeal swab		B.1.1	GR	South Africa	Undertermined	No (couldn't determine the patient status as either pre-symptomatic / asymptomatic or symptomatic conclusively)
hCoV-19/South Africa/KRISP-0283/2020	EPI_ISL_482870	16/06/2020	Africa / South Africa / KZN / eThekwini	Human		Female	50	Live	Original	Oro-pharyngeal swab		B.1.1	GR	South Africa	Undertermined	No (couldn't determine the patient status as either pre-symptomatic / asymptomatic or symptomatic conclusively)
hCoV-19/South Africa/KRISP-0284/2020	EPI_ISL_482871	16/06/2020	Africa / South Africa / KZN / eThekwini	Human		Male	50	Live	Original	Oro-pharyngeal swab		B.1.1	O	South Africa	Undertermined	No (couldn't determine the patient status as either pre-symptomatic / asymptomatic or symptomatic conclusively)
hCoV-19/South Africa/KRISP-0285/2020	EPI_ISL_482872	16/06/2020	Africa / South Africa / KZN / eThekwini	Human		Male	40	Live	Original	Oro-pharyngeal swab		B.1.1	O	South Africa	Undertermined	No (couldn't determine the patient status as either pre-symptomatic / asymptomatic or symptomatic conclusively)
hCoV-19/Senegal/64771/2020	EPI_ISL_482874	13/06/2020	Africa / Senegal / Dakar / Mbao	Human		Male	43	Released	Original	Oro/naso-pharyngeal swabs		B.1.1	GR	Senegal	Undertermined	No (couldn't determine the patient status as either pre-symptomatic / asymptomatic or symptomatic conclusively)
hCoV-19/Senegal/68550/2020	EPI_ISL_482875	16/06/2020	Africa / Senegal / Dakar	Human		Male	55	Released	Original	Oro/naso-pharyngeal swabs		B.1.5	G	Senegal	Undertermined	No (couldn't determine the patient status as either pre-symptomatic / asymptomatic or symptomatic conclusively)
hCoV-19/Senegal/68548/2020	EPI_ISL_482876	17/06/2020	Africa / Senegal / Dakar	Human		Male	45	Released	Original	Oro/naso-pharyngeal swabs		B.1.5	G	Senegal	Undertermined	No (couldn't determine the patient status as either pre-symptomatic / asymptomatic or symptomatic conclusively)
hCoV-19/Senegal/68488/2020	EPI_ISL_482877	17/06/2020	Africa / Senegal / Dakar	Human		Male	65	Released	Original	Oro/naso-pharyngeal swabs		B.1	GH	Senegal	Undertermined	No (couldn't determine the patient status as either pre-symptomatic / asymptomatic or symptomatic conclusively)
hCoV-19/Senegal/68458/2020	EPI_ISL_482878	18/06/2020	Africa / Senegal / Dakar	Human		Female	42	Released	Original	Oro/naso-pharyngeal swabs		B.1.1	GR	Senegal	Undertermined	No (couldn't determine the patient status as either pre-symptomatic / asymptomatic or symptomatic conclusively)
hCoV-19/Spain/CT-IrsiCaixa-Own160320/2020	EPI_ISL_483059	16/03/2020	Europe / Spain / Catalunya / Barcelona	Human		Male	89	Hospitalized	Original	Nasopharyngeal swab		B.1.5	G	Spain	Undertermined	No (couldn't determine the patient status as either pre-symptomatic / asymptomatic or symptomatic conclusively)
hCoV-19/India/KA-nimh-10559/2020	EPI_ISL_486400	03/05/2020	Asia / India / Karnataka	Human		Male	34	Asymptomatic	Original			B.4	O	India	Pre-symptomatic / Asymptomatic	Yes
hCoV-19/India/KA-nimh-11070/2020	EPI_ISL_486401	04/05/2020	Asia / India / Karnataka	Human		Male	23	Asymptomatic	Original			B	O	India	Pre-symptomatic / Asymptomatic	Yes
hCoV-19/India/KA-nimh-11076/2020	EPI_ISL_486402	04/05/2020	Asia / India / Karnataka	Human		Female	30	Asymptomatic	Original			B.6	O	India	Pre-symptomatic / Asymptomatic	Yes
hCoV-19/India/KA-nimh-11164/2020	EPI_ISL_486403	04/05/2020	Asia / India / Karnataka	Human		Male	45	Asymptomatic	Original			B.6	O	India	Pre-symptomatic / Asymptomatic	Yes
hCoV-19/India/KA-nimh-11877/2020	EPI_ISL_486404	06/05/2020	Asia / India / Karnataka	Human		Male	22	Asymptomatic	Original			B.4	O	India	Pre-symptomatic / Asymptomatic	Yes
hCoV-19/India/KA-nimh-14709/2020	EPI_ISL_486405	10/05/2020	Asia / India / Karnataka	Human		Male	34	Asymptomatic	Original			A	S	India	Pre-symptomatic / Asymptomatic	Yes
hCoV-19/India/KA-nimh-14712/2020	EPI_ISL_486406	10/05/2020	Asia / India / Karnataka	Human		Male	19	Asymptomatic	Original			A	S	India	Pre-symptomatic / Asymptomatic	Yes
hCoV-19/India/KA-nimh-14715/2020	EPI_ISL_486407	10/05/2020	Asia / India / Karnataka	Human		Male	16	Asymptomatic	Original			B.1	S	India	Pre-symptomatic / Asymptomatic	Yes
hCoV-19/India/KA-nimh-15819/2020	EPI_ISL_486408	10/05/2020	Asia / India / Karnataka	Human		Female	16	Asymptomatic	Original			B.6	O	India	Pre-symptomatic / Asymptomatic	Yes

hCoV-19/India/KA-nimh-15896/2020	EPI_ISL_486409	10/05/2020	Asia / India / Karnataka	Human	Male	17	Asymptomatic	Original		B.6	O	India	Pre-symptomatic / Asymptomatic	Yes
hCoV-19/Spain/PV-HUB-50378813/2020	EPI_ISL_490202	16/06/2020	Europe / Spain / Basque Country / Bizkaia	Human	Male	52	Live	Original		B.1.5	G	Spain	Undertermined	No (couldn't determine the patient status as either pre-symptomatic / asymptomatic or symptomatic conclusively)
hCoV-19/Spain/PV-HUB-50378855/2020	EPI_ISL_490203	10/06/2020	Europe / Spain / Basque Country / Bizkaia	Human	Male	58	Hospitalized	Original		B.1.5	G	Spain	Undertermined	No (couldn't determine the patient status as either pre-symptomatic / asymptomatic or symptomatic conclusively)
hCoV-19/Spain/PV-HUB-50388391/2020	EPI_ISL_490204	05/06/2020	Europe / Spain / Basque Country	Human	Female	41	Live	Original		B.1.5	G	Spain	Undertermined	No (couldn't determine the patient status as either pre-symptomatic / asymptomatic or symptomatic conclusively)
hCoV-19/Italy/CAM-INTPas-1/2020	EPI_ISL_492184	12/07/1905	Europe / Italy / Campania	Human	Male	47	Alive	Original		B.1	G	Italy	Undertermined	No (couldn't determine the patient status as either pre-symptomatic / asymptomatic or symptomatic conclusively)
hCoV-19/Italy/CAM-INTPas-4/2020	EPI_ISL_494756	12/07/1905	Europe / Italy / Campania	Human	Female	27	Alive	Original		B.1	G	Italy	Undertermined	No (couldn't determine the patient status as either pre-symptomatic / asymptomatic or symptomatic conclusively)
hCoV-19/Italy/CAM-INTPas-5/2020	EPI_ISL_494757	12/07/1905	Europe / Italy / Campania	Human	Female	84	Alive	Original		B.1	G	Italy	Undertermined	No (couldn't determine the patient status as either pre-symptomatic / asymptomatic or symptomatic conclusively)
hCoV-19/Italy/CAM-INTPas-6/2020	EPI_ISL_494759	12/07/1905	Europe / Italy / Campania	Human	Male	71	Alive	Original		B.1	O	Italy	Undertermined	No (couldn't determine the patient status as either pre-symptomatic / asymptomatic or symptomatic conclusively)
hCoV-19/Italy/CAM-INTPas-7/2020	EPI_ISL_494761	12/07/1905	Europe / Italy / Campania	Human	Female	60	Alive	Original		B.1.1	GR	Italy	Undertermined	No (couldn't determine the patient status as either pre-symptomatic / asymptomatic or symptomatic conclusively)
hCoV-19/Italy/CAM-INTPas-8/2020	EPI_ISL_494762	12/07/1905	Europe / Italy / Campania	Human	Male	61	Live	Original		B.1	G	Italy	Undertermined	No (couldn't determine the patient status as either pre-symptomatic / asymptomatic or symptomatic conclusively)
hCoV-19/Italy/CAM-INTPas-9/2020	EPI_ISL_494763	12/07/1905	Europe / Italy / Campania	Human	Male	23	Live	Original		B.1	O	Italy	Undertermined	No (couldn't determine the patient status as either pre-symptomatic / asymptomatic or symptomatic conclusively)
hCoV-19/Italy/CAM-INTPas-10/2020	EPI_ISL_494771	12/07/1905	Europe / Italy / Campania	Human	Male	78	Live	Original		B.1	G	Italy	Undertermined	No (couldn't determine the patient status as either pre-symptomatic / asymptomatic or symptomatic conclusively)
hCoV-19/Italy/CAM-INTPas-11/2020	EPI_ISL_494774	12/07/1905	Europe / Italy / Campania	Human	Male	26	Live	Original		B.1.1	GR	Italy	Undertermined	No (couldn't determine the patient status as either pre-symptomatic / asymptomatic or symptomatic conclusively)
hCoV-19/Italy/CAM-INTPas-12/2020	EPI_ISL_494775	12/07/1905	Europe / Italy / Campania	Human	Female	67	Live	Original		B.1	G	Italy	Undertermined	No (couldn't determine the patient status as either pre-symptomatic / asymptomatic or symptomatic conclusively)
hCoV-19/Italy/CAM-INTPas-13/2020	EPI_ISL_494776	12/07/1905	Europe / Italy / Campania	Human	Male	33	Live	Original		B.1	G	Italy	Undertermined	No (couldn't determine the patient status as either pre-symptomatic / asymptomatic or symptomatic conclusively)
hCoV-19/India/GJ-GBRC286/2020	EPI_ISL_495024	03/07/2020	Asia / India / Gujarat / Ghogha	Human	Male	50	Released	Original		B.1.1.32	GR	India	Undertermined	No (couldn't determine the patient status as either pre-symptomatic / asymptomatic or symptomatic conclusively)
hCoV-19/India/GJ-GBRC287/2020	EPI_ISL_495025	04/07/2020	Asia / India / Gujarat / Mahuva	Human	Male	50	Released	Original		B.1	G	India	Undertermined	No (couldn't determine the patient status as either pre-symptomatic / asymptomatic or symptomatic conclusively)
hCoV-19/India/GJ-GBRC289/2020	EPI_ISL_495026	05/06/2020	Asia / India / Gujarat / Vadodara	Human	Male	65	Released	Original		B.1	G	India	Undertermined	No (couldn't determine the patient status as either pre-symptomatic / asymptomatic or symptomatic conclusively)
hCoV-19/India/GJ-GBRC290a/2020	EPI_ISL_495027	15/06/2020	Asia / India / Gujarat / Vadodara	Human	Female	55	Released	Original		B.1.113	GH	India	Undertermined	No (couldn't determine the patient status as either pre-symptomatic / asymptomatic or symptomatic conclusively)
hCoV-19/India/GJ-GBRC290b/2020	EPI_ISL_495028	15/06/2020	Asia / India / Gujarat / Vadodara	Human	Female	55	Released	Original		B.1.1	GR	India	Undertermined	No (couldn't determine the patient status as either pre-symptomatic / asymptomatic or symptomatic conclusively)
hCoV-19/India/GJ-GBRC291/2020	EPI_ISL_495029	15/06/2020	Asia / India / Gujarat / Vadodara	Human	Female	55	Released	Original		B.1	G	India	Undertermined	No (couldn't determine the patient status as either pre-symptomatic / asymptomatic or symptomatic conclusively)
hCoV-19/India/GJ-GBRC292a/2020	EPI_ISL_495030	15/06/2020	Asia / India / Gujarat / Vadodara	Human	Male	67	Released	Original		B.1	G	India	Undertermined	No (couldn't determine the patient status as either pre-symptomatic / asymptomatic or symptomatic conclusively)
hCoV-19/India/GJ-GBRC292b/2020	EPI_ISL_495031	15/06/2020	Asia / India / Gujarat / Vadodara	Human	Male	67	Released	Original		B.1	G	India	Undertermined	No (couldn't determine the patient status as either pre-symptomatic / asymptomatic or symptomatic conclusively)
hCoV-19/India/GJ-GBRC293a/2020	EPI_ISL_495032	15/06/2020	Asia / India / Gujarat / Vadodara	Human	Male	53	Released	Original		B.1	G	India	Undertermined	No (couldn't determine the patient status as either pre-symptomatic / asymptomatic or symptomatic conclusively)
hCoV-19/India/GJ-GBRC293b/2020	EPI_ISL_495033	15/06/2020	Asia / India / Gujarat / Vadodara	Human	Male	53	Released	Original		B.1.36	GH	India	Undertermined	No (couldn't determine the patient status as either pre-symptomatic / asymptomatic or symptomatic conclusively)
hCoV-19/India/GJ-GBRC294/2020	EPI_ISL_495034	15/06/2020	Asia / India / Gujarat / Vadodara	Human	Male	37	Released	Original		B.1	G	India	Undertermined	No (couldn't determine the patient status as either pre-symptomatic / asymptomatic or symptomatic conclusively)
hCoV-19/India/GJ-GBRC295/2020	EPI_ISL_495035	15/06/2020	Asia / India / Gujarat / Vadodara	Human	Male	62	Released	Original		B.1	G	India	Undertermined	No (couldn't determine the patient status as either pre-symptomatic / asymptomatic or symptomatic conclusively)
hCoV-19/India/GJ-GBRC297a/2020	EPI_ISL_495038	15/06/2020	Asia / India / Gujarat / Vadodara	Human	Male	71	Deceased	Original		B.1	G	India	Symptomatic	Yes
hCoV-19/India/GJ-GBRC297b/2020	EPI_ISL_495039	15/06/2020	Asia / India / Gujarat / Vadodara	Human	Male	71	Deceased	Original		B.1	G	India	Symptomatic	Yes
hCoV-19/India/GJ-GBRC299a/2020	EPI_ISL_495042	15/06/2020	Asia / India / Gujarat / Vadodara	Human	Male	62	Released	Original		B.1.36	GH	India	Undertermined	No (couldn't determine the patient status as either pre-symptomatic / asymptomatic or symptomatic conclusively)
hCoV-19/India/GJ-GBRC299b/2020	EPI_ISL_495043	15/06/2020	Asia / India / Gujarat / Vadodara	Human	Male	62	Released	Original		B.1.113	GH	India	Undertermined	No (couldn't determine the patient status as either pre-symptomatic / asymptomatic or symptomatic conclusively)
hCoV-19/India/GJ-GBRC300a/2020	EPI_ISL_495044	15/06/2020	Asia / India / Gujarat / Vadodara	Human	Male	43	Released	Original		B.1.113	GH	India	Undertermined	No (couldn't determine the patient status as either pre-symptomatic / asymptomatic or symptomatic conclusively)
hCoV-19/India/GJ-GBRC300b/2020	EPI_ISL_495045	15/06/2020	Asia / India / Gujarat / Vadodara	Human	Male	43	Released	Original		B.1.113	GH	India	Undertermined	No (couldn't determine the patient status as either pre-symptomatic / asymptomatic or symptomatic conclusively)
hCoV-19/India/GJ-GBRC301a/2020	EPI_ISL_495046	15/06/2020	Asia / India / Gujarat / Vadodara	Human	Female	50	Deceased	Original		B.1.36	GH	India	Symptomatic	Yes
hCoV-19/India/GJ-GBRC301b/2020	EPI_ISL_495047	15/06/2020	Asia / India / Gujarat / Vadodara	Human	Female	50	Deceased	Original		B.1.36	GH	India	Symptomatic	Yes
hCoV-19/India/GJ-GBRC302a/2020	EPI_ISL_495048	15/06/2020	Asia / India / Gujarat / Vadodara	Human	Female	50	Released	Original		B.1.113	GH	India	Undertermined	No (couldn't determine the patient status as either pre-symptomatic / asymptomatic or symptomatic conclusively)
hCoV-19/India/GJ-GBRC302b/2020	EPI_ISL_495049	15/06/2020	Asia / India / Gujarat / Vadodara	Human	Female	50	Released	Original		B.1.113	G	India	Undertermined	No (couldn't determine the patient status as either pre-symptomatic / asymptomatic or symptomatic conclusively)
hCoV-19/India/GJ-GBRC303a/2020	EPI_ISL_495050	15/06/2020	Asia / India / Gujarat / Vadodara	Human	Male	41	Released	Original		B.1.113	GH	India	Undertermined	No (couldn't determine the patient status as either pre-symptomatic / asymptomatic or symptomatic conclusively)
hCoV-19/India/GJ-GBRC303b/2020	EPI_ISL_495051	15/06/2020	Asia / India / Gujarat / Vadodara	Human	Male	41	Released	Original		B.1.1	G	India	Undertermined	No (couldn't determine the patient status as either pre-symptomatic / asymptomatic or symptomatic conclusively)
hCoV-19/India/GJ-GBRC304a/2020	EPI_ISL_495052	15/06/2020	Asia / India / Gujarat / Vadodara	Human	Male	42	Released	Original		B.1	G	India	Undertermined	No (couldn't determine the patient status as either pre-symptomatic / asymptomatic or symptomatic conclusively)
hCoV-19/India/GJ-GBRC304b/2020	EPI_ISL_495053	15/06/2020	Asia / India / Gujarat / Vadodara	Human	Male	42	Released	Original		B.1.36	GH	India	Undertermined	No (couldn't determine the patient status as either pre-symptomatic / asymptomatic or symptomatic conclusively)
hCoV-19/India/GJ-GBRC305a/2020	EPI_ISL_495054	15/06/2020	Asia / India / Gujarat / Vadodara	Human	Male	67	Released	Original		B.1	G	India	Undertermined	No (couldn't determine the patient status as either pre-symptomatic / asymptomatic or symptomatic conclusively)
hCoV-19/India/GJ-GBRC305b/2020	EPI_ISL_495055	15/06/2020	Asia / India / Gujarat / Vadodara	Human	Male	67	Released	Original		B.1.113	GH	India	Undertermined	No (couldn't determine the patient status as either pre-symptomatic / asymptomatic or symptomatic conclusively)
hCoV-19/India/GJ-GBRC306a/2020	EPI_ISL_495056	15/06/2020	Asia / India / Gujarat / Vadodara	Human	Male	45	Deceased	Original		B.1.113	GH	India	Symptomatic	Yes
hCoV-19/India/GJ-GBRC306b/2020	EPI_ISL_495057	15/06/2020	Asia / India / Gujarat / Vadodara	Human	Male	45	Deceased	Original		B.1.113	GH	India	Symptomatic	Yes
hCoV-19/India/GJ-GBRC307a/2020	EPI_ISL_495058	15/06/2020	Asia / India / Gujarat / Vadodara	Human	Male	50	Hospitalized	Original		B.1	G	India	Undertermined	No (couldn't determine the patient status as either pre-symptomatic / asymptomatic or symptomatic conclusively)
hCoV-19/India/GJ-GBRC307b/2020	EPI_ISL_495059	15/06/2020	Asia / India / Gujarat / Vadodara	Human	Male	50	Hospitalized	Original		B.1.113	GH	India	Undertermined	No (couldn't determine the patient status as either pre-symptomatic / asymptomatic or symptomatic conclusively)
hCoV-19/India/GJ-GBRC308/2020	EPI_ISL_495060	15/06/2020	Asia / India / Gujarat / Vadodara	Human	Female	32	Released	Original		B.1.36	GH	India	Undertermined	No (couldn't determine the patient status as either pre-symptomatic / asymptomatic or symptomatic conclusively)
hCoV-19/India/GJ-GBRC309a/2020	EPI_ISL_495061	15/06/2020	Asia / India / Gujarat / Vadodara	Human	Male	75	Deceased	Original		B.1	G	India	Symptomatic	Yes
hCoV-19/India/GJ-GBRC309b/2020	EPI_ISL_495062	15/06/2020	Asia / India / Gujarat / Vadodara	Human	Male	75	Deceased	Original		B.1	G	India	Symptomatic	Yes
hCoV-19/India/GJ-GBRC310a/2020	EPI_ISL_495063	19/06/2020	Asia / India / Gujarat / Prantij	Human	Male	55	Deceased	Original		B.1.113	GH	India	Symptomatic	Yes
hCoV-19/India/GJ-GBRC310b/2020	EPI_ISL_495064	19/06/2020	Asia / India / Gujarat / Prantij	Human	Male	55	Deceased	Original		B.1.113	GH	India	Symptomatic	Yes
hCoV-19/India/GJ-GBRC311a/2020	EPI_ISL_495065	20/06/2020	Asia / India / Gujarat / Mandvi	Human	Male	26	Released	Original		B.1.36	GH	India	Undertermined	No (couldn't determine the patient status as either pre-symptomatic / asymptomatic or symptomatic conclusively)
hCoV-19/India/GJ-GBRC311b/2020	EPI_ISL_495066	20/06/2020	Asia / India / Gujarat / Mandvi	Human	Male	26	Released	Original		B.1.113	GH	India	Undertermined	No (couldn't determine the patient status as either pre-symptomatic / asymptomatic or symptomatic conclusively)
hCoV-19/India/GJ-GBRC312a/2020	EPI_ISL_495067	08/06/2020	Asia / India / Gujarat / Kheda	Human	Male	52	Released	Original		B.1	G	India	Undertermined	No (couldn't determine the patient status as either pre-symptomatic / asymptomatic or symptomatic conclusively)
hCoV-19/India/GJ-GBRC312b/2020	EPI_ISL_495068	08/06/2020	Asia / India / Gujarat / Kheda	Human	Male	52	Released	Original		B.1	G	India	Undertermined	No (couldn't determine the patient status as either pre-symptomatic / asymptomatic or symptomatic conclusively)
hCoV-19/India/GJ-GBRC313/2020	EPI_ISL_495069	08/06/2020	Asia / India / Gujarat / Nadiad	Human	Male	33	Released	Original		B.1.113	GH	India	Undertermined	No (couldn't determine the patient status as either pre-symptomatic / asymptomatic or symptomatic conclusively)
hCoV-19/India/GJ-GBRC314/2020	EPI_ISL_495070	17/06/2020	Asia / India / Gujarat / Choryasi	Human	Male	24	Released	Original		B.1	G	India	Undertermined	No (couldn't determine the patient status as either pre-symptomatic / asymptomatic or symptomatic conclusively)
hCoV-19/India/GJ-GBRC315a/2020	EPI_ISL_495071	17/06/2020	Asia / India / Gujarat / Kamrej	Human	Male	45	Released	Original		B.1	GH	India	Undertermined	No (couldn't determine the patient status as either pre-symptomatic / asymptomatic or symptomatic conclusively)
hCoV-19/India/GJ-GBRC315b/2020	EPI_ISL_495072	17/06/2020	Asia / India / Gujarat / Kamrej	Human	Male	45	Released	Original		B.1	G	India	Undertermined	No (couldn't determine the patient status as either pre-symptomatic / asymptomatic or symptomatic conclusively)
hCoV-19/India/GJ-GBRC316a/2020	EPI_ISL_495073	17/06/2020	Asia / India / Gujarat / Surat	Human	Male	53	Released	Original		B.1	GH	India	Undertermined	No (couldn't determine the patient status as either pre-symptomatic / asymptomatic or symptomatic conclusively)
hCoV-19/India/GJ-GBRC316b/2020	EPI_ISL_495074	17/06/2020	Asia / India / Gujarat / Surat	Human	Male	53	Released	Original		B.1.36	G	India	Undertermined	No (couldn't determine the patient status as either pre-symptomatic / asymptomatic or symptomatic conclusively)
hCoV-19/India/GJ-GBRC318a/2020	EPI_ISL_495075	17/06/2020	Asia / India / Gujarat / Surat	Human	Male	76	Released	Original		B.1	G	India	Undertermined	No (couldn't determine the patient status as either pre-symptomatic / asymptomatic or symptomatic conclusively)
hCoV-19/India/GJ-GBRC318b/2020	EPI_ISL_495076	17/06/2020	Asia / India / Gujarat / Surat	Human	Male	76	Released	Original		B.1	GH	India	Undertermined	No (couldn't determine the patient status as either pre-symptomatic / asymptomatic or symptomatic conclusively)
hCoV-19/India/GJ-GBRC319/2020	EPI_ISL_495077	17/06/2020	Asia / India / Gujarat / Surat	Human	Male	48	Released	Original		A	S	India	Undertermined	No (couldn't determine the patient status as either pre-symptomatic / asymptomatic or symptomatic conclusively)
hCoV-19/India/GJ-GBRC320/2020	EPI_ISL_495078	17/06/2020	Asia / India / Gujarat / Surat	Human	Male	40	Released	Original		B.1	G	India	Undertermined	No (couldn't determine the patient status as either pre-symptomatic / asymptomatic or symptomatic conclusively)
hCoV-19/India/GJ-GBRC323a/2020	EPI_ISL_495079	12/06/2020	Asia / India / Gujarat / Surat	Human	Female	79	Released	Original		B.1.113	GH	India	Undertermined	No (couldn't determine the patient status as either pre-symptomatic / asymptomatic or symptomatic conclusively)
hCoV-19/India/GJ-GBRC323b/2020	EPI_ISL_495080	12/06/2020	Asia / India / Gujarat / Surat	Human	Female	79	Released	Original		B	G	India	Undertermined	No (couldn't determine the patient status as either pre-symptomatic / asymptomatic or symptomatic conclusively)
hCoV-19/Brazil/RO-01/2020	EPI_ISL_514131	11/05/2020	South America / Brazil / Rondonia / Jaru	Human	Female	67	Live	Original		B.1.1.33	GR	Brazil	Undertermined	No (couldn't determine the patient status as either pre-symptomatic / asymptomatic or symptomatic conclusively)
hCoV-19/Brazil/RO-02/2020	EPI_ISL_514132	11/05/2020	South America / Brazil / Rondonia / Porto Velho	Human	Female	70	Live	Original		B.1.1.33	GR	Brazil	Undertermined	No (couldn't determine the patient status as either pre-symptomatic / asymptomatic or symptomatic conclusively)
hCoV-19/Brazil/RO-03/2020	EPI_ISL_514133	11/05/2020	South America / Brazil / Rondonia / Porto Velho	Human	Female	36	Live	Original		B.1.1.33	GR	Brazil	Undertermined	No (couldn't determine the patient status as either pre-symptomatic / asymptomatic or symptomatic conclusively)
hCoV-19/Brazil/RO-04/2020	EPI_ISL_514134	11/05/2020	South America / Brazil / Rondonia / Porto Velho	Human	Female	46	Live	Original		B.1	G	Brazil	Undertermined	No (couldn't determine the patient status as either pre-symptomatic / asymptomatic or symptomatic conclusively)
hCoV-19/Brazil/RO-05/2020	EPI_ISL_514135	05/05/2020	South America / Brazil / Rondonia / Porto Velho	Human	Female	41	Live	Original		B.1.1.33	GR	Brazil	Undertermined	No (couldn't determine the patient status as either pre-symptomatic / asymptomatic or symptomatic conclusively)
hCoV-19/Brazil/RO-06/2020	EPI_ISL_514136	05/05/2020	South America / Brazil / Rondonia / Porto Velho	Human	Male	38	Live	Original		B.1	G	Brazil	Undertermined	No (couldn't determine the patient status as either pre-symptomatic / asymptomatic or symptomatic conclusively)
hCoV-19/Brazil/RO-07/2020	EPI_ISL_514137	05/05/2020	South America / Brazil / Rondonia / Porto Velho	Human	Female	56	Live	Original		B.1.1.33	GR	Brazil	Undertermined	No (couldn't determine the patient status as either pre-symptomatic / asymptomatic or symptomatic conclusively)
hCoV-19/Brazil/RO-08/2020	EPI_ISL_514138	05/06/2020	South America / Brazil / Rondonia / Porto Velho	Human	Female	37	Live	Original		B.1.1.33	GR	Brazil	Undertermined	No (couldn't determine the patient status as either pre-symptomatic / asymptomatic or symptomatic conclusively)
hCoV-19/Canada/AB-Z0002/2020	EPI_ISL_515466	15/07/2020	North America / Canada / Alberta	Human	Male	45	Recovered	Original	Saliva	B.1	GH	Canada	Undertermined	No (couldn't determine the patient status as either pre-symptomatic / asymptomatic or symptomatic conclusively)
hCoV-19/Mexico/BC-InDRE-44/2020	EPI_ISL_516608	26/06/2020	North America / Mexico / Baja California	Human	Male	33	Released	Original	Oro-pharyngeal swab	B.1	G	Mexico	Undertermined	No (couldn't determine the patient status as either pre-symptomatic / asymptomatic or symptomatic conclusively)
hCoV-19/Mexico/BCN-InDRE-45/2020	EPI_ISL_516609	27/06/2020	North America / Mexico / Baja California	Human	Male	41	Released	Original	Oro-pharyngeal swab	B.1	G	Mexico	Undertermined	No (couldn't determine the patient status as either pre-symptomatic / asymptomatic or symptomatic conclusively)
hCoV-19/Mexico/BCN-InDRE-50/2020	EPI_ISL_516610	30/06/2020	North America / Mexico / Baja California	Human	Male	25	Released	Original	Oro-pharyngeal swab	B.1	G	Mexico	Undertermined	No (couldn't determine the patient status as either pre-symptomatic / asymptomatic or symptomatic conclusively)
hCoV-19/Mexico/BCN-InDRE-51/2020	EPI_ISL_516611	05/07/2020	North America / Mexico / Baja California	Human	Male	74	Deceased	Original	Oro-pharyngeal swab	B.1	G	Mexico	Symptomatic	Yes
hCoV-19/Mexico/BCN-InDRE-52/2020	EPI_ISL_516612	06/07/2020	North America / Mexico / Baja California	Human	Male	20	Released	Original	Oro-pharyngeal swab	B.1	G	Mexico	Undertermined	No (couldn't determine the patient status as either pre-symptomatic / asymptomatic or symptomatic conclusively)
hCoV-19/Mexico/CHH-InDRE-43/2020	EPI_ISL_516613	26/06/2020	North America / Mexico / Chihuahua	Human	Female	22	Released	Original	Oro-pharyngeal swab	B.1.2	GH	Mexico	Undertermined	No (couldn't determine the patient status as either pre-symptomatic / asymptomatic or symptomatic conclusively)
hCoV-19/Mexico/CHH-InDRE-46/2020	EPI_ISL_516614	28/06/2020	North America / Mexico / Chihuahua	Human	Male	63	Released	Original	Oro-pharyngeal swab	B.1.5	G	Mexico	Undertermined	No (couldn't determine the patient status as either pre-symptomatic / asymptomatic or symptomatic conclusively)
hCoV-19/Mexico/CHH-InDRE-47/2020	EPI_ISL_516615	27/06/2020	North America / Mexico / Chihuahua	Human	Male	29	Released	Original	Oro-pharyngeal swab	B.1	GH	Mexico	Undertermined	No (couldn't determine the patient status as either pre-symptomatic / asymptomatic or symptomatic conclusively)
hCoV-19/Mexico/CHH-InDRE-48/2020	EPI_ISL_516616	30/06/2020	North America / Mexico / Chihuahua	Human	Female	52	Released	Original	Oro-pharyngeal swab	B.1.5	G	Mexico	Undertermined	No (couldn't determine the patient status as either pre-symptomatic / asymptomatic or symptomatic conclusively)
hCoV-19/Mexico/CHH-InDRE-49/2020	EPI_ISL_516617	01/07/2020	North America / Mexico / Chihuahua	Human	Female	49	Released	Original	Oro-pharyngeal swab	B.1.5	G	Mexico	Undertermined	No (couldn't determine the patient status as either pre-symptomatic / asymptomatic or symptomatic conclusively)
hCoV-19/Mexico/COA-InDRE-36/2020	EPI_ISL_516618	13/06/2020	North America / Mexico / Coahuila	Human	Male	51	Released	Original	Oro-pharyngeal swab	B.1.1	GR	Mexico	Undertermined	No (couldn't determine the patient status as either pre-symptomatic / asymptomatic or symptomatic conclusively)
hCoV-19/Mexico/COA-InDRE-37/2020	EPI_ISL_516619	13/06/2020	North America / Mexico / Coahuila	Human	Female	32	Released	Original	Oro-pharyngeal swab	B.1.1	GR	Mexico	Undertermined	No (couldn't determine the patient status as either pre-symptomatic / asymptomatic or symptomatic conclusively)
hCoV-19/Mexico/COA-InDRE_40/2020	EPI_ISL_516620	19/06/2020	North America / Mexico / Coahuila	Human	Female	24	Released	Original	Oro-pharyngeal swab	B.1.1	GR	Mexico	Undertermined	No (couldn't determine the patient status as either pre-symptomatic / asymptomatic or symptomatic conclusively)
hCoV-19/Mexico/COA-InDRE_41/2020	EPI_ISL_516621	26/06/2020	North America / Mexico / Coahuila de Zaragoza	Human	Male	69	Released	Original	Oro-pharyngeal swab	B.1	G	Mexico	Undertermined	No (couldn't determine the patient status as either pre-symptomatic / asymptomatic or symptomatic conclusively)
hCoV-19/Mexico/SON-InDRE_35/2020	EPI_ISL_516622	15/05/2020	North America / Mexico / Sonora	Human	Male	20	Released	Original	Oro-pharyngeal swab	B.1	G	Mexico	Undertermined	No (couldn't determine the patient status as either pre-symptomatic / asymptomatic or symptomatic conclusively)
hCoV-19/Mexico/SON-InDRE_42/2020	EPI_ISL_516623	06/06/2020	North America / Mexico / Sonora	Human	Male	35	Deceased	Original	Oro-pharyngeal swab	B.1	G	Mexico	Symptomatic	Yes
hCoV-19/Mexico/TAM-InDRE_38/2020	EPI_ISL_516624	19/06/2020	North America / Mexico / Tamaulipas	Human	Male	49	Released	Original	Oro-pharyngeal swab	B.1	GH	Mexico	Undertermined	No (couldn't determine the patient status as either pre-symptomatic / asymptomatic or symptomatic conclusively)
hCoV-19/Mexico/TAM-InDRE_39/2020	EPI_ISL_516625	15/06/2020	North America / Mexico / Tamaulipas	Human	Male	79	Deceased	Original	Oro-pharyngeal swab	B.1.5	G	Mexico	Symptomatic	Yes
hCoV-19/USA/SC-20-0946-T07/2020	EPI_ISL_516749	17/06/2020	North America / USA / South Carolina	Human	Male	19	Live	Original		B.1.26	GH	USA	Undertermined	No (couldn't determine the patient status as either pre-symptomatic / asymptomatic or symptomatic conclusively)
hCoV-19/USA/SC-20-0946-T10/2020	EPI_ISL_516750	20/06/2020	North America / USA / South Carolina	Human	Male	19	Live	Original		B.1.26	GH	USA	Undertermined	No (couldn't determine the patient status as either pre-symptomatic / asymptomatic or symptomatic conclusively)
hCoV-19/USA/SC-20-0946-T14/2020	EPI_ISL_516751	24/06/2020	North America / USA / South Carolina	Human	Male	19	Live	Original		B.1.26	GH	USA	Undertermined	No (couldn't determine the patient status as either pre-symptomatic / asymptomatic or symptomatic conclusively)
hCoV-19/USA/SC-20-1167-T07/2020	EPI_ISL_516752	24/06/2020	North America / USA / South Carolina	Human	Male	18	Live	Original		B.1.2	GH	USA	Undertermined	No (couldn't determine the patient status as either pre-symptomatic / asymptomatic or symptomatic conclusively)
hCoV-19/USA/SC-20-1273-T07/2020	EPI_ISL_516753	30/06/2020	North America / USA / South Carolina	Human	Male	18	Live	Original		B.1	GH	USA	Undertermined	No (couldn't determine the patient status as either pre-symptomatic / asymptomatic or symptomatic conclusively)
hCoV-19/USA/SC-20-1278-T07/2020	EPI_ISL_516754	30/06/2020	North America / USA / South Carolina	Human	Male	19	Live	Original		B.1	G	USA	Undertermined	No (couldn't determine the patient status as either pre-symptomatic / asymptomatic or symptomatic conclusively)
hCoV-19/USA/SC-20-1385-T10/2020	EPI_ISL_516755	03/07/2020	North America / USA / South Carolina	Human	Male	18	Live	Original		B.1.2	GH	USA	Undertermined	No (couldn't determine the patient status as either pre-symptomatic / asymptomatic or symptomatic conclusively)
hCoV-19/USA/SC-20-1389-T14/2020	EPI_ISL_516756	07/07/2020	North America / USA / South Carolina	Human	Male	18	Live	Original		B.1.2	GH	USA	Undertermined	No (couldn't determine the patient status as either pre-symptomatic / asymptomatic or symptomatic conclusively)
hCoV-19/USA/SC-20-1392-T14/2020	EPI_ISL_516757	07/07/2020	North America / USA / South Carolina	Human	Male	18	Live	Original		B.1.2	GH	USA	Undertermined	No (couldn't determine the patient status as either pre-symptomatic / asymptomatic or symptomatic conclusively)
hCoV-19/USA/SC-20-1398-T14/2020	EPI_ISL_516758	07/07/2020	North America / USA / South Carolina	Human	Male	18	Live	Original		B.1.2	GH	USA	Undertermined	No (couldn't determine the patient status as either pre-symptomatic / asymptomatic or symptomatic conclusively)
hCoV-19/USA/SC-20-1402-T07/2020	EPI_ISL_516759	30/06/2020	North America / USA / South Carolina	Human	Male	18	Live	Original		B.1	GH	USA	Undertermined	No (couldn't determine the patient status as either pre-symptomatic / asymptomatic or symptomatic conclusively)
hCoV-19/USA/SC-20-1402-T08/2020	EPI_ISL_516760	01/07/2020	North America / USA / South Carolina	Human	Male	18	Live	Original		B.1.2	GH	USA	Undertermined	No (couldn't determine the patient status as either pre-symptomatic / asymptomatic or symptomatic conclusively)
hCoV-19/USA/SC-20-1411-T14/2020	EPI_ISL_516761	07/07/2020	North America / USA / South Carolina	Human	Male	19	Live	Original		B.1.1	GR	USA	Undertermined	No (couldn't determine the patient status as either pre-symptomatic / asymptomatic or symptomatic conclusively)
hCoV-19/USA/SC-20-1413-T10/2020	EPI_ISL_516762	03/07/2020	North America / USA / South Carolina	Human	Male	18	Live	Original		B.1.1	GR	USA	Undertermined	No (couldn't determine the patient status as either pre-symptomatic / asymptomatic or symptomatic conclusively)
hCoV-19/USA/SC-20-1415-T10/2020	EPI_ISL_516763	03/07/2020	North America / USA / South Carolina	Human	Male	18	Live	Original		B.1.2	GH	USA	Undertermined	No (couldn't determine the patient status as either pre-symptomatic / asymptomatic or symptomatic conclusively)
hCoV-19/USA/SC-20-1418-T07/2020	EPI_ISL_516764	01/07/2020	North America / USA / South Carolina	Human	Male	18	Live	Original		B.1.1	GR	USA	Undertermined	No (couldn't determine the patient status as either pre-symptomatic / asymptomatic or symptomatic conclusively)
hCoV-19/USA/SC-20-1419-T14/2020	EPI_ISL_516765	08/07/2020	North America / USA / South Carolina	Human	Male	18	Live	Original		B.1.1	GR	USA	Undertermined	No (couldn't determine the patient status as either pre-symptomatic / asymptomatic or symptomatic conclusively)
hCoV-19/USA/SC-20-1448-T14/2020	EPI_ISL_516766	08/07/2020	North America / USA / South Carolina	Human	Male	18	Live	Original		B.1.1	GR	USA	Undertermined	No (couldn't determine the patient status as either pre-symptomatic / asymptomatic or symptomatic conclusively)
hCoV-19/USA/SC-20-1505-T03/2020	EPI_ISL_516767	03/07/2020	North America / USA / South Carolina	Human	Female	18	Live	Original		B.1.1	G	USA	Undertermined	No (couldn't determine the patient status as either pre-symptomatic / asymptomatic or symptomatic conclusively)
hCoV-19/USA/SC-20-1568-T07/2020	EPI_ISL_516768	07/07/2020	North America / USA / South Carolina	Human	Male	18	Live	Original		B.1	G	USA	Undertermined	No (couldn't determine the patient status as either pre-symptomatic / asymptomatic or symptomatic conclusively)
hCoV-19/USA/SC-20-1575-T07/2020	EPI_ISL_516769	07/07/2020	North America / USA / South Carolina	Human	Male	23	Live	Original		B.1.1	G	USA	Undertermined	No (couldn't determine the patient status as either pre-symptomatic / asymptomatic or symptomatic conclusively)
hCoV-19/USA/SC-20-1577-T07/2020	EPI_ISL_516770	07/07/2020	North America / USA / South Carolina	Human	Male	18	Live	Original		B.1	G	USA	Undertermined	No (couldn't determine the patient status as either pre-symptomatic / asymptomatic or symptomatic conclusively)
hCoV-19/USA/SC-20-1588-T07/2020	EPI_ISL_516771	07/07/2020	North America / USA / South Carolina	Human	Male	19	Live	Original		B.1.2	GH	USA	Undertermined	No (couldn't determine the patient status as either pre-symptomatic / asymptomatic or symptomatic conclusively)
hCoV-19/USA/SC-20-1597-T14/2020	EPI_ISL_516772	14/07/2020	North America / USA / South Carolina	Human	Male	18	Live	Original		B.1.2	GH	USA	Undertermined	No (couldn't determine the patient status as either pre-symptomatic / asymptomatic or symptomatic conclusively)
hCoV-19/USA/SC-20-1601-T03/2020	EPI_ISL_516773	03/07/2020	North America / USA / South Carolina	Human	Male	18	Live	Original		B.1.2	GH	USA	Undertermined	No (couldn't determine the patient status as either pre-symptomatic / asymptomatic or symptomatic conclusively)
hCoV-19/USA/SC-20-1609-T07/2020	EPI_ISL_516774	07/07/2020	North America / USA / South Carolina	Human	Male	19	Live	Original		B.1.1	GR	USA	Undertermined	No (couldn't determine the patient status as either pre-symptomatic / asymptomatic or symptomatic conclusively)
hCoV-19/USA/SC-20-1615-T14/2020	EPI_ISL_516775	14/07/2020	North America / USA / South Carolina	Human	Male	23	Live	Original		B.1.2	GH	USA	Undertermined	No (couldn't determine the patient status as either pre-symptomatic / asymptomatic or symptomatic conclusively)
hCoV-19/USA/SC-20-1633-T07/2020	EPI_ISL_516776	07/07/2020	North America / USA / South Carolina	Human	Male	20	Live	Original		B.1.2	GH	USA	Undertermined	No (couldn't determine the patient status as either pre-symptomatic / asymptomatic or symptomatic conclusively)
hCoV-19/USA/SC-20-1803-T07/2020	EPI_ISL_516777	21/07/2020	North America / USA / South Carolina	Human	Male	18	Live	Original		B.1.1	G	USA	Undertermined	No (couldn't determine the patient status as either pre-symptomatic / asymptomatic or symptomatic conclusively)
hCoV-19/USA/SC-20-1820-T07/2020	EPI_ISL_516778	21/07/2020	North America / USA / South Carolina	Human	Male	18	Live	Original		B.1.1	G	USA	Undertermined	No (couldn't determine the patient status as either pre-symptomatic / asymptomatic or symptomatic conclusively)
hCoV-19/USA/SC-20-1936-T00/2020	EPI_ISL_516779	15/07/2020	North America / USA / South Carolina	Human	Male	18	Live	Original		B.1.1	G	USA	Undertermined	No (couldn't determine the patient status as either pre-symptomatic / asymptomatic or symptomatic conclusively)
hCoV-19/USA/SC-20-1958-T00/2020	EPI_ISL_516780	15/07/2020	North America / USA / South Carolina	Human	Male	18	Live	Original		B.1	G	USA	Undertermined	No (couldn't determine the patient status as either pre-symptomatic / asymptomatic or symptomatic conclusively)
hCoV-19/USA/SC-20-1963-T00/2020	EPI_ISL_516781	15/07/2020	North America / USA / South Carolina	Human	Male	18	Live	Original		B.1	G	USA	Undertermined	No (couldn't determine the patient status as either pre-symptomatic / asymptomatic or symptomatic conclusively)
hCoV-19/USA/SC-20-1963-T07/2020	EPI_ISL_516782	22/07/2020	North America / USA / South Carolina	Human	Male	18	Live	Original		B.1.1	GR	USA	Undertermined	No (couldn't determine the patient status as either pre-symptomatic / asymptomatic or symptomatic conclusively)
hCoV-19/Singapore/766_2/2020	EPI_ISL_517996	08/07/2020	Asia / Singapore	Human	Male	40	Hospitalized	Original		B.6	O	Singapore	Undertermined	No (couldn't determine the patient status as either pre-symptomatic / asymptomatic or symptomatic conclusively)
hCoV-19/Singapore/834/2020	EPI_ISL_517997	15/06/2020	Asia / Singapore	Human	Male	26	Hospitalized	Original		B.6	O	Singapore	Undertermined	No (couldn't determine the patient status as either pre-symptomatic / asymptomatic or symptomatic conclusively)
hCoV-19/Singapore/835/2020	EPI_ISL_517998	17/06/2020	Asia / Singapore	Human	Male	43	Hospitalized	Original		B.6	O	Singapore	Undertermined	No (couldn't determine the patient status as either pre-symptomatic / asymptomatic or symptomatic conclusively)
hCoV-19/Singapore/836/2020	EPI_ISL_517999	16/06/2020	Asia / Singapore	Human	Male	43	Hospitalized	Original		B.6	O	Singapore	Undertermined	No (couldn't determine the patient status as either pre-symptomatic / asymptomatic or symptomatic conclusively)
hCoV-19/Dominican Republic/ICGEB-UNIBE024/2020	EPI_ISL_522406	28/02/2020	North America / Dominican Republic	Human	Male	62	Hospitalized	Original	Oropharyngeal swab	B.1	O	Dominican Republic	Undertermined	No (couldn't determine the patient status as either pre-symptomatic / asymptomatic or symptomatic conclusively)
hCoV-19/Dominican Republic/ICGEB_UNIBE051/2020	EPI_ISL_523811	01/03/2020	North America / Dominican Republic	Human	Female	unknown	Hospitalized	Original	Nasopharyngeal Swab	B	GH	Dominican Republic	Undertermined	No (couldn't determine the patient status as either pre-symptomatic / asymptomatic or symptomatic conclusively)
hCoV-19/Dominican Republic/ICGEB_UNIBE258/2020	EPI_ISL_523812	04/03/2020	North America / Dominican Republic	Human	Male	unknown	Hospitalized	Original	Nasopharyngeal Swab	B	GH	Dominican Republic	Undertermined	No (couldn't determine the patient status as either pre-symptomatic / asymptomatic or symptomatic conclusively)
hCoV-19/India/GJ-GBRC-382a/2020	EPI_ISL_525419	18/06/2020	Asia / India / Gujarat / Ahmedabad	Human	Female	47	Released	Original		B.1.113	GH	India	Undertermined	No (couldn't determine the patient status as either pre-symptomatic / asymptomatic or symptomatic conclusively)
hCoV-19/India/GJ-GBRC-382b/2020	EPI_ISL_525420	18/06/2020	Asia / India / Gujarat / Ahmedabad	Human	Female	47	Released	Original		B.1.113	GH	India	Undertermined	No (couldn't determine the patient status as either pre-symptomatic / asymptomatic or symptomatic conclusively)
hCoV-19/India/GJ-GBRC-397a/2020	EPI_ISL_525421	25/06/2020	Asia / India / Gujarat / Ahmedabad	Human	Male	62	Released	Original		B.1.113	GH	India	Undertermined	No (couldn't determine the patient status as either pre-symptomatic / asymptomatic or symptomatic conclusively)
hCoV-19/India/GJ-GBRC-397b/2020	EPI_ISL_525422	25/06/2020	Asia / India / Gujarat / Ahmedabad	Human	Male	62	Released	Original		B.1.113	GH	India	Undertermined	No (couldn't determine the patient status as either pre-symptomatic / asymptomatic or symptomatic conclusively)
hCoV-19/Ecuador/USFQ-163/2020	EPI_ISL_525430	20/05/2020	South America / Ecuador / Pichincha / Quito	Human	Male	unknown	Live	Original	Nasopharyngeal swab	B.1.9	GH	Ecuador	Undertermined	No (couldn't determine the patient status as either pre-symptomatic / asymptomatic or symptomatic conclusively)
hCoV-19/United Arab Emirates/13/2020	EPI_ISL_528687	05/05/2020	Asia / United Arab Emirates / Abu Dhabi	Human	Male	45	Hospitalized	Original		B.1.1	GR	United Arab Emirates	Undertermined	No (couldn't determine the patient status as either pre-symptomatic / asymptomatic or symptomatic conclusively)
hCoV-19/United Arab Emirates/16/2020	EPI_ISL_528689	05/05/2020	Asia / United Arab Emirates / Abu Dhabi	Human	Female	44	Hospitalized	Original		B.1.1	GR	United Arab Emirates	Undertermined	No (couldn't determine the patient status as either pre-symptomatic / asymptomatic or symptomatic conclusively)
hCoV-19/United Arab Emirates/195/2020	EPI_ISL_528691	07/06/2020	Asia / United Arab Emirates / Abu Dhabi	Human	Male	71	Hospitalized	Original		B.1.1	GR	United Arab Emirates	Undertermined	No (couldn't determine the patient status as either pre-symptomatic / asymptomatic or symptomatic conclusively)
hCoV-19/United Arab Emirates/2/2020	EPI_ISL_528692	02/05/2020	Asia / United Arab Emirates / Abu Dhabi	Human	Female	30	Hospitalized	Original		B.1.1	GR	United Arab Emirates	Undertermined	No (couldn't determine the patient status as either pre-symptomatic / asymptomatic or symptomatic conclusively)
hCoV-19/United Arab Emirates/21R/2020	EPI_ISL_528693	05/05/2020	Asia / United Arab Emirates / Abu Dhabi	Human	Male	34	Hospitalized	Original		B.1	G	United Arab Emirates	Undertermined	No (couldn't determine the patient status as either pre-symptomatic / asymptomatic or symptomatic conclusively)
hCoV-19/United Arab Emirates/306/2020	EPI_ISL_528694	02/06/2020	Asia / United Arab Emirates / Dubai	Human	Male	29	Live	Original		A	S	United Arab Emirates	Undertermined	No (couldn't determine the patient status as either pre-symptomatic / asymptomatic or symptomatic conclusively)
hCoV-19/United Arab Emirates/310/2020	EPI_ISL_528695	02/06/2020	Asia / United Arab Emirates / Dubai	Human	Male	31	Live	Original		B.1.1	GR	United Arab Emirates	Undertermined	No (couldn't determine the patient status as either pre-symptomatic / asymptomatic or symptomatic conclusively)
hCoV-19/United Arab Emirates/313/2020	EPI_ISL_528696	02/06/2020	Asia / United Arab Emirates / Dubai	Human	Male	38	Live	Original		B.1.1.7	GR	United Arab Emirates	Undertermined	No (couldn't determine the patient status as either pre-symptomatic / asymptomatic or symptomatic conclusively)

hCoV-19/United Arab Emirates/38/2020	EPI_ISL_528698	12/05/2020	Asia / United Arab Emirates / Abu Dhabi	Human		Male	43	Hospitalized	Original			B.1.1	GR	United Arab Emirates	Undertermined	No (couldn't determine the patient status as either pre-symptomatic / asymptomatic or symptomatic conclusively)
hCoV-19/United Arab Emirates/42/2020	EPI_ISL_528699	12/05/2020	Asia / United Arab Emirates / Abu Dhabi	Human		Male	37	Hospitalized	Original			B.1.1	GR	United Arab Emirates	Undertermined	No (couldn't determine the patient status as either pre-symptomatic / asymptomatic or symptomatic conclusively)
hCoV-19/Malaysia/MGI-G873/2020	EPI_ISL_528738	07/04/2020	Asia / Malaysia / Selangor	Human		Male	32	Live	Original			B.1.36	GH	Malaysia	Undertermined	No (couldn't determine the patient status as either pre-symptomatic / asymptomatic or symptomatic conclusively)
hCoV-19/Malaysia/MGI-M32/2020	EPI_ISL_528739	02/04/2020	Asia / Malaysia / Selangor	Human		Male	unknown	Live	Original			B.6	O	Malaysia	Undertermined	No (couldn't determine the patient status as either pre-symptomatic / asymptomatic or symptomatic conclusively)
hCoV-19/Malaysia/MGI-M64/2020	EPI_ISL_528740	02/04/2020	Asia / Malaysia / Selangor	Human		Male	unknown	Live	Original			B.6	O	Malaysia	Undertermined	No (couldn't determine the patient status as either pre-symptomatic / asymptomatic or symptomatic conclusively)
hCoV-19/Malaysia/MGI-M71/2020	EPI_ISL_528741	02/04/2020	Asia / Malaysia / Selangor	Human		Male	unknown	Live	Original			B.6	O	Malaysia	Undertermined	No (couldn't determine the patient status as either pre-symptomatic / asymptomatic or symptomatic conclusively)
hCoV-19/Malaysia/MGI-M76/2020	EPI_ISL_528742	02/04/2020	Asia / Malaysia / Selangor	Human		Male	64	Live	Original			B.6	O	Malaysia	Undertermined	No (couldn't determine the patient status as either pre-symptomatic / asymptomatic or symptomatic conclusively)
hCoV-19/Malaysia/MGI-MAEPS54/2020	EPI_ISL_528743	04/06/2020	Asia / Malaysia / Selangor	Human		Male	unknown	Live	Original			B.6	O	Malaysia	Undertermined	No (couldn't determine the patient status as either pre-symptomatic / asymptomatic or symptomatic conclusively)
hCoV-19/Malaysia/MGI-MAEPS67/2020	EPI_ISL_528744	04/06/2020	Asia / Malaysia / Selangor	Human		Male	unknown	Live	Original			B.6	O	Malaysia	Undertermined	No (couldn't determine the patient status as either pre-symptomatic / asymptomatic or symptomatic conclusively)
hCoV-19/Indonesia/JB-TFRIC19-R24754/2020	EPI_ISL_528745	22/06/2020	Asia / Indonesia / West Java	Human		Male	47	Live	Original	Nasopharyngeal swab and Oropharyngeal swab		B.1.36	GH	Indonesia	Undertermined	No (couldn't determine the patient status as either pre-symptomatic / asymptomatic or symptomatic conclusively)
hCoV-19/Indonesia/JB-TFRIC19-R47609/2020	EPI_ISL_528746	21/07/2020	Asia / Indonesia / West Java	Human		Male	56	Hospitalized	Original	Nasopharyngeal swab and Oropharyngeal swab		B.1.36	GH	Indonesia	Undertermined	No (couldn't determine the patient status as either pre-symptomatic / asymptomatic or symptomatic conclusively)
hCoV-19/Indonesia/JB-TFRIC19-R30535/2020	EPI_ISL_528747	28/06/2020	Asia / Indonesia / West Java	Human		Male	22	Hospitalized	Original	Nasopharyngeal swab and Oropharyngeal swab		B.1.1	GR	Indonesia	Undertermined	No (couldn't determine the patient status as either pre-symptomatic / asymptomatic or symptomatic conclusively)
hCoV-19/Indonesia/JB-TFRIC19-R48562/2020	EPI_ISL_528748	23/07/2020	Asia / Indonesia / West Java	Human		Male	32	Live	Original	Nasopharyngeal swab and Oropharyngeal swab		B.1	G	Indonesia	Undertermined	No (couldn't determine the patient status as either pre-symptomatic / asymptomatic or symptomatic conclusively)
hCoV-19/Indonesia/JB-TFRIC19-R4975/2020	EPI_ISL_528749	07/05/2020	Asia / Indonesia / West Java	Human		Male	61	Hospitalized	Original	Nasopharyngeal swab and Oropharyngeal swab		B.1	GH	Indonesia	Undertermined	No (couldn't determine the patient status as either pre-symptomatic / asymptomatic or symptomatic conclusively)
hCoV-19/Indonesia/JB-TFRIC19-R49542/2020	EPI_ISL_528750	25/07/2020	Asia / Indonesia / West Java	Human		Male	35	Hospitalized	Original	Nasopharyngeal swab and Oropharyngeal swab		B.1.36	GH	Indonesia	Undertermined	No (couldn't determine the patient status as either pre-symptomatic / asymptomatic or symptomatic conclusively)
hCoV-19/Indonesia/JB-TFRIC19-R49344/2020	EPI_ISL_528751	23/07/2020	Asia / Indonesia / West Java	Human		Male	41	Hospitalized	Original	Nasopharyngeal swab and Oropharyngeal swab		B.1.36	GH	Indonesia	Undertermined	No (couldn't determine the patient status as either pre-symptomatic / asymptomatic or symptomatic conclusively)
hCoV-19/Indonesia/JB-TFRIC19-R48875/2020	EPI_ISL_528752	23/07/2020	Asia / Indonesia / West Java	Human		Male	47	Hospitalized	Original	Nasopharyngeal swab and Oropharyngeal swab		B.1.36	GH	Indonesia	Undertermined	No (couldn't determine the patient status as either pre-symptomatic / asymptomatic or symptomatic conclusively)
hCoV-19/Indonesia/JB-TFRIC19-R53817/2020	EPI_ISL_528753	04/08/2020	Asia / Indonesia / West Java	Human		Male	17	Live	Original	Nasopharyngeal swab and Oropharyngeal swab		B.1.36	GH	Indonesia	Undertermined	No (couldn't determine the patient status as either pre-symptomatic / asymptomatic or symptomatic conclusively)
hCoV-19/Indonesia/JB-TFRIC19-R49544/2020	EPI_ISL_528759	25/07/2020	Asia / Indonesia / West Java	Human		Male	37	Hospitalized	Original	Nasopharyngeal swab and Oropharyngeal swab		B.1.36	GH	Indonesia	Undertermined	No (couldn't determine the patient status as either pre-symptomatic / asymptomatic or symptomatic conclusively)
hCoV-19/Indonesia/JI-ITD-136N/2020	EPI_ISL_529961	12/03/2020	Asia / Indonesia / East Java / Surabaya	Human		Male	55	Hospitalized	Original	Nasopharyngeal swab		B	L	Indonesia	Undertermined	No (couldn't determine the patient status as either pre-symptomatic / asymptomatic or symptomatic conclusively)
hCoV-19/Indonesia/JI-ITD-150Sp/2020	EPI_ISL_529962	16/03/2020	Asia / Indonesia / East Java / Surabaya	Human		Female	43	Hospitalized	Original	Sputum		B	L	Indonesia	Undertermined	No (couldn't determine the patient status as either pre-symptomatic / asymptomatic or symptomatic conclusively)
hCoV-19/Indonesia/JI-ITD-17398NT/2020	EPI_ISL_529963	18/07/2020	Asia / Indonesia / East Java / Surabaya	Human		Male	42	Deceased	Original	Nasopharyngeal and Oropharyngeal swab		B.1.36	GH	Indonesia	Symptomatic	Yes
hCoV-19/Czech Republic/NRL-5555/2020	EPI_ISL_539493	23/03/2020	Europe / Czech Republic / South Moravian Region / Znojmo	Human	Import from Austria, Hollabrunn	Male	60	Mild clinical signs without hospitalization	Original	Nasopharyngeal swab		B.1	GH	Czech Republic	Symptomatic	Yes
hCoV-19/Czech Republic/NRL-5635/2020	EPI_ISL_539494	31/03/2020	Europe / Czech Republic / Usti nad Labem / Litomerice / Snedovice	Human		Male	56	Hospitalized/Deceased	Original	Nasopharyngeal swab		B.1	GH	Czech Republic	Symptomatic	Yes
hCoV-19/USA/PR-CDC-S29/2020	EPI_ISL_539495	06/07/2020	North America / USA / Puerto Rico	Human		Male	6 months	Recovered	Original	Nasopharyngeal swab		B.1.26	GH	USA	Undertermined	No (couldn't determine the patient status as either pre-symptomatic / asymptomatic or symptomatic conclusively)
hCoV-19/Andorra/202552/2020	EPI_ISL_539496	13/03/2020	Europe / Andorra	Human		Female	6 months	Hospitalized	Original	Oropharyngeal swab		B.1.5	O	Andorra	Undertermined	No (couldn't determine the patient status as either pre-symptomatic / asymptomatic or symptomatic conclusively)
hCoV-19/Spain/AN-ISCIII-201273/2020	EPI_ISL_539497	28/02/2020	Europe / Spain / Andalucia	Human		Male	59	Hospitalized	Original	Oropharyngeal swab		B	O	Spain	Undertermined	No (couldn't determine the patient status as either pre-symptomatic / asymptomatic or symptomatic conclusively)
hCoV-19/Spain/AN-ISCIII-201618/2020	EPI_ISL_539498	05/03/2020	Europe / Spain / Andalucia	Human		Male	38	Hospitalized	Original	Oropharyngeal swab		B	L	Spain	Undertermined	No (couldn't determine the patient status as either pre-symptomatic / asymptomatic or symptomatic conclusively)
hCoV-19/Spain/AN-ISCIII-202657/2020	EPI_ISL_539499	12/03/2020	Europe / Spain / Andalucia	Human		Male	62	Hospitalized	Original	Oropharyngeal swab		A.2	S	Spain	Undertermined	No (couldn't determine the patient status as either pre-symptomatic / asymptomatic or symptomatic conclusively)
hCoV-19/Czech Republic/NRL-7950/2020	EPI_ISL_546436	22/07/2020	Europe / Czech Republic / ÃšstÃ- nad Labem Region / LitomÄ›Å™ice	Human	Children sport camp held in Prague (Slavia Eden) between 13-17. 7. 2020	Male	12	Mild clinical signs without hospitalization	Original	Nasopharyngeal swab		B.1.1	GR	Czech Republic	Symptomatic	Yes
hCoV-19/Czech Republic/NRL-8631/2020	EPI_ISL_568577	04/09/2020	Europe / Czech Republic / Prague	Human	Outbreak in Ministry of Health	MAle	69	Mild clinical signs without hospitalization	Original	Nasopharyngeal swab		B.1	GH	Czech Republic	Symptomatic	Yes
hCoV-19/Italy/PIE-SLL-MS52/2020	EPI_ISL_569868	01/04/2020	Europe / Italy / Piedmont / Turin	Human		Male	5 months	Hospitalized, Live	Original	Nasopharyngeal swab		B.1	G	Italy	Undertermined	No (couldn't determine the patient status as either pre-symptomatic / asymptomatic or symptomatic conclusively)
hCoV-19/Italy/PIE-SLL-MS53/2020	EPI_ISL_569869	31/03/2020	Europe / Italy / Piedmont / Turin	Human		Male	23	Hospitalized, Live	Original	Nasopharyngeal swab		B.1.1.1	GR	Italy	Undertermined	No (couldn't determine the patient status as either pre-symptomatic / asymptomatic or symptomatic conclusively)
hCoV-19/Germany/BY-ChVir-929/2020	EPI_ISL_406862	28/01/2020	Europe / Germany / Bavaria / Munich	Human		Male	unknown	Hospitalized	Original	Sputum		B	G	Germany	Undertermined	No (couldn't determine the patient status as either pre-symptomatic / asymptomatic or symptomatic conclusively)
hCoV-19/Hangzhou/HZ-1/2020	EPI_ISL_406970	20/01/2020	Asia / China / Zhejiang / Hangzhou	Human		male	32	hospitalized	Original	Sputum	Patient return form Wuhan, China	B	L	China	Undertermined	No (couldn't determine the patient status as either pre-symptomatic / asymptomatic or symptomatic conclusively)
hCoV-19/Belgium/GHB-03021/2020	EPI_ISL_407976	03/02/2020	Europe / Belgium / Leuven	Human		Male	54	Asymptomatic	Original	Oro-pharyngael swab	Patient probably infected in Wuhan. Found positive the day of return to Belgium	A	S	Belgium	Pre-symptomatic / Asymptomatic	Yes
hCoV-19/Brazil/SP-07/2020	EPI_ISL_416028	03/03/2020	South America / Brazil / Sao Paulo / Sao Paulo	Human		Female	22	Released	Original	Naso-pharyngeal swab	Travel Venice and Rome	B.1.1	GR	Brazil	Undertermined	No (couldn't determine the patient status as either pre-symptomatic / asymptomatic or symptomatic conclusively)
hCoV-19/Brazil/SP-08/2020	EPI_ISL_416029	04/03/2020	South America / Brazil / Sao Paulo / Sao Paulo	Human		Female	13	Released	Original	Naso-pharyngeal swab	Contact	B.1.1	GR	Brazil	Undertermined	No (couldn't determine the patient status as either pre-symptomatic / asymptomatic or symptomatic conclusively)
hCoV-19/Brazil/SP-09/2020	EPI_ISL_416031	04/03/2020	South America / Brazil / Sao Paulo / Sao Paulo	Human		Male	37	Released	Original	Naso-pharyngeal swab	Contact	B.1	G	Brazil	Undertermined	No (couldn't determine the patient status as either pre-symptomatic / asymptomatic or symptomatic conclusively)
hCoV-19/Brazil/SP-10/2020	EPI_ISL_416032	04/03/2020	South America / Brazil / Distrito Federal / Brasilia	Human		Female	52	Hospitalized in ICU	Original	Naso-pharyngeal swab	Travel	B.2	O	Brazil	Symptomatic	Yes
hCoV-19/Brazil/SP-11/2020	EPI_ISL_416033	03/03/2020	South America / Brazil / Sao Paulo / Sao Paulo	Human		Male	17	Released	Original	Naso-pharyngeal swab	Travel to UK	B.3	L	Brazil	Undertermined	No (couldn't determine the patient status as either pre-symptomatic / asymptomatic or symptomatic conclusively)
hCoV-19/Brazil/SP-12/2020	EPI_ISL_416034	04/03/2020	South America / Brazil / Sao Paulo / Sao Paulo	Human		Male	35	Released	Original	Naso-pharyngeal swab	Travel to USA	B.1.1	GR	Brazil	Undertermined	No (couldn't determine the patient status as either pre-symptomatic / asymptomatic or symptomatic conclusively)
hCoV-19/Brazil/SP-13/2020	EPI_ISL_416035	05/03/2020	South America / Brazil / Sao Paulo / Sao Paulo	Human		Male	34	Released	Original	Naso-pharyngeal swab	Contact	B.1.1	GR	Brazil	Undertermined	No (couldn't determine the patient status as either pre-symptomatic / asymptomatic or symptomatic conclusively)
hCoV-19/Brazil/SP-14/2020	EPI_ISL_416036	05/03/2020	South America / Brazil / Sao Paulo / Sao Paulo	Human		Male	22	Hospitalized	Original	Naso-pharyngeal swab	Travel to Italy	B.1.1.28	GR	Brazil	Undertermined	No (couldn't determine the patient status as either pre-symptomatic / asymptomatic or symptomatic conclusively)
hCoV-19/France/Lyon_06042/2020	EPI_ISL_417333	04/03/2020	Europe / France / ARA	Human		Male	48	Hospitalized	Original	Nasopharyngeal swab		B.1	G	France	Undertermined	No (couldn't determine the patient status as either pre-symptomatic / asymptomatic or symptomatic conclusively)
hCoV-19/France/Lyon_06056/2020	EPI_ISL_417334	04/03/2020	Europe / France / ARA	Human		Female	50	Hospitalized	Original	Nasopharyngeal swab		B.1	G	France	Undertermined	No (couldn't determine the patient status as either pre-symptomatic / asymptomatic or symptomatic conclusively)
hCoV-19/France/Lyon_06573/2020	EPI_ISL_417335	06/03/2020	Europe / France / ARA	Human		Male	74	Hospitalized	Original	Nasopharyngeal swab		B	L	France	Undertermined	No (couldn't determine the patient status as either pre-symptomatic / asymptomatic or symptomatic conclusively)
hCoV-19/France/Lyon_0668/2020	EPI_ISL_417336	06/03/2020	Europe / France / ARA	Human		Male	79	Hospitalized	Original	Nasopharyngeal swab		B.1	GH	France	Undertermined	No (couldn't determine the patient status as either pre-symptomatic / asymptomatic or symptomatic conclusively)
hCoV-19/France/Lyon_06625/2020	EPI_ISL_417337	07/03/2020	Europe / France / ARA	Human		Male	78	Hospitalized	Original	Nasopharyngeal swab		B.1	G	France	Undertermined	No (couldn't determine the patient status as either pre-symptomatic / asymptomatic or symptomatic conclusively)
hCoV-19/France/Lyon_06820/2020	EPI_ISL_417339	08/03/2020	Europe / France / ARA	Human		Female	86	Hospitalized	Original	Nasopharyngeal swab		B.1	G	France	Undertermined	No (couldn't determine the patient status as either pre-symptomatic / asymptomatic or symptomatic conclusively)
hCoV-19/Belgium/ULG-6732/2020	EPI_ISL_418623	14/03/2020	Europe / Belgium / Liege	Human		Male	Male	hospitalized or to be hospitalized	Original	Nasopharynx swab		B.1.9	GH	Belgium	Undertermined	No (couldn't determine the patient status as either pre-symptomatic / asymptomatic or symptomatic conclusively)
hCoV-19/Belgium/ULG-6972/2020	EPI_ISL_418624	15/03/2020	Europe / Belgium / LiÃ¨ge	Human		Male	77	hospitalized or to be hospitalized	Original	Nasopharynx swab		B.1	G	Belgium	Undertermined	No (couldn't determine the patient status as either pre-symptomatic / asymptomatic or symptomatic conclusively)
hCoV-19/Belgium/ULG-7626/2020	EPI_ISL_418633	17/03/2020	Europe / Belgium / LiÃ¨ge	Human		Male	76	hospitalized or to be hospitalized	Original	Nasopharynx swab		B.3	L	Belgium	Undertermined	No (couldn't determine the patient status as either pre-symptomatic / asymptomatic or symptomatic conclusively)
hCoV-19/Belgium/ULG-8715/2020	EPI_ISL_418643	19/03/2020	Europe / Belgium / LiÃ¨ge	Human		Male	71	hospitalized or to be hospitalized	Original	Nasopharynx swab		B.1.12	GH	Belgium	Undertermined	No (couldn't determine the patient status as either pre-symptomatic / asymptomatic or symptomatic conclusively)
hCoV-19/Belgium/ULG-8808/2020	EPI_ISL_418644	19/03/2020	Europe / Belgium / LiÃ¨ge	Human		Female	64	hospitalized or to be hospitalized	Original	Nasopharynx swab		B.1.12	GH	Belgium	Undertermined	No (couldn't determine the patient status as either pre-symptomatic / asymptomatic or symptomatic conclusively)
hCoV-19/Belgium/ULG-9558/2020	EPI_ISL_418645	21/03/2020	Europe / Belgium / LiÃ¨ge	Human		Male	51	hospitalized or to be hospitalized	Original	Nasopharynx swab		B.1	G	Belgium	Undertermined	No (couldn't determine the patient status as either pre-symptomatic / asymptomatic or symptomatic conclusively)
hCoV-19/Belgium/ULG-9572/2020	EPI_ISL_418646	21/03/2020	Europe / Belgium / LiÃ¨ge	Human		Male	23	hospitalized or to be hospitalized	Original	Nasopharynx swab		B.1	GH	Belgium	Undertermined	No (couldn't determine the patient status as either pre-symptomatic / asymptomatic or symptomatic conclusively)
hCoV-19/Belgium/ULG-9617/2020	EPI_ISL_418647	22/03/2020	Europe / Belgium / LiÃ¨ge	Human		Female	93	hospitalized or to be hospitalized	Original	Nasopharynx swab		B.1	G	Belgium	Undertermined	No (couldn't determine the patient status as either pre-symptomatic / asymptomatic or symptomatic conclusively)
hCoV-19/Belgium/ULG-9618/2020	EPI_ISL_418648	22/03/2020	Europe / Belgium / LiÃ¨ge	Human		Female	70	hospitalized or to be hospitalized	Original	Nasopharynx swab		B.1	G	Belgium	Undertermined	No (couldn't determine the patient status as either pre-symptomatic / asymptomatic or symptomatic conclusively)
hCoV-19/Belgium/ULG-9619/2020	EPI_ISL_418649	22/03/2020	Europe / Belgium / LiÃ¨ge	Human		Female	81	hospitalized or to be hospitalized	Original	Nasopharynx swab		B.1	G	Belgium	Undertermined	No (couldn't determine the patient status as either pre-symptomatic / asymptomatic or symptomatic conclusively)
hCoV-19/Belgium/ULG-9620/2020	EPI_ISL_418650	22/03/2020	Europe / Belgium / LiÃ¨ge	Human		Male	51	hospitalized or to be hospitalized	Original	Nasopharynx swab		B.1.9	GH	Belgium	Undertermined	No (couldn't determine the patient status as either pre-symptomatic / asymptomatic or symptomatic conclusively)
hCoV-19/Belgium/ULG-9634/2020	EPI_ISL_418651	22/03/2020	Europe / Belgium / LiÃ¨ge	Human		Female	57	hospitalized or to be hospitalized	Original	Nasopharynx swab		B.1.1	GR	Belgium	Undertermined	No (couldn't determine the patient status as either pre-symptomatic / asymptomatic or symptomatic conclusively)
hCoV-19/Belgium/ULG-9641/2020	EPI_ISL_418652	22/03/2020	Europe / Belgium / LiÃ¨ge	Human		Female	63	hospitalized or to be hospitalized	Original	Nasopharynx swab		B.1.12	GH	Belgium	Undertermined	No (couldn't determine the patient status as either pre-symptomatic / asymptomatic or symptomatic conclusively)
hCoV-19/Belgium/ULG-9719/2020	EPI_ISL_418658	22/03/2020	Europe / Belgium / LiÃ¨ge	Human		Female	34	hospitalized or to be hospitalized	Original	Nasopharynx swab		B.1	G	Belgium	Undertermined	No (couldn't determine the patient status as either pre-symptomatic / asymptomatic or symptomatic conclusively)
hCoV-19/Belgium/ULG-9725/2020	EPI_ISL_418659	22/03/2020	Europe / Belgium / LiÃ¨ge	Human		Male	46	hospitalized or to be hospitalized	Original	Nasopharynx swab		B.1	G	Belgium	Undertermined	No (couldn't determine the patient status as either pre-symptomatic / asymptomatic or symptomatic conclusively)
hCoV-19/Belgium/ULG-9732/2020	EPI_ISL_418660	22/03/2020	Europe / Belgium / LiÃ¨ge	Human		Male	51	hospitalized or to be hospitalized	Original	Bronchoalveolar lavage		B.1.9	GH	Belgium	Undertermined	No (couldn't determine the patient status as either pre-symptomatic / asymptomatic or symptomatic conclusively)
hCoV-19/Belgium/ULG-9738/2020	EPI_ISL_418662	22/03/2020	Europe / Belgium / LiÃ¨ge	Human		Male	57	hospitalized or to be hospitalized	Original	Nasopharynx swab		B.1	G	Belgium	Undertermined	No (couldn't determine the patient status as either pre-symptomatic / asymptomatic or symptomatic conclusively)
hCoV-19/Belgium/ULG-9741/2020	EPI_ISL_418664	22/03/2020	Europe / Belgium / LiÃ¨ge	Human		Female	83	hospitalized or to be hospitalized	Original	Nasopharynx swab		B.1.1	GR	Belgium	Undertermined	No (couldn't determine the patient status as either pre-symptomatic / asymptomatic or symptomatic conclusively)
hCoV-19/Belgium/ULG-9742/2020	EPI_ISL_418665	22/03/2020	Europe / Belgium / LiÃ¨ge	Human		Male	77	hospitalized or to be hospitalized	Original	Nasopharynx swab		B.1.1	GR	Belgium	Undertermined	No (couldn't determine the patient status as either pre-symptomatic / asymptomatic or symptomatic conclusively)
hCoV-19/Belgium/ULG-9751/2020	EPI_ISL_418666	22/03/2020	Europe / Belgium / LiÃ¨ge	Human		Female	73	hospitalized or to be hospitalized	Original	Nasopharynx swab		B.1	GH	Belgium	Undertermined	No (couldn't determine the patient status as either pre-symptomatic / asymptomatic or symptomatic conclusively)
hCoV-19/Belgium/CS-031052/2020	EPI_ISL_418800	10/03/2020	Europe / Belgium / Gierle	Human		Female	41	Hospitalized	Original	Oro-pharyngeal swab		B.3	L	Belgium	Undertermined	No (couldn't determine the patient status as either pre-symptomatic / asymptomatic or symptomatic conclusively)
hCoV-19/Australia/QLDID924/2020	EPI_ISL_418802	18/03/2020	Oceania / Australia / Queensland / Gold Coast	Human		Female	24	unknow	Original	Swab		B.1.1	GR	Australia	Undertermined	No (couldn't determine the patient status as either pre-symptomatic / asymptomatic or symptomatic conclusively)
hCoV-19/Hong Kong/HKPU36-0702/2020	EPI_ISL_418815	09/02/2020	Asia / Hong Kong	Human		Male	23	Hospitalized; Stable	Original			B.2.8	O	Hong Kong	Undertermined	No (couldn't determine the patient status as either pre-symptomatic / asymptomatic or symptomatic conclusively)
hCoV-19/Belgium/ULG-7821/2020	EPI_ISL_424628	17/03/2020	Europe / Belgium / LiÃ¨ge	Human		Female	72	Hospitalized	Original			B.1.12	GH	Belgium	Undertermined	No (couldn't determine the patient status as either pre-symptomatic / asymptomatic or symptomatic conclusively)
hCoV-19/Belgium/ULG-10066/2020	EPI_ISL_424629	03/04/2020	Europe / Belgium / LiÃ¨ge	Human		unknown	unknown	Hospitalized	Original			B.1.1	GR	Belgium	Undertermined	No (couldn't determine the patient status as either pre-symptomatic / asymptomatic or symptomatic conclusively)
hCoV-19/Belgium/ULG-10067/2020	EPI_ISL_424630	03/04/2020	Europe / Belgium / LiÃ¨ge	Human		unknown	unknown	Hospitalized	Original			B.1.1	GR	Belgium	Undertermined	No (couldn't determine the patient status as either pre-symptomatic / asymptomatic or symptomatic conclusively)
hCoV-19/Belgium/ULG-10072/2020	EPI_ISL_424631	03/04/2020	Europe / Belgium / LiÃ¨ge	Human		unknown	unknown	Hospitalized	Original			B.1.1	GR	Belgium	Undertermined	No (couldn't determine the patient status as either pre-symptomatic / asymptomatic or symptomatic conclusively)
hCoV-19/Belgium/ULG-10069/2020	EPI_ISL_424633	03/04/2020	Europe / Belgium / LiÃ¨ge	Human		unknown	unknown	Hospitalized	Original			B.1	G	Belgium	Undertermined	No (couldn't determine the patient status as either pre-symptomatic / asymptomatic or symptomatic conclusively)
hCoV-19/Belgium/ULG-10065/2020	EPI_ISL_424635	03/04/2020	Europe / Belgium / LiÃ¨ge	Human		unknown	unknown	Hospitalized	Original			B.1	GH	Belgium	Undertermined	No (couldn't determine the patient status as either pre-symptomatic / asymptomatic or symptomatic conclusively)
hCoV-19/Belgium/ULG-10068/2020	EPI_ISL_424636	03/04/2020	Europe / Belgium / LiÃ¨ge	Human		unknown	unknown	Hospitalized	Original			B.1.1	GR	Belgium	Undertermined	No (couldn't determine the patient status as either pre-symptomatic / asymptomatic or symptomatic conclusively)
hCoV-19/Belgium/ULG-10078/2020	EPI_ISL_424637	03/04/2020	Europe / Belgium / LiÃ¨ge	Human		unknown	unknown	Hospitalized	Original			B.1	GH	Belgium	Undertermined	No (couldn't determine the patient status as either pre-symptomatic / asymptomatic or symptomatic conclusively)
hCoV-19/Belgium/ULG-10084/2020	EPI_ISL_424638	03/04/2020	Europe / Belgium / LiÃ¨ge	Human		unknown	unknown	Hospitalized	Original			B.1	G	Belgium	Undertermined	No (couldn't determine the patient status as either pre-symptomatic / asymptomatic or symptomatic conclusively)
hCoV-19/Belgium/ULG-10079/2020	EPI_ISL_424639	03/04/2020	Europe / Belgium / LiÃ¨ge	Human		unknown	unknown	Hospitalized	Original			B.1.12	GH	Belgium	Undertermined	No (couldn't determine the patient status as either pre-symptomatic / asymptomatic or symptomatic conclusively)
hCoV-19/Belgium/ULG-10081/2020	EPI_ISL_424640	03/04/2020	Europe / Belgium / LiÃ¨ge	Human		unknown	unknown	Hospitalized	Original			B.1	G	Belgium	Undertermined	No (couldn't determine the patient status as either pre-symptomatic / asymptomatic or symptomatic conclusively)
hCoV-19/Belgium/ULG-10080/2020	EPI_ISL_424641	03/04/2020	Europe / Belgium / LiÃ¨ge	Human		unknown	unknown	Hospitalized	Original			B.2.2	V	Belgium	Undertermined	No (couldn't determine the patient status as either pre-symptomatic / asymptomatic or symptomatic conclusively)
hCoV-19/Belgium/ULG-10077/2020	EPI_ISL_424642	04/04/2020	Europe / Belgium / LiÃ¨ge	Human		unknown	unknown	Hospitalized	Original			B.1	G	Belgium	Undertermined	No (couldn't determine the patient status as either pre-symptomatic / asymptomatic or symptomatic conclusively)
hCoV-19/Belgium/ULG-10089/2020	EPI_ISL_424643	04/04/2020	Europe / Belgium / LiÃ¨ge	Human		unknown	unknown	Hospitalized	Original			B.3	L	Belgium	Undertermined	No (couldn't determine the patient status as either pre-symptomatic / asymptomatic or symptomatic conclusively)
hCoV-19/Belgium/ULG-10086/2020	EPI_ISL_424644	04/04/2020	Europe / Belgium / LiÃ¨ge	Human		unknown	unknown	Hospitalized	Original			B.1	G	Belgium	Undertermined	No (couldn't determine the patient status as either pre-symptomatic / asymptomatic or symptomatic conclusively)
hCoV-19/Belgium/ULG-10092/2020	EPI_ISL_424647	04/04/2020	Europe / Belgium / LiÃ¨ge	Human		unknown	unknown	Hospitalized	Original			B.1	G	Belgium	Undertermined	No (couldn't determine the patient status as either pre-symptomatic / asymptomatic or symptomatic conclusively)
hCoV-19/Belgium/ULG-10093/2020	EPI_ISL_424648	04/04/2020	Europe / Belgium / LiÃ¨ge	Human		unknown	unknown	Hospitalized	Original			B.1.1	GR	Belgium	Undertermined	No (couldn't determine the patient status as either pre-symptomatic / asymptomatic or symptomatic conclusively)
hCoV-19/Belgium/ULG-10082/2020	EPI_ISL_424649	04/04/2020	Europe / Belgium / LiÃ¨ge	Human		unknown	unknown	Hospitalized	Original			B.1.1	GR	Belgium	Undertermined	No (couldn't determine the patient status as either pre-symptomatic / asymptomatic or symptomatic conclusively)
hCoV-19/Belgium/ULG-10094/2020	EPI_ISL_424650	04/04/2020	Europe / Belgium / LiÃ¨ge	Human		unknown	unknown	Hospitalized	Original			B.1	G	Belgium	Undertermined	No (couldn't determine the patient status as either pre-symptomatic / asymptomatic or symptomatic conclusively)
hCoV-19/Belgium/ULG-10055/2020	EPI_ISL_424652	05/04/2020	Europe / Belgium / LiÃ¨ge	Human		unknown	unknown	Hospitalized	Original			B.1	G	Belgium	Undertermined	No (couldn't determine the patient status as either pre-symptomatic / asymptomatic or symptomatic conclusively)
hCoV-19/Belgium/ULG-10058/2020	EPI_ISL_424653	05/04/2020	Europe / Belgium / LiÃ¨ge	Human		unknown	unknown	Hospitalized	Original			B.1	G	Belgium	Undertermined	No (couldn't determine the patient status as either pre-symptomatic / asymptomatic or symptomatic conclusively)
hCoV-19/Belgium/ULG-10057/2020	EPI_ISL_424654	05/04/2020	Europe / Belgium / LiÃ¨ge	Human		unknown	unknown	Hospitalized	Original			B.1.1	GR	Belgium	Undertermined	No (couldn't determine the patient status as either pre-symptomatic / asymptomatic or symptomatic conclusively)
hCoV-19/Belgium/ULG-10056/2020	EPI_ISL_424655	05/04/2020	Europe / Belgium / LiÃ¨ge	Human		unknown	unknown	Hospitalized	Original			B.1	G	Belgium	Undertermined	No (couldn't determine the patient status as either pre-symptomatic / asymptomatic or symptomatic conclusively)
hCoV-19/Belgium/ULG-10054/2020	EPI_ISL_424656	05/04/2020	Europe / Belgium / LiÃ¨ge	Human		unknown	unknown	Hospitalized	Original			B.1.9	GH	Belgium	Undertermined	No (couldn't determine the patient status as either pre-symptomatic / asymptomatic or symptomatic conclusively)
hCoV-19/Belgium/ULG-10052/2020	EPI_ISL_424657	05/04/2020	Europe / Belgium / LiÃ¨ge	Human		unknown	unknown	Hospitalized	Original			B.3	L	Belgium	Undertermined	No (couldn't determine the patient status as either pre-symptomatic / asymptomatic or symptomatic conclusively)
hCoV-19/Belgium/ULG-10049/2020	EPI_ISL_424658	05/04/2020	Europe / Belgium / LiÃ¨ge	Human		unknown	unknown	Hospitalized	Original			B.1	G	Belgium	Undertermined	No (couldn't determine the patient status as either pre-symptomatic / asymptomatic or symptomatic conclusively)
hCoV-19/Belgium/ULG-10050/2020	EPI_ISL_424659	05/04/2020	Europe / Belgium / LiÃ¨ge	Human		unknown	unknown	Hospitalized	Original			B.1.1	GR	Belgium	Undertermined	No (couldn't determine the patient status as either pre-symptomatic / asymptomatic or symptomatic conclusively)
hCoV-19/Belgium/ULG-10051/2020	EPI_ISL_424660	05/04/2020	Europe / Belgium / LiÃ¨ge	Human		unknown	unknown	Hospitalized	Original			B.1	G	Belgium	Undertermined	No (couldn't determine the patient status as either pre-symptomatic / asymptomatic or symptomatic conclusively)
hCoV-19/Belgium/ULG-10053/2020	EPI_ISL_424661	05/04/2020	Europe / Belgium / LiÃ¨ge	Human		unknown	unknown	Hospitalized	Original			B.1.1	GR	Belgium	Undertermined	No (couldn't determine the patient status as either pre-symptomatic / asymptomatic or symptomatic conclusively)
hCoV-19/Belgium/ULG-10060/2020	EPI_ISL_424663	05/04/2020	Europe / Belgium / LiÃ¨ge	Human		unknown	unknown	Hospitalized	Original			B.1	G	Belgium	Undertermined	No (couldn't determine the patient status as either pre-symptomatic / asymptomatic or symptomatic conclusively)
hCoV-19/Belgium/ULG-10062/2020	EPI_ISL_424664	05/04/2020	Europe / Belgium / LiÃ¨ge	Human		unknown	unknown	Hospitalized	Original			B.1.6	G	Belgium	Undertermined	No (couldn't determine the patient status as either pre-symptomatic / asymptomatic or symptomatic conclusively)
hCoV-19/Taiwan/TSGH-12/2020	EPI_ISL_428229	18/03/2020	Asia / Taiwan / Taipei	Human		Female	60	Released	Original			B.1	G	Taiwan	Undertermined	No (couldn't determine the patient status as either pre-symptomatic / asymptomatic or symptomatic conclusively)
hCoV-19/Turkey/GLAB-CoV012/2020	EPI_ISL_428346	17/04/2020	Europe / Turkey / Istanbul	Human		Male	49	Outpatient	Original	Oro-pharyngeal swab		B.1.9	GH	Turkey	Undertermined	No (couldn't determine the patient status as either pre-symptomatic / asymptomatic or symptomatic conclusively)
hCoV-19/France/IDF3384/2020	EPI_ISL_428347	23/03/2020	Europe / France / Ile De France / Longjumeau	Human		Female	16	hospitalized	Original	Oro-pharyngeal swab		B.1	GH	France	Undertermined	No (couldn't determine the patient status as either pre-symptomatic / asymptomatic or symptomatic conclusively)
hCoV-19/France/GE3372/2020	EPI_ISL_428348	20/03/2020	Europe / France / Grand-est / Gondrecourt-le-chateau	Human		Female	72	Physician network	Original	Oro-pharyngeal swab		B.1	G	France	Undertermined	No (couldn't determine the patient status as either pre-symptomatic / asymptomatic or symptomatic conclusively)
hCoV-19/France/IDF3386/2020	EPI_ISL_428349	23/03/2020	Europe / France / Ile De France / Longjumeau	Human		Female	72	hospitalized	Original	Oro-pharyngeal swab		B.1	GH	France	Undertermined	No (couldn't determine the patient status as either pre-symptomatic / asymptomatic or symptomatic conclusively)
hCoV-19/France/HF3419/2020	EPI_ISL_428350	24/03/2020	Europe / France / Hauts De France / Chateau-thierry	Human		Female	94	hospitalized	Original	Oro-pharyngeal swab		B.1	GH	France	Undertermined	No (couldn't determine the patient status as either pre-symptomatic / asymptomatic or symptomatic conclusively)
hCoV-19/France/IDF3509/2020	EPI_ISL_428351	24/03/2020	Europe / France / Ile De France / Orsay	Human		Male	55	hospitalized	Original	Oro-pharyngeal swab		B.1	GH	France	Undertermined	No (couldn't determine the patient status as either pre-symptomatic / asymptomatic or symptomatic conclusively)
hCoV-19/France/IDF3518/2020	EPI_ISL_428352	23/03/2020	Europe / France / Ile De France / Orsay	Human		Male	86	hospitalized	Original	Oro-pharyngeal swab		B.1	G	France	Undertermined	No (couldn't determine the patient status as either pre-symptomatic / asymptomatic or symptomatic conclusively)
hCoV-19/France/HF3534/2020	EPI_ISL_428353	24/03/2020	Europe / France / Hauts De France / Compiegne	Human		Female	unknown	hospitalized	Original	Oro-pharyngeal swab		B.1	GH	France	Undertermined	No (couldn't determine the patient status as either pre-symptomatic / asymptomatic or symptomatic conclusively)
hCoV-19/France/IDF3577/2020	EPI_ISL_428354	25/03/2020	Europe / France / Ile De France / Longjumeau	Human		Male	71	hospitalized	Original	sputum		B.1.1	GR	France	Undertermined	No (couldn't determine the patient status as either pre-symptomatic / asymptomatic or symptomatic conclusively)
hCoV-19/France/IDF2251/2020	EPI_ISL_428355	11/03/2020	Europe / France / Ile De France / Garches	Human		Male	71	hospitalized	Original	lung biopsy		B.1	GH	France	Undertermined	No (couldn't determine the patient status as either pre-symptomatic / asymptomatic or symptomatic conclusively)
hCoV-19/France/IDF2252/2020	EPI_ISL_428356	11/03/2020	Europe / France / Ile De France / Garches	Human		Male	71	hospitalized	Original	lung biopsy		B.1	GH	France	Undertermined	No (couldn't determine the patient status as either pre-symptomatic / asymptomatic or symptomatic conclusively)
hCoV-19/France/IDF2253/2020	EPI_ISL_428357	11/03/2020	Europe / France / Ile De France / Garches	Human		Male	71	hospitalized	Original	pulmonary swab		B.1	GH	France	Undertermined	No (couldn't determine the patient status as either pre-symptomatic / asymptomatic or symptomatic conclusively)
hCoV-19/France/HF3598/2020	EPI_ISL_428358	24/03/2020	Europe / France / Hauts De France / Chateau-thierry	Human		Female	92	hospitalized	Original	Oro-pharyngeal swab		B.1	GH	France	Undertermined	No (couldn't determine the patient status as either pre-symptomatic / asymptomatic or symptomatic conclusively)
hCoV-19/France/HF3677/2020	EPI_ISL_428359	23/03/2020	Europe / France / Hauts De France / Compiegne	Human		Male	66	hospitalized	Original	Oro-pharyngeal swab		B.1	G	France	Undertermined	No (couldn't determine the patient status as either pre-symptomatic / asymptomatic or symptomatic conclusively)
hCoV-19/France/HF3678/2020	EPI_ISL_428360	25/03/2020	Europe / France / Hauts De France / Compiegne	Human		Male	66	hospitalized	Original	tracheal		B.1	GH	France	Undertermined	No (couldn't determine the patient status as either pre-symptomatic / asymptomatic or symptomatic conclusively)
hCoV-19/France/IDF3703/2020	EPI_ISL_428361	25/03/2020	Europe / France / Ile De France / Longjumeau	Human		Female	66	hospitalized	Original	Oro-pharyngeal swab		B.1	G	France	Undertermined	No (couldn't determine the patient status as either pre-symptomatic / asymptomatic or symptomatic conclusively)
hCoV-19/France/IDF3709/2020	EPI_ISL_428362	25/03/2020	Europe / France / Ile De France / Longjumeau	Human		Female	40	hospitalized	Original	Oro-pharyngeal swab		B.1	GH	France	Undertermined	No (couldn't determine the patient status as either pre-symptomatic / asymptomatic or symptomatic conclusively)
hCoV-19/France/IDF3745/2020	EPI_ISL_428363	26/03/2020	Europe / France / Ile De France / Orsay	Human		Male	49	hospitalized	Original	Oro-pharyngeal swab		B.1	GH	France	Undertermined	No (couldn't determine the patient status as either pre-symptomatic / asymptomatic or symptomatic conclusively)
hCoV-19/France/IDF3831/2020	EPI_ISL_428364	23/03/2020	Europe / France / Ile De France / Moussy Le Neuf	Human		Female	59	Physician network	Original	Oro-pharyngeal swab		B.1	GH	France	Undertermined	No (couldn't determine the patient status as either pre-symptomatic / asymptomatic or symptomatic conclusively)
hCoV-19/France/IDF3930/2020	EPI_ISL_428365	26/03/2020	Europe / France / Ile De France / Longjumeau	Human		Male	29	hospitalized	Original	Oro-pharyngeal swab		B.1	GH	France	Undertermined	No (couldn't determine the patient status as either pre-symptomatic / asymptomatic or symptomatic conclusively)
hCoV-19/France/HF4220/2020	EPI_ISL_428366	30/03/2020	Europe / France / Hauts De France / Chateau-thierry	Human		Female	28	hospitalized	Original	Oro-pharyngeal swab		B.1	GH	France	Undertermined	No (couldn't determine the patient status as either pre-symptomatic / asymptomatic or symptomatic conclusively)
hCoV-19/France/N4427/2020	EPI_ISL_428367	23/03/2020	Europe / France / Normandie / Carentan	Human		Female	21	Physician network	Original	Oro-pharyngeal swab		B.1	GH	France	Undertermined	No (couldn't determine the patient status as either pre-symptomatic / asymptomatic or symptomatic conclusively)

hCoV-19/Turkey/GLAB-CoV013/2020	EPI_ISL_428368	16/04/2020	Europe / Turkey / Istanbul	Human		Female	49	Hospitalized	Original	Oro-pharyngeal swab	B.1.1	GR	Turkey	Undertermined	No (couldn't determine the patient status as either pre-symptomatic / asymptomatic or symptomatic conclusively)
hCoV-19/Italy/ABR-IZSGC-TE6644/2020	EPI_ISL_435145	24/03/2020	Europe / Italy / Abruzzo	Human		Male	67	Live	Original		B.1.1	GR	Italy	Undertermined	No (couldn't determine the patient status as either pre-symptomatic / asymptomatic or symptomatic conclusively)
hCoV-19/Italy/ABR-IZSGC-TE12337/2020	EPI_ISL_435146	07/04/2020	Europe / Italy / Abruzzo	Human		Male	36	Live	Original		B.1	G	Italy	Undertermined	No (couldn't determine the patient status as either pre-symptomatic / asymptomatic or symptomatic conclusively)
hCoV-19/Italy/ABR-IZSGC-TE12759/2020	EPI_ISL_435147	08/04/2020	Europe / Italy / Abruzzo	Human		Female	43	Live	Original		B.1	G	Italy	Undertermined	No (couldn't determine the patient status as either pre-symptomatic / asymptomatic or symptomatic conclusively)
hCoV-19/Italy/ABR-IZSGC-TE13491/2020	EPI_ISL_435148	08/04/2020	Europe / Italy / Abruzzo	Human		Female	49	Live	Original		B.1.1	GR	Italy	Undertermined	No (couldn't determine the patient status as either pre-symptomatic / asymptomatic or symptomatic conclusively)
hCoV-19/Italy/ABR-IZSGC-TE13457/2020	EPI_ISL_435149	08/04/2020	Europe / Italy / Abruzzo	Human		Female	49	Live	Original		B.1.1	GR	Italy	Undertermined	No (couldn't determine the patient status as either pre-symptomatic / asymptomatic or symptomatic conclusively)
hCoV-19/Vietnam/VNHN_022/2020	EPI_ISL_435303	07/03/2020	Asia / Vietnam / Hanoi	Human		Male	27	Released	Original	Oro-pharyngeal swab	B.1.1	GR	Vietnam	Undertermined	No (couldn't determine the patient status as either pre-symptomatic / asymptomatic or symptomatic conclusively)
hCoV-19/Vietnam/VNHN_026/2020	EPI_ISL_435305	07/03/2020	Asia / Vietnam / Hanoi	Human		Female	64	Hospitalized	Original	Oro-pharyngeal swab	B.1.1	GR	Vietnam	Undertermined	No (couldn't determine the patient status as either pre-symptomatic / asymptomatic or symptomatic conclusively)
hCoV-19/Hungary/SRC-00126/2020	EPI_ISL_435405	22/03/2020	Europe / Hungary / Baranya	Human		Female	42	Hospitalized	Original	Oro-pharyngeal swab	B.1.1	GR	Hungary	Undertermined	No (couldn't determine the patient status as either pre-symptomatic / asymptomatic or symptomatic conclusively)
hCoV-19/Hungary/SRC-00183/2020	EPI_ISL_435407	22/03/2020	Europe / Hungary / Baranya	Human		Male	87	Hospitalized	Original	Oro-pharyngeal swab	B.1	GH	Hungary	Undertermined	No (couldn't determine the patient status as either pre-symptomatic / asymptomatic or symptomatic conclusively)
hCoV-19/Hungary/SRC-00186/2020	EPI_ISL_435408	22/03/2020	Europe / Hungary / Baranya	Human		Male	54	Hospitalized	Original	Oro-pharyngeal swab	B.1.1	GR	Hungary	Undertermined	No (couldn't determine the patient status as either pre-symptomatic / asymptomatic or symptomatic conclusively)
hCoV-19/Hungary/SRC-00419/2020	EPI_ISL_435410	26/03/2020	Europe / Hungary / Baranya	Human		Female	41	Hospitalized	Original	Oro-pharyngeal swab	B.1	GH	Hungary	Undertermined	No (couldn't determine the patient status as either pre-symptomatic / asymptomatic or symptomatic conclusively)
hCoV-19/Hungary/SRC-00792/2020	EPI_ISL_435412	30/03/2020	Europe / Hungary / Baranya	Human		Female	60	Hospitalized	Original	Oro-pharyngeal swab	B.1	GH	Hungary	Undertermined	No (couldn't determine the patient status as either pre-symptomatic / asymptomatic or symptomatic conclusively)
hCoV-19/Hungary/SRC-00827/2020	EPI_ISL_435414	30/03/2020	Europe / Hungary / Baranya	Human		Male	71	Hospitalized	Original	Oro-pharyngeal swab	B.1	GH	Hungary	Undertermined	No (couldn't determine the patient status as either pre-symptomatic / asymptomatic or symptomatic conclusively)
hCoV-19/Hungary/SRC-00620/2020	EPI_ISL_435416	27/03/2020	Europe / Hungary / Baranya	Human		Male	47	Hospitalized	Original	Oro-pharyngeal swab	B.3	L	Hungary	Undertermined	No (couldn't determine the patient status as either pre-symptomatic / asymptomatic or symptomatic conclusively)
hCoV-19/Hungary/SRC-00055/2020	EPI_ISL_435419	20/03/2020	Europe / Hungary / Baranya	Human		Male	68	Hospitalized	Original	Oro-pharyngeal swab	B.1.1	GR	Hungary	Undertermined	No (couldn't determine the patient status as either pre-symptomatic / asymptomatic or symptomatic conclusively)
hCoV-19/Hungary/MBL-464/2020	EPI_ISL_435422	27/03/2020	Europe / Hungary / Balassagyarmat	Human	Hospital	Male	47	Hospitalized	Original	Oro-pharyngeal swab	B.1.5	G	Hungary	Undertermined	No (couldn't determine the patient status as either pre-symptomatic / asymptomatic or symptomatic conclusively)
hCoV-19/Hungary/MBL-465/2020	EPI_ISL_435423	27/03/2020	Europe / Hungary / Balassagyarmat	Human	Hospital	Male	53	Hospitalized	Original	Oro-pharyngeal swab	B.1.5	G	Hungary	Undertermined	No (couldn't determine the patient status as either pre-symptomatic / asymptomatic or symptomatic conclusively)
hCoV-19/Hungary/MBL-469/2020	EPI_ISL_435424	27/03/2020	Europe / Hungary / Balassagyarmat	Human	Hospital	Female	36	Hospitalized	Original	Oro-pharyngeal swab	B.1.5	G	Hungary	Undertermined	No (couldn't determine the patient status as either pre-symptomatic / asymptomatic or symptomatic conclusively)
hCoV-19/Hungary/SRC-1788lc/2020	EPI_ISL_435425	19/03/2020	Europe / Hungary / Szeged	Human		Male	48	Hospitalized	Original	Oro-pharyngeal swab	B.1.5	G	Hungary	Undertermined	No (couldn't determine the patient status as either pre-symptomatic / asymptomatic or symptomatic conclusively)
hCoV-19/Hungary/SRC-03670w/2020	EPI_ISL_435430	29/03/2020	Europe / Hungary / Kecskemet	Human		Male	43	Hospitalized	Original	Oro-pharyngeal swab	B.1.1	GR	Hungary	Undertermined	No (couldn't determine the patient status as either pre-symptomatic / asymptomatic or symptomatic conclusively)
hCoV-19/Hungary/SRC-00572w/2020	EPI_ISL_435431	25/03/2020	Europe / Hungary / Kecskemet	Human		Female	61	Hospitalized	Original	Oro-pharyngeal swab	B.1	G	Hungary	Undertermined	No (couldn't determine the patient status as either pre-symptomatic / asymptomatic or symptomatic conclusively)
hCoV-19/South Africa/KRISP-04/2020	EPI_ISL_436684	31/03/2020	Africa / South Africa / KwaZulu-Natal	Human		Female	26	Alive	Original	Oro-pharyngeal swab	B.1.1	GR	South Africa	Undertermined	No (couldn't determine the patient status as either pre-symptomatic / asymptomatic or symptomatic conclusively)
hCoV-19/South Africa/KRISP-010/2020	EPI_ISL_436685	01/04/2020	Africa / South Africa / KwaZulu-Natal	Human		Female	52	Alive	Original	Oro-pharyngeal swab	B.1.106	G	South Africa	Undertermined	No (couldn't determine the patient status as either pre-symptomatic / asymptomatic or symptomatic conclusively)
hCoV-19/South Africa/KRISP-045/2020	EPI_ISL_436686	27/03/2020	Africa / South Africa / KwaZulu-Natal	Human		Female	39	Alive	Original	Oro-pharyngeal swab	B.1.1.1	GR	South Africa	Undertermined	No (couldn't determine the patient status as either pre-symptomatic / asymptomatic or symptomatic conclusively)
hCoV-19/South Africa/KRISP-051/2020	EPI_ISL_436687	28/03/2020	Africa / South Africa / KwaZulu-Natal	Human		Female	55	Alive	Original		B.1	G	South Africa	Undertermined	No (couldn't determine the patient status as either pre-symptomatic / asymptomatic or symptomatic conclusively)
hCoV-19/Russia/Moscow-GCBL1/2020	EPI_ISL_436715	14/04/2020	Europe / Russia / Moscow	Human		Male	41	Released	Original	Oro-pharyngeal swab	B.1.1	GR	Russia	Undertermined	No (couldn't determine the patient status as either pre-symptomatic / asymptomatic or symptomatic conclusively)
hCoV-19/Russia/Moscow-GCBL2/2020	EPI_ISL_436716	14/04/2020	Europe / Russia / Moscow	Human		Male	33	Released	Original	Oro-pharyngeal swab	B.1.1	GR	Russia	Undertermined	No (couldn't determine the patient status as either pre-symptomatic / asymptomatic or symptomatic conclusively)
hCoV-19/Russia/Moscow-GCBL3/2020	EPI_ISL_436717	14/04/2020	Europe / Russia / Moscow	Human		Male	44	Released	Original	Oro-pharyngeal swab	B.1.1	GR	Russia	Undertermined	No (couldn't determine the patient status as either pre-symptomatic / asymptomatic or symptomatic conclusively)
hCoV-19/Italy/ABR-IZSGC-TE5473/2020	EPI_ISL_436718	19/03/2020	Europe / Italy / Abruzzo	Human		Female	40	Live	Original		B.1.1	GR	Italy	Undertermined	No (couldn't determine the patient status as either pre-symptomatic / asymptomatic or symptomatic conclusively)
hCoV-19/Italy/ABR-IZSGC-TE5541/2020	EPI_ISL_436719	20/03/2020	Europe / Italy / Abruzzo	Human		Male	75	Live	Original		B.1.1	GR	Italy	Undertermined	No (couldn't determine the patient status as either pre-symptomatic / asymptomatic or symptomatic conclusively)
hCoV-19/Italy/ABR-IZSGC-TE5543/2020	EPI_ISL_436720	20/03/2020	Europe / Italy / Abruzzo	Human		Male	62	Live	Original		B.1.1	GR	Italy	Undertermined	No (couldn't determine the patient status as either pre-symptomatic / asymptomatic or symptomatic conclusively)
hCoV-19/Italy/ABR-IZSGC-TE5545/2020	EPI_ISL_436721	20/03/2020	Europe / Italy / Abruzzo	Human		Female	79	Live	Original		B.1.1	GR	Italy	Undertermined	No (couldn't determine the patient status as either pre-symptomatic / asymptomatic or symptomatic conclusively)
hCoV-19/Italy/ABR-IZSGC-TE5547/2020	EPI_ISL_436722	20/03/2020	Europe / Italy / Abruzzo	Human		Male	81	Live	Original		B.1.1	GR	Italy	Undertermined	No (couldn't determine the patient status as either pre-symptomatic / asymptomatic or symptomatic conclusively)
hCoV-19/Italy/ABR-IZSGC-TE5687/2020	EPI_ISL_436723	20/03/2020	Europe / Italy / Abruzzo	Human		Male	65	Live	Original		B.1.1	G	Italy	Undertermined	No (couldn't determine the patient status as either pre-symptomatic / asymptomatic or symptomatic conclusively)
hCoV-19/Italy/ABR-IZSGC-TE5827/2020	EPI_ISL_436724	21/03/2020	Europe / Italy / Abruzzo	Human		Male	70	Hospitalized	Original		B.1.1	GR	Italy	Undertermined	No (couldn't determine the patient status as either pre-symptomatic / asymptomatic or symptomatic conclusively)
hCoV-19/Italy/ABR-IZSGC-TE27020/2020	EPI_ISL_436725	27/04/2020	Europe / Italy / Abruzzo	Human		Female	60	Live	Original		B.1.1	GR	Italy	Undertermined	No (couldn't determine the patient status as either pre-symptomatic / asymptomatic or symptomatic conclusively)
hCoV-19/Italy/ABR-IZSGC-TE26533/2020	EPI_ISL_436726	27/04/2020	Europe / Italy / Abruzzo	Human		Female	84	Live	Original		B.1	G	Italy	Undertermined	No (couldn't determine the patient status as either pre-symptomatic / asymptomatic or symptomatic conclusively)
hCoV-19/Italy/ABR-IZSGC-TE26539/2020	EPI_ISL_436727	27/04/2020	Europe / Italy / Abruzzo	Human		Female	56	Live	Original		B.1	G	Italy	Undertermined	No (couldn't determine the patient status as either pre-symptomatic / asymptomatic or symptomatic conclusively)
hCoV-19/Italy/ABR-IZSGC-TE26540/2020	EPI_ISL_436728	27/04/2020	Europe / Italy / Abruzzo	Human		Female	26	Live	Original		B.1	G	Italy	Undertermined	No (couldn't determine the patient status as either pre-symptomatic / asymptomatic or symptomatic conclusively)
hCoV-19/Italy/ABR-IZSGC-TE26643/2020	EPI_ISL_436729	27/04/2020	Europe / Italy / Abruzzo	Human		Male	49	Live	Original		B.1.1	GR	Italy	Undertermined	No (couldn't determine the patient status as either pre-symptomatic / asymptomatic or symptomatic conclusively)
hCoV-19/Italy/ABR-IZSGC-TE26969/2020	EPI_ISL_436730	27/04/2020	Europe / Italy / Abruzzo	Human		Male	93	Hospitalized	Original		B.1	G	Italy	Undertermined	No (couldn't determine the patient status as either pre-symptomatic / asymptomatic or symptomatic conclusively)
hCoV-19/Italy/ABR-IZSGC-TE26423/2020	EPI_ISL_436731	26/04/2020	Europe / Italy / Abruzzo	Human		Female	89	Live	Original		B.1.1	GR	Italy	Undertermined	No (couldn't determine the patient status as either pre-symptomatic / asymptomatic or symptomatic conclusively)
hCoV-19/Italy/ABR-IZSGC-TE26425/2020	EPI_ISL_436732	27/04/2020	Europe / Italy / Abruzzo	Human		Female	101	Live	Original		B.1	G	Italy	Undertermined	No (couldn't determine the patient status as either pre-symptomatic / asymptomatic or symptomatic conclusively)
hCoV-19/Austria/CeMM0034/2020	EPI_ISL_437923	10/03/2020	Europe / Austria / Ischgl	Human		Male	23	-	Original	Oropharyngeal swab	B.1	GH	Austria	Undertermined	No (couldn't determine the patient status as either pre-symptomatic / asymptomatic or symptomatic conclusively)
hCoV-19/Austria/CeMM0035/2020	EPI_ISL_437924	07/03/2020	Europe / Austria / Ischgl	Human		Male	36	-	Original	Oropharyngeal swab	B.1	GH	Austria	Undertermined	No (couldn't determine the patient status as either pre-symptomatic / asymptomatic or symptomatic conclusively)
hCoV-19/Austria/CeMM0036/2020	EPI_ISL_437925	09/03/2020	Europe / Austria / Ischgl	Human		Female	25	-	Original	Oropharyngeal swab	B.1	GH	Austria	Undertermined	No (couldn't determine the patient status as either pre-symptomatic / asymptomatic or symptomatic conclusively)
hCoV-19/Austria/CeMM0037/2020	EPI_ISL_437926	09/03/2020	Europe / Austria / Ischgl	Human		Male	44	-	Original	Oropharyngeal swab	B.1	GH	Austria	Undertermined	No (couldn't determine the patient status as either pre-symptomatic / asymptomatic or symptomatic conclusively)
hCoV-19/Austria/CeMM0038/2020	EPI_ISL_437927	09/03/2020	Europe / Austria / Ischgl	Human		Male	58	-	Original	Oropharyngeal swab	B.1	GH	Austria	Undertermined	No (couldn't determine the patient status as either pre-symptomatic / asymptomatic or symptomatic conclusively)
hCoV-19/Austria/CeMM0039/2020	EPI_ISL_437928	10/03/2020	Europe / Austria / Ischgl	Human		Male	22	-	Original	Oropharyngeal swab	B.1	GH	Austria	Undertermined	No (couldn't determine the patient status as either pre-symptomatic / asymptomatic or symptomatic conclusively)
hCoV-19/Austria/CeMM0041/2020	EPI_ISL_437929	05/03/2020	Europe / Austria / Ischgl	Human		Male	23	-	Original	Oropharyngeal swab	B.1	G	Austria	Undertermined	No (couldn't determine the patient status as either pre-symptomatic / asymptomatic or symptomatic conclusively)
hCoV-19/Austria/CeMM0042/2020	EPI_ISL_437930	09/03/2020	Europe / Austria / Ischgl	Human		Male	25	-	Original	Oropharyngeal swab	B.1	GH	Austria	Undertermined	No (couldn't determine the patient status as either pre-symptomatic / asymptomatic or symptomatic conclusively)
hCoV-19/Austria/CeMM0043/2020	EPI_ISL_437931	09/03/2020	Europe / Austria / Ischgl	Human		Male	23	-	Original	Oropharyngeal swab	B.3	L	Austria	Undertermined	No (couldn't determine the patient status as either pre-symptomatic / asymptomatic or symptomatic conclusively)
hCoV-19/Austria/CeMM0045/2020	EPI_ISL_437932	24/02/2020	Europe / Austria / Ischgl	Human		Female	25	-	Original	Oropharyngeal swab	B.1.1	GR	Austria	Undertermined	No (couldn't determine the patient status as either pre-symptomatic / asymptomatic or symptomatic conclusively)
hCoV-19/Austria/CeMM0047/2020	EPI_ISL_437933	25/03/2020	Europe / Austria / Innsbruck	Human		Male	53	-	Original	Oropharyngeal swab	B.1.36	GH	Austria	Undertermined	No (couldn't determine the patient status as either pre-symptomatic / asymptomatic or symptomatic conclusively)
hCoV-19/Austria/CeMM0048/2020	EPI_ISL_437934	25/03/2020	Europe / Austria / Ischgl	Human		Female	52	-	Original	Oropharyngeal swab	B.1.22	GH	Austria	Undertermined	No (couldn't determine the patient status as either pre-symptomatic / asymptomatic or symptomatic conclusively)
hCoV-19/Austria/CeMM0052/2020	EPI_ISL_437935	30/03/2020	Europe / Austria / Ischgl	Human		Male	43	-	Original	Oropharyngeal swab	B.1	GH	Austria	Undertermined	No (couldn't determine the patient status as either pre-symptomatic / asymptomatic or symptomatic conclusively)
hCoV-19/Austria/CeMM0053/2020	EPI_ISL_437936	30/03/2020	Europe / Austria / Matrei am Brenner	Human		Female	80	-	Original	Oropharyngeal swab	B.1	GH	Austria	Undertermined	No (couldn't determine the patient status as either pre-symptomatic / asymptomatic or symptomatic conclusively)
hCoV-19/Austria/CeMM0055/2020	EPI_ISL_437937	27/03/2020	Europe / Austria / Innsbruck	Human		Male	71	-	Original	Oropharyngeal swab	B.3	L	Austria	Undertermined	No (couldn't determine the patient status as either pre-symptomatic / asymptomatic or symptomatic conclusively)
hCoV-19/Austria/CeMM0058/2020	EPI_ISL_437938	03/04/2020	Europe / Austria / Innsbruck	Human		Male	50	-	Original	Oropharyngeal swab	B.1	GH	Austria	Undertermined	No (couldn't determine the patient status as either pre-symptomatic / asymptomatic or symptomatic conclusively)
hCoV-19/Austria/CeMM0067/2020	EPI_ISL_437939	06/04/2020	Europe / Austria / Strass im Zillertal	Human		Male	84	-	Original	Oropharyngeal swab	B.1.1.1	GR	Austria	Undertermined	No (couldn't determine the patient status as either pre-symptomatic / asymptomatic or symptomatic conclusively)
hCoV-19/Austria/CeMM0068/2020	EPI_ISL_437940	12/03/2020	Europe / Austria / Innsbruck	Human		Female	21	-	Original	Oropharyngeal swab	B.1	G	Austria	Undertermined	No (couldn't determine the patient status as either pre-symptomatic / asymptomatic or symptomatic conclusively)
hCoV-19/Austria/CeMM0071/2020	EPI_ISL_437941	11/03/2020	Europe / Austria / Ischgl	Human		Male	22	-	Original	Oropharyngeal swab	B.1	G	Austria	Undertermined	No (couldn't determine the patient status as either pre-symptomatic / asymptomatic or symptomatic conclusively)
hCoV-19/Austria/CeMM0073/2020	EPI_ISL_437942	11/03/2020	Europe / Austria / Ischgl	Human		Female	23	-	Original	Oropharyngeal swab	B.1	GH	Austria	Undertermined	No (couldn't determine the patient status as either pre-symptomatic / asymptomatic or symptomatic conclusively)
hCoV-19/Austria/CeMM0074/2020	EPI_ISL_437943	17/03/2020	Europe / Austria / Elbigenalp	Human		Male	36	-	Original	Oropharyngeal swab	B.1	GH	Austria	Undertermined	No (couldn't determine the patient status as either pre-symptomatic / asymptomatic or symptomatic conclusively)
hCoV-19/Austria/CeMM0076/2020	EPI_ISL_437944	17/03/2020	Europe / Austria / Aldrans	Human		Male	31	-	Original	Oropharyngeal swab	B.1	GH	Austria	Undertermined	No (couldn't determine the patient status as either pre-symptomatic / asymptomatic or symptomatic conclusively)
hCoV-19/Austria/CeMM0077/2020	EPI_ISL_437945	17/03/2020	Europe / Austria / Innsbruck	Human		Female	52	-	Original	Oropharyngeal swab	B.1	GH	Austria	Undertermined	No (couldn't determine the patient status as either pre-symptomatic / asymptomatic or symptomatic conclusively)
hCoV-19/Austria/CeMM0078/2020	EPI_ISL_437946	24/03/2020	Europe / Austria / Innsbruck	Human		Female	63	-	Original	Oropharyngeal swab	B.1.1	GR	Austria	Undertermined	No (couldn't determine the patient status as either pre-symptomatic / asymptomatic or symptomatic conclusively)
hCoV-19/Austria/CeMM0080/2020	EPI_ISL_437947	24/03/2020	Europe / Austria / Ramsau im Zillertal	Human		Male	47	-	Original	Oropharyngeal swab	B	L	Austria	Undertermined	No (couldn't determine the patient status as either pre-symptomatic / asymptomatic or symptomatic conclusively)
hCoV-19/Austria/CeMM0083/2020	EPI_ISL_437948	24/03/2020	Europe / Austria / Innsbruck	Human		Male	59	-	Original	Oropharyngeal swab	B.1.1	GR	Austria	Undertermined	No (couldn't determine the patient status as either pre-symptomatic / asymptomatic or symptomatic conclusively)
hCoV-19/Austria/CeMM0084/2020	EPI_ISL_437949	24/03/2020	Europe / Austria / Steinach am Brenner	Human		Male	56	-	Original	Oropharyngeal swab	B.1	GH	Austria	Undertermined	No (couldn't determine the patient status as either pre-symptomatic / asymptomatic or symptomatic conclusively)
hCoV-19/Austria/CeMM0086/2020	EPI_ISL_437950	24/03/2020	Europe / Austria / Zams	Human		Female	7	-	Original	Oropharyngeal swab	B.1	G	Austria	Undertermined	No (couldn't determine the patient status as either pre-symptomatic / asymptomatic or symptomatic conclusively)
hCoV-19/Austria/CeMM0088/2020	EPI_ISL_437951	24/03/2020	Europe / Austria / Innsbruck	Human		Female	49	-	Original	Oropharyngeal swab	B.1	GH	Austria	Undertermined	No (couldn't determine the patient status as either pre-symptomatic / asymptomatic or symptomatic conclusively)
hCoV-19/Austria/CeMM0089/2020	EPI_ISL_437952	24/03/2020	Europe / Austria / Innsbruck	Human		Female	77	-	Original	Oropharyngeal swab	B.1.1	GR	Austria	Undertermined	No (couldn't determine the patient status as either pre-symptomatic / asymptomatic or symptomatic conclusively)
hCoV-19/Austria/CeMM0090/2020	EPI_ISL_437953	25/03/2020	Europe / Austria / Innsbruck	Human		Female	70	-	Original	Oropharyngeal swab	B.1.1	GR	Austria	Undertermined	No (couldn't determine the patient status as either pre-symptomatic / asymptomatic or symptomatic conclusively)
hCoV-19/Austria/CeMM0091/2020	EPI_ISL_437954	25/03/2020	Europe / Austria / Seefeld in Tirol	Human		Male	67	-	Original	Oropharyngeal swab	B.1.1	GR	Austria	Undertermined	No (couldn't determine the patient status as either pre-symptomatic / asymptomatic or symptomatic conclusively)
hCoV-19/Austria/CeMM0094/2020	EPI_ISL_437955	23/03/2020	Europe / Austria / Innsbruck	Human		Female	91	-	Original	Oropharyngeal swab	B.1	GH	Austria	Undertermined	No (couldn't determine the patient status as either pre-symptomatic / asymptomatic or symptomatic conclusively)
hCoV-19/Austria/CeMM0095/2020	EPI_ISL_437956	23/03/2020	Europe / Austria / Innsbruck	Human		Female	32	-	Original	Oropharyngeal swab	B.1	GH	Austria	Undertermined	No (couldn't determine the patient status as either pre-symptomatic / asymptomatic or symptomatic conclusively)
hCoV-19/Austria/CeMM0096/2020	EPI_ISL_437957	23/03/2020	Europe / Austria / Innsbruck	Human		Male	76	-	Original	Oropharyngeal swab	B.1	GH	Austria	Undertermined	No (couldn't determine the patient status as either pre-symptomatic / asymptomatic or symptomatic conclusively)
hCoV-19/Austria/CeMM0097/2020	EPI_ISL_437958	23/03/2020	Europe / Austria / Bad Gastein	Human		Male	76	-	Original	Oropharyngeal swab	B	L	Austria	Undertermined	No (couldn't determine the patient status as either pre-symptomatic / asymptomatic or symptomatic conclusively)
hCoV-19/Austria/CeMM0098/2020	EPI_ISL_437959	20/03/2020	Europe / Austria / Kematen In Tirol	Human		Male	55	-	Original	Oropharyngeal swab	B.1.1	GR	Austria	Undertermined	No (couldn't determine the patient status as either pre-symptomatic / asymptomatic or symptomatic conclusively)
hCoV-19/Austria/CeMM0099/2020	EPI_ISL_437960	20/03/2020	Europe / Austria / Innsbruck	Human		Male	41	-	Original	Oropharyngeal swab	B.1	GH	Austria	Undertermined	No (couldn't determine the patient status as either pre-symptomatic / asymptomatic or symptomatic conclusively)
hCoV-19/Austria/CeMM0100/2020	EPI_ISL_437961	20/03/2020	Europe / Austria / Innsbruck	Human		Female	27	-	Original	Oropharyngeal swab	B.1.1	GR	Austria	Undertermined	No (couldn't determine the patient status as either pre-symptomatic / asymptomatic or symptomatic conclusively)
hCoV-19/Austria/CeMM0101/2020	EPI_ISL_437962	20/03/2020	Europe / Austria / Innsbruck	Human		Male	46	-	Original	Oropharyngeal swab	B.1	GH	Austria	Undertermined	No (couldn't determine the patient status as either pre-symptomatic / asymptomatic or symptomatic conclusively)
hCoV-19/Austria/CeMM0102/2020	EPI_ISL_437963	19/03/2020	Europe / Austria / Innsbruck	Human		Male	28	-	Original	Oropharyngeal swab	B.1.1	GR	Austria	Undertermined	No (couldn't determine the patient status as either pre-symptomatic / asymptomatic or symptomatic conclusively)
hCoV-19/Austria/CeMM0103/2020	EPI_ISL_437964	19/03/2020	Europe / Austria / Innsbruck	Human		Male	39	-	Original	Oropharyngeal swab	B.1.1	GR	Austria	Undertermined	No (couldn't determine the patient status as either pre-symptomatic / asymptomatic or symptomatic conclusively)
hCoV-19/Austria/CeMM0104/2020	EPI_ISL_437965	19/03/2020	Europe / Austria / Innsbruck	Human		Male	51	-	Original	Oropharyngeal swab	B.1	GH	Austria	Undertermined	No (couldn't determine the patient status as either pre-symptomatic / asymptomatic or symptomatic conclusively)
hCoV-19/Austria/CeMM0106/2020	EPI_ISL_437966	18/03/2020	Europe / Austria / Laengenfeld	Human		Female	34	-	Original	Oropharyngeal swab	B.1.8	G	Austria	Undertermined	No (couldn't determine the patient status as either pre-symptomatic / asymptomatic or symptomatic conclusively)
hCoV-19/Austria/CeMM0108/2020	EPI_ISL_437967	13/03/2020	Europe / Austria / St. Anton	Human		Female	63	-	Original	Oropharyngeal swab	B.2	O	Austria	Undertermined	No (couldn't determine the patient status as either pre-symptomatic / asymptomatic or symptomatic conclusively)
hCoV-19/Austria/CeMM0109/2020	EPI_ISL_437968	12/03/2020	Europe / Austria / Innsbruck	Human		Female	32	-	Original	Oropharyngeal swab	B.1	G	Austria	Undertermined	No (couldn't determine the patient status as either pre-symptomatic / asymptomatic or symptomatic conclusively)
hCoV-19/Austria/CeMM0111/2020	EPI_ISL_437969	12/03/2020	Europe / Austria / Innsbruck	Human		Female	21	-	Original	Oropharyngeal swab	B.1.1	GR	Austria	Undertermined	No (couldn't determine the patient status as either pre-symptomatic / asymptomatic or symptomatic conclusively)
hCoV-19/Austria/CeMM0112/2020	EPI_ISL_437970	17/03/2020	Europe / Austria / Sistrans	Human		Female	63	-	Original	Oropharyngeal swab	B.1.1.1	GR	Austria	Undertermined	No (couldn't determine the patient status as either pre-symptomatic / asymptomatic or symptomatic conclusively)
hCoV-19/Austria/CeMM0113/2020	EPI_ISL_437971	18/03/2020	Europe / Austria / Innsbruck	Human		Male	39	-	Original	Oropharyngeal swab	B.1	GH	Austria	Undertermined	No (couldn't determine the patient status as either pre-symptomatic / asymptomatic or symptomatic conclusively)
hCoV-19/Austria/CeMM0114/2020	EPI_ISL_437972	18/03/2020	Europe / Austria / St. Anton	Human		Female	63	-	Original	Oropharyngeal swab	B.3	O	Austria	Undertermined	No (couldn't determine the patient status as either pre-symptomatic / asymptomatic or symptomatic conclusively)
hCoV-19/Austria/CeMM0115/2020	EPI_ISL_437973	18/03/2020	Europe / Austria / Innsbruck	Human		Female	81	-	Original	Oropharyngeal swab	B.1.5	G	Austria	Undertermined	No (couldn't determine the patient status as either pre-symptomatic / asymptomatic or symptomatic conclusively)
hCoV-19/Austria/CeMM0116/2020	EPI_ISL_437974	10/03/2020	Europe / Austria / Ischgl	Human		Female	21	-	Original	Oropharyngeal swab	B.1	GH	Austria	Undertermined	No (couldn't determine the patient status as either pre-symptomatic / asymptomatic or symptomatic conclusively)
hCoV-19/Austria/CeMM0117/2020	EPI_ISL_437975	13/03/2020	Europe / Austria / Ischgl	Human		Male	63	-	Original	Oropharyngeal swab	B.1	GH	Austria	Undertermined	No (couldn't determine the patient status as either pre-symptomatic / asymptomatic or symptomatic conclusively)
hCoV-19/Austria/CeMM0119/2020	EPI_ISL_437976	11/03/2020	Europe / Austria / Ischgl	Human		Female	23	-	Original	Oropharyngeal swab	B	L	Austria	Undertermined	No (couldn't determine the patient status as either pre-symptomatic / asymptomatic or symptomatic conclusively)
hCoV-19/Austria/CeMM0120/2020	EPI_ISL_437977	15/03/2020	Europe / Austria / Ischgl	Human		Male	36	-	Original	Oropharyngeal swab	B.1	G	Austria	Undertermined	No (couldn't determine the patient status as either pre-symptomatic / asymptomatic or symptomatic conclusively)
hCoV-19/Austria/CeMM0121/2020	EPI_ISL_437978	11/03/2020	Europe / Austria / Ischgl	Human		Female	19	-	Original	Oropharyngeal swab	B.1	GH	Austria	Undertermined	No (couldn't determine the patient status as either pre-symptomatic / asymptomatic or symptomatic conclusively)
hCoV-19/Austria/CeMM0123/2020	EPI_ISL_437979	11/03/2020	Europe / Austria / Ischgl	Human		Female	31	-	Original	Oropharyngeal swab	B.1	GH	Austria	Undertermined	No (couldn't determine the patient status as either pre-symptomatic / asymptomatic or symptomatic conclusively)
hCoV-19/Austria/CeMM0124/2020	EPI_ISL_437980	11/03/2020	Europe / Austria / Ischgl	Human		Male	53	-	Original	Oropharyngeal swab	B.1	GH	Austria	Undertermined	No (couldn't determine the patient status as either pre-symptomatic / asymptomatic or symptomatic conclusively)
hCoV-19/Austria/CeMM0125/2020	EPI_ISL_437981	11/03/2020	Europe / Austria / Ischgl	Human		Female	59	-	Original	Oropharyngeal swab	B.1	GH	Austria	Undertermined	No (couldn't determine the patient status as either pre-symptomatic / asymptomatic or symptomatic conclusively)
hCoV-19/Austria/CeMM0126/2020	EPI_ISL_437982	11/03/2020	Europe / Austria / Ischgl	Human		Female	21	-	Original	Oropharyngeal swab	B.1	G	Austria	Undertermined	No (couldn't determine the patient status as either pre-symptomatic / asymptomatic or symptomatic conclusively)
hCoV-19/Austria/CeMM0127/2020	EPI_ISL_437983	11/03/2020	Europe / Austria / Ischgl	Human		Female	55	-	Original	Oropharyngeal swab	B.1	G	Austria	Undertermined	No (couldn't determine the patient status as either pre-symptomatic / asymptomatic or symptomatic conclusively)
hCoV-19/Austria/CeMM0128/2020	EPI_ISL_437984	11/03/2020	Europe / Austria / Ischgl	Human		Female	30	-	Original	Oropharyngeal swab	B.1	GH	Austria	Undertermined	No (couldn't determine the patient status as either pre-symptomatic / asymptomatic or symptomatic conclusively)
hCoV-19/Austria/CeMM0132/2020	EPI_ISL_437985	13/03/2020	Europe / Austria / Ischgl	Human		Male	57	-	Original	Oropharyngeal swab	B.1	GH	Austria	Undertermined	No (couldn't determine the patient status as either pre-symptomatic / asymptomatic or symptomatic conclusively)
hCoV-19/Austria/CeMM0133/2020	EPI_ISL_437986	13/03/2020	Europe / Austria / Ischgl	Human		Male	49	-	Original	Oropharyngeal swab	B.1	GH	Austria	Undertermined	No (couldn't determine the patient status as either pre-symptomatic / asymptomatic or symptomatic conclusively)
hCoV-19/Austria/CeMM0134/2020	EPI_ISL_437987	13/03/2020	Europe / Austria / Ischgl	Human		Female	28	-	Original	Oropharyngeal swab	B.1	GH	Austria	Undertermined	No (couldn't determine the patient status as either pre-symptomatic / asymptomatic or symptomatic conclusively)
hCoV-19/Austria/CeMM0135/2020	EPI_ISL_437988	13/03/2020	Europe / Austria / Ischgl	Human		Female	32	-	Original	Oropharyngeal swab	B.1	GH	Austria	Undertermined	No (couldn't determine the patient status as either pre-symptomatic / asymptomatic or symptomatic conclusively)
hCoV-19/Austria/CeMM0136/2020	EPI_ISL_437989	13/03/2020	Europe / Austria / Ischgl	Human		Female	42	-	Original	Oropharyngeal swab	B.1	GH	Austria	Undertermined	No (couldn't determine the patient status as either pre-symptomatic / asymptomatic or symptomatic conclusively)
hCoV-19/Austria/CeMM0137/2020	EPI_ISL_437990	13/03/2020	Europe / Austria / Ischgl	Human		Male	27	-	Original	Oropharyngeal swab	B.1.1	GR	Austria	Undertermined	No (couldn't determine the patient status as either pre-symptomatic / asymptomatic or symptomatic conclusively)
hCoV-19/Austria/CeMM0139/2020	EPI_ISL_437991	13/03/2020	Europe / Austria / Ischgl	Human		Male	69	-	Original	Oropharyngeal swab	B.1	GH	Austria	Undertermined	No (couldn't determine the patient status as either pre-symptomatic / asymptomatic or symptomatic conclusively)
hCoV-19/Austria/CeMM0140/2020	EPI_ISL_437992	13/03/2020	Europe / Austria / Ischgl	Human		Male	35	-	Original	Oropharyngeal swab	B.1	G	Austria	Undertermined	No (couldn't determine the patient status as either pre-symptomatic / asymptomatic or symptomatic conclusively)
hCoV-19/Austria/CeMM0143/2020	EPI_ISL_437993	27/02/2020	Europe / Austria / Vienna	Human		Female	46	-	Original	Oropharyngeal swab	B.1.1.6	GR	Austria	Undertermined	No (couldn't determine the patient status as either pre-symptomatic / asymptomatic or symptomatic conclusively)
hCoV-19/Austria/CeMM0146/2020	EPI_ISL_437994	28/02/2020	Europe / Austria / Vienna	Human		Female	40	-	Original	Oropharyngeal swab	B.1.1.6	GR	Austria	Undertermined	No (couldn't determine the patient status as either pre-symptomatic / asymptomatic or symptomatic conclusively)
hCoV-19/Austria/CeMM0147/2020	EPI_ISL_437995	28/02/2020	Europe / Austria / Vienna	Human		Male	36	-	Original	Oropharyngeal swab	B.1.1.6	GR	Austria	Undertermined	No (couldn't determine the patient status as either pre-symptomatic / asymptomatic or symptomatic conclusively)
hCoV-19/Austria/CeMM0148/2020	EPI_ISL_437996	02/03/2020	Europe / Austria / Vienna	Human		Female	53	-	Original	Oropharyngeal swab	B.1.1.6	GR	Austria	Undertermined	No (couldn't determine the patient status as either pre-symptomatic / asymptomatic or symptomatic conclusively)
hCoV-19/Austria/CeMM0149/2020	EPI_ISL_437997	03/03/2020	Europe / Austria / Vienna	Human		Female	55	-	Original	Oropharyngeal swab	B.1.1.6	GR	Austria	Undertermined	No (couldn't determine the patient status as either pre-symptomatic / asymptomatic or symptomatic conclusively)
hCoV-19/Austria/CeMM0150/2020	EPI_ISL_437998	03/03/2020	Europe / Austria / Vienna	Human		Female	30	-	Original	Oropharyngeal swab	B.1.1.6	GR	Austria	Undertermined	No (couldn't determine the patient status as either pre-symptomatic / asymptomatic or symptomatic conclusively)
hCoV-19/Austria/CeMM0153/2020	EPI_ISL_437999	03/03/2020	Europe / Austria / Vienna	Human		Female	25	-	Original	Oropharyngeal swab	B.1.1	GR	Austria	Undertermined	No (couldn't determine the patient status as either pre-symptomatic / asymptomatic or symptomatic conclusively)
hCoV-19/Iran/KHGRC-1.1-IPI-8206/2020	EPI_ISL_442523	09/03/2020	Asia / Iran / Semnan	Human		unknown	unknown	Hospitalized	Original		B	O	Iran	Undertermined	No (couldn't determine the patient status as either pre-symptomatic / asymptomatic or symptomatic conclusively)
hCoV-19/Guangdong/SYSU-IHV/2020	EPI_ISL_444969	16/04/2020	Asia / China / Guangdong / Guangzhou	Human		Female	21	Hospitalized	Original	Sputum	B.1	G	China	Undertermined	No (couldn't determine the patient status as either pre-symptomatic / asymptomatic or symptomatic conclusively)
hCoV-19/USA/CA_NHRC_01/2020	EPI_ISL_444994	11/03/2020	North America / USA / California	Human		Male	21	Outpatient	Original	NP	A.1	S	USA	Undertermined	No (couldn't determine the patient status as either pre-symptomatic / asymptomatic or symptomatic conclusively)
hCoV-19/USA/SC_NHRC_01/2020	EPI_ISL_444995	27/03/2020	North America / USA / South Carolina	Human		Male	29	Outpatient	Original	NP	B.1	GH	USA	Undertermined	No (couldn't determine the patient status as either pre-symptomatic / asymptomatic or symptomatic conclusively)
hCoV-19/USA/SC_NHRC_02/2020	EPI_ISL_444996	27/03/2020	North America / USA / South Carolina	Human		Female	34	Outpatient	Original	NP	B.1	GH	USA	Undertermined	No (couldn't determine the patient status as either pre-symptomatic / asymptomatic or symptomatic conclusively)
hCoV-19/Guam/GU_NHG_01/2020	EPI_ISL_444998	26/03/2020	Oceania / Guam	Human		Male	62	Outpatient	Original	NP	B.6	O	Guam	Undertermined	No (couldn't determine the patient status as either pre-symptomatic / asymptomatic or symptomatic conclusively)
hCoV-19/Israel/701002442/2020	EPI_ISL_447300	24/03/2020	Asia / Israel / South Coast District	Human		Male	37	Recovered	Original	Naso-pharyngeal swab	B.1	GH	Israel	Undertermined	No (couldn't determine the patient status as either pre-symptomatic / asymptomatic or symptomatic conclusively)
hCoV-19/Israel/701002440/2020	EPI_ISL_447301	24/03/2020	Asia / Israel / South Coast District	Human		Female	25	Recovered	Original	Naso-pharyngeal swab	B.1	GH	Israel	Undertermined	No (couldn't determine the patient status as either pre-symptomatic / asymptomatic or symptomatic conclusively)
hCoV-19/Israel/701002431/2020	EPI_ISL_447302	24/03/2020	Asia / Israel / South Coast District	Human		Male	25	Recovered	Original	Naso-pharyngeal swab	B.1.1	GR	Israel	Undertermined	No (couldn't determine the patient status as either pre-symptomatic / asymptomatic or symptomatic conclusively)
hCoV-19/Israel/701002426/2020	EPI_ISL_447303	24/03/2020	Asia / Israel / South Coast District	Human		Male	60	Recovered	Original	Naso-pharyngeal swab	B.1	GH	Israel	Undertermined	No (couldn't determine the patient status as either pre-symptomatic / asymptomatic or symptomatic conclusively)
hCoV-19/Israel/701002407/2020	EPI_ISL_447304	23/03/2020	Asia / Israel / South Coast District	Human		Male	41	Recovered	Original	Naso-pharyngeal swab	B.1	O	Israel	Undertermined	No (couldn't determine the patient status as either pre-symptomatic / asymptomatic or symptomatic conclusively)
hCoV-19/Israel/701002403/2020	EPI_ISL_447305	23/03/2020	Asia / Israel / South Coast District	Human		Male	40	Recovered	Original	Naso-pharyngeal swab	B.1	G	Israel	Undertermined	No (couldn't determine the patient status as either pre-symptomatic / asymptomatic or symptomatic conclusively)
hCoV-19/Israel/701002334/2020	EPI_ISL_447306	22/03/2020	Asia / Israel / South Coast District	Human		Male	77	Deceased	Original	Naso-pharyngeal swab	B.1	GH	Israel	Symptomatic	Yes
hCoV-19/Israel/701002327/2020	EPI_ISL_447307	22/03/2020	Asia / Israel / South Coast District	Human		Male	55	Recovered	Original	Naso-pharyngeal swab	B.1.115	GH	Israel	Undertermined	No (couldn't determine the patient status as either pre-symptomatic / asymptomatic or symptomatic conclusively)
hCoV-19/Israel/701002317/2020	EPI_ISL_447308	21/03/2020	Asia / Israel / South Coast District	Human		Male	54	Recovered	Original	Naso-pharyngeal swab	B.1	GH	Israel	Undertermined	No (couldn't determine the patient status as either pre-symptomatic / asymptomatic or symptomatic conclusively)
hCoV-19/Israel/701002314/2020	EPI_ISL_447309	21/03/2020	Asia / Israel / South Coast District	Human		Male	24	Recovered	Original	Naso-pharyngeal swab	B.1.115	GH	Israel	Undertermined	No (couldn't determine the patient status as either pre-symptomatic / asymptomatic or symptomatic conclusively)
hCoV-19/Israel/701002313/2020	EPI_ISL_447310	21/03/2020	Asia / Israel / South Coast District	Human		Male	52	Recovered	Original	Naso-pharyngeal swab	B.1	GH	Israel	Undertermined	No (couldn't determine the patient status as either pre-symptomatic / asymptomatic or symptomatic conclusively)
hCoV-19/Belgium/ITM_C184/2020	EPI_ISL_450730	30/03/2020	Europe / Belgium / Antwerp	Human		Female	76	Hospitalized	Original	Oro-pharyngeal swab	B.1.1	GR	Belgium	Undertermined	No (couldn't determine the patient status as either pre-symptomatic / asymptomatic or symptomatic conclusively)
hCoV-19/Belgium/ITM_C318/2020	EPI_ISL_450733	05/04/2020	Europe / Belgium / Antwerp	Human		Female	38	Not hospitalized	Original	Oro-pharyngeal swab	B.1	G	Belgium	Undertermined	No (couldn't determine the patient status as either pre-symptomatic / asymptomatic or symptomatic conclusively)
hCoV-19/Belgium/ITM_C359/2020	EPI_ISL_450735	07/04/2020	Europe / Belgium / Antwerp	Human		Female	35	Not hospitalized	Original	Oro-pharyngeal swab	B.1	G	Belgium	Undertermined	No (couldn't determine the patient status as either pre-symptomatic / asymptomatic or symptomatic conclusively)
hCoV-19/Poland/Pom2/2020	EPI_ISL_450746	08/05/2020	Europe / Poland / Pomerania	Human		Male	78	Hospitalized	Original	Swab	B.1.1.1	GR	Poland	Undertermined	No (couldn't determine the patient status as either pre-symptomatic / asymptomatic or symptomatic conclusively)
hCoV-19/Canada/ON_SHSC-01/2020	EPI_ISL_450747	26/02/2020	North America / Canada / Ontario	Human	Iran	unknown	unknown	unknown	Original	Nasal swab	B.4	O	Canada	Undertermined	No (couldn't determine the patient status as either pre-symptomatic / asymptomatic or symptomatic conclusively)
hCoV-19/Kazakhstan/18243/2020	EPI_ISL_454500	16/04/2020	Asia / Kazakhstan / Nur-Sultan	Human		Female	74	Released	Original		A.2	S	Kazakhstan	Undertermined	No (couldn't determine the patient status as either pre-symptomatic / asymptomatic or symptomatic conclusively)
hCoV-19/Kazakhstan/20679/2020	EPI_ISL_454501	20/04/2020	Asia / Kazakhstan / Nur-Sultan	Human		Female	26	Released	Original	Oro-pharyngeal swab	B.1.3	GH	Kazakhstan	Undertermined	No (couldn't determine the patient status as either pre-symptomatic / asymptomatic or symptomatic conclusively)
hCoV-19/Kazakhstan/21399/2020	EPI_ISL_454502	20/04/2020	Asia / Kazakhstan / Nur-Sultan	Human		Male	2	Released	Original	Oro-pharyngeal swab	B.1.36	GH	Kazakhstan	Undertermined	No (couldn't determine the patient status as either pre-symptomatic / asymptomatic or symptomatic conclusively)
hCoV-19/Kazakhstan/21927/2020	EPI_ISL_454503	22/04/2020	Asia / Kazakhstan / Nur-Sultan	Human		Male	33	Released	Original	Oro-pharyngeal swab	A.2	S	Kazakhstan	Undertermined	No (couldn't determine the patient status as either pre-symptomatic / asymptomatic or symptomatic conclusively)
hCoV-19/Kazakhstan/26473/2020	EPI_ISL_454504	26/04/2020	Asia / Kazakhstan / Nur-Sultan	Human		Male	19	Released	Original	Oro-pharyngeal swab	B.4	O	Kazakhstan	Undertermined	No (couldn't determine the patient status as either pre-symptomatic / asymptomatic or symptomatic conclusively)
hCoV-19/Kazakhstan/26474/2020	EPI_ISL_454505	26/04/2020	Asia / Kazakhstan / Nur-Sultan	Human		Male	20	Released	Original	Oro-pharyngeal swab	B.4	O	Kazakhstan	Undertermined	No (couldn't determine the patient status as either pre-symptomatic / asymptomatic or symptomatic conclusively)
hCoV-19/Kazakhstan/26478/2020	EPI_ISL_454506	26/04/2020	Asia / Kazakhstan / Nur-Sultan	Human		Female	20	Released	Original		B.4	O	Kazakhstan	Undertermined	No (couldn't determine the patient status as either pre-symptomatic / asymptomatic or symptomatic conclusively)

hCoV-19/Kazakhstan/26489/2020	EPI_ISL_454507	26/04/2020	Asia / Kazakhstan / Nur-Sultan	Human	Male	23	Released	Original	Oro-pharyngeal swab	B.4	O	Kazakhstan	Undertermined	No (couldn't determine the patient status as either pre-symptomatic / asymptomatic or symptomatic conclusively)
hCoV-19/Kazakhstan/26491/2020	EPI_ISL_454508	19/04/2020	Asia / Kazakhstan / Nur-Sultan	Human	Female	20	Released	Original	Oro-pharyngeal swab	B.4	O	Kazakhstan	Undertermined	No (couldn't determine the patient status as either pre-symptomatic / asymptomatic or symptomatic conclusively)
hCoV-19/Kazakhstan/26497/2020	EPI_ISL_454509	26/04/2020	Asia / Kazakhstan / Nur-Sultan	Human	Male	20	Released	Original	Oro-pharyngeal swab	B.4	O	Kazakhstan	Undertermined	No (couldn't determine the patient status as either pre-symptomatic / asymptomatic or symptomatic conclusively)
hCoV-19/Kazakhstan/26501/2020	EPI_ISL_454510	26/04/2020	Asia / Kazakhstan / Nur-Sultan	Human	Female	19	Released	Original	Oro-pharyngeal swab	B.4	O	Kazakhstan	Undertermined	No (couldn't determine the patient status as either pre-symptomatic / asymptomatic or symptomatic conclusively)
hCoV-19/Kazakhstan/26506/2020	EPI_ISL_454511	26/04/2020	Asia / Kazakhstan / Nur-Sultan	Human	Male	19	Released	Original	Oro-pharyngeal swab	B.4	O	Kazakhstan	Undertermined	No (couldn't determine the patient status as either pre-symptomatic / asymptomatic or symptomatic conclusively)
hCoV-19/Kazakhstan/26548/2020	EPI_ISL_454512	26/04/2020	Asia / Kazakhstan / Nur-Sultan	Human	Male	22	Released	Original	Oro-pharyngeal swab	B.4	O	Kazakhstan	Undertermined	No (couldn't determine the patient status as either pre-symptomatic / asymptomatic or symptomatic conclusively)
hCoV-19/Kazakhstan/26549/2020	EPI_ISL_454513	26/04/2020	Asia / Kazakhstan / Nur-Sultan	Human	Male	20	Released	Original	Oro-pharyngeal swab	B.4	O	Kazakhstan	Undertermined	No (couldn't determine the patient status as either pre-symptomatic / asymptomatic or symptomatic conclusively)
hCoV-19/Kazakhstan/26585/2020	EPI_ISL_454514	26/04/2020	Asia / Kazakhstan / Nur-Sultan	Human	Male	21	Released	Original	Oro-pharyngeal swab	B.4	O	Kazakhstan	Undertermined	No (couldn't determine the patient status as either pre-symptomatic / asymptomatic or symptomatic conclusively)
hCoV-19/Kazakhstan/26617/2020	EPI_ISL_454515	26/04/2020	Asia / Kazakhstan / Nur-Sultan	Human	Male	19	Released	Original	Oro-pharyngeal swab	B.4	O	Kazakhstan	Undertermined	No (couldn't determine the patient status as either pre-symptomatic / asymptomatic or symptomatic conclusively)
hCoV-19/Kazakhstan/26827/2020	EPI_ISL_454516	26/04/2020	Asia / Kazakhstan / Nur-Sultan	Human	Male	2	Released	Original	Oro-pharyngeal swab	A.2	S	Kazakhstan	Undertermined	No (couldn't determine the patient status as either pre-symptomatic / asymptomatic or symptomatic conclusively)
hCoV-19/Kazakhstan/26828/2020	EPI_ISL_454517	26/04/2020	Asia / Kazakhstan / Nur-Sultan	Human	Female	2	Released	Original	Oro-pharyngeal swab	A.2	S	Kazakhstan	Undertermined	No (couldn't determine the patient status as either pre-symptomatic / asymptomatic or symptomatic conclusively)
hCoV-19/Kazakhstan/26829/2020	EPI_ISL_454518	26/04/2020	Asia / Kazakhstan / Nur-Sultan	Human	Male	11	Released	Original	Oro-pharyngeal swab	A.2	S	Kazakhstan	Undertermined	No (couldn't determine the patient status as either pre-symptomatic / asymptomatic or symptomatic conclusively)
hCoV-19/Kazakhstan/34391/2020	EPI_ISL_454519	26/04/2020	Asia / Kazakhstan / Nur-Sultan	Human	Female	41	Released	Original	Oro-pharyngeal swab	A.2	S	Kazakhstan	Undertermined	No (couldn't determine the patient status as either pre-symptomatic / asymptomatic or symptomatic conclusively)
hCoV-19/Kazakhstan/38533/2020	EPI_ISL_454520	26/04/2020	Asia / Kazakhstan / Nur-Sultan	Human	Female	41	Released	Original	Oro-pharyngeal swab	A.2	S	Kazakhstan	Undertermined	No (couldn't determine the patient status as either pre-symptomatic / asymptomatic or symptomatic conclusively)
hCoV-19/India/MH-NIV-11182/2020	EPI_ISL_454521	18/04/2020	Asia / India / Maharashtra	Human	Female	1.5	Mild	Original		B.1.1.32	GR	India	Symptomatic	Yes
hCoV-19/India/MH-NIV-4271/2020	EPI_ISL_454530	22/03/2020	Asia / India / Maharashtra	Human	Male	46	Mild	Original		B.1.80	G	India	Symptomatic	Yes
hCoV-19/India/MH-NIV-4369/2020	EPI_ISL_454531	22/03/2020	Asia / India / Maharashtra	Human	Male	65	Mild	Original		A.7	S	India	Symptomatic	Yes
hCoV-19/India/MH-NIV-4379/2020	EPI_ISL_454532	23/03/2020	Asia / India / Maharashtra	Human	Female	45	Mild	Original		B.1	G	India	Symptomatic	Yes
hCoV-19/Croatia/14412_Koprivnica/2020	EPI_ISL_454602	21/04/2020	Europe / Croatia / Koprivnica	Human	Male	68	asymptomatic	Original	Nasopharyngeal swab	B.1.1	GR	Croatia	Pre-symptomatic / Asymptomatic	Yes
hCoV-19/Croatia/8U-S17new/2020	EPI_ISL_454605	31/03/2020	Europe / Croatia / Istria	Human	Female	61	Live	Original	Oro-pharyngeal swab	B.1	GH	Croatia	Undertermined	No (couldn't determine the patient status as either pre-symptomatic / asymptomatic or symptomatic conclusively)
hCoV-19/Croatia/I7-S21new/2020	EPI_ISL_454606	30/03/2020	Europe / Croatia / Istria	Human	Female	60	Live	Original		B.1	GH	Croatia	Undertermined	No (couldn't determine the patient status as either pre-symptomatic / asymptomatic or symptomatic conclusively)
hCoV-19/Vietnam/VNHN_1098/2020	EPI_ISL_455694	18/03/2020	Asia / Vietnam / Hanoi	Human	Male	19	Released	Original	Oro-pharyngeal swab	B.1.1	GR	Vietnam	Undertermined	No (couldn't determine the patient status as either pre-symptomatic / asymptomatic or symptomatic conclusively)
hCoV-19/Vietnam/VNHN_1099/2020	EPI_ISL_455695	18/03/2020	Asia / Vietnam / Hanoi	Human	Female	19	Released	Original	Oro-pharyngeal swab	B.1.1	GR	Vietnam	Undertermined	No (couldn't determine the patient status as either pre-symptomatic / asymptomatic or symptomatic conclusively)
hCoV-19/Vietnam/VNHN_1167/2020	EPI_ISL_455696	18/03/2020	Asia / Vietnam / Hanoi	Human	Male	21	Released	Original	Oro-pharyngeal swab	B.1	GH	Vietnam	Undertermined	No (couldn't determine the patient status as either pre-symptomatic / asymptomatic or symptomatic conclusively)
hCoV-19/Vietnam/VNHN_1528/2020	EPI_ISL_455697	22/03/2020	Asia / Vietnam / Hanoi	Human	Male	19	Released	Original	Oro-pharyngeal swab	B.1.1	GR	Vietnam	Undertermined	No (couldn't determine the patient status as either pre-symptomatic / asymptomatic or symptomatic conclusively)
hCoV-19/Vietnam/VNHN_1226/2020	EPI_ISL_455698	19/03/2020	Asia / Vietnam / Hanoi	Human	Male	19	Released	Original	Oro-pharyngeal swab	B.1.1	GR	Vietnam	Undertermined	No (couldn't determine the patient status as either pre-symptomatic / asymptomatic or symptomatic conclusively)
hCoV-19/Vietnam/VNHN_1492/2020	EPI_ISL_455699	21/03/2020	Asia / Vietnam / Hanoi	Human	Male	29	Released	Original	Oro-pharyngeal swab	B.1.1	GR	Vietnam	Undertermined	No (couldn't determine the patient status as either pre-symptomatic / asymptomatic or symptomatic conclusively)
hCoV-19/Taiwan/TSGH-38/2020	EPI_ISL_458029	24/03/2020	Asia / Taiwan / Taipei	Human	Male	34	Released	Original		B.1	GH	Taiwan	Undertermined	No (couldn't determine the patient status as either pre-symptomatic / asymptomatic or symptomatic conclusively)
hCoV-19/India/GJ-GBRC141a/2020	EPI_ISL_458100	24/05/2020	Asia / India / Gujarat / Ahmedabad	Human	Female	75	Hospitalized	Original		B.1.113	GH	India	Undertermined	No (couldn't determine the patient status as either pre-symptomatic / asymptomatic or symptomatic conclusively)
hCoV-19/India/GJ-GBRC141b/2020	EPI_ISL_458101	24/05/2020	Asia / India / Gujarat / Ahmedabad	Human	Female	75	Hospitalized	Original		B.1	GH	India	Undertermined	No (couldn't determine the patient status as either pre-symptomatic / asymptomatic or symptomatic conclusively)
hCoV-19/India/GJ-GBRC142/2020	EPI_ISL_458102	24/05/2020	Asia / India / Gujarat / Ahmedabad	Human	Female	80	Hospitalized	Original		B.1.36	GH	India	Undertermined	No (couldn't determine the patient status as either pre-symptomatic / asymptomatic or symptomatic conclusively)
hCoV-19/India/GJ-GBRC143/2020	EPI_ISL_458103	05/05/2020	Asia / India / Gujarat / Bayad	Human	Male	50	Released, Live	Original		B.1.113	GH	India	Undertermined	No (couldn't determine the patient status as either pre-symptomatic / asymptomatic or symptomatic conclusively)
hCoV-19/India/GJ-GBRC144/2020	EPI_ISL_458104	06/05/2020	Asia / India / Gujarat / Prantij	Human	Female	60	Released, Live	Original		B.1	G	India	Undertermined	No (couldn't determine the patient status as either pre-symptomatic / asymptomatic or symptomatic conclusively)
hCoV-19/India/GJ-GBRC145/2020	EPI_ISL_458105	06/05/2020	Asia / India / Gujarat / Prantij	Human	Female	70	Released, Live	Original		B.1	G	India	Undertermined	No (couldn't determine the patient status as either pre-symptomatic / asymptomatic or symptomatic conclusively)
hCoV-19/India/GJ-GBRC146a/2020	EPI_ISL_458106	16/05/2020	Asia / India / Gujarat / Himatnagar	Human	Female	45	Released, Live	Original		B.1	G	India	Undertermined	No (couldn't determine the patient status as either pre-symptomatic / asymptomatic or symptomatic conclusively)
hCoV-19/India/GJ-GBRC146b/2020	EPI_ISL_458107	16/05/2020	Asia / India / Gujarat / Himatnagar	Human	Female	45	Released, Live	Original		B.1	G	India	Undertermined	No (couldn't determine the patient status as either pre-symptomatic / asymptomatic or symptomatic conclusively)
hCoV-19/India/GJ-GBRC149/2020	EPI_ISL_458111	17/05/2020	Asia / India / Gujarat / Himatnagar	Human	Male	30	Released, Live	Original		B.1.113	GH	India	Undertermined	No (couldn't determine the patient status as either pre-symptomatic / asymptomatic or symptomatic conclusively)
hCoV-19/India/GJ-GBRC150a/2020	EPI_ISL_458112	18/05/2020	Asia / India / Gujarat / Himatnagar	Human	Male	32	Released, Live	Original		B.1.113	GH	India	Undertermined	No (couldn't determine the patient status as either pre-symptomatic / asymptomatic or symptomatic conclusively)
hCoV-19/India/GJ-GBRC150b/2020	EPI_ISL_458113	18/05/2020	Asia / India / Gujarat / Himatnagar	Human	Male	32	Released, Live	Original		B.1.113	GH	India	Undertermined	No (couldn't determine the patient status as either pre-symptomatic / asymptomatic or symptomatic conclusively)
hCoV-19/Oman/205033013/2020	EPI_ISL_458116	01/05/2020	Asia / Oman / Muscat	Human	Male	65	Deceased	Original	Tracheal swab	B.1.1.27	GR	Oman	Symptomatic	Yes
hCoV-19/Oman/205034603/2020	EPI_ISL_458117	09/05/2020	Asia / Oman / Muscat	Human	Female	53	Hospitalized	Original	Tracheal swab	B.1.1.27	GR	Oman	Undertermined	No (couldn't determine the patient status as either pre-symptomatic / asymptomatic or symptomatic conclusively)
hCoV-19/Oman/205033010/2020	EPI_ISL_458118	01/05/2020	Asia / Oman / Muscat	Human	Female	44	Hospitalized	Original	Oro-pharyngeal swab	B.1.1.27	GR	Oman	Undertermined	No (couldn't determine the patient status as either pre-symptomatic / asymptomatic or symptomatic conclusively)
hCoV-19/Oman/205028997/2020	EPI_ISL_458120	13/04/2020	Asia / Oman / Muscat	Human	Male	unknown	Hospitalized	Original	Oro-pharyngeal swab	B.1.1.27	GR	Oman	Undertermined	No (couldn't determine the patient status as either pre-symptomatic / asymptomatic or symptomatic conclusively)
hCoV-19/Bangladesh/NIB-BCSIR-02/2020	EPI_ISL_458133	11/05/2020	Asia / Bangladesh / Dhaka	Human	Female	45	Hospitalized	Original	Oropharyngeal swab	B	L	Bangladesh	Undertermined	No (couldn't determine the patient status as either pre-symptomatic / asymptomatic or symptomatic conclusively)
hCoV-19/Brazil/AP-IEC-162741/2020	EPI_ISL_458138	03/04/2020	South America / Brazil / Amapa	Human	Female	37	ï»¿unknown	Original	Sputum	B.1.1.33	GR	Brazil	Undertermined	No (couldn't determine the patient status as either pre-symptomatic / asymptomatic or symptomatic conclusively)
hCoV-19/Brazil/AC-IEC-162535/2020	EPI_ISL_458139	18/03/2020	South America / Brazil / Acre	Human	Male	81	Hospitalized	Original	Oro-pharyngeal swab	B.1.1.33	GR	Brazil	Undertermined	No (couldn't determine the patient status as either pre-symptomatic / asymptomatic or symptomatic conclusively)
hCoV-19/Brazil/PA-IEC-162802/2020	EPI_ISL_458140	07/04/2020	South America / Brazil / Para	Human	Male	63	ï»¿unknown	Original	Oro-pharyngeal swab	B.1.1.28	GR	Brazil	Undertermined	No (couldn't determine the patient status as either pre-symptomatic / asymptomatic or symptomatic conclusively)
hCoV-19/Brazil/PA-IEC-164239/2020	EPI_ISL_458141	26/04/2020	South America / Brazil / Para	Human	Male	38	ï»¿unknown	Original	Oro-pharyngeal swab	B.1.1.28	GR	Brazil	Undertermined	No (couldn't determine the patient status as either pre-symptomatic / asymptomatic or symptomatic conclusively)
hCoV-19/Brazil/AP-IEC-162966/2020	EPI_ISL_458142	05/04/2020	South America / Brazil / Amapa	Human	Female	37	ï»¿unknown	Original	Oro-pharyngeal swab	B.1.1.33	GR	Brazil	Undertermined	No (couldn't determine the patient status as either pre-symptomatic / asymptomatic or symptomatic conclusively)
hCoV-19/Brazil/AP-IEC-164082/2020	EPI_ISL_458143	15/04/2020	South America / Brazil / Amapa	Human	Male	44	ï»¿unknown	Original	Sputum	B.1.1.33	GR	Brazil	Undertermined	No (couldn't determine the patient status as either pre-symptomatic / asymptomatic or symptomatic conclusively)
hCoV-19/Brazil/AP-IEC-163972/2020	EPI_ISL_458144	15/04/2020	South America / Brazil / Amapa	Human	Male	44	ï»¿unknown	Original	Sputum	B.1.1.33	GR	Brazil	Undertermined	No (couldn't determine the patient status as either pre-symptomatic / asymptomatic or symptomatic conclusively)
hCoV-19/Brazil/AP-IEC-164346/2020	EPI_ISL_458145	20/04/2020	South America / Brazil / Amapa	Human	Female	62	ï»¿unknown	Original	Sputum	B.1.1.33	GR	Brazil	Undertermined	No (couldn't determine the patient status as either pre-symptomatic / asymptomatic or symptomatic conclusively)
hCoV-19/Brazil/PA-IEC-164173/2020	EPI_ISL_458146	23/04/2020	South America / Brazil / Para	Human	Female	38	ï»¿unknown	Original	Oro-pharyngeal swab	B.1.1.28	GR	Brazil	Undertermined	No (couldn't determine the patient status as either pre-symptomatic / asymptomatic or symptomatic conclusively)
hCoV-19/Brazil/PA-IEC-164218/2020	EPI_ISL_458147	24/04/2020	South America / Brazil / Para	Human	Male	45	ï»¿unknown	Original	Oro-pharyngeal swab	B.1.1.28	GR	Brazil	Undertermined	No (couldn't determine the patient status as either pre-symptomatic / asymptomatic or symptomatic conclusively)
hCoV-19/Brazil/PA-IEC-164684/2020	EPI_ISL_458148	27/04/2020	South America / Brazil / Para	Human	Male	38	ï»¿unknown	Original	Oro-pharyngeal swab	B.1.1.33	GR	Brazil	Undertermined	No (couldn't determine the patient status as either pre-symptomatic / asymptomatic or symptomatic conclusively)
hCoV-19/Brazil/MA-IEC-163069/2020	EPI_ISL_458149	06/04/2020	South America / Brazil / Maranhao	Human	Female	40	ï»¿unknown	Original	Oro-pharyngeal swab	B.1.1.33	GR	Brazil	Undertermined	No (couldn't determine the patient status as either pre-symptomatic / asymptomatic or symptomatic conclusively)
hCoV-19/India/GJ-GBRC151a/2020	EPI_ISL_461478	02/05/2020	Asia / India / Gujarat / Vadodara	Human	Female	45	Released	Original		B.1	G	India	Undertermined	No (couldn't determine the patient status as either pre-symptomatic / asymptomatic or symptomatic conclusively)
hCoV-19/India/GJ-GBRC151b/2020	EPI_ISL_461479	02/05/2020	Asia / India / Gujarat / Vadodara	Human	Female	45	Released	Original		B.1	G	India	Undertermined	No (couldn't determine the patient status as either pre-symptomatic / asymptomatic or symptomatic conclusively)
hCoV-19/India/GJ-GBRC152/2020	EPI_ISL_461480	02/05/2020	Asia / India / Gujarat / Vadodara	Human	Female	53	Released	Original		B.1.36	GH	India	Undertermined	No (couldn't determine the patient status as either pre-symptomatic / asymptomatic or symptomatic conclusively)
hCoV-19/India/GJ-GBRC153a/2020	EPI_ISL_461481	27/04/2020	Asia / India / Gujarat / Rajkot	Human	Male	18	Released	Original		B.1.80	G	India	Undertermined	No (couldn't determine the patient status as either pre-symptomatic / asymptomatic or symptomatic conclusively)
hCoV-19/India/GJ-GBRC153b/2020	EPI_ISL_461482	27/04/2020	Asia / India / Gujarat / Rajkot	Human	Male	18	Released	Original		A	S	India	Undertermined	No (couldn't determine the patient status as either pre-symptomatic / asymptomatic or symptomatic conclusively)
hCoV-19/India/GJ-GBRC154/2020	EPI_ISL_461483	27/05/2020	Asia / India / Gujarat / Ahmedabad	Human	Male	68	Hospitalized	Original		B.1.80	G	India	Undertermined	No (couldn't determine the patient status as either pre-symptomatic / asymptomatic or symptomatic conclusively)
hCoV-19/India/GJ-GBRC155/2020	EPI_ISL_461484	27/05/2020	Asia / India / Gujarat / Ahmedabad	Human	Female	60	Hospitalized	Original		B.1.113	GH	India	Undertermined	No (couldn't determine the patient status as either pre-symptomatic / asymptomatic or symptomatic conclusively)
hCoV-19/India/GJ-GBRC156/2020	EPI_ISL_461485	27/05/2020	Asia / India / Gujarat / Ahmedabad	Human	Female	57	Deceased	Original		B.1.113	GH	India	Symptomatic	Yes
hCoV-19/India/GJ-GBRC157a/2020	EPI_ISL_461486	27/05/2020	Asia / India / Gujarat / Ahmedabad	Human	Male	83	Hospitalized	Original		B.1	G	India	Undertermined	No (couldn't determine the patient status as either pre-symptomatic / asymptomatic or symptomatic conclusively)
hCoV-19/India/GJ-GBRC157b/2020	EPI_ISL_461487	27/05/2020	Asia / India / Gujarat / Ahmedabad	Human	Male	83	Hospitalized	Original		B.1.80	G	India	Undertermined	No (couldn't determine the patient status as either pre-symptomatic / asymptomatic or symptomatic conclusively)
hCoV-19/India/GJ-GBRC158/2020	EPI_ISL_461488	27/05/2020	Asia / India / Gujarat / Ahmedabad	Human	Female	50	Hospitalized	Original		B.1.113	GH	India	Undertermined	No (couldn't determine the patient status as either pre-symptomatic / asymptomatic or symptomatic conclusively)
hCoV-19/India/GJ-GBRC159a/2020	EPI_ISL_461489	27/05/2020	Asia / India / Gujarat / Ahmedabad	Human	Female	45	Hospitalized	Original		B.1.113	GH	India	Undertermined	No (couldn't determine the patient status as either pre-symptomatic / asymptomatic or symptomatic conclusively)
hCoV-19/India/GJ-GBRC159b/2020	EPI_ISL_461490	27/05/2020	Asia / India / Gujarat / Ahmedabad	Human	Female	45	Hospitalized	Original		B.1	G	India	Undertermined	No (couldn't determine the patient status as either pre-symptomatic / asymptomatic or symptomatic conclusively)
hCoV-19/India/GJ-GBRC160a/2020	EPI_ISL_461491	27/05/2020	Asia / India / Gujarat / Ahmedabad	Human	Male	60	Hospitalized	Original		B.1.113	G	India	Undertermined	No (couldn't determine the patient status as either pre-symptomatic / asymptomatic or symptomatic conclusively)
hCoV-19/India/GJ-GBRC160b/2020	EPI_ISL_461492	27/05/2020	Asia / India / Gujarat / Ahmedabad	Human	Male	60	Hospitalized	Original		B.1.80	GH	India	Undertermined	No (couldn't determine the patient status as either pre-symptomatic / asymptomatic or symptomatic conclusively)
hCoV-19/India/GJ-GBRC161a/2020	EPI_ISL_461493	27/05/2020	Asia / India / Gujarat / Ahmedabad	Human	Female	45	Deceased	Original		B.1.80	G	India	Symptomatic	Yes
hCoV-19/India/GJ-GBRC161b/2020	EPI_ISL_461494	27/05/2020	Asia / India / Gujarat / Ahmedabad	Human	Female	45	Deceased	Original		B.1.80	GH	India	Symptomatic	Yes
hCoV-19/India/GJ-GBRC162a/2020	EPI_ISL_461495	27/05/2020	Asia / India / Gujarat / Ahmedabad	Human	Male	54	Hospitalized	Original		B.1.113	GH	India	Undertermined	No (couldn't determine the patient status as either pre-symptomatic / asymptomatic or symptomatic conclusively)
hCoV-19/India/GJ-GBRC162b/2020	EPI_ISL_461496	27/05/2020	Asia / India / Gujarat / Ahmedabad	Human	Male	54	Hospitalized	Original		B.1.80	G	India	Undertermined	No (couldn't determine the patient status as either pre-symptomatic / asymptomatic or symptomatic conclusively)
hCoV-19/India/GJ-GBRC163a/2020	EPI_ISL_461497	27/05/2020	Asia / India / Gujarat / Ahmedabad	Human	Female	58	Released	Original		B.1	G	India	Undertermined	No (couldn't determine the patient status as either pre-symptomatic / asymptomatic or symptomatic conclusively)
hCoV-19/India/GJ-GBRC163b/2020	EPI_ISL_461498	27/05/2020	Asia / India / Gujarat / Ahmedabad	Human	Female	58	Released	Original		B.1	G	India	Undertermined	No (couldn't determine the patient status as either pre-symptomatic / asymptomatic or symptomatic conclusively)
hCoV-19/India/GJ-GBRC164/2020	EPI_ISL_461499	27/05/2020	Asia / India / Gujarat / Ahmedabad	Human	Male	73	Hospitalized	Original		B.1.113	GH	India	Undertermined	No (couldn't determine the patient status as either pre-symptomatic / asymptomatic or symptomatic conclusively)
hCoV-19/Bosnia and Herzegovina/02-Banja Luka/2020	EPI_ISL_462990	29/04/2020	Europe / Bosnia and Herzegovina / ÄŒelinac	Human	Male	78	Deceased	Original	Nasopharyngeal swab	B.1.1	GR	Bosnia and Herzegovina	Symptomatic	Yes
hCoV-19/Mongolia/Imp3989/2020	EPI_ISL_467666	25/03/2020	Asia / Mongolia	Human	Male	32	Hospitalized	Original	Oro-pharyngeal swab	B.1	G	Mongolia	Undertermined	No (couldn't determine the patient status as either pre-symptomatic / asymptomatic or symptomatic conclusively)
hCoV-19/USA/CA-CSMC1/2020	EPI_ISL_467809	27/03/2020	North America / USA / California / Los Angeles	Human	Male	60	Released	Original		B.1	GH	USA	Undertermined	No (couldn't determine the patient status as either pre-symptomatic / asymptomatic or symptomatic conclusively)
hCoV-19/Italy/APU-UniMI-703/2020	EPI_ISL_468914	18/03/2020	Europe / Italy / Apulia / Taranto	Human	Female	26	Symptomatic	Original		B.1.1	GR	Italy	Symptomatic	Yes
hCoV-19/Gambia/0214/2020	EPI_ISL_471158	29/03/2020	Africa / Gambia / Kombo	Human	Male	20	Released	Original		B.1.1	G	Gambia	Undertermined	No (couldn't determine the patient status as either pre-symptomatic / asymptomatic or symptomatic conclusively)
hCoV-19/Gambia/0268/2020	EPI_ISL_471159	21/03/2020	Africa / Gambia / Kombo	Human	Male	62	Recovered	Original		B.1.1	GR	Gambia	Undertermined	No (couldn't determine the patient status as either pre-symptomatic / asymptomatic or symptomatic conclusively)
hCoV-19/Gambia/0278/2020	EPI_ISL_471160	20/03/2020	Africa / Gambia / Kombo	Human	Female	28	Released	Original		B.1.1	GR	Gambia	Undertermined	No (couldn't determine the patient status as either pre-symptomatic / asymptomatic or symptomatic conclusively)
hCoV-19/Gambia/0528/2020	EPI_ISL_471161	30/04/2020	Africa / Gambia / Kombo	Human	Male	31	Released	Original		B.1.1	GR	Gambia	Undertermined	No (couldn't determine the patient status as either pre-symptomatic / asymptomatic or symptomatic conclusively)
hCoV-19/Gambia/0536/2020	EPI_ISL_471163	30/04/2020	Africa / Gambia / Kombo	Human	Female	22	Released	Original		B.1.1	GR	Gambia	Undertermined	No (couldn't determine the patient status as either pre-symptomatic / asymptomatic or symptomatic conclusively)
hCoV-19/Gambia/0547/2020	EPI_ISL_471164	30/04/2020	Africa / Gambia / Kombo	Human	Male	21	Released	Original		B.1.1	GR	Gambia	Undertermined	No (couldn't determine the patient status as either pre-symptomatic / asymptomatic or symptomatic conclusively)
hCoV-19/Gambia/1090/2020	EPI_ISL_471166	04/05/2020	Africa / Gambia / Kombo	Human	Male	23	Released	Original		B.1.1	GR	Gambia	Undertermined	No (couldn't determine the patient status as either pre-symptomatic / asymptomatic or symptomatic conclusively)
hCoV-19/Gambia/1094/2020	EPI_ISL_471167	04/05/2020	Africa / Gambia / Kombo	Human	Male	23	Released	Original		B.1.1	GR	Gambia	Undertermined	No (couldn't determine the patient status as either pre-symptomatic / asymptomatic or symptomatic conclusively)
hCoV-19/Gambia/1251/2020	EPI_ISL_471168	07/05/2020	Africa / Gambia / Kombo	Human	Male	6	Released	Original		B.1.1	GR	Gambia	Undertermined	No (couldn't determine the patient status as either pre-symptomatic / asymptomatic or symptomatic conclusively)
hCoV-19/Gambia/1264/2020	EPI_ISL_471169	07/05/2020	Africa / Gambia / Kombo	Human	Male	69	Released	Original		B.1.1	GR	Gambia	Undertermined	No (couldn't determine the patient status as either pre-symptomatic / asymptomatic or symptomatic conclusively)
hCoV-19/Gambia/1277/2020	EPI_ISL_471171	07/05/2020	Africa / Gambia / Kombo	Human	Male	58	Released	Original		B.1.1	GR	Gambia	Undertermined	No (couldn't determine the patient status as either pre-symptomatic / asymptomatic or symptomatic conclusively)
hCoV-19/India/MP-DRDE08/2020	EPI_ISL_476022	25/03/2020	Asia / India / Madhya Pradesh	Human	Male	23	Hospitalized	Original	Oro-pharyngeal swab	B	L	India	Undertermined	No (couldn't determine the patient status as either pre-symptomatic / asymptomatic or symptomatic conclusively)
hCoV-19/India/MP-DRDE115/2020	EPI_ISL_476023	31/03/2020	Asia / India / Madhya Pradesh	Human	Male	38	Hospitalized	Original	Oro-pharyngeal swab	B	L	India	Undertermined	No (couldn't determine the patient status as either pre-symptomatic / asymptomatic or symptomatic conclusively)
hCoV-19/Senegal/55738/2020	EPI_ISL_477141	07/06/2020	Africa / Senegal / Dakar	Human	Male	35	Released	Original	Oro/naso-pharyngeal swab	B.1.5	G	Senegal	Undertermined	No (couldn't determine the patient status as either pre-symptomatic / asymptomatic or symptomatic conclusively)
hCoV-19/Senegal/68525/2020	EPI_ISL_477142	17/06/2020	Africa / Senegal / Dakar	Human	Male	37	Released	Original	Oro/naso-pharyngeal swab	B.1.1	GR	Senegal	Undertermined	No (couldn't determine the patient status as either pre-symptomatic / asymptomatic or symptomatic conclusively)
hCoV-19/Senegal/68303/2020	EPI_ISL_477143	17/06/2020	Africa / Senegal / Dakar	Human	Male	32	Released	Original	Oro/naso-pharyngeal swab	B.1.5	G	Senegal	Undertermined	No (couldn't determine the patient status as either pre-symptomatic / asymptomatic or symptomatic conclusively)
hCoV-19/Senegal/70427/2020	EPI_ISL_477144	20/06/2020	Africa / Senegal / Dakar	Human	Male	70	Released	Original	Oro/naso-pharyngeal swab	B.1.5	G	Senegal	Undertermined	No (couldn't determine the patient status as either pre-symptomatic / asymptomatic or symptomatic conclusively)
hCoV-19/Senegal/70440/2020	EPI_ISL_477145	19/06/2020	Africa / Senegal / Dakar	Human	Female	31	Released	Original	Oro/naso-pharyngeal swabs	B.1.5	G	Senegal	Undertermined	No (couldn't determine the patient status as either pre-symptomatic / asymptomatic or symptomatic conclusively)
hCoV-19/Senegal/70339/2020	EPI_ISL_477146	20/06/2020	Africa / Senegal / Dakar	Human	Male	58	Released	Original	Oro/naso-pharyngeal swabs	B.1	G	Senegal	Undertermined	No (couldn't determine the patient status as either pre-symptomatic / asymptomatic or symptomatic conclusively)
hCoV-19/Senegal/70541/2020	EPI_ISL_477147	14/06/2020	Africa / Senegal / Dakar	Human	Male	55	Released	Original		B.1	G	Senegal	Undertermined	No (couldn't determine the patient status as either pre-symptomatic / asymptomatic or symptomatic conclusively)
hCoV-19/Senegal/55703/2020	EPI_ISL_477148	07/06/2020	Africa / Senegal / Dakar	Human	Female	24	Released	Original	Oro/naso-pharyngeal swabs	B.1	GH	Senegal	Undertermined	No (couldn't determine the patient status as either pre-symptomatic / asymptomatic or symptomatic conclusively)
hCoV-19/Senegal/55752/2020	EPI_ISL_477149	07/06/2020	Africa / Senegal / Dakar	Human	Female	36	Released	Original		B.1.5	G	Senegal	Undertermined	No (couldn't determine the patient status as either pre-symptomatic / asymptomatic or symptomatic conclusively)
hCoV-19/Senegal/55728/2020	EPI_ISL_477150	07/06/2020	Africa / Senegal / Dakar	Human	Male	38	Released	Original	Oro/naso-pharyngeal swabs	B.1.5	G	Senegal	Undertermined	No (couldn't determine the patient status as either pre-symptomatic / asymptomatic or symptomatic conclusively)
hCoV-19/Senegal/65481/2020	EPI_ISL_477151	14/06/2020	Africa / Senegal / Dakar	Human	Female	25	Released	Original	Oro/naso-pharyngeal swabs	B.1.1	GR	Senegal	Undertermined	No (couldn't determine the patient status as either pre-symptomatic / asymptomatic or symptomatic conclusively)
hCoV-19/Senegal/65526/2020	EPI_ISL_477152	14/06/2020	Africa / Senegal / Dakar	Human	Female	75	Released	Original	Oro/naso-pharyngeal swabs	B.1	G	Senegal	Undertermined	No (couldn't determine the patient status as either pre-symptomatic / asymptomatic or symptomatic conclusively)
hCoV-19/Senegal/65548/2020	EPI_ISL_477153	14/06/2020	Africa / Senegal / Dakar	Human	Male	20	Released	Original		B.1.5	G	Senegal	Undertermined	No (couldn't determine the patient status as either pre-symptomatic / asymptomatic or symptomatic conclusively)
hCoV-19/Senegal/64772/2020	EPI_ISL_477154	13/06/2020	Africa / Senegal / Dakar	Human	Female	28	Released	Original	Oro/naso-pharyngeal swabs	B.1	G	Senegal	Undertermined	No (couldn't determine the patient status as either pre-symptomatic / asymptomatic or symptomatic conclusively)
hCoV-19/Senegal/64729/2020	EPI_ISL_477155	11/06/2020	Africa / Senegal / Dakar	Human	Female	10	Released	Original	Oro/naso-pharyngeal swabs	B.1.1	GR	Senegal	Undertermined	No (couldn't determine the patient status as either pre-symptomatic / asymptomatic or symptomatic conclusively)
hCoV-19/Senegal/64916/2020	EPI_ISL_477156	13/06/2020	Africa / Senegal / Dakar	Human	Male	23	Released	Original	Oro/naso-pharyngeal swabs	B.1.5	G	Senegal	Undertermined	No (couldn't determine the patient status as either pre-symptomatic / asymptomatic or symptomatic conclusively)
hCoV-19/Senegal/65095/2020	EPI_ISL_477157	13/06/2020	Africa / Senegal / Dakar	Human	Male	82	Released	Original	Oro/naso-pharyngeal swabs	B.1	G	Senegal	Undertermined	No (couldn't determine the patient status as either pre-symptomatic / asymptomatic or symptomatic conclusively)
hCoV-19/Senegal/63930/2020	EPI_ISL_477158	12/06/2020	Africa / Senegal / Dakar	Human	Male	74	Released	Original	Oro/naso-pharyngeal swabs	B.1.5	G	Senegal	Undertermined	No (couldn't determine the patient status as either pre-symptomatic / asymptomatic or symptomatic conclusively)
hCoV-19/Senegal/64163/2020	EPI_ISL_477159	12/06/2020	Africa / Senegal / Dakar	Human	Female	37	Released	Original	Oro/naso-pharyngeal swabs	B.1.5	G	Senegal	Undertermined	No (couldn't determine the patient status as either pre-symptomatic / asymptomatic or symptomatic conclusively)
hCoV-19/Singapore/470/2020	EPI_ISL_477170	16/02/2020	Asia / Singapore	Human	Female	54	Hospitalized	Original		B	O	Singapore	Undertermined	No (couldn't determine the patient status as either pre-symptomatic / asymptomatic or symptomatic conclusively)
hCoV-19/Singapore/481/2020	EPI_ISL_477171	16/03/2020	Asia / Singapore	Human	Male	30	Hospitalized	Original		B.1.5	G	Singapore	Undertermined	No (couldn't determine the patient status as either pre-symptomatic / asymptomatic or symptomatic conclusively)
hCoV-19/Singapore/431nan/2020	EPI_ISL_477172	18/03/2020	Asia / Singapore	Human	Female	19	Hospitalized	Original		B.1.1	GR	Singapore	Undertermined	No (couldn't determine the patient status as either pre-symptomatic / asymptomatic or symptomatic conclusively)
hCoV-19/Singapore/451nan/2020	EPI_ISL_477174	20/03/2020	Asia / Singapore	Human	Male	31	Hospitalized	Original		B.1.1	GR	Singapore	Undertermined	No (couldn't determine the patient status as either pre-symptomatic / asymptomatic or symptomatic conclusively)
hCoV-19/Singapore/485/2020	EPI_ISL_477175	22/03/2020	Asia / Singapore	Human	Male	54	Hospitalized	Original		B	L	Singapore	Undertermined	No (couldn't determine the patient status as either pre-symptomatic / asymptomatic or symptomatic conclusively)
hCoV-19/Singapore/159nan/2020	EPI_ISL_477177	21/03/2020	Asia / Singapore	Human	Male	30	Hospitalized	Original		B	L	Singapore	Undertermined	No (couldn't determine the patient status as either pre-symptomatic / asymptomatic or symptomatic conclusively)
hCoV-19/Singapore/278nan/2020	EPI_ISL_477178	22/03/2020	Asia / Singapore	Human	Male	43	Hospitalized	Original		B.1	GH	Singapore	Undertermined	No (couldn't determine the patient status as either pre-symptomatic / asymptomatic or symptomatic conclusively)
hCoV-19/Singapore/268nan/2020	EPI_ISL_477180	22/06/2020	Asia / Singapore	Human	Female	18	Hospitalized	Original		B.1.5	G	Singapore	Undertermined	No (couldn't determine the patient status as either pre-symptomatic / asymptomatic or symptomatic conclusively)
hCoV-19/Singapore/488/2020	EPI_ISL_477182	13/04/2020	Asia / Singapore	Human	Male	42	Hospitalized	Original		B.6	O	Singapore	Undertermined	No (couldn't determine the patient status as either pre-symptomatic / asymptomatic or symptomatic conclusively)
hCoV-19/India/GJ-GBRC234/2020	EPI_ISL_477183	12/06/2020	Asia / India / Gujarat / Choryasi	Human	Male	42	Released	Original		B.1	G	India	Undertermined	No (couldn't determine the patient status as either pre-symptomatic / asymptomatic or symptomatic conclusively)
hCoV-19/Singapore/489/2020	EPI_ISL_477184	13/04/2020	Asia / Singapore	Human	Male	46	Hospitalized	Original		B.6	O	Singapore	Undertermined	No (couldn't determine the patient status as either pre-symptomatic / asymptomatic or symptomatic conclusively)
hCoV-19/Singapore/490/2020	EPI_ISL_477187	26/04/2020	Asia / Singapore	Human	Male	44	Hospitalized	Original		B.6	O	Singapore	Undertermined	No (couldn't determine the patient status as either pre-symptomatic / asymptomatic or symptomatic conclusively)
hCoV-19/Singapore/491/2020	EPI_ISL_477188	26/04/2020	Asia / Singapore	Human	Male	47	Hospitalized	Original		B.6	O	Singapore	Undertermined	No (couldn't determine the patient status as either pre-symptomatic / asymptomatic or symptomatic conclusively)
hCoV-19/Singapore/492/2020	EPI_ISL_477189	26/04/2020	Asia / Singapore	Human	Male	50	Hospitalized	Original		B.6	O	Singapore	Undertermined	No (couldn't determine the patient status as either pre-symptomatic / asymptomatic or symptomatic conclusively)
hCoV-19/Singapore/493/2020	EPI_ISL_477190	26/04/2020	Asia / Singapore	Human	Male	55	Hospitalized	Original		B.6	O	Singapore	Undertermined	No (couldn't determine the patient status as either pre-symptomatic / asymptomatic or symptomatic conclusively)
hCoV-19/Singapore/494/2020	EPI_ISL_477191	26/04/2020	Asia / Singapore	Human	Male	50	Hospitalized	Original		B.6	O	Singapore	Undertermined	No (couldn't determine the patient status as either pre-symptomatic / asymptomatic or symptomatic conclusively)
hCoV-19/Singapore/495/2020	EPI_ISL_477192	27/04/2020	Asia / Singapore	Human	Male	52	Hospitalized	Original		B.6	O	Singapore	Undertermined	No (couldn't determine the patient status as either pre-symptomatic / asymptomatic or symptomatic conclusively)
hCoV-19/Italy/APU-UniMI-45/2020	EPI_ISL_477193	16/03/2020	Europe / Italy / Apulia	Human	Female	62	symptomatic	Original	Nasopharyngeal swab	B.1	G	Italy	Symptomatic	Yes
hCoV-19/Italy/APU-UniMI-39/2020	EPI_ISL_477194	16/03/2020	Europe / Italy / Apulia	Human	Male	67	symptomatic	Original	Nasopharyngeal swab	B.1.1	GR	Italy	Symptomatic	Yes
hCoV-19/Italy/FVG-ICGEB-bal18/2020	EPI_ISL_479617	27/03/2020	Europe / Italy / Friuli Venezia Giulia	Human	Male	47	Hospitalized, released	Original	Alveolar lavage fluid	B.1	G	Italy	Undertermined	No (couldn't determine the patient status as either pre-symptomatic / asymptomatic or symptomatic conclusively)
hCoV-19/Italy/FVG-ICGEB-bal16/2020	EPI_ISL_479618	12/04/2020	Europe / Italy / Friuli Venezia Giulia	Human	Male	59	Hospitalized, deceased	Original	Alveolar lavage fluid	B.1	G	Italy	Symptomatic	Yes
hCoV-19/Italy/FVG-ICGEB-atr18/2020	EPI_ISL_479619	12/04/2020	Europe / Italy / Friuli Venezia Giulia	Human	Male	59	Hospitalized, deceased	Original	Tracheal swab	B.1	G	Italy	Symptomatic	Yes

hCoV-19/Russia/CRIE182061/2020	EPI_ISL_479620	31/03/2020	Europe / Russia / Moscow	Human		male	21	ICD-10 Disease: J06.9 Acute upper respiratory infection, unspecified	Original	Oro-pharyngeal swab		B.1.1	GR	Russia	Symptomatic	Yes
hCoV-19/Russia/CRIE161454/2020	EPI_ISL_479621	31/03/2020	Europe / Russia / Moscow	Human		male	28	ICD-10 Disease: J06.9 Acute upper respiratory infection, unspecified	Original	Oro-pharyngeal swab		B.1.1	GR	Russia	Symptomatic	Yes
hCoV-19/Russia/CRIE164776/2020	EPI_ISL_479622	31/03/2020	Europe / Russia / Moscow	Human		male	82	ICD-10 Disease: Z03.8 Observation for other suspected diseases and conditions	Original	Oro-pharyngeal swab		B.1	GH	Russia	Undertermined	No (couldn't determine the patient status as either pre-symptomatic / asymptomatic or symptomatic conclusively)
hCoV-19/Russia/CRIE180117/2020	EPI_ISL_479623	01/04/2020	Europe / Russia / Moscow	Human		unknown	64	ICD-10 Disease: Z03.8 Observation for other suspected diseases and conditions	Original	Oro-pharyngeal swab		B.1	GH	Russia	Undertermined	No (couldn't determine the patient status as either pre-symptomatic / asymptomatic or symptomatic conclusively)
hCoV-19/Russia/CRIE155506/2020	EPI_ISL_479624	30/03/2020	Europe / Russia / Moscow	Human		male	57	ICD-10 Disease: J06.9 Acute upper respiratory infection, unspecified	Original	Oro-pharyngeal swab		B.1.1	GR	Russia	Symptomatic	Yes
hCoV-19/India/MH-NIV-25949/2020	EPI_ISL_479657	09/05/2020	Asia / India / Maharashtra	Human		Male	75	Symptomatic	Original			B.1.80	G	India	Symptomatic	Yes
hCoV-19/India/MH-NIV-26133/2020	EPI_ISL_479658	12/05/2020	Asia / India / Maharashtra	Human		Female	57	Symptomatic	Original			B.1.1	GR	India	Symptomatic	Yes
hCoV-19/India/MH-NIV-39037/2020	EPI_ISL_479659	22/05/2020	Asia / India / Maharashtra	Human		Female	56	Symptomatic	Original			B.1.1.32	GR	India	Symptomatic	No (unusually high diversity)
hCoV-19/Italy/FVG-ICGEB-atr21/2020	EPI_ISL_479790	13/04/2020	Europe / Italy / Friuli Venezia Giulia	Human		Male	67	Hospitalized, deceased	Original	Tracheal swab		B.1	G	Italy	Symptomatic	Yes
hCoV-19/Italy/FVG-ICGEB-S26/2020	EPI_ISL_479791	08/03/2020	Europe / Italy / Friuli Venezia Giulia	Human		Male	71	Hospitalized	Original	Oropharyngeal swab		B.1	G	Italy	Undertermined	No (couldn't determine the patient status as either pre-symptomatic / asymptomatic or symptomatic conclusively)
hCoV-19/Japan/PG-0003/2020	EPI_ISL_479792	2020-02	Asia / Japan / Hokkaido	Human		unknown	unknown	Symptomatic	Original	Pharyngeal swab		B	L	Japan	Symptomatic	Yes
hCoV-19/Japan/PG-0004/2020	EPI_ISL_479793	2020-02	Asia / Japan / Hokkaido	Human		unknown	unknown	Symptomatic	Original	Pharyngeal swab		B	L	Japan	Symptomatic	Yes
hCoV-19/Japan/PG-0005/2020	EPI_ISL_479794	2020-02	Asia / Japan / Hokkaido	Human		unknown	unknown	Symptomatic	Original	Pharyngeal swab		B	L	Japan	Symptomatic	Yes
hCoV-19/Japan/PG-0006/2020	EPI_ISL_479795	2020-02	Asia / Japan / Hokkaido	Human		unknown	unknown	Symptomatic	Original	Pharyngeal swab		B	L	Japan	Symptomatic	Yes
hCoV-19/Japan/PG-0009/2020	EPI_ISL_479796	2020-02	Asia / Japan / HokurikuShinsyu	Human		unknown	unknown	Symptomatic	Original	Pharyngeal swab		B.1	GH	Japan	Symptomatic	Yes
hCoV-19/Japan/PG-0010/2020	EPI_ISL_479797	18/02/2020	Asia / Japan / Kanto	Human		unknown	unknown	Symptomatic	Original	Pharyngeal swab		B	L	Japan	Symptomatic	Yes
hCoV-19/Japan/PG-0011/2020	EPI_ISL_479798	18/02/2020	Asia / Japan / Kanto	Human		unknown	unknown	Symptomatic	Original	Pharyngeal swab		B	L	Japan	Symptomatic	Yes
hCoV-19/Japan/PG-0015/2020	EPI_ISL_479799	20/01/2020	Asia / Japan / Hokkaido	Human		unknown	unknown	Symptomatic	Original	Pharyngeal swab		B	L	Japan	Symptomatic	Yes
hCoV-19/Japan/PG-0195/2020	EPI_ISL_479900	16/03/2020	Asia / Japan / Kanto	Human		unknown	unknown	Symptomatic	Original	Pharyngeal swab		B.1.36	GH	Japan	Symptomatic	Yes
hCoV-19/Japan/PG-0196/2020	EPI_ISL_479901	19/03/2020	Asia / Japan / Kanto	Human		unknown	unknown	Symptomatic	Original	Pharyngeal swab		B.1.36	GH	Japan	Symptomatic	Yes
[truncated: 4,468,060 more chars]
